# Development of a 690 K SNP array in catfish and its application for genetic mapping and validation of the reference genome sequence

**DOI:** 10.1038/srep40347

**Published:** 2017-01-12

**Authors:** Qifan Zeng, Qiang Fu, Yun Li, Geoff Waldbieser, Brian Bosworth, Shikai Liu, Yujia Yang, Lisui Bao, Zihao Yuan, Ning Li, Zhanjiang Liu

**Affiliations:** 1 The Fish Molecular Genetics and Biotechnology Laboratory, School of Fisheries, Aquaculture, and Aquatic Sciences, and Program of Cell and Molecular Biosciences, Auburn University, Auburn, Alabama 36849, United States of America; 2 USDA-ARS Warmwater Aquaculture Research Unit, Stoneville, Mississippi 38776, United States of America

## Abstract

Single nucleotide polymorphisms (SNPs) are capable of providing the highest level of genome coverage for genomic and genetic analysis because of their abundance and relatively even distribution in the genome. Such a capacity, however, cannot be achieved without an efficient genotyping platform such as SNP arrays. In this work, we developed a high-density SNP array with 690,662 unique SNPs (herein 690 K array) that were relatively evenly distributed across the entire genome, and covered 98.6% of the reference genome sequence. Here we also report linkage mapping using the 690 K array, which allowed mapping of over 250,000 SNPs on the linkage map, the highest marker density among all the constructed linkage maps. These markers were mapped to 29 linkage groups (LGs) with 30,591 unique marker positions. This linkage map anchored 1,602 scaffolds of the reference genome sequence to LGs, accounting for over 97% of the total genome assembly. A total of 1,007 previously unmapped scaffolds were placed to LGs, allowing validation and in few instances correction of the reference genome sequence assembly. This linkage map should serve as a valuable resource for various genetic and genomic analyses, especially for GWAS and QTL mapping for genes associated with economically important traits.

Catfish is the primary aquaculture species in the US, accounting for approximately 60% of US aquaculture production. Channel catfish (*Ictalurus punctatus*) and blue catfish (*I. furcatus*) are the two most important species in catfish farming industry due to their high tolerance in a wide range of environmental conditions. They have been also used as models for comparative immunology and environmental toxicology^1^. In the past decades, various genetic and genomic resources have been produced to facilitate catfish genetic improvement and breeding programs, including development of molecular markers^2^,^3^,^4^,^5^, BAC-based physical maps^6^,^7^,^8^, expression sequence tags (ESTs)^9^,^10^,^11^,^12^,^13^,^14^,^15^ and transcriptome sequencing using RNA-Seq^16^,^17^,^18^,^19^,^20^,^21^,^22^,^23^, genetic linkage maps^24^,^25^,^26^,^27^,^28^,^29^, and generation of whole genome reference sequences^1^.

However, rapid progress in catfish genetics research has been hindered by the lack of effective and efficient genotyping platforms. Recently, we developed the catfish 250 K SNP array using Affymetrix axiom technology^30^. This array has been of great use for various genetic analysis including genetic linkage mapping^26^,^28^ and analysis of performance traits using GWAS^31^,^32^,^33^. However, the 250 K SNP array was designed without a reference genome. As a result, there were limitations with equal spacing of marker intervals and complete genome coverage. The recent catfish reference genome assembly^1^ provided a platform for the construction of a high-density SNP array with improved genome coverage. In addition, independent genetic analyses using linkage mapping should allow greater levels of integration of the reference genome sequence with linkage maps, validation of the reference genome sequence, and correction of potential mistakes in the reference genome sequence.

Linkage maps are important for genetic analysis because they serve as a chromosomal-level framework to trace inheritance of various traits. However, for several decades, progress of linkage mapping was limited to relatively low density of markers because the marker systems available then were mostly limited to microsatellites and AFLPs, for which highly efficient automated genotyping systems were not available. Until recently, linkage maps carried over thousands of SNP markers have been constructed in several aquaculture species including Atlantic salmon (*Salmo salar*)^34^,^35^, rainbow trout (*Oncorhynchus mykiss*)^36^, Japanese flounder (*Paralichthys olivaceus*)^37^,^38^, Asian seabass (*Lates calcarifer*)^39^,^40^, sea bream (*Sparus auratus*)^41^, bighead carp (*Hypophthalmichthys nobilis*)^42^, yellowtail (*Seriola quinqueradiata*)^43^, Pacific oyster (*Crassostrea gigas*)^44^,^45^, tiger shrimp (*Penaeus monodon*)^46^. kuruma prawn (*Marsupenaeus japonicas*)^47^, Pacific white shrimp (*Litopenaeus vannamei*)^48^,^49^, silver-lipped pearl oyster (*Pinctada maxima*)^50^, common carp (*Cyprinus carpio*)^51^, gudgeons of the genus *Gnathopogon*^52^, European sea bass (*Dicentrarchus labrax*)^53^, sea urchins (*Strongylocentrotus nudus* and *S. intermedius*)^54^, sea cucumber (*Apostichopus japonicus*)^55^, turbot (*Scophthalmus maximus*)^56^, large yellow croaker (*Larimichthys crocea*)^57^,^58^, Atlantic halibut (*Hippoglossus hippoglossus*)^59^, zhikong scallop (*Chlamys farreri*)^60^, chinook salmon (*Oncorhynchus tshawytscha*)^61^, triangle sail mussel (*Hyriopsis cumingii*)^62^, coho salmon (*Oncorhynchus kisutch*)^63^, Japanese eel (*Anguilla japonica*)^64^, and pearl oyster (*Pinctada fucata*)^65^. Similarly, for many years, catfish genetic maps were limited to relatively low densities^24^,^25^,^27^,^66^. It was only after the application of the automated SNP array genotyping systems, high density linkage maps were produced^26^,^28^. Although the latest channel catfish linkage map carried over 50,000 SNP markers, a large number of scaffolds of the reference genome sequence were still not anchored to chromosomes. In addition, about half of the mapped markers were completely linked due to the relatively low resolution, hindering the validation of the reference genome sequence assembly^28^. A linkage map with super-density of markers and high resolution is still desired in order to provide a high level of integration of the linkage map with the reference genome sequence. In this work, we developed a high-density SNP array with 690,662 unique markers, and used this array to construct a catfish genetic linkage map with the highest marker density among any of the constructed linkage maps. The use of four resource families with a total of 465 individuals allowed a greater resolution, with 30,591 unique mapped marker positions. Comparative analysis of marker orders on the genetic map and on the reference genome sequence allowed validation and correction of the reference genome sequence.

## Results

### SNPs identification and selection

SNPs were identified from a total of over 6.4 billion sequencing reads from 1,213 catfish (Table 1). Initially, a total of over 9.6 million putative SNPs were identified, including 8.6 million SNPs from channel catfish and 3.8 million SNPs from blue catfish. To select high-quality SNPs, 70 bp flanking sequences (35 bp upstream and downstream of the SNP site) of all the putative SNPs were mapped onto the catfish reference genome sequence and matched more than one locus were removed, resulting in approximately 5.7 million uniquely mapped SNPs from channel catfish and 3.1 million uniquely mapped SNPs from blue catfish. A total of ~1.8 million SNPs from channel catfish and ~300,000 SNPs from blue catfish were retained after additional steps of screening, including elimination of SNPs surrounded by simple sequence repeats, removal of tri-allelic SNPs, and removal of markers with low probability of conversion based on Affymetrix algorithms (Table 2).

### SNPs included on the 690 K array

The final SNPs included on the 690 K SNP array were summarized in Table 3. A total of 690,662 SNPs were selected, including 238,484 genic SNPs and 452,178 intergenic SNPs. The 238,484 genic SNPs were from 24,186 genes annotated from the channel catfish reference genome sequence, or from the transcripts assembled from RNA-Seq datasets. For most SNPs with unique flanking sequences, only one probe was designed on the array. However, two probes were designed on the array for 2,905 SNPs whose flanking sequences were less unique, leading to a total of 693,567 SNP probes on the 690 K SNP array.

The 690 K catfish SNP array also included species and strain specific SNPs (Table 3), which should be useful for genetic analysis of the interspecific hybrid system and intraspecific crossbreeding. Of the total SNPs on the array, 581,002 SNPs were specifically identified from channel catfish, 44,694 were exclusively observed in blue catfish, 19,124 were interspecific SNPs which were homozygous within but differing between species, and 45,842 SNPs that were heterozygous within and between species.

For strain identification, 48,434 SNPs from four catfish aquaculture strains that originate from different geographic locations were also included on the array. These four strains possess different production traits such as growth rates, disease resistance, and adaptation to environmental stresses. A total of 6,622 SNPs were specifically from a wild channel catfish population of the Coosa River, Alabama, which may be useful for genetic analysis of genomic regions with selective signatures. In addition to SNP probes, 2,000 data quality control (QC) probes were also included on the SNP array serving as negative controls.

**Figure 1:**
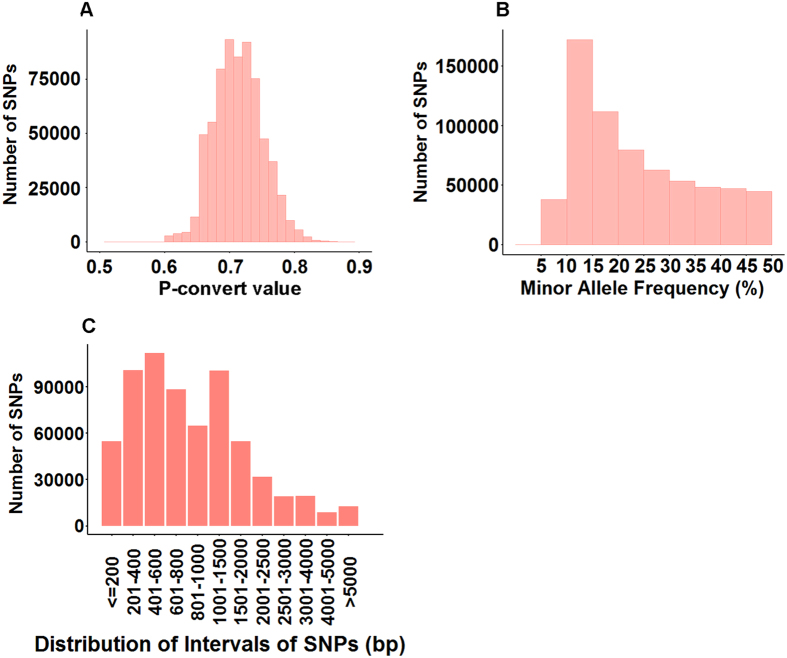
Summary of SNP metrics. (**A**) Distribution of SNP probes based on p-convert values. The x-axis represents the p-convert value; the y-axis represents the number of SNPs; (**B**) Distribution of SNPs based on MAF. The x-axis represents the Minor Allele Frequency (MAF); the y-axis represents the number of SNPs. (**C**) Distribution of inter-SNPs spacing for SNPs included on the array. The x-axis represents the length of intervals of SNPs; the y-axis represents the number of SNPs.

The SNPs included on the 690 K SNP array were of high quality, with an average p-convert value of 0.71; 97% of SNPs had a p-convert value of greater than 0.65 (Fig. 1A). A proportion of 89.6% of SNPs had a MAF greater than 0.1 (Fig. 1B). This distribution of MAF should provide the SNPs with a relatively high level of polymorphic content, a desired characteristic for linkage mapping, GWAS, and other genetic analysis.

The spacing among SNPs was evaluated using their physical position on the reference genome or on reference sequence contigs that were not mapped to chromosomes. As shown in Fig. 1C, over 86% SNPs had an inter-SNP spacing of less than 2,000 bp.

### Distribution of SNPs across the genome

**Figure 2:**
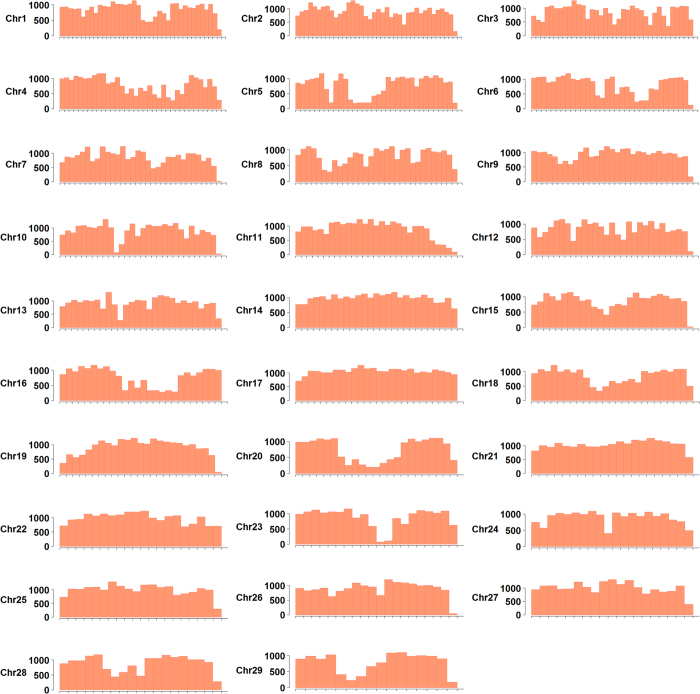
Genome distribution of SNPs on the 690 K SNP array. Each of the 29 channel catfish chromosomes was laid out in the x-axis with one million base pairs intervals. The number of SNPs residing in the interval was plotted on the y-axis.

One of the most important goals of the SNP array development is to have a good coverage of the entire genome with relatively even distribution of SNPs. The SNP locations on the reference genome sequence were used as the coordinates to assess their overall distribution. A total of 659,912 (95.5% of all the SNPs on the array) SNPs were developed from the reference genome sequence, which span a total of 778 Mb, approximately 99.4% of the assembled reference genome sequence. As shown in Fig. 2, almost all of the genomic regions were covered by SNPs, except a few highly repetitive regions from which convertible SNP probes could not be designed. The remaining 30,750 (4.5%) SNPs were developed from other genomic resources, including 15,108 SNPs from unmapped scaffolds and contigs, 521 SNPs from transcript sequences via *de novo* transcriptome assembly, and 15,121 SNPs from bacterial artificial chromosome (BAC) end sequences (BES). Taken together, all the SNPs on the array covered 98.6% of the sequences in the reference genome scaffolds and 93.9% of the BAC based physical map contigs.

### Performance of the catfish 690 K SNP array

Performance of the SNP array was examined by genotyping catfish DNA samples from hybrid backcross families and channel catfish domesticated families. As summarized in Table 4, 473 of 480 catfish samples (98.5%) were successfully genotyped after sample quality control. In backcross hybrids samples, a total of 597,323 (86.1%) SNPs were successfully genotyped, and 504,265 (72.7%) were polymorphic in these 84 individuals. The average call rate of dish quality control (DQC) qualified samples was greater than 99.2%. In channel catfish samples, a total of 578,868 (83.5%) SNPs were converted, of which 467,821 (67.5%) were polymorphic from 396 tested fish of Delta select strain.

### Construction of channel catfish linkage map

**Figure 3:**
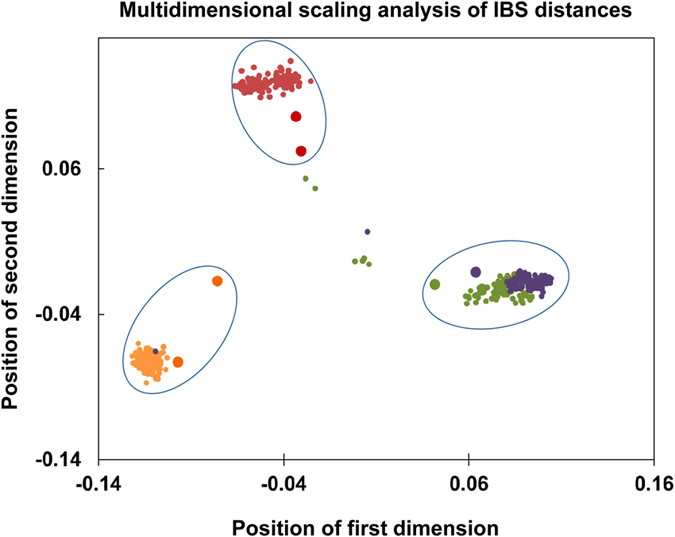
Sample structure identified by multidimensional scaling analysis of IBS distances. The coordinates were the first two clustering dimensions. Large dots represent parent samples.

A total of 478 channel catfish samples of four mapping families were used for linkage mapping. After applying the criteria of DQC score greater than 0.82 and call rate greater than 97%, 5 samples with poor qualities were eliminated. Genotyping data of the remaining 473 samples were imported into Plink for a pedigree information test (Fig. 3). The vast majority of samples fell into three clusters, with two families gathered together because they were generated from one sire. Eight individual outliers were identified and excluded from linkage analysis. The genotyping data of remaining 465 samples were imported into Lep-map2 for SNP filtering prior to linkage group assignment. According to the assessment of genotyping quality and polymorphism in all samples from the four reference families, a total of 287,583 SNPs were informative in at least two families.

A total of 287,370 qualified SNPs were successfully assigned into 29 linkage groups, which was in concordance with the number of chromosomes of the catfish haploid genome. A two-round marker ordering procedure were carried out with the four families simultaneously. The first round of marker ordering identified 116,864 representative markers. By using a hidden Markov model (HMM), the OrderMarkers module modeled recombinant haplotypes and identify duplicated markers. After filtering these duplicated markers, a second round of marker ordering was performed to improve the order of representative markers. Finally, the previously excluded duplicate and stacked markers were inserted back into the maker order to calculate the genetic distance. A total of 253,087 markers were placed onto the linkage map.

**Figure 4:**
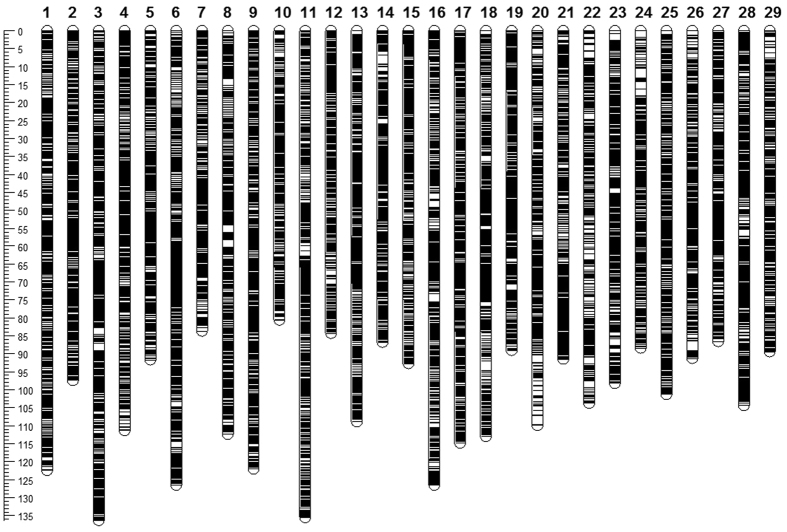
Illustration of sex-average linkage map.

The sex-average genetic distances were calculated by taking account the recombination probabilities in both sexes. As summarized in Table 5, the sex-average map consists of 253,087 markers including 30,591 unique positions, with a total genetic length of 3,004.7 cM. The marker intervals estimated based on the unique marker positions ranged from 0.08 cM/marker pair in LG12 and LG13 to 0.13 cM/marker pair in LG22, with an average marker interval of 0.1 cM/marker pair in sex-average genetic map. As illustrated in Fig. 4, there were no abnormal large gaps on the genetic map. The detailed information on marker position is provided in Supplemental Table S1.

The sex-specific genetic distances were calculated by taking account the recombination rates in only one sex. The female genetic map consisted of 23,610 unique positions, with a total genetic length of 3,582.3 cM (Table 6, Supplemental Fig. 1). The marker intervals estimated based on the unique marker positions ranged from 0.13 cM/marker pair in LG5, LG13, LG19 and LG21 to 0.18 cM/marker in LG22 and LG29, with an average marker interval of 0.15 cM/marker in the female genetic map. The male genetic map consists of 18,339 unique markers, with a total genetic length of 2,545.6 cM (Table 6, Supplemental Fig. 2). The marker intervals estimated based on the unique marker positions ranged from 0.11 cM/marker in LG12 to 0.17 cM/marker in LG22, with an average marker interval of 0.14 cM/marker on male genetic map.

The difference between female and male linkage maps was assessed according to contingency G-test^67^. Significantly higher recombination rates were observed in the female genetic map than in the male genetic map, for the majority of the linkage groups (p < 0.01). The female genetic map was 1,036.7 cM longer than the male genetic map, with an average female-to-male ratio of 1.4:1. The ratio varied by linkage groups, ranging from 0.96 in LG6 to 2.06 in LG18 (Table 6). Large differences of recombination rate were also observed in LG2 and LG23 with the ratios of female to male greater than 1.6.

### Integration and validation with reference genome sequence

**Figure 5:**
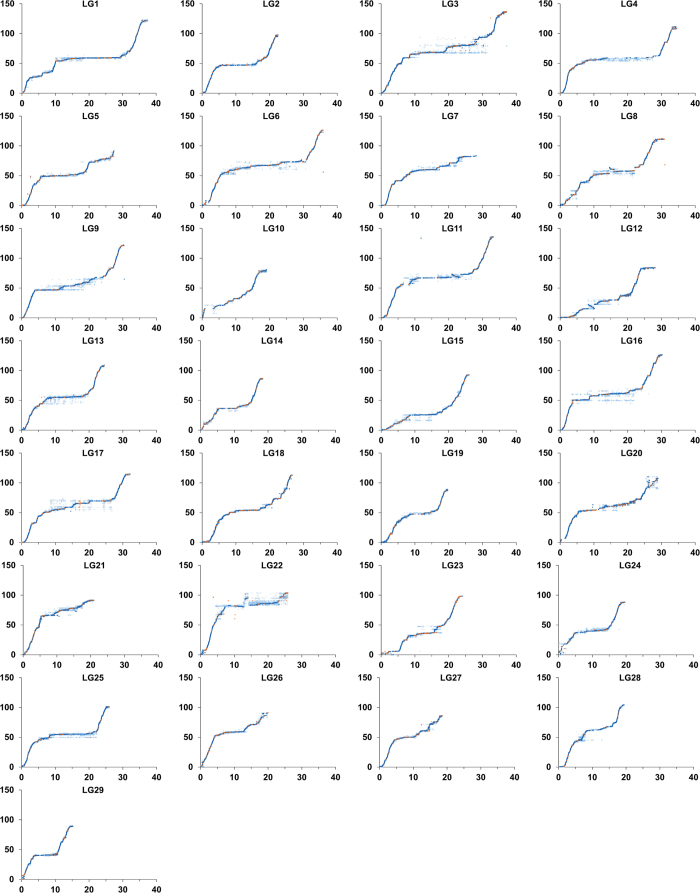
Concordance of SNP marker positions on reference sequence with those on genetic linkage map. The x-axis represents the physical position (Mb) of markers, the y-axis represents the genetic positions of markers on the linkage map (cM). Dark blue dots represent unique markers from previously mapped scaffolds; orange dots represent markers from previously unmapped genome or transcriptome sequences; markers in light blue circles were duplicated markers from previously mapped scaffolds.

The genetic map anchored 1,602 scaffolds of the reference genome sequence, corresponding to 766 Mb (97.8%) of the total 783 Mb. Comparing with the results from using the catfish 250 K SNP array, 1,007 previously unmapped scaffolds are anchored to chromosomes. However, these scaffolds are small in size, resulting in anchoring of only additional 5.7 Mb of the reference genome sequences to chromosomes. Additionally, 15 transcripts generated from de novo transcriptome assembly and 244 previously unmapped BAC-based physical contigs are also placed onto the linkage map. The positions of previously unmapped markers are illustrated in Fig. 5, making the whole genome reference sequence more ordered, continuous and connected.

**Figure 6:**
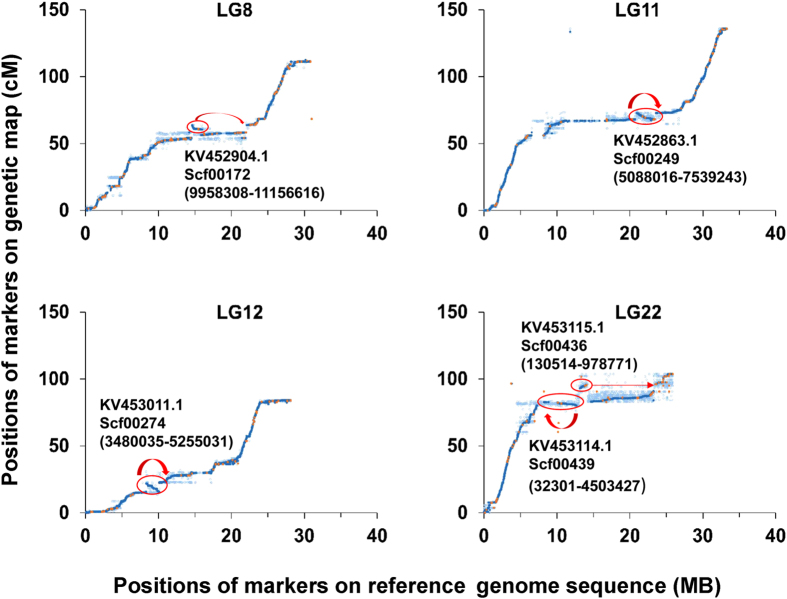
Genetic map suggested reorganization of scf00172 (LG8), scf00249 (LG11), scf00274 (LG12), scf00439 and scf00436 (LG22).

Mapping of over 250,000 SNPs across the genome validate the accuracy of the reference genome sequence assembly. As shown in Fig. 5, the marker orders from the linkage map and the reference genome are in concordance. A few differences of the marker orders between the linkage map and the reference genome sequences are observed in scf00172 (LG8), scf00249 (LG11), scf00274 (LG12), scf00439 and scf00436 (LG22) (Fig. 6), suggesting that these regions may be mis-assembled.

## Discussion

In this study, we developed the catfish 690 K SNP array using Affymetrix Axiom technology. Compared to the catfish 250 K SNP array^30^, the 690 K array has significantly more markers, more even genomic distribution, higher qualities of SNPs based on p-convert values, greater level of polymorphic contents, and greater level of coverage for the whole genome. The final set SNPs on the array consisted of channel catfish specific, blue catfish specific, and inter-species SNPs. A subset of strain-specific markers from domestic and wild channel catfish populations were also included on the array, which should be useful for detecting genomic regions with selective signatures. The 690 K SNP covered 24,186 of the 27,618 predicted genes in catfish, including 23,876 genes identified from the reference genome sequences and 310 genes predicted from RNA-seq datasets. The remaining genes were not covered because most of these genes have more than one copy on the genome; with duplicated genes, it is difficult to design reliable SNP probes. The selected SNPs were relatively evenly distributed on the reference genome sequence, which should benefit the detection of linkage disequilibrium in future genome-wide association studies and fine-scale QTL mapping. Distribution of MAF indicated that most of the SNPs included on the array were common variants (89.6% of SNPs had a MAF >0.1).

The evaluation of SNP array performance by genotyping samples from catfish backcross hybrid families and channel catfish families indicated that 72.7% of the SNPs were polymorphic in backcross hybrid catfish and 67.5% were polymorphic in channel catfish. Despite more channel catfish samples processed, higher percentages of SNPs were converted and polymorphic in backcross hybrids samples. This may be caused by the interspecific and blue-specific SNPs included on the array. Alleles from blue catfish could be detected as they segregated in the hybrid backcross genomes.

Taking advantage of the catfish 690 K SNP array, we constructed a genetic linkage map with super high density of markers for channel catfish. Not only the total number of mapped markers were increased from 50,000 to 250,000, the resolution of the map was also increased by using four reference families with a total of 465 individuals. However, because of the large number of markers and the limited recombination along the chromosomes, a large number of stacked markers were still observed. The only way to increase resolution power is to increase the sample sizes of the reference population (numbers of individuals and number of the reference families). While that can be done, the primary limitation is the cost. Due to the presence of genotyping errors and missing values, genetic mapping with these closely linked markers greatly increase the computation burden and usually introduce an overestimated genetic sizes. In our study, the total genetic distance and error estimate were dramatically reduced when we filtered the stacked SNPs. Genotyping errors in duplicate markers could also deform the marker orders, which can cause more severe problems compared with the inflation of genetic distances. To reduce the effect of clustered markers on the map construction and reduce the computing burden, we selected one representative marker to anchor the clusters. After the marker order was settled, the duplicated markers were then rejoined into the linkage group. This procedure rescued the informative markers onto the linkage maps based on the positions of their representative anchor marker. Such clustered markers were still valuable to organize the scaffolds to chromosomes using the reference genome sequences.

By integration of the genetic map with the reference genome sequences, the patterns of recombination across the whole chromosomes could be determined. As shown in Fig. 5, mild to strong localized specific recombination patterns were observed in each linkage group. Most often, the recombination rates were usually elevated towards the ends and decreased in the middle of the chromosome. Stacked markers that located in regions of strong linkage disequilibrium were observed in each linkage group, especially in regions close to the centromeres. The observed overall recombination patterns were in concordance with previous research in catfish^26^,^28^ and other aquaculture species such as tilapia^68^, medaka^69^, Atlantic salmon^70^, and rainbow trout^71^. Although the ultimate mechanism is still elusive, increasing evidence supports the hypothesis that multiple functional sequence motifs are involved in recombination regulation^72^. With the available of channel catfish reference genome, the next step of our study is to identify the sequence features within the recombination “hot zones”.

Higher recombination rate is observed in females than in males, which is consistent with previous studies in channel catfish. This phenomenon has also been observed in species with dissimilar sex chromosomes, with larger recombination rate in homogametic sex than in heterogametic sex. Some examples of this include mice^73^, zebrafish^74^, Atlantic salmon^35^, rainbow trout^71^, European seabass^75^, silver carp^76^, and grass carp^77^. One hypothesis toward sexual dimorphism in recombination fraction is that sex chromosomes in the homogametic sex are equal in size, therefore, recombination is more likely to occur. However, this does not seem to be true for channel catfish because the X and Y chromosomes are cytologically indistinguishable^78^. Interestingly, our results showed that recombination rate of LG4, which corresponds to the sex chromosome, is similar to that of other linkage groups, perhaps with the exception of the sex determination region. This suggests that other factors such as male-specific selection^79^ or chromatin differences may account for this difference^80^.

The high-density linkage map allowed validation and correction of the reference genome. In LG8, a 1.2 Mb stretch (from 9,958,308 bp to 11,156,616 bp) of scf00172 (NCBI accession KV452904.1) is supposed to be placed reversely between scf00174 and scf00637. In LG11, a 2.5 Mb subsequence (from 5,088,016 bp to 7,539,243 bp) of scf00249 (NCBI accession KV452863.1) is assumed to be assembled in the opposite direction. In LG12, a 1.8 Mb subsequence (from 3,480,035 bp to 5,255,031 bp) of scf00274 (NCBI accession KV453011.1) is presumed to be assembled in the opposite direction. It is also surmised that the whole sequence of scf00439 (NCBI accession KV453114.1) should be assembled reversely in LG22. Scf00436 (NCBI accession KV453115.1) is supposed to be placed between scf00437 (NCBI accession KV453116.1) and scf00435 (NCBI accession KV453121.1). By integrating the linkage map with the reference genome assembly, over 5.6 Mb from 1,007 previously unmapped scaffolds were successful anchored to their corresponding positions. Additionally, 15 transcripts generated from de novo transcriptome assembly, and 244 previously unmapped BAC-based physical contigs were also placed onto the linkage map. This is a significant improvement on integration of the linkage map and the reference genome sequence, which is useful for further genomic studies, QTL analysis, and whole genome-based selection.

## Materials and Methods

### Ethics statement

This study was approved by the Institutional Animal Care and Use Committee (IACUC) at Auburn University. All experiments involving the handling and treatment of fish were carried out in accordance with approved guidelines.

### SNP identification and SNP array development

In order to identify SNPs from the whole genome, Illumina sequencing data from various studies involving fish with diverse genetic background were collected. For channel catfish, a total of 3.3 billion reads from RNA-seq of 824 fish and 2.4 billion reads from whole genome sequencing of 150 fish were collected for SNP identification, with an average genome coverage of 500X (Table 1). For blue catfish, data used for SNP identification included over 359 million reads of GBS data from 190 individuals and 478 million reads of RNA-seq data from 49 individuals.

To reduce sequencing artifacts and improve the SNP quality, raw sequencing reads from all the studies were first subjected to quality control with Trimmomatic (version 0.33). Adaptor sequences, ambiguous nucleotides (N’s), extreme short reads (<25 bp) were removed. Low quality bases were identified and trimmed with a sliding window method, bases within a window size of 4 were cut once the average quality was less than 15. For reads generated from GBS, chimeric sequences were eliminated by trimming the sequence at the corresponding restriction enzyme site. The clean reads generated by WGS and GBS were then aligned to the reference genome sequence with BWA-MEM (version 0.7.12). To acquire high sensitivity and accuracy for RNA reads alignment, a 2-pass alignment method was performed using STAR aligner (version 2.4.0j). The results of the alignment were exported in BAM format for subsequent analysis^81^.

Prior to SNP identification, the BAM files were processed with Picard tools (version 1.119) to identify and remove redundant copies of duplicates. BAM files of WGS reads were subjected to local realignment of regions near INDELs with GATK (version 3.3) to improve the accuracy of variant calling. For BAM files of RNA-seq reads, “SplitNCigarReads” commands of GATK were used to split reads which spanned multiple exons and to trim overhangs. Ambiguously mapped reads were removed using SAMtools (version 0.1.19) under the criteria of a minimum MAPQ score of 20. For paired-end reads, only those mapped in a proper pair were kept for further analysis. Subsequently all alignment files were integrated for variant calling using Varscan (version 2.3.7). The putative SNPs were identified with the thresholds of minor allele frequency greater than 0.05, minimum read base quality of 20, strand-filter of 90%, and minimum read depth of 10. Sequences of 71-bp spanning each SNP were extracted, with 35-bp upstream and 35-bp downstream of the SNP base, respectively. To avoid false positive SNPs caused by ambiguous mapping of duplicated regions, the 71-bp fragments were aligned to the reference genome sequence with BLAST + (version 2.2.29). SNPs with flanking regions that mapped to multiple sites or low complexity and repetitive regions on the reference genome sequence were removed.

SNP selection was performed in multiple steps using different criteria regarding different SNP types. All the filtration parameters were set with the aims of achieving an evenly spaced coverage across the entire genome and removal of false positive sites. All the original SNPs were classified into different groups and selected in a certain order: SNPs within genes were first selected, then SNPs from intergenic regions were added, finally, species-specific SNPs and strain-specific SNPs were included in the pool of candidate SNPs. Custom scripts were used to perform the selection according to the following criteria: (1) To avoid non-specific hybridization, the flanking sequences of selected SNPs should not have other SNPs or simple nucleotide repeats; (2) For practical application in SNP genotyping assays, only bi-allelic SNPs were selected; (3) To acquire high-polymorphic rate, SNPs with a minor allele frequency greater than 0.1 were preferentially selected; (4) To acquire a high-capacity, A/T or C/G SNPs were not selected unless absolutely needed; (5) To minimize the effects of GC content on the signal intensity, the GC percentage of the SNPs flanking sequences should be between 30–70%. For SNPs within genes, once a new SNP was included into the candidate pool, SNPs from the flanking regions of 200 bp were not added. For SNPs from intergenic regions, once a new SNP was included into the candidate pool, SNPs from the flanking regions of 350 bp were not added. SNPs from unmapped scaffolds and contigs apart from reference genome sequences were also included regardless of their distances.

All catfish SNPs that passed this filter were submitted to Affymetrix Bioinformatics Services for *in-silico* probe converting test, where the performing quality of the SNPs were evaluated. Upstream and downstream probes flanking the SNPs were assigned with a p-convert value (0.0 to 1.0), respectively. Probes with high p-convert values were more likely to be successfully genotyped. A threshold for p-convert value was set to remove the lowest performing probes to facilitate selection of a high-quality SNP list. Markers with at least one probe that passed the p-convert value threshold were retained. For SNP markers with both of the probes that passed the p-convert value threshold, the probe with greater p-convert value was selected. For SNPs with only probes of low p-convert values, both probes were included to cover the genome region.

In addition to the polymorphic SNPs, 2,000 probes generated from non-polymorphic genomic regions were also introduced as QC probes of which 1,000 probes were selected with A or T at the 31st base, and 1,000 QC probes were selected with G or C at the 31th base. The QC probes along with the final list of SNPs were submitted to Affymetrix for fabrication of Axiom GW genotyping array.

### SNP array performance evaluation

A total of 480 catfish were genotyped to assess the performance of SNP array, including 396 purebred channel catfish of the Delta Select line provided by USDA-ARS Warmwater Aquaculture Research Unit, and 84 hybrids generated by backcrossing channel x blue interspecific hybrid females with male channel catfish.

DNA samples were prepared following the procedures as previous described^27^. DNA samples were diluted to 50 ng/μl and genotyped with the catfish 690 K SNP array (GeneSeek, Lincoln, Nebraska, USA). The signal intensity data of each probe on the array were reported in CEL files, which were analyzed with Axiom Analysis Suite (version 1.1.0.616) for quality control and genotype calling. Samples with a Dish value greater than 0.85 and SNP call rates greater than 95% were retained for subsequent analysis.

Following the genotyping step, SNPolisher (Affymetrix) generated quality metrics and classified all the SNPs into six types. Briefly, “PolyHighResolution” indicate that both of the two alleles of a SNP were detected. The signal data of all the samples also formed into three distinct clusters with good resolution; “NoMinorHom” indicate SNPs with two clusters of signal data, with no example of the minor homozygous genotypes; “MonoHighResolution” include SNPs with only one clusters identified. “OTV” refers to off-target variants, indicating SNPs with an OTV cluster that caused by sequence dissimilarity between probes and target genome regions^82^. “CallRateBelowThreshold” were the SNPs with call rates below threshold, but other cluster properties were above threshold. “Other” were the SNPs with one or more cluster properties below the threshold. In most cases, SNPs classified as “PolyHighResolution”, “NoMinorHom”, “MonoHighResolution”, were considered as converted SNPs. SNPs classified as “PolyHighResolution” and “NoMinorHom” were considered as polymorphic SNPs.

### Linkage map construction

A total of 478 individuals from four full-sib families of the Delta select strain of channel catfish were used for linkage mapping. SNPs classified as “PolyHighResolution” and “NoMinorHom” were retained for further analysis. The genotyping results were exported in the pre-MAKEPED LINKAGE pedigree format^83^.

The genotyping data were imported into Plink (version 1.9) to test pedigree information. A complete linkage agglomerative clustering procedure based on pairwise identity-by-state (IBS) distance were carried out. Multidimensional scaling analysis was performed on the generated IBS pairwise distances matrix. The pedigree information of outliers was identified and checked by subsequent outlier detection diagnostics, where a Z score was assigned to measure the distance between the outliers with the rest of the samples. Outlier samples were discarded once they were detected with significantly larger distances compared with the normal level.

Linkage map was constructed using Lep-MAP2 (version 0.2)^84^. First, the Filtering module was executed to filtering low quality and un-informative markers. Markers with missing values larger than 12 (about 10%) or MAF less than 6 (about 5%) in each family were discarded. Only markers with 2 or more informative families were retained. A segregation distortion test was also performed to compare the offspring genotype distribution and the expected Mendelian proportions. Markers with significant segregation distortion were eliminated from linkage analysis (χ^2^ test, *P* < 0.005). SNP Markers were assigned to linkage groups (LGs) using the SeparateChromosomes module. LGs were formed according to the threshold of logarithm of the odds (LOD) score limit of 35 and minimum LG size of 10. Singular markers were then added to the established LGs using the JoinSingles module with an LOD score limit of 10 and a minimum difference of 3 between the best LG and the second best LG of each joined marker.

Marker order of each LGs was determined by allowing different recombination probabilities in both sexes. Genotyping data of markers from the four families were analyzed simultaneously. Two-rounds of marker ordering procedures were carried out for better performance. In the first round, ten iterations were performed to acquire the best order of markers. To reduce the computational burden, a missing rate of 5% was set when determining whether two markers were duplicates. As the number of markers may beyond the resolution of recombination, there were many markers stacking up in the same locus on the genetic map, which may lead to recombination rate deformities. Therefore, the stacked markers as well as the duplicated markers were clustered and filtered. The marker with the most informative meiosis of one cluster was selected as the representative marker and retained for a new round of marker ordering. After the second round of ordering, all the previously identified duplicates and stacked markers were added to the adjacent locus and included for genetic distance calculation with Kosambi mapping function by taking account both male and female meiosis. Sex-specific recombination rates were then calculated with the same marker order. MapChart (version 2.3) was used to graphically present the genetic linkage map.

## Additional Information

**How to cite this article**: Zeng, Q. *et al*. Development of a 690 K SNP array in catfish and its application for genetic mapping and validation of the reference genome sequence. *Sci. Rep.* **7**, 40347; doi: 10.1038/srep40347 (2017).

**Publisher's note:** Springer Nature remains neutral with regard to jurisdictional claims in published maps and institutional affiliations.

## Supplementary Material

Supplementary Information

## Figures and Tables

**Table 1:** Sample and data size for development of 690 K SNP array.

Project	Species	Sample size	Data type	Data accession	Reads (x10^6^)
Gene-associated SNP mining	Channel, blue catfish	66	RNA-seq	SRA025099	506
Studies on catfish domestication	Channel catfish	150	WGS	SRX275877	2,347
Gonad development	Channel catfish	279	RNA-seq	SRP018265, SRP067841, Unpublished	1,185
Barbel regeneration	Channel catfish	90	RNA-seq	SRX1003145	395
Skin regeneration	Channel catfish	10	RNA-seq	SRX1003286	193
ESC chanllenge	Channel catfish	180	RNA-seq	SRP009069	197
Columnaris challenge	Channel catfish	218	RNA-seq	SRP017689, SRP012586	1,108
Feed deprivation	Blue catfish	30	RNA-seq	SRP020252	194
SNP mining with GBS	Blue catfish	190	GBS	SRP039640	359
Total	−	1,213	−	−	6,484

**Table 2:** Summary of SNPs identified in channel and blue catfish.

	Channel catfish							
	Thompson	Hatchery	Marion	USDA103	Wild	Subtotal	Blue catfish	Total
Number of putative SNPs	5,723,886	4,270,527	4,817,089	4,900,634	5,488,642	8,644,975	3,850,662	9,662,958
Number of uniquely mapped SNPs	3,992,954	2,861,190	3,283,718	3,345,046	3,852,203	5,667,615	3,138,197	6,535,576
Number of SNPs without adjacent SNPs in 35 bp	1,487,079	1,032,160	1,184,548	1,225,949	1,442,459	2,270,690	347,958	2,512,422
Number of SNPs passed flanking sequence complexity inspection	1,455,201	1,009,026	1,155,782	1,196,716	1,414,789	2,199,952	340,921	2,435,602
Number of bi-allelic SNPs	1,443,954	1,000,560	1,146,365	1,187,187	1,403,727	2,184,946	332,934	2,418,168
Number of SNPs passed Affymetrix *in-silico* test	1,224,483	846,622	969,303	1,004,560	1,191,020	1,839,478	294,593	2,046,175

**Table 3:** Summary of the catfish 690 K SNP array.

SNP array	Number
Number of genic SNPs	238,484
Number of intergenic SNPs	452,178
Number of SNPs tiled with single probe	687,757
Number of SNPs tiled with two probes	2,905
Channel catfish-specific SNPs	581,002
Blue catfish-specific SNPs	44,694
Inter-species SNPs	19,124
Channel-blue both possessed	45,842
Strain-specific SNPs	48,434
Domesticate strain (Thompson)	12,672
Domesticate strain (Hatchery)	9,498
Domesticate strain (Marion)	10,309
Domesticate strain (USDA 103)	9,333
Wild population	6,622
Total number of SNPs on array	690,662

**Table 4:** SNP metrics summary.

	Backcross hybrids	Channel catfish	Total
Samples processed	84	396	480
Samples passed QC	81 (96.4%)	392 (98.9%)	473 (98.5%)
PolyHighResolution	292,185 (42.1%)	326,411 (47.1%)	401,815 (57.9%)
NoMinorHom	211,980 (30.6%)	141,410 (20.4%)	133,803 (19.3%)
Total Polymorphic SNPs	504,265 (72.7%)	467,821 (67.5%)	535,618 (77.2%)
MonoHighResolution	93,058 (13.4%)	111,047 (16.0%)	62,262 (8.9%)
Total converted SNPs	597,323 (86.1%)	578,868 (83.5%)	597,880 (86.1%)

**Table 5:** Summary of the sex-average linkage map of channel catfish.

Linkage group	Chromosome	Sex-average map			
		Mapped markers	Unique positions	Genetic length (cM)	Marker interval
1	1	13,426	1,198	122.48	0.1
2	20	6,626	1,031	97.624	0.09
3	2	12,838	1,340	136.613	0.1
4	4	7,754	1,090	111.502	0.1
5	13	9,869	1,017	91.712	0.09
6	3	11,507	1,312	126.625	0.1
7	11	8,000	832	83.681	0.1
8	7	10,999	1,216	112.518	0.09
9	8	8,516	1,267	122.109	0.1
10	26	6,138	850	80.721	0.09
11	5	9,256	1,364	135.765	0.1
12	12	8,264	1,010	84.4	0.08
13	18	9,330	1,286	109.138	0.08
14	28	6,728	899	86.76	0.1
15	15	7,672	999	92.755	0.09
16	9	9,293	1,180	126.838	0.11
17	6	10,052	1,286	114.924	0.09
18	14	13,475	1,226	113.228	0.09
19	23	7,429	1,038	89.178	0.09
20	10	10,797	1,050	110.144	0.1
21	21	10,074	1,021	91.477	0.09
22	17	5,628	771	103.989	0.13
23	19	10,492	995	98.423	0.1
24	24	5,361	778	88.577	0.11
25	16	7,873	994	101.438	0.1
26	22	7,161	863	91.43	0.11
27	27	7,907	864	86.565	0.1
28	25	6,084	1,068	104.627	0.1
29	29	4,538	746	89.494	0.12
Total		253,087	30,591	3,004.735	0.1

**Table 6:** Summary of the sex-specific linkage map of channel catfish.

Linkage group	Female-specific map			Male-specific map			F:M ratio
	Unique positions	Genetic length (cM)	Marker interval (cM)	Unique positions	Genetic length (cM)	Marker interval (cM)	
1	880	150.398	0.17	764	96.932	0.13	1.55
2	914	128.927	0.14	576	80.311	0.14	1.61
3	1,005	167.41	0.17	797	107.838	0.14	1.55
4	798	133.264	0.17	608	94.902	0.16	1.4
5	797	107.165	0.13	564	76.823	0.14	1.39
6	1,020	147.84	0.14	1,088	154.592	0.14	0.96
7	593	98.666	0.17	515	69.364	0.13	1.42
8	951	139.04	0.15	642	89.167	0.14	1.56
9	1,006	150.924	0.15	754	97.856	0.13	1.54
10	643	94.239	0.15	498	72.248	0.15	1.3
11	1,012	145.177	0.14	883	127.862	0.14	1.14
12	718	97.291	0.14	640	72.927	0.11	1.33
13	975	123.13	0.13	663	95.595	0.14	1.29
14	690	102.484	0.15	517	72.634	0.14	1.41
15	754	112.926	0.15	607	73.845	0.12	1.53
16	939	154.868	0.16	639	101.333	0.16	1.53
17	924	134.912	0.15	760	96.545	0.13	1.4
18	1,030	152.931	0.15	610	74.318	0.12	2.06
19	814	103.42	0.13	552	75.193	0.14	1.38
20	773	134.035	0.17	592	84.287	0.14	1.59
21	753	94.889	0.13	612	89.754	0.15	1.06
22	700	128.097	0.18	502	87.469	0.17	1.46
23	808	126.172	0.16	578	75.567	0.13	1.67
24	632	104.576	0.17	462	72.957	0.16	1.43
25	693	113.383	0.16	662	89.252	0.13	1.27
26	637	104.682	0.16	504	78.919	0.16	1.33
27	777	108.956	0.14	528	68.471	0.13	1.59
28	755	112.341	0.15	697	98.77	0.14	1.14
29	619	110.117	0.18	525	69.86	0.13	1.58
Total	23,610	3,582.26	0.15	18,339	2,545.591	0.14	1.41
