# Supplementary material for: Development of a 690 K SNP array in catfish and its application for genetic mapping and validation of the reference genome sequence
Source: Sci Rep. 2017 Jan 12;7:40347. doi: 10.1038/srep40347 (PMC5228154; doi:10.1038/srep40347)
Supplement: Supplementary Information [file srep40347-s1.pdf]

Development of a 690K SNP array in catfish and its application for genetic mapping and validation of the reference genome sequence

Qifan Zeng[1], Qiang Fu[1], Yun Li[1], Geoff Waldbieser[2], Brian Bosworth[2], Shikai Liu[1], Yujia Yang[1], Lisui Bao[1], Zihao Yuan[1], Ning Li[1], and Zhanjiang Liu[1*]

[1]The Fish Molecular Genetics and Biotechnology Laboratory, Aquatic Genomics Unit, School of Fisheries, Aquaculture and Aquatic Sciences, and Program of Cell and Molecular Biosciences, Auburn, Alabama 36832, United States of America

[2]USDA-ARS Warmwater Aquaculture Research Unit, Stoneville, Mississippi 38776, United States of America

[*]Correspondence: E-mail: liuzhan@auburn.edu

Supplemental Figure 1. Illustration of female-specific linkage map.

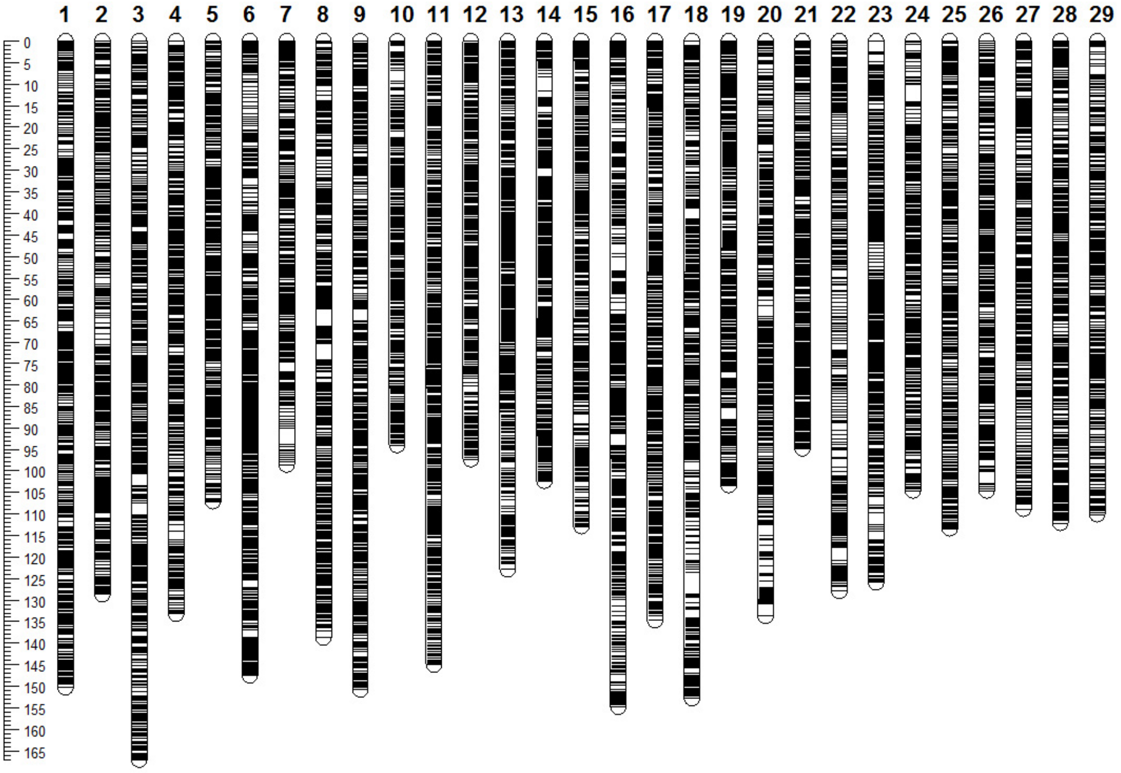

Supplemental Figure 2. Illustration of male-specific linkage map.

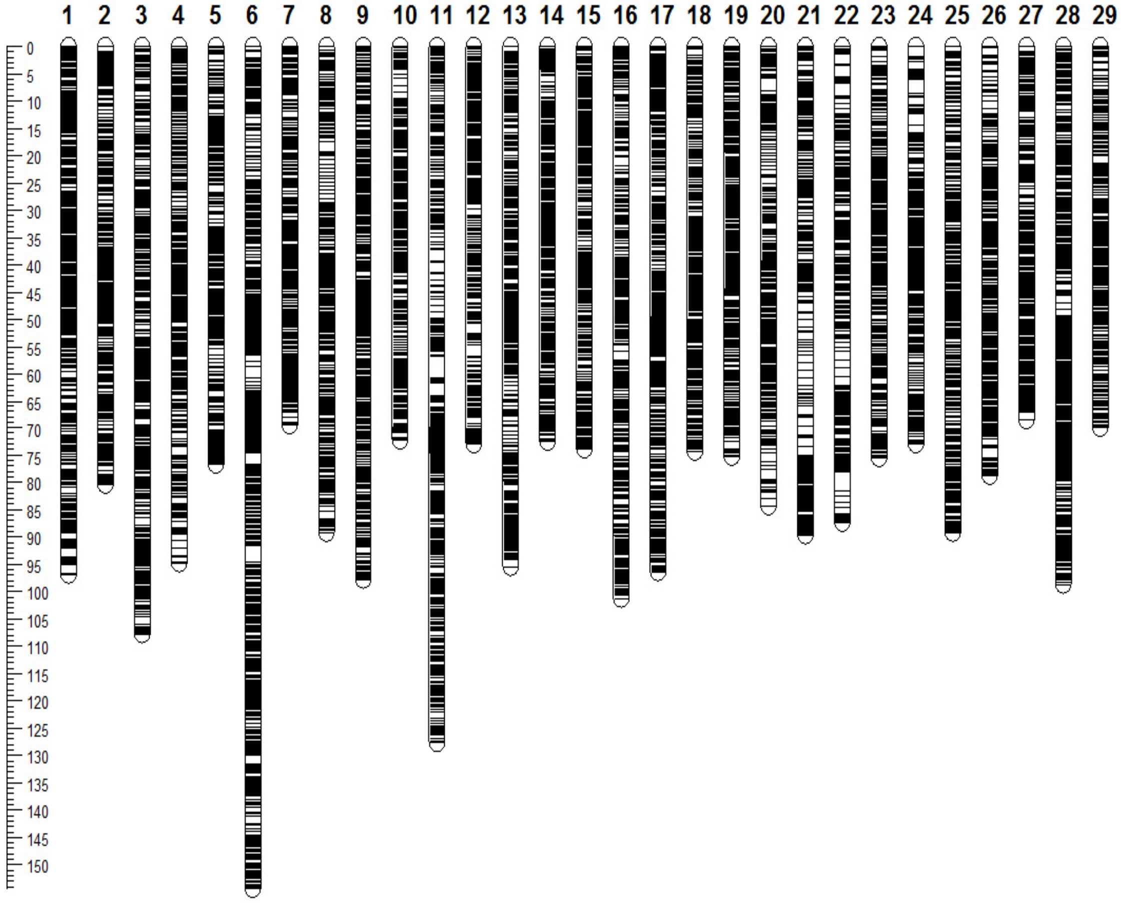

Supplemental Table S1.

Marker	Linkage group	Genetic position	Accession	Marker Position on Scaffold	Scaffold	Chrosome
AX-157889335	1	0	KV452696.1	768	1-scf00001	1
AX-157810099	1	0	KV452696.1	13675	1-scf00001	1
AX-157889348	1	0	KV452696.1	19009	1-scf00001	1
AX-157889378	1	0	KV452696.1	94512	1-scf00001	1
AX-157810111	1	0	KV452696.1	194391	1-scf00001	1
AX-157889490	1	0	KV452696.1	255063	1-scf00001	1
AX-157890091	1	0	KV452696.1	1222381	1-scf00001	1
AX-157890144	1	0	KV452696.1	1304163	1-scf00001	1
AX-158376989	1	0	KV452695.1	6200	1-scf00598	1
AX-157709104	1	0	KV452695.1	8650	1-scf00598	1
AX-158376990	1	0	KV452695.1	9182	1-scf00598	1
AX-157853360	1	0	KV452695.1	12432	1-scf00598	1
AX-157720046	1	0	KV452695.1	16327	1-scf00598	1
AX-158376996	1	0	KV452695.1	24090	1-scf00598	1
AX-158376998	1	0	KV452695.1	33689	1-scf00598	1
AX-158377000	1	0	KV452695.1	56502	1-scf00598	1
AX-157772461	1	0	KV452695.1	68810	1-scf00598	1
AX-158377004	1	0	KV452695.1	74627	1-scf00598	1
AX-157889322	1	0	KV452695.1	79314	1-scf00598	1
AX-158424003	1	0	KV452695.1	81171	1-scf00598	1
AX-157853364	1	0	KV452695.1	92819	1-scf00598	1
AX-158377006	1	0	KV452695.1	93302	1-scf00598	1
AX-158377007	1	0	KV452695.1	95855	1-scf00598	1
AX-158377008	1	0	KV452695.1	97051	1-scf00598	1
AX-158379063	1	0	KV452695.1	97725	1-scf00598	1
AX-157751599	1	0	KV452695.1	150749	1-scf00598	1
AX-157723901	1	0	KV452695.1	158105	1-scf00598	1
AX-158377036	1	0	KV452695.1	170155	1-scf00598	1
AX-158377042	1	0	KV452695.1	180739	1-scf00598	1
AX-158377052	1	0	KV452695.1	198222	1-scf00598	1
AX-158377053	1	0	KV452695.1	198977	1-scf00598	1
AX-157687664	1	0	KV452694.1	686	1-scf00962	1
AX-157680241	1	0	KV452694.1	2193	1-scf00962	1
AX-157687668	1	0	KV452694.1	7464	1-scf00962	1
AX-157680131	1	0	3ML01024458	31133	scf00715_1	UN
AX-158377012	1	0.074	KV452695.1	101726	1-scf00598	1
AX-157758848	1	0.126	KV452696.1	101253	1-scf00001	1
AX-158377014	1	0.126	KV452695.1	105509	1-scf00598	1
AX-157853366	1	0.126	KV452695.1	105695	1-scf00598	1
AX-158377016	1	0.126	KV452695.1	107887	1-scf00598	1
AX-157853367	1	0.126	KV452695.1	115316	1-scf00598	1
AX-158377020	1	0.126	KV452695.1	119746	1-scf00598	1
AX-157706029	1	0.126	3ML01031462	233	scf07075_1	UN
AX-157889337	1	0.252	KV452696.1	2443	1-scf00001	1
AX-157810098	1	0.252	KV452696.1	10510	1-scf00001	1

AX-157889344	1	0.252	KV452696.1	14506	1-scf00001	1
AX-157889346	1	0.252	KV452696.1	17276	1-scf00001	1
AX-157889351	1	0.252	KV452696.1	22454	1-scf00001	1
AX-157889352	1	0.252	KV452696.1	22608	1-scf00001	1
AX-157889353	1	0.252	KV452696.1	24201	1-scf00001	1
AX-158377011	1	0.252	KV452695.1	99372	1-scf00598	1
AX-158377029	1	0.252	KV452695.1	157313	1-scf00598	1
AX-157725922	1	0.252	KV452695.1	182840	1-scf00598	1
AX-158377049	1	0.252	KV452695.1	195530	1-scf00598	1
AX-157889369	1	0.379	KV452696.1	78184	1-scf00001	1
AX-157889370	1	0.379	KV452696.1	79159	1-scf00001	1
AX-157889371	1	0.379	KV452696.1	79440	1-scf00001	1
AX-157889373	1	0.379	KV452696.1	81452	1-scf00001	1
AX-157889377	1	0.379	KV452696.1	93960	1-scf00001	1
AX-157889381	1	0.379	KV452696.1	100522	1-scf00001	1
AX-157889388	1	0.379	KV452696.1	107998	1-scf00001	1
AX-157715345	1	0.379	KV452696.1	108410	1-scf00001	1
AX-157889390	1	0.379	KV452696.1	111068	1-scf00001	1
AX-157889409	1	0.379	KV452696.1	131675	1-scf00001	1
AX-157889410	1	0.379	KV452696.1	132058	1-scf00001	1
AX-157889389	1	0.484	KV452696.1	109156	1-scf00001	1
AX-157810106	1	0.597	KV452696.1	112210	1-scf00001	1
AX-157810108	1	0.597	KV452696.1	114828	1-scf00001	1
AX-157810109	1	0.597	KV452696.1	115061	1-scf00001	1
AX-157874194	1	0.597	KV452696.1	115177	1-scf00001	1
AX-157889393	1	0.597	KV452696.1	115406	1-scf00001	1
AX-157889395	1	0.597	KV452696.1	116775	1-scf00001	1
AX-157889398	1	0.597	KV452696.1	120550	1-scf00001	1
AX-157889400	1	0.597	KV452696.1	121327	1-scf00001	1
AX-157810110	1	0.597	KV452696.1	122791	1-scf00001	1
AX-157889403	1	0.597	KV452696.1	123879	1-scf00001	1
AX-157889404	1	0.597	KV452696.1	124921	1-scf00001	1
AX-157889412	1	0.597	KV452696.1	134343	1-scf00001	1
AX-157889413	1	0.597	KV452696.1	135161	1-scf00001	1
AX-157889414	1	0.597	KV452696.1	136370	1-scf00001	1
AX-157719822	1	0.597	KV452696.1	138643	1-scf00001	1
AX-158377002	1	0.597	KV452695.1	66859	1-scf00598	1
AX-158377027	1	0.597	KV452695.1	149611	1-scf00598	1
AX-158377032	1	0.597	KV452695.1	162389	1-scf00598	1
AX-157853371	1	0.597	KV452695.1	162875	1-scf00598	1
AX-157687665	1	0.597	KV452694.1	1503	1-scf00962	1
AX-157889418	1	0.707	KV452696.1	145286	1-scf00001	1
AX-157889419	1	0.707	KV452696.1	145492	1-scf00001	1
AX-157889421	1	0.707	KV452696.1	147400	1-scf00001	1
AX-157889422	1	0.707	KV452696.1	147766	1-scf00001	1
AX-157681017	1	0.707	KV452694.1	7771	1-scf00962	1
AX-157889424	1	0.708	KV452696.1	150436	1-scf00001	1
AX-157889427	1	1.112	KV452696.1	155625	1-scf00001	1
AX-157889429	1	1.112	KV452696.1	160866	1-scf00001	1
AX-157889430	1	1.113	KV452696.1	161691	1-scf00001	1
AX-157889432	1	1.115	KV452696.1	164763	1-scf00001	1

AX-157889433	1	1.116	KV452696.1	165520	1-scf00001	1
AX-157745518	1	1.118	KV452696.1	165747	1-scf00001	1
AX-157889436	1	1.119	KV452696.1	169660	1-scf00001	1
AX-157714401	1	1.119	KV452696.1	170217	1-scf00001	1
AX-157889437	1	1.12	KV452696.1	170650	1-scf00001	1
AX-157889345	1	1.311	KV452696.1	15068	1-scf00001	1
AX-157714644	1	1.311	KV452696.1	113213	1-scf00001	1
AX-157889391	1	1.311	KV452696.1	113880	1-scf00001	1
AX-157889402	1	1.311	KV452696.1	122502	1-scf00001	1
AX-157889438	1	1.311	KV452696.1	174265	1-scf00001	1
AX-157889439	1	1.311	KV452696.1	175420	1-scf00001	1
AX-157889442	1	1.311	KV452696.1	180968	1-scf00001	1
AX-157889492	1	1.311	KV452696.1	264313	1-scf00001	1
AX-158376991	1	1.311	KV452695.1	10948	1-scf00598	1
AX-157706556	1	1.311	3ML01032450	520	scf08071_1	UN
AX-157706555	1	1.35	3ML01032450	377	scf08071_1	UN
AX-157868440	1	1.409	KV452696.1	181217	1-scf00001	1
AX-157715146	1	1.409	KV452696.1	191389	1-scf00001	1
AX-157889444	1	1.471	KV452696.1	185961	1-scf00001	1
AX-157889445	1	1.471	KV452696.1	189173	1-scf00001	1
AX-157889446	1	1.471	KV452696.1	189478	1-scf00001	1
AX-157889447	1	1.471	KV452696.1	190487	1-scf00001	1
AX-157889448	1	1.471	KV452696.1	191500	1-scf00001	1
AX-157889452	1	1.471	KV452696.1	199229	1-scf00001	1
AX-157770650	1	1.471	KV452696.1	211881	1-scf00001	1
AX-157889456	1	1.525	KV452696.1	205397	1-scf00001	1
AX-157889458	1	1.581	KV452696.1	209111	1-scf00001	1
AX-157889460	1	1.581	KV452696.1	210774	1-scf00001	1
AX-157810112	1	1.581	KV452696.1	222686	1-scf00001	1
AX-157746184	1	1.636	KV452696.1	214757	1-scf00001	1
AX-157889463	1	1.689	KV452696.1	216181	1-scf00001	1
AX-157889464	1	1.745	KV452696.1	219261	1-scf00001	1
AX-157751316	1	1.814	KV452696.1	225190	1-scf00001	1
AX-157889472	1	1.859	KV452696.1	235803	1-scf00001	1
AX-158403602	1	1.909	KV452696.1	5245	1-scf00001	1
AX-157889340	1	1.909	KV452696.1	6405	1-scf00001	1
AX-157742990	1	1.909	KV452696.1	76830	1-scf00001	1
AX-157889368	1	1.909	KV452696.1	77144	1-scf00001	1
AX-157889372	1	1.909	KV452696.1	81171	1-scf00001	1
AX-157889379	1	1.909	KV452696.1	99163	1-scf00001	1
AX-157889397	1	1.909	KV452696.1	118152	1-scf00001	1
AX-157756124	1	1.909	KV452696.1	133715	1-scf00001	1
AX-157889416	1	1.909	KV452696.1	139038	1-scf00001	1
AX-157868439	1	1.909	KV452696.1	149537	1-scf00001	1
AX-157889435	1	1.909	KV452696.1	167913	1-scf00001	1
AX-157889462	1	1.909	KV452696.1	215470	1-scf00001	1
AX-157889469	1	1.909	KV452696.1	233879	1-scf00001	1
AX-157889473	1	1.909	KV452696.1	236107	1-scf00001	1
AX-157889476	1	1.909	KV452696.1	238305	1-scf00001	1
AX-157810113	1	1.909	KV452696.1	239495	1-scf00001	1
AX-157889477	1	1.909	KV452696.1	239723	1-scf00001	1

AX-157889479	1	1.909	KV452696.1	240351	1-scf00001	1
AX-157889480	1	1.909	KV452696.1	240516	1-scf00001	1
AX-157889481	1	1.909	KV452696.1	241204	1-scf00001	1
AX-157889486	1	1.909	KV452696.1	247281	1-scf00001	1
AX-157810115	1	1.909	KV452696.1	249165	1-scf00001	1
AX-157745729	1	1.909	KV452696.1	249258	1-scf00001	1
AX-157810116	1	1.909	KV452696.1	250033	1-scf00001	1
AX-158378693	1	1.909	KV452696.1	360598	1-scf00001	1
AX-158377003	1	1.909	KV452695.1	68321	1-scf00598	1
AX-158420677	1	1.909	KV452695.1	80955	1-scf00598	1
AX-158377013	1	1.909	KV452695.1	103475	1-scf00598	1
AX-158379064	1	1.909	KV452695.1	117412	1-scf00598	1
AX-157853372	1	1.909	KV452695.1	178837	1-scf00598	1
AX-157689695	1	1.909	3ML01031408	287	scf07020_1	UN
AX-157889488	1	2.018	KV452696.1	252160	1-scf00001	1
AX-157889493	1	2.106	KV452696.1	264442	1-scf00001	1
AX-157889494	1	2.182	KV452696.1	265169	1-scf00001	1
AX-157889499	1	2.182	KV452696.1	267912	1-scf00001	1
AX-157889497	1	2.238	KV452696.1	266584	1-scf00001	1
AX-157889455	1	2.283	KV452696.1	203604	1-scf00001	1
AX-157889468	1	2.283	KV452696.1	232253	1-scf00001	1
AX-158379081	1	2.283	KV452696.1	263874	1-scf00001	1
AX-157710376	1	2.283	KV452696.1	270140	1-scf00001	1
AX-157889505	1	2.306	KV452696.1	271730	1-scf00001	1
AX-157889496	1	2.316	KV452696.1	266076	1-scf00001	1
AX-158381854	1	2.316	KV452696.1	266835	1-scf00001	1
AX-157889500	1	2.316	KV452696.1	268282	1-scf00001	1
AX-157889501	1	2.316	KV452696.1	269125	1-scf00001	1
AX-157889504	1	2.316	KV452696.1	271144	1-scf00001	1
AX-157756012	1	2.316	KV452696.1	273884	1-scf00001	1
AX-157889516	1	2.316	KV452696.1	286337	1-scf00001	1
AX-157719435	1	2.346	KV452696.1	263786	1-scf00001	1
AX-157889498	1	2.346	KV452696.1	267087	1-scf00001	1
AX-157889502	1	2.346	KV452696.1	269637	1-scf00001	1
AX-157889509	1	2.346	KV452696.1	279852	1-scf00001	1
AX-157889510	1	2.346	KV452696.1	281131	1-scf00001	1
AX-157889511	1	2.346	KV452696.1	282450	1-scf00001	1
AX-157889514	1	2.346	KV452696.1	285532	1-scf00001	1
AX-157889519	1	2.564	KV452696.1	288656	1-scf00001	1
AX-157889521	1	2.564	KV452696.1	293995	1-scf00001	1
AX-157889524	1	2.564	KV452696.1	295931	1-scf00001	1
AX-157746161	1	2.783	KV452696.1	259783	1-scf00001	1
AX-157889523	1	2.783	KV452696.1	295463	1-scf00001	1
AX-157889526	1	2.783	KV452696.1	297790	1-scf00001	1
AX-157889529	1	2.783	KV452696.1	302394	1-scf00001	1
AX-157874198	1	2.783	KV452696.1	306243	1-scf00001	1
AX-157889533	1	2.783	KV452696.1	308400	1-scf00001	1
AX-157771493	1	2.783	KV452696.1	309599	1-scf00001	1
AX-157889534	1	2.783	KV452696.1	313075	1-scf00001	1
AX-157751748	1	2.783	KV452696.1	314204	1-scf00001	1
AX-157889536	1	2.783	KV452696.1	314924	1-scf00001	1

AX-157889537	1	2.783	KV452696.1	316300	1-scf00001	1
AX-157889538	1	2.783	KV452696.1	316710	1-scf00001	1
AX-157727150	1	2.783	KV452696.1	317877	1-scf00001	1
AX-157889540	1	2.783	KV452696.1	319284	1-scf00001	1
AX-158404104	1	2.783	KV452696.1	322627	1-scf00001	1
AX-157889541	1	2.783	KV452696.1	322996	1-scf00001	1
AX-157889545	1	2.783	KV452696.1	325795	1-scf00001	1
AX-157889547	1	2.783	KV452696.1	328467	1-scf00001	1
AX-157889548	1	2.783	KV452696.1	330832	1-scf00001	1
AX-157810119	1	2.783	KV452696.1	331639	1-scf00001	1
AX-157810120	1	2.783	KV452696.1	333217	1-scf00001	1
AX-157889552	1	2.783	KV452696.1	334243	1-scf00001	1
AX-157889553	1	2.783	KV452696.1	334727	1-scf00001	1
AX-157889554	1	2.783	KV452696.1	336961	1-scf00001	1
AX-157889555	1	2.783	KV452696.1	338735	1-scf00001	1
AX-157889556	1	2.783	KV452696.1	338844	1-scf00001	1
AX-157810121	1	2.783	KV452696.1	342183	1-scf00001	1
AX-157810122	1	2.783	KV452696.1	342361	1-scf00001	1
AX-157889561	1	2.783	KV452696.1	342450	1-scf00001	1
AX-157889563	1	2.783	KV452696.1	345925	1-scf00001	1
AX-157889566	1	2.783	KV452696.1	357829	1-scf00001	1
AX-157889567	1	2.783	KV452696.1	360869	1-scf00001	1
AX-157889571	1	2.783	KV452696.1	368841	1-scf00001	1
AX-157889573	1	2.783	KV452696.1	372306	1-scf00001	1
AX-157889588	1	2.783	KV452696.1	396406	1-scf00001	1
AX-157889565	1	3.11	KV452696.1	355814	1-scf00001	1
AX-157889581	1	3.11	KV452696.1	385208	1-scf00001	1
AX-157889583	1	3.11	KV452696.1	389120	1-scf00001	1
AX-157810125	1	3.11	KV452696.1	389521	1-scf00001	1
AX-157874201	1	3.11	KV452696.1	390488	1-scf00001	1
AX-157889584	1	3.11	KV452696.1	391208	1-scf00001	1
AX-157754048	1	3.11	KV452696.1	391338	1-scf00001	1
AX-157889585	1	3.11	KV452696.1	392318	1-scf00001	1
AX-157889586	1	3.11	KV452696.1	393053	1-scf00001	1
AX-157889589	1	3.11	KV452696.1	396769	1-scf00001	1
AX-157758291	1	3.11	KV452696.1	396875	1-scf00001	1
AX-157889590	1	3.11	KV452696.1	399060	1-scf00001	1
AX-157772256	1	3.11	KV452696.1	404019	1-scf00001	1
AX-157889595	1	3.11	KV452696.1	404914	1-scf00001	1
AX-157741017	1	3.11	KV452696.1	406411	1-scf00001	1
AX-157889596	1	3.11	KV452696.1	407064	1-scf00001	1
AX-157744835	1	3.11	KV452696.1	412670	1-scf00001	1
AX-157810127	1	3.11	KV452696.1	413768	1-scf00001	1
AX-157889598	1	3.11	KV452696.1	415096	1-scf00001	1
AX-157889603	1	3.11	KV452696.1	443000	1-scf00001	1
AX-158388940	1	3.11	KV452696.1	444988	1-scf00001	1
AX-157889605	1	3.11	KV452696.1	447058	1-scf00001	1
AX-157889607	1	3.11	KV452696.1	451043	1-scf00001	1
AX-157889608	1	3.11	KV452696.1	451633	1-scf00001	1
AX-157889609	1	3.11	KV452696.1	452176	1-scf00001	1
AX-157889610	1	3.11	KV452696.1	452520	1-scf00001	1

AX-157889611	1	3.11	KV452696.1	453026	1-scf00001	1
AX-157889612	1	3.11	KV452696.1	453512	1-scf00001	1
AX-157864281	1	3.11	KV452696.1	453977	1-scf00001	1
AX-157889613	1	3.11	KV452696.1	454853	1-scf00001	1
AX-157889614	1	3.11	KV452696.1	455243	1-scf00001	1
AX-157716152	1	3.11	KV452696.1	458402	1-scf00001	1
AX-157889570	1	3.329	KV452696.1	368482	1-scf00001	1
AX-157889620	1	3.329	KV452696.1	468871	1-scf00001	1
AX-157889622	1	3.329	KV452696.1	470135	1-scf00001	1
AX-157748641	1	3.329	KV452696.1	476383	1-scf00001	1
AX-157810137	1	3.329	KV452696.1	509514	1-scf00001	1
AX-157774141	1	3.493	KV452696.1	480231	1-scf00001	1
AX-157889619	1	3.657	KV452696.1	465454	1-scf00001	1
AX-157810133	1	3.657	KV452696.1	471962	1-scf00001	1
AX-157889628	1	3.657	KV452696.1	484061	1-scf00001	1
AX-157889631	1	3.657	KV452696.1	486206	1-scf00001	1
AX-157889632	1	3.657	KV452696.1	488223	1-scf00001	1
AX-157810134	1	3.657	KV452696.1	491318	1-scf00001	1
AX-157889634	1	3.657	KV452696.1	491530	1-scf00001	1
AX-157889635	1	3.657	KV452696.1	492649	1-scf00001	1
AX-157889636	1	3.657	KV452696.1	494103	1-scf00001	1
AX-157889638	1	3.657	KV452696.1	496040	1-scf00001	1
AX-157810135	1	3.657	KV452696.1	496700	1-scf00001	1
AX-157889640	1	3.657	KV452696.1	498302	1-scf00001	1
AX-157889641	1	3.657	KV452696.1	500249	1-scf00001	1
AX-157721405	1	3.657	KV452696.1	503025	1-scf00001	1
AX-157889644	1	3.657	KV452696.1	508589	1-scf00001	1
AX-157889645	1	3.657	KV452696.1	509811	1-scf00001	1
AX-157889646	1	3.657	KV452696.1	510482	1-scf00001	1
AX-157889647	1	3.657	KV452696.1	510841	1-scf00001	1
AX-157889650	1	3.657	KV452696.1	517711	1-scf00001	1
AX-157889651	1	3.657	KV452696.1	518788	1-scf00001	1
AX-157889652	1	3.657	KV452696.1	519011	1-scf00001	1
AX-157889655	1	3.657	KV452696.1	526301	1-scf00001	1
AX-157889656	1	3.657	KV452696.1	526797	1-scf00001	1
AX-157889658	1	3.657	KV452696.1	527711	1-scf00001	1
AX-158414477	1	3.657	KV452696.1	530164	1-scf00001	1
AX-157810138	1	3.657	KV452696.1	530759	1-scf00001	1
AX-157889659	1	3.657	KV452696.1	532904	1-scf00001	1
AX-157889660	1	3.659	KV452696.1	537125	1-scf00001	1
AX-157889661	1	3.802	KV452696.1	538993	1-scf00001	1
AX-157889669	1	3.802	KV452696.1	549268	1-scf00001	1
AX-157889665	1	3.948	KV452696.1	544184	1-scf00001	1
AX-157889667	1	3.948	KV452696.1	546164	1-scf00001	1
AX-157889668	1	3.948	KV452696.1	546783	1-scf00001	1
AX-157889671	1	4.094	KV452696.1	556171	1-scf00001	1
AX-157889672	1	4.094	KV452696.1	563943	1-scf00001	1
AX-157889674	1	4.094	KV452696.1	564979	1-scf00001	1
AX-157889677	1	4.094	KV452696.1	572672	1-scf00001	1
AX-157889679	1	4.094	KV452696.1	573895	1-scf00001	1
AX-157707266	1	4.094	3ML01033859	369	scf09525_1	UN

AX-157889680	1	4.16	KV452696.1	575302	1-scf00001	1
AX-157889670	1	4.203	KV452696.1	555656	1-scf00001	1
AX-157714799	1	4.203	KV452696.1	584693	1-scf00001	1
AX-157889684	1	4.203	KV452696.1	586628	1-scf00001	1
AX-157889685	1	4.203	KV452696.1	588010	1-scf00001	1
AX-157889687	1	4.203	KV452696.1	590546	1-scf00001	1
AX-157889689	1	4.203	KV452696.1	592522	1-scf00001	1
AX-157751020	1	4.367	KV452696.1	595206	1-scf00001	1
AX-157889691	1	4.463	KV452696.1	598593	1-scf00001	1
AX-158381857	1	4.463	KV452696.1	612588	1-scf00001	1
AX-157853501	1	4.531	KV452696.1	603185	1-scf00001	1
AX-157889696	1	4.531	KV452696.1	606839	1-scf00001	1
AX-157756520	1	4.579	KV452696.1	608887	1-scf00001	1
AX-157889702	1	4.579	KV452696.1	619901	1-scf00001	1
AX-157889703	1	4.579	KV452696.1	621982	1-scf00001	1
AX-158379083	1	4.641	KV452696.1	617051	1-scf00001	1
AX-157745552	1	4.641	KV452696.1	618418	1-scf00001	1
AX-157889701	1	4.641	KV452696.1	619275	1-scf00001	1
AX-157745372	1	4.75	KV452696.1	620843	1-scf00001	1
AX-157889704	1	4.82	KV452696.1	623815	1-scf00001	1
AX-157747755	1	4.894	KV452696.1	626051	1-scf00001	1
AX-157889713	1	4.894	KV452696.1	644477	1-scf00001	1
AX-157889706	1	4.969	KV452696.1	626217	1-scf00001	1
AX-157746363	1	4.985	KV452696.1	599728	1-scf00001	1
AX-157889708	1	4.985	KV452696.1	631038	1-scf00001	1
AX-157889711	1	4.985	KV452696.1	641263	1-scf00001	1
AX-157889728	1	4.985	KV452696.1	664140	1-scf00001	1
AX-157889710	1	5.093	KV452696.1	636922	1-scf00001	1
AX-157889693	1	5.217	KV452696.1	603581	1-scf00001	1
AX-157745845	1	5.217	KV452696.1	643774	1-scf00001	1
AX-157889714	1	5.217	KV452696.1	644905	1-scf00001	1
AX-157889716	1	5.217	KV452696.1	647726	1-scf00001	1
AX-157889717	1	5.217	KV452696.1	648099	1-scf00001	1
AX-157889719	1	5.592	KV452696.1	651212	1-scf00001	1
AX-157889720	1	5.592	KV452696.1	652508	1-scf00001	1
AX-157889722	1	5.592	KV452696.1	655575	1-scf00001	1
AX-157889723	1	5.843	KV452696.1	656418	1-scf00001	1
AX-157889725	1	5.843	KV452696.1	660019	1-scf00001	1
AX-157889733	1	5.855	KV452696.1	669952	1-scf00001	1
AX-157889734	1	6.158	KV452696.1	672407	1-scf00001	1
AX-157889735	1	6.605	KV452696.1	674082	1-scf00001	1
AX-157810145	1	6.62	KV452696.1	675703	1-scf00001	1
AX-157889746	1	7.159	KV452696.1	691482	1-scf00001	1
AX-158379084	1	7.159	KV452696.1	698299	1-scf00001	1
AX-157889755	1	7.159	KV452696.1	701135	1-scf00001	1
AX-157889757	1	7.159	KV452696.1	702627	1-scf00001	1
AX-157889759	1	7.557	KV452696.1	707649	1-scf00001	1
AX-157889760	1	7.559	KV452696.1	710665	1-scf00001	1
AX-157883017	1	7.768	KV452696.1	713871	1-scf00001	1
AX-157889763	1	7.924	KV452696.1	718362	1-scf00001	1
AX-157889765	1	7.924	KV452696.1	720720	1-scf00001	1

AX-157889766	1	7.924	KV452696.1	720936	1-scf00001	1
AX-157889767	1	7.924	KV452696.1	721946	1-scf00001	1
AX-157810148	1	7.924	KV452696.1	724066	1-scf00001	1
AX-157889769	1	7.924	KV452696.1	724240	1-scf00001	1
AX-157889770	1	7.924	KV452696.1	725555	1-scf00001	1
AX-157810149	1	7.924	KV452696.1	726897	1-scf00001	1
AX-157889772	1	7.924	KV452696.1	728294	1-scf00001	1
AX-157889773	1	7.924	KV452696.1	730475	1-scf00001	1
AX-157810150	1	7.924	KV452696.1	731115	1-scf00001	1
AX-157810151	1	8.47	KV452696.1	741311	1-scf00001	1
AX-157889775	1	8.47	KV452696.1	742530	1-scf00001	1
AX-157889776	1	8.47	KV452696.1	742963	1-scf00001	1
AX-157889777	1	8.47	KV452696.1	746931	1-scf00001	1
AX-157889778	1	8.47	KV452696.1	747516	1-scf00001	1
AX-157722180	1	8.47	KV452696.1	748053	1-scf00001	1
AX-157889779	1	8.47	KV452696.1	748657	1-scf00001	1
AX-157810152	1	8.47	KV452696.1	748818	1-scf00001	1
AX-157810153	1	8.47	KV452696.1	748996	1-scf00001	1
AX-157810154	1	8.647	KV452696.1	752686	1-scf00001	1
AX-157889782	1	8.648	KV452696.1	755342	1-scf00001	1
AX-157889788	1	8.648	KV452696.1	764041	1-scf00001	1
AX-157889783	1	8.65	KV452696.1	757010	1-scf00001	1
AX-157772334	1	8.651	KV452696.1	761413	1-scf00001	1
AX-157889787	1	8.755	KV452696.1	762678	1-scf00001	1
AX-157889789	1	9.053	KV452696.1	764455	1-scf00001	1
AX-157889792	1	9.053	KV452696.1	767021	1-scf00001	1
AX-157889793	1	9.053	KV452696.1	769435	1-scf00001	1
AX-157889794	1	9.053	KV452696.1	770749	1-scf00001	1
AX-157714747	1	9.057	KV452696.1	772703	1-scf00001	1
AX-157889799	1	9.057	KV452696.1	778383	1-scf00001	1
AX-157889798	1	9.308	KV452696.1	776762	1-scf00001	1
AX-157889802	1	9.563	KV452696.1	783575	1-scf00001	1
AX-157889803	1	9.563	KV452696.1	783794	1-scf00001	1
AX-157889790	1	9.564	KV452696.1	765641	1-scf00001	1
AX-157889804	1	9.564	KV452696.1	785388	1-scf00001	1
AX-157889807	1	9.564	KV452696.1	788111	1-scf00001	1
AX-157889808	1	9.564	KV452696.1	788564	1-scf00001	1
AX-157889812	1	9.564	KV452696.1	793933	1-scf00001	1
AX-157889814	1	9.564	KV452696.1	795350	1-scf00001	1
AX-157889815	1	9.564	KV452696.1	797873	1-scf00001	1
AX-157889817	1	9.564	KV452696.1	799171	1-scf00001	1
AX-157889820	1	9.564	KV452696.1	803164	1-scf00001	1
AX-157889824	1	9.564	KV452696.1	806618	1-scf00001	1
AX-158381861	1	9.674	KV452696.1	810017	1-scf00001	1
AX-157889825	1	9.674	KV452696.1	810880	1-scf00001	1
AX-157772660	1	9.948	KV452696.1	750201	1-scf00001	1
AX-157752610	1	9.948	KV452696.1	811832	1-scf00001	1
AX-157889827	1	9.948	KV452696.1	817037	1-scf00001	1
AX-157889830	1	9.948	KV452696.1	820162	1-scf00001	1
AX-157889836	1	9.948	KV452696.1	824374	1-scf00001	1
AX-157750971	1	9.948	KV452696.1	891116	1-scf00001	1

AX-157889892	1	9.948	KV452696.1	896228	1-scf00001	1
AX-157710186	1	9.948	KV452696.1	909176	1-scf00001	1
AX-157889978	1	9.948	KV452696.1	1020378	1-scf00001	1
AX-157889984	1	9.948	KV452696.1	1028606	1-scf00001	1
AX-157890062	1	9.948	KV452696.1	1157438	1-scf00001	1
AX-157890260	1	9.948	KV452696.1	1546100	1-scf00001	1
AX-157890313	1	9.948	KV452696.1	1630635	1-scf00001	1
AX-157890316	1	9.948	KV452696.1	1631642	1-scf00001	1
AX-157889834	1	10.002	KV452696.1	822620	1-scf00001	1
AX-157889840	1	10.057	KV452696.1	829630	1-scf00001	1
AX-157874210	1	10.086	KV452696.1	829868	1-scf00001	1
AX-157889842	1	10.211	KV452696.1	831204	1-scf00001	1
AX-157889843	1	10.213	KV452696.1	833098	1-scf00001	1
AX-157889844	1	10.215	KV452696.1	833535	1-scf00001	1
AX-157889848	1	10.215	KV452696.1	838134	1-scf00001	1
AX-157889879	1	10.215	KV452696.1	871600	1-scf00001	1
AX-157889850	1	10.218	KV452696.1	839301	1-scf00001	1
AX-157889806	1	10.22	KV452696.1	787304	1-scf00001	1
AX-157757921	1	10.22	KV452696.1	837009	1-scf00001	1
AX-157889851	1	10.22	KV452696.1	840049	1-scf00001	1
AX-157889854	1	10.22	KV452696.1	842260	1-scf00001	1
AX-157738422	1	10.22	KV452696.1	843708	1-scf00001	1
AX-157810160	1	10.22	KV452696.1	844155	1-scf00001	1
AX-157889857	1	10.22	KV452696.1	846400	1-scf00001	1
AX-157889859	1	10.22	KV452696.1	847829	1-scf00001	1
AX-157719903	1	10.22	KV452696.1	848061	1-scf00001	1
AX-157889860	1	10.22	KV452696.1	849547	1-scf00001	1
AX-157889861	1	10.22	KV452696.1	850640	1-scf00001	1
AX-157874211	1	10.22	KV452696.1	851868	1-scf00001	1
AX-157889862	1	10.22	KV452696.1	853048	1-scf00001	1
AX-157889868	1	10.22	KV452696.1	856696	1-scf00001	1
AX-157889869	1	10.22	KV452696.1	857859	1-scf00001	1
AX-157889870	1	10.22	KV452696.1	858395	1-scf00001	1
AX-157889871	1	10.22	KV452696.1	859985	1-scf00001	1
AX-157889874	1	10.22	KV452696.1	862919	1-scf00001	1
AX-157889875	1	10.22	KV452696.1	864142	1-scf00001	1
AX-157889878	1	10.22	KV452696.1	870264	1-scf00001	1
AX-157889884	1	10.22	KV452696.1	881196	1-scf00001	1
AX-157754391	1	10.657	KV452696.1	841550	1-scf00001	1
AX-157810162	1	10.657	KV452696.1	874395	1-scf00001	1
AX-157889882	1	10.657	KV452696.1	875503	1-scf00001	1
AX-157889883	1	10.657	KV452696.1	878486	1-scf00001	1
AX-157810163	1	10.657	KV452696.1	880755	1-scf00001	1
AX-157774409	1	10.708	KV452696.1	881690	1-scf00001	1
AX-157755190	1	10.708	KV452696.1	882236	1-scf00001	1
AX-157889885	1	10.766	KV452696.1	883878	1-scf00001	1
AX-157889886	1	10.766	KV452696.1	884838	1-scf00001	1
AX-157889887	1	10.766	KV452696.1	885390	1-scf00001	1
AX-157889888	1	10.766	KV452696.1	886315	1-scf00001	1
AX-157775319	1	10.766	KV452696.1	886818	1-scf00001	1
AX-157889890	1	10.985	KV452696.1	893966	1-scf00001	1

AX-157853507	1	10.985	KV452696.1	895967	1-scf00001	1
AX-157889897	1	10.985	KV452696.1	900739	1-scf00001	1
AX-157889901	1	11.094	KV452696.1	903613	1-scf00001	1
AX-157889903	1	11.203	KV452696.1	906571	1-scf00001	1
AX-157889904	1	11.222	KV452696.1	907207	1-scf00001	1
AX-157889905	1	11.24	KV452696.1	907587	1-scf00001	1
AX-157889909	1	11.24	KV452696.1	916368	1-scf00001	1
AX-157889908	1	11.256	KV452696.1	914932	1-scf00001	1
AX-157889918	1	11.256	KV452696.1	924464	1-scf00001	1
AX-157889910	1	11.272	KV452696.1	917206	1-scf00001	1
AX-157889917	1	11.282	KV452696.1	923228	1-scf00001	1
AX-157889919	1	11.297	KV452696.1	924756	1-scf00001	1
AX-157889920	1	11.312	KV452696.1	927111	1-scf00001	1
AX-157889922	1	11.489	KV452696.1	931710	1-scf00001	1
AX-157889928	1	11.625	KV452696.1	936303	1-scf00001	1
AX-157889934	1	11.833	KV452696.1	945965	1-scf00001	1
AX-157889936	1	12.25	KV452696.1	949431	1-scf00001	1
AX-157889937	1	12.25	KV452696.1	950359	1-scf00001	1
AX-157889938	1	12.25	KV452696.1	950806	1-scf00001	1
AX-157889939	1	12.308	KV452696.1	951324	1-scf00001	1
AX-157889948	1	12.308	KV452696.1	963019	1-scf00001	1
AX-157889954	1	12.308	KV452696.1	976884	1-scf00001	1
AX-157889800	1	12.356	KV452696.1	778736	1-scf00001	1
AX-157889949	1	12.356	KV452696.1	969001	1-scf00001	1
AX-157709834	1	12.356	KV452696.1	972267	1-scf00001	1
AX-157889945	1	12.407	KV452696.1	957480	1-scf00001	1
AX-157889950	1	12.407	KV452696.1	970390	1-scf00001	1
AX-157740309	1	12.407	KV452696.1	971747	1-scf00001	1
AX-158381866	1	12.407	KV452696.1	972056	1-scf00001	1
AX-157889951	1	12.407	KV452696.1	973845	1-scf00001	1
AX-157889956	1	12.407	KV452696.1	979989	1-scf00001	1
AX-158379086	1	12.407	KV452696.1	980860	1-scf00001	1
AX-157889957	1	12.407	KV452696.1	980938	1-scf00001	1
AX-157889958	1	12.407	KV452696.1	982909	1-scf00001	1
AX-158381867	1	12.407	KV452696.1	990577	1-scf00001	1
AX-157889940	1	12.735	KV452696.1	951777	1-scf00001	1
AX-157889959	1	12.735	KV452696.1	985636	1-scf00001	1
AX-157722574	1	12.735	KV452696.1	987219	1-scf00001	1
AX-157883020	1	12.735	KV452696.1	990275	1-scf00001	1
AX-157890057	1	12.735	KV452696.1	1142028	1-scf00001	1
AX-157889967	1	12.844	KV452696.1	997133	1-scf00001	1
AX-157745490	1	12.844	KV452696.1	997556	1-scf00001	1
AX-157889972	1	12.844	KV452696.1	1005580	1-scf00001	1
AX-157889973	1	13.063	KV452696.1	1006606	1-scf00001	1
AX-157740346	1	13.063	KV452696.1	1009121	1-scf00001	1
AX-157889974	1	13.063	KV452696.1	1009649	1-scf00001	1
AX-157755170	1	13.251	KV452696.1	1018652	1-scf00001	1
AX-157889979	1	13.251	KV452696.1	1024744	1-scf00001	1
AX-157889981	1	13.251	KV452696.1	1026361	1-scf00001	1
AX-157738973	1	13.272	KV452696.1	1027547	1-scf00001	1
AX-157889987	1	13.809	KV452696.1	1033921	1-scf00001	1

AX-157889980	1	13.822	KV452696.1	1025800	1-scf00001	1
AX-157889988	1	13.822	KV452696.1	1034861	1-scf00001	1
AX-157889989	1	13.835	KV452696.1	1036118	1-scf00001	1
AX-157889996	1	14.18	KV452696.1	1044433	1-scf00001	1
AX-157751620	1	14.181	KV452696.1	1048572	1-scf00001	1
AX-157889998	1	14.263	KV452696.1	1049000	1-scf00001	1
AX-157890006	1	14.703	KV452696.1	1063762	1-scf00001	1
AX-157890007	1	14.705	KV452696.1	1065027	1-scf00001	1
AX-157890008	1	14.705	KV452696.1	1065454	1-scf00001	1
AX-157890009	1	14.705	KV452696.1	1065972	1-scf00001	1
AX-157890010	1	14.924	KV452696.1	1067732	1-scf00001	1
AX-157720812	1	14.927	KV452696.1	1069010	1-scf00001	1
AX-157890012	1	14.931	KV452696.1	1071994	1-scf00001	1
AX-157758700	1	15.363	KV452696.1	1074442	1-scf00001	1
AX-157890013	1	15.363	KV452696.1	1075736	1-scf00001	1
AX-157890014	1	15.363	KV452696.1	1076641	1-scf00001	1
AX-157890016	1	15.363	KV452696.1	1078216	1-scf00001	1
AX-157890017	1	15.363	KV452696.1	1078618	1-scf00001	1
AX-157890020	1	15.363	KV452696.1	1081957	1-scf00001	1
AX-157708594	1	15.363	KV452696.1	1089878	1-scf00001	1
AX-157752804	1	15.363	KV452696.1	1091874	1-scf00001	1
AX-157890022	1	15.363	KV452696.1	1092919	1-scf00001	1
AX-157890024	1	15.363	KV452696.1	1094329	1-scf00001	1
AX-158388945	1	15.363	KV452696.1	1107380	1-scf00001	1
AX-157890029	1	15.674	KV452696.1	1099565	1-scf00001	1
AX-157890037	1	15.674	KV452696.1	1110772	1-scf00001	1
AX-157890038	1	15.674	KV452696.1	1111850	1-scf00001	1
AX-157750601	1	15.831	KV452696.1	1114618	1-scf00001	1
AX-157890043	1	15.867	KV452696.1	1122305	1-scf00001	1
AX-157890044	1	16.424	KV452696.1	1124570	1-scf00001	1
AX-157890047	1	16.584	KV452696.1	1127571	1-scf00001	1
AX-157890051	1	16.584	KV452696.1	1132072	1-scf00001	1
AX-157744890	1	16.703	KV452696.1	1128672	1-scf00001	1
AX-157890052	1	16.975	KV452696.1	1133023	1-scf00001	1
AX-157890055	1	16.975	KV452696.1	1139051	1-scf00001	1
AX-157890058	1	17.653	KV452696.1	1143545	1-scf00001	1
AX-157710971	1	17.653	KV452696.1	1143949	1-scf00001	1
AX-157890061	1	17.653	KV452696.1	1153614	1-scf00001	1
AX-157890064	1	17.89	KV452696.1	1159616	1-scf00001	1
AX-157890065	1	17.89	KV452696.1	1160539	1-scf00001	1
AX-157890068	1	18.611	KV452696.1	1169309	1-scf00001	1
AX-157890070	1	18.611	KV452696.1	1179812	1-scf00001	1
AX-157772638	1	18.611	KV452696.1	1182542	1-scf00001	1
AX-157741122	1	18.756	KV452696.1	1186796	1-scf00001	1
AX-157890074	1	18.787	KV452696.1	1187606	1-scf00001	1
AX-157890075	1	18.788	KV452696.1	1192363	1-scf00001	1
AX-157890021	1	19.092	KV452696.1	1084921	1-scf00001	1
AX-158381871	1	19.092	KV452696.1	1144484	1-scf00001	1
AX-157752620	1	19.092	KV452696.1	1198332	1-scf00001	1
AX-157890077	1	19.092	KV452696.1	1202057	1-scf00001	1
AX-157890078	1	19.092	KV452696.1	1207316	1-scf00001	1

AX-157890079	1	19.092	KV452696.1	1208339	1-scf00001	1
AX-158379087	1	19.092	KV452696.1	1209623	1-scf00001	1
AX-157890084	1	19.092	KV452696.1	1214161	1-scf00001	1
AX-157890210	1	19.092	KV452696.1	1459302	1-scf00001	1
AX-157890085	1	19.127	KV452696.1	1214488	1-scf00001	1
AX-157890086	1	19.17	KV452696.1	1216792	1-scf00001	1
AX-157890102	1	19.17	KV452696.1	1240786	1-scf00001	1
AX-157890088	1	19.229	KV452696.1	1217730	1-scf00001	1
AX-157890093	1	19.311	KV452696.1	1224560	1-scf00001	1
AX-157890094	1	19.311	KV452696.1	1227167	1-scf00001	1
AX-157890095	1	19.311	KV452696.1	1229131	1-scf00001	1
AX-157890098	1	19.311	KV452696.1	1234317	1-scf00001	1
AX-157890103	1	19.311	KV452696.1	1242934	1-scf00001	1
AX-157890105	1	19.311	KV452696.1	1246406	1-scf00001	1
AX-157754948	1	19.337	KV452696.1	1221520	1-scf00001	1
AX-157774620	1	19.337	KV452696.1	1233325	1-scf00001	1
AX-157890108	1	19.337	KV452696.1	1249077	1-scf00001	1
AX-157890109	1	19.337	KV452696.1	1249477	1-scf00001	1
AX-157890121	1	19.337	KV452696.1	1267179	1-scf00001	1
AX-157890124	1	19.337	KV452696.1	1270924	1-scf00001	1
AX-157890160	1	19.337	KV452696.1	1327956	1-scf00001	1
AX-157890229	1	19.337	KV452696.1	1495078	1-scf00001	1
AX-157890116	1	19.475	KV452696.1	1260464	1-scf00001	1
AX-157890096	1	19.639	KV452696.1	1230618	1-scf00001	1
AX-157890117	1	19.639	KV452696.1	1261810	1-scf00001	1
AX-157890118	1	19.639	KV452696.1	1263666	1-scf00001	1
AX-157750052	1	19.639	KV452696.1	1266712	1-scf00001	1
AX-157890122	1	19.693	KV452696.1	1268976	1-scf00001	1
AX-157890123	1	19.748	KV452696.1	1269779	1-scf00001	1
AX-157754707	1	19.858	KV452696.1	1274924	1-scf00001	1
AX-157890130	1	19.858	KV452696.1	1288585	1-scf00001	1
AX-157890132	1	19.858	KV452696.1	1289223	1-scf00001	1
AX-157890133	1	19.858	KV452696.1	1289563	1-scf00001	1
AX-157890139	1	19.906	KV452696.1	1295238	1-scf00001	1
AX-157890141	1	19.967	KV452696.1	1298273	1-scf00001	1
AX-157890142	1	19.967	KV452696.1	1299583	1-scf00001	1
AX-157890145	1	19.999	KV452696.1	1306067	1-scf00001	1
AX-157890172	1	19.999	KV452696.1	1351757	1-scf00001	1
AX-157890147	1	20.016	KV452696.1	1306868	1-scf00001	1
AX-157890150	1	20.103	KV452696.1	1308943	1-scf00001	1
AX-157709458	1	20.186	KV452696.1	1268814	1-scf00001	1
AX-157702551	1	20.186	KV452696.1	1283426	1-scf00001	1
AX-157890137	1	20.186	KV452696.1	1292845	1-scf00001	1
AX-157890149	1	20.186	KV452696.1	1308092	1-scf00001	1
AX-157890151	1	20.186	KV452696.1	1310670	1-scf00001	1
AX-157890154	1	20.186	KV452696.1	1316402	1-scf00001	1
AX-157890157	1	20.186	KV452696.1	1319883	1-scf00001	1
AX-157890158	1	20.186	KV452696.1	1320596	1-scf00001	1
AX-157853513	1	20.186	KV452696.1	1321660	1-scf00001	1
AX-158379088	1	20.186	KV452696.1	1345918	1-scf00001	1
AX-157890173	1	20.186	KV452696.1	1354340	1-scf00001	1

AX-157890174	1	20.186	KV452696.1	1355443	1-scf00001	1
AX-157890179	1	20.186	KV452696.1	1367158	1-scf00001	1
AX-157890297	1	20.186	KV452696.1	1614002	1-scf00001	1
AX-157810166	1	20.272	KV452696.1	1352408	1-scf00001	1
AX-157810167	1	20.295	KV452696.1	1353430	1-scf00001	1
AX-157890175	1	20.295	KV452696.1	1357229	1-scf00001	1
AX-157890176	1	20.295	KV452696.1	1360804	1-scf00001	1
AX-157890178	1	20.405	KV452696.1	1366400	1-scf00001	1
AX-157890181	1	20.405	KV452696.1	1368571	1-scf00001	1
AX-157699460	1	20.405	KV452696.1	1370145	1-scf00001	1
AX-157890186	1	20.405	KV452696.1	1378393	1-scf00001	1
AX-157890187	1	20.407	KV452696.1	1379742	1-scf00001	1
AX-157890188	1	20.411	KV452696.1	1380121	1-scf00001	1
AX-157751344	1	20.621	KV452696.1	1385336	1-scf00001	1
AX-157890191	1	20.737	KV452696.1	1383578	1-scf00001	1
AX-157890193	1	20.737	KV452696.1	1387257	1-scf00001	1
AX-157810179	1	20.737	KV452696.1	1530389	1-scf00001	1
AX-157890196	1	20.842	KV452696.1	1396611	1-scf00001	1
AX-157890197	1	20.842	KV452696.1	1399293	1-scf00001	1
AX-157715478	1	20.842	KV452696.1	1483353	1-scf00001	1
AX-157810171	1	20.842	KV452696.1	1484103	1-scf00001	1
AX-157890223	1	20.842	KV452696.1	1491084	1-scf00001	1
AX-157890225	1	20.842	KV452696.1	1493206	1-scf00001	1
AX-157890226	1	20.842	KV452696.1	1493969	1-scf00001	1
AX-157810173	1	20.842	KV452696.1	1498369	1-scf00001	1
AX-157810182	1	20.842	KV452696.1	1542128	1-scf00001	1
AX-157890222	1	20.92	KV452696.1	1489934	1-scf00001	1
AX-157748550	1	20.92	KV452696.1	1499086	1-scf00001	1
AX-157890233	1	20.92	KV452696.1	1500541	1-scf00001	1
AX-157890281	1	20.92	KV452696.1	1581390	1-scf00001	1
AX-157890289	1	20.92	KV452696.1	1596137	1-scf00001	1
AX-158379090	1	20.92	KV452696.1	1755386	1-scf00001	1
AX-157890404	1	20.92	KV452696.1	1757526	1-scf00001	1
AX-157890170	1	20.932	KV452696.1	1347204	1-scf00001	1
AX-157890240	1	20.932	KV452696.1	1510059	1-scf00001	1
AX-157716217	1	20.946	KV452696.1	1514599	1-scf00001	1
AX-157890419	1	20.946	KV452696.1	1786650	1-scf00001	1
AX-157810175	1	20.952	KV452696.1	1505658	1-scf00001	1
AX-157890243	1	20.952	KV452696.1	1517387	1-scf00001	1
AX-157810181	1	20.952	KV452696.1	1540871	1-scf00001	1
AX-157754762	1	20.952	KV452696.1	1541212	1-scf00001	1
AX-157751738	1	20.952	KV452696.1	1541963	1-scf00001	1
AX-157810177	1	21.17	KV452696.1	1522567	1-scf00001	1
AX-157890250	1	21.17	KV452696.1	1525623	1-scf00001	1
AX-157890254	1	21.17	KV452696.1	1538747	1-scf00001	1
AX-157751055	1	21.17	KV452696.1	1568156	1-scf00001	1
AX-157890271	1	21.17	KV452696.1	1571558	1-scf00001	1
AX-157890272	1	21.17	KV452696.1	1571837	1-scf00001	1
AX-157890273	1	21.17	KV452696.1	1572222	1-scf00001	1
AX-157890274	1	21.17	KV452696.1	1574078	1-scf00001	1
AX-157890279	1	21.17	KV452696.1	1577979	1-scf00001	1

AX-157890283	1	21.17	KV452696.1	1583639	1-scf00001	1
AX-157890286	1	21.17	KV452696.1	1592606	1-scf00001	1
AX-157890290	1	21.17	KV452696.1	1597062	1-scf00001	1
AX-157890292	1	21.17	KV452696.1	1604680	1-scf00001	1
AX-157810189	1	21.17	KV452696.1	1625752	1-scf00001	1
AX-157890295	1	21.205	KV452696.1	1612515	1-scf00001	1
AX-157890169	1	21.225	KV452696.1	1341096	1-scf00001	1
AX-157890232	1	21.225	KV452696.1	1500095	1-scf00001	1
AX-157890238	1	21.225	KV452696.1	1504764	1-scf00001	1
AX-157890246	1	21.225	KV452696.1	1519509	1-scf00001	1
AX-157890293	1	21.225	KV452696.1	1611632	1-scf00001	1
AX-157890296	1	21.225	KV452696.1	1613383	1-scf00001	1
AX-157890338	1	21.225	KV452696.1	1659880	1-scf00001	1
AX-157890347	1	21.225	KV452696.1	1676031	1-scf00001	1
AX-157853518	1	21.225	KV452696.1	1692487	1-scf00001	1
AX-157890364	1	21.225	KV452696.1	1694383	1-scf00001	1
AX-157890306	1	21.245	KV452696.1	1620104	1-scf00001	1
AX-157890314	1	21.279	KV452696.1	1631093	1-scf00001	1
AX-157890315	1	21.279	KV452696.1	1631329	1-scf00001	1
AX-157890317	1	21.279	KV452696.1	1632101	1-scf00001	1
AX-157810191	1	21.279	KV452696.1	1635798	1-scf00001	1
AX-157890199	1	21.374	KV452696.1	1400447	1-scf00001	1
AX-157890320	1	21.374	KV452696.1	1637003	1-scf00001	1
AX-157890319	1	21.498	KV452696.1	1636548	1-scf00001	1
AX-157890326	1	21.498	KV452696.1	1642833	1-scf00001	1
AX-157890327	1	21.498	KV452696.1	1643315	1-scf00001	1
AX-157890330	1	21.498	KV452696.1	1645520	1-scf00001	1
AX-157890336	1	21.498	KV452696.1	1655212	1-scf00001	1
AX-157890202	1	21.612	KV452696.1	1412254	1-scf00001	1
AX-157890237	1	21.612	KV452696.1	1504514	1-scf00001	1
AX-157890256	1	21.612	KV452696.1	1539747	1-scf00001	1
AX-157890263	1	21.612	KV452696.1	1549505	1-scf00001	1
AX-157810188	1	21.612	KV452696.1	1599693	1-scf00001	1
AX-157747986	1	21.612	KV452696.1	1668015	1-scf00001	1
AX-157890402	1	21.612	KV452696.1	1755968	1-scf00001	1
AX-157755314	1	21.612	KV452696.1	1761798	1-scf00001	1
AX-157890475	1	21.612	KV452696.1	1863993	1-scf00001	1
AX-157890499	1	21.612	KV452696.1	1919333	1-scf00001	1
AX-157890825	1	21.612	KV452696.1	2543426	1-scf00001	1
AX-157890919	1	21.612	KV452696.1	2746935	1-scf00001	1
AX-158379096	1	21.612	KV452696.1	2795120	1-scf00001	1
AX-157890962	1	21.612	KV452696.1	2828143	1-scf00001	1
AX-157890966	1	21.612	KV452696.1	2835233	1-scf00001	1
AX-157890967	1	21.612	KV452696.1	2837237	1-scf00001	1
AX-157890341	1	21.717	KV452696.1	1667459	1-scf00001	1
AX-157853517	1	21.717	KV452696.1	1668260	1-scf00001	1
AX-157890344	1	21.717	KV452696.1	1672200	1-scf00001	1
AX-157890345	1	21.717	KV452696.1	1673668	1-scf00001	1
AX-157774868	1	21.717	KV452696.1	1676407	1-scf00001	1
AX-157890348	1	21.717	KV452696.1	1676554	1-scf00001	1
AX-157890349	1	21.717	KV452696.1	1677547	1-scf00001	1

AX-157890350	1	21.717	KV452696.1	1678555	1-scf00001	1
AX-157890354	1	21.717	KV452696.1	1684696	1-scf00001	1
AX-157890359	1	21.826	KV452696.1	1688228	1-scf00001	1
AX-157890360	1	21.826	KV452696.1	1689225	1-scf00001	1
AX-157890366	1	21.855	KV452696.1	1698603	1-scf00001	1
AX-157772973	1	21.87	KV452696.1	1415924	1-scf00001	1
AX-157890218	1	21.87	KV452696.1	1485525	1-scf00001	1
AX-157890231	1	21.87	KV452696.1	1496571	1-scf00001	1
AX-157890239	1	21.87	KV452696.1	1509340	1-scf00001	1
AX-157810186	1	21.87	KV452696.1	1567008	1-scf00001	1
AX-157890268	1	21.87	KV452696.1	1568900	1-scf00001	1
AX-157890285	1	21.87	KV452696.1	1588712	1-scf00001	1
AX-157890323	1	21.87	KV452696.1	1641423	1-scf00001	1
AX-157890331	1	21.87	KV452696.1	1645734	1-scf00001	1
AX-157890365	1	21.87	KV452696.1	1697790	1-scf00001	1
AX-157890368	1	21.87	KV452696.1	1700267	1-scf00001	1
AX-157890372	1	21.87	KV452696.1	1707479	1-scf00001	1
AX-157890377	1	21.87	KV452696.1	1720435	1-scf00001	1
AX-157890381	1	21.87	KV452696.1	1723891	1-scf00001	1
AX-157716500	1	21.898	KV452696.1	1711787	1-scf00001	1
AX-157890373	1	21.935	KV452696.1	1712777	1-scf00001	1
AX-157890376	1	21.949	KV452696.1	1719072	1-scf00001	1
AX-157890378	1	22.044	KV452696.1	1722267	1-scf00001	1
AX-157771832	1	22.132	KV452696.1	1725285	1-scf00001	1
AX-157890392	1	22.132	KV452696.1	1738841	1-scf00001	1
AX-157890382	1	22.205	KV452696.1	1726073	1-scf00001	1
AX-157890384	1	22.263	KV452696.1	1730582	1-scf00001	1
AX-157890387	1	22.263	KV452696.1	1732020	1-scf00001	1
AX-157890388	1	22.263	KV452696.1	1732370	1-scf00001	1
AX-157746839	1	22.287	KV452696.1	1737563	1-scf00001	1
AX-157890395	1	22.287	KV452696.1	1747609	1-scf00001	1
AX-157713471	1	22.287	KV452696.1	1765411	1-scf00001	1
AX-157810197	1	22.313	KV452696.1	1748946	1-scf00001	1
AX-157890394	1	22.372	KV452696.1	1741711	1-scf00001	1
AX-157810198	1	22.372	KV452696.1	1749581	1-scf00001	1
AX-157749741	1	22.405	KV452696.1	1751358	1-scf00001	1
AX-157890406	1	22.444	KV452696.1	1759519	1-scf00001	1
AX-157890407	1	22.482	KV452696.1	1765956	1-scf00001	1
AX-157890409	1	22.482	KV452696.1	1767187	1-scf00001	1
AX-157890410	1	22.482	KV452696.1	1768845	1-scf00001	1
AX-157890416	1	22.49	KV452696.1	1779078	1-scf00001	1
AX-157890417	1	22.684	KV452696.1	1780200	1-scf00001	1
AX-157890420	1	22.891	KV452696.1	1787630	1-scf00001	1
AX-157890421	1	22.891	KV452696.1	1788678	1-scf00001	1
AX-157890423	1	22.891	KV452696.1	1789844	1-scf00001	1
AX-157890428	1	22.891	KV452696.1	1795049	1-scf00001	1
AX-157890429	1	22.891	KV452696.1	1795434	1-scf00001	1
AX-157890430	1	22.891	KV452696.1	1795844	1-scf00001	1
AX-157890431	1	22.891	KV452696.1	1796562	1-scf00001	1
AX-157890399	1	23.028	KV452696.1	1752191	1-scf00001	1
AX-158379091	1	23.028	KV452696.1	1790162	1-scf00001	1

AX-157890425	1	23.028	KV452696.1	1792122	1-scf00001	1
AX-157890434	1	23.028	KV452696.1	1800148	1-scf00001	1
AX-157890435	1	23.028	KV452696.1	1801399	1-scf00001	1
AX-157883025	1	23.028	KV452696.1	1801638	1-scf00001	1
AX-157890436	1	23.028	KV452696.1	1802019	1-scf00001	1
AX-157890438	1	23.028	KV452696.1	1803676	1-scf00001	1
AX-157890439	1	23.028	KV452696.1	1804468	1-scf00001	1
AX-158404134	1	23.028	KV452696.1	1807366	1-scf00001	1
AX-157890442	1	23.028	KV452696.1	1813283	1-scf00001	1
AX-157890444	1	23.028	KV452696.1	1815807	1-scf00001	1
AX-157890445	1	23.028	KV452696.1	1816310	1-scf00001	1
AX-157890448	1	23.028	KV452696.1	1821724	1-scf00001	1
AX-157890450	1	23.028	KV452696.1	1823285	1-scf00001	1
AX-157890452	1	23.028	KV452696.1	1825206	1-scf00001	1
AX-157890453	1	23.028	KV452696.1	1825597	1-scf00001	1
AX-157890456	1	23.028	KV452696.1	1828243	1-scf00001	1
AX-157810199	1	23.028	KV452696.1	1836655	1-scf00001	1
AX-157890482	1	23.028	KV452696.1	1873550	1-scf00001	1
AX-157874229	1	23.028	KV452696.1	1888523	1-scf00001	1
AX-157890843	1	23.028	KV452696.1	2594269	1-scf00001	1
AX-157890470	1	23.086	KV452696.1	1853177	1-scf00001	1
AX-157890443	1	23.138	KV452696.1	1813983	1-scf00001	1
AX-157890446	1	23.138	KV452696.1	1817118	1-scf00001	1
AX-157890465	1	23.138	KV452696.1	1847121	1-scf00001	1
AX-157890471	1	23.138	KV452696.1	1853837	1-scf00001	1
AX-157890472	1	23.138	KV452696.1	1860537	1-scf00001	1
AX-157810201	1	23.138	KV452696.1	1861740	1-scf00001	1
AX-157890477	1	23.138	KV452696.1	1865118	1-scf00001	1
AX-157890478	1	23.138	KV452696.1	1865328	1-scf00001	1
AX-157890497	1	23.138	KV452696.1	1914884	1-scf00001	1
AX-157774338	1	23.516	KV452696.1	1885398	1-scf00001	1
AX-157890489	1	23.516	KV452696.1	1888965	1-scf00001	1
AX-157890490	1	23.66	KV452696.1	1889709	1-scf00001	1
AX-158379092	1	23.879	KV452696.1	1894555	1-scf00001	1
AX-157890493	1	24.232	KV452696.1	1904544	1-scf00001	1
AX-157890494	1	24.232	KV452696.1	1906626	1-scf00001	1
AX-157890503	1	24.232	KV452696.1	1923799	1-scf00001	1
AX-157810202	1	24.232	KV452696.1	1929521	1-scf00001	1
AX-157717283	1	24.232	KV452696.1	1930478	1-scf00001	1
AX-157890506	1	24.232	KV452696.1	1932829	1-scf00001	1
AX-157890510	1	24.232	KV452696.1	1941222	1-scf00001	1
AX-157890512	1	24.232	KV452696.1	1945242	1-scf00001	1
AX-157890515	1	24.232	KV452696.1	1948365	1-scf00001	1
AX-157890519	1	24.451	KV452696.1	1952076	1-scf00001	1
AX-157890522	1	24.451	KV452696.1	1954983	1-scf00001	1
AX-157890526	1	24.451	KV452696.1	1959203	1-scf00001	1
AX-157708183	1	24.451	KV452696.1	1966185	1-scf00001	1
AX-157708182	1	24.451	KV452696.1	1966365	1-scf00001	1
AX-157890532	1	24.451	KV452696.1	1967942	1-scf00001	1
AX-157890534	1	24.451	KV452696.1	1969590	1-scf00001	1
AX-157890539	1	24.451	KV452696.1	1973451	1-scf00001	1

AX-157890553	1	24.451	KV452696.1	2000220	1-scf00001	1
AX-157742873	1	24.459	KV452696.1	1970302	1-scf00001	1
AX-157890541	1	24.502	KV452696.1	1976223	1-scf00001	1
AX-158388950	1	24.56	KV452696.1	1979431	1-scf00001	1
AX-157774009	1	24.56	KV452696.1	1980236	1-scf00001	1
AX-157890543	1	24.778	KV452696.1	1982471	1-scf00001	1
AX-157710358	1	24.778	KV452696.1	1986580	1-scf00001	1
AX-157713314	1	24.778	KV452696.1	2563658	1-scf00001	1
AX-157890871	1	24.778	KV452696.1	2645771	1-scf00001	1
AX-158404135	1	24.888	KV452696.1	1969248	1-scf00001	1
AX-157890535	1	24.888	KV452696.1	1971000	1-scf00001	1
AX-157890546	1	24.888	KV452696.1	1988325	1-scf00001	1
AX-157890551	1	24.888	KV452696.1	1996578	1-scf00001	1
AX-157742396	1	24.888	KV452696.1	1999239	1-scf00001	1
AX-157810206	1	24.888	KV452696.1	1999481	1-scf00001	1
AX-157810207	1	24.888	KV452696.1	1999520	1-scf00001	1
AX-157890554	1	24.888	KV452696.1	2003569	1-scf00001	1
AX-157748162	1	24.888	KV452696.1	2004910	1-scf00001	1
AX-157890557	1	24.888	KV452696.1	2006447	1-scf00001	1
AX-157890560	1	24.888	KV452696.1	2009116	1-scf00001	1
AX-157890562	1	24.888	KV452696.1	2010219	1-scf00001	1
AX-157890564	1	24.888	KV452696.1	2013437	1-scf00001	1
AX-157890565	1	24.888	KV452696.1	2014201	1-scf00001	1
AX-157890566	1	24.888	KV452696.1	2014971	1-scf00001	1
AX-157890567	1	24.888	KV452696.1	2015357	1-scf00001	1
AX-157890570	1	24.888	KV452696.1	2016948	1-scf00001	1
AX-157890571	1	24.888	KV452696.1	2017724	1-scf00001	1
AX-157890572	1	24.888	KV452696.1	2018437	1-scf00001	1
AX-157890574	1	24.888	KV452696.1	2018913	1-scf00001	1
AX-157890578	1	24.888	KV452696.1	2027427	1-scf00001	1
AX-157890587	1	24.888	KV452696.1	2042464	1-scf00001	1
AX-157890588	1	24.888	KV452696.1	2047006	1-scf00001	1
AX-157890590	1	24.888	KV452696.1	2048451	1-scf00001	1
AX-157890591	1	24.888	KV452696.1	2049656	1-scf00001	1
AX-157890549	1	25.215	KV452696.1	1994263	1-scf00001	1
AX-157890595	1	25.215	KV452696.1	2053251	1-scf00001	1
AX-157890603	1	25.215	KV452696.1	2065346	1-scf00001	1
AX-157890604	1	25.215	KV452696.1	2066241	1-scf00001	1
AX-157890609	1	25.215	KV452696.1	2069336	1-scf00001	1
AX-157890610	1	25.215	KV452696.1	2070140	1-scf00001	1
AX-157773101	1	25.215	KV452696.1	2072486	1-scf00001	1
AX-157890611	1	25.215	KV452696.1	2074048	1-scf00001	1
AX-157890612	1	25.215	KV452696.1	2075304	1-scf00001	1
AX-157810209	1	25.325	KV452696.1	2084896	1-scf00001	1
AX-157890619	1	25.325	KV452696.1	2090320	1-scf00001	1
AX-157890621	1	25.325	KV452696.1	2093945	1-scf00001	1
AX-157746192	1	25.434	KV452696.1	2088467	1-scf00001	1
AX-157890627	1	25.434	KV452696.1	2104356	1-scf00001	1
AX-157890630	1	25.434	KV452696.1	2106248	1-scf00001	1
AX-157890633	1	25.434	KV452696.1	2108735	1-scf00001	1
AX-157890634	1	25.434	KV452696.1	2109156	1-scf00001	1

AX-157704059	1	25.434	KV452696.1	2110772	1-scf00001	1
AX-157890652	1	25.434	KV452696.1	2128921	1-scf00001	1
AX-157890656	1	25.434	KV452696.1	2132090	1-scf00001	1
AX-157890828	1	25.434	KV452696.1	2560359	1-scf00001	1
AX-158403658	1	25.463	KV452696.1	2113157	1-scf00001	1
AX-158403660	1	25.491	KV452696.1	2113501	1-scf00001	1
AX-157810211	1	25.543	KV452696.1	2112719	1-scf00001	1
AX-157890638	1	25.543	KV452696.1	2115098	1-scf00001	1
AX-157890639	1	25.543	KV452696.1	2115863	1-scf00001	1
AX-157890642	1	25.543	KV452696.1	2117786	1-scf00001	1
AX-157890643	1	25.543	KV452696.1	2119252	1-scf00001	1
AX-157771685	1	25.543	KV452696.1	2123188	1-scf00001	1
AX-157890651	1	25.543	KV452696.1	2125402	1-scf00001	1
AX-157890667	1	25.543	KV452696.1	2141239	1-scf00001	1
AX-157890670	1	25.543	KV452696.1	2144197	1-scf00001	1
AX-157890657	1	25.638	KV452696.1	2133192	1-scf00001	1
AX-157890614	1	25.652	KV452696.1	2084140	1-scf00001	1
AX-157890617	1	25.652	KV452696.1	2087662	1-scf00001	1
AX-157890653	1	25.652	KV452696.1	2129091	1-scf00001	1
AX-157890659	1	25.652	KV452696.1	2136638	1-scf00001	1
AX-157890661	1	25.652	KV452696.1	2137133	1-scf00001	1
AX-157890662	1	25.652	KV452696.1	2137717	1-scf00001	1
AX-157890663	1	25.652	KV452696.1	2138176	1-scf00001	1
AX-157890664	1	25.652	KV452696.1	2138625	1-scf00001	1
AX-157890666	1	25.652	KV452696.1	2140590	1-scf00001	1
AX-157890668	1	25.652	KV452696.1	2142010	1-scf00001	1
AX-157890669	1	25.652	KV452696.1	2142351	1-scf00001	1
AX-157890673	1	25.652	KV452696.1	2148060	1-scf00001	1
AX-157714624	1	25.652	KV452696.1	2150367	1-scf00001	1
AX-157890675	1	25.652	KV452696.1	2152120	1-scf00001	1
AX-157890676	1	25.652	KV452696.1	2153737	1-scf00001	1
AX-157890677	1	25.652	KV452696.1	2155158	1-scf00001	1
AX-157890678	1	25.652	KV452696.1	2156629	1-scf00001	1
AX-157890683	1	25.652	KV452696.1	2165334	1-scf00001	1
AX-157890684	1	25.652	KV452696.1	2166004	1-scf00001	1
AX-157890685	1	25.652	KV452696.1	2166758	1-scf00001	1
AX-157890686	1	25.652	KV452696.1	2172926	1-scf00001	1
AX-157754890	1	25.652	KV452696.1	2180126	1-scf00001	1
AX-157890692	1	25.652	KV452696.1	2182636	1-scf00001	1
AX-157890702	1	25.652	KV452696.1	2188935	1-scf00001	1
AX-157890704	1	25.652	KV452696.1	2191602	1-scf00001	1
AX-157890705	1	25.652	KV452696.1	2192058	1-scf00001	1
AX-157890708	1	25.652	KV452696.1	2195971	1-scf00001	1
AX-157890709	1	25.652	KV452696.1	2196348	1-scf00001	1
AX-157890710	1	25.652	KV452696.1	2196801	1-scf00001	1
AX-157890712	1	25.652	KV452696.1	2198284	1-scf00001	1
AX-157890714	1	25.652	KV452696.1	2199150	1-scf00001	1
AX-157890717	1	25.652	KV452696.1	2207927	1-scf00001	1
AX-157810212	1	25.652	KV452696.1	2209230	1-scf00001	1
AX-157748054	1	25.652	KV452696.1	2214506	1-scf00001	1
AX-157890720	1	25.652	KV452696.1	2215437	1-scf00001	1

AX-157810213	1	25.652	KV452696.1	2216701	1-scf00001	1
AX-157890723	1	25.652	KV452696.1	2220155	1-scf00001	1
AX-157890724	1	25.652	KV452696.1	2221920	1-scf00001	1
AX-157890728	1	25.652	KV452696.1	2225202	1-scf00001	1
AX-157890729	1	25.652	KV452696.1	2226385	1-scf00001	1
AX-157890731	1	25.652	KV452696.1	2228688	1-scf00001	1
AX-157890734	1	25.652	KV452696.1	2231729	1-scf00001	1
AX-158379094	1	25.652	KV452696.1	2235452	1-scf00001	1
AX-157810215	1	25.652	KV452696.1	2235560	1-scf00001	1
AX-158403664	1	25.652	KV452696.1	2247519	1-scf00001	1
AX-157890742	1	25.652	KV452696.1	2264567	1-scf00001	1
AX-157890743	1	25.652	KV452696.1	2265790	1-scf00001	1
AX-157890744	1	25.652	KV452696.1	2266323	1-scf00001	1
AX-157810216	1	25.652	KV452696.1	2270201	1-scf00001	1
AX-158404141	1	25.652	KV452696.1	2274328	1-scf00001	1
AX-157890754	1	25.652	KV452696.1	2279784	1-scf00001	1
AX-157890760	1	25.652	KV452696.1	2293874	1-scf00001	1
AX-157890803	1	25.652	KV452696.1	2485895	1-scf00001	1
AX-157890824	1	25.652	KV452696.1	2542664	1-scf00001	1
AX-157810236	1	25.652	KV452696.1	2553625	1-scf00001	1
AX-157810238	1	25.652	KV452696.1	2600731	1-scf00001	1
AX-157810239	1	25.652	KV452696.1	2601382	1-scf00001	1
AX-157890857	1	25.652	KV452696.1	2627726	1-scf00001	1
AX-157746862	1	25.652	KV452696.1	2656620	1-scf00001	1
AX-158404151	1	25.652	KV452696.1	2661064	1-scf00001	1
AX-157890876	1	25.652	KV452696.1	2663617	1-scf00001	1
AX-157725146	1	25.652	KV452696.1	2667184	1-scf00001	1
AX-157890891	1	25.652	KV452696.1	2693129	1-scf00001	1
AX-157810252	1	25.652	KV452696.1	2741048	1-scf00001	1
AX-157890940	1	25.652	KV452696.1	2792482	1-scf00001	1
AX-157890982	1	25.652	KV452696.1	2870855	1-scf00001	1
AX-157890745	1	25.762	KV452696.1	2267086	1-scf00001	1
AX-158388953	1	25.871	KV452696.1	2055667	1-scf00001	1
AX-157890749	1	25.871	KV452696.1	2276586	1-scf00001	1
AX-157890751	1	25.871	KV452696.1	2277739	1-scf00001	1
AX-157890752	1	25.871	KV452696.1	2278448	1-scf00001	1
AX-157890753	1	25.871	KV452696.1	2279421	1-scf00001	1
AX-157890755	1	25.871	KV452696.1	2283934	1-scf00001	1
AX-157890757	1	25.871	KV452696.1	2285727	1-scf00001	1
AX-157890758	1	25.871	KV452696.1	2286434	1-scf00001	1
AX-157890764	1	25.871	KV452696.1	2299269	1-scf00001	1
AX-157810219	1	25.871	KV452696.1	2305834	1-scf00001	1
AX-157890767	1	25.871	KV452696.1	2307088	1-scf00001	1
AX-157810220	1	25.871	KV452696.1	2310254	1-scf00001	1
AX-157890770	1	25.871	KV452696.1	2315284	1-scf00001	1
AX-157890771	1	25.871	KV452696.1	2320462	1-scf00001	1
AX-157890772	1	25.871	KV452696.1	2326237	1-scf00001	1
AX-157890773	1	25.871	KV452696.1	2327728	1-scf00001	1
AX-157890774	1	25.871	KV452696.1	2332026	1-scf00001	1
AX-157810223	1	25.871	KV452696.1	2341275	1-scf00001	1
AX-157890782	1	25.871	KV452696.1	2358573	1-scf00001	1

AX-157890783	1	25.871	KV452696.1	2369579	1-scf00001	1
AX-157810225	1	25.871	KV452696.1	2370802	1-scf00001	1
AX-157890786	1	25.871	KV452696.1	2376469	1-scf00001	1
AX-157810227	1	25.871	KV452696.1	2376857	1-scf00001	1
AX-157890787	1	25.871	KV452696.1	2377576	1-scf00001	1
AX-157810228	1	25.871	KV452696.1	2384727	1-scf00001	1
AX-158381886	1	25.871	KV452696.1	2410866	1-scf00001	1
AX-157890794	1	25.871	KV452696.1	2411662	1-scf00001	1
AX-157890795	1	25.871	KV452696.1	2412258	1-scf00001	1
AX-157890796	1	25.871	KV452696.1	2413565	1-scf00001	1
AX-157810232	1	25.871	KV452696.1	2477072	1-scf00001	1
AX-157810233	1	25.871	KV452696.1	2477658	1-scf00001	1
AX-157890802	1	25.871	KV452696.1	2482288	1-scf00001	1
AX-157890813	1	25.871	KV452696.1	2523619	1-scf00001	1
AX-157890819	1	25.871	KV452696.1	2533517	1-scf00001	1
AX-157890822	1	25.871	KV452696.1	2540126	1-scf00001	1
AX-157754178	1	25.871	KV452696.1	4154421	1-scf00001	1
AX-157890834	1	25.889	KV452696.1	2576183	1-scf00001	1
AX-157890812	1	25.941	KV452696.1	2522443	1-scf00001	1
AX-157890831	1	25.941	KV452696.1	2567824	1-scf00001	1
AX-157712959	1	25.941	KV452696.1	2568589	1-scf00001	1
AX-157890835	1	25.941	KV452696.1	2577830	1-scf00001	1
AX-157890836	1	25.941	KV452696.1	2578036	1-scf00001	1
AX-157890688	1	25.98	KV452696.1	2173784	1-scf00001	1
AX-157890718	1	25.98	KV452696.1	2212568	1-scf00001	1
AX-157890719	1	25.98	KV452696.1	2213465	1-scf00001	1
AX-157890741	1	25.98	KV452696.1	2259222	1-scf00001	1
AX-157890815	1	25.98	KV452696.1	2526830	1-scf00001	1
AX-157890823	1	25.98	KV452696.1	2541478	1-scf00001	1
AX-157890830	1	25.98	KV452696.1	2563158	1-scf00001	1
AX-157890838	1	25.98	KV452696.1	2580424	1-scf00001	1
AX-157720980	1	25.98	KV452696.1	2583173	1-scf00001	1
AX-157890839	1	25.98	KV452696.1	2584025	1-scf00001	1
AX-157890840	1	25.98	KV452696.1	2584409	1-scf00001	1
AX-157890842	1	25.98	KV452696.1	2585202	1-scf00001	1
AX-157890849	1	25.98	KV452696.1	2602602	1-scf00001	1
AX-157890851	1	25.98	KV452696.1	2608769	1-scf00001	1
AX-157890852	1	25.98	KV452696.1	2610932	1-scf00001	1
AX-157890853	1	25.98	KV452696.1	2612778	1-scf00001	1
AX-157713847	1	25.98	KV452696.1	2621703	1-scf00001	1
AX-157890856	1	25.98	KV452696.1	2627039	1-scf00001	1
AX-157890858	1	25.98	KV452696.1	2628465	1-scf00001	1
AX-157890859	1	25.98	KV452696.1	2629017	1-scf00001	1
AX-157890860	1	25.98	KV452696.1	2629612	1-scf00001	1
AX-157890861	1	25.98	KV452696.1	2632490	1-scf00001	1
AX-157890863	1	25.98	KV452696.1	2633932	1-scf00001	1
AX-157715374	1	25.98	KV452696.1	2636068	1-scf00001	1
AX-157890865	1	25.98	KV452696.1	2636665	1-scf00001	1
AX-157810245	1	25.98	KV452696.1	2637584	1-scf00001	1
AX-157775331	1	25.98	KV452696.1	2637823	1-scf00001	1
AX-157810246	1	25.98	KV452696.1	2638151	1-scf00001	1

AX-157890867	1	25.98	KV452696.1	2640303	1-scf00001	1
AX-157890868	1	25.98	KV452696.1	2642081	1-scf00001	1
AX-157890870	1	25.98	KV452696.1	2644887	1-scf00001	1
AX-157890888	1	25.98	KV452696.1	2688942	1-scf00001	1
AX-157890894	1	25.98	KV452696.1	2698610	1-scf00001	1
AX-157890899	1	25.98	KV452696.1	2704058	1-scf00001	1
AX-157890901	1	25.98	KV452696.1	2707591	1-scf00001	1
AX-157890902	1	25.98	KV452696.1	2708541	1-scf00001	1
AX-157890904	1	25.98	KV452696.1	2712704	1-scf00001	1
AX-157890914	1	25.98	KV452696.1	2740853	1-scf00001	1
AX-157890920	1	25.98	KV452696.1	2750290	1-scf00001	1
AX-157890950	1	25.98	KV452696.1	2800657	1-scf00001	1
AX-157725094	1	25.98	KV452696.1	2839938	1-scf00001	1
AX-157890916	1	25.988	KV452696.1	2742516	1-scf00001	1
AX-157874233	1	25.995	KV452696.1	2273154	1-scf00001	1
AX-157810222	1	25.995	KV452696.1	2338034	1-scf00001	1
AX-157890779	1	25.995	KV452696.1	2343851	1-scf00001	1
AX-157810229	1	25.995	KV452696.1	2464471	1-scf00001	1
AX-157810231	1	25.995	KV452696.1	2465972	1-scf00001	1
AX-157890814	1	25.995	KV452696.1	2525306	1-scf00001	1
AX-157874234	1	25.995	KV452696.1	2556307	1-scf00001	1
AX-157890829	1	25.995	KV452696.1	2562002	1-scf00001	1
AX-157890909	1	25.995	KV452696.1	2732545	1-scf00001	1
AX-157890917	1	25.995	KV452696.1	2742820	1-scf00001	1
AX-157890931	1	25.995	KV452696.1	2776483	1-scf00001	1
AX-157890943	1	25.995	KV452696.1	2795834	1-scf00001	1
AX-157890944	1	25.995	KV452696.1	2796442	1-scf00001	1
AX-157890918	1	26.021	KV452696.1	2745330	1-scf00001	1
AX-157890555	1	26.041	KV452696.1	2005376	1-scf00001	1
AX-157747933	1	26.041	KV452696.1	2008210	1-scf00001	1
AX-157890879	1	26.041	KV452696.1	2669271	1-scf00001	1
AX-157890893	1	26.041	KV452696.1	2698358	1-scf00001	1
AX-157890895	1	26.041	KV452696.1	2699824	1-scf00001	1
AX-157890921	1	26.041	KV452696.1	2752331	1-scf00001	1
AX-157890924	1	26.041	KV452696.1	2753815	1-scf00001	1
AX-157890655	1	26.058	KV452696.1	2131205	1-scf00001	1
AX-157771692	1	26.058	KV452696.1	2179147	1-scf00001	1
AX-157890759	1	26.058	KV452696.1	2289130	1-scf00001	1
AX-157890806	1	26.058	KV452696.1	2500007	1-scf00001	1
AX-157890844	1	26.058	KV452696.1	2595421	1-scf00001	1
AX-157890845	1	26.058	KV452696.1	2598363	1-scf00001	1
AX-157890846	1	26.058	KV452696.1	2600029	1-scf00001	1
AX-157890854	1	26.058	KV452696.1	2613504	1-scf00001	1
AX-157762851	1	26.058	KV452696.1	2621345	1-scf00001	1
AX-157890855	1	26.058	KV452696.1	2625761	1-scf00001	1
AX-157890873	1	26.058	KV452696.1	2650293	1-scf00001	1
AX-157716740	1	26.058	KV452696.1	2652415	1-scf00001	1
AX-157890878	1	26.058	KV452696.1	2668679	1-scf00001	1
AX-157883042	1	26.058	KV452696.1	2753258	1-scf00001	1
AX-157890925	1	26.058	KV452696.1	2756207	1-scf00001	1
AX-157890947	1	26.058	KV452696.1	2798884	1-scf00001	1

AX-157890948	1	26.058	KV452696.1	2799180	1-scf00001	1
AX-158421724	1	26.069	KV452696.1	2767577	1-scf00001	1
AX-157890540	1	26.089	KV452696.1	1975652	1-scf00001	1
AX-157810241	1	26.089	KV452696.1	2621894	1-scf00001	1
AX-157890930	1	26.089	KV452696.1	2772080	1-scf00001	1
AX-157746771	1	26.089	KV452696.1	2774262	1-scf00001	1
AX-157723341	1	26.089	KV452696.1	2775790	1-scf00001	1
AX-157721480	1	26.089	KV452696.1	2776836	1-scf00001	1
AX-157890932	1	26.089	KV452696.1	2777399	1-scf00001	1
AX-158404157	1	26.089	KV452696.1	2778725	1-scf00001	1
AX-157890933	1	26.089	KV452696.1	2780231	1-scf00001	1
AX-157810255	1	26.089	KV452696.1	2783436	1-scf00001	1
AX-157890936	1	26.089	KV452696.1	2783898	1-scf00001	1
AX-157810256	1	26.089	KV452696.1	2784103	1-scf00001	1
AX-157890937	1	26.089	KV452696.1	2786913	1-scf00001	1
AX-157890938	1	26.089	KV452696.1	2787659	1-scf00001	1
AX-157890939	1	26.089	KV452696.1	2788591	1-scf00001	1
AX-157890945	1	26.089	KV452696.1	2797797	1-scf00001	1
AX-157890949	1	26.089	KV452696.1	2800424	1-scf00001	1
AX-157890952	1	26.089	KV452696.1	2805082	1-scf00001	1
AX-157890953	1	26.089	KV452696.1	2807907	1-scf00001	1
AX-157744906	1	26.089	KV452696.1	2808342	1-scf00001	1
AX-157725223	1	26.089	KV452696.1	2808710	1-scf00001	1
AX-157716820	1	26.308	KV452696.1	2811559	1-scf00001	1
AX-157890955	1	26.308	KV452696.1	2815415	1-scf00001	1
AX-157890959	1	26.308	KV452696.1	2821418	1-scf00001	1
AX-157890963	1	26.308	KV452696.1	2830056	1-scf00001	1
AX-157890964	1	26.308	KV452696.1	2831133	1-scf00001	1
AX-157890965	1	26.308	KV452696.1	2832321	1-scf00001	1
AX-157890974	1	26.308	KV452696.1	2864627	1-scf00001	1
AX-157890979	1	26.308	KV452696.1	2867965	1-scf00001	1
AX-157890980	1	26.308	KV452696.1	2868299	1-scf00001	1
AX-157890990	1	26.308	KV452696.1	2884803	1-scf00001	1
AX-157890993	1	26.308	KV452696.1	2888365	1-scf00001	1
AX-157890960	1	26.527	KV452696.1	2821785	1-scf00001	1
AX-157890988	1	26.527	KV452696.1	2880388	1-scf00001	1
AX-157890992	1	26.527	KV452696.1	2887286	1-scf00001	1
AX-157890996	1	26.527	KV452696.1	2891227	1-scf00001	1
AX-157890998	1	26.527	KV452696.1	2892448	1-scf00001	1
AX-157746582	1	26.527	KV452696.1	2897171	1-scf00001	1
AX-157891000	1	26.527	KV452696.1	2897779	1-scf00001	1
AX-157891002	1	26.527	KV452696.1	2900069	1-scf00001	1
AX-157891003	1	26.527	KV452696.1	2903503	1-scf00001	1
AX-157891005	1	26.527	KV452696.1	2904334	1-scf00001	1
AX-157891006	1	26.527	KV452696.1	2905421	1-scf00001	1
AX-157891007	1	26.527	KV452696.1	2906830	1-scf00001	1
AX-157891010	1	26.527	KV452696.1	2913005	1-scf00001	1
AX-157891012	1	26.527	KV452696.1	2914176	1-scf00001	1
AX-157891014	1	26.527	KV452696.1	2917148	1-scf00001	1
AX-157891018	1	26.527	KV452696.1	2921156	1-scf00001	1
AX-157891021	1	26.527	KV452696.1	2927043	1-scf00001	1

AX-157891024	1	26.527	KV452696.1	2928712	1-scf00001	1
AX-157891030	1	26.527	KV452696.1	2935427	1-scf00001	1
AX-157891031	1	26.527	KV452696.1	2935523	1-scf00001	1
AX-157891034	1	26.527	KV452696.1	2936738	1-scf00001	1
AX-157891035	1	26.527	KV452696.1	2936977	1-scf00001	1
AX-157891038	1	26.527	KV452696.1	2941558	1-scf00001	1
AX-157891039	1	26.527	KV452696.1	2945182	1-scf00001	1
AX-157891048	1	26.527	KV452696.1	2952828	1-scf00001	1
AX-157891063	1	26.527	KV452696.1	2978192	1-scf00001	1
AX-157810272	1	26.527	KV452696.1	2978648	1-scf00001	1
AX-157891065	1	26.527	KV452696.1	2979356	1-scf00001	1
AX-157891066	1	26.527	KV452696.1	2981598	1-scf00001	1
AX-157891068	1	26.527	KV452696.1	2987063	1-scf00001	1
AX-157891082	1	26.527	KV452696.1	3017202	1-scf00001	1
AX-157891084	1	26.527	KV452696.1	3024160	1-scf00001	1
AX-158403717	1	26.527	KV452696.1	4400336	1-scf00001	1
AX-157892323	1	26.527	KV452698.1	666489	1-scf00002	1
AX-157892477	1	26.527	KV452697.1	102792	1-scf00003	1
AX-157891086	1	26.636	KV452696.1	3028613	1-scf00001	1
AX-157891087	1	26.636	KV452696.1	3032316	1-scf00001	1
AX-157891088	1	26.636	KV452696.1	3033812	1-scf00001	1
AX-157891090	1	26.636	KV452696.1	3035242	1-scf00001	1
AX-157891093	1	26.636	KV452696.1	3039241	1-scf00001	1
AX-157891094	1	26.636	KV452696.1	3040175	1-scf00001	1
AX-157890975	1	26.745	KV452696.1	2865432	1-scf00001	1
AX-157890976	1	26.745	KV452696.1	2866415	1-scf00001	1
AX-157890977	1	26.745	KV452696.1	2866882	1-scf00001	1
AX-157890994	1	26.745	KV452696.1	2889269	1-scf00001	1
AX-157891044	1	26.745	KV452696.1	2950264	1-scf00001	1
AX-157891047	1	26.745	KV452696.1	2952071	1-scf00001	1
AX-157723083	1	26.745	KV452696.1	2953774	1-scf00001	1
AX-157891049	1	26.745	KV452696.1	2954287	1-scf00001	1
AX-157891050	1	26.745	KV452696.1	2954672	1-scf00001	1
AX-157810268	1	26.745	KV452696.1	2955861	1-scf00001	1
AX-157810269	1	26.745	KV452696.1	2956857	1-scf00001	1
AX-157891053	1	26.745	KV452696.1	2958544	1-scf00001	1
AX-157891078	1	26.745	KV452696.1	3006899	1-scf00001	1
AX-157891079	1	26.745	KV452696.1	3007158	1-scf00001	1
AX-157891098	1	26.745	KV452696.1	3046432	1-scf00001	1
AX-157891101	1	26.745	KV452696.1	3049934	1-scf00001	1
AX-157810274	1	26.745	KV452696.1	3050033	1-scf00001	1
AX-157891102	1	26.745	KV452696.1	3050332	1-scf00001	1
AX-157891103	1	26.745	KV452696.1	3050732	1-scf00001	1
AX-157891115	1	26.745	KV452696.1	3069089	1-scf00001	1
AX-157891117	1	26.745	KV452696.1	3070765	1-scf00001	1
AX-157891118	1	26.745	KV452696.1	3074253	1-scf00001	1
AX-157891229	1	26.745	KV452696.1	3302452	1-scf00001	1
AX-157891279	1	26.745	KV452696.1	3372223	1-scf00001	1
AX-157891280	1	26.745	KV452696.1	3372984	1-scf00001	1
AX-157891281	1	26.745	KV452696.1	3373460	1-scf00001	1
AX-157891283	1	26.745	KV452696.1	3374810	1-scf00001	1

AX-157891284	1	26.745	KV452696.1	3375272	1-scf00001	1
AX-157891287	1	26.745	KV452696.1	3379260	1-scf00001	1
AX-157891311	1	26.745	KV452696.1	3484315	1-scf00001	1
AX-157891324	1	26.745	KV452696.1	3508868	1-scf00001	1
AX-157891333	1	26.745	KV452696.1	3520348	1-scf00001	1
AX-157891339	1	26.745	KV452696.1	3526594	1-scf00001	1
AX-157891366	1	26.745	KV452696.1	3581574	1-scf00001	1
AX-157891370	1	26.745	KV452696.1	3608163	1-scf00001	1
AX-157891505	1	26.745	KV452696.1	3804068	1-scf00001	1
AX-157891120	1	26.778	KV452696.1	3075193	1-scf00001	1
AX-157891128	1	26.789	KV452696.1	3098196	1-scf00001	1
AX-157891129	1	26.822	KV452696.1	3100212	1-scf00001	1
AX-157740780	1	26.854	KV452696.1	3058115	1-scf00001	1
AX-157891121	1	26.854	KV452696.1	3078153	1-scf00001	1
AX-157891132	1	26.854	KV452696.1	3102460	1-scf00001	1
AX-157891134	1	26.854	KV452696.1	3107323	1-scf00001	1
AX-157891135	1	26.854	KV452696.1	3109408	1-scf00001	1
AX-157891136	1	26.854	KV452696.1	3109848	1-scf00001	1
AX-157891139	1	26.854	KV452696.1	3113734	1-scf00001	1
AX-157883044	1	26.854	KV452696.1	3115719	1-scf00001	1
AX-157891141	1	26.854	KV452696.1	3116493	1-scf00001	1
AX-157746497	1	26.854	KV452696.1	3119652	1-scf00001	1
AX-157738938	1	26.854	KV452696.1	3122154	1-scf00001	1
AX-157745144	1	26.854	KV452696.1	3122968	1-scf00001	1
AX-157891143	1	26.854	KV452696.1	3123492	1-scf00001	1
AX-157891145	1	26.854	KV452696.1	3128136	1-scf00001	1
AX-157891146	1	26.854	KV452696.1	3129373	1-scf00001	1
AX-157891148	1	26.854	KV452696.1	3130697	1-scf00001	1
AX-157891150	1	26.854	KV452696.1	3139226	1-scf00001	1
AX-157891156	1	26.854	KV452696.1	3145163	1-scf00001	1
AX-157891157	1	26.854	KV452696.1	3146997	1-scf00001	1
AX-157891160	1	26.854	KV452696.1	3152976	1-scf00001	1
AX-157810287	1	26.854	KV452696.1	3154993	1-scf00001	1
AX-157891161	1	26.854	KV452696.1	3155978	1-scf00001	1
AX-157864311	1	26.854	KV452696.1	3156990	1-scf00001	1
AX-157891163	1	26.854	KV452696.1	3158346	1-scf00001	1
AX-157864312	1	26.854	KV452696.1	3158727	1-scf00001	1
AX-157864313	1	26.854	KV452696.1	3159023	1-scf00001	1
AX-157891164	1	26.854	KV452696.1	3159706	1-scf00001	1
AX-157864314	1	26.854	KV452696.1	3163722	1-scf00001	1
AX-157864315	1	26.854	KV452696.1	3165356	1-scf00001	1
AX-157891168	1	26.854	KV452696.1	3175475	1-scf00001	1
AX-157891169	1	26.854	KV452696.1	3176813	1-scf00001	1
AX-157891170	1	26.854	KV452696.1	3177441	1-scf00001	1
AX-157891182	1	26.854	KV452696.1	3188097	1-scf00001	1
AX-157891186	1	26.854	KV452696.1	3193218	1-scf00001	1
AX-157891190	1	26.854	KV452696.1	3197028	1-scf00001	1
AX-157891191	1	26.854	KV452696.1	3199249	1-scf00001	1
AX-157810307	1	26.854	KV452696.1	3678018	1-scf00001	1
AX-157891473	1	26.854	KV452696.1	3760109	1-scf00001	1
AX-157891635	1	26.854	KV452696.1	3948943	1-scf00001	1

AX-157891641	1	26.854	KV452696.1	3961715	1-scf00001	1
AX-157891662	1	26.854	KV452696.1	3995361	1-scf00001	1
AX-157868456	1	26.886	KV452696.1	3195373	1-scf00001	1
AX-157708259	1	26.919	KV452696.1	2848046	1-scf00001	1
AX-157891064	1	26.919	KV452696.1	2978397	1-scf00001	1
AX-157810277	1	26.919	KV452696.1	3061832	1-scf00001	1
AX-157891204	1	26.919	KV452696.1	3228462	1-scf00001	1
AX-157891219	1	26.919	KV452696.1	3290257	1-scf00001	1
AX-157891228	1	26.919	KV452696.1	3301997	1-scf00001	1
AX-157891233	1	26.919	KV452696.1	3314414	1-scf00001	1
AX-157723209	1	26.919	KV452696.1	3400556	1-scf00001	1
AX-157891342	1	26.919	KV452696.1	3530980	1-scf00001	1
AX-157891343	1	26.919	KV452696.1	3533019	1-scf00001	1
AX-157891348	1	26.919	KV452696.1	3544228	1-scf00001	1
AX-157891772	1	26.919	KV452696.1	4161612	1-scf00001	1
AX-157892020	1	26.919	KV452696.1	4566080	1-scf00001	1
AX-157892277	1	26.919	KV452698.1	568104	1-scf00002	1
AX-157892440	1	26.919	KV452697.1	4891	1-scf00003	1
AX-157892480	1	26.919	KV452697.1	107494	1-scf00003	1
AX-157810290	1	26.927	KV452696.1	3241220	1-scf00001	1
AX-157891041	1	26.963	KV452696.1	2947309	1-scf00001	1
AX-157891144	1	26.963	KV452696.1	3127296	1-scf00001	1
AX-157891153	1	26.963	KV452696.1	3143772	1-scf00001	1
AX-157810293	1	26.963	KV452696.1	3272541	1-scf00001	1
AX-157891217	1	26.963	KV452696.1	3286811	1-scf00001	1
AX-157716586	1	26.963	KV452696.1	3295048	1-scf00001	1
AX-157891224	1	26.963	KV452696.1	3295740	1-scf00001	1
AX-157891230	1	26.963	KV452696.1	3308293	1-scf00001	1
AX-157776116	1	26.963	KV452696.1	3308982	1-scf00001	1
AX-157810295	1	26.963	KV452696.1	3310685	1-scf00001	1
AX-157891232	1	26.963	KV452696.1	3311647	1-scf00001	1
AX-157891234	1	26.963	KV452696.1	3314960	1-scf00001	1
AX-157868457	1	26.963	KV452696.1	3315615	1-scf00001	1
AX-157868458	1	26.963	KV452696.1	3315919	1-scf00001	1
AX-157891236	1	26.963	KV452696.1	3319742	1-scf00001	1
AX-157891238	1	26.963	KV452696.1	3322813	1-scf00001	1
AX-157891240	1	26.963	KV452696.1	3330364	1-scf00001	1
AX-157810297	1	26.963	KV452696.1	3330714	1-scf00001	1
AX-157891241	1	26.963	KV452696.1	3331435	1-scf00001	1
AX-157891244	1	26.963	KV452696.1	3333772	1-scf00001	1
AX-157891246	1	26.963	KV452696.1	3334790	1-scf00001	1
AX-157891248	1	26.963	KV452696.1	3336083	1-scf00001	1
AX-157891250	1	26.963	KV452696.1	3338915	1-scf00001	1
AX-157891251	1	26.963	KV452696.1	3339257	1-scf00001	1
AX-157891252	1	26.963	KV452696.1	3339827	1-scf00001	1
AX-157891253	1	26.963	KV452696.1	3341130	1-scf00001	1
AX-157891254	1	26.963	KV452696.1	3342250	1-scf00001	1
AX-157891259	1	26.963	KV452696.1	3345136	1-scf00001	1
AX-157891261	1	26.963	KV452696.1	3347954	1-scf00001	1
AX-157891270	1	26.963	KV452696.1	3356087	1-scf00001	1
AX-157891276	1	26.963	KV452696.1	3369967	1-scf00001	1

AX-157891278	1	26.963	KV452696.1	3371349	1-scf00001	1
AX-157891282	1	26.963	KV452696.1	3374229	1-scf00001	1
AX-157891295	1	26.963	KV452696.1	3397495	1-scf00001	1
AX-157891299	1	26.963	KV452696.1	3437548	1-scf00001	1
AX-157891314	1	26.963	KV452696.1	3494775	1-scf00001	1
AX-157891318	1	26.963	KV452696.1	3498442	1-scf00001	1
AX-157891319	1	26.963	KV452696.1	3501458	1-scf00001	1
AX-157810301	1	26.963	KV452696.1	3501949	1-scf00001	1
AX-157891320	1	26.963	KV452696.1	3503760	1-scf00001	1
AX-157891323	1	26.963	KV452696.1	3505998	1-scf00001	1
AX-157891326	1	26.963	KV452696.1	3509905	1-scf00001	1
AX-157891327	1	26.963	KV452696.1	3512138	1-scf00001	1
AX-157810302	1	26.963	KV452696.1	3512197	1-scf00001	1
AX-157891331	1	26.963	KV452696.1	3519023	1-scf00001	1
AX-157891336	1	26.963	KV452696.1	3523021	1-scf00001	1
AX-157891345	1	26.963	KV452696.1	3539460	1-scf00001	1
AX-157810303	1	26.963	KV452696.1	3546775	1-scf00001	1
AX-157891349	1	26.963	KV452696.1	3546921	1-scf00001	1
AX-157810304	1	26.963	KV452696.1	3565390	1-scf00001	1
AX-157774819	1	26.963	KV452696.1	3565688	1-scf00001	1
AX-157891383	1	26.963	KV452696.1	3625553	1-scf00001	1
AX-157891398	1	26.963	KV452696.1	3647385	1-scf00001	1
AX-158379099	1	26.963	KV452696.1	3648352	1-scf00001	1
AX-157891399	1	26.963	KV452696.1	3648433	1-scf00001	1
AX-157891447	1	26.963	KV452696.1	3713298	1-scf00001	1
AX-157891449	1	26.963	KV452696.1	3713929	1-scf00001	1
AX-157891588	1	26.963	KV452696.1	3896129	1-scf00001	1
AX-157868465	1	26.963	KV452696.1	4153178	1-scf00001	1
AX-158388964	1	26.963	KV452696.1	4163165	1-scf00001	1
AX-157891354	1	26.97	KV452696.1	3555819	1-scf00001	1
AX-157743148	1	26.975	KV452696.1	3556961	1-scf00001	1
AX-157891358	1	26.978	KV452696.1	3563660	1-scf00001	1
AX-157891364	1	26.982	KV452696.1	3578215	1-scf00001	1
AX-157891040	1	26.988	KV452696.1	2945639	1-scf00001	1
AX-157891092	1	26.988	KV452696.1	3038814	1-scf00001	1
AX-157891137	1	26.988	KV452696.1	3112827	1-scf00001	1
AX-157891267	1	26.988	KV452696.1	3353850	1-scf00001	1
AX-157891293	1	26.988	KV452696.1	3390355	1-scf00001	1
AX-157891335	1	26.988	KV452696.1	3522520	1-scf00001	1
AX-157891338	1	26.988	KV452696.1	3524321	1-scf00001	1
AX-157891380	1	26.988	KV452696.1	3623090	1-scf00001	1
AX-157891381	1	26.992	KV452696.1	3623824	1-scf00001	1
AX-157868459	1	26.997	KV452696.1	3323654	1-scf00001	1
AX-157891256	1	26.997	KV452696.1	3343303	1-scf00001	1
AX-157891390	1	26.997	KV452696.1	3633613	1-scf00001	1
AX-157891392	1	27.003	KV452696.1	3638036	1-scf00001	1
AX-157891393	1	27.008	KV452696.1	3639628	1-scf00001	1
AX-157891394	1	27.04	KV452696.1	3641044	1-scf00001	1
AX-157891225	1	27.073	KV452696.1	3297768	1-scf00001	1
AX-157891227	1	27.073	KV452696.1	3300651	1-scf00001	1
AX-157891255	1	27.073	KV452696.1	3342815	1-scf00001	1

AX-157891321	1	27.073	KV452696.1	3504704	1-scf00001	1
AX-157891322	1	27.073	KV452696.1	3505344	1-scf00001	1
AX-158379098	1	27.073	KV452696.1	3505549	1-scf00001	1
AX-157707986	1	27.073	KV452696.1	3520876	1-scf00001	1
AX-157891356	1	27.073	KV452696.1	3558641	1-scf00001	1
AX-157891368	1	27.073	KV452696.1	3590470	1-scf00001	1
AX-157891374	1	27.073	KV452696.1	3616140	1-scf00001	1
AX-157891386	1	27.073	KV452696.1	3630284	1-scf00001	1
AX-157891396	1	27.073	KV452696.1	3644066	1-scf00001	1
AX-157891408	1	27.073	KV452696.1	3665353	1-scf00001	1
AX-157891410	1	27.073	KV452696.1	3666281	1-scf00001	1
AX-157874242	1	27.073	KV452696.1	3668288	1-scf00001	1
AX-157891411	1	27.073	KV452696.1	3668324	1-scf00001	1
AX-157891412	1	27.073	KV452696.1	3669070	1-scf00001	1
AX-157891422	1	27.073	KV452696.1	3688504	1-scf00001	1
AX-157740513	1	27.073	KV452696.1	3693807	1-scf00001	1
AX-157891425	1	27.073	KV452696.1	3695914	1-scf00001	1
AX-157891426	1	27.073	KV452696.1	3696398	1-scf00001	1
AX-157891427	1	27.073	KV452696.1	3697089	1-scf00001	1
AX-157891429	1	27.073	KV452696.1	3697420	1-scf00001	1
AX-157891430	1	27.073	KV452696.1	3699228	1-scf00001	1
AX-157891433	1	27.073	KV452696.1	3701100	1-scf00001	1
AX-157891434	1	27.073	KV452696.1	3701344	1-scf00001	1
AX-157810309	1	27.073	KV452696.1	3701983	1-scf00001	1
AX-157891439	1	27.073	KV452696.1	3704621	1-scf00001	1
AX-157773019	1	27.073	KV452696.1	3704808	1-scf00001	1
AX-157891441	1	27.073	KV452696.1	3706374	1-scf00001	1
AX-157891442	1	27.073	KV452696.1	3707700	1-scf00001	1
AX-157891443	1	27.073	KV452696.1	3708235	1-scf00001	1
AX-157891455	1	27.073	KV452696.1	3720332	1-scf00001	1
AX-157891456	1	27.073	KV452696.1	3720834	1-scf00001	1
AX-157891458	1	27.073	KV452696.1	3721630	1-scf00001	1
AX-157724487	1	27.073	KV452696.1	3730998	1-scf00001	1
AX-157891466	1	27.073	KV452696.1	3749161	1-scf00001	1
AX-157891469	1	27.073	KV452696.1	3754978	1-scf00001	1
AX-158388962	1	27.073	KV452696.1	3766723	1-scf00001	1
AX-157891478	1	27.073	KV452696.1	3769325	1-scf00001	1
AX-157891480	1	27.073	KV452696.1	3771134	1-scf00001	1
AX-157891481	1	27.073	KV452696.1	3771616	1-scf00001	1
AX-157891482	1	27.073	KV452696.1	3773082	1-scf00001	1
AX-157891483	1	27.073	KV452696.1	3773579	1-scf00001	1
AX-157891486	1	27.073	KV452696.1	3778602	1-scf00001	1
AX-157891487	1	27.073	KV452696.1	3781609	1-scf00001	1
AX-157891488	1	27.073	KV452696.1	3782184	1-scf00001	1
AX-157891489	1	27.073	KV452696.1	3783154	1-scf00001	1
AX-157891490	1	27.073	KV452696.1	3783749	1-scf00001	1
AX-158388963	1	27.073	KV452696.1	3784334	1-scf00001	1
AX-157891491	1	27.073	KV452696.1	3784542	1-scf00001	1
AX-157891492	1	27.073	KV452696.1	3784843	1-scf00001	1
AX-157891497	1	27.073	KV452696.1	3790827	1-scf00001	1
AX-157891498	1	27.073	KV452696.1	3791324	1-scf00001	1

AX-157891500	1	27.073	KV452696.1	3797684	1-scf00001	1
AX-157891502	1	27.073	KV452696.1	3800596	1-scf00001	1
AX-157891506	1	27.073	KV452696.1	3804726	1-scf00001	1
AX-157891508	1	27.073	KV452696.1	3812295	1-scf00001	1
AX-157891509	1	27.073	KV452696.1	3812802	1-scf00001	1
AX-157891510	1	27.073	KV452696.1	3814361	1-scf00001	1
AX-157709680	1	27.073	KV452696.1	3817357	1-scf00001	1
AX-157891516	1	27.073	KV452696.1	3819829	1-scf00001	1
AX-157891517	1	27.073	KV452696.1	3820543	1-scf00001	1
AX-157891519	1	27.073	KV452696.1	3822509	1-scf00001	1
AX-157891521	1	27.073	KV452696.1	3826798	1-scf00001	1
AX-157891522	1	27.073	KV452696.1	3827107	1-scf00001	1
AX-157891523	1	27.073	KV452696.1	3827714	1-scf00001	1
AX-157891524	1	27.073	KV452696.1	3828208	1-scf00001	1
AX-157891525	1	27.073	KV452696.1	3830947	1-scf00001	1
AX-157891526	1	27.073	KV452696.1	3831109	1-scf00001	1
AX-157891527	1	27.073	KV452696.1	3831478	1-scf00001	1
AX-157891530	1	27.073	KV452696.1	3834321	1-scf00001	1
AX-157891532	1	27.073	KV452696.1	3835941	1-scf00001	1
AX-157891535	1	27.073	KV452696.1	3837524	1-scf00001	1
AX-157891536	1	27.073	KV452696.1	3837920	1-scf00001	1
AX-157891537	1	27.073	KV452696.1	3838794	1-scf00001	1
AX-157891538	1	27.073	KV452696.1	3841615	1-scf00001	1
AX-157891539	1	27.073	KV452696.1	3842053	1-scf00001	1
AX-157891540	1	27.073	KV452696.1	3842482	1-scf00001	1
AX-157891541	1	27.073	KV452696.1	3842560	1-scf00001	1
AX-157891545	1	27.073	KV452696.1	3845450	1-scf00001	1
AX-157743206	1	27.073	KV452696.1	3852574	1-scf00001	1
AX-157891547	1	27.073	KV452696.1	3853906	1-scf00001	1
AX-157891548	1	27.073	KV452696.1	3854365	1-scf00001	1
AX-157891549	1	27.073	KV452696.1	3854777	1-scf00001	1
AX-157891550	1	27.073	KV452696.1	3855267	1-scf00001	1
AX-157891551	1	27.073	KV452696.1	3855902	1-scf00001	1
AX-157891553	1	27.073	KV452696.1	3858771	1-scf00001	1
AX-157891554	1	27.073	KV452696.1	3859088	1-scf00001	1
AX-157891556	1	27.073	KV452696.1	3860911	1-scf00001	1
AX-157891557	1	27.073	KV452696.1	3862665	1-scf00001	1
AX-157891567	1	27.073	KV452696.1	3867577	1-scf00001	1
AX-157891568	1	27.073	KV452696.1	3868076	1-scf00001	1
AX-157891570	1	27.073	KV452696.1	3869784	1-scf00001	1
AX-157774645	1	27.073	KV452696.1	3869941	1-scf00001	1
AX-157891571	1	27.073	KV452696.1	3870498	1-scf00001	1
AX-157891572	1	27.073	KV452696.1	3873093	1-scf00001	1
AX-157891575	1	27.073	KV452696.1	3875366	1-scf00001	1
AX-157891576	1	27.073	KV452696.1	3876011	1-scf00001	1
AX-157891577	1	27.073	KV452696.1	3876982	1-scf00001	1
AX-157891578	1	27.073	KV452696.1	3881722	1-scf00001	1
AX-157891581	1	27.073	KV452696.1	3886512	1-scf00001	1
AX-157891582	1	27.073	KV452696.1	3889787	1-scf00001	1
AX-157891638	1	27.073	KV452696.1	3957069	1-scf00001	1
AX-157891653	1	27.073	KV452696.1	3978173	1-scf00001	1

AX-157891703	1	27.073	KV452696.1	4076037	1-scf00001	1
AX-157705467	1	27.073	3ML01029866	69	scf05488_1	UN
AX-157891131	1	27.093	KV452696.1	3102020	1-scf00001	1
AX-157891359	1	27.093	KV452696.1	3569617	1-scf00001	1
AX-157891377	1	27.093	KV452696.1	3618193	1-scf00001	1
AX-157891580	1	27.093	KV452696.1	3884884	1-scf00001	1
AX-157891583	1	27.093	KV452696.1	3891912	1-scf00001	1
AX-157891584	1	27.093	KV452696.1	3892236	1-scf00001	1
AX-157891618	1	27.093	KV452696.1	3935037	1-scf00001	1
AX-157891332	1	27.103	KV452696.1	3520121	1-scf00001	1
AX-157891357	1	27.103	KV452696.1	3562718	1-scf00001	1
AX-157748404	1	27.103	KV452696.1	3643093	1-scf00001	1
AX-157891503	1	27.103	KV452696.1	3802781	1-scf00001	1
AX-157891589	1	27.103	KV452696.1	3896450	1-scf00001	1
AX-157891912	1	27.103	KV452696.1	4385477	1-scf00001	1
AX-157810353	1	27.103	KV452696.1	4433906	1-scf00001	1
AX-157892437	1	27.103	KV452697.1	1398	1-scf00003	1
AX-157890978	1	27.123	KV452696.1	2867489	1-scf00001	1
AX-157891108	1	27.123	KV452696.1	3058524	1-scf00001	1
AX-157891187	1	27.123	KV452696.1	3194985	1-scf00001	1
AX-158381897	1	27.123	KV452696.1	3372749	1-scf00001	1
AX-158381898	1	27.123	KV452696.1	3396231	1-scf00001	1
AX-158381899	1	27.123	KV452696.1	3430947	1-scf00001	1
AX-157891310	1	27.123	KV452696.1	3481049	1-scf00001	1
AX-157891371	1	27.123	KV452696.1	3613622	1-scf00001	1
AX-157891372	1	27.123	KV452696.1	3614489	1-scf00001	1
AX-157891375	1	27.123	KV452696.1	3617445	1-scf00001	1
AX-157891378	1	27.123	KV452696.1	3619104	1-scf00001	1
AX-158381900	1	27.123	KV452696.1	3648388	1-scf00001	1
AX-157891400	1	27.123	KV452696.1	3649899	1-scf00001	1
AX-157755010	1	27.123	KV452696.1	3702319	1-scf00001	1
AX-157891495	1	27.123	KV452696.1	3788276	1-scf00001	1
AX-157891528	1	27.123	KV452696.1	3831895	1-scf00001	1
AX-158379100	1	27.123	KV452696.1	3832072	1-scf00001	1
AX-157749658	1	27.123	KV452696.1	3832146	1-scf00001	1
AX-157891529	1	27.123	KV452696.1	3832704	1-scf00001	1
AX-157891534	1	27.123	KV452696.1	3836751	1-scf00001	1
AX-157891544	1	27.123	KV452696.1	3843910	1-scf00001	1
AX-157891555	1	27.123	KV452696.1	3860207	1-scf00001	1
AX-157891579	1	27.123	KV452696.1	3882916	1-scf00001	1
AX-157891591	1	27.123	KV452696.1	3898600	1-scf00001	1
AX-157776108	1	27.182	KV452696.1	3199482	1-scf00001	1
AX-157891448	1	27.182	KV452696.1	3713691	1-scf00001	1
AX-157891459	1	27.182	KV452696.1	3723660	1-scf00001	1
AX-157891485	1	27.182	KV452696.1	3774368	1-scf00001	1
AX-157891543	1	27.182	KV452696.1	3843748	1-scf00001	1
AX-157891595	1	27.182	KV452696.1	3901222	1-scf00001	1
AX-157891599	1	27.182	KV452696.1	3904640	1-scf00001	1
AX-157891603	1	27.182	KV452696.1	3915135	1-scf00001	1
AX-157874249	1	27.182	KV452696.1	3933901	1-scf00001	1
AX-157891619	1	27.182	KV452696.1	3935383	1-scf00001	1

AX-157891620	1	27.182	KV452696.1	3936772	1-scf00001	1
AX-157891622	1	27.182	KV452696.1	3938264	1-scf00001	1
AX-157891624	1	27.182	KV452696.1	3940666	1-scf00001	1
AX-157891625	1	27.182	KV452696.1	3941025	1-scf00001	1
AX-157891629	1	27.182	KV452696.1	3944305	1-scf00001	1
AX-157891630	1	27.182	KV452696.1	3944713	1-scf00001	1
AX-158404187	1	27.182	KV452696.1	3947147	1-scf00001	1
AX-157891634	1	27.182	KV452696.1	3948057	1-scf00001	1
AX-157891639	1	27.182	KV452696.1	3957500	1-scf00001	1
AX-157891640	1	27.182	KV452696.1	3957996	1-scf00001	1
AX-157891642	1	27.182	KV452696.1	3963537	1-scf00001	1
AX-157891644	1	27.182	KV452696.1	3969811	1-scf00001	1
AX-157891645	1	27.182	KV452696.1	3970267	1-scf00001	1
AX-157891646	1	27.182	KV452696.1	3971123	1-scf00001	1
AX-157891647	1	27.182	KV452696.1	3971384	1-scf00001	1
AX-157891648	1	27.182	KV452696.1	3971728	1-scf00001	1
AX-157891654	1	27.182	KV452696.1	3978350	1-scf00001	1
AX-157891656	1	27.182	KV452696.1	3979899	1-scf00001	1
AX-157891659	1	27.182	KV452696.1	3983118	1-scf00001	1
AX-157891678	1	27.182	KV452696.1	4029096	1-scf00001	1
AX-157891679	1	27.182	KV452696.1	4029608	1-scf00001	1
AX-157891680	1	27.182	KV452696.1	4030083	1-scf00001	1
AX-157891681	1	27.182	KV452696.1	4030561	1-scf00001	1
AX-157891682	1	27.182	KV452696.1	4030931	1-scf00001	1
AX-157810314	1	27.182	KV452696.1	4031424	1-scf00001	1
AX-157891684	1	27.182	KV452696.1	4034503	1-scf00001	1
AX-157891688	1	27.182	KV452696.1	4039910	1-scf00001	1
AX-157891689	1	27.182	KV452696.1	4040747	1-scf00001	1
AX-157891690	1	27.182	KV452696.1	4042168	1-scf00001	1
AX-157810315	1	27.182	KV452696.1	4048936	1-scf00001	1
AX-157891695	1	27.182	KV452696.1	4056457	1-scf00001	1
AX-157891696	1	27.182	KV452696.1	4057940	1-scf00001	1
AX-157891697	1	27.182	KV452696.1	4060315	1-scf00001	1
AX-157810316	1	27.182	KV452696.1	4067716	1-scf00001	1
AX-157891701	1	27.182	KV452696.1	4072102	1-scf00001	1
AX-157891705	1	27.182	KV452696.1	4076810	1-scf00001	1
AX-157883049	1	27.182	KV452696.1	4094607	1-scf00001	1
AX-157891745	1	27.182	KV452696.1	4120703	1-scf00001	1
AX-157891765	1	27.182	KV452696.1	4150122	1-scf00001	1
AX-157810328	1	27.182	KV452696.1	4235059	1-scf00001	1
AX-157891810	1	27.182	KV452696.1	4250120	1-scf00001	1
AX-157891826	1	27.182	KV452696.1	4272718	1-scf00001	1
AX-157891838	1	27.182	KV452696.1	4288694	1-scf00001	1
AX-157891849	1	27.182	KV452696.1	4301023	1-scf00001	1
AX-157892087	1	27.182	KV452698.1	53777	1-scf00002	1
AX-157892102	1	27.182	KV452698.1	94823	1-scf00002	1
AX-157892104	1	27.182	KV452698.1	99350	1-scf00002	1
AX-157810419	1	27.182	KV452697.1	77759	1-scf00003	1
AX-157891631	1	27.219	KV452696.1	3944839	1-scf00001	1
AX-157891704	1	27.219	KV452696.1	4076310	1-scf00001	1
AX-157810294	1	27.291	KV452696.1	3275931	1-scf00001	1

AX-157891590	1	27.291	KV452696.1	3896941	1-scf00001	1
AX-157891592	1	27.291	KV452696.1	3899052	1-scf00001	1
AX-157891593	1	27.291	KV452696.1	3899852	1-scf00001	1
AX-157891594	1	27.291	KV452696.1	3900583	1-scf00001	1
AX-157891596	1	27.291	KV452696.1	3901433	1-scf00001	1
AX-157891608	1	27.291	KV452696.1	3922704	1-scf00001	1
AX-157891628	1	27.291	KV452696.1	3943946	1-scf00001	1
AX-157891706	1	27.291	KV452696.1	4077212	1-scf00001	1
AX-157891708	1	27.291	KV452696.1	4079121	1-scf00001	1
AX-157891709	1	27.291	KV452696.1	4080359	1-scf00001	1
AX-157891712	1	27.291	KV452696.1	4082571	1-scf00001	1
AX-157891713	1	27.291	KV452696.1	4083072	1-scf00001	1
AX-157891714	1	27.291	KV452696.1	4084007	1-scf00001	1
AX-157891715	1	27.291	KV452696.1	4084719	1-scf00001	1
AX-157891717	1	27.291	KV452696.1	4087507	1-scf00001	1
AX-157891718	1	27.291	KV452696.1	4088014	1-scf00001	1
AX-157891719	1	27.291	KV452696.1	4089646	1-scf00001	1
AX-157891720	1	27.291	KV452696.1	4090330	1-scf00001	1
AX-157891721	1	27.291	KV452696.1	4090485	1-scf00001	1
AX-157891722	1	27.291	KV452696.1	4092655	1-scf00001	1
AX-157891723	1	27.291	KV452696.1	4094066	1-scf00001	1
AX-157891725	1	27.291	KV452696.1	4097669	1-scf00001	1
AX-157810317	1	27.291	KV452696.1	4099758	1-scf00001	1
AX-157891731	1	27.291	KV452696.1	4104542	1-scf00001	1
AX-157891732	1	27.291	KV452696.1	4107896	1-scf00001	1
AX-157810318	1	27.291	KV452696.1	4107959	1-scf00001	1
AX-157891733	1	27.291	KV452696.1	4108848	1-scf00001	1
AX-157742311	1	27.291	KV452696.1	4110308	1-scf00001	1
AX-157891735	1	27.291	KV452696.1	4110455	1-scf00001	1
AX-158404192	1	27.291	KV452696.1	4110978	1-scf00001	1
AX-157891739	1	27.291	KV452696.1	4113741	1-scf00001	1
AX-157891741	1	27.291	KV452696.1	4115605	1-scf00001	1
AX-157891744	1	27.291	KV452696.1	4119993	1-scf00001	1
AX-157891747	1	27.291	KV452696.1	4125374	1-scf00001	1
AX-157891748	1	27.291	KV452696.1	4130055	1-scf00001	1
AX-157891750	1	27.291	KV452696.1	4131648	1-scf00001	1
AX-157891752	1	27.291	KV452696.1	4133562	1-scf00001	1
AX-157891755	1	27.291	KV452696.1	4138822	1-scf00001	1
AX-157810320	1	27.291	KV452696.1	4139150	1-scf00001	1
AX-158404194	1	27.291	KV452696.1	4140562	1-scf00001	1
AX-157891757	1	27.291	KV452696.1	4142587	1-scf00001	1
AX-157726307	1	27.291	KV452696.1	4144238	1-scf00001	1
AX-157891760	1	27.291	KV452696.1	4146002	1-scf00001	1
AX-157891762	1	27.291	KV452696.1	4147953	1-scf00001	1
AX-157891770	1	27.291	KV452696.1	4159157	1-scf00001	1
AX-157891771	1	27.291	KV452696.1	4159705	1-scf00001	1
AX-157864322	1	27.291	KV452696.1	4170454	1-scf00001	1
AX-157891777	1	27.291	KV452696.1	4173167	1-scf00001	1
AX-157810323	1	27.291	KV452696.1	4177610	1-scf00001	1
AX-157891783	1	27.291	KV452696.1	4178447	1-scf00001	1
AX-157891785	1	27.291	KV452696.1	4179958	1-scf00001	1

AX-157759031	1	27.291	KV452696.1	4181534	1-scf00001	1
AX-157891786	1	27.291	KV452696.1	4190408	1-scf00001	1
AX-157891787	1	27.291	KV452696.1	4192272	1-scf00001	1
AX-157810325	1	27.291	KV452696.1	4194767	1-scf00001	1
AX-157891796	1	27.291	KV452696.1	4228793	1-scf00001	1
AX-157891797	1	27.291	KV452696.1	4229305	1-scf00001	1
AX-157770732	1	27.291	KV452696.1	4230072	1-scf00001	1
AX-157891800	1	27.291	KV452696.1	4232785	1-scf00001	1
AX-157810327	1	27.291	KV452696.1	4234082	1-scf00001	1
AX-157891811	1	27.291	KV452696.1	4250781	1-scf00001	1
AX-157891814	1	27.291	KV452696.1	4257312	1-scf00001	1
AX-157891822	1	27.291	KV452696.1	4265692	1-scf00001	1
AX-157810336	1	27.291	KV452696.1	4278719	1-scf00001	1
AX-157891869	1	27.291	KV452696.1	4325235	1-scf00001	1
AX-157715671	1	27.291	KV452696.1	4329926	1-scf00001	1
AX-157891875	1	27.291	KV452696.1	4335923	1-scf00001	1
AX-157891804	1	27.307	KV452696.1	4242308	1-scf00001	1
AX-157891812	1	27.319	KV452696.1	4252634	1-scf00001	1
AX-157810330	1	27.345	KV452696.1	4255093	1-scf00001	1
AX-157891813	1	27.373	KV452696.1	4255997	1-scf00001	1
AX-157891401	1	27.4	KV452696.1	3651956	1-scf00001	1
AX-157891404	1	27.4	KV452696.1	3659647	1-scf00001	1
AX-157891598	1	27.4	KV452696.1	3903163	1-scf00001	1
AX-157891609	1	27.4	KV452696.1	3922958	1-scf00001	1
AX-157891610	1	27.4	KV452696.1	3924320	1-scf00001	1
AX-157891621	1	27.4	KV452696.1	3937760	1-scf00001	1
AX-157810319	1	27.4	KV452696.1	4118982	1-scf00001	1
AX-157810326	1	27.4	KV452696.1	4226616	1-scf00001	1
AX-157810329	1	27.4	KV452696.1	4241270	1-scf00001	1
AX-157891824	1	27.4	KV452696.1	4266455	1-scf00001	1
AX-157891825	1	27.4	KV452696.1	4266906	1-scf00001	1
AX-157810331	1	27.4	KV452696.1	4269858	1-scf00001	1
AX-157891827	1	27.4	KV452696.1	4272946	1-scf00001	1
AX-157810335	1	27.4	KV452696.1	4275353	1-scf00001	1
AX-157891828	1	27.4	KV452696.1	4275866	1-scf00001	1
AX-157891829	1	27.4	KV452696.1	4276443	1-scf00001	1
AX-157891835	1	27.4	KV452696.1	4284483	1-scf00001	1
AX-157741804	1	27.4	KV452696.1	4292780	1-scf00001	1
AX-157891848	1	27.4	KV452696.1	4300502	1-scf00001	1
AX-157745669	1	27.4	KV452696.1	4327789	1-scf00001	1
AX-157891830	1	27.401	KV452696.1	4280250	1-scf00001	1
AX-157891753	1	27.403	KV452696.1	4137843	1-scf00001	1
AX-157891809	1	27.403	KV452696.1	4247844	1-scf00001	1
AX-157726962	1	27.403	KV452696.1	4301367	1-scf00001	1
AX-157713554	1	27.403	KV452696.1	4332537	1-scf00001	1
AX-157853537	1	27.545	KV452696.1	4319239	1-scf00001	1
AX-157891867	1	27.545	KV452696.1	4323854	1-scf00001	1
AX-157874258	1	27.545	KV452696.1	4330650	1-scf00001	1
AX-157891876	1	27.545	KV452696.1	4336682	1-scf00001	1
AX-157891879	1	27.837	KV452696.1	4338041	1-scf00001	1
AX-157771574	1	27.837	KV452696.1	4348918	1-scf00001	1

AX-157891885	1	27.837	KV452696.1	4352048	1-scf00001	1
AX-157891887	1	27.837	KV452696.1	4356315	1-scf00001	1
AX-157891888	1	27.837	KV452696.1	4356662	1-scf00001	1
AX-157810341	1	27.837	KV452696.1	4363002	1-scf00001	1
AX-157891915	1	27.837	KV452696.1	4390448	1-scf00001	1
AX-157891929	1	27.837	KV452696.1	4416608	1-scf00001	1
AX-157891889	1	27.855	KV452696.1	4357506	1-scf00001	1
AX-157891894	1	27.876	KV452696.1	4361051	1-scf00001	1
AX-157891901	1	27.894	KV452696.1	4370771	1-scf00001	1
AX-157891836	1	27.905	KV452696.1	4284930	1-scf00001	1
AX-157891850	1	27.905	KV452696.1	4303813	1-scf00001	1
AX-157891865	1	27.905	KV452696.1	4321830	1-scf00001	1
AX-157891902	1	27.905	KV452696.1	4371576	1-scf00001	1
AX-157810349	1	27.905	KV452696.1	4419235	1-scf00001	1
AX-157891904	1	27.921	KV452696.1	4374592	1-scf00001	1
AX-157810343	1	27.934	KV452696.1	4378480	1-scf00001	1
AX-157891905	1	27.946	KV452696.1	4375988	1-scf00001	1
AX-157891907	1	27.946	KV452696.1	4379254	1-scf00001	1
AX-157891909	1	27.946	KV452696.1	4383440	1-scf00001	1
AX-157810346	1	27.946	KV452696.1	4387540	1-scf00001	1
AX-157891914	1	27.946	KV452696.1	4389095	1-scf00001	1
AX-157810347	1	27.946	KV452696.1	4395226	1-scf00001	1
AX-158403716	1	27.946	KV452696.1	4399762	1-scf00001	1
AX-157891924	1	27.946	KV452696.1	4405258	1-scf00001	1
AX-157891925	1	27.946	KV452696.1	4406956	1-scf00001	1
AX-157891926	1	27.946	KV452696.1	4408662	1-scf00001	1
AX-157892033	1	27.946	KV452696.1	4584075	1-scf00001	1
AX-158379101	1	27.995	KV452696.1	4269142	1-scf00001	1
AX-157810338	1	27.995	KV452696.1	4322222	1-scf00001	1
AX-157810344	1	27.995	KV452696.1	4381675	1-scf00001	1
AX-157891910	1	27.995	KV452696.1	4383733	1-scf00001	1
AX-157742685	1	27.995	KV452696.1	4426930	1-scf00001	1
AX-157891934	1	28.056	KV452696.1	4427249	1-scf00001	1
AX-157891937	1	28.056	KV452696.1	4434182	1-scf00001	1
AX-157892172	1	28.056	KV452698.1	324799	1-scf00002	1
AX-158403738	1	28.056	KV452697.1	116190	1-scf00003	1
AX-158421050	1	28.056	3ML01025952	299	scf01561_1	UN
AX-157707024	1	28.056	3ML01034532	283	scf10209_1	UN
AX-157707025	1	28.056	3ML01034532	409	scf10209_1	UN
AX-157891938	1	28.201	KV452696.1	4437508	1-scf00001	1
AX-157891940	1	28.201	KV452696.1	4441896	1-scf00001	1
AX-157891945	1	28.201	KV452696.1	4445590	1-scf00001	1
AX-157891948	1	28.201	KV452696.1	4447498	1-scf00001	1
AX-157891953	1	28.201	KV452696.1	4451973	1-scf00001	1
AX-157891955	1	28.201	KV452696.1	4455604	1-scf00001	1
AX-157892132	1	28.201	KV452698.1	175530	1-scf00002	1
AX-157891964	1	28.347	KV452696.1	4473230	1-scf00001	1
AX-157891054	1	28.493	KV452696.1	2961798	1-scf00001	1
AX-157891060	1	28.493	KV452696.1	2974417	1-scf00001	1
AX-157891070	1	28.493	KV452696.1	2995590	1-scf00001	1
AX-157891071	1	28.493	KV452696.1	3000098	1-scf00001	1

AX-157891072	1	28.493	KV452696.1	3001858	1-scf00001	1
AX-157891075	1	28.493	KV452696.1	3003683	1-scf00001	1
AX-157891076	1	28.493	KV452696.1	3004833	1-scf00001	1
AX-157891175	1	28.493	KV452696.1	3182006	1-scf00001	1
AX-157891193	1	28.493	KV452696.1	3201375	1-scf00001	1
AX-157810291	1	28.493	KV452696.1	3254555	1-scf00001	1
AX-157891207	1	28.493	KV452696.1	3254703	1-scf00001	1
AX-157891215	1	28.493	KV452696.1	3278872	1-scf00001	1
AX-157891226	1	28.493	KV452696.1	3300161	1-scf00001	1
AX-157891290	1	28.493	KV452696.1	3383833	1-scf00001	1
AX-157891292	1	28.493	KV452696.1	3386775	1-scf00001	1
AX-157891301	1	28.493	KV452696.1	3441835	1-scf00001	1
AX-157891415	1	28.493	KV452696.1	3670716	1-scf00001	1
AX-157891416	1	28.493	KV452696.1	3675059	1-scf00001	1
AX-157891419	1	28.493	KV452696.1	3683721	1-scf00001	1
AX-157891467	1	28.493	KV452696.1	3749707	1-scf00001	1
AX-157891784	1	28.493	KV452696.1	4179300	1-scf00001	1
AX-157891819	1	28.493	KV452696.1	4262476	1-scf00001	1
AX-157745561	1	28.493	KV452696.1	4314452	1-scf00001	1
AX-157891878	1	28.493	KV452696.1	4337576	1-scf00001	1
AX-157891886	1	28.493	KV452696.1	4354221	1-scf00001	1
AX-157891890	1	28.493	KV452696.1	4358935	1-scf00001	1
AX-157891917	1	28.493	KV452696.1	4392668	1-scf00001	1
AX-157891931	1	28.493	KV452696.1	4417631	1-scf00001	1
AX-157891941	1	28.493	KV452696.1	4442875	1-scf00001	1
AX-157810356	1	28.493	KV452696.1	4453401	1-scf00001	1
AX-157891954	1	28.493	KV452696.1	4453508	1-scf00001	1
AX-157891966	1	28.493	KV452696.1	4475134	1-scf00001	1
AX-157891972	1	28.493	KV452696.1	4479294	1-scf00001	1
AX-157891974	1	28.493	KV452696.1	4481138	1-scf00001	1
AX-157723563	1	28.493	KV452696.1	4492155	1-scf00001	1
AX-157891984	1	28.493	KV452696.1	4494687	1-scf00001	1
AX-157892000	1	28.493	KV452696.1	4528291	1-scf00001	1
AX-157709977	1	28.493	KV452696.1	4530694	1-scf00001	1
AX-157892002	1	28.493	KV452696.1	4534354	1-scf00001	1
AX-157892004	1	28.493	KV452696.1	4536917	1-scf00001	1
AX-157892006	1	28.493	KV452696.1	4538570	1-scf00001	1
AX-157892019	1	28.493	KV452696.1	4565661	1-scf00001	1
AX-158381906	1	28.493	KV452698.1	13370	1-scf00002	1
AX-157892197	1	28.493	KV452698.1	409562	1-scf00002	1
AX-157892208	1	28.493	KV452698.1	438961	1-scf00002	1
AX-157892209	1	28.493	KV452698.1	440196	1-scf00002	1
AX-157892214	1	28.493	KV452698.1	450366	1-scf00002	1
AX-157892215	1	28.493	KV452698.1	453953	1-scf00002	1
AX-157892219	1	28.493	KV452698.1	467276	1-scf00002	1
AX-157892221	1	28.493	KV452698.1	468357	1-scf00002	1
AX-158414513	1	28.517	KV452696.1	4539391	1-scf00001	1
AX-157810292	1	28.53	KV452696.1	3267461	1-scf00001	1
AX-157891213	1	28.53	KV452696.1	3269634	1-scf00001	1
AX-157891742	1	28.53	KV452696.1	4117765	1-scf00001	1
AX-157742429	1	28.53	KV452696.1	4222678	1-scf00001	1

AX-157891918	1	28.53	KV452696.1	4393507	1-scf00001	1
AX-157891969	1	28.53	KV452696.1	4476348	1-scf00001	1
AX-157891975	1	28.53	KV452696.1	4483170	1-scf00001	1
AX-157892009	1	28.53	KV452696.1	4542147	1-scf00001	1
AX-157892013	1	28.53	KV452696.1	4549500	1-scf00001	1
AX-157810370	1	28.53	KV452698.1	16555	1-scf00002	1
AX-157892085	1	28.53	KV452698.1	52531	1-scf00002	1
AX-157892090	1	28.53	KV452698.1	58935	1-scf00002	1
AX-157892096	1	28.53	KV452698.1	73993	1-scf00002	1
AX-157892103	1	28.53	KV452698.1	98721	1-scf00002	1
AX-157892108	1	28.53	KV452698.1	102549	1-scf00002	1
AX-157892110	1	28.53	KV452698.1	103777	1-scf00002	1
AX-157892114	1	28.53	KV452698.1	113060	1-scf00002	1
AX-157892119	1	28.53	KV452698.1	119369	1-scf00002	1
AX-157892123	1	28.53	KV452698.1	129619	1-scf00002	1
AX-157892127	1	28.53	KV452698.1	148555	1-scf00002	1
AX-157892130	1	28.53	KV452698.1	158089	1-scf00002	1
AX-157892133	1	28.53	KV452698.1	175893	1-scf00002	1
AX-157892135	1	28.53	KV452698.1	176869	1-scf00002	1
AX-157892136	1	28.53	KV452698.1	177114	1-scf00002	1
AX-157892141	1	28.53	KV452698.1	195843	1-scf00002	1
AX-157892142	1	28.53	KV452698.1	214451	1-scf00002	1
AX-157892146	1	28.53	KV452698.1	226634	1-scf00002	1
AX-157892150	1	28.53	KV452698.1	239834	1-scf00002	1
AX-157892153	1	28.53	KV452698.1	250989	1-scf00002	1
AX-157892156	1	28.53	KV452698.1	254087	1-scf00002	1
AX-157892157	1	28.53	KV452698.1	257001	1-scf00002	1
AX-157892158	1	28.53	KV452698.1	258298	1-scf00002	1
AX-157892159	1	28.53	KV452698.1	259615	1-scf00002	1
AX-157892160	1	28.53	KV452698.1	261381	1-scf00002	1
AX-157892165	1	28.53	KV452698.1	287691	1-scf00002	1
AX-157892168	1	28.53	KV452698.1	295938	1-scf00002	1
AX-158404207	1	28.53	KV452698.1	299445	1-scf00002	1
AX-158403722	1	28.53	KV452698.1	299779	1-scf00002	1
AX-158404208	1	28.53	KV452698.1	300014	1-scf00002	1
AX-158421762	1	28.53	KV452698.1	305253	1-scf00002	1
AX-158404211	1	28.53	KV452698.1	346486	1-scf00002	1
AX-157892181	1	28.53	KV452698.1	359513	1-scf00002	1
AX-157892185	1	28.53	KV452698.1	362992	1-scf00002	1
AX-157892190	1	28.53	KV452698.1	381385	1-scf00002	1
AX-157892194	1	28.53	KV452698.1	403460	1-scf00002	1
AX-157892195	1	28.53	KV452698.1	407045	1-scf00002	1
AX-157892199	1	28.53	KV452698.1	416793	1-scf00002	1
AX-157892201	1	28.53	KV452698.1	425616	1-scf00002	1
AX-157892262	1	28.53	KV452698.1	545778	1-scf00002	1
AX-157892263	1	28.53	KV452698.1	546388	1-scf00002	1
AX-157862577	1	28.53	KV452698.1	554991	1-scf00002	1
AX-157883053	1	28.53	KV452698.1	630712	1-scf00002	1
AX-157810396	1	28.53	KV452698.1	677023	1-scf00002	1
AX-157892442	1	28.53	KV452697.1	6633	1-scf00003	1
AX-157892454	1	28.53	KV452697.1	46994	1-scf00003	1

AX-157892517	1	28.53	KV452697.1	218841	1-scf00003	1
AX-157772233	1	28.545	KV452696.1	4412227	1-scf00001	1
AX-157892050	1	28.545	KV452696.1	4637172	1-scf00001	1
AX-157892438	1	28.575	KV452697.1	2995	1-scf00003	1
AX-157890989	1	28.602	KV452696.1	2882555	1-scf00001	1
AX-157891016	1	28.602	KV452696.1	2919304	1-scf00001	1
AX-157891173	1	28.602	KV452696.1	3180935	1-scf00001	1
AX-157891218	1	28.602	KV452696.1	3289555	1-scf00001	1
AX-157891484	1	28.602	KV452696.1	3773721	1-scf00001	1
AX-157891496	1	28.602	KV452696.1	3790520	1-scf00001	1
AX-157891651	1	28.602	KV452696.1	3973545	1-scf00001	1
AX-157891687	1	28.602	KV452696.1	4037785	1-scf00001	1
AX-157891692	1	28.602	KV452696.1	4050035	1-scf00001	1
AX-157891699	1	28.602	KV452696.1	4067297	1-scf00001	1
AX-157891780	1	28.602	KV452696.1	4174481	1-scf00001	1
AX-157891851	1	28.602	KV452696.1	4305051	1-scf00001	1
AX-157810339	1	28.602	KV452696.1	4335078	1-scf00001	1
AX-157891884	1	28.602	KV452696.1	4350529	1-scf00001	1
AX-157891932	1	28.602	KV452696.1	4421833	1-scf00001	1
AX-157891947	1	28.602	KV452696.1	4446104	1-scf00001	1
AX-157891949	1	28.602	KV452696.1	4448566	1-scf00001	1
AX-157891958	1	28.602	KV452696.1	4459544	1-scf00001	1
AX-157891962	1	28.602	KV452696.1	4470602	1-scf00001	1
AX-157891977	1	28.602	KV452696.1	4484238	1-scf00001	1
AX-157891982	1	28.602	KV452696.1	4489966	1-scf00001	1
AX-157810361	1	28.602	KV452696.1	4493939	1-scf00001	1
AX-157891988	1	28.602	KV452696.1	4499548	1-scf00001	1
AX-157727345	1	28.602	KV452696.1	4501740	1-scf00001	1
AX-157891995	1	28.602	KV452696.1	4517959	1-scf00001	1
AX-157892008	1	28.602	KV452696.1	4541288	1-scf00001	1
AX-157892037	1	28.602	KV452696.1	4590783	1-scf00001	1
AX-157892066	1	28.602	KV452698.1	11530	1-scf00002	1
AX-157892067	1	28.602	KV452698.1	13793	1-scf00002	1
AX-157892069	1	28.602	KV452698.1	15862	1-scf00002	1
AX-157892072	1	28.602	KV452698.1	31204	1-scf00002	1
AX-157892073	1	28.602	KV452698.1	32356	1-scf00002	1
AX-157892075	1	28.602	KV452698.1	34754	1-scf00002	1
AX-158381907	1	28.602	KV452698.1	36277	1-scf00002	1
AX-157892076	1	28.602	KV452698.1	38238	1-scf00002	1
AX-157892077	1	28.602	KV452698.1	39607	1-scf00002	1
AX-157892078	1	28.602	KV452698.1	40280	1-scf00002	1
AX-157892080	1	28.602	KV452698.1	47667	1-scf00002	1
AX-157892081	1	28.602	KV452698.1	48158	1-scf00002	1
AX-157892086	1	28.602	KV452698.1	53322	1-scf00002	1
AX-157892089	1	28.602	KV452698.1	58397	1-scf00002	1
AX-157892091	1	28.602	KV452698.1	59969	1-scf00002	1
AX-157892092	1	28.602	KV452698.1	63344	1-scf00002	1
AX-157892093	1	28.602	KV452698.1	70250	1-scf00002	1
AX-157892101	1	28.602	KV452698.1	92246	1-scf00002	1
AX-157892105	1	28.602	KV452698.1	101471	1-scf00002	1
AX-157892106	1	28.602	KV452698.1	101832	1-scf00002	1

AX-157892109	1	28.602	KV452698.1	103235	1-scf00002	1
AX-157722150	1	28.602	KV452698.1	105820	1-scf00002	1
AX-157892118	1	28.602	KV452698.1	118924	1-scf00002	1
AX-157892124	1	28.602	KV452698.1	133836	1-scf00002	1
AX-157892125	1	28.602	KV452698.1	139105	1-scf00002	1
AX-157892126	1	28.602	KV452698.1	147313	1-scf00002	1
AX-157892129	1	28.602	KV452698.1	153753	1-scf00002	1
AX-157892131	1	28.602	KV452698.1	174344	1-scf00002	1
AX-157892138	1	28.602	KV452698.1	181825	1-scf00002	1
AX-157868467	1	28.602	KV452698.1	184164	1-scf00002	1
AX-157892143	1	28.602	KV452698.1	216990	1-scf00002	1
AX-157892144	1	28.602	KV452698.1	219513	1-scf00002	1
AX-157892145	1	28.602	KV452698.1	223958	1-scf00002	1
AX-157892148	1	28.602	KV452698.1	236704	1-scf00002	1
AX-157892154	1	28.602	KV452698.1	252245	1-scf00002	1
AX-157892163	1	28.602	KV452698.1	279192	1-scf00002	1
AX-157892171	1	28.602	KV452698.1	317811	1-scf00002	1
AX-157892175	1	28.602	KV452698.1	333352	1-scf00002	1
AX-157892189	1	28.602	KV452698.1	376729	1-scf00002	1
AX-157892191	1	28.602	KV452698.1	382881	1-scf00002	1
AX-157892192	1	28.602	KV452698.1	383519	1-scf00002	1
AX-157892193	1	28.602	KV452698.1	389504	1-scf00002	1
AX-157722409	1	28.602	KV452698.1	418756	1-scf00002	1
AX-157755821	1	28.602	KV452698.1	433288	1-scf00002	1
AX-157892206	1	28.602	KV452698.1	435661	1-scf00002	1
AX-157892207	1	28.602	KV452698.1	438308	1-scf00002	1
AX-158388969	1	28.602	KV452698.1	439841	1-scf00002	1
AX-157892213	1	28.602	KV452698.1	449208	1-scf00002	1
AX-157892217	1	28.602	KV452698.1	458802	1-scf00002	1
AX-157892220	1	28.602	KV452698.1	467594	1-scf00002	1
AX-157892222	1	28.602	KV452698.1	468938	1-scf00002	1
AX-157810371	1	28.602	KV452698.1	473272	1-scf00002	1
AX-157892225	1	28.602	KV452698.1	473744	1-scf00002	1
AX-157892226	1	28.602	KV452698.1	474474	1-scf00002	1
AX-157810372	1	28.602	KV452698.1	474723	1-scf00002	1
AX-157892227	1	28.602	KV452698.1	475117	1-scf00002	1
AX-157892228	1	28.602	KV452698.1	475495	1-scf00002	1
AX-157892229	1	28.602	KV452698.1	476068	1-scf00002	1
AX-157892232	1	28.602	KV452698.1	477719	1-scf00002	1
AX-157892238	1	28.602	KV452698.1	488163	1-scf00002	1
AX-157892240	1	28.602	KV452698.1	489530	1-scf00002	1
AX-157892243	1	28.602	KV452698.1	497099	1-scf00002	1
AX-157892246	1	28.602	KV452698.1	507176	1-scf00002	1
AX-157892247	1	28.602	KV452698.1	508442	1-scf00002	1
AX-157810373	1	28.602	KV452698.1	537117	1-scf00002	1
AX-157810384	1	28.602	KV452698.1	609912	1-scf00002	1
AX-157892301	1	28.602	KV452698.1	618421	1-scf00002	1
AX-157810386	1	28.602	KV452698.1	622445	1-scf00002	1
AX-157748491	1	28.602	KV452698.1	622837	1-scf00002	1
AX-157810394	1	28.602	KV452698.1	672042	1-scf00002	1
AX-157892329	1	28.602	KV452698.1	684981	1-scf00002	1

AX-158388971	1	28.602	KV452698.1	687621	1-scf00002	1
AX-157774245	1	28.602	KV452698.1	723855	1-scf00002	1
AX-157892365	1	28.602	KV452698.1	745998	1-scf00002	1
AX-157892443	1	28.602	KV452697.1	7710	1-scf00003	1
AX-157726116	1	28.602	KV452697.1	18829	1-scf00003	1
AX-157892447	1	28.602	KV452697.1	22289	1-scf00003	1
AX-157892448	1	28.602	KV452697.1	24692	1-scf00003	1
AX-157810415	1	28.602	KV452697.1	49386	1-scf00003	1
AX-157810417	1	28.602	KV452697.1	55027	1-scf00003	1
AX-157892457	1	28.602	KV452697.1	58645	1-scf00003	1
AX-157864330	1	28.602	KV452697.1	59151	1-scf00003	1
AX-157892473	1	28.602	KV452697.1	96160	1-scf00003	1
AX-157892474	1	28.602	KV452697.1	97810	1-scf00003	1
AX-157892476	1	28.602	KV452697.1	101414	1-scf00003	1
AX-157892483	1	28.602	KV452697.1	115750	1-scf00003	1
AX-157892484	1	28.602	KV452697.1	120531	1-scf00003	1
AX-157751146	1	28.602	KV452697.1	126328	1-scf00003	1
AX-157810423	1	28.602	KV452697.1	161456	1-scf00003	1
AX-157810424	1	28.602	KV452697.1	171069	1-scf00003	1
AX-157892503	1	28.602	KV452697.1	172503	1-scf00003	1
AX-157892504	1	28.602	KV452697.1	174757	1-scf00003	1
AX-157892506	1	28.602	KV452697.1	179093	1-scf00003	1
AX-157892507	1	28.602	KV452697.1	179603	1-scf00003	1
AX-157892508	1	28.602	KV452697.1	179848	1-scf00003	1
AX-157892509	1	28.602	KV452697.1	180651	1-scf00003	1
AX-157892510	1	28.602	KV452697.1	182779	1-scf00003	1
AX-157892511	1	28.602	KV452697.1	187376	1-scf00003	1
AX-157892260	1	28.656	KV452698.1	535116	1-scf00002	1
AX-157892472	1	28.656	KV452697.1	95704	1-scf00003	1
AX-157892198	1	28.711	KV452698.1	412627	1-scf00002	1
AX-157810374	1	28.711	KV452698.1	537635	1-scf00002	1
AX-157892264	1	28.711	KV452698.1	547016	1-scf00002	1
AX-157892269	1	28.711	KV452698.1	554406	1-scf00002	1
AX-157892270	1	28.711	KV452698.1	554650	1-scf00002	1
AX-157892349	1	28.711	KV452698.1	710140	1-scf00002	1
AX-157892452	1	28.711	KV452697.1	33043	1-scf00003	1
AX-157892272	1	28.781	KV452698.1	559002	1-scf00002	1
AX-157892280	1	28.781	KV452698.1	577916	1-scf00002	1
AX-157892284	1	28.781	KV452698.1	586071	1-scf00002	1
AX-157891216	1	28.82	KV452696.1	3284499	1-scf00001	1
AX-157892095	1	28.82	KV452698.1	73271	1-scf00002	1
AX-157892278	1	28.82	KV452698.1	575996	1-scf00002	1
AX-157742735	1	28.82	KV452698.1	582228	1-scf00002	1
AX-157892283	1	28.82	KV452698.1	583534	1-scf00002	1
AX-157746071	1	28.82	KV452698.1	584713	1-scf00002	1
AX-157810382	1	28.82	KV452698.1	602482	1-scf00002	1
AX-157892293	1	28.82	KV452698.1	603969	1-scf00002	1
AX-157892294	1	28.82	KV452698.1	604403	1-scf00002	1
AX-157892295	1	28.82	KV452698.1	604731	1-scf00002	1
AX-157892296	1	28.82	KV452698.1	608667	1-scf00002	1
AX-157810387	1	28.82	KV452698.1	625665	1-scf00002	1

AX-157705858	1	28.82	3ML01031072	181	scf06677_1	UN
AX-157810383	1	28.854	KV452698.1	609190	1-scf00002	1
AX-157810358	1	28.883	KV452696.1	4468726	1-scf00001	1
AX-157892297	1	28.883	KV452698.1	610904	1-scf00002	1
AX-157892304	1	28.917	KV452698.1	628173	1-scf00002	1
AX-157892094	1	28.93	KV452698.1	71710	1-scf00002	1
AX-157810391	1	28.93	KV452698.1	641242	1-scf00002	1
AX-157892310	1	28.93	KV452698.1	642279	1-scf00002	1
AX-157810392	1	28.93	KV452698.1	642667	1-scf00002	1
AX-157892312	1	28.93	KV452698.1	643380	1-scf00002	1
AX-157810393	1	28.93	KV452698.1	643698	1-scf00002	1
AX-157717844	1	28.93	KV452698.1	646838	1-scf00002	1
AX-157892348	1	28.93	KV452698.1	707046	1-scf00002	1
AX-157892463	1	28.93	KV452697.1	76134	1-scf00003	1
AX-157891898	1	29.039	KV452696.1	4365717	1-scf00001	1
AX-157891943	1	29.039	KV452696.1	4443480	1-scf00001	1
AX-157892084	1	29.039	KV452698.1	49418	1-scf00002	1
AX-157892097	1	29.039	KV452698.1	74454	1-scf00002	1
AX-157892099	1	29.039	KV452698.1	88701	1-scf00002	1
AX-157892113	1	29.039	KV452698.1	112112	1-scf00002	1
AX-157892128	1	29.039	KV452698.1	153291	1-scf00002	1
AX-157892308	1	29.039	KV452698.1	636148	1-scf00002	1
AX-157892309	1	29.039	KV452698.1	637383	1-scf00002	1
AX-157892320	1	29.039	KV452698.1	656628	1-scf00002	1
AX-157892321	1	29.039	KV452698.1	660252	1-scf00002	1
AX-157751147	1	29.039	KV452698.1	662294	1-scf00002	1
AX-157810395	1	29.039	KV452698.1	673194	1-scf00002	1
AX-157892325	1	29.039	KV452698.1	673704	1-scf00002	1
AX-157892330	1	29.039	KV452698.1	685485	1-scf00002	1
AX-157892331	1	29.039	KV452698.1	686019	1-scf00002	1
AX-157892335	1	29.039	KV452698.1	690793	1-scf00002	1
AX-157892336	1	29.039	KV452698.1	692439	1-scf00002	1
AX-157892342	1	29.039	KV452698.1	698809	1-scf00002	1
AX-157718244	1	29.039	KV452698.1	700023	1-scf00002	1
AX-157892344	1	29.039	KV452698.1	700376	1-scf00002	1
AX-157892345	1	29.039	KV452698.1	700927	1-scf00002	1
AX-157810399	1	29.039	KV452698.1	713077	1-scf00002	1
AX-157883054	1	29.039	KV452698.1	713184	1-scf00002	1
AX-157892351	1	29.039	KV452698.1	713364	1-scf00002	1
AX-157892352	1	29.039	KV452698.1	716484	1-scf00002	1
AX-157892354	1	29.039	KV452698.1	718823	1-scf00002	1
AX-157892358	1	29.039	KV452698.1	722397	1-scf00002	1
AX-157709496	1	29.039	KV452698.1	723165	1-scf00002	1
AX-157771935	1	29.039	KV452698.1	725981	1-scf00002	1
AX-157892362	1	29.039	KV452698.1	726305	1-scf00002	1
AX-157744510	1	29.039	KV452698.1	727459	1-scf00002	1
AX-157810401	1	29.039	KV452698.1	734139	1-scf00002	1
AX-157892370	1	29.039	KV452698.1	762996	1-scf00002	1
AX-157892374	1	29.039	KV452698.1	766955	1-scf00002	1
AX-157773865	1	29.148	KV452696.1	3688563	1-scf00001	1
AX-157891444	1	29.148	KV452696.1	3708988	1-scf00001	1

AX-157891450	1	29.148	KV452696.1	3714364	1-scf00001	1
AX-157891452	1	29.148	KV452696.1	3718487	1-scf00001	1
AX-157891453	1	29.148	KV452696.1	3719310	1-scf00001	1
AX-157891460	1	29.148	KV452696.1	3724680	1-scf00001	1
AX-157891980	1	29.148	KV452696.1	4486958	1-scf00001	1
AX-157775123	1	29.148	KV452696.1	4518875	1-scf00001	1
AX-157892166	1	29.148	KV452698.1	288241	1-scf00002	1
AX-157892169	1	29.148	KV452698.1	298431	1-scf00002	1
AX-157892224	1	29.148	KV452698.1	471997	1-scf00002	1
AX-157892363	1	29.148	KV452698.1	738369	1-scf00002	1
AX-157714957	1	29.148	KV452698.1	752644	1-scf00002	1
AX-157892368	1	29.148	KV452698.1	758608	1-scf00002	1
AX-157892496	1	29.148	KV452697.1	157764	1-scf00003	1
AX-157892499	1	29.148	KV452697.1	163679	1-scf00003	1
AX-157892367	1	29.257	KV452698.1	754924	1-scf00002	1
AX-157715840	1	29.257	KV452698.1	756043	1-scf00002	1
AX-157892369	1	29.257	KV452698.1	759437	1-scf00002	1
AX-157892371	1	29.257	KV452698.1	765342	1-scf00002	1
AX-157810404	1	29.257	KV452698.1	765613	1-scf00002	1
AX-157892372	1	29.257	KV452698.1	765994	1-scf00002	1
AX-157892375	1	29.257	KV452698.1	767830	1-scf00002	1
AX-157810388	1	29.585	KV452698.1	631082	1-scf00002	1
AX-157892337	1	29.585	KV452698.1	693213	1-scf00002	1
AX-157892376	1	29.585	KV452698.1	771286	1-scf00002	1
AX-157892379	1	29.585	KV452698.1	779034	1-scf00002	1
AX-157892380	1	29.585	KV452698.1	780554	1-scf00002	1
AX-157738881	1	29.585	KV452698.1	787699	1-scf00002	1
AX-157892386	1	29.585	KV452698.1	790787	1-scf00002	1
AX-157716109	1	29.585	KV452698.1	795290	1-scf00002	1
AX-157892388	1	29.585	KV452698.1	795380	1-scf00002	1
AX-157892390	1	29.585	KV452698.1	801825	1-scf00002	1
AX-157723580	1	29.585	KV452698.1	803623	1-scf00002	1
AX-157892488	1	29.585	KV452697.1	125607	1-scf00003	1
AX-157892147	1	29.721	KV452698.1	231369	1-scf00002	1
AX-157892389	1	29.721	KV452698.1	795618	1-scf00002	1
AX-157892391	1	29.721	KV452698.1	805757	1-scf00002	1
AX-157892392	1	29.721	KV452698.1	809997	1-scf00002	1
AX-157892393	1	29.721	KV452698.1	810948	1-scf00002	1
AX-157892396	1	29.721	KV452698.1	815548	1-scf00002	1
AX-157892398	1	29.721	KV452698.1	818177	1-scf00002	1
AX-157892399	1	29.721	KV452698.1	818543	1-scf00002	1
AX-157892401	1	29.721	KV452698.1	821813	1-scf00002	1
AX-157892405	1	29.721	KV452698.1	826989	1-scf00002	1
AX-157892492	1	29.721	KV452697.1	147559	1-scf00003	1
AX-157892394	1	29.828	KV452698.1	811498	1-scf00002	1
AX-157892404	1	29.828	KV452698.1	825017	1-scf00002	1
AX-157892406	1	29.828	KV452698.1	827735	1-scf00002	1
AX-157892418	1	29.828	KV452698.1	848329	1-scf00002	1
AX-157892411	1	30.131	KV452698.1	841847	1-scf00002	1
AX-157892412	1	30.131	KV452698.1	842361	1-scf00002	1
AX-157892413	1	30.131	KV452698.1	844985	1-scf00002	1

AX-157892414	1	30.131	KV452698.1	845720	1-scf00002	1
AX-157892416	1	30.131	KV452698.1	846671	1-scf00002	1
AX-157741149	1	30.131	KV452698.1	848685	1-scf00002	1
AX-157744288	1	30.131	KV452698.1	850102	1-scf00002	1
AX-157742090	1	30.131	KV452698.1	851820	1-scf00002	1
AX-157810412	1	30.131	KV452698.1	872822	1-scf00002	1
AX-157868470	1	30.349	KV452698.1	851981	1-scf00002	1
AX-157892420	1	30.349	KV452698.1	853830	1-scf00002	1
AX-157892421	1	30.349	KV452698.1	854068	1-scf00002	1
AX-157810407	1	30.349	KV452698.1	854278	1-scf00002	1
AX-157746502	1	30.349	KV452698.1	855633	1-scf00002	1
AX-157746246	1	30.349	KV452698.1	857162	1-scf00002	1
AX-157892423	1	30.349	KV452698.1	858241	1-scf00002	1
AX-157892426	1	30.349	KV452698.1	862539	1-scf00002	1
AX-157892429	1	30.349	KV452698.1	868179	1-scf00002	1
AX-157715019	1	30.349	KV452698.1	872617	1-scf00002	1
AX-157892424	1	30.357	KV452698.1	859191	1-scf00002	1
AX-157892427	1	30.414	KV452698.1	866654	1-scf00002	1
AX-157810411	1	30.472	KV452698.1	866763	1-scf00002	1
AX-157892430	1	30.595	KV452698.1	868607	1-scf00002	1
AX-157743672	1	30.595	KV452698.1	869609	1-scf00002	1
AX-157714513	1	30.595	KV452698.1	871375	1-scf00002	1
AX-157772055	1	30.839	KV452698.1	879294	1-scf00002	1
AX-157726133	1	30.839	KV452698.1	880093	1-scf00002	1
AX-157874265	1	30.968	KV452698.1	883112	1-scf00002	1
AX-157724742	1	31.333	KV452698.1	885383	1-scf00002	1
AX-157715993	1	31.334	KV452698.1	892234	1-scf00002	1
AX-157741156	1	31.334	KV452698.1	892711	1-scf00002	1
AX-157810426	1	31.334	KV452698.1	893388	1-scf00002	1
AX-157892520	1	31.399	KV452698.1	898583	1-scf00002	1
AX-157892519	1	31.443	KV452698.1	895322	1-scf00002	1
AX-157892521	1	31.443	KV452698.1	898953	1-scf00002	1
AX-157892523	1	31.443	KV452698.1	901083	1-scf00002	1
AX-157892524	1	31.443	KV452698.1	901701	1-scf00002	1
AX-157892525	1	31.443	KV452698.1	902667	1-scf00002	1
AX-157892526	1	31.443	KV452698.1	903676	1-scf00002	1
AX-157892527	1	31.443	KV452698.1	904847	1-scf00002	1
AX-157892529	1	31.443	KV452698.1	907479	1-scf00002	1
AX-157751990	1	31.443	KV452698.1	907529	1-scf00002	1
AX-157892532	1	31.443	KV452698.1	914021	1-scf00002	1
AX-157892540	1	31.443	KV452698.1	927638	1-scf00002	1
AX-157892534	1	32.006	KV452698.1	919440	1-scf00002	1
AX-157745583	1	32.006	KV452698.1	921688	1-scf00002	1
AX-157892536	1	32.006	KV452698.1	922506	1-scf00002	1
AX-157892537	1	32.006	KV452698.1	923478	1-scf00002	1
AX-157750444	1	32.006	KV452698.1	925332	1-scf00002	1
AX-157892539	1	32.092	KV452698.1	926580	1-scf00002	1
AX-157892541	1	32.218	KV452698.1	929062	1-scf00002	1
AX-157771538	1	32.422	KV452698.1	930110	1-scf00002	1
AX-157892544	1	32.427	KV452698.1	931636	1-scf00002	1
AX-157892547	1	32.427	KV452698.1	934972	1-scf00002	1

AX-157892549	1	32.427	KV452698.1	938525	1-scf00002	1
AX-157892552	1	32.427	KV452698.1	941752	1-scf00002	1
AX-157892553	1	32.427	KV452698.1	942982	1-scf00002	1
AX-157892554	1	32.427	KV452698.1	944326	1-scf00002	1
AX-157723048	1	32.427	KV452698.1	947402	1-scf00002	1
AX-157755696	1	32.863	KV452698.1	946419	1-scf00002	1
AX-157892559	1	32.863	KV452698.1	956205	1-scf00002	1
AX-157892562	1	32.863	KV452698.1	958003	1-scf00002	1
AX-157892563	1	32.863	KV452698.1	958803	1-scf00002	1
AX-157892564	1	32.863	KV452698.1	959447	1-scf00002	1
AX-157892565	1	32.863	KV452698.1	960067	1-scf00002	1
AX-157892566	1	32.863	KV452698.1	960983	1-scf00002	1
AX-157892568	1	32.863	KV452698.1	964482	1-scf00002	1
AX-157892569	1	32.863	KV452698.1	966056	1-scf00002	1
AX-157892588	1	32.863	KV452698.1	1012159	1-scf00002	1
AX-157892610	1	32.863	KV452698.1	1050878	1-scf00002	1
AX-157892571	1	32.947	KV452698.1	967856	1-scf00002	1
AX-157740432	1	33.028	KV452698.1	981299	1-scf00002	1
AX-157892580	1	33.028	KV452698.1	982249	1-scf00002	1
AX-157810430	1	33.111	KV452698.1	995070	1-scf00002	1
AX-157892584	1	33.191	KV452698.1	997934	1-scf00002	1
AX-157744604	1	33.191	KV452698.1	1005201	1-scf00002	1
AX-157810433	1	33.191	KV452698.1	1005282	1-scf00002	1
AX-157892587	1	33.191	KV452698.1	1007790	1-scf00002	1
AX-157892589	1	33.191	KV452698.1	1012571	1-scf00002	1
AX-157810434	1	33.191	KV452698.1	1014128	1-scf00002	1
AX-157892591	1	33.191	KV452698.1	1016034	1-scf00002	1
AX-157892593	1	33.191	KV452698.1	1017497	1-scf00002	1
AX-157745771	1	33.191	KV452698.1	1017584	1-scf00002	1
AX-157892594	1	33.191	KV452698.1	1020366	1-scf00002	1
AX-157892596	1	33.191	KV452698.1	1022813	1-scf00002	1
AX-157892597	1	33.191	KV452698.1	1028653	1-scf00002	1
AX-157892598	1	33.191	KV452698.1	1030641	1-scf00002	1
AX-157892599	1	33.191	KV452698.1	1031541	1-scf00002	1
AX-157749280	1	33.191	KV452698.1	1032824	1-scf00002	1
AX-157892600	1	33.191	KV452698.1	1033345	1-scf00002	1
AX-157868475	1	33.191	KV452698.1	1038015	1-scf00002	1
AX-157745132	1	33.191	KV452698.1	1039146	1-scf00002	1
AX-157892633	1	33.191	KV452698.1	1085318	1-scf00002	1
AX-157892644	1	33.191	KV452698.1	1105786	1-scf00002	1
AX-157892690	1	33.191	KV452698.1	1164255	1-scf00002	1
AX-157893635	1	33.191	KV452698.1	1975338	1-scf00002	1
AX-157893652	1	33.191	KV452698.1	2021321	1-scf00002	1
AX-157723914	1	33.191	KV452698.1	2050896	1-scf00002	1
AX-157893040	1	33.191	KV452700.1	45231	1-scf00005	1
AX-157893046	1	33.191	KV452700.1	54590	1-scf00005	1
AX-157893059	1	33.191	KV452700.1	71830	1-scf00005	1
AX-157893066	1	33.191	KV452700.1	76867	1-scf00005	1
AX-157893070	1	33.191	KV452700.1	83425	1-scf00005	1
AX-157893071	1	33.191	KV452700.1	83935	1-scf00005	1
AX-157893073	1	33.191	KV452700.1	95641	1-scf00005	1

AX-157893074	1	33.191	KV452700.1	97287	1-scf00005	1
AX-157893083	1	33.191	KV452700.1	108788	1-scf00005	1
AX-157753148	1	33.191	KV452700.1	109960	1-scf00005	1
AX-157893088	1	33.191	KV452700.1	113814	1-scf00005	1
AX-157893090	1	33.191	KV452700.1	119099	1-scf00005	1
AX-157810493	1	33.191	KV452700.1	120723	1-scf00005	1
AX-157893091	1	33.191	KV452700.1	121976	1-scf00005	1
AX-157893155	1	33.191	KV452700.1	197949	1-scf00005	1
AX-157893177	1	33.191	KV452700.1	222938	1-scf00005	1
AX-157893495	1	33.191	KV452700.1	711249	1-scf00005	1
AX-157892603	1	33.194	KV452698.1	1042167	1-scf00002	1
AX-157810437	1	33.553	KV452698.1	1043227	1-scf00002	1
AX-157700592	1	33.555	KV452698.1	1044906	1-scf00002	1
AX-157892613	1	33.555	KV452698.1	1056984	1-scf00002	1
AX-157892625	1	33.555	KV452698.1	1078937	1-scf00002	1
AX-157810429	1	33.737	KV452698.1	979561	1-scf00002	1
AX-157892592	1	33.737	KV452698.1	1016869	1-scf00002	1
AX-157892626	1	33.737	KV452698.1	1079461	1-scf00002	1
AX-157740949	1	33.737	KV452698.1	1096637	1-scf00002	1
AX-157810440	1	33.737	KV452698.1	1098048	1-scf00002	1
AX-157892641	1	33.737	KV452698.1	1098902	1-scf00002	1
AX-157892642	1	33.737	KV452698.1	1099487	1-scf00002	1
AX-157892643	1	33.737	KV452698.1	1103524	1-scf00002	1
AX-157892649	1	33.737	KV452698.1	1111558	1-scf00002	1
AX-157892650	1	33.752	KV452698.1	1112069	1-scf00002	1
AX-157892655	1	33.781	KV452698.1	1117260	1-scf00002	1
AX-157892659	1	33.847	KV452698.1	1124232	1-scf00002	1
AX-157892660	1	33.847	KV452698.1	1125545	1-scf00002	1
AX-157892662	1	33.847	KV452698.1	1128216	1-scf00002	1
AX-157892663	1	33.847	KV452698.1	1131056	1-scf00002	1
AX-157892665	1	33.847	KV452698.1	1137140	1-scf00002	1
AX-157892666	1	33.847	KV452698.1	1137847	1-scf00002	1
AX-157892667	1	33.847	KV452698.1	1138955	1-scf00002	1
AX-157892670	1	33.847	KV452698.1	1141893	1-scf00002	1
AX-157892671	1	33.847	KV452698.1	1144114	1-scf00002	1
AX-157743212	1	33.847	KV452698.1	1145828	1-scf00002	1
AX-157892674	1	33.847	KV452698.1	1147579	1-scf00002	1
AX-157892678	1	33.847	KV452698.1	1153920	1-scf00002	1
AX-157892573	1	33.956	KV452698.1	969687	1-scf00002	1
AX-157892615	1	33.956	KV452698.1	1059437	1-scf00002	1
AX-157874268	1	33.956	KV452698.1	1064356	1-scf00002	1
AX-157810438	1	33.956	KV452698.1	1080703	1-scf00002	1
AX-157892630	1	33.956	KV452698.1	1082034	1-scf00002	1
AX-157892635	1	33.956	KV452698.1	1088330	1-scf00002	1
AX-157892638	1	33.956	KV452698.1	1090945	1-scf00002	1
AX-157892639	1	33.956	KV452698.1	1091717	1-scf00002	1
AX-157892657	1	33.956	KV452698.1	1122507	1-scf00002	1
AX-157892664	1	33.956	KV452698.1	1131415	1-scf00002	1
AX-157892668	1	33.956	KV452698.1	1140113	1-scf00002	1
AX-157892682	1	33.956	KV452698.1	1157282	1-scf00002	1
AX-157892688	1	33.956	KV452698.1	1161351	1-scf00002	1

AX-157892691	1	33.956	KV452698.1	1169044	1-scf00002	1
AX-157892692	1	33.956	KV452698.1	1169759	1-scf00002	1
AX-157892693	1	33.956	KV452698.1	1170285	1-scf00002	1
AX-157892695	1	33.956	KV452698.1	1172141	1-scf00002	1
AX-157810442	1	33.956	KV452698.1	1175864	1-scf00002	1
AX-157716536	1	33.956	KV452698.1	1186025	1-scf00002	1
AX-157892697	1	33.956	KV452698.1	1189183	1-scf00002	1
AX-157892698	1	33.956	KV452698.1	1189528	1-scf00002	1
AX-157892699	1	33.956	KV452698.1	1192038	1-scf00002	1
AX-157892700	1	33.956	KV452698.1	1193839	1-scf00002	1
AX-157892701	1	33.956	KV452698.1	1198110	1-scf00002	1
AX-157892702	1	33.956	KV452698.1	1201697	1-scf00002	1
AX-157892703	1	33.956	KV452698.1	1203777	1-scf00002	1
AX-157892705	1	33.956	KV452698.1	1208382	1-scf00002	1
AX-157892706	1	33.956	KV452698.1	1211226	1-scf00002	1
AX-157892710	1	33.956	KV452698.1	1219890	1-scf00002	1
AX-157892711	1	33.956	KV452698.1	1220566	1-scf00002	1
AX-157892716	1	33.956	KV452698.1	1229530	1-scf00002	1
AX-157892717	1	33.956	KV452698.1	1230965	1-scf00002	1
AX-157892718	1	33.956	KV452698.1	1233967	1-scf00002	1
AX-157892719	1	33.956	KV452698.1	1234467	1-scf00002	1
AX-157892720	1	33.956	KV452698.1	1235109	1-scf00002	1
AX-157892721	1	33.956	KV452698.1	1236314	1-scf00002	1
AX-157892722	1	33.956	KV452698.1	1237148	1-scf00002	1
AX-157853544	1	33.956	KV452698.1	1246167	1-scf00002	1
AX-157892727	1	33.956	KV452698.1	1249673	1-scf00002	1
AX-157810444	1	33.956	KV452698.1	1279497	1-scf00002	1
AX-157892739	1	33.956	KV452698.1	1289240	1-scf00002	1
AX-157892741	1	33.956	KV452698.1	1291663	1-scf00002	1
AX-157892742	1	33.956	KV452698.1	1293792	1-scf00002	1
AX-157892743	1	33.956	KV452698.1	1295257	1-scf00002	1
AX-157892744	1	33.956	KV452698.1	1295723	1-scf00002	1
AX-157892745	1	33.956	KV452698.1	1305404	1-scf00002	1
AX-157892746	1	33.956	KV452698.1	1310912	1-scf00002	1
AX-157892747	1	33.956	KV452698.1	1311139	1-scf00002	1
AX-157892748	1	33.956	KV452698.1	1311961	1-scf00002	1
AX-157892749	1	33.956	KV452698.1	1314112	1-scf00002	1
AX-157892751	1	33.956	KV452698.1	1316127	1-scf00002	1
AX-157892752	1	33.956	KV452698.1	1316903	1-scf00002	1
AX-157892757	1	33.956	KV452698.1	1328536	1-scf00002	1
AX-157892762	1	33.956	KV452698.1	1338899	1-scf00002	1
AX-158404244	1	33.956	KV452698.1	1366824	1-scf00002	1
AX-157892774	1	33.956	KV452698.1	1378578	1-scf00002	1
AX-157892775	1	33.956	KV452698.1	1380191	1-scf00002	1
AX-158379106	1	33.956	KV452698.1	1381415	1-scf00002	1
AX-157892776	1	33.956	KV452698.1	1385290	1-scf00002	1
AX-157892777	1	33.956	KV452698.1	1386187	1-scf00002	1
AX-157892778	1	33.956	KV452698.1	1390435	1-scf00002	1
AX-157892779	1	33.956	KV452698.1	1392412	1-scf00002	1
AX-157892780	1	33.956	KV452698.1	1392482	1-scf00002	1
AX-157892781	1	33.956	KV452698.1	1392722	1-scf00002	1

AX-157892782	1	33.956	KV452698.1	1398419	1-scf00002	1
AX-157892783	1	33.956	KV452698.1	1400385	1-scf00002	1
AX-157892784	1	33.956	KV452698.1	1404949	1-scf00002	1
AX-157892786	1	33.956	KV452698.1	1409194	1-scf00002	1
AX-157892789	1	33.956	KV452698.1	1411302	1-scf00002	1
AX-157892790	1	33.956	KV452698.1	1414008	1-scf00002	1
AX-157892791	1	33.956	KV452698.1	1415029	1-scf00002	1
AX-157892792	1	33.956	KV452698.1	1417913	1-scf00002	1
AX-157892793	1	33.956	KV452698.1	1422578	1-scf00002	1
AX-157810448	1	33.956	KV452698.1	1424729	1-scf00002	1
AX-157810449	1	33.956	KV452698.1	1426861	1-scf00002	1
AX-157810452	1	33.956	KV452698.1	1441872	1-scf00002	1
AX-157892800	1	33.956	KV452698.1	1448364	1-scf00002	1
AX-157892801	1	33.956	KV452698.1	1465971	1-scf00002	1
AX-157892802	1	33.956	KV452698.1	1466395	1-scf00002	1
AX-157892803	1	33.956	KV452698.1	1466893	1-scf00002	1
AX-157810454	1	33.956	KV452698.1	1468278	1-scf00002	1
AX-157810455	1	33.956	KV452698.1	1470995	1-scf00002	1
AX-157810456	1	33.956	KV452698.1	1471591	1-scf00002	1
AX-157892805	1	33.956	KV452698.1	1472049	1-scf00002	1
AX-157892808	1	33.956	KV452698.1	1478685	1-scf00002	1
AX-157892809	1	33.956	KV452698.1	1482762	1-scf00002	1
AX-157892810	1	33.956	KV452698.1	1483636	1-scf00002	1
AX-157892811	1	33.956	KV452698.1	1487423	1-scf00002	1
AX-157892812	1	33.956	KV452698.1	1489668	1-scf00002	1
AX-157892813	1	33.956	KV452698.1	1491063	1-scf00002	1
AX-157892816	1	33.956	KV452698.1	1495768	1-scf00002	1
AX-157892819	1	33.956	KV452698.1	1501736	1-scf00002	1
AX-157892821	1	33.956	KV452698.1	1507356	1-scf00002	1
AX-157892824	1	33.956	KV452698.1	1515244	1-scf00002	1
AX-157892826	1	33.956	KV452698.1	1516550	1-scf00002	1
AX-157892831	1	33.956	KV452698.1	1525076	1-scf00002	1
AX-157892832	1	33.956	KV452698.1	1525567	1-scf00002	1
AX-157892835	1	33.956	KV452698.1	1530277	1-scf00002	1
AX-157892836	1	33.956	KV452698.1	1532275	1-scf00002	1
AX-157892839	1	33.956	KV452698.1	1535547	1-scf00002	1
AX-157892842	1	33.956	KV452698.1	1539867	1-scf00002	1
AX-157810459	1	33.956	KV452698.1	1562508	1-scf00002	1
AX-157810461	1	33.956	KV452698.1	1597029	1-scf00002	1
AX-157810462	1	33.956	KV452698.1	1597139	1-scf00002	1
AX-157892872	1	33.956	KV452698.1	1604911	1-scf00002	1
AX-157892873	1	33.956	KV452698.1	1605638	1-scf00002	1
AX-157892874	1	33.956	KV452698.1	1607950	1-scf00002	1
AX-157892877	1	33.956	KV452698.1	1611944	1-scf00002	1
AX-157892878	1	33.956	KV452698.1	1612872	1-scf00002	1
AX-157892881	1	33.956	KV452698.1	1616486	1-scf00002	1
AX-157892882	1	33.956	KV452698.1	1619209	1-scf00002	1
AX-157892886	1	33.956	KV452698.1	1624674	1-scf00002	1
AX-157702714	1	33.956	KV452698.1	1626481	1-scf00002	1
AX-157892887	1	33.956	KV452698.1	1629365	1-scf00002	1
AX-157892888	1	33.956	KV452698.1	1629519	1-scf00002	1

AX-157892889	1	33.956	KV452698.1	1631037	1-scf00002	1
AX-157892898	1	33.956	KV452698.1	1645821	1-scf00002	1
AX-157892900	1	33.956	KV452698.1	1649045	1-scf00002	1
AX-157892906	1	33.956	KV452698.1	1667887	1-scf00002	1
AX-157892909	1	33.956	KV452698.1	1671139	1-scf00002	1
AX-157892910	1	33.956	KV452698.1	1671799	1-scf00002	1
AX-157892911	1	33.956	KV452698.1	1676254	1-scf00002	1
AX-157892912	1	33.956	KV452698.1	1678680	1-scf00002	1
AX-157892913	1	33.956	KV452698.1	1681187	1-scf00002	1
AX-157892916	1	33.956	KV452698.1	1685050	1-scf00002	1
AX-157892917	1	33.956	KV452698.1	1688243	1-scf00002	1
AX-157892918	1	33.956	KV452698.1	1689047	1-scf00002	1
AX-157742265	1	33.956	KV452698.1	1689110	1-scf00002	1
AX-157892919	1	33.956	KV452698.1	1689815	1-scf00002	1
AX-157713839	1	33.956	KV452698.1	1690085	1-scf00002	1
AX-157862579	1	33.956	KV452698.1	1692375	1-scf00002	1
AX-157892920	1	33.956	KV452698.1	1693110	1-scf00002	1
AX-157892921	1	33.956	KV452698.1	1693575	1-scf00002	1
AX-157892922	1	33.956	KV452698.1	1694052	1-scf00002	1
AX-157892923	1	33.956	KV452698.1	1694934	1-scf00002	1
AX-157892924	1	33.956	KV452698.1	1695136	1-scf00002	1
AX-157892925	1	33.956	KV452698.1	1696468	1-scf00002	1
AX-157892926	1	33.956	KV452698.1	1703190	1-scf00002	1
AX-157892928	1	33.956	KV452698.1	1706633	1-scf00002	1
AX-157892929	1	33.956	KV452698.1	1707840	1-scf00002	1
AX-157810470	1	33.956	KV452698.1	1723900	1-scf00002	1
AX-157810471	1	33.956	KV452698.1	1724104	1-scf00002	1
AX-157892935	1	33.956	KV452698.1	1724804	1-scf00002	1
AX-157810473	1	33.956	KV452698.1	1727555	1-scf00002	1
AX-157892936	1	33.956	KV452698.1	1729170	1-scf00002	1
AX-157892937	1	33.956	KV452698.1	1731634	1-scf00002	1
AX-157892938	1	33.956	KV452698.1	1733744	1-scf00002	1
AX-157810474	1	33.956	KV452698.1	1733976	1-scf00002	1
AX-157892940	1	33.956	KV452698.1	1740002	1-scf00002	1
AX-157719349	1	33.956	KV452698.1	1743937	1-scf00002	1
AX-157892948	1	33.956	KV452698.1	1758039	1-scf00002	1
AX-157892950	1	33.956	KV452698.1	1760046	1-scf00002	1
AX-157892952	1	33.956	KV452698.1	1761007	1-scf00002	1
AX-157892953	1	33.956	KV452698.1	1762211	1-scf00002	1
AX-157892955	1	33.956	KV452698.1	1763160	1-scf00002	1
AX-157892956	1	33.956	KV452698.1	1763807	1-scf00002	1
AX-157892957	1	33.956	KV452698.1	1765547	1-scf00002	1
AX-157892958	1	33.956	KV452698.1	1766679	1-scf00002	1
AX-157892959	1	33.956	KV452698.1	1767313	1-scf00002	1
AX-157726267	1	33.956	KV452698.1	1767916	1-scf00002	1
AX-157892960	1	33.956	KV452698.1	1769317	1-scf00002	1
AX-157892971	1	33.956	KV452698.1	1790431	1-scf00002	1
AX-157892972	1	33.956	KV452698.1	1791135	1-scf00002	1
AX-157892974	1	33.956	KV452698.1	1794068	1-scf00002	1
AX-157892977	1	33.956	KV452698.1	1796888	1-scf00002	1
AX-157892978	1	33.956	KV452698.1	1797518	1-scf00002	1

AX-157892984	1	33.956	KV452698.1	1805698	1-scf00002	1
AX-157892986	1	33.956	KV452698.1	1807712	1-scf00002	1
AX-157892990	1	33.956	KV452698.1	1810387	1-scf00002	1
AX-158388975	1	33.956	KV452698.1	1811869	1-scf00002	1
AX-157709623	1	33.956	KV452698.1	1817424	1-scf00002	1
AX-157892993	1	33.956	KV452698.1	1819971	1-scf00002	1
AX-157892994	1	33.956	KV452698.1	1820592	1-scf00002	1
AX-157892996	1	33.956	KV452698.1	1827083	1-scf00002	1
AX-157893003	1	33.956	KV452698.1	1835491	1-scf00002	1
AX-157893005	1	33.956	KV452698.1	1836625	1-scf00002	1
AX-157893006	1	33.956	KV452698.1	1838735	1-scf00002	1
AX-157893008	1	33.956	KV452698.1	1841779	1-scf00002	1
AX-157893009	1	33.956	KV452698.1	1842345	1-scf00002	1
AX-157893010	1	33.956	KV452698.1	1843438	1-scf00002	1
AX-157893011	1	33.956	KV452698.1	1844034	1-scf00002	1
AX-157810483	1	33.956	KV452698.1	1845578	1-scf00002	1
AX-157893012	1	33.956	KV452698.1	1845735	1-scf00002	1
AX-157810538	1	33.956	KV452698.1	1854328	1-scf00002	1
AX-157893588	1	33.956	KV452698.1	1855755	1-scf00002	1
AX-157893594	1	33.956	KV452698.1	1869680	1-scf00002	1
AX-157893597	1	33.956	KV452698.1	1876047	1-scf00002	1
AX-157810541	1	33.956	KV452698.1	1892515	1-scf00002	1
AX-157893599	1	33.956	KV452698.1	1893387	1-scf00002	1
AX-157893602	1	33.956	KV452698.1	1900664	1-scf00002	1
AX-157893603	1	33.956	KV452698.1	1904806	1-scf00002	1
AX-157893606	1	33.956	KV452698.1	1915419	1-scf00002	1
AX-157893636	1	33.956	KV452698.1	1977553	1-scf00002	1
AX-157893665	1	33.956	KV452698.1	2058701	1-scf00002	1
AX-157893675	1	33.956	KV452698.1	2072439	1-scf00002	1
AX-157708352	1	33.956	KV452698.1	2076825	1-scf00002	1
AX-157893680	1	33.956	KV452698.1	2078721	1-scf00002	1
AX-157893685	1	33.956	KV452698.1	2091273	1-scf00002	1
AX-157810551	1	33.956	KV452698.1	2091327	1-scf00002	1
AX-157893686	1	33.956	KV452698.1	2091760	1-scf00002	1
AX-157810552	1	33.956	KV452698.1	2093558	1-scf00002	1
AX-157893687	1	33.956	KV452698.1	2096316	1-scf00002	1
AX-158404274	1	33.956	KV452698.1	2097286	1-scf00002	1
AX-157893689	1	33.956	KV452698.1	2097517	1-scf00002	1
AX-157893690	1	33.956	KV452698.1	2100126	1-scf00002	1
AX-157893691	1	33.956	KV452698.1	2103858	1-scf00002	1
AX-157893692	1	33.956	KV452698.1	2104084	1-scf00002	1
AX-157893694	1	33.956	KV452698.1	2106277	1-scf00002	1
AX-157893695	1	33.956	KV452698.1	2107033	1-scf00002	1
AX-157893696	1	33.956	KV452698.1	2109126	1-scf00002	1
AX-157893698	1	33.956	KV452698.1	2110738	1-scf00002	1
AX-157893699	1	33.956	KV452698.1	2111421	1-scf00002	1
AX-157893702	1	33.956	KV452698.1	2116883	1-scf00002	1
AX-157893706	1	33.956	KV452698.1	2120851	1-scf00002	1
AX-158404275	1	33.956	KV452698.1	2122494	1-scf00002	1
AX-157893707	1	33.956	KV452698.1	2126208	1-scf00002	1
AX-157893708	1	33.956	KV452698.1	2126570	1-scf00002	1

AX-157893709	1	33.956	KV452698.1	2126977	1-scf00002	1
AX-157893710	1	33.956	KV452698.1	2127368	1-scf00002	1
AX-157770893	1	33.956	KV452698.1	2136533	1-scf00002	1
AX-157893138	1	33.956	KV452700.1	177449	1-scf00005	1
AX-157717543	1	33.956	KV452700.1	232235	1-scf00005	1
AX-157893194	1	33.956	KV452700.1	244115	1-scf00005	1
AX-157893205	1	33.956	KV452700.1	269980	1-scf00005	1
AX-157893218	1	33.956	KV452700.1	284764	1-scf00005	1
AX-157893391	1	33.956	KV452700.1	554830	1-scf00005	1
AX-157893499	1	33.956	KV452700.1	720592	1-scf00005	1
AX-157893584	1	33.956	KV452700.1	864277	1-scf00005	1
AX-157893897	1	33.956	KV452707.1	378107	1-scf00007	1
AX-157685190	1	33.956	KV452704.1	67401	1-scf00597	1
AX-157753971	1	33.956	KV452699.1	9483	1-scf00610	1
AX-158377554	1	33.956	KV452699.1	23691	1-scf00610	1
AX-158377556	1	33.956	KV452699.1	25796	1-scf00610	1
AX-158377561	1	33.956	KV452699.1	36074	1-scf00610	1
AX-158377563	1	33.956	KV452699.1	37343	1-scf00610	1
AX-158377564	1	33.956	KV452699.1	37956	1-scf00610	1
AX-158377565	1	33.956	KV452699.1	38499	1-scf00610	1
AX-158377566	1	33.956	KV452699.1	39149	1-scf00610	1
AX-157755343	1	33.956	KV452699.1	40367	1-scf00610	1
AX-158377568	1	33.956	KV452699.1	41424	1-scf00610	1
AX-158377569	1	33.956	KV452699.1	42044	1-scf00610	1
AX-158377571	1	33.956	KV452699.1	43636	1-scf00610	1
AX-158377575	1	33.956	KV452699.1	49094	1-scf00610	1
AX-157750007	1	33.956	KV452699.1	50450	1-scf00610	1
AX-158377577	1	33.956	KV452699.1	51161	1-scf00610	1
AX-158377578	1	33.956	KV452699.1	52133	1-scf00610	1
AX-158377580	1	33.956	KV452699.1	55029	1-scf00610	1
AX-158377581	1	33.956	KV452699.1	56219	1-scf00610	1
AX-158377582	1	33.956	KV452699.1	56522	1-scf00610	1
AX-158377583	1	33.956	KV452699.1	57216	1-scf00610	1
AX-158377584	1	33.956	KV452699.1	58169	1-scf00610	1
AX-158377588	1	33.956	KV452699.1	62106	1-scf00610	1
AX-158377590	1	33.956	KV452699.1	63571	1-scf00610	1
AX-158377600	1	33.956	KV452699.1	72453	1-scf00610	1
AX-158377602	1	33.956	KV452699.1	74723	1-scf00610	1
AX-158377616	1	33.956	KV452699.1	94147	1-scf00610	1
AX-158377630	1	33.956	KV452699.1	103629	1-scf00610	1
AX-157681771	1	33.956	KV453631.1	225	scf01063	UN
AX-157681802	1	33.956	3ML01026316	1245	scf01911_1	UN
AX-157705142	1	33.956	3ML01028779	201	scf04391_1	UN
AX-157689364	1	33.956	3ML01029325	574	scf04946_1	UN
AX-157707134	1	33.956	3ML01033571	494	scf09227_1	UN
AX-157893628	1	34.174	KV452698.1	1959955	1-scf00002	1
AX-157893629	1	34.174	KV452698.1	1960688	1-scf00002	1
AX-157810549	1	34.174	KV452698.1	1961249	1-scf00002	1
AX-157750825	1	34.174	KV452698.1	1964285	1-scf00002	1
AX-157893630	1	34.174	KV452698.1	1967295	1-scf00002	1
AX-157893632	1	34.174	KV452698.1	1969706	1-scf00002	1

AX-157893639	1	34.212	KV452698.1	1982131	1-scf00002	1
AX-158381932	1	34.212	KV452698.1	1985839	1-scf00002	1
AX-157893644	1	34.212	KV452698.1	1991651	1-scf00002	1
AX-157892806	1	34.239	KV452698.1	1474543	1-scf00002	1
AX-157893642	1	34.239	KV452698.1	1986763	1-scf00002	1
AX-157893677	1	34.239	KV452698.1	2073787	1-scf00002	1
AX-157893648	1	34.245	KV452698.1	1999681	1-scf00002	1
AX-157892982	1	34.284	KV452698.1	1803792	1-scf00002	1
AX-157892983	1	34.284	KV452698.1	1804665	1-scf00002	1
AX-157893000	1	34.284	KV452698.1	1831914	1-scf00002	1
AX-157893622	1	34.284	KV452698.1	1950351	1-scf00002	1
AX-157893634	1	34.284	KV452698.1	1972751	1-scf00002	1
AX-157893647	1	34.284	KV452698.1	1997704	1-scf00002	1
AX-157893657	1	34.284	KV452698.1	2032610	1-scf00002	1
AX-157893674	1	34.284	KV452698.1	2071414	1-scf00002	1
AX-157810553	1	34.284	KV452698.1	2096898	1-scf00002	1
AX-157893697	1	34.284	KV452698.1	2110279	1-scf00002	1
AX-157893704	1	34.284	KV452698.1	2118794	1-scf00002	1
AX-157893705	1	34.284	KV452698.1	2119494	1-scf00002	1
AX-157810554	1	34.284	KV452698.1	2127656	1-scf00002	1
AX-157740470	1	34.284	KV452698.1	2129058	1-scf00002	1
AX-157893711	1	34.284	KV452698.1	2129198	1-scf00002	1
AX-157893713	1	34.284	KV452698.1	2130410	1-scf00002	1
AX-157810555	1	34.284	KV452698.1	2130859	1-scf00002	1
AX-157893715	1	34.284	KV452698.1	2131486	1-scf00002	1
AX-157893716	1	34.284	KV452698.1	2132441	1-scf00002	1
AX-157746764	1	34.284	KV452698.1	2133041	1-scf00002	1
AX-157893717	1	34.284	KV452698.1	2133923	1-scf00002	1
AX-157893718	1	34.284	KV452698.1	2134786	1-scf00002	1
AX-157893720	1	34.284	KV452698.1	2140577	1-scf00002	1
AX-157893049	1	34.284	KV452700.1	58627	1-scf00005	1
AX-157893050	1	34.284	KV452700.1	59462	1-scf00005	1
AX-157893052	1	34.284	KV452700.1	60633	1-scf00005	1
AX-157893053	1	34.284	KV452700.1	61495	1-scf00005	1
AX-157893555	1	34.284	KV452700.1	825579	1-scf00005	1
AX-157893579	1	34.284	KV452700.1	857604	1-scf00005	1
AX-158420709	1	34.284	KV452699.1	317	1-scf00610	1
AX-158377555	1	34.284	KV452699.1	23944	1-scf00610	1
AX-158377678	1	34.284	KV452699.1	159222	1-scf00610	1
AX-157689621	1	34.284	KV453829.1	837	scf06466	UN
AX-157689882	1	34.284		239	scf10330	UN
AX-157893661	1	34.46	KV452698.1	2048303	1-scf00002	1
AX-157893051	1	34.46	KV452700.1	59843	1-scf00005	1
AX-157893662	1	34.502	KV452698.1	2050523	1-scf00002	1
AX-157893055	1	34.502	KV452700.1	63245	1-scf00005	1
AX-157810490	1	34.502	KV452700.1	65172	1-scf00005	1
AX-157717740	1	34.502	KV452700.1	65942	1-scf00005	1
AX-157893057	1	34.502	KV452700.1	68747	1-scf00005	1
AX-157893065	1	34.502	KV452700.1	76381	1-scf00005	1
AX-157893069	1	34.502	KV452700.1	82724	1-scf00005	1
AX-158403760	1	34.502	KV452700.1	84561	1-scf00005	1

AX-157893078	1	34.502	KV452700.1	105697	1-scf00005	1
AX-157810495	1	34.502	KV452700.1	121419	1-scf00005	1
AX-157810497	1	34.502	KV452700.1	122729	1-scf00005	1
AX-157893092	1	34.502	KV452700.1	123618	1-scf00005	1
AX-157893093	1	34.502	KV452700.1	126351	1-scf00005	1
AX-157893094	1	34.502	KV452700.1	126759	1-scf00005	1
AX-157893095	1	34.502	KV452700.1	127000	1-scf00005	1
AX-157893096	1	34.502	KV452700.1	127258	1-scf00005	1
AX-158404259	1	34.502	KV452700.1	127928	1-scf00005	1
AX-157893097	1	34.502	KV452700.1	128258	1-scf00005	1
AX-157893098	1	34.502	KV452700.1	128579	1-scf00005	1
AX-157893099	1	34.502	KV452700.1	129017	1-scf00005	1
AX-157893100	1	34.502	KV452700.1	129309	1-scf00005	1
AX-157893102	1	34.502	KV452700.1	131244	1-scf00005	1
AX-157893103	1	34.502	KV452700.1	132283	1-scf00005	1
AX-157893104	1	34.502	KV452700.1	132493	1-scf00005	1
AX-157893105	1	34.502	KV452700.1	133270	1-scf00005	1
AX-157893106	1	34.502	KV452700.1	133482	1-scf00005	1
AX-157893107	1	34.502	KV452700.1	135329	1-scf00005	1
AX-157893122	1	34.502	KV452700.1	151927	1-scf00005	1
AX-157810500	1	34.502	KV452700.1	174934	1-scf00005	1
AX-157893134	1	34.611	KV452700.1	172539	1-scf00005	1
AX-157893137	1	34.611	KV452700.1	176621	1-scf00005	1
AX-157810502	1	34.611	KV452700.1	176979	1-scf00005	1
AX-157893150	1	34.611	KV452700.1	188947	1-scf00005	1
AX-157893164	1	34.611	KV452700.1	209435	1-scf00005	1
AX-157810505	1	34.611	KV452700.1	212624	1-scf00005	1
AX-157718119	1	34.611	KV452700.1	215323	1-scf00005	1
AX-157893171	1	34.611	KV452700.1	218691	1-scf00005	1
AX-157893174	1	34.611	KV452700.1	221232	1-scf00005	1
AX-157745264	1	34.611	KV452700.1	221880	1-scf00005	1
AX-157893175	1	34.611	KV452700.1	222095	1-scf00005	1
AX-157893176	1	34.611	KV452700.1	222320	1-scf00005	1
AX-157893180	1	34.611	KV452700.1	227911	1-scf00005	1
AX-157893181	1	34.611	KV452700.1	228381	1-scf00005	1
AX-157893182	1	34.611	KV452700.1	228590	1-scf00005	1
AX-157893184	1	34.611	KV452700.1	230849	1-scf00005	1
AX-157893189	1	34.611	KV452700.1	238765	1-scf00005	1
AX-157893190	1	34.611	KV452700.1	239344	1-scf00005	1
AX-158414534	1	34.611	KV452700.1	240667	1-scf00005	1
AX-157893199	1	34.611	KV452700.1	253852	1-scf00005	1
AX-157893200	1	34.611	KV452700.1	254399	1-scf00005	1
AX-157893201	1	34.611	KV452700.1	256628	1-scf00005	1
AX-157893202	1	34.611	KV452700.1	257421	1-scf00005	1
AX-157893216	1	34.611	KV452700.1	281791	1-scf00005	1
AX-157893217	1	34.611	KV452700.1	283290	1-scf00005	1
AX-157718475	1	34.611	KV452700.1	300209	1-scf00005	1
AX-158381919	1	34.611	KV452700.1	300925	1-scf00005	1
AX-157893229	1	34.611	KV452700.1	310867	1-scf00005	1
AX-157893232	1	34.611	KV452700.1	315118	1-scf00005	1
AX-157893237	1	34.611	KV452700.1	321513	1-scf00005	1

AX-157893238	1	34.611	KV452700.1	325146	1-scf00005	1
AX-157893239	1	34.611	KV452700.1	325501	1-scf00005	1
AX-158404263	1	34.611	KV452700.1	327350	1-scf00005	1
AX-157893241	1	34.611	KV452700.1	327844	1-scf00005	1
AX-157893243	1	34.611	KV452700.1	329280	1-scf00005	1
AX-157893244	1	34.611	KV452700.1	329785	1-scf00005	1
AX-157893245	1	34.611	KV452700.1	329897	1-scf00005	1
AX-157893246	1	34.611	KV452700.1	330400	1-scf00005	1
AX-157893248	1	34.611	KV452700.1	332069	1-scf00005	1
AX-157893249	1	34.611	KV452700.1	332562	1-scf00005	1
AX-157893250	1	34.611	KV452700.1	332864	1-scf00005	1
AX-157893251	1	34.611	KV452700.1	333458	1-scf00005	1
AX-157893252	1	34.611	KV452700.1	334695	1-scf00005	1
AX-158414537	1	34.611	KV452700.1	348721	1-scf00005	1
AX-157810511	1	34.611	KV452700.1	387171	1-scf00005	1
AX-157810523	1	34.611	KV452700.1	546862	1-scf00005	1
AX-157853547	1	34.611	KV452700.1	568537	1-scf00005	1
AX-157893402	1	34.611	KV452700.1	571466	1-scf00005	1
AX-157893444	1	34.611	KV452700.1	628089	1-scf00005	1
AX-157709310	1	34.611	KV452700.1	652420	1-scf00005	1
AX-158377608	1	34.611	KV452699.1	80880	1-scf00610	1
AX-157893259	1	34.638	KV452700.1	341236	1-scf00005	1
AX-157893192	1	34.72	KV452700.1	241590	1-scf00005	1
AX-157893262	1	34.72	KV452700.1	345461	1-scf00005	1
AX-158379109	1	34.72	KV452700.1	361061	1-scf00005	1
AX-157713712	1	34.72	KV452700.1	363403	1-scf00005	1
AX-157748364	1	34.72	KV452700.1	366145	1-scf00005	1
AX-157893290	1	34.72	KV452700.1	404431	1-scf00005	1
AX-157893311	1	34.72	KV452700.1	440891	1-scf00005	1
AX-157893316	1	34.72	KV452700.1	452021	1-scf00005	1
AX-157853545	1	34.72	KV452700.1	452871	1-scf00005	1
AX-157893329	1	34.72	KV452700.1	470389	1-scf00005	1
AX-157893342	1	34.72	KV452700.1	484484	1-scf00005	1
AX-157893343	1	34.72	KV452700.1	485164	1-scf00005	1
AX-157893344	1	34.72	KV452700.1	485634	1-scf00005	1
AX-157893345	1	34.72	KV452700.1	487050	1-scf00005	1
AX-157893265	1	34.83	KV452700.1	354558	1-scf00005	1
AX-157893275	1	34.83	KV452700.1	384978	1-scf00005	1
AX-157893330	1	34.83	KV452700.1	471827	1-scf00005	1
AX-157893333	1	34.83	KV452700.1	474571	1-scf00005	1
AX-157893335	1	34.83	KV452700.1	475539	1-scf00005	1
AX-157893338	1	34.83	KV452700.1	478567	1-scf00005	1
AX-157893346	1	34.83	KV452700.1	487825	1-scf00005	1
AX-157893348	1	34.83	KV452700.1	490111	1-scf00005	1
AX-157893349	1	34.83	KV452700.1	490452	1-scf00005	1
AX-157893350	1	34.83	KV452700.1	490811	1-scf00005	1
AX-157893351	1	34.83	KV452700.1	491295	1-scf00005	1
AX-157893352	1	34.83	KV452700.1	491678	1-scf00005	1
AX-157893353	1	34.83	KV452700.1	492179	1-scf00005	1
AX-157893356	1	34.83	KV452700.1	495415	1-scf00005	1
AX-157893363	1	34.83	KV452700.1	510100	1-scf00005	1

AX-157893367	1	34.83	KV452700.1	512963	1-scf00005	1
AX-157893374	1	34.83	KV452700.1	523206	1-scf00005	1
AX-157810518	1	34.83	KV452700.1	530020	1-scf00005	1
AX-157893377	1	34.83	KV452700.1	530591	1-scf00005	1
AX-157893384	1	34.83	KV452700.1	542465	1-scf00005	1
AX-157893395	1	34.83	KV452700.1	560888	1-scf00005	1
AX-157893400	1	34.83	KV452700.1	568753	1-scf00005	1
AX-157893401	1	34.83	KV452700.1	570035	1-scf00005	1
AX-157748785	1	34.83	KV452700.1	573139	1-scf00005	1
AX-157883056	1	34.83	KV452700.1	574601	1-scf00005	1
AX-157893427	1	34.83	KV452700.1	600561	1-scf00005	1
AX-157893446	1	34.83	KV452700.1	630062	1-scf00005	1
AX-157893491	1	34.83	KV452700.1	707336	1-scf00005	1
AX-157893517	1	34.83	KV452700.1	743017	1-scf00005	1
AX-157893419	1	34.887	KV452700.1	594041	1-scf00005	1
AX-157893649	1	34.939	KV452698.1	2003225	1-scf00002	1
AX-157893654	1	34.939	KV452698.1	2027722	1-scf00002	1
AX-157893054	1	34.939	KV452700.1	62035	1-scf00005	1
AX-157893056	1	34.939	KV452700.1	65351	1-scf00005	1
AX-157893085	1	34.939	KV452700.1	111610	1-scf00005	1
AX-157755463	1	34.939	KV452700.1	152511	1-scf00005	1
AX-157893151	1	34.939	KV452700.1	189177	1-scf00005	1
AX-157893225	1	34.939	KV452700.1	293975	1-scf00005	1
AX-157893236	1	34.939	KV452700.1	320844	1-scf00005	1
AX-157893253	1	34.939	KV452700.1	335025	1-scf00005	1
AX-157893261	1	34.939	KV452700.1	344131	1-scf00005	1
AX-157810507	1	34.939	KV452700.1	355567	1-scf00005	1
AX-157893268	1	34.939	KV452700.1	368129	1-scf00005	1
AX-157893289	1	34.939	KV452700.1	402992	1-scf00005	1
AX-157893295	1	34.939	KV452700.1	417463	1-scf00005	1
AX-157853546	1	34.939	KV452700.1	453682	1-scf00005	1
AX-157810515	1	34.939	KV452700.1	486191	1-scf00005	1
AX-157710727	1	34.939	KV452700.1	524934	1-scf00005	1
AX-157893392	1	34.939	KV452700.1	555832	1-scf00005	1
AX-157893407	1	34.939	KV452700.1	579540	1-scf00005	1
AX-157893410	1	34.939	KV452700.1	582397	1-scf00005	1
AX-157773107	1	34.939	KV452700.1	587027	1-scf00005	1
AX-157893421	1	34.939	KV452700.1	595397	1-scf00005	1
AX-157893423	1	34.939	KV452700.1	596964	1-scf00005	1
AX-157893425	1	34.939	KV452700.1	599441	1-scf00005	1
AX-157893426	1	34.939	KV452700.1	599849	1-scf00005	1
AX-157893430	1	34.939	KV452700.1	602409	1-scf00005	1
AX-157893436	1	34.939	KV452700.1	609578	1-scf00005	1
AX-157893440	1	34.939	KV452700.1	618631	1-scf00005	1
AX-157893443	1	34.939	KV452700.1	625395	1-scf00005	1
AX-157893445	1	34.939	KV452700.1	629398	1-scf00005	1
AX-157893448	1	34.939	KV452700.1	631382	1-scf00005	1
AX-157893449	1	34.939	KV452700.1	631714	1-scf00005	1
AX-157810530	1	34.939	KV452700.1	631839	1-scf00005	1
AX-157714942	1	34.939	KV452700.1	635858	1-scf00005	1
AX-157893457	1	34.939	KV452700.1	644112	1-scf00005	1

AX-157893458	1	34.939	KV452700.1	645038	1-scf00005	1
AX-157893459	1	34.939	KV452700.1	645919	1-scf00005	1
AX-157893461	1	34.939	KV452700.1	649521	1-scf00005	1
AX-157893464	1	34.939	KV452700.1	653693	1-scf00005	1
AX-157893466	1	34.939	KV452700.1	662116	1-scf00005	1
AX-157727571	1	34.939	KV452700.1	663005	1-scf00005	1
AX-157893469	1	34.939	KV452700.1	664277	1-scf00005	1
AX-157707844	1	34.939	KV452700.1	667102	1-scf00005	1
AX-157893471	1	34.939	KV452700.1	668371	1-scf00005	1
AX-157893472	1	34.939	KV452700.1	668816	1-scf00005	1
AX-157893531	1	34.939	KV452700.1	772105	1-scf00005	1
AX-157810532	1	34.939	KV452700.1	799563	1-scf00005	1
AX-157853435	1	34.939	KV452699.1	25934	1-scf00610	1
AX-157892949	1	35.048	KV452698.1	1759812	1-scf00002	1
AX-157893468	1	35.048	KV452700.1	663874	1-scf00005	1
AX-157893475	1	35.048	KV452700.1	674954	1-scf00005	1
AX-157893476	1	35.048	KV452700.1	676524	1-scf00005	1
AX-157745747	1	35.048	KV452700.1	677296	1-scf00005	1
AX-157893477	1	35.048	KV452700.1	680613	1-scf00005	1
AX-157893478	1	35.048	KV452700.1	681645	1-scf00005	1
AX-157893479	1	35.048	KV452700.1	682687	1-scf00005	1
AX-157893480	1	35.048	KV452700.1	683442	1-scf00005	1
AX-157893484	1	35.048	KV452700.1	694214	1-scf00005	1
AX-157810565	1	35.048	KV452707.1	205291	1-scf00007	1
AX-157893875	1	35.048	KV452707.1	350674	1-scf00007	1
AX-157893905	1	35.048	KV452707.1	386504	1-scf00007	1
AX-158404270	1	35.167	KV452700.1	801263	1-scf00005	1
AX-157874269	1	35.267	KV452698.1	1067010	1-scf00002	1
AX-157892696	1	35.267	KV452698.1	1179214	1-scf00002	1
AX-157810450	1	35.267	KV452698.1	1432087	1-scf00002	1
AX-157810451	1	35.267	KV452698.1	1438585	1-scf00002	1
AX-157892899	1	35.267	KV452698.1	1648534	1-scf00002	1
AX-157810481	1	35.267	KV452698.1	1822403	1-scf00002	1
AX-157893590	1	35.267	KV452698.1	1857812	1-scf00002	1
AX-157810506	1	35.267	KV452700.1	213521	1-scf00005	1
AX-157810525	1	35.267	KV452700.1	553875	1-scf00005	1
AX-157893506	1	35.267	KV452700.1	730123	1-scf00005	1
AX-158381926	1	35.267	KV452700.1	737827	1-scf00005	1
AX-157893513	1	35.267	KV452700.1	740649	1-scf00005	1
AX-157893558	1	35.267	KV452700.1	828736	1-scf00005	1
AX-157773076	1	35.267	KV452707.1	227214	1-scf00007	1
AX-157893910	1	35.267	KV452707.1	392465	1-scf00007	1
AX-157687600	1	35.267	KV452705.1	2457	1-scf00946	1
AX-158379111	1	35.272	KV452700.1	742943	1-scf00005	1
AX-157893521	1	35.277	KV452700.1	754880	1-scf00005	1
AX-157893523	1	35.282	KV452700.1	756954	1-scf00005	1
AX-157893530	1	35.289	KV452700.1	770269	1-scf00005	1
AX-157752919	1	35.294	KV452700.1	783018	1-scf00005	1
AX-158388978	1	35.303	KV452700.1	363635	1-scf00005	1
AX-157893284	1	35.303	KV452700.1	399555	1-scf00005	1
AX-158381928	1	35.303	KV452700.1	788044	1-scf00005	1

AX-157810531	1	35.317	KV452700.1	791587	1-scf00005	1
AX-157893538	1	35.33	KV452700.1	793643	1-scf00005	1
AX-157758230	1	35.346	KV452700.1	796851	1-scf00005	1
AX-157893282	1	35.362	KV452700.1	398337	1-scf00005	1
AX-157893510	1	35.362	KV452700.1	737566	1-scf00005	1
AX-157893539	1	35.362	KV452700.1	798199	1-scf00005	1
AX-158404269	1	35.376	KV452700.1	453398	1-scf00005	1
AX-157893527	1	35.376	KV452700.1	766889	1-scf00005	1
AX-157810533	1	35.376	KV452700.1	800392	1-scf00005	1
AX-157893447	1	35.382	KV452700.1	630713	1-scf00005	1
AX-157893541	1	35.382	KV452700.1	804674	1-scf00005	1
AX-157893544	1	35.496	KV452700.1	807917	1-scf00005	1
AX-157744430	1	35.496	KV452700.1	810117	1-scf00005	1
AX-157893547	1	35.496	KV452700.1	813073	1-scf00005	1
AX-157893549	1	35.496	KV452700.1	815769	1-scf00005	1
AX-157893540	1	35.561	KV452700.1	798751	1-scf00005	1
AX-157893552	1	35.561	KV452700.1	821277	1-scf00005	1
AX-157810562	1	35.561	KV452707.1	175848	1-scf00007	1
AX-157893864	1	35.561	KV452707.1	336076	1-scf00007	1
AX-157893906	1	35.561	KV452707.1	387159	1-scf00007	1
AX-157681501	1	35.561	3ML01025279	2877	scf01061_1	UN
AX-157892798	1	35.616	KV452698.1	1439991	1-scf00002	1
AX-157893502	1	35.616	KV452700.1	726869	1-scf00005	1
AX-157893536	1	35.616	KV452700.1	786598	1-scf00005	1
AX-157893557	1	35.616	KV452700.1	828527	1-scf00005	1
AX-157893560	1	35.616	KV452700.1	830099	1-scf00005	1
AX-157893561	1	35.616	KV452700.1	831752	1-scf00005	1
AX-157893564	1	35.616	KV452700.1	835236	1-scf00005	1
AX-157893568	1	35.616	KV452700.1	839500	1-scf00005	1
AX-157893571	1	35.616	KV452700.1	841815	1-scf00005	1
AX-157893572	1	35.616	KV452700.1	842211	1-scf00005	1
AX-157810563	1	35.616	KV452707.1	186656	1-scf00007	1
AX-157810574	1	35.616	KV452707.1	265168	1-scf00007	1
AX-157893582	1	35.618	KV452700.1	862679	1-scf00005	1
AX-157687599	1	35.812	KV452705.1	1627	1-scf00946	1
AX-157893781	1	36.581	KV452707.1	194745	1-scf00007	1
AX-157893845	1	36.581	KV452707.1	312231	1-scf00007	1
AX-157893872	1	36.581	KV452707.1	348216	1-scf00007	1
AX-157893931	1	36.581	KV452707.1	417758	1-scf00007	1
AX-157893958	1	36.581	KV452707.1	467051	1-scf00007	1
AX-157893980	1	36.581	KV452707.1	497268	1-scf00007	1
AX-157893994	1	36.581	KV452707.1	514233	1-scf00007	1
AX-157893995	1	36.581	KV452707.1	516494	1-scf00007	1
AX-157893996	1	36.581	KV452707.1	517113	1-scf00007	1
AX-157893997	1	36.581	KV452707.1	517511	1-scf00007	1
AX-157893998	1	36.581	KV452707.1	518710	1-scf00007	1
AX-157893999	1	36.581	KV452707.1	520183	1-scf00007	1
AX-157810603	1	36.581	KV452707.1	523970	1-scf00007	1
AX-157894000	1	36.581	KV452707.1	524508	1-scf00007	1
AX-157883071	1	36.581	KV452707.1	527964	1-scf00007	1
AX-157894003	1	36.581	KV452707.1	531117	1-scf00007	1

AX-157894007	1	36.581	KV452707.1	533915	1-scf00007	1
AX-157719850	1	36.581	KV452707.1	536015	1-scf00007	1
AX-157894008	1	36.581	KV452707.1	536213	1-scf00007	1
AX-157810605	1	36.581	KV452707.1	544330	1-scf00007	1
AX-157713409	1	36.581	KV452707.1	545147	1-scf00007	1
AX-157894010	1	36.581	KV452707.1	551097	1-scf00007	1
AX-157894011	1	36.581	KV452707.1	552267	1-scf00007	1
AX-157810608	1	36.581	KV452707.1	557922	1-scf00007	1
AX-157894012	1	36.581	KV452707.1	562466	1-scf00007	1
AX-157810612	1	36.581	KV452707.1	564465	1-scf00007	1
AX-157894017	1	36.581	KV452707.1	570620	1-scf00007	1
AX-157810615	1	36.581	KV452707.1	581317	1-scf00007	1
AX-157894024	1	36.581	KV452707.1	590390	1-scf00007	1
AX-157894025	1	36.581	KV452707.1	591562	1-scf00007	1
AX-157894043	1	36.581	KV452708.1	22237	1-scf00008	1
AX-157893986	1	36.622	KV452707.1	506759	1-scf00007	1
AX-157771522	1	36.653	KV452707.1	295790	1-scf00007	1
AX-157893949	1	36.653	KV452707.1	457587	1-scf00007	1
AX-157741250	1	36.653	KV452707.1	505350	1-scf00007	1
AX-157893990	1	36.653	KV452707.1	509209	1-scf00007	1
AX-157893992	1	36.653	KV452707.1	511282	1-scf00007	1
AX-157893882	1	36.69	KV452707.1	361279	1-scf00007	1
AX-157893974	1	36.69	KV452707.1	489769	1-scf00007	1
AX-157893977	1	36.69	KV452707.1	494350	1-scf00007	1
AX-157893978	1	36.69	KV452707.1	495313	1-scf00007	1
AX-157893981	1	36.69	KV452707.1	497985	1-scf00007	1
AX-157755538	1	36.69	KV452707.1	498238	1-scf00007	1
AX-157893982	1	36.69	KV452707.1	498694	1-scf00007	1
AX-157893983	1	36.69	KV452707.1	499388	1-scf00007	1
AX-157893989	1	36.69	KV452707.1	508200	1-scf00007	1
AX-157893971	1	36.716	KV452707.1	485936	1-scf00007	1
AX-157893967	1	36.755	KV452707.1	480672	1-scf00007	1
AX-157893565	1	36.8	KV452700.1	836156	1-scf00005	1
AX-158403780	1	36.8	KV452707.1	277782	1-scf00007	1
AX-157715215	1	36.8	KV452707.1	437662	1-scf00007	1
AX-157893941	1	36.8	KV452707.1	447300	1-scf00007	1
AX-157893943	1	36.8	KV452707.1	450469	1-scf00007	1
AX-157893944	1	36.8	KV452707.1	451219	1-scf00007	1
AX-157810588	1	36.8	KV452707.1	453896	1-scf00007	1
AX-157810589	1	36.8	KV452707.1	454891	1-scf00007	1
AX-157893948	1	36.8	KV452707.1	456253	1-scf00007	1
AX-157893951	1	36.8	KV452707.1	459295	1-scf00007	1
AX-157893954	1	36.8	KV452707.1	462162	1-scf00007	1
AX-157810592	1	36.8	KV452707.1	463340	1-scf00007	1
AX-157810593	1	36.8	KV452707.1	464601	1-scf00007	1
AX-157810594	1	36.8	KV452707.1	464826	1-scf00007	1
AX-157893956	1	36.8	KV452707.1	465269	1-scf00007	1
AX-157893957	1	36.8	KV452707.1	466039	1-scf00007	1
AX-157810595	1	36.8	KV452707.1	466176	1-scf00007	1
AX-158388986	1	36.8	KV452707.1	466736	1-scf00007	1
AX-157893959	1	36.8	KV452707.1	469832	1-scf00007	1

AX-157893961	1	36.8	KV452707.1	473021	1-scf00007	1
AX-157810598	1	36.8	KV452707.1	478804	1-scf00007	1
AX-157893965	1	36.8	KV452707.1	478844	1-scf00007	1
AX-157893969	1	36.8	KV452707.1	483242	1-scf00007	1
AX-157893979	1	36.8	KV452707.1	495935	1-scf00007	1
AX-157893942	1	36.829	KV452707.1	449913	1-scf00007	1
AX-157810584	1	36.859	KV452707.1	436669	1-scf00007	1
AX-157893935	1	36.886	KV452707.1	427375	1-scf00007	1
AX-158404278	1	36.909	KV452707.1	207844	1-scf00007	1
AX-157893816	1	36.909	KV452707.1	250354	1-scf00007	1
AX-157893826	1	36.909	KV452707.1	282372	1-scf00007	1
AX-157893835	1	36.909	KV452707.1	298092	1-scf00007	1
AX-158379113	1	36.909	KV452707.1	308804	1-scf00007	1
AX-157893877	1	36.909	KV452707.1	353364	1-scf00007	1
AX-157893932	1	36.909	KV452707.1	420413	1-scf00007	1
AX-157893933	1	36.909	KV452707.1	420810	1-scf00007	1
AX-157893934	1	36.909	KV452707.1	425552	1-scf00007	1
AX-157774562	1	36.943	KV452707.1	418625	1-scf00007	1
AX-157718957	1	36.98	KV452707.1	416063	1-scf00007	1
AX-157810569	1	37.018	KV452707.1	244995	1-scf00007	1
AX-157893873	1	37.018	KV452707.1	349792	1-scf00007	1
AX-157893898	1	37.018	KV452707.1	378606	1-scf00007	1
AX-157727007	1	37.018	KV452707.1	401589	1-scf00007	1
AX-157893922	1	37.018	KV452707.1	409405	1-scf00007	1
AX-157893923	1	37.018	KV452707.1	410164	1-scf00007	1
AX-157893924	1	37.018	KV452707.1	410691	1-scf00007	1
AX-157893926	1	37.018	KV452707.1	412636	1-scf00007	1
AX-157893927	1	37.018	KV452707.1	412746	1-scf00007	1
AX-157725051	1	37.018	KV452707.1	415019	1-scf00007	1
AX-157893929	1	37.018	KV452707.1	415559	1-scf00007	1
AX-157738851	1	37.018	KV452707.1	470571	1-scf00007	1
AX-157893976	1	37.018	KV452707.1	493672	1-scf00007	1
AX-157894086	1	37.018	KV452708.1	110837	1-scf00008	1
AX-157892985	1	37.127	KV452698.1	1806342	1-scf00002	1
AX-157893714	1	37.127	KV452698.1	2130560	1-scf00002	1
AX-157893144	1	37.127	KV452700.1	182189	1-scf00005	1
AX-157893196	1	37.127	KV452700.1	248952	1-scf00005	1
AX-157893214	1	37.127	KV452700.1	277856	1-scf00005	1
AX-157893242	1	37.127	KV452700.1	328007	1-scf00005	1
AX-157893255	1	37.127	KV452700.1	337524	1-scf00005	1
AX-157893256	1	37.127	KV452700.1	338738	1-scf00005	1
AX-157893258	1	37.127	KV452700.1	340123	1-scf00005	1
AX-157893260	1	37.127	KV452700.1	343499	1-scf00005	1
AX-157893263	1	37.127	KV452700.1	351729	1-scf00005	1
AX-157746383	1	37.127	KV452700.1	352918	1-scf00005	1
AX-157893266	1	37.127	KV452700.1	356561	1-scf00005	1
AX-157893269	1	37.127	KV452700.1	380953	1-scf00005	1
AX-157893271	1	37.127	KV452700.1	382122	1-scf00005	1
AX-157893272	1	37.127	KV452700.1	382670	1-scf00005	1
AX-157810510	1	37.127	KV452700.1	383164	1-scf00005	1
AX-157893273	1	37.127	KV452700.1	383590	1-scf00005	1

AX-157893274	1	37.127	KV452700.1	384054	1-scf00005	1
AX-157745655	1	37.127	KV452700.1	384308	1-scf00005	1
AX-157893276	1	37.127	KV452700.1	385959	1-scf00005	1
AX-157893280	1	37.127	KV452700.1	393299	1-scf00005	1
AX-157893287	1	37.127	KV452700.1	401514	1-scf00005	1
AX-157893293	1	37.127	KV452700.1	413992	1-scf00005	1
AX-157893294	1	37.127	KV452700.1	416733	1-scf00005	1
AX-157893296	1	37.127	KV452700.1	417901	1-scf00005	1
AX-157893297	1	37.127	KV452700.1	421326	1-scf00005	1
AX-157893298	1	37.127	KV452700.1	422325	1-scf00005	1
AX-157893299	1	37.127	KV452700.1	422866	1-scf00005	1
AX-157893300	1	37.127	KV452700.1	423481	1-scf00005	1
AX-157893301	1	37.127	KV452700.1	424361	1-scf00005	1
AX-157893302	1	37.127	KV452700.1	425387	1-scf00005	1
AX-157893305	1	37.127	KV452700.1	428005	1-scf00005	1
AX-158404268	1	37.127	KV452700.1	446361	1-scf00005	1
AX-157893313	1	37.127	KV452700.1	447455	1-scf00005	1
AX-157893315	1	37.127	KV452700.1	451315	1-scf00005	1
AX-157893317	1	37.127	KV452700.1	452677	1-scf00005	1
AX-157893318	1	37.127	KV452700.1	452920	1-scf00005	1
AX-157893322	1	37.127	KV452700.1	460010	1-scf00005	1
AX-157893323	1	37.127	KV452700.1	460277	1-scf00005	1
AX-157893332	1	37.127	KV452700.1	473002	1-scf00005	1
AX-157893340	1	37.127	KV452700.1	479753	1-scf00005	1
AX-157893354	1	37.127	KV452700.1	492786	1-scf00005	1
AX-157893357	1	37.127	KV452700.1	495796	1-scf00005	1
AX-157893358	1	37.127	KV452700.1	497347	1-scf00005	1
AX-157893359	1	37.127	KV452700.1	497856	1-scf00005	1
AX-157893360	1	37.127	KV452700.1	499967	1-scf00005	1
AX-157810520	1	37.127	KV452700.1	539176	1-scf00005	1
AX-157893462	1	37.127	KV452700.1	650319	1-scf00005	1
AX-157893473	1	37.127	KV452700.1	671304	1-scf00005	1
AX-157893474	1	37.127	KV452700.1	674172	1-scf00005	1
AX-157893485	1	37.127	KV452700.1	696422	1-scf00005	1
AX-157893486	1	37.127	KV452700.1	698662	1-scf00005	1
AX-157893504	1	37.127	KV452700.1	728723	1-scf00005	1
AX-157810534	1	37.127	KV452700.1	808847	1-scf00005	1
AX-157893791	1	37.127	KV452707.1	203902	1-scf00007	1
AX-157893810	1	37.127	KV452707.1	236508	1-scf00007	1
AX-157771710	1	37.127	KV452707.1	330471	1-scf00007	1
AX-157893880	1	37.127	KV452707.1	359817	1-scf00007	1
AX-157893884	1	37.127	KV452707.1	366201	1-scf00007	1
AX-157893886	1	37.127	KV452707.1	369158	1-scf00007	1
AX-157893890	1	37.127	KV452707.1	373566	1-scf00007	1
AX-157893891	1	37.127	KV452707.1	374009	1-scf00007	1
AX-157893900	1	37.127	KV452707.1	380171	1-scf00007	1
AX-157893914	1	37.127	KV452707.1	397461	1-scf00007	1
AX-157893917	1	37.127	KV452707.1	403602	1-scf00007	1
AX-157862562	1	37.127	KV452699.1	97086	1-scf00610	1
AX-157893878	1	37.132	KV452707.1	355162	1-scf00007	1
AX-157893870	1	37.182	KV452707.1	343762	1-scf00007	1

AX-157893867	1	37.188	KV452707.1	340345	1-scf00007	1
AX-157892947	1	37.237	KV452698.1	1756394	1-scf00002	1
AX-157810482	1	37.237	KV452698.1	1823206	1-scf00002	1
AX-157892995	1	37.237	KV452698.1	1825122	1-scf00002	1
AX-157893605	1	37.237	KV452698.1	1914017	1-scf00002	1
AX-157744105	1	37.237	KV452700.1	790962	1-scf00005	1
AX-157893542	1	37.237	KV452700.1	805070	1-scf00005	1
AX-157893760	1	37.237	KV452707.1	159598	1-scf00007	1
AX-157810560	1	37.237	KV452707.1	162670	1-scf00007	1
AX-157893763	1	37.237	KV452707.1	163216	1-scf00007	1
AX-157893774	1	37.237	KV452707.1	185633	1-scf00007	1
AX-157893792	1	37.237	KV452707.1	204538	1-scf00007	1
AX-157893799	1	37.237	KV452707.1	215357	1-scf00007	1
AX-157893805	1	37.237	KV452707.1	229423	1-scf00007	1
AX-157893806	1	37.237	KV452707.1	231076	1-scf00007	1
AX-157893807	1	37.237	KV452707.1	231361	1-scf00007	1
AX-157776304	1	37.237	KV452707.1	238610	1-scf00007	1
AX-157810567	1	37.237	KV452707.1	240382	1-scf00007	1
AX-157893815	1	37.237	KV452707.1	242924	1-scf00007	1
AX-157747750	1	37.237	KV452707.1	249540	1-scf00007	1
AX-157810570	1	37.237	KV452707.1	250691	1-scf00007	1
AX-157810572	1	37.237	KV452707.1	260164	1-scf00007	1
AX-157893817	1	37.237	KV452707.1	260269	1-scf00007	1
AX-157893818	1	37.237	KV452707.1	266381	1-scf00007	1
AX-157810578	1	37.237	KV452707.1	268040	1-scf00007	1
AX-157893821	1	37.237	KV452707.1	274186	1-scf00007	1
AX-157893822	1	37.237	KV452707.1	275971	1-scf00007	1
AX-157893825	1	37.237	KV452707.1	281050	1-scf00007	1
AX-157893827	1	37.237	KV452707.1	286358	1-scf00007	1
AX-157893828	1	37.237	KV452707.1	287667	1-scf00007	1
AX-157725713	1	37.237	KV452707.1	290742	1-scf00007	1
AX-157893831	1	37.237	KV452707.1	295323	1-scf00007	1
AX-157741384	1	37.237	KV452707.1	295423	1-scf00007	1
AX-157893833	1	37.237	KV452707.1	296062	1-scf00007	1
AX-157752454	1	37.237	KV452707.1	299230	1-scf00007	1
AX-157741607	1	37.237	KV452707.1	299811	1-scf00007	1
AX-157893837	1	37.237	KV452707.1	300152	1-scf00007	1
AX-157893839	1	37.237	KV452707.1	301994	1-scf00007	1
AX-157893840	1	37.237	KV452707.1	306938	1-scf00007	1
AX-157893846	1	37.237	KV452707.1	313543	1-scf00007	1
AX-157893848	1	37.237	KV452707.1	314937	1-scf00007	1
AX-157893854	1	37.237	KV452707.1	323510	1-scf00007	1
AX-157893865	1	37.237	KV452707.1	338182	1-scf00007	1
AX-157893866	1	37.237	KV452707.1	339981	1-scf00007	1
AX-157893887	1	37.237	KV452707.1	369485	1-scf00007	1
AX-157893907	1	37.237	KV452707.1	387466	1-scf00007	1
AX-157893915	1	37.237	KV452707.1	397764	1-scf00007	1
AX-157853552	1	37.237	KV452707.1	401966	1-scf00007	1
AX-157893918	1	37.237	KV452707.1	404546	1-scf00007	1
AX-157893925	1	37.237	KV452707.1	412217	1-scf00007	1
AX-157756682	1	37.237	KV452707.1	423627	1-scf00007	1

AX-157864353	1	37.237	KV452707.1	442930	1-scf00007	1
AX-157894009	1	37.237	KV452707.1	542137	1-scf00007	1
AX-157894029	1	37.237	KV452708.1	989	1-scf00008	1
AX-157894036	1	37.237	KV452708.1	10547	1-scf00008	1
AX-157810568	1	37.294	KV452707.1	244214	1-scf00007	1
AX-157893836	1	37.294	KV452707.1	298614	1-scf00007	1
AX-157893968	1	37.294	KV452707.1	482528	1-scf00007	1
AX-157893788	1	37.346	KV452707.1	201479	1-scf00007	1
AX-157893796	1	37.346	KV452707.1	212505	1-scf00007	1
AX-157893798	1	37.346	KV452707.1	214924	1-scf00007	1
AX-157893800	1	37.346	KV452707.1	216855	1-scf00007	1
AX-157893801	1	37.346	KV452707.1	218447	1-scf00007	1
AX-157893808	1	37.346	KV452707.1	232293	1-scf00007	1
AX-157775265	1	37.346	KV452707.1	235158	1-scf00007	1
AX-157776303	1	37.346	KV452707.1	235219	1-scf00007	1
AX-157893814	1	37.346	KV452707.1	241381	1-scf00007	1
AX-157893844	1	37.346	KV452707.1	310835	1-scf00007	1
AX-157810590	1	37.346	KV452707.1	455398	1-scf00007	1
AX-157810561	1	37.369	KV452707.1	167630	1-scf00007	1
AX-157757447	1	37.369	KV452707.1	178258	1-scf00007	1
AX-157893794	1	37.369	KV452707.1	207976	1-scf00007	1
AX-157893881	1	37.369	KV452707.1	360394	1-scf00007	1
AX-157748161	1	37.369	KV452707.1	432578	1-scf00007	1
AX-157893793	1	37.39	KV452707.1	205129	1-scf00007	1
AX-157893786	1	37.408	KV452707.1	199868	1-scf00007	1
AX-157893785	1	37.423	KV452707.1	197822	1-scf00007	1
AX-157744036	1	37.423	KV452707.1	216553	1-scf00007	1
AX-157893874	1	37.423	KV452707.1	350291	1-scf00007	1
AX-157893780	1	37.438	KV452707.1	193652	1-scf00007	1
AX-157893830	1	37.438	KV452707.1	293498	1-scf00007	1
AX-157893518	1	37.456	KV452700.1	751605	1-scf00005	1
AX-157893566	1	37.456	KV452700.1	837264	1-scf00005	1
AX-157893766	1	37.456	KV452707.1	168244	1-scf00007	1
AX-157893768	1	37.456	KV452707.1	175444	1-scf00007	1
AX-157893769	1	37.456	KV452707.1	177322	1-scf00007	1
AX-157893772	1	37.456	KV452707.1	182260	1-scf00007	1
AX-157893773	1	37.456	KV452707.1	182709	1-scf00007	1
AX-157893775	1	37.456	KV452707.1	186865	1-scf00007	1
AX-157752337	1	37.456	KV452707.1	188015	1-scf00007	1
AX-157810564	1	37.456	KV452707.1	191193	1-scf00007	1
AX-157893779	1	37.456	KV452707.1	192305	1-scf00007	1
AX-157893832	1	37.456	KV452707.1	295702	1-scf00007	1
AX-157893852	1	37.456	KV452707.1	321126	1-scf00007	1
AX-157893871	1	37.456	KV452707.1	346461	1-scf00007	1
AX-157752044	1	37.457	KV452707.1	113893	1-scf00007	1
AX-157894027	1	37.726	KV452708.1	494	1-scf00008	1
AX-157893973	1	38.435	KV452707.1	488541	1-scf00007	1
AX-157893988	1	38.435	KV452707.1	507919	1-scf00007	1
AX-157883072	1	38.435	KV452708.1	15913	1-scf00008	1
AX-157810620	1	38.435	KV452708.1	19896	1-scf00008	1
AX-157894045	1	38.51	KV452708.1	26218	1-scf00008	1

AX-157894046	1	38.521	KV452708.1	26871	1-scf00008	1
AX-157894048	1	38.528	KV452708.1	28355	1-scf00008	1
AX-157894052	1	38.528	KV452708.1	32491	1-scf00008	1
AX-157894069	1	38.539	KV452708.1	67731	1-scf00008	1
AX-157772393	1	38.552	KV452708.1	70769	1-scf00008	1
AX-157894070	1	38.552	KV452708.1	71456	1-scf00008	1
AX-157894076	1	38.845	KV452708.1	83750	1-scf00008	1
AX-157894078	1	38.845	KV452708.1	85214	1-scf00008	1
AX-157894082	1	38.845	KV452708.1	104684	1-scf00008	1
AX-157810611	1	38.846	KV452707.1	562099	1-scf00007	1
AX-157894079	1	38.846	KV452708.1	87124	1-scf00008	1
AX-157810633	1	38.846	KV452708.1	136765	1-scf00008	1
AX-157894075	1	38.989	KV452708.1	83318	1-scf00008	1
AX-157773945	1	38.989	KV452708.1	116047	1-scf00008	1
AX-157894088	1	38.989	KV452708.1	119322	1-scf00008	1
AX-157894089	1	38.989	KV452708.1	119966	1-scf00008	1
AX-157894090	1	38.989	KV452708.1	120982	1-scf00008	1
AX-157894091	1	38.989	KV452708.1	122104	1-scf00008	1
AX-157894092	1	38.989	KV452708.1	127122	1-scf00008	1
AX-157748539	1	38.989	KV452708.1	128397	1-scf00008	1
AX-157894107	1	38.989	KV452708.1	159326	1-scf00008	1
AX-157894119	1	38.989	KV452708.1	180650	1-scf00008	1
AX-157894084	1	39.13	KV452708.1	109060	1-scf00008	1
AX-157810631	1	39.13	KV452708.1	130630	1-scf00008	1
AX-157894097	1	39.281	KV452708.1	140667	1-scf00008	1
AX-157810635	1	39.423	KV452708.1	141369	1-scf00008	1
AX-157894098	1	39.424	KV452708.1	146509	1-scf00008	1
AX-157810639	1	39.425	KV452708.1	147582	1-scf00008	1
AX-157810637	1	39.426	KV452708.1	145297	1-scf00008	1
AX-157894100	1	39.426	KV452708.1	150426	1-scf00008	1
AX-157894102	1	39.426	KV452708.1	152471	1-scf00008	1
AX-157894105	1	39.427	KV452708.1	155213	1-scf00008	1
AX-157894106	1	39.427	KV452708.1	157399	1-scf00008	1
AX-157894108	1	39.427	KV452708.1	159689	1-scf00008	1
AX-157893533	1	39.674	KV452700.1	777401	1-scf00005	1
AX-157894028	1	39.674	KV452708.1	867	1-scf00008	1
AX-157894116	1	39.674	KV452708.1	175524	1-scf00008	1
AX-157894120	1	39.918	KV452708.1	180813	1-scf00008	1
AX-157894124	1	40.69	KV452708.1	193416	1-scf00008	1
AX-157810641	1	41.07	KV452708.1	196197	1-scf00008	1
AX-157810643	1	41.071	KV452708.1	197572	1-scf00008	1
AX-157748602	1	41.071	KV452708.1	199154	1-scf00008	1
AX-157810644	1	41.071	KV452708.1	199448	1-scf00008	1
AX-157743025	1	41.071	KV452708.1	199830	1-scf00008	1
AX-157714203	1	41.397	KV452708.1	202762	1-scf00008	1
AX-157742218	1	41.398	KV452708.1	206901	1-scf00008	1
AX-157894127	1	41.398	KV452708.1	206975	1-scf00008	1
AX-157894128	1	41.422	KV452708.1	208475	1-scf00008	1
AX-157894133	1	41.422	KV452708.1	221951	1-scf00008	1
AX-157717355	1	41.451	KV452708.1	212828	1-scf00008	1
AX-157810648	1	41.476	KV452708.1	218721	1-scf00008	1

AX-157894135	1	41.476	KV452708.1	225485	1-scf00008	1
AX-157894132	1	41.507	KV452708.1	219595	1-scf00008	1
AX-157744060	1	41.507	KV452708.1	222753	1-scf00008	1
AX-157740986	1	41.644	KV452708.1	196429	1-scf00008	1
AX-157894134	1	41.644	KV452708.1	224909	1-scf00008	1
AX-157810630	1	41.671	KV452708.1	94648	1-scf00008	1
AX-157894081	1	41.671	KV452708.1	100864	1-scf00008	1
AX-157894136	1	41.671	KV452708.1	233008	1-scf00008	1
AX-157894192	1	41.671	KV452708.1	329783	1-scf00008	1
AX-157810650	1	41.726	KV452708.1	237092	1-scf00008	1
AX-157894143	1	41.726	KV452708.1	243137	1-scf00008	1
AX-157746042	1	41.726	KV452708.1	244690	1-scf00008	1
AX-157894148	1	41.726	KV452708.1	249062	1-scf00008	1
AX-157810651	1	41.729	KV452708.1	244529	1-scf00008	1
AX-157894147	1	41.729	KV452708.1	247737	1-scf00008	1
AX-157894151	1	42.017	KV452708.1	253738	1-scf00008	1
AX-157894155	1	42.017	KV452708.1	268950	1-scf00008	1
AX-157810653	1	42.163	KV452708.1	275378	1-scf00008	1
AX-157810654	1	42.163	KV452708.1	278198	1-scf00008	1
AX-157894158	1	42.163	KV452708.1	282750	1-scf00008	1
AX-157894159	1	42.163	KV452708.1	284215	1-scf00008	1
AX-157894161	1	42.163	KV452708.1	284940	1-scf00008	1
AX-157745024	1	42.163	KV452708.1	288196	1-scf00008	1
AX-157894168	1	42.163	KV452708.1	296808	1-scf00008	1
AX-157894180	1	42.163	KV452708.1	313509	1-scf00008	1
AX-157894169	1	42.49	KV452708.1	301068	1-scf00008	1
AX-157894176	1	42.49	KV452708.1	306711	1-scf00008	1
AX-157752861	1	42.491	KV452708.1	69303	1-scf00008	1
AX-157772201	1	42.491	KV452708.1	307292	1-scf00008	1
AX-157894178	1	42.491	KV452708.1	309359	1-scf00008	1
AX-157894179	1	42.491	KV452708.1	311576	1-scf00008	1
AX-157810660	1	42.493	KV452708.1	314673	1-scf00008	1
AX-157894184	1	42.495	KV452708.1	317659	1-scf00008	1
AX-157894186	1	42.9	KV452708.1	321372	1-scf00008	1
AX-157894187	1	42.9	KV452708.1	321553	1-scf00008	1
AX-157894188	1	42.9	KV452708.1	321955	1-scf00008	1
AX-157864361	1	42.9	KV452708.1	334303	1-scf00008	1
AX-157810663	1	42.9	KV452708.1	359679	1-scf00008	1
AX-157894190	1	42.901	KV452708.1	328449	1-scf00008	1
AX-157894195	1	42.902	KV452708.1	333253	1-scf00008	1
AX-157894201	1	42.903	KV452708.1	339183	1-scf00008	1
AX-157894212	1	43.355	KV452708.1	359636	1-scf00008	1
AX-157894217	1	43.584	KV452708.1	371930	1-scf00008	1
AX-157894218	1	43.584	KV452708.1	372326	1-scf00008	1
AX-157894221	1	43.584	KV452708.1	376124	1-scf00008	1
AX-157894223	1	43.584	KV452708.1	379965	1-scf00008	1
AX-157894234	1	43.584	KV452708.1	395091	1-scf00008	1
AX-157894233	1	43.623	KV452708.1	393249	1-scf00008	1
AX-157853554	1	43.694	KV452708.1	220820	1-scf00008	1
AX-158404287	1	43.694	KV452708.1	229082	1-scf00008	1
AX-157894171	1	43.694	KV452708.1	303136	1-scf00008	1

AX-157894229	1	43.694	KV452708.1	388964	1-scf00008	1
AX-157894235	1	43.694	KV452708.1	396529	1-scf00008	1
AX-157894238	1	43.694	KV452708.1	400455	1-scf00008	1
AX-157894239	1	43.694	KV452708.1	400942	1-scf00008	1
AX-157894241	1	43.694	KV452708.1	401610	1-scf00008	1
AX-157894240	1	43.803	KV452708.1	401146	1-scf00008	1
AX-157810667	1	43.803	KV452708.1	404216	1-scf00008	1
AX-157894242	1	43.803	KV452708.1	405494	1-scf00008	1
AX-157810668	1	43.803	KV452708.1	410617	1-scf00008	1
AX-157894200	1	44.021	KV452708.1	337513	1-scf00008	1
AX-157894252	1	44.021	KV452708.1	418976	1-scf00008	1
AX-157894257	1	44.021	KV452708.1	425466	1-scf00008	1
AX-157894258	1	44.021	KV452708.1	426435	1-scf00008	1
AX-157894260	1	44.021	KV452708.1	428036	1-scf00008	1
AX-157894261	1	44.021	KV452708.1	428862	1-scf00008	1
AX-157742409	1	44.021	KV452708.1	432101	1-scf00008	1
AX-157810670	1	44.021	KV452708.1	433330	1-scf00008	1
AX-157810671	1	44.021	KV452708.1	433882	1-scf00008	1
AX-157719298	1	44.021	KV452708.1	434853	1-scf00008	1
AX-157894262	1	44.021	KV452708.1	436574	1-scf00008	1
AX-157894263	1	44.021	KV452708.1	438485	1-scf00008	1
AX-157894264	1	44.021	KV452708.1	440698	1-scf00008	1
AX-157810672	1	44.021	KV452708.1	443687	1-scf00008	1
AX-157894268	1	44.313	KV452708.1	446261	1-scf00008	1
AX-157745593	1	44.313	KV452708.1	447730	1-scf00008	1
AX-157894269	1	44.313	KV452708.1	447859	1-scf00008	1
AX-157894277	1	44.313	KV452708.1	468407	1-scf00008	1
AX-157810646	1	44.458	KV452708.1	205235	1-scf00008	1
AX-157810665	1	44.458	KV452708.1	365505	1-scf00008	1
AX-157714780	1	44.458	KV452708.1	457473	1-scf00008	1
AX-157894273	1	44.458	KV452708.1	460570	1-scf00008	1
AX-158381941	1	44.458	KV452708.1	464469	1-scf00008	1
AX-157894275	1	44.458	KV452708.1	465406	1-scf00008	1
AX-157738363	1	44.458	KV452708.1	470542	1-scf00008	1
AX-157862589	1	44.458	KV452708.1	472678	1-scf00008	1
AX-157894280	1	44.458	KV452708.1	475561	1-scf00008	1
AX-157894281	1	44.458	KV452708.1	476433	1-scf00008	1
AX-157894282	1	44.458	KV452708.1	476832	1-scf00008	1
AX-157810677	1	44.458	KV452708.1	497378	1-scf00008	1
AX-157894288	1	44.459	KV452708.1	481493	1-scf00008	1
AX-157894253	1	44.46	KV452708.1	420722	1-scf00008	1
AX-157744986	1	44.46	KV452708.1	482111	1-scf00008	1
AX-157894289	1	44.786	KV452708.1	486776	1-scf00008	1
AX-157894290	1	44.786	KV452708.1	487348	1-scf00008	1
AX-157810675	1	44.786	KV452708.1	490987	1-scf00008	1
AX-157810676	1	44.786	KV452708.1	493506	1-scf00008	1
AX-157894292	1	44.786	KV452708.1	494378	1-scf00008	1
AX-157894293	1	44.786	KV452708.1	495076	1-scf00008	1
AX-157894139	1	44.847	KV452708.1	236464	1-scf00008	1
AX-157864360	1	44.847	KV452708.1	283646	1-scf00008	1
AX-157864362	1	44.847	KV452708.1	343927	1-scf00008	1

AX-157894230	1	44.847	KV452708.1	389839	1-scf00008	1
AX-157894295	1	44.847	KV452708.1	498616	1-scf00008	1
AX-157894294	1	44.919	KV452708.1	497758	1-scf00008	1
AX-157894296	1	44.919	KV452708.1	498999	1-scf00008	1
AX-157874291	1	45.004	KV452708.1	502660	1-scf00008	1
AX-157894297	1	45.004	KV452708.1	505314	1-scf00008	1
AX-157746873	1	45.004	KV452708.1	505577	1-scf00008	1
AX-157894298	1	45.004	KV452708.1	505910	1-scf00008	1
AX-157894299	1	45.004	KV452708.1	508319	1-scf00008	1
AX-157894300	1	45.004	KV452708.1	509160	1-scf00008	1
AX-157894301	1	45.004	KV452708.1	509855	1-scf00008	1
AX-157894305	1	45.004	KV452708.1	514825	1-scf00008	1
AX-157717580	1	45.004	KV452708.1	515335	1-scf00008	1
AX-158404300	1	45.004	KV452708.1	519304	1-scf00008	1
AX-157894310	1	45.004	KV452708.1	526586	1-scf00008	1
AX-157713083	1	45.004	KV452708.1	570843	1-scf00008	1
AX-157894311	1	45.223	KV452708.1	529520	1-scf00008	1
AX-157894314	1	45.223	KV452708.1	535421	1-scf00008	1
AX-157883075	1	45.223	KV452708.1	535666	1-scf00008	1
AX-157753568	1	45.223	KV452708.1	539419	1-scf00008	1
AX-157722002	1	45.223	KV452708.1	540582	1-scf00008	1
AX-157894322	1	45.223	KV452708.1	549368	1-scf00008	1
AX-157894323	1	45.223	KV452708.1	549890	1-scf00008	1
AX-157894325	1	45.223	KV452708.1	551933	1-scf00008	1
AX-157894319	1	45.504	KV452708.1	543245	1-scf00008	1
AX-157894332	1	45.504	KV452708.1	564129	1-scf00008	1
AX-158381942	1	45.504	KV452708.1	570385	1-scf00008	1
AX-157894334	1	45.504	KV452708.1	572972	1-scf00008	1
AX-157894335	1	45.504	KV452708.1	575069	1-scf00008	1
AX-157894336	1	45.504	KV452708.1	576502	1-scf00008	1
AX-157810684	1	45.504	KV452708.1	577621	1-scf00008	1
AX-157894338	1	45.793	KV452708.1	583056	1-scf00008	1
AX-157894328	1	45.918	KV452708.1	555936	1-scf00008	1
AX-157894340	1	45.918	KV452708.1	588621	1-scf00008	1
AX-157894342	1	45.925	KV452708.1	590838	1-scf00008	1
AX-157894343	1	45.938	KV452708.1	591321	1-scf00008	1
AX-157894345	1	46.206	KV452708.1	593665	1-scf00008	1
AX-157894346	1	46.206	KV452708.1	595106	1-scf00008	1
AX-158404301	1	46.207	KV452708.1	595373	1-scf00008	1
AX-157714029	1	46.316	KV452708.1	596644	1-scf00008	1
AX-157894347	1	46.316	KV452708.1	600248	1-scf00008	1
AX-157894348	1	46.425	KV452708.1	603364	1-scf00008	1
AX-157747147	1	46.486	KV452708.1	604504	1-scf00008	1
AX-157894349	1	46.534	KV452708.1	605999	1-scf00008	1
AX-157810685	1	46.534	KV452708.1	606254	1-scf00008	1
AX-157894351	1	46.535	KV452708.1	607899	1-scf00008	1
AX-157894352	1	46.535	KV452708.1	608337	1-scf00008	1
AX-157717501	1	46.697	KV452708.1	611507	1-scf00008	1
AX-157894353	1	46.761	KV452708.1	610393	1-scf00008	1
AX-157894355	1	46.761	KV452708.1	615659	1-scf00008	1
AX-157894388	1	46.761	KV452708.1	660282	1-scf00008	1

AX-157894357	1	46.97	KV452708.1	620221	1-scf00008	1
AX-157894356	1	47.194	KV452708.1	619413	1-scf00008	1
AX-157894359	1	47.194	KV452708.1	622464	1-scf00008	1
AX-157894360	1	47.194	KV452708.1	623017	1-scf00008	1
AX-157894372	1	47.194	KV452708.1	641442	1-scf00008	1
AX-158404304	1	47.293	KV452708.1	629620	1-scf00008	1
AX-157894364	1	47.52	KV452708.1	630224	1-scf00008	1
AX-157894365	1	47.52	KV452708.1	631416	1-scf00008	1
AX-157810687	1	47.52	KV452708.1	631740	1-scf00008	1
AX-157739123	1	47.52	KV452708.1	638414	1-scf00008	1
AX-157894375	1	47.52	KV452708.1	642753	1-scf00008	1
AX-157894376	1	47.52	KV452708.1	643909	1-scf00008	1
AX-157894377	1	47.52	KV452708.1	644682	1-scf00008	1
AX-157894379	1	47.52	KV452708.1	646198	1-scf00008	1
AX-157894380	1	47.52	KV452708.1	648208	1-scf00008	1
AX-157717518	1	47.52	KV452708.1	650151	1-scf00008	1
AX-157894382	1	47.52	KV452708.1	653355	1-scf00008	1
AX-157894383	1	47.52	KV452708.1	654018	1-scf00008	1
AX-157894384	1	47.52	KV452708.1	655154	1-scf00008	1
AX-157894403	1	47.52	KV452708.1	681921	1-scf00008	1
AX-157894350	1	47.629	KV452708.1	607032	1-scf00008	1
AX-158421772	1	47.629	KV452708.1	632231	1-scf00008	1
AX-158379119	1	47.629	KV452708.1	657290	1-scf00008	1
AX-157718264	1	47.629	KV452708.1	657470	1-scf00008	1
AX-157894385	1	47.629	KV452708.1	657825	1-scf00008	1
AX-157894391	1	47.629	KV452708.1	662409	1-scf00008	1
AX-157894378	1	47.738	KV452708.1	645277	1-scf00008	1
AX-158404309	1	47.738	KV452708.1	667258	1-scf00008	1
AX-157894394	1	47.738	KV452708.1	668044	1-scf00008	1
AX-157894395	1	47.738	KV452708.1	669186	1-scf00008	1
AX-157894396	1	47.738	KV452708.1	670863	1-scf00008	1
AX-157810688	1	47.738	KV452708.1	671833	1-scf00008	1
AX-157894398	1	47.738	KV452708.1	672637	1-scf00008	1
AX-157862590	1	47.738	KV452708.1	676508	1-scf00008	1
AX-157894401	1	47.738	KV452708.1	676610	1-scf00008	1
AX-157776381	1	47.849	KV452708.1	682460	1-scf00008	1
AX-157748592	1	47.911	KV452708.1	675189	1-scf00008	1
AX-157810690	1	47.911	KV452708.1	684587	1-scf00008	1
AX-157894407	1	47.911	KV452708.1	686113	1-scf00008	1
AX-157810691	1	47.985	KV452708.1	689537	1-scf00008	1
AX-157894409	1	47.991	KV452708.1	689867	1-scf00008	1
AX-157716394	1	48.037	KV452708.1	692939	1-scf00008	1
AX-157810693	1	48.037	KV452708.1	693129	1-scf00008	1
AX-157743585	1	48.083	KV452708.1	699588	1-scf00008	1
AX-158403798	1	48.083	KV452708.1	701303	1-scf00008	1
AX-157894413	1	48.344	KV452708.1	702568	1-scf00008	1
AX-157894414	1	48.344	KV452708.1	703721	1-scf00008	1
AX-157894417	1	48.344	KV452708.1	709286	1-scf00008	1
AX-157894422	1	48.815	KV452708.1	715474	1-scf00008	1
AX-157810694	1	49.051	KV452708.1	724170	1-scf00008	1
AX-157894430	1	49.051	KV452708.1	729433	1-scf00008	1

AX-157894432	1	49.051	KV452708.1	732054	1-scf00008	1
AX-157894438	1	49.377	KV452708.1	738832	1-scf00008	1
AX-157894439	1	49.377	KV452708.1	739927	1-scf00008	1
AX-157894429	1	49.378	KV452708.1	727301	1-scf00008	1
AX-157894440	1	49.378	KV452708.1	741056	1-scf00008	1
AX-157894442	1	49.378	KV452708.1	743424	1-scf00008	1
AX-157894443	1	49.378	KV452708.1	745233	1-scf00008	1
AX-157894444	1	49.378	KV452708.1	746105	1-scf00008	1
AX-157775920	1	49.488	KV452708.1	748368	1-scf00008	1
AX-157894445	1	49.488	KV452708.1	748864	1-scf00008	1
AX-157894446	1	49.488	KV452708.1	752159	1-scf00008	1
AX-157894448	1	49.488	KV452708.1	755736	1-scf00008	1
AX-157894450	1	49.488	KV452708.1	756996	1-scf00008	1
AX-157771278	1	49.544	KV452708.1	758860	1-scf00008	1
AX-157894434	1	49.597	KV452708.1	734183	1-scf00008	1
AX-157894452	1	49.597	KV452708.1	760323	1-scf00008	1
AX-157894455	1	49.597	KV452708.1	763683	1-scf00008	1
AX-157894456	1	49.597	KV452708.1	764879	1-scf00008	1
AX-157894457	1	49.597	KV452708.1	766468	1-scf00008	1
AX-157894463	1	49.597	KV452708.1	773896	1-scf00008	1
AX-157741669	1	49.597	KV452708.1	774652	1-scf00008	1
AX-157894465	1	49.597	KV452708.1	775443	1-scf00008	1
AX-157810697	1	49.908	KV452708.1	778854	1-scf00008	1
AX-157752983	1	49.908	KV452708.1	779936	1-scf00008	1
AX-157894467	1	49.908	KV452708.1	784634	1-scf00008	1
AX-157894454	1	49.912	KV452708.1	762585	1-scf00008	1
AX-157894466	1	49.912	KV452708.1	782062	1-scf00008	1
AX-157894468	1	50.011	KV452708.1	785515	1-scf00008	1
AX-157894469	1	50.122	KV452708.1	786489	1-scf00008	1
AX-157894471	1	50.334	KV452708.1	789499	1-scf00008	1
AX-157894472	1	50.336	KV452708.1	789917	1-scf00008	1
AX-157894474	1	50.471	KV452708.1	793335	1-scf00008	1
AX-157894475	1	50.471	KV452708.1	793764	1-scf00008	1
AX-157894480	1	50.471	KV452708.1	800074	1-scf00008	1
AX-157894481	1	50.471	KV452708.1	800988	1-scf00008	1
AX-157853557	1	50.581	KV452708.1	807073	1-scf00008	1
AX-157894486	1	50.581	KV452708.1	810340	1-scf00008	1
AX-157894488	1	50.778	KV452708.1	811815	1-scf00008	1
AX-157750675	1	50.966	KV452708.1	815076	1-scf00008	1
AX-157894492	1	51.173	KV452708.1	823498	1-scf00008	1
AX-157725168	1	51.173	KV452708.1	824455	1-scf00008	1
AX-157749973	1	51.254	KV452708.1	832138	1-scf00008	1
AX-158379121	1	51.261	KV452708.1	833385	1-scf00008	1
AX-157895193	1	51.261	KV452708.1	2081142	1-scf00008	1
AX-157894499	1	51.874	KV452708.1	838544	1-scf00008	1
AX-157894500	1	52.119	KV452708.1	841736	1-scf00008	1
AX-157894501	1	52.119	KV452708.1	843894	1-scf00008	1
AX-157894504	1	52.888	KV452708.1	848753	1-scf00008	1
AX-157894506	1	53.008	KV452708.1	850077	1-scf00008	1
AX-157894510	1	53.108	KV452708.1	859050	1-scf00008	1
AX-157894513	1	53.328	KV452708.1	864024	1-scf00008	1

AX-157810702	1	53.328	KV452708.1	867298	1-scf00008	1
AX-157894515	1	53.328	KV452708.1	870076	1-scf00008	1
AX-157894517	1	53.328	KV452708.1	871298	1-scf00008	1
AX-157894519	1	53.328	KV452708.1	872434	1-scf00008	1
AX-157894521	1	53.385	KV452708.1	875090	1-scf00008	1
AX-157894524	1	53.411	KV452708.1	877024	1-scf00008	1
AX-157894527	1	53.437	KV452708.1	884635	1-scf00008	1
AX-157894530	1	53.437	KV452708.1	894540	1-scf00008	1
AX-158379122	1	53.437	KV452708.1	896987	1-scf00008	1
AX-157894531	1	53.437	KV452708.1	897918	1-scf00008	1
AX-157894532	1	53.437	KV452708.1	900573	1-scf00008	1
AX-157810705	1	53.437	KV452708.1	905882	1-scf00008	1
AX-157894536	1	53.437	KV452708.1	906187	1-scf00008	1
AX-157810706	1	53.437	KV452708.1	913889	1-scf00008	1
AX-157810707	1	53.437	KV452708.1	916704	1-scf00008	1
AX-157894542	1	53.437	KV452708.1	918116	1-scf00008	1
AX-157894544	1	53.437	KV452708.1	922545	1-scf00008	1
AX-157725434	1	53.437	KV452708.1	927508	1-scf00008	1
AX-157894547	1	53.437	KV452708.1	927551	1-scf00008	1
AX-157894551	1	53.437	KV452708.1	930929	1-scf00008	1
AX-157894559	1	53.437	KV452708.1	937582	1-scf00008	1
AX-157894579	1	53.437	KV452708.1	985475	1-scf00008	1
AX-157897520	1	53.437	KV452709.1	2004589	1-scf00009	1
AX-157894555	1	53.479	KV452708.1	934679	1-scf00008	1
AX-157894557	1	53.546	KV452708.1	936434	1-scf00008	1
AX-157810709	1	53.546	KV452708.1	938436	1-scf00008	1
AX-157894562	1	53.546	KV452708.1	939501	1-scf00008	1
AX-157894563	1	53.546	KV452708.1	940267	1-scf00008	1
AX-157894565	1	53.546	KV452708.1	941637	1-scf00008	1
AX-157751963	1	53.655	KV452708.1	887105	1-scf00008	1
AX-157772437	1	53.655	KV452708.1	943958	1-scf00008	1
AX-157894568	1	53.655	KV452708.1	945138	1-scf00008	1
AX-157810711	1	53.655	KV452708.1	945582	1-scf00008	1
AX-157894571	1	53.655	KV452708.1	951474	1-scf00008	1
AX-157894573	1	53.655	KV452708.1	953075	1-scf00008	1
AX-157894924	1	53.655	KV452708.1	1574548	1-scf00008	1
AX-157689966	1	53.655		256	scf16754	UN
AX-157894574	1	53.686	KV452708.1	954652	1-scf00008	1
AX-157771694	1	53.713	KV452708.1	974575	1-scf00008	1
AX-157894512	1	53.738	KV452708.1	862714	1-scf00008	1
AX-157894576	1	53.738	KV452708.1	974670	1-scf00008	1
AX-157895151	1	53.738	KV452708.1	1994256	1-scf00008	1
AX-157895504	1	53.738	KV452708.1	2610457	1-scf00008	1
AX-157895505	1	53.738	KV452708.1	2610770	1-scf00008	1
AX-157894560	1	53.765	KV452708.1	938008	1-scf00008	1
AX-157894578	1	53.765	KV452708.1	984360	1-scf00008	1
AX-157894583	1	53.765	KV452708.1	991602	1-scf00008	1
AX-157894584	1	53.765	KV452708.1	992169	1-scf00008	1
AX-157810719	1	53.765	KV452708.1	993263	1-scf00008	1
AX-157775334	1	53.765	KV452708.1	995365	1-scf00008	1
AX-157894589	1	53.765	KV452708.1	1001901	1-scf00008	1

AX-157894591	1	53.765	KV452708.1	1006470	1-scf00008	1
AX-157894594	1	53.765	KV452708.1	1008583	1-scf00008	1
AX-158404313	1	53.765	KV452708.1	1009591	1-scf00008	1
AX-157894596	1	53.765	KV452708.1	1010070	1-scf00008	1
AX-157742371	1	53.765	KV452708.1	1022012	1-scf00008	1
AX-157894607	1	53.765	KV452708.1	1028624	1-scf00008	1
AX-157894608	1	53.765	KV452708.1	1029073	1-scf00008	1
AX-157894609	1	53.765	KV452708.1	1033157	1-scf00008	1
AX-157894612	1	53.765	KV452708.1	1035523	1-scf00008	1
AX-157894620	1	53.765	KV452708.1	1045841	1-scf00008	1
AX-157894621	1	53.765	KV452708.1	1047058	1-scf00008	1
AX-157810731	1	53.765	KV452708.1	1268820	1-scf00008	1
AX-157894814	1	53.765	KV452708.1	1403258	1-scf00008	1
AX-157810750	1	53.765	KV452708.1	1423747	1-scf00008	1
AX-157894911	1	53.765	KV452708.1	1561799	1-scf00008	1
AX-157894912	1	53.765	KV452708.1	1562489	1-scf00008	1
AX-157894913	1	53.765	KV452708.1	1562697	1-scf00008	1
AX-157810761	1	53.765	KV452708.1	1562973	1-scf00008	1
AX-157894914	1	53.765	KV452708.1	1564462	1-scf00008	1
AX-157810763	1	53.765	KV452708.1	1564847	1-scf00008	1
AX-157894916	1	53.765	KV452708.1	1565860	1-scf00008	1
AX-157894917	1	53.765	KV452708.1	1566814	1-scf00008	1
AX-157894918	1	53.765	KV452708.1	1566988	1-scf00008	1
AX-157894921	1	53.765	KV452708.1	1569315	1-scf00008	1
AX-157894922	1	53.765	KV452708.1	1570476	1-scf00008	1
AX-157810764	1	53.765	KV452708.1	1571991	1-scf00008	1
AX-157718608	1	53.765	KV452708.1	1572194	1-scf00008	1
AX-157894926	1	53.765	KV452708.1	1578309	1-scf00008	1
AX-157810765	1	53.765	KV452708.1	1578786	1-scf00008	1
AX-157894927	1	53.765	KV452708.1	1579144	1-scf00008	1
AX-157894928	1	53.765	KV452708.1	1579880	1-scf00008	1
AX-157894929	1	53.765	KV452708.1	1580170	1-scf00008	1
AX-157894931	1	53.765	KV452708.1	1580728	1-scf00008	1
AX-157894933	1	53.765	KV452708.1	1582072	1-scf00008	1
AX-157894934	1	53.765	KV452708.1	1587951	1-scf00008	1
AX-157894936	1	53.765	KV452708.1	1593890	1-scf00008	1
AX-157810766	1	53.765	KV452708.1	1596817	1-scf00008	1
AX-157714162	1	53.765	KV452708.1	1609180	1-scf00008	1
AX-157894945	1	53.765	KV452708.1	1618708	1-scf00008	1
AX-157894949	1	53.765	KV452708.1	1633598	1-scf00008	1
AX-157894959	1	53.765	KV452708.1	1651667	1-scf00008	1
AX-157746963	1	53.765	KV452708.1	1962059	1-scf00008	1
AX-157895177	1	53.765	KV452708.1	2054360	1-scf00008	1
AX-157895198	1	53.765	KV452708.1	2087852	1-scf00008	1
AX-157895203	1	53.765	KV452708.1	2096721	1-scf00008	1
AX-157895255	1	53.765	KV452708.1	2180317	1-scf00008	1
AX-157895269	1	53.765	KV452708.1	2198223	1-scf00008	1
AX-157810826	1	53.765	KV452708.1	2469291	1-scf00008	1
AX-157895490	1	53.765	KV452708.1	2597740	1-scf00008	1
AX-157895675	1	53.765	KV452708.1	2833446	1-scf00008	1
AX-157895676	1	53.765	KV452708.1	2834729	1-scf00008	1

AX-157895699	1	53.765	KV452708.1	2867585	1-scf00008	1
AX-157895718	1	53.765	KV452708.1	2884276	1-scf00008	1
AX-157895721	1	53.765	KV452708.1	2887879	1-scf00008	1
AX-157895734	1	53.765	KV452708.1	2907320	1-scf00008	1
AX-157895735	1	53.765	KV452708.1	2908504	1-scf00008	1
AX-157895782	1	53.765	KV452708.1	2967949	1-scf00008	1
AX-157810868	1	53.765	KV452708.1	2979285	1-scf00008	1
AX-157699310	1	53.765	FI899188.1	81	HORI212 Ictalu	UN
AX-157894567	1	53.872	KV452708.1	943855	1-scf00008	1
AX-157894624	1	53.872	KV452708.1	1049190	1-scf00008	1
AX-157894628	1	53.872	KV452708.1	1052323	1-scf00008	1
AX-157810729	1	53.872	KV452708.1	1261657	1-scf00008	1
AX-157894836	1	53.872	KV452708.1	1449265	1-scf00008	1
AX-157895459	1	53.872	KV452708.1	2559045	1-scf00008	1
AX-157741064	1	53.872	KV452708.1	2567512	1-scf00008	1
AX-157895507	1	53.872	KV452708.1	2614775	1-scf00008	1
AX-157895726	1	53.872	KV452708.1	2895722	1-scf00008	1
AX-157895833	1	53.872	KV452708.1	3023061	1-scf00008	1
AX-157895919	1	53.872	KV452708.1	3108472	1-scf00008	1
AX-157896043	1	53.872	KV452708.1	3239251	1-scf00008	1
AX-157894502	1	53.983	KV452708.1	846724	1-scf00008	1
AX-157894526	1	53.983	KV452708.1	884197	1-scf00008	1
AX-157894537	1	53.983	KV452708.1	907588	1-scf00008	1
AX-157894550	1	53.983	KV452708.1	929996	1-scf00008	1
AX-157810720	1	53.983	KV452708.1	1011976	1-scf00008	1
AX-157894598	1	53.983	KV452708.1	1014120	1-scf00008	1
AX-157894600	1	53.983	KV452708.1	1016635	1-scf00008	1
AX-157894601	1	53.983	KV452708.1	1020479	1-scf00008	1
AX-157894606	1	53.983	KV452708.1	1027463	1-scf00008	1
AX-157894613	1	53.983	KV452708.1	1035884	1-scf00008	1
AX-157894614	1	53.983	KV452708.1	1036351	1-scf00008	1
AX-157894616	1	53.983	KV452708.1	1038805	1-scf00008	1
AX-157894619	1	53.983	KV452708.1	1044544	1-scf00008	1
AX-157894631	1	53.983	KV452708.1	1054980	1-scf00008	1
AX-157756364	1	53.983	KV452708.1	1058138	1-scf00008	1
AX-157894637	1	53.983	KV452708.1	1068064	1-scf00008	1
AX-157894639	1	53.983	KV452708.1	1074709	1-scf00008	1
AX-157894643	1	53.983	KV452708.1	1084079	1-scf00008	1
AX-157894649	1	53.983	KV452708.1	1095573	1-scf00008	1
AX-157894670	1	53.983	KV452708.1	1132676	1-scf00008	1
AX-158379123	1	53.983	KV452708.1	1133108	1-scf00008	1
AX-157894676	1	53.983	KV452708.1	1141831	1-scf00008	1
AX-157894680	1	53.983	KV452708.1	1144418	1-scf00008	1
AX-157894687	1	53.983	KV452708.1	1158181	1-scf00008	1
AX-157894689	1	53.983	KV452708.1	1161612	1-scf00008	1
AX-157894690	1	53.983	KV452708.1	1162122	1-scf00008	1
AX-157894692	1	53.983	KV452708.1	1172399	1-scf00008	1
AX-157894698	1	53.983	KV452708.1	1187529	1-scf00008	1
AX-157715407	1	53.983	KV452708.1	1190030	1-scf00008	1
AX-157810723	1	53.983	KV452708.1	1223777	1-scf00008	1
AX-157894707	1	53.983	KV452708.1	1228391	1-scf00008	1

AX-157894714	1	53.983	KV452708.1	1242089	1-scf00008	1
AX-157810724	1	53.983	KV452708.1	1244788	1-scf00008	1
AX-157894715	1	53.983	KV452708.1	1245094	1-scf00008	1
AX-157810725	1	53.983	KV452708.1	1247696	1-scf00008	1
AX-157894717	1	53.983	KV452708.1	1253456	1-scf00008	1
AX-157894718	1	53.983	KV452708.1	1254270	1-scf00008	1
AX-157715667	1	53.983	KV452708.1	1254999	1-scf00008	1
AX-157894720	1	53.983	KV452708.1	1256645	1-scf00008	1
AX-157894721	1	53.983	KV452708.1	1258647	1-scf00008	1
AX-157810733	1	53.983	KV452708.1	1270536	1-scf00008	1
AX-157894726	1	53.983	KV452708.1	1271514	1-scf00008	1
AX-157894727	1	53.983	KV452708.1	1272190	1-scf00008	1
AX-157726290	1	53.983	KV452708.1	1282382	1-scf00008	1
AX-157894738	1	53.983	KV452708.1	1285167	1-scf00008	1
AX-157810736	1	53.983	KV452708.1	1286786	1-scf00008	1
AX-157894748	1	53.983	KV452708.1	1290394	1-scf00008	1
AX-157894750	1	53.983	KV452708.1	1292180	1-scf00008	1
AX-157894759	1	53.983	KV452708.1	1307108	1-scf00008	1
AX-157810739	1	53.983	KV452708.1	1309916	1-scf00008	1
AX-157894763	1	53.983	KV452708.1	1315253	1-scf00008	1
AX-157894765	1	53.983	KV452708.1	1317833	1-scf00008	1
AX-157752284	1	53.983	KV452708.1	1319431	1-scf00008	1
AX-157747703	1	53.983	KV452708.1	1320248	1-scf00008	1
AX-157894767	1	53.983	KV452708.1	1322712	1-scf00008	1
AX-157894768	1	53.983	KV452708.1	1323691	1-scf00008	1
AX-157810742	1	53.983	KV452708.1	1325550	1-scf00008	1
AX-157894769	1	53.983	KV452708.1	1325627	1-scf00008	1
AX-157894770	1	53.983	KV452708.1	1329115	1-scf00008	1
AX-157894773	1	53.983	KV452708.1	1332351	1-scf00008	1
AX-157894783	1	53.983	KV452708.1	1347414	1-scf00008	1
AX-157883083	1	53.983	KV452708.1	1361306	1-scf00008	1
AX-157894793	1	53.983	KV452708.1	1361973	1-scf00008	1
AX-157894794	1	53.983	KV452708.1	1364784	1-scf00008	1
AX-157883084	1	53.983	KV452708.1	1366601	1-scf00008	1
AX-157894798	1	53.983	KV452708.1	1373580	1-scf00008	1
AX-157894800	1	53.983	KV452708.1	1375747	1-scf00008	1
AX-157894803	1	53.983	KV452708.1	1384742	1-scf00008	1
AX-157894808	1	53.983	KV452708.1	1396946	1-scf00008	1
AX-157894812	1	53.983	KV452708.1	1400356	1-scf00008	1
AX-157894818	1	53.983	KV452708.1	1406323	1-scf00008	1
AX-157810747	1	53.983	KV452708.1	1408844	1-scf00008	1
AX-157894822	1	53.983	KV452708.1	1408902	1-scf00008	1
AX-157894823	1	53.983	KV452708.1	1409274	1-scf00008	1
AX-157894824	1	53.983	KV452708.1	1410367	1-scf00008	1
AX-157894825	1	53.983	KV452708.1	1411420	1-scf00008	1
AX-157894827	1	53.983	KV452708.1	1419993	1-scf00008	1
AX-157894828	1	53.983	KV452708.1	1420736	1-scf00008	1
AX-157894829	1	53.983	KV452708.1	1421033	1-scf00008	1
AX-157810751	1	53.983	KV452708.1	1424910	1-scf00008	1
AX-157894830	1	53.983	KV452708.1	1425397	1-scf00008	1
AX-157810752	1	53.983	KV452708.1	1430379	1-scf00008	1

AX-157894833	1	53.983	KV452708.1	1439417	1-scf00008	1
AX-157894834	1	53.983	KV452708.1	1443124	1-scf00008	1
AX-157894837	1	53.983	KV452708.1	1451243	1-scf00008	1
AX-157894838	1	53.983	KV452708.1	1451599	1-scf00008	1
AX-157894839	1	53.983	KV452708.1	1452942	1-scf00008	1
AX-157894840	1	53.983	KV452708.1	1453389	1-scf00008	1
AX-157894841	1	53.983	KV452708.1	1453916	1-scf00008	1
AX-157874301	1	53.983	KV452708.1	1462267	1-scf00008	1
AX-157695425	1	53.983	KV452708.1	1462948	1-scf00008	1
AX-157894850	1	53.983	KV452708.1	1465625	1-scf00008	1
AX-157810754	1	53.983	KV452708.1	1467549	1-scf00008	1
AX-157894851	1	53.983	KV452708.1	1468322	1-scf00008	1
AX-157894852	1	53.983	KV452708.1	1468417	1-scf00008	1
AX-157894853	1	53.983	KV452708.1	1470127	1-scf00008	1
AX-157894854	1	53.983	KV452708.1	1470695	1-scf00008	1
AX-157810755	1	53.983	KV452708.1	1471104	1-scf00008	1
AX-157894855	1	53.983	KV452708.1	1472745	1-scf00008	1
AX-157894858	1	53.983	KV452708.1	1476200	1-scf00008	1
AX-157894859	1	53.983	KV452708.1	1477848	1-scf00008	1
AX-157745868	1	53.983	KV452708.1	1485634	1-scf00008	1
AX-157894862	1	53.983	KV452708.1	1488511	1-scf00008	1
AX-157894863	1	53.983	KV452708.1	1492844	1-scf00008	1
AX-157894864	1	53.983	KV452708.1	1494570	1-scf00008	1
AX-157894866	1	53.983	KV452708.1	1501486	1-scf00008	1
AX-157894869	1	53.983	KV452708.1	1504432	1-scf00008	1
AX-157894870	1	53.983	KV452708.1	1506906	1-scf00008	1
AX-157894871	1	53.983	KV452708.1	1507063	1-scf00008	1
AX-157894880	1	53.983	KV452708.1	1517961	1-scf00008	1
AX-157810756	1	53.983	KV452708.1	1524029	1-scf00008	1
AX-157894883	1	53.983	KV452708.1	1525285	1-scf00008	1
AX-157894884	1	53.983	KV452708.1	1525642	1-scf00008	1
AX-157810757	1	53.983	KV452708.1	1526267	1-scf00008	1
AX-157810758	1	53.983	KV452708.1	1526377	1-scf00008	1
AX-157894885	1	53.983	KV452708.1	1526583	1-scf00008	1
AX-157894886	1	53.983	KV452708.1	1528084	1-scf00008	1
AX-157894887	1	53.983	KV452708.1	1530283	1-scf00008	1
AX-157894890	1	53.983	KV452708.1	1533629	1-scf00008	1
AX-157894891	1	53.983	KV452708.1	1533850	1-scf00008	1
AX-157894893	1	53.983	KV452708.1	1535856	1-scf00008	1
AX-157894894	1	53.983	KV452708.1	1536414	1-scf00008	1
AX-157894895	1	53.983	KV452708.1	1536623	1-scf00008	1
AX-157894898	1	53.983	KV452708.1	1539050	1-scf00008	1
AX-157894899	1	53.983	KV452708.1	1546444	1-scf00008	1
AX-157894903	1	53.983	KV452708.1	1547917	1-scf00008	1
AX-157894904	1	53.983	KV452708.1	1548904	1-scf00008	1
AX-157894905	1	53.983	KV452708.1	1549564	1-scf00008	1
AX-157810760	1	53.983	KV452708.1	1562180	1-scf00008	1
AX-157734809	1	53.983	KV452708.1	1563760	1-scf00008	1
AX-157894915	1	53.983	KV452708.1	1565244	1-scf00008	1
AX-157894923	1	53.983	KV452708.1	1573274	1-scf00008	1
AX-157894925	1	53.983	KV452708.1	1575403	1-scf00008	1

AX-157894930	1	53.983	KV452708.1	1580519	1-scf00008	1
AX-158403820	1	53.983	KV452708.1	1601046	1-scf00008	1
AX-157894937	1	53.983	KV452708.1	1602039	1-scf00008	1
AX-157894941	1	53.983	KV452708.1	1608592	1-scf00008	1
AX-157810768	1	53.983	KV452708.1	1614049	1-scf00008	1
AX-157894943	1	53.983	KV452708.1	1614503	1-scf00008	1
AX-157717172	1	53.983	KV452708.1	1616528	1-scf00008	1
AX-157894944	1	53.983	KV452708.1	1617915	1-scf00008	1
AX-157894947	1	53.983	KV452708.1	1624717	1-scf00008	1
AX-157894951	1	53.983	KV452708.1	1641195	1-scf00008	1
AX-157894953	1	53.983	KV452708.1	1643388	1-scf00008	1
AX-157894958	1	53.983	KV452708.1	1650919	1-scf00008	1
AX-157894962	1	53.983	KV452708.1	1656374	1-scf00008	1
AX-157894963	1	53.983	KV452708.1	1658650	1-scf00008	1
AX-157894964	1	53.983	KV452708.1	1661871	1-scf00008	1
AX-157758854	1	53.983	KV452708.1	1662979	1-scf00008	1
AX-157775413	1	53.983	KV452708.1	1664285	1-scf00008	1
AX-157894965	1	53.983	KV452708.1	1670037	1-scf00008	1
AX-157894966	1	53.983	KV452708.1	1670943	1-scf00008	1
AX-157727857	1	53.983	KV452708.1	1671544	1-scf00008	1
AX-157694228	1	53.983	KV452708.1	1677690	1-scf00008	1
AX-157894973	1	53.983	KV452708.1	1679154	1-scf00008	1
AX-157894974	1	53.983	KV452708.1	1679916	1-scf00008	1
AX-157894975	1	53.983	KV452708.1	1679964	1-scf00008	1
AX-157894976	1	53.983	KV452708.1	1682582	1-scf00008	1
AX-157894977	1	53.983	KV452708.1	1683224	1-scf00008	1
AX-157894978	1	53.983	KV452708.1	1683739	1-scf00008	1
AX-157894979	1	53.983	KV452708.1	1684300	1-scf00008	1
AX-157894981	1	53.983	KV452708.1	1685739	1-scf00008	1
AX-157894982	1	53.983	KV452708.1	1686257	1-scf00008	1
AX-157894983	1	53.983	KV452708.1	1686713	1-scf00008	1
AX-157894984	1	53.983	KV452708.1	1687556	1-scf00008	1
AX-157894986	1	53.983	KV452708.1	1689420	1-scf00008	1
AX-157894987	1	53.983	KV452708.1	1690465	1-scf00008	1
AX-157894988	1	53.983	KV452708.1	1690869	1-scf00008	1
AX-157894989	1	53.983	KV452708.1	1691316	1-scf00008	1
AX-157894991	1	53.983	KV452708.1	1693702	1-scf00008	1
AX-157894993	1	53.983	KV452708.1	1696120	1-scf00008	1
AX-157894994	1	53.983	KV452708.1	1696524	1-scf00008	1
AX-157894995	1	53.983	KV452708.1	1698212	1-scf00008	1
AX-157894998	1	53.983	KV452708.1	1706087	1-scf00008	1
AX-157775504	1	53.983	KV452708.1	1706701	1-scf00008	1
AX-157894999	1	53.983	KV452708.1	1708996	1-scf00008	1
AX-157895000	1	53.983	KV452708.1	1710248	1-scf00008	1
AX-157895003	1	53.983	KV452708.1	1715484	1-scf00008	1
AX-157895004	1	53.983	KV452708.1	1715669	1-scf00008	1
AX-157895005	1	53.983	KV452708.1	1719086	1-scf00008	1
AX-157895006	1	53.983	KV452708.1	1719451	1-scf00008	1
AX-157895008	1	53.983	KV452708.1	1720526	1-scf00008	1
AX-157895009	1	53.983	KV452708.1	1721152	1-scf00008	1
AX-157895010	1	53.983	KV452708.1	1721569	1-scf00008	1

AX-157895011	1	53.983	KV452708.1	1724085	1-scf00008	1
AX-157895012	1	53.983	KV452708.1	1724803	1-scf00008	1
AX-157895013	1	53.983	KV452708.1	1728107	1-scf00008	1
AX-157895014	1	53.983	KV452708.1	1728274	1-scf00008	1
AX-157895015	1	53.983	KV452708.1	1730740	1-scf00008	1
AX-157765837	1	53.983	KV452708.1	1731806	1-scf00008	1
AX-157895017	1	53.983	KV452708.1	1732146	1-scf00008	1
AX-157810772	1	53.983	KV452708.1	1732558	1-scf00008	1
AX-157810773	1	53.983	KV452708.1	1733014	1-scf00008	1
AX-157895018	1	53.983	KV452708.1	1733186	1-scf00008	1
AX-157895019	1	53.983	KV452708.1	1733931	1-scf00008	1
AX-157895020	1	53.983	KV452708.1	1734559	1-scf00008	1
AX-157895022	1	53.983	KV452708.1	1738080	1-scf00008	1
AX-157895023	1	53.983	KV452708.1	1738777	1-scf00008	1
AX-157895024	1	53.983	KV452708.1	1739294	1-scf00008	1
AX-157895026	1	53.983	KV452708.1	1741308	1-scf00008	1
AX-157895027	1	53.983	KV452708.1	1741869	1-scf00008	1
AX-157895028	1	53.983	KV452708.1	1743014	1-scf00008	1
AX-157895029	1	53.983	KV452708.1	1744752	1-scf00008	1
AX-157895030	1	53.983	KV452708.1	1747188	1-scf00008	1
AX-157895031	1	53.983	KV452708.1	1747554	1-scf00008	1
AX-157895032	1	53.983	KV452708.1	1748075	1-scf00008	1
AX-157895033	1	53.983	KV452708.1	1749645	1-scf00008	1
AX-157895034	1	53.983	KV452708.1	1750029	1-scf00008	1
AX-157810774	1	53.983	KV452708.1	1751007	1-scf00008	1
AX-157895035	1	53.983	KV452708.1	1752331	1-scf00008	1
AX-157810775	1	53.983	KV452708.1	1752687	1-scf00008	1
AX-157895037	1	53.983	KV452708.1	1754298	1-scf00008	1
AX-157895038	1	53.983	KV452708.1	1758816	1-scf00008	1
AX-157895040	1	53.983	KV452708.1	1759449	1-scf00008	1
AX-157895041	1	53.983	KV452708.1	1760029	1-scf00008	1
AX-157895042	1	53.983	KV452708.1	1760567	1-scf00008	1
AX-157810776	1	53.983	KV452708.1	1763583	1-scf00008	1
AX-157895044	1	53.983	KV452708.1	1765235	1-scf00008	1
AX-157895045	1	53.983	KV452708.1	1766968	1-scf00008	1
AX-158404328	1	53.983	KV452708.1	1768963	1-scf00008	1
AX-158421778	1	53.983	KV452708.1	1769923	1-scf00008	1
AX-157895047	1	53.983	KV452708.1	1773236	1-scf00008	1
AX-157895048	1	53.983	KV452708.1	1773780	1-scf00008	1
AX-157895049	1	53.983	KV452708.1	1774215	1-scf00008	1
AX-157895050	1	53.983	KV452708.1	1774574	1-scf00008	1
AX-157895052	1	53.983	KV452708.1	1776157	1-scf00008	1
AX-157723020	1	53.983	KV452708.1	1776843	1-scf00008	1
AX-157895053	1	53.983	KV452708.1	1779054	1-scf00008	1
AX-157810777	1	53.983	KV452708.1	1779949	1-scf00008	1
AX-157895054	1	53.983	KV452708.1	1780638	1-scf00008	1
AX-157895055	1	53.983	KV452708.1	1781264	1-scf00008	1
AX-157895056	1	53.983	KV452708.1	1781591	1-scf00008	1
AX-157895057	1	53.983	KV452708.1	1781953	1-scf00008	1
AX-157895058	1	53.983	KV452708.1	1782912	1-scf00008	1
AX-157895059	1	53.983	KV452708.1	1783261	1-scf00008	1

AX-157895060	1	53.983	KV452708.1	1784033	1-scf00008	1
AX-157895061	1	53.983	KV452708.1	1785165	1-scf00008	1
AX-157895062	1	53.983	KV452708.1	1786077	1-scf00008	1
AX-157895063	1	53.983	KV452708.1	1786796	1-scf00008	1
AX-157895064	1	53.983	KV452708.1	1787151	1-scf00008	1
AX-157895065	1	53.983	KV452708.1	1788169	1-scf00008	1
AX-157895067	1	53.983	KV452708.1	1789769	1-scf00008	1
AX-157895068	1	53.983	KV452708.1	1790532	1-scf00008	1
AX-157895069	1	53.983	KV452708.1	1791266	1-scf00008	1
AX-157895070	1	53.983	KV452708.1	1791765	1-scf00008	1
AX-157810780	1	53.983	KV452708.1	1792120	1-scf00008	1
AX-157895071	1	53.983	KV452708.1	1793768	1-scf00008	1
AX-157895072	1	53.983	KV452708.1	1795136	1-scf00008	1
AX-157895075	1	53.983	KV452708.1	1800496	1-scf00008	1
AX-157895076	1	53.983	KV452708.1	1801303	1-scf00008	1
AX-157895077	1	53.983	KV452708.1	1801756	1-scf00008	1
AX-157895078	1	53.983	KV452708.1	1802234	1-scf00008	1
AX-157895079	1	53.983	KV452708.1	1805075	1-scf00008	1
AX-157895080	1	53.983	KV452708.1	1805419	1-scf00008	1
AX-157810781	1	53.983	KV452708.1	1805841	1-scf00008	1
AX-158414561	1	53.983	KV452708.1	1807300	1-scf00008	1
AX-157810782	1	53.983	KV452708.1	1809595	1-scf00008	1
AX-157895081	1	53.983	KV452708.1	1809764	1-scf00008	1
AX-157810783	1	53.983	KV452708.1	1810263	1-scf00008	1
AX-157810784	1	53.983	KV452708.1	1812647	1-scf00008	1
AX-157895082	1	53.983	KV452708.1	1813155	1-scf00008	1
AX-157895083	1	53.983	KV452708.1	1813610	1-scf00008	1
AX-157895084	1	53.983	KV452708.1	1813950	1-scf00008	1
AX-157895085	1	53.983	KV452708.1	1814419	1-scf00008	1
AX-157810785	1	53.983	KV452708.1	1815391	1-scf00008	1
AX-157895086	1	53.983	KV452708.1	1816002	1-scf00008	1
AX-157810786	1	53.983	KV452708.1	1820456	1-scf00008	1
AX-157895088	1	53.983	KV452708.1	1820717	1-scf00008	1
AX-157895089	1	53.983	KV452708.1	1823745	1-scf00008	1
AX-157895090	1	53.983	KV452708.1	1824025	1-scf00008	1
AX-157895091	1	53.983	KV452708.1	1824705	1-scf00008	1
AX-157895092	1	53.983	KV452708.1	1825284	1-scf00008	1
AX-157895094	1	53.983	KV452708.1	1826779	1-scf00008	1
AX-157895095	1	53.983	KV452708.1	1828069	1-scf00008	1
AX-157895096	1	53.983	KV452708.1	1830420	1-scf00008	1
AX-157895097	1	53.983	KV452708.1	1830873	1-scf00008	1
AX-157810787	1	53.983	KV452708.1	1831357	1-scf00008	1
AX-157895099	1	53.983	KV452708.1	1832419	1-scf00008	1
AX-157810789	1	53.983	KV452708.1	1832671	1-scf00008	1
AX-157750654	1	53.983	KV452708.1	1834940	1-scf00008	1
AX-157810791	1	53.983	KV452708.1	1835590	1-scf00008	1
AX-158414562	1	53.983	KV452708.1	1838866	1-scf00008	1
AX-157895100	1	53.983	KV452708.1	1839468	1-scf00008	1
AX-157895101	1	53.983	KV452708.1	1840808	1-scf00008	1
AX-157895102	1	53.983	KV452708.1	1843438	1-scf00008	1
AX-157895103	1	53.983	KV452708.1	1844555	1-scf00008	1

AX-157868493	1	53.983	KV452708.1	1846756	1-scf00008	1
AX-157810792	1	53.983	KV452708.1	1846972	1-scf00008	1
AX-157895105	1	53.983	KV452708.1	1848038	1-scf00008	1
AX-158379126	1	53.983	KV452708.1	1850194	1-scf00008	1
AX-157721005	1	53.983	KV452708.1	1850808	1-scf00008	1
AX-157895106	1	53.983	KV452708.1	1850855	1-scf00008	1
AX-157895107	1	53.983	KV452708.1	1854041	1-scf00008	1
AX-157772542	1	53.983	KV452708.1	1854739	1-scf00008	1
AX-157810794	1	53.983	KV452708.1	1857919	1-scf00008	1
AX-157895109	1	53.983	KV452708.1	1861491	1-scf00008	1
AX-157895110	1	53.983	KV452708.1	1862563	1-scf00008	1
AX-157895112	1	53.983	KV452708.1	1865507	1-scf00008	1
AX-157895115	1	53.983	KV452708.1	1867262	1-scf00008	1
AX-157895117	1	53.983	KV452708.1	1871151	1-scf00008	1
AX-157895118	1	53.983	KV452708.1	1871538	1-scf00008	1
AX-157895119	1	53.983	KV452708.1	1872661	1-scf00008	1
AX-157895120	1	53.983	KV452708.1	1873672	1-scf00008	1
AX-157895121	1	53.983	KV452708.1	1874202	1-scf00008	1
AX-157895122	1	53.983	KV452708.1	1875075	1-scf00008	1
AX-157895124	1	53.983	KV452708.1	1878027	1-scf00008	1
AX-157895128	1	53.983	KV452708.1	1889812	1-scf00008	1
AX-157895130	1	53.983	KV452708.1	1893634	1-scf00008	1
AX-157895133	1	53.983	KV452708.1	1903324	1-scf00008	1
AX-157895134	1	53.983	KV452708.1	1903846	1-scf00008	1
AX-157868494	1	53.983	KV452708.1	1909944	1-scf00008	1
AX-157810797	1	53.983	KV452708.1	1923471	1-scf00008	1
AX-157895140	1	53.983	KV452708.1	1927960	1-scf00008	1
AX-157868495	1	53.983	KV452708.1	1929736	1-scf00008	1
AX-158388991	1	53.983	KV452708.1	1938502	1-scf00008	1
AX-157895142	1	53.983	KV452708.1	1944866	1-scf00008	1
AX-157895143	1	53.983	KV452708.1	1947400	1-scf00008	1
AX-157895144	1	53.983	KV452708.1	1952433	1-scf00008	1
AX-157726494	1	53.983	KV452708.1	1954358	1-scf00008	1
AX-157756975	1	53.983	KV452708.1	1956343	1-scf00008	1
AX-157895146	1	53.983	KV452708.1	1960158	1-scf00008	1
AX-157696678	1	53.983	KV452708.1	1970926	1-scf00008	1
AX-157895149	1	53.983	KV452708.1	1988281	1-scf00008	1
AX-157853561	1	53.983	KV452708.1	1989648	1-scf00008	1
AX-157810801	1	53.983	KV452708.1	2011061	1-scf00008	1
AX-157895156	1	53.983	KV452708.1	2019101	1-scf00008	1
AX-157810803	1	53.983	KV452708.1	2020269	1-scf00008	1
AX-157895160	1	53.983	KV452708.1	2023625	1-scf00008	1
AX-157895161	1	53.983	KV452708.1	2024430	1-scf00008	1
AX-157868496	1	53.983	KV452708.1	2031915	1-scf00008	1
AX-157895165	1	53.983	KV452708.1	2032990	1-scf00008	1
AX-157895167	1	53.983	KV452708.1	2039632	1-scf00008	1
AX-157895168	1	53.983	KV452708.1	2040620	1-scf00008	1
AX-157895170	1	53.983	KV452708.1	2043358	1-scf00008	1
AX-157895171	1	53.983	KV452708.1	2043672	1-scf00008	1
AX-157810805	1	53.983	KV452708.1	2047076	1-scf00008	1
AX-157895172	1	53.983	KV452708.1	2048397	1-scf00008	1

AX-157895173	1	53.983	KV452708.1	2050116	1-scf00008	1
AX-157895174	1	53.983	KV452708.1	2050429	1-scf00008	1
AX-157895179	1	53.983	KV452708.1	2060150	1-scf00008	1
AX-157895180	1	53.983	KV452708.1	2060728	1-scf00008	1
AX-157895181	1	53.983	KV452708.1	2063485	1-scf00008	1
AX-157895184	1	53.983	KV452708.1	2070493	1-scf00008	1
AX-157895185	1	53.983	KV452708.1	2071178	1-scf00008	1
AX-157895186	1	53.983	KV452708.1	2072111	1-scf00008	1
AX-157895187	1	53.983	KV452708.1	2074688	1-scf00008	1
AX-157895188	1	53.983	KV452708.1	2075085	1-scf00008	1
AX-157895189	1	53.983	KV452708.1	2075605	1-scf00008	1
AX-157895190	1	53.983	KV452708.1	2077742	1-scf00008	1
AX-157895194	1	53.983	KV452708.1	2083708	1-scf00008	1
AX-157895195	1	53.983	KV452708.1	2084790	1-scf00008	1
AX-157895199	1	53.983	KV452708.1	2088836	1-scf00008	1
AX-157895205	1	53.983	KV452708.1	2101331	1-scf00008	1
AX-157895210	1	53.983	KV452708.1	2111593	1-scf00008	1
AX-157895212	1	53.983	KV452708.1	2114972	1-scf00008	1
AX-157895215	1	53.983	KV452708.1	2126390	1-scf00008	1
AX-157895216	1	53.983	KV452708.1	2126801	1-scf00008	1
AX-157895218	1	53.983	KV452708.1	2132030	1-scf00008	1
AX-157810807	1	53.983	KV452708.1	2134218	1-scf00008	1
AX-157895221	1	53.983	KV452708.1	2134983	1-scf00008	1
AX-157727397	1	53.983	KV452708.1	2135635	1-scf00008	1
AX-157895223	1	53.983	KV452708.1	2137346	1-scf00008	1
AX-157895224	1	53.983	KV452708.1	2137771	1-scf00008	1
AX-157895225	1	53.983	KV452708.1	2138325	1-scf00008	1
AX-157895227	1	53.983	KV452708.1	2142313	1-scf00008	1
AX-157895228	1	53.983	KV452708.1	2143507	1-scf00008	1
AX-157895229	1	53.983	KV452708.1	2149236	1-scf00008	1
AX-157895230	1	53.983	KV452708.1	2152987	1-scf00008	1
AX-157895231	1	53.983	KV452708.1	2153249	1-scf00008	1
AX-157895232	1	53.983	KV452708.1	2154357	1-scf00008	1
AX-157895235	1	53.983	KV452708.1	2157564	1-scf00008	1
AX-157895236	1	53.983	KV452708.1	2158015	1-scf00008	1
AX-157810808	1	53.983	KV452708.1	2162890	1-scf00008	1
AX-157895240	1	53.983	KV452708.1	2167539	1-scf00008	1
AX-157895242	1	53.983	KV452708.1	2168527	1-scf00008	1
AX-157895245	1	53.983	KV452708.1	2172372	1-scf00008	1
AX-157895249	1	53.983	KV452708.1	2175392	1-scf00008	1
AX-157895250	1	53.983	KV452708.1	2177713	1-scf00008	1
AX-157895251	1	53.983	KV452708.1	2178330	1-scf00008	1
AX-157895252	1	53.983	KV452708.1	2179088	1-scf00008	1
AX-157895253	1	53.983	KV452708.1	2179519	1-scf00008	1
AX-157895254	1	53.983	KV452708.1	2179932	1-scf00008	1
AX-157895257	1	53.983	KV452708.1	2181911	1-scf00008	1
AX-157895260	1	53.983	KV452708.1	2187482	1-scf00008	1
AX-157895261	1	53.983	KV452708.1	2187788	1-scf00008	1
AX-157895262	1	53.983	KV452708.1	2188182	1-scf00008	1
AX-157895265	1	53.983	KV452708.1	2189203	1-scf00008	1
AX-157895267	1	53.983	KV452708.1	2191774	1-scf00008	1

AX-157895270	1	53.983	KV452708.1	2198751	1-scf00008	1
AX-157895272	1	53.983	KV452708.1	2201787	1-scf00008	1
AX-157810809	1	53.983	KV452708.1	2202337	1-scf00008	1
AX-157895273	1	53.983	KV452708.1	2202885	1-scf00008	1
AX-157895275	1	53.983	KV452708.1	2204929	1-scf00008	1
AX-157895276	1	53.983	KV452708.1	2206099	1-scf00008	1
AX-157895277	1	53.983	KV452708.1	2207992	1-scf00008	1
AX-157895279	1	53.983	KV452708.1	2209091	1-scf00008	1
AX-157895282	1	53.983	KV452708.1	2211874	1-scf00008	1
AX-157895283	1	53.983	KV452708.1	2212549	1-scf00008	1
AX-157895284	1	53.983	KV452708.1	2213588	1-scf00008	1
AX-157895287	1	53.983	KV452708.1	2221476	1-scf00008	1
AX-157895289	1	53.983	KV452708.1	2222660	1-scf00008	1
AX-157895290	1	53.983	KV452708.1	2223530	1-scf00008	1
AX-157895291	1	53.983	KV452708.1	2224753	1-scf00008	1
AX-157895292	1	53.983	KV452708.1	2227880	1-scf00008	1
AX-157895293	1	53.983	KV452708.1	2229569	1-scf00008	1
AX-158388992	1	53.983	KV452708.1	2230319	1-scf00008	1
AX-157895294	1	53.983	KV452708.1	2230611	1-scf00008	1
AX-157895295	1	53.983	KV452708.1	2231448	1-scf00008	1
AX-157895297	1	53.983	KV452708.1	2234906	1-scf00008	1
AX-157895300	1	53.983	KV452708.1	2236814	1-scf00008	1
AX-157710872	1	53.983	KV452708.1	2240194	1-scf00008	1
AX-157895301	1	53.983	KV452708.1	2246015	1-scf00008	1
AX-157895302	1	53.983	KV452708.1	2248371	1-scf00008	1
AX-157895303	1	53.983	KV452708.1	2248841	1-scf00008	1
AX-157895304	1	53.983	KV452708.1	2250233	1-scf00008	1
AX-157895305	1	53.983	KV452708.1	2250581	1-scf00008	1
AX-157895307	1	53.983	KV452708.1	2253074	1-scf00008	1
AX-157895308	1	53.983	KV452708.1	2253499	1-scf00008	1
AX-157895309	1	53.983	KV452708.1	2253887	1-scf00008	1
AX-157895314	1	53.983	KV452708.1	2258943	1-scf00008	1
AX-157895318	1	53.983	KV452708.1	2264955	1-scf00008	1
AX-157895319	1	53.983	KV452708.1	2266160	1-scf00008	1
AX-157895323	1	53.983	KV452708.1	2270008	1-scf00008	1
AX-157895324	1	53.983	KV452708.1	2270842	1-scf00008	1
AX-157895327	1	53.983	KV452708.1	2273276	1-scf00008	1
AX-157895329	1	53.983	KV452708.1	2275804	1-scf00008	1
AX-157895330	1	53.983	KV452708.1	2276901	1-scf00008	1
AX-157895331	1	53.983	KV452708.1	2279185	1-scf00008	1
AX-157895332	1	53.983	KV452708.1	2280797	1-scf00008	1
AX-157895333	1	53.983	KV452708.1	2282113	1-scf00008	1
AX-157895334	1	53.983	KV452708.1	2282534	1-scf00008	1
AX-157895335	1	53.983	KV452708.1	2283160	1-scf00008	1
AX-157725659	1	53.983	KV452708.1	2283816	1-scf00008	1
AX-157895336	1	53.983	KV452708.1	2285050	1-scf00008	1
AX-157895337	1	53.983	KV452708.1	2287086	1-scf00008	1
AX-157895339	1	53.983	KV452708.1	2288805	1-scf00008	1
AX-157895342	1	53.983	KV452708.1	2293586	1-scf00008	1
AX-157895343	1	53.983	KV452708.1	2294341	1-scf00008	1
AX-157895344	1	53.983	KV452708.1	2294927	1-scf00008	1

AX-157895348	1	53.983	KV452708.1	2298491	1-scf00008	1
AX-157895352	1	53.983	KV452708.1	2303689	1-scf00008	1
AX-157810810	1	53.983	KV452708.1	2307700	1-scf00008	1
AX-157868497	1	53.983	KV452708.1	2309320	1-scf00008	1
AX-157810811	1	53.983	KV452708.1	2310572	1-scf00008	1
AX-157895357	1	53.983	KV452708.1	2311146	1-scf00008	1
AX-157895358	1	53.983	KV452708.1	2311356	1-scf00008	1
AX-157810812	1	53.983	KV452708.1	2315993	1-scf00008	1
AX-157895361	1	53.983	KV452708.1	2316430	1-scf00008	1
AX-157895362	1	53.983	KV452708.1	2321770	1-scf00008	1
AX-157895363	1	53.983	KV452708.1	2322778	1-scf00008	1
AX-157895364	1	53.983	KV452708.1	2323914	1-scf00008	1
AX-157895365	1	53.983	KV452708.1	2325143	1-scf00008	1
AX-157895366	1	53.983	KV452708.1	2327503	1-scf00008	1
AX-157895368	1	53.983	KV452708.1	2329440	1-scf00008	1
AX-157895370	1	53.983	KV452708.1	2331119	1-scf00008	1
AX-157895371	1	53.983	KV452708.1	2333874	1-scf00008	1
AX-157895372	1	53.983	KV452708.1	2335690	1-scf00008	1
AX-157895373	1	53.983	KV452708.1	2336282	1-scf00008	1
AX-157895374	1	53.983	KV452708.1	2343454	1-scf00008	1
AX-157895375	1	53.983	KV452708.1	2348247	1-scf00008	1
AX-157868498	1	53.983	KV452708.1	2353414	1-scf00008	1
AX-157868499	1	53.983	KV452708.1	2354288	1-scf00008	1
AX-157895378	1	53.983	KV452708.1	2356347	1-scf00008	1
AX-157810814	1	53.983	KV452708.1	2358686	1-scf00008	1
AX-157810816	1	53.983	KV452708.1	2361048	1-scf00008	1
AX-157895383	1	53.983	KV452708.1	2371206	1-scf00008	1
AX-157895384	1	53.983	KV452708.1	2372281	1-scf00008	1
AX-158404340	1	53.983	KV452708.1	2380233	1-scf00008	1
AX-157895387	1	53.983	KV452708.1	2381591	1-scf00008	1
AX-157895390	1	53.983	KV452708.1	2383827	1-scf00008	1
AX-157895391	1	53.983	KV452708.1	2384883	1-scf00008	1
AX-157895395	1	53.983	KV452708.1	2388155	1-scf00008	1
AX-157895396	1	53.983	KV452708.1	2391991	1-scf00008	1
AX-157810817	1	53.983	KV452708.1	2393376	1-scf00008	1
AX-157810818	1	53.983	KV452708.1	2393530	1-scf00008	1
AX-157895397	1	53.983	KV452708.1	2397881	1-scf00008	1
AX-157895398	1	53.983	KV452708.1	2399772	1-scf00008	1
AX-157895399	1	53.983	KV452708.1	2403432	1-scf00008	1
AX-157895400	1	53.983	KV452708.1	2405118	1-scf00008	1
AX-157895403	1	53.983	KV452708.1	2408799	1-scf00008	1
AX-157810819	1	53.983	KV452708.1	2410111	1-scf00008	1
AX-157810820	1	53.983	KV452708.1	2412135	1-scf00008	1
AX-157810821	1	53.983	KV452708.1	2412621	1-scf00008	1
AX-157810822	1	53.983	KV452708.1	2421468	1-scf00008	1
AX-157810824	1	53.983	KV452708.1	2426937	1-scf00008	1
AX-157895407	1	53.983	KV452708.1	2429084	1-scf00008	1
AX-157895408	1	53.983	KV452708.1	2429527	1-scf00008	1
AX-157895409	1	53.983	KV452708.1	2430554	1-scf00008	1
AX-157895410	1	53.983	KV452708.1	2432715	1-scf00008	1
AX-157720016	1	53.983	KV452708.1	2436391	1-scf00008	1

AX-157895413	1	53.983	KV452708.1	2439356	1-scf00008	1
AX-157895414	1	53.983	KV452708.1	2440547	1-scf00008	1
AX-157895415	1	53.983	KV452708.1	2445829	1-scf00008	1
AX-157895416	1	53.983	KV452708.1	2451182	1-scf00008	1
AX-157749471	1	53.983	KV452708.1	2451402	1-scf00008	1
AX-157895417	1	53.983	KV452708.1	2452641	1-scf00008	1
AX-157895418	1	53.983	KV452708.1	2452943	1-scf00008	1
AX-157895419	1	53.983	KV452708.1	2454775	1-scf00008	1
AX-157895420	1	53.983	KV452708.1	2456655	1-scf00008	1
AX-157810825	1	53.983	KV452708.1	2459061	1-scf00008	1
AX-157707783	1	53.983	KV452708.1	2460472	1-scf00008	1
AX-157707781	1	53.983	KV452708.1	2460510	1-scf00008	1
AX-157719650	1	53.983	KV452708.1	2466367	1-scf00008	1
AX-157895421	1	53.983	KV452708.1	2467172	1-scf00008	1
AX-157774578	1	53.983	KV452708.1	2467281	1-scf00008	1
AX-157810827	1	53.983	KV452708.1	2469567	1-scf00008	1
AX-157895424	1	53.983	KV452708.1	2479322	1-scf00008	1
AX-157895425	1	53.983	KV452708.1	2482767	1-scf00008	1
AX-157895427	1	53.983	KV452708.1	2489294	1-scf00008	1
AX-157895428	1	53.983	KV452708.1	2490183	1-scf00008	1
AX-157895429	1	53.983	KV452708.1	2494579	1-scf00008	1
AX-157715918	1	53.983	KV452708.1	2503533	1-scf00008	1
AX-157895431	1	53.983	KV452708.1	2508653	1-scf00008	1
AX-157895432	1	53.983	KV452708.1	2509341	1-scf00008	1
AX-157895433	1	53.983	KV452708.1	2509679	1-scf00008	1
AX-157895434	1	53.983	KV452708.1	2512214	1-scf00008	1
AX-157748837	1	53.983	KV452708.1	2512650	1-scf00008	1
AX-157895435	1	53.983	KV452708.1	2512748	1-scf00008	1
AX-157895436	1	53.983	KV452708.1	2514556	1-scf00008	1
AX-157895437	1	53.983	KV452708.1	2515814	1-scf00008	1
AX-157810831	1	53.983	KV452708.1	2521607	1-scf00008	1
AX-157748407	1	53.983	KV452708.1	2524775	1-scf00008	1
AX-158414567	1	53.983	KV452708.1	2534052	1-scf00008	1
AX-157895446	1	53.983	KV452708.1	2540960	1-scf00008	1
AX-157895447	1	53.983	KV452708.1	2541624	1-scf00008	1
AX-157895448	1	53.983	KV452708.1	2542287	1-scf00008	1
AX-157895449	1	53.983	KV452708.1	2543105	1-scf00008	1
AX-157720481	1	53.983	KV452708.1	2543865	1-scf00008	1
AX-157773240	1	53.983	KV452708.1	2544894	1-scf00008	1
AX-157895450	1	53.983	KV452708.1	2546122	1-scf00008	1
AX-157895453	1	53.983	KV452708.1	2550787	1-scf00008	1
AX-157895455	1	53.983	KV452708.1	2552868	1-scf00008	1
AX-157810833	1	53.983	KV452708.1	2553138	1-scf00008	1
AX-157895456	1	53.983	KV452708.1	2557494	1-scf00008	1
AX-157895457	1	53.983	KV452708.1	2557703	1-scf00008	1
AX-157895461	1	53.983	KV452708.1	2560254	1-scf00008	1
AX-157895462	1	53.983	KV452708.1	2560477	1-scf00008	1
AX-157895463	1	53.983	KV452708.1	2561347	1-scf00008	1
AX-157895464	1	53.983	KV452708.1	2563421	1-scf00008	1
AX-157895465	1	53.983	KV452708.1	2567160	1-scf00008	1
AX-157895466	1	53.983	KV452708.1	2567894	1-scf00008	1

AX-157810834	1	53.983	KV452708.1	2570225	1-scf00008	1
AX-157895467	1	53.983	KV452708.1	2570940	1-scf00008	1
AX-158381959	1	53.983	KV452708.1	2572369	1-scf00008	1
AX-157895473	1	53.983	KV452708.1	2576262	1-scf00008	1
AX-157895474	1	53.983	KV452708.1	2576719	1-scf00008	1
AX-157810835	1	53.983	KV452708.1	2577785	1-scf00008	1
AX-157895477	1	53.983	KV452708.1	2584630	1-scf00008	1
AX-157895479	1	53.983	KV452708.1	2586041	1-scf00008	1
AX-157895483	1	53.983	KV452708.1	2589406	1-scf00008	1
AX-157895484	1	53.983	KV452708.1	2590051	1-scf00008	1
AX-157895485	1	53.983	KV452708.1	2591443	1-scf00008	1
AX-157895486	1	53.983	KV452708.1	2593094	1-scf00008	1
AX-157895487	1	53.983	KV452708.1	2593713	1-scf00008	1
AX-157895488	1	53.983	KV452708.1	2597005	1-scf00008	1
AX-157895491	1	53.983	KV452708.1	2599149	1-scf00008	1
AX-157868501	1	53.983	KV452708.1	2600261	1-scf00008	1
AX-157895494	1	53.983	KV452708.1	2601334	1-scf00008	1
AX-157895497	1	53.983	KV452708.1	2603418	1-scf00008	1
AX-157895499	1	53.983	KV452708.1	2604631	1-scf00008	1
AX-157895501	1	53.983	KV452708.1	2608121	1-scf00008	1
AX-157895502	1	53.983	KV452708.1	2609283	1-scf00008	1
AX-157895503	1	53.983	KV452708.1	2609526	1-scf00008	1
AX-158404347	1	53.983	KV452708.1	2613887	1-scf00008	1
AX-158404348	1	53.983	KV452708.1	2614846	1-scf00008	1
AX-158404350	1	53.983	KV452708.1	2615082	1-scf00008	1
AX-157895509	1	53.983	KV452708.1	2616561	1-scf00008	1
AX-157775660	1	53.983	KV452708.1	2618093	1-scf00008	1
AX-157895513	1	53.983	KV452708.1	2619458	1-scf00008	1
AX-157895514	1	53.983	KV452708.1	2622390	1-scf00008	1
AX-157895519	1	53.983	KV452708.1	2633804	1-scf00008	1
AX-157895521	1	53.983	KV452708.1	2635701	1-scf00008	1
AX-157810843	1	53.983	KV452708.1	2636013	1-scf00008	1
AX-157895524	1	53.983	KV452708.1	2644854	1-scf00008	1
AX-158379128	1	53.983	KV452708.1	2651850	1-scf00008	1
AX-157810848	1	53.983	KV452708.1	2661186	1-scf00008	1
AX-157895533	1	53.983	KV452708.1	2662413	1-scf00008	1
AX-157895544	1	53.983	KV452708.1	2677076	1-scf00008	1
AX-157895554	1	53.983	KV452708.1	2684778	1-scf00008	1
AX-157895565	1	53.983	KV452708.1	2707035	1-scf00008	1
AX-157895566	1	53.983	KV452708.1	2708267	1-scf00008	1
AX-157895576	1	53.983	KV452708.1	2722414	1-scf00008	1
AX-158379129	1	53.983	KV452708.1	2723402	1-scf00008	1
AX-157895586	1	53.983	KV452708.1	2731194	1-scf00008	1
AX-157895588	1	53.983	KV452708.1	2734981	1-scf00008	1
AX-157895589	1	53.983	KV452708.1	2736025	1-scf00008	1
AX-157895600	1	53.983	KV452708.1	2743179	1-scf00008	1
AX-157895605	1	53.983	KV452708.1	2747066	1-scf00008	1
AX-157739816	1	53.983	KV452708.1	2755009	1-scf00008	1
AX-157895612	1	53.983	KV452708.1	2755773	1-scf00008	1
AX-157895613	1	53.983	KV452708.1	2756388	1-scf00008	1
AX-157895616	1	53.983	KV452708.1	2758269	1-scf00008	1

AX-157895626	1	53.983	KV452708.1	2769694	1-scf00008	1
AX-157895633	1	53.983	KV452708.1	2786808	1-scf00008	1
AX-157895635	1	53.983	KV452708.1	2788235	1-scf00008	1
AX-157895639	1	53.983	KV452708.1	2793568	1-scf00008	1
AX-157895640	1	53.983	KV452708.1	2795234	1-scf00008	1
AX-157895650	1	53.983	KV452708.1	2807578	1-scf00008	1
AX-157895651	1	53.983	KV452708.1	2808348	1-scf00008	1
AX-157895654	1	53.983	KV452708.1	2809335	1-scf00008	1
AX-157895657	1	53.983	KV452708.1	2811387	1-scf00008	1
AX-157895665	1	53.983	KV452708.1	2819450	1-scf00008	1
AX-157895673	1	53.983	KV452708.1	2830553	1-scf00008	1
AX-157895678	1	53.983	KV452708.1	2842460	1-scf00008	1
AX-157895679	1	53.983	KV452708.1	2844937	1-scf00008	1
AX-157895681	1	53.983	KV452708.1	2847183	1-scf00008	1
AX-157895682	1	53.983	KV452708.1	2847791	1-scf00008	1
AX-157895693	1	53.983	KV452708.1	2858957	1-scf00008	1
AX-157895700	1	53.983	KV452708.1	2868177	1-scf00008	1
AX-157895708	1	53.983	KV452708.1	2876244	1-scf00008	1
AX-157895710	1	53.983	KV452708.1	2878183	1-scf00008	1
AX-157895715	1	53.983	KV452708.1	2882907	1-scf00008	1
AX-157895733	1	53.983	KV452708.1	2905327	1-scf00008	1
AX-157895741	1	53.983	KV452708.1	2914327	1-scf00008	1
AX-157895748	1	53.983	KV452708.1	2923924	1-scf00008	1
AX-157895750	1	53.983	KV452708.1	2927417	1-scf00008	1
AX-157810867	1	53.983	KV452708.1	2939225	1-scf00008	1
AX-157895764	1	53.983	KV452708.1	2946506	1-scf00008	1
AX-157895766	1	53.983	KV452708.1	2947641	1-scf00008	1
AX-157895767	1	53.983	KV452708.1	2948609	1-scf00008	1
AX-157895776	1	53.983	KV452708.1	2961024	1-scf00008	1
AX-157895798	1	53.983	KV452708.1	2988842	1-scf00008	1
AX-157895807	1	53.983	KV452708.1	2996706	1-scf00008	1
AX-157895872	1	53.983	KV452708.1	3057187	1-scf00008	1
AX-157895978	1	53.983	KV452708.1	3171724	1-scf00008	1
AX-157896307	1	53.983	KV452709.1	297379	1-scf00009	1
AX-157722127	1	53.983	KV452709.1	3617571	1-scf00009	1
AX-157902592	1	53.983	KV452713.1	995134	1-scf00011	1
AX-157904539	1	53.983	KV452713.1	3914612	1-scf00011	1
AX-157712425	1	53.983	FI885482.1	593	HORI212 Ictalu	UN
AX-157895508	1	53.992	KV452708.1	2615780	1-scf00008	1
AX-157895515	1	54.004	KV452708.1	2622979	1-scf00008	1
AX-157895517	1	54.014	KV452708.1	2629215	1-scf00008	1
AX-157894968	1	54.029	KV452708.1	1674064	1-scf00008	1
AX-157894969	1	54.029	KV452708.1	1674940	1-scf00008	1
AX-157895148	1	54.029	KV452708.1	1987802	1-scf00008	1
AX-157895152	1	54.029	KV452708.1	1998438	1-scf00008	1
AX-157895153	1	54.029	KV452708.1	1999269	1-scf00008	1
AX-157810842	1	54.029	KV452708.1	2630930	1-scf00008	1
AX-157895522	1	54.029	KV452708.1	2638679	1-scf00008	1
AX-157810845	1	54.029	KV452708.1	2650796	1-scf00008	1
AX-157895526	1	54.029	KV452708.1	2652318	1-scf00008	1
AX-157895527	1	54.029	KV452708.1	2653861	1-scf00008	1

AX-157810846	1	54.029	KV452708.1	2654402	1-scf00008	1
AX-157810847	1	54.029	KV452708.1	2657212	1-scf00008	1
AX-157895529	1	54.029	KV452708.1	2658225	1-scf00008	1
AX-157895531	1	54.029	KV452708.1	2660595	1-scf00008	1
AX-157895536	1	54.029	KV452708.1	2669839	1-scf00008	1
AX-157895537	1	54.029	KV452708.1	2670606	1-scf00008	1
AX-157895538	1	54.029	KV452708.1	2671344	1-scf00008	1
AX-157895541	1	54.029	KV452708.1	2674491	1-scf00008	1
AX-157895542	1	54.029	KV452708.1	2675180	1-scf00008	1
AX-157895543	1	54.029	KV452708.1	2676389	1-scf00008	1
AX-157895559	1	54.029	KV452708.1	2693003	1-scf00008	1
AX-157895582	1	54.029	KV452708.1	2725989	1-scf00008	1
AX-157895583	1	54.029	KV452708.1	2726666	1-scf00008	1
AX-157895584	1	54.029	KV452708.1	2726897	1-scf00008	1
AX-157895585	1	54.029	KV452708.1	2730642	1-scf00008	1
AX-157895597	1	54.029	KV452708.1	2741520	1-scf00008	1
AX-157895599	1	54.029	KV452708.1	2742607	1-scf00008	1
AX-157895602	1	54.029	KV452708.1	2745090	1-scf00008	1
AX-157895603	1	54.029	KV452708.1	2745350	1-scf00008	1
AX-157895607	1	54.029	KV452708.1	2748876	1-scf00008	1
AX-157741890	1	54.029	KV452708.1	2774431	1-scf00008	1
AX-157895630	1	54.029	KV452708.1	2777418	1-scf00008	1
AX-157895643	1	54.029	KV452708.1	2798560	1-scf00008	1
AX-157895568	1	54.042	KV452708.1	2709779	1-scf00008	1
AX-157895572	1	54.058	KV452708.1	2719275	1-scf00008	1
AX-157895574	1	54.075	KV452708.1	2719967	1-scf00008	1
AX-157810718	1	54.092	KV452708.1	976821	1-scf00008	1
AX-157894625	1	54.092	KV452708.1	1050026	1-scf00008	1
AX-157894630	1	54.092	KV452708.1	1053837	1-scf00008	1
AX-157895108	1	54.092	KV452708.1	1855242	1-scf00008	1
AX-157895123	1	54.092	KV452708.1	1877369	1-scf00008	1
AX-157895510	1	54.092	KV452708.1	2616923	1-scf00008	1
AX-157895560	1	54.092	KV452708.1	2694529	1-scf00008	1
AX-157895573	1	54.092	KV452708.1	2719659	1-scf00008	1
AX-157895575	1	54.092	KV452708.1	2720151	1-scf00008	1
AX-157895577	1	54.092	KV452708.1	2723099	1-scf00008	1
AX-157895578	1	54.092	KV452708.1	2723359	1-scf00008	1
AX-157895590	1	54.092	KV452708.1	2736861	1-scf00008	1
AX-157895592	1	54.092	KV452708.1	2737641	1-scf00008	1
AX-158403844	1	54.092	KV452708.1	2743892	1-scf00008	1
AX-157895601	1	54.092	KV452708.1	2744850	1-scf00008	1
AX-157895623	1	54.092	KV452708.1	2767468	1-scf00008	1
AX-157895625	1	54.092	KV452708.1	2768962	1-scf00008	1
AX-157710464	1	54.092	KV452708.1	2770028	1-scf00008	1
AX-157810858	1	54.092	KV452708.1	2778173	1-scf00008	1
AX-157895634	1	54.092	KV452708.1	2787694	1-scf00008	1
AX-157895637	1	54.092	KV452708.1	2790645	1-scf00008	1
AX-157810862	1	54.092	KV452708.1	2826739	1-scf00008	1
AX-157895686	1	54.092	KV452708.1	2852387	1-scf00008	1
AX-157895743	1	54.092	KV452708.1	2920836	1-scf00008	1
AX-157895779	1	54.092	KV452708.1	2963970	1-scf00008	1

AX-157810700	1	54.201	KV452708.1	853695	1-scf00008	1
AX-157894543	1	54.201	KV452708.1	920931	1-scf00008	1
AX-157894548	1	54.201	KV452708.1	928308	1-scf00008	1
AX-157894570	1	54.201	KV452708.1	950423	1-scf00008	1
AX-157894593	1	54.201	KV452708.1	1007659	1-scf00008	1
AX-157894595	1	54.201	KV452708.1	1009263	1-scf00008	1
AX-157894633	1	54.201	KV452708.1	1058555	1-scf00008	1
AX-157894635	1	54.201	KV452708.1	1066035	1-scf00008	1
AX-157708701	1	54.201	KV452708.1	1068010	1-scf00008	1
AX-157894658	1	54.201	KV452708.1	1118228	1-scf00008	1
AX-157894674	1	54.201	KV452708.1	1137827	1-scf00008	1
AX-157894724	1	54.201	KV452708.1	1269302	1-scf00008	1
AX-157894733	1	54.201	KV452708.1	1280844	1-scf00008	1
AX-157894745	1	54.201	KV452708.1	1289685	1-scf00008	1
AX-157894746	1	54.201	KV452708.1	1289880	1-scf00008	1
AX-157894747	1	54.201	KV452708.1	1290169	1-scf00008	1
AX-157894802	1	54.201	KV452708.1	1381995	1-scf00008	1
AX-157894826	1	54.201	KV452708.1	1411887	1-scf00008	1
AX-157810749	1	54.201	KV452708.1	1414873	1-scf00008	1
AX-157753320	1	54.201	KV452708.1	1422138	1-scf00008	1
AX-157894846	1	54.201	KV452708.1	1459970	1-scf00008	1
AX-157894865	1	54.201	KV452708.1	1499395	1-scf00008	1
AX-157894877	1	54.201	KV452708.1	1513069	1-scf00008	1
AX-157894878	1	54.201	KV452708.1	1513635	1-scf00008	1
AX-157894897	1	54.201	KV452708.1	1538581	1-scf00008	1
AX-157895007	1	54.201	KV452708.1	1719908	1-scf00008	1
AX-157810788	1	54.201	KV452708.1	1832001	1-scf00008	1
AX-157895191	1	54.201	KV452708.1	2078310	1-scf00008	1
AX-157895220	1	54.201	KV452708.1	2133716	1-scf00008	1
AX-157895244	1	54.201	KV452708.1	2171899	1-scf00008	1
AX-157895246	1	54.201	KV452708.1	2172726	1-scf00008	1
AX-157895263	1	54.201	KV452708.1	2188459	1-scf00008	1
AX-157895288	1	54.201	KV452708.1	2221886	1-scf00008	1
AX-158404338	1	54.201	KV452708.1	2305878	1-scf00008	1
AX-157895355	1	54.201	KV452708.1	2308401	1-scf00008	1
AX-157895369	1	54.201	KV452708.1	2330162	1-scf00008	1
AX-157895380	1	54.201	KV452708.1	2365178	1-scf00008	1
AX-157895441	1	54.201	KV452708.1	2529462	1-scf00008	1
AX-157810832	1	54.201	KV452708.1	2545654	1-scf00008	1
AX-157895460	1	54.201	KV452708.1	2559272	1-scf00008	1
AX-157810837	1	54.201	KV452708.1	2598395	1-scf00008	1
AX-158404351	1	54.201	KV452708.1	2639076	1-scf00008	1
AX-157895525	1	54.201	KV452708.1	2647727	1-scf00008	1
AX-157895528	1	54.201	KV452708.1	2657017	1-scf00008	1
AX-157895530	1	54.201	KV452708.1	2659972	1-scf00008	1
AX-157895547	1	54.201	KV452708.1	2678980	1-scf00008	1
AX-157895567	1	54.201	KV452708.1	2709343	1-scf00008	1
AX-157810854	1	54.201	KV452708.1	2712958	1-scf00008	1
AX-157895570	1	54.201	KV452708.1	2716401	1-scf00008	1
AX-157895571	1	54.201	KV452708.1	2716614	1-scf00008	1
AX-157810856	1	54.201	KV452708.1	2717256	1-scf00008	1

AX-157895598	1	54.201	KV452708.1	2742115	1-scf00008	1
AX-157895606	1	54.201	KV452708.1	2747530	1-scf00008	1
AX-157895622	1	54.201	KV452708.1	2766912	1-scf00008	1
AX-157895629	1	54.201	KV452708.1	2773432	1-scf00008	1
AX-157895631	1	54.201	KV452708.1	2781875	1-scf00008	1
AX-157895638	1	54.201	KV452708.1	2793174	1-scf00008	1
AX-157895642	1	54.201	KV452708.1	2797056	1-scf00008	1
AX-157895646	1	54.201	KV452708.1	2802933	1-scf00008	1
AX-157810859	1	54.201	KV452708.1	2805053	1-scf00008	1
AX-157721444	1	54.201	KV452708.1	2805271	1-scf00008	1
AX-157895649	1	54.201	KV452708.1	2807053	1-scf00008	1
AX-157895653	1	54.201	KV452708.1	2809117	1-scf00008	1
AX-157895658	1	54.201	KV452708.1	2811763	1-scf00008	1
AX-157895659	1	54.201	KV452708.1	2812950	1-scf00008	1
AX-157895661	1	54.201	KV452708.1	2815969	1-scf00008	1
AX-157895666	1	54.201	KV452708.1	2819843	1-scf00008	1
AX-157895667	1	54.201	KV452708.1	2820595	1-scf00008	1
AX-157810864	1	54.201	KV452708.1	2832070	1-scf00008	1
AX-157810865	1	54.201	KV452708.1	2836403	1-scf00008	1
AX-157895683	1	54.201	KV452708.1	2850425	1-scf00008	1
AX-157895685	1	54.201	KV452708.1	2851193	1-scf00008	1
AX-157895688	1	54.201	KV452708.1	2854921	1-scf00008	1
AX-157895690	1	54.201	KV452708.1	2856989	1-scf00008	1
AX-157895691	1	54.201	KV452708.1	2857979	1-scf00008	1
AX-157895696	1	54.201	KV452708.1	2864437	1-scf00008	1
AX-157895703	1	54.201	KV452708.1	2872148	1-scf00008	1
AX-157895713	1	54.201	KV452708.1	2879886	1-scf00008	1
AX-157895716	1	54.201	KV452708.1	2883469	1-scf00008	1
AX-157895719	1	54.201	KV452708.1	2885188	1-scf00008	1
AX-157895724	1	54.201	KV452708.1	2894933	1-scf00008	1
AX-157895727	1	54.201	KV452708.1	2896194	1-scf00008	1
AX-157895730	1	54.201	KV452708.1	2901028	1-scf00008	1
AX-157895736	1	54.201	KV452708.1	2911221	1-scf00008	1
AX-157895738	1	54.201	KV452708.1	2912004	1-scf00008	1
AX-157895739	1	54.201	KV452708.1	2912396	1-scf00008	1
AX-157853564	1	54.201	KV452708.1	2912755	1-scf00008	1
AX-158404358	1	54.201	KV452708.1	2920078	1-scf00008	1
AX-157895745	1	54.201	KV452708.1	2922748	1-scf00008	1
AX-157895746	1	54.201	KV452708.1	2923274	1-scf00008	1
AX-157895756	1	54.201	KV452708.1	2936176	1-scf00008	1
AX-157895850	1	54.201	KV452708.1	3040572	1-scf00008	1
AX-157757862	1	54.201	KV452708.1	3077650	1-scf00008	1
AX-157895892	1	54.201	KV452708.1	3083512	1-scf00008	1
AX-157895893	1	54.201	KV452708.1	3084163	1-scf00008	1
AX-157895895	1	54.201	KV452708.1	3085169	1-scf00008	1
AX-157895898	1	54.201	KV452708.1	3087977	1-scf00008	1
AX-157895899	1	54.201	KV452708.1	3090507	1-scf00008	1
AX-157895904	1	54.201	KV452708.1	3096000	1-scf00008	1
AX-157895906	1	54.201	KV452708.1	3096950	1-scf00008	1
AX-157895908	1	54.201	KV452708.1	3098230	1-scf00008	1
AX-157895909	1	54.201	KV452708.1	3099007	1-scf00008	1

AX-157768471	1	54.201	KV452709.1	171979	1-scf00009	1
AX-157896375	1	54.201	KV452709.1	374083	1-scf00009	1
AX-157896499	1	54.201	KV452709.1	523893	1-scf00009	1
AX-157896512	1	54.201	KV452709.1	547202	1-scf00009	1
AX-157896542	1	54.201	KV452709.1	574001	1-scf00009	1
AX-157896688	1	54.201	KV452709.1	770424	1-scf00009	1
AX-157896702	1	54.201	KV452709.1	782758	1-scf00009	1
AX-157896707	1	54.201	KV452709.1	785629	1-scf00009	1
AX-157896804	1	54.201	KV452709.1	947097	1-scf00009	1
AX-157896818	1	54.201	KV452709.1	969469	1-scf00009	1
AX-157896854	1	54.201	KV452709.1	1021937	1-scf00009	1
AX-157896856	1	54.201	KV452709.1	1023145	1-scf00009	1
AX-157897338	1	54.201	KV452709.1	1725184	1-scf00009	1
AX-157897605	1	54.201	KV452709.1	2149268	1-scf00009	1
AX-157695716	1	54.201	KV452709.1	2178883	1-scf00009	1
AX-157897643	1	54.201	KV452709.1	2203574	1-scf00009	1
AX-157897663	1	54.201	KV452709.1	2232781	1-scf00009	1
AX-157897709	1	54.201	KV452709.1	2315135	1-scf00009	1
AX-157897730	1	54.201	KV452709.1	2341802	1-scf00009	1
AX-157897805	1	54.201	KV452709.1	2465895	1-scf00009	1
AX-157751855	1	54.201	KV452709.1	2470106	1-scf00009	1
AX-157897824	1	54.201	KV452709.1	2490400	1-scf00009	1
AX-157898003	1	54.201	KV452709.1	2804697	1-scf00009	1
AX-157898027	1	54.201	KV452709.1	2834463	1-scf00009	1
AX-157898464	1	54.201	KV452709.1	3735533	1-scf00009	1
AX-157898467	1	54.201	KV452709.1	3765348	1-scf00009	1
AX-157898547	1	54.201	KV452709.1	3922585	1-scf00009	1
AX-157898600	1	54.201	KV452709.1	3983680	1-scf00009	1
AX-157898601	1	54.201	KV452709.1	3987593	1-scf00009	1
AX-157898602	1	54.201	KV452709.1	3988707	1-scf00009	1
AX-157898604	1	54.201	KV452709.1	3990715	1-scf00009	1
AX-157811184	1	54.201	KV452709.1	3992528	1-scf00009	1
AX-157898615	1	54.201	KV452709.1	4009679	1-scf00009	1
AX-157898641	1	54.201	KV452709.1	4051777	1-scf00009	1
AX-157741241	1	54.201	KV452709.1	4075498	1-scf00009	1
AX-157898755	1	54.201	KV452709.1	4225114	1-scf00009	1
AX-157898770	1	54.201	KV452709.1	4244628	1-scf00009	1
AX-157898834	1	54.201	KV452709.1	4337503	1-scf00009	1
AX-157898862	1	54.201	KV452709.1	4375808	1-scf00009	1
AX-157899135	1	54.201	KV452709.1	4720827	1-scf00009	1
AX-157899238	1	54.201	KV452709.1	4909185	1-scf00009	1
AX-157899252	1	54.201	KV452709.1	4922067	1-scf00009	1
AX-157899270	1	54.201	KV452709.1	4951774	1-scf00009	1
AX-157899280	1	54.201	KV452709.1	4966921	1-scf00009	1
AX-157899466	1	54.201	KV452709.1	5215686	1-scf00009	1
AX-157899467	1	54.201	KV452709.1	5216046	1-scf00009	1
AX-157899503	1	54.201	KV452709.1	5251766	1-scf00009	1
AX-157899537	1	54.201	KV452709.1	5288856	1-scf00009	1
AX-157899574	1	54.201	KV452709.1	5329165	1-scf00009	1
AX-157743848	1	54.201	KV452709.1	5446494	1-scf00009	1
AX-157901257	1	54.201	KV452711.1	1865834	1-scf00010	1

AX-157895753	1	54.311	KV452708.1	2930805	1-scf00008	1
AX-157895754	1	54.311	KV452708.1	2933308	1-scf00008	1
AX-157895757	1	54.311	KV452708.1	2936637	1-scf00008	1
AX-157895758	1	54.311	KV452708.1	2936871	1-scf00008	1
AX-157895759	1	54.311	KV452708.1	2938625	1-scf00008	1
AX-157895760	1	54.311	KV452708.1	2940552	1-scf00008	1
AX-157895768	1	54.311	KV452708.1	2950829	1-scf00008	1
AX-157895769	1	54.311	KV452708.1	2951812	1-scf00008	1
AX-157895773	1	54.311	KV452708.1	2956360	1-scf00008	1
AX-157895775	1	54.311	KV452708.1	2957869	1-scf00008	1
AX-157895781	1	54.311	KV452708.1	2967478	1-scf00008	1
AX-157708824	1	54.311	KV452708.1	2967706	1-scf00008	1
AX-157895786	1	54.311	KV452708.1	2971281	1-scf00008	1
AX-157895789	1	54.311	KV452708.1	2974622	1-scf00008	1
AX-157895790	1	54.42	KV452708.1	2975594	1-scf00008	1
AX-157895791	1	54.42	KV452708.1	2976946	1-scf00008	1
AX-157895793	1	54.42	KV452708.1	2983946	1-scf00008	1
AX-157895794	1	54.42	KV452708.1	2984859	1-scf00008	1
AX-157895795	1	54.42	KV452708.1	2987119	1-scf00008	1
AX-157895797	1	54.42	KV452708.1	2988327	1-scf00008	1
AX-157895800	1	54.447	KV452708.1	2990005	1-scf00008	1
AX-157895802	1	54.485	KV452708.1	2991396	1-scf00008	1
AX-157895805	1	54.529	KV452708.1	2993510	1-scf00008	1
AX-157810869	1	54.529	KV452708.1	2993951	1-scf00008	1
AX-157895806	1	54.529	KV452708.1	2995500	1-scf00008	1
AX-157743715	1	54.529	KV452708.1	2996413	1-scf00008	1
AX-157895808	1	54.584	KV452708.1	2998926	1-scf00008	1
AX-157895851	1	54.584	KV452708.1	3041729	1-scf00008	1
AX-157895811	1	54.638	KV452708.1	3001393	1-scf00008	1
AX-157895814	1	54.684	KV452708.1	3002749	1-scf00008	1
AX-157810738	1	54.725	KV452708.1	1308681	1-scf00008	1
AX-157895799	1	54.725	KV452708.1	2989050	1-scf00008	1
AX-157895801	1	54.725	KV452708.1	2990396	1-scf00008	1
AX-157895804	1	54.725	KV452708.1	2992782	1-scf00008	1
AX-157895815	1	54.725	KV452708.1	3003104	1-scf00008	1
AX-157895816	1	54.725	KV452708.1	3003605	1-scf00008	1
AX-157895822	1	54.725	KV452708.1	3012518	1-scf00008	1
AX-157895841	1	54.725	KV452708.1	3032403	1-scf00008	1
AX-157745888	1	54.748	KV452708.1	1928397	1-scf00008	1
AX-157895581	1	54.748	KV452708.1	2725589	1-scf00008	1
AX-157895817	1	54.748	KV452708.1	3004957	1-scf00008	1
AX-157895825	1	54.748	KV452708.1	3016779	1-scf00008	1
AX-157895826	1	54.748	KV452708.1	3017117	1-scf00008	1
AX-157895828	1	54.748	KV452708.1	3018478	1-scf00008	1
AX-157895830	1	54.748	KV452708.1	3019511	1-scf00008	1
AX-157895832	1	54.748	KV452708.1	3020715	1-scf00008	1
AX-157895842	1	54.748	KV452708.1	3032998	1-scf00008	1
AX-157810871	1	54.748	KV452708.1	3040804	1-scf00008	1
AX-157810872	1	54.748	KV452708.1	3040895	1-scf00008	1
AX-158388995	1	54.748	KV452708.1	3042426	1-scf00008	1
AX-157895877	1	54.748	KV452708.1	3065784	1-scf00008	1

AX-157895903	1	54.748	KV452708.1	3094920	1-scf00008	1
AX-157810875	1	54.816	KV452708.1	3045570	1-scf00008	1
AX-157810804	1	54.857	KV452708.1	2021081	1-scf00008	1
AX-157895468	1	54.857	KV452708.1	2571563	1-scf00008	1
AX-157895818	1	54.857	KV452708.1	3009695	1-scf00008	1
AX-157895853	1	54.857	KV452708.1	3042657	1-scf00008	1
AX-157810873	1	54.857	KV452708.1	3043357	1-scf00008	1
AX-157810874	1	54.857	KV452708.1	3043459	1-scf00008	1
AX-157895854	1	54.857	KV452708.1	3043868	1-scf00008	1
AX-157895855	1	54.857	KV452708.1	3044401	1-scf00008	1
AX-157895858	1	54.857	KV452708.1	3045915	1-scf00008	1
AX-157895859	1	54.857	KV452708.1	3046349	1-scf00008	1
AX-157895860	1	54.857	KV452708.1	3046522	1-scf00008	1
AX-157895861	1	54.857	KV452708.1	3046999	1-scf00008	1
AX-157895862	1	54.857	KV452708.1	3047394	1-scf00008	1
AX-157895867	1	54.857	KV452708.1	3052866	1-scf00008	1
AX-157895868	1	54.857	KV452708.1	3053814	1-scf00008	1
AX-157810878	1	54.857	KV452708.1	3055690	1-scf00008	1
AX-157757756	1	54.857	KV452708.1	3056319	1-scf00008	1
AX-157895871	1	54.857	KV452708.1	3056960	1-scf00008	1
AX-157895873	1	54.857	KV452708.1	3058324	1-scf00008	1
AX-157748588	1	54.857	KV452708.1	3064412	1-scf00008	1
AX-157895875	1	54.857	KV452708.1	3064514	1-scf00008	1
AX-157895879	1	54.857	KV452708.1	3067182	1-scf00008	1
AX-157895882	1	54.857	KV452708.1	3069179	1-scf00008	1
AX-157895883	1	54.857	KV452708.1	3072266	1-scf00008	1
AX-157895884	1	54.857	KV452708.1	3072603	1-scf00008	1
AX-157895886	1	54.857	KV452708.1	3073868	1-scf00008	1
AX-157715991	1	54.857	KV452708.1	3074219	1-scf00008	1
AX-157726873	1	54.857	KV452708.1	3078665	1-scf00008	1
AX-157895900	1	54.857	KV452708.1	3091256	1-scf00008	1
AX-157895901	1	54.857	KV452708.1	3092396	1-scf00008	1
AX-157739691	1	54.857	KV452708.1	3093121	1-scf00008	1
AX-157895907	1	54.857	KV452708.1	3097547	1-scf00008	1
AX-157895910	1	54.857	KV452708.1	3099876	1-scf00008	1
AX-157895912	1	54.857	KV452708.1	3101069	1-scf00008	1
AX-157895949	1	54.857	KV452708.1	3139689	1-scf00008	1
AX-157895983	1	54.857	KV452708.1	3178778	1-scf00008	1
AX-157896005	1	54.857	KV452708.1	3200869	1-scf00008	1
AX-157896045	1	54.857	KV452708.1	3240350	1-scf00008	1
AX-157896092	1	54.857	KV452708.1	3308065	1-scf00008	1
AX-158404373	1	54.857	KV452709.1	60401	1-scf00009	1
AX-157706132	1	54.857	3ML01031818	76	scf07439_1	UN
AX-157895662	1	54.966	KV452708.1	2816206	1-scf00008	1
AX-157895762	1	54.966	KV452708.1	2942236	1-scf00008	1
AX-157895891	1	54.966	KV452708.1	3083087	1-scf00008	1
AX-157895918	1	54.966	KV452708.1	3107932	1-scf00008	1
AX-157895920	1	54.966	KV452708.1	3109244	1-scf00008	1
AX-157895921	1	54.966	KV452708.1	3109476	1-scf00008	1
AX-157895922	1	54.966	KV452708.1	3110251	1-scf00008	1
AX-157895923	1	54.966	KV452708.1	3110987	1-scf00008	1

AX-157895924	1	54.966	KV452708.1	3111464	1-scf00008	1
AX-157895925	1	54.966	KV452708.1	3111908	1-scf00008	1
AX-157895926	1	54.966	KV452708.1	3112840	1-scf00008	1
AX-157895927	1	54.966	KV452708.1	3115416	1-scf00008	1
AX-157810879	1	54.966	KV452708.1	3116344	1-scf00008	1
AX-157810880	1	54.966	KV452708.1	3116619	1-scf00008	1
AX-157810881	1	54.966	KV452708.1	3117554	1-scf00008	1
AX-157895928	1	54.966	KV452708.1	3117812	1-scf00008	1
AX-157723708	1	54.966	KV452708.1	3117980	1-scf00008	1
AX-157895929	1	54.966	KV452708.1	3119086	1-scf00008	1
AX-157774832	1	54.966	KV452708.1	3119534	1-scf00008	1
AX-157696222	1	54.966	KV452708.1	3120581	1-scf00008	1
AX-157695408	1	54.966	KV452708.1	3123444	1-scf00008	1
AX-157895931	1	54.966	KV452708.1	3124139	1-scf00008	1
AX-157722229	1	54.966	KV452708.1	3125062	1-scf00008	1
AX-157895933	1	54.966	KV452708.1	3125239	1-scf00008	1
AX-157895934	1	54.966	KV452708.1	3125691	1-scf00008	1
AX-157810882	1	54.966	KV452708.1	3127163	1-scf00008	1
AX-157895936	1	54.966	KV452708.1	3127365	1-scf00008	1
AX-157895937	1	54.966	KV452708.1	3128240	1-scf00008	1
AX-157895939	1	54.966	KV452708.1	3129579	1-scf00008	1
AX-157895940	1	54.966	KV452708.1	3130338	1-scf00008	1
AX-157895941	1	54.966	KV452708.1	3131413	1-scf00008	1
AX-157895942	1	54.966	KV452708.1	3131834	1-scf00008	1
AX-157895943	1	54.966	KV452708.1	3132924	1-scf00008	1
AX-157895944	1	54.966	KV452708.1	3133876	1-scf00008	1
AX-157895945	1	54.966	KV452708.1	3134721	1-scf00008	1
AX-157895947	1	54.966	KV452708.1	3136646	1-scf00008	1
AX-157895948	1	54.966	KV452708.1	3139202	1-scf00008	1
AX-157895950	1	54.966	KV452708.1	3141423	1-scf00008	1
AX-157895953	1	54.966	KV452708.1	3142997	1-scf00008	1
AX-157895955	1	54.966	KV452708.1	3144037	1-scf00008	1
AX-157895957	1	54.966	KV452708.1	3147836	1-scf00008	1
AX-157895958	1	54.966	KV452708.1	3148765	1-scf00008	1
AX-157895959	1	54.966	KV452708.1	3150518	1-scf00008	1
AX-157895960	1	54.966	KV452708.1	3152382	1-scf00008	1
AX-157895961	1	54.966	KV452708.1	3152990	1-scf00008	1
AX-157895962	1	54.966	KV452708.1	3153930	1-scf00008	1
AX-157853565	1	54.966	KV452708.1	3153992	1-scf00008	1
AX-157895963	1	54.966	KV452708.1	3156574	1-scf00008	1
AX-157895964	1	54.966	KV452708.1	3157914	1-scf00008	1
AX-157895965	1	54.966	KV452708.1	3159019	1-scf00008	1
AX-157726213	1	54.966	KV452708.1	3159494	1-scf00008	1
AX-157895968	1	54.966	KV452708.1	3161608	1-scf00008	1
AX-157895969	1	54.966	KV452708.1	3162099	1-scf00008	1
AX-157895970	1	54.966	KV452708.1	3162838	1-scf00008	1
AX-157895971	1	54.966	KV452708.1	3163752	1-scf00008	1
AX-157895972	1	54.966	KV452708.1	3164650	1-scf00008	1
AX-157895973	1	54.966	KV452708.1	3165631	1-scf00008	1
AX-157895974	1	54.966	KV452708.1	3166000	1-scf00008	1
AX-157895975	1	54.966	KV452708.1	3168852	1-scf00008	1

AX-157895976	1	54.966	KV452708.1	3169994	1-scf00008	1
AX-157895979	1	54.966	KV452708.1	3172929	1-scf00008	1
AX-157895980	1	54.966	KV452708.1	3175838	1-scf00008	1
AX-157895982	1	54.966	KV452708.1	3176716	1-scf00008	1
AX-157895984	1	54.966	KV452708.1	3180812	1-scf00008	1
AX-157895985	1	54.966	KV452708.1	3181474	1-scf00008	1
AX-157895986	1	54.966	KV452708.1	3181807	1-scf00008	1
AX-157895987	1	54.966	KV452708.1	3182369	1-scf00008	1
AX-157895988	1	54.966	KV452708.1	3182974	1-scf00008	1
AX-157895989	1	54.966	KV452708.1	3184718	1-scf00008	1
AX-157895990	1	54.966	KV452708.1	3185583	1-scf00008	1
AX-157895991	1	54.966	KV452708.1	3186145	1-scf00008	1
AX-157895992	1	54.966	KV452708.1	3187179	1-scf00008	1
AX-157895993	1	54.966	KV452708.1	3188081	1-scf00008	1
AX-157895994	1	54.966	KV452708.1	3189127	1-scf00008	1
AX-157895995	1	54.966	KV452708.1	3192101	1-scf00008	1
AX-157895996	1	54.966	KV452708.1	3193092	1-scf00008	1
AX-157895997	1	54.966	KV452708.1	3194137	1-scf00008	1
AX-157895998	1	54.966	KV452708.1	3194910	1-scf00008	1
AX-157895999	1	54.966	KV452708.1	3195603	1-scf00008	1
AX-157896001	1	54.966	KV452708.1	3196840	1-scf00008	1
AX-157896002	1	54.966	KV452708.1	3197840	1-scf00008	1
AX-157896003	1	54.966	KV452708.1	3198760	1-scf00008	1
AX-157896004	1	54.966	KV452708.1	3199910	1-scf00008	1
AX-158379130	1	54.966	KV452708.1	3199968	1-scf00008	1
AX-157896006	1	54.966	KV452708.1	3201618	1-scf00008	1
AX-157896007	1	54.966	KV452708.1	3203208	1-scf00008	1
AX-157896008	1	54.966	KV452708.1	3203668	1-scf00008	1
AX-157896009	1	54.966	KV452708.1	3204577	1-scf00008	1
AX-157896010	1	54.966	KV452708.1	3205562	1-scf00008	1
AX-157896011	1	54.966	KV452708.1	3206330	1-scf00008	1
AX-157896014	1	54.966	KV452708.1	3209150	1-scf00008	1
AX-157896015	1	54.966	KV452708.1	3210456	1-scf00008	1
AX-157896016	1	54.966	KV452708.1	3211412	1-scf00008	1
AX-157896017	1	54.966	KV452708.1	3212724	1-scf00008	1
AX-157896019	1	54.966	KV452708.1	3214182	1-scf00008	1
AX-157896021	1	54.966	KV452708.1	3216080	1-scf00008	1
AX-157896022	1	54.966	KV452708.1	3216635	1-scf00008	1
AX-157896023	1	54.966	KV452708.1	3217419	1-scf00008	1
AX-157896025	1	54.966	KV452708.1	3219087	1-scf00008	1
AX-157775561	1	54.966	KV452708.1	3220042	1-scf00008	1
AX-157896027	1	54.966	KV452708.1	3222848	1-scf00008	1
AX-157896028	1	54.966	KV452708.1	3223513	1-scf00008	1
AX-157896030	1	54.966	KV452708.1	3225417	1-scf00008	1
AX-157896031	1	54.966	KV452708.1	3226148	1-scf00008	1
AX-157775535	1	54.966	KV452708.1	3227197	1-scf00008	1
AX-157896034	1	54.966	KV452708.1	3229305	1-scf00008	1
AX-157896035	1	54.966	KV452708.1	3230285	1-scf00008	1
AX-157896036	1	54.966	KV452708.1	3233770	1-scf00008	1
AX-157896037	1	54.966	KV452708.1	3234686	1-scf00008	1
AX-157896038	1	54.966	KV452708.1	3235288	1-scf00008	1

AX-157896040	1	54.966	KV452708.1	3236972	1-scf00008	1
AX-157896041	1	54.966	KV452708.1	3237582	1-scf00008	1
AX-157896047	1	54.966	KV452708.1	3244762	1-scf00008	1
AX-158404364	1	54.966	KV452708.1	3246616	1-scf00008	1
AX-157810885	1	54.966	KV452708.1	3252575	1-scf00008	1
AX-157896056	1	54.966	KV452708.1	3256307	1-scf00008	1
AX-157713399	1	54.966	KV452708.1	3267812	1-scf00008	1
AX-157896150	1	54.966	KV452709.1	87842	1-scf00009	1
AX-157896202	1	54.966	KV452709.1	156496	1-scf00009	1
AX-157896204	1	54.966	KV452709.1	158258	1-scf00009	1
AX-157896044	1	55.09	KV452708.1	3239902	1-scf00008	1
AX-157895966	1	55.185	KV452708.1	3159876	1-scf00008	1
AX-157896058	1	55.185	KV452708.1	3257697	1-scf00008	1
AX-157719587	1	55.185	KV452708.1	3257761	1-scf00008	1
AX-157868506	1	55.185	KV452708.1	3258305	1-scf00008	1
AX-157896065	1	55.185	KV452708.1	3269500	1-scf00008	1
AX-157896070	1	55.185	KV452708.1	3280263	1-scf00008	1
AX-157896071	1	55.185	KV452708.1	3282136	1-scf00008	1
AX-157896072	1	55.185	KV452708.1	3282777	1-scf00008	1
AX-157896074	1	55.185	KV452708.1	3283540	1-scf00008	1
AX-157896076	1	55.185	KV452708.1	3284392	1-scf00008	1
AX-157896078	1	55.185	KV452708.1	3288052	1-scf00008	1
AX-157896080	1	55.185	KV452708.1	3290620	1-scf00008	1
AX-157896082	1	55.185	KV452708.1	3294070	1-scf00008	1
AX-157896083	1	55.185	KV452708.1	3294522	1-scf00008	1
AX-157810890	1	55.185	KV452708.1	3296626	1-scf00008	1
AX-158404365	1	55.185	KV452708.1	3298281	1-scf00008	1
AX-157896085	1	55.185	KV452708.1	3303360	1-scf00008	1
AX-157896086	1	55.185	KV452708.1	3304072	1-scf00008	1
AX-157896088	1	55.185	KV452708.1	3304864	1-scf00008	1
AX-157896089	1	55.185	KV452708.1	3305342	1-scf00008	1
AX-157896091	1	55.185	KV452708.1	3307710	1-scf00008	1
AX-157896093	1	55.185	KV452708.1	3308675	1-scf00008	1
AX-157896094	1	55.185	KV452708.1	3311377	1-scf00008	1
AX-157896099	1	55.185	KV452709.1	20515	1-scf00009	1
AX-157896100	1	55.185	KV452709.1	20946	1-scf00009	1
AX-157896101	1	55.185	KV452709.1	23418	1-scf00009	1
AX-157896107	1	55.185	KV452709.1	28358	1-scf00009	1
AX-157896108	1	55.185	KV452709.1	29312	1-scf00009	1
AX-157896109	1	55.185	KV452709.1	30875	1-scf00009	1
AX-157896110	1	55.185	KV452709.1	32115	1-scf00009	1
AX-157896111	1	55.185	KV452709.1	32460	1-scf00009	1
AX-157896113	1	55.185	KV452709.1	33718	1-scf00009	1
AX-157896114	1	55.185	KV452709.1	33925	1-scf00009	1
AX-157810892	1	55.185	KV452709.1	34444	1-scf00009	1
AX-157896115	1	55.185	KV452709.1	36850	1-scf00009	1
AX-157896119	1	55.185	KV452709.1	40362	1-scf00009	1
AX-157896120	1	55.185	KV452709.1	41336	1-scf00009	1
AX-157896123	1	55.185	KV452709.1	45751	1-scf00009	1
AX-157896124	1	55.185	KV452709.1	46191	1-scf00009	1
AX-157896125	1	55.185	KV452709.1	46655	1-scf00009	1

AX-158388996	1	55.185	KV452709.1	51934	1-scf00009	1
AX-157896144	1	55.185	KV452709.1	80676	1-scf00009	1
AX-157896254	1	55.185	KV452709.1	211797	1-scf00009	1
AX-157896280	1	55.185	KV452709.1	245485	1-scf00009	1
AX-157896583	1	55.185	KV452709.1	629329	1-scf00009	1
AX-157896117	1	55.292	KV452709.1	38318	1-scf00009	1
AX-157896118	1	55.292	KV452709.1	39426	1-scf00009	1
AX-157895967	1	55.509	KV452708.1	3161191	1-scf00008	1
AX-157716021	1	55.509	KV452708.1	3249595	1-scf00008	1
AX-157896068	1	55.509	KV452708.1	3271838	1-scf00008	1
AX-157896127	1	55.509	KV452709.1	52321	1-scf00009	1
AX-157896128	1	55.509	KV452709.1	52876	1-scf00009	1
AX-157896130	1	55.509	KV452709.1	56847	1-scf00009	1
AX-157896131	1	55.509	KV452709.1	57261	1-scf00009	1
AX-157896132	1	55.509	KV452709.1	57819	1-scf00009	1
AX-158403860	1	55.509	KV452709.1	60277	1-scf00009	1
AX-157896133	1	55.509	KV452709.1	61557	1-scf00009	1
AX-157896134	1	55.509	KV452709.1	65551	1-scf00009	1
AX-157896135	1	55.509	KV452709.1	66040	1-scf00009	1
AX-157896136	1	55.509	KV452709.1	66241	1-scf00009	1
AX-157810897	1	55.509	KV452709.1	66744	1-scf00009	1
AX-157810898	1	55.509	KV452709.1	66959	1-scf00009	1
AX-157720868	1	55.509	KV452709.1	67494	1-scf00009	1
AX-157896145	1	55.509	KV452709.1	82622	1-scf00009	1
AX-157896146	1	55.509	KV452709.1	83805	1-scf00009	1
AX-157896166	1	55.509	KV452709.1	111752	1-scf00009	1
AX-157810903	1	55.509	KV452709.1	173533	1-scf00009	1
AX-157810899	1	55.512	KV452709.1	74156	1-scf00009	1
AX-157896143	1	55.512	KV452709.1	80318	1-scf00009	1
AX-157896141	1	55.515	KV452709.1	79303	1-scf00009	1
AX-157896147	1	55.518	KV452709.1	85170	1-scf00009	1
AX-157724475	1	55.535	KV452709.1	86680	1-scf00009	1
AX-157896151	1	55.569	KV452709.1	88698	1-scf00009	1
AX-157896152	1	55.608	KV452709.1	89851	1-scf00009	1
AX-157810746	1	55.618	KV452708.1	1387682	1-scf00008	1
AX-157810748	1	55.618	KV452708.1	1414480	1-scf00008	1
AX-157894874	1	55.618	KV452708.1	1511009	1-scf00008	1
AX-157894875	1	55.618	KV452708.1	1511707	1-scf00008	1
AX-157894876	1	55.618	KV452708.1	1511999	1-scf00008	1
AX-157895977	1	55.618	KV452708.1	3171054	1-scf00008	1
AX-157853566	1	55.618	KV452708.1	3236484	1-scf00008	1
AX-157810893	1	55.618	KV452709.1	41609	1-scf00009	1
AX-157896121	1	55.618	KV452709.1	42649	1-scf00009	1
AX-157810894	1	55.618	KV452709.1	42999	1-scf00009	1
AX-157896167	1	55.618	KV452709.1	114743	1-scf00009	1
AX-157896171	1	55.618	KV452709.1	120608	1-scf00009	1
AX-157896177	1	55.618	KV452709.1	126408	1-scf00009	1
AX-157896178	1	55.618	KV452709.1	127031	1-scf00009	1
AX-157896369	1	55.618	KV452709.1	366680	1-scf00009	1
AX-157896506	1	55.618	KV452709.1	540626	1-scf00009	1
AX-157896507	1	55.618	KV452709.1	541030	1-scf00009	1

AX-157896537	1	55.618	KV452709.1	568688	1-scf00009	1
AX-157696662	1	55.618	FI894255.1	214	HORI212 Ictalu	UN
AX-157896055	1	55.837	KV452708.1	3255723	1-scf00008	1
AX-157896067	1	55.837	KV452708.1	3270390	1-scf00008	1
AX-157896112	1	55.837	KV452709.1	33469	1-scf00009	1
AX-157896122	1	55.837	KV452709.1	44938	1-scf00009	1
AX-157896183	1	55.837	KV452709.1	135409	1-scf00009	1
AX-157896185	1	55.837	KV452709.1	139171	1-scf00009	1
AX-157896189	1	55.837	KV452709.1	141734	1-scf00009	1
AX-157896190	1	55.837	KV452709.1	142140	1-scf00009	1
AX-157896193	1	55.837	KV452709.1	145258	1-scf00009	1
AX-157896199	1	55.837	KV452709.1	152351	1-scf00009	1
AX-157896201	1	55.837	KV452709.1	154327	1-scf00009	1
AX-157896208	1	55.837	KV452709.1	166541	1-scf00009	1
AX-157896210	1	55.837	KV452709.1	167904	1-scf00009	1
AX-157810928	1	55.837	KV452709.1	486847	1-scf00009	1
AX-157896540	1	55.837	KV452709.1	572632	1-scf00009	1
AX-157896646	1	55.837	KV452709.1	720365	1-scf00009	1
AX-157810904	1	55.863	KV452709.1	174113	1-scf00009	1
AX-157896225	1	55.917	KV452709.1	184795	1-scf00009	1
AX-157896220	1	55.946	KV452709.1	180960	1-scf00009	1
AX-157896224	1	55.946	KV452709.1	184310	1-scf00009	1
AX-157896226	1	55.946	KV452709.1	185418	1-scf00009	1
AX-157896231	1	55.946	KV452709.1	189372	1-scf00009	1
AX-157896234	1	55.946	KV452709.1	193420	1-scf00009	1
AX-157810905	1	55.946	KV452709.1	195802	1-scf00009	1
AX-157896240	1	55.946	KV452709.1	198197	1-scf00009	1
AX-158388998	1	55.946	KV452709.1	201004	1-scf00009	1
AX-157810907	1	55.946	KV452709.1	202260	1-scf00009	1
AX-157896245	1	55.946	KV452709.1	202872	1-scf00009	1
AX-157896251	1	55.946	KV452709.1	210508	1-scf00009	1
AX-157896260	1	55.946	KV452709.1	218620	1-scf00009	1
AX-157896261	1	55.946	KV452709.1	219716	1-scf00009	1
AX-157896262	1	55.946	KV452709.1	220252	1-scf00009	1
AX-157810909	1	55.946	KV452709.1	220953	1-scf00009	1
AX-157896265	1	55.946	KV452709.1	223500	1-scf00009	1
AX-157896267	1	55.946	KV452709.1	226553	1-scf00009	1
AX-157810912	1	55.946	KV452709.1	228733	1-scf00009	1
AX-157748844	1	55.946	KV452709.1	228920	1-scf00009	1
AX-157896273	1	55.946	KV452709.1	234383	1-scf00009	1
AX-157810913	1	55.946	KV452709.1	234885	1-scf00009	1
AX-157896274	1	55.946	KV452709.1	235687	1-scf00009	1
AX-157810914	1	55.946	KV452709.1	237684	1-scf00009	1
AX-157894856	1	56.055	KV452708.1	1475430	1-scf00008	1
AX-157894857	1	56.055	KV452708.1	1475828	1-scf00008	1
AX-157895114	1	56.055	KV452708.1	1866451	1-scf00008	1
AX-157810851	1	56.055	KV452708.1	2690214	1-scf00008	1
AX-157895579	1	56.055	KV452708.1	2723893	1-scf00008	1
AX-157895596	1	56.055	KV452708.1	2740689	1-scf00008	1
AX-157895619	1	56.055	KV452708.1	2761959	1-scf00008	1
AX-157810886	1	56.055	KV452708.1	3259584	1-scf00008	1

AX-157896153	1	56.055	KV452709.1	90215	1-scf00009	1
AX-157896182	1	56.055	KV452709.1	134610	1-scf00009	1
AX-157896186	1	56.055	KV452709.1	140257	1-scf00009	1
AX-157896192	1	56.055	KV452709.1	143373	1-scf00009	1
AX-157896198	1	56.055	KV452709.1	151430	1-scf00009	1
AX-157896217	1	56.055	KV452709.1	178498	1-scf00009	1
AX-157896228	1	56.055	KV452709.1	186496	1-scf00009	1
AX-157896233	1	56.055	KV452709.1	191522	1-scf00009	1
AX-157896235	1	56.055	KV452709.1	194185	1-scf00009	1
AX-158379132	1	56.055	KV452709.1	199540	1-scf00009	1
AX-157896246	1	56.055	KV452709.1	203120	1-scf00009	1
AX-157896247	1	56.055	KV452709.1	203390	1-scf00009	1
AX-157896255	1	56.055	KV452709.1	215222	1-scf00009	1
AX-157810911	1	56.055	KV452709.1	228464	1-scf00009	1
AX-157748252	1	56.055	KV452709.1	239469	1-scf00009	1
AX-157896276	1	56.055	KV452709.1	240692	1-scf00009	1
AX-157896277	1	56.055	KV452709.1	241384	1-scf00009	1
AX-157896284	1	56.055	KV452709.1	258857	1-scf00009	1
AX-157896287	1	56.055	KV452709.1	263094	1-scf00009	1
AX-157896289	1	56.055	KV452709.1	264727	1-scf00009	1
AX-157896291	1	56.055	KV452709.1	266720	1-scf00009	1
AX-157868510	1	56.055	KV452709.1	270683	1-scf00009	1
AX-157896294	1	56.055	KV452709.1	270894	1-scf00009	1
AX-157745538	1	56.055	KV452709.1	287878	1-scf00009	1
AX-157896303	1	56.055	KV452709.1	288848	1-scf00009	1
AX-157868511	1	56.055	KV452709.1	292895	1-scf00009	1
AX-157810917	1	56.055	KV452709.1	293404	1-scf00009	1
AX-157896306	1	56.055	KV452709.1	293846	1-scf00009	1
AX-157896311	1	56.055	KV452709.1	303085	1-scf00009	1
AX-157896315	1	56.055	KV452709.1	307399	1-scf00009	1
AX-157896318	1	56.055	KV452709.1	309792	1-scf00009	1
AX-157896320	1	56.055	KV452709.1	311479	1-scf00009	1
AX-157896325	1	56.055	KV452709.1	316836	1-scf00009	1
AX-157896326	1	56.055	KV452709.1	317269	1-scf00009	1
AX-157722891	1	56.055	KV452709.1	318824	1-scf00009	1
AX-157896329	1	56.055	KV452709.1	319035	1-scf00009	1
AX-157896330	1	56.055	KV452709.1	319574	1-scf00009	1
AX-157896335	1	56.055	KV452709.1	326933	1-scf00009	1
AX-157896338	1	56.055	KV452709.1	332311	1-scf00009	1
AX-157896339	1	56.055	KV452709.1	334433	1-scf00009	1
AX-157896340	1	56.055	KV452709.1	336222	1-scf00009	1
AX-157896342	1	56.055	KV452709.1	337975	1-scf00009	1
AX-157853567	1	56.055	KV452709.1	338756	1-scf00009	1
AX-157896343	1	56.055	KV452709.1	339181	1-scf00009	1
AX-157896344	1	56.055	KV452709.1	340775	1-scf00009	1
AX-157896345	1	56.055	KV452709.1	341873	1-scf00009	1
AX-157896348	1	56.055	KV452709.1	348765	1-scf00009	1
AX-158388999	1	56.055	KV452709.1	352098	1-scf00009	1
AX-157896352	1	56.055	KV452709.1	352850	1-scf00009	1
AX-157896357	1	56.055	KV452709.1	356467	1-scf00009	1
AX-158421783	1	56.055	KV452709.1	357596	1-scf00009	1

AX-157896358	1	56.055	KV452709.1	358051	1-scf00009	1
AX-157896359	1	56.055	KV452709.1	359147	1-scf00009	1
AX-157896360	1	56.055	KV452709.1	360185	1-scf00009	1
AX-157896362	1	56.055	KV452709.1	363107	1-scf00009	1
AX-157896363	1	56.055	KV452709.1	363232	1-scf00009	1
AX-157896365	1	56.055	KV452709.1	364317	1-scf00009	1
AX-157896366	1	56.055	KV452709.1	364801	1-scf00009	1
AX-157896367	1	56.055	KV452709.1	365269	1-scf00009	1
AX-157896368	1	56.055	KV452709.1	366254	1-scf00009	1
AX-157896370	1	56.055	KV452709.1	367152	1-scf00009	1
AX-157701266	1	56.055	KV452709.1	367743	1-scf00009	1
AX-157896373	1	56.055	KV452709.1	372214	1-scf00009	1
AX-157696657	1	56.055	KV452709.1	372869	1-scf00009	1
AX-157896374	1	56.055	KV452709.1	373461	1-scf00009	1
AX-158389000	1	56.055	KV452709.1	376385	1-scf00009	1
AX-157896377	1	56.055	KV452709.1	377332	1-scf00009	1
AX-157896378	1	56.055	KV452709.1	377926	1-scf00009	1
AX-157896379	1	56.055	KV452709.1	378896	1-scf00009	1
AX-157896381	1	56.055	KV452709.1	380607	1-scf00009	1
AX-157896382	1	56.055	KV452709.1	381300	1-scf00009	1
AX-157896383	1	56.055	KV452709.1	382778	1-scf00009	1
AX-157896384	1	56.055	KV452709.1	383495	1-scf00009	1
AX-157896385	1	56.055	KV452709.1	384736	1-scf00009	1
AX-157896386	1	56.055	KV452709.1	386230	1-scf00009	1
AX-157896387	1	56.055	KV452709.1	386978	1-scf00009	1
AX-157896388	1	56.055	KV452709.1	387620	1-scf00009	1
AX-157896389	1	56.055	KV452709.1	387895	1-scf00009	1
AX-157896390	1	56.055	KV452709.1	388704	1-scf00009	1
AX-157896392	1	56.055	KV452709.1	390151	1-scf00009	1
AX-157896393	1	56.055	KV452709.1	390837	1-scf00009	1
AX-157896394	1	56.055	KV452709.1	391839	1-scf00009	1
AX-157896395	1	56.055	KV452709.1	392781	1-scf00009	1
AX-157896396	1	56.055	KV452709.1	393700	1-scf00009	1
AX-157896397	1	56.055	KV452709.1	393992	1-scf00009	1
AX-157896398	1	56.055	KV452709.1	396348	1-scf00009	1
AX-157810920	1	56.055	KV452709.1	396486	1-scf00009	1
AX-157896399	1	56.055	KV452709.1	397922	1-scf00009	1
AX-157722210	1	56.055	KV452709.1	399636	1-scf00009	1
AX-157896400	1	56.055	KV452709.1	400481	1-scf00009	1
AX-157896401	1	56.055	KV452709.1	401141	1-scf00009	1
AX-157718610	1	56.055	KV452709.1	402276	1-scf00009	1
AX-157896404	1	56.055	KV452709.1	404726	1-scf00009	1
AX-157896408	1	56.055	KV452709.1	407926	1-scf00009	1
AX-157896409	1	56.055	KV452709.1	409058	1-scf00009	1
AX-157896410	1	56.055	KV452709.1	410564	1-scf00009	1
AX-157896412	1	56.055	KV452709.1	414344	1-scf00009	1
AX-157896417	1	56.055	KV452709.1	419383	1-scf00009	1
AX-157896418	1	56.055	KV452709.1	420542	1-scf00009	1
AX-157896422	1	56.055	KV452709.1	426499	1-scf00009	1
AX-157896423	1	56.055	KV452709.1	427480	1-scf00009	1
AX-157896425	1	56.055	KV452709.1	428044	1-scf00009	1

AX-158404380	1	56.055	KV452709.1	435434	1-scf00009	1
AX-157896430	1	56.055	KV452709.1	439762	1-scf00009	1
AX-157896431	1	56.055	KV452709.1	440068	1-scf00009	1
AX-157810922	1	56.055	KV452709.1	440705	1-scf00009	1
AX-157810923	1	56.055	KV452709.1	443522	1-scf00009	1
AX-157896488	1	56.055	KV452709.1	507381	1-scf00009	1
AX-157896496	1	56.055	KV452709.1	518997	1-scf00009	1
AX-157896502	1	56.055	KV452709.1	532005	1-scf00009	1
AX-157896624	1	56.055	KV452709.1	691488	1-scf00009	1
AX-157749792	1	56.055	KV452709.1	729953	1-scf00009	1
AX-157896663	1	56.055	KV452709.1	740750	1-scf00009	1
AX-157810997	1	56.055	KV452709.1	1510942	1-scf00009	1
AX-157897479	1	56.055	KV452709.1	1945026	1-scf00009	1
AX-158404375	1	56.164	KV452709.1	76045	1-scf00009	1
AX-157896242	1	56.164	KV452709.1	199405	1-scf00009	1
AX-157896252	1	56.164	KV452709.1	210826	1-scf00009	1
AX-157896253	1	56.164	KV452709.1	211581	1-scf00009	1
AX-157896269	1	56.164	KV452709.1	230930	1-scf00009	1
AX-157896270	1	56.164	KV452709.1	231079	1-scf00009	1
AX-157896271	1	56.164	KV452709.1	232073	1-scf00009	1
AX-157896278	1	56.164	KV452709.1	242897	1-scf00009	1
AX-157896349	1	56.164	KV452709.1	349156	1-scf00009	1
AX-157896351	1	56.164	KV452709.1	351633	1-scf00009	1
AX-157896355	1	56.164	KV452709.1	354812	1-scf00009	1
AX-157896364	1	56.164	KV452709.1	364277	1-scf00009	1
AX-157896402	1	56.164	KV452709.1	401806	1-scf00009	1
AX-157896403	1	56.164	KV452709.1	403737	1-scf00009	1
AX-157896411	1	56.164	KV452709.1	412068	1-scf00009	1
AX-157810921	1	56.164	KV452709.1	433183	1-scf00009	1
AX-157810924	1	56.164	KV452709.1	444063	1-scf00009	1
AX-157896434	1	56.164	KV452709.1	444631	1-scf00009	1
AX-157896435	1	56.164	KV452709.1	444946	1-scf00009	1
AX-157896436	1	56.164	KV452709.1	446359	1-scf00009	1
AX-157896439	1	56.164	KV452709.1	449920	1-scf00009	1
AX-157896440	1	56.164	KV452709.1	450894	1-scf00009	1
AX-157896441	1	56.164	KV452709.1	451823	1-scf00009	1
AX-157896443	1	56.164	KV452709.1	454761	1-scf00009	1
AX-157896444	1	56.164	KV452709.1	455473	1-scf00009	1
AX-157896446	1	56.164	KV452709.1	458054	1-scf00009	1
AX-157896447	1	56.164	KV452709.1	458465	1-scf00009	1
AX-157896451	1	56.164	KV452709.1	461150	1-scf00009	1
AX-157853570	1	56.164	KV452709.1	463233	1-scf00009	1
AX-157896453	1	56.164	KV452709.1	463990	1-scf00009	1
AX-157896455	1	56.164	KV452709.1	467937	1-scf00009	1
AX-157896458	1	56.164	KV452709.1	470218	1-scf00009	1
AX-157896459	1	56.164	KV452709.1	470967	1-scf00009	1
AX-157896461	1	56.164	KV452709.1	473523	1-scf00009	1
AX-157896463	1	56.164	KV452709.1	475208	1-scf00009	1
AX-157896464	1	56.164	KV452709.1	476103	1-scf00009	1
AX-157896466	1	56.164	KV452709.1	476824	1-scf00009	1
AX-157726663	1	56.164	KV452709.1	480886	1-scf00009	1

AX-157896470	1	56.164	KV452709.1	481369	1-scf00009	1
AX-157896472	1	56.164	KV452709.1	482922	1-scf00009	1
AX-157896473	1	56.164	KV452709.1	483745	1-scf00009	1
AX-157896474	1	56.164	KV452709.1	485000	1-scf00009	1
AX-157748632	1	56.164	KV452709.1	485711	1-scf00009	1
AX-157896475	1	56.164	KV452709.1	487274	1-scf00009	1
AX-157896477	1	56.164	KV452709.1	492787	1-scf00009	1
AX-157810929	1	56.164	KV452709.1	492823	1-scf00009	1
AX-157896479	1	56.164	KV452709.1	495511	1-scf00009	1
AX-158404381	1	56.164	KV452709.1	496119	1-scf00009	1
AX-158404382	1	56.164	KV452709.1	496280	1-scf00009	1
AX-157896480	1	56.164	KV452709.1	496846	1-scf00009	1
AX-157896481	1	56.164	KV452709.1	497732	1-scf00009	1
AX-157896482	1	56.164	KV452709.1	498016	1-scf00009	1
AX-157896485	1	56.164	KV452709.1	504129	1-scf00009	1
AX-157896486	1	56.164	KV452709.1	505886	1-scf00009	1
AX-157896491	1	56.164	KV452709.1	512843	1-scf00009	1
AX-157896493	1	56.164	KV452709.1	515844	1-scf00009	1
AX-157810931	1	56.164	KV452709.1	516324	1-scf00009	1
AX-157896494	1	56.164	KV452709.1	517801	1-scf00009	1
AX-157896495	1	56.164	KV452709.1	518388	1-scf00009	1
AX-157896501	1	56.164	KV452709.1	531349	1-scf00009	1
AX-157896504	1	56.164	KV452709.1	535256	1-scf00009	1
AX-157896505	1	56.164	KV452709.1	538326	1-scf00009	1
AX-157896508	1	56.164	KV452709.1	542552	1-scf00009	1
AX-157896509	1	56.164	KV452709.1	544696	1-scf00009	1
AX-157896510	1	56.164	KV452709.1	545105	1-scf00009	1
AX-157896511	1	56.164	KV452709.1	545470	1-scf00009	1
AX-157896513	1	56.164	KV452709.1	547763	1-scf00009	1
AX-157896517	1	56.164	KV452709.1	552610	1-scf00009	1
AX-157896521	1	56.164	KV452709.1	555148	1-scf00009	1
AX-157896522	1	56.164	KV452709.1	555372	1-scf00009	1
AX-157700820	1	56.164	KV452709.1	557700	1-scf00009	1
AX-157896526	1	56.164	KV452709.1	557882	1-scf00009	1
AX-157896528	1	56.164	KV452709.1	559515	1-scf00009	1
AX-157896529	1	56.164	KV452709.1	560352	1-scf00009	1
AX-157896530	1	56.164	KV452709.1	560766	1-scf00009	1
AX-157896532	1	56.164	KV452709.1	562121	1-scf00009	1
AX-157896533	1	56.164	KV452709.1	562333	1-scf00009	1
AX-157758456	1	56.164	KV452709.1	562448	1-scf00009	1
AX-157896536	1	56.164	KV452709.1	568305	1-scf00009	1
AX-157896539	1	56.164	KV452709.1	571621	1-scf00009	1
AX-157896541	1	56.164	KV452709.1	573150	1-scf00009	1
AX-157896543	1	56.164	KV452709.1	575532	1-scf00009	1
AX-157896544	1	56.164	KV452709.1	576220	1-scf00009	1
AX-157896545	1	56.164	KV452709.1	578545	1-scf00009	1
AX-157896547	1	56.164	KV452709.1	579966	1-scf00009	1
AX-157896548	1	56.164	KV452709.1	580670	1-scf00009	1
AX-157896549	1	56.164	KV452709.1	581678	1-scf00009	1
AX-157896551	1	56.164	KV452709.1	584299	1-scf00009	1
AX-157896553	1	56.164	KV452709.1	585906	1-scf00009	1

AX-157896554	1	56.164	KV452709.1	586381	1-scf00009	1
AX-157896555	1	56.164	KV452709.1	588474	1-scf00009	1
AX-157896557	1	56.164	KV452709.1	591669	1-scf00009	1
AX-157896558	1	56.164	KV452709.1	592362	1-scf00009	1
AX-157896560	1	56.164	KV452709.1	597876	1-scf00009	1
AX-157896563	1	56.164	KV452709.1	602412	1-scf00009	1
AX-157896564	1	56.164	KV452709.1	603533	1-scf00009	1
AX-157896565	1	56.164	KV452709.1	604405	1-scf00009	1
AX-157896566	1	56.164	KV452709.1	605419	1-scf00009	1
AX-157896567	1	56.164	KV452709.1	607692	1-scf00009	1
AX-157896569	1	56.164	KV452709.1	608895	1-scf00009	1
AX-157896570	1	56.164	KV452709.1	609290	1-scf00009	1
AX-157896571	1	56.164	KV452709.1	609786	1-scf00009	1
AX-157896572	1	56.164	KV452709.1	610801	1-scf00009	1
AX-157896573	1	56.164	KV452709.1	612564	1-scf00009	1
AX-157896578	1	56.164	KV452709.1	623135	1-scf00009	1
AX-157896579	1	56.164	KV452709.1	624036	1-scf00009	1
AX-157896580	1	56.164	KV452709.1	624886	1-scf00009	1
AX-157896581	1	56.164	KV452709.1	625566	1-scf00009	1
AX-157896582	1	56.164	KV452709.1	628511	1-scf00009	1
AX-158404383	1	56.164	KV452709.1	634293	1-scf00009	1
AX-157896585	1	56.164	KV452709.1	634840	1-scf00009	1
AX-157695711	1	56.164	KV452709.1	635772	1-scf00009	1
AX-157695712	1	56.164	KV452709.1	636195	1-scf00009	1
AX-158389003	1	56.164	KV452709.1	636637	1-scf00009	1
AX-157896586	1	56.164	KV452709.1	636723	1-scf00009	1
AX-157896588	1	56.164	KV452709.1	638014	1-scf00009	1
AX-157896589	1	56.164	KV452709.1	638502	1-scf00009	1
AX-157896590	1	56.164	KV452709.1	639197	1-scf00009	1
AX-157896591	1	56.164	KV452709.1	640206	1-scf00009	1
AX-157896592	1	56.164	KV452709.1	640586	1-scf00009	1
AX-158389004	1	56.164	KV452709.1	643729	1-scf00009	1
AX-157896593	1	56.164	KV452709.1	644276	1-scf00009	1
AX-157896595	1	56.164	KV452709.1	645324	1-scf00009	1
AX-157896596	1	56.164	KV452709.1	645390	1-scf00009	1
AX-157853571	1	56.164	KV452709.1	650770	1-scf00009	1
AX-157896598	1	56.164	KV452709.1	652521	1-scf00009	1
AX-157896601	1	56.164	KV452709.1	656344	1-scf00009	1
AX-157896602	1	56.164	KV452709.1	657238	1-scf00009	1
AX-157896603	1	56.164	KV452709.1	660454	1-scf00009	1
AX-157896604	1	56.164	KV452709.1	661289	1-scf00009	1
AX-157896605	1	56.164	KV452709.1	662292	1-scf00009	1
AX-157896608	1	56.164	KV452709.1	665593	1-scf00009	1
AX-157896610	1	56.164	KV452709.1	667989	1-scf00009	1
AX-157896614	1	56.164	KV452709.1	676251	1-scf00009	1
AX-157896615	1	56.164	KV452709.1	676761	1-scf00009	1
AX-157896617	1	56.164	KV452709.1	679389	1-scf00009	1
AX-157810933	1	56.164	KV452709.1	679530	1-scf00009	1
AX-157896619	1	56.164	KV452709.1	681311	1-scf00009	1
AX-157896620	1	56.164	KV452709.1	682220	1-scf00009	1
AX-157810934	1	56.164	KV452709.1	684612	1-scf00009	1

AX-157896621	1	56.164	KV452709.1	685157	1-scf00009	1
AX-157896622	1	56.164	KV452709.1	687117	1-scf00009	1
AX-157896623	1	56.164	KV452709.1	687492	1-scf00009	1
AX-157896625	1	56.164	KV452709.1	691956	1-scf00009	1
AX-157896627	1	56.164	KV452709.1	693394	1-scf00009	1
AX-158379134	1	56.164	KV452709.1	693839	1-scf00009	1
AX-157896628	1	56.164	KV452709.1	695509	1-scf00009	1
AX-157896629	1	56.164	KV452709.1	697127	1-scf00009	1
AX-157896631	1	56.164	KV452709.1	699438	1-scf00009	1
AX-157896632	1	56.164	KV452709.1	699712	1-scf00009	1
AX-157896633	1	56.164	KV452709.1	700979	1-scf00009	1
AX-157896637	1	56.164	KV452709.1	710288	1-scf00009	1
AX-157896638	1	56.164	KV452709.1	710836	1-scf00009	1
AX-157756320	1	56.164	KV452709.1	711260	1-scf00009	1
AX-157896642	1	56.164	KV452709.1	714436	1-scf00009	1
AX-157896643	1	56.164	KV452709.1	715708	1-scf00009	1
AX-158404384	1	56.164	KV452709.1	716204	1-scf00009	1
AX-157883093	1	56.164	KV452709.1	717501	1-scf00009	1
AX-157896644	1	56.164	KV452709.1	717693	1-scf00009	1
AX-157810935	1	56.164	KV452709.1	719648	1-scf00009	1
AX-157896645	1	56.164	KV452709.1	719957	1-scf00009	1
AX-157896647	1	56.164	KV452709.1	721244	1-scf00009	1
AX-157896648	1	56.164	KV452709.1	721984	1-scf00009	1
AX-157896651	1	56.164	KV452709.1	723966	1-scf00009	1
AX-157896652	1	56.164	KV452709.1	724811	1-scf00009	1
AX-157896653	1	56.164	KV452709.1	725801	1-scf00009	1
AX-157896654	1	56.164	KV452709.1	727261	1-scf00009	1
AX-157896656	1	56.164	KV452709.1	729082	1-scf00009	1
AX-157896659	1	56.164	KV452709.1	732604	1-scf00009	1
AX-157896660	1	56.164	KV452709.1	735752	1-scf00009	1
AX-157896683	1	56.164	KV452709.1	764141	1-scf00009	1
AX-157896694	1	56.164	KV452709.1	773319	1-scf00009	1
AX-157810937	1	56.164	KV452709.1	775264	1-scf00009	1
AX-157743619	1	56.164	KV452709.1	819260	1-scf00009	1
AX-157883095	1	56.164	KV452709.1	892469	1-scf00009	1
AX-157896774	1	56.164	KV452709.1	895867	1-scf00009	1
AX-157896776	1	56.164	KV452709.1	901317	1-scf00009	1
AX-157810955	1	56.164	KV452709.1	901368	1-scf00009	1
AX-157896783	1	56.164	KV452709.1	912669	1-scf00009	1
AX-157897190	1	56.164	KV452709.1	1518957	1-scf00009	1
AX-157899014	1	56.164	KV452709.1	4556640	1-scf00009	1
AX-157811226	1	56.164	KV452709.1	4596761	1-scf00009	1
AX-157899123	1	56.164	KV452709.1	4694983	1-scf00009	1
AX-157810925	1	56.273	KV452709.1	459755	1-scf00009	1
AX-157810926	1	56.273	KV452709.1	459804	1-scf00009	1
AX-157896546	1	56.273	KV452709.1	579363	1-scf00009	1
AX-157896550	1	56.273	KV452709.1	583043	1-scf00009	1
AX-157896556	1	56.273	KV452709.1	590968	1-scf00009	1
AX-157896618	1	56.273	KV452709.1	680966	1-scf00009	1
AX-157896664	1	56.273	KV452709.1	741163	1-scf00009	1
AX-157726294	1	56.273	KV452709.1	748196	1-scf00009	1

AX-157896672	1	56.273	KV452709.1	752630	1-scf00009	1
AX-157896673	1	56.273	KV452709.1	753161	1-scf00009	1
AX-157896674	1	56.273	KV452709.1	754097	1-scf00009	1
AX-157896676	1	56.273	KV452709.1	756790	1-scf00009	1
AX-157896679	1	56.273	KV452709.1	758240	1-scf00009	1
AX-157810936	1	56.273	KV452709.1	759860	1-scf00009	1
AX-157896681	1	56.273	KV452709.1	760214	1-scf00009	1
AX-157864375	1	56.273	KV452709.1	762290	1-scf00009	1
AX-157896682	1	56.273	KV452709.1	763912	1-scf00009	1
AX-157896684	1	56.273	KV452709.1	764401	1-scf00009	1
AX-157896685	1	56.273	KV452709.1	764836	1-scf00009	1
AX-157896686	1	56.273	KV452709.1	768388	1-scf00009	1
AX-157896687	1	56.273	KV452709.1	769004	1-scf00009	1
AX-157896689	1	56.273	KV452709.1	770633	1-scf00009	1
AX-157896693	1	56.273	KV452709.1	772861	1-scf00009	1
AX-157896696	1	56.273	KV452709.1	776674	1-scf00009	1
AX-157853572	1	56.273	KV452709.1	776992	1-scf00009	1
AX-157896697	1	56.273	KV452709.1	777115	1-scf00009	1
AX-157896700	1	56.273	KV452709.1	781750	1-scf00009	1
AX-157896703	1	56.273	KV452709.1	783444	1-scf00009	1
AX-157896723	1	56.273	KV452709.1	807976	1-scf00009	1
AX-157894675	1	56.383	KV452708.1	1139062	1-scf00008	1
AX-158388997	1	56.383	KV452709.1	140669	1-scf00009	1
AX-157896195	1	56.383	KV452709.1	148040	1-scf00009	1
AX-157896670	1	56.383	KV452709.1	749928	1-scf00009	1
AX-157896698	1	56.383	KV452709.1	778016	1-scf00009	1
AX-157896704	1	56.383	KV452709.1	783930	1-scf00009	1
AX-157896705	1	56.383	KV452709.1	784357	1-scf00009	1
AX-157896706	1	56.383	KV452709.1	785038	1-scf00009	1
AX-157896708	1	56.383	KV452709.1	786302	1-scf00009	1
AX-157752575	1	56.383	KV452709.1	787266	1-scf00009	1
AX-157896712	1	56.383	KV452709.1	789852	1-scf00009	1
AX-157810938	1	56.383	KV452709.1	790341	1-scf00009	1
AX-157896715	1	56.383	KV452709.1	793183	1-scf00009	1
AX-157896716	1	56.383	KV452709.1	795014	1-scf00009	1
AX-157896718	1	56.383	KV452709.1	796364	1-scf00009	1
AX-157810939	1	56.383	KV452709.1	797429	1-scf00009	1
AX-157896719	1	56.383	KV452709.1	798085	1-scf00009	1
AX-157864376	1	56.383	KV452709.1	805058	1-scf00009	1
AX-157810943	1	56.383	KV452709.1	808813	1-scf00009	1
AX-157896726	1	56.383	KV452709.1	813882	1-scf00009	1
AX-157874333	1	56.383	KV452709.1	820253	1-scf00009	1
AX-157747623	1	56.383	KV452709.1	820473	1-scf00009	1
AX-157896731	1	56.383	KV452709.1	822871	1-scf00009	1
AX-157896733	1	56.383	KV452709.1	824982	1-scf00009	1
AX-157896739	1	56.383	KV452709.1	836172	1-scf00009	1
AX-157896741	1	56.383	KV452709.1	840415	1-scf00009	1
AX-157896742	1	56.383	KV452709.1	840504	1-scf00009	1
AX-157896743	1	56.383	KV452709.1	842188	1-scf00009	1
AX-157896744	1	56.383	KV452709.1	844327	1-scf00009	1
AX-157896745	1	56.383	KV452709.1	845007	1-scf00009	1

AX-157896747	1	56.383	KV452709.1	848525	1-scf00009	1
AX-157810946	1	56.383	KV452709.1	849718	1-scf00009	1
AX-157896750	1	56.383	KV452709.1	855558	1-scf00009	1
AX-157810949	1	56.383	KV452709.1	858493	1-scf00009	1
AX-157810950	1	56.383	KV452709.1	858545	1-scf00009	1
AX-157896752	1	56.383	KV452709.1	858701	1-scf00009	1
AX-157896769	1	56.383	KV452709.1	885668	1-scf00009	1
AX-157810956	1	56.383	KV452709.1	907812	1-scf00009	1
AX-157700938	1	56.383	KV452709.1	938266	1-scf00009	1
AX-157896810	1	56.383	KV452709.1	954646	1-scf00009	1
AX-157896811	1	56.383	KV452709.1	958265	1-scf00009	1
AX-157896812	1	56.383	KV452709.1	961490	1-scf00009	1
AX-157896814	1	56.383	KV452709.1	963752	1-scf00009	1
AX-157896816	1	56.383	KV452709.1	966433	1-scf00009	1
AX-158379135	1	56.383	KV452709.1	968440	1-scf00009	1
AX-157745592	1	56.383	KV452709.1	980000	1-scf00009	1
AX-157896843	1	56.383	KV452709.1	1007765	1-scf00009	1
AX-157897235	1	56.383	KV452709.1	1577370	1-scf00009	1
AX-157899275	1	56.383	KV452709.1	4957509	1-scf00009	1
AX-157895672	1	56.447	KV452708.1	2823776	1-scf00008	1
AX-157896232	1	56.447	KV452709.1	189566	1-scf00009	1
AX-157810945	1	56.447	KV452709.1	845730	1-scf00009	1
AX-158404688	1	56.447	KV452713.1	2856576	1-scf00011	1
AX-157896748	1	56.495	KV452709.1	852770	1-scf00009	1
AX-157896751	1	56.534	KV452709.1	857818	1-scf00009	1
AX-157724480	1	56.534	KV452709.1	908007	1-scf00009	1
AX-157810861	1	56.601	KV452708.1	2823883	1-scf00008	1
AX-157895687	1	56.601	KV452708.1	2854424	1-scf00008	1
AX-157896753	1	56.601	KV452709.1	861669	1-scf00009	1
AX-157896755	1	56.601	KV452709.1	863495	1-scf00009	1
AX-157896758	1	56.601	KV452709.1	867743	1-scf00009	1
AX-157896760	1	56.601	KV452709.1	869737	1-scf00009	1
AX-157896764	1	56.601	KV452709.1	874476	1-scf00009	1
AX-157896765	1	56.601	KV452709.1	874978	1-scf00009	1
AX-157717623	1	56.601	KV452709.1	875583	1-scf00009	1
AX-157810953	1	56.601	KV452709.1	876270	1-scf00009	1
AX-157716527	1	56.601	KV452709.1	876450	1-scf00009	1
AX-157896766	1	56.601	KV452709.1	879533	1-scf00009	1
AX-157896767	1	56.601	KV452709.1	879911	1-scf00009	1
AX-157746774	1	56.601	KV452709.1	880679	1-scf00009	1
AX-157896771	1	56.601	KV452709.1	891089	1-scf00009	1
AX-157896781	1	56.601	KV452709.1	910984	1-scf00009	1
AX-157896782	1	56.601	KV452709.1	912351	1-scf00009	1
AX-157896785	1	56.601	KV452709.1	914842	1-scf00009	1
AX-157739236	1	56.601	KV452709.1	916842	1-scf00009	1
AX-157896786	1	56.601	KV452709.1	917730	1-scf00009	1
AX-157726435	1	56.601	KV452709.1	920820	1-scf00009	1
AX-157810959	1	56.601	KV452709.1	921534	1-scf00009	1
AX-157896794	1	56.601	KV452709.1	930841	1-scf00009	1
AX-157896800	1	56.601	KV452709.1	940104	1-scf00009	1
AX-157896826	1	56.601	KV452709.1	980942	1-scf00009	1

AX-157897642	1	56.601	KV452709.1	2202899	1-scf00009	1
AX-157897710	1	56.601	KV452709.1	2315747	1-scf00009	1
AX-158379144	1	56.601	KV452709.1	4556952	1-scf00009	1
AX-157810961	1	56.819	KV452709.1	990300	1-scf00009	1
AX-157896831	1	56.819	KV452709.1	992281	1-scf00009	1
AX-157896832	1	56.819	KV452709.1	992777	1-scf00009	1
AX-157896834	1	56.819	KV452709.1	995127	1-scf00009	1
AX-157896835	1	56.819	KV452709.1	995698	1-scf00009	1
AX-157896836	1	56.819	KV452709.1	998879	1-scf00009	1
AX-157720282	1	56.819	KV452709.1	999515	1-scf00009	1
AX-157896838	1	56.819	KV452709.1	1001164	1-scf00009	1
AX-157896893	1	56.819	KV452709.1	1062112	1-scf00009	1
AX-157737484	1	56.819	KV452709.1	1062338	1-scf00009	1
AX-157896662	1	56.82	KV452709.1	739948	1-scf00009	1
AX-157896668	1	56.82	KV452709.1	747982	1-scf00009	1
AX-157896772	1	56.82	KV452709.1	891786	1-scf00009	1
AX-157896841	1	56.82	KV452709.1	1004536	1-scf00009	1
AX-157896845	1	56.82	KV452709.1	1013156	1-scf00009	1
AX-157896859	1	56.82	KV452709.1	1026598	1-scf00009	1
AX-157810963	1	56.881	KV452709.1	1041468	1-scf00009	1
AX-157896878	1	56.938	KV452709.1	1045482	1-scf00009	1
AX-158404461	1	56.938	KV452709.1	3852443	1-scf00009	1
AX-157896881	1	56.993	KV452709.1	1048209	1-scf00009	1
AX-157896885	1	56.993	KV452709.1	1053020	1-scf00009	1
AX-157896884	1	57.044	KV452709.1	1052837	1-scf00009	1
AX-157896322	1	57.091	KV452709.1	314430	1-scf00009	1
AX-157896833	1	57.091	KV452709.1	993197	1-scf00009	1
AX-157810962	1	57.091	KV452709.1	1015826	1-scf00009	1
AX-157896882	1	57.091	KV452709.1	1049625	1-scf00009	1
AX-157896890	1	57.091	KV452709.1	1058070	1-scf00009	1
AX-157896902	1	57.091	KV452709.1	1070293	1-scf00009	1
AX-157743881	1	57.091	KV452709.1	5699551	1-scf00009	1
AX-157883080	1	57.366	KV452708.1	828979	1-scf00008	1
AX-157894782	1	57.366	KV452708.1	1347033	1-scf00008	1
AX-157894786	1	57.366	KV452708.1	1350937	1-scf00008	1
AX-157894797	1	57.366	KV452708.1	1372533	1-scf00008	1
AX-157895725	1	57.366	KV452708.1	2895300	1-scf00008	1
AX-158404360	1	57.366	KV452708.1	3016404	1-scf00008	1
AX-157896432	1	57.366	KV452709.1	441089	1-scf00009	1
AX-157896534	1	57.366	KV452709.1	563163	1-scf00009	1
AX-157896815	1	57.366	KV452709.1	966139	1-scf00009	1
AX-157896823	1	57.366	KV452709.1	974663	1-scf00009	1
AX-157774979	1	57.366	KV452709.1	1052291	1-scf00009	1
AX-157810964	1	57.366	KV452709.1	1067751	1-scf00009	1
AX-157896916	1	57.366	KV452709.1	1095500	1-scf00009	1
AX-157810968	1	57.366	KV452709.1	1096021	1-scf00009	1
AX-157810969	1	57.366	KV452709.1	1097128	1-scf00009	1
AX-157748330	1	57.366	KV452709.1	1097649	1-scf00009	1
AX-157725082	1	57.366	KV452709.1	1141665	1-scf00009	1
AX-158404393	1	57.366	KV452709.1	1144579	1-scf00009	1
AX-157896972	1	57.366	KV452709.1	1181239	1-scf00009	1

AX-157896984	1	57.366	KV452709.1	1203272	1-scf00009	1
AX-157896985	1	57.366	KV452709.1	1205252	1-scf00009	1
AX-157897007	1	57.366	KV452709.1	1259347	1-scf00009	1
AX-158404402	1	57.366	KV452709.1	1278814	1-scf00009	1
AX-157700928	1	57.366	KV452709.1	1286277	1-scf00009	1
AX-157897021	1	57.366	KV452709.1	1287302	1-scf00009	1
AX-157897022	1	57.366	KV452709.1	1290651	1-scf00009	1
AX-157897023	1	57.366	KV452709.1	1291069	1-scf00009	1
AX-157868516	1	57.366	KV452709.1	1294180	1-scf00009	1
AX-157897045	1	57.366	KV452709.1	1329153	1-scf00009	1
AX-157745452	1	57.366	KV452709.1	1330330	1-scf00009	1
AX-157897058	1	57.366	KV452709.1	1350591	1-scf00009	1
AX-157897059	1	57.366	KV452709.1	1350872	1-scf00009	1
AX-157897060	1	57.366	KV452709.1	1351433	1-scf00009	1
AX-157897064	1	57.366	KV452709.1	1357588	1-scf00009	1
AX-157740004	1	57.366	KV452709.1	1366889	1-scf00009	1
AX-157810984	1	57.366	KV452709.1	1367144	1-scf00009	1
AX-157897075	1	57.366	KV452709.1	1369330	1-scf00009	1
AX-157810985	1	57.366	KV452709.1	1370747	1-scf00009	1
AX-157897086	1	57.366	KV452709.1	1375824	1-scf00009	1
AX-157897091	1	57.366	KV452709.1	1385037	1-scf00009	1
AX-157897093	1	57.366	KV452709.1	1386524	1-scf00009	1
AX-157810987	1	57.366	KV452709.1	1387593	1-scf00009	1
AX-157810988	1	57.366	KV452709.1	1394930	1-scf00009	1
AX-157810989	1	57.366	KV452709.1	1396964	1-scf00009	1
AX-157897100	1	57.366	KV452709.1	1406897	1-scf00009	1
AX-157897102	1	57.366	KV452709.1	1407980	1-scf00009	1
AX-157897103	1	57.366	KV452709.1	1408792	1-scf00009	1
AX-157897105	1	57.366	KV452709.1	1411528	1-scf00009	1
AX-157897111	1	57.366	KV452709.1	1416869	1-scf00009	1
AX-157897120	1	57.366	KV452709.1	1426750	1-scf00009	1
AX-157897129	1	57.366	KV452709.1	1437166	1-scf00009	1
AX-157897132	1	57.366	KV452709.1	1440108	1-scf00009	1
AX-157897134	1	57.366	KV452709.1	1442724	1-scf00009	1
AX-157897135	1	57.366	KV452709.1	1446959	1-scf00009	1
AX-157897136	1	57.366	KV452709.1	1447583	1-scf00009	1
AX-157897137	1	57.366	KV452709.1	1448900	1-scf00009	1
AX-157897138	1	57.366	KV452709.1	1449055	1-scf00009	1
AX-158404405	1	57.366	KV452709.1	1452087	1-scf00009	1
AX-157897141	1	57.366	KV452709.1	1452669	1-scf00009	1
AX-157897143	1	57.366	KV452709.1	1454371	1-scf00009	1
AX-157897144	1	57.366	KV452709.1	1455571	1-scf00009	1
AX-157897145	1	57.366	KV452709.1	1456867	1-scf00009	1
AX-157897146	1	57.366	KV452709.1	1457593	1-scf00009	1
AX-157897150	1	57.366	KV452709.1	1464246	1-scf00009	1
AX-157810994	1	57.366	KV452709.1	1464672	1-scf00009	1
AX-157897152	1	57.366	KV452709.1	1465546	1-scf00009	1
AX-157810995	1	57.366	KV452709.1	1466333	1-scf00009	1
AX-157897153	1	57.366	KV452709.1	1466814	1-scf00009	1
AX-157864383	1	57.366	KV452709.1	1469735	1-scf00009	1
AX-157897173	1	57.366	KV452709.1	1490990	1-scf00009	1

AX-157897174	1	57.366	KV452709.1	1492590	1-scf00009	1
AX-157747106	1	57.366	KV452709.1	1499942	1-scf00009	1
AX-157897180	1	57.366	KV452709.1	1500798	1-scf00009	1
AX-157897185	1	57.366	KV452709.1	1508336	1-scf00009	1
AX-157772134	1	57.366	KV452709.1	1524100	1-scf00009	1
AX-157897195	1	57.366	KV452709.1	1531507	1-scf00009	1
AX-157774537	1	57.366	KV452709.1	1535570	1-scf00009	1
AX-157897196	1	57.366	KV452709.1	1536116	1-scf00009	1
AX-157897197	1	57.366	KV452709.1	1538068	1-scf00009	1
AX-157897198	1	57.366	KV452709.1	1539818	1-scf00009	1
AX-157810998	1	57.366	KV452709.1	1541140	1-scf00009	1
AX-157897199	1	57.366	KV452709.1	1542757	1-scf00009	1
AX-157897200	1	57.366	KV452709.1	1544434	1-scf00009	1
AX-157897203	1	57.366	KV452709.1	1546225	1-scf00009	1
AX-157897210	1	57.366	KV452709.1	1552685	1-scf00009	1
AX-157897211	1	57.366	KV452709.1	1553283	1-scf00009	1
AX-157897212	1	57.366	KV452709.1	1553685	1-scf00009	1
AX-157897213	1	57.366	KV452709.1	1554749	1-scf00009	1
AX-157874339	1	57.366	KV452709.1	1557593	1-scf00009	1
AX-157897223	1	57.366	KV452709.1	1564639	1-scf00009	1
AX-157897232	1	57.366	KV452709.1	1574442	1-scf00009	1
AX-157897239	1	57.366	KV452709.1	1582095	1-scf00009	1
AX-157897240	1	57.366	KV452709.1	1583772	1-scf00009	1
AX-157897242	1	57.366	KV452709.1	1585767	1-scf00009	1
AX-157897249	1	57.366	KV452709.1	1590169	1-scf00009	1
AX-157897251	1	57.366	KV452709.1	1591426	1-scf00009	1
AX-157897264	1	57.366	KV452709.1	1604506	1-scf00009	1
AX-158379137	1	57.366	KV452709.1	1632730	1-scf00009	1
AX-157897283	1	57.366	KV452709.1	1633913	1-scf00009	1
AX-158404414	1	57.366	KV452709.1	1653758	1-scf00009	1
AX-157774210	1	57.366	KV452709.1	1726086	1-scf00009	1
AX-157897340	1	57.366	KV452709.1	1726336	1-scf00009	1
AX-157897341	1	57.366	KV452709.1	1726907	1-scf00009	1
AX-157811011	1	57.366	KV452709.1	1728593	1-scf00009	1
AX-157897354	1	57.366	KV452709.1	1751103	1-scf00009	1
AX-157897356	1	57.366	KV452709.1	1754457	1-scf00009	1
AX-157897357	1	57.366	KV452709.1	1756890	1-scf00009	1
AX-157722418	1	57.366	KV452709.1	1758078	1-scf00009	1
AX-157897358	1	57.366	KV452709.1	1759437	1-scf00009	1
AX-157897360	1	57.366	KV452709.1	1764000	1-scf00009	1
AX-157897362	1	57.366	KV452709.1	1765888	1-scf00009	1
AX-157774293	1	57.366	KV452709.1	1768651	1-scf00009	1
AX-157897363	1	57.366	KV452709.1	1769004	1-scf00009	1
AX-157897364	1	57.366	KV452709.1	1770224	1-scf00009	1
AX-157897365	1	57.366	KV452709.1	1770747	1-scf00009	1
AX-157897366	1	57.366	KV452709.1	1773308	1-scf00009	1
AX-157897367	1	57.366	KV452709.1	1773716	1-scf00009	1
AX-157897369	1	57.366	KV452709.1	1776563	1-scf00009	1
AX-157897370	1	57.366	KV452709.1	1779630	1-scf00009	1
AX-157714167	1	57.366	KV452709.1	1780352	1-scf00009	1
AX-157897371	1	57.366	KV452709.1	1780716	1-scf00009	1

AX-157897372	1	57.366	KV452709.1	1781028	1-scf00009	1
AX-157811017	1	57.366	KV452709.1	1785831	1-scf00009	1
AX-157897373	1	57.366	KV452709.1	1786405	1-scf00009	1
AX-157897376	1	57.366	KV452709.1	1788808	1-scf00009	1
AX-157897377	1	57.366	KV452709.1	1790054	1-scf00009	1
AX-157897378	1	57.366	KV452709.1	1791071	1-scf00009	1
AX-157897379	1	57.366	KV452709.1	1792210	1-scf00009	1
AX-157897381	1	57.366	KV452709.1	1795624	1-scf00009	1
AX-157897382	1	57.366	KV452709.1	1796001	1-scf00009	1
AX-157897388	1	57.366	KV452709.1	1799120	1-scf00009	1
AX-157811020	1	57.366	KV452709.1	1802346	1-scf00009	1
AX-157897392	1	57.366	KV452709.1	1808863	1-scf00009	1
AX-157897396	1	57.366	KV452709.1	1813528	1-scf00009	1
AX-157897399	1	57.366	KV452709.1	1821009	1-scf00009	1
AX-157897402	1	57.366	KV452709.1	1822525	1-scf00009	1
AX-157897405	1	57.366	KV452709.1	1825886	1-scf00009	1
AX-157897406	1	57.366	KV452709.1	1826705	1-scf00009	1
AX-157897407	1	57.366	KV452709.1	1827324	1-scf00009	1
AX-157897409	1	57.366	KV452709.1	1829861	1-scf00009	1
AX-158404420	1	57.366	KV452709.1	1831122	1-scf00009	1
AX-157897410	1	57.366	KV452709.1	1832393	1-scf00009	1
AX-157897411	1	57.366	KV452709.1	1832655	1-scf00009	1
AX-157897412	1	57.366	KV452709.1	1832934	1-scf00009	1
AX-157897413	1	57.366	KV452709.1	1833037	1-scf00009	1
AX-157897431	1	57.366	KV452709.1	1869838	1-scf00009	1
AX-157897432	1	57.366	KV452709.1	1870531	1-scf00009	1
AX-157897434	1	57.366	KV452709.1	1872863	1-scf00009	1
AX-157811024	1	57.366	KV452709.1	1876649	1-scf00009	1
AX-157897448	1	57.366	KV452709.1	1898332	1-scf00009	1
AX-157897457	1	57.366	KV452709.1	1915386	1-scf00009	1
AX-157868532	1	57.366	KV452709.1	1917622	1-scf00009	1
AX-157897459	1	57.366	KV452709.1	1918463	1-scf00009	1
AX-157897460	1	57.366	KV452709.1	1919719	1-scf00009	1
AX-157897461	1	57.366	KV452709.1	1920075	1-scf00009	1
AX-157897463	1	57.366	KV452709.1	1922932	1-scf00009	1
AX-157897465	1	57.366	KV452709.1	1924556	1-scf00009	1
AX-157897469	1	57.366	KV452709.1	1927569	1-scf00009	1
AX-157897472	1	57.366	KV452709.1	1933263	1-scf00009	1
AX-157751437	1	57.366	KV452709.1	1967544	1-scf00009	1
AX-157897489	1	57.366	KV452709.1	1968206	1-scf00009	1
AX-157897491	1	57.366	KV452709.1	1970826	1-scf00009	1
AX-157897509	1	57.366	KV452709.1	1993407	1-scf00009	1
AX-157897515	1	57.366	KV452709.1	1997307	1-scf00009	1
AX-157897546	1	57.366	KV452709.1	2046693	1-scf00009	1
AX-157811041	1	57.366	KV452709.1	2102794	1-scf00009	1
AX-157811042	1	57.366	KV452709.1	2103035	1-scf00009	1
AX-157874347	1	57.366	KV452709.1	2129306	1-scf00009	1
AX-157897628	1	57.366	KV452709.1	2179505	1-scf00009	1
AX-157897632	1	57.366	KV452709.1	2190114	1-scf00009	1
AX-157739159	1	57.366	KV452709.1	2212405	1-scf00009	1
AX-157897650	1	57.366	KV452709.1	2215247	1-scf00009	1

AX-157897652	1	57.366	KV452709.1	2216865	1-scf00009	1
AX-157897653	1	57.366	KV452709.1	2217884	1-scf00009	1
AX-157897684	1	57.366	KV452709.1	2259992	1-scf00009	1
AX-157811062	1	57.366	KV452709.1	2441969	1-scf00009	1
AX-157897817	1	57.366	KV452709.1	2483854	1-scf00009	1
AX-157897869	1	57.366	KV452709.1	2551422	1-scf00009	1
AX-157897886	1	57.366	KV452709.1	2572921	1-scf00009	1
AX-157811077	1	57.366	KV452709.1	2647541	1-scf00009	1
AX-157811078	1	57.366	KV452709.1	2647797	1-scf00009	1
AX-158379140	1	57.366	KV452709.1	2713177	1-scf00009	1
AX-157864392	1	57.366	KV452709.1	2722964	1-scf00009	1
AX-157694202	1	57.366	KV452709.1	2743085	1-scf00009	1
AX-157898010	1	57.366	KV452709.1	2811059	1-scf00009	1
AX-157898155	1	57.366	KV452709.1	3090760	1-scf00009	1
AX-157898160	1	57.366	KV452709.1	3098198	1-scf00009	1
AX-157898240	1	57.366	KV452709.1	3283229	1-scf00009	1
AX-157898352	1	57.366	KV452709.1	3493483	1-scf00009	1
AX-157898359	1	57.366	KV452709.1	3513088	1-scf00009	1
AX-158381995	1	57.366	KV452709.1	3519050	1-scf00009	1
AX-157898365	1	57.366	KV452709.1	3519092	1-scf00009	1
AX-158381997	1	57.366	KV452709.1	3611179	1-scf00009	1
AX-157741793	1	57.366	KV452709.1	3622381	1-scf00009	1
AX-157898459	1	57.366	KV452709.1	3721709	1-scf00009	1
AX-157898474	1	57.366	KV452709.1	3794366	1-scf00009	1
AX-157715585	1	57.366	KV452709.1	3889450	1-scf00009	1
AX-157898528	1	57.366	KV452709.1	3892182	1-scf00009	1
AX-157898607	1	57.366	KV452709.1	3992968	1-scf00009	1
AX-157811197	1	57.366	KV452709.1	4185903	1-scf00009	1
AX-157811222	1	57.366	KV452709.1	4587421	1-scf00009	1
AX-157899078	1	57.366	KV452709.1	4643288	1-scf00009	1
AX-157899139	1	57.366	KV452709.1	4728318	1-scf00009	1
AX-157899196	1	57.366	KV452709.1	4825289	1-scf00009	1
AX-157899202	1	57.366	KV452709.1	4836126	1-scf00009	1
AX-157899242	1	57.366	KV452709.1	4913273	1-scf00009	1
AX-157899306	1	57.366	KV452709.1	4991351	1-scf00009	1
AX-157899332	1	57.366	KV452709.1	5034523	1-scf00009	1
AX-157811276	1	57.366	KV452709.1	5090671	1-scf00009	1
AX-157899424	1	57.366	KV452709.1	5152443	1-scf00009	1
AX-157899566	1	57.366	KV452709.1	5321331	1-scf00009	1
AX-157811330	1	57.366	KV452709.1	5578292	1-scf00009	1
AX-157899779	1	57.366	KV452709.1	5618932	1-scf00009	1
AX-157899816	1	57.366	KV452709.1	5654869	1-scf00009	1
AX-157902457	1	57.366	KV452713.1	773925	1-scf00011	1
AX-157903852	1	57.366	KV452713.1	2629521	1-scf00011	1
AX-157903921	1	57.366	KV452713.1	2701141	1-scf00011	1
AX-157811795	1	57.366	KV452713.1	2843194	1-scf00011	1
AX-157904918	1	57.366	KV452713.1	4416525	1-scf00011	1
AX-157725962	1	57.377	KV452709.1	1559050	1-scf00009	1
AX-157897225	1	57.39	KV452709.1	1567017	1-scf00009	1
AX-157897233	1	57.405	KV452709.1	1574926	1-scf00009	1
AX-157897261	1	57.417	KV452709.1	1601502	1-scf00009	1

AX-157897281	1	57.425	KV452709.1	1633024	1-scf00009	1
AX-157897288	1	57.442	KV452709.1	1646759	1-scf00009	1
AX-157811006	1	57.458	KV452709.1	1652776	1-scf00009	1
AX-157894650	1	57.475	KV452708.1	1096936	1-scf00008	1
AX-157776573	1	57.475	KV452708.1	2665252	1-scf00008	1
AX-157895905	1	57.475	KV452708.1	3096363	1-scf00008	1
AX-157896844	1	57.475	KV452709.1	1008010	1-scf00009	1
AX-157810982	1	57.475	KV452709.1	1355979	1-scf00009	1
AX-157897079	1	57.475	KV452709.1	1371562	1-scf00009	1
AX-157897101	1	57.475	KV452709.1	1407731	1-scf00009	1
AX-157746718	1	57.475	KV452709.1	1523384	1-scf00009	1
AX-157897204	1	57.475	KV452709.1	1546475	1-scf00009	1
AX-157897247	1	57.475	KV452709.1	1588926	1-scf00009	1
AX-157897250	1	57.475	KV452709.1	1590770	1-scf00009	1
AX-157897257	1	57.475	KV452709.1	1597058	1-scf00009	1
AX-157868524	1	57.475	KV452709.1	1613443	1-scf00009	1
AX-157897306	1	57.475	KV452709.1	1678154	1-scf00009	1
AX-157897309	1	57.475	KV452709.1	1686051	1-scf00009	1
AX-157897310	1	57.475	KV452709.1	1689167	1-scf00009	1
AX-157811008	1	57.475	KV452709.1	1690700	1-scf00009	1
AX-157897314	1	57.475	KV452709.1	1696920	1-scf00009	1
AX-157897315	1	57.475	KV452709.1	1697308	1-scf00009	1
AX-157897329	1	57.475	KV452709.1	1712515	1-scf00009	1
AX-157747396	1	57.475	KV452709.1	1750396	1-scf00009	1
AX-157897359	1	57.475	KV452709.1	1762678	1-scf00009	1
AX-157897361	1	57.475	KV452709.1	1764896	1-scf00009	1
AX-157749104	1	57.475	KV452709.1	1781464	1-scf00009	1
AX-157897380	1	57.475	KV452709.1	1792363	1-scf00009	1
AX-157897386	1	57.475	KV452709.1	1798527	1-scf00009	1
AX-157897394	1	57.475	KV452709.1	1812510	1-scf00009	1
AX-157897401	1	57.475	KV452709.1	1822069	1-scf00009	1
AX-157868528	1	57.475	KV452709.1	1834634	1-scf00009	1
AX-157897416	1	57.475	KV452709.1	1835869	1-scf00009	1
AX-157897417	1	57.475	KV452709.1	1837543	1-scf00009	1
AX-157897421	1	57.475	KV452709.1	1841331	1-scf00009	1
AX-157897424	1	57.475	KV452709.1	1842792	1-scf00009	1
AX-157811025	1	57.475	KV452709.1	1878583	1-scf00009	1
AX-157897468	1	57.475	KV452709.1	1926054	1-scf00009	1
AX-157868534	1	57.475	KV452709.1	1935799	1-scf00009	1
AX-157897480	1	57.475	KV452709.1	1949703	1-scf00009	1
AX-158389021	1	57.475	KV452709.1	1949948	1-scf00009	1
AX-157897481	1	57.475	KV452709.1	1950927	1-scf00009	1
AX-158389022	1	57.475	KV452709.1	1952539	1-scf00009	1
AX-157897482	1	57.475	KV452709.1	1953534	1-scf00009	1
AX-158389023	1	57.475	KV452709.1	1958860	1-scf00009	1
AX-157811031	1	57.475	KV452709.1	1959305	1-scf00009	1
AX-157897485	1	57.475	KV452709.1	1960758	1-scf00009	1
AX-157811032	1	57.475	KV452709.1	1960827	1-scf00009	1
AX-157755107	1	57.475	KV452709.1	1968809	1-scf00009	1
AX-157897490	1	57.475	KV452709.1	1969840	1-scf00009	1
AX-157897492	1	57.475	KV452709.1	1971640	1-scf00009	1

AX-157897493	1	57.475	KV452709.1	1972450	1-scf00009	1
AX-157724119	1	57.475	KV452709.1	1973227	1-scf00009	1
AX-157897494	1	57.475	KV452709.1	1973927	1-scf00009	1
AX-157897495	1	57.475	KV452709.1	1974313	1-scf00009	1
AX-157897496	1	57.475	KV452709.1	1976708	1-scf00009	1
AX-157868536	1	57.475	KV452709.1	1977474	1-scf00009	1
AX-157897497	1	57.475	KV452709.1	1978795	1-scf00009	1
AX-157868537	1	57.475	KV452709.1	1986007	1-scf00009	1
AX-157897501	1	57.475	KV452709.1	1987935	1-scf00009	1
AX-157897502	1	57.475	KV452709.1	1988378	1-scf00009	1
AX-157897505	1	57.475	KV452709.1	1990087	1-scf00009	1
AX-157897508	1	57.475	KV452709.1	1992990	1-scf00009	1
AX-157897510	1	57.475	KV452709.1	1993681	1-scf00009	1
AX-157897511	1	57.475	KV452709.1	1994133	1-scf00009	1
AX-157897512	1	57.475	KV452709.1	1995709	1-scf00009	1
AX-157897513	1	57.475	KV452709.1	1996156	1-scf00009	1
AX-157897514	1	57.475	KV452709.1	1996778	1-scf00009	1
AX-157897521	1	57.475	KV452709.1	2005592	1-scf00009	1
AX-157897522	1	57.475	KV452709.1	2006592	1-scf00009	1
AX-157868538	1	57.475	KV452709.1	2008440	1-scf00009	1
AX-157897525	1	57.475	KV452709.1	2009074	1-scf00009	1
AX-157897528	1	57.475	KV452709.1	2011020	1-scf00009	1
AX-157897529	1	57.475	KV452709.1	2012271	1-scf00009	1
AX-157897530	1	57.475	KV452709.1	2012733	1-scf00009	1
AX-157897531	1	57.475	KV452709.1	2013861	1-scf00009	1
AX-157897532	1	57.475	KV452709.1	2014808	1-scf00009	1
AX-157897533	1	57.475	KV452709.1	2015957	1-scf00009	1
AX-157897534	1	57.475	KV452709.1	2017243	1-scf00009	1
AX-157753244	1	57.475	KV452709.1	2017528	1-scf00009	1
AX-157897536	1	57.475	KV452709.1	2018917	1-scf00009	1
AX-157897537	1	57.475	KV452709.1	2019377	1-scf00009	1
AX-157868540	1	57.475	KV452709.1	2024726	1-scf00009	1
AX-157722133	1	57.475	KV452709.1	2025123	1-scf00009	1
AX-157868541	1	57.475	KV452709.1	2025560	1-scf00009	1
AX-157868542	1	57.475	KV452709.1	2032470	1-scf00009	1
AX-157897539	1	57.475	KV452709.1	2033074	1-scf00009	1
AX-157897540	1	57.475	KV452709.1	2035697	1-scf00009	1
AX-157716422	1	57.475	KV452709.1	2037168	1-scf00009	1
AX-157713568	1	57.475	KV452709.1	2037849	1-scf00009	1
AX-158389025	1	57.475	KV452709.1	2039588	1-scf00009	1
AX-157897542	1	57.475	KV452709.1	2041259	1-scf00009	1
AX-158389026	1	57.475	KV452709.1	2043053	1-scf00009	1
AX-157897545	1	57.475	KV452709.1	2045654	1-scf00009	1
AX-157868543	1	57.475	KV452709.1	2048666	1-scf00009	1
AX-157811035	1	57.475	KV452709.1	2049673	1-scf00009	1
AX-157897550	1	57.475	KV452709.1	2050252	1-scf00009	1
AX-157897551	1	57.475	KV452709.1	2051329	1-scf00009	1
AX-157868545	1	57.475	KV452709.1	2051781	1-scf00009	1
AX-157811036	1	57.475	KV452709.1	2052042	1-scf00009	1
AX-157811037	1	57.475	KV452709.1	2058593	1-scf00009	1
AX-157868546	1	57.475	KV452709.1	2060941	1-scf00009	1

AX-157897555	1	57.475	KV452709.1	2062649	1-scf00009	1
AX-157811038	1	57.475	KV452709.1	2064482	1-scf00009	1
AX-157897557	1	57.475	KV452709.1	2065811	1-scf00009	1
AX-158389028	1	57.475	KV452709.1	2066363	1-scf00009	1
AX-157897559	1	57.475	KV452709.1	2069296	1-scf00009	1
AX-157868547	1	57.475	KV452709.1	2069563	1-scf00009	1
AX-157897560	1	57.475	KV452709.1	2072587	1-scf00009	1
AX-158389029	1	57.475	KV452709.1	2078566	1-scf00009	1
AX-157897562	1	57.475	KV452709.1	2079198	1-scf00009	1
AX-157897563	1	57.475	KV452709.1	2079604	1-scf00009	1
AX-157897564	1	57.475	KV452709.1	2080778	1-scf00009	1
AX-158389031	1	57.475	KV452709.1	2081707	1-scf00009	1
AX-157897566	1	57.475	KV452709.1	2083277	1-scf00009	1
AX-158389032	1	57.475	KV452709.1	2084041	1-scf00009	1
AX-157897567	1	57.475	KV452709.1	2086231	1-scf00009	1
AX-157897568	1	57.475	KV452709.1	2086856	1-scf00009	1
AX-157897569	1	57.475	KV452709.1	2088994	1-scf00009	1
AX-157897570	1	57.475	KV452709.1	2091486	1-scf00009	1
AX-157897571	1	57.475	KV452709.1	2092335	1-scf00009	1
AX-157868549	1	57.475	KV452709.1	2094589	1-scf00009	1
AX-157868550	1	57.475	KV452709.1	2096747	1-scf00009	1
AX-157811040	1	57.475	KV452709.1	2098884	1-scf00009	1
AX-157868551	1	57.475	KV452709.1	2100456	1-scf00009	1
AX-157868552	1	57.475	KV452709.1	2100769	1-scf00009	1
AX-157897573	1	57.475	KV452709.1	2101679	1-scf00009	1
AX-157897574	1	57.475	KV452709.1	2101984	1-scf00009	1
AX-157897575	1	57.475	KV452709.1	2105325	1-scf00009	1
AX-157897576	1	57.475	KV452709.1	2105789	1-scf00009	1
AX-157897580	1	57.475	KV452709.1	2111801	1-scf00009	1
AX-157868553	1	57.475	KV452709.1	2113086	1-scf00009	1
AX-157897583	1	57.475	KV452709.1	2114773	1-scf00009	1
AX-157868554	1	57.475	KV452709.1	2116831	1-scf00009	1
AX-157897586	1	57.475	KV452709.1	2119566	1-scf00009	1
AX-157897587	1	57.475	KV452709.1	2119878	1-scf00009	1
AX-157897591	1	57.475	KV452709.1	2124433	1-scf00009	1
AX-157868555	1	57.475	KV452709.1	2125812	1-scf00009	1
AX-157811043	1	57.475	KV452709.1	2127129	1-scf00009	1
AX-157897595	1	57.475	KV452709.1	2127691	1-scf00009	1
AX-157897596	1	57.475	KV452709.1	2128746	1-scf00009	1
AX-157868556	1	57.475	KV452709.1	2130043	1-scf00009	1
AX-157897597	1	57.475	KV452709.1	2130302	1-scf00009	1
AX-157811045	1	57.475	KV452709.1	2130890	1-scf00009	1
AX-157897599	1	57.475	KV452709.1	2131606	1-scf00009	1
AX-157897601	1	57.475	KV452709.1	2135170	1-scf00009	1
AX-157868557	1	57.475	KV452709.1	2136006	1-scf00009	1
AX-157811047	1	57.475	KV452709.1	2138578	1-scf00009	1
AX-157897613	1	57.475	KV452709.1	2164113	1-scf00009	1
AX-157897616	1	57.475	KV452709.1	2167451	1-scf00009	1
AX-157897618	1	57.475	KV452709.1	2168250	1-scf00009	1
AX-157897621	1	57.475	KV452709.1	2170331	1-scf00009	1
AX-157897626	1	57.475	KV452709.1	2178435	1-scf00009	1

AX-157811052	1	57.475	KV452709.1	2191074	1-scf00009	1
AX-157897633	1	57.475	KV452709.1	2191308	1-scf00009	1
AX-157811053	1	57.475	KV452709.1	2192441	1-scf00009	1
AX-157897647	1	57.475	KV452709.1	2212015	1-scf00009	1
AX-157897649	1	57.475	KV452709.1	2214387	1-scf00009	1
AX-157897654	1	57.475	KV452709.1	2218262	1-scf00009	1
AX-157897655	1	57.475	KV452709.1	2218987	1-scf00009	1
AX-157898011	1	57.475	KV452709.1	2812585	1-scf00009	1
AX-157898118	1	57.475	KV452709.1	3013113	1-scf00009	1
AX-157898123	1	57.475	KV452709.1	3027364	1-scf00009	1
AX-157898204	1	57.475	KV452709.1	3226215	1-scf00009	1
AX-157883112	1	57.475	KV452709.1	3994642	1-scf00009	1
AX-157899148	1	57.475	KV452709.1	4743772	1-scf00009	1
AX-157899206	1	57.475	KV452709.1	4841948	1-scf00009	1
AX-157900017	1	57.475	KV452709.1	5926800	1-scf00009	1
AX-158404510	1	57.475	KV452709.1	6196911	1-scf00009	1
AX-157706511	1	57.475	3ML01032359	593	scf07975_1	UN
AX-157707521	1	57.475	3ML01034476	78	scf10151_1	UN
AX-157897646	1	57.547	KV452709.1	2211105	1-scf00009	1
AX-157897312	1	57.584	KV452709.1	1693241	1-scf00009	1
AX-157897319	1	57.584	KV452709.1	1700817	1-scf00009	1
AX-157897320	1	57.584	KV452709.1	1703628	1-scf00009	1
AX-157897321	1	57.584	KV452709.1	1704033	1-scf00009	1
AX-157897322	1	57.584	KV452709.1	1704458	1-scf00009	1
AX-157897414	1	57.584	KV452709.1	1833420	1-scf00009	1
AX-157897415	1	57.584	KV452709.1	1833842	1-scf00009	1
AX-157897418	1	57.584	KV452709.1	1839630	1-scf00009	1
AX-157897656	1	57.584	KV452709.1	2219680	1-scf00009	1
AX-157897660	1	57.584	KV452709.1	2227188	1-scf00009	1
AX-157754241	1	57.584	KV452709.1	2236417	1-scf00009	1
AX-157897572	1	57.649	KV452709.1	2099893	1-scf00009	1
AX-157897683	1	57.649	KV452709.1	2259659	1-scf00009	1
AX-157895902	1	57.694	KV452708.1	3093682	1-scf00008	1
AX-157895911	1	57.694	KV452708.1	3100831	1-scf00008	1
AX-157896730	1	57.694	KV452709.1	821728	1-scf00009	1
AX-157718071	1	57.694	KV452709.1	889153	1-scf00009	1
AX-157896788	1	57.694	KV452709.1	923930	1-scf00009	1
AX-157897110	1	57.694	KV452709.1	1416191	1-scf00009	1
AX-158404409	1	57.694	KV452709.1	1492708	1-scf00009	1
AX-157897207	1	57.694	KV452709.1	1549401	1-scf00009	1
AX-157897218	1	57.694	KV452709.1	1560646	1-scf00009	1
AX-157897263	1	57.694	KV452709.1	1604285	1-scf00009	1
AX-157897343	1	57.694	KV452709.1	1728799	1-scf00009	1
AX-157745141	1	57.694	KV452709.1	1786790	1-scf00009	1
AX-157897398	1	57.694	KV452709.1	1820721	1-scf00009	1
AX-157718955	1	57.694	KV452709.1	1850244	1-scf00009	1
AX-157897441	1	57.694	KV452709.1	1882344	1-scf00009	1
AX-157897451	1	57.694	KV452709.1	1901726	1-scf00009	1
AX-157897498	1	57.694	KV452709.1	1979833	1-scf00009	1
AX-157897504	1	57.694	KV452709.1	1989541	1-scf00009	1
AX-157897516	1	57.694	KV452709.1	1999690	1-scf00009	1

AX-157864389	1	57.694	KV452709.1	2019551	1-scf00009	1
AX-158389033	1	57.694	KV452709.1	2085753	1-scf00009	1
AX-157897598	1	57.694	KV452709.1	2130612	1-scf00009	1
AX-157897644	1	57.694	KV452709.1	2203823	1-scf00009	1
AX-157897681	1	57.694	KV452709.1	2253864	1-scf00009	1
AX-157897688	1	57.694	KV452709.1	2266406	1-scf00009	1
AX-157897689	1	57.694	KV452709.1	2270489	1-scf00009	1
AX-157897693	1	57.694	KV452709.1	2276780	1-scf00009	1
AX-157897708	1	57.694	KV452709.1	2314216	1-scf00009	1
AX-157897711	1	57.694	KV452709.1	2316536	1-scf00009	1
AX-157897712	1	57.694	KV452709.1	2316923	1-scf00009	1
AX-157897713	1	57.694	KV452709.1	2317856	1-scf00009	1
AX-157897716	1	57.694	KV452709.1	2332257	1-scf00009	1
AX-157897717	1	57.694	KV452709.1	2332843	1-scf00009	1
AX-157897719	1	57.694	KV452709.1	2334209	1-scf00009	1
AX-157897720	1	57.694	KV452709.1	2334675	1-scf00009	1
AX-157897722	1	57.694	KV452709.1	2335721	1-scf00009	1
AX-157897724	1	57.694	KV452709.1	2338370	1-scf00009	1
AX-157897728	1	57.694	KV452709.1	2340929	1-scf00009	1
AX-157897731	1	57.694	KV452709.1	2342263	1-scf00009	1
AX-157897734	1	57.694	KV452709.1	2346570	1-scf00009	1
AX-157897737	1	57.694	KV452709.1	2355664	1-scf00009	1
AX-157897738	1	57.694	KV452709.1	2357091	1-scf00009	1
AX-157897739	1	57.694	KV452709.1	2358844	1-scf00009	1
AX-157897740	1	57.694	KV452709.1	2363371	1-scf00009	1
AX-157757338	1	57.694	KV452709.1	2364154	1-scf00009	1
AX-158404428	1	57.694	KV452709.1	2371405	1-scf00009	1
AX-157897743	1	57.694	KV452709.1	2371667	1-scf00009	1
AX-158404429	1	57.694	KV452709.1	2373702	1-scf00009	1
AX-157897745	1	57.694	KV452709.1	2373881	1-scf00009	1
AX-157897747	1	57.694	KV452709.1	2374956	1-scf00009	1
AX-157897749	1	57.694	KV452709.1	2381116	1-scf00009	1
AX-157897751	1	57.694	KV452709.1	2383993	1-scf00009	1
AX-157897756	1	57.694	KV452709.1	2390886	1-scf00009	1
AX-157897759	1	57.694	KV452709.1	2397664	1-scf00009	1
AX-157897761	1	57.694	KV452709.1	2401473	1-scf00009	1
AX-157897764	1	57.694	KV452709.1	2406592	1-scf00009	1
AX-157897765	1	57.694	KV452709.1	2408891	1-scf00009	1
AX-157897766	1	57.694	KV452709.1	2410669	1-scf00009	1
AX-157897822	1	57.694	KV452709.1	2487897	1-scf00009	1
AX-157897837	1	57.694	KV452709.1	2505883	1-scf00009	1
AX-157811082	1	57.694	KV452709.1	2686756	1-scf00009	1
AX-157898052	1	57.694	KV452709.1	2868999	1-scf00009	1
AX-157754904	1	57.694	KV452709.1	2903574	1-scf00009	1
AX-157898921	1	57.694	KV452709.1	4453022	1-scf00009	1
AX-157899023	1	57.694	KV452709.1	4561609	1-scf00009	1
AX-157899039	1	57.694	KV452709.1	4579972	1-scf00009	1
AX-158379145	1	57.694	KV452709.1	4589968	1-scf00009	1
AX-157899049	1	57.694	KV452709.1	4605087	1-scf00009	1
AX-157811247	1	57.694	KV452709.1	4752590	1-scf00009	1
AX-157723269	1	57.694	KV452709.1	4793015	1-scf00009	1

AX-157899193	1	57.694	KV452709.1	4821298	1-scf00009	1
AX-157899194	1	57.694	KV452709.1	4821760	1-scf00009	1
AX-157899195	1	57.694	KV452709.1	4822012	1-scf00009	1
AX-157899247	1	57.694	KV452709.1	4918277	1-scf00009	1
AX-157897770	1	57.742	KV452709.1	2414674	1-scf00009	1
AX-157897721	1	57.803	KV452709.1	2335134	1-scf00009	1
AX-157897741	1	57.803	KV452709.1	2364984	1-scf00009	1
AX-157897760	1	57.803	KV452709.1	2398740	1-scf00009	1
AX-157811059	1	57.803	KV452709.1	2424371	1-scf00009	1
AX-157897776	1	57.803	KV452709.1	2425685	1-scf00009	1
AX-157897777	1	57.803	KV452709.1	2426376	1-scf00009	1
AX-157897778	1	57.803	KV452709.1	2426907	1-scf00009	1
AX-157897779	1	57.803	KV452709.1	2427408	1-scf00009	1
AX-157897780	1	57.803	KV452709.1	2427587	1-scf00009	1
AX-157897782	1	57.803	KV452709.1	2428759	1-scf00009	1
AX-157897784	1	57.803	KV452709.1	2430383	1-scf00009	1
AX-157897785	1	57.803	KV452709.1	2431760	1-scf00009	1
AX-157897786	1	57.803	KV452709.1	2434286	1-scf00009	1
AX-157897787	1	57.803	KV452709.1	2437982	1-scf00009	1
AX-157897790	1	57.803	KV452709.1	2441156	1-scf00009	1
AX-157897798	1	57.803	KV452709.1	2457454	1-scf00009	1
AX-157897800	1	57.803	KV452709.1	2459922	1-scf00009	1
AX-157897801	1	57.803	KV452709.1	2461941	1-scf00009	1
AX-157897803	1	57.803	KV452709.1	2463633	1-scf00009	1
AX-157897804	1	57.803	KV452709.1	2464515	1-scf00009	1
AX-157897806	1	57.803	KV452709.1	2466096	1-scf00009	1
AX-157897807	1	57.803	KV452709.1	2467430	1-scf00009	1
AX-157811063	1	57.803	KV452709.1	2467831	1-scf00009	1
AX-157897808	1	57.803	KV452709.1	2469552	1-scf00009	1
AX-157811064	1	57.803	KV452709.1	2470563	1-scf00009	1
AX-157811065	1	57.803	KV452709.1	2471407	1-scf00009	1
AX-157897818	1	57.912	KV452709.1	2484242	1-scf00009	1
AX-157897838	1	57.912	KV452709.1	2508552	1-scf00009	1
AX-157897840	1	57.967	KV452709.1	2510468	1-scf00009	1
AX-157897819	1	58.059	KV452709.1	2484727	1-scf00009	1
AX-157897845	1	58.059	KV452709.1	2519751	1-scf00009	1
AX-157898210	1	58.059	KV452709.1	3237390	1-scf00009	1
AX-157898217	1	58.059	KV452709.1	3245514	1-scf00009	1
AX-157898244	1	58.059	KV452709.1	3288934	1-scf00009	1
AX-157898246	1	58.059	KV452709.1	3289531	1-scf00009	1
AX-157898438	1	58.059	KV452709.1	3644875	1-scf00009	1
AX-157775088	1	58.131	KV452709.1	2523539	1-scf00009	1
AX-157897849	1	58.131	KV452709.1	2527744	1-scf00009	1
AX-157897851	1	58.131	KV452709.1	2529798	1-scf00009	1
AX-157897854	1	58.131	KV452709.1	2531938	1-scf00009	1
AX-157811067	1	58.131	KV452709.1	2544224	1-scf00009	1
AX-157897863	1	58.131	KV452709.1	2546117	1-scf00009	1
AX-157897865	1	58.131	KV452709.1	2549911	1-scf00009	1
AX-157897870	1	58.131	KV452709.1	2552890	1-scf00009	1
AX-157897871	1	58.131	KV452709.1	2553751	1-scf00009	1
AX-157898119	1	58.131	KV452709.1	3019485	1-scf00009	1

AX-157895411	1	58.24	KV452708.1	2433165	1-scf00008	1
AX-157895890	1	58.24	KV452708.1	3082380	1-scf00008	1
AX-157896137	1	58.24	KV452709.1	74253	1-scf00009	1
AX-157896257	1	58.24	KV452709.1	216477	1-scf00009	1
AX-157896285	1	58.24	KV452709.1	261390	1-scf00009	1
AX-157896293	1	58.24	KV452709.1	270453	1-scf00009	1
AX-157896296	1	58.24	KV452709.1	273007	1-scf00009	1
AX-157896297	1	58.24	KV452709.1	273359	1-scf00009	1
AX-157896298	1	58.24	KV452709.1	273597	1-scf00009	1
AX-157896301	1	58.24	KV452709.1	281244	1-scf00009	1
AX-157727783	1	58.24	KV452709.1	295186	1-scf00009	1
AX-157896317	1	58.24	KV452709.1	309508	1-scf00009	1
AX-157810918	1	58.24	KV452709.1	310127	1-scf00009	1
AX-157896350	1	58.24	KV452709.1	349863	1-scf00009	1
AX-157896714	1	58.24	KV452709.1	792507	1-scf00009	1
AX-157810947	1	58.24	KV452709.1	851215	1-scf00009	1
AX-157810990	1	58.24	KV452709.1	1397028	1-scf00009	1
AX-157897107	1	58.24	KV452709.1	1413589	1-scf00009	1
AX-157897108	1	58.24	KV452709.1	1415101	1-scf00009	1
AX-157897118	1	58.24	KV452709.1	1425250	1-scf00009	1
AX-157897230	1	58.24	KV452709.1	1571986	1-scf00009	1
AX-157853575	1	58.24	KV452709.1	2213566	1-scf00009	1
AX-157754799	1	58.24	KV452709.1	2241790	1-scf00009	1
AX-157897691	1	58.24	KV452709.1	2274855	1-scf00009	1
AX-157868571	1	58.24	KV452709.1	2311406	1-scf00009	1
AX-157897750	1	58.24	KV452709.1	2382786	1-scf00009	1
AX-157853576	1	58.24	KV452709.1	2386207	1-scf00009	1
AX-157897758	1	58.24	KV452709.1	2392441	1-scf00009	1
AX-157897771	1	58.24	KV452709.1	2415367	1-scf00009	1
AX-157897797	1	58.24	KV452709.1	2456330	1-scf00009	1
AX-158414601	1	58.24	KV452709.1	2474842	1-scf00009	1
AX-157897842	1	58.24	KV452709.1	2512160	1-scf00009	1
AX-157897848	1	58.24	KV452709.1	2525265	1-scf00009	1
AX-157811066	1	58.24	KV452709.1	2537733	1-scf00009	1
AX-157897868	1	58.24	KV452709.1	2551190	1-scf00009	1
AX-157897877	1	58.24	KV452709.1	2564424	1-scf00009	1
AX-157897885	1	58.24	KV452709.1	2571600	1-scf00009	1
AX-157897892	1	58.24	KV452709.1	2585905	1-scf00009	1
AX-157897895	1	58.24	KV452709.1	2588486	1-scf00009	1
AX-157811071	1	58.24	KV452709.1	2595342	1-scf00009	1
AX-157897900	1	58.24	KV452709.1	2600810	1-scf00009	1
AX-157811072	1	58.24	KV452709.1	2602927	1-scf00009	1
AX-157897905	1	58.24	KV452709.1	2604648	1-scf00009	1
AX-157897908	1	58.24	KV452709.1	2613751	1-scf00009	1
AX-157811074	1	58.24	KV452709.1	2614232	1-scf00009	1
AX-157751735	1	58.24	KV452709.1	2627196	1-scf00009	1
AX-157897918	1	58.24	KV452709.1	2628424	1-scf00009	1
AX-157897919	1	58.24	KV452709.1	2631133	1-scf00009	1
AX-157897925	1	58.24	KV452709.1	2646533	1-scf00009	1
AX-157897928	1	58.24	KV452709.1	2650596	1-scf00009	1
AX-157897930	1	58.24	KV452709.1	2654192	1-scf00009	1

AX-157897934	1	58.24	KV452709.1	2659026	1-scf00009	1
AX-157897935	1	58.24	KV452709.1	2659703	1-scf00009	1
AX-157897938	1	58.24	KV452709.1	2665080	1-scf00009	1
AX-157897939	1	58.24	KV452709.1	2665919	1-scf00009	1
AX-157811080	1	58.24	KV452709.1	2681213	1-scf00009	1
AX-157811081	1	58.24	KV452709.1	2684443	1-scf00009	1
AX-157868574	1	58.24	KV452709.1	2685898	1-scf00009	1
AX-157897948	1	58.24	KV452709.1	2686343	1-scf00009	1
AX-157811083	1	58.24	KV452709.1	2695180	1-scf00009	1
AX-157897954	1	58.24	KV452709.1	2699027	1-scf00009	1
AX-157811086	1	58.24	KV452709.1	2705314	1-scf00009	1
AX-157897956	1	58.24	KV452709.1	2706709	1-scf00009	1
AX-157897959	1	58.24	KV452709.1	2719765	1-scf00009	1
AX-157897963	1	58.24	KV452709.1	2723901	1-scf00009	1
AX-157897964	1	58.24	KV452709.1	2724751	1-scf00009	1
AX-157811088	1	58.24	KV452709.1	2726203	1-scf00009	1
AX-157897967	1	58.24	KV452709.1	2726383	1-scf00009	1
AX-157897968	1	58.24	KV452709.1	2728819	1-scf00009	1
AX-157897976	1	58.24	KV452709.1	2742477	1-scf00009	1
AX-157897978	1	58.24	KV452709.1	2746455	1-scf00009	1
AX-157773975	1	58.24	KV452709.1	2749244	1-scf00009	1
AX-157741467	1	58.24	KV452709.1	2756331	1-scf00009	1
AX-157897983	1	58.24	KV452709.1	2757082	1-scf00009	1
AX-157897985	1	58.24	KV452709.1	2759942	1-scf00009	1
AX-157897989	1	58.24	KV452709.1	2766265	1-scf00009	1
AX-157811091	1	58.24	KV452709.1	2767235	1-scf00009	1
AX-157897992	1	58.24	KV452709.1	2772339	1-scf00009	1
AX-157897994	1	58.24	KV452709.1	2778379	1-scf00009	1
AX-157744161	1	58.24	KV452709.1	2779628	1-scf00009	1
AX-157708538	1	58.24	KV452709.1	2782681	1-scf00009	1
AX-157898000	1	58.24	KV452709.1	2798463	1-scf00009	1
AX-157811093	1	58.24	KV452709.1	2798654	1-scf00009	1
AX-157898005	1	58.24	KV452709.1	2807544	1-scf00009	1
AX-157898017	1	58.24	KV452709.1	2822177	1-scf00009	1
AX-157898018	1	58.24	KV452709.1	2824309	1-scf00009	1
AX-157711283	1	58.24	KV452709.1	2824806	1-scf00009	1
AX-157898037	1	58.24	KV452709.1	2843544	1-scf00009	1
AX-158404437	1	58.24	KV452709.1	3021834	1-scf00009	1
AX-157898156	1	58.24	KV452709.1	3090910	1-scf00009	1
AX-157898190	1	58.24	KV452709.1	3188390	1-scf00009	1
AX-157898258	1	58.24	KV452709.1	3304212	1-scf00009	1
AX-157898259	1	58.24	KV452709.1	3304826	1-scf00009	1
AX-157723534	1	58.24	KV452709.1	3310593	1-scf00009	1
AX-157776804	1	58.24	KV452709.1	3604662	1-scf00009	1
AX-157898642	1	58.24	KV452709.1	4054076	1-scf00009	1
AX-157898643	1	58.24	KV452709.1	4056381	1-scf00009	1
AX-158381999	1	58.24	KV452709.1	4167793	1-scf00009	1
AX-157898749	1	58.24	KV452709.1	4211826	1-scf00009	1
AX-157898760	1	58.24	KV452709.1	4229477	1-scf00009	1
AX-157898763	1	58.24	KV452709.1	4238393	1-scf00009	1
AX-157898811	1	58.24	KV452709.1	4300181	1-scf00009	1

AX-157898962	1	58.24	KV452709.1	4498321	1-scf00009	1
AX-157899082	1	58.24	KV452709.1	4645960	1-scf00009	1
AX-157899119	1	58.24	KV452709.1	4693616	1-scf00009	1
AX-157811265	1	58.24	KV452709.1	4923867	1-scf00009	1
AX-157899263	1	58.24	KV452709.1	4938389	1-scf00009	1
AX-157717229	1	58.24	KV452709.1	5432062	1-scf00009	1
AX-157899782	1	58.24	KV452709.1	5620567	1-scf00009	1
AX-157899917	1	58.24	KV452709.1	5758385	1-scf00009	1
AX-157899961	1	58.24	KV452709.1	5837216	1-scf00009	1
AX-157811660	1	58.24	KV452713.1	650407	1-scf00011	1
AX-157703670	1	58.24	DX099789.1	238	IORI212 Ictalu	UN
AX-157895889	1	58.349	KV452708.1	3080935	1-scf00008	1
AX-157896175	1	58.349	KV452709.1	123580	1-scf00009	1
AX-157896214	1	58.349	KV452709.1	173239	1-scf00009	1
AX-157896241	1	58.349	KV452709.1	198261	1-scf00009	1
AX-157896305	1	58.349	KV452709.1	292386	1-scf00009	1
AX-157896611	1	58.349	KV452709.1	668360	1-scf00009	1
AX-157896677	1	58.349	KV452709.1	757014	1-scf00009	1
AX-157896691	1	58.349	KV452709.1	771798	1-scf00009	1
AX-157896746	1	58.349	KV452709.1	848306	1-scf00009	1
AX-157810958	1	58.349	KV452709.1	919673	1-scf00009	1
AX-157896795	1	58.349	KV452709.1	931224	1-scf00009	1
AX-157896801	1	58.349	KV452709.1	943523	1-scf00009	1
AX-157896824	1	58.349	KV452709.1	975816	1-scf00009	1
AX-157896855	1	58.349	KV452709.1	1022671	1-scf00009	1
AX-157896905	1	58.349	KV452709.1	1072121	1-scf00009	1
AX-157896942	1	58.349	KV452709.1	1133626	1-scf00009	1
AX-157897001	1	58.349	KV452709.1	1253758	1-scf00009	1
AX-157810980	1	58.349	KV452709.1	1324880	1-scf00009	1
AX-157897087	1	58.349	KV452709.1	1378299	1-scf00009	1
AX-157897266	1	58.349	KV452709.1	1605778	1-scf00009	1
AX-157897462	1	58.349	KV452709.1	1921168	1-scf00009	1
AX-157811034	1	58.349	KV452709.1	2041324	1-scf00009	1
AX-157897543	1	58.349	KV452709.1	2042126	1-scf00009	1
AX-157897669	1	58.349	KV452709.1	2242232	1-scf00009	1
AX-157897726	1	58.349	KV452709.1	2340059	1-scf00009	1
AX-157897767	1	58.349	KV452709.1	2411817	1-scf00009	1
AX-157897833	1	58.349	KV452709.1	2501477	1-scf00009	1
AX-157897844	1	58.349	KV452709.1	2517252	1-scf00009	1
AX-157693818	1	58.349	KV452709.1	2522902	1-scf00009	1
AX-157897874	1	58.349	KV452709.1	2558509	1-scf00009	1
AX-157897882	1	58.349	KV452709.1	2569702	1-scf00009	1
AX-157708620	1	58.349	KV452709.1	2583538	1-scf00009	1
AX-157897893	1	58.349	KV452709.1	2586266	1-scf00009	1
AX-157897894	1	58.349	KV452709.1	2587040	1-scf00009	1
AX-157862606	1	58.349	KV452709.1	2587569	1-scf00009	1
AX-157897898	1	58.349	KV452709.1	2598717	1-scf00009	1
AX-157897899	1	58.349	KV452709.1	2599869	1-scf00009	1
AX-157897903	1	58.349	KV452709.1	2602799	1-scf00009	1
AX-158389046	1	58.349	KV452709.1	2607328	1-scf00009	1
AX-157811073	1	58.349	KV452709.1	2608432	1-scf00009	1

AX-157897907	1	58.349	KV452709.1	2611690	1-scf00009	1
AX-157897909	1	58.349	KV452709.1	2619322	1-scf00009	1
AX-157897910	1	58.349	KV452709.1	2619700	1-scf00009	1
AX-157897911	1	58.349	KV452709.1	2620743	1-scf00009	1
AX-157897913	1	58.349	KV452709.1	2623411	1-scf00009	1
AX-157897914	1	58.349	KV452709.1	2624065	1-scf00009	1
AX-157811075	1	58.349	KV452709.1	2624905	1-scf00009	1
AX-157897915	1	58.349	KV452709.1	2625132	1-scf00009	1
AX-157897916	1	58.349	KV452709.1	2626474	1-scf00009	1
AX-157868573	1	58.349	KV452709.1	2630568	1-scf00009	1
AX-157897921	1	58.349	KV452709.1	2633441	1-scf00009	1
AX-157897922	1	58.349	KV452709.1	2633754	1-scf00009	1
AX-157698783	1	58.349	KV452709.1	2635627	1-scf00009	1
AX-158389047	1	58.349	KV452709.1	2645997	1-scf00009	1
AX-157897926	1	58.349	KV452709.1	2646930	1-scf00009	1
AX-157897931	1	58.349	KV452709.1	2654547	1-scf00009	1
AX-158389049	1	58.349	KV452709.1	2654978	1-scf00009	1
AX-157897933	1	58.349	KV452709.1	2657389	1-scf00009	1
AX-158389050	1	58.349	KV452709.1	2664638	1-scf00009	1
AX-157897945	1	58.349	KV452709.1	2682267	1-scf00009	1
AX-157897947	1	58.349	KV452709.1	2684701	1-scf00009	1
AX-157897949	1	58.349	KV452709.1	2691844	1-scf00009	1
AX-157897952	1	58.349	KV452709.1	2694392	1-scf00009	1
AX-157897953	1	58.349	KV452709.1	2694657	1-scf00009	1
AX-157811084	1	58.349	KV452709.1	2699283	1-scf00009	1
AX-157897969	1	58.349	KV452709.1	2730042	1-scf00009	1
AX-157897970	1	58.349	KV452709.1	2730846	1-scf00009	1
AX-158404432	1	58.349	KV452709.1	2737830	1-scf00009	1
AX-157811090	1	58.349	KV452709.1	2744001	1-scf00009	1
AX-157897980	1	58.349	KV452709.1	2748328	1-scf00009	1
AX-157897982	1	58.349	KV452709.1	2750539	1-scf00009	1
AX-157897986	1	58.349	KV452709.1	2760752	1-scf00009	1
AX-157897988	1	58.349	KV452709.1	2764989	1-scf00009	1
AX-157712280	1	58.349	KV452709.1	2776047	1-scf00009	1
AX-157897993	1	58.349	KV452709.1	2777958	1-scf00009	1
AX-157868575	1	58.349	KV452709.1	2779557	1-scf00009	1
AX-157868576	1	58.349	KV452709.1	2781365	1-scf00009	1
AX-157708539	1	58.349	KV452709.1	2782738	1-scf00009	1
AX-157897996	1	58.349	KV452709.1	2784676	1-scf00009	1
AX-157811092	1	58.349	KV452709.1	2793815	1-scf00009	1
AX-157897998	1	58.349	KV452709.1	2795113	1-scf00009	1
AX-158389053	1	58.349	KV452709.1	2798176	1-scf00009	1
AX-157898001	1	58.349	KV452709.1	2799144	1-scf00009	1
AX-158389054	1	58.349	KV452709.1	2800693	1-scf00009	1
AX-157898002	1	58.349	KV452709.1	2802684	1-scf00009	1
AX-157898044	1	58.349	KV452709.1	2858068	1-scf00009	1
AX-157898047	1	58.349	KV452709.1	2862045	1-scf00009	1
AX-157758226	1	58.349	KV452709.1	2862624	1-scf00009	1
AX-157898048	1	58.349	KV452709.1	2863318	1-scf00009	1
AX-158389061	1	58.349	KV452709.1	2864492	1-scf00009	1
AX-157898050	1	58.349	KV452709.1	2864954	1-scf00009	1

AX-158389062	1	58.349	KV452709.1	2866077	1-scf00009	1
AX-157898051	1	58.349	KV452709.1	2866113	1-scf00009	1
AX-157898053	1	58.349	KV452709.1	2869596	1-scf00009	1
AX-157898057	1	58.349	KV452709.1	2874884	1-scf00009	1
AX-157898059	1	58.349	KV452709.1	2881746	1-scf00009	1
AX-157898061	1	58.349	KV452709.1	2883433	1-scf00009	1
AX-157898062	1	58.349	KV452709.1	2885047	1-scf00009	1
AX-157811100	1	58.349	KV452709.1	2885746	1-scf00009	1
AX-157898064	1	58.349	KV452709.1	2886958	1-scf00009	1
AX-157898065	1	58.349	KV452709.1	2888286	1-scf00009	1
AX-157898066	1	58.349	KV452709.1	2889911	1-scf00009	1
AX-157898067	1	58.349	KV452709.1	2891944	1-scf00009	1
AX-157898068	1	58.349	KV452709.1	2894321	1-scf00009	1
AX-157898070	1	58.349	KV452709.1	2904048	1-scf00009	1
AX-158389064	1	58.349	KV452709.1	2904371	1-scf00009	1
AX-157811101	1	58.349	KV452709.1	2904952	1-scf00009	1
AX-157868579	1	58.349	KV452709.1	2918731	1-scf00009	1
AX-157898078	1	58.349	KV452709.1	2919111	1-scf00009	1
AX-157868580	1	58.349	KV452709.1	2926666	1-scf00009	1
AX-157898082	1	58.349	KV452709.1	2928024	1-scf00009	1
AX-157811104	1	58.349	KV452709.1	2930921	1-scf00009	1
AX-158389065	1	58.349	KV452709.1	2934554	1-scf00009	1
AX-157811105	1	58.349	KV452709.1	2937210	1-scf00009	1
AX-157898086	1	58.349	KV452709.1	2939528	1-scf00009	1
AX-157868585	1	58.349	KV452709.1	2943952	1-scf00009	1
AX-157898087	1	58.349	KV452709.1	2944297	1-scf00009	1
AX-157898089	1	58.349	KV452709.1	2945793	1-scf00009	1
AX-157898090	1	58.349	KV452709.1	2946113	1-scf00009	1
AX-157811106	1	58.349	KV452709.1	2949464	1-scf00009	1
AX-157898091	1	58.349	KV452709.1	2950641	1-scf00009	1
AX-158389066	1	58.349	KV452709.1	2951467	1-scf00009	1
AX-157898096	1	58.349	KV452709.1	2956345	1-scf00009	1
AX-157811108	1	58.349	KV452709.1	2960672	1-scf00009	1
AX-157811109	1	58.349	KV452709.1	2961863	1-scf00009	1
AX-158389069	1	58.349	KV452709.1	2964301	1-scf00009	1
AX-157898100	1	58.349	KV452709.1	2966264	1-scf00009	1
AX-158389070	1	58.349	KV452709.1	2966993	1-scf00009	1
AX-157898101	1	58.349	KV452709.1	2972685	1-scf00009	1
AX-157898102	1	58.349	KV452709.1	2973095	1-scf00009	1
AX-157898107	1	58.349	KV452709.1	2987854	1-scf00009	1
AX-157898108	1	58.349	KV452709.1	2987915	1-scf00009	1
AX-157898110	1	58.349	KV452709.1	2992469	1-scf00009	1
AX-157898115	1	58.349	KV452709.1	3007351	1-scf00009	1
AX-157898116	1	58.349	KV452709.1	3008088	1-scf00009	1
AX-157898117	1	58.349	KV452709.1	3008882	1-scf00009	1
AX-157898121	1	58.349	KV452709.1	3022935	1-scf00009	1
AX-157898122	1	58.349	KV452709.1	3023701	1-scf00009	1
AX-158421791	1	58.349	KV452709.1	3025671	1-scf00009	1
AX-157898125	1	58.349	KV452709.1	3033433	1-scf00009	1
AX-157898127	1	58.349	KV452709.1	3034969	1-scf00009	1
AX-157898130	1	58.349	KV452709.1	3039053	1-scf00009	1

AX-157898131	1	58.349	KV452709.1	3041543	1-scf00009	1
AX-158389076	1	58.349	KV452709.1	3043833	1-scf00009	1
AX-157898133	1	58.349	KV452709.1	3045103	1-scf00009	1
AX-157811110	1	58.349	KV452709.1	3046724	1-scf00009	1
AX-157898137	1	58.349	KV452709.1	3051845	1-scf00009	1
AX-157868589	1	58.349	KV452709.1	3060593	1-scf00009	1
AX-157811113	1	58.349	KV452709.1	3062109	1-scf00009	1
AX-157898140	1	58.349	KV452709.1	3069352	1-scf00009	1
AX-157898142	1	58.349	KV452709.1	3072265	1-scf00009	1
AX-157898144	1	58.349	KV452709.1	3075380	1-scf00009	1
AX-157811114	1	58.349	KV452709.1	3077242	1-scf00009	1
AX-157695648	1	58.349	KV452709.1	3078727	1-scf00009	1
AX-157898145	1	58.349	KV452709.1	3079638	1-scf00009	1
AX-157898146	1	58.349	KV452709.1	3080993	1-scf00009	1
AX-157898147	1	58.349	KV452709.1	3082346	1-scf00009	1
AX-157898148	1	58.349	KV452709.1	3082933	1-scf00009	1
AX-157898149	1	58.349	KV452709.1	3083589	1-scf00009	1
AX-157898150	1	58.349	KV452709.1	3084312	1-scf00009	1
AX-158389079	1	58.349	KV452709.1	3085933	1-scf00009	1
AX-157811115	1	58.349	KV452709.1	3087997	1-scf00009	1
AX-157898158	1	58.349	KV452709.1	3095236	1-scf00009	1
AX-157898161	1	58.349	KV452709.1	3098426	1-scf00009	1
AX-157898163	1	58.349	KV452709.1	3102584	1-scf00009	1
AX-157898164	1	58.349	KV452709.1	3103931	1-scf00009	1
AX-157898169	1	58.349	KV452709.1	3109281	1-scf00009	1
AX-157898170	1	58.349	KV452709.1	3110156	1-scf00009	1
AX-157811117	1	58.349	KV452709.1	3113140	1-scf00009	1
AX-157898172	1	58.349	KV452709.1	3116834	1-scf00009	1
AX-158389082	1	58.349	KV452709.1	3119063	1-scf00009	1
AX-157898174	1	58.349	KV452709.1	3119617	1-scf00009	1
AX-157898175	1	58.349	KV452709.1	3120483	1-scf00009	1
AX-158389083	1	58.349	KV452709.1	3121211	1-scf00009	1
AX-157898178	1	58.349	KV452709.1	3123856	1-scf00009	1
AX-157811120	1	58.349	KV452709.1	3126570	1-scf00009	1
AX-157898179	1	58.349	KV452709.1	3127242	1-scf00009	1
AX-157898180	1	58.349	KV452709.1	3128260	1-scf00009	1
AX-158389085	1	58.349	KV452709.1	3130121	1-scf00009	1
AX-157898181	1	58.349	KV452709.1	3131406	1-scf00009	1
AX-157898182	1	58.349	KV452709.1	3135306	1-scf00009	1
AX-157811121	1	58.349	KV452709.1	3139449	1-scf00009	1
AX-157811122	1	58.349	KV452709.1	3143663	1-scf00009	1
AX-157898184	1	58.349	KV452709.1	3147990	1-scf00009	1
AX-157868593	1	58.349	KV452709.1	3148363	1-scf00009	1
AX-157898185	1	58.349	KV452709.1	3161460	1-scf00009	1
AX-157868595	1	58.349	KV452709.1	3164646	1-scf00009	1
AX-157868596	1	58.349	KV452709.1	3170753	1-scf00009	1
AX-157868597	1	58.349	KV452709.1	3174627	1-scf00009	1
AX-157898189	1	58.349	KV452709.1	3179717	1-scf00009	1
AX-157868598	1	58.349	KV452709.1	3184537	1-scf00009	1
AX-158389089	1	58.349	KV452709.1	3187912	1-scf00009	1
AX-157811123	1	58.349	KV452709.1	3188778	1-scf00009	1

AX-157898191	1	58.349	KV452709.1	3189031	1-scf00009	1
AX-157811124	1	58.349	KV452709.1	3189679	1-scf00009	1
AX-157868600	1	58.349	KV452709.1	3193138	1-scf00009	1
AX-158403917	1	58.349	KV452709.1	3195172	1-scf00009	1
AX-158404439	1	58.349	KV452709.1	3195242	1-scf00009	1
AX-157868601	1	58.349	KV452709.1	3199145	1-scf00009	1
AX-157811125	1	58.349	KV452709.1	3203814	1-scf00009	1
AX-158389090	1	58.349	KV452709.1	3205113	1-scf00009	1
AX-158389091	1	58.349	KV452709.1	3209115	1-scf00009	1
AX-157898196	1	58.349	KV452709.1	3212386	1-scf00009	1
AX-157898199	1	58.349	KV452709.1	3215735	1-scf00009	1
AX-157868607	1	58.349	KV452709.1	3220834	1-scf00009	1
AX-157898202	1	58.349	KV452709.1	3221956	1-scf00009	1
AX-158389093	1	58.349	KV452709.1	3224770	1-scf00009	1
AX-158389095	1	58.349	KV452709.1	3230514	1-scf00009	1
AX-157898206	1	58.349	KV452709.1	3231436	1-scf00009	1
AX-157898207	1	58.349	KV452709.1	3232280	1-scf00009	1
AX-157898208	1	58.349	KV452709.1	3232937	1-scf00009	1
AX-157898209	1	58.349	KV452709.1	3235042	1-scf00009	1
AX-157898211	1	58.349	KV452709.1	3238030	1-scf00009	1
AX-158389098	1	58.349	KV452709.1	3238756	1-scf00009	1
AX-157898212	1	58.349	KV452709.1	3239964	1-scf00009	1
AX-158389099	1	58.349	KV452709.1	3241549	1-scf00009	1
AX-157898216	1	58.349	KV452709.1	3244292	1-scf00009	1
AX-157898218	1	58.349	KV452709.1	3247681	1-scf00009	1
AX-157898219	1	58.349	KV452709.1	3250003	1-scf00009	1
AX-157898220	1	58.349	KV452709.1	3250689	1-scf00009	1
AX-157898221	1	58.349	KV452709.1	3253094	1-scf00009	1
AX-157898223	1	58.349	KV452709.1	3257959	1-scf00009	1
AX-158389102	1	58.349	KV452709.1	3258138	1-scf00009	1
AX-157898224	1	58.349	KV452709.1	3259505	1-scf00009	1
AX-157898225	1	58.349	KV452709.1	3260140	1-scf00009	1
AX-158389105	1	58.349	KV452709.1	3265741	1-scf00009	1
AX-158389107	1	58.349	KV452709.1	3271963	1-scf00009	1
AX-157898234	1	58.349	KV452709.1	3275946	1-scf00009	1
AX-157898235	1	58.349	KV452709.1	3278652	1-scf00009	1
AX-158389109	1	58.349	KV452709.1	3279301	1-scf00009	1
AX-157898238	1	58.349	KV452709.1	3281384	1-scf00009	1
AX-157898239	1	58.349	KV452709.1	3282039	1-scf00009	1
AX-158389110	1	58.349	KV452709.1	3283275	1-scf00009	1
AX-157898245	1	58.349	KV452709.1	3289014	1-scf00009	1
AX-158389111	1	58.349	KV452709.1	3289348	1-scf00009	1
AX-158389112	1	58.349	KV452709.1	3291895	1-scf00009	1
AX-157898248	1	58.349	KV452709.1	3292468	1-scf00009	1
AX-157898249	1	58.349	KV452709.1	3293411	1-scf00009	1
AX-157898250	1	58.349	KV452709.1	3294030	1-scf00009	1
AX-157898252	1	58.349	KV452709.1	3296279	1-scf00009	1
AX-157898253	1	58.349	KV452709.1	3296999	1-scf00009	1
AX-157898256	1	58.349	KV452709.1	3300324	1-scf00009	1
AX-158389115	1	58.349	KV452709.1	3307840	1-scf00009	1
AX-157898261	1	58.349	KV452709.1	3315438	1-scf00009	1

AX-157898263	1	58.349	KV452709.1	3317223	1-scf00009	1
AX-158404445	1	58.349	KV452709.1	3318724	1-scf00009	1
AX-157898264	1	58.349	KV452709.1	3321776	1-scf00009	1
AX-158389118	1	58.349	KV452709.1	3321918	1-scf00009	1
AX-157898265	1	58.349	KV452709.1	3322295	1-scf00009	1
AX-157898269	1	58.349	KV452709.1	3326978	1-scf00009	1
AX-157898272	1	58.349	KV452709.1	3331818	1-scf00009	1
AX-157898275	1	58.349	KV452709.1	3337439	1-scf00009	1
AX-158389121	1	58.349	KV452709.1	3337736	1-scf00009	1
AX-157898276	1	58.349	KV452709.1	3338727	1-scf00009	1
AX-158389122	1	58.349	KV452709.1	3342124	1-scf00009	1
AX-157898283	1	58.349	KV452709.1	3360662	1-scf00009	1
AX-158389126	1	58.349	KV452709.1	3362021	1-scf00009	1
AX-157811129	1	58.349	KV452709.1	3362860	1-scf00009	1
AX-157811130	1	58.349	KV452709.1	3363175	1-scf00009	1
AX-157898284	1	58.349	KV452709.1	3367106	1-scf00009	1
AX-158389127	1	58.349	KV452709.1	3367781	1-scf00009	1
AX-157898285	1	58.349	KV452709.1	3367860	1-scf00009	1
AX-157898286	1	58.349	KV452709.1	3368460	1-scf00009	1
AX-158389128	1	58.349	KV452709.1	3371002	1-scf00009	1
AX-157898287	1	58.349	KV452709.1	3371310	1-scf00009	1
AX-157898288	1	58.349	KV452709.1	3371735	1-scf00009	1
AX-157898289	1	58.349	KV452709.1	3372302	1-scf00009	1
AX-157748824	1	58.349	KV452709.1	3372946	1-scf00009	1
AX-157898290	1	58.349	KV452709.1	3373902	1-scf00009	1
AX-157898291	1	58.349	KV452709.1	3375358	1-scf00009	1
AX-157898292	1	58.349	KV452709.1	3376084	1-scf00009	1
AX-158389129	1	58.349	KV452709.1	3377101	1-scf00009	1
AX-157811131	1	58.349	KV452709.1	3380939	1-scf00009	1
AX-158421737	1	58.349	KV452709.1	3382895	1-scf00009	1
AX-158389130	1	58.349	KV452709.1	3384273	1-scf00009	1
AX-158389131	1	58.349	KV452709.1	3386015	1-scf00009	1
AX-158389132	1	58.349	KV452709.1	3388629	1-scf00009	1
AX-157898298	1	58.349	KV452709.1	3389492	1-scf00009	1
AX-158389133	1	58.349	KV452709.1	3390869	1-scf00009	1
AX-157898300	1	58.349	KV452709.1	3391916	1-scf00009	1
AX-157898301	1	58.349	KV452709.1	3393572	1-scf00009	1
AX-158389134	1	58.349	KV452709.1	3394228	1-scf00009	1
AX-157898303	1	58.349	KV452709.1	3396096	1-scf00009	1
AX-158389135	1	58.349	KV452709.1	3397879	1-scf00009	1
AX-157898305	1	58.349	KV452709.1	3398471	1-scf00009	1
AX-157898306	1	58.349	KV452709.1	3399523	1-scf00009	1
AX-157898307	1	58.349	KV452709.1	3399937	1-scf00009	1
AX-157898309	1	58.349	KV452709.1	3401873	1-scf00009	1
AX-158404447	1	58.349	KV452709.1	3402345	1-scf00009	1
AX-158389136	1	58.349	KV452709.1	3403579	1-scf00009	1
AX-157898310	1	58.349	KV452709.1	3403858	1-scf00009	1
AX-157898311	1	58.349	KV452709.1	3405828	1-scf00009	1
AX-157811132	1	58.349	KV452709.1	3409276	1-scf00009	1
AX-157811133	1	58.349	KV452709.1	3409892	1-scf00009	1
AX-157898312	1	58.349	KV452709.1	3412052	1-scf00009	1

AX-158389139	1	58.349	KV452709.1	3412674	1-scf00009	1
AX-158389140	1	58.349	KV452709.1	3415860	1-scf00009	1
AX-158389141	1	58.349	KV452709.1	3418903	1-scf00009	1
AX-157898315	1	58.349	KV452709.1	3420425	1-scf00009	1
AX-158389142	1	58.349	KV452709.1	3421561	1-scf00009	1
AX-157898317	1	58.349	KV452709.1	3425718	1-scf00009	1
AX-158389143	1	58.349	KV452709.1	3427379	1-scf00009	1
AX-157898319	1	58.349	KV452709.1	3428439	1-scf00009	1
AX-157898321	1	58.349	KV452709.1	3430018	1-scf00009	1
AX-158389144	1	58.349	KV452709.1	3430615	1-scf00009	1
AX-158389145	1	58.349	KV452709.1	3432937	1-scf00009	1
AX-157898323	1	58.349	KV452709.1	3433198	1-scf00009	1
AX-157898325	1	58.349	KV452709.1	3438724	1-scf00009	1
AX-158389146	1	58.349	KV452709.1	3438927	1-scf00009	1
AX-157868611	1	58.349	KV452709.1	3440282	1-scf00009	1
AX-157868612	1	58.349	KV452709.1	3440546	1-scf00009	1
AX-157811134	1	58.349	KV452709.1	3441114	1-scf00009	1
AX-157868614	1	58.349	KV452709.1	3442663	1-scf00009	1
AX-157898327	1	58.349	KV452709.1	3443095	1-scf00009	1
AX-157898328	1	58.349	KV452709.1	3444257	1-scf00009	1
AX-157868615	1	58.349	KV452709.1	3444918	1-scf00009	1
AX-157898329	1	58.349	KV452709.1	3445726	1-scf00009	1
AX-157898330	1	58.349	KV452709.1	3446494	1-scf00009	1
AX-157898331	1	58.349	KV452709.1	3448284	1-scf00009	1
AX-157898334	1	58.349	KV452709.1	3453682	1-scf00009	1
AX-157868618	1	58.349	KV452709.1	3454913	1-scf00009	1
AX-157898335	1	58.349	KV452709.1	3455375	1-scf00009	1
AX-157868619	1	58.349	KV452709.1	3455820	1-scf00009	1
AX-157898336	1	58.349	KV452709.1	3456160	1-scf00009	1
AX-157868620	1	58.349	KV452709.1	3458461	1-scf00009	1
AX-157868621	1	58.349	KV452709.1	3458843	1-scf00009	1
AX-157868622	1	58.349	KV452709.1	3459185	1-scf00009	1
AX-157868624	1	58.349	KV452709.1	3463275	1-scf00009	1
AX-157898341	1	58.349	KV452709.1	3466000	1-scf00009	1
AX-157898342	1	58.349	KV452709.1	3467348	1-scf00009	1
AX-157898344	1	58.349	KV452709.1	3468310	1-scf00009	1
AX-157868626	1	58.349	KV452709.1	3470399	1-scf00009	1
AX-157868627	1	58.349	KV452709.1	3470747	1-scf00009	1
AX-157898346	1	58.349	KV452709.1	3472154	1-scf00009	1
AX-157868628	1	58.349	KV452709.1	3473706	1-scf00009	1
AX-157898347	1	58.349	KV452709.1	3476279	1-scf00009	1
AX-157868630	1	58.349	KV452709.1	3478290	1-scf00009	1
AX-157898349	1	58.349	KV452709.1	3479296	1-scf00009	1
AX-157711139	1	58.349	KV452709.1	3482867	1-scf00009	1
AX-157811136	1	58.349	KV452709.1	3488483	1-scf00009	1
AX-157898351	1	58.349	KV452709.1	3493118	1-scf00009	1
AX-157898354	1	58.349	KV452709.1	3494867	1-scf00009	1
AX-158414605	1	58.349	KV452709.1	3497537	1-scf00009	1
AX-158404449	1	58.349	KV452709.1	3497685	1-scf00009	1
AX-157898355	1	58.349	KV452709.1	3497761	1-scf00009	1
AX-157898356	1	58.349	KV452709.1	3505584	1-scf00009	1

AX-157898357	1	58.349	KV452709.1	3506363	1-scf00009	1
AX-158389149	1	58.349	KV452709.1	3507108	1-scf00009	1
AX-158389150	1	58.349	KV452709.1	3509080	1-scf00009	1
AX-157898358	1	58.349	KV452709.1	3511655	1-scf00009	1
AX-158389152	1	58.349	KV452709.1	3513786	1-scf00009	1
AX-157898360	1	58.349	KV452709.1	3515008	1-scf00009	1
AX-158389153	1	58.349	KV452709.1	3515846	1-scf00009	1
AX-157898361	1	58.349	KV452709.1	3515913	1-scf00009	1
AX-157898364	1	58.349	KV452709.1	3518799	1-scf00009	1
AX-157898366	1	58.349	KV452709.1	3519582	1-scf00009	1
AX-157898367	1	58.349	KV452709.1	3520229	1-scf00009	1
AX-158389154	1	58.349	KV452709.1	3521128	1-scf00009	1
AX-157898370	1	58.349	KV452709.1	3522719	1-scf00009	1
AX-157898371	1	58.349	KV452709.1	3523339	1-scf00009	1
AX-157883109	1	58.349	KV452709.1	3523658	1-scf00009	1
AX-157898373	1	58.349	KV452709.1	3524566	1-scf00009	1
AX-158389156	1	58.349	KV452709.1	3526352	1-scf00009	1
AX-157898377	1	58.349	KV452709.1	3526509	1-scf00009	1
AX-157898379	1	58.349	KV452709.1	3529119	1-scf00009	1
AX-157898380	1	58.349	KV452709.1	3529529	1-scf00009	1
AX-157898382	1	58.349	KV452709.1	3530735	1-scf00009	1
AX-157868631	1	58.349	KV452709.1	3531395	1-scf00009	1
AX-157868632	1	58.349	KV452709.1	3531871	1-scf00009	1
AX-158389157	1	58.349	KV452709.1	3534043	1-scf00009	1
AX-157898386	1	58.349	KV452709.1	3541280	1-scf00009	1
AX-157898387	1	58.349	KV452709.1	3543470	1-scf00009	1
AX-157898389	1	58.349	KV452709.1	3551321	1-scf00009	1
AX-157898390	1	58.349	KV452709.1	3551800	1-scf00009	1
AX-158389160	1	58.349	KV452709.1	3552691	1-scf00009	1
AX-157898391	1	58.349	KV452709.1	3553876	1-scf00009	1
AX-157898392	1	58.349	KV452709.1	3555115	1-scf00009	1
AX-158389161	1	58.349	KV452709.1	3555432	1-scf00009	1
AX-157898393	1	58.349	KV452709.1	3555592	1-scf00009	1
AX-157898394	1	58.349	KV452709.1	3556214	1-scf00009	1
AX-157898395	1	58.349	KV452709.1	3557910	1-scf00009	1
AX-157898396	1	58.349	KV452709.1	3559918	1-scf00009	1
AX-157811137	1	58.349	KV452709.1	3563152	1-scf00009	1
AX-157811139	1	58.349	KV452709.1	3564783	1-scf00009	1
AX-157898400	1	58.349	KV452709.1	3568537	1-scf00009	1
AX-157898401	1	58.349	KV452709.1	3568898	1-scf00009	1
AX-157898402	1	58.349	KV452709.1	3569740	1-scf00009	1
AX-157898403	1	58.349	KV452709.1	3572235	1-scf00009	1
AX-157898404	1	58.349	KV452709.1	3572632	1-scf00009	1
AX-158389163	1	58.349	KV452709.1	3573655	1-scf00009	1
AX-158389164	1	58.349	KV452709.1	3575617	1-scf00009	1
AX-157898405	1	58.349	KV452709.1	3576529	1-scf00009	1
AX-157898406	1	58.349	KV452709.1	3578014	1-scf00009	1
AX-157898409	1	58.349	KV452709.1	3580453	1-scf00009	1
AX-158389165	1	58.349	KV452709.1	3582940	1-scf00009	1
AX-157898411	1	58.349	KV452709.1	3583596	1-scf00009	1
AX-157811140	1	58.349	KV452709.1	3585154	1-scf00009	1

AX-158389167	1	58.349	KV452709.1	3587414	1-scf00009	1
AX-158389168	1	58.349	KV452709.1	3591032	1-scf00009	1
AX-158389169	1	58.349	KV452709.1	3591472	1-scf00009	1
AX-157898414	1	58.349	KV452709.1	3593382	1-scf00009	1
AX-157868635	1	58.349	KV452709.1	3593640	1-scf00009	1
AX-157898415	1	58.349	KV452709.1	3597584	1-scf00009	1
AX-157868638	1	58.349	KV452709.1	3602207	1-scf00009	1
AX-157898416	1	58.349	KV452709.1	3605642	1-scf00009	1
AX-157811141	1	58.349	KV452709.1	3606823	1-scf00009	1
AX-157898418	1	58.349	KV452709.1	3611768	1-scf00009	1
AX-157898419	1	58.349	KV452709.1	3612663	1-scf00009	1
AX-157868639	1	58.349	KV452709.1	3613636	1-scf00009	1
AX-157868640	1	58.349	KV452709.1	3622617	1-scf00009	1
AX-158389170	1	58.349	KV452709.1	3626926	1-scf00009	1
AX-157898425	1	58.349	KV452709.1	3627958	1-scf00009	1
AX-157898429	1	58.349	KV452709.1	3633784	1-scf00009	1
AX-157868641	1	58.349	KV452709.1	3635168	1-scf00009	1
AX-157811148	1	58.349	KV452709.1	3635463	1-scf00009	1
AX-157898432	1	58.349	KV452709.1	3636344	1-scf00009	1
AX-157811149	1	58.349	KV452709.1	3636811	1-scf00009	1
AX-157868642	1	58.349	KV452709.1	3638374	1-scf00009	1
AX-157898433	1	58.349	KV452709.1	3639537	1-scf00009	1
AX-157868643	1	58.349	KV452709.1	3640911	1-scf00009	1
AX-157898435	1	58.349	KV452709.1	3642944	1-scf00009	1
AX-157898436	1	58.349	KV452709.1	3643499	1-scf00009	1
AX-157898437	1	58.349	KV452709.1	3644621	1-scf00009	1
AX-157868645	1	58.349	KV452709.1	3645315	1-scf00009	1
AX-157898439	1	58.349	KV452709.1	3646716	1-scf00009	1
AX-157898440	1	58.349	KV452709.1	3646855	1-scf00009	1
AX-157868647	1	58.349	KV452709.1	3648009	1-scf00009	1
AX-157898441	1	58.349	KV452709.1	3650518	1-scf00009	1
AX-157898442	1	58.349	KV452709.1	3653082	1-scf00009	1
AX-157898443	1	58.349	KV452709.1	3653392	1-scf00009	1
AX-157898444	1	58.349	KV452709.1	3653978	1-scf00009	1
AX-157898446	1	58.349	KV452709.1	3655691	1-scf00009	1
AX-157898448	1	58.349	KV452709.1	3664318	1-scf00009	1
AX-157898449	1	58.349	KV452709.1	3664802	1-scf00009	1
AX-157898450	1	58.349	KV452709.1	3664852	1-scf00009	1
AX-158421793	1	58.349	KV452709.1	3667503	1-scf00009	1
AX-157898453	1	58.349	KV452709.1	3668167	1-scf00009	1
AX-157811151	1	58.349	KV452709.1	3669509	1-scf00009	1
AX-157811152	1	58.349	KV452709.1	3678366	1-scf00009	1
AX-157811154	1	58.349	KV452709.1	3691975	1-scf00009	1
AX-157898454	1	58.349	KV452709.1	3702670	1-scf00009	1
AX-157898456	1	58.349	KV452709.1	3711806	1-scf00009	1
AX-158404454	1	58.349	KV452709.1	3713128	1-scf00009	1
AX-157898457	1	58.349	KV452709.1	3716585	1-scf00009	1
AX-157898462	1	58.349	KV452709.1	3730219	1-scf00009	1
AX-157898463	1	58.349	KV452709.1	3731017	1-scf00009	1
AX-158404458	1	58.349	KV452709.1	3738414	1-scf00009	1
AX-157868652	1	58.349	KV452709.1	3741781	1-scf00009	1

AX-157898466	1	58.349	KV452709.1	3742344	1-scf00009	1
AX-157868653	1	58.349	KV452709.1	3746363	1-scf00009	1
AX-157811160	1	58.349	KV452709.1	3755899	1-scf00009	1
AX-157811161	1	58.349	KV452709.1	3760387	1-scf00009	1
AX-157811162	1	58.349	KV452709.1	3772595	1-scf00009	1
AX-158414606	1	58.349	KV452709.1	3774978	1-scf00009	1
AX-157898473	1	58.349	KV452709.1	3793899	1-scf00009	1
AX-157898475	1	58.349	KV452709.1	3801639	1-scf00009	1
AX-157898477	1	58.349	KV452709.1	3803811	1-scf00009	1
AX-157722935	1	58.349	KV452709.1	3807592	1-scf00009	1
AX-157898479	1	58.349	KV452709.1	3812865	1-scf00009	1
AX-157898480	1	58.349	KV452709.1	3813568	1-scf00009	1
AX-157898481	1	58.349	KV452709.1	3813828	1-scf00009	1
AX-157898483	1	58.349	KV452709.1	3816786	1-scf00009	1
AX-157898484	1	58.349	KV452709.1	3823271	1-scf00009	1
AX-157898485	1	58.349	KV452709.1	3823600	1-scf00009	1
AX-157710216	1	58.349	KV452709.1	3823936	1-scf00009	1
AX-157898492	1	58.349	KV452709.1	3834554	1-scf00009	1
AX-157811163	1	58.349	KV452709.1	3834594	1-scf00009	1
AX-157898495	1	58.349	KV452709.1	3839992	1-scf00009	1
AX-157898497	1	58.349	KV452709.1	3844611	1-scf00009	1
AX-157898498	1	58.349	KV452709.1	3845765	1-scf00009	1
AX-157898499	1	58.349	KV452709.1	3847461	1-scf00009	1
AX-157898500	1	58.349	KV452709.1	3848814	1-scf00009	1
AX-157898501	1	58.349	KV452709.1	3849955	1-scf00009	1
AX-157811165	1	58.349	KV452709.1	3852097	1-scf00009	1
AX-158404460	1	58.349	KV452709.1	3852408	1-scf00009	1
AX-157719203	1	58.349	KV452709.1	3855758	1-scf00009	1
AX-157898505	1	58.349	KV452709.1	3855978	1-scf00009	1
AX-158389178	1	58.349	KV452709.1	3871532	1-scf00009	1
AX-157811167	1	58.349	KV452709.1	3872400	1-scf00009	1
AX-157898513	1	58.349	KV452709.1	3873136	1-scf00009	1
AX-157811168	1	58.349	KV452709.1	3873517	1-scf00009	1
AX-157898517	1	58.349	KV452709.1	3876351	1-scf00009	1
AX-157898518	1	58.349	KV452709.1	3877948	1-scf00009	1
AX-157898519	1	58.349	KV452709.1	3879324	1-scf00009	1
AX-157811169	1	58.349	KV452709.1	3879547	1-scf00009	1
AX-157811170	1	58.349	KV452709.1	3879714	1-scf00009	1
AX-157868660	1	58.349	KV452709.1	3880853	1-scf00009	1
AX-157811172	1	58.349	KV452709.1	3882080	1-scf00009	1
AX-157898522	1	58.349	KV452709.1	3882450	1-scf00009	1
AX-157811173	1	58.349	KV452709.1	3884353	1-scf00009	1
AX-157868662	1	58.349	KV452709.1	3884672	1-scf00009	1
AX-157898523	1	58.349	KV452709.1	3884938	1-scf00009	1
AX-157898524	1	58.349	KV452709.1	3885257	1-scf00009	1
AX-157751130	1	58.349	KV452709.1	3889013	1-scf00009	1
AX-157811174	1	58.349	KV452709.1	3893337	1-scf00009	1
AX-158404462	1	58.349	KV452709.1	3898463	1-scf00009	1
AX-158404463	1	58.349	KV452709.1	3898761	1-scf00009	1
AX-157898531	1	58.349	KV452709.1	3901528	1-scf00009	1
AX-158389179	1	58.349	KV452709.1	3901576	1-scf00009	1

AX-157898532	1	58.349	KV452709.1	3902812	1-scf00009	1
AX-157898533	1	58.349	KV452709.1	3904055	1-scf00009	1
AX-157898534	1	58.349	KV452709.1	3904972	1-scf00009	1
AX-157868664	1	58.349	KV452709.1	3907620	1-scf00009	1
AX-157898536	1	58.349	KV452709.1	3911215	1-scf00009	1
AX-157898538	1	58.349	KV452709.1	3911726	1-scf00009	1
AX-157811176	1	58.349	KV452709.1	3912202	1-scf00009	1
AX-157811177	1	58.349	KV452709.1	3913251	1-scf00009	1
AX-157811178	1	58.349	KV452709.1	3915022	1-scf00009	1
AX-157898545	1	58.349	KV452709.1	3921711	1-scf00009	1
AX-157898546	1	58.349	KV452709.1	3922189	1-scf00009	1
AX-157898548	1	58.349	KV452709.1	3923138	1-scf00009	1
AX-157898549	1	58.349	KV452709.1	3923653	1-scf00009	1
AX-157898552	1	58.349	KV452709.1	3926075	1-scf00009	1
AX-157898555	1	58.349	KV452709.1	3930105	1-scf00009	1
AX-157898556	1	58.349	KV452709.1	3930425	1-scf00009	1
AX-157898558	1	58.349	KV452709.1	3933307	1-scf00009	1
AX-157811179	1	58.349	KV452709.1	3933585	1-scf00009	1
AX-157898559	1	58.349	KV452709.1	3934830	1-scf00009	1
AX-157898561	1	58.349	KV452709.1	3936902	1-scf00009	1
AX-157811180	1	58.349	KV452709.1	3937531	1-scf00009	1
AX-157811181	1	58.349	KV452709.1	3938293	1-scf00009	1
AX-157898562	1	58.349	KV452709.1	3939714	1-scf00009	1
AX-157898563	1	58.349	KV452709.1	3940063	1-scf00009	1
AX-157898565	1	58.349	KV452709.1	3942145	1-scf00009	1
AX-157898567	1	58.349	KV452709.1	3943766	1-scf00009	1
AX-157898568	1	58.349	KV452709.1	3945291	1-scf00009	1
AX-157898570	1	58.349	KV452709.1	3948314	1-scf00009	1
AX-157811183	1	58.349	KV452709.1	3957248	1-scf00009	1
AX-157898577	1	58.349	KV452709.1	3958493	1-scf00009	1
AX-157898578	1	58.349	KV452709.1	3961753	1-scf00009	1
AX-157898579	1	58.349	KV452709.1	3962629	1-scf00009	1
AX-157898581	1	58.349	KV452709.1	3964815	1-scf00009	1
AX-157898582	1	58.349	KV452709.1	3965484	1-scf00009	1
AX-157898585	1	58.349	KV452709.1	3967922	1-scf00009	1
AX-157898588	1	58.349	KV452709.1	3972618	1-scf00009	1
AX-157898589	1	58.349	KV452709.1	3973809	1-scf00009	1
AX-157898590	1	58.349	KV452709.1	3974646	1-scf00009	1
AX-158389182	1	58.349	KV452709.1	3974682	1-scf00009	1
AX-157898591	1	58.349	KV452709.1	3975151	1-scf00009	1
AX-157898594	1	58.349	KV452709.1	3976683	1-scf00009	1
AX-157898595	1	58.349	KV452709.1	3977892	1-scf00009	1
AX-157898596	1	58.349	KV452709.1	3978287	1-scf00009	1
AX-157898598	1	58.349	KV452709.1	3980924	1-scf00009	1
AX-157883111	1	58.349	KV452709.1	3994386	1-scf00009	1
AX-157868666	1	58.349	KV452709.1	4003117	1-scf00009	1
AX-157868667	1	58.349	KV452709.1	4008041	1-scf00009	1
AX-157898614	1	58.349	KV452709.1	4009021	1-scf00009	1
AX-157898617	1	58.349	KV452709.1	4010663	1-scf00009	1
AX-157898618	1	58.349	KV452709.1	4010884	1-scf00009	1
AX-157898619	1	58.349	KV452709.1	4011208	1-scf00009	1

AX-157898620	1	58.349	KV452709.1	4011495	1-scf00009	1
AX-157898621	1	58.349	KV452709.1	4011601	1-scf00009	1
AX-157811188	1	58.349	KV452709.1	4012777	1-scf00009	1
AX-157898622	1	58.349	KV452709.1	4012838	1-scf00009	1
AX-158389183	1	58.349	KV452709.1	4012932	1-scf00009	1
AX-157898624	1	58.349	KV452709.1	4014334	1-scf00009	1
AX-157811190	1	58.349	KV452709.1	4015435	1-scf00009	1
AX-157811191	1	58.349	KV452709.1	4018038	1-scf00009	1
AX-158389185	1	58.349	KV452709.1	4018444	1-scf00009	1
AX-157898629	1	58.349	KV452709.1	4022493	1-scf00009	1
AX-157898632	1	58.349	KV452709.1	4025829	1-scf00009	1
AX-157898633	1	58.349	KV452709.1	4026747	1-scf00009	1
AX-157898636	1	58.349	KV452709.1	4034877	1-scf00009	1
AX-157868669	1	58.349	KV452709.1	4035298	1-scf00009	1
AX-157811193	1	58.349	KV452709.1	4038242	1-scf00009	1
AX-157868670	1	58.349	KV452709.1	4040896	1-scf00009	1
AX-157898640	1	58.349	KV452709.1	4050559	1-scf00009	1
AX-157898646	1	58.349	KV452709.1	4061009	1-scf00009	1
AX-157898647	1	58.349	KV452709.1	4061726	1-scf00009	1
AX-157898649	1	58.349	KV452709.1	4062902	1-scf00009	1
AX-157898650	1	58.349	KV452709.1	4063436	1-scf00009	1
AX-157727346	1	58.349	KV452709.1	4065107	1-scf00009	1
AX-157898652	1	58.349	KV452709.1	4066180	1-scf00009	1
AX-157811194	1	58.349	KV452709.1	4067931	1-scf00009	1
AX-157898654	1	58.349	KV452709.1	4069316	1-scf00009	1
AX-157898658	1	58.349	KV452709.1	4077437	1-scf00009	1
AX-157898659	1	58.349	KV452709.1	4078832	1-scf00009	1
AX-157898660	1	58.349	KV452709.1	4080397	1-scf00009	1
AX-157898661	1	58.349	KV452709.1	4082139	1-scf00009	1
AX-157898664	1	58.349	KV452709.1	4083796	1-scf00009	1
AX-157898665	1	58.349	KV452709.1	4085622	1-scf00009	1
AX-157898667	1	58.349	KV452709.1	4087145	1-scf00009	1
AX-157898668	1	58.349	KV452709.1	4088181	1-scf00009	1
AX-158389188	1	58.349	KV452709.1	4088457	1-scf00009	1
AX-157898670	1	58.349	KV452709.1	4089827	1-scf00009	1
AX-157898672	1	58.349	KV452709.1	4090368	1-scf00009	1
AX-157898673	1	58.349	KV452709.1	4090858	1-scf00009	1
AX-158389189	1	58.349	KV452709.1	4090901	1-scf00009	1
AX-157898675	1	58.349	KV452709.1	4091859	1-scf00009	1
AX-157898676	1	58.349	KV452709.1	4092287	1-scf00009	1
AX-158389190	1	58.349	KV452709.1	4093302	1-scf00009	1
AX-157898679	1	58.349	KV452709.1	4097449	1-scf00009	1
AX-157898682	1	58.349	KV452709.1	4099852	1-scf00009	1
AX-158389192	1	58.349	KV452709.1	4100381	1-scf00009	1
AX-157772809	1	58.349	KV452709.1	4101960	1-scf00009	1
AX-158389193	1	58.349	KV452709.1	4105229	1-scf00009	1
AX-157811195	1	58.349	KV452709.1	4105912	1-scf00009	1
AX-157898687	1	58.349	KV452709.1	4107484	1-scf00009	1
AX-157898689	1	58.349	KV452709.1	4111069	1-scf00009	1
AX-157758469	1	58.349	KV452709.1	4111483	1-scf00009	1
AX-157898690	1	58.349	KV452709.1	4112738	1-scf00009	1

AX-157898691	1	58.349	KV452709.1	4114269	1-scf00009	1
AX-157898693	1	58.349	KV452709.1	4117176	1-scf00009	1
AX-157898694	1	58.349	KV452709.1	4117920	1-scf00009	1
AX-157898698	1	58.349	KV452709.1	4124455	1-scf00009	1
AX-157898705	1	58.349	KV452709.1	4131603	1-scf00009	1
AX-157898706	1	58.349	KV452709.1	4133375	1-scf00009	1
AX-157898708	1	58.349	KV452709.1	4140040	1-scf00009	1
AX-158389203	1	58.349	KV452709.1	4144041	1-scf00009	1
AX-157898710	1	58.349	KV452709.1	4144362	1-scf00009	1
AX-157898713	1	58.349	KV452709.1	4147977	1-scf00009	1
AX-158389204	1	58.349	KV452709.1	4148259	1-scf00009	1
AX-158389205	1	58.349	KV452709.1	4150104	1-scf00009	1
AX-158389206	1	58.349	KV452709.1	4152152	1-scf00009	1
AX-157898718	1	58.349	KV452709.1	4154954	1-scf00009	1
AX-157702419	1	58.349	KV452709.1	4156068	1-scf00009	1
AX-157898722	1	58.349	KV452709.1	4159340	1-scf00009	1
AX-158389207	1	58.349	KV452709.1	4159399	1-scf00009	1
AX-157755199	1	58.349	KV452709.1	4159636	1-scf00009	1
AX-157898723	1	58.349	KV452709.1	4161113	1-scf00009	1
AX-157898724	1	58.349	KV452709.1	4163224	1-scf00009	1
AX-158389208	1	58.349	KV452709.1	4164335	1-scf00009	1
AX-157898727	1	58.349	KV452709.1	4164501	1-scf00009	1
AX-157898730	1	58.349	KV452709.1	4165853	1-scf00009	1
AX-157898732	1	58.349	KV452709.1	4168260	1-scf00009	1
AX-158389209	1	58.349	KV452709.1	4168451	1-scf00009	1
AX-157898733	1	58.349	KV452709.1	4169737	1-scf00009	1
AX-158389211	1	58.349	KV452709.1	4172166	1-scf00009	1
AX-157898734	1	58.349	KV452709.1	4175008	1-scf00009	1
AX-158404467	1	58.349	KV452709.1	4179130	1-scf00009	1
AX-158389212	1	58.349	KV452709.1	4180635	1-scf00009	1
AX-157811196	1	58.349	KV452709.1	4185190	1-scf00009	1
AX-157868672	1	58.349	KV452709.1	4186649	1-scf00009	1
AX-157811198	1	58.349	KV452709.1	4189432	1-scf00009	1
AX-157898738	1	58.349	KV452709.1	4193187	1-scf00009	1
AX-157898739	1	58.349	KV452709.1	4197205	1-scf00009	1
AX-157898741	1	58.349	KV452709.1	4199362	1-scf00009	1
AX-157898742	1	58.349	KV452709.1	4200547	1-scf00009	1
AX-157898745	1	58.349	KV452709.1	4204303	1-scf00009	1
AX-158389214	1	58.349	KV452709.1	4208634	1-scf00009	1
AX-158389215	1	58.349	KV452709.1	4210429	1-scf00009	1
AX-157898758	1	58.349	KV452709.1	4227173	1-scf00009	1
AX-157898759	1	58.349	KV452709.1	4228174	1-scf00009	1
AX-157811200	1	58.349	KV452709.1	4231994	1-scf00009	1
AX-157898767	1	58.349	KV452709.1	4241884	1-scf00009	1
AX-157898769	1	58.349	KV452709.1	4243665	1-scf00009	1
AX-157868673	1	58.349	KV452709.1	4246913	1-scf00009	1
AX-157898775	1	58.349	KV452709.1	4252103	1-scf00009	1
AX-157898776	1	58.349	KV452709.1	4254613	1-scf00009	1
AX-157898783	1	58.349	KV452709.1	4260542	1-scf00009	1
AX-157898785	1	58.349	KV452709.1	4262047	1-scf00009	1
AX-157898790	1	58.349	KV452709.1	4268714	1-scf00009	1

AX-157898794	1	58.349	KV452709.1	4272758	1-scf00009	1
AX-157898797	1	58.349	KV452709.1	4275050	1-scf00009	1
AX-157811201	1	58.349	KV452709.1	4276452	1-scf00009	1
AX-157898800	1	58.349	KV452709.1	4277024	1-scf00009	1
AX-157898801	1	58.349	KV452709.1	4277262	1-scf00009	1
AX-157868675	1	58.349	KV452709.1	4280575	1-scf00009	1
AX-157898803	1	58.349	KV452709.1	4284213	1-scf00009	1
AX-157883113	1	58.349	KV452709.1	4286504	1-scf00009	1
AX-157898806	1	58.349	KV452709.1	4288120	1-scf00009	1
AX-157721033	1	58.349	KV452709.1	4298791	1-scf00009	1
AX-157898815	1	58.349	KV452709.1	4301815	1-scf00009	1
AX-157811202	1	58.349	KV452709.1	4303008	1-scf00009	1
AX-157898816	1	58.349	KV452709.1	4303401	1-scf00009	1
AX-157811203	1	58.349	KV452709.1	4315818	1-scf00009	1
AX-157868677	1	58.349	KV452709.1	4321019	1-scf00009	1
AX-157868678	1	58.349	KV452709.1	4322366	1-scf00009	1
AX-157811204	1	58.349	KV452709.1	4322975	1-scf00009	1
AX-157868679	1	58.349	KV452709.1	4326767	1-scf00009	1
AX-157811205	1	58.349	KV452709.1	4327307	1-scf00009	1
AX-157898827	1	58.349	KV452709.1	4328545	1-scf00009	1
AX-157898828	1	58.349	KV452709.1	4329153	1-scf00009	1
AX-157868680	1	58.349	KV452709.1	4334734	1-scf00009	1
AX-157898839	1	58.349	KV452709.1	4342868	1-scf00009	1
AX-157898841	1	58.349	KV452709.1	4344805	1-scf00009	1
AX-157853580	1	58.349	KV452709.1	4349644	1-scf00009	1
AX-157898850	1	58.349	KV452709.1	4360056	1-scf00009	1
AX-157898854	1	58.349	KV452709.1	4363998	1-scf00009	1
AX-157898859	1	58.349	KV452709.1	4374313	1-scf00009	1
AX-157898860	1	58.349	KV452709.1	4374787	1-scf00009	1
AX-157898868	1	58.349	KV452709.1	4384362	1-scf00009	1
AX-157898869	1	58.349	KV452709.1	4385511	1-scf00009	1
AX-157898884	1	58.349	KV452709.1	4407867	1-scf00009	1
AX-157898885	1	58.349	KV452709.1	4408756	1-scf00009	1
AX-157898889	1	58.349	KV452709.1	4414067	1-scf00009	1
AX-157898893	1	58.349	KV452709.1	4416895	1-scf00009	1
AX-157898897	1	58.349	KV452709.1	4421089	1-scf00009	1
AX-157898898	1	58.349	KV452709.1	4421415	1-scf00009	1
AX-157898899	1	58.349	KV452709.1	4421898	1-scf00009	1
AX-157811209	1	58.349	KV452709.1	4435271	1-scf00009	1
AX-157898906	1	58.349	KV452709.1	4435807	1-scf00009	1
AX-157811213	1	58.349	KV452709.1	4443932	1-scf00009	1
AX-157898927	1	58.349	KV452709.1	4460399	1-scf00009	1
AX-157811214	1	58.349	KV452709.1	4466067	1-scf00009	1
AX-157898938	1	58.349	KV452709.1	4473589	1-scf00009	1
AX-157898939	1	58.349	KV452709.1	4475286	1-scf00009	1
AX-157898950	1	58.349	KV452709.1	4486998	1-scf00009	1
AX-157898954	1	58.349	KV452709.1	4489937	1-scf00009	1
AX-157898956	1	58.349	KV452709.1	4491237	1-scf00009	1
AX-158389218	1	58.349	KV452709.1	4494520	1-scf00009	1
AX-157898970	1	58.349	KV452709.1	4508137	1-scf00009	1
AX-157898977	1	58.349	KV452709.1	4518163	1-scf00009	1

AX-157898978	1	58.349	KV452709.1	4519596	1-scf00009	1
AX-157898980	1	58.349	KV452709.1	4522945	1-scf00009	1
AX-157898981	1	58.349	KV452709.1	4526159	1-scf00009	1
AX-157898984	1	58.349	KV452709.1	4528251	1-scf00009	1
AX-157898985	1	58.349	KV452709.1	4528548	1-scf00009	1
AX-157898986	1	58.349	KV452709.1	4531242	1-scf00009	1
AX-157898992	1	58.349	KV452709.1	4536562	1-scf00009	1
AX-157868682	1	58.349	KV452709.1	4538913	1-scf00009	1
AX-157811216	1	58.349	KV452709.1	4541750	1-scf00009	1
AX-157898999	1	58.349	KV452709.1	4544463	1-scf00009	1
AX-157899001	1	58.349	KV452709.1	4545339	1-scf00009	1
AX-157899003	1	58.349	KV452709.1	4546523	1-scf00009	1
AX-157899005	1	58.349	KV452709.1	4548608	1-scf00009	1
AX-157899007	1	58.349	KV452709.1	4551689	1-scf00009	1
AX-157899029	1	58.349	KV452709.1	4567875	1-scf00009	1
AX-157899030	1	58.349	KV452709.1	4570235	1-scf00009	1
AX-157899032	1	58.349	KV452709.1	4571502	1-scf00009	1
AX-157899033	1	58.349	KV452709.1	4572272	1-scf00009	1
AX-157739479	1	58.349	KV452709.1	4573576	1-scf00009	1
AX-157899035	1	58.349	KV452709.1	4574203	1-scf00009	1
AX-157899036	1	58.349	KV452709.1	4574609	1-scf00009	1
AX-157811218	1	58.349	KV452709.1	4580420	1-scf00009	1
AX-157899043	1	58.349	KV452709.1	4588212	1-scf00009	1
AX-157899044	1	58.349	KV452709.1	4589456	1-scf00009	1
AX-157811227	1	58.349	KV452709.1	4600576	1-scf00009	1
AX-157899048	1	58.349	KV452709.1	4604583	1-scf00009	1
AX-157899053	1	58.349	KV452709.1	4609058	1-scf00009	1
AX-157899055	1	58.349	KV452709.1	4610554	1-scf00009	1
AX-157899056	1	58.349	KV452709.1	4611901	1-scf00009	1
AX-157899057	1	58.349	KV452709.1	4612440	1-scf00009	1
AX-157868684	1	58.349	KV452709.1	4614521	1-scf00009	1
AX-157899061	1	58.349	KV452709.1	4617523	1-scf00009	1
AX-157899062	1	58.349	KV452709.1	4618973	1-scf00009	1
AX-157899063	1	58.349	KV452709.1	4619077	1-scf00009	1
AX-157899064	1	58.349	KV452709.1	4619519	1-scf00009	1
AX-157899067	1	58.349	KV452709.1	4621142	1-scf00009	1
AX-157899070	1	58.349	KV452709.1	4627324	1-scf00009	1
AX-157899073	1	58.349	KV452709.1	4629782	1-scf00009	1
AX-157899076	1	58.349	KV452709.1	4633975	1-scf00009	1
AX-157899077	1	58.349	KV452709.1	4642180	1-scf00009	1
AX-157899080	1	58.349	KV452709.1	4645451	1-scf00009	1
AX-157811230	1	58.349	KV452709.1	4647181	1-scf00009	1
AX-157899092	1	58.349	KV452709.1	4657638	1-scf00009	1
AX-157811231	1	58.349	KV452709.1	4659274	1-scf00009	1
AX-157899098	1	58.349	KV452709.1	4665565	1-scf00009	1
AX-157899111	1	58.349	KV452709.1	4686351	1-scf00009	1
AX-157715046	1	58.349	KV452709.1	4693306	1-scf00009	1
AX-157899126	1	58.349	KV452709.1	4698653	1-scf00009	1
AX-157899308	1	58.349	KV452709.1	4994300	1-scf00009	1
AX-157899318	1	58.349	KV452709.1	5010057	1-scf00009	1
AX-157899334	1	58.349	KV452709.1	5038930	1-scf00009	1

AX-157899510	1	58.349	KV452709.1	5261114	1-scf00009	1
AX-157899559	1	58.349	KV452709.1	5314200	1-scf00009	1
AX-157811402	1	58.349	KV452709.1	6457731	1-scf00009	1
AX-157901840	1	58.349	KV452711.1	3440998	1-scf00010	1
AX-157902906	1	58.349	KV452713.1	1446261	1-scf00011	1
AX-157902926	1	58.349	KV452713.1	1478512	1-scf00011	1
AX-157903924	1	58.349	KV452713.1	2705109	1-scf00011	1
AX-158404683	1	58.349	KV452713.1	2705455	1-scf00011	1
AX-157903925	1	58.349	KV452713.1	2708496	1-scf00011	1
AX-157903926	1	58.349	KV452713.1	2710620	1-scf00011	1
AX-157903930	1	58.349	KV452713.1	2720942	1-scf00011	1
AX-157903932	1	58.349	KV452713.1	2723612	1-scf00011	1
AX-157903940	1	58.349	KV452713.1	2730986	1-scf00011	1
AX-157903944	1	58.349	KV452713.1	2735642	1-scf00011	1
AX-157903952	1	58.349	KV452713.1	2744755	1-scf00011	1
AX-157903958	1	58.349	KV452713.1	2750337	1-scf00011	1
AX-157903960	1	58.349	KV452713.1	2753147	1-scf00011	1
AX-157903963	1	58.349	KV452713.1	2756861	1-scf00011	1
AX-157903964	1	58.349	KV452713.1	2757399	1-scf00011	1
AX-157903966	1	58.349	KV452713.1	2759000	1-scf00011	1
AX-157903967	1	58.349	KV452713.1	2759759	1-scf00011	1
AX-157903968	1	58.349	KV452713.1	2760956	1-scf00011	1
AX-157811791	1	58.349	KV452713.1	2762721	1-scf00011	1
AX-157903970	1	58.349	KV452713.1	2764370	1-scf00011	1
AX-157903971	1	58.349	KV452713.1	2765389	1-scf00011	1
AX-157903972	1	58.349	KV452713.1	2766244	1-scf00011	1
AX-157903973	1	58.349	KV452713.1	2766899	1-scf00011	1
AX-157811792	1	58.349	KV452713.1	2767948	1-scf00011	1
AX-157903981	1	58.349	KV452713.1	2792170	1-scf00011	1
AX-158389445	1	58.349	KV452713.1	2797269	1-scf00011	1
AX-157904054	1	58.349	KV452713.1	2953267	1-scf00011	1
AX-157904544	1	58.349	KV452713.1	3934090	1-scf00011	1
AX-157904915	1	58.349	KV452713.1	4412637	1-scf00011	1
AX-157895701	1	58.458	KV452708.1	2868998	1-scf00008	1
AX-157810976	1	58.458	KV452709.1	1180586	1-scf00009	1
AX-157896976	1	58.458	KV452709.1	1187224	1-scf00009	1
AX-157897131	1	58.458	KV452709.1	1439124	1-scf00009	1
AX-157811000	1	58.458	KV452709.1	1573405	1-scf00009	1
AX-157897389	1	58.458	KV452709.1	1804623	1-scf00009	1
AX-157897735	1	58.458	KV452709.1	2350388	1-scf00009	1
AX-157897879	1	58.458	KV452709.1	2566083	1-scf00009	1
AX-157897902	1	58.458	KV452709.1	2602419	1-scf00009	1
AX-157897961	1	58.458	KV452709.1	2720948	1-scf00009	1
AX-157898056	1	58.458	KV452709.1	2873447	1-scf00009	1
AX-157898188	1	58.458	KV452709.1	3179095	1-scf00009	1
AX-158403920	1	58.458	KV452709.1	3233767	1-scf00009	1
AX-157898231	1	58.458	KV452709.1	3267399	1-scf00009	1
AX-157898270	1	58.458	KV452709.1	3327746	1-scf00009	1
AX-157898271	1	58.458	KV452709.1	3330567	1-scf00009	1
AX-157898277	1	58.458	KV452709.1	3339049	1-scf00009	1
AX-157898278	1	58.458	KV452709.1	3340595	1-scf00009	1

AX-157898468	1	58.458	KV452709.1	3772304	1-scf00009	1
AX-157898470	1	58.458	KV452709.1	3785888	1-scf00009	1
AX-157898471	1	58.458	KV452709.1	3788399	1-scf00009	1
AX-158381998	1	58.458	KV452709.1	3831011	1-scf00009	1
AX-157898721	1	58.458	KV452709.1	4158850	1-scf00009	1
AX-157738585	1	58.458	KV452709.1	4176836	1-scf00009	1
AX-157898822	1	58.458	KV452709.1	4312520	1-scf00009	1
AX-157898846	1	58.458	KV452709.1	4356736	1-scf00009	1
AX-157898848	1	58.458	KV452709.1	4358777	1-scf00009	1
AX-157898855	1	58.458	KV452709.1	4368440	1-scf00009	1
AX-158404469	1	58.458	KV452709.1	4370155	1-scf00009	1
AX-157898861	1	58.458	KV452709.1	4375181	1-scf00009	1
AX-157898863	1	58.458	KV452709.1	4377013	1-scf00009	1
AX-157898864	1	58.458	KV452709.1	4377888	1-scf00009	1
AX-157750545	1	58.458	KV452709.1	4396389	1-scf00009	1
AX-157898879	1	58.458	KV452709.1	4403268	1-scf00009	1
AX-157898882	1	58.458	KV452709.1	4406725	1-scf00009	1
AX-157898894	1	58.458	KV452709.1	4417992	1-scf00009	1
AX-157811210	1	58.458	KV452709.1	4438591	1-scf00009	1
AX-157811211	1	58.458	KV452709.1	4439174	1-scf00009	1
AX-157898913	1	58.458	KV452709.1	4448240	1-scf00009	1
AX-157898916	1	58.458	KV452709.1	4449866	1-scf00009	1
AX-157898918	1	58.458	KV452709.1	4451633	1-scf00009	1
AX-157898928	1	58.458	KV452709.1	4461621	1-scf00009	1
AX-157898929	1	58.458	KV452709.1	4462335	1-scf00009	1
AX-157898933	1	58.458	KV452709.1	4465853	1-scf00009	1
AX-157898936	1	58.458	KV452709.1	4470139	1-scf00009	1
AX-157898937	1	58.458	KV452709.1	4470454	1-scf00009	1
AX-157754333	1	58.458	KV452709.1	4471619	1-scf00009	1
AX-157898947	1	58.458	KV452709.1	4485365	1-scf00009	1
AX-157898958	1	58.458	KV452709.1	4494193	1-scf00009	1
AX-157898960	1	58.458	KV452709.1	4495372	1-scf00009	1
AX-157898967	1	58.458	KV452709.1	4502201	1-scf00009	1
AX-157898969	1	58.458	KV452709.1	4506346	1-scf00009	1
AX-157883117	1	58.458	KV452709.1	4507160	1-scf00009	1
AX-157898971	1	58.458	KV452709.1	4511368	1-scf00009	1
AX-158389219	1	58.458	KV452709.1	4519292	1-scf00009	1
AX-157898979	1	58.458	KV452709.1	4521666	1-scf00009	1
AX-157811215	1	58.458	KV452709.1	4541584	1-scf00009	1
AX-157899008	1	58.458	KV452709.1	4552208	1-scf00009	1
AX-157899010	1	58.458	KV452709.1	4553887	1-scf00009	1
AX-157899012	1	58.458	KV452709.1	4555334	1-scf00009	1
AX-157899013	1	58.458	KV452709.1	4555979	1-scf00009	1
AX-157899015	1	58.458	KV452709.1	4557185	1-scf00009	1
AX-157899019	1	58.458	KV452709.1	4559137	1-scf00009	1
AX-157899022	1	58.458	KV452709.1	4561257	1-scf00009	1
AX-157695998	1	58.458	KV452709.1	4565817	1-scf00009	1
AX-157899028	1	58.458	KV452709.1	4567489	1-scf00009	1
AX-157899031	1	58.458	KV452709.1	4570961	1-scf00009	1
AX-157899037	1	58.458	KV452709.1	4575408	1-scf00009	1
AX-157811219	1	58.458	KV452709.1	4584599	1-scf00009	1

AX-157747063	1	58.458	KV452709.1	4590178	1-scf00009	1
AX-157899046	1	58.458	KV452709.1	4603088	1-scf00009	1
AX-158389220	1	58.458	KV452709.1	4611296	1-scf00009	1
AX-157899058	1	58.458	KV452709.1	4615510	1-scf00009	1
AX-157899059	1	58.458	KV452709.1	4616092	1-scf00009	1
AX-157811228	1	58.458	KV452709.1	4622981	1-scf00009	1
AX-158389222	1	58.458	KV452709.1	4623512	1-scf00009	1
AX-157899069	1	58.458	KV452709.1	4624534	1-scf00009	1
AX-157899071	1	58.458	KV452709.1	4628276	1-scf00009	1
AX-157899072	1	58.458	KV452709.1	4629139	1-scf00009	1
AX-157899081	1	58.458	KV452709.1	4645671	1-scf00009	1
AX-157811229	1	58.458	KV452709.1	4647142	1-scf00009	1
AX-157770993	1	58.458	KV452709.1	4648313	1-scf00009	1
AX-157899085	1	58.458	KV452709.1	4648810	1-scf00009	1
AX-157899086	1	58.458	KV452709.1	4650324	1-scf00009	1
AX-157899091	1	58.458	KV452709.1	4657171	1-scf00009	1
AX-157899096	1	58.458	KV452709.1	4663268	1-scf00009	1
AX-157811236	1	58.458	KV452709.1	4664658	1-scf00009	1
AX-157899099	1	58.458	KV452709.1	4668594	1-scf00009	1
AX-157713644	1	58.458	KV452709.1	4684968	1-scf00009	1
AX-157899110	1	58.458	KV452709.1	4685637	1-scf00009	1
AX-157899113	1	58.458	KV452709.1	4690567	1-scf00009	1
AX-157899117	1	58.458	KV452709.1	4693090	1-scf00009	1
AX-157899131	1	58.458	KV452709.1	4711962	1-scf00009	1
AX-157899137	1	58.458	KV452709.1	4723637	1-scf00009	1
AX-157899163	1	58.458	KV452709.1	4775906	1-scf00009	1
AX-157811251	1	58.458	KV452709.1	4805733	1-scf00009	1
AX-157899184	1	58.458	KV452709.1	4808129	1-scf00009	1
AX-157874371	1	58.458	KV452709.1	4818336	1-scf00009	1
AX-157874372	1	58.458	KV452709.1	4824583	1-scf00009	1
AX-157899198	1	58.458	KV452709.1	4826180	1-scf00009	1
AX-157899221	1	58.458	KV452709.1	4878978	1-scf00009	1
AX-157713677	1	58.458	KV452709.1	5749464	1-scf00009	1
AX-157900200	1	58.458	KV452709.1	6242379	1-scf00009	1
AX-157900201	1	58.458	KV452709.1	6243891	1-scf00009	1
AX-157900218	1	58.458	KV452709.1	6270195	1-scf00009	1
AX-157901069	1	58.458	KV452711.1	1358560	1-scf00010	1
AX-157901329	1	58.458	KV452711.1	2027012	1-scf00010	1
AX-157901392	1	58.458	KV452711.1	2218627	1-scf00010	1
AX-157901408	1	58.458	KV452711.1	2241467	1-scf00010	1
AX-157901434	1	58.458	KV452711.1	2338013	1-scf00010	1
AX-157901485	1	58.458	KV452711.1	2534177	1-scf00010	1
AX-157751271	1	58.458	KV452711.1	2710668	1-scf00010	1
AX-157901717	1	58.458	KV452711.1	3079941	1-scf00010	1
AX-157902453	1	58.458	KV452713.1	763911	1-scf00011	1
AX-157902454	1	58.458	KV452713.1	764702	1-scf00011	1
AX-157902591	1	58.458	KV452713.1	994063	1-scf00011	1
AX-158382037	1	58.458	KV452713.1	1072703	1-scf00011	1
AX-157682297	1	58.458	3ML01033942	190	scf09611_1	UN
AX-157899116	1	58.487	KV452709.1	4692242	1-scf00009	1
AX-157899128	1	58.529	KV452709.1	4700577	1-scf00009	1

AX-157894996	1	58.568	KV452708.1	1701764	1-scf00008	1
AX-157898151	1	58.568	KV452709.1	3086006	1-scf00009	1
AX-157898488	1	58.568	KV452709.1	3831432	1-scf00009	1
AX-157715204	1	58.568	KV452709.1	4352586	1-scf00009	1
AX-157898952	1	58.568	KV452709.1	4488780	1-scf00009	1
AX-157899093	1	58.568	KV452709.1	4659543	1-scf00009	1
AX-157899124	1	58.568	KV452709.1	4696165	1-scf00009	1
AX-157899134	1	58.568	KV452709.1	4716173	1-scf00009	1
AX-157899140	1	58.568	KV452709.1	4728947	1-scf00009	1
AX-157899147	1	58.568	KV452709.1	4742860	1-scf00009	1
AX-157899158	1	58.568	KV452709.1	4767635	1-scf00009	1
AX-157899159	1	58.568	KV452709.1	4768192	1-scf00009	1
AX-157899161	1	58.568	KV452709.1	4770371	1-scf00009	1
AX-157899162	1	58.568	KV452709.1	4772034	1-scf00009	1
AX-157899164	1	58.568	KV452709.1	4777355	1-scf00009	1
AX-157899170	1	58.568	KV452709.1	4783348	1-scf00009	1
AX-157899172	1	58.568	KV452709.1	4786973	1-scf00009	1
AX-157708938	1	58.568	KV452709.1	4788907	1-scf00009	1
AX-157899173	1	58.568	KV452709.1	4789665	1-scf00009	1
AX-157899179	1	58.568	KV452709.1	4801355	1-scf00009	1
AX-157772342	1	58.568	KV452709.1	4802128	1-scf00009	1
AX-157899182	1	58.568	KV452709.1	4802588	1-scf00009	1
AX-157811250	1	58.568	KV452709.1	4803221	1-scf00009	1
AX-157811252	1	58.568	KV452709.1	4810763	1-scf00009	1
AX-157694034	1	58.568	KV452709.1	4816687	1-scf00009	1
AX-157811253	1	58.568	KV452709.1	4822614	1-scf00009	1
AX-157738728	1	58.568	KV452709.1	4866071	1-scf00009	1
AX-157899212	1	58.568	KV452709.1	4873459	1-scf00009	1
AX-157899216	1	58.568	KV452709.1	4875499	1-scf00009	1
AX-157899222	1	58.568	KV452709.1	4879645	1-scf00009	1
AX-157758621	1	58.568	KV452709.1	4881042	1-scf00009	1
AX-157899241	1	58.568	KV452709.1	4913028	1-scf00009	1
AX-157755647	1	58.568	KV452709.1	5296744	1-scf00009	1
AX-157811474	1	58.568	KV452711.1	1992316	1-scf00010	1
AX-157902637	1	58.568	KV452713.1	1051589	1-scf00011	1
AX-157902660	1	58.568	KV452713.1	1085824	1-scf00011	1
AX-157902910	1	58.568	KV452713.1	1450933	1-scf00011	1
AX-157756922	1	58.568	KV452713.1	1593732	1-scf00011	1
AX-157903775	1	58.568	KV452713.1	2534677	1-scf00011	1
AX-157904535	1	58.568	KV452713.1	3908332	1-scf00011	1
AX-157904537	1	58.568	KV452713.1	3913275	1-scf00011	1
AX-157899224	1	58.62	KV452709.1	4883474	1-scf00009	1
AX-157883121	1	58.677	KV452709.1	4887916	1-scf00009	1
AX-157899230	1	58.677	KV452709.1	4891943	1-scf00009	1
AX-157899234	1	58.71	KV452709.1	4895573	1-scf00009	1
AX-157740114	1	58.753	KV452709.1	4901005	1-scf00009	1
AX-157897467	1	58.786	KV452709.1	1925549	1-scf00009	1
AX-157897796	1	58.786	KV452709.1	2452421	1-scf00009	1
AX-157899214	1	58.786	KV452709.1	4874405	1-scf00009	1
AX-157899228	1	58.786	KV452709.1	4887095	1-scf00009	1
AX-157811259	1	58.786	KV452709.1	4900267	1-scf00009	1

AX-157811261	1	58.786	KV452709.1	4902183	1-scf00009	1
AX-157899235	1	58.786	KV452709.1	4902937	1-scf00009	1
AX-157868686	1	58.786	KV452709.1	4908713	1-scf00009	1
AX-157899239	1	58.786	KV452709.1	4909602	1-scf00009	1
AX-157899240	1	58.786	KV452709.1	4912622	1-scf00009	1
AX-157899246	1	58.786	KV452709.1	4917437	1-scf00009	1
AX-157899249	1	58.786	KV452709.1	4919967	1-scf00009	1
AX-157899250	1	58.786	KV452709.1	4920822	1-scf00009	1
AX-157899253	1	58.786	KV452709.1	4923968	1-scf00009	1
AX-157747416	1	58.786	KV452709.1	4938029	1-scf00009	1
AX-157899267	1	58.786	KV452709.1	4943859	1-scf00009	1
AX-157899268	1	58.786	KV452709.1	4949710	1-scf00009	1
AX-157811267	1	58.786	KV452709.1	4951178	1-scf00009	1
AX-157899272	1	58.786	KV452709.1	4953719	1-scf00009	1
AX-157811268	1	58.786	KV452709.1	4956774	1-scf00009	1
AX-157746741	1	58.786	KV452709.1	4956899	1-scf00009	1
AX-157899279	1	58.786	KV452709.1	4964345	1-scf00009	1
AX-157899282	1	58.786	KV452709.1	4969043	1-scf00009	1
AX-157899283	1	58.786	KV452709.1	4969387	1-scf00009	1
AX-157899284	1	58.786	KV452709.1	4969740	1-scf00009	1
AX-157899290	1	58.786	KV452709.1	4976437	1-scf00009	1
AX-157899292	1	58.786	KV452709.1	4977546	1-scf00009	1
AX-157899296	1	58.786	KV452709.1	4979242	1-scf00009	1
AX-157899304	1	58.786	KV452709.1	4989502	1-scf00009	1
AX-157899311	1	58.786	KV452709.1	4997477	1-scf00009	1
AX-157899316	1	58.786	KV452709.1	5007523	1-scf00009	1
AX-157899327	1	58.786	KV452709.1	5022998	1-scf00009	1
AX-157899335	1	58.786	KV452709.1	5039665	1-scf00009	1
AX-157725602	1	58.786	KV452709.1	5045779	1-scf00009	1
AX-157811272	1	58.786	KV452709.1	5060111	1-scf00009	1
AX-157899355	1	58.786	KV452709.1	5073610	1-scf00009	1
AX-157899371	1	58.786	KV452709.1	5098624	1-scf00009	1
AX-157899623	1	58.786	KV452709.1	5397969	1-scf00009	1
AX-157727204	1	58.786	KV452709.1	5505928	1-scf00009	1
AX-157899826	1	58.786	KV452709.1	5669191	1-scf00009	1
AX-157811341	1	58.786	KV452709.1	5730979	1-scf00009	1
AX-157896918	1	58.895	KV452709.1	1100935	1-scf00009	1
AX-157810977	1	58.895	KV452709.1	1188993	1-scf00009	1
AX-157897017	1	58.895	KV452709.1	1281595	1-scf00009	1
AX-157704131	1	58.895	KV452709.1	1469824	1-scf00009	1
AX-157811005	1	58.895	KV452709.1	1647649	1-scf00009	1
AX-157897466	1	58.895	KV452709.1	1924999	1-scf00009	1
AX-157897585	1	58.895	KV452709.1	2118101	1-scf00009	1
AX-157897588	1	58.895	KV452709.1	2120193	1-scf00009	1
AX-157897634	1	58.895	KV452709.1	2192087	1-scf00009	1
AX-157898597	1	58.895	KV452709.1	3979917	1-scf00009	1
AX-157898968	1	58.895	KV452709.1	4504497	1-scf00009	1
AX-157723308	1	58.895	KV452709.1	4699756	1-scf00009	1
AX-157899133	1	58.895	KV452709.1	4714940	1-scf00009	1
AX-157899174	1	58.895	KV452709.1	4791401	1-scf00009	1
AX-157899223	1	58.895	KV452709.1	4882918	1-scf00009	1

AX-157899227	1	58.895	KV452709.1	4885703	1-scf00009	1
AX-157899237	1	58.895	KV452709.1	4906292	1-scf00009	1
AX-157853584	1	58.895	KV452709.1	4917796	1-scf00009	1
AX-157899294	1	58.895	KV452709.1	4978279	1-scf00009	1
AX-157899366	1	58.895	KV452709.1	5095273	1-scf00009	1
AX-157899368	1	58.895	KV452709.1	5096969	1-scf00009	1
AX-157899369	1	58.895	KV452709.1	5097524	1-scf00009	1
AX-157899370	1	58.895	KV452709.1	5097730	1-scf00009	1
AX-157899372	1	58.895	KV452709.1	5099597	1-scf00009	1
AX-157899373	1	58.895	KV452709.1	5100060	1-scf00009	1
AX-157883122	1	58.895	KV452709.1	5102109	1-scf00009	1
AX-157899374	1	58.895	KV452709.1	5102818	1-scf00009	1
AX-157899375	1	58.895	KV452709.1	5103288	1-scf00009	1
AX-157899376	1	58.895	KV452709.1	5103765	1-scf00009	1
AX-157899377	1	58.895	KV452709.1	5105455	1-scf00009	1
AX-157899378	1	58.895	KV452709.1	5106112	1-scf00009	1
AX-158389228	1	58.895	KV452709.1	5106540	1-scf00009	1
AX-157899379	1	58.895	KV452709.1	5106664	1-scf00009	1
AX-157899380	1	58.895	KV452709.1	5107505	1-scf00009	1
AX-157899381	1	58.895	KV452709.1	5108190	1-scf00009	1
AX-157811277	1	58.895	KV452709.1	5108653	1-scf00009	1
AX-157899383	1	58.895	KV452709.1	5110686	1-scf00009	1
AX-157899384	1	58.895	KV452709.1	5111175	1-scf00009	1
AX-157899385	1	58.895	KV452709.1	5111732	1-scf00009	1
AX-157899386	1	58.895	KV452709.1	5112416	1-scf00009	1
AX-157899387	1	58.895	KV452709.1	5113210	1-scf00009	1
AX-157899388	1	58.895	KV452709.1	5113564	1-scf00009	1
AX-157899389	1	58.895	KV452709.1	5113962	1-scf00009	1
AX-157899390	1	58.895	KV452709.1	5114772	1-scf00009	1
AX-157899391	1	58.895	KV452709.1	5115991	1-scf00009	1
AX-157899392	1	58.895	KV452709.1	5117224	1-scf00009	1
AX-157899393	1	58.895	KV452709.1	5118092	1-scf00009	1
AX-157899394	1	58.895	KV452709.1	5119680	1-scf00009	1
AX-157899395	1	58.895	KV452709.1	5120119	1-scf00009	1
AX-157899396	1	58.895	KV452709.1	5120645	1-scf00009	1
AX-157899397	1	58.895	KV452709.1	5121137	1-scf00009	1
AX-157899398	1	58.895	KV452709.1	5121658	1-scf00009	1
AX-157899399	1	58.895	KV452709.1	5122280	1-scf00009	1
AX-157899400	1	58.895	KV452709.1	5123353	1-scf00009	1
AX-157899401	1	58.895	KV452709.1	5124516	1-scf00009	1
AX-157899402	1	58.895	KV452709.1	5126760	1-scf00009	1
AX-157899403	1	58.895	KV452709.1	5128133	1-scf00009	1
AX-157899404	1	58.895	KV452709.1	5128545	1-scf00009	1
AX-158379148	1	58.895	KV452709.1	5128725	1-scf00009	1
AX-157899405	1	58.895	KV452709.1	5129247	1-scf00009	1
AX-157754560	1	58.895	KV452709.1	5129703	1-scf00009	1
AX-157899406	1	58.895	KV452709.1	5130981	1-scf00009	1
AX-157899408	1	58.895	KV452709.1	5132788	1-scf00009	1
AX-157899410	1	58.895	KV452709.1	5134582	1-scf00009	1
AX-157899412	1	58.895	KV452709.1	5138395	1-scf00009	1
AX-157899413	1	58.895	KV452709.1	5138970	1-scf00009	1

AX-157899414	1	58.895	KV452709.1	5141162	1-scf00009	1
AX-157899415	1	58.895	KV452709.1	5141415	1-scf00009	1
AX-157899416	1	58.895	KV452709.1	5141635	1-scf00009	1
AX-157772262	1	58.895	KV452709.1	5142305	1-scf00009	1
AX-157899419	1	58.895	KV452709.1	5145845	1-scf00009	1
AX-157700833	1	58.895	KV452709.1	5147173	1-scf00009	1
AX-157899420	1	58.895	KV452709.1	5147606	1-scf00009	1
AX-157811278	1	58.895	KV452709.1	5148771	1-scf00009	1
AX-157899421	1	58.895	KV452709.1	5148934	1-scf00009	1
AX-157899422	1	58.895	KV452709.1	5149720	1-scf00009	1
AX-157899423	1	58.895	KV452709.1	5151883	1-scf00009	1
AX-157811279	1	58.895	KV452709.1	5152680	1-scf00009	1
AX-157811280	1	58.895	KV452709.1	5154553	1-scf00009	1
AX-157899426	1	58.895	KV452709.1	5155677	1-scf00009	1
AX-157899427	1	58.895	KV452709.1	5158045	1-scf00009	1
AX-157899428	1	58.895	KV452709.1	5159721	1-scf00009	1
AX-157899429	1	58.895	KV452709.1	5159926	1-scf00009	1
AX-157811281	1	58.895	KV452709.1	5161313	1-scf00009	1
AX-157899430	1	58.895	KV452709.1	5162130	1-scf00009	1
AX-157811282	1	58.895	KV452709.1	5162728	1-scf00009	1
AX-157748906	1	58.895	KV452709.1	5163093	1-scf00009	1
AX-157899431	1	58.895	KV452709.1	5163393	1-scf00009	1
AX-157899432	1	58.895	KV452709.1	5165944	1-scf00009	1
AX-157899433	1	58.895	KV452709.1	5168182	1-scf00009	1
AX-157811283	1	58.895	KV452709.1	5168655	1-scf00009	1
AX-157899434	1	58.895	KV452709.1	5169220	1-scf00009	1
AX-157899435	1	58.895	KV452709.1	5170831	1-scf00009	1
AX-157899437	1	58.895	KV452709.1	5171776	1-scf00009	1
AX-157899438	1	58.895	KV452709.1	5175093	1-scf00009	1
AX-157899439	1	58.895	KV452709.1	5175546	1-scf00009	1
AX-157899440	1	58.895	KV452709.1	5178910	1-scf00009	1
AX-157899441	1	58.895	KV452709.1	5180456	1-scf00009	1
AX-157899444	1	58.895	KV452709.1	5182521	1-scf00009	1
AX-157899445	1	58.895	KV452709.1	5183425	1-scf00009	1
AX-157899447	1	58.895	KV452709.1	5189782	1-scf00009	1
AX-157899448	1	58.895	KV452709.1	5192747	1-scf00009	1
AX-157899449	1	58.895	KV452709.1	5195022	1-scf00009	1
AX-157899452	1	58.895	KV452709.1	5196693	1-scf00009	1
AX-157899454	1	58.895	KV452709.1	5197246	1-scf00009	1
AX-157899455	1	58.895	KV452709.1	5200296	1-scf00009	1
AX-157899456	1	58.895	KV452709.1	5201035	1-scf00009	1
AX-157899457	1	58.895	KV452709.1	5202478	1-scf00009	1
AX-157811284	1	58.895	KV452709.1	5203882	1-scf00009	1
AX-157899459	1	58.895	KV452709.1	5208563	1-scf00009	1
AX-157899460	1	58.895	KV452709.1	5209334	1-scf00009	1
AX-157899461	1	58.895	KV452709.1	5209631	1-scf00009	1
AX-157899462	1	58.895	KV452709.1	5209863	1-scf00009	1
AX-157899463	1	58.895	KV452709.1	5210892	1-scf00009	1
AX-157899464	1	58.895	KV452709.1	5211161	1-scf00009	1
AX-157811286	1	58.895	KV452709.1	5211553	1-scf00009	1
AX-157811287	1	58.895	KV452709.1	5211800	1-scf00009	1

AX-157899465	1	58.895	KV452709.1	5212038	1-scf00009	1
AX-157899468	1	58.895	KV452709.1	5216750	1-scf00009	1
AX-157899470	1	58.895	KV452709.1	5219820	1-scf00009	1
AX-157899471	1	58.895	KV452709.1	5220182	1-scf00009	1
AX-157811289	1	58.895	KV452709.1	5220485	1-scf00009	1
AX-157899472	1	58.895	KV452709.1	5220834	1-scf00009	1
AX-157899474	1	58.895	KV452709.1	5221758	1-scf00009	1
AX-157899475	1	58.895	KV452709.1	5222640	1-scf00009	1
AX-157899476	1	58.895	KV452709.1	5223158	1-scf00009	1
AX-157899478	1	58.895	KV452709.1	5227129	1-scf00009	1
AX-157899479	1	58.895	KV452709.1	5227687	1-scf00009	1
AX-157899480	1	58.895	KV452709.1	5228011	1-scf00009	1
AX-157899481	1	58.895	KV452709.1	5229279	1-scf00009	1
AX-157899482	1	58.895	KV452709.1	5229693	1-scf00009	1
AX-157899484	1	58.895	KV452709.1	5232997	1-scf00009	1
AX-157899485	1	58.895	KV452709.1	5233206	1-scf00009	1
AX-157899486	1	58.895	KV452709.1	5233683	1-scf00009	1
AX-157899488	1	58.895	KV452709.1	5235724	1-scf00009	1
AX-157757199	1	58.895	KV452709.1	5236135	1-scf00009	1
AX-157899490	1	58.895	KV452709.1	5238032	1-scf00009	1
AX-157899491	1	58.895	KV452709.1	5238536	1-scf00009	1
AX-157899492	1	58.895	KV452709.1	5240313	1-scf00009	1
AX-157899493	1	58.895	KV452709.1	5241778	1-scf00009	1
AX-157899494	1	58.895	KV452709.1	5243825	1-scf00009	1
AX-157899495	1	58.895	KV452709.1	5244170	1-scf00009	1
AX-157899496	1	58.895	KV452709.1	5244491	1-scf00009	1
AX-157899497	1	58.895	KV452709.1	5246769	1-scf00009	1
AX-157899498	1	58.895	KV452709.1	5247940	1-scf00009	1
AX-157773176	1	58.895	KV452709.1	5248201	1-scf00009	1
AX-157899501	1	58.895	KV452709.1	5249636	1-scf00009	1
AX-157899504	1	58.895	KV452709.1	5254125	1-scf00009	1
AX-158404481	1	58.895	KV452709.1	5254992	1-scf00009	1
AX-157899506	1	58.895	KV452709.1	5256859	1-scf00009	1
AX-158404482	1	58.895	KV452709.1	5257357	1-scf00009	1
AX-157757478	1	58.895	KV452709.1	5259741	1-scf00009	1
AX-157899511	1	58.895	KV452709.1	5262380	1-scf00009	1
AX-157899512	1	58.895	KV452709.1	5263162	1-scf00009	1
AX-157899513	1	58.895	KV452709.1	5263977	1-scf00009	1
AX-157899514	1	58.895	KV452709.1	5266288	1-scf00009	1
AX-157899515	1	58.895	KV452709.1	5268700	1-scf00009	1
AX-157899516	1	58.895	KV452709.1	5269653	1-scf00009	1
AX-157899518	1	58.895	KV452709.1	5270776	1-scf00009	1
AX-157899519	1	58.895	KV452709.1	5271308	1-scf00009	1
AX-157899522	1	58.895	KV452709.1	5274080	1-scf00009	1
AX-157899523	1	58.895	KV452709.1	5274713	1-scf00009	1
AX-157899526	1	58.895	KV452709.1	5276486	1-scf00009	1
AX-157899527	1	58.895	KV452709.1	5276988	1-scf00009	1
AX-157899528	1	58.895	KV452709.1	5277928	1-scf00009	1
AX-157899529	1	58.895	KV452709.1	5280289	1-scf00009	1
AX-157899532	1	58.895	KV452709.1	5286527	1-scf00009	1
AX-157899533	1	58.895	KV452709.1	5287009	1-scf00009	1

AX-157899534	1	58.895	KV452709.1	5287570	1-scf00009	1
AX-157899535	1	58.895	KV452709.1	5287894	1-scf00009	1
AX-157811292	1	58.895	KV452709.1	5288566	1-scf00009	1
AX-157899538	1	58.895	KV452709.1	5289289	1-scf00009	1
AX-157883123	1	58.895	KV452709.1	5290276	1-scf00009	1
AX-157899539	1	58.895	KV452709.1	5290667	1-scf00009	1
AX-157899540	1	58.895	KV452709.1	5291155	1-scf00009	1
AX-157899541	1	58.895	KV452709.1	5291816	1-scf00009	1
AX-157899542	1	58.895	KV452709.1	5292657	1-scf00009	1
AX-157899544	1	58.895	KV452709.1	5294894	1-scf00009	1
AX-157899545	1	58.895	KV452709.1	5296105	1-scf00009	1
AX-157899550	1	58.895	KV452709.1	5302584	1-scf00009	1
AX-157899556	1	58.895	KV452709.1	5307371	1-scf00009	1
AX-157754642	1	58.895	KV452709.1	5308669	1-scf00009	1
AX-157899565	1	58.895	KV452709.1	5320786	1-scf00009	1
AX-157899572	1	58.895	KV452709.1	5327116	1-scf00009	1
AX-157899575	1	58.895	KV452709.1	5329782	1-scf00009	1
AX-157722572	1	58.895	KV452709.1	5339657	1-scf00009	1
AX-157811294	1	58.895	KV452709.1	5341647	1-scf00009	1
AX-157899582	1	58.895	KV452709.1	5341890	1-scf00009	1
AX-157899584	1	58.895	KV452709.1	5344395	1-scf00009	1
AX-157899593	1	58.895	KV452709.1	5355201	1-scf00009	1
AX-157899603	1	58.895	KV452709.1	5367179	1-scf00009	1
AX-158379150	1	58.895	KV452709.1	5386655	1-scf00009	1
AX-157811296	1	58.895	KV452709.1	5391689	1-scf00009	1
AX-157811297	1	58.895	KV452709.1	5395452	1-scf00009	1
AX-157745196	1	58.895	KV452709.1	5404551	1-scf00009	1
AX-157811299	1	58.895	KV452709.1	5414063	1-scf00009	1
AX-157899634	1	58.895	KV452709.1	5415724	1-scf00009	1
AX-157899635	1	58.895	KV452709.1	5416516	1-scf00009	1
AX-157899644	1	58.895	KV452709.1	5425731	1-scf00009	1
AX-157811301	1	58.895	KV452709.1	5427742	1-scf00009	1
AX-157811302	1	58.895	KV452709.1	5429557	1-scf00009	1
AX-157811303	1	58.895	KV452709.1	5430657	1-scf00009	1
AX-157899651	1	58.895	KV452709.1	5433384	1-scf00009	1
AX-157899653	1	58.895	KV452709.1	5435299	1-scf00009	1
AX-157899654	1	58.895	KV452709.1	5435666	1-scf00009	1
AX-157811304	1	58.895	KV452709.1	5435969	1-scf00009	1
AX-157899657	1	58.895	KV452709.1	5438391	1-scf00009	1
AX-157899659	1	58.895	KV452709.1	5443976	1-scf00009	1
AX-157899661	1	58.895	KV452709.1	5450436	1-scf00009	1
AX-157899663	1	58.895	KV452709.1	5454905	1-scf00009	1
AX-158389231	1	58.895	KV452709.1	5455170	1-scf00009	1
AX-157899664	1	58.895	KV452709.1	5455329	1-scf00009	1
AX-157811308	1	58.895	KV452709.1	5458613	1-scf00009	1
AX-157899669	1	58.895	KV452709.1	5459131	1-scf00009	1
AX-157899671	1	58.895	KV452709.1	5462344	1-scf00009	1
AX-157899672	1	58.895	KV452709.1	5462746	1-scf00009	1
AX-157899673	1	58.895	KV452709.1	5463110	1-scf00009	1
AX-157742501	1	58.895	KV452709.1	5465368	1-scf00009	1
AX-157899674	1	58.895	KV452709.1	5465969	1-scf00009	1

AX-157899675	1	58.895	KV452709.1	5466540	1-scf00009	1
AX-157899678	1	58.895	KV452709.1	5468804	1-scf00009	1
AX-157899681	1	58.895	KV452709.1	5471566	1-scf00009	1
AX-157899682	1	58.895	KV452709.1	5472016	1-scf00009	1
AX-157811309	1	58.895	KV452709.1	5474382	1-scf00009	1
AX-157899684	1	58.895	KV452709.1	5474537	1-scf00009	1
AX-157899685	1	58.895	KV452709.1	5474961	1-scf00009	1
AX-157899686	1	58.895	KV452709.1	5475292	1-scf00009	1
AX-157868690	1	58.895	KV452709.1	5476124	1-scf00009	1
AX-157899687	1	58.895	KV452709.1	5476620	1-scf00009	1
AX-157899688	1	58.895	KV452709.1	5477002	1-scf00009	1
AX-157899690	1	58.895	KV452709.1	5478937	1-scf00009	1
AX-157899691	1	58.895	KV452709.1	5479551	1-scf00009	1
AX-157899692	1	58.895	KV452709.1	5480103	1-scf00009	1
AX-157899693	1	58.895	KV452709.1	5480421	1-scf00009	1
AX-157811310	1	58.895	KV452709.1	5480858	1-scf00009	1
AX-157899695	1	58.895	KV452709.1	5481956	1-scf00009	1
AX-157899696	1	58.895	KV452709.1	5482901	1-scf00009	1
AX-157899697	1	58.895	KV452709.1	5483267	1-scf00009	1
AX-157811311	1	58.895	KV452709.1	5483386	1-scf00009	1
AX-157811312	1	58.895	KV452709.1	5484546	1-scf00009	1
AX-157899699	1	58.895	KV452709.1	5486173	1-scf00009	1
AX-157899701	1	58.895	KV452709.1	5487694	1-scf00009	1
AX-157899702	1	58.895	KV452709.1	5487969	1-scf00009	1
AX-157899703	1	58.895	KV452709.1	5489099	1-scf00009	1
AX-157899704	1	58.895	KV452709.1	5491408	1-scf00009	1
AX-157811313	1	58.895	KV452709.1	5492301	1-scf00009	1
AX-157899705	1	58.895	KV452709.1	5492630	1-scf00009	1
AX-157899708	1	58.895	KV452709.1	5499129	1-scf00009	1
AX-157811315	1	58.895	KV452709.1	5499410	1-scf00009	1
AX-157899709	1	58.895	KV452709.1	5500508	1-scf00009	1
AX-157899710	1	58.895	KV452709.1	5501703	1-scf00009	1
AX-157899711	1	58.895	KV452709.1	5502992	1-scf00009	1
AX-157899712	1	58.895	KV452709.1	5503597	1-scf00009	1
AX-157899713	1	58.895	KV452709.1	5503876	1-scf00009	1
AX-157899714	1	58.895	KV452709.1	5504590	1-scf00009	1
AX-157899716	1	58.895	KV452709.1	5508553	1-scf00009	1
AX-157899717	1	58.895	KV452709.1	5509221	1-scf00009	1
AX-157899718	1	58.895	KV452709.1	5509775	1-scf00009	1
AX-157899719	1	58.895	KV452709.1	5510371	1-scf00009	1
AX-158389232	1	58.895	KV452709.1	5511861	1-scf00009	1
AX-157899723	1	58.895	KV452709.1	5516716	1-scf00009	1
AX-157899724	1	58.895	KV452709.1	5517119	1-scf00009	1
AX-157811316	1	58.895	KV452709.1	5519741	1-scf00009	1
AX-157899725	1	58.895	KV452709.1	5520182	1-scf00009	1
AX-157811317	1	58.895	KV452709.1	5520979	1-scf00009	1
AX-157811318	1	58.895	KV452709.1	5521736	1-scf00009	1
AX-158389233	1	58.895	KV452709.1	5523936	1-scf00009	1
AX-157899726	1	58.895	KV452709.1	5524687	1-scf00009	1
AX-157899728	1	58.895	KV452709.1	5528366	1-scf00009	1
AX-157811319	1	58.895	KV452709.1	5528682	1-scf00009	1

AX-157899729	1	58.895	KV452709.1	5529336	1-scf00009	1
AX-157899730	1	58.895	KV452709.1	5530536	1-scf00009	1
AX-157811323	1	58.895	KV452709.1	5532850	1-scf00009	1
AX-157899731	1	58.895	KV452709.1	5533853	1-scf00009	1
AX-157899735	1	58.895	KV452709.1	5538267	1-scf00009	1
AX-157811324	1	58.895	KV452709.1	5544721	1-scf00009	1
AX-157899736	1	58.895	KV452709.1	5545513	1-scf00009	1
AX-157811325	1	58.895	KV452709.1	5548106	1-scf00009	1
AX-157811326	1	58.895	KV452709.1	5549461	1-scf00009	1
AX-157899740	1	58.895	KV452709.1	5553131	1-scf00009	1
AX-157899743	1	58.895	KV452709.1	5559418	1-scf00009	1
AX-157899744	1	58.895	KV452709.1	5560106	1-scf00009	1
AX-157899745	1	58.895	KV452709.1	5561946	1-scf00009	1
AX-157811328	1	58.895	KV452709.1	5563824	1-scf00009	1
AX-157811329	1	58.895	KV452709.1	5564575	1-scf00009	1
AX-157899748	1	58.895	KV452709.1	5566735	1-scf00009	1
AX-157899750	1	58.895	KV452709.1	5570487	1-scf00009	1
AX-157899752	1	58.895	KV452709.1	5574686	1-scf00009	1
AX-157899754	1	58.895	KV452709.1	5576951	1-scf00009	1
AX-157811332	1	58.895	KV452709.1	5579579	1-scf00009	1
AX-157899755	1	58.895	KV452709.1	5581968	1-scf00009	1
AX-157899756	1	58.895	KV452709.1	5582884	1-scf00009	1
AX-157899757	1	58.895	KV452709.1	5583471	1-scf00009	1
AX-157899758	1	58.895	KV452709.1	5583980	1-scf00009	1
AX-157811333	1	58.895	KV452709.1	5587200	1-scf00009	1
AX-157899759	1	58.895	KV452709.1	5589384	1-scf00009	1
AX-157899760	1	58.895	KV452709.1	5589741	1-scf00009	1
AX-157811334	1	58.895	KV452709.1	5590161	1-scf00009	1
AX-157811335	1	58.895	KV452709.1	5591952	1-scf00009	1
AX-157899761	1	58.895	KV452709.1	5592284	1-scf00009	1
AX-157729591	1	58.895	KV452709.1	5592365	1-scf00009	1
AX-157899762	1	58.895	KV452709.1	5593304	1-scf00009	1
AX-157899764	1	58.895	KV452709.1	5595758	1-scf00009	1
AX-157899765	1	58.895	KV452709.1	5599385	1-scf00009	1
AX-157899766	1	58.895	KV452709.1	5600858	1-scf00009	1
AX-157899767	1	58.895	KV452709.1	5601572	1-scf00009	1
AX-157899769	1	58.895	KV452709.1	5603078	1-scf00009	1
AX-157899770	1	58.895	KV452709.1	5603893	1-scf00009	1
AX-157708200	1	58.895	KV452709.1	5604111	1-scf00009	1
AX-157899771	1	58.895	KV452709.1	5604767	1-scf00009	1
AX-158404490	1	58.895	KV452709.1	5613281	1-scf00009	1
AX-157899774	1	58.895	KV452709.1	5615108	1-scf00009	1
AX-157899776	1	58.895	KV452709.1	5616573	1-scf00009	1
AX-157899778	1	58.895	KV452709.1	5617749	1-scf00009	1
AX-158389234	1	58.895	KV452709.1	5617959	1-scf00009	1
AX-157899780	1	58.895	KV452709.1	5619549	1-scf00009	1
AX-157899781	1	58.895	KV452709.1	5620271	1-scf00009	1
AX-157899783	1	58.895	KV452709.1	5621701	1-scf00009	1
AX-157899784	1	58.895	KV452709.1	5622367	1-scf00009	1
AX-157899787	1	58.895	KV452709.1	5624003	1-scf00009	1
AX-157899789	1	58.895	KV452709.1	5626101	1-scf00009	1

AX-157899790	1	58.895	KV452709.1	5626878	1-scf00009	1
AX-157899792	1	58.895	KV452709.1	5628460	1-scf00009	1
AX-157899793	1	58.895	KV452709.1	5632025	1-scf00009	1
AX-157899794	1	58.895	KV452709.1	5632818	1-scf00009	1
AX-157899795	1	58.895	KV452709.1	5634122	1-scf00009	1
AX-157899798	1	58.895	KV452709.1	5637712	1-scf00009	1
AX-157899799	1	58.895	KV452709.1	5638286	1-scf00009	1
AX-157899801	1	58.895	KV452709.1	5640080	1-scf00009	1
AX-157899803	1	58.895	KV452709.1	5640892	1-scf00009	1
AX-158389237	1	58.895	KV452709.1	5641314	1-scf00009	1
AX-157899804	1	58.895	KV452709.1	5643485	1-scf00009	1
AX-157899805	1	58.895	KV452709.1	5643866	1-scf00009	1
AX-157899806	1	58.895	KV452709.1	5645242	1-scf00009	1
AX-157899807	1	58.895	KV452709.1	5645613	1-scf00009	1
AX-157899808	1	58.895	KV452709.1	5645984	1-scf00009	1
AX-157899809	1	58.895	KV452709.1	5646533	1-scf00009	1
AX-157899810	1	58.895	KV452709.1	5647174	1-scf00009	1
AX-157899812	1	58.895	KV452709.1	5648568	1-scf00009	1
AX-158403944	1	58.895	KV452709.1	5651119	1-scf00009	1
AX-158403946	1	58.895	KV452709.1	5651274	1-scf00009	1
AX-158414614	1	58.895	KV452709.1	5651343	1-scf00009	1
AX-157899815	1	58.895	KV452709.1	5653899	1-scf00009	1
AX-157899817	1	58.895	KV452709.1	5655565	1-scf00009	1
AX-157899819	1	58.895	KV452709.1	5656769	1-scf00009	1
AX-157899820	1	58.895	KV452709.1	5657319	1-scf00009	1
AX-157899822	1	58.895	KV452709.1	5658833	1-scf00009	1
AX-157899823	1	58.895	KV452709.1	5662807	1-scf00009	1
AX-157899824	1	58.895	KV452709.1	5667148	1-scf00009	1
AX-157899825	1	58.895	KV452709.1	5668794	1-scf00009	1
AX-157899828	1	58.895	KV452709.1	5670437	1-scf00009	1
AX-157899829	1	58.895	KV452709.1	5671171	1-scf00009	1
AX-157899830	1	58.895	KV452709.1	5671662	1-scf00009	1
AX-157899831	1	58.895	KV452709.1	5671842	1-scf00009	1
AX-157899832	1	58.895	KV452709.1	5672296	1-scf00009	1
AX-157899833	1	58.895	KV452709.1	5672682	1-scf00009	1
AX-158389238	1	58.895	KV452709.1	5673189	1-scf00009	1
AX-157899834	1	58.895	KV452709.1	5673771	1-scf00009	1
AX-157899836	1	58.895	KV452709.1	5678577	1-scf00009	1
AX-157899837	1	58.895	KV452709.1	5678928	1-scf00009	1
AX-157862613	1	58.895	KV452709.1	5679225	1-scf00009	1
AX-157899838	1	58.895	KV452709.1	5681702	1-scf00009	1
AX-157899839	1	58.895	KV452709.1	5683115	1-scf00009	1
AX-157899840	1	58.895	KV452709.1	5683477	1-scf00009	1
AX-157899842	1	58.895	KV452709.1	5685833	1-scf00009	1
AX-157899843	1	58.895	KV452709.1	5686247	1-scf00009	1
AX-157899845	1	58.895	KV452709.1	5688160	1-scf00009	1
AX-157899846	1	58.895	KV452709.1	5689299	1-scf00009	1
AX-157899847	1	58.895	KV452709.1	5689713	1-scf00009	1
AX-157899850	1	58.895	KV452709.1	5692209	1-scf00009	1
AX-157899851	1	58.895	KV452709.1	5692717	1-scf00009	1
AX-157899852	1	58.895	KV452709.1	5693125	1-scf00009	1

AX-157868692	1	58.895	KV452709.1	5694128	1-scf00009	1
AX-157899853	1	58.895	KV452709.1	5697341	1-scf00009	1
AX-157899855	1	58.895	KV452709.1	5697995	1-scf00009	1
AX-157899857	1	58.895	KV452709.1	5700038	1-scf00009	1
AX-157899858	1	58.895	KV452709.1	5701405	1-scf00009	1
AX-157899859	1	58.895	KV452709.1	5701924	1-scf00009	1
AX-157899860	1	58.895	KV452709.1	5703326	1-scf00009	1
AX-157899861	1	58.895	KV452709.1	5703805	1-scf00009	1
AX-157899862	1	58.895	KV452709.1	5704180	1-scf00009	1
AX-157899863	1	58.895	KV452709.1	5704547	1-scf00009	1
AX-157899864	1	58.895	KV452709.1	5704985	1-scf00009	1
AX-157899865	1	58.895	KV452709.1	5705422	1-scf00009	1
AX-157695270	1	58.895	KV452709.1	5707246	1-scf00009	1
AX-157899866	1	58.895	KV452709.1	5708765	1-scf00009	1
AX-157899867	1	58.895	KV452709.1	5709229	1-scf00009	1
AX-157811340	1	58.895	KV452709.1	5710133	1-scf00009	1
AX-157899868	1	58.895	KV452709.1	5712306	1-scf00009	1
AX-157899870	1	58.895	KV452709.1	5714037	1-scf00009	1
AX-157899871	1	58.895	KV452709.1	5714540	1-scf00009	1
AX-157755719	1	58.895	KV452709.1	5714579	1-scf00009	1
AX-157899872	1	58.895	KV452709.1	5715221	1-scf00009	1
AX-157899876	1	58.895	KV452709.1	5718498	1-scf00009	1
AX-157899877	1	58.895	KV452709.1	5719664	1-scf00009	1
AX-157899878	1	58.895	KV452709.1	5720161	1-scf00009	1
AX-157899879	1	58.895	KV452709.1	5721159	1-scf00009	1
AX-157899882	1	58.895	KV452709.1	5722637	1-scf00009	1
AX-157899884	1	58.895	KV452709.1	5723479	1-scf00009	1
AX-157899885	1	58.895	KV452709.1	5724185	1-scf00009	1
AX-157899888	1	58.895	KV452709.1	5725729	1-scf00009	1
AX-157899889	1	58.895	KV452709.1	5727697	1-scf00009	1
AX-157899897	1	58.895	KV452709.1	5739428	1-scf00009	1
AX-157899935	1	58.895	KV452709.1	5791346	1-scf00009	1
AX-157899941	1	58.895	KV452709.1	5798837	1-scf00009	1
AX-157899946	1	58.895	KV452709.1	5805716	1-scf00009	1
AX-157811348	1	58.895	KV452709.1	5810823	1-scf00009	1
AX-157775849	1	58.895	KV452709.1	5834143	1-scf00009	1
AX-157811351	1	58.895	KV452709.1	5834581	1-scf00009	1
AX-158379152	1	58.895	KV452709.1	5835244	1-scf00009	1
AX-157717971	1	58.895	KV452709.1	5835317	1-scf00009	1
AX-157899960	1	58.895	KV452709.1	5836928	1-scf00009	1
AX-157727172	1	58.895	KV452709.1	5839890	1-scf00009	1
AX-157899964	1	58.895	KV452709.1	5841305	1-scf00009	1
AX-157899965	1	58.895	KV452709.1	5841948	1-scf00009	1
AX-157899967	1	58.895	KV452709.1	5843202	1-scf00009	1
AX-157868696	1	58.895	KV452709.1	5859495	1-scf00009	1
AX-157899984	1	58.895	KV452709.1	5865841	1-scf00009	1
AX-157899985	1	58.895	KV452709.1	5869222	1-scf00009	1
AX-157899986	1	58.895	KV452709.1	5870872	1-scf00009	1
AX-157899988	1	58.895	KV452709.1	5872132	1-scf00009	1
AX-157868697	1	58.895	KV452709.1	5872648	1-scf00009	1
AX-157899989	1	58.895	KV452709.1	5873910	1-scf00009	1

AX-157899990	1	58.895	KV452709.1	5874170	1-scf00009	1
AX-157868699	1	58.895	KV452709.1	5877846	1-scf00009	1
AX-157868700	1	58.895	KV452709.1	5879587	1-scf00009	1
AX-157773213	1	58.895	KV452709.1	5879646	1-scf00009	1
AX-157811354	1	58.895	KV452709.1	5880131	1-scf00009	1
AX-157899993	1	58.895	KV452709.1	5883197	1-scf00009	1
AX-157868702	1	58.895	KV452709.1	5885193	1-scf00009	1
AX-157899995	1	58.895	KV452709.1	5886396	1-scf00009	1
AX-157899997	1	58.895	KV452709.1	5887172	1-scf00009	1
AX-157899999	1	58.895	KV452709.1	5891767	1-scf00009	1
AX-157900000	1	58.895	KV452709.1	5892283	1-scf00009	1
AX-157868704	1	58.895	KV452709.1	5893369	1-scf00009	1
AX-157900002	1	58.895	KV452709.1	5894210	1-scf00009	1
AX-157900003	1	58.895	KV452709.1	5894909	1-scf00009	1
AX-157900009	1	58.895	KV452709.1	5913320	1-scf00009	1
AX-157900010	1	58.895	KV452709.1	5913528	1-scf00009	1
AX-158404501	1	58.895	KV452709.1	6048280	1-scf00009	1
AX-157900090	1	58.895	KV452709.1	6048940	1-scf00009	1
AX-158389262	1	58.895	KV452709.1	6050302	1-scf00009	1
AX-157900091	1	58.895	KV452709.1	6050703	1-scf00009	1
AX-157900092	1	58.895	KV452709.1	6051690	1-scf00009	1
AX-157900093	1	58.895	KV452709.1	6052597	1-scf00009	1
AX-157900094	1	58.895	KV452709.1	6056579	1-scf00009	1
AX-157900095	1	58.895	KV452709.1	6057336	1-scf00009	1
AX-157868720	1	58.895	KV452709.1	6058795	1-scf00009	1
AX-157900097	1	58.895	KV452709.1	6059731	1-scf00009	1
AX-157900099	1	58.895	KV452709.1	6062284	1-scf00009	1
AX-157900100	1	58.895	KV452709.1	6063433	1-scf00009	1
AX-157900101	1	58.895	KV452709.1	6065874	1-scf00009	1
AX-157900102	1	58.895	KV452709.1	6066673	1-scf00009	1
AX-158389263	1	58.895	KV452709.1	6069431	1-scf00009	1
AX-157900106	1	58.895	KV452709.1	6070314	1-scf00009	1
AX-158421801	1	58.895	KV452709.1	6071441	1-scf00009	1
AX-157900107	1	58.895	KV452709.1	6071865	1-scf00009	1
AX-158389264	1	58.895	KV452709.1	6074840	1-scf00009	1
AX-157900111	1	58.895	KV452709.1	6076268	1-scf00009	1
AX-157900112	1	58.895	KV452709.1	6078482	1-scf00009	1
AX-158389265	1	58.895	KV452709.1	6079440	1-scf00009	1
AX-158389267	1	58.895	KV452709.1	6087333	1-scf00009	1
AX-158389268	1	58.895	KV452709.1	6091179	1-scf00009	1
AX-157900118	1	58.895	KV452709.1	6091282	1-scf00009	1
AX-157900120	1	58.895	KV452709.1	6092339	1-scf00009	1
AX-157900123	1	58.895	KV452709.1	6094177	1-scf00009	1
AX-157900124	1	58.895	KV452709.1	6094580	1-scf00009	1
AX-158389269	1	58.895	KV452709.1	6095801	1-scf00009	1
AX-158389270	1	58.895	KV452709.1	6097554	1-scf00009	1
AX-157900128	1	58.895	KV452709.1	6104400	1-scf00009	1
AX-157811364	1	58.895	KV452709.1	6104622	1-scf00009	1
AX-157811370	1	58.895	KV452709.1	6138222	1-scf00009	1
AX-157868722	1	58.895	KV452709.1	6140511	1-scf00009	1
AX-157811372	1	58.895	KV452709.1	6144959	1-scf00009	1

AX-157868723	1	58.895	KV452709.1	6146778	1-scf00009	1
AX-157900154	1	58.895	KV452709.1	6154186	1-scf00009	1
AX-157868726	1	58.895	KV452709.1	6154669	1-scf00009	1
AX-157868727	1	58.895	KV452709.1	6154955	1-scf00009	1
AX-157900155	1	58.895	KV452709.1	6155205	1-scf00009	1
AX-157868728	1	58.895	KV452709.1	6156050	1-scf00009	1
AX-157811374	1	58.895	KV452709.1	6156588	1-scf00009	1
AX-157900158	1	58.895	KV452709.1	6160776	1-scf00009	1
AX-157900159	1	58.895	KV452709.1	6162361	1-scf00009	1
AX-157900160	1	58.895	KV452709.1	6165506	1-scf00009	1
AX-158389273	1	58.895	KV452709.1	6168170	1-scf00009	1
AX-157900161	1	58.895	KV452709.1	6169218	1-scf00009	1
AX-157811375	1	58.895	KV452709.1	6169626	1-scf00009	1
AX-157900162	1	58.895	KV452709.1	6171244	1-scf00009	1
AX-157900163	1	58.895	KV452709.1	6172854	1-scf00009	1
AX-157900164	1	58.895	KV452709.1	6173622	1-scf00009	1
AX-157900165	1	58.895	KV452709.1	6174734	1-scf00009	1
AX-158389274	1	58.895	KV452709.1	6175392	1-scf00009	1
AX-157900167	1	58.895	KV452709.1	6179667	1-scf00009	1
AX-158389275	1	58.895	KV452709.1	6180743	1-scf00009	1
AX-157868729	1	58.895	KV452709.1	6182201	1-scf00009	1
AX-157900168	1	58.895	KV452709.1	6183373	1-scf00009	1
AX-157900169	1	58.895	KV452709.1	6186883	1-scf00009	1
AX-157900170	1	58.895	KV452709.1	6188126	1-scf00009	1
AX-157900171	1	58.895	KV452709.1	6190221	1-scf00009	1
AX-157900173	1	58.895	KV452709.1	6192022	1-scf00009	1
AX-157900174	1	58.895	KV452709.1	6193136	1-scf00009	1
AX-157900179	1	58.895	KV452709.1	6200214	1-scf00009	1
AX-157811377	1	58.895	KV452709.1	6205480	1-scf00009	1
AX-157900182	1	58.895	KV452709.1	6209738	1-scf00009	1
AX-157900185	1	58.895	KV452709.1	6211724	1-scf00009	1
AX-158404511	1	58.895	KV452709.1	6212916	1-scf00009	1
AX-158404512	1	58.895	KV452709.1	6212972	1-scf00009	1
AX-157900186	1	58.895	KV452709.1	6213472	1-scf00009	1
AX-157868731	1	58.895	KV452709.1	6216055	1-scf00009	1
AX-157811379	1	58.895	KV452709.1	6216576	1-scf00009	1
AX-157868732	1	58.895	KV452709.1	6217270	1-scf00009	1
AX-157900189	1	58.895	KV452709.1	6223726	1-scf00009	1
AX-157900190	1	58.895	KV452709.1	6225631	1-scf00009	1
AX-157811381	1	58.895	KV452709.1	6226924	1-scf00009	1
AX-157811382	1	58.895	KV452709.1	6227814	1-scf00009	1
AX-157868734	1	58.895	KV452709.1	6228006	1-scf00009	1
AX-157868735	1	58.895	KV452709.1	6228941	1-scf00009	1
AX-157900194	1	58.895	KV452709.1	6231800	1-scf00009	1
AX-157900198	1	58.895	KV452709.1	6235796	1-scf00009	1
AX-157900202	1	58.895	KV452709.1	6251149	1-scf00009	1
AX-157900204	1	58.895	KV452709.1	6254087	1-scf00009	1
AX-157868737	1	58.895	KV452709.1	6260988	1-scf00009	1
AX-157900211	1	58.895	KV452709.1	6264221	1-scf00009	1
AX-157900212	1	58.895	KV452709.1	6267112	1-scf00009	1
AX-157900215	1	58.895	KV452709.1	6269326	1-scf00009	1

AX-157900219	1	58.895	KV452709.1	6271099	1-scf00009	1
AX-157900224	1	58.895	KV452709.1	6276718	1-scf00009	1
AX-157900225	1	58.895	KV452709.1	6277748	1-scf00009	1
AX-157900227	1	58.895	KV452709.1	6281054	1-scf00009	1
AX-157900229	1	58.895	KV452709.1	6283495	1-scf00009	1
AX-158389277	1	58.895	KV452709.1	6283565	1-scf00009	1
AX-157900230	1	58.895	KV452709.1	6284326	1-scf00009	1
AX-158389278	1	58.895	KV452709.1	6287291	1-scf00009	1
AX-157900239	1	58.895	KV452709.1	6296261	1-scf00009	1
AX-158389280	1	58.895	KV452709.1	6297908	1-scf00009	1
AX-157900242	1	58.895	KV452709.1	6298930	1-scf00009	1
AX-157900243	1	58.895	KV452709.1	6300874	1-scf00009	1
AX-157900244	1	58.895	KV452709.1	6302666	1-scf00009	1
AX-157853586	1	58.895	KV452709.1	6306281	1-scf00009	1
AX-157853587	1	58.895	KV452709.1	6310456	1-scf00009	1
AX-157811392	1	58.895	KV452709.1	6312020	1-scf00009	1
AX-158389282	1	58.895	KV452709.1	6314664	1-scf00009	1
AX-157811393	1	58.895	KV452709.1	6316378	1-scf00009	1
AX-157811394	1	58.895	KV452709.1	6321011	1-scf00009	1
AX-157900248	1	58.895	KV452709.1	6321267	1-scf00009	1
AX-157868739	1	58.895	KV452709.1	6326208	1-scf00009	1
AX-157811395	1	58.895	KV452709.1	6327501	1-scf00009	1
AX-157900249	1	58.895	KV452709.1	6331469	1-scf00009	1
AX-157811396	1	58.895	KV452709.1	6333469	1-scf00009	1
AX-157868741	1	58.895	KV452709.1	6335881	1-scf00009	1
AX-157900253	1	58.895	KV452709.1	6342027	1-scf00009	1
AX-157900254	1	58.895	KV452709.1	6342267	1-scf00009	1
AX-157868742	1	58.895	KV452709.1	6343173	1-scf00009	1
AX-157868744	1	58.895	KV452709.1	6347174	1-scf00009	1
AX-157900511	1	58.895	KV452709.1	7219349	1-scf00009	1
AX-157901058	1	58.895	KV452711.1	1344274	1-scf00010	1
AX-157811458	1	58.895	KV452711.1	1651382	1-scf00010	1
AX-157749268	1	58.895	KV452711.1	1653703	1-scf00010	1
AX-157901259	1	58.895	KV452711.1	1867364	1-scf00010	1
AX-157901285	1	58.895	KV452711.1	1912300	1-scf00010	1
AX-157901294	1	58.895	KV452711.1	1931566	1-scf00010	1
AX-157901296	1	58.895	KV452711.1	1935143	1-scf00010	1
AX-157811490	1	58.895	KV452711.1	2204019	1-scf00010	1
AX-157901390	1	58.895	KV452711.1	2204587	1-scf00010	1
AX-157902664	1	58.895	KV452713.1	1093916	1-scf00011	1
AX-157703587	1	58.895	DX099273.1	48	IORI212 Ictalu	UN
AX-157899994	1	58.926	KV452709.1	5883914	1-scf00009	1
AX-158404499	1	58.962	KV452709.1	5884352	1-scf00009	1
AX-157894533	1	59.005	KV452708.1	901667	1-scf00008	1
AX-157894586	1	59.005	KV452708.1	997671	1-scf00008	1
AX-157894656	1	59.005	KV452708.1	1107368	1-scf00008	1
AX-157894660	1	59.005	KV452708.1	1119659	1-scf00008	1
AX-157894672	1	59.005	KV452708.1	1133889	1-scf00008	1
AX-157810726	1	59.005	KV452708.1	1248509	1-scf00008	1
AX-157894730	1	59.005	KV452708.1	1275550	1-scf00008	1
AX-157894842	1	59.005	KV452708.1	1454959	1-scf00008	1

AX-157894843	1	59.005	KV452708.1	1456953	1-scf00008	1
AX-157810828	1	59.005	KV452708.1	2469824	1-scf00008	1
AX-157895451	1	59.005	KV452708.1	2549009	1-scf00008	1
AX-157895452	1	59.005	KV452708.1	2550160	1-scf00008	1
AX-157895664	1	59.005	KV452708.1	2818267	1-scf00008	1
AX-157895706	1	59.005	KV452708.1	2874766	1-scf00008	1
AX-158404359	1	59.005	KV452708.1	2954021	1-scf00008	1
AX-157895778	1	59.005	KV452708.1	2963651	1-scf00008	1
AX-157895780	1	59.005	KV452708.1	2964636	1-scf00008	1
AX-157895809	1	59.005	KV452708.1	2999349	1-scf00008	1
AX-157895821	1	59.005	KV452708.1	3012240	1-scf00008	1
AX-157895915	1	59.005	KV452708.1	3104858	1-scf00008	1
AX-157896069	1	59.005	KV452708.1	3275094	1-scf00008	1
AX-157896095	1	59.005	KV452709.1	6090	1-scf00009	1
AX-157743121	1	59.005	KV452709.1	13012	1-scf00009	1
AX-157896116	1	59.005	KV452709.1	37437	1-scf00009	1
AX-157896172	1	59.005	KV452709.1	122113	1-scf00009	1
AX-157897000	1	59.005	KV452709.1	1253288	1-scf00009	1
AX-157897030	1	59.005	KV452709.1	1297581	1-scf00009	1
AX-158404417	1	59.005	KV452709.1	1661253	1-scf00009	1
AX-157897604	1	59.005	KV452709.1	2148899	1-scf00009	1
AX-157897608	1	59.005	KV452709.1	2159741	1-scf00009	1
AX-157897617	1	59.005	KV452709.1	2167624	1-scf00009	1
AX-157897622	1	59.005	KV452709.1	2172819	1-scf00009	1
AX-157897625	1	59.005	KV452709.1	2175818	1-scf00009	1
AX-157897641	1	59.005	KV452709.1	2199780	1-scf00009	1
AX-157897665	1	59.005	KV452709.1	2235035	1-scf00009	1
AX-157897690	1	59.005	KV452709.1	2273074	1-scf00009	1
AX-157897773	1	59.005	KV452709.1	2420488	1-scf00009	1
AX-157897802	1	59.005	KV452709.1	2463001	1-scf00009	1
AX-157897835	1	59.005	KV452709.1	2504257	1-scf00009	1
AX-157897936	1	59.005	KV452709.1	2660393	1-scf00009	1
AX-157811079	1	59.005	KV452709.1	2664235	1-scf00009	1
AX-157744594	1	59.005	KV452709.1	2690787	1-scf00009	1
AX-157897987	1	59.005	KV452709.1	2761696	1-scf00009	1
AX-157696576	1	59.005	KV452709.1	2825046	1-scf00009	1
AX-157898040	1	59.005	KV452709.1	2848567	1-scf00009	1
AX-157898103	1	59.005	KV452709.1	2973371	1-scf00009	1
AX-157898266	1	59.005	KV452709.1	3322651	1-scf00009	1
AX-157898408	1	59.005	KV452709.1	3579620	1-scf00009	1
AX-157811166	1	59.005	KV452709.1	3865770	1-scf00009	1
AX-157898599	1	59.005	KV452709.1	3982151	1-scf00009	1
AX-157898657	1	59.005	KV452709.1	4075999	1-scf00009	1
AX-157898761	1	59.005	KV452709.1	4235137	1-scf00009	1
AX-157898796	1	59.005	KV452709.1	4274715	1-scf00009	1
AX-157898865	1	59.005	KV452709.1	4379047	1-scf00009	1
AX-157811207	1	59.005	KV452709.1	4398123	1-scf00009	1
AX-157811212	1	59.005	KV452709.1	4441622	1-scf00009	1
AX-157899130	1	59.005	KV452709.1	4706844	1-scf00009	1
AX-157742253	1	59.005	KV452709.1	4761872	1-scf00009	1
AX-157899165	1	59.005	KV452709.1	4779363	1-scf00009	1

AX-157899166	1	59.005	KV452709.1	4779746	1-scf00009	1
AX-157727651	1	59.005	KV452709.1	4849149	1-scf00009	1
AX-157899210	1	59.005	KV452709.1	4866510	1-scf00009	1
AX-157899254	1	59.005	KV452709.1	4925618	1-scf00009	1
AX-157899258	1	59.005	KV452709.1	4931488	1-scf00009	1
AX-157811270	1	59.005	KV452709.1	4981015	1-scf00009	1
AX-157899298	1	59.005	KV452709.1	4983113	1-scf00009	1
AX-157899356	1	59.005	KV452709.1	5076682	1-scf00009	1
AX-157899357	1	59.005	KV452709.1	5079803	1-scf00009	1
AX-157899358	1	59.005	KV452709.1	5080279	1-scf00009	1
AX-157811275	1	59.005	KV452709.1	5081645	1-scf00009	1
AX-157899359	1	59.005	KV452709.1	5082513	1-scf00009	1
AX-157899360	1	59.005	KV452709.1	5084737	1-scf00009	1
AX-157899361	1	59.005	KV452709.1	5086060	1-scf00009	1
AX-157899362	1	59.005	KV452709.1	5087446	1-scf00009	1
AX-157899363	1	59.005	KV452709.1	5087988	1-scf00009	1
AX-157899364	1	59.005	KV452709.1	5088672	1-scf00009	1
AX-157899365	1	59.005	KV452709.1	5094231	1-scf00009	1
AX-157899409	1	59.005	KV452709.1	5133789	1-scf00009	1
AX-157899436	1	59.005	KV452709.1	5171314	1-scf00009	1
AX-158389229	1	59.005	KV452709.1	5176783	1-scf00009	1
AX-157899443	1	59.005	KV452709.1	5181907	1-scf00009	1
AX-157811288	1	59.005	KV452709.1	5215117	1-scf00009	1
AX-157899473	1	59.005	KV452709.1	5221302	1-scf00009	1
AX-157899502	1	59.005	KV452709.1	5250472	1-scf00009	1
AX-157811291	1	59.005	KV452709.1	5264211	1-scf00009	1
AX-157899530	1	59.005	KV452709.1	5282145	1-scf00009	1
AX-157899547	1	59.005	KV452709.1	5299326	1-scf00009	1
AX-157899552	1	59.005	KV452709.1	5305010	1-scf00009	1
AX-157899553	1	59.005	KV452709.1	5305461	1-scf00009	1
AX-157899555	1	59.005	KV452709.1	5306672	1-scf00009	1
AX-157754148	1	59.005	KV452709.1	5309776	1-scf00009	1
AX-157899558	1	59.005	KV452709.1	5311401	1-scf00009	1
AX-157899560	1	59.005	KV452709.1	5315648	1-scf00009	1
AX-157899561	1	59.005	KV452709.1	5317793	1-scf00009	1
AX-157899567	1	59.005	KV452709.1	5321795	1-scf00009	1
AX-157899569	1	59.005	KV452709.1	5323070	1-scf00009	1
AX-157899570	1	59.005	KV452709.1	5323768	1-scf00009	1
AX-157899571	1	59.005	KV452709.1	5324676	1-scf00009	1
AX-157899573	1	59.005	KV452709.1	5327664	1-scf00009	1
AX-157899576	1	59.005	KV452709.1	5331058	1-scf00009	1
AX-157899577	1	59.005	KV452709.1	5331632	1-scf00009	1
AX-157899579	1	59.005	KV452709.1	5333647	1-scf00009	1
AX-157899580	1	59.005	KV452709.1	5334289	1-scf00009	1
AX-157899581	1	59.005	KV452709.1	5335603	1-scf00009	1
AX-157727037	1	59.005	KV452709.1	5340517	1-scf00009	1
AX-157899583	1	59.005	KV452709.1	5343252	1-scf00009	1
AX-157899585	1	59.005	KV452709.1	5345236	1-scf00009	1
AX-157899587	1	59.005	KV452709.1	5347198	1-scf00009	1
AX-157899588	1	59.005	KV452709.1	5348623	1-scf00009	1
AX-157899589	1	59.005	KV452709.1	5349085	1-scf00009	1

AX-157899590	1	59.005	KV452709.1	5352100	1-scf00009	1
AX-157899594	1	59.005	KV452709.1	5356449	1-scf00009	1
AX-157899595	1	59.005	KV452709.1	5357903	1-scf00009	1
AX-157899596	1	59.005	KV452709.1	5358913	1-scf00009	1
AX-157899597	1	59.005	KV452709.1	5359617	1-scf00009	1
AX-157899598	1	59.005	KV452709.1	5360129	1-scf00009	1
AX-157899599	1	59.005	KV452709.1	5361662	1-scf00009	1
AX-157899600	1	59.005	KV452709.1	5363006	1-scf00009	1
AX-157899601	1	59.005	KV452709.1	5365514	1-scf00009	1
AX-157899604	1	59.005	KV452709.1	5367599	1-scf00009	1
AX-157899605	1	59.005	KV452709.1	5369779	1-scf00009	1
AX-157899606	1	59.005	KV452709.1	5372181	1-scf00009	1
AX-157899608	1	59.005	KV452709.1	5375446	1-scf00009	1
AX-157899609	1	59.005	KV452709.1	5375933	1-scf00009	1
AX-157899611	1	59.005	KV452709.1	5379202	1-scf00009	1
AX-157899612	1	59.005	KV452709.1	5380712	1-scf00009	1
AX-157899614	1	59.005	KV452709.1	5382552	1-scf00009	1
AX-157899615	1	59.005	KV452709.1	5383213	1-scf00009	1
AX-157899617	1	59.005	KV452709.1	5386100	1-scf00009	1
AX-157899618	1	59.005	KV452709.1	5387441	1-scf00009	1
AX-157899619	1	59.005	KV452709.1	5391438	1-scf00009	1
AX-157899622	1	59.005	KV452709.1	5394759	1-scf00009	1
AX-157899625	1	59.005	KV452709.1	5401190	1-scf00009	1
AX-158389230	1	59.005	KV452709.1	5401851	1-scf00009	1
AX-157899626	1	59.005	KV452709.1	5402221	1-scf00009	1
AX-157899627	1	59.005	KV452709.1	5402550	1-scf00009	1
AX-157868687	1	59.005	KV452709.1	5408439	1-scf00009	1
AX-157899631	1	59.005	KV452709.1	5409783	1-scf00009	1
AX-157899633	1	59.005	KV452709.1	5414516	1-scf00009	1
AX-157899639	1	59.005	KV452709.1	5421797	1-scf00009	1
AX-157899642	1	59.005	KV452709.1	5423711	1-scf00009	1
AX-157811300	1	59.005	KV452709.1	5424130	1-scf00009	1
AX-157868688	1	59.005	KV452709.1	5424822	1-scf00009	1
AX-157899643	1	59.005	KV452709.1	5425222	1-scf00009	1
AX-157899645	1	59.005	KV452709.1	5426236	1-scf00009	1
AX-158404487	1	59.005	KV452709.1	5428235	1-scf00009	1
AX-157899650	1	59.005	KV452709.1	5429858	1-scf00009	1
AX-157811306	1	59.005	KV452709.1	5440745	1-scf00009	1
AX-157899660	1	59.005	KV452709.1	5445101	1-scf00009	1
AX-157899676	1	59.005	KV452709.1	5466997	1-scf00009	1
AX-157899679	1	59.005	KV452709.1	5469585	1-scf00009	1
AX-157899720	1	59.005	KV452709.1	5512027	1-scf00009	1
AX-157811320	1	59.005	KV452709.1	5528807	1-scf00009	1
AX-157811321	1	59.005	KV452709.1	5529728	1-scf00009	1
AX-157899734	1	59.005	KV452709.1	5537303	1-scf00009	1
AX-157899737	1	59.005	KV452709.1	5546115	1-scf00009	1
AX-157899739	1	59.005	KV452709.1	5551980	1-scf00009	1
AX-157899741	1	59.005	KV452709.1	5554473	1-scf00009	1
AX-157811331	1	59.005	KV452709.1	5578892	1-scf00009	1
AX-157899773	1	59.005	KV452709.1	5612414	1-scf00009	1
AX-158389235	1	59.005	KV452709.1	5620906	1-scf00009	1

AX-158379151	1	59.005	KV452709.1	5626511	1-scf00009	1
AX-157899791	1	59.005	KV452709.1	5627985	1-scf00009	1
AX-157899797	1	59.005	KV452709.1	5635952	1-scf00009	1
AX-157899811	1	59.005	KV452709.1	5647622	1-scf00009	1
AX-157899841	1	59.005	KV452709.1	5684732	1-scf00009	1
AX-157899844	1	59.005	KV452709.1	5687036	1-scf00009	1
AX-157899854	1	59.005	KV452709.1	5697596	1-scf00009	1
AX-157899856	1	59.005	KV452709.1	5698749	1-scf00009	1
AX-157864401	1	59.005	KV452709.1	5715015	1-scf00009	1
AX-157899883	1	59.005	KV452709.1	5723163	1-scf00009	1
AX-157752976	1	59.005	KV452709.1	5783968	1-scf00009	1
AX-157899966	1	59.005	KV452709.1	5842693	1-scf00009	1
AX-157727105	1	59.005	KV452709.1	5865711	1-scf00009	1
AX-157874381	1	59.005	KV452709.1	5893002	1-scf00009	1
AX-157900001	1	59.005	KV452709.1	5893819	1-scf00009	1
AX-157900005	1	59.005	KV452709.1	5896878	1-scf00009	1
AX-157811355	1	59.005	KV452709.1	5898775	1-scf00009	1
AX-157900008	1	59.005	KV452709.1	5911932	1-scf00009	1
AX-157900013	1	59.005	KV452709.1	5919975	1-scf00009	1
AX-157900018	1	59.005	KV452709.1	5930235	1-scf00009	1
AX-157900019	1	59.005	KV452709.1	5931129	1-scf00009	1
AX-157900022	1	59.005	KV452709.1	5935679	1-scf00009	1
AX-157900027	1	59.005	KV452709.1	5943321	1-scf00009	1
AX-157748279	1	59.005	KV452709.1	5946047	1-scf00009	1
AX-157811357	1	59.005	KV452709.1	5946235	1-scf00009	1
AX-158389247	1	59.005	KV452709.1	5947241	1-scf00009	1
AX-157900029	1	59.005	KV452709.1	5948005	1-scf00009	1
AX-157900030	1	59.005	KV452709.1	5948373	1-scf00009	1
AX-158389248	1	59.005	KV452709.1	5949034	1-scf00009	1
AX-157900031	1	59.005	KV452709.1	5949084	1-scf00009	1
AX-157900032	1	59.005	KV452709.1	5949639	1-scf00009	1
AX-157900034	1	59.005	KV452709.1	5952999	1-scf00009	1
AX-157900035	1	59.005	KV452709.1	5953756	1-scf00009	1
AX-157900036	1	59.005	KV452709.1	5959925	1-scf00009	1
AX-157900037	1	59.005	KV452709.1	5960385	1-scf00009	1
AX-158389249	1	59.005	KV452709.1	5962883	1-scf00009	1
AX-157900039	1	59.005	KV452709.1	5963794	1-scf00009	1
AX-157900040	1	59.005	KV452709.1	5964755	1-scf00009	1
AX-157900041	1	59.005	KV452709.1	5970306	1-scf00009	1
AX-157900042	1	59.005	KV452709.1	5971241	1-scf00009	1
AX-157900043	1	59.005	KV452709.1	5971537	1-scf00009	1
AX-158389250	1	59.005	KV452709.1	5978153	1-scf00009	1
AX-157900046	1	59.005	KV452709.1	5980027	1-scf00009	1
AX-158389251	1	59.005	KV452709.1	5981818	1-scf00009	1
AX-157900051	1	59.005	KV452709.1	5986706	1-scf00009	1
AX-157900052	1	59.005	KV452709.1	5987778	1-scf00009	1
AX-157900053	1	59.005	KV452709.1	5990240	1-scf00009	1
AX-157900054	1	59.005	KV452709.1	5993245	1-scf00009	1
AX-157900056	1	59.005	KV452709.1	5994567	1-scf00009	1
AX-158389252	1	59.005	KV452709.1	5995652	1-scf00009	1
AX-158389253	1	59.005	KV452709.1	5999423	1-scf00009	1

AX-158389254	1	59.005	KV452709.1	6001153	1-scf00009	1
AX-158389256	1	59.005	KV452709.1	6003905	1-scf00009	1
AX-157900062	1	59.005	KV452709.1	6004478	1-scf00009	1
AX-158389257	1	59.005	KV452709.1	6005957	1-scf00009	1
AX-158389258	1	59.005	KV452709.1	6008051	1-scf00009	1
AX-157900065	1	59.005	KV452709.1	6008223	1-scf00009	1
AX-157900066	1	59.005	KV452709.1	6008649	1-scf00009	1
AX-157900069	1	59.005	KV452709.1	6012631	1-scf00009	1
AX-158389260	1	59.005	KV452709.1	6015710	1-scf00009	1
AX-157900072	1	59.005	KV452709.1	6021776	1-scf00009	1
AX-157900074	1	59.005	KV452709.1	6024832	1-scf00009	1
AX-157900075	1	59.005	KV452709.1	6025746	1-scf00009	1
AX-157868708	1	59.005	KV452709.1	6027415	1-scf00009	1
AX-157811360	1	59.005	KV452709.1	6027662	1-scf00009	1
AX-157900077	1	59.005	KV452709.1	6027869	1-scf00009	1
AX-157900078	1	59.005	KV452709.1	6030283	1-scf00009	1
AX-157900079	1	59.005	KV452709.1	6032403	1-scf00009	1
AX-157868709	1	59.005	KV452709.1	6033871	1-scf00009	1
AX-157868710	1	59.005	KV452709.1	6034795	1-scf00009	1
AX-157900082	1	59.005	KV452709.1	6035209	1-scf00009	1
AX-157900083	1	59.005	KV452709.1	6036188	1-scf00009	1
AX-157900084	1	59.005	KV452709.1	6036651	1-scf00009	1
AX-157900086	1	59.005	KV452709.1	6037714	1-scf00009	1
AX-157900089	1	59.005	KV452709.1	6041402	1-scf00009	1
AX-158414619	1	59.005	KV452709.1	6044421	1-scf00009	1
AX-157811361	1	59.005	KV452709.1	6045082	1-scf00009	1
AX-157724287	1	59.005	KV452709.1	6046458	1-scf00009	1
AX-157868718	1	59.005	KV452709.1	6046998	1-scf00009	1
AX-158389261	1	59.005	KV452709.1	6048046	1-scf00009	1
AX-158404502	1	59.005	KV452709.1	6048415	1-scf00009	1
AX-157868719	1	59.005	KV452709.1	6058131	1-scf00009	1
AX-157900096	1	59.005	KV452709.1	6059181	1-scf00009	1
AX-158404504	1	59.005	KV452709.1	6071076	1-scf00009	1
AX-157900110	1	59.005	KV452709.1	6074774	1-scf00009	1
AX-158389266	1	59.005	KV452709.1	6085428	1-scf00009	1
AX-157900117	1	59.005	KV452709.1	6089850	1-scf00009	1
AX-157900125	1	59.005	KV452709.1	6096568	1-scf00009	1
AX-157900126	1	59.005	KV452709.1	6097015	1-scf00009	1
AX-157745437	1	59.005	KV452709.1	6098333	1-scf00009	1
AX-157900129	1	59.005	KV452709.1	6105415	1-scf00009	1
AX-158389271	1	59.005	KV452709.1	6106522	1-scf00009	1
AX-157811365	1	59.005	KV452709.1	6107852	1-scf00009	1
AX-157743590	1	59.005	KV452709.1	6109032	1-scf00009	1
AX-157811366	1	59.005	KV452709.1	6109585	1-scf00009	1
AX-157900130	1	59.005	KV452709.1	6109885	1-scf00009	1
AX-157811367	1	59.005	KV452709.1	6111415	1-scf00009	1
AX-157900131	1	59.005	KV452709.1	6112814	1-scf00009	1
AX-157900132	1	59.005	KV452709.1	6114154	1-scf00009	1
AX-157900133	1	59.005	KV452709.1	6117964	1-scf00009	1
AX-157900134	1	59.005	KV452709.1	6118715	1-scf00009	1
AX-157900135	1	59.005	KV452709.1	6119653	1-scf00009	1

AX-157900137	1	59.005	KV452709.1	6121620	1-scf00009	1
AX-157900138	1	59.005	KV452709.1	6122548	1-scf00009	1
AX-157900139	1	59.005	KV452709.1	6123293	1-scf00009	1
AX-157900140	1	59.005	KV452709.1	6123737	1-scf00009	1
AX-157900141	1	59.005	KV452709.1	6124494	1-scf00009	1
AX-157900142	1	59.005	KV452709.1	6124838	1-scf00009	1
AX-157900144	1	59.005	KV452709.1	6131407	1-scf00009	1
AX-157900145	1	59.005	KV452709.1	6131856	1-scf00009	1
AX-157900146	1	59.005	KV452709.1	6132509	1-scf00009	1
AX-157811368	1	59.005	KV452709.1	6133901	1-scf00009	1
AX-157900147	1	59.005	KV452709.1	6134341	1-scf00009	1
AX-157900148	1	59.005	KV452709.1	6134818	1-scf00009	1
AX-157811369	1	59.005	KV452709.1	6135181	1-scf00009	1
AX-157900149	1	59.005	KV452709.1	6135480	1-scf00009	1
AX-157900152	1	59.005	KV452709.1	6137590	1-scf00009	1
AX-158379153	1	59.005	KV452709.1	6165831	1-scf00009	1
AX-157811376	1	59.005	KV452709.1	6172070	1-scf00009	1
AX-157900166	1	59.005	KV452709.1	6176712	1-scf00009	1
AX-157868730	1	59.005	KV452709.1	6197563	1-scf00009	1
AX-157900177	1	59.005	KV452709.1	6198880	1-scf00009	1
AX-157717342	1	59.005	KV452709.1	6199577	1-scf00009	1
AX-157900180	1	59.005	KV452709.1	6202088	1-scf00009	1
AX-157757557	1	59.005	KV452709.1	6209359	1-scf00009	1
AX-157900183	1	59.005	KV452709.1	6210457	1-scf00009	1
AX-157811378	1	59.005	KV452709.1	6211386	1-scf00009	1
AX-157883131	1	59.005	KV452709.1	6214305	1-scf00009	1
AX-157811380	1	59.005	KV452709.1	6222724	1-scf00009	1
AX-157900188	1	59.005	KV452709.1	6223075	1-scf00009	1
AX-157751664	1	59.005	KV452709.1	6223430	1-scf00009	1
AX-157868733	1	59.005	KV452709.1	6225146	1-scf00009	1
AX-157900191	1	59.005	KV452709.1	6225970	1-scf00009	1
AX-157900193	1	59.005	KV452709.1	6231183	1-scf00009	1
AX-158389276	1	59.005	KV452709.1	6231906	1-scf00009	1
AX-157811388	1	59.005	KV452709.1	6254729	1-scf00009	1
AX-157900205	1	59.005	KV452709.1	6255460	1-scf00009	1
AX-157714479	1	59.005	KV452709.1	6258423	1-scf00009	1
AX-157900208	1	59.005	KV452709.1	6258913	1-scf00009	1
AX-157811391	1	59.005	KV452709.1	6262026	1-scf00009	1
AX-157700637	1	59.005	KV452709.1	6266093	1-scf00009	1
AX-157900213	1	59.005	KV452709.1	6268035	1-scf00009	1
AX-157900217	1	59.005	KV452709.1	6270023	1-scf00009	1
AX-157900233	1	59.005	KV452709.1	6288634	1-scf00009	1
AX-157900237	1	59.005	KV452709.1	6293176	1-scf00009	1
AX-157900240	1	59.005	KV452709.1	6297407	1-scf00009	1
AX-157900241	1	59.005	KV452709.1	6298561	1-scf00009	1
AX-157900245	1	59.005	KV452709.1	6304165	1-scf00009	1
AX-157900246	1	59.005	KV452709.1	6305611	1-scf00009	1
AX-157900247	1	59.005	KV452709.1	6306033	1-scf00009	1
AX-158389281	1	59.005	KV452709.1	6312937	1-scf00009	1
AX-157868743	1	59.005	KV452709.1	6344848	1-scf00009	1
AX-157900257	1	59.005	KV452709.1	6346049	1-scf00009	1

AX-157900259	1	59.005	KV452709.1	6347514	1-scf00009	1
AX-157900260	1	59.005	KV452709.1	6347885	1-scf00009	1
AX-157900265	1	59.005	KV452709.1	6356191	1-scf00009	1
AX-157900268	1	59.005	KV452709.1	6362088	1-scf00009	1
AX-157900269	1	59.005	KV452709.1	6362981	1-scf00009	1
AX-157900272	1	59.005	KV452709.1	6375549	1-scf00009	1
AX-157811398	1	59.005	KV452709.1	6392837	1-scf00009	1
AX-157900277	1	59.005	KV452709.1	6395361	1-scf00009	1
AX-157900278	1	59.005	KV452709.1	6395931	1-scf00009	1
AX-157900279	1	59.005	KV452709.1	6396337	1-scf00009	1
AX-157900280	1	59.005	KV452709.1	6397460	1-scf00009	1
AX-157900281	1	59.005	KV452709.1	6400316	1-scf00009	1
AX-157900283	1	59.005	KV452709.1	6411062	1-scf00009	1
AX-157900292	1	59.005	KV452709.1	6425007	1-scf00009	1
AX-157900293	1	59.005	KV452709.1	6426533	1-scf00009	1
AX-157900294	1	59.005	KV452709.1	6434998	1-scf00009	1
AX-157900297	1	59.005	KV452709.1	6444214	1-scf00009	1
AX-157900298	1	59.005	KV452709.1	6445258	1-scf00009	1
AX-157900299	1	59.005	KV452709.1	6448458	1-scf00009	1
AX-157811401	1	59.005	KV452709.1	6456073	1-scf00009	1
AX-157811403	1	59.005	KV452709.1	6459138	1-scf00009	1
AX-157900306	1	59.005	KV452709.1	6464141	1-scf00009	1
AX-157900307	1	59.005	KV452709.1	6465704	1-scf00009	1
AX-157811404	1	59.005	KV452709.1	6468343	1-scf00009	1
AX-157900311	1	59.005	KV452709.1	6483073	1-scf00009	1
AX-157900312	1	59.005	KV452709.1	6484823	1-scf00009	1
AX-157900313	1	59.005	KV452709.1	6486397	1-scf00009	1
AX-157900317	1	59.005	KV452709.1	6497442	1-scf00009	1
AX-157900318	1	59.005	KV452709.1	6497853	1-scf00009	1
AX-157900319	1	59.005	KV452709.1	6498876	1-scf00009	1
AX-157900324	1	59.005	KV452709.1	6508228	1-scf00009	1
AX-157900325	1	59.005	KV452709.1	6509939	1-scf00009	1
AX-157900326	1	59.005	KV452709.1	6510480	1-scf00009	1
AX-157900327	1	59.005	KV452709.1	6511844	1-scf00009	1
AX-157900328	1	59.005	KV452709.1	6512514	1-scf00009	1
AX-157900329	1	59.005	KV452709.1	6514600	1-scf00009	1
AX-157900330	1	59.005	KV452709.1	6518846	1-scf00009	1
AX-157695681	1	59.005	KV452709.1	6526194	1-scf00009	1
AX-157900333	1	59.005	KV452709.1	6529263	1-scf00009	1
AX-157900334	1	59.005	KV452709.1	6532023	1-scf00009	1
AX-157900338	1	59.005	KV452709.1	6537912	1-scf00009	1
AX-157900342	1	59.005	KV452709.1	6553989	1-scf00009	1
AX-157900343	1	59.005	KV452709.1	6555175	1-scf00009	1
AX-157900344	1	59.005	KV452709.1	6555909	1-scf00009	1
AX-157900345	1	59.005	KV452709.1	6556334	1-scf00009	1
AX-157900346	1	59.005	KV452709.1	6564013	1-scf00009	1
AX-157900348	1	59.005	KV452709.1	6567395	1-scf00009	1
AX-157900349	1	59.005	KV452709.1	6568400	1-scf00009	1
AX-157900350	1	59.005	KV452709.1	6573380	1-scf00009	1
AX-157900352	1	59.005	KV452709.1	6574623	1-scf00009	1
AX-157900353	1	59.005	KV452709.1	6575029	1-scf00009	1

AX-157900354	1	59.005	KV452709.1	6575734	1-scf00009	1
AX-157708902	1	59.005	KV452709.1	6590719	1-scf00009	1
AX-157900363	1	59.005	KV452709.1	6593014	1-scf00009	1
AX-157900373	1	59.005	KV452709.1	6620787	1-scf00009	1
AX-157900383	1	59.005	KV452709.1	6700270	1-scf00009	1
AX-157900389	1	59.005	KV452709.1	6736846	1-scf00009	1
AX-157900391	1	59.005	KV452709.1	6755302	1-scf00009	1
AX-157900395	1	59.005	KV452709.1	6779758	1-scf00009	1
AX-158404521	1	59.005	KV452709.1	6851112	1-scf00009	1
AX-157900403	1	59.005	KV452709.1	6946696	1-scf00009	1
AX-157874383	1	59.005	KV452709.1	6948032	1-scf00009	1
AX-157900404	1	59.005	KV452709.1	6958565	1-scf00009	1
AX-157900409	1	59.005	KV452709.1	7010462	1-scf00009	1
AX-157900410	1	59.005	KV452709.1	7012386	1-scf00009	1
AX-157900412	1	59.005	KV452709.1	7020930	1-scf00009	1
AX-157900418	1	59.005	KV452709.1	7041963	1-scf00009	1
AX-157900420	1	59.005	KV452709.1	7044598	1-scf00009	1
AX-157900421	1	59.005	KV452709.1	7045153	1-scf00009	1
AX-157900423	1	59.005	KV452709.1	7047441	1-scf00009	1
AX-157900436	1	59.005	KV452709.1	7076275	1-scf00009	1
AX-157900438	1	59.005	KV452709.1	7078991	1-scf00009	1
AX-157900440	1	59.005	KV452709.1	7087634	1-scf00009	1
AX-157900443	1	59.005	KV452709.1	7101238	1-scf00009	1
AX-157900444	1	59.005	KV452709.1	7114433	1-scf00009	1
AX-157900445	1	59.005	KV452709.1	7119975	1-scf00009	1
AX-157900446	1	59.005	KV452709.1	7123991	1-scf00009	1
AX-157900447	1	59.005	KV452709.1	7127736	1-scf00009	1
AX-157900452	1	59.005	KV452709.1	7142311	1-scf00009	1
AX-157811410	1	59.005	KV452709.1	7158157	1-scf00009	1
AX-157900457	1	59.005	KV452709.1	7158271	1-scf00009	1
AX-157900459	1	59.005	KV452709.1	7160182	1-scf00009	1
AX-157900460	1	59.005	KV452709.1	7161273	1-scf00009	1
AX-157900463	1	59.005	KV452709.1	7171648	1-scf00009	1
AX-157900466	1	59.005	KV452709.1	7174803	1-scf00009	1
AX-157900477	1	59.005	KV452709.1	7193278	1-scf00009	1
AX-157900479	1	59.005	KV452709.1	7195666	1-scf00009	1
AX-157900480	1	59.005	KV452709.1	7195858	1-scf00009	1
AX-157900481	1	59.005	KV452709.1	7196361	1-scf00009	1
AX-157900482	1	59.005	KV452709.1	7197291	1-scf00009	1
AX-157900484	1	59.005	KV452709.1	7197980	1-scf00009	1
AX-157900485	1	59.005	KV452709.1	7198486	1-scf00009	1
AX-157900487	1	59.005	KV452709.1	7199733	1-scf00009	1
AX-157900488	1	59.005	KV452709.1	7201003	1-scf00009	1
AX-157900489	1	59.005	KV452709.1	7201930	1-scf00009	1
AX-157900491	1	59.005	KV452709.1	7203774	1-scf00009	1
AX-157900494	1	59.005	KV452709.1	7205683	1-scf00009	1
AX-157900497	1	59.005	KV452709.1	7207276	1-scf00009	1
AX-157900499	1	59.005	KV452709.1	7210060	1-scf00009	1
AX-157900501	1	59.005	KV452709.1	7210909	1-scf00009	1
AX-157811411	1	59.005	KV452709.1	7211000	1-scf00009	1
AX-157900502	1	59.005	KV452709.1	7211600	1-scf00009	1

AX-157900506	1	59.005	KV452709.1	7214512	1-scf00009	1
AX-157900510	1	59.005	KV452709.1	7217809	1-scf00009	1
AX-157811413	1	59.005	KV452709.1	7224057	1-scf00009	1
AX-157811414	1	59.005	KV452709.1	7224224	1-scf00009	1
AX-157900515	1	59.005	KV452709.1	7231699	1-scf00009	1
AX-157900526	1	59.005	KV452709.1	7295563	1-scf00009	1
AX-157900556	1	59.005	KV452709.1	7421681	1-scf00009	1
AX-157726885	1	59.005	KV452709.1	7426354	1-scf00009	1
AX-157900560	1	59.005	KV452709.1	7429948	1-scf00009	1
AX-157900561	1	59.005	KV452709.1	7441347	1-scf00009	1
AX-157707657	1	59.005	KV452709.1	7441465	1-scf00009	1
AX-157900562	1	59.005	KV452709.1	7443757	1-scf00009	1
AX-157900563	1	59.005	KV452709.1	7447076	1-scf00009	1
AX-157900564	1	59.005	KV452709.1	7448952	1-scf00009	1
AX-157900565	1	59.005	KV452709.1	7451755	1-scf00009	1
AX-157900568	1	59.005	KV452709.1	7459258	1-scf00009	1
AX-157900569	1	59.005	KV452709.1	7460130	1-scf00009	1
AX-157900573	1	59.005	KV452709.1	7465512	1-scf00009	1
AX-157900574	1	59.005	KV452709.1	7468935	1-scf00009	1
AX-157900575	1	59.005	KV452709.1	7469374	1-scf00009	1
AX-157900576	1	59.005	KV452709.1	7471647	1-scf00009	1
AX-157900577	1	59.005	KV452709.1	7473394	1-scf00009	1
AX-157900584	1	59.005	KV452709.1	7488657	1-scf00009	1
AX-157900586	1	59.005	KV452709.1	7491330	1-scf00009	1
AX-157900587	1	59.005	KV452709.1	7494518	1-scf00009	1
AX-157900588	1	59.005	KV452709.1	7503871	1-scf00009	1
AX-157900589	1	59.005	KV452709.1	7504131	1-scf00009	1
AX-157900591	1	59.005	KV452709.1	7516320	1-scf00009	1
AX-157900592	1	59.005	KV452709.1	7519942	1-scf00009	1
AX-157900600	1	59.005	KV452709.1	7546531	1-scf00009	1
AX-157900602	1	59.005	KV452709.1	7548995	1-scf00009	1
AX-157900604	1	59.005	KV452709.1	7552906	1-scf00009	1
AX-157900609	1	59.005	KV452709.1	7561515	1-scf00009	1
AX-157900610	1	59.005	KV452709.1	7568482	1-scf00009	1
AX-158382010	1	59.005	KV452709.1	7574028	1-scf00009	1
AX-157900612	1	59.005	KV452709.1	7575648	1-scf00009	1
AX-157900617	1	59.005	KV452709.1	7595655	1-scf00009	1
AX-157900623	1	59.005	KV452709.1	7608790	1-scf00009	1
AX-157900626	1	59.005	KV452709.1	7611649	1-scf00009	1
AX-157900637	1	59.005	KV452709.1	7635637	1-scf00009	1
AX-157900644	1	59.005	KV452709.1	7644996	1-scf00009	1
AX-157900648	1	59.005	KV452709.1	7661179	1-scf00009	1
AX-157900650	1	59.005	KV452709.1	7665773	1-scf00009	1
AX-157900651	1	59.005	KV452709.1	7672215	1-scf00009	1
AX-157900652	1	59.005	KV452709.1	7674946	1-scf00009	1
AX-157900656	1	59.005	KV452709.1	7687805	1-scf00009	1
AX-157900663	1	59.005	KV452709.1	7717978	1-scf00009	1
AX-157900667	1	59.005	KV452709.1	7722298	1-scf00009	1
AX-157900675	1	59.005	KV452709.1	7732420	1-scf00009	1
AX-157900677	1	59.005	KV452709.1	7734518	1-scf00009	1
AX-157900679	1	59.005	KV452709.1	7736095	1-scf00009	1

AX-157900681	1	59.005	KV452709.1	7743899	1-scf00009	1
AX-157900682	1	59.005	KV452709.1	7745961	1-scf00009	1
AX-157900686	1	59.005	KV452709.1	7757904	1-scf00009	1
AX-157900687	1	59.005	KV452709.1	7758996	1-scf00009	1
AX-157707591	1	59.005	KV452709.1	7764435	1-scf00009	1
AX-157900691	1	59.005	KV452709.1	7766155	1-scf00009	1
AX-157900698	1	59.005	KV452709.1	7779785	1-scf00009	1
AX-157900699	1	59.005	KV452709.1	7782658	1-scf00009	1
AX-157900704	1	59.005	KV452709.1	7787493	1-scf00009	1
AX-157900705	1	59.005	KV452709.1	7788786	1-scf00009	1
AX-157900707	1	59.005	KV452709.1	7790097	1-scf00009	1
AX-157900708	1	59.005	KV452709.1	7793453	1-scf00009	1
AX-157900710	1	59.005	KV452709.1	7795689	1-scf00009	1
AX-157900711	1	59.005	KV452709.1	7796888	1-scf00009	1
AX-157900714	1	59.005	KV452709.1	7804079	1-scf00009	1
AX-158404538	1	59.005	KV452709.1	7804821	1-scf00009	1
AX-157900715	1	59.005	KV452709.1	7811946	1-scf00009	1
AX-157900716	1	59.005	KV452709.1	7814041	1-scf00009	1
AX-157900717	1	59.005	KV452709.1	7815270	1-scf00009	1
AX-157900718	1	59.005	KV452709.1	7818235	1-scf00009	1
AX-157900719	1	59.005	KV452709.1	7823603	1-scf00009	1
AX-157900720	1	59.005	KV452709.1	7825919	1-scf00009	1
AX-157900721	1	59.005	KV452709.1	7827450	1-scf00009	1
AX-157900722	1	59.005	KV452709.1	7836575	1-scf00009	1
AX-157900725	1	59.005	KV452709.1	7855704	1-scf00009	1
AX-157900726	1	59.005	KV452709.1	7858090	1-scf00009	1
AX-157900727	1	59.005	KV452709.1	7858977	1-scf00009	1
AX-157900728	1	59.005	KV452709.1	7859474	1-scf00009	1
AX-157900729	1	59.005	KV452709.1	7871965	1-scf00009	1
AX-157900730	1	59.005	KV452709.1	7872357	1-scf00009	1
AX-157900731	1	59.005	KV452709.1	7872842	1-scf00009	1
AX-157900732	1	59.005	KV452709.1	7876735	1-scf00009	1
AX-157900733	1	59.005	KV452709.1	7880614	1-scf00009	1
AX-157900734	1	59.005	KV452709.1	7886434	1-scf00009	1
AX-157900735	1	59.005	KV452709.1	7887196	1-scf00009	1
AX-157900736	1	59.005	KV452709.1	7888984	1-scf00009	1
AX-157900739	1	59.005	KV452709.1	7897624	1-scf00009	1
AX-157900742	1	59.005	KV452711.1	11854	1-scf00010	1
AX-157900744	1	59.005	KV452711.1	14184	1-scf00010	1
AX-158404540	1	59.005	KV452711.1	18021	1-scf00010	1
AX-157811423	1	59.005	KV452711.1	38732	1-scf00010	1
AX-158404544	1	59.005	KV452711.1	79234	1-scf00010	1
AX-157744425	1	59.005	KV452711.1	125480	1-scf00010	1
AX-157900758	1	59.005	KV452711.1	128811	1-scf00010	1
AX-157900759	1	59.005	KV452711.1	129322	1-scf00010	1
AX-157900760	1	59.005	KV452711.1	131776	1-scf00010	1
AX-157900761	1	59.005	KV452711.1	139305	1-scf00010	1
AX-157900763	1	59.005	KV452711.1	145693	1-scf00010	1
AX-157900764	1	59.005	KV452711.1	161054	1-scf00010	1
AX-157900765	1	59.005	KV452711.1	162755	1-scf00010	1
AX-157900766	1	59.005	KV452711.1	170724	1-scf00010	1

AX-157900767	1	59.005	KV452711.1	171338	1-scf00010	1
AX-157900768	1	59.005	KV452711.1	176344	1-scf00010	1
AX-157900769	1	59.005	KV452711.1	177144	1-scf00010	1
AX-157900770	1	59.005	KV452711.1	182861	1-scf00010	1
AX-157900771	1	59.005	KV452711.1	186549	1-scf00010	1
AX-157900773	1	59.005	KV452711.1	198707	1-scf00010	1
AX-157900775	1	59.005	KV452711.1	208950	1-scf00010	1
AX-157900777	1	59.005	KV452711.1	217122	1-scf00010	1
AX-157722776	1	59.005	KV452711.1	221664	1-scf00010	1
AX-157900778	1	59.005	KV452711.1	222302	1-scf00010	1
AX-157900779	1	59.005	KV452711.1	222748	1-scf00010	1
AX-157900780	1	59.005	KV452711.1	224778	1-scf00010	1
AX-157900782	1	59.005	KV452711.1	229710	1-scf00010	1
AX-157900783	1	59.005	KV452711.1	231338	1-scf00010	1
AX-157900785	1	59.005	KV452711.1	243500	1-scf00010	1
AX-157900786	1	59.005	KV452711.1	245954	1-scf00010	1
AX-157900787	1	59.005	KV452711.1	249106	1-scf00010	1
AX-157900788	1	59.005	KV452711.1	254653	1-scf00010	1
AX-157900790	1	59.005	KV452711.1	261064	1-scf00010	1
AX-157900791	1	59.005	KV452711.1	261426	1-scf00010	1
AX-157900793	1	59.005	KV452711.1	264358	1-scf00010	1
AX-157900794	1	59.005	KV452711.1	265768	1-scf00010	1
AX-157900797	1	59.005	KV452711.1	270273	1-scf00010	1
AX-157709165	1	59.005	KV452711.1	276506	1-scf00010	1
AX-157900799	1	59.005	KV452711.1	277495	1-scf00010	1
AX-158404546	1	59.005	KV452711.1	279285	1-scf00010	1
AX-157900800	1	59.005	KV452711.1	282428	1-scf00010	1
AX-157900801	1	59.005	KV452711.1	289739	1-scf00010	1
AX-157900802	1	59.005	KV452711.1	291365	1-scf00010	1
AX-157900804	1	59.005	KV452711.1	296932	1-scf00010	1
AX-157811433	1	59.005	KV452711.1	305828	1-scf00010	1
AX-157900805	1	59.005	KV452711.1	306381	1-scf00010	1
AX-157900806	1	59.005	KV452711.1	308693	1-scf00010	1
AX-157900807	1	59.005	KV452711.1	312860	1-scf00010	1
AX-157900808	1	59.005	KV452711.1	314217	1-scf00010	1
AX-157900810	1	59.005	KV452711.1	318479	1-scf00010	1
AX-157900811	1	59.005	KV452711.1	319736	1-scf00010	1
AX-157900812	1	59.005	KV452711.1	321487	1-scf00010	1
AX-157900814	1	59.005	KV452711.1	323637	1-scf00010	1
AX-157900815	1	59.005	KV452711.1	325021	1-scf00010	1
AX-157900817	1	59.005	KV452711.1	328542	1-scf00010	1
AX-157900818	1	59.005	KV452711.1	329619	1-scf00010	1
AX-157900819	1	59.005	KV452711.1	331602	1-scf00010	1
AX-157900820	1	59.005	KV452711.1	334838	1-scf00010	1
AX-157900821	1	59.005	KV452711.1	337046	1-scf00010	1
AX-157727171	1	59.005	KV452711.1	342110	1-scf00010	1
AX-157900823	1	59.005	KV452711.1	343517	1-scf00010	1
AX-157900824	1	59.005	KV452711.1	360734	1-scf00010	1
AX-157900826	1	59.005	KV452711.1	368851	1-scf00010	1
AX-157900827	1	59.005	KV452711.1	372119	1-scf00010	1
AX-157900829	1	59.005	KV452711.1	374830	1-scf00010	1

AX-157900830	1	59.005	KV452711.1	377423	1-scf00010	1
AX-157811436	1	59.005	KV452711.1	387666	1-scf00010	1
AX-157900833	1	59.005	KV452711.1	390586	1-scf00010	1
AX-157900835	1	59.005	KV452711.1	396077	1-scf00010	1
AX-157900836	1	59.005	KV452711.1	397739	1-scf00010	1
AX-157900837	1	59.005	KV452711.1	399406	1-scf00010	1
AX-157900838	1	59.005	KV452711.1	403710	1-scf00010	1
AX-157714794	1	59.005	KV452711.1	415758	1-scf00010	1
AX-157900840	1	59.005	KV452711.1	417904	1-scf00010	1
AX-157900842	1	59.005	KV452711.1	432924	1-scf00010	1
AX-157900844	1	59.005	KV452711.1	445770	1-scf00010	1
AX-157900845	1	59.005	KV452711.1	454152	1-scf00010	1
AX-157900846	1	59.005	KV452711.1	456674	1-scf00010	1
AX-157900847	1	59.005	KV452711.1	458014	1-scf00010	1
AX-157900849	1	59.005	KV452711.1	466643	1-scf00010	1
AX-157900850	1	59.005	KV452711.1	486484	1-scf00010	1
AX-157900852	1	59.005	KV452711.1	491910	1-scf00010	1
AX-157900853	1	59.005	KV452711.1	497008	1-scf00010	1
AX-157900854	1	59.005	KV452711.1	511310	1-scf00010	1
AX-157900856	1	59.005	KV452711.1	520624	1-scf00010	1
AX-158404549	1	59.005	KV452711.1	523749	1-scf00010	1
AX-157900857	1	59.005	KV452711.1	526169	1-scf00010	1
AX-157900859	1	59.005	KV452711.1	539179	1-scf00010	1
AX-157900860	1	59.005	KV452711.1	540096	1-scf00010	1
AX-157900861	1	59.005	KV452711.1	556545	1-scf00010	1
AX-157900863	1	59.005	KV452711.1	559148	1-scf00010	1
AX-157900864	1	59.005	KV452711.1	565344	1-scf00010	1
AX-157900866	1	59.005	KV452711.1	567721	1-scf00010	1
AX-157900867	1	59.005	KV452711.1	568532	1-scf00010	1
AX-157900868	1	59.005	KV452711.1	570634	1-scf00010	1
AX-157900869	1	59.005	KV452711.1	571841	1-scf00010	1
AX-157900870	1	59.005	KV452711.1	575520	1-scf00010	1
AX-157900872	1	59.005	KV452711.1	598650	1-scf00010	1
AX-157900875	1	59.005	KV452711.1	617891	1-scf00010	1
AX-157900877	1	59.005	KV452711.1	622816	1-scf00010	1
AX-157900878	1	59.005	KV452711.1	624155	1-scf00010	1
AX-157900880	1	59.005	KV452711.1	641759	1-scf00010	1
AX-157900882	1	59.005	KV452711.1	643720	1-scf00010	1
AX-157900884	1	59.005	KV452711.1	647793	1-scf00010	1
AX-157900885	1	59.005	KV452711.1	653231	1-scf00010	1
AX-157900886	1	59.005	KV452711.1	653907	1-scf00010	1
AX-157900888	1	59.005	KV452711.1	662140	1-scf00010	1
AX-157900891	1	59.005	KV452711.1	685642	1-scf00010	1
AX-157900893	1	59.005	KV452711.1	689603	1-scf00010	1
AX-157900894	1	59.005	KV452711.1	691123	1-scf00010	1
AX-157900896	1	59.005	KV452711.1	697659	1-scf00010	1
AX-157900897	1	59.005	KV452711.1	699449	1-scf00010	1
AX-157900899	1	59.005	KV452711.1	707523	1-scf00010	1
AX-157900902	1	59.005	KV452711.1	719428	1-scf00010	1
AX-157743942	1	59.005	KV452711.1	724052	1-scf00010	1
AX-157900904	1	59.005	KV452711.1	742632	1-scf00010	1

AX-157900906	1	59.005	KV452711.1	747916	1-scf00010	1
AX-157811437	1	59.005	KV452711.1	749688	1-scf00010	1
AX-157900908	1	59.005	KV452711.1	754803	1-scf00010	1
AX-157900910	1	59.005	KV452711.1	759353	1-scf00010	1
AX-157900913	1	59.005	KV452711.1	776567	1-scf00010	1
AX-157900914	1	59.005	KV452711.1	778031	1-scf00010	1
AX-157900917	1	59.005	KV452711.1	788955	1-scf00010	1
AX-157900918	1	59.005	KV452711.1	790382	1-scf00010	1
AX-157900919	1	59.005	KV452711.1	795632	1-scf00010	1
AX-157900924	1	59.005	KV452711.1	814225	1-scf00010	1
AX-157708688	1	59.005	KV452711.1	820688	1-scf00010	1
AX-157900929	1	59.005	KV452711.1	821531	1-scf00010	1
AX-157900930	1	59.005	KV452711.1	822671	1-scf00010	1
AX-157811440	1	59.005	KV452711.1	824764	1-scf00010	1
AX-157900931	1	59.005	KV452711.1	825674	1-scf00010	1
AX-157900933	1	59.005	KV452711.1	831658	1-scf00010	1
AX-157811441	1	59.005	KV452711.1	834140	1-scf00010	1
AX-157716990	1	59.005	KV452711.1	834817	1-scf00010	1
AX-157900934	1	59.005	KV452711.1	843077	1-scf00010	1
AX-157811442	1	59.005	KV452711.1	856791	1-scf00010	1
AX-157900940	1	59.005	KV452711.1	863845	1-scf00010	1
AX-157900941	1	59.005	KV452711.1	866149	1-scf00010	1
AX-157900942	1	59.005	KV452711.1	870545	1-scf00010	1
AX-157900944	1	59.005	KV452711.1	881771	1-scf00010	1
AX-157900946	1	59.005	KV452711.1	901819	1-scf00010	1
AX-157900947	1	59.005	KV452711.1	904694	1-scf00010	1
AX-157900948	1	59.005	KV452711.1	906086	1-scf00010	1
AX-157900950	1	59.005	KV452711.1	912320	1-scf00010	1
AX-157900951	1	59.005	KV452711.1	914980	1-scf00010	1
AX-157900953	1	59.005	KV452711.1	920184	1-scf00010	1
AX-157900954	1	59.005	KV452711.1	922298	1-scf00010	1
AX-157900957	1	59.005	KV452711.1	925048	1-scf00010	1
AX-157900958	1	59.005	KV452711.1	936420	1-scf00010	1
AX-157900959	1	59.005	KV452711.1	938951	1-scf00010	1
AX-157900978	1	59.005	KV452711.1	1075628	1-scf00010	1
AX-157900979	1	59.005	KV452711.1	1076340	1-scf00010	1
AX-157900980	1	59.005	KV452711.1	1077980	1-scf00010	1
AX-157900981	1	59.005	KV452711.1	1080030	1-scf00010	1
AX-157811448	1	59.005	KV452711.1	1081764	1-scf00010	1
AX-157900982	1	59.005	KV452711.1	1086637	1-scf00010	1
AX-157811449	1	59.005	KV452711.1	1094084	1-scf00010	1
AX-157900986	1	59.005	KV452711.1	1112046	1-scf00010	1
AX-157900987	1	59.005	KV452711.1	1127551	1-scf00010	1
AX-157900990	1	59.005	KV452711.1	1131972	1-scf00010	1
AX-157900993	1	59.005	KV452711.1	1137073	1-scf00010	1
AX-157900999	1	59.005	KV452711.1	1169106	1-scf00010	1
AX-157901010	1	59.005	KV452711.1	1189521	1-scf00010	1
AX-157901011	1	59.005	KV452711.1	1196384	1-scf00010	1
AX-157901016	1	59.005	KV452711.1	1220918	1-scf00010	1
AX-157811451	1	59.005	KV452711.1	1224741	1-scf00010	1
AX-157901021	1	59.005	KV452711.1	1228630	1-scf00010	1

AX-157901022	1	59.005	KV452711.1	1230126	1-scf00010	1
AX-157901028	1	59.005	KV452711.1	1250683	1-scf00010	1
AX-157901032	1	59.005	KV452711.1	1257974	1-scf00010	1
AX-158382016	1	59.005	KV452711.1	1284920	1-scf00010	1
AX-157901043	1	59.005	KV452711.1	1296072	1-scf00010	1
AX-157901046	1	59.005	KV452711.1	1307074	1-scf00010	1
AX-157901048	1	59.005	KV452711.1	1312510	1-scf00010	1
AX-157901053	1	59.005	KV452711.1	1338103	1-scf00010	1
AX-157811454	1	59.005	KV452711.1	1347013	1-scf00010	1
AX-157901061	1	59.005	KV452711.1	1347871	1-scf00010	1
AX-157901062	1	59.005	KV452711.1	1348857	1-scf00010	1
AX-157901063	1	59.005	KV452711.1	1350420	1-scf00010	1
AX-157901066	1	59.005	KV452711.1	1352928	1-scf00010	1
AX-157901076	1	59.005	KV452711.1	1376657	1-scf00010	1
AX-157901078	1	59.005	KV452711.1	1378368	1-scf00010	1
AX-157901079	1	59.005	KV452711.1	1378984	1-scf00010	1
AX-157901080	1	59.005	KV452711.1	1380403	1-scf00010	1
AX-157901081	1	59.005	KV452711.1	1393147	1-scf00010	1
AX-157901082	1	59.005	KV452711.1	1395416	1-scf00010	1
AX-157901083	1	59.005	KV452711.1	1396642	1-scf00010	1
AX-157901089	1	59.005	KV452711.1	1424019	1-scf00010	1
AX-157901091	1	59.005	KV452711.1	1430984	1-scf00010	1
AX-157901093	1	59.005	KV452711.1	1434564	1-scf00010	1
AX-157901094	1	59.005	KV452711.1	1436202	1-scf00010	1
AX-157901095	1	59.005	KV452711.1	1438650	1-scf00010	1
AX-157901110	1	59.005	KV452711.1	1507593	1-scf00010	1
AX-157901111	1	59.005	KV452711.1	1511347	1-scf00010	1
AX-158404574	1	59.005	KV452711.1	1511908	1-scf00010	1
AX-157901112	1	59.005	KV452711.1	1525299	1-scf00010	1
AX-157901120	1	59.005	KV452711.1	1542497	1-scf00010	1
AX-157901129	1	59.005	KV452711.1	1586656	1-scf00010	1
AX-157901130	1	59.005	KV452711.1	1587681	1-scf00010	1
AX-157901131	1	59.005	KV452711.1	1590554	1-scf00010	1
AX-157901133	1	59.005	KV452711.1	1592792	1-scf00010	1
AX-157901156	1	59.005	KV452711.1	1643928	1-scf00010	1
AX-157901159	1	59.005	KV452711.1	1660473	1-scf00010	1
AX-157901185	1	59.005	KV452711.1	1716161	1-scf00010	1
AX-157901193	1	59.005	KV452711.1	1738178	1-scf00010	1
AX-157754358	1	59.005	KV452711.1	1739910	1-scf00010	1
AX-157901194	1	59.005	KV452711.1	1742032	1-scf00010	1
AX-157901195	1	59.005	KV452711.1	1744939	1-scf00010	1
AX-157811467	1	59.005	KV452711.1	1745953	1-scf00010	1
AX-157901207	1	59.005	KV452711.1	1774065	1-scf00010	1
AX-157901208	1	59.005	KV452711.1	1775214	1-scf00010	1
AX-157901209	1	59.005	KV452711.1	1776899	1-scf00010	1
AX-157901213	1	59.005	KV452711.1	1780497	1-scf00010	1
AX-157901243	1	59.005	KV452711.1	1823456	1-scf00010	1
AX-157901256	1	59.005	KV452711.1	1865257	1-scf00010	1
AX-157901260	1	59.005	KV452711.1	1871704	1-scf00010	1
AX-157811469	1	59.005	KV452711.1	1894057	1-scf00010	1
AX-157811470	1	59.005	KV452711.1	1894583	1-scf00010	1

AX-157901273	1	59.005	KV452711.1	1897018	1-scf00010	1
AX-157901275	1	59.005	KV452711.1	1898560	1-scf00010	1
AX-157901276	1	59.005	KV452711.1	1901689	1-scf00010	1
AX-157901277	1	59.005	KV452711.1	1904769	1-scf00010	1
AX-157725844	1	59.005	KV452711.1	1905314	1-scf00010	1
AX-157901280	1	59.005	KV452711.1	1907412	1-scf00010	1
AX-157901282	1	59.005	KV452711.1	1909553	1-scf00010	1
AX-157901283	1	59.005	KV452711.1	1910245	1-scf00010	1
AX-157756375	1	59.005	KV452711.1	1910932	1-scf00010	1
AX-157722161	1	59.005	KV452711.1	1926817	1-scf00010	1
AX-157901295	1	59.005	KV452711.1	1934390	1-scf00010	1
AX-157901298	1	59.005	KV452711.1	1944600	1-scf00010	1
AX-157756172	1	59.005	KV452711.1	1950016	1-scf00010	1
AX-157901303	1	59.005	KV452711.1	1953421	1-scf00010	1
AX-157901305	1	59.005	KV452711.1	1956224	1-scf00010	1
AX-157774143	1	59.005	KV452711.1	1968408	1-scf00010	1
AX-157901309	1	59.005	KV452711.1	1969497	1-scf00010	1
AX-157901311	1	59.005	KV452711.1	1971349	1-scf00010	1
AX-157901312	1	59.005	KV452711.1	1974071	1-scf00010	1
AX-157901313	1	59.005	KV452711.1	1974951	1-scf00010	1
AX-157901314	1	59.005	KV452711.1	1976175	1-scf00010	1
AX-157901319	1	59.005	KV452711.1	1990473	1-scf00010	1
AX-157901321	1	59.005	KV452711.1	1996394	1-scf00010	1
AX-157901322	1	59.005	KV452711.1	1998712	1-scf00010	1
AX-157901323	1	59.005	KV452711.1	1999468	1-scf00010	1
AX-157901326	1	59.005	KV452711.1	2011480	1-scf00010	1
AX-157811475	1	59.005	KV452711.1	2020260	1-scf00010	1
AX-157901337	1	59.005	KV452711.1	2044400	1-scf00010	1
AX-157901338	1	59.005	KV452711.1	2047969	1-scf00010	1
AX-157901340	1	59.005	KV452711.1	2052187	1-scf00010	1
AX-157811478	1	59.005	KV452711.1	2054370	1-scf00010	1
AX-157901343	1	59.005	KV452711.1	2080259	1-scf00010	1
AX-157901344	1	59.005	KV452711.1	2081204	1-scf00010	1
AX-157901346	1	59.005	KV452711.1	2084673	1-scf00010	1
AX-157901347	1	59.005	KV452711.1	2085305	1-scf00010	1
AX-157901353	1	59.005	KV452711.1	2097970	1-scf00010	1
AX-157901358	1	59.005	KV452711.1	2117896	1-scf00010	1
AX-157901359	1	59.005	KV452711.1	2122865	1-scf00010	1
AX-157901360	1	59.005	KV452711.1	2123432	1-scf00010	1
AX-157901368	1	59.005	KV452711.1	2138281	1-scf00010	1
AX-157901369	1	59.005	KV452711.1	2144795	1-scf00010	1
AX-157811485	1	59.005	KV452711.1	2153872	1-scf00010	1
AX-157811486	1	59.005	KV452711.1	2154297	1-scf00010	1
AX-157811487	1	59.005	KV452711.1	2157301	1-scf00010	1
AX-158379157	1	59.005	KV452711.1	2160774	1-scf00010	1
AX-157901372	1	59.005	KV452711.1	2162959	1-scf00010	1
AX-157901377	1	59.005	KV452711.1	2176466	1-scf00010	1
AX-157901380	1	59.005	KV452711.1	2187803	1-scf00010	1
AX-157901383	1	59.005	KV452711.1	2192503	1-scf00010	1
AX-157901384	1	59.005	KV452711.1	2193393	1-scf00010	1
AX-157811489	1	59.005	KV452711.1	2193665	1-scf00010	1

AX-157864406	1	59.005	KV452711.1	2194625	1-scf00010	1
AX-157901385	1	59.005	KV452711.1	2196714	1-scf00010	1
AX-157901386	1	59.005	KV452711.1	2197195	1-scf00010	1
AX-157901388	1	59.005	KV452711.1	2200381	1-scf00010	1
AX-157864407	1	59.005	KV452711.1	2203574	1-scf00010	1
AX-157901391	1	59.005	KV452711.1	2207647	1-scf00010	1
AX-157901393	1	59.005	KV452711.1	2225763	1-scf00010	1
AX-157901396	1	59.005	KV452711.1	2228327	1-scf00010	1
AX-157901397	1	59.005	KV452711.1	2228529	1-scf00010	1
AX-157811495	1	59.005	KV452711.1	2229162	1-scf00010	1
AX-157901398	1	59.005	KV452711.1	2229472	1-scf00010	1
AX-157901399	1	59.005	KV452711.1	2231277	1-scf00010	1
AX-157901404	1	59.005	KV452711.1	2237216	1-scf00010	1
AX-157901409	1	59.005	KV452711.1	2241869	1-scf00010	1
AX-157901411	1	59.005	KV452711.1	2243360	1-scf00010	1
AX-157901412	1	59.005	KV452711.1	2243971	1-scf00010	1
AX-157901413	1	59.005	KV452711.1	2247146	1-scf00010	1
AX-157901414	1	59.005	KV452711.1	2253159	1-scf00010	1
AX-157901416	1	59.005	KV452711.1	2272337	1-scf00010	1
AX-157901417	1	59.005	KV452711.1	2276625	1-scf00010	1
AX-157901418	1	59.005	KV452711.1	2278404	1-scf00010	1
AX-157720748	1	59.005	KV452711.1	2288357	1-scf00010	1
AX-157901420	1	59.005	KV452711.1	2297359	1-scf00010	1
AX-157901422	1	59.005	KV452711.1	2306983	1-scf00010	1
AX-157901423	1	59.005	KV452711.1	2307375	1-scf00010	1
AX-157811498	1	59.005	KV452711.1	2310528	1-scf00010	1
AX-157811499	1	59.005	KV452711.1	2323530	1-scf00010	1
AX-157901426	1	59.005	KV452711.1	2324476	1-scf00010	1
AX-157901427	1	59.005	KV452711.1	2325206	1-scf00010	1
AX-157901429	1	59.005	KV452711.1	2327287	1-scf00010	1
AX-157746928	1	59.005	KV452711.1	2327833	1-scf00010	1
AX-157901430	1	59.005	KV452711.1	2328450	1-scf00010	1
AX-157901431	1	59.005	KV452711.1	2333071	1-scf00010	1
AX-157901432	1	59.005	KV452711.1	2334896	1-scf00010	1
AX-157901435	1	59.005	KV452711.1	2339247	1-scf00010	1
AX-157901439	1	59.005	KV452711.1	2361674	1-scf00010	1
AX-157901440	1	59.005	KV452711.1	2364414	1-scf00010	1
AX-157901441	1	59.005	KV452711.1	2366886	1-scf00010	1
AX-157901444	1	59.005	KV452711.1	2380557	1-scf00010	1
AX-157901445	1	59.005	KV452711.1	2381013	1-scf00010	1
AX-157901447	1	59.005	KV452711.1	2389563	1-scf00010	1
AX-157756760	1	59.005	KV452711.1	2390382	1-scf00010	1
AX-157901448	1	59.005	KV452711.1	2392861	1-scf00010	1
AX-157901450	1	59.005	KV452711.1	2405028	1-scf00010	1
AX-157901451	1	59.005	KV452711.1	2407620	1-scf00010	1
AX-157901452	1	59.005	KV452711.1	2410387	1-scf00010	1
AX-157901454	1	59.005	KV452711.1	2412321	1-scf00010	1
AX-157901455	1	59.005	KV452711.1	2417396	1-scf00010	1
AX-157901474	1	59.005	KV452711.1	2486569	1-scf00010	1
AX-157719525	1	59.005	KV452711.1	2489604	1-scf00010	1
AX-157901482	1	59.005	KV452711.1	2522445	1-scf00010	1

AX-157901488	1	59.005	KV452711.1	2543655	1-scf00010	1
AX-157901489	1	59.005	KV452711.1	2545302	1-scf00010	1
AX-157901490	1	59.005	KV452711.1	2552892	1-scf00010	1
AX-157901494	1	59.005	KV452711.1	2563843	1-scf00010	1
AX-157771452	1	59.005	KV452711.1	2568722	1-scf00010	1
AX-157901498	1	59.005	KV452711.1	2571530	1-scf00010	1
AX-157901500	1	59.005	KV452711.1	2576698	1-scf00010	1
AX-157901501	1	59.005	KV452711.1	2579735	1-scf00010	1
AX-157901508	1	59.005	KV452711.1	2595040	1-scf00010	1
AX-157901509	1	59.005	KV452711.1	2595775	1-scf00010	1
AX-157901512	1	59.005	KV452711.1	2599150	1-scf00010	1
AX-157811508	1	59.005	KV452711.1	2601378	1-scf00010	1
AX-157901514	1	59.005	KV452711.1	2604768	1-scf00010	1
AX-157901515	1	59.005	KV452711.1	2604979	1-scf00010	1
AX-157901516	1	59.005	KV452711.1	2606921	1-scf00010	1
AX-157901517	1	59.005	KV452711.1	2607934	1-scf00010	1
AX-157753671	1	59.005	KV452711.1	2612919	1-scf00010	1
AX-157901519	1	59.005	KV452711.1	2613299	1-scf00010	1
AX-157747016	1	59.005	KV452711.1	2614769	1-scf00010	1
AX-157713467	1	59.005	KV452711.1	2630659	1-scf00010	1
AX-157901532	1	59.005	KV452711.1	2678533	1-scf00010	1
AX-157901533	1	59.005	KV452711.1	2679656	1-scf00010	1
AX-157901540	1	59.005	KV452711.1	2692020	1-scf00010	1
AX-157901547	1	59.005	KV452711.1	2703026	1-scf00010	1
AX-157901552	1	59.005	KV452711.1	2708635	1-scf00010	1
AX-157901624	1	59.005	KV452711.1	2830516	1-scf00010	1
AX-157901626	1	59.005	KV452711.1	2833121	1-scf00010	1
AX-157901691	1	59.005	KV452711.1	3004144	1-scf00010	1
AX-157901701	1	59.005	KV452711.1	3037185	1-scf00010	1
AX-157901704	1	59.005	KV452711.1	3043538	1-scf00010	1
AX-157901783	1	59.005	KV452711.1	3266672	1-scf00010	1
AX-157901799	1	59.005	KV452711.1	3298868	1-scf00010	1
AX-157901805	1	59.005	KV452711.1	3305209	1-scf00010	1
AX-157901917	1	59.005	KV452711.1	3657164	1-scf00010	1
AX-157901961	1	59.005	KV452711.1	3708520	1-scf00010	1
AX-157715786	1	59.005	KV452711.1	3717895	1-scf00010	1
AX-157901985	1	59.005	KV452711.1	3741554	1-scf00010	1
AX-157902017	1	59.005	KV452711.1	3788090	1-scf00010	1
AX-157811608	1	59.005	KV452711.1	3831356	1-scf00010	1
AX-157811633	1	59.005	KV452711.1	3974543	1-scf00010	1
AX-157902217	1	59.005	KV452711.1	4061483	1-scf00010	1
AX-157902232	1	59.005	KV452711.1	4096082	1-scf00010	1
AX-157902234	1	59.005	KV452711.1	4098253	1-scf00010	1
AX-157902294	1	59.005	KV452713.1	83523	1-scf00011	1
AX-157902352	1	59.005	KV452713.1	401589	1-scf00011	1
AX-157902357	1	59.005	KV452713.1	431085	1-scf00011	1
AX-157902364	1	59.005	KV452713.1	457576	1-scf00011	1
AX-157902375	1	59.005	KV452713.1	519262	1-scf00011	1
AX-157902389	1	59.005	KV452713.1	544357	1-scf00011	1
AX-157902395	1	59.005	KV452713.1	588506	1-scf00011	1
AX-157811650	1	59.005	KV452713.1	591477	1-scf00011	1

AX-157902397	1	59.005	KV452713.1	594034	1-scf00011	1
AX-157853601	1	59.005	KV452713.1	600518	1-scf00011	1
AX-157853603	1	59.005	KV452713.1	620045	1-scf00011	1
AX-157902413	1	59.005	KV452713.1	639062	1-scf00011	1
AX-157902426	1	59.005	KV452713.1	692978	1-scf00011	1
AX-157902442	1	59.005	KV452713.1	733680	1-scf00011	1
AX-157902443	1	59.005	KV452713.1	734535	1-scf00011	1
AX-157755003	1	59.005	KV452713.1	779395	1-scf00011	1
AX-157902474	1	59.005	KV452713.1	791200	1-scf00011	1
AX-157902479	1	59.005	KV452713.1	800345	1-scf00011	1
AX-157902480	1	59.005	KV452713.1	800783	1-scf00011	1
AX-157902509	1	59.005	KV452713.1	856065	1-scf00011	1
AX-157902512	1	59.005	KV452713.1	860992	1-scf00011	1
AX-157902515	1	59.005	KV452713.1	865498	1-scf00011	1
AX-157811674	1	59.005	KV452713.1	869871	1-scf00011	1
AX-157902517	1	59.005	KV452713.1	871513	1-scf00011	1
AX-157811675	1	59.005	KV452713.1	873058	1-scf00011	1
AX-157902518	1	59.005	KV452713.1	874337	1-scf00011	1
AX-157902535	1	59.005	KV452713.1	898354	1-scf00011	1
AX-157902540	1	59.005	KV452713.1	910570	1-scf00011	1
AX-157902544	1	59.005	KV452713.1	922188	1-scf00011	1
AX-157902578	1	59.005	KV452713.1	976431	1-scf00011	1
AX-157902601	1	59.005	KV452713.1	1012392	1-scf00011	1
AX-157902602	1	59.005	KV452713.1	1013026	1-scf00011	1
AX-157902603	1	59.005	KV452713.1	1015896	1-scf00011	1
AX-157902631	1	59.005	KV452713.1	1045189	1-scf00011	1
AX-157902652	1	59.005	KV452713.1	1074056	1-scf00011	1
AX-157902714	1	59.005	KV452713.1	1162561	1-scf00011	1
AX-157902721	1	59.005	KV452713.1	1168124	1-scf00011	1
AX-157902776	1	59.005	KV452713.1	1245708	1-scf00011	1
AX-157902834	1	59.005	KV452713.1	1331862	1-scf00011	1
AX-157868876	1	59.005	KV452713.1	1372442	1-scf00011	1
AX-157883304	1	59.005	KV452713.1	1472306	1-scf00011	1
AX-157811715	1	59.005	KV452713.1	1615666	1-scf00011	1
AX-157903114	1	59.005	KV452713.1	1709620	1-scf00011	1
AX-157903323	1	59.005	KV452713.1	1993855	1-scf00011	1
AX-157903410	1	59.005	KV452713.1	2099174	1-scf00011	1
AX-157903469	1	59.005	KV452713.1	2160143	1-scf00011	1
AX-158389419	1	59.005	KV452713.1	2173614	1-scf00011	1
AX-158414675	1	59.005	KV452713.1	2366803	1-scf00011	1
AX-157903688	1	59.005	KV452713.1	2396321	1-scf00011	1
AX-157903718	1	59.005	KV452713.1	2440813	1-scf00011	1
AX-157903733	1	59.005	KV452713.1	2457081	1-scf00011	1
AX-157903765	1	59.005	KV452713.1	2512820	1-scf00011	1
AX-157903800	1	59.005	KV452713.1	2574050	1-scf00011	1
AX-157903878	1	59.005	KV452713.1	2662386	1-scf00011	1
AX-157757713	1	59.005	KV452713.1	2756771	1-scf00011	1
AX-157811794	1	59.005	KV452713.1	2833237	1-scf00011	1
AX-157874425	1	59.005	KV452713.1	2842000	1-scf00011	1
AX-157904003	1	59.005	KV452713.1	2853318	1-scf00011	1
AX-157874426	1	59.005	KV452713.1	2929543	1-scf00011	1

AX-157904047	1	59.005	KV452713.1	2936482	1-scf00011	1
AX-157904056	1	59.005	KV452713.1	2968561	1-scf00011	1
AX-157904057	1	59.005	KV452713.1	2968993	1-scf00011	1
AX-157904110	1	59.005	KV452713.1	3055665	1-scf00011	1
AX-157904169	1	59.005	KV452713.1	3173978	1-scf00011	1
AX-157727174	1	59.005	KV452713.1	3359266	1-scf00011	1
AX-157904249	1	59.005	KV452713.1	3383473	1-scf00011	1
AX-157904251	1	59.005	KV452713.1	3386479	1-scf00011	1
AX-157904255	1	59.005	KV452713.1	3396528	1-scf00011	1
AX-157904260	1	59.005	KV452713.1	3422885	1-scf00011	1
AX-157904267	1	59.005	KV452713.1	3437118	1-scf00011	1
AX-157904272	1	59.005	KV452713.1	3447162	1-scf00011	1
AX-157904274	1	59.005	KV452713.1	3451075	1-scf00011	1
AX-157904275	1	59.005	KV452713.1	3452022	1-scf00011	1
AX-157811813	1	59.005	KV452713.1	3465476	1-scf00011	1
AX-157904284	1	59.005	KV452713.1	3470848	1-scf00011	1
AX-157904302	1	59.005	KV452713.1	3526454	1-scf00011	1
AX-157904304	1	59.005	KV452713.1	3528667	1-scf00011	1
AX-157904308	1	59.005	KV452713.1	3533234	1-scf00011	1
AX-157904311	1	59.005	KV452713.1	3537979	1-scf00011	1
AX-157904312	1	59.005	KV452713.1	3540623	1-scf00011	1
AX-157904386	1	59.005	KV452713.1	3647644	1-scf00011	1
AX-157904417	1	59.005	KV452713.1	3729500	1-scf00011	1
AX-157904428	1	59.005	KV452713.1	3738286	1-scf00011	1
AX-157904429	1	59.005	KV452713.1	3739147	1-scf00011	1
AX-157904479	1	59.005	KV452713.1	3814387	1-scf00011	1
AX-157904493	1	59.005	KV452713.1	3840912	1-scf00011	1
AX-157904542	1	59.005	KV452713.1	3924473	1-scf00011	1
AX-157904595	1	59.005	KV452713.1	3989786	1-scf00011	1
AX-157874432	1	59.005	KV452713.1	4013927	1-scf00011	1
AX-157904665	1	59.005	KV452713.1	4097918	1-scf00011	1
AX-157904668	1	59.005	KV452713.1	4101636	1-scf00011	1
AX-157904700	1	59.005	KV452713.1	4137711	1-scf00011	1
AX-157904767	1	59.005	KV452713.1	4219110	1-scf00011	1
AX-157904864	1	59.005	KV452713.1	4349779	1-scf00011	1
AX-158389498	1	59.005	KV452713.1	4349971	1-scf00011	1
AX-157904889	1	59.005	KV452713.1	4379517	1-scf00011	1
AX-157811853	1	59.005	KV452713.1	4385271	1-scf00011	1
AX-158382052	1	59.005	KV452713.1	4386974	1-scf00011	1
AX-157710315	1	59.005	KV452713.1	4530398	1-scf00011	1
AX-157905107	1	59.005	KV452713.1	4653336	1-scf00011	1
AX-157811872	1	59.005	KV452713.1	4690494	1-scf00011	1
AX-158376618	1	59.005	KV452710.1	30940	1-scf00588	1
AX-158376622	1	59.005	KV452710.1	69082	1-scf00588	1
AX-158376624	1	59.005	KV452710.1	101011	1-scf00588	1
AX-158376627	1	59.005	KV452710.1	140216	1-scf00588	1
AX-158376628	1	59.005	KV452710.1	156302	1-scf00588	1
AX-158376631	1	59.005	KV452710.1	164254	1-scf00588	1
AX-158376636	1	59.005	KV452710.1	176251	1-scf00588	1
AX-157717465	1	59.005	KV452710.1	194425	1-scf00588	1
AX-157853332	1	59.005	KV452710.1	196093	1-scf00588	1

AX-158376639	1	59.005	KV452710.1	196727	1-scf00588	1
AX-158376641	1	59.005	KV452710.1	208972	1-scf00588	1
AX-158376642	1	59.005	KV452710.1	211163	1-scf00588	1
AX-158376643	1	59.005	KV452710.1	211623	1-scf00588	1
AX-158379060	1	59.005	KV452710.1	224261	1-scf00588	1
AX-158376645	1	59.005	KV452710.1	227376	1-scf00588	1
AX-158376646	1	59.005	KV452710.1	234015	1-scf00588	1
AX-158376647	1	59.005	KV452710.1	237320	1-scf00588	1
AX-157853333	1	59.005	KV452710.1	238876	1-scf00588	1
AX-158376648	1	59.005	KV452710.1	240429	1-scf00588	1
AX-157853334	1	59.005	KV452710.1	245140	1-scf00588	1
AX-158376650	1	59.005	KV452710.1	250869	1-scf00588	1
AX-158376651	1	59.005	KV452710.1	253061	1-scf00588	1
AX-158376652	1	59.005	KV452710.1	260730	1-scf00588	1
AX-158376653	1	59.005	KV452710.1	261602	1-scf00588	1
AX-158376656	1	59.005	KV452710.1	270383	1-scf00588	1
AX-158376657	1	59.005	KV452710.1	274908	1-scf00588	1
AX-158376658	1	59.005	KV452710.1	278208	1-scf00588	1
AX-158421736	1	59.005	DX102434.1	56	IORI212 Ictalu	UN
AX-157695258	1	59.005	FI869408.1	39	IORI212 Ictalu	UN
AX-157706053	1	59.005	3ML01031505	492	scf07120_1	UN
AX-157743790	1	59.114	KV452708.1	978770	1-scf00008	1
AX-157853562	1	59.114	KV452708.1	2081260	1-scf00008	1
AX-157715844	1	59.114	KV452708.1	2516172	1-scf00008	1
AX-157896148	1	59.114	KV452709.1	86261	1-scf00009	1
AX-157896194	1	59.114	KV452709.1	146855	1-scf00009	1
AX-157896212	1	59.114	KV452709.1	170104	1-scf00009	1
AX-157896286	1	59.114	KV452709.1	262212	1-scf00009	1
AX-157810919	1	59.114	KV452709.1	352586	1-scf00009	1
AX-157810930	1	59.114	KV452709.1	500403	1-scf00009	1
AX-157896538	1	59.114	KV452709.1	570142	1-scf00009	1
AX-157896680	1	59.114	KV452709.1	758899	1-scf00009	1
AX-157718143	1	59.114	KV452709.1	840062	1-scf00009	1
AX-157896775	1	59.114	KV452709.1	897641	1-scf00009	1
AX-157896806	1	59.114	KV452709.1	950549	1-scf00009	1
AX-157896849	1	59.114	KV452709.1	1016230	1-scf00009	1
AX-157896865	1	59.114	KV452709.1	1029943	1-scf00009	1
AX-157896873	1	59.114	KV452709.1	1039003	1-scf00009	1
AX-157896895	1	59.114	KV452709.1	1064277	1-scf00009	1
AX-157896898	1	59.114	KV452709.1	1066104	1-scf00009	1
AX-157896906	1	59.114	KV452709.1	1072398	1-scf00009	1
AX-157810966	1	59.114	KV452709.1	1084776	1-scf00009	1
AX-157896959	1	59.114	KV452709.1	1160363	1-scf00009	1
AX-157896967	1	59.114	KV452709.1	1173998	1-scf00009	1
AX-157896968	1	59.114	KV452709.1	1176819	1-scf00009	1
AX-157897065	1	59.114	KV452709.1	1359110	1-scf00009	1
AX-157897165	1	59.114	KV452709.1	1480927	1-scf00009	1
AX-157897186	1	59.114	KV452709.1	1513041	1-scf00009	1
AX-157897234	1	59.114	KV452709.1	1576326	1-scf00009	1
AX-157897246	1	59.114	KV452709.1	1588499	1-scf00009	1
AX-157897302	1	59.114	KV452709.1	1666129	1-scf00009	1

AX-157883103	1	59.114	KV452709.1	1708032	1-scf00009	1
AX-157897427	1	59.114	KV452709.1	1848434	1-scf00009	1
AX-157897429	1	59.114	KV452709.1	1853024	1-scf00009	1
AX-157897483	1	59.114	KV452709.1	1954740	1-scf00009	1
AX-157897486	1	59.114	KV452709.1	1963014	1-scf00009	1
AX-157811057	1	59.114	KV452709.1	2289516	1-scf00009	1
AX-157897794	1	59.114	KV452709.1	2451110	1-scf00009	1
AX-157811069	1	59.114	KV452709.1	2559548	1-scf00009	1
AX-157897965	1	59.114	KV452709.1	2725630	1-scf00009	1
AX-157897973	1	59.114	KV452709.1	2738452	1-scf00009	1
AX-157898254	1	59.114	KV452709.1	3298066	1-scf00009	1
AX-157898338	1	59.114	KV452709.1	3462870	1-scf00009	1
AX-157898345	1	59.114	KV452709.1	3471015	1-scf00009	1
AX-157714756	1	59.114	KV452709.1	3473347	1-scf00009	1
AX-157898384	1	59.114	KV452709.1	3535919	1-scf00009	1
AX-157898398	1	59.114	KV452709.1	3563694	1-scf00009	1
AX-157898412	1	59.114	KV452709.1	3586190	1-scf00009	1
AX-157725412	1	59.114	KV452709.1	3712361	1-scf00009	1
AX-157898508	1	59.114	KV452709.1	3862553	1-scf00009	1
AX-157898709	1	59.114	KV452709.1	4143345	1-scf00009	1
AX-157898712	1	59.114	KV452709.1	4145956	1-scf00009	1
AX-157898717	1	59.114	KV452709.1	4154145	1-scf00009	1
AX-157898787	1	59.114	KV452709.1	4263968	1-scf00009	1
AX-157724733	1	59.114	KV452709.1	4292727	1-scf00009	1
AX-157898838	1	59.114	KV452709.1	4342379	1-scf00009	1
AX-157811245	1	59.114	KV452709.1	4733932	1-scf00009	1
AX-157899186	1	59.114	KV452709.1	4812643	1-scf00009	1
AX-157899226	1	59.114	KV452709.1	4884799	1-scf00009	1
AX-157899229	1	59.114	KV452709.1	4891384	1-scf00009	1
AX-157811266	1	59.114	KV452709.1	4949522	1-scf00009	1
AX-157899312	1	59.114	KV452709.1	5000788	1-scf00009	1
AX-157899319	1	59.114	KV452709.1	5010783	1-scf00009	1
AX-157899563	1	59.114	KV452709.1	5319643	1-scf00009	1
AX-157899610	1	59.114	KV452709.1	5376479	1-scf00009	1
AX-157742844	1	59.114	KV452709.1	5437391	1-scf00009	1
AX-157899658	1	59.114	KV452709.1	5440009	1-scf00009	1
AX-157757090	1	59.114	KV452709.1	5492573	1-scf00009	1
AX-157899796	1	59.114	KV452709.1	5635597	1-scf00009	1
AX-157899892	1	59.114	KV452709.1	5731399	1-scf00009	1
AX-157899998	1	59.114	KV452709.1	5888747	1-scf00009	1
AX-157900007	1	59.114	KV452709.1	5911266	1-scf00009	1
AX-157900011	1	59.114	KV452709.1	5914479	1-scf00009	1
AX-157900012	1	59.114	KV452709.1	5917322	1-scf00009	1
AX-157900014	1	59.114	KV452709.1	5925836	1-scf00009	1
AX-157900015	1	59.114	KV452709.1	5926183	1-scf00009	1
AX-157900016	1	59.114	KV452709.1	5926465	1-scf00009	1
AX-157900021	1	59.114	KV452709.1	5934638	1-scf00009	1
AX-157900023	1	59.114	KV452709.1	5936319	1-scf00009	1
AX-157900025	1	59.114	KV452709.1	5941987	1-scf00009	1
AX-157900028	1	59.114	KV452709.1	5946522	1-scf00009	1
AX-157811358	1	59.114	KV452709.1	5946898	1-scf00009	1

AX-158404500	1	59.114	KV452709.1	5996704	1-scf00009	1
AX-157900076	1	59.114	KV452709.1	6026537	1-scf00009	1
AX-157900151	1	59.114	KV452709.1	6136686	1-scf00009	1
AX-157811373	1	59.114	KV452709.1	6150965	1-scf00009	1
AX-157724572	1	59.114	KV452709.1	6229116	1-scf00009	1
AX-157774468	1	59.114	KV452709.1	6233505	1-scf00009	1
AX-157900196	1	59.114	KV452709.1	6234229	1-scf00009	1
AX-157900197	1	59.114	KV452709.1	6235180	1-scf00009	1
AX-157900199	1	59.114	KV452709.1	6237224	1-scf00009	1
AX-157900238	1	59.114	KV452709.1	6293744	1-scf00009	1
AX-157864402	1	59.114	KV452709.1	6321943	1-scf00009	1
AX-157900251	1	59.114	KV452709.1	6335314	1-scf00009	1
AX-157900256	1	59.114	KV452709.1	6343682	1-scf00009	1
AX-157900258	1	59.114	KV452709.1	6346481	1-scf00009	1
AX-157900275	1	59.114	KV452709.1	6392377	1-scf00009	1
AX-158404514	1	59.114	KV452709.1	6395233	1-scf00009	1
AX-157900289	1	59.114	KV452709.1	6421524	1-scf00009	1
AX-157900296	1	59.114	KV452709.1	6440791	1-scf00009	1
AX-157900300	1	59.114	KV452709.1	6456749	1-scf00009	1
AX-157900301	1	59.114	KV452709.1	6457147	1-scf00009	1
AX-157900321	1	59.114	KV452709.1	6501656	1-scf00009	1
AX-157900322	1	59.114	KV452709.1	6505236	1-scf00009	1
AX-157900335	1	59.114	KV452709.1	6532946	1-scf00009	1
AX-157900351	1	59.114	KV452709.1	6574039	1-scf00009	1
AX-157900355	1	59.114	KV452709.1	6577044	1-scf00009	1
AX-157900411	1	59.114	KV452709.1	7013461	1-scf00009	1
AX-157811409	1	59.114	KV452709.1	7126597	1-scf00009	1
AX-157900517	1	59.114	KV452709.1	7244983	1-scf00009	1
AX-157900538	1	59.114	KV452709.1	7363481	1-scf00009	1
AX-157900570	1	59.114	KV452709.1	7460994	1-scf00009	1
AX-157900749	1	59.114	KV452711.1	34514	1-scf00010	1
AX-157900756	1	59.114	KV452711.1	97383	1-scf00010	1
AX-158382013	1	59.114	KV452711.1	212443	1-scf00010	1
AX-157900858	1	59.114	KV452711.1	533570	1-scf00010	1
AX-157900915	1	59.114	KV452711.1	780905	1-scf00010	1
AX-157811439	1	59.114	KV452711.1	788698	1-scf00010	1
AX-157900939	1	59.114	KV452711.1	862919	1-scf00010	1
AX-157811446	1	59.114	KV452711.1	1038690	1-scf00010	1
AX-157900975	1	59.114	KV452711.1	1039782	1-scf00010	1
AX-157720758	1	59.114	KV452711.1	1044384	1-scf00010	1
AX-158421696	1	59.114	KV452711.1	1049944	1-scf00010	1
AX-157811447	1	59.114	KV452711.1	1064481	1-scf00010	1
AX-157901000	1	59.114	KV452711.1	1169754	1-scf00010	1
AX-157901008	1	59.114	KV452711.1	1186140	1-scf00010	1
AX-157901009	1	59.114	KV452711.1	1187895	1-scf00010	1
AX-157901033	1	59.114	KV452711.1	1258430	1-scf00010	1
AX-157901037	1	59.114	KV452711.1	1277604	1-scf00010	1
AX-157901039	1	59.114	KV452711.1	1281440	1-scf00010	1
AX-157901041	1	59.114	KV452711.1	1294165	1-scf00010	1
AX-157901042	1	59.114	KV452711.1	1295017	1-scf00010	1
AX-157901044	1	59.114	KV452711.1	1302205	1-scf00010	1

AX-157901045	1	59.114	KV452711.1	1304299	1-scf00010	1
AX-157901052	1	59.114	KV452711.1	1334887	1-scf00010	1
AX-157901054	1	59.114	KV452711.1	1338767	1-scf00010	1
AX-157901057	1	59.114	KV452711.1	1342427	1-scf00010	1
AX-157901084	1	59.114	KV452711.1	1400765	1-scf00010	1
AX-157901099	1	59.114	KV452711.1	1467497	1-scf00010	1
AX-157901100	1	59.114	KV452711.1	1470314	1-scf00010	1
AX-157901105	1	59.114	KV452711.1	1485585	1-scf00010	1
AX-158382019	1	59.114	KV452711.1	1506111	1-scf00010	1
AX-157901121	1	59.114	KV452711.1	1549541	1-scf00010	1
AX-157901134	1	59.114	KV452711.1	1594996	1-scf00010	1
AX-157901138	1	59.114	KV452711.1	1603691	1-scf00010	1
AX-157901140	1	59.114	KV452711.1	1605351	1-scf00010	1
AX-157901142	1	59.114	KV452711.1	1609659	1-scf00010	1
AX-157901143	1	59.114	KV452711.1	1610548	1-scf00010	1
AX-157901204	1	59.114	KV452711.1	1770601	1-scf00010	1
AX-157901250	1	59.114	KV452711.1	1850914	1-scf00010	1
AX-157901292	1	59.114	KV452711.1	1925879	1-scf00010	1
AX-157901293	1	59.114	KV452711.1	1927693	1-scf00010	1
AX-157901308	1	59.114	KV452711.1	1967192	1-scf00010	1
AX-157901320	1	59.114	KV452711.1	1991671	1-scf00010	1
AX-157726845	1	59.114	KV452711.1	2038649	1-scf00010	1
AX-157901342	1	59.114	KV452711.1	2062436	1-scf00010	1
AX-157901345	1	59.114	KV452711.1	2083386	1-scf00010	1
AX-157901349	1	59.114	KV452711.1	2092265	1-scf00010	1
AX-157901355	1	59.114	KV452711.1	2101590	1-scf00010	1
AX-157901371	1	59.114	KV452711.1	2152892	1-scf00010	1
AX-157901373	1	59.114	KV452711.1	2167310	1-scf00010	1
AX-157811494	1	59.114	KV452711.1	2224657	1-scf00010	1
AX-157901394	1	59.114	KV452711.1	2226950	1-scf00010	1
AX-157901395	1	59.114	KV452711.1	2227588	1-scf00010	1
AX-157901421	1	59.114	KV452711.1	2299070	1-scf00010	1
AX-157724118	1	59.114	KV452711.1	2355887	1-scf00010	1
AX-157901453	1	59.114	KV452711.1	2410780	1-scf00010	1
AX-157901462	1	59.114	KV452711.1	2451882	1-scf00010	1
AX-157901492	1	59.114	KV452711.1	2561054	1-scf00010	1
AX-157746342	1	59.114	KV452711.1	2561509	1-scf00010	1
AX-157811507	1	59.114	KV452711.1	2562229	1-scf00010	1
AX-157901510	1	59.114	KV452711.1	2596283	1-scf00010	1
AX-157901511	1	59.114	KV452711.1	2597203	1-scf00010	1
AX-157901522	1	59.114	KV452711.1	2634733	1-scf00010	1
AX-157901545	1	59.114	KV452711.1	2700620	1-scf00010	1
AX-157771059	1	59.114	KV452711.1	2703589	1-scf00010	1
AX-157901548	1	59.114	KV452711.1	2704221	1-scf00010	1
AX-157901549	1	59.114	KV452711.1	2706586	1-scf00010	1
AX-157901551	1	59.114	KV452711.1	2707711	1-scf00010	1
AX-157741562	1	59.114	KV452711.1	2709300	1-scf00010	1
AX-157901554	1	59.114	KV452711.1	2711626	1-scf00010	1
AX-157901556	1	59.114	KV452711.1	2713816	1-scf00010	1
AX-157901557	1	59.114	KV452711.1	2714942	1-scf00010	1
AX-157901558	1	59.114	KV452711.1	2716040	1-scf00010	1

AX-157901559	1	59.114	KV452711.1	2719039	1-scf00010	1
AX-157901560	1	59.114	KV452711.1	2719887	1-scf00010	1
AX-157811515	1	59.114	KV452711.1	2720225	1-scf00010	1
AX-157811516	1	59.114	KV452711.1	2721041	1-scf00010	1
AX-157901562	1	59.114	KV452711.1	2723797	1-scf00010	1
AX-157750627	1	59.114	KV452711.1	2728853	1-scf00010	1
AX-157901574	1	59.114	KV452711.1	2747128	1-scf00010	1
AX-157721039	1	59.114	KV452711.1	2763900	1-scf00010	1
AX-157901586	1	59.114	KV452711.1	2766179	1-scf00010	1
AX-157901609	1	59.114	KV452711.1	2798847	1-scf00010	1
AX-157811523	1	59.114	KV452711.1	2803631	1-scf00010	1
AX-157811524	1	59.114	KV452711.1	2804254	1-scf00010	1
AX-157901613	1	59.114	KV452711.1	2808352	1-scf00010	1
AX-157901614	1	59.114	KV452711.1	2808821	1-scf00010	1
AX-157901616	1	59.114	KV452711.1	2811309	1-scf00010	1
AX-157811525	1	59.114	KV452711.1	2811530	1-scf00010	1
AX-157811526	1	59.114	KV452711.1	2811634	1-scf00010	1
AX-157901618	1	59.114	KV452711.1	2813698	1-scf00010	1
AX-157811536	1	59.114	KV452711.1	2890566	1-scf00010	1
AX-157901649	1	59.114	KV452711.1	2894750	1-scf00010	1
AX-157901661	1	59.114	KV452711.1	2923256	1-scf00010	1
AX-157901668	1	59.114	KV452711.1	2935217	1-scf00010	1
AX-157901682	1	59.114	KV452711.1	2973813	1-scf00010	1
AX-157901696	1	59.114	KV452711.1	3019912	1-scf00010	1
AX-157901700	1	59.114	KV452711.1	3035521	1-scf00010	1
AX-157757927	1	59.114	KV452711.1	3046415	1-scf00010	1
AX-157901726	1	59.114	KV452711.1	3101260	1-scf00010	1
AX-157901729	1	59.114	KV452711.1	3104818	1-scf00010	1
AX-157811553	1	59.114	KV452711.1	3126277	1-scf00010	1
AX-157901736	1	59.114	KV452711.1	3126827	1-scf00010	1
AX-157901737	1	59.114	KV452711.1	3129403	1-scf00010	1
AX-157901741	1	59.114	KV452711.1	3143345	1-scf00010	1
AX-158379159	1	59.114	KV452711.1	3162914	1-scf00010	1
AX-157901747	1	59.114	KV452711.1	3165744	1-scf00010	1
AX-157901753	1	59.114	KV452711.1	3180095	1-scf00010	1
AX-157901754	1	59.114	KV452711.1	3180817	1-scf00010	1
AX-157901755	1	59.114	KV452711.1	3183588	1-scf00010	1
AX-157901757	1	59.114	KV452711.1	3188731	1-scf00010	1
AX-157901759	1	59.114	KV452711.1	3195574	1-scf00010	1
AX-157901760	1	59.114	KV452711.1	3196560	1-scf00010	1
AX-157901762	1	59.114	KV452711.1	3202536	1-scf00010	1
AX-157758203	1	59.114	KV452711.1	3203414	1-scf00010	1
AX-157901772	1	59.114	KV452711.1	3221441	1-scf00010	1
AX-157901778	1	59.114	KV452711.1	3245016	1-scf00010	1
AX-157901785	1	59.114	KV452711.1	3271796	1-scf00010	1
AX-157811570	1	59.114	KV452711.1	3280941	1-scf00010	1
AX-157901787	1	59.114	KV452711.1	3284967	1-scf00010	1
AX-157811571	1	59.114	KV452711.1	3285835	1-scf00010	1
AX-157901788	1	59.114	KV452711.1	3287245	1-scf00010	1
AX-157901789	1	59.114	KV452711.1	3287667	1-scf00010	1
AX-157901790	1	59.114	KV452711.1	3289424	1-scf00010	1

AX-157901791	1	59.114	KV452711.1	3291427	1-scf00010	1
AX-157752559	1	59.114	KV452711.1	3293098	1-scf00010	1
AX-157901792	1	59.114	KV452711.1	3293337	1-scf00010	1
AX-157901793	1	59.114	KV452711.1	3293944	1-scf00010	1
AX-157901794	1	59.114	KV452711.1	3296232	1-scf00010	1
AX-157901795	1	59.114	KV452711.1	3296602	1-scf00010	1
AX-157901797	1	59.114	KV452711.1	3296881	1-scf00010	1
AX-157901798	1	59.114	KV452711.1	3297683	1-scf00010	1
AX-157901800	1	59.114	KV452711.1	3300161	1-scf00010	1
AX-157901801	1	59.114	KV452711.1	3301375	1-scf00010	1
AX-157901802	1	59.114	KV452711.1	3302493	1-scf00010	1
AX-157901803	1	59.114	KV452711.1	3303196	1-scf00010	1
AX-157901806	1	59.114	KV452711.1	3322722	1-scf00010	1
AX-157864417	1	59.114	KV452711.1	3324475	1-scf00010	1
AX-157901808	1	59.114	KV452711.1	3347491	1-scf00010	1
AX-157901809	1	59.114	KV452711.1	3348449	1-scf00010	1
AX-157747264	1	59.114	KV452711.1	3350783	1-scf00010	1
AX-157901810	1	59.114	KV452711.1	3352110	1-scf00010	1
AX-157811576	1	59.114	KV452711.1	3352984	1-scf00010	1
AX-157901811	1	59.114	KV452711.1	3353088	1-scf00010	1
AX-157901813	1	59.114	KV452711.1	3359402	1-scf00010	1
AX-157901814	1	59.114	KV452711.1	3360471	1-scf00010	1
AX-157901815	1	59.114	KV452711.1	3361539	1-scf00010	1
AX-157901816	1	59.114	KV452711.1	3362420	1-scf00010	1
AX-157901818	1	59.114	KV452711.1	3368406	1-scf00010	1
AX-157901819	1	59.114	KV452711.1	3368841	1-scf00010	1
AX-157901822	1	59.114	KV452711.1	3374323	1-scf00010	1
AX-157901823	1	59.114	KV452711.1	3380238	1-scf00010	1
AX-157864419	1	59.114	KV452711.1	3382951	1-scf00010	1
AX-157901826	1	59.114	KV452711.1	3385777	1-scf00010	1
AX-157901833	1	59.114	KV452711.1	3409714	1-scf00010	1
AX-157901834	1	59.114	KV452711.1	3412499	1-scf00010	1
AX-157901838	1	59.114	KV452711.1	3438107	1-scf00010	1
AX-157717441	1	59.114	KV452711.1	3446501	1-scf00010	1
AX-157901844	1	59.114	KV452711.1	3454201	1-scf00010	1
AX-157901849	1	59.114	KV452711.1	3472104	1-scf00010	1
AX-157901850	1	59.114	KV452711.1	3474976	1-scf00010	1
AX-157901851	1	59.114	KV452711.1	3479044	1-scf00010	1
AX-157901854	1	59.114	KV452711.1	3503803	1-scf00010	1
AX-157901855	1	59.114	KV452711.1	3517488	1-scf00010	1
AX-157901856	1	59.114	KV452711.1	3519271	1-scf00010	1
AX-157901857	1	59.114	KV452711.1	3522051	1-scf00010	1
AX-157901858	1	59.114	KV452711.1	3522672	1-scf00010	1
AX-157901859	1	59.114	KV452711.1	3527454	1-scf00010	1
AX-157901860	1	59.114	KV452711.1	3530916	1-scf00010	1
AX-157757382	1	59.114	KV452711.1	3546703	1-scf00010	1
AX-157901862	1	59.114	KV452711.1	3557650	1-scf00010	1
AX-157901866	1	59.114	KV452711.1	3572091	1-scf00010	1
AX-157901869	1	59.114	KV452711.1	3597048	1-scf00010	1
AX-157901872	1	59.114	KV452711.1	3607723	1-scf00010	1
AX-157755874	1	59.114	KV452711.1	3613021	1-scf00010	1

AX-157901896	1	59.114	KV452711.1	3642234	1-scf00010	1
AX-157901900	1	59.114	KV452711.1	3643800	1-scf00010	1
AX-157901903	1	59.114	KV452711.1	3645524	1-scf00010	1
AX-157901919	1	59.114	KV452711.1	3658160	1-scf00010	1
AX-157901926	1	59.114	KV452711.1	3661186	1-scf00010	1
AX-157901929	1	59.114	KV452711.1	3665338	1-scf00010	1
AX-157901933	1	59.114	KV452711.1	3667268	1-scf00010	1
AX-157901984	1	59.114	KV452711.1	3740606	1-scf00010	1
AX-157901990	1	59.114	KV452711.1	3748067	1-scf00010	1
AX-157902065	1	59.114	KV452711.1	3841719	1-scf00010	1
AX-157902072	1	59.114	KV452711.1	3855787	1-scf00010	1
AX-157902073	1	59.114	KV452711.1	3857161	1-scf00010	1
AX-157811622	1	59.114	KV452711.1	3895491	1-scf00010	1
AX-157811635	1	59.114	KV452711.1	4005238	1-scf00010	1
AX-157902182	1	59.114	KV452711.1	4007299	1-scf00010	1
AX-157902184	1	59.114	KV452711.1	4008370	1-scf00010	1
AX-157723378	1	59.114	KV452711.1	4010125	1-scf00010	1
AX-157902186	1	59.114	KV452711.1	4012403	1-scf00010	1
AX-157902192	1	59.114	KV452711.1	4023238	1-scf00010	1
AX-157902193	1	59.114	KV452711.1	4027567	1-scf00010	1
AX-157902197	1	59.114	KV452711.1	4032659	1-scf00010	1
AX-157902199	1	59.114	KV452711.1	4035277	1-scf00010	1
AX-157902200	1	59.114	KV452711.1	4036900	1-scf00010	1
AX-157902201	1	59.114	KV452711.1	4040483	1-scf00010	1
AX-157902202	1	59.114	KV452711.1	4041074	1-scf00010	1
AX-157902208	1	59.114	KV452711.1	4049830	1-scf00010	1
AX-157902218	1	59.114	KV452711.1	4061811	1-scf00010	1
AX-158379160	1	59.114	KV452711.1	4064376	1-scf00010	1
AX-157902220	1	59.114	KV452711.1	4065019	1-scf00010	1
AX-157902224	1	59.114	KV452711.1	4070281	1-scf00010	1
AX-157902225	1	59.114	KV452711.1	4075990	1-scf00010	1
AX-157902227	1	59.114	KV452711.1	4078313	1-scf00010	1
AX-157902231	1	59.114	KV452711.1	4094878	1-scf00010	1
AX-157902233	1	59.114	KV452711.1	4096982	1-scf00010	1
AX-157902235	1	59.114	KV452711.1	4099250	1-scf00010	1
AX-157902236	1	59.114	KV452711.1	4104014	1-scf00010	1
AX-157902238	1	59.114	KV452711.1	4114513	1-scf00010	1
AX-157902239	1	59.114	KV452711.1	4116276	1-scf00010	1
AX-157902241	1	59.114	KV452711.1	4131455	1-scf00010	1
AX-157902242	1	59.114	KV452711.1	4140370	1-scf00010	1
AX-157902244	1	59.114	KV452711.1	4147911	1-scf00010	1
AX-157902245	1	59.114	KV452711.1	4149092	1-scf00010	1
AX-157902248	1	59.114	KV452711.1	4182494	1-scf00010	1
AX-157902249	1	59.114	KV452711.1	4185174	1-scf00010	1
AX-157902250	1	59.114	KV452711.1	4190154	1-scf00010	1
AX-157902251	1	59.114	KV452711.1	4212224	1-scf00010	1
AX-157902252	1	59.114	KV452711.1	4223944	1-scf00010	1
AX-157902255	1	59.114	KV452711.1	4243780	1-scf00010	1
AX-157902256	1	59.114	KV452711.1	4244532	1-scf00010	1
AX-157902257	1	59.114	KV452711.1	4258935	1-scf00010	1
AX-157902258	1	59.114	KV452711.1	4260659	1-scf00010	1

AX-157902260	1	59.114	KV452711.1	4267578	1-scf00010	1
AX-157902261	1	59.114	KV452711.1	4267880	1-scf00010	1
AX-157902265	1	59.114	KV452711.1	4283799	1-scf00010	1
AX-157902266	1	59.114	KV452711.1	4285078	1-scf00010	1
AX-157902267	1	59.114	KV452711.1	4286755	1-scf00010	1
AX-157902268	1	59.114	KV452711.1	4292191	1-scf00010	1
AX-157902269	1	59.114	KV452711.1	4298408	1-scf00010	1
AX-157902279	1	59.114	KV452713.1	9828	1-scf00011	1
AX-157902280	1	59.114	KV452713.1	19407	1-scf00011	1
AX-157902281	1	59.114	KV452713.1	19878	1-scf00011	1
AX-157902284	1	59.114	KV452713.1	39226	1-scf00011	1
AX-157902285	1	59.114	KV452713.1	41397	1-scf00011	1
AX-157902286	1	59.114	KV452713.1	43459	1-scf00011	1
AX-157902288	1	59.114	KV452713.1	48960	1-scf00011	1
AX-157902289	1	59.114	KV452713.1	53746	1-scf00011	1
AX-157902290	1	59.114	KV452713.1	55085	1-scf00011	1
AX-157902291	1	59.114	KV452713.1	60199	1-scf00011	1
AX-157902292	1	59.114	KV452713.1	61232	1-scf00011	1
AX-157902293	1	59.114	KV452713.1	81131	1-scf00011	1
AX-157902295	1	59.114	KV452713.1	104221	1-scf00011	1
AX-157902298	1	59.114	KV452713.1	108136	1-scf00011	1
AX-157902299	1	59.114	KV452713.1	121018	1-scf00011	1
AX-157902300	1	59.114	KV452713.1	121286	1-scf00011	1
AX-157902304	1	59.114	KV452713.1	140866	1-scf00011	1
AX-157902305	1	59.114	KV452713.1	146205	1-scf00011	1
AX-157902307	1	59.114	KV452713.1	148657	1-scf00011	1
AX-157902309	1	59.114	KV452713.1	155149	1-scf00011	1
AX-157902313	1	59.114	KV452713.1	173722	1-scf00011	1
AX-157902314	1	59.114	KV452713.1	178886	1-scf00011	1
AX-157902316	1	59.114	KV452713.1	207684	1-scf00011	1
AX-157902319	1	59.114	KV452713.1	225136	1-scf00011	1
AX-157902322	1	59.114	KV452713.1	245015	1-scf00011	1
AX-157902324	1	59.114	KV452713.1	276898	1-scf00011	1
AX-157902327	1	59.114	KV452713.1	296153	1-scf00011	1
AX-157902328	1	59.114	KV452713.1	296412	1-scf00011	1
AX-157902333	1	59.114	KV452713.1	319925	1-scf00011	1
AX-157902334	1	59.114	KV452713.1	320962	1-scf00011	1
AX-157902335	1	59.114	KV452713.1	322601	1-scf00011	1
AX-157902336	1	59.114	KV452713.1	337404	1-scf00011	1
AX-157902337	1	59.114	KV452713.1	340806	1-scf00011	1
AX-157902338	1	59.114	KV452713.1	344870	1-scf00011	1
AX-157811640	1	59.114	KV452713.1	358326	1-scf00011	1
AX-157902342	1	59.114	KV452713.1	374577	1-scf00011	1
AX-157902343	1	59.114	KV452713.1	380579	1-scf00011	1
AX-157902344	1	59.114	KV452713.1	381374	1-scf00011	1
AX-157902346	1	59.114	KV452713.1	382883	1-scf00011	1
AX-157811641	1	59.114	KV452713.1	390347	1-scf00011	1
AX-157902349	1	59.114	KV452713.1	391337	1-scf00011	1
AX-157902350	1	59.114	KV452713.1	394659	1-scf00011	1
AX-157902351	1	59.114	KV452713.1	398263	1-scf00011	1
AX-157902358	1	59.114	KV452713.1	432943	1-scf00011	1

AX-157902359	1	59.114	KV452713.1	437582	1-scf00011	1
AX-157902362	1	59.114	KV452713.1	455747	1-scf00011	1
AX-157902368	1	59.114	KV452713.1	484356	1-scf00011	1
AX-157902370	1	59.114	KV452713.1	491391	1-scf00011	1
AX-157902371	1	59.114	KV452713.1	502254	1-scf00011	1
AX-158403988	1	59.114	KV452713.1	507316	1-scf00011	1
AX-158404631	1	59.114	KV452713.1	507996	1-scf00011	1
AX-157902372	1	59.114	KV452713.1	512077	1-scf00011	1
AX-158404633	1	59.114	KV452713.1	515526	1-scf00011	1
AX-157902373	1	59.114	KV452713.1	515692	1-scf00011	1
AX-157902374	1	59.114	KV452713.1	518533	1-scf00011	1
AX-157902377	1	59.114	KV452713.1	521977	1-scf00011	1
AX-157902378	1	59.114	KV452713.1	525011	1-scf00011	1
AX-158414656	1	59.114	KV452713.1	526104	1-scf00011	1
AX-157902380	1	59.114	KV452713.1	527936	1-scf00011	1
AX-157902381	1	59.114	KV452713.1	529458	1-scf00011	1
AX-157811643	1	59.114	KV452713.1	529934	1-scf00011	1
AX-157902382	1	59.114	KV452713.1	531882	1-scf00011	1
AX-157811644	1	59.114	KV452713.1	533060	1-scf00011	1
AX-157902384	1	59.114	KV452713.1	533515	1-scf00011	1
AX-157902385	1	59.114	KV452713.1	536101	1-scf00011	1
AX-157902387	1	59.114	KV452713.1	538301	1-scf00011	1
AX-157811645	1	59.114	KV452713.1	539928	1-scf00011	1
AX-157811646	1	59.114	KV452713.1	540697	1-scf00011	1
AX-157811647	1	59.114	KV452713.1	540759	1-scf00011	1
AX-157811648	1	59.114	KV452713.1	542342	1-scf00011	1
AX-157902388	1	59.114	KV452713.1	542425	1-scf00011	1
AX-157902390	1	59.114	KV452713.1	545200	1-scf00011	1
AX-157902391	1	59.114	KV452713.1	550004	1-scf00011	1
AX-157902396	1	59.114	KV452713.1	589441	1-scf00011	1
AX-157902402	1	59.114	KV452713.1	621077	1-scf00011	1
AX-157902405	1	59.114	KV452713.1	624453	1-scf00011	1
AX-157902407	1	59.114	KV452713.1	625456	1-scf00011	1
AX-157902408	1	59.114	KV452713.1	626894	1-scf00011	1
AX-157902409	1	59.114	KV452713.1	629895	1-scf00011	1
AX-157811655	1	59.114	KV452713.1	639779	1-scf00011	1
AX-157811657	1	59.114	KV452713.1	645861	1-scf00011	1
AX-157902419	1	59.114	KV452713.1	668938	1-scf00011	1
AX-157811665	1	59.114	KV452713.1	672518	1-scf00011	1
AX-157902420	1	59.114	KV452713.1	676018	1-scf00011	1
AX-157902424	1	59.114	KV452713.1	679422	1-scf00011	1
AX-158404637	1	59.114	KV452713.1	697490	1-scf00011	1
AX-157902430	1	59.114	KV452713.1	704154	1-scf00011	1
AX-157902433	1	59.114	KV452713.1	711656	1-scf00011	1
AX-157902434	1	59.114	KV452713.1	712014	1-scf00011	1
AX-157902435	1	59.114	KV452713.1	712360	1-scf00011	1
AX-157902436	1	59.114	KV452713.1	718298	1-scf00011	1
AX-157902446	1	59.114	KV452713.1	739798	1-scf00011	1
AX-157902447	1	59.114	KV452713.1	741419	1-scf00011	1
AX-157902448	1	59.114	KV452713.1	742810	1-scf00011	1
AX-157902449	1	59.114	KV452713.1	744030	1-scf00011	1

AX-157902450	1	59.114	KV452713.1	749488	1-scf00011	1
AX-157902451	1	59.114	KV452713.1	758375	1-scf00011	1
AX-157902463	1	59.114	KV452713.1	777863	1-scf00011	1
AX-157902464	1	59.114	KV452713.1	778241	1-scf00011	1
AX-157902471	1	59.114	KV452713.1	789080	1-scf00011	1
AX-157902475	1	59.114	KV452713.1	794806	1-scf00011	1
AX-157811670	1	59.114	KV452713.1	795765	1-scf00011	1
AX-157902476	1	59.114	KV452713.1	795981	1-scf00011	1
AX-157902477	1	59.114	KV452713.1	796723	1-scf00011	1
AX-157902478	1	59.114	KV452713.1	797507	1-scf00011	1
AX-157902486	1	59.114	KV452713.1	807024	1-scf00011	1
AX-157902488	1	59.114	KV452713.1	808209	1-scf00011	1
AX-157715021	1	59.114	KV452713.1	823455	1-scf00011	1
AX-157902495	1	59.114	KV452713.1	825551	1-scf00011	1
AX-157902496	1	59.114	KV452713.1	825963	1-scf00011	1
AX-157749569	1	59.114	KV452713.1	830336	1-scf00011	1
AX-157902498	1	59.114	KV452713.1	830947	1-scf00011	1
AX-157902502	1	59.114	KV452713.1	833367	1-scf00011	1
AX-157902505	1	59.114	KV452713.1	835315	1-scf00011	1
AX-157902508	1	59.114	KV452713.1	852528	1-scf00011	1
AX-157902511	1	59.114	KV452713.1	860136	1-scf00011	1
AX-157902520	1	59.114	KV452713.1	878869	1-scf00011	1
AX-157902521	1	59.114	KV452713.1	880576	1-scf00011	1
AX-157902522	1	59.114	KV452713.1	882537	1-scf00011	1
AX-157902523	1	59.114	KV452713.1	883985	1-scf00011	1
AX-157902524	1	59.114	KV452713.1	885831	1-scf00011	1
AX-157902527	1	59.114	KV452713.1	888382	1-scf00011	1
AX-157902528	1	59.114	KV452713.1	889417	1-scf00011	1
AX-157902533	1	59.114	KV452713.1	897484	1-scf00011	1
AX-157902534	1	59.114	KV452713.1	897780	1-scf00011	1
AX-157902537	1	59.114	KV452713.1	906838	1-scf00011	1
AX-157902538	1	59.114	KV452713.1	908677	1-scf00011	1
AX-157902539	1	59.114	KV452713.1	909706	1-scf00011	1
AX-157811676	1	59.114	KV452713.1	917464	1-scf00011	1
AX-157902546	1	59.114	KV452713.1	924739	1-scf00011	1
AX-157902547	1	59.114	KV452713.1	931684	1-scf00011	1
AX-157902548	1	59.114	KV452713.1	933118	1-scf00011	1
AX-157811678	1	59.114	KV452713.1	936065	1-scf00011	1
AX-157902552	1	59.114	KV452713.1	943024	1-scf00011	1
AX-157902553	1	59.114	KV452713.1	943528	1-scf00011	1
AX-157902554	1	59.114	KV452713.1	944587	1-scf00011	1
AX-157902559	1	59.114	KV452713.1	954331	1-scf00011	1
AX-157902562	1	59.114	KV452713.1	959155	1-scf00011	1
AX-157902563	1	59.114	KV452713.1	961598	1-scf00011	1
AX-157902565	1	59.114	KV452713.1	962579	1-scf00011	1
AX-157902571	1	59.114	KV452713.1	970165	1-scf00011	1
AX-157902572	1	59.114	KV452713.1	972251	1-scf00011	1
AX-157902579	1	59.114	KV452713.1	977896	1-scf00011	1
AX-158421823	1	59.114	KV452713.1	979709	1-scf00011	1
AX-157902581	1	59.114	KV452713.1	981029	1-scf00011	1
AX-157902586	1	59.114	KV452713.1	987507	1-scf00011	1

AX-157902587	1	59.114	KV452713.1	989698	1-scf00011	1
AX-157902590	1	59.114	KV452713.1	992037	1-scf00011	1
AX-157811680	1	59.114	KV452713.1	993748	1-scf00011	1
AX-157902595	1	59.114	KV452713.1	1000332	1-scf00011	1
AX-157902597	1	59.114	KV452713.1	1004156	1-scf00011	1
AX-157902599	1	59.114	KV452713.1	1007697	1-scf00011	1
AX-157902600	1	59.114	KV452713.1	1010811	1-scf00011	1
AX-157811682	1	59.114	KV452713.1	1012878	1-scf00011	1
AX-157902605	1	59.114	KV452713.1	1021136	1-scf00011	1
AX-157902610	1	59.114	KV452713.1	1026339	1-scf00011	1
AX-157902615	1	59.114	KV452713.1	1032096	1-scf00011	1
AX-157902616	1	59.114	KV452713.1	1032366	1-scf00011	1
AX-157902617	1	59.114	KV452713.1	1032445	1-scf00011	1
AX-157902618	1	59.114	KV452713.1	1033486	1-scf00011	1
AX-157902619	1	59.114	KV452713.1	1035113	1-scf00011	1
AX-157902620	1	59.114	KV452713.1	1036065	1-scf00011	1
AX-157902626	1	59.114	KV452713.1	1042824	1-scf00011	1
AX-157902628	1	59.114	KV452713.1	1043601	1-scf00011	1
AX-157902632	1	59.114	KV452713.1	1045480	1-scf00011	1
AX-157902633	1	59.114	KV452713.1	1046107	1-scf00011	1
AX-157902635	1	59.114	KV452713.1	1048103	1-scf00011	1
AX-157902638	1	59.114	KV452713.1	1052245	1-scf00011	1
AX-157902642	1	59.114	KV452713.1	1058725	1-scf00011	1
AX-157902644	1	59.114	KV452713.1	1061864	1-scf00011	1
AX-157902645	1	59.114	KV452713.1	1063224	1-scf00011	1
AX-157811685	1	59.114	KV452713.1	1067960	1-scf00011	1
AX-157902650	1	59.114	KV452713.1	1070063	1-scf00011	1
AX-157902651	1	59.114	KV452713.1	1070627	1-scf00011	1
AX-157902655	1	59.114	KV452713.1	1079362	1-scf00011	1
AX-157902656	1	59.114	KV452713.1	1080537	1-scf00011	1
AX-157902659	1	59.114	KV452713.1	1082040	1-scf00011	1
AX-157902685	1	59.114	KV452713.1	1130197	1-scf00011	1
AX-157902692	1	59.114	KV452713.1	1139146	1-scf00011	1
AX-157902707	1	59.114	KV452713.1	1153597	1-scf00011	1
AX-157902723	1	59.114	KV452713.1	1170681	1-scf00011	1
AX-157747231	1	59.114	KV452713.1	1204000	1-scf00011	1
AX-157902756	1	59.114	KV452713.1	1216894	1-scf00011	1
AX-157902800	1	59.114	KV452713.1	1282202	1-scf00011	1
AX-157902829	1	59.114	KV452713.1	1323695	1-scf00011	1
AX-157902830	1	59.114	KV452713.1	1325704	1-scf00011	1
AX-157902832	1	59.114	KV452713.1	1328229	1-scf00011	1
AX-157902835	1	59.114	KV452713.1	1333297	1-scf00011	1
AX-157902896	1	59.114	KV452713.1	1431418	1-scf00011	1
AX-157902922	1	59.114	KV452713.1	1467462	1-scf00011	1
AX-158389390	1	59.114	KV452713.1	1494239	1-scf00011	1
AX-157903004	1	59.114	KV452713.1	1564414	1-scf00011	1
AX-157903006	1	59.114	KV452713.1	1567246	1-scf00011	1
AX-157811713	1	59.114	KV452713.1	1567425	1-scf00011	1
AX-157903008	1	59.114	KV452713.1	1569907	1-scf00011	1
AX-157903010	1	59.114	KV452713.1	1575407	1-scf00011	1
AX-157903012	1	59.114	KV452713.1	1578374	1-scf00011	1

AX-157903013	1	59.114	KV452713.1	1578968	1-scf00011	1
AX-157853606	1	59.114	KV452713.1	1798220	1-scf00011	1
AX-157811739	1	59.114	KV452713.1	1915860	1-scf00011	1
AX-157903296	1	59.114	KV452713.1	1959542	1-scf00011	1
AX-157903355	1	59.114	KV452713.1	2036865	1-scf00011	1
AX-157903367	1	59.114	KV452713.1	2053157	1-scf00011	1
AX-157903369	1	59.114	KV452713.1	2054753	1-scf00011	1
AX-157903370	1	59.114	KV452713.1	2055324	1-scf00011	1
AX-157903372	1	59.114	KV452713.1	2059692	1-scf00011	1
AX-157903373	1	59.114	KV452713.1	2059935	1-scf00011	1
AX-157903374	1	59.114	KV452713.1	2060564	1-scf00011	1
AX-157903508	1	59.114	KV452713.1	2198154	1-scf00011	1
AX-157903540	1	59.114	KV452713.1	2231164	1-scf00011	1
AX-157696977	1	59.114	KV452713.1	2292678	1-scf00011	1
AX-157903750	1	59.114	KV452713.1	2488518	1-scf00011	1
AX-158414680	1	59.114	KV452713.1	2597161	1-scf00011	1
AX-158404677	1	59.114	KV452713.1	2617743	1-scf00011	1
AX-157903871	1	59.114	KV452713.1	2651900	1-scf00011	1
AX-157719119	1	59.114	KV452713.1	2659863	1-scf00011	1
AX-157903876	1	59.114	KV452713.1	2660171	1-scf00011	1
AX-157903901	1	59.114	KV452713.1	2679748	1-scf00011	1
AX-157811788	1	59.114	KV452713.1	2680983	1-scf00011	1
AX-157903904	1	59.114	KV452713.1	2683841	1-scf00011	1
AX-157868917	1	59.114	KV452713.1	2683962	1-scf00011	1
AX-157903908	1	59.114	KV452713.1	2686391	1-scf00011	1
AX-157903909	1	59.114	KV452713.1	2686958	1-scf00011	1
AX-157903911	1	59.114	KV452713.1	2688118	1-scf00011	1
AX-158404682	1	59.114	KV452713.1	2694353	1-scf00011	1
AX-157903919	1	59.114	KV452713.1	2699784	1-scf00011	1
AX-157903922	1	59.114	KV452713.1	2701529	1-scf00011	1
AX-157725472	1	59.114	KV452713.1	2738960	1-scf00011	1
AX-157868920	1	59.114	KV452713.1	2742045	1-scf00011	1
AX-157904000	1	59.114	KV452713.1	2848737	1-scf00011	1
AX-157811796	1	59.114	KV452713.1	2852172	1-scf00011	1
AX-157727367	1	59.114	KV452713.1	2856366	1-scf00011	1
AX-157904007	1	59.114	KV452713.1	2865631	1-scf00011	1
AX-157708854	1	59.114	KV452713.1	2897046	1-scf00011	1
AX-157904038	1	59.114	KV452713.1	2927965	1-scf00011	1
AX-157904086	1	59.114	KV452713.1	3025407	1-scf00011	1
AX-157904113	1	59.114	KV452713.1	3064093	1-scf00011	1
AX-157904467	1	59.114	KV452713.1	3798955	1-scf00011	1
AX-157904474	1	59.114	KV452713.1	3807352	1-scf00011	1
AX-157904506	1	59.114	KV452713.1	3860284	1-scf00011	1
AX-157904515	1	59.114	KV452713.1	3874499	1-scf00011	1
AX-157811832	1	59.114	KV452713.1	3931841	1-scf00011	1
AX-157904618	1	59.114	KV452713.1	4028423	1-scf00011	1
AX-157904661	1	59.114	KV452713.1	4092503	1-scf00011	1
AX-157904662	1	59.114	KV452713.1	4093351	1-scf00011	1
AX-157757259	1	59.114	KV452713.1	4513621	1-scf00011	1
AX-157811263	1	59.145	KV452709.1	4903783	1-scf00009	1
AX-157899287	1	59.145	KV452709.1	4971807	1-scf00009	1

AX-157811274	1	59.145	KV452709.1	5076320	1-scf00009	1
AX-157899992	1	59.145	KV452709.1	5882886	1-scf00009	1
AX-157901389	1	59.145	KV452711.1	2203814	1-scf00010	1
AX-157901419	1	59.145	KV452711.1	2283339	1-scf00010	1
AX-157901541	1	59.145	KV452711.1	2694145	1-scf00010	1
AX-157901686	1	59.145	KV452711.1	2991423	1-scf00010	1
AX-157747677	1	59.145	KV452711.1	3157835	1-scf00010	1
AX-157811658	1	59.145	KV452713.1	647856	1-scf00011	1
AX-157902429	1	59.145	KV452713.1	701450	1-scf00011	1
AX-157902530	1	59.145	KV452713.1	891062	1-scf00011	1
AX-157864424	1	59.145	KV452713.1	893985	1-scf00011	1
AX-157902564	1	59.145	KV452713.1	962058	1-scf00011	1
AX-157902665	1	59.145	KV452713.1	1096004	1-scf00011	1
AX-157902821	1	59.145	KV452713.1	1313758	1-scf00011	1
AX-157811783	1	59.145	KV452713.1	2481904	1-scf00011	1
AX-157754203	1	59.145	KV452713.1	3924684	1-scf00011	1
AX-157904543	1	59.145	KV452713.1	3924879	1-scf00011	1
AX-157902686	1	59.166	KV452713.1	1132261	1-scf00011	1
AX-157903088	1	59.166	KV452713.1	1674792	1-scf00011	1
AX-157811793	1	59.166	KV452713.1	2828906	1-scf00011	1
AX-157904541	1	59.166	KV452713.1	3923523	1-scf00011	1
AX-157719713	1	59.223	KV452708.1	1045752	1-scf00008	1
AX-157896913	1	59.223	KV452709.1	1080831	1-scf00009	1
AX-158404396	1	59.223	KV452709.1	1175728	1-scf00009	1
AX-157896975	1	59.223	KV452709.1	1186314	1-scf00009	1
AX-157896988	1	59.223	KV452709.1	1214131	1-scf00009	1
AX-157897214	1	59.223	KV452709.1	1557166	1-scf00009	1
AX-157897219	1	59.223	KV452709.1	1561091	1-scf00009	1
AX-157897220	1	59.223	KV452709.1	1562664	1-scf00009	1
AX-157897221	1	59.223	KV452709.1	1563304	1-scf00009	1
AX-157897245	1	59.223	KV452709.1	1587930	1-scf00009	1
AX-157897268	1	59.223	KV452709.1	1609582	1-scf00009	1
AX-157897297	1	59.223	KV452709.1	1657690	1-scf00009	1
AX-157811033	1	59.223	KV452709.1	1998146	1-scf00009	1
AX-157897594	1	59.223	KV452709.1	2127405	1-scf00009	1
AX-157898014	1	59.223	KV452709.1	2820167	1-scf00009	1
AX-157750156	1	59.223	KV452709.1	3256500	1-scf00009	1
AX-158403924	1	59.223	KV452709.1	3909444	1-scf00009	1
AX-157898551	1	59.223	KV452709.1	3925691	1-scf00009	1
AX-157898688	1	59.223	KV452709.1	4109986	1-scf00009	1
AX-157898972	1	59.223	KV452709.1	4512019	1-scf00009	1
AX-157899211	1	59.223	KV452709.1	4870004	1-scf00009	1
AX-157899265	1	59.223	KV452709.1	4939373	1-scf00009	1
AX-157899417	1	59.223	KV452709.1	5143053	1-scf00009	1
AX-157899453	1	59.223	KV452709.1	5196906	1-scf00009	1
AX-157899458	1	59.223	KV452709.1	5204806	1-scf00009	1
AX-157899507	1	59.223	KV452709.1	5258365	1-scf00009	1
AX-157899508	1	59.223	KV452709.1	5259308	1-scf00009	1
AX-157811295	1	59.223	KV452709.1	5345292	1-scf00009	1
AX-157899649	1	59.223	KV452709.1	5429118	1-scf00009	1
AX-157746131	1	59.223	KV452709.1	5483884	1-scf00009	1

AX-157811356	1	59.223	KV452709.1	5921011	1-scf00009	1
AX-157772457	1	59.223	KV452709.1	6116915	1-scf00009	1
AX-157900143	1	59.223	KV452709.1	6127638	1-scf00009	1
AX-158404507	1	59.223	KV452709.1	6129687	1-scf00009	1
AX-157900150	1	59.223	KV452709.1	6136159	1-scf00009	1
AX-157748739	1	59.223	KV452709.1	6142507	1-scf00009	1
AX-157900232	1	59.223	KV452709.1	6285928	1-scf00009	1
AX-157900234	1	59.223	KV452709.1	6289799	1-scf00009	1
AX-157754936	1	59.223	KV452709.1	6363284	1-scf00009	1
AX-157900378	1	59.223	KV452709.1	6665898	1-scf00009	1
AX-157900385	1	59.223	KV452709.1	6714113	1-scf00009	1
AX-157900394	1	59.223	KV452709.1	6778359	1-scf00009	1
AX-157864403	1	59.223	KV452709.1	7265562	1-scf00009	1
AX-157900557	1	59.223	KV452709.1	7427312	1-scf00009	1
AX-157900567	1	59.223	KV452709.1	7458090	1-scf00009	1
AX-157900571	1	59.223	KV452709.1	7463537	1-scf00009	1
AX-157900572	1	59.223	KV452709.1	7464742	1-scf00009	1
AX-157900578	1	59.223	KV452709.1	7477057	1-scf00009	1
AX-157900582	1	59.223	KV452709.1	7481692	1-scf00009	1
AX-157900585	1	59.223	KV452709.1	7489862	1-scf00009	1
AX-157900590	1	59.223	KV452709.1	7504860	1-scf00009	1
AX-157900593	1	59.223	KV452709.1	7523134	1-scf00009	1
AX-157900594	1	59.223	KV452709.1	7524170	1-scf00009	1
AX-157900596	1	59.223	KV452709.1	7530748	1-scf00009	1
AX-157900601	1	59.223	KV452709.1	7547935	1-scf00009	1
AX-157724383	1	59.223	KV452709.1	7548297	1-scf00009	1
AX-157900605	1	59.223	KV452709.1	7553294	1-scf00009	1
AX-157900606	1	59.223	KV452709.1	7553800	1-scf00009	1
AX-157900614	1	59.223	KV452709.1	7576681	1-scf00009	1
AX-157900615	1	59.223	KV452709.1	7583499	1-scf00009	1
AX-157900618	1	59.223	KV452709.1	7598230	1-scf00009	1
AX-157900619	1	59.223	KV452709.1	7598738	1-scf00009	1
AX-157900621	1	59.223	KV452709.1	7605374	1-scf00009	1
AX-157900624	1	59.223	KV452709.1	7609556	1-scf00009	1
AX-157900629	1	59.223	KV452709.1	7617733	1-scf00009	1
AX-157900632	1	59.223	KV452709.1	7622117	1-scf00009	1
AX-157900634	1	59.223	KV452709.1	7625685	1-scf00009	1
AX-157900640	1	59.223	KV452709.1	7639340	1-scf00009	1
AX-157900642	1	59.223	KV452709.1	7643816	1-scf00009	1
AX-157900646	1	59.223	KV452709.1	7655527	1-scf00009	1
AX-157900649	1	59.223	KV452709.1	7661776	1-scf00009	1
AX-157900653	1	59.223	KV452709.1	7676927	1-scf00009	1
AX-157900654	1	59.223	KV452709.1	7684859	1-scf00009	1
AX-157900657	1	59.223	KV452709.1	7697615	1-scf00009	1
AX-157900670	1	59.223	KV452709.1	7723949	1-scf00009	1
AX-157900673	1	59.223	KV452709.1	7728109	1-scf00009	1
AX-157900684	1	59.223	KV452709.1	7749242	1-scf00009	1
AX-157900688	1	59.223	KV452709.1	7763075	1-scf00009	1
AX-157900689	1	59.223	KV452709.1	7763564	1-scf00009	1
AX-157900693	1	59.223	KV452709.1	7771067	1-scf00009	1
AX-157900701	1	59.223	KV452709.1	7784427	1-scf00009	1

AX-157900703	1	59.223	KV452709.1	7786351	1-scf00009	1
AX-157900712	1	59.223	KV452709.1	7797491	1-scf00009	1
AX-157900724	1	59.223	KV452709.1	7849042	1-scf00009	1
AX-157900747	1	59.223	KV452711.1	23344	1-scf00010	1
AX-157874387	1	59.223	KV452711.1	45838	1-scf00010	1
AX-158382012	1	59.223	KV452711.1	47526	1-scf00010	1
AX-157900900	1	59.223	KV452711.1	708179	1-scf00010	1
AX-157901013	1	59.223	KV452711.1	1216193	1-scf00010	1
AX-157901027	1	59.223	KV452711.1	1249758	1-scf00010	1
AX-157901056	1	59.223	KV452711.1	1341609	1-scf00010	1
AX-157901064	1	59.223	KV452711.1	1351035	1-scf00010	1
AX-157901101	1	59.223	KV452711.1	1477824	1-scf00010	1
AX-157901103	1	59.223	KV452711.1	1484065	1-scf00010	1
AX-157901224	1	59.223	KV452711.1	1791193	1-scf00010	1
AX-157901248	1	59.223	KV452711.1	1846729	1-scf00010	1
AX-157901252	1	59.223	KV452711.1	1854201	1-scf00010	1
AX-157901263	1	59.223	KV452711.1	1883789	1-scf00010	1
AX-157901325	1	59.223	KV452711.1	2008678	1-scf00010	1
AX-157901477	1	59.223	KV452711.1	2492674	1-scf00010	1
AX-157901542	1	59.223	KV452711.1	2696951	1-scf00010	1
AX-157901610	1	59.223	KV452711.1	2799219	1-scf00010	1
AX-157811522	1	59.223	KV452711.1	2803269	1-scf00010	1
AX-157901617	1	59.223	KV452711.1	2813269	1-scf00010	1
AX-157901620	1	59.223	KV452711.1	2822344	1-scf00010	1
AX-157901623	1	59.223	KV452711.1	2828611	1-scf00010	1
AX-157901625	1	59.223	KV452711.1	2830996	1-scf00010	1
AX-157901633	1	59.223	KV452711.1	2860921	1-scf00010	1
AX-157901637	1	59.223	KV452711.1	2866697	1-scf00010	1
AX-157901665	1	59.223	KV452711.1	2931942	1-scf00010	1
AX-157901675	1	59.223	KV452711.1	2950020	1-scf00010	1
AX-157901692	1	59.223	KV452711.1	3014048	1-scf00010	1
AX-157901713	1	59.223	KV452711.1	3065246	1-scf00010	1
AX-157902277	1	59.223	KV452713.1	3884	1-scf00011	1
AX-157902394	1	59.223	KV452713.1	585353	1-scf00011	1
AX-157902411	1	59.223	KV452713.1	635883	1-scf00011	1
AX-157902412	1	59.223	KV452713.1	637864	1-scf00011	1
AX-157811659	1	59.223	KV452713.1	649954	1-scf00011	1
AX-157902428	1	59.223	KV452713.1	697317	1-scf00011	1
AX-157902529	1	59.223	KV452713.1	890397	1-scf00011	1
AX-158389374	1	59.223	KV452713.1	972101	1-scf00011	1
AX-157749938	1	59.223	KV452713.1	1005551	1-scf00011	1
AX-157721446	1	59.223	KV452713.1	1010307	1-scf00011	1
AX-158379162	1	59.223	KV452713.1	1010440	1-scf00011	1
AX-157864426	1	59.223	KV452713.1	1038761	1-scf00011	1
AX-157902622	1	59.223	KV452713.1	1039073	1-scf00011	1
AX-157902629	1	59.223	KV452713.1	1044002	1-scf00011	1
AX-157902640	1	59.223	KV452713.1	1053697	1-scf00011	1
AX-157902648	1	59.223	KV452713.1	1067650	1-scf00011	1
AX-157902654	1	59.223	KV452713.1	1076418	1-scf00011	1
AX-157902657	1	59.223	KV452713.1	1081155	1-scf00011	1
AX-158403992	1	59.223	KV452713.1	1090945	1-scf00011	1

AX-157902662	1	59.223	KV452713.1	1092175	1-scf00011	1
AX-157902667	1	59.223	KV452713.1	1101605	1-scf00011	1
AX-157902670	1	59.223	KV452713.1	1107542	1-scf00011	1
AX-157902680	1	59.223	KV452713.1	1126176	1-scf00011	1
AX-157902694	1	59.223	KV452713.1	1140998	1-scf00011	1
AX-157902695	1	59.223	KV452713.1	1141470	1-scf00011	1
AX-157902697	1	59.223	KV452713.1	1142709	1-scf00011	1
AX-157902703	1	59.223	KV452713.1	1148763	1-scf00011	1
AX-157902705	1	59.223	KV452713.1	1152009	1-scf00011	1
AX-157902706	1	59.223	KV452713.1	1153231	1-scf00011	1
AX-157902709	1	59.223	KV452713.1	1155143	1-scf00011	1
AX-157902712	1	59.223	KV452713.1	1160010	1-scf00011	1
AX-157902713	1	59.223	KV452713.1	1162113	1-scf00011	1
AX-157902715	1	59.223	KV452713.1	1163594	1-scf00011	1
AX-157902716	1	59.223	KV452713.1	1164298	1-scf00011	1
AX-157902717	1	59.223	KV452713.1	1165409	1-scf00011	1
AX-157902718	1	59.223	KV452713.1	1166202	1-scf00011	1
AX-157902719	1	59.223	KV452713.1	1166978	1-scf00011	1
AX-157902729	1	59.223	KV452713.1	1178612	1-scf00011	1
AX-157902731	1	59.223	KV452713.1	1180711	1-scf00011	1
AX-157902732	1	59.223	KV452713.1	1181396	1-scf00011	1
AX-157902733	1	59.223	KV452713.1	1181815	1-scf00011	1
AX-157902734	1	59.223	KV452713.1	1182325	1-scf00011	1
AX-157902735	1	59.223	KV452713.1	1183255	1-scf00011	1
AX-157902736	1	59.223	KV452713.1	1183887	1-scf00011	1
AX-157902738	1	59.223	KV452713.1	1186323	1-scf00011	1
AX-157902740	1	59.223	KV452713.1	1187824	1-scf00011	1
AX-157902742	1	59.223	KV452713.1	1191343	1-scf00011	1
AX-157902745	1	59.223	KV452713.1	1194486	1-scf00011	1
AX-157902746	1	59.223	KV452713.1	1196429	1-scf00011	1
AX-157902747	1	59.223	KV452713.1	1197021	1-scf00011	1
AX-157811690	1	59.223	KV452713.1	1198346	1-scf00011	1
AX-157811691	1	59.223	KV452713.1	1204063	1-scf00011	1
AX-157902751	1	59.223	KV452713.1	1207705	1-scf00011	1
AX-157902752	1	59.223	KV452713.1	1208065	1-scf00011	1
AX-157902755	1	59.223	KV452713.1	1213771	1-scf00011	1
AX-157902757	1	59.223	KV452713.1	1217262	1-scf00011	1
AX-157902760	1	59.223	KV452713.1	1225424	1-scf00011	1
AX-157902762	1	59.223	KV452713.1	1226103	1-scf00011	1
AX-157811692	1	59.223	KV452713.1	1226393	1-scf00011	1
AX-157902763	1	59.223	KV452713.1	1226658	1-scf00011	1
AX-157712190	1	59.223	KV452713.1	1227904	1-scf00011	1
AX-157902765	1	59.223	KV452713.1	1229580	1-scf00011	1
AX-157902766	1	59.223	KV452713.1	1229784	1-scf00011	1
AX-157902769	1	59.223	KV452713.1	1232788	1-scf00011	1
AX-157902770	1	59.223	KV452713.1	1233506	1-scf00011	1
AX-157902771	1	59.223	KV452713.1	1234642	1-scf00011	1
AX-158404650	1	59.223	KV452713.1	1242268	1-scf00011	1
AX-157811693	1	59.223	KV452713.1	1255295	1-scf00011	1
AX-157902780	1	59.223	KV452713.1	1259555	1-scf00011	1
AX-157717882	1	59.223	KV452713.1	1259656	1-scf00011	1

AX-157902781	1	59.223	KV452713.1	1262489	1-scf00011	1
AX-157902782	1	59.223	KV452713.1	1263139	1-scf00011	1
AX-157902783	1	59.223	KV452713.1	1263935	1-scf00011	1
AX-157902784	1	59.223	KV452713.1	1264125	1-scf00011	1
AX-157902785	1	59.223	KV452713.1	1264532	1-scf00011	1
AX-157902786	1	59.223	KV452713.1	1265408	1-scf00011	1
AX-157902787	1	59.223	KV452713.1	1267280	1-scf00011	1
AX-157902789	1	59.223	KV452713.1	1268293	1-scf00011	1
AX-157902790	1	59.223	KV452713.1	1268978	1-scf00011	1
AX-157902791	1	59.223	KV452713.1	1269371	1-scf00011	1
AX-157902792	1	59.223	KV452713.1	1269769	1-scf00011	1
AX-157902794	1	59.223	KV452713.1	1271554	1-scf00011	1
AX-157902795	1	59.223	KV452713.1	1273092	1-scf00011	1
AX-157902796	1	59.223	KV452713.1	1273625	1-scf00011	1
AX-157902797	1	59.223	KV452713.1	1275217	1-scf00011	1
AX-157883302	1	59.223	KV452713.1	1276249	1-scf00011	1
AX-157902801	1	59.223	KV452713.1	1282424	1-scf00011	1
AX-157902803	1	59.223	KV452713.1	1283810	1-scf00011	1
AX-157902805	1	59.223	KV452713.1	1290636	1-scf00011	1
AX-157902806	1	59.223	KV452713.1	1292175	1-scf00011	1
AX-157902809	1	59.223	KV452713.1	1297268	1-scf00011	1
AX-157902815	1	59.223	KV452713.1	1304805	1-scf00011	1
AX-157902818	1	59.223	KV452713.1	1309018	1-scf00011	1
AX-157902822	1	59.223	KV452713.1	1315143	1-scf00011	1
AX-157902823	1	59.223	KV452713.1	1316228	1-scf00011	1
AX-157902824	1	59.223	KV452713.1	1316715	1-scf00011	1
AX-157902826	1	59.223	KV452713.1	1318254	1-scf00011	1
AX-157902827	1	59.223	KV452713.1	1318655	1-scf00011	1
AX-157811695	1	59.223	KV452713.1	1321817	1-scf00011	1
AX-157902828	1	59.223	KV452713.1	1321946	1-scf00011	1
AX-157902831	1	59.223	KV452713.1	1326216	1-scf00011	1
AX-157740696	1	59.223	KV452713.1	1327172	1-scf00011	1
AX-157811697	1	59.223	KV452713.1	1329450	1-scf00011	1
AX-157902837	1	59.223	KV452713.1	1334926	1-scf00011	1
AX-157902838	1	59.223	KV452713.1	1336427	1-scf00011	1
AX-157811701	1	59.223	KV452713.1	1342145	1-scf00011	1
AX-157902840	1	59.223	KV452713.1	1342944	1-scf00011	1
AX-158404653	1	59.223	KV452713.1	1351700	1-scf00011	1
AX-157902968	1	59.223	KV452713.1	1524862	1-scf00011	1
AX-157902986	1	59.223	KV452713.1	1540405	1-scf00011	1
AX-157903206	1	59.223	KV452713.1	1811920	1-scf00011	1
AX-157811738	1	59.223	KV452713.1	1892573	1-scf00011	1
AX-157903267	1	59.223	KV452713.1	1909840	1-scf00011	1
AX-157811755	1	59.223	KV452713.1	2057065	1-scf00011	1
AX-157903528	1	59.223	KV452713.1	2216221	1-scf00011	1
AX-158414674	1	59.223	KV452713.1	2231433	1-scf00011	1
AX-157903747	1	59.223	KV452713.1	2483902	1-scf00011	1
AX-157883312	1	59.223	KV452713.1	2586459	1-scf00011	1
AX-157903869	1	59.223	KV452713.1	2649285	1-scf00011	1
AX-157903889	1	59.223	KV452713.1	2670323	1-scf00011	1
AX-157903902	1	59.223	KV452713.1	2681469	1-scf00011	1

AX-157904051	1	59.223	KV452713.1	2943405	1-scf00011	1
AX-157904104	1	59.223	KV452713.1	3044776	1-scf00011	1
AX-157904147	1	59.223	KV452713.1	3123235	1-scf00011	1
AX-157904166	1	59.223	KV452713.1	3171420	1-scf00011	1
AX-157904168	1	59.223	KV452713.1	3172974	1-scf00011	1
AX-157904174	1	59.223	KV452713.1	3183268	1-scf00011	1
AX-157904245	1	59.223	KV452713.1	3378892	1-scf00011	1
AX-157904315	1	59.223	KV452713.1	3551145	1-scf00011	1
AX-157904318	1	59.223	KV452713.1	3552803	1-scf00011	1
AX-157904319	1	59.223	KV452713.1	3553336	1-scf00011	1
AX-157714746	1	59.223	KV452713.1	3852292	1-scf00011	1
AX-157904513	1	59.223	KV452713.1	3871112	1-scf00011	1
AX-157904528	1	59.223	KV452713.1	3893961	1-scf00011	1
AX-157904564	1	59.223	KV452713.1	3957591	1-scf00011	1
AX-157904632	1	59.223	KV452713.1	4050943	1-scf00011	1
AX-157904660	1	59.223	KV452713.1	4092086	1-scf00011	1
AX-157904871	1	59.223	KV452713.1	4358398	1-scf00011	1
AX-158376619	1	59.223	KV452710.1	34009	1-scf00588	1
AX-158376629	1	59.223	KV452710.1	157163	1-scf00588	1
AX-158376630	1	59.223	KV452710.1	160804	1-scf00588	1
AX-158376632	1	59.223	KV452710.1	168641	1-scf00588	1
AX-158376634	1	59.223	KV452710.1	172850	1-scf00588	1
AX-158376635	1	59.223	KV452710.1	173089	1-scf00588	1
AX-158379061	1	59.223	KV452710.1	239382	1-scf00588	1
AX-158376655	1	59.223	KV452710.1	267362	1-scf00588	1
AX-157726207	1	59.223	KV452710.1	277597	1-scf00588	1
AX-157704685	1	59.223	3ML01028149	46	scf03754_1	UN
AX-157704684	1	59.223	3ML01028149	183	scf03754_1	UN
AX-157902844	1	59.268	KV452713.1	1352259	1-scf00011	1
AX-157902845	1	59.32	KV452713.1	1353496	1-scf00011	1
AX-157902846	1	59.32	KV452713.1	1354559	1-scf00011	1
AX-157898819	1	59.332	KV452709.1	4306365	1-scf00009	1
AX-157899630	1	59.332	KV452709.1	5409145	1-scf00009	1
AX-157899835	1	59.332	KV452709.1	5675657	1-scf00009	1
AX-157902859	1	59.332	KV452713.1	1376853	1-scf00011	1
AX-157902861	1	59.332	KV452713.1	1380074	1-scf00011	1
AX-157902865	1	59.332	KV452713.1	1386620	1-scf00011	1
AX-157902866	1	59.332	KV452713.1	1391119	1-scf00011	1
AX-157752268	1	59.332	KV452713.1	1391956	1-scf00011	1
AX-157902870	1	59.332	KV452713.1	1398730	1-scf00011	1
AX-157902871	1	59.332	KV452713.1	1399199	1-scf00011	1
AX-157902872	1	59.332	KV452713.1	1399518	1-scf00011	1
AX-157902879	1	59.332	KV452713.1	1408602	1-scf00011	1
AX-157902880	1	59.332	KV452713.1	1408751	1-scf00011	1
AX-157902897	1	59.332	KV452713.1	1432120	1-scf00011	1
AX-158389387	1	59.332	KV452713.1	1432193	1-scf00011	1
AX-157903680	1	59.332	KV452713.1	2387394	1-scf00011	1
AX-157811771	1	59.332	KV452713.1	2427957	1-scf00011	1
AX-157903751	1	59.332	KV452713.1	2489438	1-scf00011	1
AX-157904209	1	59.332	KV452713.1	3277322	1-scf00011	1
AX-157902848	1	59.397	KV452713.1	1356393	1-scf00011	1

AX-157902916	1	59.397	KV452713.1	1462092	1-scf00011	1
AX-157903456	1	59.397	KV452713.1	2151412	1-scf00011	1
AX-157902927	1	59.448	KV452713.1	1479312	1-scf00011	1
AX-157698129	1	59.473	FI897710.1	40	HORI212 Ictalu	UN
AX-157902720	1	59.551	KV452713.1	1167494	1-scf00011	1
AX-157902932	1	59.551	KV452713.1	1485548	1-scf00011	1
AX-157902933	1	59.551	KV452713.1	1486349	1-scf00011	1
AX-157902934	1	59.551	KV452713.1	1487209	1-scf00011	1
AX-157902935	1	59.551	KV452713.1	1487551	1-scf00011	1
AX-157902936	1	59.551	KV452713.1	1488016	1-scf00011	1
AX-157902937	1	59.551	KV452713.1	1488426	1-scf00011	1
AX-157902938	1	59.551	KV452713.1	1490351	1-scf00011	1
AX-157902939	1	59.551	KV452713.1	1491073	1-scf00011	1
AX-157902941	1	59.551	KV452713.1	1492705	1-scf00011	1
AX-157902942	1	59.551	KV452713.1	1493336	1-scf00011	1
AX-157902948	1	59.551	KV452713.1	1500016	1-scf00011	1
AX-157902951	1	59.551	KV452713.1	1504799	1-scf00011	1
AX-157902976	1	59.551	KV452713.1	1532536	1-scf00011	1
AX-157902978	1	59.551	KV452713.1	1533645	1-scf00011	1
AX-157902979	1	59.551	KV452713.1	1535460	1-scf00011	1
AX-157902985	1	59.551	KV452713.1	1539970	1-scf00011	1
AX-157760662	1	59.551	KV452713.1	1558383	1-scf00011	1
AX-157753196	1	59.551	KV452713.1	2197562	1-scf00011	1
AX-157904902	1	59.551	KV452713.1	4397943	1-scf00011	1
AX-157903015	1	59.586	KV452713.1	1582492	1-scf00011	1
AX-157811714	1	59.619	KV452713.1	1583394	1-scf00011	1
AX-157895671	1	59.66	KV452708.1	2822713	1-scf00008	1
AX-157896487	1	59.66	KV452709.1	506900	1-scf00009	1
AX-157896896	1	59.66	KV452709.1	1064314	1-scf00009	1
AX-157897063	1	59.66	KV452709.1	1353935	1-scf00009	1
AX-157897272	1	59.66	KV452709.1	1614551	1-scf00009	1
AX-157897932	1	59.66	KV452709.1	2656081	1-scf00009	1
AX-157898316	1	59.66	KV452709.1	3424598	1-scf00009	1
AX-157898320	1	59.66	KV452709.1	3428893	1-scf00009	1
AX-157898427	1	59.66	KV452709.1	3628959	1-scf00009	1
AX-157898496	1	59.66	KV452709.1	3843688	1-scf00009	1
AX-157899016	1	59.66	KV452709.1	4557543	1-scf00009	1
AX-157899017	1	59.66	KV452709.1	4557974	1-scf00009	1
AX-157811220	1	59.66	KV452709.1	4587146	1-scf00009	1
AX-157899108	1	59.66	KV452709.1	4682930	1-scf00009	1
AX-157899112	1	59.66	KV452709.1	4688438	1-scf00009	1
AX-158404474	1	59.66	KV452709.1	4857574	1-scf00009	1
AX-157899225	1	59.66	KV452709.1	4884071	1-scf00009	1
AX-157899347	1	59.66	KV452709.1	5063221	1-scf00009	1
AX-157899655	1	59.66	KV452709.1	5436301	1-scf00009	1
AX-157745901	1	59.66	KV452709.1	5593791	1-scf00009	1
AX-157899827	1	59.66	KV452709.1	5669851	1-scf00009	1
AX-157899869	1	59.66	KV452709.1	5712350	1-scf00009	1
AX-157899899	1	59.66	KV452709.1	5741486	1-scf00009	1
AX-157725528	1	59.66	KV452709.1	5772968	1-scf00009	1
AX-157899944	1	59.66	KV452709.1	5802606	1-scf00009	1

AX-157900048	1	59.66	KV452709.1	5981378	1-scf00009	1
AX-157900080	1	59.66	KV452709.1	6032610	1-scf00009	1
AX-157900320	1	59.66	KV452709.1	6499816	1-scf00009	1
AX-157900340	1	59.66	KV452709.1	6542099	1-scf00009	1
AX-157900372	1	59.66	KV452709.1	6610403	1-scf00009	1
AX-157900380	1	59.66	KV452709.1	6680393	1-scf00009	1
AX-157900387	1	59.66	KV452709.1	6723318	1-scf00009	1
AX-157900396	1	59.66	KV452709.1	6798088	1-scf00009	1
AX-157900398	1	59.66	KV452709.1	6843455	1-scf00009	1
AX-157900399	1	59.66	KV452709.1	6879953	1-scf00009	1
AX-157811408	1	59.66	KV452709.1	7018780	1-scf00009	1
AX-157900413	1	59.66	KV452709.1	7024067	1-scf00009	1
AX-157900414	1	59.66	KV452709.1	7033489	1-scf00009	1
AX-157900415	1	59.66	KV452709.1	7035831	1-scf00009	1
AX-157900416	1	59.66	KV452709.1	7037613	1-scf00009	1
AX-157900417	1	59.66	KV452709.1	7040700	1-scf00009	1
AX-157900419	1	59.66	KV452709.1	7042524	1-scf00009	1
AX-157900422	1	59.66	KV452709.1	7046000	1-scf00009	1
AX-157900424	1	59.66	KV452709.1	7049969	1-scf00009	1
AX-157900425	1	59.66	KV452709.1	7052849	1-scf00009	1
AX-157900426	1	59.66	KV452709.1	7053633	1-scf00009	1
AX-157708064	1	59.66	KV452709.1	7054200	1-scf00009	1
AX-157900427	1	59.66	KV452709.1	7055233	1-scf00009	1
AX-157900428	1	59.66	KV452709.1	7056444	1-scf00009	1
AX-157900429	1	59.66	KV452709.1	7058194	1-scf00009	1
AX-157900431	1	59.66	KV452709.1	7060581	1-scf00009	1
AX-157900433	1	59.66	KV452709.1	7064400	1-scf00009	1
AX-157900435	1	59.66	KV452709.1	7075135	1-scf00009	1
AX-157900437	1	59.66	KV452709.1	7078404	1-scf00009	1
AX-157900439	1	59.66	KV452709.1	7079657	1-scf00009	1
AX-157900442	1	59.66	KV452709.1	7097350	1-scf00009	1
AX-157900448	1	59.66	KV452709.1	7129776	1-scf00009	1
AX-157900451	1	59.66	KV452709.1	7140569	1-scf00009	1
AX-157900453	1	59.66	KV452709.1	7146766	1-scf00009	1
AX-157900454	1	59.66	KV452709.1	7147315	1-scf00009	1
AX-157900455	1	59.66	KV452709.1	7149115	1-scf00009	1
AX-157755085	1	59.66	KV452709.1	7163321	1-scf00009	1
AX-157758519	1	59.66	KV452709.1	7170469	1-scf00009	1
AX-157900465	1	59.66	KV452709.1	7173924	1-scf00009	1
AX-157900467	1	59.66	KV452709.1	7178903	1-scf00009	1
AX-157900468	1	59.66	KV452709.1	7182071	1-scf00009	1
AX-157900469	1	59.66	KV452709.1	7184496	1-scf00009	1
AX-157900470	1	59.66	KV452709.1	7185206	1-scf00009	1
AX-157900471	1	59.66	KV452709.1	7189933	1-scf00009	1
AX-157900472	1	59.66	KV452709.1	7190236	1-scf00009	1
AX-157900473	1	59.66	KV452709.1	7191328	1-scf00009	1
AX-157900474	1	59.66	KV452709.1	7191750	1-scf00009	1
AX-157900476	1	59.66	KV452709.1	7192950	1-scf00009	1
AX-157900478	1	59.66	KV452709.1	7194803	1-scf00009	1
AX-157900486	1	59.66	KV452709.1	7199191	1-scf00009	1
AX-157900503	1	59.66	KV452709.1	7212384	1-scf00009	1

AX-158404525	1	59.66	KV452709.1	7213185	1-scf00009	1
AX-157900504	1	59.66	KV452709.1	7213253	1-scf00009	1
AX-157900505	1	59.66	KV452709.1	7214051	1-scf00009	1
AX-157900512	1	59.66	KV452709.1	7220388	1-scf00009	1
AX-157900513	1	59.66	KV452709.1	7220839	1-scf00009	1
AX-157900514	1	59.66	KV452709.1	7225554	1-scf00009	1
AX-157811415	1	59.66	KV452709.1	7227130	1-scf00009	1
AX-157900520	1	59.66	KV452709.1	7272899	1-scf00009	1
AX-157900540	1	59.66	KV452709.1	7365874	1-scf00009	1
AX-157900748	1	59.66	KV452711.1	33020	1-scf00010	1
AX-157811424	1	59.66	KV452711.1	38901	1-scf00010	1
AX-157900873	1	59.66	KV452711.1	603259	1-scf00010	1
AX-157811443	1	59.66	KV452711.1	877941	1-scf00010	1
AX-157900955	1	59.66	KV452711.1	924100	1-scf00010	1
AX-157901038	1	59.66	KV452711.1	1279836	1-scf00010	1
AX-157901049	1	59.66	KV452711.1	1316489	1-scf00010	1
AX-157901098	1	59.66	KV452711.1	1458938	1-scf00010	1
AX-157901108	1	59.66	KV452711.1	1491124	1-scf00010	1
AX-157901220	1	59.66	KV452711.1	1786946	1-scf00010	1
AX-157901225	1	59.66	KV452711.1	1791549	1-scf00010	1
AX-157901231	1	59.66	KV452711.1	1794667	1-scf00010	1
AX-157901233	1	59.66	KV452711.1	1797245	1-scf00010	1
AX-157901242	1	59.66	KV452711.1	1822446	1-scf00010	1
AX-157901382	1	59.66	KV452711.1	2190313	1-scf00010	1
AX-157901499	1	59.66	KV452711.1	2575821	1-scf00010	1
AX-157901608	1	59.66	KV452711.1	2798256	1-scf00010	1
AX-157901651	1	59.66	KV452711.1	2898900	1-scf00010	1
AX-157702280	1	59.66	KV452711.1	2905495	1-scf00010	1
AX-157763136	1	59.66	KV452711.1	3172725	1-scf00010	1
AX-157901807	1	59.66	KV452711.1	3327008	1-scf00010	1
AX-158404616	1	59.66	KV452711.1	3507773	1-scf00010	1
AX-157901873	1	59.66	KV452711.1	3616515	1-scf00010	1
AX-157901934	1	59.66	KV452711.1	3668250	1-scf00010	1
AX-157902278	1	59.66	KV452713.1	9545	1-scf00011	1
AX-157902326	1	59.66	KV452713.1	294782	1-scf00011	1
AX-157902361	1	59.66	KV452713.1	450338	1-scf00011	1
AX-157902386	1	59.66	KV452713.1	536959	1-scf00011	1
AX-157811669	1	59.66	KV452713.1	757276	1-scf00011	1
AX-157902458	1	59.66	KV452713.1	774641	1-scf00011	1
AX-157902460	1	59.66	KV452713.1	775108	1-scf00011	1
AX-157902462	1	59.66	KV452713.1	775597	1-scf00011	1
AX-157902468	1	59.66	KV452713.1	788302	1-scf00011	1
AX-157902470	1	59.66	KV452713.1	788771	1-scf00011	1
AX-157902490	1	59.66	KV452713.1	812043	1-scf00011	1
AX-157902510	1	59.66	KV452713.1	859050	1-scf00011	1
AX-157902519	1	59.66	KV452713.1	875290	1-scf00011	1
AX-157902612	1	59.66	KV452713.1	1028322	1-scf00011	1
AX-157902613	1	59.66	KV452713.1	1028798	1-scf00011	1
AX-157902614	1	59.66	KV452713.1	1029417	1-scf00011	1
AX-157902621	1	59.66	KV452713.1	1037698	1-scf00011	1
AX-157902627	1	59.66	KV452713.1	1043302	1-scf00011	1

AX-157902646	1	59.66	KV452713.1	1065487	1-scf00011	1
AX-157902647	1	59.66	KV452713.1	1067181	1-scf00011	1
AX-157902674	1	59.66	KV452713.1	1118742	1-scf00011	1
AX-157902677	1	59.66	KV452713.1	1121563	1-scf00011	1
AX-157902688	1	59.66	KV452713.1	1134894	1-scf00011	1
AX-157902728	1	59.66	KV452713.1	1177641	1-scf00011	1
AX-157902749	1	59.66	KV452713.1	1205172	1-scf00011	1
AX-157740092	1	59.66	KV452713.1	1208726	1-scf00011	1
AX-157902774	1	59.66	KV452713.1	1238193	1-scf00011	1
AX-157811699	1	59.66	KV452713.1	1339296	1-scf00011	1
AX-157811700	1	59.66	KV452713.1	1340063	1-scf00011	1
AX-158404652	1	59.66	KV452713.1	1345228	1-scf00011	1
AX-157902843	1	59.66	KV452713.1	1348139	1-scf00011	1
AX-157902849	1	59.66	KV452713.1	1357140	1-scf00011	1
AX-157902850	1	59.66	KV452713.1	1357860	1-scf00011	1
AX-157902852	1	59.66	KV452713.1	1358548	1-scf00011	1
AX-157902855	1	59.66	KV452713.1	1361550	1-scf00011	1
AX-157694874	1	59.66	KV452713.1	1364237	1-scf00011	1
AX-157811703	1	59.66	KV452713.1	1365709	1-scf00011	1
AX-157902856	1	59.66	KV452713.1	1366826	1-scf00011	1
AX-157902858	1	59.66	KV452713.1	1374720	1-scf00011	1
AX-157902860	1	59.66	KV452713.1	1378815	1-scf00011	1
AX-157902862	1	59.66	KV452713.1	1381636	1-scf00011	1
AX-157902867	1	59.66	KV452713.1	1395462	1-scf00011	1
AX-157902868	1	59.66	KV452713.1	1397049	1-scf00011	1
AX-157902869	1	59.66	KV452713.1	1398113	1-scf00011	1
AX-157902873	1	59.66	KV452713.1	1400679	1-scf00011	1
AX-157902874	1	59.66	KV452713.1	1401221	1-scf00011	1
AX-157902875	1	59.66	KV452713.1	1403168	1-scf00011	1
AX-157748454	1	59.66	KV452713.1	1403376	1-scf00011	1
AX-157902876	1	59.66	KV452713.1	1404960	1-scf00011	1
AX-157902878	1	59.66	KV452713.1	1407156	1-scf00011	1
AX-157902882	1	59.66	KV452713.1	1413766	1-scf00011	1
AX-157902883	1	59.66	KV452713.1	1414704	1-scf00011	1
AX-157902888	1	59.66	KV452713.1	1421802	1-scf00011	1
AX-157902895	1	59.66	KV452713.1	1428752	1-scf00011	1
AX-157902901	1	59.66	KV452713.1	1436690	1-scf00011	1
AX-157902903	1	59.66	KV452713.1	1439600	1-scf00011	1
AX-157902909	1	59.66	KV452713.1	1449099	1-scf00011	1
AX-157902912	1	59.66	KV452713.1	1452887	1-scf00011	1
AX-158389388	1	59.66	KV452713.1	1457507	1-scf00011	1
AX-157902917	1	59.66	KV452713.1	1462776	1-scf00011	1
AX-157902920	1	59.66	KV452713.1	1466058	1-scf00011	1
AX-157902923	1	59.66	KV452713.1	1472367	1-scf00011	1
AX-157902924	1	59.66	KV452713.1	1476382	1-scf00011	1
AX-157902940	1	59.66	KV452713.1	1491552	1-scf00011	1
AX-157902943	1	59.66	KV452713.1	1493875	1-scf00011	1
AX-157902947	1	59.66	KV452713.1	1499484	1-scf00011	1
AX-157883310	1	59.66	KV452713.1	1501291	1-scf00011	1
AX-157902949	1	59.66	KV452713.1	1501641	1-scf00011	1
AX-157902950	1	59.66	KV452713.1	1503165	1-scf00011	1

AX-157902953	1	59.66	KV452713.1	1506048	1-scf00011	1
AX-157902954	1	59.66	KV452713.1	1506835	1-scf00011	1
AX-157902955	1	59.66	KV452713.1	1507209	1-scf00011	1
AX-157902956	1	59.66	KV452713.1	1508851	1-scf00011	1
AX-157902957	1	59.66	KV452713.1	1509172	1-scf00011	1
AX-157902965	1	59.66	KV452713.1	1517933	1-scf00011	1
AX-157772188	1	59.66	KV452713.1	1520739	1-scf00011	1
AX-157902969	1	59.66	KV452713.1	1525459	1-scf00011	1
AX-157756610	1	59.66	KV452713.1	1528928	1-scf00011	1
AX-157902973	1	59.66	KV452713.1	1529644	1-scf00011	1
AX-157902974	1	59.66	KV452713.1	1530549	1-scf00011	1
AX-157902975	1	59.66	KV452713.1	1531754	1-scf00011	1
AX-157902981	1	59.66	KV452713.1	1536308	1-scf00011	1
AX-157811709	1	59.66	KV452713.1	1539056	1-scf00011	1
AX-157902988	1	59.66	KV452713.1	1541266	1-scf00011	1
AX-157902990	1	59.66	KV452713.1	1543308	1-scf00011	1
AX-157902992	1	59.66	KV452713.1	1547698	1-scf00011	1
AX-157902993	1	59.66	KV452713.1	1548216	1-scf00011	1
AX-157902994	1	59.66	KV452713.1	1549100	1-scf00011	1
AX-157811711	1	59.66	KV452713.1	1550337	1-scf00011	1
AX-157902998	1	59.66	KV452713.1	1554701	1-scf00011	1
AX-157903000	1	59.66	KV452713.1	1558140	1-scf00011	1
AX-157903002	1	59.66	KV452713.1	1562602	1-scf00011	1
AX-157903003	1	59.66	KV452713.1	1563266	1-scf00011	1
AX-158389393	1	59.66	KV452713.1	1563943	1-scf00011	1
AX-157772295	1	59.66	KV452713.1	1566344	1-scf00011	1
AX-157716409	1	59.66	KV452713.1	1580398	1-scf00011	1
AX-157868877	1	59.66	KV452713.1	1582249	1-scf00011	1
AX-157903018	1	59.66	KV452713.1	1586245	1-scf00011	1
AX-157717015	1	59.66	KV452713.1	1588827	1-scf00011	1
AX-157903021	1	59.66	KV452713.1	1590750	1-scf00011	1
AX-157903025	1	59.66	KV452713.1	1592647	1-scf00011	1
AX-157757796	1	59.66	KV452713.1	1594980	1-scf00011	1
AX-157903027	1	59.66	KV452713.1	1598300	1-scf00011	1
AX-157903028	1	59.66	KV452713.1	1599145	1-scf00011	1
AX-157903029	1	59.66	KV452713.1	1601132	1-scf00011	1
AX-157903034	1	59.66	KV452713.1	1611344	1-scf00011	1
AX-157903035	1	59.66	KV452713.1	1613707	1-scf00011	1
AX-157811716	1	59.66	KV452713.1	1623108	1-scf00011	1
AX-157811717	1	59.66	KV452713.1	1624835	1-scf00011	1
AX-157811718	1	59.66	KV452713.1	1627745	1-scf00011	1
AX-157903045	1	59.66	KV452713.1	1633178	1-scf00011	1
AX-157903046	1	59.66	KV452713.1	1633751	1-scf00011	1
AX-157903047	1	59.66	KV452713.1	1634108	1-scf00011	1
AX-157903051	1	59.66	KV452713.1	1639682	1-scf00011	1
AX-157903052	1	59.66	KV452713.1	1639943	1-scf00011	1
AX-157903053	1	59.66	KV452713.1	1642311	1-scf00011	1
AX-157903054	1	59.66	KV452713.1	1642540	1-scf00011	1
AX-157903056	1	59.66	KV452713.1	1646611	1-scf00011	1
AX-157903057	1	59.66	KV452713.1	1647118	1-scf00011	1
AX-157811720	1	59.66	KV452713.1	1647259	1-scf00011	1

AX-157903059	1	59.66	KV452713.1	1648257	1-scf00011	1
AX-157903063	1	59.66	KV452713.1	1653908	1-scf00011	1
AX-157903067	1	59.66	KV452713.1	1656992	1-scf00011	1
AX-157724979	1	59.66	KV452713.1	1660349	1-scf00011	1
AX-157903070	1	59.66	KV452713.1	1660395	1-scf00011	1
AX-157903071	1	59.66	KV452713.1	1660793	1-scf00011	1
AX-157725788	1	59.66	KV452713.1	1661246	1-scf00011	1
AX-157903081	1	59.66	KV452713.1	1668820	1-scf00011	1
AX-157903082	1	59.66	KV452713.1	1670101	1-scf00011	1
AX-157903083	1	59.66	KV452713.1	1670659	1-scf00011	1
AX-157903087	1	59.66	KV452713.1	1674401	1-scf00011	1
AX-157903090	1	59.66	KV452713.1	1676274	1-scf00011	1
AX-158404657	1	59.66	KV452713.1	1680303	1-scf00011	1
AX-158389394	1	59.66	KV452713.1	1683037	1-scf00011	1
AX-157903097	1	59.66	KV452713.1	1689070	1-scf00011	1
AX-157903098	1	59.66	KV452713.1	1689568	1-scf00011	1
AX-157903100	1	59.66	KV452713.1	1691847	1-scf00011	1
AX-158389396	1	59.66	KV452713.1	1692386	1-scf00011	1
AX-157903101	1	59.66	KV452713.1	1692457	1-scf00011	1
AX-157903102	1	59.66	KV452713.1	1692915	1-scf00011	1
AX-157725763	1	59.66	KV452713.1	1693977	1-scf00011	1
AX-157903103	1	59.66	KV452713.1	1695389	1-scf00011	1
AX-157903113	1	59.66	KV452713.1	1708799	1-scf00011	1
AX-157903117	1	59.66	KV452713.1	1711873	1-scf00011	1
AX-157903119	1	59.66	KV452713.1	1716127	1-scf00011	1
AX-157903121	1	59.66	KV452713.1	1718746	1-scf00011	1
AX-157903124	1	59.66	KV452713.1	1720805	1-scf00011	1
AX-157903125	1	59.66	KV452713.1	1722280	1-scf00011	1
AX-157903126	1	59.66	KV452713.1	1722955	1-scf00011	1
AX-157903127	1	59.66	KV452713.1	1723910	1-scf00011	1
AX-157903128	1	59.66	KV452713.1	1724383	1-scf00011	1
AX-157903130	1	59.66	KV452713.1	1725140	1-scf00011	1
AX-158389399	1	59.66	KV452713.1	1728766	1-scf00011	1
AX-157903134	1	59.66	KV452713.1	1728823	1-scf00011	1
AX-157903135	1	59.66	KV452713.1	1729074	1-scf00011	1
AX-157903137	1	59.66	KV452713.1	1730239	1-scf00011	1
AX-158389400	1	59.66	KV452713.1	1730873	1-scf00011	1
AX-158389401	1	59.66	KV452713.1	1737042	1-scf00011	1
AX-157903142	1	59.66	KV452713.1	1741243	1-scf00011	1
AX-157903143	1	59.66	KV452713.1	1741705	1-scf00011	1
AX-157903144	1	59.66	KV452713.1	1741910	1-scf00011	1
AX-157868882	1	59.66	KV452713.1	1744628	1-scf00011	1
AX-157903148	1	59.66	KV452713.1	1745274	1-scf00011	1
AX-157903150	1	59.66	KV452713.1	1749577	1-scf00011	1
AX-157903151	1	59.66	KV452713.1	1750574	1-scf00011	1
AX-157903153	1	59.66	KV452713.1	1751744	1-scf00011	1
AX-157903154	1	59.66	KV452713.1	1756403	1-scf00011	1
AX-157903156	1	59.66	KV452713.1	1758511	1-scf00011	1
AX-157903159	1	59.66	KV452713.1	1761314	1-scf00011	1
AX-157903160	1	59.66	KV452713.1	1762135	1-scf00011	1
AX-157903163	1	59.66	KV452713.1	1765009	1-scf00011	1

AX-157903168	1	59.66	KV452713.1	1773288	1-scf00011	1
AX-157903173	1	59.66	KV452713.1	1775419	1-scf00011	1
AX-157903176	1	59.66	KV452713.1	1777828	1-scf00011	1
AX-157903177	1	59.66	KV452713.1	1778280	1-scf00011	1
AX-157903185	1	59.66	KV452713.1	1784949	1-scf00011	1
AX-157714103	1	59.66	KV452713.1	1785994	1-scf00011	1
AX-157868884	1	59.66	KV452713.1	1786344	1-scf00011	1
AX-157811723	1	59.66	KV452713.1	1786759	1-scf00011	1
AX-157903186	1	59.66	KV452713.1	1789875	1-scf00011	1
AX-157811725	1	59.66	KV452713.1	1794244	1-scf00011	1
AX-157903189	1	59.66	KV452713.1	1796353	1-scf00011	1
AX-157903190	1	59.66	KV452713.1	1797150	1-scf00011	1
AX-157903195	1	59.66	KV452713.1	1805304	1-scf00011	1
AX-157903196	1	59.66	KV452713.1	1805952	1-scf00011	1
AX-157903197	1	59.66	KV452713.1	1806175	1-scf00011	1
AX-157903198	1	59.66	KV452713.1	1806753	1-scf00011	1
AX-157903201	1	59.66	KV452713.1	1809068	1-scf00011	1
AX-157903207	1	59.66	KV452713.1	1812183	1-scf00011	1
AX-157868886	1	59.66	KV452713.1	1814151	1-scf00011	1
AX-157903214	1	59.66	KV452713.1	1819610	1-scf00011	1
AX-157903216	1	59.66	KV452713.1	1820895	1-scf00011	1
AX-157903217	1	59.66	KV452713.1	1822724	1-scf00011	1
AX-157903219	1	59.66	KV452713.1	1824754	1-scf00011	1
AX-157903220	1	59.66	KV452713.1	1825620	1-scf00011	1
AX-157903224	1	59.66	KV452713.1	1831175	1-scf00011	1
AX-157811726	1	59.66	KV452713.1	1831292	1-scf00011	1
AX-157903226	1	59.66	KV452713.1	1833818	1-scf00011	1
AX-157903227	1	59.66	KV452713.1	1834633	1-scf00011	1
AX-157903228	1	59.66	KV452713.1	1836747	1-scf00011	1
AX-157903231	1	59.66	KV452713.1	1840090	1-scf00011	1
AX-157868888	1	59.66	KV452713.1	1841631	1-scf00011	1
AX-157811729	1	59.66	KV452713.1	1842677	1-scf00011	1
AX-157903233	1	59.66	KV452713.1	1842903	1-scf00011	1
AX-157903237	1	59.66	KV452713.1	1847738	1-scf00011	1
AX-157903238	1	59.66	KV452713.1	1848939	1-scf00011	1
AX-157903240	1	59.66	KV452713.1	1851289	1-scf00011	1
AX-157868889	1	59.66	KV452713.1	1852226	1-scf00011	1
AX-157811732	1	59.66	KV452713.1	1853146	1-scf00011	1
AX-157811734	1	59.66	KV452713.1	1859995	1-scf00011	1
AX-158403997	1	59.66	KV452713.1	1863592	1-scf00011	1
AX-157903244	1	59.66	KV452713.1	1865893	1-scf00011	1
AX-157903245	1	59.66	KV452713.1	1866756	1-scf00011	1
AX-157903249	1	59.66	KV452713.1	1869942	1-scf00011	1
AX-157903250	1	59.66	KV452713.1	1871062	1-scf00011	1
AX-157903251	1	59.66	KV452713.1	1874859	1-scf00011	1
AX-157903252	1	59.66	KV452713.1	1875431	1-scf00011	1
AX-157903254	1	59.66	KV452713.1	1876234	1-scf00011	1
AX-157811735	1	59.66	KV452713.1	1878064	1-scf00011	1
AX-157811736	1	59.66	KV452713.1	1880100	1-scf00011	1
AX-158389407	1	59.66	KV452713.1	1883245	1-scf00011	1
AX-157903260	1	59.66	KV452713.1	1886047	1-scf00011	1

AX-158389408	1	59.66	KV452713.1	1886906	1-scf00011	1
AX-158389409	1	59.66	KV452713.1	1891239	1-scf00011	1
AX-157903264	1	59.66	KV452713.1	1903200	1-scf00011	1
AX-157903268	1	59.66	KV452713.1	1910542	1-scf00011	1
AX-157903270	1	59.66	KV452713.1	1911814	1-scf00011	1
AX-157903271	1	59.66	KV452713.1	1913704	1-scf00011	1
AX-157811741	1	59.66	KV452713.1	1922183	1-scf00011	1
AX-157811742	1	59.66	KV452713.1	1926561	1-scf00011	1
AX-157903276	1	59.66	KV452713.1	1931327	1-scf00011	1
AX-157903278	1	59.66	KV452713.1	1933515	1-scf00011	1
AX-157773598	1	59.66	KV452713.1	1936315	1-scf00011	1
AX-157903279	1	59.66	KV452713.1	1936608	1-scf00011	1
AX-157868892	1	59.66	KV452713.1	1937963	1-scf00011	1
AX-157903282	1	59.66	KV452713.1	1939098	1-scf00011	1
AX-157903285	1	59.66	KV452713.1	1942854	1-scf00011	1
AX-157903287	1	59.66	KV452713.1	1945900	1-scf00011	1
AX-157811747	1	59.66	KV452713.1	1951006	1-scf00011	1
AX-157762597	1	59.66	KV452713.1	1955979	1-scf00011	1
AX-157903293	1	59.66	KV452713.1	1956497	1-scf00011	1
AX-157903294	1	59.66	KV452713.1	1958441	1-scf00011	1
AX-157903300	1	59.66	KV452713.1	1965639	1-scf00011	1
AX-157903301	1	59.66	KV452713.1	1966026	1-scf00011	1
AX-157768328	1	59.66	KV452713.1	1971705	1-scf00011	1
AX-157903305	1	59.66	KV452713.1	1972396	1-scf00011	1
AX-157811750	1	59.66	KV452713.1	1986029	1-scf00011	1
AX-157903316	1	59.66	KV452713.1	1986662	1-scf00011	1
AX-157903317	1	59.66	KV452713.1	1987108	1-scf00011	1
AX-157903320	1	59.66	KV452713.1	1989166	1-scf00011	1
AX-157721429	1	59.66	KV452713.1	1990325	1-scf00011	1
AX-157903321	1	59.66	KV452713.1	1992561	1-scf00011	1
AX-157903322	1	59.66	KV452713.1	1993241	1-scf00011	1
AX-157811752	1	59.66	KV452713.1	1993420	1-scf00011	1
AX-157903330	1	59.66	KV452713.1	2003645	1-scf00011	1
AX-157903333	1	59.66	KV452713.1	2005240	1-scf00011	1
AX-158389411	1	59.66	KV452713.1	2012419	1-scf00011	1
AX-157903335	1	59.66	KV452713.1	2013205	1-scf00011	1
AX-157903338	1	59.66	KV452713.1	2016937	1-scf00011	1
AX-157903341	1	59.66	KV452713.1	2019036	1-scf00011	1
AX-157811754	1	59.66	KV452713.1	2020530	1-scf00011	1
AX-157903343	1	59.66	KV452713.1	2021882	1-scf00011	1
AX-157903344	1	59.66	KV452713.1	2026602	1-scf00011	1
AX-157903356	1	59.66	KV452713.1	2038190	1-scf00011	1
AX-157903359	1	59.66	KV452713.1	2041259	1-scf00011	1
AX-157903366	1	59.66	KV452713.1	2048761	1-scf00011	1
AX-158389413	1	59.66	KV452713.1	2052180	1-scf00011	1
AX-158414672	1	59.66	KV452713.1	2052553	1-scf00011	1
AX-157903368	1	59.66	KV452713.1	2053317	1-scf00011	1
AX-157771948	1	59.66	KV452713.1	2060663	1-scf00011	1
AX-157903375	1	59.66	KV452713.1	2061069	1-scf00011	1
AX-157903376	1	59.66	KV452713.1	2061326	1-scf00011	1
AX-157868895	1	59.66	KV452713.1	2065341	1-scf00011	1

AX-157903382	1	59.66	KV452713.1	2070411	1-scf00011	1
AX-157903384	1	59.66	KV452713.1	2072801	1-scf00011	1
AX-157903385	1	59.66	KV452713.1	2073338	1-scf00011	1
AX-157903387	1	59.66	KV452713.1	2076228	1-scf00011	1
AX-157903388	1	59.66	KV452713.1	2077968	1-scf00011	1
AX-157811756	1	59.66	KV452713.1	2078169	1-scf00011	1
AX-157903389	1	59.66	KV452713.1	2079802	1-scf00011	1
AX-157903390	1	59.66	KV452713.1	2080335	1-scf00011	1
AX-157903392	1	59.66	KV452713.1	2082124	1-scf00011	1
AX-157903393	1	59.66	KV452713.1	2082847	1-scf00011	1
AX-157903394	1	59.66	KV452713.1	2083307	1-scf00011	1
AX-157903396	1	59.66	KV452713.1	2085620	1-scf00011	1
AX-157903397	1	59.66	KV452713.1	2086126	1-scf00011	1
AX-157903401	1	59.66	KV452713.1	2089953	1-scf00011	1
AX-157903402	1	59.66	KV452713.1	2091959	1-scf00011	1
AX-157903403	1	59.66	KV452713.1	2092800	1-scf00011	1
AX-157903407	1	59.66	KV452713.1	2095575	1-scf00011	1
AX-157853608	1	59.66	KV452713.1	2097022	1-scf00011	1
AX-158404662	1	59.66	KV452713.1	2098542	1-scf00011	1
AX-157903412	1	59.66	KV452713.1	2100595	1-scf00011	1
AX-157903414	1	59.66	KV452713.1	2105916	1-scf00011	1
AX-157903416	1	59.66	KV452713.1	2107078	1-scf00011	1
AX-157903417	1	59.66	KV452713.1	2107798	1-scf00011	1
AX-157903419	1	59.66	KV452713.1	2110191	1-scf00011	1
AX-157903421	1	59.66	KV452713.1	2111487	1-scf00011	1
AX-157903423	1	59.66	KV452713.1	2112510	1-scf00011	1
AX-158389415	1	59.66	KV452713.1	2114167	1-scf00011	1
AX-157740846	1	59.66	KV452713.1	2117821	1-scf00011	1
AX-157903429	1	59.66	KV452713.1	2120045	1-scf00011	1
AX-157903430	1	59.66	KV452713.1	2122391	1-scf00011	1
AX-157903431	1	59.66	KV452713.1	2124524	1-scf00011	1
AX-157903432	1	59.66	KV452713.1	2124903	1-scf00011	1
AX-157903435	1	59.66	KV452713.1	2127274	1-scf00011	1
AX-157903436	1	59.66	KV452713.1	2129854	1-scf00011	1
AX-157903438	1	59.66	KV452713.1	2132089	1-scf00011	1
AX-157903445	1	59.66	KV452713.1	2139858	1-scf00011	1
AX-157903447	1	59.66	KV452713.1	2141316	1-scf00011	1
AX-157903452	1	59.66	KV452713.1	2148180	1-scf00011	1
AX-157903453	1	59.66	KV452713.1	2149214	1-scf00011	1
AX-157903454	1	59.66	KV452713.1	2149786	1-scf00011	1
AX-157903457	1	59.66	KV452713.1	2152671	1-scf00011	1
AX-158389416	1	59.66	KV452713.1	2154302	1-scf00011	1
AX-157903460	1	59.66	KV452713.1	2154974	1-scf00011	1
AX-157903462	1	59.66	KV452713.1	2155751	1-scf00011	1
AX-157811760	1	59.66	KV452713.1	2156179	1-scf00011	1
AX-157903465	1	59.66	KV452713.1	2158305	1-scf00011	1
AX-157903467	1	59.66	KV452713.1	2159232	1-scf00011	1
AX-157903472	1	59.66	KV452713.1	2162171	1-scf00011	1
AX-157903473	1	59.66	KV452713.1	2163417	1-scf00011	1
AX-157903475	1	59.66	KV452713.1	2165032	1-scf00011	1
AX-157903476	1	59.66	KV452713.1	2165656	1-scf00011	1

AX-157903479	1	59.66	KV452713.1	2167071	1-scf00011	1
AX-157903480	1	59.66	KV452713.1	2167762	1-scf00011	1
AX-157903481	1	59.66	KV452713.1	2169136	1-scf00011	1
AX-158389418	1	59.66	KV452713.1	2170113	1-scf00011	1
AX-157903482	1	59.66	KV452713.1	2170674	1-scf00011	1
AX-157903483	1	59.66	KV452713.1	2171304	1-scf00011	1
AX-157777335	1	59.66	KV452713.1	2171968	1-scf00011	1
AX-157903484	1	59.66	KV452713.1	2172321	1-scf00011	1
AX-157903485	1	59.66	KV452713.1	2172892	1-scf00011	1
AX-157903486	1	59.66	KV452713.1	2174900	1-scf00011	1
AX-157903487	1	59.66	KV452713.1	2175380	1-scf00011	1
AX-157903488	1	59.66	KV452713.1	2175818	1-scf00011	1
AX-157903489	1	59.66	KV452713.1	2176315	1-scf00011	1
AX-157903490	1	59.66	KV452713.1	2177251	1-scf00011	1
AX-157903491	1	59.66	KV452713.1	2178357	1-scf00011	1
AX-157903493	1	59.66	KV452713.1	2180041	1-scf00011	1
AX-157903494	1	59.66	KV452713.1	2180845	1-scf00011	1
AX-158389421	1	59.66	KV452713.1	2183053	1-scf00011	1
AX-157903497	1	59.66	KV452713.1	2183842	1-scf00011	1
AX-157903498	1	59.66	KV452713.1	2184578	1-scf00011	1
AX-157903503	1	59.66	KV452713.1	2191248	1-scf00011	1
AX-157903504	1	59.66	KV452713.1	2192668	1-scf00011	1
AX-158389423	1	59.66	KV452713.1	2195050	1-scf00011	1
AX-157903506	1	59.66	KV452713.1	2195236	1-scf00011	1
AX-157903509	1	59.66	KV452713.1	2198290	1-scf00011	1
AX-157811761	1	59.66	KV452713.1	2202513	1-scf00011	1
AX-157903513	1	59.66	KV452713.1	2203558	1-scf00011	1
AX-157903514	1	59.66	KV452713.1	2204139	1-scf00011	1
AX-157903518	1	59.66	KV452713.1	2207498	1-scf00011	1
AX-157903520	1	59.66	KV452713.1	2209886	1-scf00011	1
AX-157903521	1	59.66	KV452713.1	2210590	1-scf00011	1
AX-158389424	1	59.66	KV452713.1	2210738	1-scf00011	1
AX-157903524	1	59.66	KV452713.1	2213043	1-scf00011	1
AX-157903529	1	59.66	KV452713.1	2218764	1-scf00011	1
AX-157903530	1	59.66	KV452713.1	2219691	1-scf00011	1
AX-157903532	1	59.66	KV452713.1	2226348	1-scf00011	1
AX-157903534	1	59.66	KV452713.1	2227680	1-scf00011	1
AX-157903536	1	59.66	KV452713.1	2229554	1-scf00011	1
AX-157903538	1	59.66	KV452713.1	2230057	1-scf00011	1
AX-158389427	1	59.66	KV452713.1	2233419	1-scf00011	1
AX-157903543	1	59.66	KV452713.1	2234240	1-scf00011	1
AX-157724015	1	59.66	KV452713.1	2234728	1-scf00011	1
AX-157903545	1	59.66	KV452713.1	2236350	1-scf00011	1
AX-157903546	1	59.66	KV452713.1	2238207	1-scf00011	1
AX-158389428	1	59.66	KV452713.1	2239080	1-scf00011	1
AX-157903547	1	59.66	KV452713.1	2239772	1-scf00011	1
AX-157903548	1	59.66	KV452713.1	2240408	1-scf00011	1
AX-157903549	1	59.66	KV452713.1	2242304	1-scf00011	1
AX-158389429	1	59.66	KV452713.1	2243114	1-scf00011	1
AX-157903554	1	59.66	KV452713.1	2246501	1-scf00011	1
AX-157903555	1	59.66	KV452713.1	2246657	1-scf00011	1

AX-157903556	1	59.66	KV452713.1	2247323	1-scf00011	1
AX-157903557	1	59.66	KV452713.1	2249584	1-scf00011	1
AX-157903558	1	59.66	KV452713.1	2251949	1-scf00011	1
AX-157903559	1	59.66	KV452713.1	2253245	1-scf00011	1
AX-157903561	1	59.66	KV452713.1	2254353	1-scf00011	1
AX-157868897	1	59.66	KV452713.1	2254835	1-scf00011	1
AX-157903563	1	59.66	KV452713.1	2255513	1-scf00011	1
AX-157903565	1	59.66	KV452713.1	2258649	1-scf00011	1
AX-157903567	1	59.66	KV452713.1	2259420	1-scf00011	1
AX-157903572	1	59.66	KV452713.1	2265973	1-scf00011	1
AX-157903575	1	59.66	KV452713.1	2268255	1-scf00011	1
AX-157903576	1	59.66	KV452713.1	2269008	1-scf00011	1
AX-157903577	1	59.66	KV452713.1	2271451	1-scf00011	1
AX-157903579	1	59.66	KV452713.1	2273012	1-scf00011	1
AX-157903580	1	59.66	KV452713.1	2273214	1-scf00011	1
AX-158404666	1	59.66	KV452713.1	2274483	1-scf00011	1
AX-157868901	1	59.66	KV452713.1	2276955	1-scf00011	1
AX-157903586	1	59.66	KV452713.1	2277438	1-scf00011	1
AX-157903589	1	59.66	KV452713.1	2280248	1-scf00011	1
AX-157903590	1	59.66	KV452713.1	2280603	1-scf00011	1
AX-157903594	1	59.66	KV452713.1	2283560	1-scf00011	1
AX-157903595	1	59.66	KV452713.1	2284059	1-scf00011	1
AX-157903596	1	59.66	KV452713.1	2284887	1-scf00011	1
AX-157723234	1	59.66	KV452713.1	2285105	1-scf00011	1
AX-157903598	1	59.66	KV452713.1	2286272	1-scf00011	1
AX-157903601	1	59.66	KV452713.1	2289693	1-scf00011	1
AX-157903603	1	59.66	KV452713.1	2290180	1-scf00011	1
AX-157903604	1	59.66	KV452713.1	2291338	1-scf00011	1
AX-157903605	1	59.66	KV452713.1	2293122	1-scf00011	1
AX-157903606	1	59.66	KV452713.1	2293686	1-scf00011	1
AX-157903607	1	59.66	KV452713.1	2293905	1-scf00011	1
AX-157903608	1	59.66	KV452713.1	2294243	1-scf00011	1
AX-157903609	1	59.66	KV452713.1	2295856	1-scf00011	1
AX-157903610	1	59.66	KV452713.1	2296818	1-scf00011	1
AX-157903611	1	59.66	KV452713.1	2297643	1-scf00011	1
AX-157903612	1	59.66	KV452713.1	2299503	1-scf00011	1
AX-157903615	1	59.66	KV452713.1	2301450	1-scf00011	1
AX-157903617	1	59.66	KV452713.1	2303091	1-scf00011	1
AX-157903618	1	59.66	KV452713.1	2303940	1-scf00011	1
AX-157868904	1	59.66	KV452713.1	2307133	1-scf00011	1
AX-157903621	1	59.66	KV452713.1	2307587	1-scf00011	1
AX-157903622	1	59.66	KV452713.1	2308432	1-scf00011	1
AX-157903623	1	59.66	KV452713.1	2311291	1-scf00011	1
AX-157903624	1	59.66	KV452713.1	2311662	1-scf00011	1
AX-157903625	1	59.66	KV452713.1	2313511	1-scf00011	1
AX-157903628	1	59.66	KV452713.1	2327511	1-scf00011	1
AX-157903630	1	59.66	KV452713.1	2329466	1-scf00011	1
AX-157903631	1	59.66	KV452713.1	2331360	1-scf00011	1
AX-157903635	1	59.66	KV452713.1	2334089	1-scf00011	1
AX-157903637	1	59.66	KV452713.1	2336126	1-scf00011	1
AX-157903638	1	59.66	KV452713.1	2336612	1-scf00011	1

AX-157903639	1	59.66	KV452713.1	2337057	1-scf00011	1
AX-157903640	1	59.66	KV452713.1	2337494	1-scf00011	1
AX-157903641	1	59.66	KV452713.1	2338316	1-scf00011	1
AX-157903642	1	59.66	KV452713.1	2338785	1-scf00011	1
AX-157903644	1	59.66	KV452713.1	2341839	1-scf00011	1
AX-157903645	1	59.66	KV452713.1	2342852	1-scf00011	1
AX-157903646	1	59.66	KV452713.1	2343153	1-scf00011	1
AX-157903647	1	59.66	KV452713.1	2344159	1-scf00011	1
AX-157903649	1	59.66	KV452713.1	2345630	1-scf00011	1
AX-157903652	1	59.66	KV452713.1	2346908	1-scf00011	1
AX-157903653	1	59.66	KV452713.1	2347662	1-scf00011	1
AX-157741872	1	59.66	KV452713.1	2348770	1-scf00011	1
AX-157811762	1	59.66	KV452713.1	2350983	1-scf00011	1
AX-157903656	1	59.66	KV452713.1	2353505	1-scf00011	1
AX-158389431	1	59.66	KV452713.1	2356491	1-scf00011	1
AX-157903659	1	59.66	KV452713.1	2358612	1-scf00011	1
AX-157903660	1	59.66	KV452713.1	2359014	1-scf00011	1
AX-157811763	1	59.66	KV452713.1	2360691	1-scf00011	1
AX-157903663	1	59.66	KV452713.1	2362212	1-scf00011	1
AX-157903664	1	59.66	KV452713.1	2362494	1-scf00011	1
AX-157903665	1	59.66	KV452713.1	2362621	1-scf00011	1
AX-157811765	1	59.66	KV452713.1	2363476	1-scf00011	1
AX-157903667	1	59.66	KV452713.1	2365724	1-scf00011	1
AX-158404668	1	59.66	KV452713.1	2366561	1-scf00011	1
AX-158404669	1	59.66	KV452713.1	2366767	1-scf00011	1
AX-157903668	1	59.66	KV452713.1	2367518	1-scf00011	1
AX-158414676	1	59.66	KV452713.1	2367800	1-scf00011	1
AX-157903672	1	59.66	KV452713.1	2376998	1-scf00011	1
AX-157903673	1	59.66	KV452713.1	2377358	1-scf00011	1
AX-157903675	1	59.66	KV452713.1	2383708	1-scf00011	1
AX-157903677	1	59.66	KV452713.1	2384576	1-scf00011	1
AX-158389433	1	59.66	KV452713.1	2385943	1-scf00011	1
AX-158389434	1	59.66	KV452713.1	2393778	1-scf00011	1
AX-157903694	1	59.66	KV452713.1	2400632	1-scf00011	1
AX-158404672	1	59.66	KV452713.1	2411534	1-scf00011	1
AX-157811767	1	59.66	KV452713.1	2415940	1-scf00011	1
AX-157903705	1	59.66	KV452713.1	2416676	1-scf00011	1
AX-157811768	1	59.66	KV452713.1	2417652	1-scf00011	1
AX-157903707	1	59.66	KV452713.1	2418597	1-scf00011	1
AX-157741659	1	59.66	KV452713.1	2419827	1-scf00011	1
AX-157811769	1	59.66	KV452713.1	2421343	1-scf00011	1
AX-157811770	1	59.66	KV452713.1	2421382	1-scf00011	1
AX-157903708	1	59.66	KV452713.1	2422132	1-scf00011	1
AX-157903709	1	59.66	KV452713.1	2423160	1-scf00011	1
AX-157903711	1	59.66	KV452713.1	2426229	1-scf00011	1
AX-157903712	1	59.66	KV452713.1	2426962	1-scf00011	1
AX-157903713	1	59.66	KV452713.1	2427253	1-scf00011	1
AX-157903714	1	59.66	KV452713.1	2429059	1-scf00011	1
AX-157903715	1	59.66	KV452713.1	2432119	1-scf00011	1
AX-157903717	1	59.66	KV452713.1	2435688	1-scf00011	1
AX-157903719	1	59.66	KV452713.1	2442558	1-scf00011	1

AX-157903722	1	59.66	KV452713.1	2444994	1-scf00011	1
AX-157903725	1	59.66	KV452713.1	2448783	1-scf00011	1
AX-157811773	1	59.66	KV452713.1	2450444	1-scf00011	1
AX-157903727	1	59.66	KV452713.1	2451013	1-scf00011	1
AX-157903729	1	59.66	KV452713.1	2453205	1-scf00011	1
AX-157811775	1	59.66	KV452713.1	2453723	1-scf00011	1
AX-157903736	1	59.66	KV452713.1	2459964	1-scf00011	1
AX-157868909	1	59.66	KV452713.1	2460712	1-scf00011	1
AX-157811776	1	59.66	KV452713.1	2461432	1-scf00011	1
AX-157811777	1	59.66	KV452713.1	2463609	1-scf00011	1
AX-157718006	1	59.66	KV452713.1	2466634	1-scf00011	1
AX-157811781	1	59.66	KV452713.1	2470634	1-scf00011	1
AX-157903739	1	59.66	KV452713.1	2472413	1-scf00011	1
AX-158389435	1	59.66	KV452713.1	2473387	1-scf00011	1
AX-157903740	1	59.66	KV452713.1	2474240	1-scf00011	1
AX-157903742	1	59.66	KV452713.1	2477291	1-scf00011	1
AX-157811782	1	59.66	KV452713.1	2477510	1-scf00011	1
AX-157903743	1	59.66	KV452713.1	2477988	1-scf00011	1
AX-157903745	1	59.66	KV452713.1	2481070	1-scf00011	1
AX-157903749	1	59.66	KV452713.1	2487379	1-scf00011	1
AX-157903753	1	59.66	KV452713.1	2491606	1-scf00011	1
AX-157903758	1	59.66	KV452713.1	2498449	1-scf00011	1
AX-157903759	1	59.66	KV452713.1	2500213	1-scf00011	1
AX-157903760	1	59.66	KV452713.1	2500777	1-scf00011	1
AX-157903762	1	59.66	KV452713.1	2502648	1-scf00011	1
AX-157903763	1	59.66	KV452713.1	2504290	1-scf00011	1
AX-157903764	1	59.66	KV452713.1	2507961	1-scf00011	1
AX-157811785	1	59.66	KV452713.1	2511491	1-scf00011	1
AX-157903766	1	59.66	KV452713.1	2514232	1-scf00011	1
AX-158389436	1	59.66	KV452713.1	2515185	1-scf00011	1
AX-157903767	1	59.66	KV452713.1	2515553	1-scf00011	1
AX-158404000	1	59.66	KV452713.1	2520075	1-scf00011	1
AX-158404001	1	59.66	KV452713.1	2525027	1-scf00011	1
AX-158404674	1	59.66	KV452713.1	2525500	1-scf00011	1
AX-157903771	1	59.66	KV452713.1	2526840	1-scf00011	1
AX-157903773	1	59.66	KV452713.1	2531781	1-scf00011	1
AX-157755387	1	59.66	KV452713.1	2532675	1-scf00011	1
AX-157903776	1	59.66	KV452713.1	2535102	1-scf00011	1
AX-157903779	1	59.66	KV452713.1	2539184	1-scf00011	1
AX-157868911	1	59.66	KV452713.1	2557500	1-scf00011	1
AX-157903787	1	59.66	KV452713.1	2557947	1-scf00011	1
AX-157868912	1	59.66	KV452713.1	2558439	1-scf00011	1
AX-157903788	1	59.66	KV452713.1	2561485	1-scf00011	1
AX-157903789	1	59.66	KV452713.1	2562717	1-scf00011	1
AX-157903790	1	59.66	KV452713.1	2563177	1-scf00011	1
AX-157903794	1	59.66	KV452713.1	2570569	1-scf00011	1
AX-157903795	1	59.66	KV452713.1	2571099	1-scf00011	1
AX-157903796	1	59.66	KV452713.1	2571464	1-scf00011	1
AX-157903797	1	59.66	KV452713.1	2572086	1-scf00011	1
AX-157903801	1	59.66	KV452713.1	2574965	1-scf00011	1
AX-157903803	1	59.66	KV452713.1	2576930	1-scf00011	1

AX-157903804	1	59.66	KV452713.1	2577323	1-scf00011	1
AX-157903805	1	59.66	KV452713.1	2577677	1-scf00011	1
AX-157903808	1	59.66	KV452713.1	2580554	1-scf00011	1
AX-157903810	1	59.66	KV452713.1	2581869	1-scf00011	1
AX-157903811	1	59.66	KV452713.1	2582753	1-scf00011	1
AX-157903812	1	59.66	KV452713.1	2584188	1-scf00011	1
AX-157903813	1	59.66	KV452713.1	2584889	1-scf00011	1
AX-157903815	1	59.66	KV452713.1	2587026	1-scf00011	1
AX-157903816	1	59.66	KV452713.1	2587738	1-scf00011	1
AX-157903817	1	59.66	KV452713.1	2588645	1-scf00011	1
AX-157903819	1	59.66	KV452713.1	2590905	1-scf00011	1
AX-157903820	1	59.66	KV452713.1	2591621	1-scf00011	1
AX-157903821	1	59.66	KV452713.1	2592753	1-scf00011	1
AX-157741457	1	59.66	KV452713.1	2596024	1-scf00011	1
AX-157726538	1	59.66	KV452713.1	2597491	1-scf00011	1
AX-157903826	1	59.66	KV452713.1	2600597	1-scf00011	1
AX-157903827	1	59.66	KV452713.1	2600954	1-scf00011	1
AX-157903829	1	59.66	KV452713.1	2602015	1-scf00011	1
AX-157903835	1	59.66	KV452713.1	2613265	1-scf00011	1
AX-157903837	1	59.66	KV452713.1	2614275	1-scf00011	1
AX-157903838	1	59.66	KV452713.1	2616152	1-scf00011	1
AX-157903839	1	59.66	KV452713.1	2617290	1-scf00011	1
AX-157903842	1	59.66	KV452713.1	2619443	1-scf00011	1
AX-158389442	1	59.66	KV452713.1	2621828	1-scf00011	1
AX-157903844	1	59.66	KV452713.1	2622102	1-scf00011	1
AX-157903845	1	59.66	KV452713.1	2622596	1-scf00011	1
AX-157903848	1	59.66	KV452713.1	2625509	1-scf00011	1
AX-157903849	1	59.66	KV452713.1	2626968	1-scf00011	1
AX-157903850	1	59.66	KV452713.1	2628235	1-scf00011	1
AX-157903856	1	59.66	KV452713.1	2634502	1-scf00011	1
AX-157903858	1	59.66	KV452713.1	2635745	1-scf00011	1
AX-157903859	1	59.66	KV452713.1	2638408	1-scf00011	1
AX-157903860	1	59.66	KV452713.1	2639900	1-scf00011	1
AX-157903864	1	59.66	KV452713.1	2644001	1-scf00011	1
AX-157903867	1	59.66	KV452713.1	2647523	1-scf00011	1
AX-158404680	1	59.66	KV452713.1	2658549	1-scf00011	1
AX-157903875	1	59.66	KV452713.1	2659451	1-scf00011	1
AX-157868913	1	59.66	KV452713.1	2661195	1-scf00011	1
AX-157903884	1	59.66	KV452713.1	2666515	1-scf00011	1
AX-157903885	1	59.66	KV452713.1	2667629	1-scf00011	1
AX-157903887	1	59.66	KV452713.1	2668683	1-scf00011	1
AX-157903890	1	59.66	KV452713.1	2671002	1-scf00011	1
AX-157903891	1	59.66	KV452713.1	2671308	1-scf00011	1
AX-157903892	1	59.66	KV452713.1	2671817	1-scf00011	1
AX-157903893	1	59.66	KV452713.1	2672030	1-scf00011	1
AX-157903894	1	59.66	KV452713.1	2672277	1-scf00011	1
AX-157903895	1	59.66	KV452713.1	2673392	1-scf00011	1
AX-158404681	1	59.66	KV452713.1	2678134	1-scf00011	1
AX-157868915	1	59.66	KV452713.1	2678651	1-scf00011	1
AX-157903899	1	59.66	KV452713.1	2678888	1-scf00011	1
AX-157903900	1	59.66	KV452713.1	2679309	1-scf00011	1

AX-157868916	1	59.66	KV452713.1	2680385	1-scf00011	1
AX-157724983	1	59.66	KV452713.1	2684122	1-scf00011	1
AX-157903906	1	59.66	KV452713.1	2685229	1-scf00011	1
AX-157903910	1	59.66	KV452713.1	2687889	1-scf00011	1
AX-157903912	1	59.66	KV452713.1	2688470	1-scf00011	1
AX-157903928	1	59.66	KV452713.1	2718685	1-scf00011	1
AX-157903929	1	59.66	KV452713.1	2720293	1-scf00011	1
AX-157903931	1	59.66	KV452713.1	2722258	1-scf00011	1
AX-157903934	1	59.66	KV452713.1	2724765	1-scf00011	1
AX-157903936	1	59.66	KV452713.1	2728362	1-scf00011	1
AX-157811790	1	59.66	KV452713.1	2728719	1-scf00011	1
AX-157903938	1	59.66	KV452713.1	2729554	1-scf00011	1
AX-157903939	1	59.66	KV452713.1	2730458	1-scf00011	1
AX-157903948	1	59.66	KV452713.1	2740996	1-scf00011	1
AX-157903954	1	59.66	KV452713.1	2746022	1-scf00011	1
AX-157903957	1	59.66	KV452713.1	2749997	1-scf00011	1
AX-158414681	1	59.66	KV452713.1	2751253	1-scf00011	1
AX-157903959	1	59.66	KV452713.1	2752890	1-scf00011	1
AX-157755517	1	59.66	KV452713.1	2754099	1-scf00011	1
AX-157903961	1	59.66	KV452713.1	2754173	1-scf00011	1
AX-157903962	1	59.66	KV452713.1	2756676	1-scf00011	1
AX-157903965	1	59.66	KV452713.1	2757787	1-scf00011	1
AX-157903969	1	59.66	KV452713.1	2761444	1-scf00011	1
AX-157903977	1	59.66	KV452713.1	2790233	1-scf00011	1
AX-157903978	1	59.66	KV452713.1	2790718	1-scf00011	1
AX-157903980	1	59.66	KV452713.1	2791735	1-scf00011	1
AX-157903982	1	59.66	KV452713.1	2797033	1-scf00011	1
AX-157903983	1	59.66	KV452713.1	2799248	1-scf00011	1
AX-157903987	1	59.66	KV452713.1	2807024	1-scf00011	1
AX-157903988	1	59.66	KV452713.1	2809447	1-scf00011	1
AX-157903991	1	59.66	KV452713.1	2817500	1-scf00011	1
AX-157903992	1	59.66	KV452713.1	2818186	1-scf00011	1
AX-157903998	1	59.66	KV452713.1	2842159	1-scf00011	1
AX-157811797	1	59.66	KV452713.1	2857010	1-scf00011	1
AX-158404690	1	59.66	KV452713.1	2863292	1-scf00011	1
AX-157811798	1	59.66	KV452713.1	2863984	1-scf00011	1
AX-157904011	1	59.66	KV452713.1	2878859	1-scf00011	1
AX-157744957	1	59.66	KV452713.1	2880299	1-scf00011	1
AX-157904012	1	59.66	KV452713.1	2880776	1-scf00011	1
AX-157904013	1	59.66	KV452713.1	2882888	1-scf00011	1
AX-158389446	1	59.66	KV452713.1	2891364	1-scf00011	1
AX-157811799	1	59.66	KV452713.1	2891762	1-scf00011	1
AX-157904015	1	59.66	KV452713.1	2895689	1-scf00011	1
AX-157739952	1	59.66	KV452713.1	2900585	1-scf00011	1
AX-157904016	1	59.66	KV452713.1	2901455	1-scf00011	1
AX-157904017	1	59.66	KV452713.1	2902348	1-scf00011	1
AX-157904018	1	59.66	KV452713.1	2903507	1-scf00011	1
AX-157904020	1	59.66	KV452713.1	2904285	1-scf00011	1
AX-157904022	1	59.66	KV452713.1	2905521	1-scf00011	1
AX-157904023	1	59.66	KV452713.1	2908719	1-scf00011	1
AX-157904024	1	59.66	KV452713.1	2910205	1-scf00011	1

AX-157904025	1	59.66	KV452713.1	2913828	1-scf00011	1
AX-157904030	1	59.66	KV452713.1	2919024	1-scf00011	1
AX-157904032	1	59.66	KV452713.1	2920446	1-scf00011	1
AX-157904033	1	59.66	KV452713.1	2922842	1-scf00011	1
AX-157904034	1	59.66	KV452713.1	2923654	1-scf00011	1
AX-157904035	1	59.66	KV452713.1	2923966	1-scf00011	1
AX-157904036	1	59.66	KV452713.1	2926202	1-scf00011	1
AX-157904050	1	59.66	KV452713.1	2939030	1-scf00011	1
AX-157904053	1	59.66	KV452713.1	2952278	1-scf00011	1
AX-157904055	1	59.66	KV452713.1	2957700	1-scf00011	1
AX-157904068	1	59.66	KV452713.1	3007584	1-scf00011	1
AX-158404694	1	59.66	KV452713.1	3010332	1-scf00011	1
AX-158414682	1	59.66	KV452713.1	3010505	1-scf00011	1
AX-157904069	1	59.66	KV452713.1	3010995	1-scf00011	1
AX-157754843	1	59.66	KV452713.1	3012498	1-scf00011	1
AX-157904072	1	59.66	KV452713.1	3014146	1-scf00011	1
AX-157904073	1	59.66	KV452713.1	3014305	1-scf00011	1
AX-157904074	1	59.66	KV452713.1	3014952	1-scf00011	1
AX-157904075	1	59.66	KV452713.1	3015369	1-scf00011	1
AX-157904077	1	59.66	KV452713.1	3018082	1-scf00011	1
AX-157904078	1	59.66	KV452713.1	3018772	1-scf00011	1
AX-157904080	1	59.66	KV452713.1	3020087	1-scf00011	1
AX-157904088	1	59.66	KV452713.1	3027486	1-scf00011	1
AX-157904089	1	59.66	KV452713.1	3028180	1-scf00011	1
AX-157904091	1	59.66	KV452713.1	3029446	1-scf00011	1
AX-157904092	1	59.66	KV452713.1	3029894	1-scf00011	1
AX-157904095	1	59.66	KV452713.1	3031157	1-scf00011	1
AX-157904097	1	59.66	KV452713.1	3032609	1-scf00011	1
AX-157904098	1	59.66	KV452713.1	3035704	1-scf00011	1
AX-157904100	1	59.66	KV452713.1	3037319	1-scf00011	1
AX-157904102	1	59.66	KV452713.1	3041896	1-scf00011	1
AX-157904105	1	59.66	KV452713.1	3046421	1-scf00011	1
AX-157904106	1	59.66	KV452713.1	3048530	1-scf00011	1
AX-157904107	1	59.66	KV452713.1	3049858	1-scf00011	1
AX-157811807	1	59.66	KV452713.1	3050242	1-scf00011	1
AX-157904108	1	59.66	KV452713.1	3053089	1-scf00011	1
AX-157904109	1	59.66	KV452713.1	3054003	1-scf00011	1
AX-157904111	1	59.66	KV452713.1	3061129	1-scf00011	1
AX-157868921	1	59.66	KV452713.1	3063188	1-scf00011	1
AX-157868924	1	59.66	KV452713.1	3067284	1-scf00011	1
AX-157904114	1	59.66	KV452713.1	3067997	1-scf00011	1
AX-157904117	1	59.66	KV452713.1	3069766	1-scf00011	1
AX-157904118	1	59.66	KV452713.1	3070531	1-scf00011	1
AX-157904119	1	59.66	KV452713.1	3071561	1-scf00011	1
AX-157868926	1	59.66	KV452713.1	3072006	1-scf00011	1
AX-157904122	1	59.66	KV452713.1	3073357	1-scf00011	1
AX-157904123	1	59.66	KV452713.1	3073853	1-scf00011	1
AX-157904124	1	59.66	KV452713.1	3074064	1-scf00011	1
AX-157904126	1	59.66	KV452713.1	3077059	1-scf00011	1
AX-157904127	1	59.66	KV452713.1	3079209	1-scf00011	1
AX-157904129	1	59.66	KV452713.1	3082972	1-scf00011	1

AX-157904130	1	59.66	KV452713.1	3087447	1-scf00011	1
AX-157904131	1	59.66	KV452713.1	3093452	1-scf00011	1
AX-157904133	1	59.66	KV452713.1	3097166	1-scf00011	1
AX-157904134	1	59.66	KV452713.1	3099095	1-scf00011	1
AX-157904135	1	59.66	KV452713.1	3099470	1-scf00011	1
AX-157904140	1	59.66	KV452713.1	3106624	1-scf00011	1
AX-157904144	1	59.66	KV452713.1	3120985	1-scf00011	1
AX-157904146	1	59.66	KV452713.1	3122757	1-scf00011	1
AX-157904148	1	59.66	KV452713.1	3126095	1-scf00011	1
AX-158389454	1	59.66	KV452713.1	3167217	1-scf00011	1
AX-157750372	1	59.66	KV452713.1	3167586	1-scf00011	1
AX-157904176	1	59.66	KV452713.1	3186594	1-scf00011	1
AX-158404701	1	59.66	KV452713.1	3208231	1-scf00011	1
AX-157741849	1	59.66	KV452713.1	3208706	1-scf00011	1
AX-157904183	1	59.66	KV452713.1	3221407	1-scf00011	1
AX-158379169	1	59.66	KV452713.1	3224071	1-scf00011	1
AX-157904189	1	59.66	KV452713.1	3234832	1-scf00011	1
AX-157904190	1	59.66	KV452713.1	3235467	1-scf00011	1
AX-157904199	1	59.66	KV452713.1	3261576	1-scf00011	1
AX-157904202	1	59.66	KV452713.1	3268267	1-scf00011	1
AX-157904206	1	59.66	KV452713.1	3273995	1-scf00011	1
AX-157904211	1	59.66	KV452713.1	3281554	1-scf00011	1
AX-157748973	1	59.66	KV452713.1	3284141	1-scf00011	1
AX-157904214	1	59.66	KV452713.1	3287027	1-scf00011	1
AX-157904215	1	59.66	KV452713.1	3297294	1-scf00011	1
AX-157904219	1	59.66	KV452713.1	3305979	1-scf00011	1
AX-157748299	1	59.66	KV452713.1	3306023	1-scf00011	1
AX-157904220	1	59.66	KV452713.1	3307690	1-scf00011	1
AX-157904221	1	59.66	KV452713.1	3308383	1-scf00011	1
AX-157904222	1	59.66	KV452713.1	3310023	1-scf00011	1
AX-157904223	1	59.66	KV452713.1	3310568	1-scf00011	1
AX-157904224	1	59.66	KV452713.1	3311605	1-scf00011	1
AX-157904225	1	59.66	KV452713.1	3312552	1-scf00011	1
AX-157904226	1	59.66	KV452713.1	3320408	1-scf00011	1
AX-157904230	1	59.66	KV452713.1	3326795	1-scf00011	1
AX-157904233	1	59.66	KV452713.1	3330535	1-scf00011	1
AX-157904234	1	59.66	KV452713.1	3336424	1-scf00011	1
AX-157904236	1	59.66	KV452713.1	3347673	1-scf00011	1
AX-157721885	1	59.66	KV452713.1	3356776	1-scf00011	1
AX-157904243	1	59.66	KV452713.1	3374906	1-scf00011	1
AX-157904259	1	59.66	KV452713.1	3422257	1-scf00011	1
AX-157904276	1	59.66	KV452713.1	3453275	1-scf00011	1
AX-157904277	1	59.66	KV452713.1	3457784	1-scf00011	1
AX-157904279	1	59.66	KV452713.1	3461758	1-scf00011	1
AX-157770780	1	59.66	KV452713.1	3463441	1-scf00011	1
AX-157904283	1	59.66	KV452713.1	3469875	1-scf00011	1
AX-157904285	1	59.66	KV452713.1	3476717	1-scf00011	1
AX-157904288	1	59.66	KV452713.1	3486068	1-scf00011	1
AX-158382045	1	59.66	KV452713.1	3486674	1-scf00011	1
AX-157904292	1	59.66	KV452713.1	3506993	1-scf00011	1
AX-157904294	1	59.66	KV452713.1	3509496	1-scf00011	1

AX-157749445	1	59.66	KV452713.1	3513551	1-scf00011	1
AX-157904297	1	59.66	KV452713.1	3515349	1-scf00011	1
AX-157904303	1	59.66	KV452713.1	3528105	1-scf00011	1
AX-157904307	1	59.66	KV452713.1	3531950	1-scf00011	1
AX-157904316	1	59.66	KV452713.1	3551863	1-scf00011	1
AX-157904320	1	59.66	KV452713.1	3553707	1-scf00011	1
AX-157753349	1	59.66	KV452713.1	3555540	1-scf00011	1
AX-157904322	1	59.66	KV452713.1	3557937	1-scf00011	1
AX-157756461	1	59.66	KV452713.1	3559704	1-scf00011	1
AX-157904325	1	59.66	KV452713.1	3560941	1-scf00011	1
AX-157749630	1	59.66	KV452713.1	3565310	1-scf00011	1
AX-157904330	1	59.66	KV452713.1	3569356	1-scf00011	1
AX-157904332	1	59.66	KV452713.1	3571290	1-scf00011	1
AX-157904334	1	59.66	KV452713.1	3575945	1-scf00011	1
AX-157904335	1	59.66	KV452713.1	3576872	1-scf00011	1
AX-157904336	1	59.66	KV452713.1	3578305	1-scf00011	1
AX-157904340	1	59.66	KV452713.1	3579524	1-scf00011	1
AX-157904342	1	59.66	KV452713.1	3580579	1-scf00011	1
AX-157904343	1	59.66	KV452713.1	3580891	1-scf00011	1
AX-157811816	1	59.66	KV452713.1	3581696	1-scf00011	1
AX-157904352	1	59.66	KV452713.1	3591572	1-scf00011	1
AX-157904353	1	59.66	KV452713.1	3592387	1-scf00011	1
AX-157904355	1	59.66	KV452713.1	3596321	1-scf00011	1
AX-157904356	1	59.66	KV452713.1	3596790	1-scf00011	1
AX-157904359	1	59.66	KV452713.1	3600628	1-scf00011	1
AX-157904367	1	59.66	KV452713.1	3613462	1-scf00011	1
AX-157883319	1	59.66	KV452713.1	3613725	1-scf00011	1
AX-157904369	1	59.66	KV452713.1	3615671	1-scf00011	1
AX-157904374	1	59.66	KV452713.1	3627964	1-scf00011	1
AX-157904375	1	59.66	KV452713.1	3631654	1-scf00011	1
AX-157904381	1	59.66	KV452713.1	3642113	1-scf00011	1
AX-157904382	1	59.66	KV452713.1	3644002	1-scf00011	1
AX-157904383	1	59.66	KV452713.1	3645841	1-scf00011	1
AX-157904384	1	59.66	KV452713.1	3646241	1-scf00011	1
AX-157904400	1	59.66	KV452713.1	3690359	1-scf00011	1
AX-157904409	1	59.66	KV452713.1	3715734	1-scf00011	1
AX-157904422	1	59.66	KV452713.1	3735133	1-scf00011	1
AX-157904426	1	59.66	KV452713.1	3736783	1-scf00011	1
AX-157904434	1	59.66	KV452713.1	3752796	1-scf00011	1
AX-157904440	1	59.66	KV452713.1	3757580	1-scf00011	1
AX-157811819	1	59.66	KV452713.1	3758431	1-scf00011	1
AX-157904456	1	59.66	KV452713.1	3777946	1-scf00011	1
AX-157811820	1	59.66	KV452713.1	3778522	1-scf00011	1
AX-157744673	1	59.66	KV452713.1	3778590	1-scf00011	1
AX-157718626	1	59.66	KV452713.1	3779185	1-scf00011	1
AX-157752085	1	59.66	KV452713.1	3781506	1-scf00011	1
AX-157716151	1	59.66	KV452713.1	3782154	1-scf00011	1
AX-157904461	1	59.66	KV452713.1	3786480	1-scf00011	1
AX-157748870	1	59.66	KV452713.1	3790262	1-scf00011	1
AX-157904473	1	59.66	KV452713.1	3806751	1-scf00011	1
AX-157904490	1	59.66	KV452713.1	3835124	1-scf00011	1

AX-157904491	1	59.66	KV452713.1	3836056	1-scf00011	1
AX-157904496	1	59.66	KV452713.1	3848434	1-scf00011	1
AX-157904497	1	59.66	KV452713.1	3849092	1-scf00011	1
AX-157811826	1	59.66	KV452713.1	3850994	1-scf00011	1
AX-157904498	1	59.66	KV452713.1	3851400	1-scf00011	1
AX-157904508	1	59.66	KV452713.1	3861304	1-scf00011	1
AX-157904511	1	59.66	KV452713.1	3865427	1-scf00011	1
AX-157904512	1	59.66	KV452713.1	3866397	1-scf00011	1
AX-157904517	1	59.66	KV452713.1	3875505	1-scf00011	1
AX-157904518	1	59.66	KV452713.1	3876556	1-scf00011	1
AX-157904519	1	59.66	KV452713.1	3877033	1-scf00011	1
AX-157904521	1	59.66	KV452713.1	3878428	1-scf00011	1
AX-157904527	1	59.66	KV452713.1	3892595	1-scf00011	1
AX-157738847	1	59.66	KV452713.1	3901394	1-scf00011	1
AX-157904533	1	59.66	KV452713.1	3902819	1-scf00011	1
AX-157904534	1	59.66	KV452713.1	3907404	1-scf00011	1
AX-157742608	1	59.66	KV452713.1	3909870	1-scf00011	1
AX-157904536	1	59.66	KV452713.1	3912938	1-scf00011	1
AX-157811829	1	59.66	KV452713.1	3920296	1-scf00011	1
AX-157774890	1	59.66	KV452713.1	3920397	1-scf00011	1
AX-157904547	1	59.66	KV452713.1	3938077	1-scf00011	1
AX-157904549	1	59.66	KV452713.1	3939605	1-scf00011	1
AX-157904551	1	59.66	KV452713.1	3941661	1-scf00011	1
AX-157811833	1	59.66	KV452713.1	3942092	1-scf00011	1
AX-157904554	1	59.66	KV452713.1	3945962	1-scf00011	1
AX-157904555	1	59.66	KV452713.1	3946552	1-scf00011	1
AX-157904559	1	59.66	KV452713.1	3952319	1-scf00011	1
AX-157904561	1	59.66	KV452713.1	3954621	1-scf00011	1
AX-157713253	1	59.66	KV452713.1	3956607	1-scf00011	1
AX-157904563	1	59.66	KV452713.1	3957437	1-scf00011	1
AX-157904565	1	59.66	KV452713.1	3958061	1-scf00011	1
AX-157904566	1	59.66	KV452713.1	3958468	1-scf00011	1
AX-157904567	1	59.66	KV452713.1	3958560	1-scf00011	1
AX-157904568	1	59.66	KV452713.1	3959711	1-scf00011	1
AX-157904570	1	59.66	KV452713.1	3960457	1-scf00011	1
AX-157904573	1	59.66	KV452713.1	3965150	1-scf00011	1
AX-157904583	1	59.66	KV452713.1	3978940	1-scf00011	1
AX-157904585	1	59.66	KV452713.1	3980278	1-scf00011	1
AX-157904588	1	59.66	KV452713.1	3984034	1-scf00011	1
AX-157904599	1	59.66	KV452713.1	4003932	1-scf00011	1
AX-157904611	1	59.66	KV452713.1	4018409	1-scf00011	1
AX-157904624	1	59.66	KV452713.1	4033392	1-scf00011	1
AX-157904631	1	59.66	KV452713.1	4045019	1-scf00011	1
AX-157740616	1	59.66	KV452713.1	4045363	1-scf00011	1
AX-157904645	1	59.66	KV452713.1	4073268	1-scf00011	1
AX-157751529	1	59.66	KV452713.1	4082290	1-scf00011	1
AX-158379171	1	59.66	KV452713.1	4083243	1-scf00011	1
AX-157904673	1	59.66	KV452713.1	4105547	1-scf00011	1
AX-157904675	1	59.66	KV452713.1	4107285	1-scf00011	1
AX-157904684	1	59.66	KV452713.1	4118734	1-scf00011	1
AX-157904692	1	59.66	KV452713.1	4127292	1-scf00011	1

AX-157904698	1	59.66	KV452713.1	4134918	1-scf00011	1
AX-157904733	1	59.66	KV452713.1	4175841	1-scf00011	1
AX-157904750	1	59.66	KV452713.1	4199483	1-scf00011	1
AX-157904816	1	59.66	KV452713.1	4288585	1-scf00011	1
AX-157904828	1	59.66	KV452713.1	4303295	1-scf00011	1
AX-157811847	1	59.66	KV452713.1	4324372	1-scf00011	1
AX-157904867	1	59.66	KV452713.1	4354625	1-scf00011	1
AX-157904884	1	59.66	KV452713.1	4376328	1-scf00011	1
AX-157904887	1	59.66	KV452713.1	4378134	1-scf00011	1
AX-157904912	1	59.66	KV452713.1	4410437	1-scf00011	1
AX-157904950	1	59.66	KV452713.1	4465216	1-scf00011	1
AX-157687384	1	59.66	3ML01024870	3121	scf00867_1	UN
AX-157687391	1	59.66	3ML01024870	12672	scf00867_1	UN
AX-157687392	1	59.66	3ML01024870	14351	scf00867_1	UN
AX-157896887	1	59.769	KV452709.1	1054491	1-scf00009	1
AX-157747008	1	59.769	KV452709.1	1099739	1-scf00009	1
AX-157749391	1	59.769	KV452709.1	1107958	1-scf00009	1
AX-157897072	1	59.769	KV452709.1	1366529	1-scf00009	1
AX-157864384	1	59.769	KV452709.1	1547255	1-scf00009	1
AX-157897353	1	59.769	KV452709.1	1749034	1-scf00009	1
AX-157897458	1	59.769	KV452709.1	1915842	1-scf00009	1
AX-157703113	1	59.769	KV452709.1	2347394	1-scf00009	1
AX-157868572	1	59.769	KV452709.1	2558083	1-scf00009	1
AX-157899886	1	59.769	KV452709.1	5724501	1-scf00009	1
AX-157900207	1	59.769	KV452709.1	6257302	1-scf00009	1
AX-157900221	1	59.769	KV452709.1	6273055	1-scf00009	1
AX-157874384	1	59.769	KV452709.1	7175786	1-scf00009	1
AX-157901141	1	59.769	KV452711.1	1606783	1-scf00010	1
AX-157901229	1	59.769	KV452711.1	1793707	1-scf00010	1
AX-157902320	1	59.769	KV452713.1	230769	1-scf00011	1
AX-157811683	1	59.769	KV452713.1	1016476	1-scf00011	1
AX-157902699	1	59.769	KV452713.1	1144582	1-scf00011	1
AX-158382038	1	59.769	KV452713.1	1386304	1-scf00011	1
AX-157903001	1	59.769	KV452713.1	1560867	1-scf00011	1
AX-157903036	1	59.769	KV452713.1	1614328	1-scf00011	1
AX-157903055	1	59.769	KV452713.1	1643264	1-scf00011	1
AX-157903107	1	59.769	KV452713.1	1701892	1-scf00011	1
AX-157903108	1	59.769	KV452713.1	1702692	1-scf00011	1
AX-157853607	1	59.769	KV452713.1	1833595	1-scf00011	1
AX-157903284	1	59.769	KV452713.1	1942408	1-scf00011	1
AX-157903328	1	59.769	KV452713.1	2001809	1-scf00011	1
AX-157903581	1	59.769	KV452713.1	2274287	1-scf00011	1
AX-157903584	1	59.769	KV452713.1	2276311	1-scf00011	1
AX-157903585	1	59.769	KV452713.1	2276676	1-scf00011	1
AX-157903591	1	59.769	KV452713.1	2280906	1-scf00011	1
AX-157903592	1	59.769	KV452713.1	2281934	1-scf00011	1
AX-157811780	1	59.769	KV452713.1	2467395	1-scf00011	1
AX-157874423	1	59.769	KV452713.1	2652637	1-scf00011	1
AX-157903873	1	59.769	KV452713.1	2653518	1-scf00011	1
AX-157903984	1	59.769	KV452713.1	2799863	1-scf00011	1
AX-157904002	1	59.769	KV452713.1	2850107	1-scf00011	1

AX-157904031	1	59.769	KV452713.1	2920072	1-scf00011	1
AX-157904037	1	59.769	KV452713.1	2926854	1-scf00011	1
AX-157904046	1	59.769	KV452713.1	2935768	1-scf00011	1
AX-157904076	1	59.769	KV452713.1	3015816	1-scf00011	1
AX-157904120	1	59.769	KV452713.1	3072227	1-scf00011	1
AX-157904138	1	59.769	KV452713.1	3104910	1-scf00011	1
AX-157904139	1	59.769	KV452713.1	3106235	1-scf00011	1
AX-157904141	1	59.769	KV452713.1	3107250	1-scf00011	1
AX-157904346	1	59.769	KV452713.1	3582208	1-scf00011	1
AX-157904516	1	59.769	KV452713.1	3875174	1-scf00011	1
AX-157716189	1	59.769	KV452713.1	3889347	1-scf00011	1
AX-157777367	1	59.769	KV452713.1	3922318	1-scf00011	1
AX-157904556	1	59.769	KV452713.1	3947553	1-scf00011	1
AX-157904571	1	59.769	KV452713.1	3963692	1-scf00011	1
AX-157904579	1	59.769	KV452713.1	3971950	1-scf00011	1
AX-157904581	1	59.769	KV452713.1	3973994	1-scf00011	1
AX-157904602	1	59.769	KV452713.1	4007917	1-scf00011	1
AX-157754041	1	59.769	KV452713.1	4015354	1-scf00011	1
AX-157811838	1	59.769	KV452713.1	4020875	1-scf00011	1
AX-157904616	1	59.769	KV452713.1	4024255	1-scf00011	1
AX-157904619	1	59.769	KV452713.1	4029911	1-scf00011	1
AX-157904620	1	59.769	KV452713.1	4030290	1-scf00011	1
AX-157904621	1	59.769	KV452713.1	4031492	1-scf00011	1
AX-157755547	1	59.769	KV452713.1	4034989	1-scf00011	1
AX-157811839	1	59.769	KV452713.1	4035038	1-scf00011	1
AX-157904625	1	59.769	KV452713.1	4035285	1-scf00011	1
AX-157904628	1	59.769	KV452713.1	4039820	1-scf00011	1
AX-157904629	1	59.769	KV452713.1	4041981	1-scf00011	1
AX-157904630	1	59.769	KV452713.1	4044625	1-scf00011	1
AX-157904634	1	59.769	KV452713.1	4051917	1-scf00011	1
AX-157775615	1	59.769	KV452713.1	4055129	1-scf00011	1
AX-157904636	1	59.769	KV452713.1	4055212	1-scf00011	1
AX-157904638	1	59.769	KV452713.1	4056110	1-scf00011	1
AX-157811842	1	59.769	KV452713.1	4058316	1-scf00011	1
AX-157904642	1	59.769	KV452713.1	4070359	1-scf00011	1
AX-157904649	1	59.769	KV452713.1	4075478	1-scf00011	1
AX-157811843	1	59.769	KV452713.1	4076416	1-scf00011	1
AX-157756925	1	59.769	KV452713.1	4077450	1-scf00011	1
AX-157904652	1	59.769	KV452713.1	4078108	1-scf00011	1
AX-157904656	1	59.769	KV452713.1	4086010	1-scf00011	1
AX-157904658	1	59.769	KV452713.1	4088522	1-scf00011	1
AX-157904664	1	59.769	KV452713.1	4097096	1-scf00011	1
AX-157904666	1	59.769	KV452713.1	4100099	1-scf00011	1
AX-157904669	1	59.769	KV452713.1	4102185	1-scf00011	1
AX-157904676	1	59.769	KV452713.1	4108566	1-scf00011	1
AX-157904677	1	59.769	KV452713.1	4110198	1-scf00011	1
AX-157904678	1	59.769	KV452713.1	4111093	1-scf00011	1
AX-157904680	1	59.769	KV452713.1	4113179	1-scf00011	1
AX-157904682	1	59.769	KV452713.1	4116010	1-scf00011	1
AX-157904683	1	59.769	KV452713.1	4116472	1-scf00011	1
AX-157749619	1	59.769	KV452713.1	4122156	1-scf00011	1

AX-157904690	1	59.769	KV452713.1	4124742	1-scf00011	1
AX-157904691	1	59.769	KV452713.1	4126641	1-scf00011	1
AX-157904693	1	59.769	KV452713.1	4128279	1-scf00011	1
AX-157904696	1	59.769	KV452713.1	4131687	1-scf00011	1
AX-157904699	1	59.769	KV452713.1	4135330	1-scf00011	1
AX-157904709	1	59.769	KV452713.1	4144926	1-scf00011	1
AX-157904711	1	59.769	KV452713.1	4146712	1-scf00011	1
AX-157904712	1	59.769	KV452713.1	4147386	1-scf00011	1
AX-157904713	1	59.769	KV452713.1	4148380	1-scf00011	1
AX-157726504	1	59.769	KV452713.1	4164083	1-scf00011	1
AX-157904732	1	59.769	KV452713.1	4175165	1-scf00011	1
AX-157904786	1	59.769	KV452713.1	4245764	1-scf00011	1
AX-157904801	1	59.769	KV452713.1	4266098	1-scf00011	1
AX-157698925	1	59.769	KV452713.1	4266636	1-scf00011	1
AX-157904812	1	59.769	KV452713.1	4285098	1-scf00011	1
AX-157748006	1	59.769	KV452713.1	4386014	1-scf00011	1
AX-157904901	1	59.769	KV452713.1	4396481	1-scf00011	1
AX-157904909	1	59.769	KV452713.1	4407015	1-scf00011	1
AX-157904911	1	59.769	KV452713.1	4409215	1-scf00011	1
AX-157904921	1	59.769	KV452713.1	4423889	1-scf00011	1
AX-157904966	1	59.769	KV452713.1	4485808	1-scf00011	1
AX-157901761	1	59.878	KV452711.1	3197693	1-scf00010	1
AX-157904715	1	59.878	KV452713.1	4150183	1-scf00011	1
AX-157904720	1	59.878	KV452713.1	4152648	1-scf00011	1
AX-157904725	1	59.878	KV452713.1	4166315	1-scf00011	1
AX-157904727	1	59.878	KV452713.1	4168030	1-scf00011	1
AX-157749560	1	59.89	KV452713.1	4169690	1-scf00011	1
AX-157904728	1	59.907	KV452713.1	4170513	1-scf00011	1
AX-157904729	1	59.919	KV452713.1	4171446	1-scf00011	1
AX-157902921	1	59.928	KV452713.1	1466930	1-scf00011	1
AX-157903818	1	59.928	KV452713.1	2590216	1-scf00011	1
AX-157903830	1	59.928	KV452713.1	2605842	1-scf00011	1
AX-157904736	1	59.928	KV452713.1	4180375	1-scf00011	1
AX-157771035	1	59.937	KV452713.1	2182243	1-scf00011	1
AX-157903507	1	59.937	KV452713.1	2196852	1-scf00011	1
AX-157904039	1	59.937	KV452713.1	2928393	1-scf00011	1
AX-157904103	1	59.937	KV452713.1	3042593	1-scf00011	1
AX-157904738	1	59.937	KV452713.1	4181879	1-scf00011	1
AX-157904744	1	59.937	KV452713.1	4188044	1-scf00011	1
AX-157904745	1	59.937	KV452713.1	4192496	1-scf00011	1
AX-157904747	1	59.946	KV452713.1	4196343	1-scf00011	1
AX-157904758	1	59.946	KV452713.1	4207452	1-scf00011	1
AX-157904776	1	59.946	KV452713.1	4228493	1-scf00011	1
AX-157904847	1	59.946	KV452713.1	4327907	1-scf00011	1
AX-157904753	1	59.957	KV452713.1	4203392	1-scf00011	1
AX-157904756	1	59.966	KV452713.1	4205988	1-scf00011	1
AX-157904717	1	59.978	KV452713.1	4151276	1-scf00011	1
AX-157749885	1	59.978	KV452713.1	4208377	1-scf00011	1
AX-157904792	1	59.978	KV452713.1	4252988	1-scf00011	1
AX-157896279	1	59.988	KV452709.1	244300	1-scf00009	1
AX-157757737	1	59.988	KV452713.1	4018767	1-scf00011	1

AX-157904686	1	59.988	KV452713.1	4120776	1-scf00011	1
AX-157811846	1	59.988	KV452713.1	4133893	1-scf00011	1
AX-158379172	1	59.988	KV452713.1	4214569	1-scf00011	1
AX-157904764	1	59.988	KV452713.1	4215320	1-scf00011	1
AX-157904765	1	59.988	KV452713.1	4216527	1-scf00011	1
AX-157904771	1	59.988	KV452713.1	4224557	1-scf00011	1
AX-157904775	1	59.988	KV452713.1	4227007	1-scf00011	1
AX-157904778	1	59.988	KV452713.1	4233155	1-scf00011	1
AX-157904780	1	59.988	KV452713.1	4236169	1-scf00011	1
AX-157904781	1	59.988	KV452713.1	4238278	1-scf00011	1
AX-157904782	1	59.988	KV452713.1	4238793	1-scf00011	1
AX-157904783	1	59.988	KV452713.1	4242166	1-scf00011	1
AX-157904818	1	59.988	KV452713.1	4291155	1-scf00011	1
AX-157904819	1	59.988	KV452713.1	4291795	1-scf00011	1
AX-157904835	1	59.988	KV452713.1	4312744	1-scf00011	1
AX-157742642	1	59.988	KV452713.1	4448607	1-scf00011	1
AX-157853612	1	59.988	KV452713.1	4452284	1-scf00011	1
AX-157898343	1	60.097	KV452709.1	3467787	1-scf00009	1
AX-157904526	1	60.097	KV452713.1	3890179	1-scf00011	1
AX-157904651	1	60.097	KV452713.1	4077303	1-scf00011	1
AX-157904743	1	60.097	KV452713.1	4185844	1-scf00011	1
AX-157758572	1	60.097	KV452713.1	4240835	1-scf00011	1
AX-157904789	1	60.097	KV452713.1	4250846	1-scf00011	1
AX-157904790	1	60.097	KV452713.1	4251302	1-scf00011	1
AX-157904813	1	60.097	KV452713.1	4285701	1-scf00011	1
AX-157904842	1	60.097	KV452713.1	4321643	1-scf00011	1
AX-157904191	1	60.206	KV452713.1	3236501	1-scf00011	1
AX-157904193	1	60.206	KV452713.1	3241040	1-scf00011	1
AX-157904262	1	60.206	KV452713.1	3428796	1-scf00011	1
AX-157904723	1	60.206	KV452713.1	4157228	1-scf00011	1
AX-157726436	1	60.206	KV452713.1	4173518	1-scf00011	1
AX-157904739	1	60.206	KV452713.1	4183091	1-scf00011	1
AX-157904740	1	60.206	KV452713.1	4183399	1-scf00011	1
AX-157904800	1	60.206	KV452713.1	4263751	1-scf00011	1
AX-157904810	1	60.206	KV452713.1	4280468	1-scf00011	1
AX-157904811	1	60.206	KV452713.1	4284569	1-scf00011	1
AX-157904814	1	60.206	KV452713.1	4287307	1-scf00011	1
AX-157904817	1	60.206	KV452713.1	4289120	1-scf00011	1
AX-157904822	1	60.206	KV452713.1	4295377	1-scf00011	1
AX-157904823	1	60.206	KV452713.1	4296954	1-scf00011	1
AX-157904832	1	60.206	KV452713.1	4308806	1-scf00011	1
AX-157904837	1	60.206	KV452713.1	4314390	1-scf00011	1
AX-157904841	1	60.206	KV452713.1	4320683	1-scf00011	1
AX-157904843	1	60.206	KV452713.1	4323572	1-scf00011	1
AX-157811848	1	60.206	KV452713.1	4333361	1-scf00011	1
AX-157904851	1	60.206	KV452713.1	4333899	1-scf00011	1
AX-157811849	1	60.206	KV452713.1	4334183	1-scf00011	1
AX-157904852	1	60.206	KV452713.1	4336060	1-scf00011	1
AX-157904854	1	60.206	KV452713.1	4337625	1-scf00011	1
AX-157709946	1	60.206	KV452713.1	4340444	1-scf00011	1
AX-157904857	1	60.206	KV452713.1	4340877	1-scf00011	1

AX-157874436	1	60.206	KV452713.1	4341679	1-scf00011	1
AX-157721362	1	60.206	KV452713.1	4344982	1-scf00011	1
AX-157904878	1	60.206	KV452713.1	4368366	1-scf00011	1
AX-157904880	1	60.206	KV452713.1	4370755	1-scf00011	1
AX-157904881	1	60.206	KV452713.1	4373139	1-scf00011	1
AX-157904885	1	60.206	KV452713.1	4377320	1-scf00011	1
AX-157904894	1	60.424	KV452713.1	4388507	1-scf00011	1
AX-157756869	1	60.424	KV452713.1	4389321	1-scf00011	1
AX-157904895	1	60.424	KV452713.1	4389927	1-scf00011	1
AX-157904896	1	60.424	KV452713.1	4392422	1-scf00011	1
AX-157904897	1	60.424	KV452713.1	4393573	1-scf00011	1
AX-157904900	1	60.424	KV452713.1	4395231	1-scf00011	1
AX-157904903	1	60.564	KV452713.1	4398564	1-scf00011	1
AX-157904860	1	60.643	KV452713.1	4345376	1-scf00011	1
AX-157904904	1	60.643	KV452713.1	4401234	1-scf00011	1
AX-157904908	1	60.643	KV452713.1	4404857	1-scf00011	1
AX-157904980	1	60.643	KV452713.1	4502533	1-scf00011	1
AX-157904907	1	60.691	KV452713.1	4404004	1-scf00011	1
AX-157904641	1	60.752	KV452713.1	4069414	1-scf00011	1
AX-158382049	1	60.752	KV452713.1	4248726	1-scf00011	1
AX-157904913	1	60.752	KV452713.1	4410843	1-scf00011	1
AX-157904919	1	60.752	KV452713.1	4417000	1-scf00011	1
AX-157725230	1	60.752	KV452713.1	4420792	1-scf00011	1
AX-157811856	1	60.815	KV452713.1	4422393	1-scf00011	1
AX-157904926	1	60.891	KV452713.1	4430350	1-scf00011	1
AX-157904979	1	60.891	KV452713.1	4501690	1-scf00011	1
AX-157905012	1	60.891	KV452713.1	4542178	1-scf00011	1
AX-157905013	1	60.891	KV452713.1	4542750	1-scf00011	1
AX-157721106	1	60.891	KV452713.1	4547613	1-scf00011	1
AX-157904798	1	60.971	KV452713.1	4257429	1-scf00011	1
AX-157904846	1	60.971	KV452713.1	4327334	1-scf00011	1
AX-157740009	1	60.971	KV452713.1	4421791	1-scf00011	1
AX-157904927	1	60.971	KV452713.1	4432409	1-scf00011	1
AX-157904928	1	60.971	KV452713.1	4433055	1-scf00011	1
AX-157904930	1	60.971	KV452713.1	4435935	1-scf00011	1
AX-157904931	1	60.971	KV452713.1	4437628	1-scf00011	1
AX-157717071	1	60.971	KV452713.1	4439419	1-scf00011	1
AX-157904934	1	60.971	KV452713.1	4444302	1-scf00011	1
AX-157904935	1	60.971	KV452713.1	4449270	1-scf00011	1
AX-157904936	1	60.971	KV452713.1	4451446	1-scf00011	1
AX-157904937	1	60.971	KV452713.1	4452762	1-scf00011	1
AX-157904938	1	60.971	KV452713.1	4453271	1-scf00011	1
AX-157904939	1	60.971	KV452713.1	4453681	1-scf00011	1
AX-157904943	1	60.971	KV452713.1	4456496	1-scf00011	1
AX-157904944	1	60.971	KV452713.1	4457139	1-scf00011	1
AX-157775262	1	60.971	KV452713.1	4471449	1-scf00011	1
AX-157904962	1	60.971	KV452713.1	4482700	1-scf00011	1
AX-157904970	1	60.971	KV452713.1	4490645	1-scf00011	1
AX-157811857	1	61.085	KV452713.1	4463375	1-scf00011	1
AX-157904958	1	61.154	KV452713.1	4474055	1-scf00011	1
AX-157904959	1	61.154	KV452713.1	4475477	1-scf00011	1

AX-157904960	1	61.154	KV452713.1	4477929	1-scf00011	1
AX-157904961	1	61.154	KV452713.1	4479064	1-scf00011	1
AX-157904963	1	61.154	KV452713.1	4483236	1-scf00011	1
AX-157904964	1	61.154	KV452713.1	4484382	1-scf00011	1
AX-157904975	1	61.241	KV452713.1	4495400	1-scf00011	1
AX-157904976	1	61.241	KV452713.1	4496493	1-scf00011	1
AX-157904984	1	61.336	KV452713.1	4510339	1-scf00011	1
AX-157904986	1	61.336	KV452713.1	4511347	1-scf00011	1
AX-157904990	1	61.336	KV452713.1	4515619	1-scf00011	1
AX-157904995	1	61.336	KV452713.1	4525130	1-scf00011	1
AX-157905355	1	61.336	KV452713.1	5033049	1-scf00011	1
AX-157743591	1	61.336	KV452713.1	5062417	1-scf00011	1
AX-157905390	1	61.336	KV452713.1	5091607	1-scf00011	1
AX-157905893	1	61.336	KV452713.1	5832868	1-scf00011	1
AX-157905898	1	61.336	KV452713.1	5841279	1-scf00011	1
AX-157905958	1	61.336	KV452713.1	5912319	1-scf00011	1
AX-157904993	1	61.43	KV452713.1	4521165	1-scf00011	1
AX-157905001	1	61.43	KV452713.1	4530110	1-scf00011	1
AX-157905006	1	61.43	KV452713.1	4538525	1-scf00011	1
AX-157905007	1	61.43	KV452713.1	4538881	1-scf00011	1
AX-157905042	1	61.43	KV452713.1	4576718	1-scf00011	1
AX-157905103	1	61.43	KV452713.1	4648141	1-scf00011	1
AX-157905010	1	61.515	KV452713.1	4540716	1-scf00011	1
AX-157905011	1	61.515	KV452713.1	4541781	1-scf00011	1
AX-157905003	1	61.518	KV452713.1	4536668	1-scf00011	1
AX-157905008	1	61.518	KV452713.1	4539524	1-scf00011	1
AX-157905015	1	61.518	KV452713.1	4545936	1-scf00011	1
AX-157905022	1	61.871	KV452713.1	4561217	1-scf00011	1
AX-158382053	1	61.871	KV452713.1	4562096	1-scf00011	1
AX-157905026	1	61.872	KV452713.1	4565348	1-scf00011	1
AX-157904985	1	61.874	KV452713.1	4510940	1-scf00011	1
AX-157904987	1	61.874	KV452713.1	4512994	1-scf00011	1
AX-157811860	1	61.874	KV452713.1	4523417	1-scf00011	1
AX-157905023	1	61.874	KV452713.1	4562051	1-scf00011	1
AX-157724576	1	61.874	KV452713.1	4563816	1-scf00011	1
AX-157905031	1	61.874	KV452713.1	4568950	1-scf00011	1
AX-157905033	1	61.874	KV452713.1	4570902	1-scf00011	1
AX-157905038	1	61.874	KV452713.1	4575022	1-scf00011	1
AX-157905039	1	61.874	KV452713.1	4575474	1-scf00011	1
AX-157905040	1	61.874	KV452713.1	4575842	1-scf00011	1
AX-157905041	1	61.874	KV452713.1	4576194	1-scf00011	1
AX-157905043	1	61.874	KV452713.1	4579277	1-scf00011	1
AX-157905051	1	61.874	KV452713.1	4584377	1-scf00011	1
AX-157905053	1	61.874	KV452713.1	4588078	1-scf00011	1
AX-157905054	1	61.874	KV452713.1	4588929	1-scf00011	1
AX-157811866	1	61.874	KV452713.1	4591126	1-scf00011	1
AX-157905056	1	61.874	KV452713.1	4596091	1-scf00011	1
AX-157905057	1	61.874	KV452713.1	4597428	1-scf00011	1
AX-157905059	1	61.874	KV452713.1	4599083	1-scf00011	1
AX-157905071	1	61.874	KV452713.1	4611658	1-scf00011	1
AX-157905078	1	61.874	KV452713.1	4621112	1-scf00011	1

AX-157905095	1	61.874	KV452713.1	4638295	1-scf00011	1
AX-157811881	1	61.874	KV452713.1	4811040	1-scf00011	1
AX-157904836	1	62.053	KV452713.1	4313396	1-scf00011	1
AX-157904838	1	62.053	KV452713.1	4315351	1-scf00011	1
AX-157904965	1	62.053	KV452713.1	4485349	1-scf00011	1
AX-157904983	1	62.053	KV452713.1	4508415	1-scf00011	1
AX-158414694	1	62.053	KV452713.1	4556970	1-scf00011	1
AX-157905025	1	62.053	KV452713.1	4564937	1-scf00011	1
AX-157905027	1	62.053	KV452713.1	4565871	1-scf00011	1
AX-157905036	1	62.053	KV452713.1	4572733	1-scf00011	1
AX-157715560	1	62.053	KV452713.1	4589561	1-scf00011	1
AX-157905055	1	62.053	KV452713.1	4594957	1-scf00011	1
AX-157811868	1	62.053	KV452713.1	4600364	1-scf00011	1
AX-157905062	1	62.053	KV452713.1	4601535	1-scf00011	1
AX-157905063	1	62.053	KV452713.1	4602122	1-scf00011	1
AX-157905065	1	62.053	KV452713.1	4604030	1-scf00011	1
AX-157905072	1	62.053	KV452713.1	4612610	1-scf00011	1
AX-157905074	1	62.053	KV452713.1	4614398	1-scf00011	1
AX-157905076	1	62.053	KV452713.1	4617830	1-scf00011	1
AX-157905077	1	62.053	KV452713.1	4620732	1-scf00011	1
AX-157905081	1	62.053	KV452713.1	4625512	1-scf00011	1
AX-157905083	1	62.053	KV452713.1	4626499	1-scf00011	1
AX-157905084	1	62.053	KV452713.1	4627066	1-scf00011	1
AX-157714260	1	62.053	KV452713.1	4628152	1-scf00011	1
AX-157714075	1	62.053	KV452713.1	4628616	1-scf00011	1
AX-157905085	1	62.053	KV452713.1	4629595	1-scf00011	1
AX-157905090	1	62.053	KV452713.1	4632017	1-scf00011	1
AX-157905091	1	62.053	KV452713.1	4633174	1-scf00011	1
AX-157739760	1	62.053	KV452713.1	4634048	1-scf00011	1
AX-157905092	1	62.053	KV452713.1	4634969	1-scf00011	1
AX-157905094	1	62.053	KV452713.1	4636071	1-scf00011	1
AX-157905096	1	62.053	KV452713.1	4639669	1-scf00011	1
AX-157905098	1	62.053	KV452713.1	4642311	1-scf00011	1
AX-157905099	1	62.053	KV452713.1	4642805	1-scf00011	1
AX-157905100	1	62.053	KV452713.1	4643619	1-scf00011	1
AX-157905114	1	62.053	KV452713.1	4668469	1-scf00011	1
AX-157905131	1	62.053	KV452713.1	4699203	1-scf00011	1
AX-157905318	1	62.053	KV452713.1	4987777	1-scf00011	1
AX-158379174	1	62.054	KV452713.1	4643815	1-scf00011	1
AX-157905112	1	62.56	KV452713.1	4664032	1-scf00011	1
AX-157739647	1	62.591	KV452713.1	4666773	1-scf00011	1
AX-157905115	1	62.591	KV452713.1	4668732	1-scf00011	1
AX-157905117	1	62.591	KV452713.1	4669957	1-scf00011	1
AX-157905118	1	62.591	KV452713.1	4673333	1-scf00011	1
AX-157905119	1	62.591	KV452713.1	4674327	1-scf00011	1
AX-157905122	1	62.591	KV452713.1	4677189	1-scf00011	1
AX-157752214	1	62.591	KV452713.1	4677837	1-scf00011	1
AX-157905219	1	62.591	KV452713.1	4835888	1-scf00011	1
AX-157775082	1	62.591	KV452713.1	4836574	1-scf00011	1
AX-157905124	1	62.864	KV452713.1	4688384	1-scf00011	1
AX-157709616	1	62.864	KV452713.1	4689228	1-scf00011	1

AX-157905130	1	62.865	KV452713.1	4698473	1-scf00011	1
AX-157905137	1	63.118	KV452713.1	4706203	1-scf00011	1
AX-157905158	1	63.118	KV452713.1	4738236	1-scf00011	1
AX-157905138	1	63.128	KV452713.1	4707206	1-scf00011	1
AX-157905140	1	63.128	KV452713.1	4709049	1-scf00011	1
AX-157905155	1	63.128	KV452713.1	4735653	1-scf00011	1
AX-157905156	1	63.128	KV452713.1	4736069	1-scf00011	1
AX-157905157	1	63.128	KV452713.1	4736459	1-scf00011	1
AX-157811875	1	63.128	KV452713.1	4736923	1-scf00011	1
AX-157741498	1	63.128	KV452713.1	4737374	1-scf00011	1
AX-157905162	1	63.128	KV452713.1	4751596	1-scf00011	1
AX-157905164	1	63.128	KV452713.1	4753369	1-scf00011	1
AX-157905171	1	63.128	KV452713.1	4777683	1-scf00011	1
AX-157905178	1	63.128	KV452713.1	4785627	1-scf00011	1
AX-157905179	1	63.128	KV452713.1	4788036	1-scf00011	1
AX-157905181	1	63.128	KV452713.1	4790928	1-scf00011	1
AX-157905184	1	63.128	KV452713.1	4795488	1-scf00011	1
AX-157905185	1	63.128	KV452713.1	4795699	1-scf00011	1
AX-157905189	1	63.128	KV452713.1	4797662	1-scf00011	1
AX-157905190	1	63.128	KV452713.1	4798527	1-scf00011	1
AX-157905192	1	63.128	KV452713.1	4801137	1-scf00011	1
AX-157905193	1	63.128	KV452713.1	4802522	1-scf00011	1
AX-157811879	1	63.128	KV452713.1	4802794	1-scf00011	1
AX-157905194	1	63.128	KV452713.1	4803847	1-scf00011	1
AX-157905196	1	63.128	KV452713.1	4810127	1-scf00011	1
AX-157811880	1	63.128	KV452713.1	4810237	1-scf00011	1
AX-157746967	1	63.128	KV452713.1	4812135	1-scf00011	1
AX-157905197	1	63.128	KV452713.1	4813022	1-scf00011	1
AX-157772049	1	63.128	KV452713.1	4813793	1-scf00011	1
AX-157905200	1	63.128	KV452713.1	4815946	1-scf00011	1
AX-157905201	1	63.128	KV452713.1	4816860	1-scf00011	1
AX-157905203	1	63.128	KV452713.1	4819351	1-scf00011	1
AX-157905208	1	63.128	KV452713.1	4827087	1-scf00011	1
AX-157905209	1	63.128	KV452713.1	4827673	1-scf00011	1
AX-157905213	1	63.128	KV452713.1	4831592	1-scf00011	1
AX-157905220	1	63.128	KV452713.1	4837721	1-scf00011	1
AX-157905227	1	63.128	KV452713.1	4841589	1-scf00011	1
AX-157905232	1	63.128	KV452713.1	4844872	1-scf00011	1
AX-157905234	1	63.128	KV452713.1	4846564	1-scf00011	1
AX-157905237	1	63.128	KV452713.1	4849336	1-scf00011	1
AX-157905241	1	63.128	KV452713.1	4854442	1-scf00011	1
AX-157905242	1	63.128	KV452713.1	4854790	1-scf00011	1
AX-157811887	1	63.128	KV452713.1	4861673	1-scf00011	1
AX-157905246	1	63.128	KV452713.1	4863682	1-scf00011	1
AX-157905247	1	63.128	KV452713.1	4865258	1-scf00011	1
AX-157883325	1	63.128	KV452713.1	4865892	1-scf00011	1
AX-158379175	1	63.128	KV452713.1	4866992	1-scf00011	1
AX-157905249	1	63.128	KV452713.1	4867630	1-scf00011	1
AX-157905250	1	63.128	KV452713.1	4868235	1-scf00011	1
AX-157712223	1	63.128	KV452713.1	4874627	1-scf00011	1
AX-157905253	1	63.128	KV452713.1	4876013	1-scf00011	1

AX-157905256	1	63.128	KV452713.1	4880594	1-scf00011	1
AX-157746548	1	63.128	KV452713.1	4883064	1-scf00011	1
AX-157811891	1	63.128	KV452713.1	4883143	1-scf00011	1
AX-157811892	1	63.128	KV452713.1	4883756	1-scf00011	1
AX-157905259	1	63.128	KV452713.1	4883868	1-scf00011	1
AX-157905553	1	63.128	KV452713.1	5339838	1-scf00011	1
AX-157700384	1	63.128	KV452713.1	5445675	1-scf00011	1
AX-157905797	1	63.128	KV452713.1	5699250	1-scf00011	1
AX-157905932	1	63.128	KV452713.1	5877358	1-scf00011	1
AX-157905264	1	63.604	KV452713.1	4896661	1-scf00011	1
AX-157775723	1	63.604	KV452713.1	4899512	1-scf00011	1
AX-157905045	1	63.609	KV452713.1	4580186	1-scf00011	1
AX-157714577	1	63.609	KV452713.1	4901613	1-scf00011	1
AX-157905269	1	63.609	KV452713.1	4903074	1-scf00011	1
AX-157905270	1	63.609	KV452713.1	4903515	1-scf00011	1
AX-157905271	1	63.609	KV452713.1	4904598	1-scf00011	1
AX-157697596	1	63.609	KV452713.1	4919357	1-scf00011	1
AX-157905278	1	63.609	KV452713.1	4926043	1-scf00011	1
AX-157905290	1	63.609	KV452713.1	4943040	1-scf00011	1
AX-157905303	1	63.609	KV452713.1	4968259	1-scf00011	1
AX-157905319	1	63.609	KV452713.1	4988437	1-scf00011	1
AX-157811905	1	63.609	KV452713.1	5042685	1-scf00011	1
AX-157905366	1	63.609	KV452713.1	5055916	1-scf00011	1
AX-157905424	1	63.609	KV452713.1	5144951	1-scf00011	1
AX-157905461	1	63.609	KV452713.1	5201766	1-scf00011	1
AX-157905511	1	63.609	KV452713.1	5275230	1-scf00011	1
AX-157905517	1	63.609	KV452713.1	5285768	1-scf00011	1
AX-157721274	1	63.609	KV452713.1	5295015	1-scf00011	1
AX-157905529	1	63.609	KV452713.1	5311808	1-scf00011	1
AX-157905537	1	63.609	KV452713.1	5319606	1-scf00011	1
AX-157905541	1	63.609	KV452713.1	5326808	1-scf00011	1
AX-157905558	1	63.609	KV452713.1	5351944	1-scf00011	1
AX-158421836	1	63.609	KV452713.1	5887756	1-scf00011	1
AX-157905301	1	63.681	KV452713.1	4962598	1-scf00011	1
AX-157905312	1	63.762	KV452713.1	4982506	1-scf00011	1
AX-157905314	1	63.838	KV452713.1	4983471	1-scf00011	1
AX-157905371	1	63.838	KV452713.1	5061916	1-scf00011	1
AX-157897010	1	63.845	KV452709.1	1261299	1-scf00009	1
AX-157897015	1	63.845	KV452709.1	1270337	1-scf00009	1
AX-158389491	1	63.845	KV452713.1	3883028	1-scf00011	1
AX-157905123	1	63.845	KV452713.1	4678942	1-scf00011	1
AX-157905266	1	63.845	KV452713.1	4897857	1-scf00011	1
AX-157905272	1	63.845	KV452713.1	4905588	1-scf00011	1
AX-157905292	1	63.845	KV452713.1	4947796	1-scf00011	1
AX-157905295	1	63.845	KV452713.1	4952825	1-scf00011	1
AX-157905296	1	63.845	KV452713.1	4953723	1-scf00011	1
AX-157905310	1	63.845	KV452713.1	4981024	1-scf00011	1
AX-157905324	1	63.845	KV452713.1	4995962	1-scf00011	1
AX-157738178	1	63.845	KV452713.1	4997763	1-scf00011	1
AX-157905325	1	63.845	KV452713.1	4997996	1-scf00011	1
AX-157905330	1	63.845	KV452713.1	5003122	1-scf00011	1

AX-157905336	1	63.845	KV452713.1	5008082	1-scf00011	1
AX-157905338	1	63.845	KV452713.1	5011224	1-scf00011	1
AX-157905339	1	63.845	KV452713.1	5013194	1-scf00011	1
AX-157905343	1	63.845	KV452713.1	5019191	1-scf00011	1
AX-157905344	1	63.845	KV452713.1	5019661	1-scf00011	1
AX-157905348	1	63.845	KV452713.1	5023284	1-scf00011	1
AX-157905349	1	63.845	KV452713.1	5023882	1-scf00011	1
AX-157905350	1	63.845	KV452713.1	5024549	1-scf00011	1
AX-157905351	1	63.845	KV452713.1	5027698	1-scf00011	1
AX-157905353	1	63.845	KV452713.1	5031401	1-scf00011	1
AX-157905359	1	63.845	KV452713.1	5039986	1-scf00011	1
AX-157905361	1	63.845	KV452713.1	5042093	1-scf00011	1
AX-157905362	1	63.845	KV452713.1	5047786	1-scf00011	1
AX-157811906	1	63.845	KV452713.1	5051786	1-scf00011	1
AX-157905372	1	63.845	KV452713.1	5062773	1-scf00011	1
AX-157905382	1	63.845	KV452713.1	5076199	1-scf00011	1
AX-157905384	1	63.845	KV452713.1	5081309	1-scf00011	1
AX-157905385	1	63.845	KV452713.1	5081757	1-scf00011	1
AX-157905395	1	63.845	KV452713.1	5099491	1-scf00011	1
AX-157905398	1	63.845	KV452713.1	5102081	1-scf00011	1
AX-157905399	1	63.845	KV452713.1	5104703	1-scf00011	1
AX-157905400	1	63.845	KV452713.1	5105117	1-scf00011	1
AX-157905402	1	63.845	KV452713.1	5110113	1-scf00011	1
AX-157905403	1	63.845	KV452713.1	5111123	1-scf00011	1
AX-157905409	1	63.845	KV452713.1	5116910	1-scf00011	1
AX-157905411	1	63.845	KV452713.1	5118438	1-scf00011	1
AX-157905412	1	63.845	KV452713.1	5119475	1-scf00011	1
AX-157905413	1	63.845	KV452713.1	5119956	1-scf00011	1
AX-157905414	1	63.845	KV452713.1	5120649	1-scf00011	1
AX-157905416	1	63.845	KV452713.1	5133842	1-scf00011	1
AX-157905418	1	63.845	KV452713.1	5135716	1-scf00011	1
AX-157905419	1	63.845	KV452713.1	5139348	1-scf00011	1
AX-157905421	1	63.845	KV452713.1	5140155	1-scf00011	1
AX-157905427	1	63.845	KV452713.1	5155644	1-scf00011	1
AX-157905460	1	63.845	KV452713.1	5201139	1-scf00011	1
AX-158404730	1	63.845	KV452713.1	5201597	1-scf00011	1
AX-157905465	1	63.845	KV452713.1	5208325	1-scf00011	1
AX-157905466	1	63.845	KV452713.1	5209430	1-scf00011	1
AX-157905467	1	63.845	KV452713.1	5210329	1-scf00011	1
AX-157905470	1	63.845	KV452713.1	5223433	1-scf00011	1
AX-157905471	1	63.845	KV452713.1	5224015	1-scf00011	1
AX-157905473	1	63.845	KV452713.1	5226699	1-scf00011	1
AX-157811911	1	63.845	KV452713.1	5235730	1-scf00011	1
AX-157905480	1	63.845	KV452713.1	5235794	1-scf00011	1
AX-157811912	1	63.845	KV452713.1	5236515	1-scf00011	1
AX-157720843	1	63.845	KV452713.1	5236985	1-scf00011	1
AX-157905481	1	63.845	KV452713.1	5238038	1-scf00011	1
AX-157905483	1	63.845	KV452713.1	5240496	1-scf00011	1
AX-157905485	1	63.845	KV452713.1	5241977	1-scf00011	1
AX-158382057	1	63.845	KV452713.1	5243025	1-scf00011	1
AX-157905486	1	63.845	KV452713.1	5244921	1-scf00011	1

AX-157905488	1	63.845	KV452713.1	5246951	1-scf00011	1
AX-157905489	1	63.845	KV452713.1	5247618	1-scf00011	1
AX-157905491	1	63.845	KV452713.1	5251004	1-scf00011	1
AX-157811917	1	63.845	KV452713.1	5254693	1-scf00011	1
AX-157905499	1	63.845	KV452713.1	5258883	1-scf00011	1
AX-157713520	1	63.845	KV452713.1	5259680	1-scf00011	1
AX-157905501	1	63.845	KV452713.1	5265147	1-scf00011	1
AX-157905502	1	63.845	KV452713.1	5266896	1-scf00011	1
AX-157811920	1	63.845	KV452713.1	5267691	1-scf00011	1
AX-157905503	1	63.845	KV452713.1	5268375	1-scf00011	1
AX-157905506	1	63.845	KV452713.1	5271554	1-scf00011	1
AX-157905507	1	63.845	KV452713.1	5272557	1-scf00011	1
AX-157905508	1	63.845	KV452713.1	5273224	1-scf00011	1
AX-157905510	1	63.845	KV452713.1	5274542	1-scf00011	1
AX-157905514	1	63.845	KV452713.1	5279239	1-scf00011	1
AX-157905515	1	63.845	KV452713.1	5282195	1-scf00011	1
AX-157905516	1	63.845	KV452713.1	5282613	1-scf00011	1
AX-157709200	1	63.845	KV452713.1	5283180	1-scf00011	1
AX-157905519	1	63.845	KV452713.1	5294262	1-scf00011	1
AX-157905520	1	63.845	KV452713.1	5294658	1-scf00011	1
AX-157905522	1	63.845	KV452713.1	5299268	1-scf00011	1
AX-157905525	1	63.845	KV452713.1	5305563	1-scf00011	1
AX-157905526	1	63.845	KV452713.1	5306361	1-scf00011	1
AX-157811921	1	63.845	KV452713.1	5308868	1-scf00011	1
AX-157905528	1	63.845	KV452713.1	5310762	1-scf00011	1
AX-157905534	1	63.845	KV452713.1	5316484	1-scf00011	1
AX-157905535	1	63.845	KV452713.1	5318001	1-scf00011	1
AX-157905538	1	63.845	KV452713.1	5320215	1-scf00011	1
AX-157905544	1	63.845	KV452713.1	5328292	1-scf00011	1
AX-157905545	1	63.845	KV452713.1	5328950	1-scf00011	1
AX-157905547	1	63.845	KV452713.1	5334818	1-scf00011	1
AX-157905548	1	63.845	KV452713.1	5336175	1-scf00011	1
AX-157905549	1	63.845	KV452713.1	5336804	1-scf00011	1
AX-157905559	1	63.845	KV452713.1	5353437	1-scf00011	1
AX-157905560	1	63.845	KV452713.1	5355298	1-scf00011	1
AX-157905563	1	63.845	KV452713.1	5359081	1-scf00011	1
AX-157905605	1	63.845	KV452713.1	5425263	1-scf00011	1
AX-157905617	1	63.845	KV452713.1	5441140	1-scf00011	1
AX-157905619	1	63.845	KV452713.1	5444189	1-scf00011	1
AX-157905626	1	63.845	KV452713.1	5453173	1-scf00011	1
AX-157905634	1	63.845	KV452713.1	5462660	1-scf00011	1
AX-157905652	1	63.845	KV452713.1	5483016	1-scf00011	1
AX-157905662	1	63.845	KV452713.1	5493944	1-scf00011	1
AX-157905692	1	63.845	KV452713.1	5540833	1-scf00011	1
AX-157905708	1	63.845	KV452713.1	5560154	1-scf00011	1
AX-157905719	1	63.845	KV452713.1	5577186	1-scf00011	1
AX-157905729	1	63.845	KV452713.1	5596581	1-scf00011	1
AX-157905739	1	63.845	KV452713.1	5609827	1-scf00011	1
AX-157905749	1	63.845	KV452713.1	5628296	1-scf00011	1
AX-157905750	1	63.845	KV452713.1	5629193	1-scf00011	1
AX-157905789	1	63.845	KV452713.1	5687809	1-scf00011	1

AX-157811927	1	63.845	KV452713.1	5688513	1-scf00011	1
AX-157726694	1	63.845	KV452713.1	5689704	1-scf00011	1
AX-157905793	1	63.845	KV452713.1	5696100	1-scf00011	1
AX-157905798	1	63.845	KV452713.1	5701027	1-scf00011	1
AX-157905823	1	63.845	KV452713.1	5735498	1-scf00011	1
AX-157905836	1	63.845	KV452713.1	5754419	1-scf00011	1
AX-157905855	1	63.845	KV452713.1	5784570	1-scf00011	1
AX-157874443	1	63.896	KV452713.1	5334137	1-scf00011	1
AX-157905570	1	63.94	KV452713.1	5381230	1-scf00011	1
AX-157905455	1	63.974	KV452713.1	5195274	1-scf00011	1
AX-157905571	1	63.974	KV452713.1	5382134	1-scf00011	1
AX-157811371	1	64.024	KV452709.1	6141152	1-scf00009	1
AX-157905173	1	64.024	KV452713.1	4780435	1-scf00011	1
AX-157811898	1	64.024	KV452713.1	4939869	1-scf00011	1
AX-157811899	1	64.024	KV452713.1	4945045	1-scf00011	1
AX-157905305	1	64.024	KV452713.1	4970737	1-scf00011	1
AX-157905315	1	64.024	KV452713.1	4983945	1-scf00011	1
AX-157905333	1	64.024	KV452713.1	5006100	1-scf00011	1
AX-157905346	1	64.024	KV452713.1	5021293	1-scf00011	1
AX-157905363	1	64.024	KV452713.1	5049338	1-scf00011	1
AX-157905379	1	64.024	KV452713.1	5071872	1-scf00011	1
AX-157905380	1	64.024	KV452713.1	5073110	1-scf00011	1
AX-157905381	1	64.024	KV452713.1	5073686	1-scf00011	1
AX-157905386	1	64.024	KV452713.1	5082811	1-scf00011	1
AX-157905401	1	64.024	KV452713.1	5108805	1-scf00011	1
AX-157905405	1	64.024	KV452713.1	5112457	1-scf00011	1
AX-157719781	1	64.024	KV452713.1	5177178	1-scf00011	1
AX-157905476	1	64.024	KV452713.1	5230699	1-scf00011	1
AX-157747904	1	64.024	KV452713.1	5243720	1-scf00011	1
AX-157905494	1	64.024	KV452713.1	5252560	1-scf00011	1
AX-157905498	1	64.024	KV452713.1	5257993	1-scf00011	1
AX-157811919	1	64.024	KV452713.1	5260826	1-scf00011	1
AX-157905504	1	64.024	KV452713.1	5269456	1-scf00011	1
AX-157905524	1	64.024	KV452713.1	5302987	1-scf00011	1
AX-157905533	1	64.024	KV452713.1	5316244	1-scf00011	1
AX-157811922	1	64.024	KV452713.1	5318501	1-scf00011	1
AX-157905542	1	64.024	KV452713.1	5327444	1-scf00011	1
AX-157811923	1	64.024	KV452713.1	5335363	1-scf00011	1
AX-157905550	1	64.024	KV452713.1	5337493	1-scf00011	1
AX-157905554	1	64.024	KV452713.1	5341429	1-scf00011	1
AX-157905569	1	64.024	KV452713.1	5377401	1-scf00011	1
AX-157905576	1	64.024	KV452713.1	5387098	1-scf00011	1
AX-157811924	1	64.024	KV452713.1	5388099	1-scf00011	1
AX-157905578	1	64.024	KV452713.1	5389509	1-scf00011	1
AX-157707976	1	64.024	KV452713.1	5391564	1-scf00011	1
AX-157905580	1	64.024	KV452713.1	5392114	1-scf00011	1
AX-157905581	1	64.024	KV452713.1	5393942	1-scf00011	1
AX-157905582	1	64.024	KV452713.1	5397564	1-scf00011	1
AX-157905585	1	64.024	KV452713.1	5401141	1-scf00011	1
AX-157905587	1	64.024	KV452713.1	5402041	1-scf00011	1
AX-157905594	1	64.024	KV452713.1	5409623	1-scf00011	1

AX-158421834	1	64.024	KV452713.1	5411040	1-scf00011	1
AX-157905599	1	64.024	KV452713.1	5420183	1-scf00011	1
AX-157905600	1	64.024	KV452713.1	5421081	1-scf00011	1
AX-157905601	1	64.024	KV452713.1	5422316	1-scf00011	1
AX-157905602	1	64.024	KV452713.1	5423498	1-scf00011	1
AX-157905603	1	64.024	KV452713.1	5424209	1-scf00011	1
AX-157905604	1	64.024	KV452713.1	5424707	1-scf00011	1
AX-157905609	1	64.024	KV452713.1	5428653	1-scf00011	1
AX-157905610	1	64.024	KV452713.1	5429506	1-scf00011	1
AX-157905611	1	64.024	KV452713.1	5430842	1-scf00011	1
AX-157905612	1	64.024	KV452713.1	5432243	1-scf00011	1
AX-157905618	1	64.024	KV452713.1	5443321	1-scf00011	1
AX-157905624	1	64.024	KV452713.1	5451368	1-scf00011	1
AX-157905625	1	64.024	KV452713.1	5451840	1-scf00011	1
AX-157905630	1	64.024	KV452713.1	5457624	1-scf00011	1
AX-157905632	1	64.024	KV452713.1	5461505	1-scf00011	1
AX-157905633	1	64.024	KV452713.1	5461989	1-scf00011	1
AX-157905635	1	64.024	KV452713.1	5463200	1-scf00011	1
AX-157722538	1	64.024	KV452713.1	5464087	1-scf00011	1
AX-157905636	1	64.024	KV452713.1	5466810	1-scf00011	1
AX-157905637	1	64.024	KV452713.1	5467180	1-scf00011	1
AX-157905638	1	64.024	KV452713.1	5469427	1-scf00011	1
AX-157905640	1	64.024	KV452713.1	5470893	1-scf00011	1
AX-157905642	1	64.024	KV452713.1	5471960	1-scf00011	1
AX-157905644	1	64.024	KV452713.1	5473098	1-scf00011	1
AX-157905645	1	64.024	KV452713.1	5475243	1-scf00011	1
AX-157905649	1	64.024	KV452713.1	5480015	1-scf00011	1
AX-157905651	1	64.024	KV452713.1	5481721	1-scf00011	1
AX-157905654	1	64.024	KV452713.1	5484533	1-scf00011	1
AX-157905659	1	64.024	KV452713.1	5490616	1-scf00011	1
AX-157811925	1	64.024	KV452713.1	5490826	1-scf00011	1
AX-157905660	1	64.024	KV452713.1	5491634	1-scf00011	1
AX-157905669	1	64.024	KV452713.1	5504284	1-scf00011	1
AX-157905671	1	64.024	KV452713.1	5507438	1-scf00011	1
AX-157905679	1	64.024	KV452713.1	5514379	1-scf00011	1
AX-157905680	1	64.024	KV452713.1	5516335	1-scf00011	1
AX-157905681	1	64.024	KV452713.1	5516766	1-scf00011	1
AX-157905684	1	64.024	KV452713.1	5521938	1-scf00011	1
AX-157905685	1	64.024	KV452713.1	5522906	1-scf00011	1
AX-157905687	1	64.024	KV452713.1	5526181	1-scf00011	1
AX-157905688	1	64.024	KV452713.1	5526891	1-scf00011	1
AX-157905689	1	64.024	KV452713.1	5533019	1-scf00011	1
AX-157905694	1	64.024	KV452713.1	5547254	1-scf00011	1
AX-157905695	1	64.024	KV452713.1	5549783	1-scf00011	1
AX-157905696	1	64.024	KV452713.1	5550735	1-scf00011	1
AX-157905697	1	64.024	KV452713.1	5551261	1-scf00011	1
AX-157905699	1	64.024	KV452713.1	5552728	1-scf00011	1
AX-157905700	1	64.024	KV452713.1	5553244	1-scf00011	1
AX-157905702	1	64.024	KV452713.1	5556946	1-scf00011	1
AX-157905703	1	64.024	KV452713.1	5557740	1-scf00011	1
AX-157905705	1	64.024	KV452713.1	5558543	1-scf00011	1

AX-157905706	1	64.024	KV452713.1	5559313	1-scf00011	1
AX-157905707	1	64.024	KV452713.1	5559777	1-scf00011	1
AX-157905712	1	64.024	KV452713.1	5565618	1-scf00011	1
AX-157905717	1	64.024	KV452713.1	5575421	1-scf00011	1
AX-157905718	1	64.024	KV452713.1	5575981	1-scf00011	1
AX-157905723	1	64.024	KV452713.1	5585031	1-scf00011	1
AX-157905724	1	64.024	KV452713.1	5589117	1-scf00011	1
AX-157905725	1	64.024	KV452713.1	5590020	1-scf00011	1
AX-157905726	1	64.024	KV452713.1	5591182	1-scf00011	1
AX-157905727	1	64.024	KV452713.1	5592739	1-scf00011	1
AX-157905733	1	64.024	KV452713.1	5600238	1-scf00011	1
AX-157905734	1	64.024	KV452713.1	5601011	1-scf00011	1
AX-157905735	1	64.024	KV452713.1	5601729	1-scf00011	1
AX-157905736	1	64.024	KV452713.1	5602992	1-scf00011	1
AX-157905738	1	64.024	KV452713.1	5604663	1-scf00011	1
AX-157905740	1	64.024	KV452713.1	5611070	1-scf00011	1
AX-157905744	1	64.024	KV452713.1	5617418	1-scf00011	1
AX-157905745	1	64.024	KV452713.1	5618024	1-scf00011	1
AX-157905747	1	64.024	KV452713.1	5623833	1-scf00011	1
AX-157905748	1	64.024	KV452713.1	5627390	1-scf00011	1
AX-157905754	1	64.024	KV452713.1	5639373	1-scf00011	1
AX-157905756	1	64.024	KV452713.1	5642272	1-scf00011	1
AX-157905757	1	64.024	KV452713.1	5642417	1-scf00011	1
AX-158414704	1	64.024	KV452713.1	5642541	1-scf00011	1
AX-158404741	1	64.024	KV452713.1	5642608	1-scf00011	1
AX-157905767	1	64.024	KV452713.1	5652065	1-scf00011	1
AX-157905769	1	64.024	KV452713.1	5657446	1-scf00011	1
AX-157905773	1	64.024	KV452713.1	5670560	1-scf00011	1
AX-157905785	1	64.024	KV452713.1	5685234	1-scf00011	1
AX-157905787	1	64.024	KV452713.1	5686871	1-scf00011	1
AX-157905790	1	64.024	KV452713.1	5690255	1-scf00011	1
AX-157905791	1	64.024	KV452713.1	5693768	1-scf00011	1
AX-158389508	1	64.024	KV452713.1	5695344	1-scf00011	1
AX-158404744	1	64.024	KV452713.1	5714032	1-scf00011	1
AX-157905822	1	64.024	KV452713.1	5734848	1-scf00011	1
AX-157905858	1	64.024	KV452713.1	5790155	1-scf00011	1
AX-157905972	1	64.024	KV452713.1	5934630	1-scf00011	1
AX-157905977	1	64.024	KV452713.1	5943351	1-scf00011	1
AX-157906001	1	64.024	KV452713.1	5982225	1-scf00011	1
AX-157703884	1	64.381	DX100978.1	134	IORI212 Ictalu	UN
AX-157905323	1	64.383	KV452713.1	4995640	1-scf00011	1
AX-157905459	1	64.383	KV452713.1	5200879	1-scf00011	1
AX-157721413	1	64.383	KV452713.1	5520439	1-scf00011	1
AX-157905701	1	64.383	KV452713.1	5556249	1-scf00011	1
AX-157905743	1	64.383	KV452713.1	5616781	1-scf00011	1
AX-157905801	1	64.383	KV452713.1	5704475	1-scf00011	1
AX-157905803	1	64.383	KV452713.1	5707118	1-scf00011	1
AX-157905804	1	64.383	KV452713.1	5709415	1-scf00011	1
AX-157905810	1	64.383	KV452713.1	5721116	1-scf00011	1
AX-157725531	1	64.383	KV452713.1	5724272	1-scf00011	1
AX-157905814	1	64.383	KV452713.1	5724992	1-scf00011	1

AX-157905817	1	64.383	KV452713.1	5727924	1-scf00011	1
AX-157905818	1	64.383	KV452713.1	5729382	1-scf00011	1
AX-157905819	1	64.383	KV452713.1	5731385	1-scf00011	1
AX-157905820	1	64.383	KV452713.1	5732876	1-scf00011	1
AX-157905834	1	64.383	KV452713.1	5753355	1-scf00011	1
AX-157905835	1	64.383	KV452713.1	5753800	1-scf00011	1
AX-157905966	1	64.383	KV452713.1	5925489	1-scf00011	1
AX-157905982	1	64.383	KV452713.1	5950868	1-scf00011	1
AX-157905674	1	64.408	KV452713.1	5510716	1-scf00011	1
AX-157905800	1	64.408	KV452713.1	5703766	1-scf00011	1
AX-157905845	1	64.408	KV452713.1	5766319	1-scf00011	1
AX-157905847	1	64.442	KV452713.1	5769339	1-scf00011	1
AX-157905848	1	64.48	KV452713.1	5771174	1-scf00011	1
AX-157742121	1	64.519	KV452713.1	4549401	1-scf00011	1
AX-157905824	1	64.519	KV452713.1	5736339	1-scf00011	1
AX-157905849	1	64.519	KV452713.1	5776278	1-scf00011	1
AX-157905861	1	64.519	KV452713.1	5793922	1-scf00011	1
AX-157905806	1	64.562	KV452713.1	5710968	1-scf00011	1
AX-157905838	1	64.562	KV452713.1	5757790	1-scf00011	1
AX-157905856	1	64.562	KV452713.1	5785436	1-scf00011	1
AX-157905857	1	64.562	KV452713.1	5789422	1-scf00011	1
AX-157905423	1	64.741	KV452713.1	5143716	1-scf00011	1
AX-157905454	1	64.741	KV452713.1	5194866	1-scf00011	1
AX-157905556	1	64.741	KV452713.1	5347977	1-scf00011	1
AX-157905584	1	64.741	KV452713.1	5400690	1-scf00011	1
AX-157905595	1	64.741	KV452713.1	5411931	1-scf00011	1
AX-157905710	1	64.741	KV452713.1	5563757	1-scf00011	1
AX-157905842	1	64.741	KV452713.1	5761159	1-scf00011	1
AX-157905843	1	64.741	KV452713.1	5762484	1-scf00011	1
AX-157905852	1	64.741	KV452713.1	5780339	1-scf00011	1
AX-157905853	1	64.741	KV452713.1	5780905	1-scf00011	1
AX-157905860	1	64.741	KV452713.1	5792998	1-scf00011	1
AX-157905862	1	64.741	KV452713.1	5794419	1-scf00011	1
AX-157713308	1	64.741	KV452713.1	5795316	1-scf00011	1
AX-157905865	1	64.741	KV452713.1	5797547	1-scf00011	1
AX-157905868	1	64.741	KV452713.1	5800298	1-scf00011	1
AX-157811934	1	64.741	KV452713.1	5801322	1-scf00011	1
AX-157905873	1	64.741	KV452713.1	5812085	1-scf00011	1
AX-157905878	1	64.741	KV452713.1	5814367	1-scf00011	1
AX-157905879	1	64.741	KV452713.1	5814759	1-scf00011	1
AX-157905881	1	64.741	KV452713.1	5815844	1-scf00011	1
AX-157905886	1	64.741	KV452713.1	5822567	1-scf00011	1
AX-157905891	1	64.741	KV452713.1	5829640	1-scf00011	1
AX-158379179	1	64.741	KV452713.1	5830296	1-scf00011	1
AX-157719321	1	64.741	KV452713.1	5832501	1-scf00011	1
AX-157905897	1	64.741	KV452713.1	5840717	1-scf00011	1
AX-157905900	1	64.741	KV452713.1	5845805	1-scf00011	1
AX-157905902	1	64.741	KV452713.1	5847423	1-scf00011	1
AX-157811937	1	64.741	KV452713.1	5847482	1-scf00011	1
AX-157905907	1	64.741	KV452713.1	5850466	1-scf00011	1
AX-157905913	1	64.741	KV452713.1	5855161	1-scf00011	1

AX-157905929	1	64.741	KV452713.1	5874228	1-scf00011	1
AX-157905930	1	64.741	KV452713.1	5875976	1-scf00011	1
AX-157905934	1	64.741	KV452713.1	5883487	1-scf00011	1
AX-157905935	1	64.741	KV452713.1	5883989	1-scf00011	1
AX-157905943	1	64.741	KV452713.1	5894356	1-scf00011	1
AX-157905946	1	64.741	KV452713.1	5896066	1-scf00011	1
AX-157905950	1	64.741	KV452713.1	5899982	1-scf00011	1
AX-157905951	1	64.741	KV452713.1	5900889	1-scf00011	1
AX-157905952	1	64.741	KV452713.1	5903051	1-scf00011	1
AX-157905964	1	64.741	KV452713.1	5923307	1-scf00011	1
AX-157905965	1	64.741	KV452713.1	5924166	1-scf00011	1
AX-157905967	1	64.741	KV452713.1	5926181	1-scf00011	1
AX-157905968	1	64.741	KV452713.1	5926862	1-scf00011	1
AX-157720965	1	64.741	KV452713.1	5927244	1-scf00011	1
AX-157905970	1	64.741	KV452713.1	5931733	1-scf00011	1
AX-157905975	1	64.741	KV452713.1	5939011	1-scf00011	1
AX-157905976	1	64.741	KV452713.1	5941910	1-scf00011	1
AX-157905979	1	64.741	KV452713.1	5947796	1-scf00011	1
AX-157883328	1	64.741	KV452713.1	5951018	1-scf00011	1
AX-157905996	1	64.741	KV452713.1	5973170	1-scf00011	1
AX-157906005	1	64.741	KV452713.1	5986515	1-scf00011	1
AX-157906011	1	64.741	KV452713.1	5993475	1-scf00011	1
AX-157699337	1	64.741	KV452713.1	6003317	1-scf00011	1
AX-157906026	1	64.741	KV452713.1	6019726	1-scf00011	1
AX-157906028	1	64.741	KV452713.1	6024255	1-scf00011	1
AX-157906054	1	64.741	KV452713.1	6055682	1-scf00011	1
AX-157906055	1	64.741	KV452713.1	6056165	1-scf00011	1
AX-157906082	1	64.741	KV452713.1	6087905	1-scf00011	1
AX-157699092	1	64.741	FI899105.1	780	HORI212 Ictalu	UN
AX-157905983	1	64.92	KV452713.1	5952777	1-scf00011	1
AX-157755596	1	64.92	KV452713.1	5954168	1-scf00011	1
AX-157811939	1	64.92	KV452713.1	5962169	1-scf00011	1
AX-157905988	1	64.92	KV452713.1	5963865	1-scf00011	1
AX-157905991	1	64.92	KV452713.1	5969693	1-scf00011	1
AX-157905994	1	64.92	KV452713.1	5972077	1-scf00011	1
AX-157699047	1	65.1	KV452713.1	5810836	1-scf00011	1
AX-157905876	1	65.1	KV452713.1	5813210	1-scf00011	1
AX-157811936	1	65.1	KV452713.1	5837882	1-scf00011	1
AX-157905947	1	65.1	KV452713.1	5897798	1-scf00011	1
AX-158382070	1	65.1	KV452713.1	5923364	1-scf00011	1
AX-157811942	1	65.1	KV452713.1	5978612	1-scf00011	1
AX-157906000	1	65.1	KV452713.1	5981635	1-scf00011	1
AX-157906004	1	65.1	KV452713.1	5984832	1-scf00011	1
AX-157906006	1	65.1	KV452713.1	5987350	1-scf00011	1
AX-157906012	1	65.1	KV452713.1	5994981	1-scf00011	1
AX-157906014	1	65.1	KV452713.1	6000819	1-scf00011	1
AX-157906015	1	65.1	KV452713.1	6001348	1-scf00011	1
AX-157906017	1	65.1	KV452713.1	6004078	1-scf00011	1
AX-158404751	1	65.1	KV452713.1	6009071	1-scf00011	1
AX-157811944	1	65.1	KV452713.1	6009838	1-scf00011	1
AX-157906023	1	65.1	KV452713.1	6012435	1-scf00011	1

AX-157726776	1	65.1	KV452713.1	6017309	1-scf00011	1
AX-157906030	1	65.1	KV452713.1	6025100	1-scf00011	1
AX-157906044	1	65.1	KV452713.1	6045917	1-scf00011	1
AX-157906049	1	65.1	KV452713.1	6050876	1-scf00011	1
AX-157906154	1	65.1	KV452713.1	6182119	1-scf00011	1
AX-157811940	1	65.279	KV452713.1	5962362	1-scf00011	1
AX-157811941	1	65.279	KV452713.1	5977504	1-scf00011	1
AX-157755635	1	65.279	KV452713.1	6030597	1-scf00011	1
AX-157906032	1	65.279	KV452713.1	6031920	1-scf00011	1
AX-157906033	1	65.279	KV452713.1	6033296	1-scf00011	1
AX-157811947	1	65.279	KV452713.1	6034884	1-scf00011	1
AX-157811949	1	65.279	KV452713.1	6038688	1-scf00011	1
AX-157906037	1	65.279	KV452713.1	6039798	1-scf00011	1
AX-157906038	1	65.279	KV452713.1	6041778	1-scf00011	1
AX-157906043	1	65.326	KV452713.1	6045178	1-scf00011	1
AX-157905332	1	65.458	KV452713.1	5004585	1-scf00011	1
AX-157905358	1	65.458	KV452713.1	5039516	1-scf00011	1
AX-157874441	1	65.458	KV452713.1	5178456	1-scf00011	1
AX-157905539	1	65.458	KV452713.1	5321749	1-scf00011	1
AX-157905565	1	65.458	KV452713.1	5362284	1-scf00011	1
AX-157905591	1	65.458	KV452713.1	5404727	1-scf00011	1
AX-157905598	1	65.458	KV452713.1	5417549	1-scf00011	1
AX-157905607	1	65.458	KV452713.1	5426961	1-scf00011	1
AX-157905672	1	65.458	KV452713.1	5508572	1-scf00011	1
AX-157869039	1	65.458	KV452713.1	5809451	1-scf00011	1
AX-157905941	1	65.458	KV452713.1	5892628	1-scf00011	1
AX-157905949	1	65.458	KV452713.1	5899253	1-scf00011	1
AX-157906018	1	65.458	KV452713.1	6006220	1-scf00011	1
AX-157906053	1	65.458	KV452713.1	6055088	1-scf00011	1
AX-157906057	1	65.458	KV452713.1	6058859	1-scf00011	1
AX-157906060	1	65.458	KV452713.1	6061764	1-scf00011	1
AX-157906062	1	65.458	KV452713.1	6063724	1-scf00011	1
AX-157906063	1	65.458	KV452713.1	6064862	1-scf00011	1
AX-157906064	1	65.458	KV452713.1	6067046	1-scf00011	1
AX-157772224	1	65.458	KV452713.1	6067848	1-scf00011	1
AX-157906067	1	65.458	KV452713.1	6071295	1-scf00011	1
AX-158404752	1	65.522	KV452713.1	6069096	1-scf00011	1
AX-157906065	1	65.577	KV452713.1	6069839	1-scf00011	1
AX-157905377	1	65.637	KV452713.1	5070358	1-scf00011	1
AX-157905837	1	65.637	KV452713.1	5756858	1-scf00011	1
AX-157905928	1	65.637	KV452713.1	5871774	1-scf00011	1
AX-157905954	1	65.637	KV452713.1	5905797	1-scf00011	1
AX-157906066	1	65.637	KV452713.1	6070525	1-scf00011	1
AX-157906069	1	65.637	KV452713.1	6074969	1-scf00011	1
AX-157906071	1	65.637	KV452713.1	6076643	1-scf00011	1
AX-157906072	1	65.637	KV452713.1	6077038	1-scf00011	1
AX-157906073	1	65.637	KV452713.1	6077810	1-scf00011	1
AX-157906074	1	65.637	KV452713.1	6079635	1-scf00011	1
AX-157906076	1	65.637	KV452713.1	6081742	1-scf00011	1
AX-157906077	1	65.637	KV452713.1	6082453	1-scf00011	1
AX-157906079	1	65.637	KV452713.1	6083673	1-scf00011	1

AX-157906084	1	65.637	KV452713.1	6091154	1-scf00011	1
AX-157906086	1	65.637	KV452713.1	6092197	1-scf00011	1
AX-157906088	1	65.637	KV452713.1	6095734	1-scf00011	1
AX-157906100	1	65.637	KV452713.1	6110015	1-scf00011	1
AX-157905885	1	65.816	KV452713.1	5821900	1-scf00011	1
AX-157905904	1	65.816	KV452713.1	5848758	1-scf00011	1
AX-157905953	1	65.816	KV452713.1	5904560	1-scf00011	1
AX-157905956	1	65.816	KV452713.1	5907724	1-scf00011	1
AX-158379180	1	65.816	KV452713.1	5986829	1-scf00011	1
AX-157906090	1	65.816	KV452713.1	6098839	1-scf00011	1
AX-157906092	1	65.816	KV452713.1	6101039	1-scf00011	1
AX-157906094	1	65.816	KV452713.1	6102650	1-scf00011	1
AX-157906095	1	65.816	KV452713.1	6104299	1-scf00011	1
AX-157906096	1	65.816	KV452713.1	6106755	1-scf00011	1
AX-157906101	1	65.816	KV452713.1	6110665	1-scf00011	1
AX-157906102	1	65.816	KV452713.1	6111444	1-scf00011	1
AX-157906103	1	65.816	KV452713.1	6111856	1-scf00011	1
AX-157906105	1	65.816	KV452713.1	6112969	1-scf00011	1
AX-157906106	1	65.816	KV452713.1	6114972	1-scf00011	1
AX-157906107	1	65.816	KV452713.1	6115678	1-scf00011	1
AX-157906108	1	65.816	KV452713.1	6116726	1-scf00011	1
AX-157906109	1	65.816	KV452713.1	6117172	1-scf00011	1
AX-157906110	1	65.816	KV452713.1	6119944	1-scf00011	1
AX-158379181	1	65.907	KV452713.1	6127241	1-scf00011	1
AX-157906091	1	65.995	KV452713.1	6100491	1-scf00011	1
AX-157906115	1	65.995	KV452713.1	6128616	1-scf00011	1
AX-157906119	1	65.995	KV452713.1	6133864	1-scf00011	1
AX-157906145	1	65.995	KV452713.1	6164729	1-scf00011	1
AX-157906152	1	65.995	KV452713.1	6175783	1-scf00011	1
AX-157883326	1	66.061	KV452713.1	5018229	1-scf00011	1
AX-157905367	1	66.061	KV452713.1	5056202	1-scf00011	1
AX-157905397	1	66.061	KV452713.1	5101341	1-scf00011	1
AX-157905487	1	66.061	KV452713.1	5246572	1-scf00011	1
AX-157905593	1	66.061	KV452713.1	5406417	1-scf00011	1
AX-157905686	1	66.061	KV452713.1	5524143	1-scf00011	1
AX-157905742	1	66.061	KV452713.1	5616152	1-scf00011	1
AX-157905909	1	66.061	KV452713.1	5852137	1-scf00011	1
AX-157744511	1	66.061	KV452713.1	6026746	1-scf00011	1
AX-157709067	1	66.061	KV452713.1	6085794	1-scf00011	1
AX-157906097	1	66.061	KV452713.1	6107431	1-scf00011	1
AX-157906099	1	66.061	KV452713.1	6109369	1-scf00011	1
AX-157906117	1	66.061	KV452713.1	6130190	1-scf00011	1
AX-157906130	1	66.061	KV452713.1	6144941	1-scf00011	1
AX-157906121	1	66.109	KV452713.1	6136425	1-scf00011	1
AX-157906081	1	66.174	KV452713.1	6085017	1-scf00011	1
AX-157699014	1	66.174	KV452713.1	6137801	1-scf00011	1
AX-157906124	1	66.174	KV452713.1	6138596	1-scf00011	1
AX-157906125	1	66.174	KV452713.1	6139205	1-scf00011	1
AX-157906126	1	66.174	KV452713.1	6140164	1-scf00011	1
AX-157906127	1	66.174	KV452713.1	6141729	1-scf00011	1
AX-157906128	1	66.174	KV452713.1	6142138	1-scf00011	1

AX-157906131	1	66.174	KV452713.1	6145610	1-scf00011	1
AX-157906137	1	66.353	KV452713.1	6150601	1-scf00011	1
AX-157906139	1	66.353	KV452713.1	6153248	1-scf00011	1
AX-157906140	1	66.353	KV452713.1	6154221	1-scf00011	1
AX-157739044	1	66.353	KV452713.1	6155632	1-scf00011	1
AX-157906144	1	66.532	KV452713.1	6162854	1-scf00011	1
AX-157906147	1	66.532	KV452713.1	6166078	1-scf00011	1
AX-157906158	1	66.532	KV452713.1	6185491	1-scf00011	1
AX-157756797	1	66.532	KV452713.1	6186937	1-scf00011	1
AX-157906162	1	66.711	KV452713.1	6191825	1-scf00011	1
AX-157906164	1	66.711	KV452713.1	6193963	1-scf00011	1
AX-157906165	1	66.711	KV452713.1	6195180	1-scf00011	1
AX-157906166	1	66.711	KV452713.1	6196061	1-scf00011	1
AX-157906142	1	66.89	KV452713.1	6156584	1-scf00011	1
AX-157906151	1	66.89	KV452713.1	6175038	1-scf00011	1
AX-157721681	1	66.89	KV452713.1	6190985	1-scf00011	1
AX-157906168	1	66.89	KV452713.1	6199273	1-scf00011	1
AX-157906170	1	66.89	KV452713.1	6200718	1-scf00011	1
AX-157906171	1	66.89	KV452713.1	6201292	1-scf00011	1
AX-157750105	1	66.89	KV452713.1	6203708	1-scf00011	1
AX-157906173	1	66.89	KV452713.1	6204569	1-scf00011	1
AX-157906180	1	66.89	KV452713.1	6213469	1-scf00011	1
AX-158382082	1	66.89	KV452713.1	6218403	1-scf00011	1
AX-157906182	1	66.89	KV452713.1	6218566	1-scf00011	1
AX-157906184	1	66.89	KV452713.1	6219626	1-scf00011	1
AX-157906190	1	66.89	KV452713.1	6226680	1-scf00011	1
AX-157906191	1	66.89	KV452713.1	6229178	1-scf00011	1
AX-157906194	1	66.89	KV452713.1	6232712	1-scf00011	1
AX-157811951	1	66.89	KV452713.1	6235852	1-scf00011	1
AX-157906198	1	66.89	KV452713.1	6237413	1-scf00011	1
AX-157906199	1	66.89	KV452713.1	6238632	1-scf00011	1
AX-157906202	1	66.89	KV452713.1	6242294	1-scf00011	1
AX-157906204	1	66.89	KV452713.1	6245052	1-scf00011	1
AX-157906206	1	66.89	KV452713.1	6248943	1-scf00011	1
AX-157906207	1	66.89	KV452713.1	6250648	1-scf00011	1
AX-157906208	1	66.89	KV452713.1	6250987	1-scf00011	1
AX-157906210	1	66.89	KV452713.1	6253362	1-scf00011	1
AX-157906212	1	66.89	KV452713.1	6255082	1-scf00011	1
AX-157906220	1	66.89	KV452713.1	6266572	1-scf00011	1
AX-157906222	1	66.89	KV452713.1	6268462	1-scf00011	1
AX-157906224	1	66.89	KV452713.1	6269527	1-scf00011	1
AX-157906225	1	66.89	KV452713.1	6271337	1-scf00011	1
AX-157906227	1	66.89	KV452713.1	6274511	1-scf00011	1
AX-157906237	1	66.89	KV452713.1	6284643	1-scf00011	1
AX-157906238	1	66.89	KV452713.1	6287466	1-scf00011	1
AX-157906239	1	66.89	KV452713.1	6288301	1-scf00011	1
AX-157758032	1	66.89	KV452713.1	6288694	1-scf00011	1
AX-157906241	1	66.89	KV452713.1	6289686	1-scf00011	1
AX-158382084	1	66.89	KV452713.1	6290070	1-scf00011	1
AX-157906242	1	66.89	KV452713.1	6291715	1-scf00011	1
AX-157906244	1	66.89	KV452713.1	6292974	1-scf00011	1

AX-157906246	1	66.89	KV452713.1	6294282	1-scf00011	1
AX-157906257	1	66.89	KV452713.1	6312656	1-scf00011	1
AX-157906271	1	66.89	KV452713.1	6328504	1-scf00011	1
AX-157906616	1	66.89	KV452713.1	6917782	1-scf00011	1
AX-157726279	1	66.89	KV452713.1	6919710	1-scf00011	1
AX-157906247	1	66.974	KV452713.1	6294871	1-scf00011	1
AX-157906167	1	67.069	KV452713.1	6197401	1-scf00011	1
AX-157906175	1	67.069	KV452713.1	6207602	1-scf00011	1
AX-157906187	1	67.069	KV452713.1	6222043	1-scf00011	1
AX-157906189	1	67.069	KV452713.1	6223598	1-scf00011	1
AX-157906223	1	67.069	KV452713.1	6269015	1-scf00011	1
AX-157906232	1	67.069	KV452713.1	6280701	1-scf00011	1
AX-157906250	1	67.069	KV452713.1	6301019	1-scf00011	1
AX-157906252	1	67.069	KV452713.1	6303248	1-scf00011	1
AX-157906253	1	67.069	KV452713.1	6304468	1-scf00011	1
AX-157906255	1	67.069	KV452713.1	6310836	1-scf00011	1
AX-157906256	1	67.069	KV452713.1	6311658	1-scf00011	1
AX-157906258	1	67.069	KV452713.1	6313526	1-scf00011	1
AX-157906261	1	67.069	KV452713.1	6317402	1-scf00011	1
AX-157906264	1	67.069	KV452713.1	6319871	1-scf00011	1
AX-157906266	1	67.069	KV452713.1	6321989	1-scf00011	1
AX-157906267	1	67.069	KV452713.1	6323585	1-scf00011	1
AX-157906269	1	67.069	KV452713.1	6327280	1-scf00011	1
AX-157906272	1	67.069	KV452713.1	6328757	1-scf00011	1
AX-157906273	1	67.069	KV452713.1	6335308	1-scf00011	1
AX-157906281	1	67.069	KV452713.1	6343078	1-scf00011	1
AX-157811952	1	67.15	KV452713.1	6335393	1-scf00011	1
AX-157906275	1	67.249	KV452713.1	6337058	1-scf00011	1
AX-157906277	1	67.249	KV452713.1	6338915	1-scf00011	1
AX-157906279	1	67.249	KV452713.1	6340776	1-scf00011	1
AX-157906280	1	67.249	KV452713.1	6342073	1-scf00011	1
AX-157906282	1	67.249	KV452713.1	6344152	1-scf00011	1
AX-157906285	1	67.249	KV452713.1	6346056	1-scf00011	1
AX-157883330	1	67.249	KV452713.1	6350648	1-scf00011	1
AX-157906274	1	67.258	KV452713.1	6336608	1-scf00011	1
AX-157906287	1	67.258	KV452713.1	6347642	1-scf00011	1
AX-157811954	1	67.258	KV452713.1	6353980	1-scf00011	1
AX-157906291	1	67.259	KV452713.1	6350443	1-scf00011	1
AX-157906293	1	67.609	KV452713.1	6360565	1-scf00011	1
AX-157906295	1	67.609	KV452713.1	6361799	1-scf00011	1
AX-157906296	1	67.609	KV452713.1	6363778	1-scf00011	1
AX-157906297	1	67.609	KV452713.1	6364064	1-scf00011	1
AX-157906298	1	67.609	KV452713.1	6365956	1-scf00011	1
AX-157905709	1	67.823	KV452713.1	5560856	1-scf00011	1
AX-157905728	1	67.823	KV452713.1	5595668	1-scf00011	1
AX-157905759	1	67.823	KV452713.1	5644140	1-scf00011	1
AX-157905774	1	67.823	KV452713.1	5671060	1-scf00011	1
AX-157772094	1	67.823	KV452713.1	6202608	1-scf00011	1
AX-157811953	1	67.823	KV452713.1	6353873	1-scf00011	1
AX-157752153	1	67.823	KV452713.1	6372379	1-scf00011	1
AX-157906574	1	67.823	KV452713.1	6849583	1-scf00011	1

AX-157906588	1	67.823	KV452713.1	6877607	1-scf00011	1
AX-157874469	1	67.823	KV452713.1	7026393	1-scf00011	1
AX-157906697	1	67.823	KV452713.1	7031101	1-scf00011	1
AX-157751560	1	67.967	KV452713.1	6378034	1-scf00011	1
AX-157906305	1	67.969	KV452713.1	6378649	1-scf00011	1
AX-158404760	1	67.969	KV452713.1	6379885	1-scf00011	1
AX-157906306	1	67.97	KV452713.1	6381197	1-scf00011	1
AX-157906307	1	67.971	KV452713.1	6384738	1-scf00011	1
AX-157906308	1	68.322	KV452713.1	6388029	1-scf00011	1
AX-157906309	1	68.324	KV452713.1	6388586	1-scf00011	1
AX-157905614	1	68.326	KV452713.1	5435816	1-scf00011	1
AX-157906310	1	68.326	KV452713.1	6391152	1-scf00011	1
AX-157739655	1	68.326	KV452713.1	6392374	1-scf00011	1
AX-158382087	1	68.326	KV452713.1	6393293	1-scf00011	1
AX-157758620	1	68.326	KV452713.1	6393471	1-scf00011	1
AX-157906322	1	68.328	KV452713.1	6407709	1-scf00011	1
AX-157906317	1	68.681	KV452713.1	6401149	1-scf00011	1
AX-157906326	1	68.681	KV452713.1	6413869	1-scf00011	1
AX-157906213	1	68.682	KV452713.1	6260517	1-scf00011	1
AX-157906327	1	68.682	KV452713.1	6414719	1-scf00011	1
AX-157906349	1	68.682	KV452713.1	6440584	1-scf00011	1
AX-157906356	1	68.682	KV452713.1	6448252	1-scf00011	1
AX-157906363	1	68.682	KV452713.1	6461704	1-scf00011	1
AX-157906366	1	68.682	KV452713.1	6464845	1-scf00011	1
AX-157906325	1	68.684	KV452713.1	6412287	1-scf00011	1
AX-157906335	1	68.684	KV452713.1	6424621	1-scf00011	1
AX-157906336	1	68.684	KV452713.1	6425202	1-scf00011	1
AX-157906339	1	68.684	KV452713.1	6427229	1-scf00011	1
AX-157906340	1	68.684	KV452713.1	6429721	1-scf00011	1
AX-157883334	1	68.684	KV452713.1	6432300	1-scf00011	1
AX-157906342	1	68.684	KV452713.1	6433088	1-scf00011	1
AX-157906346	1	68.684	KV452713.1	6438275	1-scf00011	1
AX-157906347	1	68.776	KV452713.1	6439397	1-scf00011	1
AX-157811956	1	68.863	KV452713.1	6397840	1-scf00011	1
AX-157724049	1	68.863	KV452713.1	6441282	1-scf00011	1
AX-157906352	1	68.863	KV452713.1	6443674	1-scf00011	1
AX-157906353	1	68.863	KV452713.1	6445227	1-scf00011	1
AX-157906354	1	68.863	KV452713.1	6446112	1-scf00011	1
AX-157906365	1	68.863	KV452713.1	6464282	1-scf00011	1
AX-157906355	1	68.95	KV452713.1	6447809	1-scf00011	1
AX-157906358	1	69.042	KV452713.1	6453066	1-scf00011	1
AX-157906315	1	69.047	KV452713.1	6398437	1-scf00011	1
AX-157906348	1	69.047	KV452713.1	6439982	1-scf00011	1
AX-157906362	1	69.047	KV452713.1	6460783	1-scf00011	1
AX-157811959	1	69.572	KV452713.1	6474525	1-scf00011	1
AX-157906422	1	69.572	KV452713.1	6540873	1-scf00011	1
AX-157906429	1	69.572	KV452713.1	6551636	1-scf00011	1
AX-157906437	1	69.572	KV452713.1	6567099	1-scf00011	1
AX-157906357	1	69.58	KV452713.1	6452169	1-scf00011	1
AX-157906378	1	69.58	KV452713.1	6487084	1-scf00011	1
AX-157906379	1	69.58	KV452713.1	6487398	1-scf00011	1

AX-157906386	1	69.58	KV452713.1	6494873	1-scf00011	1
AX-157906389	1	69.58	KV452713.1	6498585	1-scf00011	1
AX-157906391	1	69.58	KV452713.1	6499556	1-scf00011	1
AX-157906394	1	69.58	KV452713.1	6502145	1-scf00011	1
AX-157906800	1	69.58	KV452713.1	7159374	1-scf00011	1
AX-157906376	1	69.668	KV452713.1	6484919	1-scf00011	1
AX-157906395	1	69.668	KV452713.1	6502539	1-scf00011	1
AX-157906132	1	69.759	KV452713.1	6146249	1-scf00011	1
AX-157906369	1	69.759	KV452713.1	6471477	1-scf00011	1
AX-157906370	1	69.759	KV452713.1	6473807	1-scf00011	1
AX-157906380	1	69.759	KV452713.1	6488478	1-scf00011	1
AX-157906400	1	69.759	KV452713.1	6508120	1-scf00011	1
AX-157906403	1	69.759	KV452713.1	6511187	1-scf00011	1
AX-157906405	1	69.759	KV452713.1	6514828	1-scf00011	1
AX-157906408	1	69.759	KV452713.1	6518123	1-scf00011	1
AX-157906410	1	69.759	KV452713.1	6520906	1-scf00011	1
AX-157906415	1	69.759	KV452713.1	6529447	1-scf00011	1
AX-157719326	1	69.759	KV452713.1	6533114	1-scf00011	1
AX-157906418	1	69.759	KV452713.1	6536215	1-scf00011	1
AX-157906419	1	69.759	KV452713.1	6537101	1-scf00011	1
AX-157906421	1	69.759	KV452713.1	6539731	1-scf00011	1
AX-157906423	1	69.759	KV452713.1	6541389	1-scf00011	1
AX-157906427	1	69.759	KV452713.1	6549252	1-scf00011	1
AX-157906430	1	69.759	KV452713.1	6552126	1-scf00011	1
AX-157906432	1	69.759	KV452713.1	6553740	1-scf00011	1
AX-157906436	1	69.759	KV452713.1	6563362	1-scf00011	1
AX-157906439	1	69.759	KV452713.1	6570750	1-scf00011	1
AX-157906440	1	69.759	KV452713.1	6571802	1-scf00011	1
AX-157906707	1	69.759	KV452713.1	7037811	1-scf00011	1
AX-158389514	1	69.938	KV452713.1	6523002	1-scf00011	1
AX-157906425	1	69.938	KV452713.1	6543215	1-scf00011	1
AX-157906428	1	69.938	KV452713.1	6551084	1-scf00011	1
AX-157742740	1	69.938	KV452713.1	6560366	1-scf00011	1
AX-157906443	1	69.938	KV452713.1	6579112	1-scf00011	1
AX-157906444	1	69.938	KV452713.1	6580328	1-scf00011	1
AX-157906446	1	69.938	KV452713.1	6582832	1-scf00011	1
AX-157906448	1	69.938	KV452713.1	6585065	1-scf00011	1
AX-157751506	1	69.938	KV452713.1	6595614	1-scf00011	1
AX-157906451	1	69.938	KV452713.1	6597428	1-scf00011	1
AX-157906452	1	69.938	KV452713.1	6598211	1-scf00011	1
AX-157906454	1	69.938	KV452713.1	6600612	1-scf00011	1
AX-157906459	1	69.938	KV452713.1	6611757	1-scf00011	1
AX-157906460	1	69.938	KV452713.1	6612134	1-scf00011	1
AX-157906462	1	69.938	KV452713.1	6613331	1-scf00011	1
AX-157906464	1	69.938	KV452713.1	6614790	1-scf00011	1
AX-157906465	1	69.938	KV452713.1	6615699	1-scf00011	1
AX-157811967	1	69.938	KV452713.1	6778933	1-scf00011	1
AX-157906553	1	69.938	KV452713.1	6785409	1-scf00011	1
AX-157906324	1	69.992	KV452713.1	6409876	1-scf00011	1
AX-157811957	1	69.992	KV452713.1	6432525	1-scf00011	1
AX-157906466	1	69.992	KV452713.1	6619407	1-scf00011	1

AX-157906548	1	69.992	KV452713.1	6776963	1-scf00011	1
AX-157906467	1	70.047	KV452713.1	6628115	1-scf00011	1
AX-157906480	1	70.063	KV452713.1	6641547	1-scf00011	1
AX-157906148	1	70.117	KV452713.1	6167772	1-scf00011	1
AX-157906300	1	70.117	KV452713.1	6366668	1-scf00011	1
AX-157724048	1	70.117	KV452713.1	6385713	1-scf00011	1
AX-157906411	1	70.117	KV452713.1	6522910	1-scf00011	1
AX-157906469	1	70.117	KV452713.1	6629660	1-scf00011	1
AX-157906471	1	70.117	KV452713.1	6633431	1-scf00011	1
AX-157906475	1	70.117	KV452713.1	6635905	1-scf00011	1
AX-157906483	1	70.117	KV452713.1	6644214	1-scf00011	1
AX-157874459	1	70.117	KV452713.1	6656762	1-scf00011	1
AX-157906490	1	70.117	KV452713.1	6660591	1-scf00011	1
AX-157906491	1	70.117	KV452713.1	6661098	1-scf00011	1
AX-157906492	1	70.117	KV452713.1	6662498	1-scf00011	1
AX-157906493	1	70.117	KV452713.1	6662853	1-scf00011	1
AX-157906494	1	70.117	KV452713.1	6663809	1-scf00011	1
AX-157906495	1	70.117	KV452713.1	6664841	1-scf00011	1
AX-157906496	1	70.117	KV452713.1	6665707	1-scf00011	1
AX-157906500	1	70.117	KV452713.1	6672973	1-scf00011	1
AX-157906504	1	70.117	KV452713.1	6681578	1-scf00011	1
AX-157906505	1	70.117	KV452713.1	6687699	1-scf00011	1
AX-158389517	1	70.117	KV452713.1	6689339	1-scf00011	1
AX-157906508	1	70.117	KV452713.1	6693122	1-scf00011	1
AX-157906514	1	70.117	KV452713.1	6704687	1-scf00011	1
AX-158379185	1	70.117	KV452713.1	6704844	1-scf00011	1
AX-157906515	1	70.117	KV452713.1	6705334	1-scf00011	1
AX-157906517	1	70.117	KV452713.1	6707964	1-scf00011	1
AX-157906519	1	70.117	KV452713.1	6710338	1-scf00011	1
AX-157906520	1	70.117	KV452713.1	6712929	1-scf00011	1
AX-157906525	1	70.117	KV452713.1	6722714	1-scf00011	1
AX-157906526	1	70.117	KV452713.1	6724230	1-scf00011	1
AX-157906528	1	70.117	KV452713.1	6730723	1-scf00011	1
AX-157906529	1	70.117	KV452713.1	6732176	1-scf00011	1
AX-157906531	1	70.117	KV452713.1	6735224	1-scf00011	1
AX-157906532	1	70.117	KV452713.1	6738455	1-scf00011	1
AX-157906533	1	70.117	KV452713.1	6739670	1-scf00011	1
AX-157811964	1	70.117	KV452713.1	6753940	1-scf00011	1
AX-157906537	1	70.117	KV452713.1	6754986	1-scf00011	1
AX-157906538	1	70.117	KV452713.1	6755335	1-scf00011	1
AX-157906541	1	70.117	KV452713.1	6758964	1-scf00011	1
AX-157906542	1	70.117	KV452713.1	6759504	1-scf00011	1
AX-157906547	1	70.117	KV452713.1	6768396	1-scf00011	1
AX-157906550	1	70.117	KV452713.1	6777735	1-scf00011	1
AX-157906587	1	70.117	KV452713.1	6875409	1-scf00011	1
AX-157906603	1	70.117	KV452713.1	6898332	1-scf00011	1
AX-157906612	1	70.117	KV452713.1	6910449	1-scf00011	1
AX-157770940	1	70.117	KV452713.1	6912707	1-scf00011	1
AX-157906614	1	70.117	KV452713.1	6914681	1-scf00011	1
AX-157906620	1	70.117	KV452713.1	6926153	1-scf00011	1
AX-157753110	1	70.117	KV452713.1	6927202	1-scf00011	1

AX-157906623	1	70.117	KV452713.1	6928120	1-scf00011	1
AX-157906625	1	70.117	KV452713.1	6929043	1-scf00011	1
AX-157906626	1	70.117	KV452713.1	6929542	1-scf00011	1
AX-157906628	1	70.117	KV452713.1	6930487	1-scf00011	1
AX-157906629	1	70.117	KV452713.1	6931190	1-scf00011	1
AX-157906630	1	70.117	KV452713.1	6936559	1-scf00011	1
AX-157906631	1	70.117	KV452713.1	6937648	1-scf00011	1
AX-157906632	1	70.117	KV452713.1	6938492	1-scf00011	1
AX-157709758	1	70.117	KV452713.1	6939300	1-scf00011	1
AX-157906633	1	70.117	KV452713.1	6939875	1-scf00011	1
AX-157906634	1	70.117	KV452713.1	6940139	1-scf00011	1
AX-157906636	1	70.117	KV452713.1	6941848	1-scf00011	1
AX-157906637	1	70.117	KV452713.1	6942430	1-scf00011	1
AX-157906640	1	70.117	KV452713.1	6950785	1-scf00011	1
AX-158382100	1	70.117	KV452713.1	6952651	1-scf00011	1
AX-157906641	1	70.117	KV452713.1	6954105	1-scf00011	1
AX-157906643	1	70.117	KV452713.1	6957194	1-scf00011	1
AX-157906651	1	70.117	KV452713.1	6972008	1-scf00011	1
AX-157753038	1	70.117	KV452713.1	6975935	1-scf00011	1
AX-157811969	1	70.117	KV452713.1	6986981	1-scf00011	1
AX-157906678	1	70.117	KV452713.1	6999481	1-scf00011	1
AX-157906679	1	70.117	KV452713.1	7001111	1-scf00011	1
AX-157906681	1	70.117	KV452713.1	7008500	1-scf00011	1
AX-157906682	1	70.117	KV452713.1	7008860	1-scf00011	1
AX-157719872	1	70.117	KV452713.1	7014476	1-scf00011	1
AX-157811970	1	70.117	KV452713.1	7019185	1-scf00011	1
AX-157906693	1	70.117	KV452713.1	7024775	1-scf00011	1
AX-157906706	1	70.117	KV452713.1	7037176	1-scf00011	1
AX-157906714	1	70.117	KV452713.1	7051870	1-scf00011	1
AX-157906717	1	70.117	KV452713.1	7056443	1-scf00011	1
AX-157906719	1	70.117	KV452713.1	7058931	1-scf00011	1
AX-157906720	1	70.117	KV452713.1	7060456	1-scf00011	1
AX-157906721	1	70.117	KV452713.1	7061500	1-scf00011	1
AX-157906722	1	70.117	KV452713.1	7061878	1-scf00011	1
AX-157906730	1	70.117	KV452713.1	7072300	1-scf00011	1
AX-157906750	1	70.117	KV452713.1	7105437	1-scf00011	1
AX-157906798	1	70.117	KV452713.1	7157785	1-scf00011	1
AX-157906851	1	70.117	KV452713.1	7224061	1-scf00011	1
AX-157906409	1	70.12	KV452713.1	6520275	1-scf00011	1
AX-157906726	1	70.12	KV452713.1	7067308	1-scf00011	1
AX-157906396	1	70.471	KV452713.1	6506099	1-scf00011	1
AX-157906735	1	70.471	KV452713.1	7081488	1-scf00011	1
AX-157906736	1	70.472	KV452713.1	7083045	1-scf00011	1
AX-157906417	1	70.473	KV452713.1	6531138	1-scf00011	1
AX-157906739	1	70.473	KV452713.1	7086084	1-scf00011	1
AX-157724777	1	70.474	KV452713.1	7092126	1-scf00011	1
AX-157906744	1	70.475	KV452713.1	7094081	1-scf00011	1
AX-157906746	1	70.475	KV452713.1	7099780	1-scf00011	1
AX-157906747	1	70.475	KV452713.1	7100853	1-scf00011	1
AX-157906710	1	70.573	KV452713.1	7046156	1-scf00011	1
AX-157906732	1	70.573	KV452713.1	7074180	1-scf00011	1

AX-157906749	1	70.573	KV452713.1	7104350	1-scf00011	1
AX-157721703	1	70.573	KV452713.1	7120035	1-scf00011	1
AX-157906764	1	70.573	KV452713.1	7122188	1-scf00011	1
AX-157906768	1	70.573	KV452713.1	7126505	1-scf00011	1
AX-157906816	1	70.573	KV452713.1	7176439	1-scf00011	1
AX-157853621	1	70.573	KV452713.1	7202172	1-scf00011	1
AX-157906861	1	70.573	KV452713.1	7238870	1-scf00011	1
AX-157906756	1	70.654	KV452713.1	7110950	1-scf00011	1
AX-157906759	1	70.654	KV452713.1	7114969	1-scf00011	1
AX-157906760	1	70.654	KV452713.1	7116694	1-scf00011	1
AX-157906767	1	70.654	KV452713.1	7126034	1-scf00011	1
AX-157906769	1	70.654	KV452713.1	7127214	1-scf00011	1
AX-157906774	1	70.654	KV452713.1	7131542	1-scf00011	1
AX-157906779	1	70.701	KV452713.1	7136351	1-scf00011	1
AX-157906901	1	70.701	KV452713.1	7306042	1-scf00011	1
AX-157906780	1	70.749	KV452713.1	7137671	1-scf00011	1
AX-157906463	1	70.792	KV452713.1	6613947	1-scf00011	1
AX-157906777	1	70.792	KV452713.1	7134572	1-scf00011	1
AX-157906782	1	70.792	KV452713.1	7139113	1-scf00011	1
AX-157906865	1	70.792	KV452713.1	7249246	1-scf00011	1
AX-157906304	1	70.833	KV452713.1	6376769	1-scf00011	1
AX-157906372	1	70.833	KV452713.1	6476614	1-scf00011	1
AX-157906373	1	70.833	KV452713.1	6478173	1-scf00011	1
AX-157728211	1	70.833	KV452713.1	6621821	1-scf00011	1
AX-157906530	1	70.833	KV452713.1	6734574	1-scf00011	1
AX-157906552	1	70.833	KV452713.1	6782374	1-scf00011	1
AX-157906602	1	70.833	KV452713.1	6897463	1-scf00011	1
AX-157906696	1	70.833	KV452713.1	7029594	1-scf00011	1
AX-157906729	1	70.833	KV452713.1	7070692	1-scf00011	1
AX-157906734	1	70.833	KV452713.1	7079816	1-scf00011	1
AX-157906743	1	70.833	KV452713.1	7093589	1-scf00011	1
AX-157715705	1	70.833	KV452713.1	7102904	1-scf00011	1
AX-157906770	1	70.833	KV452713.1	7128301	1-scf00011	1
AX-157906786	1	70.833	KV452713.1	7141236	1-scf00011	1
AX-157906795	1	70.833	KV452713.1	7148358	1-scf00011	1
AX-157723172	1	70.833	KV452713.1	7150254	1-scf00011	1
AX-157749441	1	70.833	KV452713.1	7152310	1-scf00011	1
AX-157738537	1	70.833	KV452713.1	7172959	1-scf00011	1
AX-157770633	1	70.833	KV452713.1	7205229	1-scf00011	1
AX-157906858	1	70.833	KV452713.1	7236327	1-scf00011	1
AX-157741897	1	70.833	KV452713.1	7242567	1-scf00011	1
AX-157906864	1	70.833	KV452713.1	7246453	1-scf00011	1
AX-157906869	1	70.833	KV452713.1	7263842	1-scf00011	1
AX-157906874	1	70.833	KV452713.1	7270463	1-scf00011	1
AX-157906761	1	71.019	KV452713.1	7118206	1-scf00011	1
AX-157906796	1	71.019	KV452713.1	7151071	1-scf00011	1
AX-157906805	1	71.019	KV452713.1	7164590	1-scf00011	1
AX-157906806	1	71.191	KV452713.1	7165370	1-scf00011	1
AX-157906809	1	71.191	KV452713.1	7167765	1-scf00011	1
AX-157906811	1	71.191	KV452713.1	7171371	1-scf00011	1
AX-158404066	1	71.191	KV452713.1	7171504	1-scf00011	1

AX-158404768	1	71.191	KV452713.1	7171630	1-scf00011	1
AX-157906812	1	71.193	KV452713.1	7172437	1-scf00011	1
AX-157906815	1	71.548	KV452713.1	7174984	1-scf00011	1
AX-157906783	1	71.55	KV452713.1	7139534	1-scf00011	1
AX-157906797	1	71.55	KV452713.1	7157288	1-scf00011	1
AX-157906821	1	71.55	KV452713.1	7187153	1-scf00011	1
AX-157906824	1	71.55	KV452713.1	7190742	1-scf00011	1
AX-157906826	1	71.55	KV452713.1	7192250	1-scf00011	1
AX-157906835	1	71.55	KV452713.1	7201076	1-scf00011	1
AX-157906839	1	71.55	KV452713.1	7207499	1-scf00011	1
AX-157906840	1	71.55	KV452713.1	7207946	1-scf00011	1
AX-157906844	1	71.55	KV452713.1	7215206	1-scf00011	1
AX-157906847	1	71.55	KV452713.1	7217866	1-scf00011	1
AX-158382104	1	71.55	KV452713.1	7218174	1-scf00011	1
AX-157906848	1	71.55	KV452713.1	7218328	1-scf00011	1
AX-157906875	1	71.55	KV452713.1	7271107	1-scf00011	1
AX-157906946	1	71.55	KV452713.1	7388458	1-scf00011	1
AX-157757752	1	71.601	KV452713.1	7229751	1-scf00011	1
AX-157906813	1	71.664	KV452713.1	7173702	1-scf00011	1
AX-157719191	1	71.664	KV452713.1	7265036	1-scf00011	1
AX-157906882	1	71.664	KV452713.1	7280709	1-scf00011	1
AX-157874464	1	71.729	KV452713.1	6893182	1-scf00011	1
AX-157874465	1	71.729	KV452713.1	6901698	1-scf00011	1
AX-157906753	1	71.729	KV452713.1	7108765	1-scf00011	1
AX-157906754	1	71.729	KV452713.1	7109251	1-scf00011	1
AX-157906823	1	71.729	KV452713.1	7189075	1-scf00011	1
AX-157906828	1	71.729	KV452713.1	7193419	1-scf00011	1
AX-157906831	1	71.729	KV452713.1	7197501	1-scf00011	1
AX-157906833	1	71.729	KV452713.1	7198413	1-scf00011	1
AX-157906838	1	71.729	KV452713.1	7206416	1-scf00011	1
AX-157906842	1	71.729	KV452713.1	7210930	1-scf00011	1
AX-157906845	1	71.729	KV452713.1	7215859	1-scf00011	1
AX-157853623	1	71.729	KV452713.1	7263933	1-scf00011	1
AX-157906871	1	71.729	KV452713.1	7267995	1-scf00011	1
AX-157906872	1	71.729	KV452713.1	7269049	1-scf00011	1
AX-157906876	1	71.729	KV452713.1	7271888	1-scf00011	1
AX-157906877	1	71.729	KV452713.1	7274153	1-scf00011	1
AX-157811974	1	71.729	KV452713.1	7275045	1-scf00011	1
AX-157906879	1	71.729	KV452713.1	7276443	1-scf00011	1
AX-157764040	1	71.729	KV452713.1	7277442	1-scf00011	1
AX-157874472	1	71.729	KV452713.1	7278740	1-scf00011	1
AX-158382106	1	71.729	KV452713.1	7281735	1-scf00011	1
AX-157906887	1	71.729	KV452713.1	7286412	1-scf00011	1
AX-157906889	1	71.729	KV452713.1	7287468	1-scf00011	1
AX-157906891	1	71.729	KV452713.1	7290096	1-scf00011	1
AX-157906935	1	71.729	KV452713.1	7370628	1-scf00011	1
AX-157906853	1	71.73	KV452713.1	7228107	1-scf00011	1
AX-157906905	1	71.73	KV452713.1	7312538	1-scf00011	1
AX-157906906	1	71.73	KV452713.1	7316611	1-scf00011	1
AX-157906447	1	72.087	KV452713.1	6584168	1-scf00011	1
AX-157811973	1	72.087	KV452713.1	7273504	1-scf00011	1

AX-157906884	1	72.087	KV452713.1	7282802	1-scf00011	1
AX-157906892	1	72.087	KV452713.1	7290341	1-scf00011	1
AX-157906897	1	72.087	KV452713.1	7300038	1-scf00011	1
AX-157906907	1	72.087	KV452713.1	7319567	1-scf00011	1
AX-157906910	1	72.087	KV452713.1	7321200	1-scf00011	1
AX-157853624	1	72.087	KV452713.1	7321447	1-scf00011	1
AX-157753642	1	72.087	KV452713.1	7331997	1-scf00011	1
AX-157906913	1	72.087	KV452713.1	7332403	1-scf00011	1
AX-157906915	1	72.087	KV452713.1	7333292	1-scf00011	1
AX-157906924	1	72.087	KV452713.1	7348763	1-scf00011	1
AX-157906925	1	72.087	KV452713.1	7352456	1-scf00011	1
AX-157811975	1	72.087	KV452713.1	7355670	1-scf00011	1
AX-157906927	1	72.087	KV452713.1	7356669	1-scf00011	1
AX-157906928	1	72.087	KV452713.1	7358482	1-scf00011	1
AX-157906929	1	72.087	KV452713.1	7359710	1-scf00011	1
AX-157740149	1	72.087	KV452713.1	7359843	1-scf00011	1
AX-157906930	1	72.087	KV452713.1	7360728	1-scf00011	1
AX-157738522	1	72.087	KV452713.1	7363485	1-scf00011	1
AX-157716298	1	72.087	KV452713.1	7385999	1-scf00011	1
AX-157811979	1	72.087	KV452713.1	7394233	1-scf00011	1
AX-157906939	1	72.266	KV452713.1	7376648	1-scf00011	1
AX-157906941	1	72.266	KV452713.1	7380003	1-scf00011	1
AX-157906949	1	72.266	KV452713.1	7395821	1-scf00011	1
AX-157883341	1	72.266	KV452713.1	7409622	1-scf00011	1
AX-157906911	1	72.267	KV452713.1	7327297	1-scf00011	1
AX-157906933	1	72.267	KV452713.1	7366711	1-scf00011	1
AX-157743557	1	72.267	KV452713.1	7389688	1-scf00011	1
AX-157703916	1	72.269	KV452713.1	7254437	1-scf00011	1
AX-157906873	1	72.269	KV452713.1	7269454	1-scf00011	1
AX-157906888	1	72.269	KV452713.1	7287117	1-scf00011	1
AX-157906940	1	72.269	KV452713.1	7377883	1-scf00011	1
AX-157906951	1	72.591	KV452713.1	7398892	1-scf00011	1
AX-157906898	1	72.657	KV452713.1	7301159	1-scf00011	1
AX-157744315	1	72.657	KV452713.1	7400070	1-scf00011	1
AX-157811980	1	72.658	KV452713.1	7405785	1-scf00011	1
AX-157906956	1	72.983	KV452713.1	7410044	1-scf00011	1
AX-157906958	1	72.983	KV452713.1	7412873	1-scf00011	1
AX-157906961	1	72.983	KV452713.1	7415214	1-scf00011	1
AX-157906962	1	72.984	KV452713.1	7421007	1-scf00011	1
AX-157906950	1	73.148	KV452713.1	7397215	1-scf00011	1
AX-157750018	1	73.148	KV452713.1	7421971	1-scf00011	1
AX-157811984	1	73.148	KV452713.1	7425195	1-scf00011	1
AX-157906964	1	73.341	KV452713.1	7427092	1-scf00011	1
AX-157906908	1	73.342	KV452713.1	7319991	1-scf00011	1
AX-157906954	1	73.342	KV452713.1	7408788	1-scf00011	1
AX-157750988	1	73.342	KV452713.1	7418045	1-scf00011	1
AX-157906965	1	73.342	KV452713.1	7428480	1-scf00011	1
AX-157906966	1	73.342	KV452713.1	7432252	1-scf00011	1
AX-157811988	1	73.342	KV452713.1	7434332	1-scf00011	1
AX-157811989	1	73.342	KV452713.1	7435311	1-scf00011	1
AX-157906968	1	73.342	KV452713.1	7435515	1-scf00011	1

AX-157739299	1	73.342	KV452713.1	7436401	1-scf00011	1
AX-157744273	1	73.342	KV452713.1	7437538	1-scf00011	1
AX-157906970	1	73.342	KV452713.1	7438202	1-scf00011	1
AX-157906972	1	73.342	KV452713.1	7439803	1-scf00011	1
AX-157853629	1	73.342	KV452713.1	7458128	1-scf00011	1
AX-157906989	1	73.342	KV452713.1	7477320	1-scf00011	1
AX-157773972	1	73.342	KV452713.1	7487760	1-scf00011	1
AX-157907003	1	73.342	KV452713.1	7500164	1-scf00011	1
AX-157713642	1	73.492	KV452713.1	7439202	1-scf00011	1
AX-157906978	1	73.683	KV452713.1	7456610	1-scf00011	1
AX-157906973	1	73.701	KV452713.1	7443060	1-scf00011	1
AX-157906977	1	73.701	KV452713.1	7455224	1-scf00011	1
AX-157906979	1	73.701	KV452713.1	7456893	1-scf00011	1
AX-157906981	1	73.701	KV452713.1	7462392	1-scf00011	1
AX-157906982	1	73.701	KV452713.1	7464443	1-scf00011	1
AX-157906983	1	73.701	KV452713.1	7466189	1-scf00011	1
AX-157906984	1	73.701	KV452713.1	7468392	1-scf00011	1
AX-157717147	1	73.744	KV452713.1	7471330	1-scf00011	1
AX-157906986	1	73.788	KV452713.1	7474301	1-scf00011	1
AX-157906990	1	73.788	KV452713.1	7479932	1-scf00011	1
AX-157906987	1	73.835	KV452713.1	7474913	1-scf00011	1
AX-157906988	1	73.88	KV452713.1	7475370	1-scf00011	1
AX-157906995	1	73.88	KV452713.1	7489241	1-scf00011	1
AX-157906996	1	73.88	KV452713.1	7490193	1-scf00011	1
AX-158404773	1	73.88	KV452713.1	7491271	1-scf00011	1
AX-157907000	1	73.88	KV452713.1	7495996	1-scf00011	1
AX-157907002	1	73.88	KV452713.1	7499167	1-scf00011	1
AX-157907004	1	73.88	KV452713.1	7501548	1-scf00011	1
AX-157907006	1	73.905	KV452713.1	7503115	1-scf00011	1
AX-157907014	1	74.059	KV452713.1	7511713	1-scf00011	1
AX-157907018	1	74.059	KV452713.1	7518236	1-scf00011	1
AX-157906991	1	74.06	KV452713.1	7481110	1-scf00011	1
AX-157907023	1	74.06	KV452713.1	7528118	1-scf00011	1
AX-157907159	1	74.06	KV452713.1	7738308	1-scf00011	1
AX-157907029	1	74.416	KV452713.1	7539152	1-scf00011	1
AX-157907031	1	74.417	KV452713.1	7539885	1-scf00011	1
AX-157907033	1	74.417	KV452713.1	7543726	1-scf00011	1
AX-158404774	1	74.417	KV452713.1	7552454	1-scf00011	1
AX-157907040	1	74.417	KV452713.1	7552540	1-scf00011	1
AX-157907052	1	74.417	KV452713.1	7565325	1-scf00011	1
AX-157907054	1	74.417	KV452713.1	7566786	1-scf00011	1
AX-157907057	1	74.417	KV452713.1	7571400	1-scf00011	1
AX-157907058	1	74.417	KV452713.1	7572046	1-scf00011	1
AX-157907064	1	74.417	KV452713.1	7581476	1-scf00011	1
AX-157907075	1	74.417	KV452713.1	7592466	1-scf00011	1
AX-157907061	1	74.418	KV452713.1	7575080	1-scf00011	1
AX-157723478	1	74.418	KV452713.1	7575903	1-scf00011	1
AX-157907063	1	74.418	KV452713.1	7580529	1-scf00011	1
AX-158404784	1	74.418	KV452713.1	7669529	1-scf00011	1
AX-158404786	1	74.418	KV452713.1	7669770	1-scf00011	1
AX-157907126	1	74.418	KV452713.1	7680341	1-scf00011	1

AX-157907070	1	74.773	KV452713.1	7586575	1-scf00011	1
AX-157907036	1	74.775	KV452713.1	7547556	1-scf00011	1
AX-157907056	1	74.775	KV452713.1	7570795	1-scf00011	1
AX-157907072	1	74.775	KV452713.1	7589677	1-scf00011	1
AX-157907073	1	74.775	KV452713.1	7590531	1-scf00011	1
AX-157907076	1	74.775	KV452713.1	7597385	1-scf00011	1
AX-157907078	1	74.775	KV452713.1	7601565	1-scf00011	1
AX-157907079	1	74.775	KV452713.1	7602185	1-scf00011	1
AX-157907080	1	74.775	KV452713.1	7602982	1-scf00011	1
AX-157907143	1	74.775	KV452713.1	7714962	1-scf00011	1
AX-157907145	1	74.775	KV452713.1	7716309	1-scf00011	1
AX-157907149	1	74.775	KV452713.1	7722887	1-scf00011	1
AX-157907229	1	74.775	KV452713.1	7840844	1-scf00011	1
AX-157907231	1	74.775	KV452713.1	7843937	1-scf00011	1
AX-157907232	1	74.775	KV452713.1	7844385	1-scf00011	1
AX-157907251	1	74.775	KV452713.1	7869090	1-scf00011	1
AX-157906980	1	74.954	KV452713.1	7461719	1-scf00011	1
AX-157906992	1	74.954	KV452713.1	7482454	1-scf00011	1
AX-157740894	1	74.954	KV452713.1	7489840	1-scf00011	1
AX-157906997	1	74.954	KV452713.1	7492665	1-scf00011	1
AX-157907013	1	74.954	KV452713.1	7510105	1-scf00011	1
AX-157869043	1	74.954	KV452713.1	7542711	1-scf00011	1
AX-157907062	1	74.954	KV452713.1	7578444	1-scf00011	1
AX-157907082	1	74.954	KV452713.1	7604469	1-scf00011	1
AX-157757146	1	74.954	KV452713.1	7605633	1-scf00011	1
AX-157907087	1	74.954	KV452713.1	7611586	1-scf00011	1
AX-157907093	1	74.954	KV452713.1	7620002	1-scf00011	1
AX-157907095	1	74.954	KV452713.1	7621738	1-scf00011	1
AX-157907097	1	74.954	KV452713.1	7624126	1-scf00011	1
AX-157907099	1	74.954	KV452713.1	7626656	1-scf00011	1
AX-157907155	1	74.954	KV452713.1	7734845	1-scf00011	1
AX-157907184	1	74.954	KV452713.1	7773316	1-scf00011	1
AX-157907074	1	75.133	KV452713.1	7591468	1-scf00011	1
AX-157907100	1	75.133	KV452713.1	7628045	1-scf00011	1
AX-157907101	1	75.133	KV452713.1	7629649	1-scf00011	1
AX-157722197	1	75.133	KV452713.1	7635194	1-scf00011	1
AX-157907105	1	75.133	KV452713.1	7636712	1-scf00011	1
AX-157907107	1	75.133	KV452713.1	7641625	1-scf00011	1
AX-157907108	1	75.133	KV452713.1	7643327	1-scf00011	1
AX-157811998	1	75.133	KV452713.1	7649886	1-scf00011	1
AX-157907111	1	75.133	KV452713.1	7650295	1-scf00011	1
AX-157907114	1	75.133	KV452713.1	7652245	1-scf00011	1
AX-157907116	1	75.222	KV452713.1	7659329	1-scf00011	1
AX-158404779	1	75.241	KV452713.1	7667808	1-scf00011	1
AX-157811990	1	75.329	KV452713.1	7446792	1-scf00011	1
AX-157907067	1	75.329	KV452713.1	7584970	1-scf00011	1
AX-158404782	1	75.329	KV452713.1	7668716	1-scf00011	1
AX-158404783	1	75.329	KV452713.1	7668886	1-scf00011	1
AX-157811994	1	75.402	KV452713.1	7472742	1-scf00011	1
AX-157874477	1	75.402	KV452713.1	7514895	1-scf00011	1
AX-157907027	1	75.402	KV452713.1	7536130	1-scf00011	1

AX-157907066	1	75.402	KV452713.1	7584430	1-scf00011	1
AX-157907122	1	75.402	KV452713.1	7675015	1-scf00011	1
AX-157907124	1	75.402	KV452713.1	7678696	1-scf00011	1
AX-157907127	1	75.402	KV452713.1	7682294	1-scf00011	1
AX-157907128	1	75.402	KV452713.1	7684461	1-scf00011	1
AX-157907131	1	75.402	KV452713.1	7688321	1-scf00011	1
AX-157907135	1	75.402	KV452713.1	7693119	1-scf00011	1
AX-157907136	1	75.402	KV452713.1	7696354	1-scf00011	1
AX-157907138	1	75.402	KV452713.1	7706782	1-scf00011	1
AX-157907139	1	75.402	KV452713.1	7708684	1-scf00011	1
AX-157874484	1	75.513	KV452713.1	7709468	1-scf00011	1
AX-157907141	1	75.609	KV452713.1	7711270	1-scf00011	1
AX-157907035	1	75.673	KV452713.1	7546513	1-scf00011	1
AX-157907081	1	75.673	KV452713.1	7603877	1-scf00011	1
AX-157907084	1	75.673	KV452713.1	7607520	1-scf00011	1
AX-157907106	1	75.673	KV452713.1	7640701	1-scf00011	1
AX-157907142	1	75.673	KV452713.1	7712809	1-scf00011	1
AX-157907146	1	75.673	KV452713.1	7717636	1-scf00011	1
AX-157907148	1	75.673	KV452713.1	7720619	1-scf00011	1
AX-157907150	1	75.673	KV452713.1	7723566	1-scf00011	1
AX-157907151	1	75.673	KV452713.1	7728102	1-scf00011	1
AX-157907154	1	75.673	KV452713.1	7734221	1-scf00011	1
AX-157907156	1	75.673	KV452713.1	7735214	1-scf00011	1
AX-157907158	1	75.673	KV452713.1	7737493	1-scf00011	1
AX-157706430	1	75.673	3ML01032225	280	scf07841_1	UN
AX-157907015	1	75.814	KV452713.1	7514181	1-scf00011	1
AX-157907115	1	75.814	KV452713.1	7655465	1-scf00011	1
AX-157907121	1	75.814	KV452713.1	7674295	1-scf00011	1
AX-157907125	1	75.814	KV452713.1	7679759	1-scf00011	1
AX-157907161	1	75.814	KV452713.1	7744386	1-scf00011	1
AX-157907162	1	75.814	KV452713.1	7745668	1-scf00011	1
AX-157907167	1	75.814	KV452713.1	7752654	1-scf00011	1
AX-157907176	1	75.814	KV452713.1	7764989	1-scf00011	1
AX-157907190	1	75.814	KV452713.1	7780676	1-scf00011	1
AX-157907068	1	75.942	KV452713.1	7585634	1-scf00011	1
AX-157811999	1	75.942	KV452713.1	7746121	1-scf00011	1
AX-157907168	1	75.942	KV452713.1	7753170	1-scf00011	1
AX-157907178	1	75.942	KV452713.1	7767539	1-scf00011	1
AX-157907179	1	76.075	KV452713.1	7768636	1-scf00011	1
AX-157907181	1	76.211	KV452713.1	7771406	1-scf00011	1
AX-157907182	1	76.211	KV452713.1	7772102	1-scf00011	1
AX-157907188	1	76.211	KV452713.1	7776620	1-scf00011	1
AX-157907191	1	76.211	KV452713.1	7783871	1-scf00011	1
AX-157907445	1	76.211	KV452713.1	8104285	1-scf00011	1
AX-157907163	1	76.256	KV452713.1	7746478	1-scf00011	1
AX-158404788	1	76.256	KV452713.1	7825809	1-scf00011	1
AX-157907262	1	76.256	KV452713.1	7876888	1-scf00011	1
AX-157720836	1	76.313	KV452713.1	7838905	1-scf00011	1
AX-157907185	1	76.39	KV452713.1	7774029	1-scf00011	1
AX-157907222	1	76.39	KV452713.1	7823629	1-scf00011	1
AX-157907225	1	76.39	KV452713.1	7832524	1-scf00011	1

AX-157907228	1	76.39	KV452713.1	7840593	1-scf00011	1
AX-157907230	1	76.39	KV452713.1	7841342	1-scf00011	1
AX-157907233	1	76.39	KV452713.1	7844782	1-scf00011	1
AX-157907234	1	76.39	KV452713.1	7846487	1-scf00011	1
AX-157907235	1	76.39	KV452713.1	7849517	1-scf00011	1
AX-157907236	1	76.39	KV452713.1	7851755	1-scf00011	1
AX-157907238	1	76.39	KV452713.1	7853673	1-scf00011	1
AX-157907242	1	76.39	KV452713.1	7857691	1-scf00011	1
AX-158389522	1	76.39	KV452713.1	7858659	1-scf00011	1
AX-157907245	1	76.39	KV452713.1	7860135	1-scf00011	1
AX-157907246	1	76.39	KV452713.1	7860545	1-scf00011	1
AX-157907255	1	76.39	KV452713.1	7871845	1-scf00011	1
AX-157907256	1	76.39	KV452713.1	7872742	1-scf00011	1
AX-157907260	1	76.39	KV452713.1	7874629	1-scf00011	1
AX-157755882	1	76.39	KV452713.1	7875255	1-scf00011	1
AX-157907259	1	76.651	KV452713.1	7874006	1-scf00011	1
AX-157907266	1	76.929	KV452713.1	7883290	1-scf00011	1
AX-157907267	1	76.929	KV452713.1	7884101	1-scf00011	1
AX-157907270	1	76.929	KV452713.1	7886676	1-scf00011	1
AX-157907271	1	76.929	KV452713.1	7888100	1-scf00011	1
AX-157907273	1	76.929	KV452713.1	7888888	1-scf00011	1
AX-157869044	1	76.929	KV452713.1	7891465	1-scf00011	1
AX-157907277	1	76.929	KV452713.1	7893846	1-scf00011	1
AX-157907274	1	77.015	KV452713.1	7889607	1-scf00011	1
AX-157907280	1	77.015	KV452713.1	7897256	1-scf00011	1
AX-157907278	1	77.037	KV452713.1	7895630	1-scf00011	1
AX-157907279	1	77.108	KV452713.1	7896805	1-scf00011	1
AX-157907281	1	77.108	KV452713.1	7897771	1-scf00011	1
AX-157907282	1	77.108	KV452713.1	7898115	1-scf00011	1
AX-157869045	1	77.108	KV452713.1	7898561	1-scf00011	1
AX-157907283	1	77.108	KV452713.1	7898945	1-scf00011	1
AX-157907285	1	77.108	KV452713.1	7899842	1-scf00011	1
AX-157907286	1	77.108	KV452713.1	7900228	1-scf00011	1
AX-157907289	1	77.108	KV452713.1	7904447	1-scf00011	1
AX-157907291	1	77.108	KV452713.1	7909433	1-scf00011	1
AX-157907292	1	77.108	KV452713.1	7910855	1-scf00011	1
AX-157907293	1	77.108	KV452713.1	7912062	1-scf00011	1
AX-157812003	1	77.108	KV452713.1	7914500	1-scf00011	1
AX-157907294	1	77.108	KV452713.1	7915410	1-scf00011	1
AX-157907295	1	77.108	KV452713.1	7916040	1-scf00011	1
AX-157812004	1	77.108	KV452713.1	7917253	1-scf00011	1
AX-157907297	1	77.108	KV452713.1	7918606	1-scf00011	1
AX-157907298	1	77.108	KV452713.1	7919487	1-scf00011	1
AX-157722722	1	77.108	KV452713.1	7922395	1-scf00011	1
AX-157907300	1	77.108	KV452713.1	7924159	1-scf00011	1
AX-157907301	1	77.108	KV452713.1	7924752	1-scf00011	1
AX-157907302	1	77.108	KV452713.1	7925262	1-scf00011	1
AX-157907304	1	77.108	KV452713.1	7926933	1-scf00011	1
AX-158379191	1	77.108	KV452713.1	7927425	1-scf00011	1
AX-157907305	1	77.108	KV452713.1	7927867	1-scf00011	1
AX-157907306	1	77.108	KV452713.1	7929879	1-scf00011	1

AX-157907308	1	77.108	KV452713.1	7932332	1-scf00011	1
AX-157907309	1	77.108	KV452713.1	7933060	1-scf00011	1
AX-157907312	1	77.108	KV452713.1	7936812	1-scf00011	1
AX-157907313	1	77.108	KV452713.1	7937172	1-scf00011	1
AX-157907314	1	77.108	KV452713.1	7937872	1-scf00011	1
AX-157907316	1	77.108	KV452713.1	7942223	1-scf00011	1
AX-157907317	1	77.108	KV452713.1	7942532	1-scf00011	1
AX-157907318	1	77.108	KV452713.1	7946020	1-scf00011	1
AX-157907324	1	77.108	KV452713.1	7953421	1-scf00011	1
AX-157907325	1	77.108	KV452713.1	7953789	1-scf00011	1
AX-157907334	1	77.108	KV452713.1	7959896	1-scf00011	1
AX-157907338	1	77.108	KV452713.1	7963449	1-scf00011	1
AX-157907345	1	77.108	KV452713.1	7977096	1-scf00011	1
AX-157907354	1	77.108	KV452713.1	7991238	1-scf00011	1
AX-157869047	1	77.287	KV452713.1	7916417	1-scf00011	1
AX-157907320	1	77.287	KV452713.1	7948156	1-scf00011	1
AX-157907321	1	77.287	KV452713.1	7948512	1-scf00011	1
AX-157907323	1	77.287	KV452713.1	7952399	1-scf00011	1
AX-157907326	1	77.287	KV452713.1	7954202	1-scf00011	1
AX-157907328	1	77.287	KV452713.1	7955563	1-scf00011	1
AX-157907329	1	77.287	KV452713.1	7956000	1-scf00011	1
AX-157907333	1	77.287	KV452713.1	7959055	1-scf00011	1
AX-157907337	1	77.287	KV452713.1	7962113	1-scf00011	1
AX-157907340	1	77.287	KV452713.1	7969460	1-scf00011	1
AX-157907341	1	77.287	KV452713.1	7972994	1-scf00011	1
AX-157907342	1	77.287	KV452713.1	7973813	1-scf00011	1
AX-157907343	1	77.287	KV452713.1	7974478	1-scf00011	1
AX-157907346	1	77.287	KV452713.1	7977541	1-scf00011	1
AX-157907351	1	77.287	KV452713.1	7987947	1-scf00011	1
AX-157907352	1	77.287	KV452713.1	7988885	1-scf00011	1
AX-158404790	1	77.287	KV452713.1	7989468	1-scf00011	1
AX-157907361	1	77.287	KV452713.1	7997412	1-scf00011	1
AX-157907355	1	77.292	KV452713.1	7992424	1-scf00011	1
AX-157907360	1	77.597	KV452713.1	7997191	1-scf00011	1
AX-157907364	1	77.89	KV452713.1	8003365	1-scf00011	1
AX-157907374	1	78.184	KV452713.1	8014552	1-scf00011	1
AX-157907380	1	78.184	KV452713.1	8025780	1-scf00011	1
AX-157907375	1	78.188	KV452713.1	8016324	1-scf00011	1
AX-157907376	1	78.188	KV452713.1	8017446	1-scf00011	1
AX-157907379	1	78.188	KV452713.1	8022885	1-scf00011	1
AX-157907382	1	78.276	KV452713.1	8030969	1-scf00011	1
AX-157757060	1	78.289	KV452713.1	8034842	1-scf00011	1
AX-157721770	1	78.367	KV452713.1	8030088	1-scf00011	1
AX-157907386	1	78.367	KV452713.1	8035934	1-scf00011	1
AX-157907387	1	78.367	KV452713.1	8036767	1-scf00011	1
AX-157907388	1	78.367	KV452713.1	8037322	1-scf00011	1
AX-157907389	1	78.367	KV452713.1	8037769	1-scf00011	1
AX-157907391	1	78.367	KV452713.1	8038718	1-scf00011	1
AX-157907392	1	78.367	KV452713.1	8039435	1-scf00011	1
AX-157907394	1	78.367	KV452713.1	8040774	1-scf00011	1
AX-157907395	1	78.367	KV452713.1	8043263	1-scf00011	1

AX-157907397	1	78.367	KV452713.1	8044916	1-scf00011	1
AX-157907400	1	78.367	KV452713.1	8048800	1-scf00011	1
AX-157907402	1	78.367	KV452713.1	8050527	1-scf00011	1
AX-157907390	1	78.546	KV452713.1	8038435	1-scf00011	1
AX-157907404	1	78.546	KV452713.1	8052128	1-scf00011	1
AX-157907406	1	78.546	KV452713.1	8053741	1-scf00011	1
AX-157907407	1	78.726	KV452713.1	8054678	1-scf00011	1
AX-157907409	1	78.817	KV452713.1	8056031	1-scf00011	1
AX-157907408	1	78.905	KV452713.1	8054935	1-scf00011	1
AX-157907416	1	78.905	KV452713.1	8064544	1-scf00011	1
AX-157907420	1	78.905	KV452713.1	8071978	1-scf00011	1
AX-158382131	1	78.905	KV452713.1	8080359	1-scf00011	1
AX-157907414	1	79.084	KV452713.1	8063698	1-scf00011	1
AX-157907421	1	79.084	KV452713.1	8074780	1-scf00011	1
AX-157907422	1	79.084	KV452713.1	8074876	1-scf00011	1
AX-157907423	1	79.084	KV452713.1	8075371	1-scf00011	1
AX-157907426	1	79.084	KV452713.1	8080977	1-scf00011	1
AX-158404075	1	79.084	KV452713.1	8084972	1-scf00011	1
AX-157907429	1	79.084	KV452713.1	8085725	1-scf00011	1
AX-158379192	1	79.084	KV452713.1	8085926	1-scf00011	1
AX-157907430	1	79.084	KV452713.1	8086094	1-scf00011	1
AX-157907431	1	79.084	KV452713.1	8086635	1-scf00011	1
AX-157907432	1	79.084	KV452713.1	8087323	1-scf00011	1
AX-157742909	1	79.084	KV452713.1	8088321	1-scf00011	1
AX-157907433	1	79.084	KV452713.1	8090526	1-scf00011	1
AX-157907434	1	79.084	KV452713.1	8091039	1-scf00011	1
AX-157907435	1	79.084	KV452713.1	8091560	1-scf00011	1
AX-157907437	1	79.084	KV452713.1	8095570	1-scf00011	1
AX-157907438	1	79.084	KV452713.1	8096454	1-scf00011	1
AX-157907439	1	79.084	KV452713.1	8097175	1-scf00011	1
AX-157907440	1	79.084	KV452713.1	8097831	1-scf00011	1
AX-157907441	1	79.084	KV452713.1	8099151	1-scf00011	1
AX-157724857	1	79.084	KV452713.1	8100844	1-scf00011	1
AX-157907443	1	79.084	KV452713.1	8103130	1-scf00011	1
AX-157907444	1	79.084	KV452713.1	8103523	1-scf00011	1
AX-157907462	1	79.084	KV452713.1	8129176	1-scf00011	1
AX-157907469	1	79.084	KV452713.1	8138568	1-scf00011	1
AX-157907405	1	79.265	KV452713.1	8053370	1-scf00011	1
AX-157907417	1	79.265	KV452713.1	8065118	1-scf00011	1
AX-157907448	1	79.265	KV452713.1	8110741	1-scf00011	1
AX-157907450	1	79.265	KV452713.1	8113071	1-scf00011	1
AX-157907452	1	79.265	KV452713.1	8114954	1-scf00011	1
AX-157907456	1	79.265	KV452713.1	8120760	1-scf00011	1
AX-157751998	1	79.265	KV452713.1	8122269	1-scf00011	1
AX-157724596	1	79.265	KV452713.1	8143117	1-scf00011	1
AX-157907480	1	79.265	KV452713.1	8151237	1-scf00011	1
AX-157907496	1	79.265	KV452713.1	8178910	1-scf00011	1
AX-157907458	1	79.337	KV452713.1	8124269	1-scf00011	1
AX-157907427	1	79.353	KV452713.1	8084244	1-scf00011	1
AX-157907459	1	79.353	KV452713.1	8126875	1-scf00011	1
AX-157907467	1	79.353	KV452713.1	8133897	1-scf00011	1

AX-157739050	1	79.353	KV452713.1	8135489	1-scf00011	1
AX-157907454	1	79.446	KV452713.1	8119175	1-scf00011	1
AX-157907468	1	79.446	KV452713.1	8134594	1-scf00011	1
AX-158404794	1	79.446	KV452713.1	8139839	1-scf00011	1
AX-157907470	1	79.446	KV452713.1	8140356	1-scf00011	1
AX-157907476	1	79.446	KV452713.1	8147929	1-scf00011	1
AX-157907477	1	79.446	KV452713.1	8148768	1-scf00011	1
AX-157907473	1	79.626	KV452713.1	8145353	1-scf00011	1
AX-157907483	1	79.626	KV452713.1	8154077	1-scf00011	1
AX-157718445	1	79.626	KV452713.1	8159379	1-scf00011	1
AX-157770999	1	79.626	KV452713.1	8163560	1-scf00011	1
AX-157907492	1	79.626	KV452713.1	8165608	1-scf00011	1
AX-157907502	1	79.626	KV452713.1	8186063	1-scf00011	1
AX-157723811	1	79.634	KV452713.1	8169053	1-scf00011	1
AX-157907494	1	79.642	KV452713.1	8170671	1-scf00011	1
AX-157907498	1	79.651	KV452713.1	8181224	1-scf00011	1
AX-157907500	1	80.169	KV452713.1	8184401	1-scf00011	1
AX-157907504	1	80.169	KV452713.1	8188015	1-scf00011	1
AX-157907507	1	80.17	KV452713.1	8193493	1-scf00011	1
AX-157907503	1	80.351	KV452713.1	8187538	1-scf00011	1
AX-157907510	1	80.351	KV452713.1	8197052	1-scf00011	1
AX-157723369	1	80.351	KV452713.1	8200928	1-scf00011	1
AX-158404799	1	80.351	KV452713.1	8203208	1-scf00011	1
AX-157907514	1	80.351	KV452713.1	8206797	1-scf00011	1
AX-157907518	1	80.351	KV452713.1	8210451	1-scf00011	1
AX-157907520	1	80.351	KV452713.1	8212485	1-scf00011	1
AX-157907521	1	80.351	KV452713.1	8214193	1-scf00011	1
AX-157907522	1	80.351	KV452713.1	8216003	1-scf00011	1
AX-157907523	1	80.351	KV452713.1	8216672	1-scf00011	1
AX-157907528	1	80.351	KV452713.1	8222758	1-scf00011	1
AX-157907532	1	80.351	KV452713.1	8226163	1-scf00011	1
AX-157907549	1	80.351	KV452713.1	8242895	1-scf00011	1
AX-157907428	1	80.531	KV452713.1	8085245	1-scf00011	1
AX-157874494	1	80.531	KV452713.1	8131755	1-scf00011	1
AX-157907466	1	80.531	KV452713.1	8132872	1-scf00011	1
AX-157907472	1	80.531	KV452713.1	8144598	1-scf00011	1
AX-157907474	1	80.531	KV452713.1	8145743	1-scf00011	1
AX-157752041	1	80.531	KV452713.1	8146171	1-scf00011	1
AX-157907479	1	80.531	KV452713.1	8150884	1-scf00011	1
AX-157907481	1	80.531	KV452713.1	8152309	1-scf00011	1
AX-157907485	1	80.531	KV452713.1	8156188	1-scf00011	1
AX-157907505	1	80.531	KV452713.1	8189748	1-scf00011	1
AX-157874495	1	80.531	KV452713.1	8225016	1-scf00011	1
AX-157907534	1	80.531	KV452713.1	8227438	1-scf00011	1
AX-157907536	1	80.531	KV452713.1	8228557	1-scf00011	1
AX-157907537	1	80.531	KV452713.1	8231950	1-scf00011	1
AX-157907539	1	80.531	KV452713.1	8234661	1-scf00011	1
AX-157907540	1	80.531	KV452713.1	8235139	1-scf00011	1
AX-157907542	1	80.531	KV452713.1	8236573	1-scf00011	1
AX-157771227	1	80.531	KV452713.1	8237836	1-scf00011	1
AX-157907544	1	80.531	KV452713.1	8238506	1-scf00011	1

AX-157907546	1	80.531	KV452713.1	8239943	1-scf00011	1
AX-157907547	1	80.531	KV452713.1	8240665	1-scf00011	1
AX-157720493	1	80.531	KV452713.1	8258741	1-scf00011	1
AX-157907548	1	80.632	KV452713.1	8241243	1-scf00011	1
AX-157907550	1	80.712	KV452713.1	8244549	1-scf00011	1
AX-157907551	1	80.712	KV452713.1	8246103	1-scf00011	1
AX-157907552	1	80.734	KV452713.1	8248058	1-scf00011	1
AX-157907564	1	80.734	KV452713.1	8267975	1-scf00011	1
AX-157907553	1	80.761	KV452713.1	8248415	1-scf00011	1
AX-157907554	1	80.795	KV452713.1	8248934	1-scf00011	1
AX-157907555	1	80.809	KV452713.1	8250364	1-scf00011	1
AX-157907557	1	80.843	KV452713.1	8252757	1-scf00011	1
AX-158382134	1	80.869	KV452713.1	8245849	1-scf00011	1
AX-157907559	1	80.869	KV452713.1	8255630	1-scf00011	1
AX-157907561	1	80.893	KV452713.1	8263060	1-scf00011	1
AX-157907562	1	80.893	KV452713.1	8265892	1-scf00011	1
AX-157907563	1	80.893	KV452713.1	8266203	1-scf00011	1
AX-157907567	1	80.895	KV452713.1	8270220	1-scf00011	1
AX-157907568	1	81.253	KV452713.1	8271278	1-scf00011	1
AX-157907569	1	81.255	KV452713.1	8271409	1-scf00011	1
AX-157907572	1	81.992	KV452713.1	8274897	1-scf00011	1
AX-157907573	1	82.339	KV452713.1	8279442	1-scf00011	1
AX-157907575	1	82.346	KV452713.1	8285492	1-scf00011	1
AX-157907576	1	82.347	KV452713.1	8289067	1-scf00011	1
AX-157907587	1	82.347	KV452713.1	8299443	1-scf00011	1
AX-157907590	1	82.355	KV452713.1	8303502	1-scf00011	1
AX-157907591	1	82.527	KV452713.1	8304197	1-scf00011	1
AX-157907595	1	82.527	KV452713.1	8309910	1-scf00011	1
AX-157907597	1	82.569	KV452713.1	8316564	1-scf00011	1
AX-157907596	1	82.637	KV452713.1	8314275	1-scf00011	1
AX-157907599	1	82.637	KV452713.1	8317584	1-scf00011	1
AX-157907601	1	82.637	KV452713.1	8318984	1-scf00011	1
AX-158404801	1	82.637	KV452713.1	8319329	1-scf00011	1
AX-157743070	1	82.637	KV452713.1	8319843	1-scf00011	1
AX-157907603	1	82.637	KV452713.1	8324390	1-scf00011	1
AX-157727463	1	82.637	KV452713.1	8325442	1-scf00011	1
AX-157907605	1	82.637	KV452713.1	8327573	1-scf00011	1
AX-157907606	1	82.637	KV452713.1	8328476	1-scf00011	1
AX-157907607	1	82.637	KV452713.1	8328800	1-scf00011	1
AX-157907608	1	82.637	KV452713.1	8329460	1-scf00011	1
AX-157907609	1	82.637	KV452713.1	8329967	1-scf00011	1
AX-157907610	1	82.637	KV452713.1	8332423	1-scf00011	1
AX-157907611	1	83.001	KV452713.1	8336953	1-scf00011	1
AX-157907614	1	83.063	KV452713.1	8339406	1-scf00011	1
AX-158404800	1	83.12	KV452713.1	8302390	1-scf00011	1
AX-157907612	1	83.12	KV452713.1	8338051	1-scf00011	1
AX-157907615	1	83.12	KV452713.1	8339598	1-scf00011	1
AX-157907616	1	83.12	KV452713.1	8340191	1-scf00011	1
AX-157907629	1	83.12	KV452713.1	8362715	1-scf00011	1
AX-157907545	1	83.183	KV452713.1	8239253	1-scf00011	1
AX-157907558	1	83.183	KV452713.1	8253429	1-scf00011	1

AX-157907618	1	83.183	KV452713.1	8342772	1-scf00011	1
AX-158379194	1	83.183	KV452713.1	8342877	1-scf00011	1
AX-157907619	1	83.183	KV452713.1	8343155	1-scf00011	1
AX-157907621	1	83.183	KV452713.1	8347350	1-scf00011	1
AX-157751872	1	83.183	KV452713.1	8348844	1-scf00011	1
AX-157907623	1	83.183	KV452713.1	8350181	1-scf00011	1
AX-157907624	1	83.183	KV452713.1	8350779	1-scf00011	1
AX-158421842	1	83.183	KV452713.1	8354392	1-scf00011	1
AX-157907626	1	83.183	KV452713.1	8357874	1-scf00011	1
AX-157907628	1	83.183	KV452713.1	8360513	1-scf00011	1
AX-157907633	1	83.183	KV452713.1	8366436	1-scf00011	1
AX-157706333	1	83.183	3ML01032078	436	scf07691_1	UN
AX-157907630	1	83.474	KV452713.1	8364503	1-scf00011	1
AX-157907631	1	83.474	KV452713.1	8365467	1-scf00011	1
AX-157907637	1	83.548	KV452713.1	8372522	1-scf00011	1
AX-157907638	1	83.62	KV452713.1	8373543	1-scf00011	1
AX-157907641	1	83.62	KV452713.1	8376107	1-scf00011	1
AX-157907642	1	83.62	KV452713.1	8376994	1-scf00011	1
AX-157756483	1	83.62	KV452713.1	8378978	1-scf00011	1
AX-157907643	1	83.62	KV452713.1	8379857	1-scf00011	1
AX-157907647	1	83.62	KV452713.1	8382278	1-scf00011	1
AX-157714989	1	83.668	KV452713.1	8388962	1-scf00011	1
AX-157907651	1	83.729	KV452713.1	8393895	1-scf00011	1
AX-157907653	1	83.729	KV452713.1	8399036	1-scf00011	1
AX-157907654	1	83.729	KV452713.1	8399785	1-scf00011	1
AX-157772386	1	83.729	KV452713.1	8400737	1-scf00011	1
AX-157907659	1	83.729	KV452713.1	8404174	1-scf00011	1
AX-157907661	1	83.729	KV452713.1	8405231	1-scf00011	1
AX-157907663	1	83.729	KV452713.1	8405974	1-scf00011	1
AX-157907664	1	83.729	KV452713.1	8408302	1-scf00011	1
AX-157907665	1	83.729	KV452713.1	8408432	1-scf00011	1
AX-157907671	1	83.729	KV452713.1	8418343	1-scf00011	1
AX-157907646	1	83.79	KV452713.1	8381414	1-scf00011	1
AX-157907670	1	83.79	KV452713.1	8415687	1-scf00011	1
AX-157907674	1	83.838	KV452713.1	8421092	1-scf00011	1
AX-157907675	1	83.948	KV452713.1	8422035	1-scf00011	1
AX-157907676	1	83.948	KV452713.1	8423041	1-scf00011	1
AX-157907678	1	83.948	KV452713.1	8425687	1-scf00011	1
AX-157907685	1	84.111	KV452713.1	8439230	1-scf00011	1
AX-157907686	1	84.275	KV452713.1	8439772	1-scf00011	1
AX-158389528	1	84.275	KV452713.1	8440638	1-scf00011	1
AX-157907687	1	84.275	KV452713.1	8440999	1-scf00011	1
AX-157907691	1	84.275	KV452713.1	8448708	1-scf00011	1
AX-157907692	1	84.275	KV452713.1	8449734	1-scf00011	1
AX-157907693	1	84.275	KV452713.1	8450199	1-scf00011	1
AX-157907694	1	84.275	KV452713.1	8453909	1-scf00011	1
AX-157720865	1	84.275	KV452713.1	8454860	1-scf00011	1
AX-158414744	1	84.275	KV452713.1	8455825	1-scf00011	1
AX-157907695	1	84.275	KV452713.1	8458940	1-scf00011	1
AX-157907696	1	84.275	KV452713.1	8459341	1-scf00011	1
AX-157907699	1	84.275	KV452713.1	8465871	1-scf00011	1

AX-157907702	1	84.275	KV452713.1	8468719	1-scf00011	1
AX-157907703	1	84.275	KV452713.1	8470278	1-scf00011	1
AX-157907704	1	84.275	KV452713.1	8472536	1-scf00011	1
AX-157907706	1	84.275	KV452713.1	8474139	1-scf00011	1
AX-157907711	1	84.275	KV452713.1	8480182	1-scf00011	1
AX-157907712	1	84.275	KV452713.1	8480828	1-scf00011	1
AX-157907713	1	84.275	KV452713.1	8482980	1-scf00011	1
AX-157907714	1	84.275	KV452713.1	8484985	1-scf00011	1
AX-157907752	1	84.275	KV452713.1	8527232	1-scf00011	1
AX-157907715	1	84.334	KV452713.1	8487026	1-scf00011	1
AX-157907681	1	84.384	KV452713.1	8428625	1-scf00011	1
AX-157907709	1	84.384	KV452713.1	8478573	1-scf00011	1
AX-157907717	1	84.384	KV452713.1	8488745	1-scf00011	1
AX-157907718	1	84.384	KV452713.1	8489088	1-scf00011	1
AX-157907719	1	84.384	KV452713.1	8490182	1-scf00011	1
AX-157907720	1	84.384	KV452713.1	8491336	1-scf00011	1
AX-157907721	1	84.384	KV452713.1	8491637	1-scf00011	1
AX-157907722	1	84.384	KV452713.1	8494110	1-scf00011	1
AX-157907723	1	84.384	KV452713.1	8494399	1-scf00011	1
AX-157907724	1	84.485	KV452713.1	8495174	1-scf00011	1
AX-157907729	1	84.603	KV452713.1	8500617	1-scf00011	1
AX-157727831	1	84.603	KV452713.1	8502225	1-scf00011	1
AX-157907730	1	84.603	KV452713.1	8502318	1-scf00011	1
AX-157907734	1	84.603	KV452713.1	8508543	1-scf00011	1
AX-157907735	1	84.603	KV452713.1	8509030	1-scf00011	1
AX-157773102	1	84.603	KV452713.1	8510283	1-scf00011	1
AX-157907737	1	84.603	KV452713.1	8510503	1-scf00011	1
AX-157907738	1	84.603	KV452713.1	8511320	1-scf00011	1
AX-157907739	1	84.603	KV452713.1	8511696	1-scf00011	1
AX-157907740	1	84.603	KV452713.1	8511929	1-scf00011	1
AX-157739131	1	84.603	KV452713.1	8515260	1-scf00011	1
AX-157907743	1	84.603	KV452713.1	8515410	1-scf00011	1
AX-157907748	1	84.603	KV452713.1	8523250	1-scf00011	1
AX-157741065	1	84.603	KV452713.1	8525573	1-scf00011	1
AX-157907750	1	84.603	KV452713.1	8526221	1-scf00011	1
AX-157907751	1	84.603	KV452713.1	8526925	1-scf00011	1
AX-157907753	1	84.603	KV452713.1	8528994	1-scf00011	1
AX-157907757	1	84.603	KV452713.1	8530861	1-scf00011	1
AX-157812006	1	84.645	KV452713.1	8531252	1-scf00011	1
AX-157732146	1	84.645	KV452713.1	8538446	1-scf00011	1
AX-157907758	1	84.688	KV452713.1	8532495	1-scf00011	1
AX-157864440	1	84.688	KV452713.1	8539668	1-scf00011	1
AX-157907759	1	84.712	KV452713.1	8533944	1-scf00011	1
AX-157907760	1	84.712	KV452713.1	8540279	1-scf00011	1
AX-157907761	1	84.712	KV452713.1	8540428	1-scf00011	1
AX-157812007	1	84.712	KV452713.1	8542794	1-scf00011	1
AX-157907763	1	84.712	KV452713.1	8545226	1-scf00011	1
AX-157907764	1	84.93	KV452713.1	8546131	1-scf00011	1
AX-157907766	1	84.93	KV452713.1	8547062	1-scf00011	1
AX-157745708	1	84.93	KV452713.1	8548613	1-scf00011	1
AX-157907772	1	84.93	KV452713.1	8554831	1-scf00011	1

AX-157907773	1	84.93	KV452713.1	8555651	1-scf00011	1
AX-157907775	1	84.93	KV452713.1	8556925	1-scf00011	1
AX-157907776	1	84.93	KV452713.1	8557831	1-scf00011	1
AX-157812012	1	84.93	KV452713.1	8558529	1-scf00011	1
AX-157907777	1	84.93	KV452713.1	8558654	1-scf00011	1
AX-157853633	1	84.93	KV452713.1	8559670	1-scf00011	1
AX-157907780	1	85.149	KV452713.1	8563685	1-scf00011	1
AX-158404806	1	85.149	KV452713.1	8563960	1-scf00011	1
AX-158404807	1	85.149	KV452713.1	8564132	1-scf00011	1
AX-157907781	1	85.149	KV452713.1	8565697	1-scf00011	1
AX-157907783	1	85.198	KV452713.1	8567885	1-scf00011	1
AX-157751117	1	85.239	KV452713.1	8568352	1-scf00011	1
AX-157907785	1	85.239	KV452713.1	8571016	1-scf00011	1
AX-157907791	1	85.239	KV452713.1	8578108	1-scf00011	1
AX-157907767	1	85.258	KV452713.1	8547997	1-scf00011	1
AX-157907774	1	85.258	KV452713.1	8556014	1-scf00011	1
AX-157812013	1	85.258	KV452713.1	8561702	1-scf00011	1
AX-157907784	1	85.258	KV452713.1	8568887	1-scf00011	1
AX-157907786	1	85.258	KV452713.1	8572075	1-scf00011	1
AX-157907787	1	85.258	KV452713.1	8572650	1-scf00011	1
AX-157907788	1	85.258	KV452713.1	8574905	1-scf00011	1
AX-157907789	1	85.258	KV452713.1	8576353	1-scf00011	1
AX-157726212	1	85.258	KV452713.1	8576682	1-scf00011	1
AX-157907797	1	85.258	KV452713.1	8586511	1-scf00011	1
AX-157907769	1	85.259	KV452713.1	8550237	1-scf00011	1
AX-157907793	1	85.259	KV452713.1	8580652	1-scf00011	1
AX-157907794	1	85.259	KV452713.1	8581647	1-scf00011	1
AX-157907800	1	85.668	KV452713.1	8603160	1-scf00011	1
AX-157721740	1	85.668	KV452713.1	8610374	1-scf00011	1
AX-157907802	1	85.668	KV452713.1	8611969	1-scf00011	1
AX-157907803	1	85.668	KV452713.1	8615514	1-scf00011	1
AX-157907804	1	85.713	KV452713.1	8617430	1-scf00011	1
AX-157716618	1	85.804	KV452713.1	8619983	1-scf00011	1
AX-157812019	1	85.804	KV452713.1	8622954	1-scf00011	1
AX-157907809	1	85.804	KV452713.1	8623896	1-scf00011	1
AX-157907810	1	85.804	KV452713.1	8626513	1-scf00011	1
AX-157907812	1	85.804	KV452713.1	8629240	1-scf00011	1
AX-157907815	1	85.804	KV452713.1	8634207	1-scf00011	1
AX-157907807	1	86.023	KV452713.1	8622216	1-scf00011	1
AX-157715258	1	86.023	KV452713.1	8631783	1-scf00011	1
AX-157907817	1	86.023	KV452713.1	8635269	1-scf00011	1
AX-157907819	1	86.023	KV452713.1	8637325	1-scf00011	1
AX-157907820	1	86.023	KV452713.1	8639287	1-scf00011	1
AX-157812024	1	86.023	KV452713.1	8639982	1-scf00011	1
AX-157743676	1	86.023	KV452713.1	8640624	1-scf00011	1
AX-157812025	1	86.023	KV452713.1	8640736	1-scf00011	1
AX-157812026	1	86.023	KV452713.1	8642964	1-scf00011	1
AX-157907821	1	86.023	KV452713.1	8644036	1-scf00011	1
AX-157907822	1	86.023	KV452713.1	8644604	1-scf00011	1
AX-157907823	1	86.023	KV452713.1	8645147	1-scf00011	1
AX-157713598	1	86.241	KV452713.1	8656802	1-scf00011	1

AX-157907831	1	86.46	KV452713.1	8657281	1-scf00011	1
AX-157907832	1	86.46	KV452713.1	8659063	1-scf00011	1
AX-157812027	1	86.46	KV452713.1	8659966	1-scf00011	1
AX-157907806	1	86.787	KV452713.1	8619672	1-scf00011	1
AX-157748507	1	86.787	KV452713.1	8665179	1-scf00011	1
AX-157907836	1	86.787	KV452713.1	8670714	1-scf00011	1
AX-157907838	1	86.787	KV452713.1	8673235	1-scf00011	1
AX-157907841	1	86.787	KV452713.1	8677855	1-scf00011	1
AX-157907848	1	86.787	KV452713.1	8684534	1-scf00011	1
AX-157907849	1	86.787	KV452713.1	8686748	1-scf00011	1
AX-157713517	1	86.835	KV452713.1	8660743	1-scf00011	1
AX-157907839	1	86.835	KV452713.1	8674527	1-scf00011	1
AX-157874503	1	86.835	KV452713.1	8681447	1-scf00011	1
AX-157907851	1	86.835	KV452713.1	8687384	1-scf00011	1
AX-157812028	1	86.863	KV452713.1	8669753	1-scf00011	1
AX-157722220	1	86.863	KV452713.1	8693183	1-scf00011	1
AX-157907854	1	86.896	KV452713.1	8697541	1-scf00011	1
AX-157907857	1	86.896	KV452713.1	8700828	1-scf00011	1
AX-157907860	1	86.896	KV452713.1	8702986	1-scf00011	1
AX-158389532	1	86.896	KV452713.1	8704200	1-scf00011	1
AX-157907865	1	87.115	KV452713.1	8712358	1-scf00011	1
AX-157743349	1	87.145	KV452713.1	8713373	1-scf00011	1
AX-157907866	1	87.174	KV452713.1	8714488	1-scf00011	1
AX-157907870	1	87.2	KV452713.1	8720796	1-scf00011	1
AX-157907871	1	87.224	KV452713.1	8721628	1-scf00011	1
AX-157907876	1	87.271	KV452713.1	8727982	1-scf00011	1
AX-157907877	1	87.293	KV452713.1	8729469	1-scf00011	1
AX-157907878	1	87.333	KV452713.1	8733308	1-scf00011	1
AX-157907879	1	87.333	KV452713.1	8735043	1-scf00011	1
AX-157907881	1	87.333	KV452713.1	8736055	1-scf00011	1
AX-157907883	1	87.333	KV452713.1	8738425	1-scf00011	1
AX-157907884	1	87.333	KV452713.1	8739849	1-scf00011	1
AX-157907887	1	87.333	KV452713.1	8743018	1-scf00011	1
AX-157907889	1	87.333	KV452713.1	8751086	1-scf00011	1
AX-157907890	1	87.333	KV452713.1	8751483	1-scf00011	1
AX-158379197	1	87.333	KV452713.1	8755215	1-scf00011	1
AX-157742017	1	87.333	KV452713.1	8755360	1-scf00011	1
AX-157907896	1	87.333	KV452713.1	8758730	1-scf00011	1
AX-157907898	1	87.333	KV452713.1	8759611	1-scf00011	1
AX-157907900	1	87.34	KV452713.1	8762890	1-scf00011	1
AX-157907927	1	87.34	KV452713.1	8791794	1-scf00011	1
AX-157747173	1	87.371	KV452713.1	8765674	1-scf00011	1
AX-157907902	1	87.371	KV452713.1	8768115	1-scf00011	1
AX-157907904	1	87.408	KV452713.1	8771731	1-scf00011	1
AX-157907862	1	87.443	KV452713.1	8704569	1-scf00011	1
AX-157907874	1	87.443	KV452713.1	8725659	1-scf00011	1
AX-157907891	1	87.443	KV452713.1	8752230	1-scf00011	1
AX-157907908	1	87.443	KV452713.1	8773855	1-scf00011	1
AX-157907912	1	87.443	KV452713.1	8777744	1-scf00011	1
AX-157907913	1	87.443	KV452713.1	8779642	1-scf00011	1
AX-157907914	1	87.443	KV452713.1	8779852	1-scf00011	1

AX-157907880	1	87.77	KV452713.1	8735393	1-scf00011	1
AX-157907894	1	87.77	KV452713.1	8758007	1-scf00011	1
AX-157744893	1	87.77	KV452713.1	8764809	1-scf00011	1
AX-157907911	1	87.77	KV452713.1	8777250	1-scf00011	1
AX-157907917	1	87.77	KV452713.1	8781973	1-scf00011	1
AX-157907919	1	87.77	KV452713.1	8782482	1-scf00011	1
AX-157907920	1	87.77	KV452713.1	8783408	1-scf00011	1
AX-157907921	1	87.77	KV452713.1	8783795	1-scf00011	1
AX-157907923	1	87.77	KV452713.1	8786810	1-scf00011	1
AX-157907924	1	87.77	KV452713.1	8790616	1-scf00011	1
AX-157907926	1	88	KV452713.1	8791371	1-scf00011	1
AX-157907949	1	88	KV452713.1	8821226	1-scf00011	1
AX-157907929	1	88.098	KV452713.1	8792809	1-scf00011	1
AX-157907932	1	88.099	KV452713.1	8796275	1-scf00011	1
AX-157907933	1	88.099	KV452713.1	8796422	1-scf00011	1
AX-157907934	1	88.099	KV452713.1	8797643	1-scf00011	1
AX-157907936	1	88.099	KV452713.1	8798505	1-scf00011	1
AX-157907938	1	88.099	KV452713.1	8802213	1-scf00011	1
AX-157883357	1	88.099	KV452713.1	8802449	1-scf00011	1
AX-157756056	1	88.099	KV452713.1	8804881	1-scf00011	1
AX-157907940	1	88.099	KV452713.1	8806538	1-scf00011	1
AX-157907941	1	88.099	KV452713.1	8809130	1-scf00011	1
AX-157907943	1	88.099	KV452713.1	8811396	1-scf00011	1
AX-157770970	1	88.099	KV452713.1	8811812	1-scf00011	1
AX-157907944	1	88.099	KV452713.1	8812343	1-scf00011	1
AX-157907963	1	88.099	KV452713.1	8841309	1-scf00011	1
AX-157907945	1	88.244	KV452713.1	8817184	1-scf00011	1
AX-157750674	1	88.245	KV452713.1	8803396	1-scf00011	1
AX-157907942	1	88.245	KV452713.1	8810659	1-scf00011	1
AX-157907946	1	88.245	KV452713.1	8818080	1-scf00011	1
AX-157907951	1	88.356	KV452713.1	8822424	1-scf00011	1
AX-157907953	1	88.532	KV452713.1	8826030	1-scf00011	1
AX-157907895	1	88.534	KV452713.1	8758454	1-scf00011	1
AX-157907955	1	88.534	KV452713.1	8828095	1-scf00011	1
AX-157907899	1	88.536	KV452713.1	8762667	1-scf00011	1
AX-157907922	1	88.536	KV452713.1	8785497	1-scf00011	1
AX-157907956	1	88.536	KV452713.1	8829949	1-scf00011	1
AX-157907957	1	88.536	KV452713.1	8832972	1-scf00011	1
AX-157775584	1	88.536	KV452713.1	8834786	1-scf00011	1
AX-157907958	1	88.536	KV452713.1	8836519	1-scf00011	1
AX-157907961	1	88.548	KV452713.1	8838883	1-scf00011	1
AX-157907965	1	89.076	KV452713.1	8842975	1-scf00011	1
AX-157907966	1	89.076	KV452713.1	8843746	1-scf00011	1
AX-157907968	1	89.077	KV452713.1	8847080	1-scf00011	1
AX-157907969	1	89.1	KV452713.1	8847456	1-scf00011	1
AX-157907972	1	89.228	KV452713.1	8851446	1-scf00011	1
AX-157757991	1	89.228	KV452713.1	8853050	1-scf00011	1
AX-157907975	1	89.434	KV452713.1	8856598	1-scf00011	1
AX-157907976	1	89.55	KV452713.1	8857977	1-scf00011	1
AX-157812031	1	89.848	KV452713.1	8862161	1-scf00011	1
AX-157907979	1	89.848	KV452713.1	8863124	1-scf00011	1

AX-157746769	1	89.848	KV452713.1	8863499	1-scf00011	1
AX-157853635	1	89.919	KV452713.1	8867991	1-scf00011	1
AX-157907983	1	90.177	KV452713.1	8870236	1-scf00011	1
AX-157740449	1	90.177	KV452713.1	8876006	1-scf00011	1
AX-157907985	1	90.177	KV452713.1	8880205	1-scf00011	1
AX-157907986	1	90.177	KV452713.1	8880616	1-scf00011	1
AX-157907996	1	90.177	KV452713.1	8892653	1-scf00011	1
AX-157907997	1	90.177	KV452713.1	8892762	1-scf00011	1
AX-157907987	1	90.179	KV452713.1	8881288	1-scf00011	1
AX-157907992	1	90.329	KV452713.1	8886281	1-scf00011	1
AX-157907989	1	90.458	KV452713.1	8882760	1-scf00011	1
AX-157907993	1	90.458	KV452713.1	8887728	1-scf00011	1
AX-157725188	1	90.458	KV452713.1	8890549	1-scf00011	1
AX-157907995	1	90.458	KV452713.1	8891186	1-scf00011	1
AX-158382142	1	90.458	KV452713.1	8892304	1-scf00011	1
AX-157908000	1	90.458	KV452713.1	8898109	1-scf00011	1
AX-157707156	1	90.547	KV452713.1	8865023	1-scf00011	1
AX-157907981	1	90.547	KV452713.1	8868904	1-scf00011	1
AX-157750112	1	90.547	KV452713.1	8889254	1-scf00011	1
AX-157908013	1	90.547	KV452713.1	8913314	1-scf00011	1
AX-157812038	1	90.547	KV452713.1	8913470	1-scf00011	1
AX-157908021	1	90.547	KV452713.1	8921286	1-scf00011	1
AX-157908002	1	90.66	KV452713.1	8902887	1-scf00011	1
AX-157812037	1	90.753	KV452713.1	8903460	1-scf00011	1
AX-157907998	1	90.801	KV452713.1	8895873	1-scf00011	1
AX-157907999	1	90.801	KV452713.1	8897026	1-scf00011	1
AX-157908003	1	90.801	KV452713.1	8905339	1-scf00011	1
AX-157908010	1	90.825	KV452713.1	8911197	1-scf00011	1
AX-157908012	1	90.912	KV452713.1	8912487	1-scf00011	1
AX-157908014	1	90.943	KV452713.1	8914067	1-scf00011	1
AX-157721234	1	90.943	KV452713.1	8915948	1-scf00011	1
AX-157908017	1	90.943	KV452713.1	8917120	1-scf00011	1
AX-157908020	1	90.943	KV452713.1	8919433	1-scf00011	1
AX-157908022	1	90.943	KV452713.1	8921957	1-scf00011	1
AX-157908023	1	90.943	KV452713.1	8922606	1-scf00011	1
AX-157908025	1	91.278	KV452713.1	8924189	1-scf00011	1
AX-157908028	1	91.293	KV452713.1	8933869	1-scf00011	1
AX-157908030	1	91.538	KV452713.1	8934829	1-scf00011	1
AX-157908031	1	91.539	KV452713.1	8935819	1-scf00011	1
AX-157908033	1	91.86	KV452713.1	8938498	1-scf00011	1
AX-157908037	1	92.313	KV452713.1	8943721	1-scf00011	1
AX-157715304	1	92.34	KV452713.1	8945136	1-scf00011	1
AX-157908039	1	92.389	KV452713.1	8948925	1-scf00011	1
AX-157908042	1	92.389	KV452713.1	8952555	1-scf00011	1
AX-157908043	1	92.389	KV452713.1	8954165	1-scf00011	1
AX-157908044	1	92.573	KV452713.1	8954719	1-scf00011	1
AX-157908056	1	92.573	KV452713.1	8970151	1-scf00011	1
AX-157726401	1	92.778	KV452713.1	8957252	1-scf00011	1
AX-157908045	1	92.778	KV452713.1	8959625	1-scf00011	1
AX-157908046	1	92.778	KV452713.1	8960067	1-scf00011	1
AX-157908049	1	92.778	KV452713.1	8962418	1-scf00011	1

AX-157908050	1	92.778	KV452713.1	8963691	1-scf00011	1
AX-157908051	1	92.778	KV452713.1	8964858	1-scf00011	1
AX-157908057	1	92.969	KV452713.1	8971239	1-scf00011	1
AX-157908058	1	93.165	KV452713.1	8973159	1-scf00011	1
AX-157853636	1	93.173	KV452713.1	8975233	1-scf00011	1
AX-157908059	1	93.173	KV452713.1	8975355	1-scf00011	1
AX-157908061	1	93.437	KV452713.1	8978675	1-scf00011	1
AX-157908053	1	93.662	KV452713.1	8968808	1-scf00011	1
AX-157853638	1	93.662	KV452713.1	8979929	1-scf00011	1
AX-157908064	1	93.662	KV452713.1	8983167	1-scf00011	1
AX-157719036	1	93.662	KV452713.1	8984125	1-scf00011	1
AX-157908066	1	93.662	KV452713.1	8987076	1-scf00011	1
AX-157908067	1	94.006	KV452713.1	8987834	1-scf00011	1
AX-157908073	1	94.025	KV452713.1	8995416	1-scf00011	1
AX-157908085	1	94.716	KV452713.1	9011773	1-scf00011	1
AX-157908089	1	94.89	KV452713.1	9019890	1-scf00011	1
AX-157908090	1	94.89	KV452713.1	9020554	1-scf00011	1
AX-157908096	1	94.891	KV452713.1	9027277	1-scf00011	1
AX-157874511	1	95.054	KV452713.1	9009959	1-scf00011	1
AX-157908095	1	95.054	KV452713.1	9026867	1-scf00011	1
AX-157908097	1	95.054	KV452713.1	9028087	1-scf00011	1
AX-157908100	1	95.09	KV452713.1	9032343	1-scf00011	1
AX-157908102	1	95.218	KV452713.1	9036590	1-scf00011	1
AX-157746531	1	95.218	KV452713.1	9037113	1-scf00011	1
AX-157908107	1	95.218	KV452713.1	9050819	1-scf00011	1
AX-157908111	1	95.218	KV452713.1	9056857	1-scf00011	1
AX-157774213	1	95.218	KV452713.1	9060588	1-scf00011	1
AX-157908116	1	95.218	KV452713.1	9067862	1-scf00011	1
AX-157862641	1	95.218	KV452713.1	9070679	1-scf00011	1
AX-157812041	1	95.218	KV452713.1	9072005	1-scf00011	1
AX-157908103	1	95.437	KV452713.1	9042706	1-scf00011	1
AX-157908119	1	95.437	KV452713.1	9076021	1-scf00011	1
AX-157812042	1	95.437	KV452713.1	9076615	1-scf00011	1
AX-157908120	1	95.437	KV452713.1	9076754	1-scf00011	1
AX-157908121	1	95.437	KV452713.1	9077670	1-scf00011	1
AX-157812043	1	95.437	KV452713.1	9079123	1-scf00011	1
AX-157908123	1	95.437	KV452713.1	9079791	1-scf00011	1
AX-157908125	1	95.498	KV452713.1	9081904	1-scf00011	1
AX-157908118	1	95.546	KV452713.1	9074772	1-scf00011	1
AX-157752088	1	95.546	KV452713.1	9084953	1-scf00011	1
AX-157812044	1	95.546	KV452713.1	9085482	1-scf00011	1
AX-157908127	1	95.546	KV452713.1	9086848	1-scf00011	1
AX-157874516	1	95.546	KV452713.1	9090122	1-scf00011	1
AX-157812045	1	95.546	KV452713.1	9090726	1-scf00011	1
AX-157908130	1	95.546	KV452713.1	9091331	1-scf00011	1
AX-157908131	1	95.546	KV452713.1	9091874	1-scf00011	1
AX-157908132	1	95.546	KV452713.1	9098075	1-scf00011	1
AX-157908133	1	95.546	KV452713.1	9099346	1-scf00011	1
AX-157908134	1	95.546	KV452713.1	9100530	1-scf00011	1
AX-157908135	1	95.546	KV452713.1	9101007	1-scf00011	1
AX-158379200	1	95.546	KV452713.1	9115499	1-scf00011	1

AX-157908138	1	95.764	KV452713.1	9106114	1-scf00011	1
AX-157908139	1	95.764	KV452713.1	9106349	1-scf00011	1
AX-157908140	1	95.764	KV452713.1	9106748	1-scf00011	1
AX-157741691	1	96.092	KV452713.1	9114106	1-scf00011	1
AX-157713151	1	96.092	KV452713.1	9120129	1-scf00011	1
AX-157719373	1	96.102	KV452713.1	9134414	1-scf00011	1
AX-157908151	1	96.102	KV452713.1	9147113	1-scf00011	1
AX-157908156	1	96.102	KV452713.1	9154423	1-scf00011	1
AX-157908147	1	96.106	KV452713.1	9136763	1-scf00011	1
AX-157883359	1	96.106	KV452713.1	9160547	1-scf00011	1
AX-157908148	1	96.11	KV452713.1	9137682	1-scf00011	1
AX-157713439	1	96.127	KV452713.1	9138644	1-scf00011	1
AX-157908149	1	96.138	KV452713.1	9141057	1-scf00011	1
AX-157908150	1	96.169	KV452713.1	9145529	1-scf00011	1
AX-157908152	1	96.201	KV452713.1	9148406	1-scf00011	1
AX-157908157	1	96.201	KV452713.1	9155575	1-scf00011	1
AX-157908161	1	96.31	KV452713.1	9165837	1-scf00011	1
AX-157908163	1	96.389	KV452713.1	9168196	1-scf00011	1
AX-157908164	1	96.419	KV452713.1	9168548	1-scf00011	1
AX-157908165	1	96.443	KV452713.1	9169321	1-scf00011	1
AX-157908167	1	96.466	KV452713.1	9170850	1-scf00011	1
AX-157908168	1	96.485	KV452713.1	9171618	1-scf00011	1
AX-157751083	1	96.505	KV452713.1	9184077	1-scf00011	1
AX-157908172	1	96.529	KV452713.1	9187284	1-scf00011	1
AX-157908173	1	96.529	KV452713.1	9188571	1-scf00011	1
AX-157775878	1	96.614	KV452713.1	9194199	1-scf00011	1
AX-157756463	1	96.747	KV452713.1	9199661	1-scf00011	1
AX-157908177	1	96.747	KV452713.1	9202751	1-scf00011	1
AX-158382146	1	97.18	KV452713.1	9208862	1-scf00011	1
AX-157741723	1	97.184	KV452713.1	9219052	1-scf00011	1
AX-157908185	1	97.294	KV452713.1	9223226	1-scf00011	1
AX-157908186	1	97.393	KV452713.1	9223891	1-scf00011	1
AX-158379201	1	97.513	KV452713.1	9214138	1-scf00011	1
AX-157719162	1	97.513	KV452713.1	9228392	1-scf00011	1
AX-157908189	1	97.513	KV452713.1	9232981	1-scf00011	1
AX-157908191	1	97.513	KV452713.1	9234449	1-scf00011	1
AX-157908193	1	97.513	KV452713.1	9235519	1-scf00011	1
AX-157908194	1	97.513	KV452713.1	9236816	1-scf00011	1
AX-157774919	1	97.731	KV452713.1	9161536	1-scf00011	1
AX-157741279	1	97.731	KV452713.1	9199226	1-scf00011	1
AX-157908179	1	97.731	KV452713.1	9210529	1-scf00011	1
AX-157908184	1	97.731	KV452713.1	9222737	1-scf00011	1
AX-157908190	1	97.731	KV452713.1	9233801	1-scf00011	1
AX-157908196	1	97.731	KV452713.1	9241301	1-scf00011	1
AX-157908197	1	97.731	KV452713.1	9241586	1-scf00011	1
AX-157908198	1	97.731	KV452713.1	9245265	1-scf00011	1
AX-157908199	1	97.731	KV452713.1	9245907	1-scf00011	1
AX-157908200	1	97.731	KV452713.1	9246333	1-scf00011	1
AX-157908203	1	97.731	KV452713.1	9252203	1-scf00011	1
AX-157908204	1	97.731	KV452713.1	9252863	1-scf00011	1
AX-157706586	1	97.731	3ML01032524	371	scf08146_1	UN

AX-157908201	1	97.804	KV452713.1	9249368	1-scf00011	1
AX-157908206	1	97.804	KV452713.1	9257288	1-scf00011	1
AX-157908207	1	97.804	KV452713.1	9258023	1-scf00011	1
AX-157908219	1	97.804	KV452713.1	9274010	1-scf00011	1
AX-158382147	1	97.95	KV452713.1	9257957	1-scf00011	1
AX-157908209	1	97.95	KV452713.1	9259129	1-scf00011	1
AX-157908210	1	97.95	KV452713.1	9260609	1-scf00011	1
AX-157716619	1	97.95	KV452713.1	9261620	1-scf00011	1
AX-157908213	1	97.95	KV452713.1	9263986	1-scf00011	1
AX-157757758	1	97.95	KV452713.1	9271140	1-scf00011	1
AX-157812053	1	97.95	KV452713.1	9341792	1-scf00011	1
AX-157908217	1	98.277	KV452713.1	9272130	1-scf00011	1
AX-157908218	1	98.277	KV452713.1	9272384	1-scf00011	1
AX-157908215	1	98.387	KV452713.1	9269628	1-scf00011	1
AX-157908221	1	98.387	KV452713.1	9276113	1-scf00011	1
AX-157724421	1	98.387	KV452713.1	9282709	1-scf00011	1
AX-157874520	1	98.387	KV452713.1	9284965	1-scf00011	1
AX-157908227	1	98.387	KV452713.1	9287978	1-scf00011	1
AX-157812052	1	98.387	KV452713.1	9294229	1-scf00011	1
AX-157908230	1	98.387	KV452713.1	9294752	1-scf00011	1
AX-157908232	1	98.387	KV452713.1	9309602	1-scf00011	1
AX-157908233	1	98.387	KV452713.1	9310796	1-scf00011	1
AX-157693250	1	98.387	KV452713.1	9339145	1-scf00011	1
AX-157908236	1	98.389	KV452713.1	9333237	1-scf00011	1
AX-157908238	1	98.78	KV452713.1	9343757	1-scf00011	1
AX-157908239	1	98.78	KV452713.1	9358557	1-scf00011	1
AX-157908240	1	98.78	KV452713.1	9359352	1-scf00011	1
AX-157908241	1	98.912	KV452713.1	9363887	1-scf00011	1
AX-157908242	1	98.912	KV452713.1	9364652	1-scf00011	1
AX-157750852	1	98.912	KV452713.1	9369352	1-scf00011	1
AX-157908246	1	99.043	KV452713.1	9370249	1-scf00011	1
AX-157908247	1	99.043	KV452713.1	9371488	1-scf00011	1
AX-157908248	1	99.043	KV452713.1	9371990	1-scf00011	1
AX-157908249	1	99.043	KV452713.1	9372954	1-scf00011	1
AX-157908254	1	99.371	KV452713.1	9383192	1-scf00011	1
AX-157908255	1	99.371	KV452713.1	9383576	1-scf00011	1
AX-157908257	1	99.371	KV452713.1	9385528	1-scf00011	1
AX-157908280	1	99.371	KV452713.1	9414648	1-scf00011	1
AX-157908260	1	99.395	KV452713.1	9389209	1-scf00011	1
AX-157908262	1	99.433	KV452713.1	9391071	1-scf00011	1
AX-157908263	1	99.455	KV452713.1	9393342	1-scf00011	1
AX-157908265	1	99.48	KV452713.1	9399709	1-scf00011	1
AX-157908266	1	99.626	KV452713.1	9400389	1-scf00011	1
AX-157908268	1	99.699	KV452713.1	9402426	1-scf00011	1
AX-157908270	1	99.699	KV452713.1	9403772	1-scf00011	1
AX-157908271	1	99.726	KV452713.1	9406196	1-scf00011	1
AX-157908272	1	99.785	KV452713.1	9407167	1-scf00011	1
AX-157908273	1	99.841	KV452713.1	9407631	1-scf00011	1
AX-157908275	1	99.885	KV452713.1	9409800	1-scf00011	1
AX-157908277	1	99.885	KV452713.1	9412686	1-scf00011	1
AX-157908278	1	99.917	KV452713.1	9413554	1-scf00011	1

AX-157908281	1	99.917	KV452713.1	9415271	1-scf00011	1
AX-157908287	1	99.917	KV452713.1	9421393	1-scf00011	1
AX-157908285	1	99.918	KV452713.1	9420091	1-scf00011	1
AX-157908291	1	99.921	KV452713.1	9426794	1-scf00011	1
AX-157908294	1	99.923	KV452713.1	9429107	1-scf00011	1
AX-157908296	1	99.926	KV452713.1	9430775	1-scf00011	1
AX-157773967	1	100.354	KV452713.1	9393795	1-scf00011	1
AX-157908299	1	100.354	KV452713.1	9434977	1-scf00011	1
AX-157738961	1	100.354	KV452713.1	9441771	1-scf00011	1
AX-157908302	1	100.354	KV452713.1	9442410	1-scf00011	1
AX-157908304	1	100.354	KV452713.1	9444052	1-scf00011	1
AX-157908306	1	100.354	KV452713.1	9446188	1-scf00011	1
AX-157853640	1	100.354	KV452713.1	9446242	1-scf00011	1
AX-157908308	1	100.354	KV452713.1	9448634	1-scf00011	1
AX-157908309	1	100.355	KV452713.1	9450057	1-scf00011	1
AX-158379202	1	100.626	KV452713.1	9454087	1-scf00011	1
AX-157908311	1	100.626	KV452713.1	9454284	1-scf00011	1
AX-157908313	1	100.901	KV452713.1	9457704	1-scf00011	1
AX-157908314	1	100.901	KV452713.1	9458551	1-scf00011	1
AX-157908316	1	100.901	KV452713.1	9460713	1-scf00011	1
AX-157908318	1	101.01	KV452713.1	9462045	1-scf00011	1
AX-157908319	1	101.01	KV452713.1	9463489	1-scf00011	1
AX-157908320	1	101.049	KV452713.1	9468249	1-scf00011	1
AX-157908325	1	101.175	KV452713.1	9478305	1-scf00011	1
AX-157812054	1	101.274	KV452713.1	9480129	1-scf00011	1
AX-157908321	1	101.337	KV452713.1	9470350	1-scf00011	1
AX-157908322	1	101.337	KV452713.1	9473184	1-scf00011	1
AX-157908326	1	101.337	KV452713.1	9480869	1-scf00011	1
AX-157715587	1	101.337	KV452713.1	9480947	1-scf00011	1
AX-157812057	1	101.337	KV452713.1	9544770	1-scf00011	1
AX-157908327	1	101.338	KV452713.1	9481458	1-scf00011	1
AX-157775225	1	101.338	KV452713.1	9487637	1-scf00011	1
AX-157908330	1	101.557	KV452713.1	9488204	1-scf00011	1
AX-157908335	1	101.557	KV452713.1	9492241	1-scf00011	1
AX-157908338	1	101.557	KV452713.1	9494224	1-scf00011	1
AX-157908339	1	101.557	KV452713.1	9495783	1-scf00011	1
AX-157908357	1	101.557	KV452713.1	9523505	1-scf00011	1
AX-157908332	1	101.666	KV452713.1	9489855	1-scf00011	1
AX-157908345	1	101.666	KV452713.1	9504952	1-scf00011	1
AX-157908348	1	101.666	KV452713.1	9507634	1-scf00011	1
AX-157908349	1	101.666	KV452713.1	9510261	1-scf00011	1
AX-157908351	1	101.666	KV452713.1	9512444	1-scf00011	1
AX-157908352	1	101.666	KV452713.1	9512880	1-scf00011	1
AX-157908353	1	101.718	KV452713.1	9513222	1-scf00011	1
AX-157908356	1	101.775	KV452713.1	9521060	1-scf00011	1
AX-157908358	1	101.856	KV452713.1	9524760	1-scf00011	1
AX-157908363	1	101.94	KV452713.1	9529922	1-scf00011	1
AX-157908364	1	102.102	KV452713.1	9530346	1-scf00011	1
AX-157908365	1	102.102	KV452713.1	9530830	1-scf00011	1
AX-157908366	1	102.102	KV452713.1	9533518	1-scf00011	1
AX-157754376	1	102.102	KV452713.1	9562974	1-scf00011	1

AX-157908354	1	102.103	KV452713.1	9514607	1-scf00011	1
AX-157908360	1	102.103	KV452713.1	9527229	1-scf00011	1
AX-157721168	1	102.103	KV452713.1	9534213	1-scf00011	1
AX-157908368	1	102.103	KV452713.1	9536196	1-scf00011	1
AX-157908369	1	102.103	KV452713.1	9537206	1-scf00011	1
AX-157853644	1	102.103	KV452713.1	9538675	1-scf00011	1
AX-157908371	1	102.103	KV452713.1	9539939	1-scf00011	1
AX-157908372	1	102.103	KV452713.1	9540755	1-scf00011	1
AX-157812058	1	102.103	KV452713.1	9545393	1-scf00011	1
AX-157812059	1	102.103	KV452713.1	9546843	1-scf00011	1
AX-157908376	1	102.322	KV452713.1	9553922	1-scf00011	1
AX-157741338	1	102.322	KV452713.1	9554606	1-scf00011	1
AX-157708399	1	102.322	KV452713.1	9557220	1-scf00011	1
AX-157908379	1	102.361	KV452713.1	9558589	1-scf00011	1
AX-157908380	1	102.392	KV452713.1	9559548	1-scf00011	1
AX-157771508	1	102.431	KV452713.1	9563423	1-scf00011	1
AX-157753411	1	102.65	KV452713.1	9585657	1-scf00011	1
AX-157908394	1	102.671	KV452713.1	9598679	1-scf00011	1
AX-157812063	1	102.691	KV452713.1	9599303	1-scf00011	1
AX-157908395	1	102.729	KV452713.1	9599793	1-scf00011	1
AX-157771004	1	102.759	KV452713.1	9565264	1-scf00011	1
AX-157908390	1	102.759	KV452713.1	9584358	1-scf00011	1
AX-157908396	1	102.759	KV452713.1	9600677	1-scf00011	1
AX-157721315	1	102.759	KV452713.1	9601479	1-scf00011	1
AX-157908397	1	102.759	KV452713.1	9602382	1-scf00011	1
AX-157812064	1	102.759	KV452713.1	9602577	1-scf00011	1
AX-157746701	1	102.977	KV452713.1	9607673	1-scf00011	1
AX-157908398	1	102.977	KV452713.1	9607901	1-scf00011	1
AX-157908399	1	102.977	KV452713.1	9608189	1-scf00011	1
AX-157908400	1	102.977	KV452713.1	9608565	1-scf00011	1
AX-157908402	1	102.978	KV452713.1	9615688	1-scf00011	1
AX-157812068	1	102.979	KV452713.1	9616116	1-scf00011	1
AX-157908403	1	103.051	KV452713.1	9616903	1-scf00011	1
AX-157743068	1	103.133	KV452713.1	9620089	1-scf00011	1
AX-158379204	1	103.133	KV452713.1	9620485	1-scf00011	1
AX-157908405	1	103.153	KV452713.1	9625115	1-scf00011	1
AX-157712204	1	103.331	KV452713.1	9605352	1-scf00011	1
AX-157908406	1	103.331	KV452713.1	9625519	1-scf00011	1
AX-157812069	1	103.331	KV452713.1	9628453	1-scf00011	1
AX-157772119	1	103.331	KV452713.1	9628798	1-scf00011	1
AX-157908407	1	103.335	KV452713.1	9627656	1-scf00011	1
AX-157908413	1	103.335	KV452713.1	9634788	1-scf00011	1
AX-157726895	1	103.408	KV452713.1	9640679	1-scf00011	1
AX-157908408	1	103.471	KV452713.1	9628232	1-scf00011	1
AX-157908415	1	103.471	KV452713.1	9641996	1-scf00011	1
AX-157908416	1	103.71	KV452713.1	9642801	1-scf00011	1
AX-157908417	1	103.714	KV452713.1	9643872	1-scf00011	1
AX-157908418	1	103.714	KV452713.1	9645082	1-scf00011	1
AX-157908421	1	103.779	KV452713.1	9647379	1-scf00011	1
AX-157908424	1	103.841	KV452713.1	9649914	1-scf00011	1
AX-157908425	1	103.968	KV452713.1	9650457	1-scf00011	1

AX-157908426	1	103.968	KV452713.1	9651235	1-scf00011	1
AX-157908427	1	103.968	KV452713.1	9652249	1-scf00011	1
AX-157908429	1	104.095	KV452713.1	9653929	1-scf00011	1
AX-157908431	1	104.095	KV452713.1	9654885	1-scf00011	1
AX-157752029	1	104.095	KV452713.1	9655452	1-scf00011	1
AX-157908433	1	104.095	KV452713.1	9659526	1-scf00011	1
AX-157742312	1	104.095	KV452713.1	9660034	1-scf00011	1
AX-157740596	1	104.222	KV452713.1	9664558	1-scf00011	1
AX-157908436	1	104.412	KV452713.1	9665670	1-scf00011	1
AX-157908442	1	104.603	KV452713.1	9685589	1-scf00011	1
AX-157908444	1	104.603	KV452713.1	9688359	1-scf00011	1
AX-157908446	1	104.603	KV452713.1	9690263	1-scf00011	1
AX-157908449	1	104.603	KV452713.1	9694950	1-scf00011	1
AX-157908477	1	104.603	KV452713.1	9734499	1-scf00011	1
AX-157908478	1	104.603	KV452713.1	9736054	1-scf00011	1
AX-157908448	1	104.647	KV452713.1	9694739	1-scf00011	1
AX-157908453	1	104.725	KV452713.1	9699316	1-scf00011	1
AX-157908445	1	104.729	KV452713.1	9689534	1-scf00011	1
AX-157908450	1	104.729	KV452713.1	9696345	1-scf00011	1
AX-157908454	1	104.729	KV452713.1	9703922	1-scf00011	1
AX-157908455	1	104.729	KV452713.1	9704534	1-scf00011	1
AX-157908457	1	104.729	KV452713.1	9706172	1-scf00011	1
AX-157908458	1	104.729	KV452713.1	9707034	1-scf00011	1
AX-157908460	1	104.729	KV452713.1	9708070	1-scf00011	1
AX-157908461	1	104.729	KV452713.1	9708312	1-scf00011	1
AX-157908462	1	104.729	KV452713.1	9709808	1-scf00011	1
AX-157908464	1	104.729	KV452713.1	9711309	1-scf00011	1
AX-157908465	1	104.947	KV452713.1	9712868	1-scf00011	1
AX-157908463	1	105.083	KV452713.1	9710272	1-scf00011	1
AX-157908467	1	105.083	KV452713.1	9715500	1-scf00011	1
AX-157908468	1	105.083	KV452713.1	9718300	1-scf00011	1
AX-157812073	1	105.083	KV452713.1	9718391	1-scf00011	1
AX-157908469	1	105.474	KV452713.1	9722329	1-scf00011	1
AX-157812075	1	105.493	KV452713.1	9737095	1-scf00011	1
AX-157908482	1	105.82	KV452713.1	9739953	1-scf00011	1
AX-157908483	1	105.82	KV452713.1	9740203	1-scf00011	1
AX-157908486	1	105.82	KV452713.1	9744948	1-scf00011	1
AX-157908488	1	105.821	KV452713.1	9747799	1-scf00011	1
AX-157908489	1	105.821	KV452713.1	9750109	1-scf00011	1
AX-157908491	1	105.821	KV452713.1	9751030	1-scf00011	1
AX-157908495	1	106.081	KV452713.1	9761990	1-scf00011	1
AX-157751315	1	106.26	KV452713.1	9768128	1-scf00011	1
AX-158379205	1	106.26	KV452713.1	9769271	1-scf00011	1
AX-157908497	1	106.26	KV452713.1	9769473	1-scf00011	1
AX-157908500	1	106.369	KV452713.1	9772826	1-scf00011	1
AX-157908501	1	106.369	KV452713.1	9773393	1-scf00011	1
AX-157908502	1	106.369	KV452713.1	9775651	1-scf00011	1
AX-157908504	1	106.393	KV452713.1	9783291	1-scf00011	1
AX-157908506	1	106.434	KV452713.1	9784492	1-scf00011	1
AX-157750077	1	106.479	KV452713.1	9785148	1-scf00011	1
AX-157908509	1	106.479	KV452713.1	9792076	1-scf00011	1

AX-157754566	1	106.479	KV452713.1	9792774	1-scf00011	1
AX-157908510	1	106.806	KV452713.1	9794868	1-scf00011	1
AX-157908515	1	106.806	KV452713.1	9804616	1-scf00011	1
AX-157908516	1	106.849	KV452713.1	9807934	1-scf00011	1
AX-157908526	1	106.849	KV452713.1	9819458	1-scf00011	1
AX-157908518	1	106.888	KV452713.1	9809266	1-scf00011	1
AX-157908522	1	106.931	KV452713.1	9814256	1-scf00011	1
AX-157908527	1	106.974	KV452713.1	9820834	1-scf00011	1
AX-157908546	1	106.974	KV452713.1	9844965	1-scf00011	1
AX-158404820	1	107.025	KV452713.1	9821168	1-scf00011	1
AX-157908528	1	107.025	KV452713.1	9822753	1-scf00011	1
AX-157908529	1	107.134	KV452713.1	9823666	1-scf00011	1
AX-157908530	1	107.134	KV452713.1	9824232	1-scf00011	1
AX-157908534	1	107.134	KV452713.1	9829312	1-scf00011	1
AX-157908540	1	107.134	KV452713.1	9834681	1-scf00011	1
AX-157755157	1	107.171	KV452713.1	9838768	1-scf00011	1
AX-157908543	1	107.244	KV452713.1	9840608	1-scf00011	1
AX-157908550	1	107.244	KV452713.1	9848523	1-scf00011	1
AX-157908553	1	107.395	KV452713.1	9850346	1-scf00011	1
AX-157706535	1	107.636	3ML01032399	274	scf08018_1	UN
AX-157908559	1	107.639	KV452713.1	9868410	1-scf00011	1
AX-157738524	1	107.639	KV452713.1	9870819	1-scf00011	1
AX-157708223	1	107.639	KV452713.1	9871099	1-scf00011	1
AX-157908565	1	107.639	KV452713.1	9879709	1-scf00011	1
AX-157908566	1	107.639	KV452713.1	9880266	1-scf00011	1
AX-157908571	1	107.639	KV452713.1	9883964	1-scf00011	1
AX-157908562	1	107.678	KV452713.1	9872040	1-scf00011	1
AX-157908570	1	107.678	KV452713.1	9883206	1-scf00011	1
AX-157908572	1	107.79	KV452713.1	9884785	1-scf00011	1
AX-157908573	1	107.79	KV452713.1	9885324	1-scf00011	1
AX-157908575	1	107.9	KV452713.1	9886723	1-scf00011	1
AX-157908576	1	107.9	KV452713.1	9892211	1-scf00011	1
AX-157741678	1	108.064	KV452713.1	9895189	1-scf00011	1
AX-157908579	1	108.228	KV452713.1	9896884	1-scf00011	1
AX-157740853	1	108.228	KV452713.1	9897273	1-scf00011	1
AX-157908583	1	108.228	KV452713.1	9902519	1-scf00011	1
AX-157908584	1	108.228	KV452713.1	9903542	1-scf00011	1
AX-157908586	1	108.228	KV452713.1	9906407	1-scf00011	1
AX-157812077	1	108.228	KV452713.1	9907994	1-scf00011	1
AX-157708524	1	108.228	KV452713.1	9908447	1-scf00011	1
AX-157908588	1	108.228	KV452713.1	9908858	1-scf00011	1
AX-157908589	1	108.228	KV452713.1	9910430	1-scf00011	1
AX-157908591	1	108.228	KV452713.1	9912642	1-scf00011	1
AX-157908597	1	108.757	KV452713.1	9919237	1-scf00011	1
AX-157908603	1	108.757	KV452713.1	9923549	1-scf00011	1
AX-157864445	1	108.776	KV452713.1	9920695	1-scf00011	1
AX-157908601	1	108.776	KV452713.1	9921457	1-scf00011	1
AX-157908618	1	108.776	KV452713.1	9943594	1-scf00011	1
AX-157908606	1	109.43	KV452713.1	9927472	1-scf00011	1
AX-157908607	1	109.43	KV452713.1	9929257	1-scf00011	1
AX-157908609	1	109.43	KV452713.1	9931897	1-scf00011	1

AX-157908610	1	109.43	KV452713.1	9933674	1-scf00011	1
AX-157908611	1	109.43	KV452713.1	9935025	1-scf00011	1
AX-157694127	1	109.43	KV452713.1	9935315	1-scf00011	1
AX-157908612	1	109.43	KV452713.1	9938221	1-scf00011	1
AX-157908617	1	109.43	KV452713.1	9942764	1-scf00011	1
AX-157853645	1	109.43	KV452713.1	9945220	1-scf00011	1
AX-157697396	1	109.43	KV452713.1	9947262	1-scf00011	1
AX-157908620	1	109.43	KV452713.1	9949312	1-scf00011	1
AX-157908623	1	109.43	KV452713.1	9954895	1-scf00011	1
AX-157743347	1	109.43	KV452713.1	9955232	1-scf00011	1
AX-157908631	1	109.43	KV452713.1	9969679	1-scf00011	1
AX-157908613	1	109.431	KV452713.1	9938899	1-scf00011	1
AX-157908621	1	109.431	KV452713.1	9949827	1-scf00011	1
AX-157740895	1	109.431	KV452713.1	9951763	1-scf00011	1
AX-157908622	1	109.434	KV452713.1	9952471	1-scf00011	1
AX-157908624	1	109.887	KV452713.1	9957481	1-scf00011	1
AX-157874530	1	109.89	KV452713.1	9962336	1-scf00011	1
AX-157908627	1	109.964	KV452713.1	9963098	1-scf00011	1
AX-157908629	1	109.966	KV452713.1	9964260	1-scf00011	1
AX-157908630	1	110.023	KV452713.1	9966640	1-scf00011	1
AX-157908634	1	110.116	KV452713.1	9972022	1-scf00011	1
AX-157908638	1	110.116	KV452713.1	9976611	1-scf00011	1
AX-157908642	1	110.116	KV452713.1	9980884	1-scf00011	1
AX-157908643	1	110.116	KV452713.1	9981530	1-scf00011	1
AX-157908645	1	110.116	KV452713.1	9983031	1-scf00011	1
AX-157908646	1	110.118	KV452713.1	9986287	1-scf00011	1
AX-157724378	1	110.523	KV452713.1	9987477	1-scf00011	1
AX-157908647	1	110.523	KV452713.1	9990104	1-scf00011	1
AX-157908649	1	110.523	KV452713.1	9990950	1-scf00011	1
AX-157725039	1	110.523	KV452713.1	9991609	1-scf00011	1
AX-157908650	1	110.523	KV452713.1	9992717	1-scf00011	1
AX-157908654	1	110.523	KV452713.1	9996510	1-scf00011	1
AX-157908656	1	110.523	KV452713.1	10001690	1-scf00011	1
AX-157883365	1	110.523	KV452713.1	10002913	1-scf00011	1
AX-157908660	1	110.554	KV452713.1	10005971	1-scf00011	1
AX-157908661	1	110.566	KV452713.1	10006331	1-scf00011	1
AX-157908662	1	110.633	KV452713.1	10007523	1-scf00011	1
AX-157908664	1	110.633	KV452713.1	10008566	1-scf00011	1
AX-157908665	1	110.633	KV452713.1	10009235	1-scf00011	1
AX-157908666	1	110.662	KV452713.1	10010264	1-scf00011	1
AX-157774741	1	110.702	KV452713.1	10011691	1-scf00011	1
AX-157908668	1	110.742	KV452713.1	10013600	1-scf00011	1
AX-157908672	1	110.742	KV452713.1	10019236	1-scf00011	1
AX-157908673	1	110.742	KV452713.1	10021217	1-scf00011	1
AX-157908651	1	110.851	KV452713.1	9993982	1-scf00011	1
AX-157908670	1	110.851	KV452713.1	10017270	1-scf00011	1
AX-157908676	1	110.851	KV452713.1	10025436	1-scf00011	1
AX-157710534	1	110.851	KV452713.1	10026955	1-scf00011	1
AX-157908677	1	110.851	KV452713.1	10027541	1-scf00011	1
AX-157908678	1	110.851	KV452713.1	10028303	1-scf00011	1
AX-157908680	1	110.851	KV452713.1	10031127	1-scf00011	1

AX-157908681	1	110.851	KV452713.1	10031226	1-scf00011	1
AX-157908685	1	110.851	KV452713.1	10034824	1-scf00011	1
AX-157908688	1	110.851	KV452713.1	10040776	1-scf00011	1
AX-157908689	1	110.861	KV452713.1	10041272	1-scf00011	1
AX-157908691	1	111.397	KV452713.1	10042678	1-scf00011	1
AX-157908695	1	111.397	KV452713.1	10047448	1-scf00011	1
AX-157908697	1	111.397	KV452713.1	10048160	1-scf00011	1
AX-157708799	1	111.397	KV452713.1	10048510	1-scf00011	1
AX-157708800	1	111.397	KV452713.1	10048602	1-scf00011	1
AX-157908699	1	111.397	KV452713.1	10049894	1-scf00011	1
AX-157908701	1	111.397	KV452713.1	10052311	1-scf00011	1
AX-157908703	1	111.397	KV452713.1	10054064	1-scf00011	1
AX-157908704	1	111.397	KV452713.1	10055092	1-scf00011	1
AX-157908705	1	111.397	KV452713.1	10057243	1-scf00011	1
AX-157908707	1	111.398	KV452713.1	10061365	1-scf00011	1
AX-157908709	1	111.398	KV452713.1	10062658	1-scf00011	1
AX-157908711	1	111.725	KV452713.1	10065472	1-scf00011	1
AX-157908715	1	111.769	KV452713.1	10068078	1-scf00011	1
AX-157812078	1	111.769	KV452713.1	10087078	1-scf00011	1
AX-157908712	1	111.834	KV452713.1	10066758	1-scf00011	1
AX-157908717	1	111.834	KV452713.1	10073389	1-scf00011	1
AX-157908718	1	111.834	KV452713.1	10074237	1-scf00011	1
AX-157908719	1	111.834	KV452713.1	10074547	1-scf00011	1
AX-157908720	1	111.834	KV452713.1	10075299	1-scf00011	1
AX-157908723	1	111.834	KV452713.1	10079227	1-scf00011	1
AX-157908724	1	111.834	KV452713.1	10079545	1-scf00011	1
AX-157908725	1	111.834	KV452713.1	10080131	1-scf00011	1
AX-157908726	1	111.834	KV452713.1	10081053	1-scf00011	1
AX-157908727	1	111.834	KV452713.1	10081735	1-scf00011	1
AX-157908728	1	111.834	KV452713.1	10082354	1-scf00011	1
AX-157908731	1	111.834	KV452713.1	10084739	1-scf00011	1
AX-157713446	1	111.834	KV452713.1	10085742	1-scf00011	1
AX-157746433	1	111.834	KV452713.1	10086822	1-scf00011	1
AX-157908735	1	112.052	KV452713.1	10093457	1-scf00011	1
AX-157908736	1	112.052	KV452713.1	10094008	1-scf00011	1
AX-157908737	1	112.052	KV452713.1	10094510	1-scf00011	1
AX-157908740	1	112.052	KV452713.1	10101653	1-scf00011	1
AX-157908743	1	112.052	KV452713.1	10104240	1-scf00011	1
AX-157908747	1	112.052	KV452713.1	10109025	1-scf00011	1
AX-157908750	1	112.052	KV452713.1	10112540	1-scf00011	1
AX-157908751	1	112.052	KV452713.1	10114663	1-scf00011	1
AX-157908753	1	112.052	KV452713.1	10117433	1-scf00011	1
AX-157908754	1	112.052	KV452713.1	10118593	1-scf00011	1
AX-157908756	1	112.353	KV452713.1	10120773	1-scf00011	1
AX-157908760	1	112.372	KV452713.1	10129495	1-scf00011	1
AX-157705776	1	112.372	3ML01030844	820	scf06448_1	UN
AX-157908763	1	112.767	KV452713.1	10140186	1-scf00011	1
AX-157908765	1	112.818	KV452713.1	10142724	1-scf00011	1
AX-157749275	1	112.818	KV452713.1	10150683	1-scf00011	1
AX-157908773	1	112.818	KV452713.1	10151284	1-scf00011	1
AX-157908774	1	112.818	KV452713.1	10152648	1-scf00011	1

AX-157908775	1	112.818	KV452713.1	10155823	1-scf00011	1
AX-157883368	1	112.818	KV452713.1	10155957	1-scf00011	1
AX-157908776	1	112.818	KV452713.1	10156010	1-scf00011	1
AX-157908777	1	112.818	KV452713.1	10157179	1-scf00011	1
AX-157908778	1	112.818	KV452713.1	10158436	1-scf00011	1
AX-157908780	1	112.818	KV452713.1	10158958	1-scf00011	1
AX-157908781	1	112.818	KV452713.1	10159165	1-scf00011	1
AX-157908785	1	112.818	KV452713.1	10162490	1-scf00011	1
AX-157726700	1	112.818	KV452713.1	10165464	1-scf00011	1
AX-157908788	1	112.818	KV452713.1	10168953	1-scf00011	1
AX-157908792	1	112.818	KV452713.1	10171889	1-scf00011	1
AX-157908789	1	112.927	KV452713.1	10169385	1-scf00011	1
AX-157908790	1	112.927	KV452713.1	10169958	1-scf00011	1
AX-157775562	1	112.927	KV452713.1	10172004	1-scf00011	1
AX-157908793	1	112.927	KV452713.1	10172362	1-scf00011	1
AX-157908794	1	112.927	KV452713.1	10175136	1-scf00011	1
AX-157908795	1	112.944	KV452713.1	10176292	1-scf00011	1
AX-157908796	1	112.978	KV452713.1	10178062	1-scf00011	1
AX-157908797	1	112.991	KV452713.1	10178460	1-scf00011	1
AX-157908815	1	112.991	KV452713.1	10194010	1-scf00011	1
AX-157874537	1	112.991	KV452713.1	10197885	1-scf00011	1
AX-157908802	1	113.002	KV452713.1	10184542	1-scf00011	1
AX-157908805	1	113.002	KV452713.1	10186684	1-scf00011	1
AX-157908806	1	113.019	KV452713.1	10187166	1-scf00011	1
AX-157908807	1	113.036	KV452713.1	10189352	1-scf00011	1
AX-157908808	1	113.036	KV452713.1	10189968	1-scf00011	1
AX-157908810	1	113.036	KV452713.1	10191083	1-scf00011	1
AX-157908812	1	113.085	KV452713.1	10191713	1-scf00011	1
AX-158414761	1	113.105	KV452713.1	10192101	1-scf00011	1
AX-157908787	1	113.132	KV452713.1	10165747	1-scf00011	1
AX-157908817	1	113.132	KV452713.1	10194765	1-scf00011	1
AX-157908798	1	113.146	KV452713.1	10178910	1-scf00011	1
AX-157908823	1	113.146	KV452713.1	10198694	1-scf00011	1
AX-157908824	1	113.146	KV452713.1	10199702	1-scf00011	1
AX-157908825	1	113.146	KV452713.1	10202437	1-scf00011	1
AX-157908826	1	113.146	KV452713.1	10202719	1-scf00011	1
AX-157908831	1	113.539	KV452713.1	10212513	1-scf00011	1
AX-158404827	1	113.671	KV452713.1	10213207	1-scf00011	1
AX-157908833	1	113.802	KV452713.1	10214784	1-scf00011	1
AX-157741278	1	113.803	KV452713.1	10216646	1-scf00011	1
AX-157908835	1	113.803	KV452713.1	10217203	1-scf00011	1
AX-157908838	1	113.907	KV452713.1	10221243	1-scf00011	1
AX-157908840	1	113.907	KV452713.1	10222296	1-scf00011	1
AX-157908841	1	114.021	KV452713.1	10222972	1-scf00011	1
AX-157908843	1	114.24	KV452713.1	10224608	1-scf00011	1
AX-157908844	1	114.24	KV452713.1	10224948	1-scf00011	1
AX-157739622	1	114.24	KV452713.1	10225357	1-scf00011	1
AX-157908845	1	114.24	KV452713.1	10225488	1-scf00011	1
AX-157908847	1	114.349	KV452713.1	10226051	1-scf00011	1
AX-157908848	1	114.349	KV452713.1	10226855	1-scf00011	1
AX-157908849	1	114.349	KV452713.1	10227721	1-scf00011	1

AX-157908851	1	114.349	KV452713.1	10228948	1-scf00011	1
AX-157908852	1	114.349	KV452713.1	10230796	1-scf00011	1
AX-157908853	1	114.349	KV452713.1	10231716	1-scf00011	1
AX-157908890	1	114.349	KV452713.1	10275499	1-scf00011	1
AX-157908854	1	114.379	KV452713.1	10232569	1-scf00011	1
AX-157908858	1	114.409	KV452713.1	10236167	1-scf00011	1
AX-157772453	1	114.459	KV452713.1	10238800	1-scf00011	1
AX-157908861	1	114.459	KV452713.1	10239161	1-scf00011	1
AX-157908865	1	114.459	KV452713.1	10244755	1-scf00011	1
AX-157908866	1	114.459	KV452713.1	10245474	1-scf00011	1
AX-157908867	1	114.568	KV452713.1	10247995	1-scf00011	1
AX-157908870	1	114.677	KV452713.1	10252047	1-scf00011	1
AX-157908871	1	114.677	KV452713.1	10257603	1-scf00011	1
AX-157739364	1	114.677	KV452713.1	10258338	1-scf00011	1
AX-157755932	1	114.677	KV452713.1	10259111	1-scf00011	1
AX-157750330	1	114.677	KV452713.1	10259798	1-scf00011	1
AX-157908872	1	114.677	KV452713.1	10260446	1-scf00011	1
AX-157908873	1	114.677	KV452713.1	10260987	1-scf00011	1
AX-157908876	1	114.677	KV452713.1	10262948	1-scf00011	1
AX-157908877	1	114.677	KV452713.1	10263459	1-scf00011	1
AX-157908903	1	114.677	KV452713.1	10287740	1-scf00011	1
AX-157908879	1	114.786	KV452713.1	10266175	1-scf00011	1
AX-157908882	1	114.786	KV452713.1	10269078	1-scf00011	1
AX-157908886	1	114.786	KV452713.1	10271864	1-scf00011	1
AX-157908887	1	114.786	KV452713.1	10273112	1-scf00011	1
AX-157908891	1	114.786	KV452713.1	10275616	1-scf00011	1
AX-157908892	1	114.786	KV452713.1	10277599	1-scf00011	1
AX-157908895	1	114.786	KV452713.1	10280193	1-scf00011	1
AX-157908896	1	114.786	KV452713.1	10282235	1-scf00011	1
AX-157908897	1	114.786	KV452713.1	10282690	1-scf00011	1
AX-157908898	1	114.786	KV452713.1	10283204	1-scf00011	1
AX-157908902	1	114.786	KV452713.1	10286700	1-scf00011	1
AX-157908908	1	114.786	KV452713.1	10293659	1-scf00011	1
AX-157908909	1	114.786	KV452713.1	10294546	1-scf00011	1
AX-157908910	1	114.786	KV452713.1	10296275	1-scf00011	1
AX-157908915	1	114.786	KV452713.1	10302497	1-scf00011	1
AX-157908936	1	114.786	KV452713.1	10327570	1-scf00011	1
AX-157908850	1	115.005	KV452713.1	10228277	1-scf00011	1
AX-157908900	1	115.005	KV452713.1	10285469	1-scf00011	1
AX-157908906	1	115.005	KV452713.1	10291931	1-scf00011	1
AX-157908907	1	115.005	KV452713.1	10292686	1-scf00011	1
AX-157908922	1	115.005	KV452713.1	10311707	1-scf00011	1
AX-157758598	1	115.005	KV452713.1	10315037	1-scf00011	1
AX-157908924	1	115.005	KV452713.1	10316899	1-scf00011	1
AX-157908925	1	115.005	KV452713.1	10317526	1-scf00011	1
AX-157908929	1	115.005	KV452713.1	10322091	1-scf00011	1
AX-157908930	1	115.005	KV452713.1	10322806	1-scf00011	1
AX-157908934	1	115.005	KV452713.1	10325519	1-scf00011	1
AX-157908937	1	115.005	KV452713.1	10329717	1-scf00011	1
AX-157908938	1	115.005	KV452713.1	10330843	1-scf00011	1
AX-157908943	1	115.005	KV452713.1	10338827	1-scf00011	1

AX-157908889	1	115.114	KV452713.1	10274858	1-scf00011	1
AX-157908940	1	115.114	KV452713.1	10334394	1-scf00011	1
AX-157908942	1	115.114	KV452713.1	10337606	1-scf00011	1
AX-157908949	1	115.22	KV452713.1	10344856	1-scf00011	1
AX-157908916	1	115.333	KV452713.1	10303303	1-scf00011	1
AX-157774455	1	115.333	KV452713.1	10328796	1-scf00011	1
AX-157883372	1	115.333	KV452713.1	10346350	1-scf00011	1
AX-157908955	1	115.333	KV452713.1	10356352	1-scf00011	1
AX-157908957	1	115.333	KV452713.1	10357215	1-scf00011	1
AX-157908958	1	115.333	KV452713.1	10359008	1-scf00011	1
AX-157908959	1	115.333	KV452713.1	10359506	1-scf00011	1
AX-157908960	1	115.333	KV452713.1	10360126	1-scf00011	1
AX-158389540	1	115.333	KV452713.1	10361516	1-scf00011	1
AX-157908969	1	115.333	KV452713.1	10370797	1-scf00011	1
AX-157908961	1	115.396	KV452713.1	10361557	1-scf00011	1
AX-157908964	1	115.442	KV452713.1	10363404	1-scf00011	1
AX-157908965	1	115.442	KV452713.1	10365584	1-scf00011	1
AX-157908968	1	115.442	KV452713.1	10370134	1-scf00011	1
AX-157770946	1	115.442	KV452713.1	10379615	1-scf00011	1
AX-157864446	1	115.442	KV452713.1	10397587	1-scf00011	1
AX-157909005	1	115.442	KV452713.1	10414925	1-scf00011	1
AX-157909013	1	115.442	KV452713.1	10423727	1-scf00011	1
AX-157908976	1	115.465	KV452713.1	10379087	1-scf00011	1
AX-157908980	1	115.511	KV452713.1	10385980	1-scf00011	1
AX-157908981	1	115.551	KV452713.1	10386371	1-scf00011	1
AX-157908982	1	115.686	KV452713.1	10387494	1-scf00011	1
AX-157747484	1	115.686	KV452713.1	10413676	1-scf00011	1
AX-157908945	1	115.77	KV452713.1	10341286	1-scf00011	1
AX-157908962	1	115.77	KV452713.1	10362174	1-scf00011	1
AX-157908987	1	115.77	KV452713.1	10393910	1-scf00011	1
AX-157908989	1	115.77	KV452713.1	10395993	1-scf00011	1
AX-157908993	1	115.77	KV452713.1	10398914	1-scf00011	1
AX-157874539	1	115.77	KV452713.1	10399319	1-scf00011	1
AX-157908994	1	115.77	KV452713.1	10400577	1-scf00011	1
AX-157874540	1	115.77	KV452713.1	10404326	1-scf00011	1
AX-157908999	1	115.77	KV452713.1	10405679	1-scf00011	1
AX-157909000	1	115.77	KV452713.1	10407987	1-scf00011	1
AX-157909001	1	115.77	KV452713.1	10411178	1-scf00011	1
AX-157909002	1	115.77	KV452713.1	10411843	1-scf00011	1
AX-157909003	1	115.77	KV452713.1	10412216	1-scf00011	1
AX-157909004	1	115.77	KV452713.1	10413250	1-scf00011	1
AX-157909006	1	115.77	KV452713.1	10415644	1-scf00011	1
AX-157909008	1	115.77	KV452713.1	10416456	1-scf00011	1
AX-157909009	1	115.77	KV452713.1	10420337	1-scf00011	1
AX-157812082	1	115.77	KV452713.1	10427838	1-scf00011	1
AX-157812086	1	115.77	KV452713.1	10441535	1-scf00011	1
AX-157909027	1	115.77	KV452713.1	10442613	1-scf00011	1
AX-157909030	1	115.77	KV452713.1	10446980	1-scf00011	1
AX-157909036	1	115.77	KV452713.1	10451278	1-scf00011	1
AX-157909010	1	115.921	KV452713.1	10420687	1-scf00011	1
AX-157908983	1	115.969	KV452713.1	10388932	1-scf00011	1

AX-157909012	1	115.969	KV452713.1	10422410	1-scf00011	1
AX-157756039	1	116.036	KV452713.1	10425227	1-scf00011	1
AX-157909021	1	116.081	KV452713.1	10432012	1-scf00011	1
AX-157909023	1	116.135	KV452713.1	10435330	1-scf00011	1
AX-157777486	1	116.135	KV452713.1	10463469	1-scf00011	1
AX-157773008	1	116.135	KV452713.1	10486182	1-scf00011	1
AX-157909024	1	116.136	KV452713.1	10438838	1-scf00011	1
AX-157909028	1	116.45	KV452713.1	10444162	1-scf00011	1
AX-157909034	1	116.451	KV452713.1	10450476	1-scf00011	1
AX-157909040	1	116.451	KV452713.1	10457358	1-scf00011	1
AX-157909041	1	116.451	KV452713.1	10458056	1-scf00011	1
AX-157909042	1	116.451	KV452713.1	10458534	1-scf00011	1
AX-157909044	1	116.451	KV452713.1	10461133	1-scf00011	1
AX-157909045	1	116.451	KV452713.1	10462548	1-scf00011	1
AX-157909053	1	116.451	KV452713.1	10471379	1-scf00011	1
AX-157812090	1	116.451	KV452713.1	10471611	1-scf00011	1
AX-157750876	1	116.671	KV452713.1	10463624	1-scf00011	1
AX-157812089	1	116.765	KV452713.1	10469708	1-scf00011	1
AX-158379210	1	116.828	KV452713.1	10487710	1-scf00011	1
AX-157909061	1	116.852	KV452713.1	10488375	1-scf00011	1
AX-157812088	1	116.915	KV452713.1	10463505	1-scf00011	1
AX-157745509	1	116.915	KV452713.1	10489301	1-scf00011	1
AX-157909063	1	116.915	KV452713.1	10493107	1-scf00011	1
AX-157909019	1	117.008	KV452713.1	10429052	1-scf00011	1
AX-157909067	1	117.008	KV452713.1	10497422	1-scf00011	1
AX-157717728	1	117.008	KV452713.1	10537684	1-scf00011	1
AX-157909068	1	117.082	KV452713.1	10499246	1-scf00011	1
AX-157909069	1	117.082	KV452713.1	10501268	1-scf00011	1
AX-157909070	1	117.082	KV452713.1	10501530	1-scf00011	1
AX-157909071	1	117.082	KV452713.1	10503924	1-scf00011	1
AX-157909072	1	117.082	KV452713.1	10504562	1-scf00011	1
AX-157909074	1	117.082	KV452713.1	10506414	1-scf00011	1
AX-157909080	1	117.082	KV452713.1	10514084	1-scf00011	1
AX-157909150	1	117.082	KV452713.1	10588020	1-scf00011	1
AX-157908918	1	117.191	KV452713.1	10304830	1-scf00011	1
AX-157909054	1	117.191	KV452713.1	10473327	1-scf00011	1
AX-157909084	1	117.191	KV452713.1	10517585	1-scf00011	1
AX-157909086	1	117.191	KV452713.1	10519007	1-scf00011	1
AX-157909087	1	117.191	KV452713.1	10519908	1-scf00011	1
AX-157744451	1	117.191	KV452713.1	10521100	1-scf00011	1
AX-157909088	1	117.191	KV452713.1	10523751	1-scf00011	1
AX-157739371	1	117.298	KV452713.1	10529310	1-scf00011	1
AX-157909159	1	117.298	KV452713.1	10596714	1-scf00011	1
AX-157812094	1	117.41	KV452713.1	10533493	1-scf00011	1
AX-157909096	1	117.41	KV452713.1	10535297	1-scf00011	1
AX-157722901	1	117.519	KV452713.1	10540802	1-scf00011	1
AX-157909101	1	117.519	KV452713.1	10542687	1-scf00011	1
AX-157909102	1	117.519	KV452713.1	10543367	1-scf00011	1
AX-157909140	1	117.769	KV452713.1	10582110	1-scf00011	1
AX-157909051	1	117.956	KV452713.1	10470187	1-scf00011	1
AX-157909052	1	117.956	KV452713.1	10470800	1-scf00011	1

AX-157812091	1	117.956	KV452713.1	10472126	1-scf00011	1
AX-157909057	1	117.956	KV452713.1	10477260	1-scf00011	1
AX-157909104	1	117.956	KV452713.1	10544808	1-scf00011	1
AX-157909141	1	117.956	KV452713.1	10582587	1-scf00011	1
AX-157909149	1	117.956	KV452713.1	10587028	1-scf00011	1
AX-157874545	1	117.956	KV452713.1	10587328	1-scf00011	1
AX-157909152	1	117.956	KV452713.1	10589181	1-scf00011	1
AX-157909154	1	117.956	KV452713.1	10590261	1-scf00011	1
AX-157909156	1	117.956	KV452713.1	10592264	1-scf00011	1
AX-157909157	1	117.956	KV452713.1	10593918	1-scf00011	1
AX-157909158	1	117.956	KV452713.1	10594734	1-scf00011	1
AX-157812095	1	117.956	KV452713.1	10601601	1-scf00011	1
AX-157745829	1	117.956	KV452713.1	10602840	1-scf00011	1
AX-157812096	1	117.956	KV452713.1	10603070	1-scf00011	1
AX-157812098	1	117.956	KV452713.1	10605736	1-scf00011	1
AX-157909162	1	117.956	KV452713.1	10606235	1-scf00011	1
AX-157909163	1	117.956	KV452713.1	10606750	1-scf00011	1
AX-157869057	1	117.956	KV452713.1	10607463	1-scf00011	1
AX-157909095	1	118.066	KV452713.1	10534737	1-scf00011	1
AX-157909146	1	118.066	KV452713.1	10584729	1-scf00011	1
AX-157775072	1	118.066	KV452713.1	10609199	1-scf00011	1
AX-157909169	1	118.066	KV452713.1	10613585	1-scf00011	1
AX-157909172	1	118.066	KV452713.1	10616798	1-scf00011	1
AX-158421843	1	118.066	KV452713.1	10620932	1-scf00011	1
AX-158404834	1	118.066	KV452713.1	10620970	1-scf00011	1
AX-157812099	1	118.066	KV452713.1	10621643	1-scf00011	1
AX-157812100	1	118.066	KV452713.1	10626180	1-scf00011	1
AX-157909177	1	118.066	KV452713.1	10627917	1-scf00011	1
AX-157909178	1	118.066	KV452713.1	10629335	1-scf00011	1
AX-157909181	1	118.066	KV452713.1	10631629	1-scf00011	1
AX-157909183	1	118.066	KV452713.1	10633462	1-scf00011	1
AX-157909186	1	118.066	KV452713.1	10644985	1-scf00011	1
AX-157772362	1	118.175	KV452713.1	10646032	1-scf00011	1
AX-157909189	1	118.175	KV452713.1	10653015	1-scf00011	1
AX-157909191	1	118.284	KV452713.1	10657799	1-scf00011	1
AX-157909194	1	118.284	KV452713.1	10661102	1-scf00011	1
AX-157909195	1	118.393	KV452713.1	10661529	1-scf00011	1
AX-157909202	1	118.461	KV452713.1	10672278	1-scf00011	1
AX-158389544	1	118.461	KV452715.1	53120	1-scf00013	1
AX-157682307	1	118.461	KV452714.1	5209	1-scf00014	1
AX-157682314	1	118.461	KV452714.1	9821	1-scf00014	1
AX-157909200	1	118.503	KV452713.1	10670989	1-scf00011	1
AX-157909205	1	118.503	KV452715.1	1961	1-scf00013	1
AX-157909207	1	118.503	KV452715.1	3708	1-scf00013	1
AX-157909208	1	118.503	KV452715.1	5577	1-scf00013	1
AX-157909213	1	118.503	KV452715.1	10537	1-scf00013	1
AX-157909221	1	118.503	KV452715.1	16462	1-scf00013	1
AX-157812103	1	118.503	KV452715.1	27088	1-scf00013	1
AX-157682308	1	118.503	KV452714.1	5806	1-scf00014	1
AX-157682309	1	118.503	KV452714.1	5984	1-scf00014	1
AX-157682310	1	118.503	KV452714.1	7542	1-scf00014	1

AX-157682313	1	118.503	KV452714.1	9165	1-scf00014	1
AX-157682316	1	118.503	KV452714.1	10384	1-scf00014	1
AX-157909973	1	118.503	KV452717.1	443169	1-scf00018	1
AX-157909216	1	118.52	KV452715.1	12702	1-scf00013	1
AX-157909222	1	118.539	KV452715.1	16688	1-scf00013	1
AX-157909223	1	118.558	KV452715.1	17161	1-scf00013	1
AX-157909225	1	118.582	KV452715.1	21230	1-scf00013	1
AX-157753994	1	118.612	KV452715.1	1375	1-scf00013	1
AX-157909215	1	118.612	KV452715.1	12117	1-scf00013	1
AX-157909227	1	118.612	KV452715.1	25085	1-scf00013	1
AX-157909230	1	118.612	KV452715.1	26511	1-scf00013	1
AX-157909233	1	118.612	KV452715.1	28340	1-scf00013	1
AX-157909234	1	118.612	KV452715.1	30287	1-scf00013	1
AX-157812104	1	118.612	KV452715.1	30990	1-scf00013	1
AX-157883373	1	118.612	KV452715.1	31644	1-scf00013	1
AX-157909244	1	118.612	KV452715.1	39167	1-scf00013	1
AX-157909246	1	118.612	KV452715.1	41083	1-scf00013	1
AX-157909247	1	118.612	KV452715.1	41805	1-scf00013	1
AX-157756718	1	118.612	KV452715.1	46790	1-scf00013	1
AX-157909249	1	118.612	KV452715.1	50522	1-scf00013	1
AX-157909250	1	118.612	KV452715.1	51151	1-scf00013	1
AX-157749761	1	118.612	KV452715.1	57151	1-scf00013	1
AX-157909255	1	118.629	KV452715.1	59141	1-scf00013	1
AX-158389545	1	118.646	KV452715.1	69523	1-scf00013	1
AX-157909211	1	118.728	KV452715.1	8545	1-scf00013	1
AX-157909232	1	118.728	KV452715.1	27940	1-scf00013	1
AX-157909264	1	118.728	KV452715.1	70505	1-scf00013	1
AX-157909265	1	118.728	KV452715.1	70946	1-scf00013	1
AX-157909266	1	118.728	KV452715.1	71596	1-scf00013	1
AX-157909268	1	118.728	KV452715.1	76189	1-scf00013	1
AX-157909269	1	118.728	KV452715.1	76922	1-scf00013	1
AX-157909270	1	118.728	KV452715.1	80009	1-scf00013	1
AX-157682312	1	118.728	KV452714.1	8431	1-scf00014	1
AX-157710678	1	118.867	KV452715.1	80185	1-scf00013	1
AX-157909334	1	119.925	KV452716.1	153172	1-scf00015	1
AX-157883378	1	119.925	KV452719.1	137859	1-scf00016	1
AX-157909654	1	119.925	KV452718.1	263453	1-scf00017	1
AX-157909676	1	119.925	KV452717.1	21000	1-scf00018	1
AX-157909678	1	119.925	KV452717.1	23754	1-scf00018	1
AX-157909679	1	119.925	KV452717.1	28733	1-scf00018	1
AX-157909687	1	119.925	KV452717.1	50816	1-scf00018	1
AX-157909688	1	119.925	KV452717.1	51378	1-scf00018	1
AX-157909689	1	119.925	KV452717.1	52411	1-scf00018	1
AX-157716888	1	119.925	KV452717.1	52503	1-scf00018	1
AX-157909691	1	119.925	KV452717.1	54150	1-scf00018	1
AX-157909694	1	119.925	KV452717.1	61543	1-scf00018	1
AX-157909695	1	119.925	KV452717.1	65374	1-scf00018	1
AX-157909697	1	119.925	KV452717.1	66170	1-scf00018	1
AX-157909698	1	119.925	KV452717.1	66813	1-scf00018	1
AX-157909701	1	119.925	KV452717.1	71172	1-scf00018	1
AX-157812149	1	119.925	KV452717.1	71583	1-scf00018	1

AX-157909702	1	119.925	KV452717.1	75218	1-scf00018	1
AX-157909703	1	119.925	KV452717.1	75655	1-scf00018	1
AX-157909706	1	119.925	KV452717.1	78535	1-scf00018	1
AX-157689653	1	119.925	3ML01031131	736	scf06737_1	UN
AX-157853658	1	119.981	KV452717.1	89094	1-scf00018	1
AX-157909712	1	120.035	KV452717.1	90541	1-scf00018	1
AX-157909715	1	120.035	KV452717.1	93343	1-scf00018	1
AX-157869063	1	120.035	KV452717.1	93583	1-scf00018	1
AX-157909716	1	120.035	KV452717.1	95035	1-scf00018	1
AX-157909717	1	120.035	KV452717.1	95472	1-scf00018	1
AX-157909718	1	120.035	KV452717.1	96563	1-scf00018	1
AX-157909719	1	120.035	KV452717.1	97017	1-scf00018	1
AX-157909729	1	120.035	KV452717.1	116137	1-scf00018	1
AX-157909732	1	120.035	KV452717.1	118867	1-scf00018	1
AX-157909733	1	120.035	KV452717.1	120724	1-scf00018	1
AX-157909734	1	120.035	KV452717.1	122792	1-scf00018	1
AX-157909735	1	120.035	KV452717.1	124269	1-scf00018	1
AX-157771411	1	120.035	KV452717.1	125350	1-scf00018	1
AX-157909737	1	120.035	KV452717.1	127184	1-scf00018	1
AX-157909738	1	120.035	KV452717.1	127680	1-scf00018	1
AX-157909739	1	120.035	KV452717.1	128646	1-scf00018	1
AX-157909740	1	120.035	KV452717.1	132330	1-scf00018	1
AX-157724602	1	120.035	KV452717.1	134548	1-scf00018	1
AX-157864455	1	120.035	KV452717.1	135073	1-scf00018	1
AX-157909743	1	120.035	KV452717.1	136216	1-scf00018	1
AX-157773634	1	120.035	KV452717.1	136771	1-scf00018	1
AX-157909746	1	120.035	KV452717.1	137765	1-scf00018	1
AX-157909792	1	120.035	KV452717.1	224929	1-scf00018	1
AX-157909793	1	120.035	KV452717.1	225280	1-scf00018	1
AX-157812165	1	120.035	KV452717.1	237934	1-scf00018	1
AX-157909749	1	120.065	KV452717.1	140754	1-scf00018	1
AX-157909751	1	120.094	KV452717.1	142259	1-scf00018	1
AX-157909754	1	120.122	KV452717.1	152415	1-scf00018	1
AX-157812153	1	120.144	KV452717.1	155415	1-scf00018	1
AX-157909758	1	120.144	KV452717.1	162936	1-scf00018	1
AX-157853659	1	120.144	KV452717.1	167071	1-scf00018	1
AX-157909759	1	120.144	KV452717.1	167283	1-scf00018	1
AX-157909760	1	120.144	KV452717.1	172770	1-scf00018	1
AX-157909761	1	120.144	KV452717.1	178620	1-scf00018	1
AX-157812155	1	120.144	KV452717.1	186612	1-scf00018	1
AX-157909776	1	120.144	KV452717.1	204295	1-scf00018	1
AX-157909783	1	120.144	KV452717.1	213236	1-scf00018	1
AX-157909787	1	120.144	KV452717.1	218357	1-scf00018	1
AX-157909788	1	120.144	KV452717.1	219627	1-scf00018	1
AX-157909791	1	120.144	KV452717.1	222862	1-scf00018	1
AX-157812160	1	120.144	KV452717.1	224882	1-scf00018	1
AX-157812161	1	120.144	KV452717.1	224967	1-scf00018	1
AX-157812162	1	120.144	KV452717.1	225031	1-scf00018	1
AX-157909795	1	120.144	KV452717.1	233312	1-scf00018	1
AX-157751950	1	120.144	KV452717.1	234981	1-scf00018	1
AX-157909799	1	120.144	KV452717.1	245874	1-scf00018	1

AX-157909886	1	120.144	KV452717.1	331166	1-scf00018	1
AX-157909898	1	120.144	KV452717.1	344850	1-scf00018	1
AX-157812168	1	120.144	KV452717.1	346280	1-scf00018	1
AX-157909909	1	120.144	KV452717.1	359369	1-scf00018	1
AX-157680518	1	120.253	KV452714.1	518	1-scf00014	1
AX-157721864	1	120.253	KV452716.1	42666	1-scf00015	1
AX-157909343	1	120.253	KV452716.1	172773	1-scf00015	1
AX-157909392	1	120.253	KV452716.1	272820	1-scf00015	1
AX-157909416	1	120.253	KV452719.1	110873	1-scf00016	1
AX-157909419	1	120.253	KV452719.1	117506	1-scf00016	1
AX-157909436	1	120.253	KV452719.1	140491	1-scf00016	1
AX-157771125	1	120.253	KV452719.1	146989	1-scf00016	1
AX-157864451	1	120.253	KV452719.1	299549	1-scf00016	1
AX-157909510	1	120.253	KV452719.1	384261	1-scf00016	1
AX-157909511	1	120.253	KV452719.1	390647	1-scf00016	1
AX-157710703	1	120.253	KV452719.1	391004	1-scf00016	1
AX-157909542	1	120.253	KV452719.1	507740	1-scf00016	1
AX-157909550	1	120.253	KV452719.1	540344	1-scf00016	1
AX-157909557	1	120.253	KV452719.1	560675	1-scf00016	1
AX-157909565	1	120.253	KV452719.1	623033	1-scf00016	1
AX-157909566	1	120.253	KV452719.1	624985	1-scf00016	1
AX-157909570	1	120.253	KV452719.1	633141	1-scf00016	1
AX-157909576	1	120.253	KV452719.1	654169	1-scf00016	1
AX-157812134	1	120.253	KV452719.1	663198	1-scf00016	1
AX-157909748	1	120.253	KV452717.1	139912	1-scf00018	1
AX-157909780	1	120.253	KV452717.1	210995	1-scf00018	1
AX-157812158	1	120.253	KV452717.1	212483	1-scf00018	1
AX-157909785	1	120.253	KV452717.1	215889	1-scf00018	1
AX-157909786	1	120.253	KV452717.1	217101	1-scf00018	1
AX-157812163	1	120.253	KV452717.1	232401	1-scf00018	1
AX-157909796	1	120.253	KV452717.1	236784	1-scf00018	1
AX-157715715	1	120.253	KV452717.1	244352	1-scf00018	1
AX-157772818	1	120.253	KV452717.1	249182	1-scf00018	1
AX-157909804	1	120.253	KV452717.1	249825	1-scf00018	1
AX-157909806	1	120.253	KV452717.1	252705	1-scf00018	1
AX-157909808	1	120.253	KV452717.1	255844	1-scf00018	1
AX-157812166	1	120.253	KV452717.1	259186	1-scf00018	1
AX-157909812	1	120.253	KV452717.1	259303	1-scf00018	1
AX-157909813	1	120.253	KV452717.1	259744	1-scf00018	1
AX-157909816	1	120.253	KV452717.1	262018	1-scf00018	1
AX-157909817	1	120.253	KV452717.1	263672	1-scf00018	1
AX-157909820	1	120.253	KV452717.1	266125	1-scf00018	1
AX-157909822	1	120.253	KV452717.1	267881	1-scf00018	1
AX-157909824	1	120.253	KV452717.1	269168	1-scf00018	1
AX-157909826	1	120.253	KV452717.1	270119	1-scf00018	1
AX-157909828	1	120.253	KV452717.1	271385	1-scf00018	1
AX-157909835	1	120.253	KV452717.1	274906	1-scf00018	1
AX-157752702	1	120.253	KV452717.1	278050	1-scf00018	1
AX-157909837	1	120.253	KV452717.1	278255	1-scf00018	1
AX-157909838	1	120.253	KV452717.1	279256	1-scf00018	1
AX-157909839	1	120.253	KV452717.1	279498	1-scf00018	1

AX-157909840	1	120.253	KV452717.1	279893	1-scf00018	1
AX-157909841	1	120.253	KV452717.1	280470	1-scf00018	1
AX-157909842	1	120.253	KV452717.1	282992	1-scf00018	1
AX-157909844	1	120.253	KV452717.1	285129	1-scf00018	1
AX-157909845	1	120.253	KV452717.1	286854	1-scf00018	1
AX-157909846	1	120.253	KV452717.1	287160	1-scf00018	1
AX-157909847	1	120.253	KV452717.1	288445	1-scf00018	1
AX-157909848	1	120.253	KV452717.1	289500	1-scf00018	1
AX-157909851	1	120.253	KV452717.1	292233	1-scf00018	1
AX-157909852	1	120.253	KV452717.1	292514	1-scf00018	1
AX-157909858	1	120.253	KV452717.1	295942	1-scf00018	1
AX-157725914	1	120.253	KV452717.1	299235	1-scf00018	1
AX-157909861	1	120.253	KV452717.1	299331	1-scf00018	1
AX-157909862	1	120.253	KV452717.1	299649	1-scf00018	1
AX-157883381	1	120.253	KV452717.1	300135	1-scf00018	1
AX-157909865	1	120.253	KV452717.1	304806	1-scf00018	1
AX-157909867	1	120.253	KV452717.1	308418	1-scf00018	1
AX-157909868	1	120.253	KV452717.1	309050	1-scf00018	1
AX-157909869	1	120.253	KV452717.1	310430	1-scf00018	1
AX-157909871	1	120.253	KV452717.1	311560	1-scf00018	1
AX-157740083	1	120.253	KV452717.1	312544	1-scf00018	1
AX-157812167	1	120.253	KV452717.1	312962	1-scf00018	1
AX-157909872	1	120.253	KV452717.1	313395	1-scf00018	1
AX-157909873	1	120.253	KV452717.1	313880	1-scf00018	1
AX-157909877	1	120.253	KV452717.1	320046	1-scf00018	1
AX-157909881	1	120.253	KV452717.1	322319	1-scf00018	1
AX-157909884	1	120.253	KV452717.1	328948	1-scf00018	1
AX-157909887	1	120.253	KV452717.1	334048	1-scf00018	1
AX-157909888	1	120.253	KV452717.1	335893	1-scf00018	1
AX-157909889	1	120.253	KV452717.1	336645	1-scf00018	1
AX-157909890	1	120.253	KV452717.1	337141	1-scf00018	1
AX-157909893	1	120.253	KV452717.1	340401	1-scf00018	1
AX-157909895	1	120.253	KV452717.1	340895	1-scf00018	1
AX-157909901	1	120.253	KV452717.1	345861	1-scf00018	1
AX-157909903	1	120.253	KV452717.1	348155	1-scf00018	1
AX-157909906	1	120.253	KV452717.1	355337	1-scf00018	1
AX-157909928	1	120.253	KV452717.1	381182	1-scf00018	1
AX-157909957	1	120.253	KV452717.1	416137	1-scf00018	1
AX-157909958	1	120.253	KV452717.1	416556	1-scf00018	1
AX-157909961	1	120.253	KV452717.1	419117	1-scf00018	1
AX-158421180	1	120.253	3ML01031455	182	scf07068_1	UN
AX-157909900	1	120.306	KV452717.1	345427	1-scf00018	1
AX-157909908	1	120.36	KV452717.1	357238	1-scf00018	1
AX-157909913	1	120.361	KV452717.1	364629	1-scf00018	1
AX-157909954	1	120.361	KV452717.1	411420	1-scf00018	1
AX-157909914	1	120.362	KV452717.1	366000	1-scf00018	1
AX-157909916	1	120.362	KV452717.1	367406	1-scf00018	1
AX-157909917	1	120.362	KV452717.1	368110	1-scf00018	1
AX-157909919	1	120.362	KV452717.1	371151	1-scf00018	1
AX-157812169	1	120.362	KV452717.1	371827	1-scf00018	1
AX-157909921	1	120.362	KV452717.1	373186	1-scf00018	1

AX-157909923	1	120.362	KV452717.1	375131	1-scf00018	1
AX-157909925	1	120.362	KV452717.1	377419	1-scf00018	1
AX-157909931	1	120.362	KV452717.1	384565	1-scf00018	1
AX-157909932	1	120.362	KV452717.1	385058	1-scf00018	1
AX-157909934	1	120.362	KV452717.1	386633	1-scf00018	1
AX-157909935	1	120.362	KV452717.1	387024	1-scf00018	1
AX-157909937	1	120.362	KV452717.1	389298	1-scf00018	1
AX-157722621	1	120.362	KV452717.1	391815	1-scf00018	1
AX-157909951	1	120.362	KV452717.1	406926	1-scf00018	1
AX-157909942	1	120.451	KV452717.1	394357	1-scf00018	1
AX-157909918	1	120.472	KV452717.1	370748	1-scf00018	1
AX-157909924	1	120.472	KV452717.1	376281	1-scf00018	1
AX-157909946	1	120.472	KV452717.1	403800	1-scf00018	1
AX-157909949	1	120.472	KV452717.1	405839	1-scf00018	1
AX-157689159	1	120.472	3ML01028406	518	scf04013_1	UN
AX-157909953	1	120.581	KV452717.1	409800	1-scf00018	1
AX-157909955	1	120.581	KV452717.1	412761	1-scf00018	1
AX-157909956	1	120.683	KV452717.1	413572	1-scf00018	1
AX-157909943	1	120.799	KV452717.1	395770	1-scf00018	1
AX-157909959	1	120.799	KV452717.1	417595	1-scf00018	1
AX-157909962	1	120.799	KV452717.1	419791	1-scf00018	1
AX-157909967	1	120.799	KV452717.1	431448	1-scf00018	1
AX-157909968	1	120.799	KV452717.1	432929	1-scf00018	1
AX-157909972	1	120.922	KV452717.1	442209	1-scf00018	1
AX-157909161	1	121.018	KV452713.1	10605274	1-scf00011	1
AX-157909192	1	121.018	KV452713.1	10658696	1-scf00011	1
AX-157909193	1	121.018	KV452713.1	10660701	1-scf00011	1
AX-157909219	1	121.018	KV452715.1	14730	1-scf00013	1
AX-157909260	1	121.018	KV452715.1	67052	1-scf00013	1
AX-157909882	1	121.018	KV452717.1	322877	1-scf00018	1
AX-157909979	1	121.018	KV452717.1	453886	1-scf00018	1
AX-157909980	1	121.018	KV452717.1	454605	1-scf00018	1
AX-157710523	1	121.018	KV452717.1	454738	1-scf00018	1
AX-157909983	1	121.018	KV452717.1	461239	1-scf00018	1
AX-157909985	1	121.018	KV452717.1	462763	1-scf00018	1
AX-157909986	1	121.018	KV452717.1	462988	1-scf00018	1
AX-157909994	1	121.018	KV452717.1	474114	1-scf00018	1
AX-157909236	1	121.075	KV452715.1	32478	1-scf00013	1
AX-157909988	1	121.075	KV452717.1	466717	1-scf00018	1
AX-157909212	1	121.127	KV452715.1	8680	1-scf00013	1
AX-157909241	1	121.127	KV452715.1	37384	1-scf00013	1
AX-157909242	1	121.127	KV452715.1	38287	1-scf00013	1
AX-157909243	1	121.127	KV452715.1	38497	1-scf00013	1
AX-157909263	1	121.127	KV452715.1	69892	1-scf00013	1
AX-157909970	1	121.127	KV452717.1	438147	1-scf00018	1
AX-157770704	1	121.127	KV452717.1	464299	1-scf00018	1
AX-157909989	1	121.127	KV452717.1	467035	1-scf00018	1
AX-157909990	1	121.127	KV452717.1	470116	1-scf00018	1
AX-157909992	1	121.127	KV452717.1	471827	1-scf00018	1
AX-157910000	1	121.127	KV452717.1	480275	1-scf00018	1
AX-157910001	1	121.127	KV452717.1	481194	1-scf00018	1

AX-157910002	1	121.127	KV452717.1	482106	1-scf00018	1
AX-157874562	1	121.588	KV452718.1	58614	1-scf00017	1
AX-157909237	1	122.335	KV452715.1	33176	1-scf00013	1
AX-157909272	1	122.335	KV452716.1	1912	1-scf00015	1
AX-157909273	1	122.335	KV452716.1	2039	1-scf00015	1
AX-157812105	1	122.335	KV452716.1	10605	1-scf00015	1
AX-157909274	1	122.335	KV452716.1	13186	1-scf00015	1
AX-157909275	1	122.335	KV452716.1	13616	1-scf00015	1
AX-157909277	1	122.335	KV452716.1	16075	1-scf00015	1
AX-157909278	1	122.335	KV452716.1	21251	1-scf00015	1
AX-157722132	1	122.335	KV452716.1	22276	1-scf00015	1
AX-157772309	1	122.335	KV452716.1	22818	1-scf00015	1
AX-157909281	1	122.335	KV452716.1	31824	1-scf00015	1
AX-157909282	1	122.335	KV452716.1	32959	1-scf00015	1
AX-157909286	1	122.335	KV452716.1	51367	1-scf00015	1
AX-157909287	1	122.335	KV452716.1	53655	1-scf00015	1
AX-157909289	1	122.335	KV452716.1	60476	1-scf00015	1
AX-157812109	1	122.335	KV452716.1	60511	1-scf00015	1
AX-157749768	1	122.335	KV452716.1	104559	1-scf00015	1
AX-157741123	1	122.335	KV452716.1	104945	1-scf00015	1
AX-157909299	1	122.335	KV452716.1	105035	1-scf00015	1
AX-157909300	1	122.335	KV452716.1	105202	1-scf00015	1
AX-157812113	1	122.335	KV452716.1	105506	1-scf00015	1
AX-157812115	1	122.335	KV452716.1	111221	1-scf00015	1
AX-157909303	1	122.335	KV452716.1	111828	1-scf00015	1
AX-157909305	1	122.335	KV452716.1	114128	1-scf00015	1
AX-157909307	1	122.335	KV452716.1	115707	1-scf00015	1
AX-157909308	1	122.335	KV452716.1	118262	1-scf00015	1
AX-157727213	1	122.335	KV452716.1	119524	1-scf00015	1
AX-157909309	1	122.335	KV452716.1	119642	1-scf00015	1
AX-157719930	1	122.335	KV452716.1	122509	1-scf00015	1
AX-157909312	1	122.335	KV452716.1	125310	1-scf00015	1
AX-157909313	1	122.335	KV452716.1	125689	1-scf00015	1
AX-157909315	1	122.335	KV452716.1	128952	1-scf00015	1
AX-157909317	1	122.335	KV452716.1	129417	1-scf00015	1
AX-157757909	1	122.335	KV452716.1	131881	1-scf00015	1
AX-157909321	1	122.335	KV452716.1	134240	1-scf00015	1
AX-158379212	1	122.335	KV452716.1	136947	1-scf00015	1
AX-157909325	1	122.335	KV452716.1	138783	1-scf00015	1
AX-157869060	1	122.335	KV452716.1	142986	1-scf00015	1
AX-157909328	1	122.335	KV452716.1	143795	1-scf00015	1
AX-157909331	1	122.335	KV452716.1	149829	1-scf00015	1
AX-157909332	1	122.335	KV452716.1	150502	1-scf00015	1
AX-157740023	1	122.335	KV452716.1	158157	1-scf00015	1
AX-157742363	1	122.335	KV452716.1	158862	1-scf00015	1
AX-157909336	1	122.335	KV452716.1	159254	1-scf00015	1
AX-157909337	1	122.335	KV452716.1	159996	1-scf00015	1
AX-157909338	1	122.335	KV452716.1	160239	1-scf00015	1
AX-157909339	1	122.335	KV452716.1	161018	1-scf00015	1
AX-157909340	1	122.335	KV452716.1	162992	1-scf00015	1
AX-157909341	1	122.335	KV452716.1	164975	1-scf00015	1

AX-157909344	1	122.335	KV452716.1	173138	1-scf00015	1
AX-157909347	1	122.335	KV452716.1	180363	1-scf00015	1
AX-157909348	1	122.335	KV452716.1	181237	1-scf00015	1
AX-157909350	1	122.335	KV452716.1	182178	1-scf00015	1
AX-157812120	1	122.335	KV452716.1	182978	1-scf00015	1
AX-158404840	1	122.335	KV452716.1	183687	1-scf00015	1
AX-157909353	1	122.335	KV452716.1	187156	1-scf00015	1
AX-157909354	1	122.335	KV452716.1	187461	1-scf00015	1
AX-157883376	1	122.335	KV452716.1	188066	1-scf00015	1
AX-157909357	1	122.335	KV452716.1	195139	1-scf00015	1
AX-157909358	1	122.335	KV452716.1	195949	1-scf00015	1
AX-157909359	1	122.335	KV452716.1	198287	1-scf00015	1
AX-157909360	1	122.335	KV452716.1	198972	1-scf00015	1
AX-157909363	1	122.335	KV452716.1	200517	1-scf00015	1
AX-157909365	1	122.335	KV452716.1	201954	1-scf00015	1
AX-157909366	1	122.335	KV452716.1	204749	1-scf00015	1
AX-157909368	1	122.335	KV452716.1	209035	1-scf00015	1
AX-157909373	1	122.335	KV452716.1	214322	1-scf00015	1
AX-157909374	1	122.335	KV452716.1	215184	1-scf00015	1
AX-157909377	1	122.335	KV452716.1	222853	1-scf00015	1
AX-157909406	1	122.335	KV452719.1	75602	1-scf00016	1
AX-157909409	1	122.335	KV452719.1	78321	1-scf00016	1
AX-157909412	1	122.335	KV452719.1	96812	1-scf00016	1
AX-158404849	1	122.335	KV452719.1	100008	1-scf00016	1
AX-157909418	1	122.335	KV452719.1	115919	1-scf00016	1
AX-157909420	1	122.335	KV452719.1	118593	1-scf00016	1
AX-157909421	1	122.335	KV452719.1	119414	1-scf00016	1
AX-157909422	1	122.335	KV452719.1	120296	1-scf00016	1
AX-157909428	1	122.335	KV452719.1	128967	1-scf00016	1
AX-157909429	1	122.335	KV452719.1	129809	1-scf00016	1
AX-157812126	1	122.335	KV452719.1	132462	1-scf00016	1
AX-157909430	1	122.335	KV452719.1	133887	1-scf00016	1
AX-157909431	1	122.335	KV452719.1	134718	1-scf00016	1
AX-157909433	1	122.335	KV452719.1	135451	1-scf00016	1
AX-157909434	1	122.335	KV452719.1	137967	1-scf00016	1
AX-157909435	1	122.335	KV452719.1	139079	1-scf00016	1
AX-157771777	1	122.335	KV452719.1	141067	1-scf00016	1
AX-157752184	1	122.335	KV452719.1	143417	1-scf00016	1
AX-157812128	1	122.335	KV452719.1	152547	1-scf00016	1
AX-157812129	1	122.335	KV452719.1	152954	1-scf00016	1
AX-157909440	1	122.335	KV452719.1	154866	1-scf00016	1
AX-157874554	1	122.335	KV452719.1	160644	1-scf00016	1
AX-157909443	1	122.335	KV452719.1	165322	1-scf00016	1
AX-157742300	1	122.335	KV452719.1	170078	1-scf00016	1
AX-157909444	1	122.335	KV452719.1	171038	1-scf00016	1
AX-157909445	1	122.335	KV452719.1	171767	1-scf00016	1
AX-157909448	1	122.335	KV452719.1	182511	1-scf00016	1
AX-157909449	1	122.335	KV452719.1	182980	1-scf00016	1
AX-157909450	1	122.335	KV452719.1	183625	1-scf00016	1
AX-157874556	1	122.335	KV452719.1	187648	1-scf00016	1
AX-157909454	1	122.335	KV452719.1	192691	1-scf00016	1

AX-157909455	1	122.335	KV452719.1	194392	1-scf00016	1
AX-157909456	1	122.335	KV452719.1	195802	1-scf00016	1
AX-157909457	1	122.335	KV452719.1	197559	1-scf00016	1
AX-157883379	1	122.335	KV452719.1	200107	1-scf00016	1
AX-157765875	1	122.335	KV452719.1	201143	1-scf00016	1
AX-157909460	1	122.335	KV452719.1	202962	1-scf00016	1
AX-157909461	1	122.335	KV452719.1	209746	1-scf00016	1
AX-157909463	1	122.335	KV452719.1	218509	1-scf00016	1
AX-157909464	1	122.335	KV452719.1	225789	1-scf00016	1
AX-157909465	1	122.335	KV452719.1	226167	1-scf00016	1
AX-157909470	1	122.335	KV452719.1	236838	1-scf00016	1
AX-157909472	1	122.335	KV452719.1	238952	1-scf00016	1
AX-157909475	1	122.335	KV452719.1	252876	1-scf00016	1
AX-157909476	1	122.335	KV452719.1	253236	1-scf00016	1
AX-157743163	1	122.335	KV452719.1	264241	1-scf00016	1
AX-157757175	1	122.335	KV452719.1	272464	1-scf00016	1
AX-157909483	1	122.335	KV452719.1	272901	1-scf00016	1
AX-157909484	1	122.335	KV452719.1	273370	1-scf00016	1
AX-157909485	1	122.335	KV452719.1	274371	1-scf00016	1
AX-157909488	1	122.335	KV452719.1	288153	1-scf00016	1
AX-157909490	1	122.335	KV452719.1	306468	1-scf00016	1
AX-157909491	1	122.335	KV452719.1	309158	1-scf00016	1
AX-157909492	1	122.335	KV452719.1	319343	1-scf00016	1
AX-157909494	1	122.335	KV452719.1	323085	1-scf00016	1
AX-157909495	1	122.335	KV452719.1	325552	1-scf00016	1
AX-157909496	1	122.335	KV452719.1	325917	1-scf00016	1
AX-157909497	1	122.335	KV452719.1	331499	1-scf00016	1
AX-157909498	1	122.335	KV452719.1	335058	1-scf00016	1
AX-158404854	1	122.335	KV452719.1	338305	1-scf00016	1
AX-157909500	1	122.335	KV452719.1	340258	1-scf00016	1
AX-157909503	1	122.335	KV452719.1	344860	1-scf00016	1
AX-157909509	1	122.335	KV452719.1	382490	1-scf00016	1
AX-157909512	1	122.335	KV452719.1	407164	1-scf00016	1
AX-157909514	1	122.335	KV452719.1	421238	1-scf00016	1
AX-158382167	1	122.335	KV452719.1	429097	1-scf00016	1
AX-157909517	1	122.335	KV452719.1	434376	1-scf00016	1
AX-157909518	1	122.335	KV452719.1	436483	1-scf00016	1
AX-157909519	1	122.335	KV452719.1	437153	1-scf00016	1
AX-157909520	1	122.335	KV452719.1	437978	1-scf00016	1
AX-157909521	1	122.335	KV452719.1	438677	1-scf00016	1
AX-157812131	1	122.335	KV452719.1	439935	1-scf00016	1
AX-157909523	1	122.335	KV452719.1	445996	1-scf00016	1
AX-157909524	1	122.335	KV452719.1	449982	1-scf00016	1
AX-157909525	1	122.335	KV452719.1	452696	1-scf00016	1
AX-157909526	1	122.335	KV452719.1	455860	1-scf00016	1
AX-157909527	1	122.335	KV452719.1	457552	1-scf00016	1
AX-158404855	1	122.335	KV452719.1	473225	1-scf00016	1
AX-157909529	1	122.335	KV452719.1	476138	1-scf00016	1
AX-157909530	1	122.335	KV452719.1	476855	1-scf00016	1
AX-157909531	1	122.335	KV452719.1	477658	1-scf00016	1
AX-157909532	1	122.335	KV452719.1	478617	1-scf00016	1

AX-157909538	1	122.335	KV452719.1	495599	1-scf00016	1
AX-157909539	1	122.335	KV452719.1	498180	1-scf00016	1
AX-157909541	1	122.335	KV452719.1	507056	1-scf00016	1
AX-157812132	1	122.335	KV452719.1	512419	1-scf00016	1
AX-157909543	1	122.335	KV452719.1	518731	1-scf00016	1
AX-158404860	1	122.335	KV452719.1	524376	1-scf00016	1
AX-157909547	1	122.335	KV452719.1	529982	1-scf00016	1
AX-157909551	1	122.335	KV452719.1	541422	1-scf00016	1
AX-157909554	1	122.335	KV452719.1	546078	1-scf00016	1
AX-157909556	1	122.335	KV452719.1	558512	1-scf00016	1
AX-157909559	1	122.335	KV452719.1	564429	1-scf00016	1
AX-157909569	1	122.335	KV452719.1	630473	1-scf00016	1
AX-157909572	1	122.335	KV452719.1	639605	1-scf00016	1
AX-157909575	1	122.335	KV452719.1	646610	1-scf00016	1
AX-157812135	1	122.335	KV452719.1	677657	1-scf00016	1
AX-157909580	1	122.335	KV452719.1	683134	1-scf00016	1
AX-158382168	1	122.335	KV452718.1	55105	1-scf00017	1
AX-157909594	1	122.335	KV452718.1	61065	1-scf00017	1
AX-157739670	1	122.335	KV452718.1	150352	1-scf00017	1
AX-157909626	1	122.335	KV452718.1	194989	1-scf00017	1
AX-157909628	1	122.335	KV452718.1	199041	1-scf00017	1
AX-157909630	1	122.335	KV452718.1	201235	1-scf00017	1
AX-157909631	1	122.335	KV452718.1	202352	1-scf00017	1
AX-157909632	1	122.335	KV452718.1	202629	1-scf00017	1
AX-157909633	1	122.335	KV452718.1	204334	1-scf00017	1
AX-157909634	1	122.335	KV452718.1	208520	1-scf00017	1
AX-157909639	1	122.335	KV452718.1	218278	1-scf00017	1
AX-157812138	1	122.335	KV452718.1	228210	1-scf00017	1
AX-157812140	1	122.335	KV452718.1	235274	1-scf00017	1
AX-157909646	1	122.335	KV452718.1	235741	1-scf00017	1
AX-157717006	1	122.335	KV452718.1	235881	1-scf00017	1
AX-157909647	1	122.335	KV452718.1	236468	1-scf00017	1
AX-157740956	1	122.335	KV452718.1	237176	1-scf00017	1
AX-158379214	1	122.335	KV452718.1	237482	1-scf00017	1
AX-157715370	1	122.335	KV452718.1	237829	1-scf00017	1
AX-157812144	1	122.335	KV452718.1	261511	1-scf00017	1
AX-157745879	1	122.335	KV452718.1	262270	1-scf00017	1
AX-157909656	1	122.335	KV452718.1	267962	1-scf00017	1
AX-157748685	1	122.335	KV452718.1	269096	1-scf00017	1
AX-157716849	1	122.335	KV452718.1	270718	1-scf00017	1
AX-157909660	1	122.335	KV452718.1	276795	1-scf00017	1
AX-157909661	1	122.335	KV452718.1	277474	1-scf00017	1
AX-157874564	1	122.335	KV452718.1	278226	1-scf00017	1
AX-157909667	1	122.335	KV452718.1	285697	1-scf00017	1
AX-157909671	1	122.335	KV452717.1	13153	1-scf00018	1
AX-157909673	1	122.335	KV452717.1	16490	1-scf00018	1
AX-157909680	1	122.335	KV452717.1	37454	1-scf00018	1
AX-157909681	1	122.335	KV452717.1	40904	1-scf00018	1
AX-157909682	1	122.335	KV452717.1	41618	1-scf00018	1
AX-157909683	1	122.335	KV452717.1	43289	1-scf00018	1
AX-157909684	1	122.335	KV452717.1	45368	1-scf00018	1

AX-158382172	1	122.335	KV452717.1	47103	1-scf00018	1
AX-157909690	1	122.335	KV452717.1	53211	1-scf00018	1
AX-157909696	1	122.335	KV452717.1	65752	1-scf00018	1
AX-157909709	1	122.335	KV452717.1	83044	1-scf00018	1
AX-157909726	1	122.335	KV452717.1	110873	1-scf00018	1
AX-157909747	1	122.335	KV452717.1	139459	1-scf00018	1
AX-157909750	1	122.335	KV452717.1	141339	1-scf00018	1
AX-157909766	1	122.335	KV452717.1	188074	1-scf00018	1
AX-157909772	1	122.335	KV452717.1	197279	1-scf00018	1
AX-157909810	1	122.335	KV452717.1	258317	1-scf00018	1
AX-157909831	1	122.335	KV452717.1	272322	1-scf00018	1
AX-157909843	1	122.335	KV452717.1	283862	1-scf00018	1
AX-157909874	1	122.335	KV452717.1	315948	1-scf00018	1
AX-157722927	1	122.335	KV452717.1	447631	1-scf00018	1
AX-157687751	1	122.335	KV453595.1	5937	scf00999	UN
AX-157909552	1	122.345	KV452719.1	542896	1-scf00016	1
AX-157909553	1	122.345	KV452719.1	544730	1-scf00016	1
AX-157909560	1	122.345	KV452719.1	565597	1-scf00016	1
AX-157689701	1	122.48	3ML01031455	744	scf07068_1	UN
AX-157910010	2	0	KV453182.1	7839	20-scf00019	20
AX-157864457	2	0.296	KV453182.1	22246	20-scf00019	20
AX-157910006	2	0.301	KV453182.1	4841	20-scf00019	20
AX-157910007	2	0.301	KV453182.1	4892	20-scf00019	20
AX-157910008	2	0.301	KV453182.1	5643	20-scf00019	20
AX-157910011	2	0.301	KV453182.1	8037	20-scf00019	20
AX-157910012	2	0.301	KV453182.1	9100	20-scf00019	20
AX-157910013	2	0.301	KV453182.1	9614	20-scf00019	20
AX-157812171	2	0.301	KV453182.1	12229	20-scf00019	20
AX-157910014	2	0.301	KV453182.1	16843	20-scf00019	20
AX-157910017	2	0.301	KV453182.1	19336	20-scf00019	20
AX-157910018	2	0.301	KV453182.1	23769	20-scf00019	20
AX-157910019	2	0.301	KV453182.1	24661	20-scf00019	20
AX-157910022	2	0.301	KV453182.1	28094	20-scf00019	20
AX-157812176	2	0.301	KV453182.1	30086	20-scf00019	20
AX-157910023	2	0.301	KV453182.1	33651	20-scf00019	20
AX-157910024	2	0.301	KV453182.1	35663	20-scf00019	20
AX-157910025	2	0.301	KV453182.1	36702	20-scf00019	20
AX-157910026	2	0.301	KV453182.1	39185	20-scf00019	20
AX-157910027	2	0.301	KV453182.1	39574	20-scf00019	20
AX-157910031	2	0.301	KV453182.1	43769	20-scf00019	20
AX-157910032	2	0.301	KV453182.1	45897	20-scf00019	20
AX-157910034	2	0.301	KV453182.1	47893	20-scf00019	20
AX-157910036	2	0.301	KV453182.1	49231	20-scf00019	20
AX-158382178	2	0.301	KV453182.1	49802	20-scf00019	20
AX-157910038	2	0.301	KV453182.1	50994	20-scf00019	20
AX-157910040	2	0.301	KV453182.1	52364	20-scf00019	20
AX-157910041	2	0.301	KV453182.1	52659	20-scf00019	20
AX-157910042	2	0.301	KV453182.1	55425	20-scf00019	20
AX-157910046	2	0.301	KV453182.1	61042	20-scf00019	20
AX-157910047	2	0.301	KV453182.1	61809	20-scf00019	20
AX-157910049	2	0.301	KV453182.1	64923	20-scf00019	20

AX-157910052	2	0.301	KV453182.1	71439	20-scf00019	20
AX-157725436	2	0.301	KV453182.1	72683	20-scf00019	20
AX-157910055	2	0.301	KV453182.1	77008	20-scf00019	20
AX-157910056	2	0.301	KV453182.1	79388	20-scf00019	20
AX-157910057	2	0.301	KV453182.1	79995	20-scf00019	20
AX-158382180	2	0.301	KV453182.1	92777	20-scf00019	20
AX-157812180	2	0.301	KV453182.1	104900	20-scf00019	20
AX-157910067	2	0.301	KV453182.1	105672	20-scf00019	20
AX-157853663	2	0.301	KV453182.1	110957	20-scf00019	20
AX-157910070	2	0.301	KV453182.1	111408	20-scf00019	20
AX-158382181	2	0.301	KV453182.1	116442	20-scf00019	20
AX-157910075	2	0.301	KV453182.1	117864	20-scf00019	20
AX-157874574	2	0.301	KV453182.1	119087	20-scf00019	20
AX-157726115	2	0.301	KV453182.1	119855	20-scf00019	20
AX-157910078	2	0.301	KV453182.1	121918	20-scf00019	20
AX-157910079	2	0.301	KV453182.1	122546	20-scf00019	20
AX-157910081	2	0.301	KV453182.1	126335	20-scf00019	20
AX-157910083	2	0.301	KV453182.1	127812	20-scf00019	20
AX-158382182	2	0.301	KV453182.1	133063	20-scf00019	20
AX-157910087	2	0.301	KV453182.1	137397	20-scf00019	20
AX-157883384	2	0.301	KV453182.1	139284	20-scf00019	20
AX-157910090	2	0.301	KV453182.1	140171	20-scf00019	20
AX-157812183	2	0.301	KV453182.1	145829	20-scf00019	20
AX-157812185	2	0.301	KV453182.1	161770	20-scf00019	20
AX-157714843	2	0.301	KV453182.1	163247	20-scf00019	20
AX-157910097	2	0.301	KV453182.1	164399	20-scf00019	20
AX-157910098	2	0.301	KV453182.1	165841	20-scf00019	20
AX-157726823	2	0.301	KV453182.1	171868	20-scf00019	20
AX-157910103	2	0.301	KV453182.1	171904	20-scf00019	20
AX-157910105	2	0.301	KV453182.1	174177	20-scf00019	20
AX-158404877	2	0.301	KV453182.1	183500	20-scf00019	20
AX-157910109	2	0.301	KV453182.1	186403	20-scf00019	20
AX-157812187	2	0.301	KV453182.1	186800	20-scf00019	20
AX-157720122	2	0.301	KV453182.1	200240	20-scf00019	20
AX-157910111	2	0.301	KV453182.1	200372	20-scf00019	20
AX-157910113	2	0.301	KV453182.1	204453	20-scf00019	20
AX-157910115	2	0.301	KV453182.1	205570	20-scf00019	20
AX-157864458	2	0.301	KV453182.1	209533	20-scf00019	20
AX-157742227	2	0.301	KV453182.1	209906	20-scf00019	20
AX-157748635	2	0.301	KV453182.1	210636	20-scf00019	20
AX-157910120	2	0.301	KV453182.1	213015	20-scf00019	20
AX-157723371	2	0.301	KV453182.1	213399	20-scf00019	20
AX-157910125	2	0.301	KV453182.1	224971	20-scf00019	20
AX-157910130	2	0.301	KV453182.1	228864	20-scf00019	20
AX-157910132	2	0.301	KV453182.1	231518	20-scf00019	20
AX-157853664	2	0.301	KV453182.1	232290	20-scf00019	20
AX-157910133	2	0.301	KV453182.1	232327	20-scf00019	20
AX-157747669	2	0.301	KV453182.1	240384	20-scf00019	20
AX-157910138	2	0.301	KV453182.1	245817	20-scf00019	20
AX-157910139	2	0.301	KV453182.1	246749	20-scf00019	20
AX-157864460	2	0.301	KV453182.1	249032	20-scf00019	20

AX-157910147	2	0.301	KV453182.1	265843	20-scf00019	20
AX-157910148	2	0.301	KV453182.1	269083	20-scf00019	20
AX-157864461	2	0.301	KV453182.1	269624	20-scf00019	20
AX-157910156	2	0.301	KV453182.1	278833	20-scf00019	20
AX-157910158	2	0.301	KV453182.1	282143	20-scf00019	20
AX-157910172	2	0.301	KV453182.1	297926	20-scf00019	20
AX-157910174	2	0.301	KV453182.1	300616	20-scf00019	20
AX-157910190	2	0.301	KV453182.1	328242	20-scf00019	20
AX-157910202	2	0.301	KV453182.1	340920	20-scf00019	20
AX-157910203	2	0.301	KV453182.1	342323	20-scf00019	20
AX-157812199	2	0.301	KV453182.1	373799	20-scf00019	20
AX-157910244	2	0.301	KV453182.1	419007	20-scf00019	20
AX-157710576	2	0.301	KV453182.1	436905	20-scf00019	20
AX-157716383	2	0.301	KV453182.1	443828	20-scf00019	20
AX-157812206	2	0.301	KV453182.1	494042	20-scf00019	20
AX-157704694	2	0.301	3ML01027054	772	scf02658_1	UN
AX-157874577	2	0.317	KV453182.1	239219	20-scf00019	20
AX-157910135	2	0.317	KV453182.1	243087	20-scf00019	20
AX-157910149	2	0.317	KV453182.1	269916	20-scf00019	20
AX-157910249	2	0.317	KV453182.1	429791	20-scf00019	20
AX-157910252	2	0.317	KV453182.1	435728	20-scf00019	20
AX-157910259	2	0.317	KV453182.1	448321	20-scf00019	20
AX-157910266	2	0.317	KV453182.1	456859	20-scf00019	20
AX-157910137	2	0.364	KV453182.1	245102	20-scf00019	20
AX-157910150	2	0.411	KV453182.1	270488	20-scf00019	20
AX-157910152	2	0.411	KV453182.1	273174	20-scf00019	20
AX-157812177	2	0.52	KV453182.1	34447	20-scf00019	20
AX-157812178	2	0.52	KV453182.1	34648	20-scf00019	20
AX-157910029	2	0.52	KV453182.1	42055	20-scf00019	20
AX-157910037	2	0.52	KV453182.1	49863	20-scf00019	20
AX-157853661	2	0.52	KV453182.1	53536	20-scf00019	20
AX-157910045	2	0.52	KV453182.1	59011	20-scf00019	20
AX-157910051	2	0.52	KV453182.1	66977	20-scf00019	20
AX-157910089	2	0.52	KV453182.1	139201	20-scf00019	20
AX-158421856	2	0.52	KV453182.1	184522	20-scf00019	20
AX-157910112	2	0.52	KV453182.1	203359	20-scf00019	20
AX-157864459	2	0.52	KV453182.1	212171	20-scf00019	20
AX-157910143	2	0.52	KV453182.1	258401	20-scf00019	20
AX-157910163	2	0.52	KV453182.1	286826	20-scf00019	20
AX-157910164	2	0.52	KV453182.1	287932	20-scf00019	20
AX-157910167	2	0.52	KV453182.1	290021	20-scf00019	20
AX-157910178	2	0.52	KV453182.1	307579	20-scf00019	20
AX-157726129	2	0.52	KV453182.1	333018	20-scf00019	20
AX-157910205	2	0.52	KV453182.1	344386	20-scf00019	20
AX-157812197	2	0.52	KV453182.1	355191	20-scf00019	20
AX-157738392	2	0.52	KV453182.1	356848	20-scf00019	20
AX-157910213	2	0.52	KV453182.1	361125	20-scf00019	20
AX-157812198	2	0.52	KV453182.1	372937	20-scf00019	20
AX-157910230	2	0.52	KV453182.1	401475	20-scf00019	20
AX-157910232	2	0.52	KV453182.1	404298	20-scf00019	20
AX-157910234	2	0.52	KV453182.1	406705	20-scf00019	20

AX-157910239	2	0.52	KV453182.1	415432	20-scf00019	20
AX-157910241	2	0.52	KV453182.1	417089	20-scf00019	20
AX-157910242	2	0.52	KV453182.1	417534	20-scf00019	20
AX-157864462	2	0.52	KV453182.1	420180	20-scf00019	20
AX-157910246	2	0.52	KV453182.1	422268	20-scf00019	20
AX-157709185	2	0.52	KV453182.1	437435	20-scf00019	20
AX-157910254	2	0.52	KV453182.1	437665	20-scf00019	20
AX-157910255	2	0.52	KV453182.1	440225	20-scf00019	20
AX-157713245	2	0.52	KV453182.1	442323	20-scf00019	20
AX-157910265	2	0.629	KV453182.1	456434	20-scf00019	20
AX-157910297	2	0.629	KV453182.1	506590	20-scf00019	20
AX-157910269	2	0.739	KV453182.1	460685	20-scf00019	20
AX-157812204	2	0.848	KV453182.1	448984	20-scf00019	20
AX-157910263	2	0.848	KV453182.1	454888	20-scf00019	20
AX-157910270	2	0.848	KV453182.1	461077	20-scf00019	20
AX-157910272	2	0.848	KV453182.1	462911	20-scf00019	20
AX-157742855	2	0.848	KV453182.1	462966	20-scf00019	20
AX-158379217	2	0.848	KV453182.1	471348	20-scf00019	20
AX-157910277	2	0.848	KV453182.1	471789	20-scf00019	20
AX-157910281	2	0.848	KV453182.1	481990	20-scf00019	20
AX-157910282	2	0.848	KV453182.1	483099	20-scf00019	20
AX-157910284	2	0.848	KV453182.1	486797	20-scf00019	20
AX-157910285	2	0.848	KV453182.1	488416	20-scf00019	20
AX-157910318	2	0.848	KV453182.1	536589	20-scf00019	20
AX-157910385	2	0.848	KV453182.1	624287	20-scf00019	20
AX-157910394	2	0.848	KV453182.1	641696	20-scf00019	20
AX-157910397	2	0.848	KV453182.1	645165	20-scf00019	20
AX-157910434	2	0.848	KV453182.1	698322	20-scf00019	20
AX-157812215	2	0.848	KV453182.1	771496	20-scf00019	20
AX-157910515	2	0.848	KV453182.1	803754	20-scf00019	20
AX-157910554	2	0.848	KV453182.1	870318	20-scf00019	20
AX-157812207	2	1.012	KV453182.1	513797	20-scf00019	20
AX-158404882	2	1.012	KV453182.1	514567	20-scf00019	20
AX-157910304	2	1.012	KV453182.1	518985	20-scf00019	20
AX-157910309	2	1.012	KV453182.1	523429	20-scf00019	20
AX-157910310	2	1.012	KV453182.1	523838	20-scf00019	20
AX-157910312	2	1.012	KV453182.1	527651	20-scf00019	20
AX-157910030	2	1.175	KV453182.1	42396	20-scf00019	20
AX-157874572	2	1.175	KV453182.1	81544	20-scf00019	20
AX-157910068	2	1.175	KV453182.1	109449	20-scf00019	20
AX-157874573	2	1.175	KV453182.1	111473	20-scf00019	20
AX-157910072	2	1.175	KV453182.1	115889	20-scf00019	20
AX-157910076	2	1.175	KV453182.1	118603	20-scf00019	20
AX-157812181	2	1.175	KV453182.1	128463	20-scf00019	20
AX-158404878	2	1.175	KV453182.1	184353	20-scf00019	20
AX-157910260	2	1.175	KV453182.1	451308	20-scf00019	20
AX-157910315	2	1.175	KV453182.1	531551	20-scf00019	20
AX-157812208	2	1.175	KV453182.1	533297	20-scf00019	20
AX-157910335	2	1.175	KV453182.1	559007	20-scf00019	20
AX-157910340	2	1.175	KV453182.1	563107	20-scf00019	20
AX-157910342	2	1.175	KV453182.1	566709	20-scf00019	20

AX-157910347	2	1.175	KV453182.1	573345	20-scf00019	20
AX-157740783	2	1.175	KV453182.1	574942	20-scf00019	20
AX-157910351	2	1.175	KV453182.1	576629	20-scf00019	20
AX-157910352	2	1.175	KV453182.1	579454	20-scf00019	20
AX-157910353	2	1.175	KV453182.1	582939	20-scf00019	20
AX-157910356	2	1.175	KV453182.1	584997	20-scf00019	20
AX-157910357	2	1.175	KV453182.1	586435	20-scf00019	20
AX-157910359	2	1.175	KV453182.1	587867	20-scf00019	20
AX-157910361	2	1.175	KV453182.1	590487	20-scf00019	20
AX-157910365	2	1.175	KV453182.1	597494	20-scf00019	20
AX-157910367	2	1.175	KV453182.1	600599	20-scf00019	20
AX-157910370	2	1.175	KV453182.1	602804	20-scf00019	20
AX-157910405	2	1.175	KV453182.1	651415	20-scf00019	20
AX-157910537	2	1.175	KV453182.1	842124	20-scf00019	20
AX-157910547	2	1.175	KV453182.1	852457	20-scf00019	20
AX-157713930	2	1.206	KV453182.1	483585	20-scf00019	20
AX-157910355	2	1.206	KV453182.1	584676	20-scf00019	20
AX-157910371	2	1.206	KV453182.1	603215	20-scf00019	20
AX-157812209	2	1.206	KV453182.1	603910	20-scf00019	20
AX-158379220	2	1.206	KV453182.1	848823	20-scf00019	20
AX-157910552	2	1.206	KV453182.1	865499	20-scf00019	20
AX-157745912	2	1.285	KV453182.1	620227	20-scf00019	20
AX-157812213	2	1.285	KV453182.1	620411	20-scf00019	20
AX-157910386	2	1.285	KV453182.1	624978	20-scf00019	20
AX-157910388	2	1.285	KV453182.1	627261	20-scf00019	20
AX-157717115	2	1.285	KV453182.1	631938	20-scf00019	20
AX-157910390	2	1.285	KV453182.1	634618	20-scf00019	20
AX-157853669	2	1.285	KV453182.1	638522	20-scf00019	20
AX-157910393	2	1.285	KV453182.1	640111	20-scf00019	20
AX-157910333	2	1.503	KV453182.1	557770	20-scf00019	20
AX-157910338	2	1.503	KV453182.1	561002	20-scf00019	20
AX-158389551	2	1.503	KV453182.1	567517	20-scf00019	20
AX-157910400	2	1.503	KV453182.1	648178	20-scf00019	20
AX-157910401	2	1.503	KV453182.1	648702	20-scf00019	20
AX-157910404	2	1.503	KV453182.1	650646	20-scf00019	20
AX-157910408	2	1.503	KV453182.1	654136	20-scf00019	20
AX-157910415	2	1.503	KV453182.1	667384	20-scf00019	20
AX-157910416	2	1.503	KV453182.1	668587	20-scf00019	20
AX-157910418	2	1.503	KV453182.1	671006	20-scf00019	20
AX-157910421	2	1.503	KV453182.1	674289	20-scf00019	20
AX-157910423	2	1.503	KV453182.1	675867	20-scf00019	20
AX-157744215	2	1.503	KV453182.1	678404	20-scf00019	20
AX-157910459	2	1.503	KV453182.1	727183	20-scf00019	20
AX-157910426	2	1.545	KV453182.1	680016	20-scf00019	20
AX-157910440	2	1.612	KV453182.1	705569	20-scf00019	20
AX-157910441	2	1.612	KV453182.1	706737	20-scf00019	20
AX-157910444	2	1.612	KV453182.1	709215	20-scf00019	20
AX-157910446	2	1.612	KV453182.1	713757	20-scf00019	20
AX-157910447	2	1.612	KV453182.1	715222	20-scf00019	20
AX-157910451	2	1.612	KV453182.1	719021	20-scf00019	20
AX-157910453	2	1.612	KV453182.1	719704	20-scf00019	20

AX-157910471	2	1.612	KV453182.1	746161	20-scf00019	20
AX-157910437	2	1.722	KV453182.1	700812	20-scf00019	20
AX-157910461	2	1.722	KV453182.1	732377	20-scf00019	20
AX-157910462	2	1.722	KV453182.1	733046	20-scf00019	20
AX-157910464	2	1.722	KV453182.1	735967	20-scf00019	20
AX-157910465	2	1.722	KV453182.1	736343	20-scf00019	20
AX-157910472	2	1.722	KV453182.1	746692	20-scf00019	20
AX-157910474	2	1.722	KV453182.1	749875	20-scf00019	20
AX-157910475	2	1.722	KV453182.1	751169	20-scf00019	20
AX-157910476	2	1.722	KV453182.1	751869	20-scf00019	20
AX-157910483	2	1.722	KV453182.1	760511	20-scf00019	20
AX-157812214	2	1.722	KV453182.1	765686	20-scf00019	20
AX-157774029	2	1.722	KV453182.1	807815	20-scf00019	20
AX-157910599	2	1.722	KV453182.1	918723	20-scf00019	20
AX-157812235	2	1.722	KV453182.1	1030165	20-scf00019	20
AX-157739872	2	1.722	KV453182.1	1035359	20-scf00019	20
AX-157910493	2	1.78	KV453182.1	771031	20-scf00019	20
AX-157910082	2	1.838	KV453182.1	127198	20-scf00019	20
AX-157910151	2	1.838	KV453182.1	270825	20-scf00019	20
AX-157910161	2	1.838	KV453182.1	284014	20-scf00019	20
AX-157910177	2	1.838	KV453182.1	306992	20-scf00019	20
AX-157910316	2	1.838	KV453182.1	533818	20-scf00019	20
AX-157910336	2	1.838	KV453182.1	559607	20-scf00019	20
AX-157743795	2	1.838	KV453182.1	612885	20-scf00019	20
AX-157883389	2	1.838	KV453182.1	656509	20-scf00019	20
AX-157910480	2	1.838	KV453182.1	758885	20-scf00019	20
AX-157883392	2	1.838	KV453182.1	762086	20-scf00019	20
AX-157910494	2	1.838	KV453182.1	772002	20-scf00019	20
AX-157910562	2	1.838	KV453182.1	879577	20-scf00019	20
AX-157910625	2	1.838	KV453182.1	957590	20-scf00019	20
AX-157812173	2	1.886	KV453182.1	24060	20-scf00019	20
AX-157910054	2	1.886	KV453182.1	73814	20-scf00019	20
AX-157812193	2	1.886	KV453182.1	222861	20-scf00019	20
AX-157910129	2	1.886	KV453182.1	228282	20-scf00019	20
AX-157910136	2	1.886	KV453182.1	243581	20-scf00019	20
AX-157910154	2	1.886	KV453182.1	276376	20-scf00019	20
AX-157910195	2	1.886	KV453182.1	333483	20-scf00019	20
AX-157910216	2	1.886	KV453182.1	363927	20-scf00019	20
AX-157910219	2	1.886	KV453182.1	369170	20-scf00019	20
AX-157758150	2	1.886	KV453182.1	407932	20-scf00019	20
AX-157812201	2	1.886	KV453182.1	408313	20-scf00019	20
AX-157910236	2	1.886	KV453182.1	413196	20-scf00019	20
AX-157910238	2	1.886	KV453182.1	415148	20-scf00019	20
AX-157910250	2	1.886	KV453182.1	430297	20-scf00019	20
AX-157910251	2	1.886	KV453182.1	431748	20-scf00019	20
AX-157910273	2	1.886	KV453182.1	464058	20-scf00019	20
AX-157910278	2	1.886	KV453182.1	472424	20-scf00019	20
AX-157910280	2	1.886	KV453182.1	476573	20-scf00019	20
AX-157910287	2	1.886	KV453182.1	494813	20-scf00019	20
AX-157910295	2	1.886	KV453182.1	504624	20-scf00019	20
AX-157910306	2	1.886	KV453182.1	521875	20-scf00019	20

AX-157910327	2	1.886	KV453182.1	550780	20-scf00019	20
AX-157910372	2	1.886	KV453182.1	604229	20-scf00019	20
AX-157910427	2	1.886	KV453182.1	681026	20-scf00019	20
AX-157864464	2	1.886	KV453182.1	693323	20-scf00019	20
AX-157910467	2	1.886	KV453182.1	740462	20-scf00019	20
AX-157910468	2	1.886	KV453182.1	741022	20-scf00019	20
AX-157910469	2	1.886	KV453182.1	742483	20-scf00019	20
AX-157910473	2	1.886	KV453182.1	748910	20-scf00019	20
AX-157910491	2	1.886	KV453182.1	765436	20-scf00019	20
AX-157910495	2	1.886	KV453182.1	773136	20-scf00019	20
AX-157812216	2	1.886	KV453182.1	779856	20-scf00019	20
AX-158404887	2	1.886	KV453182.1	785130	20-scf00019	20
AX-157853673	2	1.886	KV453182.1	802687	20-scf00019	20
AX-157910522	2	1.886	KV453182.1	817518	20-scf00019	20
AX-157910523	2	1.886	KV453182.1	819293	20-scf00019	20
AX-157910526	2	1.886	KV453182.1	823560	20-scf00019	20
AX-157910528	2	1.886	KV453182.1	824768	20-scf00019	20
AX-157720043	2	1.886	KV453182.1	829746	20-scf00019	20
AX-157812222	2	1.886	KV453182.1	851321	20-scf00019	20
AX-157910548	2	1.886	KV453182.1	858370	20-scf00019	20
AX-157910556	2	1.886	KV453182.1	871183	20-scf00019	20
AX-157775495	2	1.886	KV453182.1	877102	20-scf00019	20
AX-158404889	2	1.886	KV453182.1	895800	20-scf00019	20
AX-157910606	2	1.886	KV453182.1	929606	20-scf00019	20
AX-157910615	2	1.886	KV453182.1	941839	20-scf00019	20
AX-157910617	2	1.886	KV453182.1	944889	20-scf00019	20
AX-157910648	2	1.886	KV453182.1	976955	20-scf00019	20
AX-157910660	2	1.886	KV453182.1	991833	20-scf00019	20
AX-157910667	2	1.886	KV453182.1	1000230	20-scf00019	20
AX-157910499	2	2.049	KV453182.1	779054	20-scf00019	20
AX-157754541	2	2.049	KV453182.1	805427	20-scf00019	20
AX-157910534	2	2.049	KV453182.1	835388	20-scf00019	20
AX-157910549	2	2.049	KV453182.1	859866	20-scf00019	20
AX-157751049	2	2.049	KV453182.1	862559	20-scf00019	20
AX-157910557	2	2.049	KV453182.1	873745	20-scf00019	20
AX-157751035	2	2.049	KV453182.1	883410	20-scf00019	20
AX-157910569	2	2.049	KV453182.1	884576	20-scf00019	20
AX-157910570	2	2.049	KV453182.1	886852	20-scf00019	20
AX-157910574	2	2.049	KV453182.1	890020	20-scf00019	20
AX-157910576	2	2.049	KV453182.1	891310	20-scf00019	20
AX-157910584	2	2.049	KV453182.1	899480	20-scf00019	20
AX-157910586	2	2.049	KV453182.1	901866	20-scf00019	20
AX-157910590	2	2.049	KV453182.1	908304	20-scf00019	20
AX-157910592	2	2.049	KV453182.1	910055	20-scf00019	20
AX-157910602	2	2.049	KV453182.1	923424	20-scf00019	20
AX-157739660	2	2.049	KV453182.1	925368	20-scf00019	20
AX-157910603	2	2.049	KV453182.1	925756	20-scf00019	20
AX-157724646	2	2.049	KV453182.1	935956	20-scf00019	20
AX-157910633	2	2.049	KV453182.1	962042	20-scf00019	20
AX-157910641	2	2.049	KV453182.1	970980	20-scf00019	20
AX-157910652	2	2.049	KV453182.1	980722	20-scf00019	20

AX-157910659	2	2.049	KV453182.1	990587	20-scf00019	20
AX-157706940	2	2.049	3ML01033190	196	scf08830_1	UN
AX-157910593	2	2.074	KV453182.1	911120	20-scf00019	20
AX-157910454	2	2.096	KV453182.1	720661	20-scf00019	20
AX-157910567	2	2.096	KV453182.1	882915	20-scf00019	20
AX-157910595	2	2.096	KV453182.1	912647	20-scf00019	20
AX-157910604	2	2.096	KV453182.1	927628	20-scf00019	20
AX-157910610	2	2.096	KV453182.1	934209	20-scf00019	20
AX-157910689	2	2.096	KV453182.1	1037528	20-scf00019	20
AX-157910613	2	2.115	KV453182.1	937909	20-scf00019	20
AX-157910573	2	2.129	KV453182.1	889140	20-scf00019	20
AX-157910575	2	2.129	KV453182.1	890545	20-scf00019	20
AX-158382187	2	2.129	KV453182.1	943611	20-scf00019	20
AX-157910609	2	2.147	KV453182.1	933865	20-scf00019	20
AX-157910616	2	2.147	KV453182.1	943957	20-scf00019	20
AX-157812227	2	2.159	KV453182.1	868346	20-scf00019	20
AX-157910558	2	2.159	KV453182.1	875529	20-scf00019	20
AX-157910619	2	2.159	KV453182.1	947899	20-scf00019	20
AX-157910620	2	2.159	KV453182.1	949482	20-scf00019	20
AX-157772238	2	2.159	KV453182.1	952198	20-scf00019	20
AX-157910622	2	2.159	KV453182.1	952399	20-scf00019	20
AX-157910623	2	2.159	KV453182.1	955534	20-scf00019	20
AX-157910628	2	2.159	KV453182.1	959625	20-scf00019	20
AX-157910630	2	2.159	KV453182.1	960678	20-scf00019	20
AX-157910635	2	2.159	KV453182.1	963864	20-scf00019	20
AX-157910636	2	2.159	KV453182.1	966564	20-scf00019	20
AX-157910639	2	2.159	KV453182.1	968879	20-scf00019	20
AX-157910642	2	2.159	KV453182.1	971451	20-scf00019	20
AX-157910535	2	2.377	KV453182.1	836646	20-scf00019	20
AX-157739000	2	2.377	KV453182.1	975615	20-scf00019	20
AX-157910649	2	2.377	KV453182.1	978772	20-scf00019	20
AX-157910651	2	2.377	KV453182.1	980134	20-scf00019	20
AX-157910653	2	2.377	KV453182.1	981405	20-scf00019	20
AX-157883396	2	2.377	KV453182.1	991289	20-scf00019	20
AX-157910662	2	2.377	KV453182.1	995739	20-scf00019	20
AX-157910668	2	2.442	KV453182.1	1001119	20-scf00019	20
AX-157910669	2	2.511	KV453182.1	1002193	20-scf00019	20
AX-157910673	2	2.568	KV453182.1	1004790	20-scf00019	20
AX-157883397	2	2.891	KV453182.1	1014070	20-scf00019	20
AX-157910168	2	2.899	KV453182.1	291258	20-scf00019	20
AX-157910678	2	2.899	KV453182.1	1014549	20-scf00019	20
AX-157910672	2	2.937	KV453182.1	1004386	20-scf00019	20
AX-157910679	2	2.937	KV453182.1	1024813	20-scf00019	20
AX-157910681	2	2.937	KV453182.1	1027885	20-scf00019	20
AX-157812233	2	2.937	KV453182.1	1028094	20-scf00019	20
AX-157910682	2	2.937	KV453182.1	1028322	20-scf00019	20
AX-157910687	2	2.937	KV453182.1	1035767	20-scf00019	20
AX-157910705	2	3.265	KV453182.1	1062494	20-scf00019	20
AX-157910704	2	3.266	KV453182.1	1061985	20-scf00019	20
AX-157910706	2	3.266	KV453182.1	1063315	20-scf00019	20
AX-157910710	2	3.578	KV453182.1	1068502	20-scf00019	20

AX-157910664	2	3.581	KV453182.1	997316	20-scf00019	20
AX-157910684	2	3.581	KV453182.1	1031844	20-scf00019	20
AX-157742827	2	3.581	KV453182.1	1036399	20-scf00019	20
AX-157910691	2	3.581	KV453182.1	1042115	20-scf00019	20
AX-157771968	2	3.581	KV453182.1	1043825	20-scf00019	20
AX-157910711	2	3.581	KV453182.1	1069074	20-scf00019	20
AX-157910713	2	3.581	KV453182.1	1075625	20-scf00019	20
AX-157755039	2	3.581	KV453182.1	1076951	20-scf00019	20
AX-157910716	2	3.581	KV453182.1	1078539	20-scf00019	20
AX-157910720	2	3.581	KV453182.1	1082891	20-scf00019	20
AX-157910723	2	3.581	KV453182.1	1086252	20-scf00019	20
AX-157720572	2	3.581	KV453182.1	1093786	20-scf00019	20
AX-157910724	2	3.607	KV453182.1	1088989	20-scf00019	20
AX-157772560	2	3.635	KV453182.1	1092844	20-scf00019	20
AX-158379222	2	3.661	KV453182.1	1099730	20-scf00019	20
AX-157910735	2	3.69	KV453182.1	1100831	20-scf00019	20
AX-157910736	2	3.69	KV453182.1	1101752	20-scf00019	20
AX-157910739	2	3.69	KV453182.1	1105039	20-scf00019	20
AX-157910708	2	3.76	KV453182.1	1064895	20-scf00019	20
AX-157910722	2	3.76	KV453182.1	1083592	20-scf00019	20
AX-157910727	2	3.76	KV453182.1	1093568	20-scf00019	20
AX-157910734	2	3.76	KV453182.1	1099770	20-scf00019	20
AX-157910743	2	3.76	KV453182.1	1108263	20-scf00019	20
AX-157910769	2	3.76	KV453182.1	1143898	20-scf00019	20
AX-157910773	2	3.76	KV453182.1	1152962	20-scf00019	20
AX-157910778	2	3.76	KV453182.1	1158671	20-scf00019	20
AX-157910728	2	3.799	KV453182.1	1094096	20-scf00019	20
AX-157910738	2	3.799	KV453182.1	1104629	20-scf00019	20
AX-157910744	2	3.799	KV453182.1	1110039	20-scf00019	20
AX-157910745	2	3.799	KV453182.1	1110459	20-scf00019	20
AX-157910746	2	3.799	KV453182.1	1112942	20-scf00019	20
AX-157910752	2	3.873	KV453182.1	1120135	20-scf00019	20
AX-157910757	2	3.958	KV453182.1	1128663	20-scf00019	20
AX-157910764	2	4.047	KV453182.1	1137715	20-scf00019	20
AX-158382189	2	4.145	KV453182.1	1120884	20-scf00019	20
AX-157910755	2	4.145	KV453182.1	1124542	20-scf00019	20
AX-157910756	2	4.145	KV453182.1	1126879	20-scf00019	20
AX-157910768	2	4.145	KV453182.1	1142841	20-scf00019	20
AX-157910771	2	4.237	KV453182.1	1149461	20-scf00019	20
AX-157910747	2	4.456	KV453182.1	1113631	20-scf00019	20
AX-157910774	2	4.456	KV453182.1	1153407	20-scf00019	20
AX-157910775	2	4.456	KV453182.1	1154582	20-scf00019	20
AX-157910777	2	4.456	KV453182.1	1157213	20-scf00019	20
AX-157910783	2	4.456	KV453182.1	1166110	20-scf00019	20
AX-157910785	2	4.456	KV453182.1	1167922	20-scf00019	20
AX-157910748	2	4.711	KV453182.1	1114010	20-scf00019	20
AX-157910786	2	4.711	KV453182.1	1170223	20-scf00019	20
AX-157910790	2	4.711	KV453182.1	1177396	20-scf00019	20
AX-157910810	2	4.711	KV453182.1	1211598	20-scf00019	20
AX-157910792	2	4.717	KV453182.1	1180149	20-scf00019	20
AX-157910794	2	5.221	KV453182.1	1181901	20-scf00019	20

AX-157910797	2	5.221	KV453182.1	1185520	20-scf00019	20
AX-157910798	2	5.221	KV453182.1	1187696	20-scf00019	20
AX-157910800	2	5.221	KV453182.1	1189054	20-scf00019	20
AX-157910801	2	5.221	KV453182.1	1194114	20-scf00019	20
AX-157720186	2	5.506	KV453182.1	1202736	20-scf00019	20
AX-157910808	2	5.693	KV453182.1	1208009	20-scf00019	20
AX-157754933	2	5.695	KV453182.1	1208538	20-scf00019	20
AX-157910820	2	5.91	KV453182.1	1224221	20-scf00019	20
AX-157910802	2	6.095	KV453182.1	1194333	20-scf00019	20
AX-157910815	2	6.095	KV453182.1	1218118	20-scf00019	20
AX-158379223	2	6.095	KV453182.1	1229642	20-scf00019	20
AX-157910842	2	6.095	KV453182.1	1252491	20-scf00019	20
AX-157910826	2	6.096	KV453182.1	1229759	20-scf00019	20
AX-157910831	2	6.096	KV453182.1	1240014	20-scf00019	20
AX-157910833	2	6.096	KV453182.1	1242595	20-scf00019	20
AX-157910835	2	6.096	KV453182.1	1245617	20-scf00019	20
AX-157883401	2	6.096	KV453182.1	1247446	20-scf00019	20
AX-157910837	2	6.096	KV453182.1	1247745	20-scf00019	20
AX-157910839	2	6.096	KV453182.1	1250387	20-scf00019	20
AX-157910840	2	6.096	KV453182.1	1251412	20-scf00019	20
AX-157910829	2	6.307	KV453182.1	1232934	20-scf00019	20
AX-157910841	2	6.307	KV453182.1	1252381	20-scf00019	20
AX-157910844	2	6.307	KV453182.1	1255448	20-scf00019	20
AX-157874589	2	6.307	KV453182.1	1257855	20-scf00019	20
AX-157812236	2	6.313	KV453182.1	1263141	20-scf00019	20
AX-157910851	2	6.32	KV453182.1	1265292	20-scf00019	20
AX-157910853	2	6.638	KV453182.1	1269317	20-scf00019	20
AX-157726774	2	6.638	KV453182.1	1291325	20-scf00019	20
AX-157910856	2	6.64	KV453182.1	1271852	20-scf00019	20
AX-157910854	2	6.642	KV453182.1	1269788	20-scf00019	20
AX-157910857	2	6.642	KV453182.1	1272405	20-scf00019	20
AX-157772910	2	6.642	KV453182.1	1272577	20-scf00019	20
AX-157910858	2	6.642	KV453182.1	1273606	20-scf00019	20
AX-157910860	2	6.642	KV453182.1	1274572	20-scf00019	20
AX-157910861	2	6.642	KV453182.1	1274776	20-scf00019	20
AX-157910862	2	6.642	KV453182.1	1275471	20-scf00019	20
AX-157910863	2	6.752	KV453182.1	1278779	20-scf00019	20
AX-157910864	2	6.838	KV453182.1	1279379	20-scf00019	20
AX-157910866	2	6.838	KV453182.1	1280796	20-scf00019	20
AX-157910867	2	6.919	KV453182.1	1281068	20-scf00019	20
AX-157910868	2	7.11	KV453182.1	1281481	20-scf00019	20
AX-157910849	2	7.253	KV453182.1	1260792	20-scf00019	20
AX-157812237	2	7.253	KV453182.1	1287516	20-scf00019	20
AX-157910873	2	7.253	KV453182.1	1290225	20-scf00019	20
AX-157910875	2	7.253	KV453182.1	1291837	20-scf00019	20
AX-157910877	2	7.253	KV453182.1	1293362	20-scf00019	20
AX-157910878	2	7.253	KV453182.1	1294547	20-scf00019	20
AX-157910884	2	7.253	KV453182.1	1301382	20-scf00019	20
AX-157751152	2	7.253	KV453182.1	1305489	20-scf00019	20
AX-157722214	2	7.253	KV453182.1	1309385	20-scf00019	20
AX-158404893	2	7.253	KV453182.1	1311562	20-scf00019	20

AX-158404894	2	7.253	KV453182.1	1311818	20-scf00019	20
AX-157910889	2	7.253	KV453182.1	1312164	20-scf00019	20
AX-157910890	2	7.253	KV453182.1	1312411	20-scf00019	20
AX-157910894	2	7.253	KV453182.1	1323495	20-scf00019	20
AX-157910906	2	7.253	KV453182.1	1337464	20-scf00019	20
AX-157910882	2	7.292	KV453182.1	1297647	20-scf00019	20
AX-157910891	2	7.292	KV453182.1	1316384	20-scf00019	20
AX-157853675	2	7.292	KV453182.1	1319472	20-scf00019	20
AX-157910896	2	7.292	KV453182.1	1325471	20-scf00019	20
AX-157910895	2	7.362	KV453182.1	1323766	20-scf00019	20
AX-157740103	2	7.362	KV453182.1	1329843	20-scf00019	20
AX-157910899	2	7.362	KV453182.1	1330566	20-scf00019	20
AX-157910892	2	7.471	KV453182.1	1318214	20-scf00019	20
AX-157910904	2	7.471	KV453182.1	1334264	20-scf00019	20
AX-157910908	2	7.471	KV453182.1	1339683	20-scf00019	20
AX-157910912	2	7.471	KV453182.1	1342142	20-scf00019	20
AX-157910913	2	7.471	KV453182.1	1343302	20-scf00019	20
AX-157772274	2	7.471	KV453182.1	1344821	20-scf00019	20
AX-157910915	2	7.471	KV453182.1	1346292	20-scf00019	20
AX-157910919	2	7.471	KV453182.1	1350948	20-scf00019	20
AX-157910921	2	7.471	KV453182.1	1353300	20-scf00019	20
AX-157910924	2	8.083	KV453182.1	1358243	20-scf00019	20
AX-157910925	2	8.083	KV453182.1	1359168	20-scf00019	20
AX-157910930	2	8.136	KV453182.1	1363402	20-scf00019	20
AX-157910910	2	8.237	KV453182.1	1341194	20-scf00019	20
AX-157910933	2	8.237	KV453182.1	1366924	20-scf00019	20
AX-157910934	2	8.237	KV453182.1	1368185	20-scf00019	20
AX-157910935	2	8.338	KV453182.1	1369805	20-scf00019	20
AX-157910936	2	8.367	KV453182.1	1371157	20-scf00019	20
AX-157910937	2	8.449	KV453182.1	1372248	20-scf00019	20
AX-157910939	2	8.449	KV453182.1	1374431	20-scf00019	20
AX-157910946	2	8.449	KV453182.1	1383980	20-scf00019	20
AX-157721375	2	8.449	KV453182.1	1385591	20-scf00019	20
AX-157910940	2	8.455	KV453182.1	1375114	20-scf00019	20
AX-157910943	2	8.46	KV453182.1	1380289	20-scf00019	20
AX-157910948	2	8.461	KV453182.1	1386173	20-scf00019	20
AX-158379224	2	8.76	KV453182.1	1391058	20-scf00019	20
AX-157910954	2	8.771	KV453182.1	1391193	20-scf00019	20
AX-157910955	2	8.774	KV453182.1	1391917	20-scf00019	20
AX-157910957	2	8.774	KV453182.1	1395461	20-scf00019	20
AX-157911007	2	9.516	KV453182.1	1452916	20-scf00019	20
AX-157911009	2	9.517	KV453182.1	1458737	20-scf00019	20
AX-157911012	2	9.646	KV453182.1	1460144	20-scf00019	20
AX-157911013	2	9.657	KV453182.1	1460692	20-scf00019	20
AX-157911014	2	9.66	KV453182.1	1463061	20-scf00019	20
AX-157911015	2	9.66	KV453182.1	1464163	20-scf00019	20
AX-157911017	2	9.66	KV453182.1	1468812	20-scf00019	20
AX-157911018	2	9.66	KV453182.1	1469817	20-scf00019	20
AX-157911019	2	9.66	KV453182.1	1470332	20-scf00019	20
AX-157911020	2	9.66	KV453182.1	1470777	20-scf00019	20
AX-157911021	2	9.66	KV453182.1	1472893	20-scf00019	20

AX-157911022	2	9.66	KV453182.1	1473455	20-scf00019	20
AX-157911023	2	9.66	KV453182.1	1473817	20-scf00019	20
AX-157911027	2	10.314	KV453182.1	1483949	20-scf00019	20
AX-157911036	2	10.314	KV453182.1	1503413	20-scf00019	20
AX-157753787	2	10.315	KV453182.1	1485133	20-scf00019	20
AX-157911028	2	10.315	KV453182.1	1491339	20-scf00019	20
AX-157911029	2	10.315	KV453182.1	1493169	20-scf00019	20
AX-157911030	2	10.315	KV453182.1	1493639	20-scf00019	20
AX-157911031	2	10.315	KV453182.1	1497532	20-scf00019	20
AX-157911032	2	10.315	KV453182.1	1498582	20-scf00019	20
AX-157911034	2	10.429	KV453182.1	1502127	20-scf00019	20
AX-157911038	2	10.534	KV453182.1	1504499	20-scf00019	20
AX-157911039	2	10.534	KV453182.1	1507493	20-scf00019	20
AX-157911040	2	10.534	KV453182.1	1508628	20-scf00019	20
AX-157911042	2	10.534	KV453182.1	1509919	20-scf00019	20
AX-157812240	2	10.534	KV453182.1	1510126	20-scf00019	20
AX-157911045	2	10.815	KV453182.1	1515375	20-scf00019	20
AX-157911046	2	10.924	KV453182.1	1516763	20-scf00019	20
AX-157911057	2	10.924	KV453182.1	1533638	20-scf00019	20
AX-157911049	2	11.027	KV453182.1	1518884	20-scf00019	20
AX-157911050	2	11.029	KV453182.1	1521733	20-scf00019	20
AX-158404895	2	11.03	KV453182.1	1522439	20-scf00019	20
AX-157911054	2	11.031	KV453182.1	1529446	20-scf00019	20
AX-157911066	2	11.031	KV453182.1	1554169	20-scf00019	20
AX-157911055	2	11.092	KV453182.1	1531518	20-scf00019	20
AX-157911059	2	11.181	KV453182.1	1540018	20-scf00019	20
AX-157911060	2	11.21	KV453182.1	1542116	20-scf00019	20
AX-157911047	2	11.518	KV453182.1	1517312	20-scf00019	20
AX-157911062	2	11.518	KV453182.1	1548890	20-scf00019	20
AX-157911064	2	11.518	KV453182.1	1550256	20-scf00019	20
AX-157911068	2	11.518	KV453182.1	1562751	20-scf00019	20
AX-157753662	2	11.518	KV453182.1	1564897	20-scf00019	20
AX-157911072	2	11.518	KV453182.1	1567005	20-scf00019	20
AX-157911073	2	11.518	KV453182.1	1567354	20-scf00019	20
AX-157911074	2	11.518	KV453182.1	1567722	20-scf00019	20
AX-157812242	2	11.518	KV453182.1	1568771	20-scf00019	20
AX-157911076	2	11.518	KV453182.1	1571797	20-scf00019	20
AX-157812244	2	11.518	KV453182.1	1573088	20-scf00019	20
AX-157911077	2	11.518	KV453182.1	1575254	20-scf00019	20
AX-157911078	2	11.518	KV453182.1	1576930	20-scf00019	20
AX-158421861	2	11.518	KV453182.1	1583279	20-scf00019	20
AX-157911107	2	11.518	KV453182.1	1624140	20-scf00019	20
AX-158421862	2	11.521	KV453182.1	1583435	20-scf00019	20
AX-157911085	2	11.521	KV453182.1	1589376	20-scf00019	20
AX-157911101	2	11.521	KV453182.1	1617921	20-scf00019	20
AX-157775100	2	11.521	KV453182.1	1680173	20-scf00019	20
AX-157911146	2	11.521	KV453182.1	1682001	20-scf00019	20
AX-157911084	2	11.955	KV453182.1	1587582	20-scf00019	20
AX-157911088	2	11.955	KV453182.1	1593352	20-scf00019	20
AX-157911090	2	11.955	KV453182.1	1597920	20-scf00019	20
AX-157742973	2	11.955	KV453182.1	1606727	20-scf00019	20

AX-157911131	2	11.955	KV453182.1	1660108	20-scf00019	20
AX-157911079	2	12.11	KV453182.1	1578197	20-scf00019	20
AX-157812245	2	12.11	KV453182.1	1602461	20-scf00019	20
AX-157777594	2	12.11	KV453182.1	1606146	20-scf00019	20
AX-157911094	2	12.11	KV453182.1	1607601	20-scf00019	20
AX-157812246	2	12.11	KV453182.1	1613366	20-scf00019	20
AX-157911122	2	12.11	KV453182.1	1647228	20-scf00019	20
AX-157911123	2	12.11	KV453182.1	1648194	20-scf00019	20
AX-157911124	2	12.11	KV453182.1	1652052	20-scf00019	20
AX-158404898	2	12.232	KV453182.1	1615460	20-scf00019	20
AX-157812248	2	12.325	KV453182.1	1620543	20-scf00019	20
AX-157911104	2	12.325	KV453182.1	1621682	20-scf00019	20
AX-157911106	2	12.325	KV453182.1	1623022	20-scf00019	20
AX-157911110	2	12.325	KV453182.1	1625710	20-scf00019	20
AX-157911115	2	12.325	KV453182.1	1629819	20-scf00019	20
AX-157740378	2	12.325	KV453182.1	1634625	20-scf00019	20
AX-157911116	2	12.325	KV453182.1	1634994	20-scf00019	20
AX-157911117	2	12.325	KV453182.1	1637203	20-scf00019	20
AX-157911125	2	12.325	KV453182.1	1653677	20-scf00019	20
AX-157911127	2	12.539	KV453182.1	1655409	20-scf00019	20
AX-157911129	2	12.754	KV453182.1	1657831	20-scf00019	20
AX-157911128	2	12.88	KV453182.1	1657265	20-scf00019	20
AX-157911130	2	12.88	KV453182.1	1658594	20-scf00019	20
AX-157716080	2	12.88	KV453182.1	1664954	20-scf00019	20
AX-158389556	2	12.88	KV453182.1	1674655	20-scf00019	20
AX-157911135	2	13.183	KV453182.1	1668684	20-scf00019	20
AX-157911137	2	13.183	KV453182.1	1669923	20-scf00019	20
AX-157911141	2	13.183	KV453182.1	1674386	20-scf00019	20
AX-157911144	2	13.184	KV453182.1	1677095	20-scf00019	20
AX-157911149	2	13.185	KV453182.1	1685388	20-scf00019	20
AX-157911151	2	13.794	KV453182.1	1687550	20-scf00019	20
AX-157911152	2	13.806	KV453182.1	1689646	20-scf00019	20
AX-157911155	2	13.827	KV453182.1	1694782	20-scf00019	20
AX-157911156	2	13.827	KV453182.1	1695539	20-scf00019	20
AX-157812251	2	13.934	KV453182.1	1678885	20-scf00019	20
AX-157812252	2	13.934	KV453182.1	1694029	20-scf00019	20
AX-157911160	2	13.934	KV453182.1	1705124	20-scf00019	20
AX-157911162	2	13.988	KV453182.1	1707432	20-scf00019	20
AX-157812254	2	13.988	KV453182.1	1708677	20-scf00019	20
AX-157911164	2	13.988	KV453182.1	1712600	20-scf00019	20
AX-157911167	2	14.146	KV453182.1	1717257	20-scf00019	20
AX-157911158	2	14.147	KV453182.1	1700824	20-scf00019	20
AX-157750432	2	14.147	KV453182.1	1717477	20-scf00019	20
AX-157738362	2	14.147	KV453182.1	1720255	20-scf00019	20
AX-157911169	2	14.147	KV453182.1	1722035	20-scf00019	20
AX-157911170	2	14.147	KV453182.1	1722557	20-scf00019	20
AX-158382196	2	14.147	KV453182.1	1722602	20-scf00019	20
AX-157911171	2	14.147	KV453182.1	1723308	20-scf00019	20
AX-157812256	2	14.147	KV453182.1	1725059	20-scf00019	20
AX-157911173	2	14.147	KV453182.1	1726429	20-scf00019	20
AX-157744058	2	14.147	KV453182.1	1728741	20-scf00019	20

AX-157911175	2	14.147	KV453182.1	1732582	20-scf00019	20
AX-157911176	2	14.147	KV453182.1	1732891	20-scf00019	20
AX-157715337	2	14.147	KV453182.1	1733969	20-scf00019	20
AX-157911177	2	14.147	KV453182.1	1735364	20-scf00019	20
AX-158379227	2	14.147	KV453182.1	1735641	20-scf00019	20
AX-157911180	2	14.147	KV453182.1	1738919	20-scf00019	20
AX-157911183	2	14.147	KV453182.1	1741498	20-scf00019	20
AX-157911184	2	14.147	KV453182.1	1741667	20-scf00019	20
AX-157869072	2	14.147	KV453182.1	1742082	20-scf00019	20
AX-157911186	2	14.147	KV453182.1	1744568	20-scf00019	20
AX-157911187	2	14.147	KV453182.1	1745418	20-scf00019	20
AX-157911189	2	14.147	KV453182.1	1747963	20-scf00019	20
AX-157911191	2	14.147	KV453182.1	1757349	20-scf00019	20
AX-157911192	2	14.147	KV453182.1	1758386	20-scf00019	20
AX-157911193	2	14.147	KV453182.1	1759782	20-scf00019	20
AX-157754943	2	14.147	KV453182.1	1760239	20-scf00019	20
AX-157911194	2	14.147	KV453182.1	1760847	20-scf00019	20
AX-157911195	2	14.147	KV453182.1	1763302	20-scf00019	20
AX-157911196	2	14.147	KV453182.1	1765405	20-scf00019	20
AX-157911197	2	14.147	KV453182.1	1772502	20-scf00019	20
AX-157812260	2	14.147	KV453182.1	1776996	20-scf00019	20
AX-157911198	2	14.147	KV453182.1	1782589	20-scf00019	20
AX-157911199	2	14.147	KV453182.1	1786202	20-scf00019	20
AX-158404139	2	14.366	KV453182.1	1737011	20-scf00019	20
AX-157911202	2	14.366	KV453182.1	1791029	20-scf00019	20
AX-157911206	2	14.366	KV453182.1	1796809	20-scf00019	20
AX-157911208	2	14.366	KV453182.1	1799980	20-scf00019	20
AX-157911209	2	14.366	KV453182.1	1801685	20-scf00019	20
AX-157911213	2	14.366	KV453182.1	1804671	20-scf00019	20
AX-157911161	2	14.693	KV453182.1	1705688	20-scf00019	20
AX-157911172	2	14.693	KV453182.1	1725813	20-scf00019	20
AX-157911174	2	14.693	KV453182.1	1729808	20-scf00019	20
AX-157812258	2	14.693	KV453182.1	1762304	20-scf00019	20
AX-157812259	2	14.693	KV453182.1	1762433	20-scf00019	20
AX-157703363	2	14.693	KV453182.1	1771818	20-scf00019	20
AX-157911203	2	14.693	KV453182.1	1791430	20-scf00019	20
AX-157911214	2	14.693	KV453182.1	1806225	20-scf00019	20
AX-157911217	2	14.693	KV453182.1	1810520	20-scf00019	20
AX-157911218	2	14.693	KV453182.1	1811009	20-scf00019	20
AX-157911219	2	14.693	KV453182.1	1811505	20-scf00019	20
AX-157911220	2	14.693	KV453182.1	1811864	20-scf00019	20
AX-157812261	2	14.693	KV453182.1	1814828	20-scf00019	20
AX-157911221	2	14.693	KV453182.1	1816458	20-scf00019	20
AX-157911222	2	14.693	KV453182.1	1817660	20-scf00019	20
AX-157911223	2	14.693	KV453182.1	1818687	20-scf00019	20
AX-157812263	2	14.693	KV453182.1	1822570	20-scf00019	20
AX-157746031	2	15.021	KV453182.1	1827462	20-scf00019	20
AX-157911229	2	15.021	KV453182.1	1831565	20-scf00019	20
AX-157911230	2	15.021	KV453182.1	1832405	20-scf00019	20
AX-157722909	2	15.021	KV453182.1	1834332	20-scf00019	20
AX-157751630	2	15.032	KV453182.1	1846054	20-scf00019	20

AX-157911239	2	15.13	KV453182.1	1852590	20-scf00019	20
AX-157911240	2	15.13	KV453182.1	1853480	20-scf00019	20
AX-157911241	2	15.13	KV453182.1	1853833	20-scf00019	20
AX-157738691	2	15.13	KV453182.1	1854319	20-scf00019	20
AX-157911242	2	15.13	KV453182.1	1854372	20-scf00019	20
AX-157750368	2	15.13	KV453182.1	1856472	20-scf00019	20
AX-157864470	2	15.13	KV453182.1	1864373	20-scf00019	20
AX-157911250	2	15.13	KV453182.1	1865315	20-scf00019	20
AX-157812272	2	15.537	KV453182.1	1873774	20-scf00019	20
AX-158379229	2	15.54	KV453182.1	1874447	20-scf00019	20
AX-157812273	2	15.54	KV453182.1	1876578	20-scf00019	20
AX-157812274	2	15.54	KV453182.1	1877697	20-scf00019	20
AX-157744333	2	15.54	KV453182.1	1877949	20-scf00019	20
AX-157911253	2	15.711	KV453182.1	1879834	20-scf00019	20
AX-157727619	2	16.225	KV453182.1	1901172	20-scf00019	20
AX-157911266	2	16.225	KV453182.1	1902941	20-scf00019	20
AX-157911267	2	16.225	KV453182.1	1903699	20-scf00019	20
AX-157911277	2	16.225	KV453182.1	1915756	20-scf00019	20
AX-157774221	2	16.225	KV453182.1	1926729	20-scf00019	20
AX-157911271	2	16.335	KV453182.1	1907626	20-scf00019	20
AX-157911272	2	16.335	KV453182.1	1908846	20-scf00019	20
AX-157911273	2	16.335	KV453182.1	1909944	20-scf00019	20
AX-157911274	2	16.335	KV453182.1	1910634	20-scf00019	20
AX-157911275	2	16.444	KV453182.1	1912049	20-scf00019	20
AX-157812275	2	16.553	KV453182.1	1924235	20-scf00019	20
AX-157911285	2	16.553	KV453182.1	1924578	20-scf00019	20
AX-157911286	2	16.553	KV453182.1	1924998	20-scf00019	20
AX-157911287	2	16.553	KV453182.1	1927448	20-scf00019	20
AX-157911290	2	16.554	KV453182.1	1931342	20-scf00019	20
AX-157911292	2	16.653	KV453182.1	1933232	20-scf00019	20
AX-157911284	2	16.717	KV453182.1	1924083	20-scf00019	20
AX-157911294	2	16.717	KV453182.1	1937979	20-scf00019	20
AX-157853679	2	16.881	KV453182.1	1938630	20-scf00019	20
AX-157911295	2	16.881	KV453182.1	1938684	20-scf00019	20
AX-157911297	2	16.881	KV453182.1	1941520	20-scf00019	20
AX-157911300	2	16.881	KV453182.1	1945604	20-scf00019	20
AX-157721313	2	16.881	KV453182.1	1945979	20-scf00019	20
AX-157911301	2	16.881	KV453182.1	1946361	20-scf00019	20
AX-157754823	2	16.882	KV453182.1	1946782	20-scf00019	20
AX-157911303	2	16.992	KV453182.1	1948375	20-scf00019	20
AX-157911304	2	17.045	KV453182.1	1949690	20-scf00019	20
AX-157911306	2	17.045	KV453182.1	1950581	20-scf00019	20
AX-157911311	2	17.208	KV453182.1	1954496	20-scf00019	20
AX-157911313	2	17.209	KV453182.1	1955789	20-scf00019	20
AX-157911315	2	17.209	KV453182.1	1960379	20-scf00019	20
AX-158382202	2	17.209	KV453182.1	1963902	20-scf00019	20
AX-157911320	2	17.209	KV453182.1	1966963	20-scf00019	20
AX-157911321	2	17.209	KV453182.1	1967355	20-scf00019	20
AX-157911322	2	17.209	KV453182.1	1968374	20-scf00019	20
AX-158382203	2	17.209	KV453182.1	1969089	20-scf00019	20
AX-157911324	2	17.209	KV453182.1	1969475	20-scf00019	20

AX-157743939	2	17.215	KV453182.1	1970738	20-scf00019	20
AX-157911327	2	17.64	KV453182.1	1978923	20-scf00019	20
AX-157911329	2	17.643	KV453182.1	1984427	20-scf00019	20
AX-157911330	2	17.643	KV453182.1	1984774	20-scf00019	20
AX-157911332	2	17.646	KV453182.1	1985497	20-scf00019	20
AX-157911335	2	17.646	KV453182.1	1988663	20-scf00019	20
AX-157864471	2	17.646	KV453182.1	1988982	20-scf00019	20
AX-157911339	2	17.646	KV453182.1	1992887	20-scf00019	20
AX-157911343	2	17.646	KV453182.1	1994719	20-scf00019	20
AX-157911379	2	17.646	KV453182.1	2031939	20-scf00019	20
AX-157911342	2	17.788	KV453182.1	1994276	20-scf00019	20
AX-157911331	2	17.908	KV453182.1	1985287	20-scf00019	20
AX-157911338	2	17.908	KV453182.1	1991986	20-scf00019	20
AX-157911341	2	17.908	KV453182.1	1993894	20-scf00019	20
AX-157911349	2	17.908	KV453182.1	1999089	20-scf00019	20
AX-157911350	2	17.908	KV453182.1	1999474	20-scf00019	20
AX-157911354	2	17.908	KV453182.1	2005511	20-scf00019	20
AX-157911357	2	17.908	KV453182.1	2008150	20-scf00019	20
AX-157911361	2	18.039	KV453182.1	2010827	20-scf00019	20
AX-157911358	2	18.302	KV453182.1	2009145	20-scf00019	20
AX-157755518	2	18.302	KV453182.1	2022513	20-scf00019	20
AX-157772719	2	18.302	KV453182.1	2024362	20-scf00019	20
AX-157911374	2	18.302	KV453182.1	2025338	20-scf00019	20
AX-157911375	2	18.302	KV453182.1	2026268	20-scf00019	20
AX-157726676	2	18.302	KV453182.1	2028282	20-scf00019	20
AX-157911376	2	18.326	KV453182.1	2029508	20-scf00019	20
AX-157911377	2	18.369	KV453182.1	2030075	20-scf00019	20
AX-157911380	2	18.411	KV453182.1	2032709	20-scf00019	20
AX-157911367	2	18.456	KV453182.1	2016660	20-scf00019	20
AX-157864473	2	18.456	KV453182.1	2028142	20-scf00019	20
AX-158404906	2	18.456	KV453182.1	2038120	20-scf00019	20
AX-157911402	2	18.456	KV453182.1	2066761	20-scf00019	20
AX-157911384	2	18.493	KV453182.1	2040237	20-scf00019	20
AX-157753464	2	18.576	KV453182.1	2044258	20-scf00019	20
AX-157911389	2	18.651	KV453182.1	2046666	20-scf00019	20
AX-157911391	2	18.74	KV453182.1	2048918	20-scf00019	20
AX-157753589	2	18.74	KV453182.1	2054684	20-scf00019	20
AX-157911398	2	18.74	KV453182.1	2060740	20-scf00019	20
AX-157911401	2	18.742	KV453182.1	2064602	20-scf00019	20
AX-157911406	2	19.063	KV453182.1	2073004	20-scf00019	20
AX-157911413	2	19.067	KV453182.1	2084696	20-scf00019	20
AX-157911414	2	19.067	KV453182.1	2085481	20-scf00019	20
AX-157775366	2	19.067	KV453182.1	2085896	20-scf00019	20
AX-157911417	2	19.067	KV453182.1	2088147	20-scf00019	20
AX-157911418	2	19.067	KV453182.1	2090934	20-scf00019	20
AX-157740388	2	19.34	KV453182.1	2012332	20-scf00019	20
AX-158404910	2	19.34	KV453182.1	2092922	20-scf00019	20
AX-157911421	2	19.34	KV453182.1	2094509	20-scf00019	20
AX-157911481	2	19.34	KV453182.1	2191295	20-scf00019	20
AX-157911499	2	19.34	KV453182.1	2218012	20-scf00019	20
AX-157911424	2	19.613	KV453182.1	2102090	20-scf00019	20

AX-157911425	2	19.613	KV453182.1	2103213	20-scf00019	20
AX-157911428	2	19.613	KV453182.1	2106036	20-scf00019	20
AX-157911429	2	19.613	KV453182.1	2108593	20-scf00019	20
AX-157911433	2	19.613	KV453182.1	2113850	20-scf00019	20
AX-157911435	2	19.759	KV453182.1	2116934	20-scf00019	20
AX-157911436	2	20.05	KV453182.1	2119101	20-scf00019	20
AX-157911437	2	20.05	KV453182.1	2121077	20-scf00019	20
AX-157911438	2	20.05	KV453182.1	2122200	20-scf00019	20
AX-157883407	2	20.05	KV453182.1	2125213	20-scf00019	20
AX-157911442	2	20.05	KV453182.1	2125611	20-scf00019	20
AX-157911443	2	20.05	KV453182.1	2127682	20-scf00019	20
AX-157721623	2	20.05	KV453182.1	2129111	20-scf00019	20
AX-157911444	2	20.05	KV453182.1	2130327	20-scf00019	20
AX-157721917	2	20.05	KV453182.1	2131715	20-scf00019	20
AX-157738508	2	20.05	KV453182.1	2133968	20-scf00019	20
AX-157911447	2	20.487	KV453182.1	2137434	20-scf00019	20
AX-157911450	2	20.487	KV453182.1	2140917	20-scf00019	20
AX-158404144	2	20.487	KV453182.1	2144189	20-scf00019	20
AX-158404911	2	20.487	KV453182.1	2144243	20-scf00019	20
AX-157911459	2	20.487	KV453182.1	2156146	20-scf00019	20
AX-157911463	2	20.487	KV453182.1	2161116	20-scf00019	20
AX-157911454	2	20.596	KV453182.1	2151491	20-scf00019	20
AX-157911457	2	20.596	KV453182.1	2154388	20-scf00019	20
AX-157911458	2	20.596	KV453182.1	2155134	20-scf00019	20
AX-157911464	2	20.596	KV453182.1	2162464	20-scf00019	20
AX-157753399	2	20.596	KV453182.1	2164469	20-scf00019	20
AX-157911467	2	20.596	KV453182.1	2164947	20-scf00019	20
AX-157874600	2	20.596	KV453182.1	2168366	20-scf00019	20
AX-157710812	2	20.596	KV453182.1	2170780	20-scf00019	20
AX-157911470	2	20.596	KV453182.1	2171193	20-scf00019	20
AX-157758628	2	20.596	KV453182.1	2173451	20-scf00019	20
AX-157911471	2	20.596	KV453182.1	2174375	20-scf00019	20
AX-157911472	2	20.596	KV453182.1	2176930	20-scf00019	20
AX-157812278	2	20.705	KV453182.1	2183637	20-scf00019	20
AX-157911474	2	20.705	KV453182.1	2184215	20-scf00019	20
AX-157812279	2	20.705	KV453182.1	2187728	20-scf00019	20
AX-157911477	2	20.705	KV453182.1	2188156	20-scf00019	20
AX-157911480	2	20.815	KV453182.1	2190383	20-scf00019	20
AX-157812280	2	21.033	KV453182.1	2192039	20-scf00019	20
AX-157812281	2	21.033	KV453182.1	2195999	20-scf00019	20
AX-157911483	2	21.033	KV453182.1	2197204	20-scf00019	20
AX-157911484	2	21.033	KV453182.1	2197633	20-scf00019	20
AX-157911485	2	21.033	KV453182.1	2198237	20-scf00019	20
AX-157722892	2	21.033	KV453182.1	2215292	20-scf00019	20
AX-157911487	2	21.088	KV453182.1	2201435	20-scf00019	20
AX-157708496	2	21.143	KV453182.1	2207223	20-scf00019	20
AX-157911494	2	21.143	KV453182.1	2209191	20-scf00019	20
AX-157911496	2	21.143	KV453182.1	2213726	20-scf00019	20
AX-157911498	2	21.143	KV453182.1	2216207	20-scf00019	20
AX-157883409	2	21.143	KV453182.1	2217974	20-scf00019	20
AX-157911430	2	21.17	KV453182.1	2109696	20-scf00019	20

AX-157911493	2	21.17	KV453182.1	2205938	20-scf00019	20
AX-157750881	2	21.237	KV453182.1	2218590	20-scf00019	20
AX-157911502	2	21.294	KV453182.1	2221040	20-scf00019	20
AX-157911503	2	21.534	KV453182.1	2222040	20-scf00019	20
AX-157911508	2	21.536	KV453182.1	2227684	20-scf00019	20
AX-157911506	2	21.538	KV453182.1	2224494	20-scf00019	20
AX-157755835	2	21.538	KV453182.1	2232320	20-scf00019	20
AX-157911515	2	21.689	KV453182.1	2239586	20-scf00019	20
AX-158404913	2	21.689	KV453182.1	2240832	20-scf00019	20
AX-158421866	2	21.689	KV453182.1	2241522	20-scf00019	20
AX-157911516	2	21.689	KV453182.1	2242946	20-scf00019	20
AX-157717259	2	21.689	KV453182.1	2244061	20-scf00019	20
AX-157911518	2	21.689	KV453182.1	2244808	20-scf00019	20
AX-158389560	2	21.689	KV453182.1	2248449	20-scf00019	20
AX-157812285	2	21.689	KV453182.1	2249610	20-scf00019	20
AX-157869075	2	21.689	KV453182.1	2256507	20-scf00019	20
AX-157874602	2	21.689	KV453182.1	2260281	20-scf00019	20
AX-157739420	2	21.689	KV453182.1	2261928	20-scf00019	20
AX-157911522	2	21.735	KV453182.1	2260436	20-scf00019	20
AX-157911524	2	21.82	KV453182.1	2263847	20-scf00019	20
AX-157911525	2	21.82	KV453182.1	2264553	20-scf00019	20
AX-157911526	2	21.832	KV453182.1	2265510	20-scf00019	20
AX-157911540	2	22.344	KV453182.1	2285350	20-scf00019	20
AX-157749613	2	22.344	KV453182.1	2286177	20-scf00019	20
AX-157911541	2	22.344	KV453182.1	2286822	20-scf00019	20
AX-157911542	2	22.344	KV453182.1	2290804	20-scf00019	20
AX-157911543	2	22.344	KV453182.1	2292284	20-scf00019	20
AX-157911547	2	22.344	KV453182.1	2296364	20-scf00019	20
AX-157911549	2	22.344	KV453182.1	2297354	20-scf00019	20
AX-157911557	2	22.344	KV453182.1	2314121	20-scf00019	20
AX-157812287	2	22.344	KV453182.1	2343172	20-scf00019	20
AX-157911580	2	22.344	KV453182.1	2346021	20-scf00019	20
AX-157911550	2	22.569	KV453182.1	2297583	20-scf00019	20
AX-157911559	2	22.671	KV453182.1	2318836	20-scf00019	20
AX-157911565	2	22.671	KV453182.1	2326948	20-scf00019	20
AX-157911553	2	22.672	KV453182.1	2310810	20-scf00019	20
AX-157911563	2	22.672	KV453182.1	2323366	20-scf00019	20
AX-157911566	2	22.672	KV453182.1	2327785	20-scf00019	20
AX-157911569	2	22.672	KV453182.1	2329816	20-scf00019	20
AX-157744259	2	22.672	KV453182.1	2330950	20-scf00019	20
AX-157911570	2	22.672	KV453182.1	2332351	20-scf00019	20
AX-157911574	2	22.672	KV453182.1	2337583	20-scf00019	20
AX-157812286	2	22.672	KV453182.1	2339213	20-scf00019	20
AX-157911576	2	22.672	KV453182.1	2339841	20-scf00019	20
AX-157911578	2	22.672	KV453182.1	2344298	20-scf00019	20
AX-157746098	2	22.672	KV453182.1	2344617	20-scf00019	20
AX-157911582	2	22.672	KV453182.1	2355676	20-scf00019	20
AX-157719594	2	22.672	KV453182.1	2365032	20-scf00019	20
AX-157812293	2	22.672	KV453182.1	2365267	20-scf00019	20
AX-157911589	2	22.672	KV453182.1	2367934	20-scf00019	20
AX-157911585	2	22.781	KV453182.1	2361524	20-scf00019	20

AX-157911587	2	22.781	KV453182.1	2363611	20-scf00019	20
AX-157911590	2	22.781	KV453182.1	2368296	20-scf00019	20
AX-157707537	2	22.781	3ML01034512	388	scf10188_1	UN
AX-157911545	2	22.925	KV453182.1	2295025	20-scf00019	20
AX-157911555	2	22.925	KV453182.1	2313311	20-scf00019	20
AX-157911562	2	22.925	KV453182.1	2321886	20-scf00019	20
AX-157911594	2	22.925	KV453182.1	2370688	20-scf00019	20
AX-157911595	2	22.925	KV453182.1	2372327	20-scf00019	20
AX-157911596	2	22.925	KV453182.1	2373902	20-scf00019	20
AX-157911599	2	22.925	KV453182.1	2377877	20-scf00019	20
AX-157725026	2	22.925	KV453182.1	2378818	20-scf00019	20
AX-157911602	2	22.925	KV453182.1	2380267	20-scf00019	20
AX-157911604	2	22.925	KV453182.1	2381785	20-scf00019	20
AX-157911605	2	22.925	KV453182.1	2382849	20-scf00019	20
AX-157911606	2	22.925	KV453182.1	2383339	20-scf00019	20
AX-157812297	2	22.925	KV453182.1	2383706	20-scf00019	20
AX-157911607	2	22.925	KV453182.1	2383948	20-scf00019	20
AX-157741883	2	22.925	KV453182.1	2384406	20-scf00019	20
AX-157911608	2	22.925	KV453182.1	2384759	20-scf00019	20
AX-157775338	2	22.925	KV453182.1	2387049	20-scf00019	20
AX-157812298	2	22.925	KV453182.1	2387132	20-scf00019	20
AX-157812301	2	22.925	KV453182.1	2390811	20-scf00019	20
AX-157812302	2	22.925	KV453182.1	2392585	20-scf00019	20
AX-157911560	2	23.052	KV453182.1	2320498	20-scf00019	20
AX-157911598	2	23.052	KV453182.1	2375037	20-scf00019	20
AX-157869078	2	23.052	KV453182.1	2393720	20-scf00019	20
AX-157727768	2	23.069	KV453182.1	2396751	20-scf00019	20
AX-157911610	2	23.069	KV453182.1	2399867	20-scf00019	20
AX-157911613	2	23.276	KV453182.1	2404070	20-scf00019	20
AX-157911614	2	23.492	KV453182.1	2404326	20-scf00019	20
AX-157911615	2	23.494	KV453182.1	2404724	20-scf00019	20
AX-157911616	2	23.494	KV453182.1	2407003	20-scf00019	20
AX-157911619	2	23.494	KV453182.1	2408441	20-scf00019	20
AX-157911622	2	23.494	KV453182.1	2411912	20-scf00019	20
AX-157911620	2	23.506	KV453182.1	2409034	20-scf00019	20
AX-157911621	2	23.509	KV453182.1	2411029	20-scf00019	20
AX-157740559	2	23.787	KV453182.1	2414476	20-scf00019	20
AX-157911624	2	23.787	KV453182.1	2414972	20-scf00019	20
AX-157812305	2	23.787	KV453182.1	2415250	20-scf00019	20
AX-157714223	2	23.787	KV453182.1	2417460	20-scf00019	20
AX-157771622	2	23.787	KV453182.1	2418418	20-scf00019	20
AX-157911626	2	23.787	KV453182.1	2420007	20-scf00019	20
AX-157911627	2	23.787	KV453182.1	2420341	20-scf00019	20
AX-157911652	2	23.787	KV453182.1	2455268	20-scf00019	20
AX-157773418	2	23.88	KV453182.1	2421134	20-scf00019	20
AX-157812306	2	24.075	KV453182.1	2425232	20-scf00019	20
AX-157911629	2	24.075	KV453182.1	2426553	20-scf00019	20
AX-157911631	2	24.075	KV453182.1	2430654	20-scf00019	20
AX-157911633	2	24.075	KV453182.1	2432134	20-scf00019	20
AX-157911634	2	24.075	KV453182.1	2432531	20-scf00019	20
AX-157911635	2	24.075	KV453182.1	2434019	20-scf00019	20

AX-157911657	2	24.075	KV453182.1	2460943	20-scf00019	20
AX-157911666	2	24.075	KV453182.1	2477554	20-scf00019	20
AX-157911675	2	24.075	KV453182.1	2494349	20-scf00019	20
AX-157911692	2	24.075	KV453182.1	2525374	20-scf00019	20
AX-157911639	2	24.12	KV453182.1	2437161	20-scf00019	20
AX-157812308	2	24.173	KV453182.1	2441212	20-scf00019	20
AX-157911648	2	24.173	KV453182.1	2450647	20-scf00019	20
AX-157812311	2	24.173	KV453182.1	2475500	20-scf00019	20
AX-157911637	2	24.219	KV453182.1	2435095	20-scf00019	20
AX-157812310	2	24.219	KV453182.1	2447468	20-scf00019	20
AX-157911655	2	24.219	KV453182.1	2458257	20-scf00019	20
AX-157911658	2	24.219	KV453182.1	2462603	20-scf00019	20
AX-157911661	2	24.219	KV453182.1	2468484	20-scf00019	20
AX-157911662	2	24.219	KV453182.1	2469467	20-scf00019	20
AX-157911663	2	24.219	KV453182.1	2470990	20-scf00019	20
AX-157777625	2	24.219	KV453182.1	2474454	20-scf00019	20
AX-157911665	2	24.219	KV453182.1	2475799	20-scf00019	20
AX-158379234	2	24.219	KV453182.1	2479495	20-scf00019	20
AX-157696588	2	24.219	KV453182.1	2486179	20-scf00019	20
AX-157911693	2	24.219	KV453182.1	2528914	20-scf00019	20
AX-157911716	2	24.219	KV453182.1	2568728	20-scf00019	20
AX-157911670	2	24.506	KV453182.1	2483750	20-scf00019	20
AX-157812312	2	24.506	KV453182.1	2484248	20-scf00019	20
AX-157911678	2	24.578	KV453182.1	2498291	20-scf00019	20
AX-157911727	2	24.578	KV453182.1	2588450	20-scf00019	20
AX-157911642	2	24.65	KV453182.1	2442497	20-scf00019	20
AX-157911643	2	24.65	KV453182.1	2443095	20-scf00019	20
AX-157723393	2	24.65	KV453182.1	2489607	20-scf00019	20
AX-157911676	2	24.65	KV453182.1	2495138	20-scf00019	20
AX-157911679	2	24.65	KV453182.1	2498923	20-scf00019	20
AX-157911681	2	24.65	KV453182.1	2500742	20-scf00019	20
AX-157911682	2	24.65	KV453182.1	2503551	20-scf00019	20
AX-157911683	2	24.65	KV453182.1	2503956	20-scf00019	20
AX-157911686	2	24.65	KV453182.1	2509349	20-scf00019	20
AX-157911687	2	24.65	KV453182.1	2511226	20-scf00019	20
AX-157770891	2	24.65	KV453182.1	2512247	20-scf00019	20
AX-157911688	2	24.65	KV453182.1	2512320	20-scf00019	20
AX-158404917	2	24.65	KV453182.1	2521672	20-scf00019	20
AX-157771521	2	24.65	KV453182.1	2524122	20-scf00019	20
AX-157812317	2	24.65	KV453182.1	2533426	20-scf00019	20
AX-157911695	2	24.65	KV453182.1	2536901	20-scf00019	20
AX-157911696	2	24.65	KV453182.1	2540377	20-scf00019	20
AX-157727075	2	24.65	KV453182.1	2546297	20-scf00019	20
AX-157911698	2	24.794	KV453182.1	2542177	20-scf00019	20
AX-157911699	2	24.794	KV453182.1	2542339	20-scf00019	20
AX-157911702	2	24.831	KV453182.1	2546475	20-scf00019	20
AX-157911703	2	24.831	KV453182.1	2547517	20-scf00019	20
AX-157911706	2	24.831	KV453182.1	2556303	20-scf00019	20
AX-157812319	2	24.866	KV453182.1	2546851	20-scf00019	20
AX-157911737	2	24.866	KV453182.1	2603706	20-scf00019	20
AX-157911704	2	24.91	KV453182.1	2547831	20-scf00019	20

AX-157719706	2	24.938	KV453182.1	2555922	20-scf00019	20
AX-157911708	2	24.938	KV453182.1	2557467	20-scf00019	20
AX-157911709	2	24.938	KV453182.1	2558329	20-scf00019	20
AX-158389563	2	24.978	KV453182.1	2558729	20-scf00019	20
AX-157911711	2	25.018	KV453182.1	2561273	20-scf00019	20
AX-157812320	2	25.042	KV453182.1	2563439	20-scf00019	20
AX-157911714	2	25.082	KV453182.1	2566411	20-scf00019	20
AX-157911715	2	25.082	KV453182.1	2567383	20-scf00019	20
AX-157864479	2	25.082	KV453182.1	2578807	20-scf00019	20
AX-157911723	2	25.082	KV453182.1	2581434	20-scf00019	20
AX-158379235	2	25.191	KV453182.1	2593460	20-scf00019	20
AX-157911731	2	25.191	KV453182.1	2594227	20-scf00019	20
AX-157911735	2	25.205	KV453182.1	2599526	20-scf00019	20
AX-157911741	2	25.605	KV453182.1	2609651	20-scf00019	20
AX-157911742	2	25.605	KV453182.1	2611630	20-scf00019	20
AX-157911744	2	25.609	KV453182.1	2613537	20-scf00019	20
AX-157911745	2	25.819	KV453182.1	2614367	20-scf00019	20
AX-157911764	2	26.393	KV453182.1	2642086	20-scf00019	20
AX-157911765	2	26.445	KV453182.1	2642706	20-scf00019	20
AX-157911768	2	26.585	KV453182.1	2647184	20-scf00019	20
AX-157911769	2	26.725	KV453182.1	2649088	20-scf00019	20
AX-157911771	2	26.725	KV453182.1	2654040	20-scf00019	20
AX-157883414	2	26.725	KV453182.1	2654874	20-scf00019	20
AX-157911773	2	26.725	KV453182.1	2655327	20-scf00019	20
AX-157911777	2	26.725	KV453182.1	2658709	20-scf00019	20
AX-157911776	2	26.888	KV453182.1	2657514	20-scf00019	20
AX-157911779	2	26.977	KV453182.1	2662176	20-scf00019	20
AX-157911782	2	26.977	KV453182.1	2665178	20-scf00019	20
AX-157911778	2	27.052	KV453182.1	2660320	20-scf00019	20
AX-157911780	2	27.052	KV453182.1	2663180	20-scf00019	20
AX-157911784	2	27.052	KV453182.1	2668672	20-scf00019	20
AX-157911786	2	27.052	KV453182.1	2670131	20-scf00019	20
AX-157911794	2	27.052	KV453182.1	2680733	20-scf00019	20
AX-157911790	2	27.053	KV453182.1	2677023	20-scf00019	20
AX-157911793	2	27.053	KV453182.1	2680063	20-scf00019	20
AX-157911796	2	27.053	KV453182.1	2685149	20-scf00019	20
AX-158379236	2	27.065	KV453182.1	2686068	20-scf00019	20
AX-157911799	2	27.42	KV453182.1	2690999	20-scf00019	20
AX-157911800	2	27.584	KV453182.1	2692497	20-scf00019	20
AX-157883416	2	27.584	KV453182.1	2733395	20-scf00019	20
AX-157911806	2	27.599	KV453182.1	2704920	20-scf00019	20
AX-157911807	2	27.599	KV453182.1	2706865	20-scf00019	20
AX-157911809	2	27.599	KV453182.1	2709053	20-scf00019	20
AX-157911810	2	27.599	KV453182.1	2710026	20-scf00019	20
AX-157911811	2	27.599	KV453182.1	2710767	20-scf00019	20
AX-157911813	2	27.599	KV453182.1	2716309	20-scf00019	20
AX-157911812	2	27.763	KV453182.1	2712473	20-scf00019	20
AX-157911815	2	27.927	KV453182.1	2718278	20-scf00019	20
AX-157752048	2	27.927	KV453182.1	2732518	20-scf00019	20
AX-157911819	2	27.942	KV453182.1	2733435	20-scf00019	20
AX-157911822	2	27.958	KV453182.1	2738867	20-scf00019	20

AX-157911823	2	28.083	KV453182.1	2739353	20-scf00019	20
AX-157911804	2	28.127	KV453182.1	2699599	20-scf00019	20
AX-157911826	2	28.246	KV453182.1	2746652	20-scf00019	20
AX-157723080	2	28.246	KV453182.1	2750716	20-scf00019	20
AX-157738457	2	28.438	KV453182.1	2752331	20-scf00019	20
AX-158382219	2	28.911	KV453182.1	2754793	20-scf00019	20
AX-157727268	2	28.911	KV453182.1	2757712	20-scf00019	20
AX-158389569	2	28.911	KV453182.1	2759200	20-scf00019	20
AX-157721805	2	29.021	KV453182.1	2759744	20-scf00019	20
AX-157911834	2	29.021	KV453182.1	2761623	20-scf00019	20
AX-157911734	2	29.03	KV453182.1	2599109	20-scf00019	20
AX-157911835	2	29.03	KV453182.1	2767020	20-scf00019	20
AX-157725743	2	29.03	KV453182.1	2768802	20-scf00019	20
AX-157911836	2	29.03	KV453182.1	2770021	20-scf00019	20
AX-157911837	2	29.183	KV453182.1	2770456	20-scf00019	20
AX-157726935	2	29.348	KV453182.1	2777857	20-scf00019	20
AX-157911829	2	29.677	KV453182.1	2752865	20-scf00019	20
AX-157753129	2	29.677	KV453182.1	2767523	20-scf00019	20
AX-157911845	2	29.677	KV453182.1	2782781	20-scf00019	20
AX-157911846	2	29.677	KV453182.1	2785860	20-scf00019	20
AX-157911848	2	29.677	KV453182.1	2789910	20-scf00019	20
AX-157911849	2	29.677	KV453182.1	2790670	20-scf00019	20
AX-157911852	2	29.677	KV453182.1	2793281	20-scf00019	20
AX-157911855	2	29.677	KV453182.1	2796640	20-scf00019	20
AX-157750800	2	29.677	KV453182.1	2799150	20-scf00019	20
AX-157756444	2	29.677	KV453182.1	2801784	20-scf00019	20
AX-157911856	2	29.677	KV453182.1	2804434	20-scf00019	20
AX-157911857	2	29.677	KV453182.1	2804589	20-scf00019	20
AX-157911859	2	29.677	KV453182.1	2805571	20-scf00019	20
AX-157911860	2	29.677	KV453182.1	2805932	20-scf00019	20
AX-157911861	2	29.677	KV453182.1	2809386	20-scf00019	20
AX-157911862	2	29.677	KV453182.1	2819202	20-scf00019	20
AX-157911863	2	29.677	KV453182.1	2820911	20-scf00019	20
AX-157911866	2	29.677	KV453182.1	2822590	20-scf00019	20
AX-157773838	2	29.677	KV453182.1	2827649	20-scf00019	20
AX-157911870	2	29.677	KV453182.1	2829269	20-scf00019	20
AX-157911871	2	29.677	KV453182.1	2830328	20-scf00019	20
AX-157911875	2	29.677	KV453182.1	2835423	20-scf00019	20
AX-157911876	2	29.677	KV453182.1	2835783	20-scf00019	20
AX-157911881	2	29.677	KV453182.1	2841727	20-scf00019	20
AX-157911883	2	29.677	KV453182.1	2843346	20-scf00019	20
AX-157874619	2	29.677	KV453182.1	2844181	20-scf00019	20
AX-157911885	2	29.677	KV453182.1	2846645	20-scf00019	20
AX-157911886	2	29.677	KV453182.1	2850208	20-scf00019	20
AX-157755138	2	29.677	KV453182.1	2852575	20-scf00019	20
AX-157911887	2	29.677	KV453182.1	2854222	20-scf00019	20
AX-157911888	2	29.677	KV453182.1	2854996	20-scf00019	20
AX-157911889	2	29.677	KV453182.1	2855855	20-scf00019	20
AX-157911897	2	29.677	KV453182.1	2863956	20-scf00019	20
AX-157911891	2	29.888	KV453182.1	2858956	20-scf00019	20
AX-157911896	2	29.906	KV453182.1	2862514	20-scf00019	20

AX-157911907	2	30.166	KV453182.1	2879553	20-scf00019	20
AX-157911909	2	30.176	KV453182.1	2883333	20-scf00019	20
AX-157911912	2	30.179	KV453182.1	2886739	20-scf00019	20
AX-157911910	2	30.553	KV453182.1	2884543	20-scf00019	20
AX-157755409	2	30.553	KV453182.1	2887921	20-scf00019	20
AX-157911915	2	30.553	KV453182.1	2890808	20-scf00019	20
AX-157911917	2	30.553	KV453182.1	2892424	20-scf00019	20
AX-158382221	2	30.553	KV453182.1	2893749	20-scf00019	20
AX-157911918	2	30.662	KV453182.1	2895670	20-scf00019	20
AX-157911921	2	30.662	KV453182.1	2898122	20-scf00019	20
AX-157721326	2	30.662	KV453182.1	2900404	20-scf00019	20
AX-157754548	2	30.662	KV453182.1	2902160	20-scf00019	20
AX-157911926	2	30.662	KV453182.1	2903789	20-scf00019	20
AX-157911927	2	30.668	KV453182.1	2904664	20-scf00019	20
AX-157911920	2	31.186	KV453182.1	2897176	20-scf00019	20
AX-157911938	2	31.186	KV453182.1	2919604	20-scf00019	20
AX-157911939	2	31.186	KV453182.1	2921009	20-scf00019	20
AX-157756004	2	31.186	KV453182.1	2921816	20-scf00019	20
AX-157911941	2	31.186	KV453182.1	2924441	20-scf00019	20
AX-157911944	2	31.186	KV453182.1	2935144	20-scf00019	20
AX-157911956	2	31.186	KV453182.1	2949919	20-scf00019	20
AX-157864481	2	31.186	KV453182.1	2955781	20-scf00019	20
AX-157911972	2	31.186	KV453182.1	2963010	20-scf00019	20
AX-157911943	2	31.224	KV453182.1	2932269	20-scf00019	20
AX-157911945	2	31.224	KV453182.1	2936758	20-scf00019	20
AX-157911946	2	31.224	KV453182.1	2937187	20-scf00019	20
AX-157911948	2	31.224	KV453182.1	2940450	20-scf00019	20
AX-157911949	2	31.224	KV453182.1	2941711	20-scf00019	20
AX-157911951	2	31.224	KV453182.1	2943149	20-scf00019	20
AX-157911953	2	31.224	KV453182.1	2945502	20-scf00019	20
AX-157911955	2	31.224	KV453182.1	2947548	20-scf00019	20
AX-157911957	2	31.224	KV453182.1	2950318	20-scf00019	20
AX-157911958	2	31.224	KV453182.1	2950904	20-scf00019	20
AX-157911959	2	31.224	KV453182.1	2951336	20-scf00019	20
AX-157911964	2	31.224	KV453182.1	2954115	20-scf00019	20
AX-157911975	2	31.224	KV453182.1	2967639	20-scf00019	20
AX-157911960	2	31.459	KV453182.1	2952360	20-scf00019	20
AX-157911963	2	31.618	KV453182.1	2953613	20-scf00019	20
AX-157911968	2	31.782	KV453182.1	2960264	20-scf00019	20
AX-157911969	2	31.782	KV453182.1	2960519	20-scf00019	20
AX-157911970	2	31.783	KV453182.1	2960967	20-scf00019	20
AX-157911973	2	31.783	KV453182.1	2964557	20-scf00019	20
AX-157812321	2	31.783	KV453182.1	2970385	20-scf00019	20
AX-157721782	2	32.05	KV453182.1	2965141	20-scf00019	20
AX-157911974	2	32.085	KV453182.1	2966566	20-scf00019	20
AX-157911976	2	32.085	KV453182.1	2968765	20-scf00019	20
AX-157911977	2	32.085	KV453182.1	2968961	20-scf00019	20
AX-157911978	2	32.085	KV453182.1	2969596	20-scf00019	20
AX-157911980	2	32.085	KV453182.1	2971289	20-scf00019	20
AX-157911982	2	32.085	KV453182.1	2974897	20-scf00019	20
AX-157812323	2	32.086	KV453182.1	2981336	20-scf00019	20

AX-157911986	2	32.086	KV453182.1	2983582	20-scf00019	20
AX-157911987	2	32.413	KV453182.1	2984562	20-scf00019	20
AX-157911991	2	32.413	KV453182.1	2988980	20-scf00019	20
AX-158404922	2	32.413	KV453182.1	2993140	20-scf00019	20
AX-158404923	2	32.413	KV453182.1	2993729	20-scf00019	20
AX-157911995	2	32.413	KV453182.1	2997583	20-scf00019	20
AX-157911997	2	32.413	KV453182.1	2999698	20-scf00019	20
AX-157912000	2	32.413	KV453182.1	3001740	20-scf00019	20
AX-157912001	2	32.413	KV453182.1	3002305	20-scf00019	20
AX-157748481	2	32.414	KV453182.1	3003168	20-scf00019	20
AX-157912002	2	32.414	KV453182.1	3003680	20-scf00019	20
AX-157912006	2	32.741	KV453182.1	3014904	20-scf00019	20
AX-157912007	2	32.741	KV453182.1	3015224	20-scf00019	20
AX-157714131	2	32.741	KV453182.1	3016589	20-scf00019	20
AX-157750995	2	32.741	KV453182.1	3017258	20-scf00019	20
AX-157745253	2	32.741	KV453182.1	3017963	20-scf00019	20
AX-157912008	2	32.741	KV453182.1	3018501	20-scf00019	20
AX-157812326	2	32.959	KV453182.1	3028033	20-scf00019	20
AX-157912016	2	32.959	KV453182.1	3031732	20-scf00019	20
AX-157912018	2	32.974	KV453182.1	3035499	20-scf00019	20
AX-157912020	2	33.147	KV453182.1	3038327	20-scf00019	20
AX-157869083	2	33.198	KV453182.1	3041379	20-scf00019	20
AX-157912023	2	33.34	KV453182.1	3043067	20-scf00019	20
AX-157912029	2	33.725	KV453182.1	3055495	20-scf00019	20
AX-157912034	2	33.725	KV453182.1	3060304	20-scf00019	20
AX-157750532	2	33.725	KV453182.1	3060775	20-scf00019	20
AX-157912036	2	33.725	KV453182.1	3061838	20-scf00019	20
AX-157912037	2	33.725	KV453182.1	3062484	20-scf00019	20
AX-158379239	2	33.725	KV453182.1	3065179	20-scf00019	20
AX-157912040	2	33.749	KV453182.1	3066456	20-scf00019	20
AX-158389573	2	33.749	KV453182.1	3066497	20-scf00019	20
AX-157722172	2	33.794	KV453182.1	3067656	20-scf00019	20
AX-157911988	2	33.834	KV453182.1	2986354	20-scf00019	20
AX-157912045	2	33.834	KV453182.1	3073017	20-scf00019	20
AX-157912048	2	33.872	KV453182.1	3075479	20-scf00019	20
AX-157912049	2	33.906	KV453182.1	3077434	20-scf00019	20
AX-157720553	2	33.944	KV453182.1	3051281	20-scf00019	20
AX-157912028	2	33.944	KV453182.1	3055023	20-scf00019	20
AX-157912032	2	33.944	KV453182.1	3058353	20-scf00019	20
AX-157912042	2	33.944	KV453182.1	3068577	20-scf00019	20
AX-157912051	2	33.944	KV453182.1	3079011	20-scf00019	20
AX-157912052	2	33.944	KV453182.1	3080397	20-scf00019	20
AX-157912053	2	33.944	KV453182.1	3080847	20-scf00019	20
AX-157912055	2	33.944	KV453182.1	3083679	20-scf00019	20
AX-157912056	2	33.944	KV453182.1	3084363	20-scf00019	20
AX-157912058	2	33.944	KV453182.1	3086729	20-scf00019	20
AX-157912061	2	33.944	KV453182.1	3089136	20-scf00019	20
AX-157912062	2	33.944	KV453182.1	3089801	20-scf00019	20
AX-157912063	2	33.944	KV453182.1	3090620	20-scf00019	20
AX-157874627	2	33.944	KV453182.1	3096509	20-scf00019	20
AX-157912079	2	33.944	KV453182.1	3113135	20-scf00019	20

AX-157912066	2	33.946	KV453182.1	3096586	20-scf00019	20
AX-157912074	2	34.271	KV453182.1	3107188	20-scf00019	20
AX-157912069	2	34.6	KV453182.1	3098036	20-scf00019	20
AX-157912070	2	34.6	KV453182.1	3099634	20-scf00019	20
AX-157912071	2	34.6	KV453182.1	3100961	20-scf00019	20
AX-157912076	2	34.6	KV453182.1	3109988	20-scf00019	20
AX-157912078	2	34.6	KV453182.1	3112126	20-scf00019	20
AX-157912083	2	34.6	KV453182.1	3119830	20-scf00019	20
AX-157752086	2	34.6	KV453182.1	3121156	20-scf00019	20
AX-157721443	2	34.6	KV453182.1	3129052	20-scf00019	20
AX-157912088	2	34.6	KV453182.1	3132895	20-scf00019	20
AX-157739642	2	34.6	KV453182.1	3134533	20-scf00019	20
AX-157912089	2	34.6	KV453182.1	3136121	20-scf00019	20
AX-157912092	2	34.6	KV453182.1	3137692	20-scf00019	20
AX-157912094	2	34.6	KV453182.1	3140587	20-scf00019	20
AX-157912095	2	34.6	KV453182.1	3141014	20-scf00019	20
AX-157733184	2	34.6	KV453182.1	3143108	20-scf00019	20
AX-157693393	2	34.6	FI859113.1	541	ORI212 Ictalu	UN
AX-157707301	2	34.6	3ML01033955	237	scf09624_1	UN
AX-157721677	2	34.711	KV453182.1	3094390	20-scf00019	20
AX-157746025	2	34.711	KV453182.1	3149476	20-scf00019	20
AX-157812329	2	34.819	KV453182.1	3122422	20-scf00019	20
AX-157812334	2	34.819	KV453182.1	3137945	20-scf00019	20
AX-157912099	2	34.819	KV453182.1	3149811	20-scf00019	20
AX-157912100	2	34.819	KV453182.1	3150260	20-scf00019	20
AX-157912102	2	34.819	KV453182.1	3154228	20-scf00019	20
AX-157912104	2	34.819	KV453182.1	3155043	20-scf00019	20
AX-157912105	2	34.819	KV453182.1	3155574	20-scf00019	20
AX-157912107	2	34.819	KV453182.1	3159165	20-scf00019	20
AX-157912108	2	34.819	KV453182.1	3162714	20-scf00019	20
AX-157912111	2	34.819	KV453182.1	3168147	20-scf00019	20
AX-157706828	2	34.819	3ML01032992	520	scf08626_1	UN
AX-157912112	2	34.998	KV453182.1	3171268	20-scf00019	20
AX-157912114	2	35.146	KV453182.1	3172329	20-scf00019	20
AX-157912115	2	35.146	KV453182.1	3174879	20-scf00019	20
AX-157693912	2	35.147	KV453182.1	3171757	20-scf00019	20
AX-157701843	2	35.147	KV453182.1	3174164	20-scf00019	20
AX-157695724	2	35.147	KV453182.1	3177980	20-scf00019	20
AX-157912119	2	35.147	KV453182.1	3179756	20-scf00019	20
AX-157719461	2	35.147	KV453182.1	3182118	20-scf00019	20
AX-157812339	2	35.147	KV453182.1	3182754	20-scf00019	20
AX-157912123	2	35.147	KV453182.1	3185794	20-scf00019	20
AX-157912125	2	35.147	KV453182.1	3189470	20-scf00019	20
AX-157912129	2	35.147	KV453182.1	3195004	20-scf00019	20
AX-157912130	2	35.147	KV453182.1	3199104	20-scf00019	20
AX-158379240	2	35.169	KV453182.1	3203263	20-scf00019	20
AX-157912134	2	35.253	KV453182.1	3205615	20-scf00019	20
AX-157912135	2	35.338	KV453182.1	3207129	20-scf00019	20
AX-157912136	2	35.366	KV453182.1	3208130	20-scf00019	20
AX-157912139	2	35.474	KV453182.1	3212029	20-scf00019	20
AX-157912141	2	35.531	KV453182.1	3213672	20-scf00019	20

AX-157912142	2	35.583	KV453182.1	3215211	20-scf00019	20
AX-157773823	2	35.583	KV453182.1	3223581	20-scf00019	20
AX-157912117	2	35.635	KV453182.1	3177103	20-scf00019	20
AX-157912128	2	35.635	KV453182.1	3194392	20-scf00019	20
AX-157912147	2	35.635	KV453182.1	3226627	20-scf00019	20
AX-157912149	2	35.635	KV453182.1	3227683	20-scf00019	20
AX-157912152	2	35.635	KV453182.1	3231787	20-scf00019	20
AX-157912148	2	35.693	KV453182.1	3227096	20-scf00019	20
AX-157912150	2	35.693	KV453182.1	3230347	20-scf00019	20
AX-157874632	2	35.693	KV453182.1	3230925	20-scf00019	20
AX-157912154	2	35.693	KV453182.1	3234400	20-scf00019	20
AX-157912155	2	35.927	KV453182.1	3235622	20-scf00019	20
AX-157912156	2	36.02	KV453182.1	3236746	20-scf00019	20
AX-157912144	2	36.021	KV453182.1	3219296	20-scf00019	20
AX-157883423	2	36.021	KV453182.1	3222981	20-scf00019	20
AX-157912158	2	36.021	KV453182.1	3237604	20-scf00019	20
AX-157912159	2	36.021	KV453182.1	3239088	20-scf00019	20
AX-157912161	2	36.021	KV453182.1	3246683	20-scf00019	20
AX-157912165	2	36.021	KV453182.1	3250196	20-scf00019	20
AX-157912167	2	36.021	KV453182.1	3252010	20-scf00019	20
AX-157912168	2	36.021	KV453182.1	3252156	20-scf00019	20
AX-157912172	2	36.021	KV453182.1	3255773	20-scf00019	20
AX-157912173	2	36.021	KV453182.1	3256258	20-scf00019	20
AX-157912174	2	36.021	KV453182.1	3260327	20-scf00019	20
AX-157912178	2	36.021	KV453182.1	3263786	20-scf00019	20
AX-157912170	2	36.22	KV453182.1	3254122	20-scf00019	20
AX-157912175	2	36.22	KV453182.1	3261475	20-scf00019	20
AX-157912176	2	36.22	KV453182.1	3262225	20-scf00019	20
AX-157912184	2	36.22	KV453182.1	3270103	20-scf00019	20
AX-157869086	2	36.22	KV453182.1	3296801	20-scf00019	20
AX-157912234	2	36.22	KV453182.1	3344277	20-scf00019	20
AX-157912235	2	36.22	KV453182.1	3344983	20-scf00019	20
AX-157912243	2	36.22	KV453182.1	3353120	20-scf00019	20
AX-157864487	2	36.22	KV453182.1	3360599	20-scf00019	20
AX-157912253	2	36.22	KV453182.1	3363590	20-scf00019	20
AX-157912260	2	36.22	KV453182.1	3377449	20-scf00019	20
AX-157912157	2	36.459	KV453182.1	3237177	20-scf00019	20
AX-157912180	2	36.459	KV453182.1	3265535	20-scf00019	20
AX-157912181	2	36.459	KV453182.1	3267660	20-scf00019	20
AX-157775398	2	36.459	KV453182.1	3269804	20-scf00019	20
AX-157912188	2	36.459	KV453182.1	3273172	20-scf00019	20
AX-157912189	2	36.459	KV453182.1	3274098	20-scf00019	20
AX-157912192	2	36.459	KV453182.1	3276246	20-scf00019	20
AX-157912193	2	36.459	KV453182.1	3276705	20-scf00019	20
AX-157912194	2	36.459	KV453182.1	3276958	20-scf00019	20
AX-157912199	2	36.459	KV453182.1	3282520	20-scf00019	20
AX-157912201	2	36.495	KV453182.1	3285928	20-scf00019	20
AX-157912202	2	36.533	KV453182.1	3287069	20-scf00019	20
AX-157912203	2	36.568	KV453182.1	3288820	20-scf00019	20
AX-157912204	2	36.568	KV453182.1	3290995	20-scf00019	20
AX-157758771	2	36.568	KV453182.1	3291205	20-scf00019	20

AX-157912220	2	36.568	KV453182.1	3326229	20-scf00019	20
AX-157812349	2	36.568	KV453182.1	3326273	20-scf00019	20
AX-157912221	2	36.568	KV453182.1	3328836	20-scf00019	20
AX-157747006	2	36.568	KV453182.1	3330972	20-scf00019	20
AX-158379241	2	36.568	KV453182.1	3333078	20-scf00019	20
AX-157883425	2	36.568	KV453182.1	3333874	20-scf00019	20
AX-157748596	2	36.677	KV453182.1	3335465	20-scf00019	20
AX-157912229	2	36.677	KV453182.1	3338401	20-scf00019	20
AX-157912230	2	36.677	KV453182.1	3338759	20-scf00019	20
AX-158421868	2	36.677	KV453182.1	3338959	20-scf00019	20
AX-157912233	2	36.677	KV453182.1	3343917	20-scf00019	20
AX-157912231	2	36.799	KV453182.1	3342441	20-scf00019	20
AX-157912236	2	36.841	KV453182.1	3345879	20-scf00019	20
AX-157912241	2	36.841	KV453182.1	3351650	20-scf00019	20
AX-157912259	2	36.841	KV453182.1	3376555	20-scf00019	20
AX-157912222	2	37.005	KV453182.1	3329530	20-scf00019	20
AX-157912245	2	37.005	KV453182.1	3356891	20-scf00019	20
AX-157912246	2	37.005	KV453182.1	3357355	20-scf00019	20
AX-157912247	2	37.005	KV453182.1	3358318	20-scf00019	20
AX-157912250	2	37.005	KV453182.1	3360818	20-scf00019	20
AX-157912251	2	37.005	KV453182.1	3361188	20-scf00019	20
AX-157912252	2	37.005	KV453182.1	3362808	20-scf00019	20
AX-157912264	2	37.005	KV453182.1	3381387	20-scf00019	20
AX-157912267	2	37.005	KV453182.1	3383920	20-scf00019	20
AX-157755368	2	37.005	KV453182.1	3395554	20-scf00019	20
AX-157774565	2	37.005	KV453182.1	3403469	20-scf00019	20
AX-157812357	2	37.005	KV453182.1	3420236	20-scf00019	20
AX-157912272	2	37.114	KV453182.1	3389549	20-scf00019	20
AX-157812351	2	37.169	KV453182.1	3396861	20-scf00019	20
AX-157748763	2	37.169	KV453182.1	3397305	20-scf00019	20
AX-157812352	2	37.169	KV453182.1	3397449	20-scf00019	20
AX-157912279	2	37.169	KV453182.1	3409221	20-scf00019	20
AX-157912282	2	37.169	KV453182.1	3410351	20-scf00019	20
AX-157722280	2	37.169	KV453182.1	3411288	20-scf00019	20
AX-157912283	2	37.169	KV453182.1	3411896	20-scf00019	20
AX-157812355	2	37.169	KV453182.1	3412310	20-scf00019	20
AX-157812356	2	37.333	KV453182.1	3413744	20-scf00019	20
AX-157912285	2	37.442	KV453182.1	3416994	20-scf00019	20
AX-157912287	2	37.442	KV453182.1	3418599	20-scf00019	20
AX-157912288	2	37.466	KV453182.1	3420017	20-scf00019	20
AX-157912291	2	37.466	KV453182.1	3423604	20-scf00019	20
AX-157912294	2	37.506	KV453182.1	3434560	20-scf00019	20
AX-157912297	2	37.551	KV453182.1	3438731	20-scf00019	20
AX-157812359	2	37.552	KV453182.1	3442675	20-scf00019	20
AX-157912301	2	37.552	KV453182.1	3444328	20-scf00019	20
AX-157912303	2	37.552	KV453182.1	3446726	20-scf00019	20
AX-157743823	2	37.552	KV453182.1	3447997	20-scf00019	20
AX-157812361	2	37.552	KV453182.1	3450380	20-scf00019	20
AX-157912306	2	37.552	KV453182.1	3451078	20-scf00019	20
AX-157812362	2	37.552	KV453182.1	3451428	20-scf00019	20
AX-157912308	2	37.552	KV453182.1	3456256	20-scf00019	20

AX-157912307	2	37.553	KV453182.1	3454331	20-scf00019	20
AX-157750698	2	37.553	KV453182.1	3454634	20-scf00019	20
AX-158389575	2	37.715	KV453182.1	3436272	20-scf00019	20
AX-157812364	2	37.715	KV453182.1	3458173	20-scf00019	20
AX-157912309	2	37.715	KV453182.1	3459043	20-scf00019	20
AX-157912310	2	37.715	KV453182.1	3461433	20-scf00019	20
AX-157912313	2	37.715	KV453182.1	3464833	20-scf00019	20
AX-157812365	2	37.715	KV453182.1	3465049	20-scf00019	20
AX-157747664	2	37.879	KV453182.1	3495290	20-scf00019	20
AX-157912326	2	37.879	KV453182.1	3497887	20-scf00019	20
AX-157912328	2	37.903	KV453182.1	3499921	20-scf00019	20
AX-157912331	2	37.93	KV453182.1	3504430	20-scf00019	20
AX-157912299	2	37.961	KV453182.1	3441847	20-scf00019	20
AX-157912332	2	37.961	KV453182.1	3505421	20-scf00019	20
AX-157912334	2	37.988	KV453182.1	3508209	20-scf00019	20
AX-157812368	2	37.988	KV453182.1	3509424	20-scf00019	20
AX-157912327	2	38.18	KV453182.1	3498754	20-scf00019	20
AX-157912336	2	38.18	KV453182.1	3511444	20-scf00019	20
AX-157912339	2	38.18	KV453182.1	3515139	20-scf00019	20
AX-157912340	2	38.18	KV453182.1	3516470	20-scf00019	20
AX-157912341	2	38.18	KV453182.1	3517554	20-scf00019	20
AX-157912344	2	38.18	KV453182.1	3519959	20-scf00019	20
AX-157714236	2	38.234	KV453182.1	3363436	20-scf00019	20
AX-157912323	2	38.234	KV453182.1	3490499	20-scf00019	20
AX-157912347	2	38.234	KV453182.1	3523489	20-scf00019	20
AX-157764025	2	38.234	KV453182.1	3523875	20-scf00019	20
AX-157812372	2	38.234	KV453182.1	3529954	20-scf00019	20
AX-157812373	2	38.234	KV453182.1	3531642	20-scf00019	20
AX-157812374	2	38.234	KV453182.1	3538425	20-scf00019	20
AX-157912355	2	38.234	KV453182.1	3543955	20-scf00019	20
AX-157912362	2	38.234	KV453182.1	3554426	20-scf00019	20
AX-157912370	2	38.234	KV453182.1	3563952	20-scf00019	20
AX-157912371	2	38.234	KV453182.1	3564633	20-scf00019	20
AX-157912385	2	38.234	KV453182.1	3583789	20-scf00019	20
AX-157912388	2	38.234	KV453182.1	3588603	20-scf00019	20
AX-157912337	2	38.425	KV453182.1	3512531	20-scf00019	20
AX-157912354	2	38.425	KV453182.1	3541677	20-scf00019	20
AX-157756336	2	38.425	KV453182.1	3542445	20-scf00019	20
AX-157912358	2	38.425	KV453182.1	3547344	20-scf00019	20
AX-157812376	2	38.425	KV453182.1	3550343	20-scf00019	20
AX-157912372	2	38.425	KV453182.1	3566098	20-scf00019	20
AX-157912373	2	38.425	KV453182.1	3567127	20-scf00019	20
AX-157912375	2	38.425	KV453182.1	3569968	20-scf00019	20
AX-157912376	2	38.425	KV453182.1	3571468	20-scf00019	20
AX-157912377	2	38.425	KV453182.1	3573630	20-scf00019	20
AX-157912391	2	38.425	KV453182.1	3595704	20-scf00019	20
AX-157912402	2	38.425	KV453182.1	3610715	20-scf00019	20
AX-157912381	2	38.534	KV453182.1	3578527	20-scf00019	20
AX-157774792	2	38.534	KV453182.1	3593890	20-scf00019	20
AX-157912390	2	38.534	KV453182.1	3594277	20-scf00019	20
AX-157912360	2	38.611	KV453182.1	3549879	20-scf00019	20

AX-157912361	2	38.611	KV453182.1	3551319	20-scf00019	20
AX-157912398	2	38.611	KV453182.1	3607189	20-scf00019	20
AX-157912379	2	38.644	KV453182.1	3576538	20-scf00019	20
AX-157912401	2	38.644	KV453182.1	3610308	20-scf00019	20
AX-158404935	2	38.644	KV453182.1	3614331	20-scf00019	20
AX-157912405	2	38.644	KV453182.1	3615151	20-scf00019	20
AX-157912407	2	38.644	KV453182.1	3617747	20-scf00019	20
AX-157912406	2	38.686	KV453182.1	3616133	20-scf00019	20
AX-157693955	2	38.753	KV453182.1	3620984	20-scf00019	20
AX-158404938	2	38.761	KV453182.1	3624370	20-scf00019	20
AX-157912412	2	38.778	KV453182.1	3625598	20-scf00019	20
AX-157912413	2	38.778	KV453182.1	3626195	20-scf00019	20
AX-157912414	2	38.778	KV453182.1	3628144	20-scf00019	20
AX-157770879	2	38.799	KV453182.1	3634487	20-scf00019	20
AX-157912420	2	38.828	KV453182.1	3635760	20-scf00019	20
AX-157912425	2	38.828	KV453182.1	3641366	20-scf00019	20
AX-157912429	2	38.828	KV453182.1	3645269	20-scf00019	20
AX-157912430	2	38.828	KV453182.1	3648316	20-scf00019	20
AX-157912421	2	38.862	KV453182.1	3636486	20-scf00019	20
AX-157812379	2	38.862	KV453182.1	3638090	20-scf00019	20
AX-157912423	2	38.862	KV453182.1	3638665	20-scf00019	20
AX-157912460	2	38.862	KV453182.1	3681596	20-scf00019	20
AX-157912403	2	39.026	KV453182.1	3612063	20-scf00019	20
AX-157912417	2	39.026	KV453182.1	3630335	20-scf00019	20
AX-157912424	2	39.026	KV453182.1	3640493	20-scf00019	20
AX-157912427	2	39.026	KV453182.1	3643579	20-scf00019	20
AX-157912433	2	39.026	KV453182.1	3654622	20-scf00019	20
AX-157912434	2	39.026	KV453182.1	3656993	20-scf00019	20
AX-157912435	2	39.026	KV453182.1	3657463	20-scf00019	20
AX-157912451	2	39.026	KV453182.1	3672826	20-scf00019	20
AX-157912459	2	39.026	KV453182.1	3680059	20-scf00019	20
AX-157912509	2	39.026	KV453182.1	3735063	20-scf00019	20
AX-157912580	2	39.026	KV453182.1	3858320	20-scf00019	20
AX-157752843	2	39.189	KV453182.1	3650007	20-scf00019	20
AX-157708810	2	39.189	KV453182.1	3659984	20-scf00019	20
AX-157912441	2	39.19	KV453182.1	3663498	20-scf00019	20
AX-157912443	2	39.19	KV453182.1	3665533	20-scf00019	20
AX-157722276	2	39.19	KV453182.1	3670909	20-scf00019	20
AX-157912449	2	39.19	KV453182.1	3671613	20-scf00019	20
AX-157912450	2	39.19	KV453182.1	3672269	20-scf00019	20
AX-157912453	2	39.19	KV453182.1	3674278	20-scf00019	20
AX-157912454	2	39.19	KV453182.1	3675171	20-scf00019	20
AX-157912456	2	39.19	KV453182.1	3676611	20-scf00019	20
AX-157812382	2	39.19	KV453182.1	3687224	20-scf00019	20
AX-157727955	2	39.19	KV453182.1	3687605	20-scf00019	20
AX-157912465	2	39.19	KV453182.1	3690821	20-scf00019	20
AX-157912470	2	39.19	KV453182.1	3694823	20-scf00019	20
AX-157912481	2	39.19	KV453182.1	3706257	20-scf00019	20
AX-157912489	2	39.19	KV453182.1	3715618	20-scf00019	20
AX-157912490	2	39.19	KV453182.1	3716403	20-scf00019	20
AX-157912491	2	39.19	KV453182.1	3716749	20-scf00019	20

AX-157912492	2	39.19	KV453182.1	3717438	20-scf00019	20
AX-157912494	2	39.19	KV453182.1	3718791	20-scf00019	20
AX-157912495	2	39.19	KV453182.1	3719780	20-scf00019	20
AX-157812386	2	39.19	KV453182.1	3719910	20-scf00019	20
AX-157812388	2	39.19	KV453182.1	3724459	20-scf00019	20
AX-157912498	2	39.19	KV453182.1	3725429	20-scf00019	20
AX-157812390	2	39.19	KV453182.1	3726428	20-scf00019	20
AX-157912499	2	39.19	KV453182.1	3726682	20-scf00019	20
AX-157912500	2	39.19	KV453182.1	3727241	20-scf00019	20
AX-157912503	2	39.19	KV453182.1	3729483	20-scf00019	20
AX-157912505	2	39.19	KV453182.1	3731363	20-scf00019	20
AX-157912508	2	39.19	KV453182.1	3734145	20-scf00019	20
AX-157812392	2	39.19	KV453182.1	3739361	20-scf00019	20
AX-157775033	2	39.19	KV453182.1	3753067	20-scf00019	20
AX-157912515	2	39.408	KV453182.1	3750031	20-scf00019	20
AX-157912517	2	39.408	KV453182.1	3752306	20-scf00019	20
AX-157812396	2	39.408	KV453182.1	3753603	20-scf00019	20
AX-157912520	2	39.408	KV453182.1	3757045	20-scf00019	20
AX-157912468	2	39.554	KV453182.1	3692793	20-scf00019	20
AX-157912474	2	39.554	KV453182.1	3698247	20-scf00019	20
AX-157912525	2	39.554	KV453182.1	3762510	20-scf00019	20
AX-157771923	2	39.554	KV453182.1	3762604	20-scf00019	20
AX-157912527	2	39.554	KV453182.1	3763835	20-scf00019	20
AX-157912531	2	39.7	KV453182.1	3771880	20-scf00019	20
AX-157912532	2	39.791	KV453182.1	3775387	20-scf00019	20
AX-157912522	2	39.846	KV453182.1	3759188	20-scf00019	20
AX-157912529	2	39.846	KV453182.1	3770026	20-scf00019	20
AX-157912535	2	39.846	KV453182.1	3778440	20-scf00019	20
AX-157912537	2	39.846	KV453182.1	3780821	20-scf00019	20
AX-157912541	2	39.846	KV453182.1	3788251	20-scf00019	20
AX-157912543	2	39.846	KV453182.1	3790454	20-scf00019	20
AX-157883430	2	39.846	KV453182.1	3792674	20-scf00019	20
AX-157912545	2	39.846	KV453182.1	3794517	20-scf00019	20
AX-157743226	2	39.846	KV453182.1	3797283	20-scf00019	20
AX-157912547	2	39.846	KV453182.1	3798641	20-scf00019	20
AX-157912551	2	39.846	KV453182.1	3804182	20-scf00019	20
AX-157912553	2	39.846	KV453182.1	3807923	20-scf00019	20
AX-157812406	2	39.846	KV453182.1	3833199	20-scf00019	20
AX-157912594	2	39.846	KV453182.1	3888442	20-scf00019	20
AX-157707495	2	39.848	3ML01034540	172	scf10217_1	UN
AX-157753839	2	39.85	KV453182.1	3816725	20-scf00019	20
AX-157715998	2	39.85	KV453182.1	3880412	20-scf00019	20
AX-157912616	2	39.85	KV453182.1	3938583	20-scf00019	20
AX-157771763	2	39.99	KV453182.1	3783985	20-scf00019	20
AX-157912558	2	39.99	KV453182.1	3817841	20-scf00019	20
AX-157912559	2	39.99	KV453182.1	3818686	20-scf00019	20
AX-157912562	2	39.99	KV453182.1	3825602	20-scf00019	20
AX-157812403	2	39.99	KV453182.1	3826557	20-scf00019	20
AX-157812405	2	39.99	KV453182.1	3830776	20-scf00019	20
AX-157912566	2	39.99	KV453182.1	3831740	20-scf00019	20
AX-157912568	2	39.99	KV453182.1	3834237	20-scf00019	20

AX-157912569	2	39.99	KV453182.1	3835178	20-scf00019	20
AX-157912570	2	39.99	KV453182.1	3843068	20-scf00019	20
AX-157912575	2	39.99	KV453182.1	3848887	20-scf00019	20
AX-157912602	2	39.99	KV453182.1	3900291	20-scf00019	20
AX-157717274	2	39.99	KV453182.1	3986101	20-scf00019	20
AX-157912579	2	40.082	KV453182.1	3854212	20-scf00019	20
AX-157715124	2	40.283	KV453182.1	3745555	20-scf00019	20
AX-157741699	2	40.283	KV453182.1	3793733	20-scf00019	20
AX-157774530	2	40.283	KV453182.1	3838988	20-scf00019	20
AX-157912582	2	40.283	KV453182.1	3863027	20-scf00019	20
AX-157912584	2	40.283	KV453182.1	3867309	20-scf00019	20
AX-157912586	2	40.283	KV453182.1	3870700	20-scf00019	20
AX-157912588	2	40.283	KV453182.1	3876774	20-scf00019	20
AX-157853692	2	40.283	KV453182.1	3877405	20-scf00019	20
AX-157912591	2	40.283	KV453182.1	3879796	20-scf00019	20
AX-157744770	2	40.283	KV453182.1	3882512	20-scf00019	20
AX-157912593	2	40.283	KV453182.1	3883408	20-scf00019	20
AX-157912595	2	40.283	KV453182.1	3888949	20-scf00019	20
AX-157912596	2	40.283	KV453182.1	3890045	20-scf00019	20
AX-157912597	2	40.283	KV453182.1	3891222	20-scf00019	20
AX-157912599	2	40.283	KV453182.1	3893955	20-scf00019	20
AX-157912605	2	40.283	KV453182.1	3905916	20-scf00019	20
AX-157753694	2	40.283	KV453182.1	3938840	20-scf00019	20
AX-157912618	2	40.283	KV453182.1	3941908	20-scf00019	20
AX-157718197	2	40.567	KV453182.1	3805682	20-scf00019	20
AX-157912613	2	40.567	KV453182.1	3932996	20-scf00019	20
AX-157912615	2	40.568	KV453182.1	3938379	20-scf00019	20
AX-157912621	2	40.568	KV453182.1	3947178	20-scf00019	20
AX-157912623	2	40.711	KV453182.1	3949103	20-scf00019	20
AX-157912629	2	40.779	KV453182.1	3956586	20-scf00019	20
AX-157912512	2	40.854	KV453182.1	3743466	20-scf00019	20
AX-157812401	2	40.854	KV453182.1	3790089	20-scf00019	20
AX-157912626	2	40.854	KV453182.1	3952199	20-scf00019	20
AX-157912628	2	40.854	KV453182.1	3954411	20-scf00019	20
AX-157912633	2	40.854	KV453182.1	3960444	20-scf00019	20
AX-157912634	2	40.854	KV453182.1	3961532	20-scf00019	20
AX-157912635	2	40.854	KV453182.1	3963631	20-scf00019	20
AX-157912636	2	40.854	KV453182.1	3966062	20-scf00019	20
AX-157812413	2	40.854	KV453182.1	3971147	20-scf00019	20
AX-157912645	2	40.854	KV453182.1	3990544	20-scf00019	20
AX-157715218	2	40.854	KV453182.1	3992797	20-scf00019	20
AX-157812414	2	40.997	KV453182.1	3976134	20-scf00019	20
AX-157912638	2	40.997	KV453182.1	3982288	20-scf00019	20
AX-157912639	2	41.281	KV453182.1	3983173	20-scf00019	20
AX-157775492	2	41.282	KV453182.1	3985137	20-scf00019	20
AX-157812416	2	41.282	KV453182.1	3985616	20-scf00019	20
AX-157912646	2	41.569	KV453182.1	3992300	20-scf00019	20
AX-157912649	2	41.57	KV453182.1	3996734	20-scf00019	20
AX-157912651	2	41.57	KV453182.1	4000291	20-scf00019	20
AX-157912696	2	41.57	KV453182.1	4064010	20-scf00019	20
AX-157912655	2	41.711	KV453182.1	4003852	20-scf00019	20

AX-157912657	2	41.711	KV453182.1	4005815	20-scf00019	20
AX-157912664	2	41.711	KV453182.1	4016271	20-scf00019	20
AX-157912668	2	41.711	KV453182.1	4026379	20-scf00019	20
AX-157912667	2	41.72	KV453182.1	4018997	20-scf00019	20
AX-157853698	2	41.72	KV453182.1	4252967	20-scf00019	20
AX-157912842	2	41.72	KV453182.1	4265189	20-scf00019	20
AX-157912669	2	41.753	KV453182.1	4026864	20-scf00019	20
AX-157716542	2	41.77	KV453182.1	4030192	20-scf00019	20
AX-158389577	2	41.801	KV453182.1	4030693	20-scf00019	20
AX-157812426	2	41.801	KV453182.1	4031247	20-scf00019	20
AX-157912672	2	41.804	KV453182.1	4035987	20-scf00019	20
AX-157912659	2	41.813	KV453182.1	4008412	20-scf00019	20
AX-157912676	2	41.813	KV453182.1	4038782	20-scf00019	20
AX-157812423	2	41.815	KV453182.1	4026303	20-scf00019	20
AX-157775251	2	41.815	KV453182.1	4030905	20-scf00019	20
AX-157912681	2	41.815	KV453182.1	4046463	20-scf00019	20
AX-157912702	2	41.815	KV453182.1	4073968	20-scf00019	20
AX-157912726	2	41.815	KV453182.1	4110797	20-scf00019	20
AX-157912686	2	41.826	KV453182.1	4052678	20-scf00019	20
AX-157912688	2	41.84	KV453182.1	4056759	20-scf00019	20
AX-157912523	2	41.855	KV453182.1	3761272	20-scf00019	20
AX-157912692	2	41.855	KV453182.1	4061414	20-scf00019	20
AX-157912695	2	41.855	KV453182.1	4062921	20-scf00019	20
AX-157912704	2	41.896	KV453182.1	4077072	20-scf00019	20
AX-158404949	2	41.937	KV453182.1	4078855	20-scf00019	20
AX-157812429	2	41.997	KV453182.1	4094179	20-scf00019	20
AX-157749170	2	41.997	KV453182.1	4095840	20-scf00019	20
AX-157912716	2	41.997	KV453182.1	4097456	20-scf00019	20
AX-157912717	2	41.997	KV453182.1	4098308	20-scf00019	20
AX-157912719	2	41.997	KV453182.1	4101416	20-scf00019	20
AX-157912722	2	41.997	KV453182.1	4106096	20-scf00019	20
AX-157912724	2	41.997	KV453182.1	4108838	20-scf00019	20
AX-157912727	2	41.997	KV453182.1	4112652	20-scf00019	20
AX-157912733	2	41.997	KV453182.1	4118931	20-scf00019	20
AX-157774531	2	41.997	KV453182.1	4659380	20-scf00019	20
AX-157727719	2	42.14	KV453182.1	4120668	20-scf00019	20
AX-157912735	2	42.161	KV453182.1	4122153	20-scf00019	20
AX-157912757	2	42.161	KV453182.1	4147482	20-scf00019	20
AX-157762272	2	42.189	KV453182.1	4123957	20-scf00019	20
AX-157912737	2	42.218	KV453182.1	4124859	20-scf00019	20
AX-157912749	2	42.251	KV453182.1	4138794	20-scf00019	20
AX-157707721	2	42.283	KV453182.1	4123382	20-scf00019	20
AX-157912754	2	42.283	KV453182.1	4146165	20-scf00019	20
AX-157912756	2	42.283	KV453182.1	4146913	20-scf00019	20
AX-157912758	2	42.283	KV453182.1	4149323	20-scf00019	20
AX-157912759	2	42.283	KV453182.1	4150341	20-scf00019	20
AX-157912760	2	42.283	KV453182.1	4151648	20-scf00019	20
AX-157912765	2	42.283	KV453182.1	4155455	20-scf00019	20
AX-157912768	2	42.283	KV453182.1	4158985	20-scf00019	20
AX-157724652	2	42.283	KV453182.1	4173785	20-scf00019	20
AX-157912778	2	42.283	KV453182.1	4173905	20-scf00019	20

AX-157912780	2	42.283	KV453182.1	4177998	20-scf00019	20
AX-157912781	2	42.283	KV453182.1	4179746	20-scf00019	20
AX-157912832	2	42.283	KV453182.1	4248596	20-scf00019	20
AX-157912773	2	42.569	KV453182.1	4170113	20-scf00019	20
AX-157912774	2	42.625	KV453182.1	4170868	20-scf00019	20
AX-157812436	2	42.678	KV453182.1	4182647	20-scf00019	20
AX-157853697	2	42.678	KV453182.1	4237065	20-scf00019	20
AX-158404952	2	42.712	KV453182.1	4081327	20-scf00019	20
AX-157812437	2	42.712	KV453182.1	4185654	20-scf00019	20
AX-157912791	2	42.712	KV453182.1	4194418	20-scf00019	20
AX-157883435	2	42.712	KV453182.1	4491617	20-scf00019	20
AX-157913076	2	42.712	KV453182.1	4600944	20-scf00019	20
AX-157913100	2	42.712	KV453182.1	4636157	20-scf00019	20
AX-157912786	2	42.725	KV453182.1	4186680	20-scf00019	20
AX-157869090	2	42.736	KV453182.1	4177134	20-scf00019	20
AX-157912793	2	42.736	KV453182.1	4197404	20-scf00019	20
AX-157912794	2	42.775	KV453182.1	4202560	20-scf00019	20
AX-157912799	2	42.814	KV453182.1	4208587	20-scf00019	20
AX-157912776	2	42.855	KV453182.1	4172487	20-scf00019	20
AX-157812438	2	42.855	KV453182.1	4190314	20-scf00019	20
AX-157912802	2	42.855	KV453182.1	4214910	20-scf00019	20
AX-157912805	2	42.855	KV453182.1	4217239	20-scf00019	20
AX-157912807	2	42.855	KV453182.1	4219366	20-scf00019	20
AX-157912810	2	42.855	KV453182.1	4221349	20-scf00019	20
AX-157912811	2	42.855	KV453182.1	4221986	20-scf00019	20
AX-157912814	2	42.855	KV453182.1	4225337	20-scf00019	20
AX-157912815	2	42.855	KV453182.1	4226814	20-scf00019	20
AX-157912824	2	42.855	KV453182.1	4236873	20-scf00019	20
AX-157912825	2	42.855	KV453182.1	4237832	20-scf00019	20
AX-157912827	2	42.855	KV453182.1	4240059	20-scf00019	20
AX-157912835	2	42.855	KV453182.1	4251651	20-scf00019	20
AX-157912838	2	42.855	KV453182.1	4259281	20-scf00019	20
AX-157912790	2	42.971	KV453182.1	4193568	20-scf00019	20
AX-157812440	2	42.971	KV453182.1	4258976	20-scf00019	20
AX-157812441	2	42.998	KV453182.1	4262988	20-scf00019	20
AX-157912847	2	42.998	KV453182.1	4272666	20-scf00019	20
AX-157912848	2	42.998	KV453182.1	4273518	20-scf00019	20
AX-157912849	2	42.998	KV453182.1	4273905	20-scf00019	20
AX-157912839	2	43.08	KV453182.1	4261098	20-scf00019	20
AX-157912841	2	43.08	KV453182.1	4262022	20-scf00019	20
AX-157912850	2	43.08	KV453182.1	4275007	20-scf00019	20
AX-157912804	2	43.141	KV453182.1	4216436	20-scf00019	20
AX-157912817	2	43.141	KV453182.1	4228627	20-scf00019	20
AX-157912818	2	43.141	KV453182.1	4229597	20-scf00019	20
AX-157912846	2	43.141	KV453182.1	4271758	20-scf00019	20
AX-157912852	2	43.141	KV453182.1	4276576	20-scf00019	20
AX-157912855	2	43.141	KV453182.1	4283021	20-scf00019	20
AX-157912860	2	43.141	KV453182.1	4287827	20-scf00019	20
AX-157912861	2	43.141	KV453182.1	4288709	20-scf00019	20
AX-157912863	2	43.141	KV453182.1	4290544	20-scf00019	20
AX-157912864	2	43.141	KV453182.1	4291380	20-scf00019	20

AX-157912865	2	43.141	KV453182.1	4291727	20-scf00019	20
AX-157812443	2	43.141	KV453182.1	4294585	20-scf00019	20
AX-157912872	2	43.141	KV453182.1	4306243	20-scf00019	20
AX-157912874	2	43.141	KV453182.1	4311936	20-scf00019	20
AX-157912875	2	43.141	KV453182.1	4312425	20-scf00019	20
AX-157912876	2	43.141	KV453182.1	4314264	20-scf00019	20
AX-157912880	2	43.141	KV453182.1	4318645	20-scf00019	20
AX-157912881	2	43.141	KV453182.1	4319796	20-scf00019	20
AX-157912883	2	43.141	KV453182.1	4320904	20-scf00019	20
AX-157912893	2	43.141	KV453182.1	4333423	20-scf00019	20
AX-158404959	2	43.141	KV453182.1	4335761	20-scf00019	20
AX-157912898	2	43.141	KV453182.1	4338625	20-scf00019	20
AX-157912901	2	43.141	KV453182.1	4341920	20-scf00019	20
AX-157912905	2	43.141	KV453182.1	4349285	20-scf00019	20
AX-157912911	2	43.141	KV453182.1	4356692	20-scf00019	20
AX-157812447	2	43.141	KV453182.1	4363598	20-scf00019	20
AX-157716524	2	43.141	KV453182.1	4368254	20-scf00019	20
AX-157912930	2	43.141	KV453182.1	4385375	20-scf00019	20
AX-157912939	2	43.141	KV453182.1	4398715	20-scf00019	20
AX-157912946	2	43.141	KV453182.1	4407845	20-scf00019	20
AX-158389583	2	43.141	KV453182.1	4413287	20-scf00019	20
AX-157912958	2	43.141	KV453182.1	4425512	20-scf00019	20
AX-157775591	2	43.141	KV453182.1	4426474	20-scf00019	20
AX-157913019	2	43.141	KV453182.1	4528554	20-scf00019	20
AX-157874651	2	43.141	KV453182.1	4535854	20-scf00019	20
AX-157913051	2	43.141	KV453182.1	4565614	20-scf00019	20
AX-157913117	2	43.141	KV453182.1	4653652	20-scf00019	20
AX-157912918	2	43.199	KV453182.1	4365208	20-scf00019	20
AX-157758651	2	43.259	KV453182.1	4273055	20-scf00019	20
AX-157912892	2	43.259	KV453182.1	4330660	20-scf00019	20
AX-157912894	2	43.259	KV453182.1	4334104	20-scf00019	20
AX-157912914	2	43.259	KV453182.1	4360663	20-scf00019	20
AX-157812445	2	43.259	KV453182.1	4360980	20-scf00019	20
AX-157912921	2	43.259	KV453182.1	4369407	20-scf00019	20
AX-157853699	2	43.283	KV453182.1	4282905	20-scf00019	20
AX-157912870	2	43.283	KV453182.1	4300031	20-scf00019	20
AX-157912891	2	43.283	KV453182.1	4329867	20-scf00019	20
AX-157912910	2	43.283	KV453182.1	4354916	20-scf00019	20
AX-157812449	2	43.283	KV453182.1	4373740	20-scf00019	20
AX-157812453	2	43.283	KV453182.1	4385304	20-scf00019	20
AX-157912931	2	43.283	KV453182.1	4385804	20-scf00019	20
AX-157912932	2	43.283	KV453182.1	4386876	20-scf00019	20
AX-157912934	2	43.283	KV453182.1	4390419	20-scf00019	20
AX-157812456	2	43.283	KV453182.1	4392466	20-scf00019	20
AX-157912935	2	43.283	KV453182.1	4393037	20-scf00019	20
AX-157812458	2	43.283	KV453182.1	4399806	20-scf00019	20
AX-157912952	2	43.283	KV453182.1	4418129	20-scf00019	20
AX-157912953	2	43.283	KV453182.1	4419702	20-scf00019	20
AX-157912955	2	43.283	KV453182.1	4420205	20-scf00019	20
AX-157912957	2	43.283	KV453182.1	4423793	20-scf00019	20
AX-157912960	2	43.283	KV453182.1	4432747	20-scf00019	20

AX-157912972	2	43.283	KV453182.1	4446182	20-scf00019	20
AX-157912974	2	43.283	KV453182.1	4447357	20-scf00019	20
AX-157912975	2	43.283	KV453182.1	4449012	20-scf00019	20
AX-157812460	2	43.283	KV453182.1	4484023	20-scf00019	20
AX-157874650	2	43.283	KV453182.1	4534576	20-scf00019	20
AX-157746532	2	43.283	KV453182.1	4569479	20-scf00019	20
AX-157913068	2	43.283	KV453182.1	4588346	20-scf00019	20
AX-158414797	2	43.283	KV453182.1	4633009	20-scf00019	20
AX-157912862	2	43.426	KV453182.1	4289539	20-scf00019	20
AX-157712154	2	43.426	KV453182.1	4386177	20-scf00019	20
AX-157864492	2	43.426	KV453182.1	4433374	20-scf00019	20
AX-157912963	2	43.426	KV453182.1	4436847	20-scf00019	20
AX-157912968	2	43.426	KV453182.1	4441084	20-scf00019	20
AX-157912971	2	43.426	KV453182.1	4445201	20-scf00019	20
AX-157724151	2	43.426	KV453182.1	4447205	20-scf00019	20
AX-157912977	2	43.426	KV453182.1	4453196	20-scf00019	20
AX-157912978	2	43.426	KV453182.1	4453890	20-scf00019	20
AX-157912981	2	43.426	KV453182.1	4456789	20-scf00019	20
AX-157912982	2	43.426	KV453182.1	4458778	20-scf00019	20
AX-157912983	2	43.426	KV453182.1	4458926	20-scf00019	20
AX-157912991	2	43.426	KV453182.1	4472008	20-scf00019	20
AX-157874648	2	43.426	KV453182.1	4473723	20-scf00019	20
AX-157912999	2	43.426	KV453182.1	4481423	20-scf00019	20
AX-157913005	2	43.426	KV453182.1	4487908	20-scf00019	20
AX-157913017	2	43.426	KV453182.1	4521370	20-scf00019	20
AX-157913018	2	43.426	KV453182.1	4523328	20-scf00019	20
AX-157913049	2	43.426	KV453182.1	4562382	20-scf00019	20
AX-157812471	2	43.426	KV453182.1	4591795	20-scf00019	20
AX-157913118	2	43.426	KV453182.1	4654799	20-scf00019	20
AX-157715389	2	43.426	KV453182.1	4761397	20-scf00019	20
AX-157913183	2	43.426	KV453182.1	4764495	20-scf00019	20
AX-157913199	2	43.426	KV453182.1	4782323	20-scf00019	20
AX-157812459	2	43.468	KV453182.1	4415936	20-scf00019	20
AX-157912992	2	43.468	KV453182.1	4472701	20-scf00019	20
AX-157913030	2	43.468	KV453182.1	4538694	20-scf00019	20
AX-157748246	2	43.468	KV453182.1	4543620	20-scf00019	20
AX-157912993	2	43.506	KV453182.1	4474201	20-scf00019	20
AX-157913020	2	43.506	KV453182.1	4529555	20-scf00019	20
AX-157913031	2	43.506	KV453182.1	4540187	20-scf00019	20
AX-157913185	2	43.506	KV453182.1	4766062	20-scf00019	20
AX-157912995	2	43.52	KV453182.1	4476091	20-scf00019	20
AX-157912851	2	43.569	KV453182.1	4276128	20-scf00019	20
AX-157912895	2	43.569	KV453182.1	4334812	20-scf00019	20
AX-157912902	2	43.569	KV453182.1	4344246	20-scf00019	20
AX-158404960	2	43.569	KV453182.1	4347984	20-scf00019	20
AX-157912949	2	43.569	KV453182.1	4413489	20-scf00019	20
AX-157912956	2	43.569	KV453182.1	4423097	20-scf00019	20
AX-157912964	2	43.569	KV453182.1	4437872	20-scf00019	20
AX-157912973	2	43.569	KV453182.1	4446545	20-scf00019	20
AX-157912979	2	43.569	KV453182.1	4454011	20-scf00019	20
AX-157912985	2	43.569	KV453182.1	4461388	20-scf00019	20

AX-157912997	2	43.569	KV453182.1	4477222	20-scf00019	20
AX-157912998	2	43.569	KV453182.1	4478922	20-scf00019	20
AX-157913009	2	43.569	KV453182.1	4497343	20-scf00019	20
AX-157913012	2	43.569	KV453182.1	4508976	20-scf00019	20
AX-157913013	2	43.569	KV453182.1	4512911	20-scf00019	20
AX-157714807	2	43.569	KV453182.1	4523961	20-scf00019	20
AX-157913025	2	43.569	KV453182.1	4532176	20-scf00019	20
AX-157874649	2	43.569	KV453182.1	4533250	20-scf00019	20
AX-157913028	2	43.569	KV453182.1	4535221	20-scf00019	20
AX-157771897	2	43.569	KV453182.1	4537967	20-scf00019	20
AX-157913033	2	43.569	KV453182.1	4541254	20-scf00019	20
AX-157713181	2	43.569	KV453182.1	4547413	20-scf00019	20
AX-157913040	2	43.569	KV453182.1	4550684	20-scf00019	20
AX-158404961	2	43.569	KV453182.1	4551304	20-scf00019	20
AX-157913041	2	43.569	KV453182.1	4552689	20-scf00019	20
AX-157913042	2	43.569	KV453182.1	4553395	20-scf00019	20
AX-157724189	2	43.569	KV453182.1	4554866	20-scf00019	20
AX-157913044	2	43.569	KV453182.1	4556807	20-scf00019	20
AX-157913047	2	43.569	KV453182.1	4559584	20-scf00019	20
AX-157913050	2	43.569	KV453182.1	4563648	20-scf00019	20
AX-157812469	2	43.569	KV453182.1	4564859	20-scf00019	20
AX-157812470	2	43.569	KV453182.1	4566812	20-scf00019	20
AX-157913055	2	43.569	KV453182.1	4569814	20-scf00019	20
AX-157913058	2	43.569	KV453182.1	4572770	20-scf00019	20
AX-158379248	2	43.569	KV453182.1	4573153	20-scf00019	20
AX-157913059	2	43.569	KV453182.1	4573345	20-scf00019	20
AX-157913062	2	43.569	KV453182.1	4576482	20-scf00019	20
AX-157913065	2	43.569	KV453182.1	4584248	20-scf00019	20
AX-157913067	2	43.569	KV453182.1	4587015	20-scf00019	20
AX-157913069	2	43.569	KV453182.1	4589309	20-scf00019	20
AX-157699847	2	43.569	KV453182.1	4596735	20-scf00019	20
AX-157913073	2	43.569	KV453182.1	4598442	20-scf00019	20
AX-157913081	2	43.569	KV453182.1	4608838	20-scf00019	20
AX-157913086	2	43.569	KV453182.1	4612328	20-scf00019	20
AX-157913092	2	43.569	KV453182.1	4628322	20-scf00019	20
AX-157913097	2	43.569	KV453182.1	4633530	20-scf00019	20
AX-157913106	2	43.569	KV453182.1	4642594	20-scf00019	20
AX-157913114	2	43.569	KV453182.1	4650166	20-scf00019	20
AX-157912967	2	43.712	KV453182.1	4440174	20-scf00019	20
AX-157913039	2	43.712	KV453182.1	4549731	20-scf00019	20
AX-157913082	2	43.712	KV453182.1	4609058	20-scf00019	20
AX-157913084	2	43.712	KV453182.1	4610993	20-scf00019	20
AX-157913085	2	43.712	KV453182.1	4611627	20-scf00019	20
AX-157812473	2	43.712	KV453182.1	4625719	20-scf00019	20
AX-157913107	2	43.712	KV453182.1	4643382	20-scf00019	20
AX-157913116	2	43.712	KV453182.1	4652247	20-scf00019	20
AX-157913109	2	43.713	KV453182.1	4645043	20-scf00019	20
AX-157913112	2	43.713	KV453182.1	4648390	20-scf00019	20
AX-157913010	2	43.997	KV453182.1	4499871	20-scf00019	20
AX-157722192	2	43.997	KV453182.1	4657428	20-scf00019	20
AX-157812474	2	43.997	KV453182.1	4659199	20-scf00019	20

AX-157913127	2	43.997	KV453182.1	4666067	20-scf00019	20
AX-157913133	2	43.997	KV453182.1	4674386	20-scf00019	20
AX-157913186	2	43.997	KV453182.1	4766658	20-scf00019	20
AX-157913267	2	43.997	KV453182.1	4883679	20-scf00019	20
AX-157742866	2	43.997	KV453182.1	4931484	20-scf00019	20
AX-157913477	2	43.997	KV453182.1	5146750	20-scf00019	20
AX-157812502	2	43.997	KV453182.1	5242236	20-scf00019	20
AX-157913559	2	43.997	KV453182.1	5245119	20-scf00019	20
AX-157912859	2	44.14	KV453182.1	4287436	20-scf00019	20
AX-157912879	2	44.14	KV453182.1	4316261	20-scf00019	20
AX-157912886	2	44.14	KV453182.1	4323484	20-scf00019	20
AX-157912908	2	44.14	KV453182.1	4352777	20-scf00019	20
AX-158414796	2	44.14	KV453182.1	4488712	20-scf00019	20
AX-157913078	2	44.14	KV453182.1	4606527	20-scf00019	20
AX-157913119	2	44.14	KV453182.1	4656763	20-scf00019	20
AX-157913134	2	44.14	KV453182.1	4678040	20-scf00019	20
AX-157717941	2	44.14	KV453182.1	4686170	20-scf00019	20
AX-157913141	2	44.14	KV453182.1	4696489	20-scf00019	20
AX-157913142	2	44.14	KV453182.1	4696963	20-scf00019	20
AX-157812478	2	44.14	KV453182.1	4699880	20-scf00019	20
AX-157812479	2	44.14	KV453182.1	4699994	20-scf00019	20
AX-157913147	2	44.14	KV453182.1	4705984	20-scf00019	20
AX-157913148	2	44.14	KV453182.1	4706279	20-scf00019	20
AX-157869092	2	44.14	KV453182.1	4706628	20-scf00019	20
AX-157913149	2	44.14	KV453182.1	4709072	20-scf00019	20
AX-157913150	2	44.14	KV453182.1	4709665	20-scf00019	20
AX-157913152	2	44.14	KV453182.1	4714463	20-scf00019	20
AX-157913157	2	44.14	KV453182.1	4725700	20-scf00019	20
AX-157913163	2	44.14	KV453182.1	4738006	20-scf00019	20
AX-157913170	2	44.14	KV453182.1	4745371	20-scf00019	20
AX-157913177	2	44.14	KV453182.1	4755684	20-scf00019	20
AX-157913181	2	44.14	KV453182.1	4759445	20-scf00019	20
AX-157913254	2	44.14	KV453182.1	4869608	20-scf00019	20
AX-157913169	2	44.141	KV453182.1	4744961	20-scf00019	20
AX-157913180	2	44.141	KV453182.1	4757297	20-scf00019	20
AX-157913201	2	44.141	KV453182.1	4784484	20-scf00019	20
AX-157913205	2	44.361	KV453182.1	4792850	20-scf00019	20
AX-157913206	2	44.425	KV453182.1	4793038	20-scf00019	20
AX-157913207	2	44.426	KV453182.1	4793813	20-scf00019	20
AX-157913208	2	44.426	KV453182.1	4794896	20-scf00019	20
AX-157741963	2	44.426	KV453182.1	4798608	20-scf00019	20
AX-157912671	2	44.569	KV453182.1	4031943	20-scf00019	20
AX-157912683	2	44.569	KV453182.1	4048124	20-scf00019	20
AX-157912697	2	44.569	KV453182.1	4065278	20-scf00019	20
AX-157912714	2	44.569	KV453182.1	4091967	20-scf00019	20
AX-157912723	2	44.569	KV453182.1	4106425	20-scf00019	20
AX-157912736	2	44.569	KV453182.1	4124137	20-scf00019	20
AX-157912741	2	44.569	KV453182.1	4128716	20-scf00019	20
AX-157812433	2	44.569	KV453182.1	4161208	20-scf00019	20
AX-157912858	2	44.569	KV453182.1	4286363	20-scf00019	20
AX-157912877	2	44.569	KV453182.1	4314808	20-scf00019	20

AX-157912878	2	44.569	KV453182.1	4315883	20-scf00019	20
AX-157912882	2	44.569	KV453182.1	4320427	20-scf00019	20
AX-157912884	2	44.569	KV453182.1	4321158	20-scf00019	20
AX-157912919	2	44.569	KV453182.1	4365929	20-scf00019	20
AX-157853702	2	44.569	KV453182.1	4369833	20-scf00019	20
AX-157913007	2	44.569	KV453182.1	4492080	20-scf00019	20
AX-157913022	2	44.569	KV453182.1	4531252	20-scf00019	20
AX-157913023	2	44.569	KV453182.1	4531735	20-scf00019	20
AX-157913053	2	44.569	KV453182.1	4568286	20-scf00019	20
AX-157913064	2	44.569	KV453182.1	4581503	20-scf00019	20
AX-157699848	2	44.569	KV453182.1	4596596	20-scf00019	20
AX-157913075	2	44.569	KV453182.1	4599549	20-scf00019	20
AX-157913093	2	44.569	KV453182.1	4628967	20-scf00019	20
AX-157913108	2	44.569	KV453182.1	4643535	20-scf00019	20
AX-157913115	2	44.569	KV453182.1	4651823	20-scf00019	20
AX-157913129	2	44.569	KV453182.1	4671158	20-scf00019	20
AX-157812476	2	44.569	KV453182.1	4675404	20-scf00019	20
AX-157913156	2	44.569	KV453182.1	4725298	20-scf00019	20
AX-157913175	2	44.569	KV453182.1	4752222	20-scf00019	20
AX-157913212	2	44.569	KV453182.1	4798678	20-scf00019	20
AX-157738423	2	44.569	KV453182.1	4811018	20-scf00019	20
AX-157913226	2	44.569	KV453182.1	4817122	20-scf00019	20
AX-157913227	2	44.569	KV453182.1	4820761	20-scf00019	20
AX-157913228	2	44.569	KV453182.1	4821414	20-scf00019	20
AX-157913230	2	44.569	KV453182.1	4823331	20-scf00019	20
AX-157913231	2	44.569	KV453182.1	4825312	20-scf00019	20
AX-157913237	2	44.569	KV453182.1	4830587	20-scf00019	20
AX-157812485	2	44.569	KV453182.1	4837727	20-scf00019	20
AX-157812486	2	44.569	KV453182.1	4839122	20-scf00019	20
AX-157913241	2	44.569	KV453182.1	4839353	20-scf00019	20
AX-157812487	2	44.569	KV453182.1	4839736	20-scf00019	20
AX-157913242	2	44.569	KV453182.1	4840680	20-scf00019	20
AX-157913243	2	44.569	KV453182.1	4841870	20-scf00019	20
AX-157913245	2	44.569	KV453182.1	4846881	20-scf00019	20
AX-157812490	2	44.569	KV453182.1	4847757	20-scf00019	20
AX-157913247	2	44.569	KV453182.1	4853606	20-scf00019	20
AX-157913250	2	44.569	KV453182.1	4857269	20-scf00019	20
AX-157913251	2	44.569	KV453182.1	4859609	20-scf00019	20
AX-157913252	2	44.569	KV453182.1	4860392	20-scf00019	20
AX-157812492	2	44.569	KV453182.1	4862163	20-scf00019	20
AX-157812494	2	44.569	KV453182.1	4863387	20-scf00019	20
AX-157913258	2	44.569	KV453182.1	4874944	20-scf00019	20
AX-157913262	2	44.569	KV453182.1	4879949	20-scf00019	20
AX-157913263	2	44.569	KV453182.1	4880535	20-scf00019	20
AX-157913265	2	44.569	KV453182.1	4881709	20-scf00019	20
AX-157913268	2	44.569	KV453182.1	4888422	20-scf00019	20
AX-157913270	2	44.569	KV453182.1	4890939	20-scf00019	20
AX-157913272	2	44.569	KV453182.1	4895672	20-scf00019	20
AX-157913273	2	44.569	KV453182.1	4896374	20-scf00019	20
AX-157913275	2	44.569	KV453182.1	4898454	20-scf00019	20
AX-157913276	2	44.569	KV453182.1	4899358	20-scf00019	20

AX-157913277	2	44.569	KV453182.1	4900993	20-scf00019	20
AX-157913279	2	44.569	KV453182.1	4905238	20-scf00019	20
AX-157743560	2	44.699	KV453182.1	4865420	20-scf00019	20
AX-157913285	2	44.699	KV453182.1	4921921	20-scf00019	20
AX-157913286	2	44.699	KV453182.1	4922772	20-scf00019	20
AX-157913287	2	44.699	KV453182.1	4923294	20-scf00019	20
AX-157913288	2	44.699	KV453182.1	4923495	20-scf00019	20
AX-157913289	2	44.699	KV453182.1	4925316	20-scf00019	20
AX-157913295	2	44.75	KV453182.1	4932664	20-scf00019	20
AX-157913298	2	44.789	KV453182.1	4935595	20-scf00019	20
AX-157913234	2	44.83	KV453182.1	4827378	20-scf00019	20
AX-157913253	2	44.83	KV453182.1	4869034	20-scf00019	20
AX-157913299	2	44.83	KV453182.1	4936637	20-scf00019	20
AX-157913301	2	44.83	KV453182.1	4943313	20-scf00019	20
AX-157913305	2	44.83	KV453182.1	4947168	20-scf00019	20
AX-157913308	2	44.83	KV453182.1	4951263	20-scf00019	20
AX-157914530	2	44.83	KV453182.1	7074571	20-scf00019	20
AX-157914901	2	44.83	KV453193.1	21807	20-scf00021	20
AX-157914953	2	44.83	KV453193.1	102558	20-scf00021	20
AX-157913266	2	45.09	KV453182.1	4882205	20-scf00019	20
AX-157913311	2	45.09	KV453182.1	4954193	20-scf00019	20
AX-157913313	2	45.09	KV453182.1	4955682	20-scf00019	20
AX-157913314	2	45.09	KV453182.1	4956537	20-scf00019	20
AX-157913315	2	45.09	KV453182.1	4957160	20-scf00019	20
AX-157913316	2	45.09	KV453182.1	4958299	20-scf00019	20
AX-157913318	2	45.09	KV453182.1	4961909	20-scf00019	20
AX-157874656	2	45.09	KV453182.1	4962635	20-scf00019	20
AX-157913321	2	45.09	KV453182.1	4964486	20-scf00019	20
AX-157913323	2	45.09	KV453182.1	4967028	20-scf00019	20
AX-157913325	2	45.09	KV453182.1	4968247	20-scf00019	20
AX-157913335	2	45.09	KV453182.1	4976201	20-scf00019	20
AX-157913339	2	45.09	KV453182.1	4979890	20-scf00019	20
AX-157913343	2	45.09	KV453182.1	4983777	20-scf00019	20
AX-157913350	2	45.09	KV453182.1	4991145	20-scf00019	20
AX-157853709	2	45.09	KV453182.1	4998155	20-scf00019	20
AX-157913365	2	45.09	KV453182.1	5011412	20-scf00019	20
AX-157750067	2	45.09	KV453182.1	5013471	20-scf00019	20
AX-157913366	2	45.09	KV453182.1	5014017	20-scf00019	20
AX-157913382	2	45.09	KV453182.1	5028928	20-scf00019	20
AX-157913387	2	45.09	KV453182.1	5032810	20-scf00019	20
AX-157913389	2	45.09	KV453182.1	5034101	20-scf00019	20
AX-157913404	2	45.09	KV453182.1	5052990	20-scf00019	20
AX-157913424	2	45.09	KV453182.1	5080530	20-scf00019	20
AX-157913452	2	45.09	KV453182.1	5122204	20-scf00019	20
AX-157913460	2	45.09	KV453182.1	5129338	20-scf00019	20
AX-157913461	2	45.09	KV453182.1	5129856	20-scf00019	20
AX-157738750	2	45.09	KV453182.1	5198075	20-scf00019	20
AX-157913535	2	45.09	KV453182.1	5219353	20-scf00019	20
AX-158382243	2	45.09	KV453182.1	5220132	20-scf00019	20
AX-157913548	2	45.09	KV453182.1	5237636	20-scf00019	20
AX-157913317	2	45.152	KV453182.1	4959965	20-scf00019	20

AX-157913338	2	45.152	KV453182.1	4978658	20-scf00019	20
AX-157695124	2	45.152	KV453182.1	5014744	20-scf00019	20
AX-157913213	2	45.22	KV453182.1	4800192	20-scf00019	20
AX-157913214	2	45.22	KV453182.1	4800581	20-scf00019	20
AX-157913290	2	45.22	KV453182.1	4926317	20-scf00019	20
AX-157913342	2	45.22	KV453182.1	4982538	20-scf00019	20
AX-157913344	2	45.22	KV453182.1	4984579	20-scf00019	20
AX-157913353	2	45.22	KV453182.1	4993439	20-scf00019	20
AX-157913354	2	45.22	KV453182.1	4995519	20-scf00019	20
AX-157913358	2	45.22	KV453182.1	5000493	20-scf00019	20
AX-157913364	2	45.22	KV453182.1	5008913	20-scf00019	20
AX-157741401	2	45.22	KV453182.1	5014670	20-scf00019	20
AX-157913371	2	45.22	KV453182.1	5018103	20-scf00019	20
AX-157913380	2	45.22	KV453182.1	5026898	20-scf00019	20
AX-157913385	2	45.22	KV453182.1	5031618	20-scf00019	20
AX-157913386	2	45.22	KV453182.1	5032230	20-scf00019	20
AX-157913388	2	45.22	KV453182.1	5033492	20-scf00019	20
AX-157913390	2	45.22	KV453182.1	5034697	20-scf00019	20
AX-158382238	2	45.22	KV453182.1	5091144	20-scf00019	20
AX-157913478	2	45.22	KV453182.1	5147313	20-scf00019	20
AX-157913518	2	45.22	KV453182.1	5196989	20-scf00019	20
AX-157913526	2	45.22	KV453182.1	5209654	20-scf00019	20
AX-157913532	2	45.22	KV453182.1	5215564	20-scf00019	20
AX-157913605	2	45.22	KV453182.1	5310299	20-scf00019	20
AX-157705742	2	45.22	3ML01030766	516	scf06368_1	UN
AX-157913396	2	45.29	KV453182.1	5046888	20-scf00019	20
AX-157915433	2	45.29	KV453193.1	715053	20-scf00021	20
AX-157920156	2	45.29	KV453183.1	913603	20-scf00571	20
AX-157913402	2	45.349	KV453182.1	5051411	20-scf00019	20
AX-157913376	2	45.411	KV453182.1	5023450	20-scf00019	20
AX-157913407	2	45.411	KV453182.1	5055396	20-scf00019	20
AX-157913408	2	45.411	KV453182.1	5055848	20-scf00019	20
AX-157913569	2	45.411	KV453182.1	5261076	20-scf00019	20
AX-157913570	2	45.411	KV453182.1	5261381	20-scf00019	20
AX-157812509	2	45.411	KV453182.1	5261493	20-scf00019	20
AX-157913624	2	45.411	KV453182.1	5338310	20-scf00019	20
AX-157913415	2	45.481	KV453182.1	5073558	20-scf00019	20
AX-157853710	2	45.481	KV453182.1	5083287	20-scf00019	20
AX-157913300	2	45.529	KV453182.1	4941108	20-scf00019	20
AX-157744873	2	45.529	KV453182.1	5086580	20-scf00019	20
AX-157913428	2	45.529	KV453182.1	5088172	20-scf00019	20
AX-157913455	2	45.529	KV453182.1	5125493	20-scf00019	20
AX-157913482	2	45.529	KV453182.1	5151600	20-scf00019	20
AX-157913446	2	45.572	KV453182.1	5113422	20-scf00019	20
AX-157913246	2	45.611	KV453182.1	4853151	20-scf00019	20
AX-157913330	2	45.611	KV453182.1	4971975	20-scf00019	20
AX-157913378	2	45.611	KV453182.1	5024508	20-scf00019	20
AX-157913405	2	45.611	KV453182.1	5054475	20-scf00019	20
AX-157913417	2	45.611	KV453182.1	5074766	20-scf00019	20
AX-157913421	2	45.611	KV453182.1	5077483	20-scf00019	20
AX-157913422	2	45.611	KV453182.1	5078588	20-scf00019	20

AX-157723218	2	45.611	KV453182.1	5115110	20-scf00019	20
AX-157913449	2	45.611	KV453182.1	5117085	20-scf00019	20
AX-157913450	2	45.611	KV453182.1	5119012	20-scf00019	20
AX-157913459	2	45.611	KV453182.1	5127425	20-scf00019	20
AX-157913462	2	45.611	KV453182.1	5131478	20-scf00019	20
AX-157913465	2	45.611	KV453182.1	5134579	20-scf00019	20
AX-157913470	2	45.611	KV453182.1	5141270	20-scf00019	20
AX-157913474	2	45.611	KV453182.1	5144339	20-scf00019	20
AX-157913476	2	45.611	KV453182.1	5146189	20-scf00019	20
AX-157913483	2	45.611	KV453182.1	5152196	20-scf00019	20
AX-157913484	2	45.611	KV453182.1	5156066	20-scf00019	20
AX-157913487	2	45.611	KV453182.1	5159144	20-scf00019	20
AX-157913490	2	45.611	KV453182.1	5164180	20-scf00019	20
AX-157913492	2	45.611	KV453182.1	5165934	20-scf00019	20
AX-157913493	2	45.611	KV453182.1	5169203	20-scf00019	20
AX-157913494	2	45.611	KV453182.1	5170313	20-scf00019	20
AX-157913498	2	45.611	KV453182.1	5177126	20-scf00019	20
AX-158404973	2	45.611	KV453182.1	5186343	20-scf00019	20
AX-157709871	2	45.611	KV453182.1	5187752	20-scf00019	20
AX-157913511	2	45.611	KV453182.1	5191348	20-scf00019	20
AX-157913516	2	45.611	KV453182.1	5195409	20-scf00019	20
AX-157913519	2	45.611	KV453182.1	5197420	20-scf00019	20
AX-157913522	2	45.611	KV453182.1	5205877	20-scf00019	20
AX-157913524	2	45.611	KV453182.1	5208491	20-scf00019	20
AX-157913528	2	45.611	KV453182.1	5211875	20-scf00019	20
AX-157913541	2	45.611	KV453182.1	5226210	20-scf00019	20
AX-157913543	2	45.611	KV453182.1	5231986	20-scf00019	20
AX-157913544	2	45.611	KV453182.1	5233464	20-scf00019	20
AX-157913167	2	45.742	KV453182.1	4741099	20-scf00019	20
AX-157913557	2	45.742	KV453182.1	5243685	20-scf00019	20
AX-157913561	2	45.742	KV453182.1	5247268	20-scf00019	20
AX-157913562	2	45.742	KV453182.1	5250399	20-scf00019	20
AX-157913563	2	45.742	KV453182.1	5251460	20-scf00019	20
AX-157812504	2	45.742	KV453182.1	5251770	20-scf00019	20
AX-157812505	2	45.742	KV453182.1	5252303	20-scf00019	20
AX-157812506	2	45.742	KV453182.1	5255113	20-scf00019	20
AX-157913565	2	45.742	KV453182.1	5256286	20-scf00019	20
AX-157913618	2	45.742	KV453182.1	5327048	20-scf00019	20
AX-157812507	2	45.779	KV453182.1	5256823	20-scf00019	20
AX-157913566	2	45.805	KV453182.1	5257055	20-scf00019	20
AX-157745615	2	45.805	KV453182.1	5257453	20-scf00019	20
AX-157812508	2	45.805	KV453182.1	5259318	20-scf00019	20
AX-157913567	2	45.81	KV453182.1	5260210	20-scf00019	20
AX-157913571	2	45.81	KV453182.1	5266507	20-scf00019	20
AX-157913576	2	45.81	KV453182.1	5268864	20-scf00019	20
AX-157913620	2	45.81	KV453182.1	5330062	20-scf00019	20
AX-157913662	2	45.81	KV453182.1	5402247	20-scf00019	20
AX-157913746	2	45.81	KV453182.1	5537531	20-scf00019	20
AX-157913507	2	45.841	KV453182.1	5188278	20-scf00019	20
AX-157913580	2	45.841	KV453182.1	5271822	20-scf00019	20
AX-157913581	2	45.872	KV453182.1	5272186	20-scf00019	20

AX-157913583	2	45.872	KV453182.1	5273663	20-scf00019	20
AX-157913587	2	45.872	KV453182.1	5279177	20-scf00019	20
AX-157913588	2	45.872	KV453182.1	5282895	20-scf00019	20
AX-157913590	2	45.872	KV453182.1	5284746	20-scf00019	20
AX-157913597	2	45.872	KV453182.1	5298024	20-scf00019	20
AX-157913607	2	45.872	KV453182.1	5312609	20-scf00019	20
AX-157913630	2	45.872	KV453182.1	5346717	20-scf00019	20
AX-157913603	2	45.882	KV453182.1	5308497	20-scf00019	20
AX-157913609	2	45.882	KV453182.1	5315702	20-scf00019	20
AX-157913610	2	45.882	KV453182.1	5318391	20-scf00019	20
AX-157913663	2	45.882	KV453182.1	5402686	20-scf00019	20
AX-157913612	2	45.913	KV453182.1	5319481	20-scf00019	20
AX-157913616	2	45.946	KV453182.1	5321989	20-scf00019	20
AX-157913619	2	45.988	KV453182.1	5327458	20-scf00019	20
AX-157913626	2	46.002	KV453182.1	5341396	20-scf00019	20
AX-157913629	2	46.002	KV453182.1	5345936	20-scf00019	20
AX-157913803	2	46.002	KV453182.1	5604894	20-scf00019	20
AX-157914421	2	46.002	KV453182.1	6613100	20-scf00019	20
AX-157702170	2	46.002	KV453189.1	1513647	20-scf00020	20
AX-158375408	2	46.002	KV453187.1	502035	20-scf00563	20
AX-158375413	2	46.002	KV453187.1	527634	20-scf00563	20
AX-157913634	2	46.245	KV453182.1	5350197	20-scf00019	20
AX-157913336	2	46.251	KV453182.1	4977289	20-scf00019	20
AX-157913410	2	46.251	KV453182.1	5059104	20-scf00019	20
AX-157709319	2	46.251	KV453182.1	5327351	20-scf00019	20
AX-157913622	2	46.251	KV453182.1	5332487	20-scf00019	20
AX-157913635	2	46.251	KV453182.1	5351064	20-scf00019	20
AX-157913637	2	46.251	KV453182.1	5358807	20-scf00019	20
AX-157812515	2	46.251	KV453182.1	5368953	20-scf00019	20
AX-157913645	2	46.251	KV453182.1	5372789	20-scf00019	20
AX-157812516	2	46.251	KV453182.1	5378195	20-scf00019	20
AX-157752786	2	46.251	KV453182.1	5378572	20-scf00019	20
AX-157913654	2	46.251	KV453182.1	5387205	20-scf00019	20
AX-157913660	2	46.251	KV453182.1	5399988	20-scf00019	20
AX-157914366	2	46.251	KV453182.1	6248243	20-scf00019	20
AX-157741556	2	46.374	KV453182.1	5314813	20-scf00019	20
AX-157913657	2	46.374	KV453182.1	5392767	20-scf00019	20
AX-157913666	2	46.374	KV453182.1	5408140	20-scf00019	20
AX-157913667	2	46.374	KV453182.1	5409109	20-scf00019	20
AX-157913696	2	46.374	KV453182.1	5465186	20-scf00019	20
AX-157693618	2	46.374	KV453182.1	5515457	20-scf00019	20
AX-157913573	2	46.495	KV453182.1	5267516	20-scf00019	20
AX-157913664	2	46.495	KV453182.1	5404101	20-scf00019	20
AX-157913672	2	46.495	KV453182.1	5424806	20-scf00019	20
AX-157913686	2	46.495	KV453182.1	5449445	20-scf00019	20
AX-157913695	2	46.495	KV453182.1	5464474	20-scf00019	20
AX-157812523	2	46.495	KV453182.1	5467717	20-scf00019	20
AX-157913725	2	46.495	KV453182.1	5505999	20-scf00019	20
AX-157915030	2	46.495	KV453193.1	194168	20-scf00021	20
AX-157747048	2	46.495	KV453193.1	440762	20-scf00021	20
AX-157915644	2	46.495	KV453193.1	1040526	20-scf00021	20

AX-157915720	2	46.495	KV453193.1	1148575	20-scf00021	20
AX-157913591	2	46.496	KV453182.1	5286003	20-scf00019	20
AX-157913596	2	46.496	KV453182.1	5295163	20-scf00019	20
AX-157913602	2	46.496	KV453182.1	5308329	20-scf00019	20
AX-157913614	2	46.496	KV453182.1	5320050	20-scf00019	20
AX-157913633	2	46.496	KV453182.1	5349035	20-scf00019	20
AX-157710689	2	46.496	KV453182.1	5393834	20-scf00019	20
AX-157717773	2	46.496	KV453182.1	5405852	20-scf00019	20
AX-157913671	2	46.496	KV453182.1	5421526	20-scf00019	20
AX-157913676	2	46.496	KV453182.1	5434492	20-scf00019	20
AX-157812522	2	46.496	KV453182.1	5438151	20-scf00019	20
AX-157913680	2	46.496	KV453182.1	5441803	20-scf00019	20
AX-157913683	2	46.496	KV453182.1	5444981	20-scf00019	20
AX-157913684	2	46.496	KV453182.1	5447356	20-scf00019	20
AX-157913692	2	46.496	KV453182.1	5461391	20-scf00019	20
AX-157726905	2	46.496	KV453182.1	5464028	20-scf00019	20
AX-157913703	2	46.496	KV453182.1	5477323	20-scf00019	20
AX-157913704	2	46.496	KV453182.1	5477793	20-scf00019	20
AX-157913706	2	46.496	KV453182.1	5479057	20-scf00019	20
AX-157913708	2	46.496	KV453182.1	5481674	20-scf00019	20
AX-157913710	2	46.496	KV453182.1	5483306	20-scf00019	20
AX-157913715	2	46.496	KV453182.1	5495448	20-scf00019	20
AX-157913717	2	46.496	KV453182.1	5496292	20-scf00019	20
AX-157913738	2	46.496	KV453182.1	5521569	20-scf00019	20
AX-157913745	2	46.496	KV453182.1	5537018	20-scf00019	20
AX-157913756	2	46.496	KV453182.1	5550538	20-scf00019	20
AX-157913761	2	46.496	KV453182.1	5554376	20-scf00019	20
AX-157913762	2	46.496	KV453182.1	5559031	20-scf00019	20
AX-157913767	2	46.496	KV453182.1	5566491	20-scf00019	20
AX-157913769	2	46.496	KV453182.1	5568756	20-scf00019	20
AX-157913771	2	46.496	KV453182.1	5570067	20-scf00019	20
AX-157913772	2	46.496	KV453182.1	5570981	20-scf00019	20
AX-157913774	2	46.496	KV453182.1	5573549	20-scf00019	20
AX-157812525	2	46.496	KV453182.1	5577489	20-scf00019	20
AX-157913779	2	46.496	KV453182.1	5577642	20-scf00019	20
AX-157913780	2	46.496	KV453182.1	5578416	20-scf00019	20
AX-157913781	2	46.496	KV453182.1	5578931	20-scf00019	20
AX-157913785	2	46.496	KV453182.1	5587606	20-scf00019	20
AX-157913786	2	46.496	KV453182.1	5588583	20-scf00019	20
AX-157913787	2	46.496	KV453182.1	5589439	20-scf00019	20
AX-157913789	2	46.496	KV453182.1	5590564	20-scf00019	20
AX-157913790	2	46.496	KV453182.1	5591319	20-scf00019	20
AX-157913791	2	46.496	KV453182.1	5591882	20-scf00019	20
AX-157913792	2	46.496	KV453182.1	5592391	20-scf00019	20
AX-157913794	2	46.496	KV453182.1	5595427	20-scf00019	20
AX-157812526	2	46.496	KV453182.1	5596350	20-scf00019	20
AX-157913795	2	46.496	KV453182.1	5596806	20-scf00019	20
AX-157913796	2	46.496	KV453182.1	5597348	20-scf00019	20
AX-157913798	2	46.496	KV453182.1	5599037	20-scf00019	20
AX-157913799	2	46.496	KV453182.1	5599393	20-scf00019	20
AX-157913801	2	46.496	KV453182.1	5603484	20-scf00019	20

AX-157913804	2	46.496	KV453182.1	5606427	20-scf00019	20
AX-157913805	2	46.496	KV453182.1	5609073	20-scf00019	20
AX-157913806	2	46.496	KV453182.1	5609470	20-scf00019	20
AX-157913808	2	46.496	KV453182.1	5610061	20-scf00019	20
AX-157913810	2	46.496	KV453182.1	5613624	20-scf00019	20
AX-157913813	2	46.496	KV453182.1	5616326	20-scf00019	20
AX-157913817	2	46.496	KV453182.1	5620083	20-scf00019	20
AX-157913822	2	46.496	KV453182.1	5624511	20-scf00019	20
AX-157913823	2	46.496	KV453182.1	5627379	20-scf00019	20
AX-157913824	2	46.496	KV453182.1	5627909	20-scf00019	20
AX-157913825	2	46.496	KV453182.1	5628271	20-scf00019	20
AX-157812527	2	46.496	KV453182.1	5629965	20-scf00019	20
AX-157913827	2	46.496	KV453182.1	5630640	20-scf00019	20
AX-157913828	2	46.496	KV453182.1	5631269	20-scf00019	20
AX-157913829	2	46.496	KV453182.1	5631629	20-scf00019	20
AX-157913830	2	46.496	KV453182.1	5631984	20-scf00019	20
AX-157812528	2	46.496	KV453182.1	5633672	20-scf00019	20
AX-157812529	2	46.496	KV453182.1	5634140	20-scf00019	20
AX-157913831	2	46.496	KV453182.1	5634330	20-scf00019	20
AX-157913832	2	46.496	KV453182.1	5636005	20-scf00019	20
AX-157913833	2	46.496	KV453182.1	5637340	20-scf00019	20
AX-157913834	2	46.496	KV453182.1	5639098	20-scf00019	20
AX-157913835	2	46.496	KV453182.1	5640533	20-scf00019	20
AX-157913836	2	46.496	KV453182.1	5641232	20-scf00019	20
AX-157913837	2	46.496	KV453182.1	5642162	20-scf00019	20
AX-157716014	2	46.496	KV453182.1	5642490	20-scf00019	20
AX-157913838	2	46.496	KV453182.1	5642768	20-scf00019	20
AX-157739827	2	46.496	KV453182.1	5645888	20-scf00019	20
AX-157913840	2	46.496	KV453182.1	5646221	20-scf00019	20
AX-157913841	2	46.496	KV453182.1	5646628	20-scf00019	20
AX-157913843	2	46.496	KV453182.1	5648234	20-scf00019	20
AX-157913844	2	46.496	KV453182.1	5648726	20-scf00019	20
AX-157913846	2	46.496	KV453182.1	5649754	20-scf00019	20
AX-157913847	2	46.496	KV453182.1	5650614	20-scf00019	20
AX-157913849	2	46.496	KV453182.1	5654862	20-scf00019	20
AX-157913850	2	46.496	KV453182.1	5655649	20-scf00019	20
AX-157740485	2	46.496	KV453182.1	5658311	20-scf00019	20
AX-157812531	2	46.496	KV453182.1	5660463	20-scf00019	20
AX-157913855	2	46.496	KV453182.1	5660774	20-scf00019	20
AX-157913857	2	46.496	KV453182.1	5662915	20-scf00019	20
AX-157913858	2	46.496	KV453182.1	5664431	20-scf00019	20
AX-157913859	2	46.496	KV453182.1	5668186	20-scf00019	20
AX-157812552	2	46.496	KV453182.1	5864372	20-scf00019	20
AX-157914080	2	46.496	KV453182.1	5911807	20-scf00019	20
AX-157915325	2	46.496	KV453193.1	579024	20-scf00021	20
AX-157920238	2	46.496	KV453183.1	1205658	20-scf00571	20
AX-157920264	2	46.496	KV453183.1	1293065	20-scf00571	20
AX-157913860	2	46.513	KV453182.1	5669337	20-scf00019	20
AX-157913747	2	46.53	KV453182.1	5538134	20-scf00019	20
AX-157913793	2	46.53	KV453182.1	5594229	20-scf00019	20
AX-157913861	2	46.53	KV453182.1	5670578	20-scf00019	20

AX-157913877	2	46.53	KV453182.1	5687895	20-scf00019	20
AX-157914145	2	46.53	KV453182.1	5974050	20-scf00019	20
AX-157914243	2	46.53	KV453182.1	6093399	20-scf00019	20
AX-157812570	2	46.53	KV453182.1	6117179	20-scf00019	20
AX-157914259	2	46.53	KV453182.1	6118111	20-scf00019	20
AX-157914260	2	46.53	KV453182.1	6118444	20-scf00019	20
AX-157812571	2	46.53	KV453182.1	6119139	20-scf00019	20
AX-157914348	2	46.53	KV453182.1	6208749	20-scf00019	20
AX-157726428	2	46.53	KV453182.1	6221293	20-scf00019	20
AX-157812578	2	46.53	KV453182.1	6296629	20-scf00019	20
AX-157914392	2	46.53	KV453182.1	6343259	20-scf00019	20
AX-158404223	2	46.53	KV453182.1	6487229	20-scf00019	20
AX-157914413	2	46.53	KV453182.1	6546603	20-scf00019	20
AX-158404994	2	46.53	KV453182.1	6846495	20-scf00019	20
AX-157914481	2	46.53	KV453182.1	6907238	20-scf00019	20
AX-157914483	2	46.53	KV453182.1	6908689	20-scf00019	20
AX-157914487	2	46.53	KV453182.1	6923325	20-scf00019	20
AX-158404998	2	46.53	KV453182.1	6924875	20-scf00019	20
AX-157722019	2	46.53	KV453182.1	6946617	20-scf00019	20
AX-157914513	2	46.53	KV453182.1	7022451	20-scf00019	20
AX-157914531	2	46.53	KV453182.1	7075282	20-scf00019	20
AX-157914564	2	46.53	KV453182.1	7176308	20-scf00019	20
AX-157914574	2	46.53	KV453182.1	7219617	20-scf00019	20
AX-157914581	2	46.53	KV453182.1	7259741	20-scf00019	20
AX-157914583	2	46.53	KV453182.1	7263912	20-scf00019	20
AX-157914589	2	46.53	KV453182.1	7360780	20-scf00019	20
AX-158375425	2	46.53	KV453187.1	613363	20-scf00563	20
AX-158375479	2	46.53	KV453187.1	1230543	20-scf00563	20
AX-157913698	2	46.57	KV453182.1	5467044	20-scf00019	20
AX-157913864	2	46.57	KV453182.1	5678581	20-scf00019	20
AX-157913869	2	46.57	KV453182.1	5682847	20-scf00019	20
AX-157913870	2	46.57	KV453182.1	5683411	20-scf00019	20
AX-157914149	2	46.57	KV453182.1	5980392	20-scf00019	20
AX-157913862	2	46.636	KV453182.1	5672303	20-scf00019	20
AX-157913863	2	46.636	KV453182.1	5676613	20-scf00019	20
AX-157913866	2	46.636	KV453182.1	5679947	20-scf00019	20
AX-157913871	2	46.636	KV453182.1	5684065	20-scf00019	20
AX-157913872	2	46.636	KV453182.1	5684288	20-scf00019	20
AX-157913873	2	46.636	KV453182.1	5685479	20-scf00019	20
AX-157913875	2	46.636	KV453182.1	5686742	20-scf00019	20
AX-157913879	2	46.636	KV453182.1	5692326	20-scf00019	20
AX-157913882	2	46.636	KV453182.1	5696461	20-scf00019	20
AX-157913883	2	46.636	KV453182.1	5696695	20-scf00019	20
AX-157913884	2	46.636	KV453182.1	5697132	20-scf00019	20
AX-157913885	2	46.636	KV453182.1	5698043	20-scf00019	20
AX-157913886	2	46.636	KV453182.1	5698432	20-scf00019	20
AX-157913887	2	46.636	KV453182.1	5699166	20-scf00019	20
AX-157913888	2	46.636	KV453182.1	5701586	20-scf00019	20
AX-157746362	2	46.636	KV453182.1	5704582	20-scf00019	20
AX-157913889	2	46.636	KV453182.1	5707343	20-scf00019	20
AX-157913890	2	46.636	KV453182.1	5707895	20-scf00019	20

AX-157913891	2	46.636	KV453182.1	5708603	20-scf00019	20
AX-157913894	2	46.636	KV453182.1	5710332	20-scf00019	20
AX-157913895	2	46.636	KV453182.1	5711565	20-scf00019	20
AX-158389594	2	46.636	KV453182.1	5711904	20-scf00019	20
AX-157913896	2	46.636	KV453182.1	5712226	20-scf00019	20
AX-157913897	2	46.636	KV453182.1	5712961	20-scf00019	20
AX-157913898	2	46.636	KV453182.1	5713524	20-scf00019	20
AX-157913899	2	46.636	KV453182.1	5714145	20-scf00019	20
AX-157913900	2	46.636	KV453182.1	5715894	20-scf00019	20
AX-157913902	2	46.636	KV453182.1	5718143	20-scf00019	20
AX-157913903	2	46.636	KV453182.1	5718852	20-scf00019	20
AX-157913904	2	46.636	KV453182.1	5719649	20-scf00019	20
AX-157913905	2	46.636	KV453182.1	5719762	20-scf00019	20
AX-157913906	2	46.636	KV453182.1	5720125	20-scf00019	20
AX-157913907	2	46.636	KV453182.1	5721313	20-scf00019	20
AX-157913909	2	46.636	KV453182.1	5723725	20-scf00019	20
AX-157913911	2	46.636	KV453182.1	5727578	20-scf00019	20
AX-157812533	2	46.636	KV453182.1	5727694	20-scf00019	20
AX-157913912	2	46.636	KV453182.1	5731588	20-scf00019	20
AX-157913915	2	46.636	KV453182.1	5732739	20-scf00019	20
AX-157913917	2	46.636	KV453182.1	5733621	20-scf00019	20
AX-157913918	2	46.636	KV453182.1	5734062	20-scf00019	20
AX-157913920	2	46.636	KV453182.1	5734938	20-scf00019	20
AX-157812534	2	46.636	KV453182.1	5735209	20-scf00019	20
AX-157913921	2	46.636	KV453182.1	5735512	20-scf00019	20
AX-157913923	2	46.636	KV453182.1	5737848	20-scf00019	20
AX-157913924	2	46.636	KV453182.1	5738583	20-scf00019	20
AX-158404975	2	46.636	KV453182.1	5738987	20-scf00019	20
AX-157812535	2	46.636	KV453182.1	5739127	20-scf00019	20
AX-157913926	2	46.636	KV453182.1	5741287	20-scf00019	20
AX-157812538	2	46.636	KV453182.1	5741768	20-scf00019	20
AX-157913927	2	46.636	KV453182.1	5742169	20-scf00019	20
AX-157913928	2	46.636	KV453182.1	5742635	20-scf00019	20
AX-157913931	2	46.636	KV453182.1	5745682	20-scf00019	20
AX-157913933	2	46.636	KV453182.1	5747119	20-scf00019	20
AX-157913934	2	46.636	KV453182.1	5748638	20-scf00019	20
AX-157913935	2	46.636	KV453182.1	5749811	20-scf00019	20
AX-157913937	2	46.636	KV453182.1	5751342	20-scf00019	20
AX-157913938	2	46.636	KV453182.1	5753058	20-scf00019	20
AX-157913939	2	46.636	KV453182.1	5754269	20-scf00019	20
AX-157913942	2	46.636	KV453182.1	5755478	20-scf00019	20
AX-157812539	2	46.636	KV453182.1	5756475	20-scf00019	20
AX-157913944	2	46.636	KV453182.1	5757195	20-scf00019	20
AX-157913945	2	46.636	KV453182.1	5757869	20-scf00019	20
AX-157748075	2	46.636	KV453182.1	5759570	20-scf00019	20
AX-157913946	2	46.636	KV453182.1	5760552	20-scf00019	20
AX-157913947	2	46.636	KV453182.1	5760767	20-scf00019	20
AX-157716884	2	46.636	KV453182.1	5760920	20-scf00019	20
AX-157812540	2	46.636	KV453182.1	5761393	20-scf00019	20
AX-157913948	2	46.636	KV453182.1	5762812	20-scf00019	20
AX-157716738	2	46.636	KV453182.1	5765359	20-scf00019	20

AX-157750528	2	46.636	KV453182.1	5767021	20-scf00019	20
AX-157913951	2	46.636	KV453182.1	5768887	20-scf00019	20
AX-157913953	2	46.636	KV453182.1	5775086	20-scf00019	20
AX-157913954	2	46.636	KV453182.1	5775353	20-scf00019	20
AX-157812544	2	46.636	KV453182.1	5775639	20-scf00019	20
AX-157913956	2	46.636	KV453182.1	5777330	20-scf00019	20
AX-157913961	2	46.636	KV453182.1	5780451	20-scf00019	20
AX-157913963	2	46.636	KV453182.1	5781169	20-scf00019	20
AX-157913967	2	46.636	KV453182.1	5786412	20-scf00019	20
AX-157913968	2	46.636	KV453182.1	5787276	20-scf00019	20
AX-158404977	2	46.636	KV453182.1	5788580	20-scf00019	20
AX-157913969	2	46.636	KV453182.1	5789163	20-scf00019	20
AX-157913970	2	46.636	KV453182.1	5789654	20-scf00019	20
AX-157913971	2	46.636	KV453182.1	5789905	20-scf00019	20
AX-157913974	2	46.636	KV453182.1	5791949	20-scf00019	20
AX-157913976	2	46.636	KV453182.1	5793264	20-scf00019	20
AX-157727814	2	46.636	KV453182.1	5793513	20-scf00019	20
AX-157913977	2	46.636	KV453182.1	5794049	20-scf00019	20
AX-157913982	2	46.636	KV453182.1	5799763	20-scf00019	20
AX-157913984	2	46.636	KV453182.1	5802495	20-scf00019	20
AX-157913985	2	46.636	KV453182.1	5802899	20-scf00019	20
AX-157913986	2	46.636	KV453182.1	5803820	20-scf00019	20
AX-157913988	2	46.636	KV453182.1	5804791	20-scf00019	20
AX-157913990	2	46.636	KV453182.1	5812048	20-scf00019	20
AX-157913991	2	46.636	KV453182.1	5812743	20-scf00019	20
AX-157913992	2	46.636	KV453182.1	5816734	20-scf00019	20
AX-157913994	2	46.636	KV453182.1	5819070	20-scf00019	20
AX-157913995	2	46.636	KV453182.1	5819391	20-scf00019	20
AX-157913996	2	46.636	KV453182.1	5821542	20-scf00019	20
AX-157913997	2	46.636	KV453182.1	5822084	20-scf00019	20
AX-157913998	2	46.636	KV453182.1	5822536	20-scf00019	20
AX-157913999	2	46.636	KV453182.1	5823827	20-scf00019	20
AX-157914000	2	46.636	KV453182.1	5824162	20-scf00019	20
AX-157914002	2	46.636	KV453182.1	5828195	20-scf00019	20
AX-158404979	2	46.636	KV453182.1	5829232	20-scf00019	20
AX-157914006	2	46.636	KV453182.1	5831020	20-scf00019	20
AX-157719608	2	46.636	KV453182.1	5831641	20-scf00019	20
AX-157914008	2	46.636	KV453182.1	5833141	20-scf00019	20
AX-157812546	2	46.636	KV453182.1	5833697	20-scf00019	20
AX-157914009	2	46.636	KV453182.1	5834190	20-scf00019	20
AX-157914010	2	46.636	KV453182.1	5834480	20-scf00019	20
AX-157914012	2	46.636	KV453182.1	5839862	20-scf00019	20
AX-157914014	2	46.636	KV453182.1	5840948	20-scf00019	20
AX-157914015	2	46.636	KV453182.1	5841972	20-scf00019	20
AX-157914016	2	46.636	KV453182.1	5842497	20-scf00019	20
AX-157914017	2	46.636	KV453182.1	5842793	20-scf00019	20
AX-157914018	2	46.636	KV453182.1	5843586	20-scf00019	20
AX-157914019	2	46.636	KV453182.1	5844949	20-scf00019	20
AX-157914020	2	46.636	KV453182.1	5845928	20-scf00019	20
AX-157914021	2	46.636	KV453182.1	5846936	20-scf00019	20
AX-157914022	2	46.636	KV453182.1	5847698	20-scf00019	20

AX-157914023	2	46.636	KV453182.1	5848257	20-scf00019	20
AX-157914024	2	46.636	KV453182.1	5848941	20-scf00019	20
AX-157812549	2	46.636	KV453182.1	5849702	20-scf00019	20
AX-157914025	2	46.636	KV453182.1	5850332	20-scf00019	20
AX-157756648	2	46.636	KV453182.1	5852032	20-scf00019	20
AX-157914027	2	46.636	KV453182.1	5855718	20-scf00019	20
AX-157914028	2	46.636	KV453182.1	5856445	20-scf00019	20
AX-157914029	2	46.636	KV453182.1	5856712	20-scf00019	20
AX-157914031	2	46.636	KV453182.1	5859176	20-scf00019	20
AX-157914033	2	46.636	KV453182.1	5860344	20-scf00019	20
AX-157914034	2	46.636	KV453182.1	5860952	20-scf00019	20
AX-157914035	2	46.636	KV453182.1	5861679	20-scf00019	20
AX-157914036	2	46.636	KV453182.1	5863714	20-scf00019	20
AX-157914038	2	46.636	KV453182.1	5866115	20-scf00019	20
AX-157914039	2	46.636	KV453182.1	5866724	20-scf00019	20
AX-157914040	2	46.636	KV453182.1	5867723	20-scf00019	20
AX-157812554	2	46.636	KV453182.1	5867768	20-scf00019	20
AX-157914041	2	46.636	KV453182.1	5868376	20-scf00019	20
AX-157914042	2	46.636	KV453182.1	5869781	20-scf00019	20
AX-157914043	2	46.636	KV453182.1	5871785	20-scf00019	20
AX-157914044	2	46.636	KV453182.1	5873117	20-scf00019	20
AX-157914048	2	46.636	KV453182.1	5878385	20-scf00019	20
AX-157773414	2	46.636	KV453182.1	5878861	20-scf00019	20
AX-157914049	2	46.636	KV453182.1	5879383	20-scf00019	20
AX-157914050	2	46.636	KV453182.1	5882993	20-scf00019	20
AX-157914055	2	46.636	KV453182.1	5889876	20-scf00019	20
AX-157914056	2	46.636	KV453182.1	5893396	20-scf00019	20
AX-157914057	2	46.636	KV453182.1	5893946	20-scf00019	20
AX-157914058	2	46.636	KV453182.1	5894260	20-scf00019	20
AX-157914059	2	46.636	KV453182.1	5894463	20-scf00019	20
AX-157914060	2	46.636	KV453182.1	5895441	20-scf00019	20
AX-157914063	2	46.636	KV453182.1	5896792	20-scf00019	20
AX-157914064	2	46.636	KV453182.1	5896957	20-scf00019	20
AX-157914066	2	46.636	KV453182.1	5898681	20-scf00019	20
AX-157812558	2	46.636	KV453182.1	5899534	20-scf00019	20
AX-157914067	2	46.636	KV453182.1	5900044	20-scf00019	20
AX-157914069	2	46.636	KV453182.1	5902576	20-scf00019	20
AX-157755791	2	46.636	KV453182.1	5902878	20-scf00019	20
AX-157914070	2	46.636	KV453182.1	5903177	20-scf00019	20
AX-158404985	2	46.636	KV453182.1	5903670	20-scf00019	20
AX-157869095	2	46.636	KV453182.1	5905453	20-scf00019	20
AX-157914072	2	46.636	KV453182.1	5906226	20-scf00019	20
AX-157914073	2	46.636	KV453182.1	5907285	20-scf00019	20
AX-157914074	2	46.636	KV453182.1	5907991	20-scf00019	20
AX-157914075	2	46.636	KV453182.1	5908560	20-scf00019	20
AX-157914076	2	46.636	KV453182.1	5909731	20-scf00019	20
AX-157914077	2	46.636	KV453182.1	5910228	20-scf00019	20
AX-157914081	2	46.636	KV453182.1	5912843	20-scf00019	20
AX-157812559	2	46.636	KV453182.1	5913019	20-scf00019	20
AX-157914082	2	46.636	KV453182.1	5913509	20-scf00019	20
AX-157914083	2	46.636	KV453182.1	5913953	20-scf00019	20

AX-157914084	2	46.636	KV453182.1	5914721	20-scf00019	20
AX-157914085	2	46.636	KV453182.1	5915353	20-scf00019	20
AX-157914086	2	46.636	KV453182.1	5915789	20-scf00019	20
AX-157914087	2	46.636	KV453182.1	5916112	20-scf00019	20
AX-157914089	2	46.636	KV453182.1	5917368	20-scf00019	20
AX-157914090	2	46.636	KV453182.1	5918813	20-scf00019	20
AX-157914091	2	46.636	KV453182.1	5919126	20-scf00019	20
AX-157914092	2	46.636	KV453182.1	5919689	20-scf00019	20
AX-157914093	2	46.636	KV453182.1	5920957	20-scf00019	20
AX-157914094	2	46.636	KV453182.1	5925351	20-scf00019	20
AX-157914095	2	46.636	KV453182.1	5925842	20-scf00019	20
AX-157914099	2	46.636	KV453182.1	5930176	20-scf00019	20
AX-157914100	2	46.636	KV453182.1	5930989	20-scf00019	20
AX-157914101	2	46.636	KV453182.1	5931410	20-scf00019	20
AX-157914102	2	46.636	KV453182.1	5932347	20-scf00019	20
AX-157914103	2	46.636	KV453182.1	5933815	20-scf00019	20
AX-157914104	2	46.636	KV453182.1	5934656	20-scf00019	20
AX-157914106	2	46.636	KV453182.1	5936827	20-scf00019	20
AX-157914107	2	46.636	KV453182.1	5937402	20-scf00019	20
AX-157914108	2	46.636	KV453182.1	5938589	20-scf00019	20
AX-157914109	2	46.636	KV453182.1	5939707	20-scf00019	20
AX-157914112	2	46.636	KV453182.1	5942278	20-scf00019	20
AX-157914114	2	46.636	KV453182.1	5943718	20-scf00019	20
AX-157914115	2	46.636	KV453182.1	5944659	20-scf00019	20
AX-157914116	2	46.636	KV453182.1	5946780	20-scf00019	20
AX-157914117	2	46.636	KV453182.1	5947335	20-scf00019	20
AX-157914118	2	46.636	KV453182.1	5948072	20-scf00019	20
AX-157914120	2	46.636	KV453182.1	5950225	20-scf00019	20
AX-157914124	2	46.636	KV453182.1	5956659	20-scf00019	20
AX-157914125	2	46.636	KV453182.1	5957779	20-scf00019	20
AX-157914126	2	46.636	KV453182.1	5957905	20-scf00019	20
AX-157812560	2	46.636	KV453182.1	5958612	20-scf00019	20
AX-157914128	2	46.636	KV453182.1	5958890	20-scf00019	20
AX-157716225	2	46.636	KV453182.1	5960225	20-scf00019	20
AX-157703700	2	46.636	KV453182.1	5961557	20-scf00019	20
AX-157914129	2	46.636	KV453182.1	5961997	20-scf00019	20
AX-157914130	2	46.636	KV453182.1	5962765	20-scf00019	20
AX-157914132	2	46.636	KV453182.1	5963399	20-scf00019	20
AX-157914134	2	46.636	KV453182.1	5965147	20-scf00019	20
AX-157914136	2	46.636	KV453182.1	5968635	20-scf00019	20
AX-157914137	2	46.636	KV453182.1	5969566	20-scf00019	20
AX-157914138	2	46.636	KV453182.1	5969890	20-scf00019	20
AX-157914139	2	46.636	KV453182.1	5970173	20-scf00019	20
AX-157914140	2	46.636	KV453182.1	5970597	20-scf00019	20
AX-157812562	2	46.636	KV453182.1	5978023	20-scf00019	20
AX-158404986	2	46.636	KV453182.1	5978411	20-scf00019	20
AX-157914148	2	46.636	KV453182.1	5979755	20-scf00019	20
AX-157914150	2	46.636	KV453182.1	5981290	20-scf00019	20
AX-157914151	2	46.636	KV453182.1	5982058	20-scf00019	20
AX-157914152	2	46.636	KV453182.1	5983572	20-scf00019	20
AX-157914153	2	46.636	KV453182.1	5984150	20-scf00019	20

AX-158404987	2	46.636	KV453182.1	5985500	20-scf00019	20
AX-157914154	2	46.636	KV453182.1	5985633	20-scf00019	20
AX-157914157	2	46.636	KV453182.1	5987572	20-scf00019	20
AX-157914158	2	46.636	KV453182.1	5987954	20-scf00019	20
AX-157914161	2	46.636	KV453182.1	5991321	20-scf00019	20
AX-157914164	2	46.636	KV453182.1	5994308	20-scf00019	20
AX-157914166	2	46.636	KV453182.1	5995762	20-scf00019	20
AX-158414802	2	46.636	KV453182.1	5997142	20-scf00019	20
AX-157914168	2	46.636	KV453182.1	5998564	20-scf00019	20
AX-157914170	2	46.636	KV453182.1	6000197	20-scf00019	20
AX-157914173	2	46.636	KV453182.1	6002460	20-scf00019	20
AX-157914174	2	46.636	KV453182.1	6003174	20-scf00019	20
AX-157914175	2	46.636	KV453182.1	6003313	20-scf00019	20
AX-157914178	2	46.636	KV453182.1	6004935	20-scf00019	20
AX-157914180	2	46.636	KV453182.1	6010475	20-scf00019	20
AX-157914181	2	46.636	KV453182.1	6013052	20-scf00019	20
AX-157914182	2	46.636	KV453182.1	6016513	20-scf00019	20
AX-157914188	2	46.636	KV453182.1	6022422	20-scf00019	20
AX-157914192	2	46.636	KV453182.1	6029543	20-scf00019	20
AX-157914205	2	46.636	KV453182.1	6042081	20-scf00019	20
AX-157914209	2	46.636	KV453182.1	6055428	20-scf00019	20
AX-157914218	2	46.636	KV453182.1	6062464	20-scf00019	20
AX-157914221	2	46.636	KV453182.1	6066169	20-scf00019	20
AX-157914224	2	46.636	KV453182.1	6071130	20-scf00019	20
AX-158389596	2	46.636	KV453182.1	6072067	20-scf00019	20
AX-157914225	2	46.636	KV453182.1	6072290	20-scf00019	20
AX-157914226	2	46.636	KV453182.1	6074119	20-scf00019	20
AX-157914227	2	46.636	KV453182.1	6074996	20-scf00019	20
AX-157914229	2	46.636	KV453182.1	6076826	20-scf00019	20
AX-158404989	2	46.636	KV453182.1	6077821	20-scf00019	20
AX-157914237	2	46.636	KV453182.1	6085402	20-scf00019	20
AX-157914239	2	46.636	KV453182.1	6087436	20-scf00019	20
AX-157812567	2	46.636	KV453182.1	6090094	20-scf00019	20
AX-157914240	2	46.636	KV453182.1	6090610	20-scf00019	20
AX-157914241	2	46.636	KV453182.1	6091690	20-scf00019	20
AX-157914242	2	46.636	KV453182.1	6092591	20-scf00019	20
AX-157914244	2	46.636	KV453182.1	6093941	20-scf00019	20
AX-157914246	2	46.636	KV453182.1	6094798	20-scf00019	20
AX-157914247	2	46.636	KV453182.1	6095591	20-scf00019	20
AX-157914248	2	46.636	KV453182.1	6096173	20-scf00019	20
AX-157914249	2	46.636	KV453182.1	6096821	20-scf00019	20
AX-157914250	2	46.636	KV453182.1	6097959	20-scf00019	20
AX-157914251	2	46.636	KV453182.1	6106614	20-scf00019	20
AX-157914255	2	46.636	KV453182.1	6112425	20-scf00019	20
AX-157914262	2	46.636	KV453182.1	6120825	20-scf00019	20
AX-157914264	2	46.636	KV453182.1	6124856	20-scf00019	20
AX-158379255	2	46.636	KV453182.1	6128653	20-scf00019	20
AX-157914266	2	46.636	KV453182.1	6133639	20-scf00019	20
AX-157914267	2	46.636	KV453182.1	6134206	20-scf00019	20
AX-157914271	2	46.636	KV453182.1	6136171	20-scf00019	20
AX-157914272	2	46.636	KV453182.1	6137257	20-scf00019	20

AX-157914276	2	46.636	KV453182.1	6139547	20-scf00019	20
AX-157914278	2	46.636	KV453182.1	6141845	20-scf00019	20
AX-157914279	2	46.636	KV453182.1	6143806	20-scf00019	20
AX-157914282	2	46.636	KV453182.1	6148030	20-scf00019	20
AX-157914283	2	46.636	KV453182.1	6148131	20-scf00019	20
AX-157914285	2	46.636	KV453182.1	6149277	20-scf00019	20
AX-157914286	2	46.636	KV453182.1	6149565	20-scf00019	20
AX-157869098	2	46.636	KV453182.1	6150577	20-scf00019	20
AX-157914288	2	46.636	KV453182.1	6153600	20-scf00019	20
AX-157812572	2	46.636	KV453182.1	6155616	20-scf00019	20
AX-157914289	2	46.636	KV453182.1	6156638	20-scf00019	20
AX-157914290	2	46.636	KV453182.1	6157832	20-scf00019	20
AX-157914292	2	46.636	KV453182.1	6159409	20-scf00019	20
AX-157914293	2	46.636	KV453182.1	6159939	20-scf00019	20
AX-157914294	2	46.636	KV453182.1	6161562	20-scf00019	20
AX-157914295	2	46.636	KV453182.1	6163138	20-scf00019	20
AX-157914296	2	46.636	KV453182.1	6165060	20-scf00019	20
AX-157812573	2	46.636	KV453182.1	6165847	20-scf00019	20
AX-157914297	2	46.636	KV453182.1	6166201	20-scf00019	20
AX-157914298	2	46.636	KV453182.1	6166365	20-scf00019	20
AX-157914300	2	46.636	KV453182.1	6169420	20-scf00019	20
AX-157914308	2	46.636	KV453182.1	6174948	20-scf00019	20
AX-157914309	2	46.636	KV453182.1	6175385	20-scf00019	20
AX-157812574	2	46.636	KV453182.1	6180094	20-scf00019	20
AX-157914313	2	46.636	KV453182.1	6180281	20-scf00019	20
AX-157709432	2	46.636	KV453182.1	6184129	20-scf00019	20
AX-157914316	2	46.636	KV453182.1	6185305	20-scf00019	20
AX-157914317	2	46.636	KV453182.1	6186304	20-scf00019	20
AX-157914318	2	46.636	KV453182.1	6186760	20-scf00019	20
AX-157914322	2	46.636	KV453182.1	6190942	20-scf00019	20
AX-157914323	2	46.636	KV453182.1	6191319	20-scf00019	20
AX-157812576	2	46.636	KV453182.1	6191694	20-scf00019	20
AX-157914324	2	46.636	KV453182.1	6191959	20-scf00019	20
AX-157914326	2	46.636	KV453182.1	6192891	20-scf00019	20
AX-157914327	2	46.636	KV453182.1	6193199	20-scf00019	20
AX-157914328	2	46.636	KV453182.1	6193265	20-scf00019	20
AX-157738801	2	46.636	KV453182.1	6195018	20-scf00019	20
AX-157914331	2	46.636	KV453182.1	6195117	20-scf00019	20
AX-157914332	2	46.636	KV453182.1	6195640	20-scf00019	20
AX-157914334	2	46.636	KV453182.1	6196731	20-scf00019	20
AX-157914338	2	46.636	KV453182.1	6199519	20-scf00019	20
AX-157914340	2	46.636	KV453182.1	6202333	20-scf00019	20
AX-157914341	2	46.636	KV453182.1	6202854	20-scf00019	20
AX-157914343	2	46.636	KV453182.1	6204246	20-scf00019	20
AX-157914344	2	46.636	KV453182.1	6204960	20-scf00019	20
AX-157914345	2	46.636	KV453182.1	6206479	20-scf00019	20
AX-157914346	2	46.636	KV453182.1	6206797	20-scf00019	20
AX-157914347	2	46.636	KV453182.1	6207723	20-scf00019	20
AX-157914350	2	46.636	KV453182.1	6211431	20-scf00019	20
AX-158382250	2	46.636	KV453182.1	6211773	20-scf00019	20
AX-157723671	2	46.636	KV453182.1	6214268	20-scf00019	20

AX-157914352	2	46.636	KV453182.1	6214732	20-scf00019	20
AX-157914353	2	46.636	KV453182.1	6215091	20-scf00019	20
AX-157914354	2	46.636	KV453182.1	6215596	20-scf00019	20
AX-157914355	2	46.636	KV453182.1	6217313	20-scf00019	20
AX-157914356	2	46.636	KV453182.1	6218585	20-scf00019	20
AX-157914357	2	46.636	KV453182.1	6219320	20-scf00019	20
AX-157914358	2	46.636	KV453182.1	6221847	20-scf00019	20
AX-157914359	2	46.636	KV453182.1	6223213	20-scf00019	20
AX-157914362	2	46.636	KV453182.1	6234709	20-scf00019	20
AX-157914430	2	46.636	KV453182.1	6676046	20-scf00019	20
AX-157914460	2	46.636	KV453182.1	6866461	20-scf00019	20
AX-157812583	2	46.636	KV453182.1	6867129	20-scf00019	20
AX-157914467	2	46.636	KV453182.1	6890844	20-scf00019	20
AX-157874664	2	46.636	KV453182.1	6892539	20-scf00019	20
AX-157914472	2	46.636	KV453182.1	6899945	20-scf00019	20
AX-157914475	2	46.636	KV453182.1	6900921	20-scf00019	20
AX-157914478	2	46.636	KV453182.1	6904588	20-scf00019	20
AX-157914499	2	46.636	KV453182.1	6957864	20-scf00019	20
AX-157812591	2	46.636	KV453182.1	6979017	20-scf00019	20
AX-157914550	2	46.636	KV453182.1	7148707	20-scf00019	20
AX-157914557	2	46.636	KV453182.1	7158725	20-scf00019	20
AX-157914560	2	46.636	KV453182.1	7169597	20-scf00019	20
AX-158375388	2	46.636	KV453187.1	161065	20-scf00563	20
AX-158375456	2	46.636	KV453187.1	887468	20-scf00563	20
AX-157920119	2	46.636	KV453183.1	758216	20-scf00571	20
AX-157920140	2	46.636	KV453183.1	817709	20-scf00571	20
AX-157920149	2	46.636	KV453183.1	888010	20-scf00571	20
AX-157726087	2	46.636	KV453183.1	891710	20-scf00571	20
AX-157920154	2	46.636	KV453183.1	904944	20-scf00571	20
AX-157679959	2	46.636	KV453185.1	155635	20-scf00994	20
AX-157685170	2	46.636	KV453185.1	167588	20-scf00994	20
AX-157697809	2	46.636	FI873218.1	157	ORI212 Ictalu	UN
AX-157705834	2	46.636	3ML01031009	86	scf06613_1	UN
AX-157706397	2	46.636	3ML01032184	230	scf07800_1	UN
AX-157913842	2	46.637	KV453182.1	5647522	20-scf00019	20
AX-157913845	2	46.637	KV453182.1	5649242	20-scf00019	20
AX-157913851	2	46.637	KV453182.1	5655948	20-scf00019	20
AX-157913865	2	46.637	KV453182.1	5679345	20-scf00019	20
AX-157913867	2	46.637	KV453182.1	5680616	20-scf00019	20
AX-157913868	2	46.637	KV453182.1	5681755	20-scf00019	20
AX-157913876	2	46.637	KV453182.1	5687457	20-scf00019	20
AX-157913880	2	46.637	KV453182.1	5695352	20-scf00019	20
AX-157774742	2	46.637	KV453182.1	5703171	20-scf00019	20
AX-157913901	2	46.637	KV453182.1	5717154	20-scf00019	20
AX-157913908	2	46.637	KV453182.1	5721462	20-scf00019	20
AX-157913913	2	46.637	KV453182.1	5732280	20-scf00019	20
AX-157913914	2	46.637	KV453182.1	5732617	20-scf00019	20
AX-157913919	2	46.637	KV453182.1	5734564	20-scf00019	20
AX-157913922	2	46.637	KV453182.1	5736689	20-scf00019	20
AX-157913925	2	46.637	KV453182.1	5738920	20-scf00019	20
AX-157812536	2	46.637	KV453182.1	5739551	20-scf00019	20

AX-157913929	2	46.637	KV453182.1	5744226	20-scf00019	20
AX-157913932	2	46.637	KV453182.1	5746760	20-scf00019	20
AX-157913936	2	46.637	KV453182.1	5750598	20-scf00019	20
AX-157913943	2	46.637	KV453182.1	5756670	20-scf00019	20
AX-157812541	2	46.637	KV453182.1	5767127	20-scf00019	20
AX-157812542	2	46.637	KV453182.1	5767490	20-scf00019	20
AX-157913952	2	46.637	KV453182.1	5774388	20-scf00019	20
AX-157913960	2	46.637	KV453182.1	5779068	20-scf00019	20
AX-157913972	2	46.637	KV453182.1	5790112	20-scf00019	20
AX-157913973	2	46.637	KV453182.1	5790792	20-scf00019	20
AX-157714597	2	46.637	KV453182.1	5791102	20-scf00019	20
AX-157913975	2	46.637	KV453182.1	5792850	20-scf00019	20
AX-157913978	2	46.637	KV453182.1	5795718	20-scf00019	20
AX-157913979	2	46.637	KV453182.1	5797284	20-scf00019	20
AX-157747431	2	46.637	KV453182.1	5798388	20-scf00019	20
AX-157913981	2	46.637	KV453182.1	5798600	20-scf00019	20
AX-157913987	2	46.637	KV453182.1	5804435	20-scf00019	20
AX-157913989	2	46.637	KV453182.1	5806038	20-scf00019	20
AX-157913993	2	46.637	KV453182.1	5818837	20-scf00019	20
AX-157740049	2	46.637	KV453182.1	5823132	20-scf00019	20
AX-157914003	2	46.637	KV453182.1	5828750	20-scf00019	20
AX-158404980	2	46.637	KV453182.1	5829430	20-scf00019	20
AX-157914004	2	46.637	KV453182.1	5829789	20-scf00019	20
AX-157914005	2	46.637	KV453182.1	5830474	20-scf00019	20
AX-157914007	2	46.637	KV453182.1	5832624	20-scf00019	20
AX-157812547	2	46.637	KV453182.1	5836200	20-scf00019	20
AX-157812548	2	46.637	KV453182.1	5837597	20-scf00019	20
AX-157914011	2	46.637	KV453182.1	5839354	20-scf00019	20
AX-158389595	2	46.637	KV453182.1	5847642	20-scf00019	20
AX-157812551	2	46.637	KV453182.1	5851096	20-scf00019	20
AX-157914030	2	46.637	KV453182.1	5858073	20-scf00019	20
AX-157914032	2	46.637	KV453182.1	5859608	20-scf00019	20
AX-157914037	2	46.637	KV453182.1	5864759	20-scf00019	20
AX-157812553	2	46.637	KV453182.1	5867462	20-scf00019	20
AX-158404983	2	46.637	KV453182.1	5870713	20-scf00019	20
AX-157914047	2	46.637	KV453182.1	5877682	20-scf00019	20
AX-157724366	2	46.637	KV453182.1	5881487	20-scf00019	20
AX-157914051	2	46.637	KV453182.1	5884313	20-scf00019	20
AX-157914052	2	46.637	KV453182.1	5885049	20-scf00019	20
AX-157812555	2	46.637	KV453182.1	5888047	20-scf00019	20
AX-157812557	2	46.637	KV453182.1	5889410	20-scf00019	20
AX-157715005	2	46.637	KV453182.1	5891535	20-scf00019	20
AX-157914061	2	46.637	KV453182.1	5895724	20-scf00019	20
AX-157914062	2	46.637	KV453182.1	5896216	20-scf00019	20
AX-157914065	2	46.637	KV453182.1	5898054	20-scf00019	20
AX-157914068	2	46.637	KV453182.1	5902004	20-scf00019	20
AX-157914071	2	46.637	KV453182.1	5904959	20-scf00019	20
AX-157914078	2	46.637	KV453182.1	5910653	20-scf00019	20
AX-157914079	2	46.637	KV453182.1	5911653	20-scf00019	20
AX-157914088	2	46.637	KV453182.1	5916614	20-scf00019	20
AX-157914096	2	46.637	KV453182.1	5926351	20-scf00019	20

AX-157914097	2	46.637	KV453182.1	5927602	20-scf00019	20
AX-157914098	2	46.637	KV453182.1	5928892	20-scf00019	20
AX-157914113	2	46.637	KV453182.1	5942856	20-scf00019	20
AX-157914122	2	46.637	KV453182.1	5951512	20-scf00019	20
AX-157914127	2	46.637	KV453182.1	5958287	20-scf00019	20
AX-157717144	2	46.637	KV453182.1	5961742	20-scf00019	20
AX-157914131	2	46.637	KV453182.1	5963084	20-scf00019	20
AX-157869096	2	46.637	KV453182.1	5967687	20-scf00019	20
AX-157914141	2	46.637	KV453182.1	5971403	20-scf00019	20
AX-157914142	2	46.637	KV453182.1	5971845	20-scf00019	20
AX-157914143	2	46.637	KV453182.1	5972891	20-scf00019	20
AX-157914144	2	46.637	KV453182.1	5973479	20-scf00019	20
AX-157914146	2	46.637	KV453182.1	5975292	20-scf00019	20
AX-157914147	2	46.637	KV453182.1	5977472	20-scf00019	20
AX-157812563	2	46.637	KV453182.1	5982439	20-scf00019	20
AX-157914155	2	46.637	KV453182.1	5986186	20-scf00019	20
AX-157914156	2	46.637	KV453182.1	5986964	20-scf00019	20
AX-157914159	2	46.637	KV453182.1	5988818	20-scf00019	20
AX-157914163	2	46.637	KV453182.1	5993158	20-scf00019	20
AX-157914167	2	46.637	KV453182.1	5996468	20-scf00019	20
AX-157914172	2	46.637	KV453182.1	6002172	20-scf00019	20
AX-157752752	2	46.637	KV453182.1	6002510	20-scf00019	20
AX-157812565	2	46.637	KV453182.1	6003622	20-scf00019	20
AX-157914176	2	46.637	KV453182.1	6003992	20-scf00019	20
AX-157914177	2	46.637	KV453182.1	6004412	20-scf00019	20
AX-157914184	2	46.637	KV453182.1	6018522	20-scf00019	20
AX-157914185	2	46.637	KV453182.1	6019083	20-scf00019	20
AX-157869097	2	46.637	KV453182.1	6021074	20-scf00019	20
AX-157914189	2	46.637	KV453182.1	6025196	20-scf00019	20
AX-157914190	2	46.637	KV453182.1	6027386	20-scf00019	20
AX-157914191	2	46.637	KV453182.1	6028884	20-scf00019	20
AX-157914200	2	46.637	KV453182.1	6038162	20-scf00019	20
AX-157914202	2	46.637	KV453182.1	6039062	20-scf00019	20
AX-157812566	2	46.637	KV453182.1	6054953	20-scf00019	20
AX-157914214	2	46.637	KV453182.1	6059446	20-scf00019	20
AX-157914217	2	46.637	KV453182.1	6062062	20-scf00019	20
AX-157914219	2	46.637	KV453182.1	6063890	20-scf00019	20
AX-157914220	2	46.637	KV453182.1	6064644	20-scf00019	20
AX-157914223	2	46.637	KV453182.1	6070918	20-scf00019	20
AX-157750068	2	46.637	KV453182.1	6071733	20-scf00019	20
AX-157914232	2	46.637	KV453182.1	6079037	20-scf00019	20
AX-157914233	2	46.637	KV453182.1	6082199	20-scf00019	20
AX-157914235	2	46.637	KV453182.1	6083074	20-scf00019	20
AX-157914245	2	46.637	KV453182.1	6094248	20-scf00019	20
AX-157914253	2	46.637	KV453182.1	6110224	20-scf00019	20
AX-157914258	2	46.637	KV453182.1	6117835	20-scf00019	20
AX-157914261	2	46.637	KV453182.1	6118961	20-scf00019	20
AX-157715457	2	46.637	KV453182.1	6128251	20-scf00019	20
AX-157914265	2	46.637	KV453182.1	6130368	20-scf00019	20
AX-158404991	2	46.637	KV453182.1	6131432	20-scf00019	20
AX-157914268	2	46.637	KV453182.1	6134478	20-scf00019	20

AX-157914269	2	46.637	KV453182.1	6134991	20-scf00019	20
AX-157914273	2	46.637	KV453182.1	6137477	20-scf00019	20
AX-157914280	2	46.637	KV453182.1	6144527	20-scf00019	20
AX-157914281	2	46.637	KV453182.1	6147198	20-scf00019	20
AX-157914284	2	46.637	KV453182.1	6148840	20-scf00019	20
AX-157914291	2	46.637	KV453182.1	6159151	20-scf00019	20
AX-157914306	2	46.637	KV453182.1	6173620	20-scf00019	20
AX-157914329	2	46.637	KV453182.1	6193573	20-scf00019	20
AX-157914330	2	46.637	KV453182.1	6194302	20-scf00019	20
AX-157914333	2	46.637	KV453182.1	6196308	20-scf00019	20
AX-157914335	2	46.637	KV453182.1	6197275	20-scf00019	20
AX-157914336	2	46.637	KV453182.1	6198238	20-scf00019	20
AX-157914339	2	46.637	KV453182.1	6201080	20-scf00019	20
AX-157914351	2	46.637	KV453182.1	6213118	20-scf00019	20
AX-157914361	2	46.637	KV453182.1	6232667	20-scf00019	20
AX-157914363	2	46.637	KV453182.1	6246205	20-scf00019	20
AX-157914364	2	46.637	KV453182.1	6246657	20-scf00019	20
AX-157914365	2	46.637	KV453182.1	6246769	20-scf00019	20
AX-157812577	2	46.637	KV453182.1	6292467	20-scf00019	20
AX-157914381	2	46.637	KV453182.1	6294330	20-scf00019	20
AX-157914389	2	46.637	KV453182.1	6315751	20-scf00019	20
AX-157914405	2	46.637	KV453182.1	6474043	20-scf00019	20
AX-157914419	2	46.637	KV453182.1	6596355	20-scf00019	20
AX-157914420	2	46.637	KV453182.1	6596889	20-scf00019	20
AX-157914424	2	46.637	KV453182.1	6650813	20-scf00019	20
AX-157914425	2	46.637	KV453182.1	6651805	20-scf00019	20
AX-157914436	2	46.637	KV453182.1	6739213	20-scf00019	20
AX-157914437	2	46.637	KV453182.1	6740338	20-scf00019	20
AX-157914440	2	46.637	KV453182.1	6763009	20-scf00019	20
AX-157914441	2	46.637	KV453182.1	6771933	20-scf00019	20
AX-157812581	2	46.637	KV453182.1	6778662	20-scf00019	20
AX-157914442	2	46.637	KV453182.1	6804226	20-scf00019	20
AX-157914444	2	46.637	KV453182.1	6807265	20-scf00019	20
AX-157914445	2	46.637	KV453182.1	6822174	20-scf00019	20
AX-157914446	2	46.637	KV453182.1	6823881	20-scf00019	20
AX-157914447	2	46.637	KV453182.1	6826283	20-scf00019	20
AX-157914451	2	46.637	KV453182.1	6856092	20-scf00019	20
AX-157914452	2	46.637	KV453182.1	6858368	20-scf00019	20
AX-157914459	2	46.637	KV453182.1	6865959	20-scf00019	20
AX-157914462	2	46.637	KV453182.1	6869330	20-scf00019	20
AX-157812584	2	46.637	KV453182.1	6869413	20-scf00019	20
AX-157812585	2	46.637	KV453182.1	6869558	20-scf00019	20
AX-157914463	2	46.637	KV453182.1	6870142	20-scf00019	20
AX-157883445	2	46.637	KV453182.1	6988474	20-scf00019	20
AX-157914539	2	46.637	KV453182.1	7107179	20-scf00019	20
AX-157914541	2	46.637	KV453182.1	7112124	20-scf00019	20
AX-158414809	2	46.637	KV453182.1	7113176	20-scf00019	20
AX-157914543	2	46.637	KV453182.1	7117235	20-scf00019	20
AX-157914562	2	46.637	KV453182.1	7173777	20-scf00019	20
AX-157914563	2	46.637	KV453182.1	7176225	20-scf00019	20
AX-157914565	2	46.637	KV453182.1	7177497	20-scf00019	20

AX-157914601	2	46.637	KV453182.1	7410766	20-scf00019	20
AX-157914664	2	46.637	KV453189.1	1217935	20-scf00020	20
AX-157914647	2	46.637	KV453189.1	1299282	20-scf00020	20
AX-157853781	2	46.637	KV453189.1	1790791	20-scf00020	20
AX-157914948	2	46.637	KV453193.1	99100	20-scf00021	20
AX-157812668	2	46.637	KV453193.1	1018094	20-scf00021	20
AX-158375397	2	46.637	KV453187.1	316478	20-scf00563	20
AX-158375401	2	46.637	KV453187.1	458049	20-scf00563	20
AX-158375402	2	46.637	KV453187.1	460598	20-scf00563	20
AX-158375403	2	46.637	KV453187.1	464400	20-scf00563	20
AX-158375404	2	46.637	KV453187.1	473702	20-scf00563	20
AX-158375405	2	46.637	KV453187.1	479894	20-scf00563	20
AX-158375406	2	46.637	KV453187.1	483214	20-scf00563	20
AX-158420373	2	46.637	KV453187.1	513924	20-scf00563	20
AX-158375426	2	46.637	KV453187.1	633754	20-scf00563	20
AX-158375428	2	46.637	KV453187.1	635820	20-scf00563	20
AX-158375432	2	46.637	KV453187.1	677808	20-scf00563	20
AX-158375440	2	46.637	KV453187.1	714430	20-scf00563	20
AX-158375457	2	46.637	KV453187.1	898081	20-scf00563	20
AX-157748177	2	46.637	KV453187.1	1029804	20-scf00563	20
AX-158375462	2	46.637	KV453187.1	1029971	20-scf00563	20
AX-158375464	2	46.637	KV453187.1	1035454	20-scf00563	20
AX-158375477	2	46.637	KV453187.1	1204784	20-scf00563	20
AX-157687888	2	46.637	KV453187.1	1265457	20-scf00563	20
AX-157680870	2	46.637	KV453187.1	1369581	20-scf00563	20
AX-158376085	2	46.637	KV453183.1	5064	20-scf00571	20
AX-158376108	2	46.637	KV453183.1	257575	20-scf00571	20
AX-158376111	2	46.637	KV453183.1	288359	20-scf00571	20
AX-158376121	2	46.637	KV453183.1	412422	20-scf00571	20
AX-157864546	2	46.637	KV453183.1	597603	20-scf00571	20
AX-157920091	2	46.637	KV453183.1	633301	20-scf00571	20
AX-157920094	2	46.637	KV453183.1	644744	20-scf00571	20
AX-158403385	2	46.637	KV453183.1	670871	20-scf00571	20
AX-157920103	2	46.637	KV453183.1	693857	20-scf00571	20
AX-157920104	2	46.637	KV453183.1	696101	20-scf00571	20
AX-157920105	2	46.637	KV453183.1	698316	20-scf00571	20
AX-157813128	2	46.637	KV453183.1	699556	20-scf00571	20
AX-157920106	2	46.637	KV453183.1	701067	20-scf00571	20
AX-157920107	2	46.637	KV453183.1	706896	20-scf00571	20
AX-157920109	2	46.637	KV453183.1	729375	20-scf00571	20
AX-157813129	2	46.637	KV453183.1	740869	20-scf00571	20
AX-157716776	2	46.637	KV453183.1	746048	20-scf00571	20
AX-157920113	2	46.637	KV453183.1	747621	20-scf00571	20
AX-157920116	2	46.637	KV453183.1	755758	20-scf00571	20
AX-157920117	2	46.637	KV453183.1	756763	20-scf00571	20
AX-157920118	2	46.637	KV453183.1	757049	20-scf00571	20
AX-157920122	2	46.637	KV453183.1	762537	20-scf00571	20
AX-157920123	2	46.637	KV453183.1	764206	20-scf00571	20
AX-157920125	2	46.637	KV453183.1	774250	20-scf00571	20
AX-157920126	2	46.637	KV453183.1	775219	20-scf00571	20
AX-157920127	2	46.637	KV453183.1	777863	20-scf00571	20

AX-157920128	2	46.637	KV453183.1	784696	20-scf00571	20
AX-157920129	2	46.637	KV453183.1	786765	20-scf00571	20
AX-157920132	2	46.637	KV453183.1	793236	20-scf00571	20
AX-157920135	2	46.637	KV453183.1	800287	20-scf00571	20
AX-157920143	2	46.637	KV453183.1	836615	20-scf00571	20
AX-157920145	2	46.637	KV453183.1	842968	20-scf00571	20
AX-157920146	2	46.637	KV453183.1	869363	20-scf00571	20
AX-157920147	2	46.637	KV453183.1	871717	20-scf00571	20
AX-157920155	2	46.637	KV453183.1	908030	20-scf00571	20
AX-157716174	2	46.637	KV453183.1	908979	20-scf00571	20
AX-157741947	2	46.637	KV453183.1	910520	20-scf00571	20
AX-157920158	2	46.637	KV453183.1	921625	20-scf00571	20
AX-157813135	2	46.637	KV453183.1	926117	20-scf00571	20
AX-158376166	2	46.637	KV453190.1	345966	20-scf00573	20
AX-158376169	2	46.637	KV453190.1	356042	20-scf00573	20
AX-157685024	2	46.637	KV453191.1	93468	20-scf00582	20
AX-158420974	2	46.637	KV453188.1	6907	20-scf00886	20
AX-157685179	2	46.637	KV453185.1	28411	20-scf00994	20
AX-157685177	2	46.637	KV453185.1	65869	20-scf00994	20
AX-157685172	2	46.637	KV453185.1	141854	20-scf00994	20
AX-157685169	2	46.637	KV453185.1	170653	20-scf00994	20
AX-157682201	2	46.637	KV453185.1	254087	20-scf00994	20
AX-157685920	2	46.637	KV453185.1	255164	20-scf00994	20
AX-157685921	2	46.637	KV453185.1	267294	20-scf00994	20
AX-157681178	2	46.637	KV453359.1	48623	scf00584	UN
AX-157704865	2	46.637	3ML01027692	957	scf03298_1	UN
AX-157813132	2	46.789	KV453183.1	885147	20-scf00571	20
AX-157716475	2	46.789	KV453183.1	886390	20-scf00571	20
AX-157920150	2	46.789	KV453183.1	888441	20-scf00571	20
AX-157913674	2	46.812	KV453182.1	5432053	20-scf00019	20
AX-157913689	2	46.812	KV453182.1	5459129	20-scf00019	20
AX-157913777	2	46.812	KV453182.1	5576087	20-scf00019	20
AX-157914429	2	46.812	KV453182.1	6667387	20-scf00019	20
AX-157812594	2	46.812	KV453182.1	7121503	20-scf00019	20
AX-157914545	2	46.812	KV453182.1	7125918	20-scf00019	20
AX-157723318	2	46.812	KV453189.1	2046823	20-scf00020	20
AX-157914926	2	46.812	KV453193.1	64431	20-scf00021	20
AX-157915102	2	46.812	KV453193.1	272008	20-scf00021	20
AX-157915149	2	46.812	KV453193.1	322575	20-scf00021	20
AX-157915178	2	46.812	KV453193.1	350375	20-scf00021	20
AX-157915180	2	46.812	KV453193.1	352502	20-scf00021	20
AX-157915188	2	46.812	KV453193.1	362238	20-scf00021	20
AX-157853721	2	46.812	KV453193.1	437661	20-scf00021	20
AX-157915247	2	46.812	KV453193.1	439902	20-scf00021	20
AX-158375419	2	46.812	KV453187.1	582108	20-scf00563	20
AX-157920096	2	46.812	KV453183.1	651929	20-scf00571	20
AX-157920098	2	46.812	KV453183.1	658261	20-scf00571	20
AX-157813133	2	46.812	KV453183.1	907308	20-scf00571	20
AX-157920160	2	46.812	KV453183.1	938862	20-scf00571	20
AX-157920162	2	46.812	KV453183.1	944320	20-scf00571	20
AX-157914817	2	46.823	KV453189.1	428233	20-scf00020	20

AX-157920489	2	46.823	KV453189.1	1998846	20-scf00020	20
AX-157812612	2	46.823	KV453193.1	70296	20-scf00021	20
AX-157920163	2	46.823	KV453183.1	945080	20-scf00571	20
AX-157920270	2	46.823	KV453183.1	1316318	20-scf00571	20
AX-157920164	2	46.832	KV453183.1	947511	20-scf00571	20
AX-157920203	2	46.832	KV453183.1	1081011	20-scf00571	20
AX-157920206	2	46.846	KV453183.1	1084807	20-scf00571	20
AX-157920209	2	46.861	KV453183.1	1086182	20-scf00571	20
AX-157920214	2	46.876	KV453183.1	1089503	20-scf00571	20
AX-157920369	2	46.891	KV453189.1	1586527	20-scf00020	20
AX-157813144	2	46.891	KV453183.1	1090621	20-scf00571	20
AX-157920223	2	46.912	KV453183.1	1133580	20-scf00571	20
AX-157920293	2	46.931	KV453183.1	1493617	20-scf00571	20
AX-157812500	2	46.942	KV453182.1	5179495	20-scf00019	20
AX-157812521	2	46.942	KV453182.1	5410326	20-scf00019	20
AX-157913735	2	46.942	KV453182.1	5518255	20-scf00019	20
AX-157913782	2	46.942	KV453182.1	5581084	20-scf00019	20
AX-157913783	2	46.942	KV453182.1	5584936	20-scf00019	20
AX-157914274	2	46.942	KV453182.1	6138347	20-scf00019	20
AX-157914315	2	46.942	KV453182.1	6182655	20-scf00019	20
AX-157914374	2	46.942	KV453182.1	6280108	20-scf00019	20
AX-157914383	2	46.942	KV453182.1	6298990	20-scf00019	20
AX-157914407	2	46.942	KV453182.1	6475972	20-scf00019	20
AX-157914414	2	46.942	KV453182.1	6557873	20-scf00019	20
AX-157914428	2	46.942	KV453182.1	6656207	20-scf00019	20
AX-157914498	2	46.942	KV453182.1	6953623	20-scf00019	20
AX-157914511	2	46.942	KV453182.1	7019026	20-scf00019	20
AX-157914514	2	46.942	KV453182.1	7025492	20-scf00019	20
AX-157914570	2	46.942	KV453182.1	7197240	20-scf00019	20
AX-157914580	2	46.942	KV453182.1	7257641	20-scf00019	20
AX-157914892	2	46.942	KV453189.1	6177	20-scf00020	20
AX-157914889	2	46.942	KV453189.1	24699	20-scf00020	20
AX-157914886	2	46.942	KV453189.1	59006	20-scf00020	20
AX-157914883	2	46.942	KV453189.1	74729	20-scf00020	20
AX-157914882	2	46.942	KV453189.1	76419	20-scf00020	20
AX-157914879	2	46.942	KV453189.1	102338	20-scf00020	20
AX-157914878	2	46.942	KV453189.1	119057	20-scf00020	20
AX-157914876	2	46.942	KV453189.1	135460	20-scf00020	20
AX-157914871	2	46.942	KV453189.1	192593	20-scf00020	20
AX-157914869	2	46.942	KV453189.1	211779	20-scf00020	20
AX-157914868	2	46.942	KV453189.1	240483	20-scf00020	20
AX-157914866	2	46.942	KV453189.1	254932	20-scf00020	20
AX-157914864	2	46.942	KV453189.1	257721	20-scf00020	20
AX-157914863	2	46.942	KV453189.1	258458	20-scf00020	20
AX-157914862	2	46.942	KV453189.1	260117	20-scf00020	20
AX-158405010	2	46.942	KV453189.1	265415	20-scf00020	20
AX-157914861	2	46.942	KV453189.1	266509	20-scf00020	20
AX-157914860	2	46.942	KV453189.1	277168	20-scf00020	20
AX-157914859	2	46.942	KV453189.1	277644	20-scf00020	20
AX-157914858	2	46.942	KV453189.1	279859	20-scf00020	20
AX-157914856	2	46.942	KV453189.1	284635	20-scf00020	20

AX-157914855	2	46.942	KV453189.1	286638	20-scf00020	20
AX-157914854	2	46.942	KV453189.1	288632	20-scf00020	20
AX-157914853	2	46.942	KV453189.1	290029	20-scf00020	20
AX-157914852	2	46.942	KV453189.1	290423	20-scf00020	20
AX-157914850	2	46.942	KV453189.1	298215	20-scf00020	20
AX-157914849	2	46.942	KV453189.1	299482	20-scf00020	20
AX-157914848	2	46.942	KV453189.1	300644	20-scf00020	20
AX-157914847	2	46.942	KV453189.1	303891	20-scf00020	20
AX-157914846	2	46.942	KV453189.1	304994	20-scf00020	20
AX-157914844	2	46.942	KV453189.1	328551	20-scf00020	20
AX-157914843	2	46.942	KV453189.1	331238	20-scf00020	20
AX-157914840	2	46.942	KV453189.1	356934	20-scf00020	20
AX-157914839	2	46.942	KV453189.1	361278	20-scf00020	20
AX-157914837	2	46.942	KV453189.1	364806	20-scf00020	20
AX-157914834	2	46.942	KV453189.1	387686	20-scf00020	20
AX-157914833	2	46.942	KV453189.1	388355	20-scf00020	20
AX-157914832	2	46.942	KV453189.1	389343	20-scf00020	20
AX-157914830	2	46.942	KV453189.1	401198	20-scf00020	20
AX-157914829	2	46.942	KV453189.1	402446	20-scf00020	20
AX-158404241	2	46.942	KV453189.1	405262	20-scf00020	20
AX-157914827	2	46.942	KV453189.1	407825	20-scf00020	20
AX-157914826	2	46.942	KV453189.1	408991	20-scf00020	20
AX-157914824	2	46.942	KV453189.1	418377	20-scf00020	20
AX-157914822	2	46.942	KV453189.1	421270	20-scf00020	20
AX-157914820	2	46.942	KV453189.1	424738	20-scf00020	20
AX-157914819	2	46.942	KV453189.1	425440	20-scf00020	20
AX-157914818	2	46.942	KV453189.1	426632	20-scf00020	20
AX-157914816	2	46.942	KV453189.1	431668	20-scf00020	20
AX-157914815	2	46.942	KV453189.1	432735	20-scf00020	20
AX-157914814	2	46.942	KV453189.1	441734	20-scf00020	20
AX-157914813	2	46.942	KV453189.1	444056	20-scf00020	20
AX-157914812	2	46.942	KV453189.1	444953	20-scf00020	20
AX-157914811	2	46.942	KV453189.1	445322	20-scf00020	20
AX-157914810	2	46.942	KV453189.1	456648	20-scf00020	20
AX-157914809	2	46.942	KV453189.1	457415	20-scf00020	20
AX-157914808	2	46.942	KV453189.1	460165	20-scf00020	20
AX-157914807	2	46.942	KV453189.1	460998	20-scf00020	20
AX-157914806	2	46.942	KV453189.1	462766	20-scf00020	20
AX-157914805	2	46.942	KV453189.1	472190	20-scf00020	20
AX-157914804	2	46.942	KV453189.1	480714	20-scf00020	20
AX-157914803	2	46.942	KV453189.1	485086	20-scf00020	20
AX-157914801	2	46.942	KV453189.1	495329	20-scf00020	20
AX-157914799	2	46.942	KV453189.1	497911	20-scf00020	20
AX-157914798	2	46.942	KV453189.1	498380	20-scf00020	20
AX-157914797	2	46.942	KV453189.1	499933	20-scf00020	20
AX-157883448	2	46.942	KV453189.1	500667	20-scf00020	20
AX-157914796	2	46.942	KV453189.1	504290	20-scf00020	20
AX-157914795	2	46.942	KV453189.1	505993	20-scf00020	20
AX-157914794	2	46.942	KV453189.1	508456	20-scf00020	20
AX-157914793	2	46.942	KV453189.1	512704	20-scf00020	20
AX-157742419	2	46.942	KV453189.1	517737	20-scf00020	20

AX-157914792	2	46.942	KV453189.1	521579	20-scf00020	20
AX-157914791	2	46.942	KV453189.1	522005	20-scf00020	20
AX-157725475	2	46.942	KV453189.1	526523	20-scf00020	20
AX-157914788	2	46.942	KV453189.1	527908	20-scf00020	20
AX-157914787	2	46.942	KV453189.1	529716	20-scf00020	20
AX-157914786	2	46.942	KV453189.1	531245	20-scf00020	20
AX-157914785	2	46.942	KV453189.1	533808	20-scf00020	20
AX-157914783	2	46.942	KV453189.1	540259	20-scf00020	20
AX-157914782	2	46.942	KV453189.1	541150	20-scf00020	20
AX-157914781	2	46.942	KV453189.1	544568	20-scf00020	20
AX-157914779	2	46.942	KV453189.1	548899	20-scf00020	20
AX-157914778	2	46.942	KV453189.1	551617	20-scf00020	20
AX-157883447	2	46.942	KV453189.1	556583	20-scf00020	20
AX-157914777	2	46.942	KV453189.1	558113	20-scf00020	20
AX-157914776	2	46.942	KV453189.1	561017	20-scf00020	20
AX-157914775	2	46.942	KV453189.1	574094	20-scf00020	20
AX-157914773	2	46.942	KV453189.1	578900	20-scf00020	20
AX-157914771	2	46.942	KV453189.1	588196	20-scf00020	20
AX-157914770	2	46.942	KV453189.1	590241	20-scf00020	20
AX-157914768	2	46.942	KV453189.1	594241	20-scf00020	20
AX-157914767	2	46.942	KV453189.1	600564	20-scf00020	20
AX-157914766	2	46.942	KV453189.1	602323	20-scf00020	20
AX-157914765	2	46.942	KV453189.1	602458	20-scf00020	20
AX-157914764	2	46.942	KV453189.1	603633	20-scf00020	20
AX-157914763	2	46.942	KV453189.1	609212	20-scf00020	20
AX-157914762	2	46.942	KV453189.1	612903	20-scf00020	20
AX-157914761	2	46.942	KV453189.1	617245	20-scf00020	20
AX-157914760	2	46.942	KV453189.1	619223	20-scf00020	20
AX-157914759	2	46.942	KV453189.1	620061	20-scf00020	20
AX-157914757	2	46.942	KV453189.1	629617	20-scf00020	20
AX-157914754	2	46.942	KV453189.1	637806	20-scf00020	20
AX-157883446	2	46.942	KV453189.1	638957	20-scf00020	20
AX-157914752	2	46.942	KV453189.1	653381	20-scf00020	20
AX-157914750	2	46.942	KV453189.1	657906	20-scf00020	20
AX-157914749	2	46.942	KV453189.1	659213	20-scf00020	20
AX-157914748	2	46.942	KV453189.1	660115	20-scf00020	20
AX-157914747	2	46.942	KV453189.1	665347	20-scf00020	20
AX-157914746	2	46.942	KV453189.1	667137	20-scf00020	20
AX-158405009	2	46.942	KV453189.1	672689	20-scf00020	20
AX-158405008	2	46.942	KV453189.1	674706	20-scf00020	20
AX-157914744	2	46.942	KV453189.1	679509	20-scf00020	20
AX-157914742	2	46.942	KV453189.1	699715	20-scf00020	20
AX-157914741	2	46.942	KV453189.1	701037	20-scf00020	20
AX-157914740	2	46.942	KV453189.1	730594	20-scf00020	20
AX-157874684	2	46.942	KV453189.1	740222	20-scf00020	20
AX-157874683	2	46.942	KV453189.1	751387	20-scf00020	20
AX-157914735	2	46.942	KV453189.1	753325	20-scf00020	20
AX-157914733	2	46.942	KV453189.1	760186	20-scf00020	20
AX-157914731	2	46.942	KV453189.1	764127	20-scf00020	20
AX-157914730	2	46.942	KV453189.1	764924	20-scf00020	20
AX-157914729	2	46.942	KV453189.1	766126	20-scf00020	20

AX-157914726	2	46.942	KV453189.1	782285	20-scf00020	20
AX-157874682	2	46.942	KV453189.1	805875	20-scf00020	20
AX-157914725	2	46.942	KV453189.1	807137	20-scf00020	20
AX-157914724	2	46.942	KV453189.1	810077	20-scf00020	20
AX-157914723	2	46.942	KV453189.1	810694	20-scf00020	20
AX-157914722	2	46.942	KV453189.1	812216	20-scf00020	20
AX-157914717	2	46.942	KV453189.1	836377	20-scf00020	20
AX-157914715	2	46.942	KV453189.1	856762	20-scf00020	20
AX-157914714	2	46.942	KV453189.1	863161	20-scf00020	20
AX-157914712	2	46.942	KV453189.1	876557	20-scf00020	20
AX-157914710	2	46.942	KV453189.1	893007	20-scf00020	20
AX-157914708	2	46.942	KV453189.1	947334	20-scf00020	20
AX-157914706	2	46.942	KV453189.1	955916	20-scf00020	20
AX-157914705	2	46.942	KV453189.1	957261	20-scf00020	20
AX-157751821	2	46.942	KV453189.1	963072	20-scf00020	20
AX-157914704	2	46.942	KV453189.1	973886	20-scf00020	20
AX-157914703	2	46.942	KV453189.1	976448	20-scf00020	20
AX-157914702	2	46.942	KV453189.1	988784	20-scf00020	20
AX-157874681	2	46.942	KV453189.1	1011108	20-scf00020	20
AX-158405007	2	46.942	KV453189.1	1017994	20-scf00020	20
AX-158405006	2	46.942	KV453189.1	1026143	20-scf00020	20
AX-158382256	2	46.942	KV453189.1	1036230	20-scf00020	20
AX-157914698	2	46.942	KV453189.1	1042576	20-scf00020	20
AX-157874679	2	46.942	KV453189.1	1054581	20-scf00020	20
AX-157914695	2	46.942	KV453189.1	1055544	20-scf00020	20
AX-158382255	2	46.942	KV453189.1	1055948	20-scf00020	20
AX-157914694	2	46.942	KV453189.1	1061995	20-scf00020	20
AX-157914693	2	46.942	KV453189.1	1063163	20-scf00020	20
AX-157914691	2	46.942	KV453189.1	1077147	20-scf00020	20
AX-158382254	2	46.942	KV453189.1	1081061	20-scf00020	20
AX-157914690	2	46.942	KV453189.1	1081453	20-scf00020	20
AX-157914688	2	46.942	KV453189.1	1083418	20-scf00020	20
AX-157914687	2	46.942	KV453189.1	1087987	20-scf00020	20
AX-157914684	2	46.942	KV453189.1	1112447	20-scf00020	20
AX-157914683	2	46.942	KV453189.1	1114565	20-scf00020	20
AX-157914681	2	46.942	KV453189.1	1128093	20-scf00020	20
AX-157914679	2	46.942	KV453189.1	1145303	20-scf00020	20
AX-157914676	2	46.942	KV453189.1	1156573	20-scf00020	20
AX-157914673	2	46.942	KV453189.1	1173265	20-scf00020	20
AX-157874678	2	46.942	KV453189.1	1189953	20-scf00020	20
AX-157914670	2	46.942	KV453189.1	1190136	20-scf00020	20
AX-157914669	2	46.942	KV453189.1	1192660	20-scf00020	20
AX-157914668	2	46.942	KV453189.1	1194044	20-scf00020	20
AX-157752965	2	46.942	KV453189.1	1212707	20-scf00020	20
AX-157914666	2	46.942	KV453189.1	1215748	20-scf00020	20
AX-157874677	2	46.942	KV453189.1	1239377	20-scf00020	20
AX-157914663	2	46.942	KV453189.1	1240336	20-scf00020	20
AX-157914662	2	46.942	KV453189.1	1245229	20-scf00020	20
AX-157727342	2	46.942	KV453189.1	1247855	20-scf00020	20
AX-157874676	2	46.942	KV453189.1	1257939	20-scf00020	20
AX-157914660	2	46.942	KV453189.1	1258923	20-scf00020	20

AX-157914658	2	46.942	KV453189.1	1262815	20-scf00020	20
AX-157874675	2	46.942	KV453189.1	1265199	20-scf00020	20
AX-157914656	2	46.942	KV453189.1	1265708	20-scf00020	20
AX-157874674	2	46.942	KV453189.1	1266792	20-scf00020	20
AX-157914654	2	46.942	KV453189.1	1276013	20-scf00020	20
AX-157914653	2	46.942	KV453189.1	1277395	20-scf00020	20
AX-157914649	2	46.942	KV453189.1	1285286	20-scf00020	20
AX-157914646	2	46.942	KV453189.1	1307090	20-scf00020	20
AX-157914640	2	46.942	KV453189.1	1317545	20-scf00020	20
AX-157914639	2	46.942	KV453189.1	1329498	20-scf00020	20
AX-157874672	2	46.942	KV453189.1	1332729	20-scf00020	20
AX-157914636	2	46.942	KV453189.1	1344908	20-scf00020	20
AX-157914635	2	46.942	KV453189.1	1345942	20-scf00020	20
AX-157914634	2	46.942	KV453189.1	1351166	20-scf00020	20
AX-157914633	2	46.942	KV453189.1	1353311	20-scf00020	20
AX-157914632	2	46.942	KV453189.1	1354719	20-scf00020	20
AX-157914629	2	46.942	KV453189.1	1385529	20-scf00020	20
AX-157914628	2	46.942	KV453189.1	1389178	20-scf00020	20
AX-157812603	2	46.942	KV453189.1	1394089	20-scf00020	20
AX-157914626	2	46.942	KV453189.1	1395464	20-scf00020	20
AX-157874670	2	46.942	KV453189.1	1409646	20-scf00020	20
AX-157914621	2	46.942	KV453189.1	1417878	20-scf00020	20
AX-157914616	2	46.942	KV453189.1	1436848	20-scf00020	20
AX-157874668	2	46.942	KV453189.1	1436975	20-scf00020	20
AX-157914615	2	46.942	KV453189.1	1439940	20-scf00020	20
AX-157920346	2	46.942	KV453189.1	1508259	20-scf00020	20
AX-157920348	2	46.942	KV453189.1	1520873	20-scf00020	20
AX-157920355	2	46.942	KV453189.1	1538904	20-scf00020	20
AX-157920356	2	46.942	KV453189.1	1540662	20-scf00020	20
AX-157920359	2	46.942	KV453189.1	1551836	20-scf00020	20
AX-157744186	2	46.942	KV453189.1	1560318	20-scf00020	20
AX-157920361	2	46.942	KV453189.1	1564292	20-scf00020	20
AX-157920365	2	46.942	KV453189.1	1576325	20-scf00020	20
AX-157920370	2	46.942	KV453189.1	1589702	20-scf00020	20
AX-158405224	2	46.942	KV453189.1	1619753	20-scf00020	20
AX-157920380	2	46.942	KV453189.1	1621414	20-scf00020	20
AX-157813163	2	46.942	KV453189.1	1622164	20-scf00020	20
AX-157753717	2	46.942	KV453189.1	1626038	20-scf00020	20
AX-157920381	2	46.942	KV453189.1	1627980	20-scf00020	20
AX-157920382	2	46.942	KV453189.1	1636341	20-scf00020	20
AX-157920385	2	46.942	KV453189.1	1647845	20-scf00020	20
AX-157920392	2	46.942	KV453189.1	1675333	20-scf00020	20
AX-157920395	2	46.942	KV453189.1	1679561	20-scf00020	20
AX-157920397	2	46.942	KV453189.1	1680925	20-scf00020	20
AX-157920409	2	46.942	KV453189.1	1735975	20-scf00020	20
AX-157920411	2	46.942	KV453189.1	1742979	20-scf00020	20
AX-157920412	2	46.942	KV453189.1	1745858	20-scf00020	20
AX-157920414	2	46.942	KV453189.1	1750423	20-scf00020	20
AX-157920417	2	46.942	KV453189.1	1758060	20-scf00020	20
AX-157920418	2	46.942	KV453189.1	1759111	20-scf00020	20
AX-157920426	2	46.942	KV453189.1	1788447	20-scf00020	20

AX-157920432	2	46.942	KV453189.1	1832666	20-scf00020	20
AX-157920442	2	46.942	KV453189.1	1862006	20-scf00020	20
AX-157920447	2	46.942	KV453189.1	1870282	20-scf00020	20
AX-157920448	2	46.942	KV453189.1	1871789	20-scf00020	20
AX-157920455	2	46.942	KV453189.1	1883973	20-scf00020	20
AX-157920483	2	46.942	KV453189.1	1984917	20-scf00020	20
AX-157920484	2	46.942	KV453189.1	1986260	20-scf00020	20
AX-157914899	2	46.942	KV453193.1	17101	20-scf00021	20
AX-157914902	2	46.942	KV453193.1	24733	20-scf00021	20
AX-157812609	2	46.942	KV453193.1	39222	20-scf00021	20
AX-157914916	2	46.942	KV453193.1	53206	20-scf00021	20
AX-157914991	2	46.942	KV453193.1	149375	20-scf00021	20
AX-157915077	2	46.942	KV453193.1	246412	20-scf00021	20
AX-157915095	2	46.942	KV453193.1	264146	20-scf00021	20
AX-157915243	2	46.942	KV453193.1	434983	20-scf00021	20
AX-157915262	2	46.942	KV453193.1	470307	20-scf00021	20
AX-157915267	2	46.942	KV453193.1	489490	20-scf00021	20
AX-157915281	2	46.942	KV453193.1	506940	20-scf00021	20
AX-157915294	2	46.942	KV453193.1	536503	20-scf00021	20
AX-157915307	2	46.942	KV453193.1	551550	20-scf00021	20
AX-157915318	2	46.942	KV453193.1	565496	20-scf00021	20
AX-157915324	2	46.942	KV453193.1	578512	20-scf00021	20
AX-157772040	2	46.942	KV453193.1	592062	20-scf00021	20
AX-157915384	2	46.942	KV453193.1	655493	20-scf00021	20
AX-157915398	2	46.942	KV453193.1	678485	20-scf00021	20
AX-157812650	2	46.942	KV453193.1	709396	20-scf00021	20
AX-157915430	2	46.942	KV453193.1	711546	20-scf00021	20
AX-157915494	2	46.942	KV453193.1	780350	20-scf00021	20
AX-157915569	2	46.942	KV453193.1	943168	20-scf00021	20
AX-157915604	2	46.942	KV453193.1	987786	20-scf00021	20
AX-158405046	2	46.942	KV453193.1	1024410	20-scf00021	20
AX-157915663	2	46.942	KV453193.1	1084556	20-scf00021	20
AX-157682324	2	46.942	KV453186.1	42167	20-scf00024	20
AX-158375412	2	46.942	KV453187.1	520712	20-scf00563	20
AX-158375415	2	46.942	KV453187.1	555942	20-scf00563	20
AX-158375424	2	46.942	KV453187.1	605857	20-scf00563	20
AX-157920114	2	46.942	KV453183.1	748515	20-scf00571	20
AX-157920133	2	46.942	KV453183.1	795109	20-scf00571	20
AX-157920148	2	46.942	KV453183.1	872560	20-scf00571	20
AX-157920165	2	46.942	KV453183.1	949038	20-scf00571	20
AX-157920170	2	46.942	KV453183.1	963793	20-scf00571	20
AX-157920172	2	46.942	KV453183.1	965517	20-scf00571	20
AX-157813138	2	46.942	KV453183.1	973334	20-scf00571	20
AX-157920188	2	46.942	KV453183.1	1032416	20-scf00571	20
AX-157920193	2	46.942	KV453183.1	1053102	20-scf00571	20
AX-157920194	2	46.942	KV453183.1	1056603	20-scf00571	20
AX-157920195	2	46.942	KV453183.1	1058212	20-scf00571	20
AX-157920196	2	46.942	KV453183.1	1065150	20-scf00571	20
AX-157920198	2	46.942	KV453183.1	1071967	20-scf00571	20
AX-157920199	2	46.942	KV453183.1	1073144	20-scf00571	20
AX-157920200	2	46.942	KV453183.1	1074207	20-scf00571	20

AX-157920201	2	46.942	KV453183.1	1074747	20-scf00571	20
AX-157920202	2	46.942	KV453183.1	1075929	20-scf00571	20
AX-157813141	2	46.942	KV453183.1	1076370	20-scf00571	20
AX-157813142	2	46.942	KV453183.1	1078384	20-scf00571	20
AX-157920207	2	46.942	KV453183.1	1085304	20-scf00571	20
AX-157920208	2	46.942	KV453183.1	1085814	20-scf00571	20
AX-157920211	2	46.942	KV453183.1	1087651	20-scf00571	20
AX-157920212	2	46.942	KV453183.1	1087882	20-scf00571	20
AX-157920215	2	46.942	KV453183.1	1090479	20-scf00571	20
AX-157920221	2	46.942	KV453183.1	1105399	20-scf00571	20
AX-157813150	2	46.942	KV453183.1	1167142	20-scf00571	20
AX-157813151	2	46.942	KV453183.1	1175054	20-scf00571	20
AX-157920233	2	46.942	KV453183.1	1182909	20-scf00571	20
AX-157920236	2	46.942	KV453183.1	1196631	20-scf00571	20
AX-157920237	2	46.942	KV453183.1	1205299	20-scf00571	20
AX-157920239	2	46.942	KV453183.1	1212020	20-scf00571	20
AX-157920241	2	46.942	KV453183.1	1218998	20-scf00571	20
AX-157920247	2	46.942	KV453183.1	1235584	20-scf00571	20
AX-157920249	2	46.942	KV453183.1	1243446	20-scf00571	20
AX-157920254	2	46.942	KV453183.1	1252168	20-scf00571	20
AX-157920258	2	46.942	KV453183.1	1264815	20-scf00571	20
AX-157920259	2	46.942	KV453183.1	1266885	20-scf00571	20
AX-157813153	2	46.942	KV453183.1	1268068	20-scf00571	20
AX-157920260	2	46.942	KV453183.1	1276279	20-scf00571	20
AX-157920263	2	46.942	KV453183.1	1284190	20-scf00571	20
AX-157920265	2	46.942	KV453183.1	1296052	20-scf00571	20
AX-157920266	2	46.942	KV453183.1	1296326	20-scf00571	20
AX-157920268	2	46.942	KV453183.1	1301293	20-scf00571	20
AX-157723246	2	46.942	KV453183.1	1311610	20-scf00571	20
AX-157920283	2	46.942	KV453183.1	1374699	20-scf00571	20
AX-157920284	2	46.942	KV453183.1	1386219	20-scf00571	20
AX-157883527	2	46.942	KV453183.1	1414565	20-scf00571	20
AX-157883528	2	46.942	KV453183.1	1467574	20-scf00571	20
AX-157920289	2	46.942	KV453183.1	1469300	20-scf00571	20
AX-157920290	2	46.942	KV453183.1	1469829	20-scf00571	20
AX-157920291	2	46.942	KV453183.1	1470777	20-scf00571	20
AX-157920294	2	46.942	KV453183.1	1494424	20-scf00571	20
AX-157727968	2	46.942	KV453183.1	1502138	20-scf00571	20
AX-157718462	2	46.942	KV453183.1	1502491	20-scf00571	20
AX-157920297	2	46.942	KV453183.1	1514531	20-scf00571	20
AX-157883530	2	46.942	KV453183.1	1529723	20-scf00571	20
AX-157920302	2	46.942	KV453183.1	1538029	20-scf00571	20
AX-157920303	2	46.942	KV453183.1	1540729	20-scf00571	20
AX-157920305	2	46.942	KV453183.1	1571133	20-scf00571	20
AX-157920306	2	46.942	KV453183.1	1578413	20-scf00571	20
AX-157883531	2	46.942	KV453183.1	1596264	20-scf00571	20
AX-157920310	2	46.942	KV453183.1	1599771	20-scf00571	20
AX-157920311	2	46.942	KV453183.1	1605859	20-scf00571	20
AX-157883533	2	46.942	KV453183.1	1616747	20-scf00571	20
AX-157920315	2	46.942	KV453183.1	1663735	20-scf00571	20
AX-157920316	2	46.942	KV453183.1	1676866	20-scf00571	20

AX-157920317	2	46.942	KV453183.1	1680577	20-scf00571	20
AX-157920318	2	46.942	KV453183.1	1685787	20-scf00571	20
AX-157920320	2	46.942	KV453183.1	1705160	20-scf00571	20
AX-157920321	2	46.942	KV453183.1	1707548	20-scf00571	20
AX-157920322	2	46.942	KV453183.1	1722671	20-scf00571	20
AX-157920323	2	46.942	KV453183.1	1725855	20-scf00571	20
AX-157883536	2	46.942	KV453183.1	1742086	20-scf00571	20
AX-158377164	2	46.942	KV453184.1	32349	20-scf00601	20
AX-158377168	2	46.942	KV453184.1	60764	20-scf00601	20
AX-157686610	2	46.942	KV453409.1	31660	scf00709	UN
AX-157686611	2	46.942	KV453409.1	32719	scf00709	UN
AX-157704366	2	46.942	3ML01025786	1083	scf01402_1	UN
AX-157705259	2	46.942	3ML01029205	1024	scf04826_1	UN
AX-157920451	2	47.095	KV453189.1	1879605	20-scf00020	20
AX-157914555	2	47.246	KV453182.1	7155786	20-scf00019	20
AX-158405019	2	47.246	KV453193.1	130294	20-scf00021	20
AX-157913430	2	47.247	KV453182.1	5096055	20-scf00019	20
AX-157913718	2	47.247	KV453182.1	5497767	20-scf00019	20
AX-157913765	2	47.247	KV453182.1	5562912	20-scf00019	20
AX-157913856	2	47.247	KV453182.1	5662641	20-scf00019	20
AX-157914236	2	47.247	KV453182.1	6084059	20-scf00019	20
AX-157914372	2	47.247	KV453182.1	6266861	20-scf00019	20
AX-157914431	2	47.247	KV453182.1	6692106	20-scf00019	20
AX-157914453	2	47.247	KV453182.1	6859783	20-scf00019	20
AX-157914474	2	47.247	KV453182.1	6900617	20-scf00019	20
AX-157914535	2	47.247	KV453182.1	7085101	20-scf00019	20
AX-157914536	2	47.247	KV453182.1	7086621	20-scf00019	20
AX-157914552	2	47.247	KV453182.1	7149868	20-scf00019	20
AX-157914577	2	47.247	KV453182.1	7233262	20-scf00019	20
AX-157920386	2	47.247	KV453189.1	1653249	20-scf00020	20
AX-157813164	2	47.247	KV453189.1	1671815	20-scf00020	20
AX-157915020	2	47.247	KV453193.1	186339	20-scf00021	20
AX-157915033	2	47.247	KV453193.1	200697	20-scf00021	20
AX-157883451	2	47.247	KV453193.1	295956	20-scf00021	20
AX-157915339	2	47.247	KV453193.1	593465	20-scf00021	20
AX-157746117	2	47.247	KV453193.1	635841	20-scf00021	20
AX-157915377	2	47.247	KV453193.1	644821	20-scf00021	20
AX-157915796	2	47.247	KV453193.1	1261124	20-scf00021	20
AX-157682317	2	47.247	KV453186.1	4626	20-scf00024	20
AX-158375399	2	47.247	KV453187.1	421624	20-scf00563	20
AX-158375411	2	47.247	KV453187.1	516877	20-scf00563	20
AX-158375414	2	47.247	KV453187.1	555041	20-scf00563	20
AX-158375435	2	47.247	KV453187.1	694590	20-scf00563	20
AX-157853229	2	47.247	KV453187.1	710906	20-scf00563	20
AX-158375442	2	47.247	KV453187.1	752403	20-scf00563	20
AX-158375458	2	47.247	KV453187.1	917997	20-scf00563	20
AX-158375463	2	47.247	KV453187.1	1031489	20-scf00563	20
AX-158375465	2	47.247	KV453187.1	1049960	20-scf00563	20
AX-158375466	2	47.247	KV453187.1	1055674	20-scf00563	20
AX-158375478	2	47.247	KV453187.1	1208657	20-scf00563	20
AX-157687889	2	47.247	KV453187.1	1269031	20-scf00563	20

AX-158376092	2	47.247	KV453183.1	92751	20-scf00571	20
AX-158376103	2	47.247	KV453183.1	184655	20-scf00571	20
AX-158376104	2	47.247	KV453183.1	196068	20-scf00571	20
AX-158376106	2	47.247	KV453183.1	251669	20-scf00571	20
AX-158376133	2	47.247	KV453183.1	538350	20-scf00571	20
AX-157920088	2	47.247	KV453183.1	614876	20-scf00571	20
AX-157920089	2	47.247	KV453183.1	624805	20-scf00571	20
AX-157920093	2	47.247	KV453183.1	638342	20-scf00571	20
AX-157920099	2	47.247	KV453183.1	660512	20-scf00571	20
AX-157920100	2	47.247	KV453183.1	668344	20-scf00571	20
AX-157920151	2	47.247	KV453183.1	892146	20-scf00571	20
AX-158376141	2	47.247	KV453190.1	89060	20-scf00573	20
AX-158376149	2	47.247	KV453190.1	146237	20-scf00573	20
AX-158376152	2	47.247	KV453190.1	173383	20-scf00573	20
AX-157746392	2	47.247	KV453190.1	262312	20-scf00573	20
AX-158376165	2	47.247	KV453190.1	342580	20-scf00573	20
AX-158376167	2	47.247	KV453190.1	346807	20-scf00573	20
AX-158376168	2	47.247	KV453190.1	355185	20-scf00573	20
AX-158376172	2	47.247	KV453190.1	369478	20-scf00573	20
AX-158376178	2	47.247	KV453190.1	411945	20-scf00573	20
AX-157685018	2	47.247	KV453191.1	27152	20-scf00582	20
AX-157685020	2	47.247	KV453191.1	34516	20-scf00582	20
AX-157685026	2	47.247	KV453191.1	100767	20-scf00582	20
AX-157685032	2	47.247	KV453191.1	168644	20-scf00582	20
AX-157685045	2	47.247	KV453191.1	255762	20-scf00582	20
AX-157685367	2	47.247	KV453188.1	25819	20-scf00886	20
AX-157685375	2	47.247	KV453188.1	124505	20-scf00886	20
AX-157685376	2	47.247	KV453188.1	126929	20-scf00886	20
AX-157685377	2	47.247	KV453188.1	129952	20-scf00886	20
AX-157685379	2	47.247	KV453188.1	155769	20-scf00886	20
AX-157685173	2	47.247	KV453185.1	138406	20-scf00994	20
AX-157685918	2	47.247	KV453185.1	248553	20-scf00994	20
AX-157685065	2	47.247	KV453359.1	62686	scf00584	UN
AX-157685081	2	47.247	KV453359.1	233990	scf00584	UN
AX-157685083	2	47.247	KV453359.1	248652	scf00584	UN
AX-157685102	2	47.247	KV453360.1	190342	scf00585	UN
AX-157913592	2	47.551	KV453182.1	5286973	20-scf00019	20
AX-157913748	2	47.551	KV453182.1	5538660	20-scf00019	20
AX-157914394	2	47.551	KV453182.1	6364142	20-scf00019	20
AX-157914397	2	47.551	KV453182.1	6386624	20-scf00019	20
AX-157914587	2	47.551	KV453182.1	7337856	20-scf00019	20
AX-157914592	2	47.551	KV453182.1	7374826	20-scf00019	20
AX-157914772	2	47.551	KV453189.1	580738	20-scf00020	20
AX-157914739	2	47.551	KV453189.1	738346	20-scf00020	20
AX-157914614	2	47.551	KV453189.1	1441027	20-scf00020	20
AX-157920353	2	47.551	KV453189.1	1537295	20-scf00020	20
AX-157920358	2	47.551	KV453189.1	1549293	20-scf00020	20
AX-157920374	2	47.551	KV453189.1	1597748	20-scf00020	20
AX-157920384	2	47.551	KV453189.1	1643021	20-scf00020	20
AX-157920410	2	47.551	KV453189.1	1739107	20-scf00020	20
AX-157920420	2	47.551	KV453189.1	1768783	20-scf00020	20

AX-157694501	2	47.551	KV453189.1	1799953	20-scf00020	20
AX-157920430	2	47.551	KV453189.1	1811645	20-scf00020	20
AX-157883542	2	47.551	KV453189.1	1833382	20-scf00020	20
AX-157920438	2	47.551	KV453189.1	1854902	20-scf00020	20
AX-157920452	2	47.551	KV453189.1	1880241	20-scf00020	20
AX-157920453	2	47.551	KV453189.1	1881679	20-scf00020	20
AX-157813172	2	47.551	KV453189.1	1882310	20-scf00020	20
AX-157920454	2	47.551	KV453189.1	1883190	20-scf00020	20
AX-157920457	2	47.551	KV453189.1	1888894	20-scf00020	20
AX-157920469	2	47.551	KV453189.1	1929977	20-scf00020	20
AX-157813175	2	47.551	KV453189.1	1959082	20-scf00020	20
AX-157813177	2	47.551	KV453189.1	1968705	20-scf00020	20
AX-157760127	2	47.551	KV453189.1	1978743	20-scf00020	20
AX-157920523	2	47.551	KV453189.1	2113881	20-scf00020	20
AX-157914913	2	47.551	KV453193.1	51522	20-scf00021	20
AX-157914919	2	47.551	KV453193.1	57241	20-scf00021	20
AX-157914921	2	47.551	KV453193.1	61431	20-scf00021	20
AX-157914922	2	47.551	KV453193.1	61772	20-scf00021	20
AX-157914923	2	47.551	KV453193.1	62176	20-scf00021	20
AX-157914924	2	47.551	KV453193.1	62418	20-scf00021	20
AX-157914925	2	47.551	KV453193.1	62674	20-scf00021	20
AX-157914929	2	47.551	KV453193.1	69050	20-scf00021	20
AX-157812613	2	47.551	KV453193.1	71830	20-scf00021	20
AX-157914939	2	47.551	KV453193.1	82312	20-scf00021	20
AX-157914940	2	47.551	KV453193.1	83114	20-scf00021	20
AX-157914941	2	47.551	KV453193.1	83554	20-scf00021	20
AX-158389598	2	47.551	KV453193.1	84633	20-scf00021	20
AX-157914947	2	47.551	KV453193.1	98347	20-scf00021	20
AX-157914949	2	47.551	KV453193.1	100713	20-scf00021	20
AX-157914951	2	47.551	KV453193.1	101743	20-scf00021	20
AX-157914961	2	47.551	KV453193.1	109018	20-scf00021	20
AX-157914963	2	47.551	KV453193.1	110604	20-scf00021	20
AX-157914964	2	47.551	KV453193.1	111509	20-scf00021	20
AX-157914971	2	47.551	KV453193.1	119968	20-scf00021	20
AX-157914976	2	47.551	KV453193.1	125616	20-scf00021	20
AX-157914980	2	47.551	KV453193.1	129079	20-scf00021	20
AX-157914981	2	47.551	KV453193.1	129585	20-scf00021	20
AX-158405018	2	47.551	KV453193.1	129822	20-scf00021	20
AX-157914982	2	47.551	KV453193.1	130475	20-scf00021	20
AX-157914983	2	47.551	KV453193.1	131265	20-scf00021	20
AX-158405024	2	47.551	KV453193.1	148368	20-scf00021	20
AX-157915004	2	47.551	KV453193.1	167930	20-scf00021	20
AX-157915006	2	47.551	KV453193.1	169687	20-scf00021	20
AX-157915007	2	47.551	KV453193.1	170343	20-scf00021	20
AX-157915009	2	47.551	KV453193.1	172190	20-scf00021	20
AX-157915010	2	47.551	KV453193.1	173180	20-scf00021	20
AX-157915080	2	47.551	KV453193.1	248491	20-scf00021	20
AX-157915084	2	47.551	KV453193.1	252977	20-scf00021	20
AX-157915103	2	47.551	KV453193.1	272221	20-scf00021	20
AX-157915114	2	47.551	KV453193.1	286273	20-scf00021	20
AX-157812628	2	47.551	KV453193.1	287687	20-scf00021	20

AX-157915117	2	47.551	KV453193.1	290147	20-scf00021	20
AX-157812629	2	47.551	KV453193.1	290456	20-scf00021	20
AX-157915122	2	47.551	KV453193.1	295701	20-scf00021	20
AX-157915129	2	47.551	KV453193.1	302253	20-scf00021	20
AX-157915130	2	47.551	KV453193.1	302609	20-scf00021	20
AX-157915132	2	47.551	KV453193.1	303214	20-scf00021	20
AX-157915135	2	47.551	KV453193.1	310618	20-scf00021	20
AX-157915137	2	47.551	KV453193.1	312135	20-scf00021	20
AX-157915147	2	47.551	KV453193.1	321487	20-scf00021	20
AX-158405029	2	47.551	KV453193.1	333144	20-scf00021	20
AX-157915187	2	47.551	KV453193.1	361878	20-scf00021	20
AX-157915189	2	47.551	KV453193.1	364302	20-scf00021	20
AX-157757975	2	47.551	KV453193.1	368402	20-scf00021	20
AX-157915195	2	47.551	KV453193.1	376147	20-scf00021	20
AX-157915220	2	47.551	KV453193.1	402801	20-scf00021	20
AX-157915228	2	47.551	KV453193.1	408431	20-scf00021	20
AX-157915232	2	47.551	KV453193.1	412328	20-scf00021	20
AX-157915235	2	47.551	KV453193.1	421150	20-scf00021	20
AX-157915236	2	47.551	KV453193.1	423921	20-scf00021	20
AX-157915240	2	47.551	KV453193.1	430879	20-scf00021	20
AX-157915242	2	47.551	KV453193.1	432381	20-scf00021	20
AX-157915244	2	47.551	KV453193.1	435520	20-scf00021	20
AX-157915245	2	47.551	KV453193.1	436410	20-scf00021	20
AX-157749240	2	47.551	KV453193.1	450606	20-scf00021	20
AX-157812638	2	47.551	KV453193.1	456886	20-scf00021	20
AX-157915256	2	47.551	KV453193.1	463467	20-scf00021	20
AX-157812639	2	47.551	KV453193.1	464218	20-scf00021	20
AX-157915257	2	47.551	KV453193.1	464486	20-scf00021	20
AX-157915264	2	47.551	KV453193.1	485948	20-scf00021	20
AX-157812642	2	47.551	KV453193.1	585575	20-scf00021	20
AX-157874691	2	47.551	KV453193.1	594759	20-scf00021	20
AX-157812644	2	47.551	KV453193.1	603608	20-scf00021	20
AX-157915364	2	47.551	KV453193.1	625774	20-scf00021	20
AX-157915370	2	47.551	KV453193.1	633064	20-scf00021	20
AX-157740863	2	47.551	KV453193.1	658460	20-scf00021	20
AX-157915389	2	47.551	KV453193.1	666122	20-scf00021	20
AX-157915427	2	47.551	KV453193.1	710001	20-scf00021	20
AX-157915457	2	47.551	KV453193.1	744239	20-scf00021	20
AX-157915477	2	47.551	KV453193.1	766844	20-scf00021	20
AX-158405035	2	47.551	KV453193.1	828551	20-scf00021	20
AX-157915528	2	47.551	KV453193.1	894094	20-scf00021	20
AX-157915551	2	47.551	KV453193.1	918300	20-scf00021	20
AX-157915561	2	47.551	KV453193.1	935701	20-scf00021	20
AX-157915565	2	47.551	KV453193.1	939938	20-scf00021	20
AX-157915610	2	47.551	KV453193.1	998336	20-scf00021	20
AX-157915611	2	47.551	KV453193.1	999972	20-scf00021	20
AX-157915613	2	47.551	KV453193.1	1001569	20-scf00021	20
AX-157915615	2	47.551	KV453193.1	1005079	20-scf00021	20
AX-157915623	2	47.551	KV453193.1	1017758	20-scf00021	20
AX-157915626	2	47.551	KV453193.1	1022125	20-scf00021	20
AX-157915652	2	47.551	KV453193.1	1062222	20-scf00021	20

AX-157915668	2	47.551	KV453193.1	1095109	20-scf00021	20
AX-157915674	2	47.551	KV453193.1	1099670	20-scf00021	20
AX-157915682	2	47.551	KV453193.1	1112380	20-scf00021	20
AX-157753544	2	47.551	KV453193.1	1118827	20-scf00021	20
AX-157915758	2	47.551	KV453193.1	1204652	20-scf00021	20
AX-157915777	2	47.551	KV453193.1	1231464	20-scf00021	20
AX-157915990	2	47.551	KV453193.1	1496051	20-scf00021	20
AX-157915991	2	47.551	KV453193.1	1499644	20-scf00021	20
AX-157709950	2	47.551	KV453187.1	785190	20-scf00563	20
AX-157920144	2	47.551	KV453183.1	837806	20-scf00571	20
AX-157920231	2	47.551	KV453183.1	1165538	20-scf00571	20
AX-157920246	2	47.551	KV453183.1	1234890	20-scf00571	20
AX-157920256	2	47.551	KV453183.1	1259230	20-scf00571	20
AX-158376181	2	47.551	KV453190.1	460470	20-scf00573	20
AX-157689772	2	47.551	3ML01032180	276	scf07796_1	UN
AX-157914906	2	47.583	KV453193.1	36009	20-scf00021	20
AX-157915260	2	47.583	KV453193.1	468438	20-scf00021	20
AX-157920095	2	47.583	KV453183.1	648226	20-scf00571	20
AX-157685029	2	47.583	KV453191.1	133343	20-scf00582	20
AX-157920388	2	47.616	KV453189.1	1659303	20-scf00020	20
AX-157920479	2	47.616	KV453189.1	1974469	20-scf00020	20
AX-157920500	2	47.616	KV453189.1	2018077	20-scf00020	20
AX-157914928	2	47.616	KV453193.1	68745	20-scf00021	20
AX-157915101	2	47.616	KV453193.1	270499	20-scf00021	20
AX-157915261	2	47.616	KV453193.1	469674	20-scf00021	20
AX-157915269	2	47.616	KV453193.1	491677	20-scf00021	20
AX-157915291	2	47.649	KV453193.1	532134	20-scf00021	20
AX-157915456	2	47.649	KV453193.1	743287	20-scf00021	20
AX-157914320	2	47.696	KV453182.1	6190403	20-scf00019	20
AX-157914485	2	47.696	KV453182.1	6911357	20-scf00019	20
AX-158376091	2	47.696	KV453183.1	67989	20-scf00571	20
AX-157920157	2	47.696	KV453183.1	919615	20-scf00571	20
AX-157685034	2	47.696	KV453191.1	181939	20-scf00582	20
AX-157685058	2	47.696	KV453359.1	39338	scf00584	UN
AX-157685060	2	47.696	KV453359.1	45069	scf00584	UN
AX-157685107	2	47.696	KV453360.1	284023	scf00585	UN
AX-157913739	2	47.993	KV453182.1	5525301	20-scf00019	20
AX-157914187	2	47.993	KV453182.1	6021718	20-scf00019	20
AX-157914195	2	47.993	KV453182.1	6031968	20-scf00019	20
AX-157914196	2	47.993	KV453182.1	6032526	20-scf00019	20
AX-157771590	2	47.993	KV453182.1	6040146	20-scf00019	20
AX-157914203	2	47.993	KV453182.1	6040455	20-scf00019	20
AX-157914222	2	47.993	KV453182.1	6068332	20-scf00019	20
AX-157914484	2	47.993	KV453182.1	6910977	20-scf00019	20
AX-157914504	2	47.993	KV453182.1	6980734	20-scf00019	20
AX-157914517	2	47.993	KV453182.1	7035383	20-scf00019	20
AX-157914525	2	47.993	KV453182.1	7059782	20-scf00019	20
AX-157914529	2	47.993	KV453182.1	7066903	20-scf00019	20
AX-157914534	2	47.993	KV453182.1	7083652	20-scf00019	20
AX-157914537	2	47.993	KV453182.1	7087405	20-scf00019	20
AX-157914538	2	47.993	KV453182.1	7093532	20-scf00019	20

AX-157914561	2	47.993	KV453182.1	7170345	20-scf00019	20
AX-157914567	2	47.993	KV453182.1	7188854	20-scf00019	20
AX-157914568	2	47.993	KV453182.1	7196002	20-scf00019	20
AX-157914569	2	47.993	KV453182.1	7196758	20-scf00019	20
AX-157914571	2	47.993	KV453182.1	7204737	20-scf00019	20
AX-157914575	2	47.993	KV453182.1	7222738	20-scf00019	20
AX-158404224	2	47.993	KV453182.1	7238780	20-scf00019	20
AX-158389597	2	47.993	KV453182.1	7359333	20-scf00019	20
AX-157914590	2	47.993	KV453182.1	7361794	20-scf00019	20
AX-157914596	2	47.993	KV453182.1	7395581	20-scf00019	20
AX-157914599	2	47.993	KV453182.1	7401425	20-scf00019	20
AX-157914600	2	47.993	KV453182.1	7406451	20-scf00019	20
AX-157914881	2	47.993	KV453189.1	89957	20-scf00020	20
AX-157914835	2	47.993	KV453189.1	376953	20-scf00020	20
AX-157914789	2	47.993	KV453189.1	527191	20-scf00020	20
AX-157914769	2	47.993	KV453189.1	592617	20-scf00020	20
AX-157914758	2	47.993	KV453189.1	629226	20-scf00020	20
AX-157914745	2	47.993	KV453189.1	678615	20-scf00020	20
AX-157914736	2	47.993	KV453189.1	751161	20-scf00020	20
AX-157914734	2	47.993	KV453189.1	756330	20-scf00020	20
AX-157914732	2	47.993	KV453189.1	763392	20-scf00020	20
AX-157914728	2	47.993	KV453189.1	769061	20-scf00020	20
AX-157914727	2	47.993	KV453189.1	778596	20-scf00020	20
AX-157914719	2	47.993	KV453189.1	828556	20-scf00020	20
AX-157914713	2	47.993	KV453189.1	873032	20-scf00020	20
AX-157914699	2	47.993	KV453189.1	1029314	20-scf00020	20
AX-157812606	2	47.993	KV453189.1	1136574	20-scf00020	20
AX-157914674	2	47.993	KV453189.1	1168024	20-scf00020	20
AX-157914672	2	47.993	KV453189.1	1179118	20-scf00020	20
AX-157914641	2	47.993	KV453189.1	1317177	20-scf00020	20
AX-157771518	2	47.993	KV453189.1	1538185	20-scf00020	20
AX-157920379	2	47.993	KV453189.1	1619148	20-scf00020	20
AX-157920415	2	47.993	KV453189.1	1755329	20-scf00020	20
AX-158405227	2	47.993	KV453189.1	1767616	20-scf00020	20
AX-157920422	2	47.993	KV453189.1	1782330	20-scf00020	20
AX-157920427	2	47.993	KV453189.1	1794947	20-scf00020	20
AX-157920436	2	47.993	KV453189.1	1846340	20-scf00020	20
AX-157920437	2	47.993	KV453189.1	1849252	20-scf00020	20
AX-157920441	2	47.993	KV453189.1	1860029	20-scf00020	20
AX-157813171	2	47.993	KV453189.1	1869530	20-scf00020	20
AX-157920460	2	47.993	KV453189.1	1899576	20-scf00020	20
AX-157920461	2	47.993	KV453189.1	1908143	20-scf00020	20
AX-157920465	2	47.993	KV453189.1	1917948	20-scf00020	20
AX-157920466	2	47.993	KV453189.1	1919759	20-scf00020	20
AX-157813173	2	47.993	KV453189.1	1943017	20-scf00020	20
AX-157920474	2	47.993	KV453189.1	1944100	20-scf00020	20
AX-157749101	2	47.993	KV453189.1	1951068	20-scf00020	20
AX-157920475	2	47.993	KV453189.1	1955774	20-scf00020	20
AX-157813174	2	47.993	KV453189.1	1956619	20-scf00020	20
AX-157920476	2	47.993	KV453189.1	1959485	20-scf00020	20
AX-157920478	2	47.993	KV453189.1	1963479	20-scf00020	20

AX-157813176	2	47.993	KV453189.1	1964578	20-scf00020	20
AX-157920491	2	47.993	KV453189.1	1999447	20-scf00020	20
AX-157812610	2	47.993	KV453193.1	45138	20-scf00021	20
AX-157914914	2	47.993	KV453193.1	52189	20-scf00021	20
AX-157812614	2	47.993	KV453193.1	78594	20-scf00021	20
AX-157914937	2	47.993	KV453193.1	78849	20-scf00021	20
AX-157914938	2	47.993	KV453193.1	80707	20-scf00021	20
AX-157914944	2	47.993	KV453193.1	93932	20-scf00021	20
AX-157914970	2	47.993	KV453193.1	117025	20-scf00021	20
AX-157914978	2	47.993	KV453193.1	127345	20-scf00021	20
AX-157914987	2	47.993	KV453193.1	141206	20-scf00021	20
AX-157915081	2	47.993	KV453193.1	249694	20-scf00021	20
AX-157915148	2	47.993	KV453193.1	322045	20-scf00021	20
AX-157915305	2	47.993	KV453193.1	548164	20-scf00021	20
AX-157915326	2	47.993	KV453193.1	579668	20-scf00021	20
AX-157915328	2	47.993	KV453193.1	582417	20-scf00021	20
AX-157915343	2	47.993	KV453193.1	598261	20-scf00021	20
AX-157915344	2	47.993	KV453193.1	598651	20-scf00021	20
AX-157915345	2	47.993	KV453193.1	599400	20-scf00021	20
AX-157915351	2	47.993	KV453193.1	603811	20-scf00021	20
AX-157915352	2	47.993	KV453193.1	605926	20-scf00021	20
AX-157915358	2	47.993	KV453193.1	612963	20-scf00021	20
AX-157775436	2	47.993	KV453193.1	620564	20-scf00021	20
AX-157812646	2	47.993	KV453193.1	622151	20-scf00021	20
AX-157915363	2	47.993	KV453193.1	624918	20-scf00021	20
AX-157725340	2	47.993	KV453193.1	625096	20-scf00021	20
AX-157915365	2	47.993	KV453193.1	627819	20-scf00021	20
AX-157915369	2	47.993	KV453193.1	631139	20-scf00021	20
AX-157915371	2	47.993	KV453193.1	634464	20-scf00021	20
AX-157915382	2	47.993	KV453193.1	654214	20-scf00021	20
AX-157915388	2	47.993	KV453193.1	663123	20-scf00021	20
AX-157915487	2	47.993	KV453193.1	774652	20-scf00021	20
AX-157915587	2	47.993	KV453193.1	965408	20-scf00021	20
AX-157915606	2	47.993	KV453193.1	991346	20-scf00021	20
AX-157915736	2	47.993	KV453193.1	1173059	20-scf00021	20
AX-158375380	2	47.993	KV453187.1	45791	20-scf00563	20
AX-158375384	2	47.993	KV453187.1	111152	20-scf00563	20
AX-158375385	2	47.993	KV453187.1	133325	20-scf00563	20
AX-158375386	2	47.993	KV453187.1	134905	20-scf00563	20
AX-158375387	2	47.993	KV453187.1	155629	20-scf00563	20
AX-158375389	2	47.993	KV453187.1	166569	20-scf00563	20
AX-158375449	2	47.993	KV453187.1	833890	20-scf00563	20
AX-158375453	2	47.993	KV453187.1	865109	20-scf00563	20
AX-158375470	2	47.993	KV453187.1	1114836	20-scf00563	20
AX-158376098	2	47.993	KV453183.1	151617	20-scf00571	20
AX-158376115	2	47.993	KV453183.1	333626	20-scf00571	20
AX-157813130	2	47.993	KV453183.1	744142	20-scf00571	20
AX-157920224	2	47.993	KV453183.1	1135678	20-scf00571	20
AX-157920267	2	47.993	KV453183.1	1297342	20-scf00571	20
AX-157920271	2	47.993	KV453183.1	1318310	20-scf00571	20
AX-157920272	2	47.993	KV453183.1	1320046	20-scf00571	20

AX-157920278	2	47.993	KV453183.1	1346609	20-scf00571	20
AX-157695780	2	47.993	KV453183.1	1380167	20-scf00571	20
AX-157920296	2	47.993	KV453183.1	1512093	20-scf00571	20
AX-157920299	2	47.993	KV453183.1	1526273	20-scf00571	20
AX-157920312	2	47.993	KV453183.1	1639348	20-scf00571	20
AX-157920324	2	47.993	KV453183.1	1730239	20-scf00571	20
AX-158376138	2	47.993	KV453190.1	1044	20-scf00573	20
AX-158376147	2	47.993	KV453190.1	134212	20-scf00573	20
AX-158376157	2	47.993	KV453190.1	213859	20-scf00573	20
AX-157685030	2	47.993	KV453191.1	136605	20-scf00582	20
AX-157685378	2	47.993	KV453188.1	144436	20-scf00886	20
AX-157685062	2	47.993	KV453359.1	57595	scf00584	UN
AX-157685066	2	47.993	KV453359.1	74384	scf00584	UN
AX-158420586	2	47.993	KV453360.1	4131	scf00585	UN
AX-157705855	2	47.993	3ML01031065	369	scf06670_1	UN
AX-157915391	2	48.145	KV453193.1	670400	20-scf00021	20
AX-157914301	2	48.297	KV453182.1	6170337	20-scf00019	20
AX-157914551	2	48.297	KV453182.1	7148960	20-scf00019	20
AX-157914558	2	48.297	KV453182.1	7159593	20-scf00019	20
AX-157914576	2	48.297	KV453182.1	7230878	20-scf00019	20
AX-157914605	2	48.297	KV453182.1	7483195	20-scf00019	20
AX-157920376	2	48.297	KV453189.1	1599803	20-scf00020	20
AX-157813162	2	48.297	KV453189.1	1605796	20-scf00020	20
AX-157920383	2	48.297	KV453189.1	1640068	20-scf00020	20
AX-157920402	2	48.297	KV453189.1	1703415	20-scf00020	20
AX-157920406	2	48.297	KV453189.1	1715845	20-scf00020	20
AX-157920440	2	48.297	KV453189.1	1858334	20-scf00020	20
AX-157812611	2	48.297	KV453193.1	60637	20-scf00021	20
AX-157914962	2	48.297	KV453193.1	109477	20-scf00021	20
AX-157915002	2	48.297	KV453193.1	162939	20-scf00021	20
AX-157915005	2	48.297	KV453193.1	168878	20-scf00021	20
AX-157746441	2	48.297	KV453193.1	291351	20-scf00021	20
AX-157812630	2	48.297	KV453193.1	293552	20-scf00021	20
AX-157915125	2	48.297	KV453193.1	298606	20-scf00021	20
AX-157915126	2	48.297	KV453193.1	299263	20-scf00021	20
AX-157915127	2	48.297	KV453193.1	300524	20-scf00021	20
AX-157915133	2	48.297	KV453193.1	306713	20-scf00021	20
AX-157915166	2	48.297	KV453193.1	337411	20-scf00021	20
AX-157915167	2	48.297	KV453193.1	337928	20-scf00021	20
AX-157915168	2	48.297	KV453193.1	339146	20-scf00021	20
AX-157915169	2	48.297	KV453193.1	339813	20-scf00021	20
AX-157915170	2	48.297	KV453193.1	340357	20-scf00021	20
AX-157915171	2	48.297	KV453193.1	340660	20-scf00021	20
AX-157915174	2	48.297	KV453193.1	343660	20-scf00021	20
AX-157915179	2	48.297	KV453193.1	351580	20-scf00021	20
AX-157915183	2	48.297	KV453193.1	353825	20-scf00021	20
AX-157812631	2	48.297	KV453193.1	354855	20-scf00021	20
AX-157915184	2	48.297	KV453193.1	356141	20-scf00021	20
AX-158379257	2	48.297	KV453193.1	357089	20-scf00021	20
AX-157727381	2	48.297	KV453193.1	357843	20-scf00021	20
AX-157719764	2	48.297	KV453193.1	358908	20-scf00021	20

AX-157915186	2	48.297	KV453193.1	361380	20-scf00021	20
AX-157915191	2	48.297	KV453193.1	365818	20-scf00021	20
AX-157812633	2	48.297	KV453193.1	366073	20-scf00021	20
AX-157915192	2	48.297	KV453193.1	369794	20-scf00021	20
AX-157915196	2	48.297	KV453193.1	376684	20-scf00021	20
AX-157915197	2	48.297	KV453193.1	378605	20-scf00021	20
AX-157915204	2	48.297	KV453193.1	390859	20-scf00021	20
AX-158405031	2	48.297	KV453193.1	395126	20-scf00021	20
AX-157915210	2	48.297	KV453193.1	395936	20-scf00021	20
AX-157915211	2	48.297	KV453193.1	396356	20-scf00021	20
AX-157915213	2	48.297	KV453193.1	396954	20-scf00021	20
AX-157915216	2	48.297	KV453193.1	399928	20-scf00021	20
AX-157725624	2	48.297	KV453193.1	400500	20-scf00021	20
AX-157915230	2	48.297	KV453193.1	410879	20-scf00021	20
AX-157777949	2	48.297	KV453193.1	411137	20-scf00021	20
AX-157915231	2	48.297	KV453193.1	411410	20-scf00021	20
AX-157812636	2	48.297	KV453193.1	411937	20-scf00021	20
AX-157915233	2	48.297	KV453193.1	414474	20-scf00021	20
AX-157915317	2	48.297	KV453193.1	564246	20-scf00021	20
AX-157915327	2	48.297	KV453193.1	580470	20-scf00021	20
AX-157915331	2	48.297	KV453193.1	586776	20-scf00021	20
AX-157915355	2	48.297	KV453193.1	609787	20-scf00021	20
AX-157915361	2	48.297	KV453193.1	623919	20-scf00021	20
AX-157915362	2	48.297	KV453193.1	624405	20-scf00021	20
AX-157915368	2	48.297	KV453193.1	630862	20-scf00021	20
AX-157915376	2	48.297	KV453193.1	643065	20-scf00021	20
AX-157915385	2	48.297	KV453193.1	656761	20-scf00021	20
AX-157915386	2	48.297	KV453193.1	657297	20-scf00021	20
AX-157915393	2	48.297	KV453193.1	673413	20-scf00021	20
AX-157724786	2	48.297	KV453193.1	675030	20-scf00021	20
AX-157915394	2	48.297	KV453193.1	675775	20-scf00021	20
AX-157915395	2	48.297	KV453193.1	676342	20-scf00021	20
AX-157915396	2	48.297	KV453193.1	676856	20-scf00021	20
AX-157915410	2	48.297	KV453193.1	689509	20-scf00021	20
AX-157915411	2	48.297	KV453193.1	691377	20-scf00021	20
AX-157915412	2	48.297	KV453193.1	693586	20-scf00021	20
AX-157915413	2	48.297	KV453193.1	694439	20-scf00021	20
AX-157915416	2	48.297	KV453193.1	698670	20-scf00021	20
AX-157915418	2	48.297	KV453193.1	700307	20-scf00021	20
AX-157915421	2	48.297	KV453193.1	702943	20-scf00021	20
AX-157915422	2	48.297	KV453193.1	703777	20-scf00021	20
AX-157915428	2	48.297	KV453193.1	710474	20-scf00021	20
AX-157915429	2	48.297	KV453193.1	710693	20-scf00021	20
AX-157915439	2	48.297	KV453193.1	721447	20-scf00021	20
AX-157915441	2	48.297	KV453193.1	725523	20-scf00021	20
AX-157915442	2	48.297	KV453193.1	727622	20-scf00021	20
AX-157812652	2	48.297	KV453193.1	727836	20-scf00021	20
AX-157915443	2	48.297	KV453193.1	728647	20-scf00021	20
AX-157915445	2	48.297	KV453193.1	729753	20-scf00021	20
AX-157915448	2	48.297	KV453193.1	737233	20-scf00021	20
AX-157915449	2	48.297	KV453193.1	738096	20-scf00021	20

AX-157915453	2	48.297	KV453193.1	741223	20-scf00021	20
AX-157915454	2	48.297	KV453193.1	741532	20-scf00021	20
AX-157915455	2	48.297	KV453193.1	742228	20-scf00021	20
AX-157915466	2	48.297	KV453193.1	750484	20-scf00021	20
AX-157812655	2	48.297	KV453193.1	755685	20-scf00021	20
AX-157812656	2	48.297	KV453193.1	756084	20-scf00021	20
AX-157915474	2	48.297	KV453193.1	760480	20-scf00021	20
AX-157915485	2	48.297	KV453193.1	774029	20-scf00021	20
AX-157915486	2	48.297	KV453193.1	774305	20-scf00021	20
AX-157915490	2	48.297	KV453193.1	777473	20-scf00021	20
AX-157915491	2	48.297	KV453193.1	777891	20-scf00021	20
AX-157812661	2	48.297	KV453193.1	784801	20-scf00021	20
AX-157915498	2	48.297	KV453193.1	795152	20-scf00021	20
AX-157915501	2	48.297	KV453193.1	800246	20-scf00021	20
AX-157915506	2	48.297	KV453193.1	806021	20-scf00021	20
AX-158414820	2	48.297	KV453193.1	871273	20-scf00021	20
AX-157915566	2	48.297	KV453193.1	940683	20-scf00021	20
AX-157915572	2	48.297	KV453193.1	946671	20-scf00021	20
AX-157915573	2	48.297	KV453193.1	947783	20-scf00021	20
AX-157771664	2	48.297	KV453193.1	949438	20-scf00021	20
AX-157915575	2	48.297	KV453193.1	949523	20-scf00021	20
AX-157915576	2	48.297	KV453193.1	951244	20-scf00021	20
AX-157915584	2	48.297	KV453193.1	961210	20-scf00021	20
AX-157915595	2	48.297	KV453193.1	980500	20-scf00021	20
AX-157883453	2	48.297	KV453193.1	981149	20-scf00021	20
AX-157915600	2	48.297	KV453193.1	984467	20-scf00021	20
AX-157915608	2	48.297	KV453193.1	996902	20-scf00021	20
AX-157915609	2	48.297	KV453193.1	997923	20-scf00021	20
AX-157915612	2	48.297	KV453193.1	1000639	20-scf00021	20
AX-157915614	2	48.297	KV453193.1	1003982	20-scf00021	20
AX-157915616	2	48.297	KV453193.1	1007269	20-scf00021	20
AX-157915619	2	48.297	KV453193.1	1010710	20-scf00021	20
AX-157915620	2	48.297	KV453193.1	1011593	20-scf00021	20
AX-157915621	2	48.297	KV453193.1	1013113	20-scf00021	20
AX-158405045	2	48.297	KV453193.1	1024375	20-scf00021	20
AX-157915635	2	48.297	KV453193.1	1033747	20-scf00021	20
AX-157915641	2	48.297	KV453193.1	1037906	20-scf00021	20
AX-157915643	2	48.297	KV453193.1	1040092	20-scf00021	20
AX-157915646	2	48.297	KV453193.1	1045136	20-scf00021	20
AX-157742703	2	48.297	KV453193.1	1045857	20-scf00021	20
AX-158405048	2	48.297	KV453193.1	1045920	20-scf00021	20
AX-157915647	2	48.297	KV453193.1	1048116	20-scf00021	20
AX-157915662	2	48.297	KV453193.1	1084259	20-scf00021	20
AX-157915692	2	48.297	KV453193.1	1122104	20-scf00021	20
AX-157915706	2	48.297	KV453193.1	1135797	20-scf00021	20
AX-157915718	2	48.297	KV453193.1	1147011	20-scf00021	20
AX-157915760	2	48.297	KV453193.1	1207946	20-scf00021	20
AX-157915850	2	48.297	KV453193.1	1332674	20-scf00021	20
AX-157915903	2	48.297	KV453193.1	1393542	20-scf00021	20
AX-158375448	2	48.297	KV453187.1	810624	20-scf00563	20
AX-158376093	2	48.297	KV453183.1	107313	20-scf00571	20

AX-158376094	2	48.297	KV453183.1	108852	20-scf00571	20
AX-158414822	2	48.298	KV453193.1	1045963	20-scf00021	20
AX-157915650	2	48.298	KV453193.1	1051455	20-scf00021	20
AX-157915656	2	48.31	KV453193.1	1075421	20-scf00021	20
AX-157915493	2	48.467	KV453193.1	779949	20-scf00021	20
AX-157915655	2	48.467	KV453193.1	1073194	20-scf00021	20
AX-157915657	2	48.467	KV453193.1	1078215	20-scf00021	20
AX-157915677	2	48.636	KV453193.1	1107357	20-scf00021	20
AX-157915679	2	48.636	KV453193.1	1109401	20-scf00021	20
AX-157915680	2	48.636	KV453193.1	1111284	20-scf00021	20
AX-157915681	2	48.636	KV453193.1	1111955	20-scf00021	20
AX-157915684	2	48.636	KV453193.1	1115383	20-scf00021	20
AX-157915685	2	48.636	KV453193.1	1115653	20-scf00021	20
AX-157915686	2	48.636	KV453193.1	1115974	20-scf00021	20
AX-157915688	2	48.636	KV453193.1	1117529	20-scf00021	20
AX-157915696	2	48.636	KV453193.1	1123822	20-scf00021	20
AX-157915697	2	48.636	KV453193.1	1124693	20-scf00021	20
AX-157883456	2	48.636	KV453193.1	1125940	20-scf00021	20
AX-157915699	2	48.636	KV453193.1	1126240	20-scf00021	20
AX-157915719	2	48.636	KV453193.1	1148141	20-scf00021	20
AX-157915725	2	48.636	KV453193.1	1153514	20-scf00021	20
AX-157915726	2	48.636	KV453193.1	1154345	20-scf00021	20
AX-157915728	2	48.636	KV453193.1	1157821	20-scf00021	20
AX-157727189	2	48.636	KV453193.1	1167122	20-scf00021	20
AX-157915278	2	48.94	KV453193.1	504213	20-scf00021	20
AX-157915279	2	48.94	KV453193.1	505492	20-scf00021	20
AX-157915426	2	48.94	KV453193.1	706860	20-scf00021	20
AX-157915602	2	48.94	KV453193.1	985263	20-scf00021	20
AX-157915730	2	48.94	KV453193.1	1162117	20-scf00021	20
AX-157915732	2	48.94	KV453193.1	1169280	20-scf00021	20
AX-157920352	2	49.143	KV453189.1	1528013	20-scf00020	20
AX-157915037	2	49.143	KV453193.1	202523	20-scf00021	20
AX-157915068	2	49.143	KV453193.1	236208	20-scf00021	20
AX-157719937	2	49.143	KV453193.1	665363	20-scf00021	20
AX-157915518	2	49.143	KV453193.1	862459	20-scf00021	20
AX-158389603	2	49.143	KV453193.1	883805	20-scf00021	20
AX-157915532	2	49.143	KV453193.1	898934	20-scf00021	20
AX-157915538	2	49.143	KV453193.1	903406	20-scf00021	20
AX-157915543	2	49.143	KV453193.1	908225	20-scf00021	20
AX-157915563	2	49.143	KV453193.1	937084	20-scf00021	20
AX-157915577	2	49.143	KV453193.1	952196	20-scf00021	20
AX-157915752	2	49.143	KV453193.1	1194019	20-scf00021	20
AX-157915756	2	49.143	KV453193.1	1199221	20-scf00021	20
AX-157915848	2	49.143	KV453193.1	1329545	20-scf00021	20
AX-157920279	2	49.143	KV453183.1	1350976	20-scf00571	20
AX-157920298	2	49.143	KV453183.1	1519201	20-scf00571	20
AX-157920314	2	49.143	KV453183.1	1651684	20-scf00571	20
AX-157920325	2	49.143	KV453183.1	1766040	20-scf00571	20
AX-157749420	2	49.144	KV453193.1	1177155	20-scf00021	20
AX-157915747	2	49.144	KV453193.1	1185862	20-scf00021	20
AX-157915764	2	49.144	KV453193.1	1212265	20-scf00021	20

AX-157915767	2	49.144	KV453193.1	1217302	20-scf00021	20
AX-157915768	2	49.144	KV453193.1	1217940	20-scf00021	20
AX-157915769	2	49.144	KV453193.1	1218800	20-scf00021	20
AX-157915789	2	49.144	KV453193.1	1253792	20-scf00021	20
AX-157915911	2	49.144	KV453193.1	1400484	20-scf00021	20
AX-157812692	2	49.144	KV453193.1	1441868	20-scf00021	20
AX-157915974	2	49.144	KV453193.1	1474525	20-scf00021	20
AX-157915742	2	49.313	KV453193.1	1179464	20-scf00021	20
AX-157708660	2	49.313	KV453193.1	1193026	20-scf00021	20
AX-157915778	2	49.313	KV453193.1	1233881	20-scf00021	20
AX-157915779	2	49.313	KV453193.1	1236328	20-scf00021	20
AX-157883459	2	49.313	KV453193.1	1237544	20-scf00021	20
AX-157915781	2	49.313	KV453193.1	1239387	20-scf00021	20
AX-157745433	2	49.313	KV453193.1	1242846	20-scf00021	20
AX-157741242	2	49.313	KV453193.1	1252446	20-scf00021	20
AX-157915788	2	49.313	KV453193.1	1252619	20-scf00021	20
AX-157774441	2	49.532	KV453193.1	1246132	20-scf00021	20
AX-157915795	2	49.532	KV453193.1	1259840	20-scf00021	20
AX-157812677	2	49.532	KV453193.1	1261891	20-scf00021	20
AX-157915829	2	49.532	KV453193.1	1310391	20-scf00021	20
AX-157915799	2	49.57	KV453193.1	1264736	20-scf00021	20
AX-157915801	2	49.57	KV453193.1	1267333	20-scf00021	20
AX-157915802	2	49.57	KV453193.1	1268423	20-scf00021	20
AX-157915808	2	49.57	KV453193.1	1280417	20-scf00021	20
AX-157853725	2	49.57	KV453193.1	1311442	20-scf00021	20
AX-157915806	2	49.619	KV453193.1	1275375	20-scf00021	20
AX-157915836	2	49.663	KV453193.1	1317683	20-scf00021	20
AX-157915839	2	49.698	KV453193.1	1319741	20-scf00021	20
AX-158405054	2	49.739	KV453193.1	1320113	20-scf00021	20
AX-157915841	2	49.779	KV453193.1	1321184	20-scf00021	20
AX-157915845	2	49.808	KV453193.1	1324386	20-scf00021	20
AX-157743484	2	49.832	KV453193.1	1163899	20-scf00021	20
AX-157915854	2	49.832	KV453193.1	1338132	20-scf00021	20
AX-157812672	2	49.86	KV453193.1	1164071	20-scf00021	20
AX-157722905	2	49.86	KV453193.1	1178033	20-scf00021	20
AX-157915754	2	49.86	KV453193.1	1197086	20-scf00021	20
AX-157915774	2	49.86	KV453193.1	1222905	20-scf00021	20
AX-157915786	2	49.86	KV453193.1	1245259	20-scf00021	20
AX-157915791	2	49.86	KV453193.1	1257277	20-scf00021	20
AX-157915803	2	49.86	KV453193.1	1272098	20-scf00021	20
AX-157774503	2	49.86	KV453193.1	1306441	20-scf00021	20
AX-157915855	2	49.86	KV453193.1	1339850	20-scf00021	20
AX-157915856	2	49.86	KV453193.1	1340637	20-scf00021	20
AX-157915857	2	49.86	KV453193.1	1341143	20-scf00021	20
AX-157915858	2	49.86	KV453193.1	1342799	20-scf00021	20
AX-157915887	2	49.86	KV453193.1	1378226	20-scf00021	20
AX-157915936	2	49.86	KV453193.1	1422848	20-scf00021	20
AX-157915945	2	49.86	KV453193.1	1435725	20-scf00021	20
AX-157915946	2	49.86	KV453193.1	1436013	20-scf00021	20
AX-157915948	2	49.86	KV453193.1	1439455	20-scf00021	20
AX-157915951	2	49.86	KV453193.1	1445235	20-scf00021	20

AX-157916073	2	49.86	KV453193.1	1627814	20-scf00021	20
AX-157853782	2	49.915	KV453189.1	1798676	20-scf00020	20
AX-157920429	2	49.915	KV453189.1	1805463	20-scf00020	20
AX-158382312	2	49.915	KV453189.1	1824225	20-scf00020	20
AX-157853783	2	49.915	KV453189.1	1824438	20-scf00020	20
AX-157813170	2	49.915	KV453189.1	1845943	20-scf00020	20
AX-157915093	2	49.915	KV453193.1	263158	20-scf00021	20
AX-157915096	2	49.915	KV453193.1	264749	20-scf00021	20
AX-157915097	2	49.915	KV453193.1	265445	20-scf00021	20
AX-157915098	2	49.915	KV453193.1	266288	20-scf00021	20
AX-157915099	2	49.915	KV453193.1	267224	20-scf00021	20
AX-158389599	2	49.915	KV453193.1	267946	20-scf00021	20
AX-157915121	2	49.915	KV453193.1	293022	20-scf00021	20
AX-157915176	2	49.915	KV453193.1	347347	20-scf00021	20
AX-157915181	2	49.915	KV453193.1	352863	20-scf00021	20
AX-157812632	2	49.915	KV453193.1	355345	20-scf00021	20
AX-157723698	2	49.915	KV453193.1	356955	20-scf00021	20
AX-157915190	2	49.915	KV453193.1	365250	20-scf00021	20
AX-157915219	2	49.915	KV453193.1	402064	20-scf00021	20
AX-157853720	2	49.915	KV453193.1	403578	20-scf00021	20
AX-157915226	2	49.915	KV453193.1	407218	20-scf00021	20
AX-157915227	2	49.915	KV453193.1	407818	20-scf00021	20
AX-157915285	2	49.915	KV453193.1	514936	20-scf00021	20
AX-157812659	2	49.915	KV453193.1	778386	20-scf00021	20
AX-157915505	2	49.915	KV453193.1	805222	20-scf00021	20
AX-157915582	2	49.915	KV453193.1	958061	20-scf00021	20
AX-157915599	2	49.915	KV453193.1	984110	20-scf00021	20
AX-157812667	2	49.915	KV453193.1	1014595	20-scf00021	20
AX-157883454	2	49.915	KV453193.1	1032285	20-scf00021	20
AX-157915700	2	49.915	KV453193.1	1128188	20-scf00021	20
AX-157915714	2	49.915	KV453193.1	1144232	20-scf00021	20
AX-157758998	2	49.915	KV453193.1	1245611	20-scf00021	20
AX-157915804	2	49.915	KV453193.1	1272910	20-scf00021	20
AX-157739374	2	49.915	KV453193.1	1324577	20-scf00021	20
AX-157915859	2	49.915	KV453193.1	1343250	20-scf00021	20
AX-157751254	2	49.915	KV453193.1	1353814	20-scf00021	20
AX-157812684	2	49.915	KV453193.1	1355082	20-scf00021	20
AX-157812691	2	49.915	KV453193.1	1437519	20-scf00021	20
AX-157915866	2	49.969	KV453193.1	1356473	20-scf00021	20
AX-157915314	2	50.078	KV453193.1	561509	20-scf00021	20
AX-157759069	2	50.078	KV453193.1	1343966	20-scf00021	20
AX-157915872	2	50.078	KV453193.1	1362697	20-scf00021	20
AX-157915875	2	50.078	KV453193.1	1364528	20-scf00021	20
AX-157915876	2	50.078	KV453193.1	1366713	20-scf00021	20
AX-157915877	2	50.078	KV453193.1	1367207	20-scf00021	20
AX-157915878	2	50.078	KV453193.1	1367569	20-scf00021	20
AX-157812685	2	50.078	KV453193.1	1368421	20-scf00021	20
AX-157915881	2	50.078	KV453193.1	1370157	20-scf00021	20
AX-157915882	2	50.078	KV453193.1	1371366	20-scf00021	20
AX-157915883	2	50.078	KV453193.1	1373503	20-scf00021	20
AX-157915885	2	50.078	KV453193.1	1376390	20-scf00021	20

AX-157812686	2	50.078	KV453193.1	1379865	20-scf00021	20
AX-157915889	2	50.078	KV453193.1	1380080	20-scf00021	20
AX-157915892	2	50.078	KV453193.1	1382239	20-scf00021	20
AX-157915893	2	50.078	KV453193.1	1382884	20-scf00021	20
AX-157915897	2	50.078	KV453193.1	1387270	20-scf00021	20
AX-157915898	2	50.078	KV453193.1	1388775	20-scf00021	20
AX-157915901	2	50.078	KV453193.1	1391297	20-scf00021	20
AX-157915928	2	50.078	KV453193.1	1413201	20-scf00021	20
AX-157915944	2	50.078	KV453193.1	1434875	20-scf00021	20
AX-157915999	2	50.078	KV453193.1	1512362	20-scf00021	20
AX-157916013	2	50.078	KV453193.1	1530289	20-scf00021	20
AX-157915902	2	50.103	KV453193.1	1392407	20-scf00021	20
AX-157739905	2	50.136	KV453193.1	1392537	20-scf00021	20
AX-157915510	2	50.188	KV453193.1	813739	20-scf00021	20
AX-157915675	2	50.188	KV453193.1	1100293	20-scf00021	20
AX-157812670	2	50.188	KV453193.1	1105357	20-scf00021	20
AX-157915784	2	50.188	KV453193.1	1243498	20-scf00021	20
AX-157915843	2	50.188	KV453193.1	1322012	20-scf00021	20
AX-157915906	2	50.188	KV453193.1	1394366	20-scf00021	20
AX-157915907	2	50.188	KV453193.1	1394983	20-scf00021	20
AX-157915910	2	50.188	KV453193.1	1400062	20-scf00021	20
AX-157915913	2	50.188	KV453193.1	1401538	20-scf00021	20
AX-157915915	2	50.188	KV453193.1	1402590	20-scf00021	20
AX-157713003	2	50.188	KV453193.1	1403454	20-scf00021	20
AX-157874699	2	50.188	KV453193.1	1403722	20-scf00021	20
AX-157915919	2	50.188	KV453193.1	1405196	20-scf00021	20
AX-157915920	2	50.188	KV453193.1	1406160	20-scf00021	20
AX-157915930	2	50.188	KV453193.1	1417300	20-scf00021	20
AX-157715078	2	50.188	KV453193.1	1419451	20-scf00021	20
AX-157915938	2	50.188	KV453193.1	1425548	20-scf00021	20
AX-157812689	2	50.188	KV453193.1	1426960	20-scf00021	20
AX-157812690	2	50.188	KV453193.1	1430259	20-scf00021	20
AX-157915943	2	50.188	KV453193.1	1434270	20-scf00021	20
AX-157915947	2	50.188	KV453193.1	1438071	20-scf00021	20
AX-157812693	2	50.188	KV453193.1	1443429	20-scf00021	20
AX-157915952	2	50.188	KV453193.1	1446185	20-scf00021	20
AX-157915953	2	50.188	KV453193.1	1448765	20-scf00021	20
AX-157915954	2	50.188	KV453193.1	1449784	20-scf00021	20
AX-157915956	2	50.188	KV453193.1	1450655	20-scf00021	20
AX-157915960	2	50.188	KV453193.1	1453952	20-scf00021	20
AX-157915961	2	50.188	KV453193.1	1454520	20-scf00021	20
AX-157874702	2	50.188	KV453193.1	1461320	20-scf00021	20
AX-157915985	2	50.515	KV453193.1	1488290	20-scf00021	20
AX-157915986	2	50.515	KV453193.1	1489798	20-scf00021	20
AX-157915996	2	50.515	KV453193.1	1505857	20-scf00021	20
AX-157915997	2	50.515	KV453193.1	1509540	20-scf00021	20
AX-157916001	2	50.842	KV453193.1	1516753	20-scf00021	20
AX-157916004	2	50.842	KV453193.1	1520279	20-scf00021	20
AX-157916017	2	50.842	KV453193.1	1533563	20-scf00021	20
AX-157916019	2	50.842	KV453193.1	1534718	20-scf00021	20
AX-157916049	2	50.842	KV453193.1	1575263	20-scf00021	20

AX-157915891	2	50.843	KV453193.1	1381680	20-scf00021	20
AX-157916002	2	50.843	KV453193.1	1519396	20-scf00021	20
AX-157853727	2	50.843	KV453193.1	1520503	20-scf00021	20
AX-157916007	2	50.843	KV453193.1	1522248	20-scf00021	20
AX-157916010	2	50.843	KV453193.1	1526216	20-scf00021	20
AX-157916033	2	50.843	KV453193.1	1554759	20-scf00021	20
AX-157916012	2	50.875	KV453193.1	1529775	20-scf00021	20
AX-157916026	2	50.875	KV453193.1	1542829	20-scf00021	20
AX-157732739	2	50.875	KV453193.1	1553784	20-scf00021	20
AX-157916018	2	50.893	KV453193.1	1533989	20-scf00021	20
AX-157916020	2	50.952	KV453193.1	1537196	20-scf00021	20
AX-157916022	2	50.952	KV453193.1	1538731	20-scf00021	20
AX-157916025	2	50.952	KV453193.1	1541259	20-scf00021	20
AX-157916030	2	50.952	KV453193.1	1548907	20-scf00021	20
AX-157812696	2	50.952	KV453193.1	1549142	20-scf00021	20
AX-157916034	2	51.061	KV453193.1	1555530	20-scf00021	20
AX-157916037	2	51.061	KV453193.1	1559974	20-scf00021	20
AX-157916038	2	51.061	KV453193.1	1561531	20-scf00021	20
AX-157916040	2	51.067	KV453193.1	1562743	20-scf00021	20
AX-157916024	2	51.071	KV453193.1	1540761	20-scf00021	20
AX-157916041	2	51.071	KV453193.1	1565447	20-scf00021	20
AX-157916005	2	51.076	KV453193.1	1520705	20-scf00021	20
AX-157916042	2	51.076	KV453193.1	1567749	20-scf00021	20
AX-157916043	2	51.076	KV453193.1	1569437	20-scf00021	20
AX-157916045	2	51.076	KV453193.1	1571129	20-scf00021	20
AX-157916047	2	51.078	KV453193.1	1573281	20-scf00021	20
AX-157916048	2	51.214	KV453193.1	1574151	20-scf00021	20
AX-157916050	2	51.314	KV453193.1	1576116	20-scf00021	20
AX-157916028	2	51.329	KV453193.1	1544010	20-scf00021	20
AX-157751695	2	51.329	KV453193.1	1577588	20-scf00021	20
AX-157916053	2	51.703	KV453193.1	1587219	20-scf00021	20
AX-157812699	2	51.718	KV453193.1	1588513	20-scf00021	20
AX-157916055	2	51.718	KV453193.1	1589370	20-scf00021	20
AX-157916058	2	51.718	KV453193.1	1595852	20-scf00021	20
AX-157715653	2	51.718	KV453193.1	1598148	20-scf00021	20
AX-157916061	2	51.718	KV453193.1	1600856	20-scf00021	20
AX-157916063	2	51.718	KV453193.1	1602076	20-scf00021	20
AX-157916064	2	51.718	KV453193.1	1604551	20-scf00021	20
AX-158379262	2	51.718	KV453193.1	1605768	20-scf00021	20
AX-157812701	2	51.718	KV453193.1	1605864	20-scf00021	20
AX-157916065	2	51.718	KV453193.1	1609259	20-scf00021	20
AX-157812702	2	51.718	KV453193.1	1611610	20-scf00021	20
AX-157916066	2	51.854	KV453193.1	1612641	20-scf00021	20
AX-157709338	2	51.854	KV453193.1	1614099	20-scf00021	20
AX-157916068	2	51.856	KV453193.1	1617154	20-scf00021	20
AX-157916071	2	52.264	KV453193.1	1625196	20-scf00021	20
AX-157916076	2	52.264	KV453193.1	1636125	20-scf00021	20
AX-157916081	2	52.264	KV453193.1	1644703	20-scf00021	20
AX-157916084	2	52.264	KV453193.1	1650606	20-scf00021	20
AX-157916085	2	52.264	KV453193.1	1651091	20-scf00021	20
AX-157719287	2	52.264	KV453193.1	1652063	20-scf00021	20

AX-157916086	2	52.264	KV453193.1	1654389	20-scf00021	20
AX-157745268	2	52.264	KV453193.1	1657922	20-scf00021	20
AX-157916095	2	52.264	KV453193.1	1662156	20-scf00021	20
AX-157916097	2	52.264	KV453193.1	1663722	20-scf00021	20
AX-157916099	2	52.264	KV453193.1	1670404	20-scf00021	20
AX-157916100	2	52.264	KV453193.1	1671022	20-scf00021	20
AX-157916103	2	52.264	KV453193.1	1678160	20-scf00021	20
AX-157916108	2	52.264	KV453193.1	1688433	20-scf00021	20
AX-157916109	2	52.264	KV453193.1	1689941	20-scf00021	20
AX-157916111	2	52.264	KV453193.1	1692954	20-scf00021	20
AX-157812710	2	52.264	KV453193.1	1698280	20-scf00021	20
AX-157750268	2	52.264	KV453193.1	1700759	20-scf00021	20
AX-157916121	2	52.264	KV453193.1	1710772	20-scf00021	20
AX-157916150	2	52.264	KV453193.1	1750661	20-scf00021	20
AX-157707079	2	52.264	3ML01033465	263	scf09116_1	UN
AX-157916117	2	52.482	KV453193.1	1705271	20-scf00021	20
AX-157916115	2	52.514	KV453193.1	1703524	20-scf00021	20
AX-157916122	2	52.514	KV453193.1	1712105	20-scf00021	20
AX-157916130	2	52.514	KV453193.1	1724409	20-scf00021	20
AX-157916201	2	52.514	KV453193.1	1812883	20-scf00021	20
AX-157916123	2	52.663	KV453193.1	1712407	20-scf00021	20
AX-157916126	2	52.81	KV453193.1	1719032	20-scf00021	20
AX-157772466	2	52.81	KV453193.1	1719465	20-scf00021	20
AX-157916127	2	52.81	KV453193.1	1720393	20-scf00021	20
AX-157916132	2	52.81	KV453193.1	1725745	20-scf00021	20
AX-157725969	2	52.81	KV453193.1	1730944	20-scf00021	20
AX-157812713	2	52.81	KV453193.1	1731960	20-scf00021	20
AX-157916136	2	52.81	KV453193.1	1733742	20-scf00021	20
AX-157812716	2	52.81	KV453193.1	1734458	20-scf00021	20
AX-157916138	2	52.81	KV453193.1	1736249	20-scf00021	20
AX-157916140	2	52.81	KV453193.1	1737752	20-scf00021	20
AX-157755524	2	52.81	KV453193.1	1739650	20-scf00021	20
AX-157916154	2	52.81	KV453193.1	1753246	20-scf00021	20
AX-157916155	2	52.81	KV453193.1	1754112	20-scf00021	20
AX-157748971	2	52.81	KV453193.1	1794269	20-scf00021	20
AX-157916268	2	52.81	KV453193.1	1877707	20-scf00021	20
AX-157916128	2	52.92	KV453193.1	1720959	20-scf00021	20
AX-157916145	2	52.92	KV453193.1	1745762	20-scf00021	20
AX-157916146	2	52.92	KV453193.1	1746858	20-scf00021	20
AX-157916148	2	52.938	KV453193.1	1749537	20-scf00021	20
AX-157812719	2	52.938	KV453193.1	1751504	20-scf00021	20
AX-157916149	2	52.986	KV453193.1	1749689	20-scf00021	20
AX-157916158	2	53.029	KV453193.1	1756525	20-scf00021	20
AX-157812720	2	53.051	KV453193.1	1762002	20-scf00021	20
AX-157812725	2	53.071	KV453193.1	1772328	20-scf00021	20
AX-157812718	2	53.094	KV453193.1	1748077	20-scf00021	20
AX-157916168	2	53.094	KV453193.1	1774625	20-scf00021	20
AX-157916254	2	53.094	KV453193.1	1855948	20-scf00021	20
AX-157916170	2	53.118	KV453193.1	1775621	20-scf00021	20
AX-157916183	2	53.118	KV453193.1	1793378	20-scf00021	20
AX-157916192	2	53.118	KV453193.1	1802765	20-scf00021	20

AX-157916330	2	53.118	KV453193.1	1969214	20-scf00021	20
AX-157916178	2	53.125	KV453193.1	1785766	20-scf00021	20
AX-157812717	2	53.138	KV453193.1	1746456	20-scf00021	20
AX-157812721	2	53.138	KV453193.1	1762806	20-scf00021	20
AX-157812722	2	53.138	KV453193.1	1762880	20-scf00021	20
AX-157916164	2	53.138	KV453193.1	1769709	20-scf00021	20
AX-157916167	2	53.138	KV453193.1	1773470	20-scf00021	20
AX-157812726	2	53.138	KV453193.1	1775445	20-scf00021	20
AX-157916171	2	53.138	KV453193.1	1776480	20-scf00021	20
AX-157812729	2	53.138	KV453193.1	1790819	20-scf00021	20
AX-157916184	2	53.138	KV453193.1	1796013	20-scf00021	20
AX-157916189	2	53.138	KV453193.1	1799564	20-scf00021	20
AX-157916191	2	53.138	KV453193.1	1800903	20-scf00021	20
AX-157916193	2	53.138	KV453193.1	1803168	20-scf00021	20
AX-157916194	2	53.138	KV453193.1	1803860	20-scf00021	20
AX-157916195	2	53.138	KV453193.1	1803967	20-scf00021	20
AX-157916206	2	53.138	KV453193.1	1816615	20-scf00021	20
AX-157916207	2	53.138	KV453193.1	1816826	20-scf00021	20
AX-157916210	2	53.138	KV453193.1	1818439	20-scf00021	20
AX-157916212	2	53.138	KV453193.1	1819887	20-scf00021	20
AX-157753331	2	53.138	KV453193.1	1820329	20-scf00021	20
AX-157916216	2	53.138	KV453193.1	1823246	20-scf00021	20
AX-157916217	2	53.138	KV453193.1	1823647	20-scf00021	20
AX-157916218	2	53.138	KV453193.1	1824247	20-scf00021	20
AX-157916220	2	53.138	KV453193.1	1825776	20-scf00021	20
AX-157916222	2	53.138	KV453193.1	1826386	20-scf00021	20
AX-157916223	2	53.138	KV453193.1	1827558	20-scf00021	20
AX-157916224	2	53.138	KV453193.1	1827806	20-scf00021	20
AX-157758917	2	53.138	KV453193.1	1827880	20-scf00021	20
AX-157916225	2	53.138	KV453193.1	1828075	20-scf00021	20
AX-157916227	2	53.138	KV453193.1	1828984	20-scf00021	20
AX-157916228	2	53.138	KV453193.1	1829401	20-scf00021	20
AX-157721905	2	53.138	KV453193.1	1830243	20-scf00021	20
AX-157916229	2	53.138	KV453193.1	1831338	20-scf00021	20
AX-157869102	2	53.138	KV453193.1	1832225	20-scf00021	20
AX-157719098	2	53.138	KV453193.1	1832736	20-scf00021	20
AX-157916231	2	53.138	KV453193.1	1833402	20-scf00021	20
AX-157756370	2	53.138	KV453193.1	1833764	20-scf00021	20
AX-157916232	2	53.138	KV453193.1	1834221	20-scf00021	20
AX-157740784	2	53.138	KV453193.1	1834260	20-scf00021	20
AX-157916235	2	53.138	KV453193.1	1836776	20-scf00021	20
AX-157916237	2	53.138	KV453193.1	1838547	20-scf00021	20
AX-157916238	2	53.138	KV453193.1	1838742	20-scf00021	20
AX-157916239	2	53.138	KV453193.1	1839173	20-scf00021	20
AX-157916241	2	53.138	KV453193.1	1841440	20-scf00021	20
AX-157916242	2	53.138	KV453193.1	1842008	20-scf00021	20
AX-157709210	2	53.138	KV453193.1	1842866	20-scf00021	20
AX-157916244	2	53.138	KV453193.1	1844189	20-scf00021	20
AX-157812731	2	53.138	KV453193.1	1847037	20-scf00021	20
AX-157916260	2	53.138	KV453193.1	1866608	20-scf00021	20
AX-157726486	2	53.138	KV453193.1	1935245	20-scf00021	20

AX-157916627	2	53.138	KV453193.1	2358752	20-scf00021	20
AX-157916782	2	53.138	KV453193.1	2657911	20-scf00021	20
AX-157916836	2	53.138	KV453193.1	2789031	20-scf00021	20
AX-157916258	2	53.247	KV453193.1	1864558	20-scf00021	20
AX-157916263	2	53.247	KV453193.1	1869752	20-scf00021	20
AX-157916264	2	53.247	KV453193.1	1870477	20-scf00021	20
AX-157916273	2	53.266	KV453193.1	1884070	20-scf00021	20
AX-157916281	2	53.289	KV453193.1	1901104	20-scf00021	20
AX-157916286	2	53.311	KV453193.1	1907430	20-scf00021	20
AX-157916252	2	53.34	KV453193.1	1853144	20-scf00021	20
AX-157812735	2	53.34	KV453193.1	1913139	20-scf00021	20
AX-157757230	2	53.407	KV453193.1	1915889	20-scf00021	20
AX-157916205	2	53.466	KV453193.1	1816353	20-scf00021	20
AX-157916261	2	53.466	KV453193.1	1867237	20-scf00021	20
AX-157812733	2	53.466	KV453193.1	1875435	20-scf00021	20
AX-157869104	2	53.466	KV453193.1	1876987	20-scf00021	20
AX-157727502	2	53.466	KV453193.1	1888329	20-scf00021	20
AX-157916284	2	53.466	KV453193.1	1903005	20-scf00021	20
AX-157916289	2	53.466	KV453193.1	1911244	20-scf00021	20
AX-157916294	2	53.466	KV453193.1	1926793	20-scf00021	20
AX-157916296	2	53.466	KV453193.1	1931053	20-scf00021	20
AX-157916298	2	53.466	KV453193.1	1935357	20-scf00021	20
AX-157916299	2	53.466	KV453193.1	1935869	20-scf00021	20
AX-157916302	2	53.466	KV453193.1	1938519	20-scf00021	20
AX-157916303	2	53.466	KV453193.1	1939361	20-scf00021	20
AX-157916304	2	53.466	KV453193.1	1939716	20-scf00021	20
AX-157916306	2	53.466	KV453193.1	1941990	20-scf00021	20
AX-157916308	2	53.466	KV453193.1	1945600	20-scf00021	20
AX-157916310	2	53.466	KV453193.1	1947771	20-scf00021	20
AX-157916311	2	53.466	KV453193.1	1949384	20-scf00021	20
AX-157916315	2	53.466	KV453193.1	1952816	20-scf00021	20
AX-157916322	2	53.466	KV453193.1	1962579	20-scf00021	20
AX-157916332	2	53.466	KV453193.1	1970291	20-scf00021	20
AX-157916451	2	53.466	KV453193.1	2119527	20-scf00021	20
AX-157916452	2	53.466	KV453193.1	2120441	20-scf00021	20
AX-157916453	2	53.466	KV453193.1	2122482	20-scf00021	20
AX-157916648	2	53.466	KV453193.1	2427356	20-scf00021	20
AX-157916675	2	53.466	KV453193.1	2487484	20-scf00021	20
AX-157812791	2	53.466	KV453193.1	2835950	20-scf00021	20
AX-157916323	2	53.599	KV453193.1	1963739	20-scf00021	20
AX-157874707	2	53.684	KV453193.1	1971331	20-scf00021	20
AX-157916334	2	53.684	KV453193.1	1972157	20-scf00021	20
AX-157916335	2	53.684	KV453193.1	1975286	20-scf00021	20
AX-157916343	2	53.721	KV453193.1	1988711	20-scf00021	20
AX-157916348	2	53.757	KV453193.1	1995359	20-scf00021	20
AX-157916351	2	53.757	KV453193.1	1998982	20-scf00021	20
AX-157812754	2	53.757	KV453193.1	2208106	20-scf00021	20
AX-157916253	2	53.794	KV453193.1	1855507	20-scf00021	20
AX-157916318	2	53.794	KV453193.1	1956494	20-scf00021	20
AX-157916320	2	53.794	KV453193.1	1960314	20-scf00021	20
AX-157916326	2	53.794	KV453193.1	1965803	20-scf00021	20

AX-157916336	2	53.794	KV453193.1	1978155	20-scf00021	20
AX-157916360	2	53.794	KV453193.1	2007217	20-scf00021	20
AX-157916361	2	53.794	KV453193.1	2007572	20-scf00021	20
AX-157916364	2	53.794	KV453193.1	2012630	20-scf00021	20
AX-157916367	2	53.794	KV453193.1	2015960	20-scf00021	20
AX-157916368	2	53.794	KV453193.1	2016775	20-scf00021	20
AX-157916370	2	53.794	KV453193.1	2018529	20-scf00021	20
AX-157916375	2	53.794	KV453193.1	2022539	20-scf00021	20
AX-157916382	2	53.794	KV453193.1	2032862	20-scf00021	20
AX-157916384	2	53.794	KV453193.1	2034610	20-scf00021	20
AX-157916391	2	53.794	KV453193.1	2047256	20-scf00021	20
AX-157916392	2	53.794	KV453193.1	2047670	20-scf00021	20
AX-157916394	2	53.794	KV453193.1	2052551	20-scf00021	20
AX-157916402	2	53.794	KV453193.1	2061980	20-scf00021	20
AX-157916407	2	53.794	KV453193.1	2069254	20-scf00021	20
AX-157916408	2	53.794	KV453193.1	2069915	20-scf00021	20
AX-157916449	2	53.794	KV453193.1	2117677	20-scf00021	20
AX-157916418	2	53.847	KV453193.1	2079284	20-scf00021	20
AX-157720223	2	53.903	KV453193.1	1607294	20-scf00021	20
AX-157916067	2	53.903	KV453193.1	1616409	20-scf00021	20
AX-157916125	2	53.903	KV453193.1	1717848	20-scf00021	20
AX-157812712	2	53.903	KV453193.1	1724093	20-scf00021	20
AX-157916291	2	53.903	KV453193.1	1920488	20-scf00021	20
AX-157916331	2	53.903	KV453193.1	1969688	20-scf00021	20
AX-157916373	2	53.903	KV453193.1	2020666	20-scf00021	20
AX-157916374	2	53.903	KV453193.1	2021425	20-scf00021	20
AX-157916376	2	53.903	KV453193.1	2023284	20-scf00021	20
AX-157916377	2	53.903	KV453193.1	2023665	20-scf00021	20
AX-157916389	2	53.903	KV453193.1	2043532	20-scf00021	20
AX-157741059	2	53.903	KV453193.1	2054623	20-scf00021	20
AX-157916397	2	53.903	KV453193.1	2055658	20-scf00021	20
AX-157916412	2	53.903	KV453193.1	2074767	20-scf00021	20
AX-157916414	2	53.903	KV453193.1	2076573	20-scf00021	20
AX-157916421	2	53.903	KV453193.1	2081642	20-scf00021	20
AX-157916423	2	53.903	KV453193.1	2083394	20-scf00021	20
AX-157916426	2	53.903	KV453193.1	2084726	20-scf00021	20
AX-157916430	2	53.903	KV453193.1	2088858	20-scf00021	20
AX-157916431	2	53.903	KV453193.1	2090182	20-scf00021	20
AX-157916433	2	53.903	KV453193.1	2092560	20-scf00021	20
AX-157916435	2	53.903	KV453193.1	2093940	20-scf00021	20
AX-157916436	2	53.903	KV453193.1	2096765	20-scf00021	20
AX-157916437	2	53.903	KV453193.1	2097578	20-scf00021	20
AX-157916438	2	53.903	KV453193.1	2098122	20-scf00021	20
AX-157812739	2	53.903	KV453193.1	2103587	20-scf00021	20
AX-157916440	2	53.903	KV453193.1	2105660	20-scf00021	20
AX-157916446	2	53.903	KV453193.1	2113313	20-scf00021	20
AX-157916447	2	53.903	KV453193.1	2115161	20-scf00021	20
AX-157916450	2	53.903	KV453193.1	2118187	20-scf00021	20
AX-157916457	2	53.903	KV453193.1	2128448	20-scf00021	20
AX-157916460	2	53.903	KV453193.1	2132835	20-scf00021	20
AX-157916461	2	53.903	KV453193.1	2133504	20-scf00021	20

AX-157916462	2	53.903	KV453193.1	2134012	20-scf00021	20
AX-158405064	2	53.903	KV453193.1	2136412	20-scf00021	20
AX-157916464	2	53.903	KV453193.1	2137673	20-scf00021	20
AX-158389608	2	53.903	KV453193.1	2140185	20-scf00021	20
AX-157916466	2	53.903	KV453193.1	2140909	20-scf00021	20
AX-157812745	2	53.903	KV453193.1	2161171	20-scf00021	20
AX-157916480	2	53.903	KV453193.1	2165862	20-scf00021	20
AX-157916482	2	53.903	KV453193.1	2168697	20-scf00021	20
AX-157916495	2	53.903	KV453193.1	2182660	20-scf00021	20
AX-157916498	2	53.903	KV453193.1	2185097	20-scf00021	20
AX-157916504	2	53.903	KV453193.1	2191857	20-scf00021	20
AX-157916508	2	53.903	KV453193.1	2196159	20-scf00021	20
AX-157916517	2	53.903	KV453193.1	2205054	20-scf00021	20
AX-158382271	2	53.903	KV453193.1	2903266	20-scf00021	20
AX-157706587	2	53.903	3ML01032525	348	scf08147_1	UN
AX-157916259	2	54.012	KV453193.1	1865715	20-scf00021	20
AX-157753731	2	54.012	KV453193.1	2098776	20-scf00021	20
AX-157916476	2	54.012	KV453193.1	2156642	20-scf00021	20
AX-157718164	2	54.012	KV453193.1	2156794	20-scf00021	20
AX-157812743	2	54.012	KV453193.1	2159188	20-scf00021	20
AX-157916478	2	54.012	KV453193.1	2163268	20-scf00021	20
AX-157916479	2	54.012	KV453193.1	2164553	20-scf00021	20
AX-157742347	2	54.027	KV453193.1	2166714	20-scf00021	20
AX-157916333	2	54.072	KV453193.1	1970415	20-scf00021	20
AX-157916338	2	54.072	KV453193.1	1981015	20-scf00021	20
AX-157916442	2	54.072	KV453193.1	2108173	20-scf00021	20
AX-157916484	2	54.072	KV453193.1	2170069	20-scf00021	20
AX-157916505	2	54.072	KV453193.1	2192895	20-scf00021	20
AX-157916269	2	54.121	KV453193.1	1878405	20-scf00021	20
AX-157916316	2	54.121	KV453193.1	1954828	20-scf00021	20
AX-157916387	2	54.121	KV453193.1	2040576	20-scf00021	20
AX-157916455	2	54.121	KV453193.1	2126906	20-scf00021	20
AX-157916488	2	54.121	KV453193.1	2176190	20-scf00021	20
AX-157916490	2	54.121	KV453193.1	2177071	20-scf00021	20
AX-157916493	2	54.121	KV453193.1	2179549	20-scf00021	20
AX-157916494	2	54.121	KV453193.1	2181973	20-scf00021	20
AX-157916496	2	54.121	KV453193.1	2182805	20-scf00021	20
AX-157916497	2	54.121	KV453193.1	2184738	20-scf00021	20
AX-157916502	2	54.121	KV453193.1	2191314	20-scf00021	20
AX-157916503	2	54.121	KV453193.1	2191753	20-scf00021	20
AX-157916507	2	54.121	KV453193.1	2195438	20-scf00021	20
AX-157916509	2	54.121	KV453193.1	2196996	20-scf00021	20
AX-157748078	2	54.121	KV453193.1	2199087	20-scf00021	20
AX-157916512	2	54.121	KV453193.1	2201293	20-scf00021	20
AX-157916513	2	54.121	KV453193.1	2202808	20-scf00021	20
AX-157916514	2	54.121	KV453193.1	2203253	20-scf00021	20
AX-157916516	2	54.121	KV453193.1	2204287	20-scf00021	20
AX-157812753	2	54.121	KV453193.1	2204842	20-scf00021	20
AX-157916523	2	54.233	KV453193.1	2213392	20-scf00021	20
AX-157916528	2	54.233	KV453193.1	2218484	20-scf00021	20
AX-157812756	2	54.328	KV453193.1	2215405	20-scf00021	20

AX-157916535	2	54.328	KV453193.1	2228451	20-scf00021	20
AX-157710914	2	54.351	KV453193.1	2093365	20-scf00021	20
AX-157812760	2	54.351	KV453193.1	2232824	20-scf00021	20
AX-157916159	2	54.558	KV453193.1	1759773	20-scf00021	20
AX-157916410	2	54.558	KV453193.1	2073115	20-scf00021	20
AX-158389610	2	54.558	KV453193.1	2179384	20-scf00021	20
AX-157916530	2	54.558	KV453193.1	2219810	20-scf00021	20
AX-157916546	2	54.558	KV453193.1	2246586	20-scf00021	20
AX-157916547	2	54.558	KV453193.1	2249668	20-scf00021	20
AX-157916552	2	54.558	KV453193.1	2253893	20-scf00021	20
AX-157916555	2	54.558	KV453193.1	2256377	20-scf00021	20
AX-157916556	2	54.558	KV453193.1	2257418	20-scf00021	20
AX-157916557	2	54.558	KV453193.1	2258834	20-scf00021	20
AX-157916558	2	54.558	KV453193.1	2259735	20-scf00021	20
AX-157916559	2	54.558	KV453193.1	2260016	20-scf00021	20
AX-158389611	2	54.558	KV453193.1	2260942	20-scf00021	20
AX-157916560	2	54.558	KV453193.1	2261257	20-scf00021	20
AX-157916561	2	54.558	KV453193.1	2262431	20-scf00021	20
AX-157916562	2	54.558	KV453193.1	2262796	20-scf00021	20
AX-157916571	2	54.558	KV453193.1	2269296	20-scf00021	20
AX-157916575	2	54.558	KV453193.1	2275393	20-scf00021	20
AX-157916578	2	54.558	KV453193.1	2278456	20-scf00021	20
AX-157916591	2	54.558	KV453193.1	2304021	20-scf00021	20
AX-157773514	2	54.558	KV453193.1	2482249	20-scf00021	20
AX-157916681	2	54.558	KV453193.1	2499087	20-scf00021	20
AX-157916707	2	54.558	KV453193.1	2537565	20-scf00021	20
AX-157916724	2	54.558	KV453193.1	2555722	20-scf00021	20
AX-157916828	2	54.558	KV453193.1	2778714	20-scf00021	20
AX-157916894	2	54.558	KV453193.1	2869842	20-scf00021	20
AX-157916582	2	54.559	KV453193.1	2281409	20-scf00021	20
AX-157916282	2	54.56	KV453193.1	1901584	20-scf00021	20
AX-157916327	2	54.56	KV453193.1	1966251	20-scf00021	20
AX-157916434	2	54.56	KV453193.1	2093815	20-scf00021	20
AX-157916456	2	54.56	KV453193.1	2127369	20-scf00021	20
AX-157916587	2	54.56	KV453193.1	2291754	20-scf00021	20
AX-157916606	2	54.56	KV453193.1	2329479	20-scf00021	20
AX-157916607	2	54.56	KV453193.1	2330119	20-scf00021	20
AX-157916610	2	54.56	KV453193.1	2333437	20-scf00021	20
AX-157916615	2	54.56	KV453193.1	2341142	20-scf00021	20
AX-157916616	2	54.56	KV453193.1	2341840	20-scf00021	20
AX-157916617	2	54.56	KV453193.1	2342391	20-scf00021	20
AX-157705649	2	54.56	3ML01030519	363	scf06119_1	UN
AX-157916678	2	54.562	KV453193.1	2494753	20-scf00021	20
AX-158405079	2	54.563	KV453193.1	2496073	20-scf00021	20
AX-157916689	2	54.565	KV453193.1	2511803	20-scf00021	20
AX-157748651	2	54.628	KV453193.1	2229547	20-scf00021	20
AX-157916538	2	54.628	KV453193.1	2231934	20-scf00021	20
AX-157916573	2	54.628	KV453193.1	2272478	20-scf00021	20
AX-157812769	2	54.628	KV453193.1	2337747	20-scf00021	20
AX-157717553	2	54.628	KV453193.1	2347320	20-scf00021	20
AX-157916621	2	54.628	KV453193.1	2347474	20-scf00021	20

AX-157916635	2	54.628	KV453193.1	2405622	20-scf00021	20
AX-158405071	2	54.628	KV453193.1	2446026	20-scf00021	20
AX-158405073	2	54.628	KV453193.1	2446820	20-scf00021	20
AX-157916657	2	54.628	KV453193.1	2451314	20-scf00021	20
AX-157745133	2	54.628	KV453193.1	2452390	20-scf00021	20
AX-157916658	2	54.628	KV453193.1	2452607	20-scf00021	20
AX-157916660	2	54.628	KV453193.1	2458035	20-scf00021	20
AX-157754250	2	54.628	KV453193.1	2467216	20-scf00021	20
AX-157916690	2	54.628	KV453193.1	2515382	20-scf00021	20
AX-157916691	2	54.628	KV453193.1	2515936	20-scf00021	20
AX-157916697	2	54.628	KV453193.1	2524358	20-scf00021	20
AX-157916762	2	54.628	KV453193.1	2630839	20-scf00021	20
AX-157916766	2	54.628	KV453193.1	2638371	20-scf00021	20
AX-157916769	2	54.628	KV453193.1	2642943	20-scf00021	20
AX-157916772	2	54.628	KV453193.1	2645557	20-scf00021	20
AX-157916698	2	54.637	KV453193.1	2526284	20-scf00021	20
AX-158405080	2	54.648	KV453193.1	2534611	20-scf00021	20
AX-157916737	2	54.658	KV453193.1	2584038	20-scf00021	20
AX-157916694	2	54.661	KV453193.1	2521013	20-scf00021	20
AX-157916736	2	54.661	KV453193.1	2582327	20-scf00021	20
AX-157916738	2	54.661	KV453193.1	2586472	20-scf00021	20
AX-157916742	2	54.661	KV453193.1	2595070	20-scf00021	20
AX-158405083	2	54.661	KV453193.1	2612954	20-scf00021	20
AX-157916763	2	54.661	KV453193.1	2632635	20-scf00021	20
AX-157916739	2	54.665	KV453193.1	2588512	20-scf00021	20
AX-157916312	2	54.667	KV453193.1	1950779	20-scf00021	20
AX-157916344	2	54.667	KV453193.1	1990671	20-scf00021	20
AX-157916399	2	54.667	KV453193.1	2058330	20-scf00021	20
AX-157916404	2	54.667	KV453193.1	2064717	20-scf00021	20
AX-157916506	2	54.667	KV453193.1	2193674	20-scf00021	20
AX-157916524	2	54.667	KV453193.1	2214219	20-scf00021	20
AX-157916542	2	54.667	KV453193.1	2240275	20-scf00021	20
AX-157723257	2	54.667	KV453193.1	2241744	20-scf00021	20
AX-157916544	2	54.667	KV453193.1	2242727	20-scf00021	20
AX-157916550	2	54.667	KV453193.1	2252579	20-scf00021	20
AX-157916551	2	54.667	KV453193.1	2253295	20-scf00021	20
AX-157916564	2	54.667	KV453193.1	2264345	20-scf00021	20
AX-157916568	2	54.667	KV453193.1	2267023	20-scf00021	20
AX-157916572	2	54.667	KV453193.1	2269944	20-scf00021	20
AX-157916579	2	54.667	KV453193.1	2279281	20-scf00021	20
AX-157916580	2	54.667	KV453193.1	2279504	20-scf00021	20
AX-157916595	2	54.667	KV453193.1	2309042	20-scf00021	20
AX-157916601	2	54.667	KV453193.1	2318309	20-scf00021	20
AX-157812768	2	54.667	KV453193.1	2337461	20-scf00021	20
AX-157812770	2	54.667	KV453193.1	2355021	20-scf00021	20
AX-157916643	2	54.667	KV453193.1	2412468	20-scf00021	20
AX-157744325	2	54.667	KV453193.1	2417024	20-scf00021	20
AX-157773305	2	54.667	KV453193.1	2418664	20-scf00021	20
AX-157916644	2	54.667	KV453193.1	2419990	20-scf00021	20
AX-157750389	2	54.667	KV453193.1	2421143	20-scf00021	20
AX-157916646	2	54.667	KV453193.1	2422385	20-scf00021	20

AX-157916647	2	54.667	KV453193.1	2422749	20-scf00021	20
AX-157774472	2	54.667	KV453193.1	2423177	20-scf00021	20
AX-157812778	2	54.667	KV453193.1	2423479	20-scf00021	20
AX-157916662	2	54.667	KV453193.1	2460301	20-scf00021	20
AX-157916669	2	54.667	KV453193.1	2473886	20-scf00021	20
AX-157916670	2	54.667	KV453193.1	2474579	20-scf00021	20
AX-157883468	2	54.667	KV453193.1	2482859	20-scf00021	20
AX-157916680	2	54.667	KV453193.1	2497566	20-scf00021	20
AX-157916682	2	54.667	KV453193.1	2500206	20-scf00021	20
AX-157812784	2	54.667	KV453193.1	2508006	20-scf00021	20
AX-157916710	2	54.667	KV453193.1	2539568	20-scf00021	20
AX-157916711	2	54.667	KV453193.1	2540610	20-scf00021	20
AX-157916718	2	54.667	KV453193.1	2551552	20-scf00021	20
AX-157916725	2	54.667	KV453193.1	2556096	20-scf00021	20
AX-157916734	2	54.667	KV453193.1	2577419	20-scf00021	20
AX-157916745	2	54.667	KV453193.1	2596852	20-scf00021	20
AX-157916747	2	54.667	KV453193.1	2601651	20-scf00021	20
AX-157916750	2	54.667	KV453193.1	2603861	20-scf00021	20
AX-157916751	2	54.667	KV453193.1	2604717	20-scf00021	20
AX-157916752	2	54.667	KV453193.1	2606064	20-scf00021	20
AX-157916755	2	54.667	KV453193.1	2620314	20-scf00021	20
AX-157916756	2	54.667	KV453193.1	2621312	20-scf00021	20
AX-157916774	2	54.667	KV453193.1	2650135	20-scf00021	20
AX-157916778	2	54.667	KV453193.1	2653697	20-scf00021	20
AX-157916779	2	54.667	KV453193.1	2654759	20-scf00021	20
AX-157916790	2	54.667	KV453193.1	2673839	20-scf00021	20
AX-157812787	2	54.667	KV453193.1	2678579	20-scf00021	20
AX-157812788	2	54.667	KV453193.1	2682671	20-scf00021	20
AX-157916798	2	54.667	KV453193.1	2703239	20-scf00021	20
AX-157916799	2	54.667	KV453193.1	2703946	20-scf00021	20
AX-157916800	2	54.667	KV453193.1	2704462	20-scf00021	20
AX-157916801	2	54.667	KV453193.1	2705116	20-scf00021	20
AX-157745631	2	54.667	KV453193.1	2708259	20-scf00021	20
AX-157916807	2	54.667	KV453193.1	2728884	20-scf00021	20
AX-158405084	2	54.667	KV453193.1	2754723	20-scf00021	20
AX-157916814	2	54.667	KV453193.1	2765110	20-scf00021	20
AX-157916817	2	54.667	KV453193.1	2768367	20-scf00021	20
AX-157916822	2	54.667	KV453193.1	2773639	20-scf00021	20
AX-157916826	2	54.667	KV453193.1	2777447	20-scf00021	20
AX-157812789	2	54.667	KV453193.1	2777660	20-scf00021	20
AX-157916830	2	54.667	KV453193.1	2781681	20-scf00021	20
AX-157916832	2	54.667	KV453193.1	2784127	20-scf00021	20
AX-157916838	2	54.667	KV453193.1	2795546	20-scf00021	20
AX-157916843	2	54.667	KV453193.1	2798901	20-scf00021	20
AX-157916847	2	54.667	KV453193.1	2802414	20-scf00021	20
AX-157916848	2	54.667	KV453193.1	2802857	20-scf00021	20
AX-157916849	2	54.667	KV453193.1	2803611	20-scf00021	20
AX-157916852	2	54.667	KV453193.1	2804382	20-scf00021	20
AX-157916853	2	54.667	KV453193.1	2805380	20-scf00021	20
AX-157916854	2	54.667	KV453193.1	2807376	20-scf00021	20
AX-157916858	2	54.667	KV453193.1	2814604	20-scf00021	20

AX-157916861	2	54.667	KV453193.1	2819359	20-scf00021	20
AX-157916866	2	54.667	KV453193.1	2827612	20-scf00021	20
AX-157916870	2	54.667	KV453193.1	2833365	20-scf00021	20
AX-157916873	2	54.667	KV453193.1	2835218	20-scf00021	20
AX-157742446	2	54.667	KV453193.1	3015351	20-scf00021	20
AX-157695028	2	54.667	FI864260.1	599	ORI212 Ictalu	UN
AX-157916180	2	54.777	KV453193.1	1787995	20-scf00021	20
AX-157916347	2	54.777	KV453193.1	1993890	20-scf00021	20
AX-157916622	2	54.777	KV453193.1	2353698	20-scf00021	20
AX-157916626	2	54.777	KV453193.1	2358351	20-scf00021	20
AX-157916641	2	54.777	KV453193.1	2410562	20-scf00021	20
AX-157916728	2	54.777	KV453193.1	2567132	20-scf00021	20
AX-157916735	2	54.777	KV453193.1	2579601	20-scf00021	20
AX-157916770	2	54.777	KV453193.1	2644120	20-scf00021	20
AX-157916794	2	54.777	KV453193.1	2685605	20-scf00021	20
AX-157916813	2	54.777	KV453193.1	2752869	20-scf00021	20
AX-157916856	2	54.777	KV453193.1	2812566	20-scf00021	20
AX-157916869	2	54.777	KV453193.1	2832319	20-scf00021	20
AX-157916879	2	54.777	KV453193.1	2846845	20-scf00021	20
AX-157916808	2	54.886	KV453193.1	2732622	20-scf00021	20
AX-157916882	2	54.886	KV453193.1	2852012	20-scf00021	20
AX-157916883	2	54.886	KV453193.1	2852231	20-scf00021	20
AX-157717852	2	54.886	KV453193.1	2855899	20-scf00021	20
AX-157717002	2	54.886	KV453193.1	2857736	20-scf00021	20
AX-157916888	2	54.886	KV453193.1	2861307	20-scf00021	20
AX-157916889	2	54.886	KV453193.1	2861826	20-scf00021	20
AX-157916890	2	54.886	KV453193.1	2862382	20-scf00021	20
AX-157916892	2	54.886	KV453193.1	2868459	20-scf00021	20
AX-157916893	2	54.886	KV453193.1	2869159	20-scf00021	20
AX-157916933	2	54.886	KV453193.1	2928027	20-scf00021	20
AX-157916363	2	54.94	KV453193.1	2011516	20-scf00021	20
AX-157916365	2	54.94	KV453193.1	2014138	20-scf00021	20
AX-157916625	2	54.94	KV453193.1	2356074	20-scf00021	20
AX-157916761	2	54.94	KV453193.1	2628851	20-scf00021	20
AX-157916875	2	54.94	KV453193.1	2840176	20-scf00021	20
AX-157916897	2	54.94	KV453193.1	2873482	20-scf00021	20
AX-157916902	2	54.94	KV453193.1	2881957	20-scf00021	20
AX-157916567	2	54.995	KV453193.1	2266079	20-scf00021	20
AX-157812763	2	54.995	KV453193.1	2278959	20-scf00021	20
AX-157916589	2	54.995	KV453193.1	2300401	20-scf00021	20
AX-157916703	2	54.995	KV453193.1	2529814	20-scf00021	20
AX-157916705	2	54.995	KV453193.1	2531434	20-scf00021	20
AX-157916708	2	54.995	KV453193.1	2537918	20-scf00021	20
AX-157916709	2	54.995	KV453193.1	2538504	20-scf00021	20
AX-157916899	2	54.995	KV453193.1	2877160	20-scf00021	20
AX-157812793	2	54.995	KV453193.1	2883358	20-scf00021	20
AX-157916907	2	54.995	KV453193.1	2888912	20-scf00021	20
AX-157812794	2	54.995	KV453193.1	2889723	20-scf00021	20
AX-157916908	2	54.995	KV453193.1	2893319	20-scf00021	20
AX-157916909	2	54.995	KV453193.1	2894901	20-scf00021	20
AX-157916910	2	54.995	KV453193.1	2896983	20-scf00021	20

AX-157916912	2	54.995	KV453193.1	2898721	20-scf00021	20
AX-157916922	2	54.995	KV453193.1	2911817	20-scf00021	20
AX-157916926	2	54.995	KV453193.1	2918713	20-scf00021	20
AX-157917000	2	54.995	KV453193.1	2992689	20-scf00021	20
AX-157917022	2	54.995	KV453193.1	3014426	20-scf00021	20
AX-157917036	2	54.995	KV453193.1	3028604	20-scf00021	20
AX-157916913	2	55.097	KV453193.1	2900356	20-scf00021	20
AX-157812742	2	55.199	KV453193.1	2153992	20-scf00021	20
AX-157916916	2	55.199	KV453193.1	2903488	20-scf00021	20
AX-157916917	2	55.199	KV453193.1	2904060	20-scf00021	20
AX-157916919	2	55.199	KV453193.1	2905498	20-scf00021	20
AX-157916923	2	55.199	KV453193.1	2915229	20-scf00021	20
AX-157916957	2	55.199	KV453193.1	2954362	20-scf00021	20
AX-157723609	2	55.208	KV453193.1	2915771	20-scf00021	20
AX-157916918	2	55.218	KV453193.1	2904878	20-scf00021	20
AX-157916924	2	55.218	KV453193.1	2916669	20-scf00021	20
AX-157916914	2	55.228	KV453193.1	2901389	20-scf00021	20
AX-157703245	2	55.228	KV453193.1	2919244	20-scf00021	20
AX-157916928	2	55.228	KV453193.1	2920969	20-scf00021	20
AX-157916932	2	55.281	KV453193.1	2926329	20-scf00021	20
AX-157916935	2	55.432	KV453193.1	2929521	20-scf00021	20
AX-157916936	2	55.432	KV453193.1	2930796	20-scf00021	20
AX-157743562	2	55.432	KV453193.1	2933006	20-scf00021	20
AX-157916938	2	55.432	KV453193.1	2933603	20-scf00021	20
AX-157916939	2	55.432	KV453193.1	2934831	20-scf00021	20
AX-157916940	2	55.432	KV453193.1	2935904	20-scf00021	20
AX-157916941	2	55.432	KV453193.1	2937505	20-scf00021	20
AX-157916944	2	55.432	KV453193.1	2940841	20-scf00021	20
AX-157916945	2	55.432	KV453193.1	2941777	20-scf00021	20
AX-157916955	2	55.432	KV453193.1	2952872	20-scf00021	20
AX-157916960	2	55.432	KV453193.1	2955503	20-scf00021	20
AX-157916942	2	55.462	KV453193.1	2939152	20-scf00021	20
AX-157916948	2	55.462	KV453193.1	2944563	20-scf00021	20
AX-157916949	2	55.462	KV453193.1	2945909	20-scf00021	20
AX-158405092	2	55.462	KV453193.1	2961878	20-scf00021	20
AX-157917021	2	55.462	KV453193.1	3012709	20-scf00021	20
AX-157916931	2	55.501	KV453193.1	2925835	20-scf00021	20
AX-157883472	2	55.501	KV453193.1	2950153	20-scf00021	20
AX-157916952	2	55.501	KV453193.1	2951058	20-scf00021	20
AX-157916953	2	55.541	KV453193.1	2951617	20-scf00021	20
AX-157916958	2	55.541	KV453193.1	2954522	20-scf00021	20
AX-157916959	2	55.541	KV453193.1	2954966	20-scf00021	20
AX-157916961	2	55.541	KV453193.1	2955893	20-scf00021	20
AX-157916962	2	55.541	KV453193.1	2957531	20-scf00021	20
AX-157916963	2	55.541	KV453193.1	2961151	20-scf00021	20
AX-157916964	2	55.541	KV453193.1	2963020	20-scf00021	20
AX-157916966	2	55.541	KV453193.1	2965004	20-scf00021	20
AX-157916969	2	55.65	KV453193.1	2967030	20-scf00021	20
AX-157916970	2	55.677	KV453193.1	2967525	20-scf00021	20
AX-157916973	2	55.714	KV453193.1	2970085	20-scf00021	20
AX-157916975	2	55.714	KV453193.1	2972482	20-scf00021	20

AX-157916992	2	55.714	KV453193.1	2986860	20-scf00021	20
AX-157916993	2	55.714	KV453193.1	2987139	20-scf00021	20
AX-157916995	2	55.714	KV453193.1	2989187	20-scf00021	20
AX-157917017	2	55.714	KV453193.1	3006944	20-scf00021	20
AX-157916978	2	55.76	KV453193.1	2974033	20-scf00021	20
AX-157916930	2	55.839	KV453193.1	2924965	20-scf00021	20
AX-157916981	2	55.839	KV453193.1	2976959	20-scf00021	20
AX-157916988	2	55.839	KV453193.1	2983530	20-scf00021	20
AX-157916989	2	55.978	KV453193.1	2984109	20-scf00021	20
AX-157916997	2	55.978	KV453193.1	2990429	20-scf00021	20
AX-157916991	2	56.088	KV453193.1	2986339	20-scf00021	20
AX-157916999	2	56.088	KV453193.1	2992441	20-scf00021	20
AX-157917011	2	56.088	KV453193.1	3003928	20-scf00021	20
AX-157917014	2	56.088	KV453193.1	3005927	20-scf00021	20
AX-157917015	2	56.088	KV453193.1	3006361	20-scf00021	20
AX-157917019	2	56.088	KV453193.1	3008300	20-scf00021	20
AX-157917025	2	56.088	KV453193.1	3017213	20-scf00021	20
AX-157917030	2	56.088	KV453193.1	3022974	20-scf00021	20
AX-157917031	2	56.201	KV453193.1	3023481	20-scf00021	20
AX-157714067	2	56.306	KV453193.1	3033452	20-scf00021	20
AX-157917038	2	56.306	KV453193.1	3035027	20-scf00021	20
AX-157917054	2	56.306	KV453193.1	3055795	20-scf00021	20
AX-157717676	2	56.306	KV453193.1	3089269	20-scf00021	20
AX-157812808	2	56.306	KV453193.1	3119039	20-scf00021	20
AX-157917099	2	56.306	KV453193.1	3119483	20-scf00021	20
AX-157917046	2	56.349	KV453193.1	3046877	20-scf00021	20
AX-157917051	2	56.382	KV453193.1	3052348	20-scf00021	20
AX-157917086	2	56.382	KV453193.1	3099047	20-scf00021	20
AX-157812709	2	56.415	KV453193.1	1693675	20-scf00021	20
AX-157916147	2	56.415	KV453193.1	1748533	20-scf00021	20
AX-157916531	2	56.415	KV453193.1	2220226	20-scf00021	20
AX-157916614	2	56.415	KV453193.1	2339956	20-scf00021	20
AX-157916686	2	56.415	KV453193.1	2503161	20-scf00021	20
AX-157812792	2	56.415	KV453193.1	2842562	20-scf00021	20
AX-157916895	2	56.415	KV453193.1	2870750	20-scf00021	20
AX-157917016	2	56.415	KV453193.1	3006742	20-scf00021	20
AX-157917028	2	56.415	KV453193.1	3019209	20-scf00021	20
AX-157917034	2	56.415	KV453193.1	3026755	20-scf00021	20
AX-157917040	2	56.415	KV453193.1	3040137	20-scf00021	20
AX-157917041	2	56.415	KV453193.1	3040520	20-scf00021	20
AX-157917045	2	56.415	KV453193.1	3045550	20-scf00021	20
AX-157812797	2	56.415	KV453193.1	3046970	20-scf00021	20
AX-157917050	2	56.415	KV453193.1	3051649	20-scf00021	20
AX-157917052	2	56.415	KV453193.1	3054374	20-scf00021	20
AX-157917053	2	56.415	KV453193.1	3054607	20-scf00021	20
AX-157917056	2	56.415	KV453193.1	3060178	20-scf00021	20
AX-157917057	2	56.415	KV453193.1	3060440	20-scf00021	20
AX-157917058	2	56.415	KV453193.1	3060653	20-scf00021	20
AX-157812799	2	56.415	KV453193.1	3062310	20-scf00021	20
AX-157917060	2	56.415	KV453193.1	3062926	20-scf00021	20
AX-157917061	2	56.415	KV453193.1	3063315	20-scf00021	20

AX-157812801	2	56.415	KV453193.1	3065041	20-scf00021	20
AX-157917062	2	56.415	KV453193.1	3065548	20-scf00021	20
AX-157917063	2	56.415	KV453193.1	3065703	20-scf00021	20
AX-157917064	2	56.415	KV453193.1	3066574	20-scf00021	20
AX-157917065	2	56.415	KV453193.1	3067686	20-scf00021	20
AX-157917067	2	56.415	KV453193.1	3071561	20-scf00021	20
AX-157917068	2	56.415	KV453193.1	3072024	20-scf00021	20
AX-157917071	2	56.415	KV453193.1	3074751	20-scf00021	20
AX-157917072	2	56.415	KV453193.1	3075174	20-scf00021	20
AX-157917075	2	56.415	KV453193.1	3076083	20-scf00021	20
AX-157812802	2	56.415	KV453193.1	3076396	20-scf00021	20
AX-158379267	2	56.415	KV453193.1	3080869	20-scf00021	20
AX-157917083	2	56.415	KV453193.1	3091761	20-scf00021	20
AX-157812804	2	56.415	KV453193.1	3098602	20-scf00021	20
AX-157812805	2	56.415	KV453193.1	3098778	20-scf00021	20
AX-157812806	2	56.415	KV453193.1	3101065	20-scf00021	20
AX-157715427	2	56.415	KV453193.1	3106771	20-scf00021	20
AX-157917093	2	56.415	KV453193.1	3113160	20-scf00021	20
AX-157812807	2	56.415	KV453193.1	3114534	20-scf00021	20
AX-157917097	2	56.415	KV453193.1	3117543	20-scf00021	20
AX-157917098	2	56.415	KV453193.1	3118480	20-scf00021	20
AX-157713713	2	56.415	KV453193.1	3122162	20-scf00021	20
AX-157812810	2	56.415	KV453193.1	3124364	20-scf00021	20
AX-157917104	2	56.415	KV453193.1	3129837	20-scf00021	20
AX-157853736	2	56.415	KV453193.1	3130239	20-scf00021	20
AX-157917105	2	56.415	KV453193.1	3130498	20-scf00021	20
AX-157741795	2	56.415	KV453193.1	3132615	20-scf00021	20
AX-157812811	2	56.415	KV453193.1	3134975	20-scf00021	20
AX-157751678	2	56.415	KV453193.1	3135312	20-scf00021	20
AX-157917106	2	56.415	KV453193.1	3135694	20-scf00021	20
AX-157812812	2	56.415	KV453193.1	3136888	20-scf00021	20
AX-157917108	2	56.415	KV453193.1	3137517	20-scf00021	20
AX-157917110	2	56.415	KV453193.1	3140258	20-scf00021	20
AX-157917133	2	56.415	KV453193.1	3166009	20-scf00021	20
AX-157917181	2	56.415	KV453193.1	3224616	20-scf00021	20
AX-157716125	2	56.634	KV453193.1	3091036	20-scf00021	20
AX-157917111	2	56.634	KV453193.1	3142063	20-scf00021	20
AX-157917112	2	56.634	KV453193.1	3143326	20-scf00021	20
AX-157714884	2	56.634	KV453193.1	3143962	20-scf00021	20
AX-157917113	2	56.634	KV453193.1	3144139	20-scf00021	20
AX-157917118	2	56.634	KV453193.1	3149279	20-scf00021	20
AX-157917120	2	56.634	KV453193.1	3150409	20-scf00021	20
AX-157917130	2	56.634	KV453193.1	3164012	20-scf00021	20
AX-157917121	2	56.743	KV453193.1	3152536	20-scf00021	20
AX-157917122	2	56.743	KV453193.1	3154303	20-scf00021	20
AX-157917128	2	56.743	KV453193.1	3163038	20-scf00021	20
AX-157917129	2	56.743	KV453193.1	3163802	20-scf00021	20
AX-157917131	2	56.743	KV453193.1	3164375	20-scf00021	20
AX-157917137	2	56.743	KV453193.1	3169034	20-scf00021	20
AX-157917153	2	56.743	KV453193.1	3180701	20-scf00021	20
AX-157917187	2	56.743	KV453193.1	3232553	20-scf00021	20

AX-157917138	2	56.785	KV453193.1	3169818	20-scf00021	20
AX-157917140	2	56.818	KV453193.1	3170612	20-scf00021	20
AX-157874709	2	56.818	KV453193.1	3171985	20-scf00021	20
AX-157917215	2	56.818	KV453193.1	3262394	20-scf00021	20
AX-157917146	2	56.852	KV453193.1	3174833	20-scf00021	20
AX-157917154	2	56.881	KV453193.1	3181369	20-scf00021	20
AX-157917160	2	56.917	KV453193.1	3197178	20-scf00021	20
AX-157916173	2	56.962	KV453193.1	1781469	20-scf00021	20
AX-157917066	2	56.962	KV453193.1	3070580	20-scf00021	20
AX-157917074	2	56.962	KV453193.1	3075762	20-scf00021	20
AX-157917089	2	56.962	KV453193.1	3100481	20-scf00021	20
AX-157917119	2	56.962	KV453193.1	3149997	20-scf00021	20
AX-157917162	2	56.962	KV453193.1	3197881	20-scf00021	20
AX-157812817	2	56.962	KV453193.1	3200248	20-scf00021	20
AX-157917167	2	56.962	KV453193.1	3205813	20-scf00021	20
AX-157917170	2	56.962	KV453193.1	3209639	20-scf00021	20
AX-158379268	2	56.962	KV453193.1	3210721	20-scf00021	20
AX-157917172	2	56.962	KV453193.1	3210787	20-scf00021	20
AX-157750870	2	56.962	KV453193.1	3212282	20-scf00021	20
AX-157917174	2	56.962	KV453193.1	3213464	20-scf00021	20
AX-157917175	2	56.962	KV453193.1	3214567	20-scf00021	20
AX-157917176	2	56.962	KV453193.1	3215147	20-scf00021	20
AX-158382272	2	56.962	KV453193.1	3215541	20-scf00021	20
AX-157720697	2	56.962	KV453193.1	3220687	20-scf00021	20
AX-157812818	2	56.962	KV453193.1	3220982	20-scf00021	20
AX-157917182	2	56.962	KV453193.1	3226036	20-scf00021	20
AX-157917184	2	56.962	KV453193.1	3230942	20-scf00021	20
AX-157917185	2	56.962	KV453193.1	3231292	20-scf00021	20
AX-157917197	2	56.962	KV453193.1	3246145	20-scf00021	20
AX-157917201	2	56.962	KV453193.1	3250685	20-scf00021	20
AX-157917203	2	56.962	KV453193.1	3252542	20-scf00021	20
AX-157917206	2	56.962	KV453193.1	3254782	20-scf00021	20
AX-157917207	2	56.962	KV453193.1	3256725	20-scf00021	20
AX-157917212	2	56.962	KV453193.1	3259436	20-scf00021	20
AX-157917213	2	56.962	KV453193.1	3259778	20-scf00021	20
AX-157917220	2	56.962	KV453193.1	3268121	20-scf00021	20
AX-157917275	2	56.962	KV453193.1	3366706	20-scf00021	20
AX-157917366	2	56.962	KV453193.1	3476605	20-scf00021	20
AX-157917396	2	56.962	KV453193.1	3512845	20-scf00021	20
AX-157706518	2	56.962	3ML01032367	348	scf07984_1	UN
AX-157917217	2	57.003	KV453193.1	3263461	20-scf00021	20
AX-157812825	2	57.021	KV453193.1	3275675	20-scf00021	20
AX-157917232	2	57.052	KV453193.1	3288872	20-scf00021	20
AX-157917135	2	57.071	KV453193.1	3167704	20-scf00021	20
AX-157917155	2	57.071	KV453193.1	3183045	20-scf00021	20
AX-157917183	2	57.071	KV453193.1	3226667	20-scf00021	20
AX-157812824	2	57.071	KV453193.1	3263105	20-scf00021	20
AX-157853739	2	57.071	KV453193.1	3266138	20-scf00021	20
AX-157917233	2	57.071	KV453193.1	3290131	20-scf00021	20
AX-157812828	2	57.071	KV453193.1	3295057	20-scf00021	20
AX-157917236	2	57.071	KV453193.1	3296412	20-scf00021	20

AX-157917147	2	57.18	KV453193.1	3175379	20-scf00021	20
AX-157917179	2	57.18	KV453193.1	3221664	20-scf00021	20
AX-157917204	2	57.18	KV453193.1	3253385	20-scf00021	20
AX-157917239	2	57.18	KV453193.1	3306703	20-scf00021	20
AX-157917240	2	57.18	KV453193.1	3307317	20-scf00021	20
AX-157917242	2	57.18	KV453193.1	3312103	20-scf00021	20
AX-158405096	2	57.18	KV453193.1	3313375	20-scf00021	20
AX-157917243	2	57.18	KV453193.1	3313514	20-scf00021	20
AX-157917246	2	57.18	KV453193.1	3317146	20-scf00021	20
AX-157917249	2	57.18	KV453193.1	3322254	20-scf00021	20
AX-157917250	2	57.18	KV453193.1	3323694	20-scf00021	20
AX-157917254	2	57.18	KV453193.1	3331801	20-scf00021	20
AX-157917255	2	57.18	KV453193.1	3333579	20-scf00021	20
AX-157917257	2	57.18	KV453193.1	3334566	20-scf00021	20
AX-157753313	2	57.18	KV453193.1	3336925	20-scf00021	20
AX-157917262	2	57.18	KV453193.1	3344007	20-scf00021	20
AX-157917287	2	57.18	KV453193.1	3374442	20-scf00021	20
AX-157917294	2	57.18	KV453193.1	3385019	20-scf00021	20
AX-157917300	2	57.18	KV453193.1	3391401	20-scf00021	20
AX-156402670	2	57.18	KV453193.1	3461640	20-scf00021	20
AX-157917219	2	57.199	KV453193.1	3264631	20-scf00021	20
AX-157812832	2	57.199	KV453193.1	3350376	20-scf00021	20
AX-157812833	2	57.199	KV453193.1	3350867	20-scf00021	20
AX-157917272	2	57.199	KV453193.1	3362031	20-scf00021	20
AX-158379269	2	57.199	KV453193.1	3372995	20-scf00021	20
AX-157917271	2	57.23	KV453193.1	3359812	20-scf00021	20
AX-157917259	2	57.25	KV453193.1	3337648	20-scf00021	20
AX-157917268	2	57.25	KV453193.1	3355991	20-scf00021	20
AX-157917276	2	57.25	KV453193.1	3367334	20-scf00021	20
AX-157689636	2	57.25	3ML01030984	391	scf06588_1	UN
AX-157917277	2	57.269	KV453193.1	3367735	20-scf00021	20
AX-157917152	2	57.289	KV453193.1	3179939	20-scf00021	20
AX-157917171	2	57.289	KV453193.1	3210408	20-scf00021	20
AX-157812823	2	57.289	KV453193.1	3256354	20-scf00021	20
AX-157812837	2	57.289	KV453193.1	3358951	20-scf00021	20
AX-157917274	2	57.289	KV453193.1	3363411	20-scf00021	20
AX-157917280	2	57.289	KV453193.1	3369387	20-scf00021	20
AX-157917283	2	57.289	KV453193.1	3370469	20-scf00021	20
AX-157917285	2	57.289	KV453193.1	3371906	20-scf00021	20
AX-157724536	2	57.289	KV453193.1	3372283	20-scf00021	20
AX-157917289	2	57.289	KV453193.1	3378852	20-scf00021	20
AX-157695260	2	57.289	KV453193.1	3379673	20-scf00021	20
AX-157917293	2	57.289	KV453193.1	3382641	20-scf00021	20
AX-158405097	2	57.289	KV453193.1	3383867	20-scf00021	20
AX-157917297	2	57.289	KV453193.1	3388662	20-scf00021	20
AX-158421899	2	57.289	KV453193.1	3416218	20-scf00021	20
AX-157917344	2	57.289	KV453193.1	3443770	20-scf00021	20
AX-157917350	2	57.289	KV453193.1	3459048	20-scf00021	20
AX-157917354	2	57.289	KV453193.1	3466738	20-scf00021	20
AX-157917301	2	57.339	KV453193.1	3391871	20-scf00021	20
AX-157757068	2	57.383	KV453193.1	3214085	20-scf00021	20

AX-157917305	2	57.383	KV453193.1	3394338	20-scf00021	20
AX-157917327	2	57.383	KV453193.1	3425962	20-scf00021	20
AX-157917224	2	57.425	KV453193.1	3273622	20-scf00021	20
AX-157917282	2	57.425	KV453193.1	3370311	20-scf00021	20
AX-157917299	2	57.425	KV453193.1	3389797	20-scf00021	20
AX-157917308	2	57.425	KV453193.1	3398149	20-scf00021	20
AX-157917309	2	57.464	KV453193.1	3399150	20-scf00021	20
AX-157917340	2	57.464	KV453193.1	3441109	20-scf00021	20
AX-157812820	2	57.508	KV453193.1	3233073	20-scf00021	20
AX-157917198	2	57.508	KV453193.1	3247321	20-scf00021	20
AX-157721996	2	57.508	KV453193.1	3268982	20-scf00021	20
AX-157917310	2	57.508	KV453193.1	3399536	20-scf00021	20
AX-157917311	2	57.508	KV453193.1	3401298	20-scf00021	20
AX-157917312	2	57.508	KV453193.1	3401990	20-scf00021	20
AX-157917313	2	57.508	KV453193.1	3403073	20-scf00021	20
AX-157917314	2	57.508	KV453193.1	3404188	20-scf00021	20
AX-157812839	2	57.508	KV453193.1	3405706	20-scf00021	20
AX-157917315	2	57.508	KV453193.1	3406018	20-scf00021	20
AX-157812840	2	57.508	KV453193.1	3407350	20-scf00021	20
AX-157917316	2	57.508	KV453193.1	3407814	20-scf00021	20
AX-157917318	2	57.508	KV453193.1	3409350	20-scf00021	20
AX-157853741	2	57.508	KV453193.1	3410806	20-scf00021	20
AX-158405098	2	57.508	KV453193.1	3413450	20-scf00021	20
AX-157917321	2	57.508	KV453193.1	3413618	20-scf00021	20
AX-157917322	2	57.508	KV453193.1	3416513	20-scf00021	20
AX-157917324	2	57.508	KV453193.1	3417285	20-scf00021	20
AX-157917328	2	57.508	KV453193.1	3426314	20-scf00021	20
AX-157917329	2	57.508	KV453193.1	3426750	20-scf00021	20
AX-157917333	2	57.508	KV453193.1	3432853	20-scf00021	20
AX-157917334	2	57.508	KV453193.1	3433779	20-scf00021	20
AX-157812841	2	57.508	KV453193.1	3435430	20-scf00021	20
AX-157917335	2	57.508	KV453193.1	3435971	20-scf00021	20
AX-157917336	2	57.508	KV453193.1	3436277	20-scf00021	20
AX-157812842	2	57.508	KV453193.1	3438137	20-scf00021	20
AX-157917338	2	57.508	KV453193.1	3438512	20-scf00021	20
AX-157746154	2	57.508	KV453193.1	3439137	20-scf00021	20
AX-157917341	2	57.508	KV453193.1	3441351	20-scf00021	20
AX-157917345	2	57.508	KV453193.1	3448264	20-scf00021	20
AX-157917347	2	57.508	KV453193.1	3456892	20-scf00021	20
AX-157812845	2	57.508	KV453193.1	3457377	20-scf00021	20
AX-157917348	2	57.508	KV453193.1	3457623	20-scf00021	20
AX-157917349	2	57.508	KV453193.1	3458339	20-scf00021	20
AX-157917351	2	57.508	KV453193.1	3459531	20-scf00021	20
AX-157917352	2	57.508	KV453193.1	3460690	20-scf00021	20
AX-157917356	2	57.508	KV453193.1	3467661	20-scf00021	20
AX-157917357	2	57.508	KV453193.1	3468507	20-scf00021	20
AX-157917359	2	57.508	KV453193.1	3471171	20-scf00021	20
AX-157917361	2	57.508	KV453193.1	3472970	20-scf00021	20
AX-157917364	2	57.508	KV453193.1	3475325	20-scf00021	20
AX-157917365	2	57.671	KV453193.1	3475818	20-scf00021	20
AX-157917368	2	57.731	KV453193.1	3477611	20-scf00021	20

AX-157917370	2	57.78	KV453193.1	3478335	20-scf00021	20
AX-157812850	2	57.78	KV453193.1	3558779	20-scf00021	20
AX-157812868	2	57.78	KV453193.1	3764794	20-scf00021	20
AX-157917372	2	57.835	KV453193.1	3486966	20-scf00021	20
AX-157917373	2	57.835	KV453193.1	3488054	20-scf00021	20
AX-157917374	2	57.835	KV453193.1	3488991	20-scf00021	20
AX-157917358	2	57.945	KV453193.1	3470107	20-scf00021	20
AX-157917376	2	57.945	KV453193.1	3491292	20-scf00021	20
AX-157917377	2	57.945	KV453193.1	3492804	20-scf00021	20
AX-157738498	2	58.054	KV453193.1	3488477	20-scf00021	20
AX-157917379	2	58.054	KV453193.1	3494471	20-scf00021	20
AX-157917380	2	58.054	KV453193.1	3495526	20-scf00021	20
AX-157917382	2	58.054	KV453193.1	3498250	20-scf00021	20
AX-157917383	2	58.054	KV453193.1	3498743	20-scf00021	20
AX-157917385	2	58.054	KV453193.1	3499973	20-scf00021	20
AX-157917387	2	58.054	KV453193.1	3502165	20-scf00021	20
AX-157917388	2	58.054	KV453193.1	3503350	20-scf00021	20
AX-157917389	2	58.054	KV453193.1	3504373	20-scf00021	20
AX-157917390	2	58.054	KV453193.1	3507290	20-scf00021	20
AX-157917391	2	58.054	KV453193.1	3508348	20-scf00021	20
AX-157917393	2	58.054	KV453193.1	3509044	20-scf00021	20
AX-157917395	2	58.054	KV453193.1	3512018	20-scf00021	20
AX-157917397	2	58.054	KV453193.1	3513554	20-scf00021	20
AX-157917410	2	58.054	KV453193.1	3524962	20-scf00021	20
AX-157917422	2	58.054	KV453193.1	3534827	20-scf00021	20
AX-157917425	2	58.054	KV453193.1	3540213	20-scf00021	20
AX-157917429	2	58.054	KV453193.1	3548286	20-scf00021	20
AX-157917400	2	58.163	KV453193.1	3516020	20-scf00021	20
AX-157917403	2	58.163	KV453193.1	3518184	20-scf00021	20
AX-157917409	2	58.252	KV453193.1	3524610	20-scf00021	20
AX-157917416	2	58.328	KV453193.1	3529390	20-scf00021	20
AX-157917420	2	58.328	KV453193.1	3533134	20-scf00021	20
AX-157917426	2	58.328	KV453193.1	3542365	20-scf00021	20
AX-157853744	2	58.328	KV453193.1	3545422	20-scf00021	20
AX-157917427	2	58.328	KV453193.1	3546932	20-scf00021	20
AX-158405104	2	58.328	KV453193.1	3547401	20-scf00021	20
AX-157812847	2	58.49	KV453193.1	3550547	20-scf00021	20
AX-157812849	2	58.491	KV453193.1	3552895	20-scf00021	20
AX-157715690	2	58.491	KV453193.1	3552984	20-scf00021	20
AX-157917434	2	58.491	KV453193.1	3555504	20-scf00021	20
AX-157812852	2	58.491	KV453193.1	3584173	20-scf00021	20
AX-157812855	2	58.491	KV453193.1	3613187	20-scf00021	20
AX-157917488	2	58.491	KV453193.1	3621925	20-scf00021	20
AX-157917437	2	58.54	KV453193.1	3557294	20-scf00021	20
AX-157812865	2	58.54	KV453193.1	3668997	20-scf00021	20
AX-157917441	2	58.6	KV453193.1	3563311	20-scf00021	20
AX-157917442	2	58.6	KV453193.1	3566113	20-scf00021	20
AX-157917460	2	58.6	KV453193.1	3590259	20-scf00021	20
AX-157917448	2	58.627	KV453193.1	3575174	20-scf00021	20
AX-157727883	2	58.627	KV453193.1	3854117	20-scf00021	20
AX-157917469	2	58.649	KV453193.1	3598531	20-scf00021	20

AX-157757879	2	58.649	KV453193.1	3613914	20-scf00021	20
AX-157812854	2	58.673	KV453193.1	3603597	20-scf00021	20
AX-157917475	2	58.695	KV453193.1	3606736	20-scf00021	20
AX-157917462	2	58.71	KV453193.1	3591228	20-scf00021	20
AX-157917472	2	58.71	KV453193.1	3603036	20-scf00021	20
AX-157917476	2	58.71	KV453193.1	3607035	20-scf00021	20
AX-157917478	2	58.71	KV453193.1	3608005	20-scf00021	20
AX-157917479	2	58.71	KV453193.1	3608243	20-scf00021	20
AX-157917480	2	58.71	KV453193.1	3609251	20-scf00021	20
AX-157742093	2	58.71	KV453193.1	3611262	20-scf00021	20
AX-157917484	2	58.71	KV453193.1	3614302	20-scf00021	20
AX-157917493	2	58.817	KV453193.1	3629080	20-scf00021	20
AX-157917494	2	58.928	KV453193.1	3629776	20-scf00021	20
AX-157917496	2	58.928	KV453193.1	3633872	20-scf00021	20
AX-157917500	2	58.928	KV453193.1	3637141	20-scf00021	20
AX-157917501	2	58.966	KV453193.1	3637897	20-scf00021	20
AX-157917503	2	58.992	KV453193.1	3638945	20-scf00021	20
AX-157917412	2	59.038	KV453193.1	3527147	20-scf00021	20
AX-157917430	2	59.038	KV453193.1	3550237	20-scf00021	20
AX-157917495	2	59.038	KV453193.1	3631914	20-scf00021	20
AX-157917499	2	59.038	KV453193.1	3636471	20-scf00021	20
AX-157917504	2	59.038	KV453193.1	3639568	20-scf00021	20
AX-157917505	2	59.038	KV453193.1	3639898	20-scf00021	20
AX-157917507	2	59.038	KV453193.1	3641208	20-scf00021	20
AX-157917508	2	59.038	KV453193.1	3641775	20-scf00021	20
AX-157812858	2	59.038	KV453193.1	3649006	20-scf00021	20
AX-157812859	2	59.038	KV453193.1	3649392	20-scf00021	20
AX-157917514	2	59.038	KV453193.1	3651112	20-scf00021	20
AX-157917521	2	59.038	KV453193.1	3663643	20-scf00021	20
AX-157812863	2	59.038	KV453193.1	3667680	20-scf00021	20
AX-157812864	2	59.038	KV453193.1	3668509	20-scf00021	20
AX-157917524	2	59.038	KV453193.1	3671260	20-scf00021	20
AX-157917560	2	59.038	KV453193.1	3740895	20-scf00021	20
AX-157917707	2	59.038	KV453193.1	3964690	20-scf00021	20
AX-157746995	2	59.057	KV453193.1	3681145	20-scf00021	20
AX-157812857	2	59.075	KV453193.1	3644911	20-scf00021	20
AX-157917534	2	59.075	KV453193.1	3690434	20-scf00021	20
AX-157917543	2	59.075	KV453193.1	3709137	20-scf00021	20
AX-157917405	2	59.086	KV453193.1	3520408	20-scf00021	20
AX-157917423	2	59.086	KV453193.1	3535372	20-scf00021	20
AX-157748590	2	59.086	KV453193.1	3656079	20-scf00021	20
AX-157917528	2	59.086	KV453193.1	3678146	20-scf00021	20
AX-157917531	2	59.086	KV453193.1	3683608	20-scf00021	20
AX-157754487	2	59.086	KV453193.1	3690481	20-scf00021	20
AX-157917538	2	59.086	KV453193.1	3696055	20-scf00021	20
AX-157744267	2	59.105	KV453193.1	3581691	20-scf00021	20
AX-157864513	2	59.105	KV453193.1	3584889	20-scf00021	20
AX-157917539	2	59.105	KV453193.1	3697552	20-scf00021	20
AX-157917540	2	59.105	KV453193.1	3706380	20-scf00021	20
AX-157917554	2	59.105	KV453193.1	3729205	20-scf00021	20
AX-157883489	2	59.105	KV453193.1	4299911	20-scf00021	20

AX-157917548	2	59.119	KV453193.1	3722754	20-scf00021	20
AX-157917556	2	59.147	KV453193.1	3731458	20-scf00021	20
AX-157917557	2	59.147	KV453193.1	3734057	20-scf00021	20
AX-157917563	2	59.147	KV453193.1	3745515	20-scf00021	20
AX-157917564	2	59.147	KV453193.1	3747719	20-scf00021	20
AX-157917570	2	59.173	KV453193.1	3754972	20-scf00021	20
AX-157917581	2	59.173	KV453193.1	3774812	20-scf00021	20
AX-157917574	2	59.198	KV453193.1	3759782	20-scf00021	20
AX-157917572	2	59.223	KV453193.1	3756886	20-scf00021	20
AX-157917575	2	59.223	KV453193.1	3760909	20-scf00021	20
AX-157917473	2	59.24	KV453193.1	3604399	20-scf00021	20
AX-157917578	2	59.24	KV453193.1	3769921	20-scf00021	20
AX-157917421	2	59.256	KV453193.1	3533499	20-scf00021	20
AX-157917523	2	59.256	KV453193.1	3668937	20-scf00021	20
AX-157717942	2	59.256	KV453193.1	3780899	20-scf00021	20
AX-157917584	2	59.256	KV453193.1	3782563	20-scf00021	20
AX-157917585	2	59.323	KV453193.1	3783728	20-scf00021	20
AX-158405108	2	59.365	KV453193.1	3711731	20-scf00021	20
AX-157744021	2	59.365	KV453193.1	3715842	20-scf00021	20
AX-157917579	2	59.365	KV453193.1	3771682	20-scf00021	20
AX-157917582	2	59.365	KV453193.1	3776694	20-scf00021	20
AX-157917586	2	59.365	KV453193.1	3784594	20-scf00021	20
AX-157917587	2	59.365	KV453193.1	3788617	20-scf00021	20
AX-157917589	2	59.365	KV453193.1	3794177	20-scf00021	20
AX-157917596	2	59.365	KV453193.1	3802455	20-scf00021	20
AX-157772771	2	59.365	KV453193.1	3802870	20-scf00021	20
AX-157917597	2	59.365	KV453193.1	3804918	20-scf00021	20
AX-157917599	2	59.365	KV453193.1	3805905	20-scf00021	20
AX-157853746	2	59.365	KV453193.1	3810393	20-scf00021	20
AX-157812869	2	59.365	KV453193.1	3826198	20-scf00021	20
AX-157917611	2	59.365	KV453193.1	3827536	20-scf00021	20
AX-157741615	2	59.365	KV453193.1	3828895	20-scf00021	20
AX-157917614	2	59.365	KV453193.1	3833787	20-scf00021	20
AX-157917616	2	59.365	KV453193.1	3837597	20-scf00021	20
AX-157812870	2	59.365	KV453193.1	3838499	20-scf00021	20
AX-157812871	2	59.365	KV453193.1	3848851	20-scf00021	20
AX-157812872	2	59.365	KV453193.1	3850845	20-scf00021	20
AX-157917623	2	59.365	KV453193.1	3851484	20-scf00021	20
AX-157812873	2	59.365	KV453193.1	3852598	20-scf00021	20
AX-157748792	2	59.365	KV453193.1	3852885	20-scf00021	20
AX-157812874	2	59.365	KV453193.1	3852926	20-scf00021	20
AX-157812875	2	59.365	KV453193.1	3853030	20-scf00021	20
AX-157917624	2	59.365	KV453193.1	3853920	20-scf00021	20
AX-157751838	2	59.365	KV453193.1	3858130	20-scf00021	20
AX-157917626	2	59.365	KV453193.1	3861408	20-scf00021	20
AX-157917627	2	59.365	KV453193.1	3861725	20-scf00021	20
AX-157917630	2	59.365	KV453193.1	3864374	20-scf00021	20
AX-157917631	2	59.365	KV453193.1	3866528	20-scf00021	20
AX-158405114	2	59.365	KV453193.1	3867744	20-scf00021	20
AX-157917634	2	59.365	KV453193.1	3869298	20-scf00021	20
AX-157917641	2	59.365	KV453193.1	3878832	20-scf00021	20

AX-157917642	2	59.365	KV453193.1	3879306	20-scf00021	20
AX-157917644	2	59.365	KV453193.1	3881525	20-scf00021	20
AX-157917645	2	59.365	KV453193.1	3882177	20-scf00021	20
AX-157917646	2	59.365	KV453193.1	3883509	20-scf00021	20
AX-157917647	2	59.365	KV453193.1	3884525	20-scf00021	20
AX-157917693	2	59.365	KV453193.1	3946503	20-scf00021	20
AX-157812881	2	59.365	KV453193.1	3948498	20-scf00021	20
AX-157719658	2	59.365	KV453193.1	4012229	20-scf00021	20
AX-157917801	2	59.365	KV453193.1	4183722	20-scf00021	20
AX-157917655	2	59.399	KV453193.1	3897358	20-scf00021	20
AX-157917657	2	59.43	KV453193.1	3898671	20-scf00021	20
AX-157917466	2	59.474	KV453193.1	3596105	20-scf00021	20
AX-157917467	2	59.474	KV453193.1	3597202	20-scf00021	20
AX-157864514	2	59.474	KV453193.1	3609956	20-scf00021	20
AX-157917512	2	59.474	KV453193.1	3646827	20-scf00021	20
AX-157696578	2	59.474	KV453193.1	3691216	20-scf00021	20
AX-157917536	2	59.474	KV453193.1	3691800	20-scf00021	20
AX-157717550	2	59.474	KV453193.1	3705451	20-scf00021	20
AX-157874713	2	59.474	KV453193.1	3709215	20-scf00021	20
AX-157917555	2	59.474	KV453193.1	3730481	20-scf00021	20
AX-157917559	2	59.474	KV453193.1	3738441	20-scf00021	20
AX-157883482	2	59.474	KV453193.1	3752968	20-scf00021	20
AX-157917603	2	59.474	KV453193.1	3810739	20-scf00021	20
AX-157917615	2	59.474	KV453193.1	3837057	20-scf00021	20
AX-157917639	2	59.474	KV453193.1	3875869	20-scf00021	20
AX-157917648	2	59.474	KV453193.1	3884870	20-scf00021	20
AX-157812877	2	59.474	KV453193.1	3891385	20-scf00021	20
AX-157917660	2	59.474	KV453193.1	3904881	20-scf00021	20
AX-157917661	2	59.474	KV453193.1	3907716	20-scf00021	20
AX-157917665	2	59.474	KV453193.1	3912960	20-scf00021	20
AX-157917666	2	59.474	KV453193.1	3914780	20-scf00021	20
AX-157917668	2	59.474	KV453193.1	3917885	20-scf00021	20
AX-157917669	2	59.474	KV453193.1	3918383	20-scf00021	20
AX-157917670	2	59.474	KV453193.1	3919288	20-scf00021	20
AX-157917672	2	59.474	KV453193.1	3922614	20-scf00021	20
AX-157917676	2	59.474	KV453193.1	3927185	20-scf00021	20
AX-157917677	2	59.474	KV453193.1	3929725	20-scf00021	20
AX-157917682	2	59.474	KV453193.1	3934676	20-scf00021	20
AX-157812879	2	59.474	KV453193.1	3937135	20-scf00021	20
AX-157812880	2	59.474	KV453193.1	3938239	20-scf00021	20
AX-157716003	2	59.474	KV453193.1	3938299	20-scf00021	20
AX-157917687	2	59.474	KV453193.1	3942671	20-scf00021	20
AX-157917691	2	59.474	KV453193.1	3945555	20-scf00021	20
AX-157917692	2	59.474	KV453193.1	3946134	20-scf00021	20
AX-157917695	2	59.474	KV453193.1	3949033	20-scf00021	20
AX-157917696	2	59.474	KV453193.1	3950004	20-scf00021	20
AX-157917697	2	59.474	KV453193.1	3952498	20-scf00021	20
AX-158404294	2	59.474	KV453193.1	3954616	20-scf00021	20
AX-157917698	2	59.474	KV453193.1	3954878	20-scf00021	20
AX-157717539	2	59.474	KV453193.1	3955284	20-scf00021	20
AX-157917700	2	59.474	KV453193.1	3956588	20-scf00021	20

AX-157917702	2	59.474	KV453193.1	3960219	20-scf00021	20
AX-157812887	2	59.474	KV453193.1	3964131	20-scf00021	20
AX-157917708	2	59.474	KV453193.1	3964932	20-scf00021	20
AX-157917712	2	59.474	KV453193.1	3968839	20-scf00021	20
AX-157772160	2	59.474	KV453193.1	3970249	20-scf00021	20
AX-157812888	2	59.474	KV453193.1	3970614	20-scf00021	20
AX-157917719	2	59.474	KV453193.1	3992023	20-scf00021	20
AX-157749354	2	59.474	KV453193.1	4004770	20-scf00021	20
AX-157917731	2	59.474	KV453193.1	4012664	20-scf00021	20
AX-157917741	2	59.474	KV453193.1	4091307	20-scf00021	20
AX-157713823	2	59.474	KV453193.1	4183481	20-scf00021	20
AX-157917820	2	59.474	KV453193.1	4209033	20-scf00021	20
AX-158405123	2	59.474	KV453193.1	4238219	20-scf00021	20
AX-157917865	2	59.474	KV453193.1	4264763	20-scf00021	20
AX-157742170	2	59.547	KV453193.1	3984515	20-scf00021	20
AX-157917418	2	59.584	KV453193.1	3531449	20-scf00021	20
AX-157917550	2	59.584	KV453193.1	3724557	20-scf00021	20
AX-157917640	2	59.584	KV453193.1	3876468	20-scf00021	20
AX-157917688	2	59.584	KV453193.1	3943542	20-scf00021	20
AX-157812884	2	59.584	KV453193.1	3961140	20-scf00021	20
AX-157812889	2	59.584	KV453193.1	3986067	20-scf00021	20
AX-157812890	2	59.584	KV453193.1	3990208	20-scf00021	20
AX-157775717	2	59.584	KV453193.1	4005731	20-scf00021	20
AX-157917723	2	59.584	KV453193.1	4007016	20-scf00021	20
AX-157917724	2	59.584	KV453193.1	4007403	20-scf00021	20
AX-157812891	2	59.584	KV453193.1	4007738	20-scf00021	20
AX-157917725	2	59.584	KV453193.1	4007994	20-scf00021	20
AX-157917727	2	59.584	KV453193.1	4009298	20-scf00021	20
AX-157917728	2	59.584	KV453193.1	4009584	20-scf00021	20
AX-157917737	2	59.584	KV453193.1	4086658	20-scf00021	20
AX-157917740	2	59.584	KV453193.1	4089183	20-scf00021	20
AX-158405117	2	59.584	KV453193.1	4090643	20-scf00021	20
AX-157917742	2	59.584	KV453193.1	4091681	20-scf00021	20
AX-157745295	2	59.584	KV453193.1	4092210	20-scf00021	20
AX-157812893	2	59.584	KV453193.1	4092511	20-scf00021	20
AX-157917744	2	59.723	KV453193.1	4096411	20-scf00021	20
AX-157917654	2	59.802	KV453193.1	3894277	20-scf00021	20
AX-157917658	2	59.802	KV453193.1	3898833	20-scf00021	20
AX-157917662	2	59.802	KV453193.1	3908700	20-scf00021	20
AX-157917680	2	59.802	KV453193.1	3932639	20-scf00021	20
AX-157718247	2	59.802	KV453193.1	4009240	20-scf00021	20
AX-157917736	2	59.802	KV453193.1	4086320	20-scf00021	20
AX-157917743	2	59.802	KV453193.1	4095432	20-scf00021	20
AX-157917746	2	59.802	KV453193.1	4098870	20-scf00021	20
AX-158405120	2	59.802	KV453193.1	4099654	20-scf00021	20
AX-157917748	2	59.802	KV453193.1	4103324	20-scf00021	20
AX-157917751	2	59.802	KV453193.1	4108915	20-scf00021	20
AX-157917752	2	59.802	KV453193.1	4109339	20-scf00021	20
AX-157917755	2	59.802	KV453193.1	4111344	20-scf00021	20
AX-157917757	2	59.802	KV453193.1	4112540	20-scf00021	20
AX-157917759	2	59.802	KV453193.1	4114801	20-scf00021	20

AX-157917761	2	59.802	KV453193.1	4118200	20-scf00021	20
AX-157917762	2	59.802	KV453193.1	4119624	20-scf00021	20
AX-157725438	2	59.802	KV453193.1	4120604	20-scf00021	20
AX-157917765	2	59.802	KV453193.1	4123580	20-scf00021	20
AX-157853748	2	59.802	KV453193.1	4129126	20-scf00021	20
AX-157917769	2	59.802	KV453193.1	4132127	20-scf00021	20
AX-157917772	2	59.802	KV453193.1	4134219	20-scf00021	20
AX-157917773	2	59.802	KV453193.1	4135006	20-scf00021	20
AX-157917774	2	59.802	KV453193.1	4135357	20-scf00021	20
AX-157917775	2	59.802	KV453193.1	4136852	20-scf00021	20
AX-157749677	2	59.802	KV453193.1	4164024	20-scf00021	20
AX-157917791	2	59.802	KV453193.1	4169081	20-scf00021	20
AX-157917794	2	59.802	KV453193.1	4171963	20-scf00021	20
AX-157917799	2	59.802	KV453193.1	4180403	20-scf00021	20
AX-157752266	2	59.802	KV453193.1	4192273	20-scf00021	20
AX-157917822	2	59.802	KV453193.1	4210177	20-scf00021	20
AX-157812901	2	59.802	KV453193.1	4218803	20-scf00021	20
AX-157812907	2	59.802	KV453193.1	4321110	20-scf00021	20
AX-157917913	2	59.802	KV453193.1	4335212	20-scf00021	20
AX-157917928	2	59.802	KV453193.1	4359259	20-scf00021	20
AX-157680314	2	59.802	BML01030984	188	scf06588_1	UN
AX-157917659	2	60.021	KV453193.1	3902527	20-scf00021	20
AX-157917771	2	60.021	KV453193.1	4133838	20-scf00021	20
AX-157716169	2	60.021	KV453193.1	4191618	20-scf00021	20
AX-157917812	2	60.021	KV453193.1	4203576	20-scf00021	20
AX-157758359	2	60.021	KV453193.1	4205180	20-scf00021	20
AX-157917814	2	60.021	KV453193.1	4205225	20-scf00021	20
AX-157917816	2	60.021	KV453193.1	4205883	20-scf00021	20
AX-157917819	2	60.021	KV453193.1	4207776	20-scf00021	20
AX-157917821	2	60.021	KV453193.1	4209475	20-scf00021	20
AX-157917823	2	60.021	KV453193.1	4211520	20-scf00021	20
AX-157917824	2	60.021	KV453193.1	4212230	20-scf00021	20
AX-157917825	2	60.021	KV453193.1	4213587	20-scf00021	20
AX-157738349	2	60.021	KV453193.1	4216003	20-scf00021	20
AX-157917828	2	60.021	KV453193.1	4216661	20-scf00021	20
AX-157917830	2	60.021	KV453193.1	4218241	20-scf00021	20
AX-157917831	2	60.021	KV453193.1	4219333	20-scf00021	20
AX-157716040	2	60.021	KV453193.1	4221217	20-scf00021	20
AX-157917835	2	60.021	KV453193.1	4226266	20-scf00021	20
AX-157917846	2	60.021	KV453193.1	4236698	20-scf00021	20
AX-157917887	2	60.021	KV453193.1	4308200	20-scf00021	20
AX-157917894	2	60.021	KV453193.1	4316797	20-scf00021	20
AX-157917839	2	60.077	KV453193.1	4230805	20-scf00021	20
AX-157917840	2	60.077	KV453193.1	4231176	20-scf00021	20
AX-157917841	2	60.13	KV453193.1	4232604	20-scf00021	20
AX-157917458	2	60.348	KV453193.1	3589166	20-scf00021	20
AX-157917516	2	60.348	KV453193.1	3653201	20-scf00021	20
AX-157917549	2	60.348	KV453193.1	3723901	20-scf00021	20
AX-157917602	2	60.348	KV453193.1	3810100	20-scf00021	20
AX-158389619	2	60.348	KV453193.1	3862926	20-scf00021	20
AX-157917632	2	60.348	KV453193.1	3868490	20-scf00021	20

AX-157917689	2	60.348	KV453193.1	3944507	20-scf00021	20
AX-157917711	2	60.348	KV453193.1	3967560	20-scf00021	20
AX-157917749	2	60.348	KV453193.1	4105684	20-scf00021	20
AX-157917750	2	60.348	KV453193.1	4108507	20-scf00021	20
AX-157778196	2	60.348	KV453193.1	4179013	20-scf00021	20
AX-157720514	2	60.348	KV453193.1	4182659	20-scf00021	20
AX-157917817	2	60.348	KV453193.1	4206632	20-scf00021	20
AX-157917829	2	60.348	KV453193.1	4217603	20-scf00021	20
AX-157917844	2	60.348	KV453193.1	4235328	20-scf00021	20
AX-157917845	2	60.348	KV453193.1	4236451	20-scf00021	20
AX-157720926	2	60.348	KV453193.1	4239126	20-scf00021	20
AX-157917850	2	60.348	KV453193.1	4242794	20-scf00021	20
AX-157917851	2	60.348	KV453193.1	4244889	20-scf00021	20
AX-157917855	2	60.348	KV453193.1	4248250	20-scf00021	20
AX-157917856	2	60.348	KV453193.1	4250338	20-scf00021	20
AX-157917860	2	60.348	KV453193.1	4255178	20-scf00021	20
AX-157812902	2	60.348	KV453193.1	4265174	20-scf00021	20
AX-157917866	2	60.348	KV453193.1	4265216	20-scf00021	20
AX-158414846	2	60.348	KV453193.1	4268364	20-scf00021	20
AX-157917868	2	60.348	KV453193.1	4268777	20-scf00021	20
AX-158389623	2	60.348	KV453193.1	4269213	20-scf00021	20
AX-157719614	2	60.348	KV453193.1	4270382	20-scf00021	20
AX-157917871	2	60.348	KV453193.1	4274604	20-scf00021	20
AX-157917872	2	60.348	KV453193.1	4275225	20-scf00021	20
AX-157917874	2	60.348	KV453193.1	4276761	20-scf00021	20
AX-157917877	2	60.348	KV453193.1	4280116	20-scf00021	20
AX-157713258	2	60.348	KV453193.1	4282559	20-scf00021	20
AX-157917892	2	60.348	KV453193.1	4315456	20-scf00021	20
AX-157706276	2	60.348	3ML01031978	90	scf07595_1	UN
AX-157706681	2	60.348	3ML01032730	101	scf08357_1	UN
AX-157706682	2	60.348	3ML01032730	258	scf08357_1	UN
AX-157718422	2	60.457	KV453193.1	4292171	20-scf00021	20
AX-157917883	2	60.457	KV453193.1	4294704	20-scf00021	20
AX-157917886	2	60.457	KV453193.1	4305117	20-scf00021	20
AX-157749990	2	60.457	KV453193.1	4307215	20-scf00021	20
AX-157812906	2	60.457	KV453193.1	4309706	20-scf00021	20
AX-158405127	2	60.457	KV453193.1	4316062	20-scf00021	20
AX-157917896	2	60.457	KV453193.1	4317935	20-scf00021	20
AX-157917903	2	60.457	KV453193.1	4326562	20-scf00021	20
AX-157917912	2	60.457	KV453193.1	4334935	20-scf00021	20
AX-157917848	2	60.56	KV453193.1	4239866	20-scf00021	20
AX-157917849	2	60.56	KV453193.1	4240217	20-scf00021	20
AX-157917918	2	60.56	KV453193.1	4340064	20-scf00021	20
AX-157917923	2	60.56	KV453193.1	4346303	20-scf00021	20
AX-157918048	2	60.56	KV453193.1	4503485	20-scf00021	20
AX-157748314	2	60.676	KV453193.1	4309392	20-scf00021	20
AX-157917920	2	60.676	KV453193.1	4340818	20-scf00021	20
AX-157917922	2	60.676	KV453193.1	4344316	20-scf00021	20
AX-157917925	2	60.676	KV453193.1	4350717	20-scf00021	20
AX-157917927	2	60.676	KV453193.1	4356508	20-scf00021	20
AX-157752089	2	60.676	KV453193.1	4358191	20-scf00021	20

AX-157917929	2	60.676	KV453193.1	4367261	20-scf00021	20
AX-157917930	2	60.676	KV453193.1	4367364	20-scf00021	20
AX-157917936	2	60.676	KV453193.1	4371047	20-scf00021	20
AX-157812912	2	60.676	KV453193.1	4374083	20-scf00021	20
AX-157917942	2	60.676	KV453193.1	4376016	20-scf00021	20
AX-157917944	2	60.676	KV453193.1	4376460	20-scf00021	20
AX-157917947	2	60.676	KV453193.1	4379161	20-scf00021	20
AX-157917951	2	60.7	KV453193.1	4383304	20-scf00021	20
AX-157917945	2	60.709	KV453193.1	4377674	20-scf00021	20
AX-157883493	2	60.709	KV453193.1	4379784	20-scf00021	20
AX-157917958	2	60.709	KV453193.1	4392575	20-scf00021	20
AX-157917961	2	60.747	KV453193.1	4396478	20-scf00021	20
AX-157917965	2	60.785	KV453193.1	4400478	20-scf00021	20
AX-157917967	2	60.82	KV453193.1	4402476	20-scf00021	20
AX-157753986	2	60.855	KV453193.1	4259868	20-scf00021	20
AX-157917904	2	60.855	KV453193.1	4328531	20-scf00021	20
AX-157917937	2	60.855	KV453193.1	4371683	20-scf00021	20
AX-157714745	2	60.855	KV453193.1	4403562	20-scf00021	20
AX-157812914	2	60.855	KV453193.1	4403658	20-scf00021	20
AX-157812915	2	60.855	KV453193.1	4406367	20-scf00021	20
AX-157917941	2	60.894	KV453193.1	4374453	20-scf00021	20
AX-157864519	2	60.894	KV453193.1	4380312	20-scf00021	20
AX-157917966	2	60.894	KV453193.1	4401838	20-scf00021	20
AX-157917968	2	60.894	KV453193.1	4406753	20-scf00021	20
AX-157917969	2	60.894	KV453193.1	4407498	20-scf00021	20
AX-157917973	2	60.894	KV453193.1	4415199	20-scf00021	20
AX-157917974	2	60.894	KV453193.1	4416244	20-scf00021	20
AX-157917975	2	60.894	KV453193.1	4419260	20-scf00021	20
AX-157917976	2	60.894	KV453193.1	4420395	20-scf00021	20
AX-157917979	2	60.894	KV453193.1	4422818	20-scf00021	20
AX-157917980	2	60.894	KV453193.1	4425710	20-scf00021	20
AX-157917982	2	60.894	KV453193.1	4426830	20-scf00021	20
AX-157917983	2	60.894	KV453193.1	4427317	20-scf00021	20
AX-157917984	2	60.894	KV453193.1	4427754	20-scf00021	20
AX-157917985	2	60.953	KV453193.1	4431544	20-scf00021	20
AX-157917962	2	61.004	KV453193.1	4397313	20-scf00021	20
AX-157917988	2	61.004	KV453193.1	4434493	20-scf00021	20
AX-157917990	2	61.004	KV453193.1	4437586	20-scf00021	20
AX-157752115	2	61.004	KV453193.1	4438478	20-scf00021	20
AX-157917991	2	61.004	KV453193.1	4439331	20-scf00021	20
AX-157918004	2	61.004	KV453193.1	4451739	20-scf00021	20
AX-158405132	2	61.004	KV453193.1	4495446	20-scf00021	20
AX-157853751	2	61.044	KV453193.1	4334225	20-scf00021	20
AX-157917939	2	61.044	KV453193.1	4372439	20-scf00021	20
AX-157917957	2	61.044	KV453193.1	4392129	20-scf00021	20
AX-157917986	2	61.044	KV453193.1	4433142	20-scf00021	20
AX-157917992	2	61.044	KV453193.1	4439997	20-scf00021	20
AX-157918014	2	61.044	KV453193.1	4463890	20-scf00021	20
AX-157917607	2	61.113	KV453193.1	3815129	20-scf00021	20
AX-157917622	2	61.113	KV453193.1	3848377	20-scf00021	20
AX-157917993	2	61.113	KV453193.1	4440710	20-scf00021	20

AX-157917994	2	61.113	KV453193.1	4441090	20-scf00021	20
AX-157917997	2	61.113	KV453193.1	4444744	20-scf00021	20
AX-157917998	2	61.113	KV453193.1	4446534	20-scf00021	20
AX-157918005	2	61.113	KV453193.1	4452465	20-scf00021	20
AX-157918006	2	61.113	KV453193.1	4454927	20-scf00021	20
AX-157918007	2	61.113	KV453193.1	4455349	20-scf00021	20
AX-157918008	2	61.113	KV453193.1	4455938	20-scf00021	20
AX-158404302	2	61.113	KV453193.1	4457576	20-scf00021	20
AX-157918012	2	61.113	KV453193.1	4461599	20-scf00021	20
AX-157918013	2	61.113	KV453193.1	4463068	20-scf00021	20
AX-157812918	2	61.113	KV453193.1	4465827	20-scf00021	20
AX-157918016	2	61.113	KV453193.1	4472654	20-scf00021	20
AX-157918018	2	61.113	KV453193.1	4473679	20-scf00021	20
AX-157918019	2	61.113	KV453193.1	4474170	20-scf00021	20
AX-157918030	2	61.113	KV453193.1	4488484	20-scf00021	20
AX-157812920	2	61.113	KV453193.1	4499154	20-scf00021	20
AX-157918162	2	61.113	KV453193.1	4669112	20-scf00021	20
AX-157917893	2	61.222	KV453193.1	4316446	20-scf00021	20
AX-157722647	2	61.222	KV453193.1	4334161	20-scf00021	20
AX-157918022	2	61.222	KV453193.1	4480123	20-scf00021	20
AX-157918026	2	61.222	KV453193.1	4483474	20-scf00021	20
AX-157918027	2	61.222	KV453193.1	4485770	20-scf00021	20
AX-157918028	2	61.222	KV453193.1	4487510	20-scf00021	20
AX-157918029	2	61.222	KV453193.1	4487898	20-scf00021	20
AX-157750712	2	61.222	KV453193.1	4490059	20-scf00021	20
AX-157918032	2	61.222	KV453193.1	4492439	20-scf00021	20
AX-157918033	2	61.222	KV453193.1	4492589	20-scf00021	20
AX-157918036	2	61.222	KV453193.1	4495365	20-scf00021	20
AX-157918037	2	61.222	KV453193.1	4496249	20-scf00021	20
AX-157864522	2	61.222	KV453193.1	4496557	20-scf00021	20
AX-157918038	2	61.222	KV453193.1	4497266	20-scf00021	20
AX-157918040	2	61.222	KV453193.1	4498336	20-scf00021	20
AX-157918041	2	61.222	KV453193.1	4499599	20-scf00021	20
AX-157716431	2	61.222	KV453193.1	4508958	20-scf00021	20
AX-157918044	2	61.223	KV453193.1	4501524	20-scf00021	20
AX-157918062	2	61.466	KV453193.1	4535140	20-scf00021	20
AX-157918063	2	61.466	KV453193.1	4536164	20-scf00021	20
AX-157918064	2	61.466	KV453193.1	4536533	20-scf00021	20
AX-157812921	2	61.466	KV453193.1	4540092	20-scf00021	20
AX-157918151	2	61.466	KV453193.1	4660009	20-scf00021	20
AX-157918200	2	61.466	KV453193.1	4726672	20-scf00021	20
AX-157714360	2	61.466	KV453193.1	4736232	20-scf00021	20
AX-157707452	2	61.466	3ML01034314	196	scf09986_1	UN
AX-157812922	2	61.525	KV453193.1	4542037	20-scf00021	20
AX-157918066	2	61.768	KV453193.1	4543414	20-scf00021	20
AX-157918068	2	61.768	KV453193.1	4544797	20-scf00021	20
AX-157918074	2	61.768	KV453193.1	4554897	20-scf00021	20
AX-157812933	2	61.768	KV453193.1	4670210	20-scf00021	20
AX-157918070	2	61.769	KV453193.1	4550258	20-scf00021	20
AX-157918075	2	61.769	KV453193.1	4555569	20-scf00021	20
AX-158405133	2	61.769	KV453193.1	4557003	20-scf00021	20

AX-157869122	2	61.769	KV453193.1	4559410	20-scf00021	20
AX-157918080	2	61.769	KV453193.1	4560644	20-scf00021	20
AX-157918081	2	61.769	KV453193.1	4560701	20-scf00021	20
AX-157918082	2	61.769	KV453193.1	4561765	20-scf00021	20
AX-157918083	2	61.769	KV453193.1	4563462	20-scf00021	20
AX-157774673	2	61.769	KV453193.1	4566354	20-scf00021	20
AX-157918087	2	61.769	KV453193.1	4566566	20-scf00021	20
AX-157918089	2	61.769	KV453193.1	4571350	20-scf00021	20
AX-157918104	2	61.769	KV453193.1	4589346	20-scf00021	20
AX-157812934	2	61.769	KV453193.1	4676739	20-scf00021	20
AX-157707545	2	61.769	3ML01034524	437	scf10201_1	UN
AX-157918079	2	61.878	KV453193.1	4560217	20-scf00021	20
AX-157918085	2	61.878	KV453193.1	4565512	20-scf00021	20
AX-157918088	2	61.878	KV453193.1	4567701	20-scf00021	20
AX-157918091	2	61.878	KV453193.1	4572743	20-scf00021	20
AX-157771287	2	61.878	KV453193.1	4574059	20-scf00021	20
AX-157918092	2	61.878	KV453193.1	4574118	20-scf00021	20
AX-157918095	2	61.878	KV453193.1	4576532	20-scf00021	20
AX-157918096	2	61.878	KV453193.1	4577139	20-scf00021	20
AX-157918099	2	61.878	KV453193.1	4583386	20-scf00021	20
AX-157918100	2	61.878	KV453193.1	4584375	20-scf00021	20
AX-157918102	2	61.878	KV453193.1	4586436	20-scf00021	20
AX-157918103	2	61.918	KV453193.1	4587449	20-scf00021	20
AX-157918106	2	61.918	KV453193.1	4592461	20-scf00021	20
AX-157918184	2	61.918	KV453193.1	4696683	20-scf00021	20
AX-157918105	2	61.957	KV453193.1	4590705	20-scf00021	20
AX-157714921	2	61.987	KV453193.1	4594680	20-scf00021	20
AX-157812923	2	62.03	KV453193.1	4597766	20-scf00021	20
AX-157918110	2	62.03	KV453193.1	4606960	20-scf00021	20
AX-157918109	2	62.097	KV453193.1	4606113	20-scf00021	20
AX-157918111	2	62.097	KV453193.1	4607269	20-scf00021	20
AX-157918112	2	62.097	KV453193.1	4607490	20-scf00021	20
AX-157812926	2	62.097	KV453193.1	4607974	20-scf00021	20
AX-157918114	2	62.097	KV453193.1	4610114	20-scf00021	20
AX-157918115	2	62.097	KV453193.1	4611477	20-scf00021	20
AX-158382281	2	62.097	KV453193.1	4624721	20-scf00021	20
AX-157812929	2	62.097	KV453193.1	4628753	20-scf00021	20
AX-157918174	2	62.097	KV453193.1	4686529	20-scf00021	20
AX-157918204	2	62.097	KV453193.1	4734819	20-scf00021	20
AX-157918078	2	62.098	KV453193.1	4559122	20-scf00021	20
AX-157918101	2	62.098	KV453193.1	4585813	20-scf00021	20
AX-157812927	2	62.098	KV453193.1	4616592	20-scf00021	20
AX-157715382	2	62.098	KV453193.1	4617265	20-scf00021	20
AX-157918143	2	62.098	KV453193.1	4651814	20-scf00021	20
AX-157720507	2	62.099	KV453193.1	4623264	20-scf00021	20
AX-157918121	2	62.099	KV453193.1	4628563	20-scf00021	20
AX-157918125	2	62.099	KV453193.1	4633403	20-scf00021	20
AX-157771765	2	62.099	KV453193.1	4656995	20-scf00021	20
AX-158405137	2	62.099	KV453193.1	4687731	20-scf00021	20
AX-157918183	2	62.099	KV453193.1	4696114	20-scf00021	20
AX-157918187	2	62.099	KV453193.1	4703272	20-scf00021	20

AX-157918189	2	62.099	KV453193.1	4704848	20-scf00021	20
AX-157918215	2	62.099	KV453193.1	4747762	20-scf00021	20
AX-157918127	2	62.26	KV453193.1	4636956	20-scf00021	20
AX-157918129	2	62.305	KV453193.1	4637934	20-scf00021	20
AX-157918132	2	62.425	KV453193.1	4640675	20-scf00021	20
AX-157918141	2	62.425	KV453193.1	4649955	20-scf00021	20
AX-157918142	2	62.425	KV453193.1	4650521	20-scf00021	20
AX-157918144	2	62.425	KV453193.1	4653042	20-scf00021	20
AX-157918148	2	62.425	KV453193.1	4656419	20-scf00021	20
AX-157918150	2	62.425	KV453193.1	4659025	20-scf00021	20
AX-157751791	2	62.425	KV453193.1	4659645	20-scf00021	20
AX-157918153	2	62.425	KV453193.1	4661402	20-scf00021	20
AX-157918155	2	62.425	KV453193.1	4664783	20-scf00021	20
AX-157918161	2	62.425	KV453193.1	4668689	20-scf00021	20
AX-157918172	2	62.425	KV453193.1	4683286	20-scf00021	20
AX-157918177	2	62.425	KV453193.1	4689109	20-scf00021	20
AX-157918178	2	62.425	KV453193.1	4690930	20-scf00021	20
AX-157918202	2	62.425	KV453193.1	4728535	20-scf00021	20
AX-157710065	2	62.444	KV453193.1	4608342	20-scf00021	20
AX-158405136	2	62.47	KV453193.1	4686880	20-scf00021	20
AX-157918181	2	62.47	KV453193.1	4694276	20-scf00021	20
AX-157869124	2	62.534	KV453193.1	4699907	20-scf00021	20
AX-157707453	2	62.534	KV453193.1	4701005	20-scf00021	20
AX-157918191	2	62.535	KV453193.1	4706480	20-scf00021	20
AX-157918194	2	62.535	KV453193.1	4715153	20-scf00021	20
AX-158405142	2	62.535	KV453193.1	4729296	20-scf00021	20
AX-157918197	2	62.536	KV453193.1	4719345	20-scf00021	20
AX-157918201	2	62.537	KV453193.1	4728177	20-scf00021	20
AX-158405145	2	62.61	KV453193.1	4729875	20-scf00021	20
AX-157918210	2	62.61	KV453193.1	4742474	20-scf00021	20
AX-157812939	2	62.681	KV453193.1	4731401	20-scf00021	20
AX-157918203	2	62.681	KV453193.1	4732905	20-scf00021	20
AX-157918206	2	62.681	KV453193.1	4737892	20-scf00021	20
AX-157918207	2	62.685	KV453193.1	4738442	20-scf00021	20
AX-157744041	2	62.902	KV453193.1	4740752	20-scf00021	20
AX-157918219	2	63.127	KV453193.1	4753608	20-scf00021	20
AX-158379277	2	63.127	KV453193.1	4754599	20-scf00021	20
AX-157918223	2	63.127	KV453193.1	4756672	20-scf00021	20
AX-157918226	2	63.127	KV453193.1	4761294	20-scf00021	20
AX-157918227	2	63.128	KV453193.1	4761860	20-scf00021	20
AX-157918229	2	63.128	KV453193.1	4763180	20-scf00021	20
AX-157918230	2	63.368	KV453193.1	4766466	20-scf00021	20
AX-157918232	2	63.368	KV453193.1	4767274	20-scf00021	20
AX-157918235	2	63.515	KV453193.1	4770020	20-scf00021	20
AX-157918237	2	63.516	KV453193.1	4772343	20-scf00021	20
AX-157812941	2	63.516	KV453193.1	4772402	20-scf00021	20
AX-157812942	2	63.517	KV453193.1	4772763	20-scf00021	20
AX-157812943	2	63.517	KV453193.1	4773408	20-scf00021	20
AX-157918238	2	63.518	KV453193.1	4775075	20-scf00021	20
AX-157918086	2	63.627	KV453193.1	4566221	20-scf00021	20
AX-157918117	2	63.627	KV453193.1	4614588	20-scf00021	20

AX-157918122	2	63.627	KV453193.1	4628911	20-scf00021	20
AX-157918159	2	63.627	KV453193.1	4667772	20-scf00021	20
AX-157883498	2	63.627	KV453193.1	4668085	20-scf00021	20
AX-157918171	2	63.627	KV453193.1	4681621	20-scf00021	20
AX-157812935	2	63.627	KV453193.1	4699702	20-scf00021	20
AX-157918196	2	63.627	KV453193.1	4718976	20-scf00021	20
AX-157918212	2	63.627	KV453193.1	4743538	20-scf00021	20
AX-157918242	2	63.627	KV453193.1	4778720	20-scf00021	20
AX-157775904	2	63.627	KV453193.1	4778758	20-scf00021	20
AX-157918244	2	63.627	KV453193.1	4779330	20-scf00021	20
AX-157918246	2	63.627	KV453193.1	4783447	20-scf00021	20
AX-157918247	2	63.627	KV453193.1	4785223	20-scf00021	20
AX-157812944	2	63.627	KV453193.1	4787002	20-scf00021	20
AX-157918251	2	63.627	KV453193.1	4791780	20-scf00021	20
AX-157918252	2	63.627	KV453193.1	4792861	20-scf00021	20
AX-157918268	2	63.627	KV453193.1	4819993	20-scf00021	20
AX-157918269	2	63.627	KV453193.1	4820394	20-scf00021	20
AX-157918254	2	63.737	KV453193.1	4794208	20-scf00021	20
AX-157740230	2	63.737	KV453193.1	4801403	20-scf00021	20
AX-157918263	2	63.737	KV453193.1	4806733	20-scf00021	20
AX-157918264	2	63.737	KV453193.1	4807437	20-scf00021	20
AX-157918265	2	63.737	KV453193.1	4812434	20-scf00021	20
AX-157771097	2	63.738	KV453193.1	4811318	20-scf00021	20
AX-157812946	2	63.996	KV453193.1	4817111	20-scf00021	20
AX-157748280	2	64.034	KV453193.1	4821007	20-scf00021	20
AX-157918270	2	64.034	KV453193.1	4821918	20-scf00021	20
AX-157758804	2	64.063	KV453193.1	4823382	20-scf00021	20
AX-157918271	2	64.11	KV453193.1	4824285	20-scf00021	20
AX-157812949	2	64.273	KV453193.1	4829312	20-scf00021	20
AX-157918278	2	64.273	KV453193.1	4832203	20-scf00021	20
AX-157918279	2	64.64	KV453193.1	4833220	20-scf00021	20
AX-157918282	2	64.65	KV453193.1	4836026	20-scf00021	20
AX-157918283	2	64.65	KV453193.1	4836439	20-scf00021	20
AX-157918284	2	64.65	KV453193.1	4836731	20-scf00021	20
AX-157918286	2	64.65	KV453193.1	4838340	20-scf00021	20
AX-157812951	2	64.65	KV453193.1	4841971	20-scf00021	20
AX-157918281	2	64.696	KV453193.1	4834943	20-scf00021	20
AX-157918290	2	64.696	KV453193.1	4842678	20-scf00021	20
AX-157918370	2	64.696	KV453193.1	4959119	20-scf00021	20
AX-157862663	2	64.83	KV453193.1	4868115	20-scf00021	20
AX-157754521	2	64.83	KV453193.1	4872183	20-scf00021	20
AX-157918313	2	64.83	KV453193.1	4877984	20-scf00021	20
AX-157714735	2	64.83	KV453193.1	4878162	20-scf00021	20
AX-157918316	2	64.83	KV453193.1	4880270	20-scf00021	20
AX-157918317	2	64.83	KV453193.1	4882802	20-scf00021	20
AX-157918318	2	64.83	KV453193.1	4883933	20-scf00021	20
AX-157918319	2	64.83	KV453193.1	4884274	20-scf00021	20
AX-157812956	2	64.83	KV453193.1	4884964	20-scf00021	20
AX-157918323	2	64.83	KV453193.1	4889861	20-scf00021	20
AX-157812957	2	64.83	KV453193.1	4890930	20-scf00021	20
AX-157746162	2	64.83	KV453193.1	4891743	20-scf00021	20

AX-157918325	2	64.83	KV453193.1	4892145	20-scf00021	20
AX-157918327	2	64.83	KV453193.1	4895146	20-scf00021	20
AX-157918328	2	64.83	KV453193.1	4895625	20-scf00021	20
AX-157719948	2	64.889	KV453193.1	4898723	20-scf00021	20
AX-157812958	2	64.939	KV453193.1	4898766	20-scf00021	20
AX-157738889	2	65.158	KV453193.1	4906085	20-scf00021	20
AX-157918330	2	65.158	KV453193.1	4906659	20-scf00021	20
AX-157918331	2	65.158	KV453193.1	4909092	20-scf00021	20
AX-157918333	2	65.158	KV453193.1	4910097	20-scf00021	20
AX-157812960	2	65.158	KV453193.1	4913364	20-scf00021	20
AX-157918334	2	65.158	KV453193.1	4914903	20-scf00021	20
AX-157918335	2	65.158	KV453193.1	4915752	20-scf00021	20
AX-157772133	2	65.158	KV453193.1	4917752	20-scf00021	20
AX-158379278	2	65.158	KV453193.1	4920668	20-scf00021	20
AX-157918338	2	65.158	KV453193.1	4921237	20-scf00021	20
AX-157918343	2	65.158	KV453193.1	4929598	20-scf00021	20
AX-157918345	2	65.158	KV453193.1	4931649	20-scf00021	20
AX-157918350	2	65.158	KV453193.1	4935489	20-scf00021	20
AX-158389629	2	65.158	KV453193.1	4960475	20-scf00021	20
AX-157918375	2	65.158	KV453193.1	4964482	20-scf00021	20
AX-157918381	2	65.158	KV453193.1	4976724	20-scf00021	20
AX-157812961	2	65.377	KV453193.1	4921994	20-scf00021	20
AX-157918339	2	65.377	KV453193.1	4922811	20-scf00021	20
AX-157918340	2	65.377	KV453193.1	4923353	20-scf00021	20
AX-157918341	2	65.377	KV453193.1	4928497	20-scf00021	20
AX-157918342	2	65.377	KV453193.1	4928905	20-scf00021	20
AX-157918344	2	65.377	KV453193.1	4930884	20-scf00021	20
AX-157742518	2	65.377	KV453193.1	4931249	20-scf00021	20
AX-157918349	2	65.377	KV453193.1	4934596	20-scf00021	20
AX-157918353	2	65.377	KV453193.1	4937047	20-scf00021	20
AX-157918352	2	65.497	KV453193.1	4936678	20-scf00021	20
AX-157918357	2	65.595	KV453193.1	4941960	20-scf00021	20
AX-157918358	2	65.595	KV453193.1	4945393	20-scf00021	20
AX-157918359	2	65.595	KV453193.1	4946980	20-scf00021	20
AX-157918360	2	65.595	KV453193.1	4947789	20-scf00021	20
AX-157918361	2	65.595	KV453193.1	4948006	20-scf00021	20
AX-157918362	2	65.645	KV453193.1	4949384	20-scf00021	20
AX-157918374	2	65.645	KV453193.1	4963753	20-scf00021	20
AX-157918367	2	65.926	KV453193.1	4956603	20-scf00021	20
AX-157918369	2	66.253	KV453193.1	4957898	20-scf00021	20
AX-157703269	2	66.253	KV453193.1	4960193	20-scf00021	20
AX-157918371	2	66.358	KV453193.1	4960412	20-scf00021	20
AX-157918365	2	66.472	KV453193.1	4954368	20-scf00021	20
AX-157918376	2	66.472	KV453193.1	4965624	20-scf00021	20
AX-157750639	2	66.472	KV453193.1	4978557	20-scf00021	20
AX-157918383	2	66.472	KV453193.1	4980920	20-scf00021	20
AX-157918385	2	66.473	KV453193.1	4982685	20-scf00021	20
AX-157918387	2	66.473	KV453193.1	4984117	20-scf00021	20
AX-157918388	2	66.635	KV453193.1	4984563	20-scf00021	20
AX-157918390	2	66.8	KV453193.1	4986651	20-scf00021	20
AX-157918392	2	66.8	KV453193.1	4987848	20-scf00021	20

AX-157918394	2	66.8	KV453193.1	4990139	20-scf00021	20
AX-157918395	2	66.8	KV453193.1	4990945	20-scf00021	20
AX-157918402	2	66.8	KV453193.1	5000933	20-scf00021	20
AX-157918396	2	66.909	KV453193.1	4994056	20-scf00021	20
AX-157918397	2	66.909	KV453193.1	4994593	20-scf00021	20
AX-157918400	2	66.909	KV453193.1	4999591	20-scf00021	20
AX-157918401	2	66.909	KV453193.1	5000418	20-scf00021	20
AX-157752118	2	66.909	KV453193.1	5002026	20-scf00021	20
AX-157918403	2	66.909	KV453193.1	5003486	20-scf00021	20
AX-157918404	2	66.909	KV453193.1	5004432	20-scf00021	20
AX-157723316	2	66.909	KV453193.1	5006130	20-scf00021	20
AX-157918405	2	66.909	KV453193.1	5008384	20-scf00021	20
AX-157918406	2	66.909	KV453193.1	5008927	20-scf00021	20
AX-157918407	2	66.909	KV453193.1	5009381	20-scf00021	20
AX-157714872	2	66.909	KV453193.1	5011438	20-scf00021	20
AX-157918411	2	67.208	KV453193.1	5016320	20-scf00021	20
AX-157709379	2	67.25	KV453193.1	5019373	20-scf00021	20
AX-157918416	2	67.25	KV453193.1	5025584	20-scf00021	20
AX-158379279	2	67.25	KV453193.1	5034411	20-scf00021	20
AX-157918471	2	67.25	KV453193.1	5119138	20-scf00021	20
AX-157812967	2	67.294	KV453193.1	5033734	20-scf00021	20
AX-157747358	2	67.322	KV453193.1	5035096	20-scf00021	20
AX-157918422	2	67.365	KV453193.1	5042445	20-scf00021	20
AX-157720573	2	67.408	KV453193.1	5048057	20-scf00021	20
AX-157918425	2	67.408	KV453193.1	5048288	20-scf00021	20
AX-157918451	2	67.408	KV453193.1	5096861	20-scf00021	20
AX-157812974	2	67.408	KV453193.1	5098310	20-scf00021	20
AX-157709378	2	67.565	KV453193.1	5019446	20-scf00021	20
AX-157719229	2	67.565	KV453193.1	5045590	20-scf00021	20
AX-157918428	2	67.565	KV453193.1	5054388	20-scf00021	20
AX-157918432	2	67.565	KV453193.1	5056826	20-scf00021	20
AX-157812969	2	67.565	KV453193.1	5059766	20-scf00021	20
AX-157918433	2	67.565	KV453193.1	5060495	20-scf00021	20
AX-157918434	2	67.565	KV453193.1	5062040	20-scf00021	20
AX-158405149	2	67.565	KV453193.1	5062852	20-scf00021	20
AX-157918437	2	67.565	KV453193.1	5063974	20-scf00021	20
AX-157918438	2	67.565	KV453193.1	5064458	20-scf00021	20
AX-157918439	2	67.565	KV453193.1	5067888	20-scf00021	20
AX-157918440	2	67.589	KV453193.1	5068758	20-scf00021	20
AX-157775044	2	67.62	KV453193.1	5069114	20-scf00021	20
AX-157918412	2	67.674	KV453193.1	5020442	20-scf00021	20
AX-157753675	2	67.674	KV453193.1	5075476	20-scf00021	20
AX-157745153	2	67.674	KV453193.1	5078241	20-scf00021	20
AX-157918442	2	67.674	KV453193.1	5080813	20-scf00021	20
AX-157874724	2	67.674	KV453193.1	5083026	20-scf00021	20
AX-157812971	2	67.674	KV453193.1	5090008	20-scf00021	20
AX-157715649	2	67.674	KV453193.1	5091068	20-scf00021	20
AX-157918449	2	67.674	KV453193.1	5092617	20-scf00021	20
AX-157812972	2	67.674	KV453193.1	5094727	20-scf00021	20
AX-157918452	2	67.674	KV453193.1	5097778	20-scf00021	20
AX-157918453	2	67.675	KV453193.1	5098575	20-scf00021	20

AX-157695471	2	67.893	KV453193.1	5099309	20-scf00021	20
AX-157812975	2	67.893	KV453193.1	5099892	20-scf00021	20
AX-157812976	2	67.893	KV453193.1	5100623	20-scf00021	20
AX-157918457	2	68.305	KV453193.1	5104878	20-scf00021	20
AX-157918458	2	68.364	KV453193.1	5105595	20-scf00021	20
AX-157918459	2	68.365	KV453193.1	5106812	20-scf00021	20
AX-157918460	2	68.606	KV453193.1	5109084	20-scf00021	20
AX-157918463	2	68.607	KV453193.1	5111824	20-scf00021	20
AX-157918464	2	68.607	KV453193.1	5113389	20-scf00021	20
AX-157918465	2	68.607	KV453193.1	5113645	20-scf00021	20
AX-157918466	2	68.607	KV453193.1	5114067	20-scf00021	20
AX-157918467	2	68.607	KV453193.1	5114567	20-scf00021	20
AX-157918470	2	68.685	KV453193.1	5117632	20-scf00021	20
AX-157750070	2	68.867	KV453193.1	5123867	20-scf00021	20
AX-157918475	2	68.876	KV453193.1	5125028	20-scf00021	20
AX-157918476	2	69.242	KV453193.1	5129151	20-scf00021	20
AX-157740674	2	69.242	KV453193.1	5130071	20-scf00021	20
AX-157918477	2	69.244	KV453193.1	5130457	20-scf00021	20
AX-157918481	2	69.269	KV453193.1	5133018	20-scf00021	20
AX-157918478	2	69.33	KV453193.1	5131861	20-scf00021	20
AX-157918482	2	69.33	KV453193.1	5133531	20-scf00021	20
AX-157741631	2	69.33	KV453193.1	5160926	20-scf00021	20
AX-157918490	2	69.545	KV453193.1	5144483	20-scf00021	20
AX-157918493	2	69.748	KV453193.1	5148484	20-scf00021	20
AX-157773742	2	69.754	KV453193.1	5148675	20-scf00021	20
AX-157918496	2	70.517	KV453193.1	5154224	20-scf00021	20
AX-157710193	2	70.517	KV453193.1	5154691	20-scf00021	20
AX-157918504	2	70.517	KV453193.1	5166455	20-scf00021	20
AX-157918505	2	70.517	KV453193.1	5167230	20-scf00021	20
AX-157918507	2	70.517	KV453193.1	5169780	20-scf00021	20
AX-157918511	2	70.517	KV453193.1	5173216	20-scf00021	20
AX-157918514	2	70.569	KV453193.1	5174661	20-scf00021	20
AX-157918516	2	70.605	KV453193.1	5177597	20-scf00021	20
AX-157918519	2	70.627	KV453193.1	5179918	20-scf00021	20
AX-157918520	2	70.627	KV453193.1	5181703	20-scf00021	20
AX-157918484	2	70.628	KV453193.1	5138322	20-scf00021	20
AX-157918522	2	70.628	KV453193.1	5185562	20-scf00021	20
AX-157918523	2	70.628	KV453193.1	5186152	20-scf00021	20
AX-157918525	2	70.628	KV453193.1	5191859	20-scf00021	20
AX-157918524	2	70.634	KV453193.1	5191508	20-scf00021	20
AX-157918528	2	70.726	KV453193.1	5197594	20-scf00021	20
AX-158405151	2	70.736	KV453193.1	5202116	20-scf00021	20
AX-158405152	2	70.736	KV453193.1	5202264	20-scf00021	20
AX-158404315	2	70.736	KV453193.1	5202869	20-scf00021	20
AX-157812980	2	70.88	KV453193.1	5209468	20-scf00021	20
AX-157918534	2	70.895	KV453193.1	5212621	20-scf00021	20
AX-157918506	2	71.029	KV453193.1	5169210	20-scf00021	20
AX-157918537	2	71.029	KV453193.1	5214911	20-scf00021	20
AX-157918538	2	71.029	KV453193.1	5215707	20-scf00021	20
AX-157918546	2	71.029	KV453193.1	5223778	20-scf00021	20
AX-157918539	2	71.171	KV453193.1	5216756	20-scf00021	20

AX-157918542	2	71.174	KV453193.1	5219905	20-scf00021	20
AX-157812982	2	71.392	KV453193.1	5222551	20-scf00021	20
AX-157918548	2	71.392	KV453193.1	5226606	20-scf00021	20
AX-157918549	2	71.392	KV453193.1	5227801	20-scf00021	20
AX-157918551	2	71.392	KV453193.1	5231820	20-scf00021	20
AX-157756979	2	71.392	KV453193.1	5232340	20-scf00021	20
AX-157740022	2	71.392	KV453193.1	5232745	20-scf00021	20
AX-157918555	2	71.829	KV453193.1	5238950	20-scf00021	20
AX-157918556	2	71.829	KV453193.1	5239432	20-scf00021	20
AX-157918557	2	71.829	KV453193.1	5240500	20-scf00021	20
AX-157918559	2	71.829	KV453193.1	5243909	20-scf00021	20
AX-157918563	2	71.829	KV453193.1	5245800	20-scf00021	20
AX-157918558	2	71.927	KV453193.1	5241909	20-scf00021	20
AX-157918564	2	72.094	KV453193.1	5248294	20-scf00021	20
AX-157918565	2	72.267	KV453193.1	5249572	20-scf00021	20
AX-157918566	2	72.346	KV453193.1	5250336	20-scf00021	20
AX-157918561	2	72.431	KV453193.1	5244796	20-scf00021	20
AX-157918568	2	72.431	KV453193.1	5252183	20-scf00021	20
AX-157918570	2	72.455	KV453193.1	5253245	20-scf00021	20
AX-157725122	2	72.594	KV453193.1	5254554	20-scf00021	20
AX-157918571	2	72.594	KV453193.1	5255188	20-scf00021	20
AX-157918572	2	72.595	KV453193.1	5255686	20-scf00021	20
AX-157918576	2	72.595	KV453193.1	5260096	20-scf00021	20
AX-157918585	2	72.704	KV453193.1	5267280	20-scf00021	20
AX-157918588	2	72.85	KV453193.1	5273284	20-scf00021	20
AX-157918595	2	72.85	KV453193.1	5281805	20-scf00021	20
AX-157771813	2	73.141	KV453193.1	5268283	20-scf00021	20
AX-157918598	2	73.141	KV453193.1	5287743	20-scf00021	20
AX-157918600	2	73.141	KV453193.1	5290237	20-scf00021	20
AX-157918601	2	73.141	KV453193.1	5291009	20-scf00021	20
AX-157918602	2	73.141	KV453193.1	5291220	20-scf00021	20
AX-157918604	2	73.141	KV453193.1	5295099	20-scf00021	20
AX-157918605	2	73.141	KV453193.1	5296590	20-scf00021	20
AX-157918612	2	73.141	KV453193.1	5304179	20-scf00021	20
AX-157812995	2	73.141	KV453193.1	5407408	20-scf00021	20
AX-157754377	2	73.141	KV453193.1	5434425	20-scf00021	20
AX-157918608	2	73.225	KV453193.1	5300384	20-scf00021	20
AX-157918610	2	73.292	KV453193.1	5302924	20-scf00021	20
AX-157918611	2	73.36	KV453193.1	5303683	20-scf00021	20
AX-157918626	2	73.36	KV453193.1	5317601	20-scf00021	20
AX-157918628	2	73.36	KV453193.1	5318749	20-scf00021	20
AX-157918631	2	73.578	KV453193.1	5320640	20-scf00021	20
AX-157918633	2	73.578	KV453193.1	5322949	20-scf00021	20
AX-157918634	2	73.578	KV453193.1	5324043	20-scf00021	20
AX-157918636	2	73.578	KV453193.1	5324699	20-scf00021	20
AX-157918637	2	73.797	KV453193.1	5325901	20-scf00021	20
AX-157812986	2	73.86	KV453193.1	5328640	20-scf00021	20
AX-157918593	2	73.906	KV453193.1	5279326	20-scf00021	20
AX-157812987	2	73.906	KV453193.1	5329527	20-scf00021	20
AX-157918638	2	73.906	KV453193.1	5331571	20-scf00021	20
AX-157918641	2	73.906	KV453193.1	5339281	20-scf00021	20

AX-157918642	2	73.906	KV453193.1	5342112	20-scf00021	20
AX-157812988	2	73.906	KV453193.1	5342587	20-scf00021	20
AX-157812989	2	73.906	KV453193.1	5344536	20-scf00021	20
AX-157918644	2	73.906	KV453193.1	5348399	20-scf00021	20
AX-157714190	2	73.906	KV453193.1	5353238	20-scf00021	20
AX-157918648	2	73.906	KV453193.1	5355168	20-scf00021	20
AX-157918661	2	73.906	KV453193.1	5372049	20-scf00021	20
AX-157918666	2	73.906	KV453193.1	5380723	20-scf00021	20
AX-157720658	2	74.124	KV453193.1	5354484	20-scf00021	20
AX-157751720	2	74.124	KV453193.1	5356394	20-scf00021	20
AX-157918650	2	74.124	KV453193.1	5359558	20-scf00021	20
AX-157918653	2	74.124	KV453193.1	5363856	20-scf00021	20
AX-157918654	2	74.124	KV453193.1	5365209	20-scf00021	20
AX-157918655	2	74.124	KV453193.1	5367192	20-scf00021	20
AX-157918658	2	74.124	KV453193.1	5369581	20-scf00021	20
AX-157918662	2	74.124	KV453193.1	5373122	20-scf00021	20
AX-157918664	2	74.124	KV453193.1	5374172	20-scf00021	20
AX-157812990	2	74.124	KV453193.1	5374422	20-scf00021	20
AX-157918687	2	74.124	KV453193.1	5422998	20-scf00021	20
AX-157918672	2	74.288	KV453193.1	5390088	20-scf00021	20
AX-157812993	2	74.288	KV453193.1	5392519	20-scf00021	20
AX-157918693	2	74.288	KV453193.1	5431061	20-scf00021	20
AX-157918673	2	74.37	KV453193.1	5393560	20-scf00021	20
AX-157773474	2	74.452	KV453193.1	5394598	20-scf00021	20
AX-157812994	2	74.452	KV453193.1	5397980	20-scf00021	20
AX-157918676	2	74.452	KV453193.1	5399051	20-scf00021	20
AX-157748384	2	74.452	KV453193.1	5400456	20-scf00021	20
AX-157918677	2	74.452	KV453193.1	5401941	20-scf00021	20
AX-157918679	2	74.452	KV453193.1	5406065	20-scf00021	20
AX-157918681	2	74.511	KV453193.1	5410807	20-scf00021	20
AX-157918689	2	74.511	KV453193.1	5426126	20-scf00021	20
AX-157918682	2	74.561	KV453193.1	5413247	20-scf00021	20
AX-157918685	2	74.561	KV453193.1	5420154	20-scf00021	20
AX-157918686	2	74.561	KV453193.1	5422379	20-scf00021	20
AX-157812997	2	74.561	KV453193.1	5423442	20-scf00021	20
AX-157918688	2	74.561	KV453193.1	5423751	20-scf00021	20
AX-157812998	2	74.561	KV453193.1	5424692	20-scf00021	20
AX-157918694	2	74.561	KV453193.1	5433400	20-scf00021	20
AX-157918695	2	74.561	KV453193.1	5435456	20-scf00021	20
AX-157716120	2	74.561	KV453193.1	5439082	20-scf00021	20
AX-157918698	2	74.561	KV453193.1	5441094	20-scf00021	20
AX-157918699	2	74.561	KV453193.1	5442137	20-scf00021	20
AX-157918700	2	74.561	KV453193.1	5443043	20-scf00021	20
AX-157812991	2	74.707	KV453193.1	5382061	20-scf00021	20
AX-157710424	2	74.707	KV453193.1	5444665	20-scf00021	20
AX-157918702	2	74.888	KV453193.1	5446243	20-scf00021	20
AX-157812996	2	74.998	KV453193.1	5420965	20-scf00021	20
AX-157918705	2	74.998	KV453193.1	5449407	20-scf00021	20
AX-157918706	2	74.998	KV453193.1	5450302	20-scf00021	20
AX-157918707	2	74.998	KV453193.1	5451112	20-scf00021	20
AX-157703235	2	74.998	KV453193.1	5464770	20-scf00021	20

AX-157918714	2	74.998	KV453193.1	5465568	20-scf00021	20
AX-157918715	2	74.998	KV453193.1	5465632	20-scf00021	20
AX-157918716	2	74.998	KV453193.1	5466297	20-scf00021	20
AX-157918717	2	74.998	KV453193.1	5467153	20-scf00021	20
AX-157918718	2	74.998	KV453193.1	5467775	20-scf00021	20
AX-157714241	2	74.998	KV453193.1	5469619	20-scf00021	20
AX-157918720	2	74.998	KV453193.1	5470211	20-scf00021	20
AX-157918723	2	75.144	KV453193.1	5472340	20-scf00021	20
AX-157918771	2	75.435	KV453193.1	5526142	20-scf00021	20
AX-157918772	2	75.435	KV453193.1	5526690	20-scf00021	20
AX-157918773	2	75.435	KV453193.1	5528958	20-scf00021	20
AX-158405162	2	75.435	KV453193.1	5530997	20-scf00021	20
AX-157918777	2	75.435	KV453193.1	5532847	20-scf00021	20
AX-157918779	2	75.435	KV453193.1	5537727	20-scf00021	20
AX-157918780	2	75.435	KV453193.1	5538625	20-scf00021	20
AX-157918781	2	75.435	KV453193.1	5539435	20-scf00021	20
AX-157918782	2	75.435	KV453193.1	5540054	20-scf00021	20
AX-157918783	2	75.435	KV453193.1	5540662	20-scf00021	20
AX-157918786	2	75.435	KV453193.1	5546810	20-scf00021	20
AX-157918788	2	75.435	KV453193.1	5550200	20-scf00021	20
AX-158379283	2	75.435	KV453193.1	5550866	20-scf00021	20
AX-157918789	2	75.435	KV453193.1	5550954	20-scf00021	20
AX-157918790	2	75.435	KV453193.1	5553132	20-scf00021	20
AX-157918794	2	75.435	KV453193.1	5557659	20-scf00021	20
AX-157739204	2	75.435	KV453193.1	5558920	20-scf00021	20
AX-157918796	2	75.435	KV453193.1	5559490	20-scf00021	20
AX-157918797	2	75.435	KV453193.1	5560390	20-scf00021	20
AX-157918798	2	75.435	KV453193.1	5560886	20-scf00021	20
AX-157918802	2	75.435	KV453193.1	5566608	20-scf00021	20
AX-157918803	2	75.435	KV453193.1	5566877	20-scf00021	20
AX-157918805	2	75.435	KV453193.1	5569187	20-scf00021	20
AX-157918806	2	75.435	KV453193.1	5571467	20-scf00021	20
AX-157918808	2	75.435	KV453193.1	5575579	20-scf00021	20
AX-157918861	2	75.435	KV453193.1	5644884	20-scf00021	20
AX-157918775	2	75.436	KV453193.1	5531286	20-scf00021	20
AX-157813005	2	75.436	KV453193.1	5543116	20-scf00021	20
AX-157918792	2	75.436	KV453193.1	5554732	20-scf00021	20
AX-157714320	2	75.436	KV453193.1	5580292	20-scf00021	20
AX-157918811	2	75.436	KV453193.1	5580705	20-scf00021	20
AX-157918812	2	75.599	KV453193.1	5581153	20-scf00021	20
AX-157918815	2	75.686	KV453193.1	5583779	20-scf00021	20
AX-157918817	2	75.763	KV453193.1	5588008	20-scf00021	20
AX-157918820	2	75.763	KV453193.1	5592679	20-scf00021	20
AX-157918821	2	75.763	KV453193.1	5594321	20-scf00021	20
AX-157918822	2	75.763	KV453193.1	5595970	20-scf00021	20
AX-157724520	2	75.785	KV453193.1	5600737	20-scf00021	20
AX-157918825	2	75.81	KV453193.1	5602105	20-scf00021	20
AX-157918827	2	75.836	KV453193.1	5604794	20-scf00021	20
AX-157918828	2	75.856	KV453193.1	5605932	20-scf00021	20
AX-157741322	2	75.873	KV453193.1	5611005	20-scf00021	20
AX-157918835	2	75.896	KV453193.1	5611420	20-scf00021	20

AX-157918850	2	75.896	KV453193.1	5631495	20-scf00021	20
AX-157918873	2	75.896	KV453193.1	5666045	20-scf00021	20
AX-157918900	2	75.896	KV453193.1	5713168	20-scf00021	20
AX-157709857	2	75.938	KV453193.1	5612002	20-scf00021	20
AX-157918837	2	75.982	KV453193.1	5614668	20-scf00021	20
AX-157725138	2	75.982	KV453193.1	5615487	20-scf00021	20
AX-157918838	2	75.982	KV453193.1	5615775	20-scf00021	20
AX-157853766	2	75.982	KV453193.1	5618640	20-scf00021	20
AX-157918842	2	76.022	KV453193.1	5622262	20-scf00021	20
AX-157918836	2	76.061	KV453193.1	5613688	20-scf00021	20
AX-157918847	2	76.061	KV453193.1	5625159	20-scf00021	20
AX-157918857	2	76.061	KV453193.1	5638280	20-scf00021	20
AX-157918839	2	76.091	KV453193.1	5618981	20-scf00021	20
AX-157918845	2	76.091	KV453193.1	5624374	20-scf00021	20
AX-157918849	2	76.091	KV453193.1	5628254	20-scf00021	20
AX-158379284	2	76.091	KV453193.1	5631783	20-scf00021	20
AX-157918854	2	76.091	KV453193.1	5634989	20-scf00021	20
AX-157918855	2	76.091	KV453193.1	5636842	20-scf00021	20
AX-157741019	2	76.091	KV453193.1	5636964	20-scf00021	20
AX-157869130	2	76.091	KV453193.1	5650730	20-scf00021	20
AX-157918864	2	76.091	KV453193.1	5651789	20-scf00021	20
AX-157918866	2	76.091	KV453193.1	5653792	20-scf00021	20
AX-157918867	2	76.091	KV453193.1	5657301	20-scf00021	20
AX-157918869	2	76.091	KV453193.1	5660132	20-scf00021	20
AX-157918870	2	76.091	KV453193.1	5660752	20-scf00021	20
AX-157813011	2	76.091	KV453193.1	5662243	20-scf00021	20
AX-157918871	2	76.091	KV453193.1	5662702	20-scf00021	20
AX-157813013	2	76.091	KV453193.1	5675023	20-scf00021	20
AX-157918878	2	76.091	KV453193.1	5675482	20-scf00021	20
AX-157813015	2	76.091	KV453193.1	5677905	20-scf00021	20
AX-157813019	2	76.153	KV453193.1	5689598	20-scf00021	20
AX-157918886	2	76.222	KV453193.1	5695622	20-scf00021	20
AX-157918816	2	76.31	KV453193.1	5585501	20-scf00021	20
AX-157918824	2	76.31	KV453193.1	5600975	20-scf00021	20
AX-157739385	2	76.31	KV453193.1	5601549	20-scf00021	20
AX-157918846	2	76.31	KV453193.1	5624623	20-scf00021	20
AX-158389631	2	76.31	KV453193.1	5665766	20-scf00021	20
AX-157745026	2	76.31	KV453193.1	5677574	20-scf00021	20
AX-157918891	2	76.31	KV453193.1	5699611	20-scf00021	20
AX-157918892	2	76.31	KV453193.1	5700436	20-scf00021	20
AX-157918893	2	76.31	KV453193.1	5700741	20-scf00021	20
AX-157918896	2	76.31	KV453193.1	5702529	20-scf00021	20
AX-157813022	2	76.31	KV453193.1	5702940	20-scf00021	20
AX-157918899	2	76.31	KV453193.1	5707815	20-scf00021	20
AX-157752942	2	76.31	KV453193.1	5711472	20-scf00021	20
AX-157918901	2	76.31	KV453193.1	5713688	20-scf00021	20
AX-157918904	2	76.31	KV453193.1	5716707	20-scf00021	20
AX-157918908	2	76.31	KV453193.1	5719431	20-scf00021	20
AX-157918910	2	76.31	KV453193.1	5720578	20-scf00021	20
AX-157918912	2	76.31	KV453193.1	5721627	20-scf00021	20
AX-157918880	2	76.362	KV453193.1	5684085	20-scf00021	20

AX-157918882	2	76.362	KV453193.1	5687308	20-scf00021	20
AX-157918915	2	76.362	KV453193.1	5722764	20-scf00021	20
AX-157918922	2	76.362	KV453193.1	5727778	20-scf00021	20
AX-157918877	2	76.414	KV453193.1	5674619	20-scf00021	20
AX-157918916	2	76.414	KV453193.1	5724285	20-scf00021	20
AX-157715648	2	76.414	KV453193.1	5732859	20-scf00021	20
AX-157918934	2	76.414	KV453193.1	5738893	20-scf00021	20
AX-157918918	2	76.473	KV453193.1	5725379	20-scf00021	20
AX-157918907	2	76.528	KV453193.1	5718996	20-scf00021	20
AX-157918913	2	76.528	KV453193.1	5721893	20-scf00021	20
AX-157918920	2	76.528	KV453193.1	5726627	20-scf00021	20
AX-157918921	2	76.528	KV453193.1	5727457	20-scf00021	20
AX-157918926	2	76.528	KV453193.1	5729642	20-scf00021	20
AX-157710989	2	76.529	KV453193.1	5674260	20-scf00021	20
AX-157918927	2	76.529	KV453193.1	5730674	20-scf00021	20
AX-157813023	2	76.529	KV453193.1	5734254	20-scf00021	20
AX-157918931	2	76.855	KV453193.1	5735509	20-scf00021	20
AX-157918932	2	76.856	KV453193.1	5736198	20-scf00021	20
AX-157918933	2	76.856	KV453193.1	5736707	20-scf00021	20
AX-157918937	2	76.856	KV453193.1	5740644	20-scf00021	20
AX-157918938	2	76.856	KV453193.1	5741225	20-scf00021	20
AX-157813024	2	76.856	KV453193.1	5741559	20-scf00021	20
AX-157918939	2	76.87	KV453193.1	5743992	20-scf00021	20
AX-157869132	2	76.894	KV453193.1	5746305	20-scf00021	20
AX-157853767	2	77.189	KV453193.1	5754323	20-scf00021	20
AX-157714776	2	77.189	KV453193.1	5755259	20-scf00021	20
AX-157918947	2	77.191	KV453193.1	5760206	20-scf00021	20
AX-157771024	2	77.364	KV453193.1	5766738	20-scf00021	20
AX-157918950	2	77.364	KV453193.1	5767567	20-scf00021	20
AX-157918953	2	77.452	KV453193.1	5774301	20-scf00021	20
AX-157918943	2	77.622	KV453193.1	5748917	20-scf00021	20
AX-157918946	2	77.622	KV453193.1	5759802	20-scf00021	20
AX-157918956	2	77.622	KV453193.1	5776434	20-scf00021	20
AX-157918959	2	77.622	KV453193.1	5778604	20-scf00021	20
AX-157918961	2	77.622	KV453193.1	5781166	20-scf00021	20
AX-157918963	2	77.779	KV453193.1	5789132	20-scf00021	20
AX-157918952	2	77.91	KV453193.1	5773589	20-scf00021	20
AX-157813026	2	77.91	KV453193.1	5791836	20-scf00021	20
AX-157918949	2	77.984	KV453193.1	5763612	20-scf00021	20
AX-157918967	2	77.984	KV453193.1	5794807	20-scf00021	20
AX-157757567	2	78.054	KV453193.1	5796535	20-scf00021	20
AX-157918986	2	78.063	KV453193.1	5819351	20-scf00021	20
AX-157918972	2	78.075	KV453193.1	5801598	20-scf00021	20
AX-157918973	2	78.147	KV453193.1	5804335	20-scf00021	20
AX-157918974	2	78.219	KV453193.1	5807433	20-scf00021	20
AX-157918975	2	78.3	KV453193.1	5808935	20-scf00021	20
AX-157918964	2	78.387	KV453193.1	5793333	20-scf00021	20
AX-157918977	2	78.387	KV453193.1	5812453	20-scf00021	20
AX-157918979	2	78.387	KV453193.1	5815138	20-scf00021	20
AX-157918980	2	78.387	KV453193.1	5816233	20-scf00021	20
AX-157918982	2	78.387	KV453193.1	5817368	20-scf00021	20

AX-157918987	2	78.387	KV453193.1	5819959	20-scf00021	20
AX-157918988	2	78.388	KV453193.1	5820243	20-scf00021	20
AX-157918991	2	79.125	KV453193.1	5824471	20-scf00021	20
AX-157918992	2	79.175	KV453193.1	5827413	20-scf00021	20
AX-157718293	2	79.268	KV453193.1	5828488	20-scf00021	20
AX-157918994	2	79.372	KV453193.1	5831931	20-scf00021	20
AX-157918995	2	79.372	KV453193.1	5832935	20-scf00021	20
AX-157918996	2	79.59	KV453193.1	5833925	20-scf00021	20
AX-157813027	2	79.59	KV453193.1	5835585	20-scf00021	20
AX-157918997	2	79.59	KV453193.1	5836442	20-scf00021	20
AX-157918998	2	79.59	KV453193.1	5838164	20-scf00021	20
AX-157918999	2	79.59	KV453193.1	5839434	20-scf00021	20
AX-157853768	2	79.59	KV453193.1	5839932	20-scf00021	20
AX-157919000	2	79.59	KV453193.1	5840298	20-scf00021	20
AX-157919001	2	79.59	KV453193.1	5841392	20-scf00021	20
AX-157919002	2	79.59	KV453193.1	5841662	20-scf00021	20
AX-157758604	2	79.59	KV453193.1	5843123	20-scf00021	20
AX-157919003	2	79.59	KV453193.1	5843660	20-scf00021	20
AX-157727464	2	79.59	KV453193.1	5844464	20-scf00021	20
AX-157919006	2	79.59	KV453193.1	5845079	20-scf00021	20
AX-157919009	2	79.59	KV453193.1	5847705	20-scf00021	20
AX-157919010	2	79.809	KV453193.1	5848821	20-scf00021	20
AX-157919011	2	79.809	KV453193.1	5849387	20-scf00021	20
AX-157919012	2	79.809	KV453193.1	5849990	20-scf00021	20
AX-157919013	2	79.809	KV453193.1	5852034	20-scf00021	20
AX-157919015	2	79.809	KV453193.1	5853557	20-scf00021	20
AX-157919017	2	79.809	KV453193.1	5854310	20-scf00021	20
AX-157919018	2	79.843	KV453193.1	5854765	20-scf00021	20
AX-157919019	2	79.881	KV453193.1	5855073	20-scf00021	20
AX-157720086	2	79.918	KV453193.1	5858734	20-scf00021	20
AX-157919023	2	79.918	KV453193.1	5859913	20-scf00021	20
AX-157919028	2	79.918	KV453193.1	5865121	20-scf00021	20
AX-157919030	2	79.918	KV453193.1	5867968	20-scf00021	20
AX-157919033	2	80.082	KV453193.1	5871685	20-scf00021	20
AX-157869133	2	80.123	KV453193.1	5875927	20-scf00021	20
AX-157919048	2	80.123	KV453193.1	5886331	20-scf00021	20
AX-157755038	2	80.162	KV453193.1	5876427	20-scf00021	20
AX-157919039	2	80.207	KV453193.1	5878449	20-scf00021	20
AX-157919140	2	80.207	KV453193.1	6016537	20-scf00021	20
AX-157919038	2	80.246	KV453193.1	5877722	20-scf00021	20
AX-157919040	2	80.246	KV453193.1	5878693	20-scf00021	20
AX-157919041	2	80.246	KV453193.1	5879309	20-scf00021	20
AX-157919042	2	80.246	KV453193.1	5879939	20-scf00021	20
AX-157919043	2	80.246	KV453193.1	5880501	20-scf00021	20
AX-157919044	2	80.246	KV453193.1	5880639	20-scf00021	20
AX-157919045	2	80.246	KV453193.1	5882281	20-scf00021	20
AX-157919050	2	80.246	KV453193.1	5887739	20-scf00021	20
AX-157919051	2	80.246	KV453193.1	5888977	20-scf00021	20
AX-157726894	2	80.246	KV453193.1	5889333	20-scf00021	20
AX-157919052	2	80.246	KV453193.1	5892679	20-scf00021	20
AX-157919053	2	80.246	KV453193.1	5893974	20-scf00021	20

AX-157919054	2	80.246	KV453193.1	5896246	20-scf00021	20
AX-157919055	2	80.246	KV453193.1	5896499	20-scf00021	20
AX-157813030	2	80.246	KV453193.1	5930161	20-scf00021	20
AX-157919135	2	80.246	KV453193.1	6008719	20-scf00021	20
AX-157919059	2	80.247	KV453193.1	5902651	20-scf00021	20
AX-157919063	2	80.574	KV453193.1	5908941	20-scf00021	20
AX-157919066	2	80.574	KV453193.1	5911816	20-scf00021	20
AX-157919083	2	80.574	KV453193.1	5934661	20-scf00021	20
AX-157919068	2	80.584	KV453193.1	5916113	20-scf00021	20
AX-157757511	2	80.635	KV453193.1	5916600	20-scf00021	20
AX-157919073	2	80.635	KV453193.1	5924849	20-scf00021	20
AX-157919086	2	80.635	KV453193.1	5941998	20-scf00021	20
AX-158389634	2	80.646	KV453193.1	5917932	20-scf00021	20
AX-157919069	2	80.683	KV453193.1	5918339	20-scf00021	20
AX-157919076	2	80.683	KV453193.1	5926569	20-scf00021	20
AX-157919077	2	80.683	KV453193.1	5926837	20-scf00021	20
AX-157919079	2	80.683	KV453193.1	5928200	20-scf00021	20
AX-157748251	2	80.683	KV453193.1	5930770	20-scf00021	20
AX-157919081	2	80.683	KV453193.1	5933366	20-scf00021	20
AX-157919088	2	80.683	KV453193.1	5942924	20-scf00021	20
AX-157919090	2	80.683	KV453193.1	5943956	20-scf00021	20
AX-157919094	2	80.683	KV453193.1	5947018	20-scf00021	20
AX-157919106	2	80.683	KV453193.1	5961205	20-scf00021	20
AX-157919070	2	81.065	KV453193.1	5919406	20-scf00021	20
AX-157919097	2	81.065	KV453193.1	5950538	20-scf00021	20
AX-157919108	2	81.449	KV453193.1	5964722	20-scf00021	20
AX-157919109	2	81.449	KV453193.1	5964977	20-scf00021	20
AX-157919110	2	81.449	KV453193.1	5965239	20-scf00021	20
AX-157919114	2	81.614	KV453193.1	5969717	20-scf00021	20
AX-157919115	2	81.614	KV453193.1	5970066	20-scf00021	20
AX-157919116	2	81.778	KV453193.1	5971383	20-scf00021	20
AX-157813031	2	81.778	KV453193.1	5975622	20-scf00021	20
AX-157919118	2	81.778	KV453193.1	5975849	20-scf00021	20
AX-157919119	2	81.778	KV453193.1	5980281	20-scf00021	20
AX-157919089	2	82.044	KV453193.1	5943410	20-scf00021	20
AX-157919112	2	82.044	KV453193.1	5969034	20-scf00021	20
AX-157919121	2	82.044	KV453193.1	5985593	20-scf00021	20
AX-157919122	2	82.044	KV453193.1	5986204	20-scf00021	20
AX-157919123	2	82.044	KV453193.1	5991636	20-scf00021	20
AX-157919124	2	82.044	KV453193.1	5993336	20-scf00021	20
AX-157919126	2	82.145	KV453193.1	5993991	20-scf00021	20
AX-157919130	2	82.324	KV453193.1	5997673	20-scf00021	20
AX-157919132	2	82.324	KV453193.1	6001013	20-scf00021	20
AX-157919133	2	82.424	KV453193.1	6003436	20-scf00021	20
AX-157813033	2	82.488	KV453193.1	6010703	20-scf00021	20
AX-157919137	2	82.542	KV453193.1	6011141	20-scf00021	20
AX-157919138	2	82.542	KV453193.1	6011995	20-scf00021	20
AX-157813034	2	82.542	KV453193.1	6016175	20-scf00021	20
AX-157919141	2	82.652	KV453193.1	6017295	20-scf00021	20
AX-157919142	2	82.652	KV453193.1	6017695	20-scf00021	20
AX-157919143	2	82.652	KV453193.1	6018828	20-scf00021	20

AX-157813036	2	82.652	KV453193.1	6022359	20-scf00021	20
AX-157813037	2	82.761	KV453193.1	6024281	20-scf00021	20
AX-157699065	2	82.761	KV453193.1	6025481	20-scf00021	20
AX-157724874	2	82.87	KV453193.1	6030078	20-scf00021	20
AX-157720784	2	82.87	KV453193.1	6030400	20-scf00021	20
AX-157919148	2	82.87	KV453193.1	6031973	20-scf00021	20
AX-157919149	2	82.87	KV453193.1	6032472	20-scf00021	20
AX-157702971	2	82.87	DX095465.1	158	IORI212 Ictalu	UN
AX-157919152	2	82.978	KV453193.1	6040385	20-scf00021	20
AX-157919150	2	83.089	KV453193.1	6037113	20-scf00021	20
AX-157919154	2	83.089	KV453193.1	6041327	20-scf00021	20
AX-157919157	2	83.089	KV453193.1	6043197	20-scf00021	20
AX-157919158	2	83.089	KV453193.1	6044458	20-scf00021	20
AX-157919159	2	83.089	KV453193.1	6044981	20-scf00021	20
AX-157708490	2	83.089	KV453193.1	6115050	20-scf00021	20
AX-157919301	2	83.089	KV453193.1	6242750	20-scf00021	20
AX-157919162	2	83.198	KV453193.1	6048435	20-scf00021	20
AX-157919164	2	83.217	KV453193.1	6052180	20-scf00021	20
AX-157919168	2	83.244	KV453193.1	6054913	20-scf00021	20
AX-157919169	2	83.271	KV453193.1	6055512	20-scf00021	20
AX-157919171	2	83.307	KV453193.1	6056697	20-scf00021	20
AX-157919160	2	83.398	KV453193.1	6047476	20-scf00021	20
AX-157919173	2	83.398	KV453193.1	6058260	20-scf00021	20
AX-157919174	2	83.398	KV453193.1	6059709	20-scf00021	20
AX-157919189	2	83.398	KV453193.1	6078312	20-scf00021	20
AX-157919198	2	83.398	KV453193.1	6082738	20-scf00021	20
AX-157919209	2	83.398	KV453193.1	6099500	20-scf00021	20
AX-157919175	2	83.437	KV453193.1	6060614	20-scf00021	20
AX-157919172	2	83.526	KV453193.1	6057828	20-scf00021	20
AX-157919177	2	83.526	KV453193.1	6062629	20-scf00021	20
AX-157775300	2	83.526	KV453193.1	6063679	20-scf00021	20
AX-158405176	2	83.526	KV453193.1	6064836	20-scf00021	20
AX-157919181	2	83.526	KV453193.1	6069783	20-scf00021	20
AX-157813041	2	83.526	KV453193.1	6070008	20-scf00021	20
AX-157919182	2	83.526	KV453193.1	6070394	20-scf00021	20
AX-157919183	2	83.526	KV453193.1	6071259	20-scf00021	20
AX-157919187	2	83.526	KV453193.1	6075159	20-scf00021	20
AX-157919188	2	83.526	KV453193.1	6077689	20-scf00021	20
AX-157919190	2	83.526	KV453193.1	6079024	20-scf00021	20
AX-158379287	2	83.526	KV453193.1	6079132	20-scf00021	20
AX-157919191	2	83.526	KV453193.1	6079267	20-scf00021	20
AX-157919195	2	83.526	KV453193.1	6080766	20-scf00021	20
AX-157919196	2	83.526	KV453193.1	6081343	20-scf00021	20
AX-157919197	2	83.526	KV453193.1	6081858	20-scf00021	20
AX-157919199	2	83.526	KV453193.1	6082962	20-scf00021	20
AX-157919200	2	83.526	KV453193.1	6083769	20-scf00021	20
AX-157919202	2	83.526	KV453193.1	6085185	20-scf00021	20
AX-157919205	2	83.526	KV453193.1	6096160	20-scf00021	20
AX-157919206	2	83.526	KV453193.1	6097129	20-scf00021	20
AX-157919207	2	83.526	KV453193.1	6098211	20-scf00021	20
AX-157919210	2	83.526	KV453193.1	6099917	20-scf00021	20

AX-157919213	2	83.526	KV453193.1	6103276	20-scf00021	20
AX-157919216	2	83.526	KV453193.1	6106131	20-scf00021	20
AX-157743875	2	83.526	KV453193.1	6107584	20-scf00021	20
AX-157708048	2	83.526	KV453193.1	6118468	20-scf00021	20
AX-157758957	2	83.526	KV453193.1	6125525	20-scf00021	20
AX-157716011	2	83.853	KV453193.1	6105229	20-scf00021	20
AX-157813049	2	83.853	KV453193.1	6121652	20-scf00021	20
AX-157723881	2	83.853	KV453193.1	6123046	20-scf00021	20
AX-157919224	2	83.853	KV453193.1	6123935	20-scf00021	20
AX-157742407	2	83.853	KV453193.1	6123998	20-scf00021	20
AX-157919225	2	83.853	KV453193.1	6125482	20-scf00021	20
AX-157919226	2	83.853	KV453193.1	6127196	20-scf00021	20
AX-157720976	2	83.853	KV453193.1	6129279	20-scf00021	20
AX-157919227	2	83.853	KV453193.1	6130580	20-scf00021	20
AX-157758944	2	83.853	KV453193.1	6132746	20-scf00021	20
AX-157919229	2	83.853	KV453193.1	6133225	20-scf00021	20
AX-157919230	2	83.853	KV453193.1	6134314	20-scf00021	20
AX-157919253	2	83.853	KV453193.1	6171617	20-scf00021	20
AX-157919263	2	83.853	KV453193.1	6184315	20-scf00021	20
AX-157919232	2	83.89	KV453193.1	6137856	20-scf00021	20
AX-157919235	2	83.933	KV453193.1	6141032	20-scf00021	20
AX-157919220	2	83.962	KV453193.1	6117508	20-scf00021	20
AX-157919231	2	83.962	KV453193.1	6135676	20-scf00021	20
AX-157773796	2	83.962	KV453193.1	6142875	20-scf00021	20
AX-157919237	2	83.962	KV453193.1	6144525	20-scf00021	20
AX-157919238	2	83.962	KV453193.1	6145651	20-scf00021	20
AX-157919240	2	83.962	KV453193.1	6146663	20-scf00021	20
AX-157864537	2	83.962	KV453193.1	6147843	20-scf00021	20
AX-157919241	2	83.962	KV453193.1	6148301	20-scf00021	20
AX-157919243	2	83.962	KV453193.1	6149105	20-scf00021	20
AX-157813053	2	83.962	KV453193.1	6153455	20-scf00021	20
AX-157919246	2	84.181	KV453193.1	6158239	20-scf00021	20
AX-157919247	2	84.181	KV453193.1	6162741	20-scf00021	20
AX-157919249	2	84.181	KV453193.1	6164597	20-scf00021	20
AX-157919250	2	84.181	KV453193.1	6164902	20-scf00021	20
AX-157919251	2	84.181	KV453193.1	6165391	20-scf00021	20
AX-157813054	2	84.181	KV453193.1	6166119	20-scf00021	20
AX-157919254	2	84.181	KV453193.1	6172310	20-scf00021	20
AX-157919256	2	84.181	KV453193.1	6177015	20-scf00021	20
AX-157919257	2	84.181	KV453193.1	6178317	20-scf00021	20
AX-157919259	2	84.181	KV453193.1	6180430	20-scf00021	20
AX-157919260	2	84.181	KV453193.1	6182433	20-scf00021	20
AX-157724838	2	84.181	KV453193.1	6183697	20-scf00021	20
AX-157919262	2	84.181	KV453193.1	6183799	20-scf00021	20
AX-157919286	2	84.181	KV453193.1	6219853	20-scf00021	20
AX-157771387	2	84.181	KV453193.1	6223841	20-scf00021	20
AX-157919264	2	84.4	KV453193.1	6185845	20-scf00021	20
AX-157919266	2	84.4	KV453193.1	6187090	20-scf00021	20
AX-157919269	2	84.4	KV453193.1	6192216	20-scf00021	20
AX-157919282	2	84.4	KV453193.1	6215956	20-scf00021	20
AX-157919267	2	84.509	KV453193.1	6188353	20-scf00021	20

AX-157773923	2	84.509	KV453193.1	6199532	20-scf00021	20
AX-157778281	2	84.509	KV453193.1	6201619	20-scf00021	20
AX-157919274	2	84.509	KV453193.1	6202307	20-scf00021	20
AX-157919275	2	84.509	KV453193.1	6206289	20-scf00021	20
AX-157919277	2	84.509	KV453193.1	6207740	20-scf00021	20
AX-157919279	2	84.509	KV453193.1	6208846	20-scf00021	20
AX-157869136	2	84.509	KV453193.1	6213239	20-scf00021	20
AX-157919281	2	84.509	KV453193.1	6215216	20-scf00021	20
AX-157919287	2	84.509	KV453193.1	6220625	20-scf00021	20
AX-157919288	2	84.509	KV453193.1	6224013	20-scf00021	20
AX-157919289	2	84.509	KV453193.1	6225043	20-scf00021	20
AX-157919290	2	84.509	KV453193.1	6225476	20-scf00021	20
AX-157813058	2	84.525	KV453193.1	6227252	20-scf00021	20
AX-157813059	2	84.525	KV453193.1	6229006	20-scf00021	20
AX-157919305	2	84.525	KV453193.1	6246692	20-scf00021	20
AX-157919291	2	84.618	KV453193.1	6225967	20-scf00021	20
AX-157919293	2	84.618	KV453193.1	6230419	20-scf00021	20
AX-157919294	2	84.618	KV453193.1	6230836	20-scf00021	20
AX-157919295	2	84.618	KV453193.1	6232589	20-scf00021	20
AX-157919300	2	84.618	KV453193.1	6241897	20-scf00021	20
AX-157919302	2	84.618	KV453193.1	6243186	20-scf00021	20
AX-157919303	2	84.618	KV453193.1	6243642	20-scf00021	20
AX-157745462	2	84.618	KV453193.1	6244862	20-scf00021	20
AX-157758367	2	84.618	KV453193.1	6247587	20-scf00021	20
AX-157813061	2	84.618	KV453193.1	6250940	20-scf00021	20
AX-157713493	2	84.618	KV453193.1	6251145	20-scf00021	20
AX-157919307	2	84.618	KV453193.1	6252575	20-scf00021	20
AX-157919384	2	84.618	KV453193.1	6358062	20-scf00021	20
AX-157919306	2	84.644	KV453193.1	6248749	20-scf00021	20
AX-157919308	2	84.644	KV453193.1	6253309	20-scf00021	20
AX-157919311	2	84.644	KV453193.1	6257837	20-scf00021	20
AX-157813063	2	84.644	KV453193.1	6268473	20-scf00021	20
AX-157919310	2	84.781	KV453193.1	6257185	20-scf00021	20
AX-157919315	2	84.945	KV453193.1	6265582	20-scf00021	20
AX-157714238	2	84.945	KV453193.1	6269042	20-scf00021	20
AX-157919316	2	84.945	KV453193.1	6272163	20-scf00021	20
AX-157813064	2	84.945	KV453193.1	6273969	20-scf00021	20
AX-157919317	2	84.945	KV453193.1	6274048	20-scf00021	20
AX-157813065	2	84.945	KV453193.1	6274290	20-scf00021	20
AX-157919319	2	84.945	KV453193.1	6276182	20-scf00021	20
AX-157919321	2	84.945	KV453193.1	6277695	20-scf00021	20
AX-157919323	2	84.945	KV453193.1	6280256	20-scf00021	20
AX-157813066	2	84.945	KV453193.1	6280538	20-scf00021	20
AX-157754570	2	84.945	KV453193.1	6281284	20-scf00021	20
AX-157919324	2	84.945	KV453193.1	6282746	20-scf00021	20
AX-157919329	2	84.945	KV453193.1	6289186	20-scf00021	20
AX-157919336	2	84.945	KV453193.1	6304965	20-scf00021	20
AX-157919339	2	84.945	KV453193.1	6309427	20-scf00021	20
AX-157919347	2	84.945	KV453193.1	6315459	20-scf00021	20
AX-158405178	2	84.945	KV453193.1	6354083	20-scf00021	20
AX-157919404	2	84.945	KV453193.1	6382347	20-scf00021	20

AX-157919325	2	84.98	KV453193.1	6283193	20-scf00021	20
AX-157919326	2	85.012	KV453193.1	6284269	20-scf00021	20
AX-157919327	2	85.027	KV453193.1	6285190	20-scf00021	20
AX-157919332	2	85.027	KV453193.1	6294627	20-scf00021	20
AX-157813068	2	85.027	KV453193.1	6300376	20-scf00021	20
AX-157727577	2	85.027	KV453193.1	6356958	20-scf00021	20
AX-157919330	2	85.031	KV453193.1	6289971	20-scf00021	20
AX-157919333	2	85.055	KV453193.1	6303033	20-scf00021	20
AX-157919340	2	85.112	KV453193.1	6310372	20-scf00021	20
AX-157919342	2	85.164	KV453193.1	6312423	20-scf00021	20
AX-157919343	2	85.164	KV453193.1	6312824	20-scf00021	20
AX-157813069	2	85.164	KV453193.1	6313385	20-scf00021	20
AX-157919344	2	85.164	KV453193.1	6313810	20-scf00021	20
AX-157919345	2	85.164	KV453193.1	6314109	20-scf00021	20
AX-157919349	2	85.164	KV453193.1	6316462	20-scf00021	20
AX-157919350	2	85.164	KV453193.1	6316822	20-scf00021	20
AX-157919351	2	85.164	KV453193.1	6317307	20-scf00021	20
AX-157919352	2	85.164	KV453193.1	6317570	20-scf00021	20
AX-157919353	2	85.164	KV453193.1	6318093	20-scf00021	20
AX-157716871	2	85.164	KV453193.1	6319323	20-scf00021	20
AX-157919356	2	85.164	KV453193.1	6321609	20-scf00021	20
AX-157919358	2	85.164	KV453193.1	6324067	20-scf00021	20
AX-157919361	2	85.164	KV453193.1	6325489	20-scf00021	20
AX-157919362	2	85.164	KV453193.1	6330859	20-scf00021	20
AX-157694378	2	85.164	FI877934.1	384	ORI212 Ictalu	UN
AX-157689824	2	85.164	3ML01033034	298	scf08671_1	UN
AX-157919363	2	85.261	KV453193.1	6331426	20-scf00021	20
AX-157724624	2	85.382	KV453193.1	6335550	20-scf00021	20
AX-157919368	2	85.382	KV453193.1	6336424	20-scf00021	20
AX-157919371	2	85.382	KV453193.1	6339428	20-scf00021	20
AX-157919372	2	85.382	KV453193.1	6341190	20-scf00021	20
AX-157919373	2	85.382	KV453193.1	6342307	20-scf00021	20
AX-157919374	2	85.382	KV453193.1	6343978	20-scf00021	20
AX-157919377	2	85.382	KV453193.1	6347914	20-scf00021	20
AX-157919379	2	85.382	KV453193.1	6350472	20-scf00021	20
AX-157919385	2	85.382	KV453193.1	6359309	20-scf00021	20
AX-157919312	2	85.6	KV453193.1	6259368	20-scf00021	20
AX-157919383	2	85.6	KV453193.1	6353687	20-scf00021	20
AX-157919400	2	85.6	KV453193.1	6376761	20-scf00021	20
AX-157919402	2	85.6	KV453193.1	6380854	20-scf00021	20
AX-157919409	2	85.6	KV453193.1	6387350	20-scf00021	20
AX-157919389	2	85.817	KV453193.1	6364530	20-scf00021	20
AX-157919391	2	85.822	KV453193.1	6366376	20-scf00021	20
AX-157919392	2	85.822	KV453193.1	6366772	20-scf00021	20
AX-157919399	2	85.822	KV453193.1	6375694	20-scf00021	20
AX-157919405	2	85.985	KV453193.1	6382935	20-scf00021	20
AX-157919407	2	85.985	KV453193.1	6384544	20-scf00021	20
AX-157919408	2	85.985	KV453193.1	6385546	20-scf00021	20
AX-157757704	2	86.147	KV453193.1	6389270	20-scf00021	20
AX-158379290	2	86.147	KV453193.1	6391508	20-scf00021	20
AX-157919415	2	86.147	KV453193.1	6397368	20-scf00021	20

AX-158382296	2	86.147	KV453193.1	6398680	20-scf00021	20
AX-157919418	2	86.147	KV453193.1	6400231	20-scf00021	20
AX-157738976	2	86.147	KV453193.1	6405767	20-scf00021	20
AX-157919423	2	86.147	KV453193.1	6406906	20-scf00021	20
AX-157740245	2	86.147	KV453193.1	6408287	20-scf00021	20
AX-157919425	2	86.147	KV453193.1	6409351	20-scf00021	20
AX-157919438	2	86.147	KV453193.1	6429311	20-scf00021	20
AX-157919440	2	86.147	KV453193.1	6430654	20-scf00021	20
AX-157746978	2	86.257	KV453193.1	6413065	20-scf00021	20
AX-157883513	2	86.257	KV453193.1	6414565	20-scf00021	20
AX-157864539	2	86.257	KV453193.1	6416801	20-scf00021	20
AX-157919432	2	86.257	KV453193.1	6422414	20-scf00021	20
AX-157719682	2	86.257	KV453193.1	6423514	20-scf00021	20
AX-157919433	2	86.257	KV453193.1	6424625	20-scf00021	20
AX-157919434	2	86.257	KV453193.1	6425675	20-scf00021	20
AX-157919436	2	86.257	KV453193.1	6427089	20-scf00021	20
AX-157749730	2	86.257	KV453193.1	6428893	20-scf00021	20
AX-157919439	2	86.257	KV453193.1	6430123	20-scf00021	20
AX-157919446	2	86.269	KV453193.1	6435850	20-scf00021	20
AX-157919428	2	86.289	KV453193.1	6417769	20-scf00021	20
AX-157853770	2	86.289	KV453193.1	6436548	20-scf00021	20
AX-157919447	2	86.685	KV453193.1	6436686	20-scf00021	20
AX-157744629	2	86.688	KV453193.1	6439678	20-scf00021	20
AX-157710611	2	86.688	KV453193.1	6441229	20-scf00021	20
AX-157919450	2	86.913	KV453193.1	6442796	20-scf00021	20
AX-157919453	2	86.913	KV453193.1	6447765	20-scf00021	20
AX-157919454	2	86.913	KV453193.1	6447807	20-scf00021	20
AX-157853771	2	86.913	KV453193.1	6448204	20-scf00021	20
AX-157919455	2	86.913	KV453193.1	6448496	20-scf00021	20
AX-157919457	2	86.913	KV453193.1	6450728	20-scf00021	20
AX-157919459	2	86.913	KV453193.1	6451706	20-scf00021	20
AX-157919462	2	87.022	KV453193.1	6453630	20-scf00021	20
AX-157919463	2	87.022	KV453193.1	6454299	20-scf00021	20
AX-157919464	2	87.022	KV453193.1	6455583	20-scf00021	20
AX-157919466	2	87.131	KV453193.1	6457721	20-scf00021	20
AX-157747734	2	87.131	KV453193.1	6458320	20-scf00021	20
AX-157919467	2	87.131	KV453193.1	6460976	20-scf00021	20
AX-157919469	2	87.131	KV453193.1	6463066	20-scf00021	20
AX-157919470	2	87.131	KV453193.1	6464068	20-scf00021	20
AX-157919475	2	87.131	KV453193.1	6471793	20-scf00021	20
AX-157919476	2	87.131	KV453193.1	6472644	20-scf00021	20
AX-157919472	2	87.206	KV453193.1	6466734	20-scf00021	20
AX-157723611	2	87.274	KV453193.1	6468029	20-scf00021	20
AX-157919474	2	87.315	KV453193.1	6471253	20-scf00021	20
AX-157919477	2	87.35	KV453193.1	6473791	20-scf00021	20
AX-157919478	2	87.35	KV453193.1	6473968	20-scf00021	20
AX-157720679	2	87.35	KV453193.1	6478657	20-scf00021	20
AX-157718213	2	87.35	KV453193.1	6479290	20-scf00021	20
AX-157919480	2	87.539	KV453193.1	6479712	20-scf00021	20
AX-157919481	2	87.539	KV453193.1	6480868	20-scf00021	20
AX-157720111	2	87.539	KV453193.1	6484264	20-scf00021	20

AX-157773337	2	87.773	KV453193.1	6488407	20-scf00021	20
AX-157753265	2	87.773	KV453193.1	6491128	20-scf00021	20
AX-157919483	2	87.773	KV453193.1	6491625	20-scf00021	20
AX-157919499	2	88.526	KV453193.1	6519720	20-scf00021	20
AX-157754349	2	88.526	KV453193.1	6526784	20-scf00021	20
AX-157919506	2	88.53	KV453193.1	6538118	20-scf00021	20
AX-157919507	2	88.535	KV453193.1	6539016	20-scf00021	20
AX-157813077	2	88.692	KV453193.1	6535015	20-scf00021	20
AX-157919508	2	88.692	KV453193.1	6541471	20-scf00021	20
AX-157919511	2	89.099	KV453193.1	6545918	20-scf00021	20
AX-157919512	2	89.099	KV453193.1	6549326	20-scf00021	20
AX-158389640	2	89.099	KV453193.1	6553385	20-scf00021	20
AX-157813079	2	89.099	KV453193.1	6559268	20-scf00021	20
AX-157919515	2	89.099	KV453193.1	6561286	20-scf00021	20
AX-157919516	2	89.099	KV453193.1	6562430	20-scf00021	20
AX-157919517	2	89.099	KV453193.1	6563486	20-scf00021	20
AX-157919518	2	89.099	KV453193.1	6563841	20-scf00021	20
AX-157919533	2	89.099	KV453193.1	6597724	20-scf00021	20
AX-157919543	2	89.099	KV453193.1	6610593	20-scf00021	20
AX-157757054	2	89.205	KV453193.1	6565585	20-scf00021	20
AX-157919513	2	89.318	KV453193.1	6551628	20-scf00021	20
AX-157919520	2	89.318	KV453193.1	6565160	20-scf00021	20
AX-157755976	2	89.318	KV453193.1	6567193	20-scf00021	20
AX-157919521	2	89.318	KV453193.1	6570472	20-scf00021	20
AX-157919523	2	89.318	KV453193.1	6571929	20-scf00021	20
AX-157713232	2	89.318	KV453193.1	6576794	20-scf00021	20
AX-157919526	2	89.318	KV453193.1	6584114	20-scf00021	20
AX-157919527	2	89.318	KV453193.1	6586975	20-scf00021	20
AX-157919528	2	89.318	KV453193.1	6590196	20-scf00021	20
AX-157919529	2	89.318	KV453193.1	6591088	20-scf00021	20
AX-157919530	2	89.318	KV453193.1	6591694	20-scf00021	20
AX-157919531	2	89.318	KV453193.1	6593075	20-scf00021	20
AX-157919532	2	89.318	KV453193.1	6596247	20-scf00021	20
AX-157919534	2	89.318	KV453193.1	6599700	20-scf00021	20
AX-157919535	2	89.318	KV453193.1	6600249	20-scf00021	20
AX-157722235	2	89.318	KV453193.1	6603394	20-scf00021	20
AX-157727420	2	89.318	KV453193.1	6607207	20-scf00021	20
AX-157813080	2	89.318	KV453193.1	6608442	20-scf00021	20
AX-157919544	2	89.318	KV453193.1	6610803	20-scf00021	20
AX-157919546	2	89.318	KV453193.1	6614304	20-scf00021	20
AX-157725768	2	89.318	KV453193.1	6622306	20-scf00021	20
AX-157813082	2	89.318	KV453193.1	6629540	20-scf00021	20
AX-157919558	2	89.318	KV453193.1	6640643	20-scf00021	20
AX-157919552	2	89.646	KV453193.1	6631579	20-scf00021	20
AX-157919553	2	89.646	KV453193.1	6632164	20-scf00021	20
AX-157919554	2	89.755	KV453193.1	6634815	20-scf00021	20
AX-157919555	2	89.757	KV453193.1	6636061	20-scf00021	20
AX-157869138	2	90.079	KV453193.1	6637561	20-scf00021	20
AX-157714152	2	90.082	KV453193.1	6639671	20-scf00021	20
AX-157919557	2	90.082	KV453193.1	6640350	20-scf00021	20
AX-157919551	2	90.083	KV453193.1	6628645	20-scf00021	20

AX-157919560	2	90.083	KV453193.1	6650440	20-scf00021	20
AX-157813083	2	90.083	KV453193.1	6677766	20-scf00021	20
AX-157919579	2	90.083	KV453193.1	6682366	20-scf00021	20
AX-157713305	2	90.084	KV453193.1	6652515	20-scf00021	20
AX-157919547	2	90.361	KV453193.1	6615656	20-scf00021	20
AX-157919565	2	90.361	KV453193.1	6663116	20-scf00021	20
AX-157919566	2	90.361	KV453193.1	6663834	20-scf00021	20
AX-158405187	2	90.442	KV453193.1	6668448	20-scf00021	20
AX-157919569	2	90.467	KV453193.1	6670506	20-scf00021	20
AX-157919574	2	90.467	KV453193.1	6675004	20-scf00021	20
AX-157919571	2	90.489	KV453193.1	6671916	20-scf00021	20
AX-157919572	2	90.513	KV453193.1	6672790	20-scf00021	20
AX-157919580	2	90.513	KV453193.1	6685635	20-scf00021	20
AX-157919575	2	90.531	KV453193.1	6675589	20-scf00021	20
AX-157752206	2	90.554	KV453193.1	6677802	20-scf00021	20
AX-157919582	2	90.584	KV453193.1	6687785	20-scf00021	20
AX-157919586	2	90.584	KV453193.1	6694014	20-scf00021	20
AX-157919592	2	90.584	KV453193.1	6699692	20-scf00021	20
AX-157719981	2	90.62	KV453193.1	6688154	20-scf00021	20
AX-157919588	2	90.641	KV453193.1	6696188	20-scf00021	20
AX-157919593	2	90.641	KV453193.1	6701116	20-scf00021	20
AX-157919594	2	90.641	KV453193.1	6701478	20-scf00021	20
AX-157919596	2	90.641	KV453193.1	6703630	20-scf00021	20
AX-157919598	2	91.066	KV453193.1	6706097	20-scf00021	20
AX-157919601	2	91.066	KV453193.1	6711660	20-scf00021	20
AX-157919602	2	91.066	KV453193.1	6712752	20-scf00021	20
AX-157919604	2	91.066	KV453193.1	6713966	20-scf00021	20
AX-157919606	2	91.066	KV453193.1	6715150	20-scf00021	20
AX-157919608	2	91.066	KV453193.1	6716644	20-scf00021	20
AX-157919611	2	91.066	KV453193.1	6719730	20-scf00021	20
AX-157919612	2	91.066	KV453193.1	6720393	20-scf00021	20
AX-157919614	2	91.066	KV453193.1	6723660	20-scf00021	20
AX-157919621	2	91.066	KV453193.1	6737343	20-scf00021	20
AX-157919599	2	91.067	KV453193.1	6708419	20-scf00021	20
AX-158379293	2	91.067	KV453193.1	6722625	20-scf00021	20
AX-157919622	2	91.067	KV453193.1	6738026	20-scf00021	20
AX-157738302	2	91.067	KV453193.1	6740533	20-scf00021	20
AX-157919626	2	91.067	KV453193.1	6741093	20-scf00021	20
AX-157919631	2	91.067	KV453193.1	6747266	20-scf00021	20
AX-157741476	2	91.266	KV453193.1	6745052	20-scf00021	20
AX-157919632	2	91.394	KV453193.1	6748748	20-scf00021	20
AX-157709177	2	91.394	KV453193.1	6750172	20-scf00021	20
AX-157919636	2	91.436	KV453193.1	6755405	20-scf00021	20
AX-157919615	2	91.503	KV453193.1	6724380	20-scf00021	20
AX-157919633	2	91.503	KV453193.1	6750799	20-scf00021	20
AX-157919637	2	91.503	KV453193.1	6755895	20-scf00021	20
AX-157919641	2	91.503	KV453193.1	6761136	20-scf00021	20
AX-157919643	2	91.503	KV453193.1	6763352	20-scf00021	20
AX-157919644	2	91.503	KV453193.1	6764434	20-scf00021	20
AX-157919651	2	91.503	KV453193.1	6773277	20-scf00021	20
AX-157919653	2	91.584	KV453193.1	6777042	20-scf00021	20

AX-157919656	2	91.646	KV453193.1	6780273	20-scf00021	20
AX-157919652	2	91.722	KV453193.1	6776326	20-scf00021	20
AX-157755330	2	91.722	KV453193.1	6777533	20-scf00021	20
AX-157919657	2	91.722	KV453193.1	6780966	20-scf00021	20
AX-157919658	2	91.722	KV453193.1	6781748	20-scf00021	20
AX-157919660	2	91.722	KV453193.1	6784966	20-scf00021	20
AX-158379294	2	91.723	KV453193.1	6785300	20-scf00021	20
AX-157919664	2	91.723	KV453193.1	6788099	20-scf00021	20
AX-157919667	2	92.05	KV453193.1	6790476	20-scf00021	20
AX-157883514	2	92.05	KV453193.1	6790913	20-scf00021	20
AX-157919668	2	92.06	KV453193.1	6792736	20-scf00021	20
AX-157919673	2	92.596	KV453193.1	6796291	20-scf00021	20
AX-157919675	2	92.596	KV453193.1	6798205	20-scf00021	20
AX-157919680	2	92.596	KV453193.1	6805375	20-scf00021	20
AX-157726766	2	92.596	KV453193.1	6806689	20-scf00021	20
AX-157919683	2	92.596	KV453193.1	6819761	20-scf00021	20
AX-157919685	2	92.596	KV453193.1	6820689	20-scf00021	20
AX-157919724	2	92.596	KV453193.1	6881568	20-scf00021	20
AX-157706996	2	92.596	3ML01033304	400	scf08948_1	UN
AX-158389641	2	92.641	KV453193.1	6821221	20-scf00021	20
AX-157919686	2	92.669	KV453193.1	6823434	20-scf00021	20
AX-157919701	2	92.669	KV453193.1	6842297	20-scf00021	20
AX-157725312	2	92.677	KV453193.1	6826106	20-scf00021	20
AX-157919688	2	92.705	KV453193.1	6826714	20-scf00021	20
AX-157919689	2	92.705	KV453193.1	6827565	20-scf00021	20
AX-157919690	2	92.705	KV453193.1	6828429	20-scf00021	20
AX-157919692	2	92.705	KV453193.1	6829918	20-scf00021	20
AX-157919693	2	92.747	KV453193.1	6832406	20-scf00021	20
AX-157919629	2	92.815	KV453193.1	6743893	20-scf00021	20
AX-157919676	2	92.815	KV453193.1	6798747	20-scf00021	20
AX-157919682	2	92.815	KV453193.1	6810824	20-scf00021	20
AX-157853774	2	92.815	KV453193.1	6824642	20-scf00021	20
AX-157919695	2	92.815	KV453193.1	6834274	20-scf00021	20
AX-157772600	2	92.815	KV453193.1	6837021	20-scf00021	20
AX-157919697	2	92.815	KV453193.1	6838261	20-scf00021	20
AX-157874732	2	92.815	KV453193.1	6838329	20-scf00021	20
AX-157919699	2	92.815	KV453193.1	6839675	20-scf00021	20
AX-157919703	2	92.815	KV453193.1	6843797	20-scf00021	20
AX-157919704	2	92.815	KV453193.1	6844294	20-scf00021	20
AX-157919707	2	92.815	KV453193.1	6847534	20-scf00021	20
AX-157919708	2	92.815	KV453193.1	6848438	20-scf00021	20
AX-157722989	2	92.815	KV453193.1	6848729	20-scf00021	20
AX-157919709	2	92.815	KV453193.1	6849818	20-scf00021	20
AX-157919712	2	92.815	KV453193.1	6853329	20-scf00021	20
AX-157770718	2	92.815	KV453193.1	6872413	20-scf00021	20
AX-157740856	2	92.815	KV453193.1	6873017	20-scf00021	20
AX-157919735	2	92.815	KV453193.1	6896415	20-scf00021	20
AX-157919736	2	92.815	KV453193.1	6896823	20-scf00021	20
AX-157919737	2	92.815	KV453193.1	6897296	20-scf00021	20
AX-157813087	2	92.924	KV453193.1	6857329	20-scf00021	20
AX-157919713	2	92.924	KV453193.1	6857702	20-scf00021	20

AX-157770977	2	92.924	KV453193.1	6858322	20-scf00021	20
AX-157919714	2	92.924	KV453193.1	6861610	20-scf00021	20
AX-157919715	2	92.924	KV453193.1	6863815	20-scf00021	20
AX-157919717	2	92.924	KV453193.1	6866969	20-scf00021	20
AX-157919719	2	92.924	KV453193.1	6871029	20-scf00021	20
AX-157752373	2	92.924	KV453193.1	6883777	20-scf00021	20
AX-157919745	2	92.924	KV453193.1	6907892	20-scf00021	20
AX-157919789	2	92.924	KV453193.1	6986699	20-scf00021	20
AX-157680402	2	92.924	3ML01032679	296	scf08306_1	UN
AX-157919728	2	93.008	KV453193.1	6889689	20-scf00021	20
AX-157773974	2	93.142	KV453193.1	6894324	20-scf00021	20
AX-157919731	2	93.142	KV453193.1	6894838	20-scf00021	20
AX-157919739	2	93.142	KV453193.1	6899789	20-scf00021	20
AX-157919742	2	93.142	KV453193.1	6905022	20-scf00021	20
AX-157919744	2	93.142	KV453193.1	6907010	20-scf00021	20
AX-157919747	2	93.143	KV453193.1	6912541	20-scf00021	20
AX-157919749	2	93.143	KV453193.1	6915008	20-scf00021	20
AX-157724799	2	93.467	KV453193.1	6928695	20-scf00021	20
AX-157726108	2	93.467	KV453193.1	6933280	20-scf00021	20
AX-157919755	2	93.467	KV453193.1	6935144	20-scf00021	20
AX-157709449	2	93.468	KV453193.1	6939552	20-scf00021	20
AX-157919756	2	93.468	KV453193.1	6940134	20-scf00021	20
AX-157919758	2	93.468	KV453193.1	6942807	20-scf00021	20
AX-157919760	2	93.472	KV453193.1	6943996	20-scf00021	20
AX-157919761	2	93.472	KV453193.1	6947100	20-scf00021	20
AX-157919764	2	93.472	KV453193.1	6950041	20-scf00021	20
AX-157689764	2	93.472	3ML01032028	389	scf07645_1	UN
AX-157919768	2	93.688	KV453193.1	6956518	20-scf00021	20
AX-158382302	2	93.688	KV453193.1	6956880	20-scf00021	20
AX-157919769	2	93.688	KV453193.1	6957325	20-scf00021	20
AX-157772535	2	93.688	KV453193.1	6959687	20-scf00021	20
AX-157919774	2	93.688	KV453193.1	6963498	20-scf00021	20
AX-157883515	2	93.688	KV453193.1	6964504	20-scf00021	20
AX-157919776	2	93.688	KV453193.1	6965568	20-scf00021	20
AX-157919777	2	93.688	KV453193.1	6968479	20-scf00021	20
AX-157919784	2	93.688	KV453193.1	6979758	20-scf00021	20
AX-157752882	2	93.688	KV453193.1	6990170	20-scf00021	20
AX-157919778	2	93.712	KV453193.1	6970107	20-scf00021	20
AX-157919779	2	93.736	KV453193.1	6972969	20-scf00021	20
AX-157919782	2	93.756	KV453193.1	6974948	20-scf00021	20
AX-157919783	2	93.78	KV453193.1	6976976	20-scf00021	20
AX-157919741	2	93.797	KV453193.1	6904599	20-scf00021	20
AX-157919786	2	93.797	KV453193.1	6983907	20-scf00021	20
AX-157739166	2	93.797	KV453193.1	6986483	20-scf00021	20
AX-157919793	2	93.797	KV453193.1	6993177	20-scf00021	20
AX-157919794	2	93.797	KV453193.1	6994099	20-scf00021	20
AX-157751167	2	93.797	KV453193.1	6994695	20-scf00021	20
AX-157919802	2	93.797	KV453193.1	7013595	20-scf00021	20
AX-157919795	2	93.831	KV453193.1	6995816	20-scf00021	20
AX-157721594	2	93.907	KV453193.1	6997245	20-scf00021	20
AX-157919796	2	93.907	KV453193.1	6998539	20-scf00021	20

AX-157813089	2	93.907	KV453193.1	7000871	20-scf00021	20
AX-157714972	2	93.907	KV453193.1	7001574	20-scf00021	20
AX-157919798	2	94.016	KV453193.1	7004779	20-scf00021	20
AX-157726438	2	94.083	KV453193.1	7006535	20-scf00021	20
AX-158404346	2	94.235	KV453193.1	7011098	20-scf00021	20
AX-157919803	2	94.235	KV453193.1	7014490	20-scf00021	20
AX-157919807	2	94.235	KV453193.1	7016736	20-scf00021	20
AX-157919810	2	94.235	KV453193.1	7018770	20-scf00021	20
AX-158379296	2	94.235	KV453193.1	7023214	20-scf00021	20
AX-157813090	2	94.399	KV453193.1	7035542	20-scf00021	20
AX-157919826	2	94.471	KV453193.1	7038787	20-scf00021	20
AX-157751123	2	94.563	KV453193.1	7041182	20-scf00021	20
AX-157919828	2	94.563	KV453193.1	7043116	20-scf00021	20
AX-157919829	2	94.563	KV453193.1	7043997	20-scf00021	20
AX-157727700	2	94.563	KV453193.1	7044371	20-scf00021	20
AX-157919832	2	94.563	KV453193.1	7049913	20-scf00021	20
AX-157919839	2	94.563	KV453193.1	7061925	20-scf00021	20
AX-157874735	2	94.563	KV453193.1	7062520	20-scf00021	20
AX-157920072	2	94.563	KV453193.1	7460499	20-scf00021	20
AX-157919835	2	94.616	KV453193.1	7059392	20-scf00021	20
AX-157919824	2	94.672	KV453193.1	7037877	20-scf00021	20
AX-157919834	2	94.672	KV453193.1	7054433	20-scf00021	20
AX-157919836	2	94.672	KV453193.1	7059980	20-scf00021	20
AX-157919837	2	94.672	KV453193.1	7060955	20-scf00021	20
AX-157753177	2	94.672	KV453193.1	7066593	20-scf00021	20
AX-157813091	2	94.672	KV453193.1	7068251	20-scf00021	20
AX-157853776	2	94.672	KV453193.1	7068831	20-scf00021	20
AX-157813092	2	94.672	KV453193.1	7069940	20-scf00021	20
AX-157715230	2	94.672	KV453193.1	7070416	20-scf00021	20
AX-157813093	2	94.672	KV453193.1	7072018	20-scf00021	20
AX-157919846	2	94.672	KV453193.1	7076150	20-scf00021	20
AX-157919849	2	94.672	KV453193.1	7086124	20-scf00021	20
AX-157919922	2	94.672	KV453193.1	7193643	20-scf00021	20
AX-157813109	2	94.672	KV453193.1	7268948	20-scf00021	20
AX-157919973	2	94.672	KV453193.1	7274387	20-scf00021	20
AX-157750887	2	94.672	KV453193.1	7438166	20-scf00021	20
AX-157682408	2	94.672	KV453196.1	17673	20-scf00028	20
AX-157919813	2	94.721	KV453193.1	7023072	20-scf00021	20
AX-157919817	2	94.721	KV453193.1	7029423	20-scf00021	20
AX-157919847	2	94.721	KV453193.1	7076609	20-scf00021	20
AX-157919850	2	94.721	KV453193.1	7086492	20-scf00021	20
AX-157713353	2	94.818	KV453193.1	7093533	20-scf00021	20
AX-157919854	2	94.859	KV453193.1	7096942	20-scf00021	20
AX-157813098	2	94.908	KV453193.1	7097992	20-scf00021	20
AX-157919860	2	94.964	KV453193.1	7106231	20-scf00021	20
AX-157919861	2	94.964	KV453193.1	7108194	20-scf00021	20
AX-157813100	2	94.964	KV453193.1	7111220	20-scf00021	20
AX-158405191	2	94.964	KV453193.1	7112981	20-scf00021	20
AX-157919864	2	94.964	KV453193.1	7114776	20-scf00021	20
AX-158389643	2	94.964	KV453193.1	7115711	20-scf00021	20
AX-157919865	2	94.964	KV453193.1	7115888	20-scf00021	20

AX-157743784	2	94.964	KV453193.1	7116798	20-scf00021	20
AX-157919869	2	94.964	KV453193.1	7119259	20-scf00021	20
AX-157716923	2	95.014	KV453193.1	7116328	20-scf00021	20
AX-157919867	2	95.056	KV453193.1	7117686	20-scf00021	20
AX-157919872	2	95.07	KV453193.1	7122745	20-scf00021	20
AX-157919863	2	95.108	KV453193.1	7112207	20-scf00021	20
AX-157919874	2	95.108	KV453193.1	7126990	20-scf00021	20
AX-157919876	2	95.108	KV453193.1	7128810	20-scf00021	20
AX-157813102	2	95.108	KV453193.1	7163983	20-scf00021	20
AX-157919938	2	95.108	KV453193.1	7219136	20-scf00021	20
AX-157919877	2	95.109	KV453193.1	7129282	20-scf00021	20
AX-157919878	2	95.111	KV453193.1	7130874	20-scf00021	20
AX-157919879	2	95.113	KV453193.1	7133904	20-scf00021	20
AX-157919933	2	95.113	KV453193.1	7212305	20-scf00021	20
AX-157744247	2	95.113	KV453193.1	7413478	20-scf00021	20
AX-157920586	2	95.113	KV453198.1	119712	20-scf00025	20
AX-157920587	2	95.113	KV453198.1	120154	20-scf00025	20
AX-157920592	2	95.113	KV453198.1	130251	20-scf00025	20
AX-157682366	2	95.113	KV453195.1	59160	20-scf00026	20
AX-157680600	2	95.113	KV453195.1	71514	20-scf00026	20
AX-157680399	2	95.113	KV453196.1	9500	20-scf00028	20
AX-157919886	2	95.253	KV453193.1	7143418	20-scf00021	20
AX-157919887	2	95.4	KV453193.1	7148287	20-scf00021	20
AX-157919880	2	95.546	KV453193.1	7134444	20-scf00021	20
AX-157919889	2	95.546	KV453193.1	7152121	20-scf00021	20
AX-157919890	2	95.546	KV453193.1	7152563	20-scf00021	20
AX-157919897	2	95.546	KV453193.1	7156104	20-scf00021	20
AX-157919898	2	95.546	KV453193.1	7156330	20-scf00021	20
AX-157919903	2	95.694	KV453193.1	7162102	20-scf00021	20
AX-157919905	2	95.694	KV453193.1	7163829	20-scf00021	20
AX-157813110	2	95.694	KV453193.1	7289618	20-scf00021	20
AX-157919899	2	95.765	KV453193.1	7157544	20-scf00021	20
AX-157778299	2	95.765	KV453193.1	7164680	20-scf00021	20
AX-157919908	2	95.765	KV453193.1	7167231	20-scf00021	20
AX-157720169	2	95.765	KV453193.1	7179478	20-scf00021	20
AX-157919966	2	95.765	KV453193.1	7264346	20-scf00021	20
AX-157813101	2	95.874	KV453193.1	7156994	20-scf00021	20
AX-157919913	2	95.874	KV453193.1	7180726	20-scf00021	20
AX-157743160	2	95.874	KV453193.1	7181863	20-scf00021	20
AX-157919916	2	95.874	KV453193.1	7188542	20-scf00021	20
AX-157919918	2	95.874	KV453193.1	7190207	20-scf00021	20
AX-157919919	2	95.874	KV453193.1	7192219	20-scf00021	20
AX-157919920	2	95.874	KV453193.1	7192545	20-scf00021	20
AX-157919921	2	95.874	KV453193.1	7193453	20-scf00021	20
AX-157919923	2	95.874	KV453193.1	7194252	20-scf00021	20
AX-157919927	2	95.874	KV453193.1	7200622	20-scf00021	20
AX-157919928	2	95.874	KV453193.1	7200893	20-scf00021	20
AX-157919935	2	95.874	KV453193.1	7215028	20-scf00021	20
AX-157919936	2	95.874	KV453193.1	7217408	20-scf00021	20
AX-157919937	2	95.874	KV453193.1	7217778	20-scf00021	20
AX-157919940	2	95.874	KV453193.1	7220612	20-scf00021	20

AX-157919943	2	95.874	KV453193.1	7223033	20-scf00021	20
AX-157919944	2	95.874	KV453193.1	7223827	20-scf00021	20
AX-157919945	2	95.874	KV453193.1	7224217	20-scf00021	20
AX-157919946	2	95.874	KV453193.1	7224594	20-scf00021	20
AX-157919947	2	95.874	KV453193.1	7228217	20-scf00021	20
AX-157919953	2	95.874	KV453193.1	7239817	20-scf00021	20
AX-157919959	2	95.874	KV453193.1	7252324	20-scf00021	20
AX-157745386	2	95.874	KV453193.1	7268739	20-scf00021	20
AX-157919970	2	95.874	KV453193.1	7272486	20-scf00021	20
AX-157919978	2	95.874	KV453193.1	7276670	20-scf00021	20
AX-157919983	2	95.874	KV453193.1	7293101	20-scf00021	20
AX-157919984	2	95.874	KV453193.1	7293324	20-scf00021	20
AX-157919990	2	95.874	KV453193.1	7299729	20-scf00021	20
AX-157919993	2	95.874	KV453193.1	7305896	20-scf00021	20
AX-157919998	2	95.874	KV453193.1	7311318	20-scf00021	20
AX-157813114	2	95.874	KV453193.1	7311868	20-scf00021	20
AX-157919999	2	95.874	KV453193.1	7314724	20-scf00021	20
AX-157920001	2	95.874	KV453193.1	7321628	20-scf00021	20
AX-157723749	2	95.874	KV453193.1	7325923	20-scf00021	20
AX-158404356	2	95.874	KV453193.1	7341128	20-scf00021	20
AX-157920027	2	95.874	KV453193.1	7372943	20-scf00021	20
AX-157919967	2	96.033	KV453193.1	7264866	20-scf00021	20
AX-157813108	2	96.033	KV453193.1	7265730	20-scf00021	20
AX-157715015	2	96.033	KV453193.1	7288343	20-scf00021	20
AX-157919971	2	96.28	KV453193.1	7273244	20-scf00021	20
AX-157919974	2	96.361	KV453193.1	7274788	20-scf00021	20
AX-157813111	2	96.439	KV453193.1	7290009	20-scf00021	20
AX-157717869	2	96.439	KV453193.1	7312066	20-scf00021	20
AX-157919992	2	96.597	KV453193.1	7303378	20-scf00021	20
AX-157919995	2	96.613	KV453193.1	7309478	20-scf00021	20
AX-157919996	2	96.627	KV453193.1	7309808	20-scf00021	20
AX-157773344	2	96.641	KV453193.1	7099184	20-scf00021	20
AX-157919914	2	96.641	KV453193.1	7182273	20-scf00021	20
AX-157920010	2	96.641	KV453193.1	7331824	20-scf00021	20
AX-157920011	2	96.641	KV453193.1	7333145	20-scf00021	20
AX-158379298	2	96.641	KV453193.1	7338523	20-scf00021	20
AX-158404355	2	96.641	KV453193.1	7340326	20-scf00021	20
AX-157920577	2	96.641	KV453198.1	90643	20-scf00025	20
AX-157721041	2	96.641	KV453198.1	97334	20-scf00025	20
AX-157920012	2	96.75	KV453193.1	7337460	20-scf00021	20
AX-157920016	2	96.75	KV453193.1	7344197	20-scf00021	20
AX-157920020	2	96.75	KV453193.1	7353228	20-scf00021	20
AX-157748198	2	96.75	KV453193.1	7357378	20-scf00021	20
AX-157920022	2	96.75	KV453193.1	7364386	20-scf00021	20
AX-157920023	2	96.75	KV453193.1	7367640	20-scf00021	20
AX-157716107	2	96.75	KV453193.1	7368366	20-scf00021	20
AX-157920025	2	96.75	KV453193.1	7371671	20-scf00021	20
AX-157920047	2	96.75	KV453193.1	7406365	20-scf00021	20
AX-157920030	2	96.79	KV453193.1	7376972	20-scf00021	20
AX-157920031	2	96.805	KV453193.1	7379889	20-scf00021	20
AX-157920026	2	96.841	KV453193.1	7372063	20-scf00021	20

AX-157813115	2	96.841	KV453193.1	7384404	20-scf00021	20
AX-157743217	2	96.859	KV453193.1	7378877	20-scf00021	20
AX-157920032	2	96.859	KV453193.1	7382801	20-scf00021	20
AX-157920037	2	96.859	KV453193.1	7391625	20-scf00021	20
AX-157920038	2	96.859	KV453193.1	7392686	20-scf00021	20
AX-157813116	2	96.859	KV453193.1	7393649	20-scf00021	20
AX-157920039	2	96.859	KV453193.1	7394462	20-scf00021	20
AX-157920040	2	96.859	KV453193.1	7395301	20-scf00021	20
AX-157920044	2	96.859	KV453193.1	7403233	20-scf00021	20
AX-157920045	2	96.859	KV453193.1	7403851	20-scf00021	20
AX-157920051	2	96.859	KV453193.1	7417047	20-scf00021	20
AX-157745200	2	96.859	KV453193.1	7420264	20-scf00021	20
AX-157920054	2	96.859	KV453193.1	7422533	20-scf00021	20
AX-157920055	2	96.859	KV453193.1	7425773	20-scf00021	20
AX-157813121	2	96.859	KV453193.1	7427460	20-scf00021	20
AX-157813122	2	96.859	KV453193.1	7429186	20-scf00021	20
AX-157682341	2	96.859	KV453195.1	25233	20-scf00026	20
AX-157682363	2	96.859	KV453195.1	51649	20-scf00026	20
AX-157680599	2	96.859	KV453195.1	57368	20-scf00026	20
AX-157681316	2	96.859	KV453195.1	62793	20-scf00026	20
AX-157682388	2	96.859	KV453197.1	32196	20-scf00027	20
AX-157682403	2	96.859	KV453196.1	15253	20-scf00028	20
AX-157706256	2	96.859	BML01031912	84	scf07535_1	UN
AX-157727652	2	96.936	KV453193.1	7284112	20-scf00021	20
AX-157920046	2	96.936	KV453193.1	7405958	20-scf00021	20
AX-157920060	2	96.936	KV453193.1	7432363	20-scf00021	20
AX-157771183	2	96.936	KV453193.1	7478199	20-scf00021	20
AX-157682336	2	96.936	KV453195.1	16503	20-scf00026	20
AX-157682405	2	96.936	KV453196.1	16537	20-scf00028	20
AX-157712141	2	96.981	KV453193.1	7439164	20-scf00021	20
AX-157883524	2	97.03	KV453193.1	7430802	20-scf00021	20
AX-157757243	2	97.03	KV453193.1	7440097	20-scf00021	20
AX-157682398	2	97.03	KV453196.1	10419	20-scf00028	20
AX-157813126	2	97.078	KV453193.1	7445600	20-scf00021	20
AX-157920066	2	97.078	KV453193.1	7447377	20-scf00021	20
AX-157869139	2	97.078	KV453193.1	7451092	20-scf00021	20
AX-157920005	2	97.187	KV453193.1	7324913	20-scf00021	20
AX-157920029	2	97.187	KV453193.1	7375687	20-scf00021	20
AX-157920053	2	97.187	KV453193.1	7419896	20-scf00021	20
AX-157920069	2	97.187	KV453193.1	7455001	20-scf00021	20
AX-157710434	2	97.187	KV453193.1	7461209	20-scf00021	20
AX-157920074	2	97.187	KV453193.1	7463289	20-scf00021	20
AX-157920079	2	97.187	KV453193.1	7472561	20-scf00021	20
AX-157874739	2	97.187	KV453193.1	7475138	20-scf00021	20
AX-157920081	2	97.187	KV453193.1	7476748	20-scf00021	20
AX-157920085	2	97.187	KV453193.1	7479122	20-scf00021	20
AX-157750991	2	97.187	KV453193.1	7480159	20-scf00021	20
AX-157920562	2	97.187	KV453198.1	55730	20-scf00025	20
AX-157682381	2	97.187	KV453197.1	18295	20-scf00027	20
AX-157682382	2	97.187	KV453197.1	23763	20-scf00027	20
AX-157682390	2	97.187	KV453196.1	876	20-scf00028	20

AX-157682393	2	97.187	KV453196.1	4231	20-scf00028	20
AX-157682397	2	97.187	KV453196.1	9059	20-scf00028	20
AX-157682401	2	97.187	KV453196.1	13334	20-scf00028	20
AX-157682407	2	97.187	KV453196.1	17133	20-scf00028	20
AX-157681259	2	97.187	3ML01030031	335	scf05653_1	UN
AX-157919843	2	97.248	KV453193.1	7071164	20-scf00021	20
AX-157689178	2	97.248	KV453194.1	88	20-scf04092	20
AX-157920077	2	97.296	KV453193.1	7468521	20-scf00021	20
AX-157682337	2	97.296	KV453195.1	18911	20-scf00026	20
AX-157682340	2	97.296	KV453195.1	22742	20-scf00026	20
AX-157682343	2	97.296	KV453195.1	28587	20-scf00026	20
AX-157682346	2	97.296	KV453195.1	30269	20-scf00026	20
AX-157682347	2	97.296	KV453195.1	30460	20-scf00026	20
AX-157680086	2	97.296	KV453195.1	30900	20-scf00026	20
AX-157682349	2	97.296	KV453195.1	31829	20-scf00026	20
AX-157682351	2	97.296	KV453195.1	35793	20-scf00026	20
AX-157682354	2	97.296	KV453195.1	39468	20-scf00026	20
AX-157682355	2	97.296	KV453195.1	41188	20-scf00026	20
AX-157682358	2	97.296	KV453195.1	45452	20-scf00026	20
AX-157682359	2	97.296	KV453195.1	46472	20-scf00026	20
AX-157682365	2	97.296	KV453195.1	55564	20-scf00026	20
AX-157712135	2	97.296	KV453195.1	61513	20-scf00026	20
AX-157682376	2	97.296	KV453197.1	9543	20-scf00027	20
AX-157682385	2	97.296	KV453197.1	29418	20-scf00027	20
AX-157706138	2	97.296	3ML01031693	184	scf07312_1	UN
AX-157682391	2	97.405	KV453196.1	2121	20-scf00028	20
AX-157682400	2	97.405	KV453196.1	11593	20-scf00028	20
AX-157682411	2	97.405	KV453196.1	22314	20-scf00028	20
AX-157680509	2	97.405	KV453196.1	22649	20-scf00028	20
AX-157682374	2	97.515	KV453197.1	3268	20-scf00027	20
AX-157682375	2	97.515	KV453197.1	7799	20-scf00027	20
AX-157682377	2	97.515	KV453197.1	10565	20-scf00027	20
AX-157680492	2	97.515	KV453197.1	13083	20-scf00027	20
AX-157682379	2	97.515	KV453197.1	13549	20-scf00027	20
AX-157682384	2	97.515	KV453197.1	27395	20-scf00027	20
AX-157920028	2	97.624	KV453193.1	7375202	20-scf00021	20
AX-157920035	2	97.624	KV453193.1	7389144	20-scf00021	20
AX-157920041	2	97.624	KV453193.1	7398713	20-scf00021	20
AX-157920538	2	97.624	KV453198.1	16414	20-scf00025	20
AX-157853787	2	97.624	KV453198.1	46244	20-scf00025	20
AX-157920556	2	97.624	KV453198.1	48341	20-scf00025	20
AX-157920565	2	97.624	KV453198.1	58837	20-scf00025	20
AX-157920571	2	97.624	KV453198.1	66128	20-scf00025	20
AX-157920572	2	97.624	KV453198.1	67076	20-scf00025	20
AX-157713902	2	97.624	KV453198.1	67157	20-scf00025	20
AX-157920573	2	97.624	KV453198.1	68117	20-scf00025	20
AX-157920574	2	97.624	KV453198.1	69792	20-scf00025	20
AX-157742938	2	97.624	KV453198.1	75996	20-scf00025	20
AX-157813203	2	97.624	KV453198.1	82343	20-scf00025	20
AX-157920578	2	97.624	KV453198.1	93095	20-scf00025	20
AX-157920590	2	97.624	KV453198.1	127618	20-scf00025	20

AX-157920591	2	97.624	KV453198.1	128267	20-scf00025	20
AX-157753212	2	97.624	KV453198.1	128960	20-scf00025	20
AX-157745250	2	97.624	KV453198.1	129629	20-scf00025	20
AX-157920595	2	97.624	KV453198.1	144554	20-scf00025	20
AX-157920597	2	97.624	KV453198.1	145117	20-scf00025	20
AX-157680477	2	97.624	KV453195.1	50451	20-scf00026	20
AX-157682367	2	97.624	KV453195.1	61086	20-scf00026	20
AX-157708139	2	97.624	KV453195.1	61720	20-scf00026	20
AX-157682372	2	97.624	KV453195.1	70555	20-scf00026	20
AX-157682387	2	97.624	KV453197.1	31324	20-scf00027	20
AX-157682416	2	97.624	KV453199.1	14138	20-scf00029	20
AX-157682427	2	97.624	KV453199.1	28242	20-scf00029	20
AX-157682439	2	97.624	KV453199.1	38499	20-scf00029	20
AX-157681258	2	97.624	3ML01030031	239	scf05653_1	UN
AX-157705978	2	97.624	3ML01031356	309	scf06967_1	UN
AX-157920667	3	0	KV452720.1	8217	2-scf00030	2
AX-157920666	3	0	KV452720.1	10579	2-scf00030	2
AX-157920664	3	0	KV452720.1	15576	2-scf00030	2
AX-157920663	3	0	KV452720.1	15780	2-scf00030	2
AX-157920662	3	0	KV452720.1	16072	2-scf00030	2
AX-157920661	3	0	KV452720.1	17725	2-scf00030	2
AX-157920660	3	0	KV452720.1	20184	2-scf00030	2
AX-157920659	3	0	KV452720.1	21076	2-scf00030	2
AX-157920658	3	0	KV452720.1	21700	2-scf00030	2
AX-157920656	3	0	KV452720.1	31446	2-scf00030	2
AX-157920655	3	0	KV452720.1	36847	2-scf00030	2
AX-157920654	3	0	KV452720.1	39162	2-scf00030	2
AX-157920653	3	0	KV452720.1	39297	2-scf00030	2
AX-157920652	3	0	KV452720.1	40547	2-scf00030	2
AX-157920651	3	0	KV452720.1	42028	2-scf00030	2
AX-157813213	3	0	KV452720.1	44232	2-scf00030	2
AX-157920648	3	0	KV452720.1	46856	2-scf00030	2
AX-157920646	3	0	KV452720.1	48937	2-scf00030	2
AX-157920645	3	0	KV452720.1	50583	2-scf00030	2
AX-157920644	3	0	KV452720.1	51383	2-scf00030	2
AX-157920643	3	0	KV452720.1	52209	2-scf00030	2
AX-157920641	3	0	KV452720.1	55196	2-scf00030	2
AX-157813212	3	0	KV452720.1	55600	2-scf00030	2
AX-157740266	3	0	KV452720.1	57165	2-scf00030	2
AX-157920639	3	0	KV452720.1	58422	2-scf00030	2
AX-157813211	3	0	KV452720.1	61227	2-scf00030	2
AX-157813210	3	0	KV452720.1	61588	2-scf00030	2
AX-157920638	3	0	KV452720.1	63251	2-scf00030	2
AX-157750488	3	0	KV452720.1	64018	2-scf00030	2
AX-157920637	3	0	KV452720.1	65628	2-scf00030	2
AX-157920636	3	0	KV452720.1	71159	2-scf00030	2
AX-157920635	3	0	KV452720.1	75011	2-scf00030	2
AX-157920634	3	0	KV452720.1	78013	2-scf00030	2
AX-157920633	3	0	KV452720.1	78539	2-scf00030	2
AX-157920632	3	0	KV452720.1	79021	2-scf00030	2
AX-157920631	3	0	KV452720.1	83423	2-scf00030	2

AX-157813209	3	0	KV452720.1	84296	2-scf00030	2
AX-157920630	3	0	KV452720.1	84679	2-scf00030	2
AX-157920629	3	0	KV452720.1	85364	2-scf00030	2
AX-157920627	3	0	KV452720.1	93968	2-scf00030	2
AX-157920626	3	0	KV452720.1	95582	2-scf00030	2
AX-157748858	3	0	KV452720.1	97135	2-scf00030	2
AX-157920625	3	0	KV452720.1	99106	2-scf00030	2
AX-157813206	3	0	KV452720.1	106681	2-scf00030	2
AX-157744463	3	0	KV452720.1	106815	2-scf00030	2
AX-157920624	3	0	KV452720.1	107429	2-scf00030	2
AX-157920623	3	0	KV452720.1	108961	2-scf00030	2
AX-157920622	3	0	KV452720.1	113357	2-scf00030	2
AX-157920621	3	0	KV452720.1	115542	2-scf00030	2
AX-157920620	3	0	KV452720.1	119161	2-scf00030	2
AX-157920618	3	0	KV452720.1	120813	2-scf00030	2
AX-157920615	3	0	KV452720.1	123881	2-scf00030	2
AX-157813205	3	0	KV452720.1	123978	2-scf00030	2
AX-157920614	3	0	KV452720.1	124525	2-scf00030	2
AX-157920613	3	0	KV452720.1	125472	2-scf00030	2
AX-157920612	3	0	KV452720.1	126812	2-scf00030	2
AX-157920611	3	0	KV452720.1	127497	2-scf00030	2
AX-157920610	3	0	KV452720.1	128957	2-scf00030	2
AX-157813204	3	0	KV452720.1	134801	2-scf00030	2
AX-157920606	3	0	KV452720.1	141972	2-scf00030	2
AX-157920605	3	0	KV452720.1	144079	2-scf00030	2
AX-157920604	3	0	KV452720.1	149686	2-scf00030	2
AX-157920601	3	0	KV452720.1	152361	2-scf00030	2
AX-157920599	3	0	KV452720.1	153759	2-scf00030	2
AX-157920598	3	0	KV452720.1	154370	2-scf00030	2
AX-157920669	3	0	KV452720.1	156871	2-scf00030	2
AX-157920675	3	0	KV452720.1	182046	2-scf00030	2
AX-157920676	3	0	KV452720.1	183480	2-scf00030	2
AX-157920677	3	0	KV452720.1	183851	2-scf00030	2
AX-157920679	3	0	KV452720.1	188970	2-scf00030	2
AX-157920689	3	0	KV452720.1	204531	2-scf00030	2
AX-157920694	3	0	KV452720.1	221088	2-scf00030	2
AX-157813217	3	0	KV452720.1	236128	2-scf00030	2
AX-157920700	3	0	KV452720.1	239045	2-scf00030	2
AX-157920704	3	0	KV452720.1	260022	2-scf00030	2
AX-157920708	3	0	KV452720.1	290587	2-scf00030	2
AX-157920712	3	0	KV452720.1	298887	2-scf00030	2
AX-157920713	3	0	KV452720.1	299025	2-scf00030	2
AX-157920791	3	0	KV452720.1	312812	2-scf00030	2
AX-157920786	3	0	KV452720.1	318850	2-scf00030	2
AX-157920782	3	0	KV452720.1	327975	2-scf00030	2
AX-157920780	3	0	KV452720.1	330959	2-scf00030	2
AX-157920778	3	0	KV452720.1	333586	2-scf00030	2
AX-157853791	3	0	KV452720.1	346648	2-scf00030	2
AX-157920758	3	0	KV452720.1	374515	2-scf00030	2
AX-157920741	3	0	KV452720.1	404630	2-scf00030	2
AX-157920732	3	0	KV452720.1	421548	2-scf00030	2

AX-157813219	3	0	KV452720.1	422374	2-scf00030	2
AX-157869145	3	0	KV452720.1	430341	2-scf00030	2
AX-157920880	3	0	KV452722.1	2657	2-scf00034	2
AX-157920881	3	0	KV452722.1	4481	2-scf00034	2
AX-158389649	3	0	KV452722.1	7846	2-scf00034	2
AX-157920739	3	0.218	KV452720.1	407400	2-scf00030	2
AX-157920738	3	0.218	KV452720.1	408972	2-scf00030	2
AX-157920737	3	0.218	KV452720.1	410961	2-scf00030	2
AX-157920735	3	0.218	KV452720.1	416072	2-scf00030	2
AX-157920727	3	0.218	KV452720.1	432410	2-scf00030	2
AX-157920726	3	0.218	KV452720.1	434619	2-scf00030	2
AX-157920725	3	0.218	KV452720.1	434966	2-scf00030	2
AX-157920723	3	0.218	KV452720.1	437028	2-scf00030	2
AX-157853789	3	0.218	KV452720.1	441960	2-scf00030	2
AX-157738987	3	0.218	KV452722.1	27232	2-scf00034	2
AX-157920709	3	0.253	KV452720.1	291348	2-scf00030	2
AX-157813221	3	0.253	KV452720.1	391816	2-scf00030	2
AX-157920748	3	0.292	KV452720.1	390889	2-scf00030	2
AX-157920764	3	0.328	KV452720.1	354748	2-scf00030	2
AX-157920762	3	0.328	KV452720.1	356248	2-scf00030	2
AX-157920754	3	0.328	KV452720.1	381760	2-scf00030	2
AX-157920750	3	0.328	KV452720.1	389083	2-scf00030	2
AX-157813228	3	0.328	KV452721.1	12899	2-scf00033	2
AX-157774481	3	0.328	KV452721.1	18710	2-scf00033	2
AX-157920917	3	0.328	KV452722.1	73057	2-scf00034	2
AX-157705926	3	0.328	3ML01031248	585	scf06855_1	UN
AX-157853790	3	0.359	KV452720.1	359197	2-scf00030	2
AX-157920788	3	0.383	KV452720.1	315911	2-scf00030	2
AX-157920792	3	0.409	KV452720.1	302988	2-scf00030	2
AX-157920790	3	0.409	KV452720.1	313923	2-scf00030	2
AX-157920784	3	0.409	KV452720.1	324482	2-scf00030	2
AX-157883547	3	0.409	KV452720.1	352109	2-scf00030	2
AX-157920751	3	0.409	KV452720.1	388392	2-scf00030	2
AX-157920745	3	0.409	KV452720.1	393823	2-scf00030	2
AX-157853788	3	0.437	KV452720.1	141495	2-scf00030	2
AX-157920602	3	0.437	KV452720.1	152237	2-scf00030	2
AX-157920668	3	0.437	KV452720.1	156570	2-scf00030	2
AX-157813216	3	0.437	KV452720.1	187660	2-scf00030	2
AX-157920681	3	0.437	KV452720.1	193388	2-scf00030	2
AX-157920683	3	0.437	KV452720.1	194634	2-scf00030	2
AX-157920685	3	0.437	KV452720.1	197556	2-scf00030	2
AX-157920702	3	0.437	KV452720.1	246173	2-scf00030	2
AX-157920705	3	0.437	KV452720.1	261293	2-scf00030	2
AX-157864556	3	0.437	KV452720.1	263232	2-scf00030	2
AX-157813224	3	0.437	KV452720.1	306904	2-scf00030	2
AX-157920781	3	0.437	KV452720.1	330683	2-scf00030	2
AX-157920793	3	1.092	KV452721.1	2726	2-scf00033	2
AX-157920797	3	1.092	KV452721.1	11230	2-scf00033	2
AX-157813231	3	1.092	KV452721.1	30831	2-scf00033	2
AX-157813232	3	1.092	KV452721.1	31448	2-scf00033	2
AX-157920804	3	1.092	KV452721.1	32324	2-scf00033	2

AX-157920805	3	1.092	KV452721.1	32896	2-scf00033	2
AX-157920807	3	1.092	KV452721.1	34839	2-scf00033	2
AX-157920809	3	1.092	KV452721.1	39343	2-scf00033	2
AX-157920810	3	1.092	KV452721.1	39911	2-scf00033	2
AX-157920815	3	1.092	KV452721.1	44350	2-scf00033	2
AX-157725027	3	1.092	KV452721.1	49384	2-scf00033	2
AX-157920820	3	1.092	KV452721.1	50815	2-scf00033	2
AX-157920831	3	1.092	KV452721.1	65107	2-scf00033	2
AX-157920848	3	1.092	KV452721.1	98921	2-scf00033	2
AX-157920898	3	1.092	KV452722.1	30806	2-scf00034	2
AX-157920902	3	1.092	KV452722.1	36391	2-scf00034	2
AX-157920910	3	1.092	KV452722.1	59668	2-scf00034	2
AX-157920833	3	1.126	KV452721.1	71891	2-scf00033	2
AX-157721508	3	1.126	KV452721.1	73888	2-scf00033	2
AX-157874750	3	1.158	KV452721.1	79121	2-scf00033	2
AX-157920835	3	1.18	KV452721.1	80473	2-scf00033	2
AX-157920819	3	1.201	KV452721.1	50349	2-scf00033	2
AX-157920836	3	1.201	KV452721.1	80822	2-scf00033	2
AX-157920838	3	1.201	KV452721.1	83438	2-scf00033	2
AX-157745541	3	1.201	KV452721.1	83669	2-scf00033	2
AX-157920900	3	1.201	KV452722.1	32808	2-scf00034	2
AX-157920832	3	1.227	KV452721.1	67808	2-scf00033	2
AX-157920839	3	1.227	KV452721.1	88631	2-scf00033	2
AX-157920841	3	1.227	KV452721.1	89810	2-scf00033	2
AX-157920849	3	1.227	KV452721.1	99176	2-scf00033	2
AX-157920854	3	1.268	KV452721.1	105960	2-scf00033	2
AX-157748570	3	1.268	KV452722.1	62392	2-scf00034	2
AX-157920858	3	1.308	KV452721.1	123532	2-scf00033	2
AX-157920859	3	1.308	KV452721.1	124219	2-scf00033	2
AX-157920862	3	1.308	KV452721.1	127647	2-scf00033	2
AX-157920870	3	1.384	KV452721.1	141987	2-scf00033	2
AX-157920872	3	1.384	KV452721.1	143512	2-scf00033	2
AX-158405249	3	1.384	KV452723.1	241275	2-scf00035	2
AX-157688215	3	1.384	3ML01025761	524	scf01379_1	UN
AX-157920876	3	1.385	KV452721.1	150528	2-scf00033	2
AX-157920730	3	1.976	KV452720.1	423133	2-scf00030	2
AX-157920879	3	1.976	KV452722.1	815	2-scf00034	2
AX-157920882	3	1.976	KV452722.1	4957	2-scf00034	2
AX-157920884	3	1.976	KV452722.1	6295	2-scf00034	2
AX-157920885	3	1.976	KV452722.1	6963	2-scf00034	2
AX-157920888	3	1.976	KV452722.1	18111	2-scf00034	2
AX-157920891	3	1.976	KV452722.1	20064	2-scf00034	2
AX-157920892	3	1.976	KV452722.1	20441	2-scf00034	2
AX-157717961	3	1.976	KV452722.1	22124	2-scf00034	2
AX-157813244	3	1.976	KV452722.1	24396	2-scf00034	2
AX-157717763	3	2.085	KV452722.1	29535	2-scf00034	2
AX-157920897	3	2.085	KV452722.1	29927	2-scf00034	2
AX-157920801	3	2.302	KV452721.1	25388	2-scf00033	2
AX-157920903	3	2.302	KV452722.1	38655	2-scf00034	2
AX-157920905	3	2.302	KV452722.1	46966	2-scf00034	2
AX-157920906	3	2.302	KV452722.1	48658	2-scf00034	2

AX-157920909	3	2.303	KV452722.1	56760	2-scf00034	2
AX-157920914	3	2.303	KV452722.1	67154	2-scf00034	2
AX-157920915	3	2.304	KV452722.1	67970	2-scf00034	2
AX-157920922	3	2.714	KV452723.1	8559	2-scf00035	2
AX-157920925	3	2.714	KV452723.1	11205	2-scf00035	2
AX-157920926	3	2.714	KV452723.1	11491	2-scf00035	2
AX-157920927	3	2.714	KV452723.1	11860	2-scf00035	2
AX-157709115	3	2.714	KV452723.1	12629	2-scf00035	2
AX-157920798	3	3.124	KV452721.1	19573	2-scf00033	2
AX-157920938	3	3.124	KV452723.1	29748	2-scf00035	2
AX-157920939	3	3.124	KV452723.1	32451	2-scf00035	2
AX-157920946	3	3.124	KV452723.1	42316	2-scf00035	2
AX-157920947	3	3.124	KV452723.1	44166	2-scf00035	2
AX-157920948	3	3.124	KV452723.1	45265	2-scf00035	2
AX-157920949	3	3.124	KV452723.1	45666	2-scf00035	2
AX-157738580	3	3.124	KV452723.1	47638	2-scf00035	2
AX-157920950	3	3.124	KV452723.1	48229	2-scf00035	2
AX-157775086	3	3.124	KV452723.1	53126	2-scf00035	2
AX-157715533	3	3.124	KV452723.1	56124	2-scf00035	2
AX-157869152	3	3.124	KV452723.1	57709	2-scf00035	2
AX-157920953	3	3.124	KV452723.1	59597	2-scf00035	2
AX-157725114	3	3.124	KV452723.1	60360	2-scf00035	2
AX-157725690	3	3.261	KV452723.1	14850	2-scf00035	2
AX-157920955	3	3.261	KV452723.1	67088	2-scf00035	2
AX-157920957	3	3.261	KV452723.1	70446	2-scf00035	2
AX-157920962	3	3.261	KV452723.1	80376	2-scf00035	2
AX-158404389	3	3.261	KV452723.1	225655	2-scf00035	2
AX-157920964	3	3.392	KV452723.1	90605	2-scf00035	2
AX-157920972	3	3.392	KV452723.1	110295	2-scf00035	2
AX-157920987	3	3.392	KV452723.1	133768	2-scf00035	2
AX-157740623	3	3.396	KV452723.1	92982	2-scf00035	2
AX-157920970	3	3.396	KV452723.1	102334	2-scf00035	2
AX-157920971	3	3.396	KV452723.1	109591	2-scf00035	2
AX-157920974	3	3.396	KV452723.1	114138	2-scf00035	2
AX-157813252	3	3.396	KV452723.1	124338	2-scf00035	2
AX-157813253	3	3.396	KV452723.1	124612	2-scf00035	2
AX-157813254	3	3.396	KV452723.1	125806	2-scf00035	2
AX-157920982	3	3.396	KV452723.1	128078	2-scf00035	2
AX-157813255	3	3.396	KV452723.1	132118	2-scf00035	2
AX-157813257	3	3.396	KV452723.1	154186	2-scf00035	2
AX-157921026	3	3.396	KV452723.1	198980	2-scf00035	2
AX-157921027	3	3.396	KV452723.1	199443	2-scf00035	2
AX-157921058	3	3.396	KV452723.1	284162	2-scf00035	2
AX-157720610	3	3.412	KV452723.1	127670	2-scf00035	2
AX-157920988	3	3.429	KV452723.1	134238	2-scf00035	2
AX-158389650	3	3.45	KV452723.1	134435	2-scf00035	2
AX-157920992	3	3.454	KV452723.1	141155	2-scf00035	2
AX-157920996	3	3.468	KV452723.1	147562	2-scf00035	2
AX-157921004	3	3.489	KV452723.1	160152	2-scf00035	2
AX-157813251	3	3.505	KV452723.1	123728	2-scf00035	2
AX-157920984	3	3.505	KV452723.1	130041	2-scf00035	2

AX-157920989	3	3.505	KV452723.1	138983	2-scf00035	2
AX-157920993	3	3.505	KV452723.1	141561	2-scf00035	2
AX-157921007	3	3.505	KV452723.1	163895	2-scf00035	2
AX-157921012	3	3.505	KV452723.1	168996	2-scf00035	2
AX-157921015	3	3.505	KV452723.1	173187	2-scf00035	2
AX-157813259	3	3.505	KV452723.1	187162	2-scf00035	2
AX-157921019	3	3.505	KV452723.1	188592	2-scf00035	2
AX-157813260	3	3.505	KV452723.1	205803	2-scf00035	2
AX-158405247	3	3.505	KV452723.1	225609	2-scf00035	2
AX-157740670	3	3.505	KV452723.1	237604	2-scf00035	2
AX-158405248	3	3.505	KV452723.1	240855	2-scf00035	2
AX-158405256	3	3.505	KV452723.1	258871	2-scf00035	2
AX-157921044	3	3.505	KV452723.1	267223	2-scf00035	2
AX-157921043	3	3.589	KV452723.1	266782	2-scf00035	2
AX-157921046	3	3.656	KV452723.1	268265	2-scf00035	2
AX-157921050	3	3.724	KV452723.1	269745	2-scf00035	2
AX-157813263	3	3.942	KV452723.1	276852	2-scf00035	2
AX-157921053	3	3.942	KV452723.1	280651	2-scf00035	2
AX-157921057	3	3.942	KV452723.1	283546	2-scf00035	2
AX-157714642	3	3.943	KV452723.1	289902	2-scf00035	2
AX-157921061	3	3.943	KV452723.1	294047	2-scf00035	2
AX-157719596	3	3.984	KV452723.1	299524	2-scf00035	2
AX-157921065	3	4.161	KV452723.1	304281	2-scf00035	2
AX-157813266	3	4.161	KV452723.1	310557	2-scf00035	2
AX-157719160	3	4.161	KV452723.1	311288	2-scf00035	2
AX-157921071	3	4.161	KV452723.1	312801	2-scf00035	2
AX-157921072	3	4.161	KV452723.1	313631	2-scf00035	2
AX-157921073	3	4.261	KV452723.1	314448	2-scf00035	2
AX-157921074	3	4.379	KV452723.1	315126	2-scf00035	2
AX-157921076	3	4.379	KV452723.1	316035	2-scf00035	2
AX-157813268	3	4.379	KV452723.1	319990	2-scf00035	2
AX-157921077	3	4.379	KV452723.1	320568	2-scf00035	2
AX-157771251	3	4.379	KV452723.1	322956	2-scf00035	2
AX-157921088	3	4.379	KV452723.1	337595	2-scf00035	2
AX-157921089	3	4.379	KV452723.1	340807	2-scf00035	2
AX-157743714	3	4.379	KV452723.1	346241	2-scf00035	2
AX-157921064	3	4.489	KV452723.1	303104	2-scf00035	2
AX-157921066	3	4.489	KV452723.1	304773	2-scf00035	2
AX-157721330	3	4.489	KV452723.1	326708	2-scf00035	2
AX-157778477	3	4.489	KV452723.1	329168	2-scf00035	2
AX-157921086	3	4.489	KV452723.1	334517	2-scf00035	2
AX-157921087	3	4.489	KV452723.1	335190	2-scf00035	2
AX-157813271	3	4.489	KV452723.1	350851	2-scf00035	2
AX-157921092	3	4.489	KV452723.1	352059	2-scf00035	2
AX-157921169	3	4.489	KV452723.1	462594	2-scf00035	2
AX-157754789	3	4.515	KV452723.1	343986	2-scf00035	2
AX-157750017	3	4.598	KV452723.1	102659	2-scf00035	2
AX-157714016	3	4.598	KV452723.1	322551	2-scf00035	2
AX-157921085	3	4.598	KV452723.1	333449	2-scf00035	2
AX-157921091	3	4.598	KV452723.1	351661	2-scf00035	2
AX-157921093	3	4.598	KV452723.1	354131	2-scf00035	2

AX-157921096	3	4.598	KV452723.1	359110	2-scf00035	2
AX-157921097	3	4.598	KV452723.1	359601	2-scf00035	2
AX-157921098	3	4.598	KV452723.1	360306	2-scf00035	2
AX-157921101	3	4.598	KV452723.1	365028	2-scf00035	2
AX-157921102	3	4.598	KV452723.1	365740	2-scf00035	2
AX-157719329	3	4.598	KV452723.1	379136	2-scf00035	2
AX-157921109	3	4.598	KV452723.1	383006	2-scf00035	2
AX-157921110	3	4.598	KV452723.1	384491	2-scf00035	2
AX-157921113	3	4.598	KV452723.1	387292	2-scf00035	2
AX-157921115	3	4.598	KV452723.1	389862	2-scf00035	2
AX-157921116	3	4.598	KV452723.1	390377	2-scf00035	2
AX-157921123	3	4.598	KV452723.1	404797	2-scf00035	2
AX-157921124	3	4.598	KV452723.1	405424	2-scf00035	2
AX-157755144	3	4.598	KV452723.1	412060	2-scf00035	2
AX-157921128	3	4.598	KV452723.1	413701	2-scf00035	2
AX-157921132	3	4.598	KV452723.1	419011	2-scf00035	2
AX-157921135	3	4.598	KV452723.1	420529	2-scf00035	2
AX-157921141	3	4.598	KV452723.1	423799	2-scf00035	2
AX-157921144	3	4.598	KV452723.1	428452	2-scf00035	2
AX-157921147	3	4.598	KV452723.1	432333	2-scf00035	2
AX-157921122	3	4.823	KV452723.1	404021	2-scf00035	2
AX-157921148	3	4.823	KV452723.1	433312	2-scf00035	2
AX-157921152	3	4.823	KV452723.1	437237	2-scf00035	2
AX-157921158	3	4.826	KV452723.1	450238	2-scf00035	2
AX-157921160	3	4.832	KV452723.1	451532	2-scf00035	2
AX-157921162	3	5.235	KV452723.1	453207	2-scf00035	2
AX-157741007	3	5.237	KV452723.1	366886	2-scf00035	2
AX-157874754	3	5.237	KV452723.1	374467	2-scf00035	2
AX-157921126	3	5.237	KV452723.1	411622	2-scf00035	2
AX-157921166	3	5.237	KV452723.1	459836	2-scf00035	2
AX-157921167	3	5.237	KV452723.1	460247	2-scf00035	2
AX-157921175	3	5.237	KV452723.1	473735	2-scf00035	2
AX-157744444	3	5.237	KV452723.1	479955	2-scf00035	2
AX-157921177	3	5.237	KV452723.1	482950	2-scf00035	2
AX-157921179	3	5.237	KV452723.1	486340	2-scf00035	2
AX-157813273	3	5.237	KV452723.1	501553	2-scf00035	2
AX-157921184	3	5.237	KV452723.1	502601	2-scf00035	2
AX-157709016	3	5.238	KV452723.1	494656	2-scf00035	2
AX-157921183	3	5.238	KV452723.1	501393	2-scf00035	2
AX-157921189	3	5.8	KV452723.1	507393	2-scf00035	2
AX-157921192	3	5.8	KV452723.1	514045	2-scf00035	2
AX-157921197	3	5.8	KV452723.1	518694	2-scf00035	2
AX-157921200	3	5.8	KV452723.1	522279	2-scf00035	2
AX-157921204	3	5.8	KV452723.1	526923	2-scf00035	2
AX-157921206	3	5.8	KV452723.1	530041	2-scf00035	2
AX-157921207	3	5.8	KV452723.1	532038	2-scf00035	2
AX-157921208	3	5.946	KV452723.1	533643	2-scf00035	2
AX-157921214	3	5.99	KV452723.1	547885	2-scf00035	2
AX-157921215	3	6.062	KV452723.1	548493	2-scf00035	2
AX-158382324	3	6.092	KV452723.1	548981	2-scf00035	2
AX-157921216	3	6.092	KV452723.1	550772	2-scf00035	2

AX-157921194	3	6.238	KV452723.1	514797	2-scf00035	2
AX-157921196	3	6.238	KV452723.1	517177	2-scf00035	2
AX-157921205	3	6.238	KV452723.1	527291	2-scf00035	2
AX-157921221	3	6.238	KV452723.1	557236	2-scf00035	2
AX-157921222	3	6.238	KV452723.1	558123	2-scf00035	2
AX-157921223	3	6.238	KV452723.1	560434	2-scf00035	2
AX-157921225	3	6.238	KV452723.1	564570	2-scf00035	2
AX-157921224	3	6.326	KV452723.1	561015	2-scf00035	2
AX-157813276	3	6.408	KV452723.1	566486	2-scf00035	2
AX-157742718	3	6.454	KV452723.1	566999	2-scf00035	2
AX-157921229	3	6.5	KV452723.1	567517	2-scf00035	2
AX-157921230	3	6.539	KV452723.1	571341	2-scf00035	2
AX-157722931	3	6.539	KV452723.1	573778	2-scf00035	2
AX-157770683	3	6.539	KV452723.1	575468	2-scf00035	2
AX-157725269	3	6.539	KV452723.1	577080	2-scf00035	2
AX-157751736	3	6.539	KV452723.1	578626	2-scf00035	2
AX-157741062	3	6.539	KV452723.1	579909	2-scf00035	2
AX-157921235	3	6.77	KV452723.1	584170	2-scf00035	2
AX-157874756	3	6.77	KV452723.1	588523	2-scf00035	2
AX-157921239	3	6.77	KV452723.1	588948	2-scf00035	2
AX-157921240	3	6.77	KV452723.1	589985	2-scf00035	2
AX-157853795	3	6.801	KV452723.1	600269	2-scf00035	2
AX-157921245	3	7.003	KV452723.1	606080	2-scf00035	2
AX-157921247	3	7.003	KV452723.1	608517	2-scf00035	2
AX-157921249	3	7.003	KV452723.1	610223	2-scf00035	2
AX-157921251	3	7.003	KV452723.1	613270	2-scf00035	2
AX-157921255	3	7.254	KV452723.1	622340	2-scf00035	2
AX-157921257	3	7.254	KV452723.1	624560	2-scf00035	2
AX-157874758	3	7.254	KV452723.1	626064	2-scf00035	2
AX-157921258	3	7.254	KV452723.1	627172	2-scf00035	2
AX-157921260	3	7.254	KV452723.1	630266	2-scf00035	2
AX-157749986	3	7.254	KV452723.1	633871	2-scf00035	2
AX-157921264	3	7.639	KV452723.1	637217	2-scf00035	2
AX-157921284	3	7.639	KV452723.1	662178	2-scf00035	2
AX-157921286	3	7.639	KV452723.1	664668	2-scf00035	2
AX-157813280	3	7.639	KV452723.1	674966	2-scf00035	2
AX-157757812	3	7.64	KV452723.1	639145	2-scf00035	2
AX-157921265	3	7.642	KV452723.1	639954	2-scf00035	2
AX-157921266	3	7.642	KV452723.1	642154	2-scf00035	2
AX-157921268	3	7.642	KV452723.1	643287	2-scf00035	2
AX-157921269	3	7.878	KV452723.1	643971	2-scf00035	2
AX-157921273	3	7.974	KV452723.1	650481	2-scf00035	2
AX-157750691	3	7.993	KV452723.1	651112	2-scf00035	2
AX-157921275	3	7.993	KV452723.1	652505	2-scf00035	2
AX-157921278	3	8.128	KV452723.1	655808	2-scf00035	2
AX-157921281	3	8.207	KV452723.1	659646	2-scf00035	2
AX-157741544	3	8.208	KV452723.1	661071	2-scf00035	2
AX-157921285	3	8.305	KV452723.1	663053	2-scf00035	2
AX-158405259	3	8.398	KV452723.1	665554	2-scf00035	2
AX-157921289	3	8.436	KV452723.1	671378	2-scf00035	2
AX-158389654	3	8.536	KV452723.1	673592	2-scf00035	2

AX-157921291	3	8.536	KV452723.1	675086	2-scf00035	2
AX-157921292	3	8.536	KV452723.1	677619	2-scf00035	2
AX-157921294	3	8.536	KV452723.1	679491	2-scf00035	2
AX-157921297	3	8.536	KV452723.1	683456	2-scf00035	2
AX-157853796	3	8.536	KV452723.1	685962	2-scf00035	2
AX-157719848	3	8.536	KV452723.1	687086	2-scf00035	2
AX-157921307	3	8.552	KV452723.1	703532	2-scf00035	2
AX-157921309	3	9.039	KV452723.1	706748	2-scf00035	2
AX-157921299	3	9.053	KV452723.1	686695	2-scf00035	2
AX-157921311	3	9.053	KV452723.1	708848	2-scf00035	2
AX-157921313	3	9.053	KV452723.1	713371	2-scf00035	2
AX-157921314	3	9.053	KV452723.1	714389	2-scf00035	2
AX-157921316	3	9.239	KV452723.1	716268	2-scf00035	2
AX-157921317	3	9.259	KV452723.1	718154	2-scf00035	2
AX-157921322	3	9.482	KV452724.1	9922	2-scf00036	2
AX-157921324	3	9.482	KV452724.1	16166	2-scf00036	2
AX-157921327	3	9.599	KV452724.1	23220	2-scf00036	2
AX-157921328	3	9.73	KV452724.1	25249	2-scf00036	2
AX-157921333	3	9.73	KV452724.1	33101	2-scf00036	2
AX-157921325	3	9.959	KV452724.1	17723	2-scf00036	2
AX-157921329	3	9.959	KV452724.1	25730	2-scf00036	2
AX-157921332	3	9.959	KV452724.1	31953	2-scf00036	2
AX-157921334	3	9.959	KV452724.1	34081	2-scf00036	2
AX-157921337	3	9.959	KV452724.1	37681	2-scf00036	2
AX-157921339	3	9.959	KV452724.1	40226	2-scf00036	2
AX-157921340	3	9.959	KV452724.1	41470	2-scf00036	2
AX-157921342	3	9.959	KV452724.1	44654	2-scf00036	2
AX-157921345	3	9.959	KV452724.1	46358	2-scf00036	2
AX-157921347	3	9.959	KV452724.1	48921	2-scf00036	2
AX-157921370	3	9.959	KV452724.1	73848	2-scf00036	2
AX-157921389	3	9.959	KV452724.1	106723	2-scf00036	2
AX-157921450	3	9.959	KV452724.1	229934	2-scf00036	2
AX-157921462	3	9.959	KV452724.1	244158	2-scf00036	2
AX-157921476	3	9.959	KV452724.1	265194	2-scf00036	2
AX-157921497	3	9.959	KV452724.1	295496	2-scf00036	2
AX-157689905	3	9.959		298	scf11685	UN
AX-157921352	3	10.063	KV452724.1	53650	2-scf00036	2
AX-157921335	3	10.105	KV452724.1	34486	2-scf00036	2
AX-157921357	3	10.105	KV452724.1	59207	2-scf00036	2
AX-158382325	3	10.105	KV452724.1	59430	2-scf00036	2
AX-157921358	3	10.105	KV452724.1	59520	2-scf00036	2
AX-157921359	3	10.105	KV452724.1	60192	2-scf00036	2
AX-157921360	3	10.105	KV452724.1	61678	2-scf00036	2
AX-157921362	3	10.105	KV452724.1	62672	2-scf00036	2
AX-157921367	3	10.105	KV452724.1	70798	2-scf00036	2
AX-157921371	3	10.105	KV452724.1	74532	2-scf00036	2
AX-157746223	3	10.105	KV452724.1	83054	2-scf00036	2
AX-157921363	3	10.542	KV452724.1	63675	2-scf00036	2
AX-157921381	3	10.542	KV452724.1	92924	2-scf00036	2
AX-157813285	3	10.542	KV452724.1	94642	2-scf00036	2
AX-157921383	3	10.542	KV452724.1	94832	2-scf00036	2

AX-157752799	3	10.542	KV452724.1	180780	2-scf00036	2
AX-157921386	3	10.834	KV452724.1	103905	2-scf00036	2
AX-157921388	3	10.834	KV452724.1	105738	2-scf00036	2
AX-157751711	3	10.942	KV452724.1	121492	2-scf00036	2
AX-157813289	3	10.942	KV452724.1	122946	2-scf00036	2
AX-157752678	3	11.162	KV452724.1	127539	2-scf00036	2
AX-158421922	3	11.162	KV452724.1	129120	2-scf00036	2
AX-157921398	3	11.162	KV452724.1	129491	2-scf00036	2
AX-157921399	3	11.162	KV452724.1	132427	2-scf00036	2
AX-157921402	3	11.162	KV452724.1	134962	2-scf00036	2
AX-157813291	3	11.162	KV452724.1	135065	2-scf00036	2
AX-157921406	3	11.162	KV452724.1	147538	2-scf00036	2
AX-157921409	3	11.162	KV452724.1	150078	2-scf00036	2
AX-157921412	3	11.162	KV452724.1	159227	2-scf00036	2
AX-157707832	3	11.162	KV452724.1	234900	2-scf00036	2
AX-157921404	3	11.163	KV452724.1	143825	2-scf00036	2
AX-157921414	3	11.163	KV452724.1	160820	2-scf00036	2
AX-157771275	3	11.163	KV452724.1	161208	2-scf00036	2
AX-157921433	3	11.163	KV452724.1	196151	2-scf00036	2
AX-157921471	3	11.163	KV452724.1	257115	2-scf00036	2
AX-157921656	3	11.163	KV452724.1	598150	2-scf00036	2
AX-157757928	3	11.489	KV452724.1	137116	2-scf00036	2
AX-157921418	3	11.489	KV452724.1	174870	2-scf00036	2
AX-157921421	3	11.489	KV452724.1	177594	2-scf00036	2
AX-157813293	3	11.489	KV452724.1	182981	2-scf00036	2
AX-157921428	3	11.489	KV452724.1	190221	2-scf00036	2
AX-157921429	3	11.489	KV452724.1	191510	2-scf00036	2
AX-157921430	3	11.489	KV452724.1	191835	2-scf00036	2
AX-157921431	3	11.489	KV452724.1	193619	2-scf00036	2
AX-157775412	3	11.489	KV452724.1	194165	2-scf00036	2
AX-157921432	3	11.489	KV452724.1	194696	2-scf00036	2
AX-157709581	3	11.489	KV452724.1	203495	2-scf00036	2
AX-157921436	3	11.489	KV452724.1	203908	2-scf00036	2
AX-157921440	3	11.489	KV452724.1	210824	2-scf00036	2
AX-157921445	3	11.489	KV452724.1	221599	2-scf00036	2
AX-157921478	3	11.489	KV452724.1	269471	2-scf00036	2
AX-157921479	3	11.489	KV452724.1	270871	2-scf00036	2
AX-158405268	3	11.489	KV452724.1	286754	2-scf00036	2
AX-157707404	3	11.489	3ML01034210	257	scf09880_1	UN
AX-157921437	3	11.598	KV452724.1	204676	2-scf00036	2
AX-157813294	3	11.598	KV452724.1	208490	2-scf00036	2
AX-157921438	3	11.598	KV452724.1	208539	2-scf00036	2
AX-157921439	3	11.598	KV452724.1	209978	2-scf00036	2
AX-157921441	3	11.598	KV452724.1	212277	2-scf00036	2
AX-157921447	3	11.598	KV452724.1	225897	2-scf00036	2
AX-157921454	3	11.598	KV452724.1	235213	2-scf00036	2
AX-157921458	3	11.598	KV452724.1	239491	2-scf00036	2
AX-157921460	3	11.598	KV452724.1	241232	2-scf00036	2
AX-157921461	3	11.598	KV452724.1	242316	2-scf00036	2
AX-157921467	3	11.598	KV452724.1	249388	2-scf00036	2
AX-157921475	3	11.598	KV452724.1	264147	2-scf00036	2

AX-157755011	3	11.598	KV452724.1	267506	2-scf00036	2
AX-157921482	3	12.144	KV452724.1	275353	2-scf00036	2
AX-157813295	3	12.144	KV452724.1	278089	2-scf00036	2
AX-157921484	3	12.144	KV452724.1	278588	2-scf00036	2
AX-157813296	3	12.144	KV452724.1	279646	2-scf00036	2
AX-158405264	3	12.184	KV452724.1	281268	2-scf00036	2
AX-157921486	3	12.224	KV452724.1	282651	2-scf00036	2
AX-157921488	3	12.306	KV452724.1	285521	2-scf00036	2
AX-158405266	3	12.383	KV452724.1	286373	2-scf00036	2
AX-157921491	3	12.472	KV452724.1	288789	2-scf00036	2
AX-157921495	3	12.472	KV452724.1	293083	2-scf00036	2
AX-157921496	3	12.472	KV452724.1	293754	2-scf00036	2
AX-157774336	3	12.483	KV452724.1	295449	2-scf00036	2
AX-157921498	3	12.733	KV452724.1	299821	2-scf00036	2
AX-157921499	3	12.797	KV452724.1	300788	2-scf00036	2
AX-157757304	3	12.865	KV452724.1	304322	2-scf00036	2
AX-157921503	3	12.865	KV452724.1	305940	2-scf00036	2
AX-157921504	3	12.865	KV452724.1	307550	2-scf00036	2
AX-157813299	3	12.865	KV452724.1	308234	2-scf00036	2
AX-157751639	3	13.128	KV452724.1	321241	2-scf00036	2
AX-157921508	3	13.128	KV452724.1	321561	2-scf00036	2
AX-157921506	3	13.129	KV452724.1	311548	2-scf00036	2
AX-157813301	3	13.129	KV452724.1	313803	2-scf00036	2
AX-157921516	3	13.129	KV452724.1	332134	2-scf00036	2
AX-157813303	3	13.129	KV452724.1	336003	2-scf00036	2
AX-157717925	3	13.129	KV452724.1	337005	2-scf00036	2
AX-157921524	3	13.129	KV452724.1	348482	2-scf00036	2
AX-157921526	3	13.129	KV452724.1	350595	2-scf00036	2
AX-157921529	3	13.129	KV452724.1	358081	2-scf00036	2
AX-157921530	3	13.129	KV452724.1	358708	2-scf00036	2
AX-157744458	3	13.129	KV452724.1	368890	2-scf00036	2
AX-157813307	3	13.129	KV452724.1	383161	2-scf00036	2
AX-158389656	3	13.129	KV452724.1	404219	2-scf00036	2
AX-157771066	3	13.129	KV452724.1	524550	2-scf00036	2
AX-157921518	3	13.162	KV452724.1	334082	2-scf00036	2
AX-157749259	3	13.162	KV452724.1	359056	2-scf00036	2
AX-157813309	3	13.162	KV452724.1	386573	2-scf00036	2
AX-157921559	3	13.162	KV452724.1	412327	2-scf00036	2
AX-157921563	3	13.162	KV452724.1	417248	2-scf00036	2
AX-157921565	3	13.162	KV452724.1	426537	2-scf00036	2
AX-157921571	3	13.162	KV452724.1	430544	2-scf00036	2
AX-157755492	3	13.162	KV452724.1	437115	2-scf00036	2
AX-157921588	3	13.162	KV452724.1	447531	2-scf00036	2
AX-157874764	3	13.162	KV452724.1	484495	2-scf00036	2
AX-158405274	3	13.162	KV452724.1	509224	2-scf00036	2
AX-157921623	3	13.162	KV452724.1	516497	2-scf00036	2
AX-157921635	3	13.162	KV452724.1	537262	2-scf00036	2
AX-157921637	3	13.162	KV452724.1	541666	2-scf00036	2
AX-157921531	3	13.177	KV452724.1	361371	2-scf00036	2
AX-157720357	3	13.177	KV452724.1	362475	2-scf00036	2
AX-157813306	3	13.177	KV452724.1	381857	2-scf00036	2

AX-157921548	3	13.177	KV452724.1	397783	2-scf00036	2
AX-157813316	3	13.177	KV452724.1	521329	2-scf00036	2
AX-157921519	3	13.21	KV452724.1	335653	2-scf00036	2
AX-157921533	3	13.21	KV452724.1	364606	2-scf00036	2
AX-157682472	3	13.21	KV452727.1	59031	2-scf00038	2
AX-157921540	3	13.242	KV452724.1	383277	2-scf00036	2
AX-157813310	3	13.242	KV452724.1	399059	2-scf00036	2
AX-157921561	3	13.242	KV452724.1	413412	2-scf00036	2
AX-157813308	3	13.271	KV452724.1	385477	2-scf00036	2
AX-157921544	3	13.293	KV452724.1	393960	2-scf00036	2
AX-157921545	3	13.293	KV452724.1	395974	2-scf00036	2
AX-157712049	3	13.293	3ML01032515	259	scf08136_1	UN
AX-157921507	3	13.456	KV452724.1	315075	2-scf00036	2
AX-157921549	3	13.456	KV452724.1	398914	2-scf00036	2
AX-157921550	3	13.456	KV452724.1	399437	2-scf00036	2
AX-157921552	3	13.456	KV452724.1	402664	2-scf00036	2
AX-157813311	3	13.456	KV452724.1	405894	2-scf00036	2
AX-157747130	3	13.456	KV452724.1	408147	2-scf00036	2
AX-157921557	3	13.456	KV452724.1	410052	2-scf00036	2
AX-157853800	3	13.456	KV452724.1	415242	2-scf00036	2
AX-157775092	3	13.456	KV452724.1	415522	2-scf00036	2
AX-157921567	3	13.456	KV452724.1	427435	2-scf00036	2
AX-157921570	3	13.456	KV452724.1	429392	2-scf00036	2
AX-157921586	3	13.456	KV452724.1	447066	2-scf00036	2
AX-157921595	3	13.456	KV452724.1	453624	2-scf00036	2
AX-157921599	3	13.456	KV452724.1	457957	2-scf00036	2
AX-157921600	3	13.456	KV452724.1	458360	2-scf00036	2
AX-157921601	3	13.456	KV452724.1	459524	2-scf00036	2
AX-157921602	3	13.456	KV452724.1	465681	2-scf00036	2
AX-157921603	3	13.456	KV452724.1	469753	2-scf00036	2
AX-157921604	3	13.456	KV452724.1	470698	2-scf00036	2
AX-157921606	3	13.456	KV452724.1	480347	2-scf00036	2
AX-157921610	3	13.456	KV452724.1	485618	2-scf00036	2
AX-157921613	3	13.456	KV452724.1	494851	2-scf00036	2
AX-157921624	3	13.456	KV452724.1	520877	2-scf00036	2
AX-157921625	3	13.456	KV452724.1	522451	2-scf00036	2
AX-157921629	3	13.456	KV452724.1	527421	2-scf00036	2
AX-157921634	3	13.456	KV452724.1	535513	2-scf00036	2
AX-157921642	3	13.456	KV452724.1	559425	2-scf00036	2
AX-157813318	3	13.456	KV452724.1	559931	2-scf00036	2
AX-157921644	3	13.456	KV452724.1	560703	2-scf00036	2
AX-157921645	3	13.456	KV452724.1	565539	2-scf00036	2
AX-157921650	3	13.456	KV452724.1	577599	2-scf00036	2
AX-157682452	3	13.456	KV452726.1	10352	2-scf00037	2
AX-157682461	3	13.456	KV452726.1	33724	2-scf00037	2
AX-157687303	3	13.456	KV452727.1	98950	2-scf00038	2
AX-157679947	3	13.676	KV452727.1	80560	2-scf00038	2
AX-157681587	3	14.222	KV452727.1	61324	2-scf00038	2
AX-157680499	3	14.226	KV452727.1	60841	2-scf00038	2
AX-157682481	3	14.481	KV452727.1	42347	2-scf00038	2
AX-157682476	3	14.481	KV452727.1	55380	2-scf00038	2

AX-157682474	3	14.481	KV452727.1	56949	2-scf00038	2
AX-157682471	3	14.481	KV452727.1	59695	2-scf00038	2
AX-157681320	3	14.481	KV452727.1	60131	2-scf00038	2
AX-157682477	3	15.188	KV452727.1	54577	2-scf00038	2
AX-157682483	3	15.549	KV452727.1	36356	2-scf00038	2
AX-157682484	3	15.669	KV452727.1	29560	2-scf00038	2
AX-157682486	3	15.677	KV452727.1	26330	2-scf00038	2
AX-157682490	3	15.805	KV452727.1	16564	2-scf00038	2
AX-157681321	3	15.805	KV452727.1	31313	2-scf00038	2
AX-157682491	3	15.951	KV452727.1	13356	2-scf00038	2
AX-157687301	3	16.278	KV452727.1	95736	2-scf00038	2
AX-157682502	3	16.278	KV452729.1	2135	2-scf00039	2
AX-157705956	3	16.356	KV452729.1	4234	2-scf00039	2
AX-157921657	3	16.513	KV452730.1	579	2-scf00040	2
AX-158421929	3	16.513	KV452730.1	2505	2-scf00040	2
AX-157813319	3	16.613	KV452730.1	2668	2-scf00040	2
AX-157921663	3	16.649	KV452730.1	3495	2-scf00040	2
AX-157921664	3	16.97	KV452730.1	7148	2-scf00040	2
AX-157719839	3	16.97	KV452730.1	26479	2-scf00040	2
AX-157921723	3	16.97	KV452730.1	77572	2-scf00040	2
AX-157921668	3	16.976	KV452730.1	12667	2-scf00040	2
AX-157921672	3	16.976	KV452730.1	15403	2-scf00040	2
AX-157921682	3	17.135	KV452730.1	26946	2-scf00040	2
AX-158405280	3	17.135	KV452730.1	27571	2-scf00040	2
AX-157921684	3	17.173	KV452730.1	28298	2-scf00040	2
AX-157921687	3	17.295	KV452730.1	32008	2-scf00040	2
AX-157921693	3	17.295	KV452730.1	35358	2-scf00040	2
AX-157750723	3	17.295	KV452730.1	38249	2-scf00040	2
AX-157718314	3	17.295	KV452730.1	38894	2-scf00040	2
AX-157921698	3	17.295	KV452730.1	47620	2-scf00040	2
AX-157773208	3	17.55	KV452730.1	57651	2-scf00040	2
AX-157813320	3	17.55	KV452730.1	58753	2-scf00040	2
AX-157713100	3	17.55	KV452730.1	61248	2-scf00040	2
AX-157682504	3	17.644	KV452729.1	5863	2-scf00039	2
AX-157748444	3	17.644	KV452730.1	57073	2-scf00040	2
AX-157752306	3	17.644	KV452730.1	64815	2-scf00040	2
AX-157921726	3	17.644	KV452730.1	82094	2-scf00040	2
AX-157921731	3	17.644	KV452730.1	87099	2-scf00040	2
AX-157921719	3	17.934	KV452730.1	75378	2-scf00040	2
AX-157921721	3	17.934	KV452730.1	76657	2-scf00040	2
AX-157921727	3	18.061	KV452730.1	82518	2-scf00040	2
AX-157921732	3	18.061	KV452730.1	87997	2-scf00040	2
AX-157696702	3	18.061	KV452730.1	88240	2-scf00040	2
AX-157921735	3	18.061	KV452730.1	92612	2-scf00040	2
AX-157921738	3	18.061	KV452730.1	95916	2-scf00040	2
AX-157921740	3	18.061	KV452730.1	103040	2-scf00040	2
AX-157921741	3	18.061	KV452730.1	103299	2-scf00040	2
AX-157921744	3	18.089	KV452730.1	105742	2-scf00040	2
AX-157921749	3	18.122	KV452730.1	108210	2-scf00040	2
AX-157921750	3	18.171	KV452730.1	108998	2-scf00040	2
AX-157921754	3	18.171	KV452730.1	111682	2-scf00040	2

AX-157883558	3	18.171	KV452730.1	155654	2-scf00040	2
AX-157813323	3	18.171	KV452730.1	170703	2-scf00040	2
AX-158382336	3	18.172	KV452730.1	107609	2-scf00040	2
AX-157921762	3	18.172	KV452730.1	118323	2-scf00040	2
AX-157921763	3	18.172	KV452730.1	118843	2-scf00040	2
AX-157740456	3	18.172	KV452730.1	196717	2-scf00040	2
AX-157921768	3	18.173	KV452730.1	123177	2-scf00040	2
AX-157921775	3	18.496	KV452730.1	136199	2-scf00040	2
AX-157921778	3	18.496	KV452730.1	139127	2-scf00040	2
AX-157921788	3	18.502	KV452730.1	148209	2-scf00040	2
AX-157921791	3	18.502	KV452730.1	150685	2-scf00040	2
AX-157921795	3	18.502	KV452730.1	153436	2-scf00040	2
AX-157874768	3	18.692	KV452730.1	159602	2-scf00040	2
AX-157921805	3	18.883	KV452730.1	161604	2-scf00040	2
AX-157921809	3	18.883	KV452730.1	164374	2-scf00040	2
AX-157921812	3	18.883	KV452730.1	166721	2-scf00040	2
AX-157770923	3	18.883	KV452730.1	170070	2-scf00040	2
AX-157921821	3	18.883	KV452730.1	181428	2-scf00040	2
AX-157921824	3	19.263	KV452730.1	201994	2-scf00040	2
AX-157813322	3	19.264	KV452730.1	168366	2-scf00040	2
AX-157921825	3	19.264	KV452730.1	202218	2-scf00040	2
AX-157921831	3	19.264	KV452730.1	207310	2-scf00040	2
AX-157921833	3	19.264	KV452730.1	212283	2-scf00040	2
AX-157921834	3	19.264	KV452730.1	212831	2-scf00040	2
AX-157921835	3	19.264	KV452730.1	213557	2-scf00040	2
AX-157813329	3	19.264	KV452730.1	214145	2-scf00040	2
AX-157754974	3	19.264	KV452730.1	219519	2-scf00040	2
AX-157715963	3	19.264	KV452730.1	220411	2-scf00040	2
AX-157921842	3	19.264	KV452730.1	226542	2-scf00040	2
AX-157813334	3	19.264	KV452730.1	241183	2-scf00040	2
AX-157813336	3	19.264	KV452730.1	243616	2-scf00040	2
AX-157921865	3	19.264	KV452730.1	245386	2-scf00040	2
AX-157921886	3	19.264	KV452730.1	264867	2-scf00040	2
AX-157735700	3	19.264	KV452730.1	264947	2-scf00040	2
AX-157921839	3	19.289	KV452730.1	225247	2-scf00040	2
AX-157921854	3	19.335	KV452730.1	238015	2-scf00040	2
AX-157874770	3	19.335	KV452730.1	238464	2-scf00040	2
AX-157921856	3	19.335	KV452730.1	242261	2-scf00040	2
AX-157921860	3	19.373	KV452730.1	243570	2-scf00040	2
AX-157813337	3	19.373	KV452730.1	244186	2-scf00040	2
AX-157921862	3	19.373	KV452730.1	244845	2-scf00040	2
AX-156906872	3	19.373	KV452730.1	252361	2-scf00040	2
AX-157813338	3	19.458	KV452730.1	247463	2-scf00040	2
AX-157921872	3	19.543	KV452730.1	252405	2-scf00040	2
AX-157921875	3	19.59	KV452730.1	256363	2-scf00040	2
AX-157921879	3	19.59	KV452730.1	259507	2-scf00040	2
AX-157921888	3	19.59	KV452730.1	265486	2-scf00040	2
AX-157921889	3	19.59	KV452730.1	266412	2-scf00040	2
AX-157700819	3	19.59	FI872934.1	60	IORI212 Ictalu	UN
AX-157921891	3	19.7	KV452730.1	269591	2-scf00040	2
AX-157921894	3	19.81	KV452730.1	273896	2-scf00040	2

AX-157699907	3	19.811	KV452730.1	277144	2-scf00040	2
AX-157813343	3	19.812	KV452730.1	277401	2-scf00040	2
AX-157921898	3	19.914	KV452730.1	278200	2-scf00040	2
AX-157921899	3	19.955	KV452730.1	280236	2-scf00040	2
AX-157724930	3	19.955	KV452730.1	283427	2-scf00040	2
AX-157921901	3	19.955	KV452730.1	284528	2-scf00040	2
AX-157921904	3	19.955	KV452730.1	286721	2-scf00040	2
AX-157921905	3	19.955	KV452730.1	287287	2-scf00040	2
AX-157753180	3	19.955	KV452730.1	287514	2-scf00040	2
AX-157921919	3	19.955	KV452730.1	310336	2-scf00040	2
AX-157921869	3	19.956	KV452730.1	248603	2-scf00040	2
AX-157921902	3	19.956	KV452730.1	284808	2-scf00040	2
AX-157921909	3	19.956	KV452730.1	289738	2-scf00040	2
AX-157719985	3	20.246	KV452730.1	291347	2-scf00040	2
AX-157714225	3	20.246	KV452730.1	333445	2-scf00040	2
AX-157921873	3	20.247	KV452730.1	254363	2-scf00040	2
AX-157723031	3	20.247	KV452730.1	263268	2-scf00040	2
AX-157715865	3	20.247	KV452730.1	294542	2-scf00040	2
AX-157813344	3	20.247	KV452730.1	308981	2-scf00040	2
AX-157813345	3	20.247	KV452730.1	310976	2-scf00040	2
AX-157921921	3	20.247	KV452730.1	311281	2-scf00040	2
AX-157921931	3	20.247	KV452730.1	318128	2-scf00040	2
AX-157921933	3	20.247	KV452730.1	318594	2-scf00040	2
AX-157921939	3	20.247	KV452730.1	321440	2-scf00040	2
AX-157921941	3	20.247	KV452730.1	322266	2-scf00040	2
AX-157921946	3	20.247	KV452730.1	326209	2-scf00040	2
AX-157921947	3	20.247	KV452730.1	326865	2-scf00040	2
AX-157724188	3	20.247	KV452730.1	327867	2-scf00040	2
AX-157921949	3	20.247	KV452730.1	328805	2-scf00040	2
AX-157921951	3	20.247	KV452730.1	329680	2-scf00040	2
AX-157921954	3	20.247	KV452730.1	330707	2-scf00040	2
AX-157921956	3	20.247	KV452730.1	332451	2-scf00040	2
AX-157721704	3	20.247	KV452730.1	332957	2-scf00040	2
AX-157921962	3	20.247	KV452730.1	334749	2-scf00040	2
AX-157921965	3	20.247	KV452730.1	336001	2-scf00040	2
AX-157921967	3	20.247	KV452730.1	337130	2-scf00040	2
AX-157921969	3	20.247	KV452730.1	338117	2-scf00040	2
AX-157921972	3	20.247	KV452730.1	339310	2-scf00040	2
AX-158389660	3	20.247	KV452730.1	447529	2-scf00040	2
AX-157921978	3	20.466	KV452730.1	346106	2-scf00040	2
AX-157739939	3	20.466	KV452730.1	349447	2-scf00040	2
AX-157726124	3	20.466	KV452730.1	353951	2-scf00040	2
AX-157921982	3	20.466	KV452730.1	356229	2-scf00040	2
AX-157921983	3	20.684	KV452730.1	356564	2-scf00040	2
AX-157921986	3	20.684	KV452730.1	358398	2-scf00040	2
AX-157773636	3	20.684	KV452730.1	364839	2-scf00040	2
AX-157921988	3	20.79	KV452730.1	367704	2-scf00040	2
AX-157813346	3	21.012	KV452730.1	311104	2-scf00040	2
AX-158382339	3	21.012	KV452730.1	322815	2-scf00040	2
AX-157921970	3	21.012	KV452730.1	338392	2-scf00040	2
AX-158379303	3	21.012	KV452730.1	365034	2-scf00040	2

AX-157745556	3	21.012	KV452730.1	374596	2-scf00040	2
AX-157921993	3	21.012	KV452730.1	384957	2-scf00040	2
AX-157921995	3	21.012	KV452730.1	386916	2-scf00040	2
AX-157921999	3	21.012	KV452730.1	389052	2-scf00040	2
AX-157922001	3	21.012	KV452730.1	389759	2-scf00040	2
AX-157922003	3	21.012	KV452730.1	390383	2-scf00040	2
AX-157922007	3	21.012	KV452730.1	394521	2-scf00040	2
AX-157716426	3	21.012	KV452730.1	401072	2-scf00040	2
AX-157714467	3	21.012	KV452730.1	415504	2-scf00040	2
AX-157922030	3	21.012	KV452730.1	415905	2-scf00040	2
AX-158379304	3	21.012	KV452730.1	420344	2-scf00040	2
AX-157922055	3	21.012	KV452730.1	450909	2-scf00040	2
AX-157813357	3	21.012	KV452730.1	453550	2-scf00040	2
AX-157813351	3	21.121	KV452730.1	427878	2-scf00040	2
AX-157813352	3	21.121	KV452730.1	428349	2-scf00040	2
AX-157743934	3	21.149	KV452730.1	429775	2-scf00040	2
AX-157813353	3	21.179	KV452730.1	430204	2-scf00040	2
AX-157922043	3	21.186	KV452730.1	431968	2-scf00040	2
AX-157751391	3	21.21	KV452730.1	370879	2-scf00040	2
AX-157921990	3	21.21	KV452730.1	376604	2-scf00040	2
AX-157922045	3	21.21	KV452730.1	433550	2-scf00040	2
AX-157922046	3	21.21	KV452730.1	435789	2-scf00040	2
AX-157718139	3	21.231	KV452730.1	444679	2-scf00040	2
AX-157922050	3	21.231	KV452730.1	445311	2-scf00040	2
AX-157813356	3	21.231	KV452730.1	445625	2-scf00040	2
AX-157922060	3	21.449	KV452730.1	455700	2-scf00040	2
AX-157922065	3	21.668	KV452730.1	457611	2-scf00040	2
AX-157813359	3	21.668	KV452730.1	463399	2-scf00040	2
AX-157922078	3	21.668	KV452730.1	474354	2-scf00040	2
AX-157922079	3	21.668	KV452730.1	474933	2-scf00040	2
AX-157758335	3	21.668	KV452730.1	479657	2-scf00040	2
AX-157922089	3	21.668	KV452730.1	487405	2-scf00040	2
AX-157922095	3	21.832	KV452730.1	490459	2-scf00040	2
AX-157922097	3	21.832	KV452730.1	492370	2-scf00040	2
AX-157922100	3	21.832	KV452730.1	494680	2-scf00040	2
AX-157922101	3	21.832	KV452730.1	495303	2-scf00040	2
AX-157922104	3	21.908	KV452730.1	498599	2-scf00040	2
AX-157922105	3	21.908	KV452730.1	500631	2-scf00040	2
AX-157922109	3	21.94	KV452730.1	502733	2-scf00040	2
AX-157922110	3	21.996	KV452730.1	503254	2-scf00040	2
AX-157922112	3	22.106	KV452730.1	503623	2-scf00040	2
AX-157922114	3	22.106	KV452730.1	507164	2-scf00040	2
AX-157922116	3	22.106	KV452730.1	507784	2-scf00040	2
AX-157922117	3	22.106	KV452730.1	508238	2-scf00040	2
AX-157922124	3	22.106	KV452730.1	510392	2-scf00040	2
AX-157922125	3	22.106	KV452730.1	512335	2-scf00040	2
AX-157922147	3	22.106	KV452730.1	538066	2-scf00040	2
AX-158405290	3	22.106	KV452730.1	550005	2-scf00040	2
AX-157813364	3	22.324	KV452730.1	517476	2-scf00040	2
AX-157922128	3	22.324	KV452730.1	519591	2-scf00040	2
AX-157922133	3	22.324	KV452730.1	522209	2-scf00040	2

AX-157922137	3	22.324	KV452730.1	525181	2-scf00040	2
AX-158379305	3	22.324	KV452730.1	527509	2-scf00040	2
AX-157922141	3	22.324	KV452730.1	532219	2-scf00040	2
AX-157922142	3	22.324	KV452730.1	532690	2-scf00040	2
AX-157922145	3	22.429	KV452730.1	537621	2-scf00040	2
AX-157922148	3	22.525	KV452730.1	538593	2-scf00040	2
AX-157922155	3	22.821	KV452730.1	540350	2-scf00040	2
AX-157922157	3	22.824	KV452730.1	544234	2-scf00040	2
AX-157922169	3	23.053	KV452730.1	559647	2-scf00040	2
AX-157922174	3	23.747	KV452730.1	563813	2-scf00040	2
AX-157922180	3	23.77	KV452730.1	569216	2-scf00040	2
AX-157922181	3	23.783	KV452730.1	571704	2-scf00040	2
AX-157922166	3	23.788	KV452730.1	558384	2-scf00040	2
AX-157922185	3	23.788	KV452730.1	574992	2-scf00040	2
AX-157922198	3	24.364	KV452730.1	587998	2-scf00040	2
AX-157922199	3	24.364	KV452730.1	588486	2-scf00040	2
AX-157922202	3	24.364	KV452730.1	589412	2-scf00040	2
AX-157922213	3	24.982	KV452730.1	600117	2-scf00040	2
AX-157744614	3	24.982	KV452730.1	600550	2-scf00040	2
AX-157709769	3	24.982	KV452730.1	603910	2-scf00040	2
AX-157922215	3	24.982	KV452730.1	604807	2-scf00040	2
AX-157922217	3	24.982	KV452730.1	607834	2-scf00040	2
AX-157922219	3	24.982	KV452730.1	608204	2-scf00040	2
AX-157922230	3	24.985	KV452730.1	615318	2-scf00040	2
AX-157922232	3	24.988	KV452730.1	615801	2-scf00040	2
AX-158389661	3	24.991	KV452730.1	623037	2-scf00040	2
AX-157922240	3	25.184	KV452730.1	628740	2-scf00040	2
AX-157775628	3	25.184	KV452730.1	630353	2-scf00040	2
AX-157922239	3	25.395	KV452730.1	627207	2-scf00040	2
AX-157922246	3	25.395	KV452730.1	637730	2-scf00040	2
AX-157922247	3	25.395	KV452730.1	638199	2-scf00040	2
AX-157922249	3	25.395	KV452730.1	638657	2-scf00040	2
AX-157813367	3	25.395	KV452730.1	638840	2-scf00040	2
AX-157922252	3	25.395	KV452730.1	641585	2-scf00040	2
AX-157720153	3	25.825	KV452730.1	644563	2-scf00040	2
AX-157922253	3	25.829	KV452730.1	645061	2-scf00040	2
AX-157922255	3	25.831	KV452730.1	645916	2-scf00040	2
AX-157922257	3	25.832	KV452730.1	649409	2-scf00040	2
AX-157741436	3	25.832	KV452730.1	654240	2-scf00040	2
AX-157922261	3	25.832	KV452730.1	656094	2-scf00040	2
AX-157853807	3	25.832	KV452730.1	658366	2-scf00040	2
AX-157922262	3	25.969	KV452730.1	658961	2-scf00040	2
AX-157922264	3	26.084	KV452730.1	659231	2-scf00040	2
AX-157922265	3	26.217	KV452730.1	662087	2-scf00040	2
AX-157813368	3	26.218	KV452730.1	665145	2-scf00040	2
AX-157922270	3	26.597	KV452730.1	668496	2-scf00040	2
AX-157922276	3	26.599	KV452730.1	670997	2-scf00040	2
AX-157922278	3	26.602	KV452730.1	671526	2-scf00040	2
AX-157725301	3	26.604	KV452730.1	671857	2-scf00040	2
AX-157922280	3	26.721	KV452730.1	673370	2-scf00040	2
AX-157813369	3	26.801	KV452730.1	677079	2-scf00040	2

AX-157922288	3	26.858	KV452730.1	684433	2-scf00040	2
AX-157922293	3	26.858	KV452730.1	687925	2-scf00040	2
AX-157922294	3	26.858	KV452730.1	689275	2-scf00040	2
AX-157922295	3	26.862	KV452730.1	690717	2-scf00040	2
AX-157922298	3	27.12	KV452730.1	693286	2-scf00040	2
AX-157922299	3	27.382	KV452730.1	694651	2-scf00040	2
AX-157922301	3	27.387	KV452730.1	694901	2-scf00040	2
AX-157922303	3	27.387	KV452730.1	695746	2-scf00040	2
AX-157922307	3	27.422	KV452730.1	699612	2-scf00040	2
AX-157922312	3	27.422	KV452730.1	702834	2-scf00040	2
AX-157922322	3	27.422	KV452730.1	715552	2-scf00040	2
AX-157750956	3	27.422	KV452730.1	723835	2-scf00040	2
AX-157922318	3	27.648	KV452730.1	708048	2-scf00040	2
AX-157922328	3	27.648	KV452730.1	726376	2-scf00040	2
AX-157756246	3	27.733	KV452730.1	708408	2-scf00040	2
AX-157922323	3	27.814	KV452730.1	721160	2-scf00040	2
AX-157922325	3	27.911	KV452730.1	722584	2-scf00040	2
AX-157922327	3	27.911	KV452730.1	725368	2-scf00040	2
AX-157755001	3	27.911	KV452730.1	728183	2-scf00040	2
AX-157922330	3	28.085	KV452730.1	729100	2-scf00040	2
AX-157744516	3	28.085	KV452730.1	733734	2-scf00040	2
AX-157883565	3	28.085	KV452730.1	740087	2-scf00040	2
AX-157717484	3	28.086	KV452730.1	740370	2-scf00040	2
AX-157922338	3	28.435	KV452730.1	742229	2-scf00040	2
AX-157922342	3	28.436	KV452730.1	745264	2-scf00040	2
AX-157922339	3	28.785	KV452730.1	743308	2-scf00040	2
AX-157922344	3	28.785	KV452730.1	746874	2-scf00040	2
AX-157717275	3	28.785	KV452730.1	756360	2-scf00040	2
AX-157771089	3	28.785	KV452730.1	758544	2-scf00040	2
AX-157922347	3	28.785	KV452730.1	759312	2-scf00040	2
AX-157741154	3	28.785	KV452730.1	760384	2-scf00040	2
AX-157922350	3	28.785	KV452730.1	761474	2-scf00040	2
AX-157813377	3	28.785	KV452730.1	764637	2-scf00040	2
AX-157922358	3	28.785	KV452730.1	776723	2-scf00040	2
AX-157922360	3	28.785	KV452730.1	776990	2-scf00040	2
AX-157922364	3	28.785	KV452730.1	777890	2-scf00040	2
AX-157922429	3	28.785	KV452730.1	875065	2-scf00040	2
AX-157751521	3	29.003	KV452730.1	745652	2-scf00040	2
AX-157715922	3	29.003	KV452730.1	772144	2-scf00040	2
AX-157741174	3	29.003	KV452730.1	777582	2-scf00040	2
AX-157750200	3	29.003	KV452730.1	779384	2-scf00040	2
AX-157922367	3	29.003	KV452730.1	786708	2-scf00040	2
AX-157922368	3	29.003	KV452730.1	787811	2-scf00040	2
AX-157922369	3	29.003	KV452730.1	790315	2-scf00040	2
AX-157813381	3	29.003	KV452730.1	791447	2-scf00040	2
AX-157922371	3	29.003	KV452730.1	793267	2-scf00040	2
AX-157864573	3	29.003	KV452730.1	795099	2-scf00040	2
AX-157922374	3	29.003	KV452730.1	795758	2-scf00040	2
AX-157775894	3	29.374	KV452730.1	823313	2-scf00040	2
AX-157853809	3	29.374	KV452730.1	828206	2-scf00040	2
AX-157813383	3	29.534	KV452730.1	828854	2-scf00040	2

AX-157813384	3	29.635	KV452730.1	833179	2-scf00040	2
AX-157853810	3	29.756	KV452730.1	836885	2-scf00040	2
AX-157922398	3	29.756	KV452730.1	837792	2-scf00040	2
AX-157922402	3	29.763	KV452730.1	844387	2-scf00040	2
AX-157922427	3	29.763	KV452730.1	873288	2-scf00040	2
AX-157922444	3	29.763	KV452730.1	886831	2-scf00040	2
AX-157813382	3	29.771	KV452730.1	810340	2-scf00040	2
AX-157922404	3	29.771	KV452730.1	845465	2-scf00040	2
AX-157813386	3	29.771	KV452730.1	885006	2-scf00040	2
AX-157922456	3	29.771	KV452730.1	892671	2-scf00040	2
AX-157922405	3	29.782	KV452730.1	845777	2-scf00040	2
AX-157922411	3	29.988	KV452730.1	857360	2-scf00040	2
AX-157922413	3	29.988	KV452730.1	859631	2-scf00040	2
AX-157922414	3	29.988	KV452730.1	862594	2-scf00040	2
AX-158405294	3	29.988	KV452730.1	885855	2-scf00040	2
AX-157922454	3	29.988	KV452730.1	891932	2-scf00040	2
AX-157922464	3	29.988	KV452730.1	906757	2-scf00040	2
AX-157922422	3	30.097	KV452730.1	870163	2-scf00040	2
AX-157922426	3	30.097	KV452730.1	872470	2-scf00040	2
AX-157922428	3	30.097	KV452730.1	874639	2-scf00040	2
AX-157922430	3	30.097	KV452730.1	875975	2-scf00040	2
AX-157716214	3	30.097	KV452730.1	904744	2-scf00040	2
AX-157922432	3	30.106	KV452730.1	879994	2-scf00040	2
AX-157922434	3	30.11	KV452730.1	881423	2-scf00040	2
AX-157922441	3	30.116	KV452730.1	885455	2-scf00040	2
AX-157922445	3	30.119	KV452730.1	888005	2-scf00040	2
AX-157922417	3	30.121	KV452730.1	865308	2-scf00040	2
AX-157922421	3	30.121	KV452730.1	869668	2-scf00040	2
AX-157922424	3	30.121	KV452730.1	871598	2-scf00040	2
AX-157922433	3	30.121	KV452730.1	880541	2-scf00040	2
AX-157724498	3	30.121	KV452730.1	888774	2-scf00040	2
AX-157741705	3	30.121	KV452730.1	901174	2-scf00040	2
AX-157922440	3	30.223	KV452730.1	884962	2-scf00040	2
AX-157922458	3	30.223	KV452730.1	895192	2-scf00040	2
AX-157742768	3	30.224	KV452730.1	903951	2-scf00040	2
AX-157922467	3	30.224	KV452730.1	909721	2-scf00040	2
AX-157773128	3	30.228	KV452730.1	916135	2-scf00040	2
AX-157714363	3	30.42	KV452730.1	916510	2-scf00040	2
AX-157813388	3	30.749	KV452730.1	918807	2-scf00040	2
AX-157813389	3	30.754	KV452730.1	918966	2-scf00040	2
AX-157722875	3	30.754	KV452730.1	939377	2-scf00040	2
AX-158389662	3	30.754	KV452730.1	939444	2-scf00040	2
AX-157922475	3	30.754	KV452730.1	941556	2-scf00040	2
AX-157922478	3	30.754	KV452730.1	945540	2-scf00040	2
AX-157922482	3	30.754	KV452730.1	947256	2-scf00040	2
AX-157922490	3	30.754	KV452730.1	951802	2-scf00040	2
AX-157720141	3	30.754	KV452730.1	954325	2-scf00040	2
AX-157922495	3	30.754	KV452730.1	958247	2-scf00040	2
AX-157813390	3	30.852	KV452730.1	961144	2-scf00040	2
AX-157733966	3	30.936	KV452730.1	918641	2-scf00040	2
AX-157738619	3	30.936	KV452730.1	962446	2-scf00040	2

AX-157922503	3	30.936	KV452730.1	967843	2-scf00040	2
AX-157922509	3	30.936	KV452730.1	973611	2-scf00040	2
AX-157922507	3	31.027	KV452730.1	973235	2-scf00040	2
AX-157922525	3	31.305	KV452730.1	981359	2-scf00040	2
AX-157922529	3	31.305	KV452730.1	983887	2-scf00040	2
AX-157922531	3	31.305	KV452730.1	987409	2-scf00040	2
AX-157922535	3	31.306	KV452730.1	990172	2-scf00040	2
AX-157922547	3	31.474	KV452730.1	996694	2-scf00040	2
AX-157922548	3	31.532	KV452730.1	997240	2-scf00040	2
AX-157922556	3	31.532	KV452730.1	1008415	2-scf00040	2
AX-157922569	3	31.532	KV452730.1	1021769	2-scf00040	2
AX-157922572	3	31.532	KV452730.1	1024634	2-scf00040	2
AX-157922557	3	31.587	KV452730.1	1009374	2-scf00040	2
AX-157922521	3	31.847	KV452730.1	979205	2-scf00040	2
AX-157922538	3	31.847	KV452730.1	991759	2-scf00040	2
AX-157922559	3	31.847	KV452730.1	1010163	2-scf00040	2
AX-157922560	3	31.847	KV452730.1	1010730	2-scf00040	2
AX-157755150	3	31.847	KV452730.1	1011881	2-scf00040	2
AX-157922562	3	31.847	KV452730.1	1014237	2-scf00040	2
AX-157756932	3	31.847	KV452730.1	1018212	2-scf00040	2
AX-157922566	3	31.847	KV452730.1	1018687	2-scf00040	2
AX-157922568	3	31.847	KV452730.1	1021075	2-scf00040	2
AX-157720197	3	31.847	KV452730.1	1026278	2-scf00040	2
AX-157922575	3	31.847	KV452730.1	1026753	2-scf00040	2
AX-157922577	3	31.847	KV452730.1	1027988	2-scf00040	2
AX-157922579	3	31.847	KV452730.1	1030055	2-scf00040	2
AX-157922580	3	31.847	KV452730.1	1030503	2-scf00040	2
AX-157758386	3	31.847	KV452730.1	1033077	2-scf00040	2
AX-157748188	3	31.847	KV452730.1	1034400	2-scf00040	2
AX-157922585	3	31.847	KV452730.1	1035588	2-scf00040	2
AX-157922588	3	31.847	KV452730.1	1037572	2-scf00040	2
AX-157922593	3	31.847	KV452730.1	1042260	2-scf00040	2
AX-157922599	3	31.847	KV452730.1	1047827	2-scf00040	2
AX-157922600	3	31.847	KV452730.1	1048399	2-scf00040	2
AX-157922603	3	31.847	KV452730.1	1050010	2-scf00040	2
AX-157922617	3	31.847	KV452730.1	1065803	2-scf00040	2
AX-157922639	3	31.847	KV452730.1	1082620	2-scf00040	2
AX-158389663	3	31.907	KV452730.1	1043067	2-scf00040	2
AX-157922596	3	31.907	KV452730.1	1046648	2-scf00040	2
AX-157922607	3	31.907	KV452730.1	1053006	2-scf00040	2
AX-157922501	3	31.956	KV452730.1	963755	2-scf00040	2
AX-157755244	3	31.956	KV452730.1	1055458	2-scf00040	2
AX-157922610	3	31.956	KV452730.1	1056177	2-scf00040	2
AX-157922611	3	31.956	KV452730.1	1057736	2-scf00040	2
AX-158405297	3	31.956	KV452730.1	1060655	2-scf00040	2
AX-158405298	3	31.956	KV452730.1	1062123	2-scf00040	2
AX-157753214	3	31.956	KV452730.1	1063260	2-scf00040	2
AX-157813392	3	31.956	KV452730.1	1065284	2-scf00040	2
AX-157922620	3	31.956	KV452730.1	1069112	2-scf00040	2
AX-157922622	3	31.956	KV452730.1	1069725	2-scf00040	2
AX-157922625	3	31.956	KV452730.1	1071601	2-scf00040	2

AX-158378696	3	31.956	KV452730.1	1081865	2-scf00040	2
AX-157922641	3	31.956	KV452730.1	1084383	2-scf00040	2
AX-157922654	3	31.956	KV452730.1	1093098	2-scf00040	2
AX-157922545	3	32.21	KV452730.1	995531	2-scf00040	2
AX-157922646	3	32.21	KV452730.1	1090992	2-scf00040	2
AX-157716466	3	32.21	KV452730.1	1091467	2-scf00040	2
AX-157922652	3	32.21	KV452730.1	1092342	2-scf00040	2
AX-157922657	3	32.476	KV452730.1	1098162	2-scf00040	2
AX-158382342	3	32.722	KV452730.1	1003064	2-scf00040	2
AX-157862674	3	32.722	KV452730.1	1076675	2-scf00040	2
AX-157922659	3	32.722	KV452730.1	1099702	2-scf00040	2
AX-157922662	3	32.722	KV452730.1	1104767	2-scf00040	2
AX-158382347	3	32.722	KV452730.1	1105762	2-scf00040	2
AX-157723270	3	32.722	KV452730.1	1107539	2-scf00040	2
AX-157922670	3	32.722	KV452730.1	1113426	2-scf00040	2
AX-157922673	3	32.722	KV452730.1	1114350	2-scf00040	2
AX-157922680	3	32.722	KV452730.1	1118511	2-scf00040	2
AX-157922726	3	32.722	KV452730.1	1146507	2-scf00040	2
AX-157883576	3	32.94	KV452730.1	1120530	2-scf00040	2
AX-157922684	3	32.94	KV452730.1	1120570	2-scf00040	2
AX-157922695	3	32.94	KV452730.1	1124691	2-scf00040	2
AX-158382348	3	32.94	KV452730.1	1127201	2-scf00040	2
AX-157922699	3	32.94	KV452730.1	1128180	2-scf00040	2
AX-157922700	3	32.94	KV452730.1	1128280	2-scf00040	2
AX-157922702	3	32.94	KV452730.1	1131523	2-scf00040	2
AX-157922706	3	32.94	KV452730.1	1134505	2-scf00040	2
AX-157922707	3	32.94	KV452730.1	1135322	2-scf00040	2
AX-157922710	3	32.94	KV452730.1	1136518	2-scf00040	2
AX-157922712	3	32.94	KV452730.1	1136891	2-scf00040	2
AX-157922719	3	32.94	KV452730.1	1142276	2-scf00040	2
AX-157853822	3	32.94	KV452730.1	1173759	2-scf00040	2
AX-157922721	3	33.267	KV452730.1	1142829	2-scf00040	2
AX-157883578	3	33.267	KV452730.1	1159049	2-scf00040	2
AX-157922760	3	33.267	KV452730.1	1178425	2-scf00040	2
AX-157922742	3	33.268	KV452730.1	1161908	2-scf00040	2
AX-157922745	3	33.268	KV452730.1	1163543	2-scf00040	2
AX-157883579	3	33.268	KV452730.1	1165172	2-scf00040	2
AX-157922747	3	33.268	KV452730.1	1166092	2-scf00040	2
AX-157922750	3	33.268	KV452730.1	1168193	2-scf00040	2
AX-157922751	3	33.268	KV452730.1	1168566	2-scf00040	2
AX-157922754	3	33.268	KV452730.1	1169244	2-scf00040	2
AX-157922757	3	33.268	KV452730.1	1175335	2-scf00040	2
AX-158382352	3	33.34	KV452730.1	1169032	2-scf00040	2
AX-157922762	3	33.34	KV452730.1	1179167	2-scf00040	2
AX-157922783	3	33.34	KV452730.1	1192279	2-scf00040	2
AX-157922786	3	33.34	KV452730.1	1195235	2-scf00040	2
AX-157853824	3	33.477	KV452730.1	1185164	2-scf00040	2
AX-157922772	3	33.499	KV452730.1	1185916	2-scf00040	2
AX-157922774	3	33.617	KV452730.1	1186965	2-scf00040	2
AX-157922777	3	33.705	KV452730.1	1188668	2-scf00040	2
AX-157922779	3	33.705	KV452730.1	1190092	2-scf00040	2

AX-157922780	3	33.705	KV452730.1	1190666	2-scf00040	2
AX-157922785	3	33.705	KV452730.1	1194774	2-scf00040	2
AX-157922789	3	33.705	KV452730.1	1196178	2-scf00040	2
AX-157922791	3	33.705	KV452730.1	1197410	2-scf00040	2
AX-157853825	3	33.705	KV452730.1	1199862	2-scf00040	2
AX-157922795	3	33.705	KV452730.1	1200241	2-scf00040	2
AX-157922798	3	33.705	KV452730.1	1205219	2-scf00040	2
AX-157922834	3	33.705	KV452730.1	1227181	2-scf00040	2
AX-157922836	3	33.705	KV452730.1	1227756	2-scf00040	2
AX-157922842	3	33.705	KV452730.1	1231199	2-scf00040	2
AX-157922802	3	33.918	KV452730.1	1208965	2-scf00040	2
AX-157922817	3	33.923	KV452730.1	1217990	2-scf00040	2
AX-157922832	3	34.034	KV452730.1	1226748	2-scf00040	2
AX-157922838	3	34.149	KV452730.1	1228770	2-scf00040	2
AX-157922844	3	34.255	KV452730.1	1232101	2-scf00040	2
AX-157922849	3	34.258	KV452730.1	1235812	2-scf00040	2
AX-157922804	3	34.471	KV452730.1	1209631	2-scf00040	2
AX-157922852	3	34.471	KV452730.1	1237405	2-scf00040	2
AX-157922855	3	34.471	KV452730.1	1238385	2-scf00040	2
AX-157922857	3	34.471	KV452730.1	1238828	2-scf00040	2
AX-157922858	3	34.471	KV452730.1	1239553	2-scf00040	2
AX-157922860	3	34.471	KV452730.1	1240649	2-scf00040	2
AX-157922865	3	34.471	KV452730.1	1245624	2-scf00040	2
AX-157922867	3	34.471	KV452730.1	1246341	2-scf00040	2
AX-157922869	3	34.471	KV452730.1	1247893	2-scf00040	2
AX-157922872	3	34.471	KV452730.1	1249215	2-scf00040	2
AX-157922874	3	34.471	KV452730.1	1249572	2-scf00040	2
AX-157813398	3	34.471	KV452730.1	1253812	2-scf00040	2
AX-157922878	3	34.471	KV452730.1	1255421	2-scf00040	2
AX-157922882	3	34.471	KV452730.1	1258022	2-scf00040	2
AX-157922887	3	34.471	KV452730.1	1259484	2-scf00040	2
AX-157869162	3	34.471	KV452730.1	1260188	2-scf00040	2
AX-157723979	3	34.471	KV452730.1	1264495	2-scf00040	2
AX-157922896	3	34.471	KV452730.1	1269243	2-scf00040	2
AX-157922897	3	34.471	KV452730.1	1270676	2-scf00040	2
AX-157922903	3	34.471	KV452730.1	1276764	2-scf00040	2
AX-157853828	3	34.471	KV452730.1	1290421	2-scf00040	2
AX-157922910	3	34.689	KV452730.1	1281365	2-scf00040	2
AX-157741302	3	34.799	KV452730.1	1283724	2-scf00040	2
AX-157883586	3	34.799	KV452730.1	1284439	2-scf00040	2
AX-157853827	3	34.799	KV452730.1	1285472	2-scf00040	2
AX-157922919	3	34.799	KV452730.1	1285823	2-scf00040	2
AX-157922921	3	34.799	KV452730.1	1287264	2-scf00040	2
AX-157922923	3	34.799	KV452730.1	1287991	2-scf00040	2
AX-157922925	3	34.799	KV452730.1	1289326	2-scf00040	2
AX-157922926	3	34.799	KV452730.1	1290124	2-scf00040	2
AX-157755081	3	34.799	KV452730.1	1292828	2-scf00040	2
AX-157922930	3	34.799	KV452730.1	1295289	2-scf00040	2
AX-157922935	3	34.799	KV452730.1	1298084	2-scf00040	2
AX-157922938	3	34.799	KV452730.1	1300977	2-scf00040	2
AX-157883587	3	34.799	KV452730.1	1312929	2-scf00040	2

AX-158378698	3	35.147	KV452730.1	1309774	2-scf00040	2
AX-157744301	3	35.147	KV452730.1	1309974	2-scf00040	2
AX-157778575	3	35.269	KV452730.1	1310116	2-scf00040	2
AX-157739339	3	35.269	KV452730.1	1311686	2-scf00040	2
AX-158378699	3	35.269	KV452730.1	1315799	2-scf00040	2
AX-157922975	3	35.281	KV452730.1	1332196	2-scf00040	2
AX-157922982	3	35.415	KV452730.1	1337917	2-scf00040	2
AX-157922985	3	35.562	KV452730.1	1340019	2-scf00040	2
AX-157922988	3	35.562	KV452730.1	1340957	2-scf00040	2
AX-157853831	3	35.562	KV452730.1	1345293	2-scf00040	2
AX-157813404	3	35.562	KV452730.1	1353120	2-scf00040	2
AX-157922995	3	35.608	KV452730.1	1356137	2-scf00040	2
AX-158378701	3	35.614	KV452730.1	1379338	2-scf00040	2
AX-157778591	3	35.65	KV452730.1	1385538	2-scf00040	2
AX-157922949	3	35.68	KV452730.1	1311642	2-scf00040	2
AX-157922989	3	35.68	KV452730.1	1344800	2-scf00040	2
AX-157923010	3	35.68	KV452730.1	1375104	2-scf00040	2
AX-157778592	3	35.68	KV452730.1	1387043	2-scf00040	2
AX-157727523	3	35.68	KV452730.1	1387484	2-scf00040	2
AX-157923021	3	35.68	KV452730.1	1395115	2-scf00040	2
AX-157923023	3	35.68	KV452730.1	1397108	2-scf00040	2
AX-157923098	3	35.68	KV452730.1	1492859	2-scf00040	2
AX-157923025	3	35.709	KV452730.1	1400932	2-scf00040	2
AX-157923028	3	35.834	KV452730.1	1402461	2-scf00040	2
AX-157744037	3	35.856	KV452730.1	1404785	2-scf00040	2
AX-157923033	3	35.856	KV452730.1	1408277	2-scf00040	2
AX-157813416	3	35.856	KV452730.1	1409711	2-scf00040	2
AX-157923034	3	35.982	KV452730.1	1411622	2-scf00040	2
AX-157923030	3	36.002	KV452730.1	1405209	2-scf00040	2
AX-157717701	3	36.002	KV452730.1	1415226	2-scf00040	2
AX-157923040	3	36.002	KV452730.1	1419812	2-scf00040	2
AX-157923046	3	36.002	KV452730.1	1422660	2-scf00040	2
AX-157713283	3	36.002	KV452730.1	1423993	2-scf00040	2
AX-157923050	3	36.002	KV452730.1	1424768	2-scf00040	2
AX-157923053	3	36.002	KV452730.1	1426155	2-scf00040	2
AX-157923056	3	36.002	KV452730.1	1429390	2-scf00040	2
AX-157923059	3	36.002	KV452730.1	1431761	2-scf00040	2
AX-157754513	3	36.002	KV452730.1	1433363	2-scf00040	2
AX-157923068	3	36.002	KV452730.1	1439762	2-scf00040	2
AX-157813418	3	36.002	KV452730.1	1440231	2-scf00040	2
AX-157714526	3	36.002	KV452730.1	1440323	2-scf00040	2
AX-157923069	3	36.002	KV452730.1	1445890	2-scf00040	2
AX-157923071	3	36.002	KV452730.1	1448086	2-scf00040	2
AX-157853832	3	36.002	KV452730.1	1450386	2-scf00040	2
AX-157883591	3	36.439	KV452730.1	1457323	2-scf00040	2
AX-157923083	3	36.439	KV452730.1	1460233	2-scf00040	2
AX-157748142	3	36.439	KV452730.1	1464036	2-scf00040	2
AX-157813422	3	36.439	KV452730.1	1468350	2-scf00040	2
AX-157923086	3	36.439	KV452730.1	1471611	2-scf00040	2
AX-157923089	3	36.439	KV452730.1	1474541	2-scf00040	2
AX-157715588	3	36.439	KV452730.1	1475814	2-scf00040	2

AX-157923091	3	36.439	KV452730.1	1479791	2-scf00040	2
AX-157874786	3	36.439	KV452730.1	1480921	2-scf00040	2
AX-157923092	3	36.439	KV452730.1	1481466	2-scf00040	2
AX-157719484	3	36.439	KV452730.1	1487802	2-scf00040	2
AX-157713405	3	36.439	KV452730.1	1488685	2-scf00040	2
AX-157712237	3	36.439	KV452730.1	1489811	2-scf00040	2
AX-157923107	3	36.439	KV452730.1	1507769	2-scf00040	2
AX-157813424	3	36.526	KV452730.1	1476365	2-scf00040	2
AX-157712238	3	36.526	KV452730.1	1489969	2-scf00040	2
AX-157813425	3	36.526	KV452730.1	1491727	2-scf00040	2
AX-157923110	3	36.526	KV452730.1	1510513	2-scf00040	2
AX-157813429	3	36.526	KV452730.1	1511482	2-scf00040	2
AX-157813430	3	36.526	KV452730.1	1512372	2-scf00040	2
AX-157922966	3	36.549	KV452730.1	1322927	2-scf00040	2
AX-157923015	3	36.549	KV452730.1	1381450	2-scf00040	2
AX-157923081	3	36.549	KV452730.1	1455965	2-scf00040	2
AX-157720090	3	36.549	KV452730.1	1480159	2-scf00040	2
AX-157923101	3	36.549	KV452730.1	1496578	2-scf00040	2
AX-157923104	3	36.549	KV452730.1	1498848	2-scf00040	2
AX-157923105	3	36.549	KV452730.1	1505849	2-scf00040	2
AX-157923109	3	36.549	KV452730.1	1509032	2-scf00040	2
AX-157923113	3	36.634	KV452730.1	1513600	2-scf00040	2
AX-157923115	3	36.67	KV452730.1	1514221	2-scf00040	2
AX-157923117	3	36.713	KV452730.1	1518359	2-scf00040	2
AX-157923119	3	36.758	KV452730.1	1519201	2-scf00040	2
AX-157923272	3	36.758	KV452730.1	1697505	2-scf00040	2
AX-157923121	3	36.77	KV452730.1	1521390	2-scf00040	2
AX-157923130	3	36.77	KV452730.1	1529370	2-scf00040	2
AX-157923145	3	36.77	KV452730.1	1556246	2-scf00040	2
AX-157923133	3	36.787	KV452730.1	1540540	2-scf00040	2
AX-158389676	3	36.814	KV452730.1	1525178	2-scf00040	2
AX-157923131	3	36.814	KV452730.1	1529931	2-scf00040	2
AX-157923141	3	36.814	KV452730.1	1552350	2-scf00040	2
AX-157923148	3	36.814	KV452730.1	1559155	2-scf00040	2
AX-157869163	3	36.814	KV452730.1	1560124	2-scf00040	2
AX-157923159	3	36.814	KV452730.1	1570727	2-scf00040	2
AX-157923163	3	36.814	KV452730.1	1576829	2-scf00040	2
AX-157755060	3	36.814	KV452730.1	1584270	2-scf00040	2
AX-157869164	3	36.814	KV452730.1	1584897	2-scf00040	2
AX-157923177	3	36.814	KV452730.1	1589649	2-scf00040	2
AX-157923185	3	36.814	KV452730.1	1599166	2-scf00040	2
AX-157923174	3	36.846	KV452730.1	1586339	2-scf00040	2
AX-157923186	3	36.846	KV452730.1	1603603	2-scf00040	2
AX-157713601	3	36.877	KV452730.1	1599481	2-scf00040	2
AX-157755501	3	36.981	KV452730.1	1611350	2-scf00040	2
AX-157923199	3	37.095	KV452730.1	1611513	2-scf00040	2
AX-157923202	3	37.095	KV452730.1	1613138	2-scf00040	2
AX-157923203	3	37.095	KV452730.1	1614351	2-scf00040	2
AX-157869165	3	37.095	KV452730.1	1615239	2-scf00040	2
AX-157923206	3	37.095	KV452730.1	1615884	2-scf00040	2
AX-157923210	3	37.095	KV452730.1	1621943	2-scf00040	2

AX-157923277	3	37.095	KV452730.1	1701516	2-scf00040	2
AX-157923212	3	37.204	KV452730.1	1623071	2-scf00040	2
AX-157923214	3	37.204	KV452730.1	1624648	2-scf00040	2
AX-157923215	3	37.204	KV452730.1	1626185	2-scf00040	2
AX-157923217	3	37.204	KV452730.1	1627462	2-scf00040	2
AX-157923220	3	37.204	KV452730.1	1633754	2-scf00040	2
AX-157813434	3	37.204	KV452730.1	1637162	2-scf00040	2
AX-157869168	3	37.204	KV452730.1	1643664	2-scf00040	2
AX-157923219	3	37.423	KV452730.1	1633105	2-scf00040	2
AX-157923222	3	37.423	KV452730.1	1634803	2-scf00040	2
AX-157874790	3	37.423	KV452730.1	1635862	2-scf00040	2
AX-157923224	3	37.423	KV452730.1	1635995	2-scf00040	2
AX-157813435	3	37.423	KV452730.1	1637459	2-scf00040	2
AX-157923226	3	37.423	KV452730.1	1638617	2-scf00040	2
AX-157923230	3	37.423	KV452730.1	1640463	2-scf00040	2
AX-157923236	3	37.423	KV452730.1	1644987	2-scf00040	2
AX-157813436	3	37.423	KV452730.1	1647583	2-scf00040	2
AX-157813438	3	37.423	KV452730.1	1658938	2-scf00040	2
AX-157923239	3	37.423	KV452730.1	1660435	2-scf00040	2
AX-157923264	3	37.423	KV452730.1	1693215	2-scf00040	2
AX-157923335	3	37.423	KV452730.1	1746082	2-scf00040	2
AX-157923243	3	37.492	KV452730.1	1667067	2-scf00040	2
AX-157923246	3	37.532	KV452730.1	1671058	2-scf00040	2
AX-157923249	3	37.532	KV452730.1	1676354	2-scf00040	2
AX-157724443	3	37.532	KV452730.1	1679913	2-scf00040	2
AX-157923248	3	37.714	KV452730.1	1675631	2-scf00040	2
AX-157923253	3	37.714	KV452730.1	1681070	2-scf00040	2
AX-157923254	3	37.714	KV452730.1	1682870	2-scf00040	2
AX-157923256	3	37.714	KV452730.1	1683831	2-scf00040	2
AX-157883600	3	37.714	KV452730.1	1714563	2-scf00040	2
AX-157813440	3	37.716	KV452730.1	1688786	2-scf00040	2
AX-157874792	3	38.078	KV452730.1	1710846	2-scf00040	2
AX-157813446	3	38.078	KV452730.1	1715225	2-scf00040	2
AX-157717076	3	38.078	KV452730.1	1718004	2-scf00040	2
AX-157923298	3	38.078	KV452730.1	1718454	2-scf00040	2
AX-157923311	3	38.078	KV452730.1	1729053	2-scf00040	2
AX-157923313	3	38.078	KV452730.1	1731243	2-scf00040	2
AX-157923314	3	38.078	KV452730.1	1731706	2-scf00040	2
AX-157923316	3	38.078	KV452730.1	1732109	2-scf00040	2
AX-157923329	3	38.078	KV452730.1	1739645	2-scf00040	2
AX-157694581	3	38.217	KV452730.1	1735292	2-scf00040	2
AX-157923339	3	38.217	KV452730.1	1750751	2-scf00040	2
AX-157923395	3	38.217	KV452730.1	1807995	2-scf00040	2
AX-157853840	3	38.287	KV452730.1	1739752	2-scf00040	2
AX-157883601	3	38.91	KV452730.1	1752202	2-scf00040	2
AX-157923346	3	38.91	KV452730.1	1761172	2-scf00040	2
AX-157923341	3	38.918	KV452730.1	1753002	2-scf00040	2
AX-157923342	3	38.918	KV452730.1	1753927	2-scf00040	2
AX-157923345	3	38.918	KV452730.1	1760349	2-scf00040	2
AX-157923347	3	38.918	KV452730.1	1761692	2-scf00040	2
AX-157923344	3	39.225	KV452730.1	1755431	2-scf00040	2

AX-157751057	3	39.225	KV452730.1	1762716	2-scf00040	2
AX-157923350	3	39.225	KV452730.1	1763141	2-scf00040	2
AX-157813452	3	39.225	KV452730.1	1789450	2-scf00040	2
AX-157923381	3	39.225	KV452730.1	1801422	2-scf00040	2
AX-157813447	3	39.473	KV452730.1	1765062	2-scf00040	2
AX-157754538	3	39.473	KV452730.1	1789957	2-scf00040	2
AX-158382371	3	39.473	KV452730.1	1804353	2-scf00040	2
AX-157923355	3	39.487	KV452730.1	1768263	2-scf00040	2
AX-157923391	3	39.487	KV452730.1	1806245	2-scf00040	2
AX-157923356	3	39.502	KV452730.1	1769358	2-scf00040	2
AX-157813450	3	39.502	KV452730.1	1774070	2-scf00040	2
AX-157923359	3	39.502	KV452730.1	1774962	2-scf00040	2
AX-157813451	3	39.503	KV452730.1	1780516	2-scf00040	2
AX-157923363	3	39.666	KV452730.1	1788585	2-scf00040	2
AX-157813453	3	39.713	KV452730.1	1789904	2-scf00040	2
AX-157923366	3	39.83	KV452730.1	1792422	2-scf00040	2
AX-157744353	3	39.83	KV452730.1	1793633	2-scf00040	2
AX-158404448	3	39.83	KV452730.1	1794538	2-scf00040	2
AX-157923372	3	39.83	KV452730.1	1795420	2-scf00040	2
AX-157923375	3	39.83	KV452730.1	1797199	2-scf00040	2
AX-157923376	3	39.83	KV452730.1	1798346	2-scf00040	2
AX-157923377	3	39.83	KV452730.1	1799982	2-scf00040	2
AX-157923382	3	39.83	KV452730.1	1802608	2-scf00040	2
AX-157923387	3	39.83	KV452730.1	1805106	2-scf00040	2
AX-157923402	3	39.83	KV452730.1	1816934	2-scf00040	2
AX-157923405	3	39.83	KV452730.1	1817932	2-scf00040	2
AX-157923409	3	39.83	KV452730.1	1820332	2-scf00040	2
AX-157923412	3	39.83	KV452730.1	1825529	2-scf00040	2
AX-157923416	3	39.83	KV452730.1	1829126	2-scf00040	2
AX-157923418	3	39.86	KV452730.1	1830379	2-scf00040	2
AX-157923422	3	39.941	KV452730.1	1844216	2-scf00040	2
AX-157743029	3	39.941	KV452730.1	1845516	2-scf00040	2
AX-157813462	3	39.941	KV452730.1	1898505	2-scf00040	2
AX-157923479	3	39.941	KV452730.1	1924591	2-scf00040	2
AX-157874797	3	39.996	KV452730.1	1845976	2-scf00040	2
AX-157813456	3	40.049	KV452730.1	1846590	2-scf00040	2
AX-157813457	3	40.049	KV452730.1	1846849	2-scf00040	2
AX-157923424	3	40.109	KV452730.1	1847758	2-scf00040	2
AX-157923429	3	40.158	KV452730.1	1854762	2-scf00040	2
AX-157923432	3	40.158	KV452730.1	1858760	2-scf00040	2
AX-157923433	3	40.158	KV452730.1	1859201	2-scf00040	2
AX-157923436	3	40.158	KV452730.1	1863450	2-scf00040	2
AX-157739275	3	40.485	KV452730.1	1865256	2-scf00040	2
AX-157923437	3	40.486	KV452730.1	1867463	2-scf00040	2
AX-157923438	3	40.486	KV452730.1	1868426	2-scf00040	2
AX-157714398	3	40.486	KV452730.1	1947888	2-scf00040	2
AX-157923442	3	40.541	KV452730.1	1875519	2-scf00040	2
AX-157923443	3	40.596	KV452730.1	1876075	2-scf00040	2
AX-157923444	3	40.596	KV452730.1	1876507	2-scf00040	2
AX-157923446	3	40.596	KV452730.1	1877218	2-scf00040	2
AX-157923451	3	40.596	KV452730.1	1883441	2-scf00040	2

AX-157923457	3	40.596	KV452730.1	1896491	2-scf00040	2
AX-157923460	3	40.596	KV452730.1	1898130	2-scf00040	2
AX-157923482	3	40.596	KV452730.1	1930774	2-scf00040	2
AX-157923506	3	40.596	KV452730.1	1953607	2-scf00040	2
AX-157923547	3	40.596	KV452730.1	2037675	2-scf00040	2
AX-157748392	3	40.657	KV452730.1	1885240	2-scf00040	2
AX-157755565	3	40.704	KV452730.1	1889136	2-scf00040	2
AX-157923456	3	40.704	KV452730.1	1896144	2-scf00040	2
AX-157923459	3	40.704	KV452730.1	1897492	2-scf00040	2
AX-157923462	3	40.704	KV452730.1	1898987	2-scf00040	2
AX-157720949	3	40.704	KV452730.1	1900987	2-scf00040	2
AX-157923465	3	40.704	KV452730.1	1902795	2-scf00040	2
AX-157714442	3	40.704	KV452730.1	1903092	2-scf00040	2
AX-157923468	3	40.704	KV452730.1	1904381	2-scf00040	2
AX-157923484	3	40.704	KV452730.1	1932232	2-scf00040	2
AX-157923466	3	40.923	KV452730.1	1903232	2-scf00040	2
AX-157923469	3	40.923	KV452730.1	1904906	2-scf00040	2
AX-157883609	3	40.923	KV452730.1	1910437	2-scf00040	2
AX-157813466	3	40.923	KV452730.1	1912057	2-scf00040	2
AX-157923474	3	40.923	KV452730.1	1916255	2-scf00040	2
AX-157740951	3	40.923	KV452730.1	1921378	2-scf00040	2
AX-157813470	3	40.923	KV452730.1	1961388	2-scf00040	2
AX-157923529	3	40.923	KV452730.1	1984805	2-scf00040	2
AX-157864585	3	40.958	KV452730.1	1922562	2-scf00040	2
AX-157923487	3	41.005	KV452730.1	1934148	2-scf00040	2
AX-157923489	3	41.052	KV452730.1	1935936	2-scf00040	2
AX-157923512	3	41.052	KV452730.1	1960048	2-scf00040	2
AX-157813468	3	41.088	KV452730.1	1936066	2-scf00040	2
AX-157923490	3	41.088	KV452730.1	1936308	2-scf00040	2
AX-157741009	3	41.088	KV452730.1	1941831	2-scf00040	2
AX-157853841	3	41.248	KV452730.1	1942328	2-scf00040	2
AX-157813469	3	41.249	KV452730.1	1936438	2-scf00040	2
AX-157923499	3	41.249	KV452730.1	1945417	2-scf00040	2
AX-157853842	3	41.249	KV452730.1	1945694	2-scf00040	2
AX-157923500	3	41.249	KV452730.1	1945843	2-scf00040	2
AX-157923530	3	41.249	KV452730.1	1986045	2-scf00040	2
AX-157773668	3	41.249	KV452730.1	2013004	2-scf00040	2
AX-157923501	3	41.25	KV452730.1	1949984	2-scf00040	2
AX-157923504	3	41.251	KV452730.1	1951869	2-scf00040	2
AX-157923505	3	41.291	KV452730.1	1953118	2-scf00040	2
AX-157923511	3	41.333	KV452730.1	1958929	2-scf00040	2
AX-157923514	3	41.36	KV452730.1	1964548	2-scf00040	2
AX-157923515	3	41.36	KV452730.1	1964971	2-scf00040	2
AX-157923516	3	41.36	KV452730.1	1965197	2-scf00040	2
AX-157813473	3	41.36	KV452730.1	1966149	2-scf00040	2
AX-157745897	3	41.407	KV452730.1	1966805	2-scf00040	2
AX-157923454	3	41.469	KV452730.1	1890406	2-scf00040	2
AX-157773895	3	41.469	KV452730.1	1927285	2-scf00040	2
AX-157813474	3	41.469	KV452730.1	1969949	2-scf00040	2
AX-157923523	3	41.469	KV452730.1	1977586	2-scf00040	2
AX-157923525	3	41.469	KV452730.1	1978337	2-scf00040	2

AX-157754117	3	41.469	KV452730.1	1982286	2-scf00040	2
AX-157853844	3	41.469	KV452730.1	1982904	2-scf00040	2
AX-157745693	3	41.469	KV452730.1	1988026	2-scf00040	2
AX-157719154	3	41.469	KV452730.1	1993995	2-scf00040	2
AX-157813477	3	41.469	KV452730.1	1994144	2-scf00040	2
AX-157923532	3	41.469	KV452730.1	1994782	2-scf00040	2
AX-157923535	3	41.469	KV452730.1	1997465	2-scf00040	2
AX-157813478	3	41.469	KV452730.1	2000441	2-scf00040	2
AX-157813480	3	41.469	KV452730.1	2006927	2-scf00040	2
AX-157813481	3	41.469	KV452730.1	2008073	2-scf00040	2
AX-158405309	3	41.469	KV452730.1	2009221	2-scf00040	2
AX-157747886	3	41.469	KV452730.1	2024319	2-scf00040	2
AX-157813483	3	41.469	KV452730.1	2024855	2-scf00040	2
AX-157813485	3	41.469	KV452730.1	2044164	2-scf00040	2
AX-157813486	3	41.469	KV452730.1	2044508	2-scf00040	2
AX-157813490	3	41.469	KV452730.1	2046923	2-scf00040	2
AX-157813491	3	41.469	KV452730.1	2047218	2-scf00040	2
AX-157923548	3	41.469	KV452730.1	2047532	2-scf00040	2
AX-157923550	3	41.469	KV452730.1	2050292	2-scf00040	2
AX-157813492	3	41.469	KV452730.1	2051514	2-scf00040	2
AX-157923553	3	41.469	KV452730.1	2053925	2-scf00040	2
AX-157923557	3	41.469	KV452730.1	2055611	2-scf00040	2
AX-157923564	3	41.469	KV452730.1	2060016	2-scf00040	2
AX-157923528	3	41.687	KV452730.1	1983307	2-scf00040	2
AX-157883612	3	41.687	KV452730.1	1992832	2-scf00040	2
AX-157869172	3	41.687	KV452730.1	1995540	2-scf00040	2
AX-157719526	3	41.687	KV452730.1	2014760	2-scf00040	2
AX-157813482	3	41.687	KV452730.1	2019851	2-scf00040	2
AX-157813484	3	41.687	KV452730.1	2033978	2-scf00040	2
AX-157813487	3	41.687	KV452730.1	2045927	2-scf00040	2
AX-157923559	3	41.687	KV452730.1	2055870	2-scf00040	2
AX-157869174	3	41.687	KV452730.1	2056578	2-scf00040	2
AX-157923566	3	41.687	KV452730.1	2060292	2-scf00040	2
AX-157923570	3	41.687	KV452730.1	2065507	2-scf00040	2
AX-157923572	3	41.687	KV452730.1	2066000	2-scf00040	2
AX-157923574	3	41.687	KV452730.1	2068982	2-scf00040	2
AX-157923576	3	41.687	KV452730.1	2069531	2-scf00040	2
AX-157923577	3	41.687	KV452730.1	2069690	2-scf00040	2
AX-157923579	3	41.687	KV452730.1	2070385	2-scf00040	2
AX-157923583	3	41.687	KV452730.1	2073614	2-scf00040	2
AX-157923586	3	41.687	KV452730.1	2074784	2-scf00040	2
AX-157923587	3	41.687	KV452730.1	2075207	2-scf00040	2
AX-157923588	3	41.687	KV452730.1	2078534	2-scf00040	2
AX-157923589	3	41.687	KV452730.1	2079873	2-scf00040	2
AX-157923592	3	41.687	KV452730.1	2082466	2-scf00040	2
AX-157923596	3	41.687	KV452730.1	2086369	2-scf00040	2
AX-157923597	3	41.687	KV452730.1	2086599	2-scf00040	2
AX-157923608	3	41.824	KV452730.1	2095694	2-scf00040	2
AX-157923609	3	41.824	KV452730.1	2095821	2-scf00040	2
AX-157923610	3	41.824	KV452730.1	2099516	2-scf00040	2
AX-157923612	3	42.234	KV452730.1	2103965	2-scf00040	2

AX-157923614	3	42.234	KV452730.1	2107997	2-scf00040	2
AX-158382376	3	42.234	KV452730.1	2109302	2-scf00040	2
AX-157923615	3	42.234	KV452730.1	2109343	2-scf00040	2
AX-157923619	3	42.234	KV452730.1	2113432	2-scf00040	2
AX-157923622	3	42.234	KV452730.1	2114582	2-scf00040	2
AX-157923623	3	42.234	KV452730.1	2116257	2-scf00040	2
AX-157923626	3	42.234	KV452730.1	2118188	2-scf00040	2
AX-157923628	3	42.234	KV452730.1	2119317	2-scf00040	2
AX-157813495	3	42.277	KV452730.1	2125521	2-scf00040	2
AX-157923631	3	42.315	KV452730.1	2123179	2-scf00040	2
AX-157923632	3	42.315	KV452730.1	2125874	2-scf00040	2
AX-157813497	3	42.343	KV452730.1	2134124	2-scf00040	2
AX-157813498	3	42.343	KV452730.1	2136123	2-scf00040	2
AX-157923640	3	42.343	KV452730.1	2137534	2-scf00040	2
AX-157923641	3	42.343	KV452730.1	2138231	2-scf00040	2
AX-157813500	3	42.343	KV452730.1	2143815	2-scf00040	2
AX-157923654	3	42.343	KV452730.1	2165793	2-scf00040	2
AX-158379315	3	42.343	KV452730.1	2184898	2-scf00040	2
AX-157746906	3	42.343	KV452730.1	2222035	2-scf00040	2
AX-157923701	3	42.343	KV452730.1	2254386	2-scf00040	2
AX-157923762	3	42.343	KV452730.1	2313982	2-scf00040	2
AX-157745719	3	42.452	KV452730.1	2150586	2-scf00040	2
AX-157813502	3	42.562	KV452730.1	2157222	2-scf00040	2
AX-157923648	3	42.562	KV452730.1	2158204	2-scf00040	2
AX-157813503	3	42.562	KV452730.1	2158411	2-scf00040	2
AX-157923650	3	42.562	KV452730.1	2159011	2-scf00040	2
AX-157923651	3	42.671	KV452730.1	2161745	2-scf00040	2
AX-157747964	3	42.728	KV452730.1	2162198	2-scf00040	2
AX-157923656	3	42.728	KV452730.1	2170444	2-scf00040	2
AX-157923655	3	42.78	KV452730.1	2167147	2-scf00040	2
AX-157710068	3	42.78	KV452730.1	2169933	2-scf00040	2
AX-157923657	3	42.78	KV452730.1	2172217	2-scf00040	2
AX-157923661	3	42.78	KV452730.1	2174273	2-scf00040	2
AX-157923664	3	42.78	KV452730.1	2176821	2-scf00040	2
AX-157923666	3	42.78	KV452730.1	2177286	2-scf00040	2
AX-157813505	3	42.78	KV452730.1	2179105	2-scf00040	2
AX-157714600	3	42.78	KV452730.1	2179265	2-scf00040	2
AX-157813506	3	42.78	KV452730.1	2183948	2-scf00040	2
AX-158405315	3	42.78	KV452730.1	2242640	2-scf00040	2
AX-157765037	3	42.933	KV452730.1	2180482	2-scf00040	2
AX-157713940	3	43.064	KV452730.1	2191375	2-scf00040	2
AX-157813509	3	43.218	KV452730.1	2195881	2-scf00040	2
AX-157923677	3	43.218	KV452730.1	2197879	2-scf00040	2
AX-157923696	3	43.218	KV452730.1	2251603	2-scf00040	2
AX-157923700	3	43.218	KV452730.1	2253797	2-scf00040	2
AX-157923726	3	43.218	KV452730.1	2288160	2-scf00040	2
AX-157923764	3	43.218	KV452730.1	2314629	2-scf00040	2
AX-157923697	3	43.222	KV452730.1	2252634	2-scf00040	2
AX-157719297	3	43.226	KV452730.1	2264423	2-scf00040	2
AX-157813511	3	43.23	KV452730.1	2264978	2-scf00040	2
AX-157709173	3	43.23	KV452730.1	2284893	2-scf00040	2

AX-157923716	3	43.236	KV452730.1	2279658	2-scf00040	2
AX-157923719	3	43.241	KV452730.1	2282390	2-scf00040	2
AX-157923745	3	43.241	KV452730.1	2298425	2-scf00040	2
AX-157704710	3	43.241	3ML01027109	880	scf02712_1	UN
AX-157923720	3	43.243	KV452730.1	2283314	2-scf00040	2
AX-157923729	3	43.247	KV452730.1	2289782	2-scf00040	2
AX-157923730	3	43.251	KV452730.1	2290304	2-scf00040	2
AX-157923731	3	43.259	KV452730.1	2290790	2-scf00040	2
AX-157923755	3	43.259	KV452730.1	2309424	2-scf00040	2
AX-158405311	3	43.339	KV452730.1	2201717	2-scf00040	2
AX-157923710	3	43.339	KV452730.1	2274209	2-scf00040	2
AX-157883614	3	43.339	KV452730.1	2300851	2-scf00040	2
AX-157923748	3	43.339	KV452730.1	2301364	2-scf00040	2
AX-157923756	3	43.339	KV452730.1	2310659	2-scf00040	2
AX-157923771	3	43.548	KV452730.1	2317953	2-scf00040	2
AX-157923782	3	43.672	KV452730.1	2325541	2-scf00040	2
AX-157923791	3	43.672	KV452730.1	2328635	2-scf00040	2
AX-158389684	3	43.672	KV452730.1	2333106	2-scf00040	2
AX-157923799	3	43.672	KV452730.1	2334456	2-scf00040	2
AX-158382386	3	43.672	KV452730.1	2337401	2-scf00040	2
AX-157874803	3	43.672	KV452730.1	2374530	2-scf00040	2
AX-157923793	3	43.983	KV452730.1	2329676	2-scf00040	2
AX-157923795	3	43.983	KV452730.1	2331669	2-scf00040	2
AX-157923805	3	43.983	KV452730.1	2338086	2-scf00040	2
AX-157923806	3	43.983	KV452730.1	2339230	2-scf00040	2
AX-157923809	3	43.983	KV452730.1	2341480	2-scf00040	2
AX-157923813	3	43.983	KV452730.1	2345376	2-scf00040	2
AX-157923819	3	43.983	KV452730.1	2350083	2-scf00040	2
AX-157923821	3	43.983	KV452730.1	2351124	2-scf00040	2
AX-157923823	3	43.983	KV452730.1	2353098	2-scf00040	2
AX-157883616	3	43.983	KV452730.1	2357980	2-scf00040	2
AX-157923832	3	43.983	KV452730.1	2358488	2-scf00040	2
AX-157923837	3	43.983	KV452730.1	2360728	2-scf00040	2
AX-157853851	3	43.983	KV452730.1	2361100	2-scf00040	2
AX-157923838	3	43.983	KV452730.1	2362466	2-scf00040	2
AX-157923839	3	43.983	KV452730.1	2364384	2-scf00040	2
AX-157923841	3	43.983	KV452730.1	2365299	2-scf00040	2
AX-157923842	3	43.983	KV452730.1	2366402	2-scf00040	2
AX-157923851	3	43.983	KV452730.1	2371104	2-scf00040	2
AX-157923855	3	43.984	KV452730.1	2373448	2-scf00040	2
AX-157923859	3	44.04	KV452730.1	2379264	2-scf00040	2
AX-157923865	3	44.04	KV452730.1	2383228	2-scf00040	2
AX-157883618	3	44.085	KV452730.1	2380070	2-scf00040	2
AX-157923888	3	44.085	KV452730.1	2395391	2-scf00040	2
AX-157923870	3	44.114	KV452730.1	2385830	2-scf00040	2
AX-157923871	3	44.147	KV452730.1	2387437	2-scf00040	2
AX-157923868	3	44.311	KV452730.1	2385438	2-scf00040	2
AX-157923874	3	44.311	KV452730.1	2388474	2-scf00040	2
AX-157923876	3	44.311	KV452730.1	2389643	2-scf00040	2
AX-157923881	3	44.311	KV452730.1	2391576	2-scf00040	2
AX-157923884	3	44.311	KV452730.1	2393174	2-scf00040	2

AX-157923886	3	44.311	KV452730.1	2393896	2-scf00040	2
AX-157883619	3	44.311	KV452730.1	2394599	2-scf00040	2
AX-157923894	3	44.316	KV452730.1	2400925	2-scf00040	2
AX-157923825	3	44.498	KV452730.1	2353689	2-scf00040	2
AX-157923895	3	44.498	KV452730.1	2402087	2-scf00040	2
AX-157923879	3	44.747	KV452730.1	2391230	2-scf00040	2
AX-157756783	3	44.747	KV452730.1	2407711	2-scf00040	2
AX-157923948	3	44.747	KV452730.1	2451183	2-scf00040	2
AX-157924032	3	44.747	KV452730.1	2515221	2-scf00040	2
AX-157923906	3	44.75	KV452730.1	2413934	2-scf00040	2
AX-157923911	3	44.75	KV452730.1	2416914	2-scf00040	2
AX-157923913	3	44.75	KV452730.1	2418228	2-scf00040	2
AX-157923917	3	44.75	KV452730.1	2420767	2-scf00040	2
AX-157923900	3	44.751	KV452730.1	2410734	2-scf00040	2
AX-157923916	3	44.751	KV452730.1	2419259	2-scf00040	2
AX-157923935	3	44.982	KV452730.1	2433676	2-scf00040	2
AX-157923926	3	44.993	KV452730.1	2426474	2-scf00040	2
AX-157883621	3	44.993	KV452730.1	2434807	2-scf00040	2
AX-158379317	3	44.993	KV452730.1	2474710	2-scf00040	2
AX-157739809	3	44.993	KV452730.1	2526005	2-scf00040	2
AX-157923940	3	45.003	KV452730.1	2439322	2-scf00040	2
AX-157923941	3	45.016	KV452730.1	2439932	2-scf00040	2
AX-157923945	3	45.028	KV452730.1	2444612	2-scf00040	2
AX-157923955	3	45.036	KV452730.1	2457107	2-scf00040	2
AX-157923965	3	45.036	KV452730.1	2461824	2-scf00040	2
AX-157923961	3	45.055	KV452730.1	2457941	2-scf00040	2
AX-157923971	3	45.218	KV452730.1	2466755	2-scf00040	2
AX-158404466	3	45.218	KV452730.1	2466979	2-scf00040	2
AX-157923972	3	45.223	KV452730.1	2468161	2-scf00040	2
AX-157923977	3	45.341	KV452730.1	2471740	2-scf00040	2
AX-157923994	3	45.341	KV452730.1	2485572	2-scf00040	2
AX-157924000	3	45.341	KV452730.1	2489585	2-scf00040	2
AX-157923985	3	45.482	KV452730.1	2480902	2-scf00040	2
AX-157741266	3	45.482	KV452730.1	2490189	2-scf00040	2
AX-157923990	3	45.803	KV452730.1	2482030	2-scf00040	2
AX-157923995	3	46.099	KV452730.1	2487347	2-scf00040	2
AX-157924005	3	46.117	KV452730.1	2494491	2-scf00040	2
AX-157924011	3	46.167	KV452730.1	2497914	2-scf00040	2
AX-157924010	3	46.174	KV452730.1	2497263	2-scf00040	2
AX-157725781	3	46.174	KV452730.1	2511133	2-scf00040	2
AX-157924029	3	46.174	KV452730.1	2514027	2-scf00040	2
AX-157924039	3	46.174	KV452730.1	2520815	2-scf00040	2
AX-157924058	3	46.33	KV452730.1	2533741	2-scf00040	2
AX-157750369	3	46.33	KV452730.1	2534829	2-scf00040	2
AX-157924062	3	46.33	KV452730.1	2536714	2-scf00040	2
AX-157924096	3	46.789	KV452730.1	2579178	2-scf00040	2
AX-157924100	3	47.049	KV452730.1	2587362	2-scf00040	2
AX-157924104	3	47.049	KV452730.1	2588625	2-scf00040	2
AX-157924106	3	47.049	KV452730.1	2590461	2-scf00040	2
AX-157924107	3	47.049	KV452730.1	2593189	2-scf00040	2
AX-157924108	3	47.049	KV452730.1	2594454	2-scf00040	2

AX-157862678	3	47.049	KV452730.1	2605019	2-scf00040	2
AX-157775650	3	47.049	KV452730.1	2605349	2-scf00040	2
AX-157924125	3	47.049	KV452730.1	2612595	2-scf00040	2
AX-157924127	3	47.049	KV452730.1	2615778	2-scf00040	2
AX-157924128	3	47.049	KV452730.1	2616547	2-scf00040	2
AX-157924143	3	47.049	KV452730.1	2631937	2-scf00040	2
AX-157924151	3	47.049	KV452730.1	2637628	2-scf00040	2
AX-157924163	3	47.049	KV452730.1	2647820	2-scf00040	2
AX-158405323	3	47.049	KV452730.1	2648769	2-scf00040	2
AX-157924184	3	47.049	KV452730.1	2664911	2-scf00040	2
AX-157924244	3	47.049	KV452730.1	2726286	2-scf00040	2
AX-157924249	3	47.049	KV452730.1	2733923	2-scf00040	2
AX-157924130	3	47.115	KV452730.1	2617988	2-scf00040	2
AX-157718556	3	47.115	KV452730.1	2618504	2-scf00040	2
AX-157923920	3	47.159	KV452730.1	2422835	2-scf00040	2
AX-157923942	3	47.159	KV452730.1	2440259	2-scf00040	2
AX-157771485	3	47.159	KV452730.1	2492228	2-scf00040	2
AX-157924014	3	47.159	KV452730.1	2498930	2-scf00040	2
AX-157924026	3	47.159	KV452730.1	2508130	2-scf00040	2
AX-157924034	3	47.159	KV452730.1	2518102	2-scf00040	2
AX-157924043	3	47.159	KV452730.1	2521941	2-scf00040	2
AX-157924047	3	47.159	KV452730.1	2523906	2-scf00040	2
AX-157924111	3	47.159	KV452730.1	2595683	2-scf00040	2
AX-157924118	3	47.159	KV452730.1	2607731	2-scf00040	2
AX-157924133	3	47.159	KV452730.1	2627637	2-scf00040	2
AX-157924141	3	47.159	KV452730.1	2630836	2-scf00040	2
AX-157924147	3	47.159	KV452730.1	2633595	2-scf00040	2
AX-157924149	3	47.159	KV452730.1	2635342	2-scf00040	2
AX-157924150	3	47.159	KV452730.1	2636147	2-scf00040	2
AX-157924153	3	47.159	KV452730.1	2639170	2-scf00040	2
AX-157924158	3	47.159	KV452730.1	2642578	2-scf00040	2
AX-157924160	3	47.159	KV452730.1	2645722	2-scf00040	2
AX-157924166	3	47.159	KV452730.1	2648948	2-scf00040	2
AX-157924168	3	47.159	KV452730.1	2650056	2-scf00040	2
AX-157924169	3	47.159	KV452730.1	2650325	2-scf00040	2
AX-157721142	3	47.159	KV452730.1	2650895	2-scf00040	2
AX-157924172	3	47.159	KV452730.1	2651650	2-scf00040	2
AX-157813520	3	47.159	KV452730.1	2653668	2-scf00040	2
AX-157924175	3	47.159	KV452730.1	2654059	2-scf00040	2
AX-157924177	3	47.159	KV452730.1	2657945	2-scf00040	2
AX-157924180	3	47.159	KV452730.1	2660417	2-scf00040	2
AX-158379318	3	47.159	KV452730.1	2662571	2-scf00040	2
AX-157924183	3	47.159	KV452730.1	2664054	2-scf00040	2
AX-157924187	3	47.159	KV452730.1	2669447	2-scf00040	2
AX-157813521	3	47.159	KV452730.1	2671161	2-scf00040	2
AX-157924194	3	47.159	KV452730.1	2674002	2-scf00040	2
AX-157924202	3	47.159	KV452730.1	2680904	2-scf00040	2
AX-157924191	3	47.257	KV452730.1	2671683	2-scf00040	2
AX-157924197	3	47.341	KV452730.1	2675673	2-scf00040	2
AX-157924212	3	47.705	KV452730.1	2688204	2-scf00040	2
AX-157924215	3	47.705	KV452730.1	2689358	2-scf00040	2

AX-157719289	3	47.705	KV452730.1	2722337	2-scf00040	2
AX-157853855	3	47.813	KV452730.1	2703906	2-scf00040	2
AX-157924228	3	47.813	KV452730.1	2705111	2-scf00040	2
AX-157924232	3	47.813	KV452730.1	2710224	2-scf00040	2
AX-157924234	3	47.867	KV452730.1	2712519	2-scf00040	2
AX-157924207	3	47.924	KV452730.1	2685545	2-scf00040	2
AX-157924220	3	47.924	KV452730.1	2691707	2-scf00040	2
AX-157924230	3	47.924	KV452730.1	2708089	2-scf00040	2
AX-157924236	3	47.924	KV452730.1	2714263	2-scf00040	2
AX-157924237	3	47.924	KV452730.1	2715437	2-scf00040	2
AX-157924238	3	47.924	KV452730.1	2715974	2-scf00040	2
AX-157924245	3	47.924	KV452730.1	2727137	2-scf00040	2
AX-157924246	3	47.924	KV452730.1	2728457	2-scf00040	2
AX-157772855	3	47.924	KV452730.1	2731922	2-scf00040	2
AX-157924257	3	47.924	KV452730.1	2738925	2-scf00040	2
AX-157924262	3	47.924	KV452730.1	2740595	2-scf00040	2
AX-157754038	3	47.924	KV452730.1	2741346	2-scf00040	2
AX-157924263	3	47.924	KV452730.1	2742041	2-scf00040	2
AX-157924269	3	48.142	KV452730.1	2748392	2-scf00040	2
AX-157924271	3	48.142	KV452730.1	2749687	2-scf00040	2
AX-158414927	3	48.142	KV452730.1	2751782	2-scf00040	2
AX-157924296	3	48.142	KV452730.1	2767664	2-scf00040	2
AX-157924301	3	48.142	KV452730.1	2771206	2-scf00040	2
AX-157924319	3	48.142	KV452730.1	2781726	2-scf00040	2
AX-157924324	3	48.142	KV452730.1	2783636	2-scf00040	2
AX-157924404	3	48.142	KV452730.1	2845223	2-scf00040	2
AX-157924426	3	48.142	KV452730.1	2870040	2-scf00040	2
AX-157924450	3	48.142	KV452730.1	2900550	2-scf00040	2
AX-157924455	3	48.142	KV452730.1	2904777	2-scf00040	2
AX-157924456	3	48.142	KV452730.1	2906308	2-scf00040	2
AX-157924484	3	48.142	KV452730.1	2935901	2-scf00040	2
AX-157924495	3	48.142	KV452730.1	2944864	2-scf00040	2
AX-157924526	3	48.142	KV452730.1	2966871	2-scf00040	2
AX-157924549	3	48.142	KV452730.1	2992851	2-scf00040	2
AX-157924560	3	48.142	KV452730.1	3018961	2-scf00040	2
AX-158382397	3	48.142	KV452730.1	3024541	2-scf00040	2
AX-157753485	3	48.142	KV452730.1	3080384	2-scf00040	2
AX-157924625	3	48.142	KV452730.1	3082772	2-scf00040	2
AX-157924665	3	48.142	KV452730.1	3115593	2-scf00040	2
AX-157924679	3	48.142	KV452730.1	3125283	2-scf00040	2
AX-157924703	3	48.142	KV452730.1	3136588	2-scf00040	2
AX-157924791	3	48.142	KV452730.1	3200282	2-scf00040	2
AX-157924807	3	48.142	KV452730.1	3219827	2-scf00040	2
AX-157924889	3	48.142	KV452730.1	3298459	2-scf00040	2
AX-157924930	3	48.142	KV452730.1	3343376	2-scf00040	2
AX-157924954	3	48.142	KV452730.1	3374503	2-scf00040	2
AX-157924961	3	48.142	KV452730.1	3379032	2-scf00040	2
AX-157925262	3	48.142	KV452730.1	3730405	2-scf00040	2
AX-157924275	3	48.215	KV452730.1	2753440	2-scf00040	2
AX-157924282	3	48.252	KV452730.1	2758182	2-scf00040	2
AX-157924295	3	48.29	KV452730.1	2766983	2-scf00040	2

AX-157924298	3	48.361	KV452730.1	2769480	2-scf00040	2
AX-157924303	3	48.47	KV452730.1	2771614	2-scf00040	2
AX-157924304	3	48.47	KV452730.1	2772098	2-scf00040	2
AX-157747932	3	48.47	KV452730.1	2778189	2-scf00040	2
AX-157924311	3	48.47	KV452730.1	2778608	2-scf00040	2
AX-157924313	3	48.47	KV452730.1	2779128	2-scf00040	2
AX-157924315	3	48.47	KV452730.1	2779713	2-scf00040	2
AX-157924316	3	48.47	KV452730.1	2780203	2-scf00040	2
AX-157924318	3	48.47	KV452730.1	2781007	2-scf00040	2
AX-157924323	3	48.47	KV452730.1	2782972	2-scf00040	2
AX-157924327	3	48.47	KV452730.1	2787190	2-scf00040	2
AX-157924328	3	48.47	KV452730.1	2789921	2-scf00040	2
AX-157924333	3	48.47	KV452730.1	2793326	2-scf00040	2
AX-157924337	3	48.47	KV452730.1	2794766	2-scf00040	2
AX-157924342	3	48.47	KV452730.1	2796680	2-scf00040	2
AX-157924343	3	48.47	KV452730.1	2797375	2-scf00040	2
AX-157924344	3	48.47	KV452730.1	2798087	2-scf00040	2
AX-158414928	3	48.47	KV452730.1	2800119	2-scf00040	2
AX-157924362	3	48.47	KV452730.1	2816731	2-scf00040	2
AX-157924366	3	48.47	KV452730.1	2818385	2-scf00040	2
AX-157924374	3	48.47	KV452730.1	2824344	2-scf00040	2
AX-157924398	3	48.47	KV452730.1	2838658	2-scf00040	2
AX-157853857	3	48.47	KV452730.1	2882196	2-scf00040	2
AX-157924542	3	48.47	KV452730.1	2988990	2-scf00040	2
AX-157924907	3	48.47	KV452730.1	3316443	2-scf00040	2
AX-157924912	3	48.47	KV452730.1	3321755	2-scf00040	2
AX-157924340	3	48.688	KV452730.1	2795843	2-scf00040	2
AX-157924364	3	48.688	KV452730.1	2817378	2-scf00040	2
AX-157924370	3	48.688	KV452730.1	2820199	2-scf00040	2
AX-157924371	3	48.688	KV452730.1	2822554	2-scf00040	2
AX-157924377	3	48.688	KV452730.1	2827012	2-scf00040	2
AX-158389687	3	48.688	KV452730.1	2828987	2-scf00040	2
AX-157924380	3	48.688	KV452730.1	2830774	2-scf00040	2
AX-157924387	3	48.688	KV452730.1	2832754	2-scf00040	2
AX-157924390	3	48.688	KV452730.1	2834672	2-scf00040	2
AX-158405330	3	48.688	KV452730.1	2835276	2-scf00040	2
AX-157924393	3	48.688	KV452730.1	2837116	2-scf00040	2
AX-157924394	3	48.688	KV452730.1	2837630	2-scf00040	2
AX-157924396	3	48.688	KV452730.1	2837949	2-scf00040	2
AX-157924402	3	48.688	KV452730.1	2842864	2-scf00040	2
AX-157924425	3	48.688	KV452730.1	2869300	2-scf00040	2
AX-157924789	3	48.688	KV452730.1	3199261	2-scf00040	2
AX-157924227	3	48.893	KV452730.1	2699239	2-scf00040	2
AX-157924240	3	48.893	KV452730.1	2717417	2-scf00040	2
AX-157924388	3	48.893	KV452730.1	2833977	2-scf00040	2
AX-157924397	3	48.893	KV452730.1	2838446	2-scf00040	2
AX-157924416	3	48.893	KV452730.1	2861537	2-scf00040	2
AX-157924418	3	48.893	KV452730.1	2862701	2-scf00040	2
AX-157924422	3	48.893	KV452730.1	2867180	2-scf00040	2
AX-157924423	3	48.893	KV452730.1	2867650	2-scf00040	2
AX-157924431	3	48.893	KV452730.1	2875618	2-scf00040	2

AX-157924436	3	48.893	KV452730.1	2882848	2-scf00040	2
AX-157924467	3	48.893	KV452730.1	2917892	2-scf00040	2
AX-157924468	3	48.893	KV452730.1	2919339	2-scf00040	2
AX-157924517	3	48.893	KV452730.1	2960402	2-scf00040	2
AX-157924557	3	48.893	KV452730.1	3018509	2-scf00040	2
AX-157864590	3	48.893	KV452730.1	3052595	2-scf00040	2
AX-157924634	3	48.893	KV452730.1	3090570	2-scf00040	2
AX-157924861	3	48.893	KV452730.1	3274547	2-scf00040	2
AX-157813564	3	48.893	KV452730.1	3361309	2-scf00040	2
AX-157813565	3	48.893	KV452730.1	3363823	2-scf00040	2
AX-157924998	3	48.893	KV452730.1	3412518	2-scf00040	2
AX-157925007	3	48.893	KV452730.1	3420680	2-scf00040	2
AX-157925287	3	48.893	KV452730.1	3762163	2-scf00040	2
AX-157745428	3	48.893	KV452730.1	3764070	2-scf00040	2
AX-157925291	3	48.893	KV452730.1	3771096	2-scf00040	2
AX-157925339	3	48.893	KV452730.1	3825728	2-scf00040	2
AX-157925343	3	48.893	KV452730.1	3832579	2-scf00040	2
AX-157924430	3	48.921	KV452730.1	2875323	2-scf00040	2
AX-157924216	3	49.125	KV452730.1	2690260	2-scf00040	2
AX-157772068	3	49.125	KV452730.1	2884485	2-scf00040	2
AX-157924444	3	49.125	KV452730.1	2895242	2-scf00040	2
AX-157924445	3	49.125	KV452730.1	2895906	2-scf00040	2
AX-157924448	3	49.125	KV452730.1	2897735	2-scf00040	2
AX-157924461	3	49.125	KV452730.1	2909656	2-scf00040	2
AX-157741712	3	49.125	KV452730.1	2918959	2-scf00040	2
AX-157924473	3	49.125	KV452730.1	2924390	2-scf00040	2
AX-157813529	3	49.125	KV452730.1	2926799	2-scf00040	2
AX-157924480	3	49.125	KV452730.1	2930586	2-scf00040	2
AX-157924489	3	49.125	KV452730.1	2939663	2-scf00040	2
AX-157924493	3	49.125	KV452730.1	2941079	2-scf00040	2
AX-157924496	3	49.125	KV452730.1	2945240	2-scf00040	2
AX-157924501	3	49.125	KV452730.1	2947893	2-scf00040	2
AX-157924502	3	49.125	KV452730.1	2948672	2-scf00040	2
AX-157924505	3	49.125	KV452730.1	2950636	2-scf00040	2
AX-157924507	3	49.125	KV452730.1	2952546	2-scf00040	2
AX-157924515	3	49.125	KV452730.1	2959594	2-scf00040	2
AX-157924520	3	49.125	KV452730.1	2962550	2-scf00040	2
AX-157924525	3	49.125	KV452730.1	2965640	2-scf00040	2
AX-157924527	3	49.125	KV452730.1	2968351	2-scf00040	2
AX-157813531	3	49.125	KV452730.1	2973612	2-scf00040	2
AX-157864589	3	49.125	KV452730.1	2981545	2-scf00040	2
AX-157924539	3	49.125	KV452730.1	2986647	2-scf00040	2
AX-157924547	3	49.125	KV452730.1	2990695	2-scf00040	2
AX-157743595	3	49.125	KV452730.1	2995369	2-scf00040	2
AX-157813534	3	49.125	KV452730.1	3006553	2-scf00040	2
AX-157924563	3	49.125	KV452730.1	3023649	2-scf00040	2
AX-157924565	3	49.125	KV452730.1	3025009	2-scf00040	2
AX-157924568	3	49.125	KV452730.1	3025361	2-scf00040	2
AX-157924571	3	49.125	KV452730.1	3026553	2-scf00040	2
AX-157753492	3	49.125	KV452730.1	3029313	2-scf00040	2
AX-157924613	3	49.125	KV452730.1	3076983	2-scf00040	2

AX-157924616	3	49.125	KV452730.1	3077819	2-scf00040	2
AX-157924686	3	49.125	KV452730.1	3129188	2-scf00040	2
AX-157924699	3	49.125	KV452730.1	3134011	2-scf00040	2
AX-157924709	3	49.125	KV452730.1	3139905	2-scf00040	2
AX-157924726	3	49.125	KV452730.1	3147025	2-scf00040	2
AX-157924727	3	49.125	KV452730.1	3147542	2-scf00040	2
AX-157924749	3	49.125	KV452730.1	3163132	2-scf00040	2
AX-157924754	3	49.125	KV452730.1	3166904	2-scf00040	2
AX-157924758	3	49.125	KV452730.1	3170742	2-scf00040	2
AX-157924770	3	49.125	KV452730.1	3180257	2-scf00040	2
AX-157924910	3	49.125	KV452730.1	3319542	2-scf00040	2
AX-157813554	3	49.125	KV452730.1	3339644	2-scf00040	2
AX-157924946	3	49.125	KV452730.1	3358935	2-scf00040	2
AX-157924972	3	49.125	KV452730.1	3399862	2-scf00040	2
AX-157925049	3	49.125	KV452730.1	3469565	2-scf00040	2
AX-157925121	3	49.125	KV452730.1	3554153	2-scf00040	2
AX-157925145	3	49.125	KV452730.1	3586100	2-scf00040	2
AX-157813594	3	49.125	KV452730.1	3679611	2-scf00040	2
AX-157925274	3	49.125	KV452730.1	3747207	2-scf00040	2
AX-157925278	3	49.125	KV452730.1	3752893	2-scf00040	2
AX-157925295	3	49.125	KV452730.1	3772239	2-scf00040	2
AX-157706369	3	49.125	3ML01032142	299	scf07755_1	UN
AX-157924561	3	49.344	KV452730.1	3022298	2-scf00040	2
AX-157924572	3	49.344	KV452730.1	3027077	2-scf00040	2
AX-157924580	3	49.344	KV452730.1	3037050	2-scf00040	2
AX-157924582	3	49.344	KV452730.1	3037926	2-scf00040	2
AX-157924588	3	49.344	KV452730.1	3047782	2-scf00040	2
AX-157924591	3	49.344	KV452730.1	3048333	2-scf00040	2
AX-157924594	3	49.344	KV452730.1	3048954	2-scf00040	2
AX-157924598	3	49.344	KV452730.1	3057186	2-scf00040	2
AX-157924604	3	49.344	KV452730.1	3067830	2-scf00040	2
AX-157924607	3	49.344	KV452730.1	3068970	2-scf00040	2
AX-157924609	3	49.344	KV452730.1	3069465	2-scf00040	2
AX-157924615	3	49.344	KV452730.1	3077408	2-scf00040	2
AX-157924637	3	49.344	KV452730.1	3094916	2-scf00040	2
AX-157924650	3	49.344	KV452730.1	3103754	2-scf00040	2
AX-157813538	3	49.344	KV452730.1	3107639	2-scf00040	2
AX-157924658	3	49.344	KV452730.1	3111263	2-scf00040	2
AX-157924669	3	49.344	KV452730.1	3117850	2-scf00040	2
AX-157924767	3	49.344	KV452730.1	3178658	2-scf00040	2
AX-157746616	3	49.344	KV452730.1	3178873	2-scf00040	2
AX-157924813	3	49.344	KV452730.1	3224420	2-scf00040	2
AX-157924834	3	49.344	KV452730.1	3251849	2-scf00040	2
AX-157924836	3	49.344	KV452730.1	3253707	2-scf00040	2
AX-157813547	3	49.344	KV452730.1	3258832	2-scf00040	2
AX-157924855	3	49.344	KV452730.1	3268350	2-scf00040	2
AX-157924884	3	49.344	KV452730.1	3294250	2-scf00040	2
AX-157925172	3	49.344	KV452730.1	3634763	2-scf00040	2
AX-157924642	3	49.432	KV452730.1	3096361	2-scf00040	2
AX-157924993	3	49.432	KV452730.1	3409645	2-scf00040	2
AX-158379323	3	49.432	KV452730.1	3514270	2-scf00040	2

AX-157924644	3	49.495	KV452730.1	3098581	2-scf00040	2
AX-157853858	3	49.562	KV452730.1	2905034	2-scf00040	2
AX-157924466	3	49.562	KV452730.1	2916724	2-scf00040	2
AX-157924541	3	49.562	KV452730.1	2988009	2-scf00040	2
AX-157924575	3	49.562	KV452730.1	3030688	2-scf00040	2
AX-157853859	3	49.562	KV452730.1	3033240	2-scf00040	2
AX-157924576	3	49.562	KV452730.1	3035235	2-scf00040	2
AX-157924584	3	49.562	KV452730.1	3041899	2-scf00040	2
AX-157924629	3	49.562	KV452730.1	3085645	2-scf00040	2
AX-157727673	3	49.562	KV452730.1	3102441	2-scf00040	2
AX-157924654	3	49.562	KV452730.1	3109306	2-scf00040	2
AX-157924660	3	49.562	KV452730.1	3112428	2-scf00040	2
AX-157924667	3	49.562	KV452730.1	3117223	2-scf00040	2
AX-157924670	3	49.562	KV452730.1	3119547	2-scf00040	2
AX-157853860	3	49.562	KV452730.1	3120095	2-scf00040	2
AX-157853861	3	49.562	KV452730.1	3122116	2-scf00040	2
AX-157874817	3	49.562	KV452730.1	3123149	2-scf00040	2
AX-157924677	3	49.562	KV452730.1	3123379	2-scf00040	2
AX-157924681	3	49.562	KV452730.1	3125732	2-scf00040	2
AX-157756502	3	49.562	KV452730.1	3128436	2-scf00040	2
AX-157924688	3	49.562	KV452730.1	3130083	2-scf00040	2
AX-157924689	3	49.562	KV452730.1	3130478	2-scf00040	2
AX-157924692	3	49.562	KV452730.1	3131069	2-scf00040	2
AX-157924696	3	49.562	KV452730.1	3132382	2-scf00040	2
AX-157924697	3	49.562	KV452730.1	3132776	2-scf00040	2
AX-157924701	3	49.562	KV452730.1	3135183	2-scf00040	2
AX-157924702	3	49.562	KV452730.1	3135972	2-scf00040	2
AX-157924705	3	49.562	KV452730.1	3137100	2-scf00040	2
AX-157924706	3	49.562	KV452730.1	3137649	2-scf00040	2
AX-157924707	3	49.562	KV452730.1	3138259	2-scf00040	2
AX-157924708	3	49.562	KV452730.1	3139402	2-scf00040	2
AX-157723285	3	49.562	KV452730.1	3140276	2-scf00040	2
AX-157853862	3	49.562	KV452730.1	3140962	2-scf00040	2
AX-157724788	3	49.562	KV452730.1	3141742	2-scf00040	2
AX-157924719	3	49.562	KV452730.1	3143733	2-scf00040	2
AX-157924729	3	49.562	KV452730.1	3149163	2-scf00040	2
AX-157924731	3	49.562	KV452730.1	3149922	2-scf00040	2
AX-157727054	3	49.562	KV452730.1	3150601	2-scf00040	2
AX-157924736	3	49.562	KV452730.1	3154909	2-scf00040	2
AX-157924737	3	49.562	KV452730.1	3155416	2-scf00040	2
AX-157924741	3	49.562	KV452730.1	3157763	2-scf00040	2
AX-157924757	3	49.562	KV452730.1	3168182	2-scf00040	2
AX-157746291	3	49.562	KV452730.1	3170875	2-scf00040	2
AX-157924760	3	49.562	KV452730.1	3171822	2-scf00040	2
AX-157924768	3	49.562	KV452730.1	3180099	2-scf00040	2
AX-157924771	3	49.562	KV452730.1	3181545	2-scf00040	2
AX-157924773	3	49.562	KV452730.1	3182093	2-scf00040	2
AX-157813541	3	49.562	KV452730.1	3183442	2-scf00040	2
AX-157924778	3	49.562	KV452730.1	3185642	2-scf00040	2
AX-157924787	3	49.562	KV452730.1	3196030	2-scf00040	2
AX-157924788	3	49.562	KV452730.1	3197072	2-scf00040	2

AX-157924793	3	49.562	KV452730.1	3201656	2-scf00040	2
AX-157924794	3	49.562	KV452730.1	3202918	2-scf00040	2
AX-157924795	3	49.562	KV452730.1	3204250	2-scf00040	2
AX-157717176	3	49.562	KV452730.1	3204335	2-scf00040	2
AX-157924798	3	49.562	KV452730.1	3207039	2-scf00040	2
AX-157924803	3	49.562	KV452730.1	3216131	2-scf00040	2
AX-157774164	3	49.562	KV452730.1	3223797	2-scf00040	2
AX-157924816	3	49.562	KV452730.1	3224921	2-scf00040	2
AX-157924818	3	49.562	KV452730.1	3226835	2-scf00040	2
AX-157743435	3	49.562	KV452730.1	3229680	2-scf00040	2
AX-157813545	3	49.562	KV452730.1	3231892	2-scf00040	2
AX-157924823	3	49.562	KV452730.1	3232416	2-scf00040	2
AX-157924826	3	49.562	KV452730.1	3236989	2-scf00040	2
AX-157924828	3	49.562	KV452730.1	3242769	2-scf00040	2
AX-157924837	3	49.562	KV452730.1	3256534	2-scf00040	2
AX-157924848	3	49.562	KV452730.1	3263295	2-scf00040	2
AX-157924853	3	49.562	KV452730.1	3266544	2-scf00040	2
AX-157723530	3	49.562	KV452730.1	3280601	2-scf00040	2
AX-157924866	3	49.562	KV452730.1	3280768	2-scf00040	2
AX-157924878	3	49.562	KV452730.1	3285474	2-scf00040	2
AX-157924882	3	49.562	KV452730.1	3289200	2-scf00040	2
AX-157771770	3	49.562	KV452730.1	3297835	2-scf00040	2
AX-157924890	3	49.562	KV452730.1	3299068	2-scf00040	2
AX-157924902	3	49.562	KV452730.1	3312113	2-scf00040	2
AX-157924918	3	49.562	KV452730.1	3325921	2-scf00040	2
AX-157924923	3	49.562	KV452730.1	3329605	2-scf00040	2
AX-157813553	3	49.562	KV452730.1	3329944	2-scf00040	2
AX-157813563	3	49.562	KV452730.1	3349339	2-scf00040	2
AX-157924957	3	49.562	KV452730.1	3377224	2-scf00040	2
AX-157925004	3	49.562	KV452730.1	3414671	2-scf00040	2
AX-157925060	3	49.562	KV452730.1	3487273	2-scf00040	2
AX-157813592	3	49.562	KV452730.1	3544878	2-scf00040	2
AX-157925128	3	49.562	KV452730.1	3566369	2-scf00040	2
AX-157925192	3	49.562	KV452730.1	3670940	2-scf00040	2
AX-157925249	3	49.562	KV452730.1	3718906	2-scf00040	2
AX-157704209	3	49.612	KV452730.1	3320347	2-scf00040	2
AX-157924470	3	49.672	KV452730.1	2922356	2-scf00040	2
AX-157719340	3	49.672	KV452730.1	2951205	2-scf00040	2
AX-157924514	3	49.672	KV452730.1	2954805	2-scf00040	2
AX-157924645	3	49.672	KV452730.1	3100312	2-scf00040	2
AX-157924721	3	49.672	KV452730.1	3144352	2-scf00040	2
AX-157924724	3	49.672	KV452730.1	3145876	2-scf00040	2
AX-157924746	3	49.672	KV452730.1	3160858	2-scf00040	2
AX-157924796	3	49.672	KV452730.1	3204681	2-scf00040	2
AX-157924870	3	49.672	KV452730.1	3282235	2-scf00040	2
AX-157924895	3	49.672	KV452730.1	3305913	2-scf00040	2
AX-157924897	3	49.672	KV452730.1	3308586	2-scf00040	2
AX-157813555	3	49.672	KV452730.1	3340057	2-scf00040	2
AX-157813556	3	49.672	KV452730.1	3342413	2-scf00040	2
AX-157813558	3	49.672	KV452730.1	3343551	2-scf00040	2
AX-157813560	3	49.672	KV452730.1	3345446	2-scf00040	2

AX-157717940	3	49.672	KV452730.1	3345543	2-scf00040	2
AX-157813561	3	49.672	KV452730.1	3345932	2-scf00040	2
AX-157813562	3	49.672	KV452730.1	3346414	2-scf00040	2
AX-157924933	3	49.672	KV452730.1	3349115	2-scf00040	2
AX-157924935	3	49.672	KV452730.1	3351462	2-scf00040	2
AX-157924936	3	49.672	KV452730.1	3353014	2-scf00040	2
AX-157924937	3	49.672	KV452730.1	3353806	2-scf00040	2
AX-157924953	3	49.672	KV452730.1	3373668	2-scf00040	2
AX-157924963	3	49.672	KV452730.1	3381186	2-scf00040	2
AX-157708032	3	49.672	KV452730.1	3381750	2-scf00040	2
AX-157924966	3	49.672	KV452730.1	3383037	2-scf00040	2
AX-157924967	3	49.672	KV452730.1	3384864	2-scf00040	2
AX-157925006	3	49.672	KV452730.1	3417609	2-scf00040	2
AX-157925023	3	49.672	KV452730.1	3443346	2-scf00040	2
AX-157813575	3	49.672	KV452730.1	3444624	2-scf00040	2
AX-157925031	3	49.672	KV452730.1	3448499	2-scf00040	2
AX-157925194	3	49.672	KV452730.1	3673592	2-scf00040	2
AX-157925230	3	49.672	KV452730.1	3698477	2-scf00040	2
AX-157925233	3	49.672	KV452730.1	3705233	2-scf00040	2
AX-157925255	3	49.672	KV452730.1	3725722	2-scf00040	2
AX-157874824	3	49.672	KV452730.1	3756718	2-scf00040	2
AX-157813604	3	49.672	KV452730.1	3769722	2-scf00040	2
AX-157925309	3	49.672	KV452730.1	3784194	2-scf00040	2
AX-157770774	3	49.781	KV452730.1	3108936	2-scf00040	2
AX-157883628	3	49.781	KV452730.1	3157103	2-scf00040	2
AX-157924952	3	49.781	KV452730.1	3371235	2-scf00040	2
AX-157924975	3	49.781	KV452730.1	3400857	2-scf00040	2
AX-157924977	3	49.781	KV452730.1	3402254	2-scf00040	2
AX-157924978	3	49.781	KV452730.1	3402660	2-scf00040	2
AX-157924982	3	49.781	KV452730.1	3403571	2-scf00040	2
AX-157924988	3	49.781	KV452730.1	3407430	2-scf00040	2
AX-157924994	3	49.781	KV452730.1	3410181	2-scf00040	2
AX-157924995	3	49.781	KV452730.1	3411335	2-scf00040	2
AX-157813569	3	49.781	KV452730.1	3415098	2-scf00040	2
AX-157925009	3	49.781	KV452730.1	3422400	2-scf00040	2
AX-157925011	3	49.781	KV452730.1	3423289	2-scf00040	2
AX-157813570	3	49.781	KV452730.1	3425286	2-scf00040	2
AX-157925014	3	49.781	KV452730.1	3427496	2-scf00040	2
AX-157925015	3	49.781	KV452730.1	3428081	2-scf00040	2
AX-157925017	3	49.781	KV452730.1	3429522	2-scf00040	2
AX-157925021	3	49.781	KV452730.1	3441422	2-scf00040	2
AX-157925028	3	49.781	KV452730.1	3445203	2-scf00040	2
AX-157925030	3	49.781	KV452730.1	3446852	2-scf00040	2
AX-157813577	3	49.781	KV452730.1	3462942	2-scf00040	2
AX-157741653	3	49.781	KV452730.1	3465191	2-scf00040	2
AX-157925040	3	49.781	KV452730.1	3465759	2-scf00040	2
AX-157925042	3	49.781	KV452730.1	3466585	2-scf00040	2
AX-157813579	3	49.781	KV452730.1	3470319	2-scf00040	2
AX-157925050	3	49.781	KV452730.1	3471296	2-scf00040	2
AX-157813581	3	49.781	KV452730.1	3479070	2-scf00040	2
AX-157925057	3	49.781	KV452730.1	3480789	2-scf00040	2

AX-157813584	3	49.781	KV452730.1	3487386	2-scf00040	2
AX-157813585	3	49.781	KV452730.1	3498127	2-scf00040	2
AX-157813587	3	49.781	KV452730.1	3501122	2-scf00040	2
AX-157925073	3	49.781	KV452730.1	3510508	2-scf00040	2
AX-157750390	3	49.781	KV452730.1	3512362	2-scf00040	2
AX-157813589	3	49.781	KV452730.1	3515123	2-scf00040	2
AX-157925077	3	49.781	KV452730.1	3519929	2-scf00040	2
AX-157925081	3	49.781	KV452730.1	3522018	2-scf00040	2
AX-157813591	3	49.781	KV452730.1	3524016	2-scf00040	2
AX-157925085	3	49.781	KV452730.1	3524305	2-scf00040	2
AX-157925089	3	49.781	KV452730.1	3525138	2-scf00040	2
AX-157864592	3	49.781	KV452730.1	3529801	2-scf00040	2
AX-157925099	3	49.781	KV452730.1	3533579	2-scf00040	2
AX-157925101	3	49.781	KV452730.1	3534359	2-scf00040	2
AX-157925102	3	49.781	KV452730.1	3535013	2-scf00040	2
AX-157925103	3	49.781	KV452730.1	3535395	2-scf00040	2
AX-157925104	3	49.781	KV452730.1	3535907	2-scf00040	2
AX-157925106	3	49.781	KV452730.1	3537928	2-scf00040	2
AX-157925109	3	49.781	KV452730.1	3541137	2-scf00040	2
AX-157925111	3	49.781	KV452730.1	3543937	2-scf00040	2
AX-157925113	3	49.781	KV452730.1	3544107	2-scf00040	2
AX-157925116	3	49.781	KV452730.1	3549367	2-scf00040	2
AX-157925119	3	49.781	KV452730.1	3553042	2-scf00040	2
AX-157925131	3	49.781	KV452730.1	3569707	2-scf00040	2
AX-157925133	3	49.781	KV452730.1	3572024	2-scf00040	2
AX-157925146	3	49.781	KV452730.1	3595920	2-scf00040	2
AX-157925204	3	49.781	KV452730.1	3679547	2-scf00040	2
AX-157925215	3	49.781	KV452730.1	3684948	2-scf00040	2
AX-157813595	3	49.781	KV452730.1	3686517	2-scf00040	2
AX-157813599	3	49.781	KV452730.1	3713493	2-scf00040	2
AX-157925246	3	49.781	KV452730.1	3717559	2-scf00040	2
AX-158379324	3	49.781	KV452730.1	3724451	2-scf00040	2
AX-157925257	3	49.781	KV452730.1	3726746	2-scf00040	2
AX-157925259	3	49.781	KV452730.1	3729852	2-scf00040	2
AX-157925261	3	49.781	KV452730.1	3729903	2-scf00040	2
AX-157718163	3	49.781	KV452730.1	3732407	2-scf00040	2
AX-157925288	3	49.781	KV452730.1	3762809	2-scf00040	2
AX-157925290	3	49.781	KV452730.1	3770591	2-scf00040	2
AX-157925294	3	49.781	KV452730.1	3771752	2-scf00040	2
AX-157925304	3	49.781	KV452730.1	3778514	2-scf00040	2
AX-157925087	3	49.89	KV452730.1	3524637	2-scf00040	2
AX-157925130	3	49.89	KV452730.1	3568066	2-scf00040	2
AX-157925136	3	49.89	KV452730.1	3573076	2-scf00040	2
AX-157925137	3	49.89	KV452730.1	3573501	2-scf00040	2
AX-157925138	3	49.89	KV452730.1	3575040	2-scf00040	2
AX-157925167	3	49.89	KV452730.1	3632126	2-scf00040	2
AX-157925171	3	49.89	KV452730.1	3634263	2-scf00040	2
AX-157925175	3	49.89	KV452730.1	3662793	2-scf00040	2
AX-157925180	3	49.89	KV452730.1	3664409	2-scf00040	2
AX-157925182	3	49.89	KV452730.1	3664833	2-scf00040	2
AX-157925183	3	49.89	KV452730.1	3665294	2-scf00040	2

AX-157925188	3	49.89	KV452730.1	3666295	2-scf00040	2
AX-157925201	3	49.89	KV452730.1	3677747	2-scf00040	2
AX-157925217	3	49.89	KV452730.1	3685409	2-scf00040	2
AX-157925302	3	49.89	KV452730.1	3777970	2-scf00040	2
AX-157925219	3	49.911	KV452730.1	3690162	2-scf00040	2
AX-157925220	3	49.936	KV452730.1	3691091	2-scf00040	2
AX-157925222	3	49.968	KV452730.1	3693210	2-scf00040	2
AX-157925162	3	49.999	KV452730.1	3630167	2-scf00040	2
AX-157745710	3	49.999	KV452730.1	3713985	2-scf00040	2
AX-157772562	3	49.999	KV452730.1	3715385	2-scf00040	2
AX-157925254	3	49.999	KV452730.1	3725365	2-scf00040	2
AX-157713610	3	49.999	KV452730.1	3735609	2-scf00040	2
AX-157925263	3	49.999	KV452730.1	3736116	2-scf00040	2
AX-157925301	3	49.999	KV452730.1	3776670	2-scf00040	2
AX-157925266	3	50.027	KV452730.1	3736636	2-scf00040	2
AX-157925286	3	50.074	KV452730.1	3761563	2-scf00040	2
AX-157813586	3	50.108	KV452730.1	3500836	2-scf00040	2
AX-157925123	3	50.108	KV452730.1	3560558	2-scf00040	2
AX-157925297	3	50.108	KV452730.1	3774481	2-scf00040	2
AX-157925305	3	50.108	KV452730.1	3782080	2-scf00040	2
AX-157925314	3	50.108	KV452730.1	3790701	2-scf00040	2
AX-157925316	3	50.108	KV452730.1	3791031	2-scf00040	2
AX-157925319	3	50.108	KV452730.1	3791353	2-scf00040	2
AX-158405336	3	50.108	KV452730.1	3792367	2-scf00040	2
AX-157924481	3	50.109	KV452730.1	2931789	2-scf00040	2
AX-158405333	3	50.109	KV452730.1	3060961	2-scf00040	2
AX-157924743	3	50.109	KV452730.1	3158398	2-scf00040	2
AX-157924986	3	50.109	KV452730.1	3405634	2-scf00040	2
AX-157925001	3	50.109	KV452730.1	3413792	2-scf00040	2
AX-157925034	3	50.109	KV452730.1	3457373	2-scf00040	2
AX-157925062	3	50.109	KV452730.1	3492150	2-scf00040	2
AX-157925092	3	50.109	KV452730.1	3528784	2-scf00040	2
AX-157925095	3	50.109	KV452730.1	3529184	2-scf00040	2
AX-157925153	3	50.109	KV452730.1	3618813	2-scf00040	2
AX-157925160	3	50.109	KV452730.1	3629801	2-scf00040	2
AX-157925173	3	50.109	KV452730.1	3635725	2-scf00040	2
AX-157925191	3	50.109	KV452730.1	3669596	2-scf00040	2
AX-157925231	3	50.109	KV452730.1	3699983	2-scf00040	2
AX-157813600	3	50.109	KV452730.1	3720799	2-scf00040	2
AX-157813601	3	50.109	KV452730.1	3723461	2-scf00040	2
AX-157925298	3	50.109	KV452730.1	3775106	2-scf00040	2
AX-157853865	3	50.109	KV452730.1	3775712	2-scf00040	2
AX-157925320	3	50.109	KV452730.1	3792556	2-scf00040	2
AX-157925065	3	50.417	KV452730.1	3501336	2-scf00040	2
AX-157925223	3	50.417	KV452730.1	3694056	2-scf00040	2
AX-157925323	3	50.417	KV452730.1	3798255	2-scf00040	2
AX-157925324	3	50.417	KV452730.1	3799063	2-scf00040	2
AX-157925329	3	50.865	KV452730.1	3810401	2-scf00040	2
AX-157724876	3	50.865	KV452730.1	3816444	2-scf00040	2
AX-157925239	3	50.868	KV452730.1	3709877	2-scf00040	2
AX-157925332	3	50.868	KV452730.1	3811878	2-scf00040	2

AX-157925353	3	50.868	KV452730.1	3840052	2-scf00040	2
AX-157925360	3	50.868	KV452730.1	3846590	2-scf00040	2
AX-157925338	3	50.871	KV452730.1	3824709	2-scf00040	2
AX-157771877	3	50.874	KV452730.1	3833730	2-scf00040	2
AX-157925346	3	50.874	KV452730.1	3834562	2-scf00040	2
AX-157925347	3	50.874	KV452730.1	3835266	2-scf00040	2
AX-157925350	3	50.874	KV452730.1	3837101	2-scf00040	2
AX-157925352	3	50.874	KV452730.1	3838670	2-scf00040	2
AX-158389695	3	51.092	KV452730.1	3851585	2-scf00040	2
AX-157720876	3	51.092	KV452730.1	3852000	2-scf00040	2
AX-157925368	3	51.092	KV452730.1	3853158	2-scf00040	2
AX-157925369	3	51.092	KV452730.1	3855965	2-scf00040	2
AX-158379325	3	51.092	KV452730.1	3856281	2-scf00040	2
AX-157925371	3	51.092	KV452730.1	3857954	2-scf00040	2
AX-157925372	3	51.092	KV452730.1	3859333	2-scf00040	2
AX-157925374	3	51.092	KV452730.1	3860034	2-scf00040	2
AX-157925375	3	51.092	KV452730.1	3862676	2-scf00040	2
AX-157925377	3	51.092	KV452730.1	3862921	2-scf00040	2
AX-157925378	3	51.092	KV452730.1	3863805	2-scf00040	2
AX-157925380	3	51.092	KV452730.1	3864579	2-scf00040	2
AX-157925381	3	51.092	KV452730.1	3864787	2-scf00040	2
AX-157713725	3	51.092	KV452730.1	3865253	2-scf00040	2
AX-157925386	3	51.092	KV452730.1	3871097	2-scf00040	2
AX-157925388	3	51.092	KV452730.1	3871732	2-scf00040	2
AX-157925389	3	51.092	KV452730.1	3871961	2-scf00040	2
AX-157740719	3	51.092	KV452730.1	3872798	2-scf00040	2
AX-157925390	3	51.092	KV452730.1	3873160	2-scf00040	2
AX-157925391	3	51.092	KV452730.1	3873789	2-scf00040	2
AX-157925394	3	51.092	KV452730.1	3874452	2-scf00040	2
AX-157925397	3	51.092	KV452730.1	3875070	2-scf00040	2
AX-157749971	3	51.092	KV452730.1	3902165	2-scf00040	2
AX-157925407	3	51.215	KV452730.1	3887921	2-scf00040	2
AX-157925410	3	51.455	KV452730.1	3891754	2-scf00040	2
AX-157719369	3	51.456	KV452730.1	3818067	2-scf00040	2
AX-157925405	3	51.456	KV452730.1	3882311	2-scf00040	2
AX-157925411	3	51.456	KV452730.1	3892368	2-scf00040	2
AX-157925434	3	51.456	KV452730.1	3914996	2-scf00040	2
AX-157925451	3	51.456	KV452730.1	3937702	2-scf00040	2
AX-157925452	3	51.456	KV452730.1	3938510	2-scf00040	2
AX-157925413	3	51.638	KV452730.1	3896758	2-scf00040	2
AX-157925414	3	51.638	KV452730.1	3898040	2-scf00040	2
AX-157813610	3	51.638	KV452730.1	3900297	2-scf00040	2
AX-157925417	3	51.638	KV452730.1	3901535	2-scf00040	2
AX-157813611	3	51.638	KV452730.1	3901741	2-scf00040	2
AX-157717850	3	51.638	KV452730.1	3904538	2-scf00040	2
AX-157925420	3	51.638	KV452730.1	3904991	2-scf00040	2
AX-157925421	3	51.638	KV452730.1	3905546	2-scf00040	2
AX-157925428	3	51.638	KV452730.1	3908928	2-scf00040	2
AX-157775691	3	51.721	KV452730.1	3907245	2-scf00040	2
AX-157925427	3	51.806	KV452730.1	3908375	2-scf00040	2
AX-157925419	3	51.857	KV452730.1	3904465	2-scf00040	2

AX-157925424	3	51.857	KV452730.1	3907056	2-scf00040	2
AX-157813612	3	51.857	KV452730.1	3912048	2-scf00040	2
AX-157925432	3	51.857	KV452730.1	3913820	2-scf00040	2
AX-157925439	3	51.857	KV452730.1	3919306	2-scf00040	2
AX-157741264	3	51.857	KV452730.1	3922519	2-scf00040	2
AX-157925444	3	51.857	KV452730.1	3926785	2-scf00040	2
AX-157925445	3	51.857	KV452730.1	3929314	2-scf00040	2
AX-157925447	3	51.857	KV452730.1	3929749	2-scf00040	2
AX-157925450	3	51.857	KV452730.1	3935978	2-scf00040	2
AX-157925456	3	51.857	KV452730.1	3944815	2-scf00040	2
AX-157925457	3	51.857	KV452730.1	3945958	2-scf00040	2
AX-157925459	3	51.857	KV452730.1	3949194	2-scf00040	2
AX-157925460	3	51.857	KV452730.1	3949529	2-scf00040	2
AX-157925467	3	52.289	KV452730.1	3959455	2-scf00040	2
AX-157925465	3	52.294	KV452730.1	3955122	2-scf00040	2
AX-157724456	3	52.294	KV452730.1	3959714	2-scf00040	2
AX-157925468	3	52.294	KV452730.1	3960023	2-scf00040	2
AX-157813617	3	52.294	KV452730.1	3960719	2-scf00040	2
AX-157925469	3	52.294	KV452730.1	3961861	2-scf00040	2
AX-157723674	3	52.294	KV452730.1	3964029	2-scf00040	2
AX-157925475	3	52.294	KV452730.1	3966647	2-scf00040	2
AX-157925476	3	52.294	KV452730.1	3967609	2-scf00040	2
AX-157722092	3	52.294	KV452730.1	3968672	2-scf00040	2
AX-157874830	3	52.294	KV452730.1	3969243	2-scf00040	2
AX-157925479	3	52.294	KV452730.1	3971573	2-scf00040	2
AX-157925480	3	52.294	KV452730.1	3972808	2-scf00040	2
AX-157925481	3	52.294	KV452730.1	3974250	2-scf00040	2
AX-157925484	3	52.294	KV452730.1	3976611	2-scf00040	2
AX-157925485	3	52.294	KV452730.1	3978587	2-scf00040	2
AX-157925486	3	52.512	KV452730.1	3980200	2-scf00040	2
AX-157925487	3	52.622	KV452730.1	3980858	2-scf00040	2
AX-157925489	3	52.627	KV452730.1	3981243	2-scf00040	2
AX-157925491	3	52.634	KV452730.1	3981926	2-scf00040	2
AX-157925492	3	52.641	KV452730.1	3983127	2-scf00040	2
AX-157925493	3	52.656	KV452730.1	3983978	2-scf00040	2
AX-157925494	3	52.892	KV452730.1	3984986	2-scf00040	2
AX-157925500	3	52.892	KV452730.1	3990130	2-scf00040	2
AX-157925515	3	52.892	KV452730.1	4003624	2-scf00040	2
AX-157925526	3	52.892	KV452730.1	4010822	2-scf00040	2
AX-157925507	3	52.963	KV452730.1	3998080	2-scf00040	2
AX-157925509	3	53.031	KV452730.1	3998811	2-scf00040	2
AX-157813619	3	53.09	KV452730.1	3999640	2-scf00040	2
AX-157925513	3	53.13	KV452730.1	4002218	2-scf00040	2
AX-157925463	3	53.168	KV452730.1	3950767	2-scf00040	2
AX-157925478	3	53.168	KV452730.1	3969526	2-scf00040	2
AX-157925510	3	53.168	KV452730.1	4000461	2-scf00040	2
AX-157813620	3	53.168	KV452730.1	4003449	2-scf00040	2
AX-157925529	3	53.168	KV452730.1	4012887	2-scf00040	2
AX-157925531	3	53.168	KV452730.1	4014426	2-scf00040	2
AX-157925533	3	53.168	KV452730.1	4014789	2-scf00040	2
AX-157925543	3	53.168	KV452730.1	4025903	2-scf00040	2

AX-157925552	3	53.168	KV452730.1	4039403	2-scf00040	2
AX-157813623	3	53.278	KV452730.1	4023123	2-scf00040	2
AX-157925546	3	53.286	KV452730.1	4034268	2-scf00040	2
AX-157925534	3	53.332	KV452730.1	4015371	2-scf00040	2
AX-157925542	3	53.402	KV452730.1	4025193	2-scf00040	2
AX-157925545	3	53.402	KV452730.1	4030462	2-scf00040	2
AX-157925554	3	53.402	KV452730.1	4042282	2-scf00040	2
AX-157925556	3	53.402	KV452730.1	4045217	2-scf00040	2
AX-157723258	3	53.402	KV452730.1	4046324	2-scf00040	2
AX-157925561	3	53.402	KV452730.1	4050714	2-scf00040	2
AX-157813626	3	54.134	KV452730.1	4056419	2-scf00040	2
AX-157813627	3	54.135	KV452730.1	4059421	2-scf00040	2
AX-157925579	3	54.135	KV452730.1	4080168	2-scf00040	2
AX-157925566	3	54.137	KV452730.1	4060001	2-scf00040	2
AX-157925570	3	54.137	KV452730.1	4065026	2-scf00040	2
AX-157749825	3	54.274	KV452730.1	4069345	2-scf00040	2
AX-157925571	3	54.439	KV452730.1	4069574	2-scf00040	2
AX-157925572	3	54.439	KV452730.1	4070089	2-scf00040	2
AX-157925573	3	54.59	KV452730.1	4071153	2-scf00040	2
AX-157771210	3	54.59	KV452730.1	4071751	2-scf00040	2
AX-157813630	3	54.59	KV452730.1	4073995	2-scf00040	2
AX-157813631	3	54.59	KV452730.1	4075862	2-scf00040	2
AX-157925578	3	54.59	KV452730.1	4075908	2-scf00040	2
AX-157743393	3	54.59	KV452730.1	4076262	2-scf00040	2
AX-157925581	3	54.59	KV452730.1	4082809	2-scf00040	2
AX-157925584	3	54.59	KV452730.1	4083901	2-scf00040	2
AX-157925586	3	54.59	KV452730.1	4085511	2-scf00040	2
AX-157925585	3	54.663	KV452730.1	4084808	2-scf00040	2
AX-157813632	3	54.689	KV452730.1	4087135	2-scf00040	2
AX-157925588	3	54.735	KV452730.1	4087235	2-scf00040	2
AX-157813634	3	55.019	KV452730.1	4092398	2-scf00040	2
AX-157710120	3	55.019	KV452730.1	4093407	2-scf00040	2
AX-157925590	3	55.03	KV452730.1	4090061	2-scf00040	2
AX-157925599	3	55.03	KV452730.1	4096374	2-scf00040	2
AX-157723596	3	55.03	KV452730.1	4104957	2-scf00040	2
AX-157925603	3	55.03	KV452730.1	4105498	2-scf00040	2
AX-157925608	3	55.03	KV452730.1	4107292	2-scf00040	2
AX-157925609	3	55.03	KV452730.1	4108176	2-scf00040	2
AX-157925610	3	55.03	KV452730.1	4108779	2-scf00040	2
AX-157751785	3	55.03	KV452730.1	4112825	2-scf00040	2
AX-157710719	3	55.031	KV452730.1	4102410	2-scf00040	2
AX-157925616	3	55.155	KV452730.1	4119677	2-scf00040	2
AX-157925624	3	55.155	KV452730.1	4122556	2-scf00040	2
AX-157925619	3	55.302	KV452730.1	4121034	2-scf00040	2
AX-157925626	3	55.465	KV452730.1	4122882	2-scf00040	2
AX-157925627	3	55.465	KV452730.1	4123141	2-scf00040	2
AX-157925629	3	55.497	KV452730.1	4124383	2-scf00040	2
AX-157749910	3	55.574	KV452730.1	4126494	2-scf00040	2
AX-157925633	3	55.574	KV452730.1	4129795	2-scf00040	2
AX-157925634	3	55.574	KV452730.1	4132424	2-scf00040	2
AX-157925638	3	55.793	KV452730.1	4134064	2-scf00040	2

AX-157925644	3	55.793	KV452730.1	4139927	2-scf00040	2
AX-157717781	3	55.793	KV452730.1	4140515	2-scf00040	2
AX-157710555	3	55.793	KV452730.1	4140815	2-scf00040	2
AX-157925646	3	55.793	KV452730.1	4141998	2-scf00040	2
AX-157925649	3	55.934	KV452730.1	4146007	2-scf00040	2
AX-157925651	3	55.937	KV452730.1	4147113	2-scf00040	2
AX-157925654	3	56.06	KV452730.1	4148762	2-scf00040	2
AX-157813637	3	56.132	KV452730.1	4149476	2-scf00040	2
AX-157925655	3	56.132	KV452730.1	4151638	2-scf00040	2
AX-157925647	3	56.279	KV452730.1	4145391	2-scf00040	2
AX-157925657	3	56.279	KV452730.1	4152389	2-scf00040	2
AX-157925665	3	56.42	KV452730.1	4160651	2-scf00040	2
AX-157925667	3	56.42	KV452730.1	4161253	2-scf00040	2
AX-158379327	3	56.777	KV452730.1	4169008	2-scf00040	2
AX-157813638	3	56.777	KV452730.1	4169435	2-scf00040	2
AX-157925672	3	56.777	KV452730.1	4169498	2-scf00040	2
AX-157853868	3	56.777	KV452730.1	4176947	2-scf00040	2
AX-157739467	3	56.779	KV452730.1	4171804	2-scf00040	2
AX-157744274	3	57.159	KV452730.1	4164020	2-scf00040	2
AX-157925674	3	57.159	KV452730.1	4172757	2-scf00040	2
AX-157925675	3	57.159	KV452730.1	4173027	2-scf00040	2
AX-157925676	3	57.159	KV452730.1	4174327	2-scf00040	2
AX-157925678	3	57.159	KV452730.1	4175275	2-scf00040	2
AX-157925679	3	57.159	KV452730.1	4176071	2-scf00040	2
AX-157925681	3	57.159	KV452730.1	4176714	2-scf00040	2
AX-157713630	3	57.286	KV452730.1	4178221	2-scf00040	2
AX-157925683	3	57.286	KV452730.1	4179613	2-scf00040	2
AX-157925686	3	57.286	KV452730.1	4180613	2-scf00040	2
AX-157925687	3	57.286	KV452730.1	4181235	2-scf00040	2
AX-157925688	3	57.286	KV452730.1	4182384	2-scf00040	2
AX-157925695	3	57.286	KV452730.1	4185816	2-scf00040	2
AX-157925692	3	57.54	KV452730.1	4185130	2-scf00040	2
AX-157770931	3	57.554	KV452730.1	4194932	2-scf00040	2
AX-158379328	3	57.683	KV452730.1	4186594	2-scf00040	2
AX-157925704	3	57.683	KV452730.1	4198949	2-scf00040	2
AX-157925706	3	57.683	KV452730.1	4202443	2-scf00040	2
AX-157925708	3	57.683	KV452730.1	4202909	2-scf00040	2
AX-157925762	3	57.916	KV452730.1	4261320	2-scf00040	2
AX-157925766	3	57.916	KV452730.1	4263233	2-scf00040	2
AX-157925767	3	57.916	KV452730.1	4263472	2-scf00040	2
AX-157773629	3	57.916	KV452730.1	4264102	2-scf00040	2
AX-157925750	3	58.198	KV452730.1	4248683	2-scf00040	2
AX-157925772	3	58.198	KV452730.1	4264976	2-scf00040	2
AX-157925774	3	58.198	KV452730.1	4267425	2-scf00040	2
AX-157813652	3	58.198	KV452730.1	4268804	2-scf00040	2
AX-158379329	3	58.198	KV452730.1	4269210	2-scf00040	2
AX-157925777	3	58.198	KV452730.1	4270846	2-scf00040	2
AX-157925780	3	58.198	KV452730.1	4272383	2-scf00040	2
AX-157721346	3	58.198	KV452730.1	4272638	2-scf00040	2
AX-157753275	3	58.198	KV452730.1	4273179	2-scf00040	2
AX-157703546	3	58.198	KV452730.1	4273325	2-scf00040	2

AX-157748523	3	58.198	KV452730.1	4279156	2-scf00040	2
AX-157925782	3	58.198	KV452730.1	4280921	2-scf00040	2
AX-157747315	3	58.198	KV452730.1	4288054	2-scf00040	2
AX-157925794	3	58.198	KV452730.1	4290264	2-scf00040	2
AX-157925795	3	58.198	KV452730.1	4290658	2-scf00040	2
AX-157925800	3	58.198	KV452730.1	4296742	2-scf00040	2
AX-157770030	3	58.198	KV452730.1	4297657	2-scf00040	2
AX-157925802	3	58.198	KV452730.1	4299172	2-scf00040	2
AX-157925804	3	58.198	KV452730.1	4300076	2-scf00040	2
AX-157925807	3	58.198	KV452730.1	4303153	2-scf00040	2
AX-157925815	3	58.198	KV452730.1	4309860	2-scf00040	2
AX-157925816	3	58.198	KV452730.1	4312187	2-scf00040	2
AX-157925822	3	58.198	KV452730.1	4322722	2-scf00040	2
AX-157925824	3	58.198	KV452730.1	4325741	2-scf00040	2
AX-157925826	3	58.198	KV452730.1	4328084	2-scf00040	2
AX-157925849	3	58.198	KV452730.1	4349224	2-scf00040	2
AX-157925857	3	58.198	KV452730.1	4357057	2-scf00040	2
AX-158389698	3	58.198	KV452730.1	4376195	2-scf00040	2
AX-157925881	3	58.198	KV452730.1	4383433	2-scf00040	2
AX-157925827	3	58.416	KV452730.1	4330930	2-scf00040	2
AX-157925831	3	58.416	KV452730.1	4334005	2-scf00040	2
AX-157925833	3	58.416	KV452730.1	4335440	2-scf00040	2
AX-157925840	3	58.416	KV452730.1	4340038	2-scf00040	2
AX-157925843	3	58.416	KV452730.1	4342317	2-scf00040	2
AX-157813658	3	58.416	KV452730.1	4342735	2-scf00040	2
AX-157925858	3	58.416	KV452730.1	4358235	2-scf00040	2
AX-157925865	3	58.416	KV452730.1	4364142	2-scf00040	2
AX-157925872	3	58.416	KV452730.1	4369664	2-scf00040	2
AX-157813661	3	58.416	KV452730.1	4388890	2-scf00040	2
AX-157753044	3	58.626	KV452730.1	4387569	2-scf00040	2
AX-157925883	3	58.626	KV452730.1	4387863	2-scf00040	2
AX-157925888	3	58.644	KV452730.1	4392715	2-scf00040	2
AX-157925891	3	58.644	KV452730.1	4394296	2-scf00040	2
AX-157925892	3	58.685	KV452730.1	4395001	2-scf00040	2
AX-157925914	3	58.722	KV452730.1	4418603	2-scf00040	2
AX-157725021	3	58.853	KV452730.1	4422055	2-scf00040	2
AX-157925920	3	58.853	KV452730.1	4425147	2-scf00040	2
AX-157925926	3	58.853	KV452730.1	4426643	2-scf00040	2
AX-157925938	3	58.853	KV452730.1	4438719	2-scf00040	2
AX-157925941	3	58.853	KV452730.1	4440525	2-scf00040	2
AX-157926074	3	58.853	KV452730.1	4582327	2-scf00040	2
AX-157926356	3	58.853	KV452730.1	4904344	2-scf00040	2
AX-157925905	3	58.909	KV452730.1	4406221	2-scf00040	2
AX-157925924	3	58.909	KV452730.1	4426304	2-scf00040	2
AX-157925942	3	58.909	KV452730.1	4441144	2-scf00040	2
AX-157925953	3	58.909	KV452730.1	4454001	2-scf00040	2
AX-157925959	3	58.909	KV452730.1	4460833	2-scf00040	2
AX-157926019	3	58.909	KV452730.1	4526534	2-scf00040	2
AX-157926066	3	58.909	KV452730.1	4577555	2-scf00040	2
AX-157926092	3	58.909	KV452730.1	4597990	2-scf00040	2
AX-157926094	3	58.909	KV452730.1	4599414	2-scf00040	2

AX-157926165	3	58.909	KV452730.1	4661834	2-scf00040	2
AX-157926232	3	58.909	KV452730.1	4740069	2-scf00040	2
AX-157926314	3	58.909	KV452730.1	4855852	2-scf00040	2
AX-157926349	3	58.909	KV452730.1	4899222	2-scf00040	2
AX-157926355	3	58.909	KV452730.1	4903911	2-scf00040	2
AX-157926459	3	58.909	KV452730.1	5002544	2-scf00040	2
AX-157874835	3	58.909	KV452730.1	5315245	2-scf00040	2
AX-157927154	3	58.909	KV452730.1	5589687	2-scf00040	2
AX-157927156	3	58.909	KV452730.1	5589984	2-scf00040	2
AX-157927157	3	58.909	KV452730.1	5591289	2-scf00040	2
AX-157927206	3	58.909	KV452730.1	5625704	2-scf00040	2
AX-157853877	3	58.909	KV452730.1	5802140	2-scf00040	2
AX-157927480	3	58.909	KV452730.1	5873705	2-scf00040	2
AX-157927481	3	58.909	KV452730.1	5873839	2-scf00040	2
AX-157927488	3	58.909	KV452730.1	5882290	2-scf00040	2
AX-157927489	3	58.909	KV452730.1	5882971	2-scf00040	2
AX-157927491	3	58.909	KV452730.1	5885031	2-scf00040	2
AX-157927563	3	58.909	KV452730.1	5964387	2-scf00040	2
AX-157813776	3	58.909	KV452730.1	6001935	2-scf00040	2
AX-157927623	3	58.909	KV452730.1	6011017	2-scf00040	2
AX-157927688	3	58.909	KV452730.1	6068418	2-scf00040	2
AX-157927720	3	58.909	KV452730.1	6088646	2-scf00040	2
AX-157925850	3	58.963	KV452730.1	4350530	2-scf00040	2
AX-157925875	3	58.963	KV452730.1	4375811	2-scf00040	2
AX-157925913	3	58.963	KV452730.1	4416133	2-scf00040	2
AX-157925918	3	58.963	KV452730.1	4419894	2-scf00040	2
AX-157925945	3	58.963	KV452730.1	4442872	2-scf00040	2
AX-157925954	3	58.963	KV452730.1	4455839	2-scf00040	2
AX-157760818	3	58.963	KV452730.1	4457718	2-scf00040	2
AX-157925956	3	58.963	KV452730.1	4459721	2-scf00040	2
AX-157925961	3	58.963	KV452730.1	4463234	2-scf00040	2
AX-157925965	3	58.963	KV452730.1	4469858	2-scf00040	2
AX-157925968	3	58.963	KV452730.1	4474143	2-scf00040	2
AX-157925970	3	58.963	KV452730.1	4479314	2-scf00040	2
AX-157925973	3	58.963	KV452730.1	4482179	2-scf00040	2
AX-157925976	3	58.963	KV452730.1	4484212	2-scf00040	2
AX-157925978	3	58.963	KV452730.1	4485166	2-scf00040	2
AX-157925981	3	58.963	KV452730.1	4486435	2-scf00040	2
AX-157925982	3	58.963	KV452730.1	4487968	2-scf00040	2
AX-157813670	3	58.963	KV452730.1	4488437	2-scf00040	2
AX-157925985	3	58.963	KV452730.1	4490850	2-scf00040	2
AX-157925992	3	58.963	KV452730.1	4496536	2-scf00040	2
AX-157925993	3	58.963	KV452730.1	4496945	2-scf00040	2
AX-157925994	3	58.963	KV452730.1	4498107	2-scf00040	2
AX-157925997	3	58.963	KV452730.1	4500938	2-scf00040	2
AX-157926004	3	58.963	KV452730.1	4505967	2-scf00040	2
AX-157746766	3	58.963	KV452730.1	4522970	2-scf00040	2
AX-157926016	3	58.963	KV452730.1	4524482	2-scf00040	2
AX-157926018	3	58.963	KV452730.1	4525949	2-scf00040	2
AX-157869181	3	58.963	KV452730.1	4532369	2-scf00040	2
AX-157869182	3	58.963	KV452730.1	4541411	2-scf00040	2

AX-157926032	3	58.963	KV452730.1	4544747	2-scf00040	2
AX-157883638	3	58.963	KV452730.1	4553804	2-scf00040	2
AX-157813675	3	58.963	KV452730.1	4561684	2-scf00040	2
AX-157813677	3	58.963	KV452730.1	4596669	2-scf00040	2
AX-157926104	3	58.963	KV452730.1	4604858	2-scf00040	2
AX-157813678	3	58.963	KV452730.1	4611272	2-scf00040	2
AX-157926113	3	58.963	KV452730.1	4616199	2-scf00040	2
AX-157926136	3	58.963	KV452730.1	4638250	2-scf00040	2
AX-157926199	3	58.963	KV452730.1	4692308	2-scf00040	2
AX-157926202	3	58.963	KV452730.1	4695104	2-scf00040	2
AX-157926226	3	58.963	KV452730.1	4733716	2-scf00040	2
AX-158404494	3	58.963	KV452730.1	4941229	2-scf00040	2
AX-157927499	3	58.963	KV452730.1	5898765	2-scf00040	2
AX-157926015	3	58.998	KV452730.1	4524016	2-scf00040	2
AX-157926017	3	59.024	KV452730.1	4525493	2-scf00040	2
AX-157757942	3	59.052	KV452730.1	4527215	2-scf00040	2
AX-157813671	3	59.072	KV452730.1	4534965	2-scf00040	2
AX-157926025	3	59.072	KV452730.1	4536313	2-scf00040	2
AX-157926026	3	59.072	KV452730.1	4540252	2-scf00040	2
AX-157926085	3	59.072	KV452730.1	4590333	2-scf00040	2
AX-157926090	3	59.072	KV452730.1	4595557	2-scf00040	2
AX-157926029	3	59.079	KV452730.1	4542100	2-scf00040	2
AX-157926030	3	59.079	KV452730.1	4542769	2-scf00040	2
AX-157926034	3	59.086	KV452730.1	4545904	2-scf00040	2
AX-158421943	3	59.088	KV452730.1	4562615	2-scf00040	2
AX-157925711	3	59.098	KV452730.1	4205960	2-scf00040	2
AX-157926056	3	59.098	KV452730.1	4568012	2-scf00040	2
AX-157926194	3	59.098	KV452730.1	4686013	2-scf00040	2
AX-157926060	3	59.109	KV452730.1	4574412	2-scf00040	2
AX-157926069	3	59.119	KV452730.1	4578697	2-scf00040	2
AX-157926071	3	59.129	KV452730.1	4580757	2-scf00040	2
AX-157926080	3	59.139	KV452730.1	4589081	2-scf00040	2
AX-157926091	3	59.153	KV452730.1	4596515	2-scf00040	2
AX-157926098	3	59.166	KV452730.1	4600471	2-scf00040	2
AX-157926167	3	59.166	KV452730.1	4664145	2-scf00040	2
AX-157739356	3	59.176	KV452730.1	4615110	2-scf00040	2
AX-157925934	3	59.181	KV452730.1	4433784	2-scf00040	2
AX-157813666	3	59.181	KV452730.1	4458370	2-scf00040	2
AX-157925958	3	59.181	KV452730.1	4460449	2-scf00040	2
AX-157813667	3	59.181	KV452730.1	4464892	2-scf00040	2
AX-157813669	3	59.181	KV452730.1	4478994	2-scf00040	2
AX-157925983	3	59.181	KV452730.1	4489442	2-scf00040	2
AX-157925998	3	59.181	KV452730.1	4502192	2-scf00040	2
AX-157926007	3	59.181	KV452730.1	4507336	2-scf00040	2
AX-157926020	3	59.181	KV452730.1	4531096	2-scf00040	2
AX-157926031	3	59.181	KV452730.1	4543103	2-scf00040	2
AX-157926078	3	59.181	KV452730.1	4586475	2-scf00040	2
AX-157926081	3	59.181	KV452730.1	4589671	2-scf00040	2
AX-157926086	3	59.181	KV452730.1	4591277	2-scf00040	2
AX-157813676	3	59.181	KV452730.1	4591516	2-scf00040	2
AX-157926114	3	59.181	KV452730.1	4616618	2-scf00040	2

AX-157926119	3	59.181	KV452730.1	4622330	2-scf00040	2
AX-157926123	3	59.181	KV452730.1	4625742	2-scf00040	2
AX-157926127	3	59.181	KV452730.1	4631347	2-scf00040	2
AX-157926128	3	59.181	KV452730.1	4631895	2-scf00040	2
AX-157926135	3	59.181	KV452730.1	4637178	2-scf00040	2
AX-157926138	3	59.181	KV452730.1	4638501	2-scf00040	2
AX-158405347	3	59.181	KV452730.1	4640631	2-scf00040	2
AX-158405348	3	59.181	KV452730.1	4640718	2-scf00040	2
AX-158405349	3	59.181	KV452730.1	4640769	2-scf00040	2
AX-157767968	3	59.181	KV452730.1	4643660	2-scf00040	2
AX-157772682	3	59.181	KV452730.1	4644870	2-scf00040	2
AX-157926147	3	59.181	KV452730.1	4648435	2-scf00040	2
AX-157926149	3	59.181	KV452730.1	4650668	2-scf00040	2
AX-157926152	3	59.181	KV452730.1	4652042	2-scf00040	2
AX-157926158	3	59.181	KV452730.1	4655616	2-scf00040	2
AX-157926159	3	59.181	KV452730.1	4657906	2-scf00040	2
AX-157926163	3	59.181	KV452730.1	4661574	2-scf00040	2
AX-157926168	3	59.181	KV452730.1	4664751	2-scf00040	2
AX-157926172	3	59.181	KV452730.1	4666247	2-scf00040	2
AX-157926175	3	59.181	KV452730.1	4671259	2-scf00040	2
AX-157926177	3	59.181	KV452730.1	4672272	2-scf00040	2
AX-157926179	3	59.181	KV452730.1	4672697	2-scf00040	2
AX-157926181	3	59.181	KV452730.1	4673377	2-scf00040	2
AX-157753769	3	59.181	KV452730.1	4675365	2-scf00040	2
AX-157926184	3	59.181	KV452730.1	4677207	2-scf00040	2
AX-157926185	3	59.181	KV452730.1	4680529	2-scf00040	2
AX-157926187	3	59.181	KV452730.1	4681469	2-scf00040	2
AX-157926188	3	59.181	KV452730.1	4681800	2-scf00040	2
AX-157813681	3	59.181	KV452730.1	4699686	2-scf00040	2
AX-157813682	3	59.181	KV452730.1	4700628	2-scf00040	2
AX-157926209	3	59.181	KV452730.1	4708903	2-scf00040	2
AX-158405350	3	59.181	KV452730.1	4710154	2-scf00040	2
AX-157926211	3	59.181	KV452730.1	4711791	2-scf00040	2
AX-157926215	3	59.181	KV452730.1	4719761	2-scf00040	2
AX-157926216	3	59.181	KV452730.1	4720855	2-scf00040	2
AX-157926222	3	59.181	KV452730.1	4732846	2-scf00040	2
AX-157926224	3	59.181	KV452730.1	4733272	2-scf00040	2
AX-157926227	3	59.181	KV452730.1	4734125	2-scf00040	2
AX-157926229	3	59.181	KV452730.1	4735589	2-scf00040	2
AX-157926231	3	59.181	KV452730.1	4737612	2-scf00040	2
AX-157813686	3	59.181	KV452730.1	4781185	2-scf00040	2
AX-157926248	3	59.181	KV452730.1	4781976	2-scf00040	2
AX-157926257	3	59.181	KV452730.1	4791310	2-scf00040	2
AX-157926262	3	59.181	KV452730.1	4794780	2-scf00040	2
AX-157926324	3	59.181	KV452730.1	4868323	2-scf00040	2
AX-157926339	3	59.181	KV452730.1	4889426	2-scf00040	2
AX-157927447	3	59.181	KV452730.1	5826166	2-scf00040	2
AX-157813767	3	59.181	KV452730.1	5861102	2-scf00040	2
AX-157927590	3	59.181	KV452730.1	5986177	2-scf00040	2
AX-157926251	3	59.29	KV452730.1	4785834	2-scf00040	2
AX-157926101	3	59.399	KV452730.1	4602197	2-scf00040	2

AX-157774895	3	59.399	KV452730.1	4790109	2-scf00040	2
AX-157926253	3	59.399	KV452730.1	4790226	2-scf00040	2
AX-157926260	3	59.399	KV452730.1	4793418	2-scf00040	2
AX-157926263	3	59.399	KV452730.1	4795262	2-scf00040	2
AX-157926282	3	59.399	KV452730.1	4815061	2-scf00040	2
AX-157709287	3	59.399	KV452730.1	4818146	2-scf00040	2
AX-157926290	3	59.399	KV452730.1	4827818	2-scf00040	2
AX-158414941	3	59.399	KV452730.1	4831998	2-scf00040	2
AX-157813689	3	59.399	KV452730.1	4838818	2-scf00040	2
AX-157926298	3	59.399	KV452730.1	4839788	2-scf00040	2
AX-157927706	3	59.399	KV452730.1	6077639	2-scf00040	2
AX-157926320	3	59.405	KV452730.1	4863311	2-scf00040	2
AX-157926322	3	59.41	KV452730.1	4864752	2-scf00040	2
AX-157926338	3	59.41	KV452730.1	4888892	2-scf00040	2
AX-157744225	3	59.41	KV452730.1	4918439	2-scf00040	2
AX-157813730	3	59.41	KV452730.1	5607088	2-scf00040	2
AX-157927692	3	59.41	KV452730.1	6071683	2-scf00040	2
AX-157926254	3	59.433	KV452730.1	4790672	2-scf00040	2
AX-157853874	3	59.433	KV452730.1	4835980	2-scf00040	2
AX-157926335	3	59.433	KV452730.1	4883521	2-scf00040	2
AX-157813698	3	59.433	KV452730.1	4911176	2-scf00040	2
AX-157813699	3	59.433	KV452730.1	4911524	2-scf00040	2
AX-157926359	3	59.433	KV452730.1	4912617	2-scf00040	2
AX-158405358	3	59.433	KV452730.1	4941375	2-scf00040	2
AX-157927429	3	59.433	KV452730.1	5798072	2-scf00040	2
AX-157926341	3	59.471	KV452730.1	4891296	2-scf00040	2
AX-157926350	3	59.471	KV452730.1	4902568	2-scf00040	2
AX-157927635	3	59.471	KV452730.1	6025603	2-scf00040	2
AX-157926395	3	59.509	KV452730.1	4950746	2-scf00040	2
AX-157926399	3	59.509	KV452730.1	4954182	2-scf00040	2
AX-157926416	3	59.509	KV452730.1	4970429	2-scf00040	2
AX-157926419	3	59.509	KV452730.1	4971763	2-scf00040	2
AX-157926420	3	59.509	KV452730.1	4972132	2-scf00040	2
AX-157926435	3	59.509	KV452730.1	4977794	2-scf00040	2
AX-157926444	3	59.509	KV452730.1	4984140	2-scf00040	2
AX-157926452	3	59.509	KV452730.1	4992648	2-scf00040	2
AX-157926478	3	59.509	KV452730.1	5025549	2-scf00040	2
AX-157926479	3	59.509	KV452730.1	5025955	2-scf00040	2
AX-157813704	3	59.509	KV452730.1	5044987	2-scf00040	2
AX-157813705	3	59.509	KV452730.1	5048832	2-scf00040	2
AX-157926522	3	59.509	KV452730.1	5067912	2-scf00040	2
AX-157926530	3	59.509	KV452730.1	5071011	2-scf00040	2
AX-157813707	3	59.509	KV452730.1	5071684	2-scf00040	2
AX-157813708	3	59.509	KV452730.1	5071953	2-scf00040	2
AX-157926546	3	59.509	KV452730.1	5076614	2-scf00040	2
AX-157869187	3	59.509	KV452730.1	5078900	2-scf00040	2
AX-157869188	3	59.509	KV452730.1	5083653	2-scf00040	2
AX-157926581	3	59.509	KV452730.1	5107179	2-scf00040	2
AX-157926589	3	59.509	KV452730.1	5114871	2-scf00040	2
AX-157926660	3	59.509	KV452730.1	5176260	2-scf00040	2
AX-157926662	3	59.509	KV452730.1	5177499	2-scf00040	2

AX-157926672	3	59.509	KV452730.1	5186528	2-scf00040	2
AX-157926673	3	59.509	KV452730.1	5187476	2-scf00040	2
AX-157926681	3	59.509	KV452730.1	5193068	2-scf00040	2
AX-158389712	3	59.509	KV452730.1	5194710	2-scf00040	2
AX-157926686	3	59.509	KV452730.1	5196159	2-scf00040	2
AX-157926982	3	59.509	KV452730.1	5427143	2-scf00040	2
AX-157926984	3	59.509	KV452730.1	5429636	2-scf00040	2
AX-157926986	3	59.509	KV452730.1	5430803	2-scf00040	2
AX-157926988	3	59.509	KV452730.1	5432728	2-scf00040	2
AX-157869189	3	59.509	KV452730.1	5442690	2-scf00040	2
AX-157927075	3	59.509	KV452730.1	5523554	2-scf00040	2
AX-157927093	3	59.509	KV452730.1	5538318	2-scf00040	2
AX-157927106	3	59.509	KV452730.1	5544715	2-scf00040	2
AX-157813725	3	59.509	KV452730.1	5572943	2-scf00040	2
AX-157927134	3	59.509	KV452730.1	5575207	2-scf00040	2
AX-157927145	3	59.509	KV452730.1	5582682	2-scf00040	2
AX-157869194	3	59.509	KV452730.1	5647788	2-scf00040	2
AX-157927243	3	59.509	KV452730.1	5653135	2-scf00040	2
AX-157927248	3	59.509	KV452730.1	5655061	2-scf00040	2
AX-157869195	3	59.509	KV452730.1	5668943	2-scf00040	2
AX-157927521	3	59.509	KV452730.1	5927346	2-scf00040	2
AX-157813772	3	59.509	KV452730.1	5952388	2-scf00040	2
AX-157926738	3	59.541	KV452730.1	5236485	2-scf00040	2
AX-157926761	3	59.572	KV452730.1	5250634	2-scf00040	2
AX-157853869	3	59.618	KV452730.1	4256792	2-scf00040	2
AX-157925896	3	59.618	KV452730.1	4396307	2-scf00040	2
AX-157926204	3	59.618	KV452730.1	4695985	2-scf00040	2
AX-157926252	3	59.618	KV452730.1	4788434	2-scf00040	2
AX-157926261	3	59.618	KV452730.1	4793881	2-scf00040	2
AX-157718746	3	59.618	KV452730.1	4797379	2-scf00040	2
AX-157753120	3	59.618	KV452730.1	4800258	2-scf00040	2
AX-157813687	3	59.618	KV452730.1	4819301	2-scf00040	2
AX-157926288	3	59.618	KV452730.1	4827323	2-scf00040	2
AX-157926302	3	59.618	KV452730.1	4846213	2-scf00040	2
AX-157813690	3	59.618	KV452730.1	4849790	2-scf00040	2
AX-157926329	3	59.618	KV452730.1	4874841	2-scf00040	2
AX-157715253	3	59.618	KV452730.1	4878703	2-scf00040	2
AX-157813694	3	59.618	KV452730.1	4879423	2-scf00040	2
AX-157926333	3	59.618	KV452730.1	4879837	2-scf00040	2
AX-157721952	3	59.618	KV452730.1	4884966	2-scf00040	2
AX-157926343	3	59.618	KV452730.1	4891713	2-scf00040	2
AX-158389702	3	59.618	KV452730.1	4894159	2-scf00040	2
AX-157926357	3	59.618	KV452730.1	4910789	2-scf00040	2
AX-157926363	3	59.618	KV452730.1	4914432	2-scf00040	2
AX-157926364	3	59.618	KV452730.1	4921756	2-scf00040	2
AX-157926367	3	59.618	KV452730.1	4925774	2-scf00040	2
AX-157926369	3	59.618	KV452730.1	4926078	2-scf00040	2
AX-157813700	3	59.618	KV452730.1	4926834	2-scf00040	2
AX-157926370	3	59.618	KV452730.1	4927528	2-scf00040	2
AX-157926371	3	59.618	KV452730.1	4927771	2-scf00040	2
AX-157926372	3	59.618	KV452730.1	4929502	2-scf00040	2

AX-157926373	3	59.618	KV452730.1	4929928	2-scf00040	2
AX-157926374	3	59.618	KV452730.1	4931360	2-scf00040	2
AX-157926375	3	59.618	KV452730.1	4932873	2-scf00040	2
AX-157926376	3	59.618	KV452730.1	4933395	2-scf00040	2
AX-157926379	3	59.618	KV452730.1	4935358	2-scf00040	2
AX-157926380	3	59.618	KV452730.1	4936202	2-scf00040	2
AX-157926381	3	59.618	KV452730.1	4936992	2-scf00040	2
AX-157710837	3	59.618	KV452730.1	4938571	2-scf00040	2
AX-157926382	3	59.618	KV452730.1	4939128	2-scf00040	2
AX-157926384	3	59.618	KV452730.1	4942821	2-scf00040	2
AX-157926385	3	59.618	KV452730.1	4943943	2-scf00040	2
AX-157926388	3	59.618	KV452730.1	4947067	2-scf00040	2
AX-157926391	3	59.618	KV452730.1	4948351	2-scf00040	2
AX-157926392	3	59.618	KV452730.1	4949196	2-scf00040	2
AX-157926394	3	59.618	KV452730.1	4950317	2-scf00040	2
AX-157733232	3	59.618	KV452730.1	4953404	2-scf00040	2
AX-157926398	3	59.618	KV452730.1	4953512	2-scf00040	2
AX-157926400	3	59.618	KV452730.1	4954471	2-scf00040	2
AX-157926401	3	59.618	KV452730.1	4956937	2-scf00040	2
AX-157926408	3	59.618	KV452730.1	4962109	2-scf00040	2
AX-157926410	3	59.618	KV452730.1	4964387	2-scf00040	2
AX-157926411	3	59.618	KV452730.1	4964738	2-scf00040	2
AX-157926412	3	59.618	KV452730.1	4965631	2-scf00040	2
AX-157869186	3	59.618	KV452730.1	4966657	2-scf00040	2
AX-157926413	3	59.618	KV452730.1	4967831	2-scf00040	2
AX-157926414	3	59.618	KV452730.1	4970025	2-scf00040	2
AX-157926418	3	59.618	KV452730.1	4970889	2-scf00040	2
AX-157926424	3	59.618	KV452730.1	4972960	2-scf00040	2
AX-157926425	3	59.618	KV452730.1	4973202	2-scf00040	2
AX-157926428	3	59.618	KV452730.1	4973499	2-scf00040	2
AX-157926429	3	59.618	KV452730.1	4973855	2-scf00040	2
AX-157926431	3	59.618	KV452730.1	4975081	2-scf00040	2
AX-157926434	3	59.618	KV452730.1	4976262	2-scf00040	2
AX-157926436	3	59.618	KV452730.1	4978300	2-scf00040	2
AX-157926437	3	59.618	KV452730.1	4979065	2-scf00040	2
AX-157926438	3	59.618	KV452730.1	4980240	2-scf00040	2
AX-157926439	3	59.618	KV452730.1	4980649	2-scf00040	2
AX-157695063	3	59.618	KV452730.1	4981296	2-scf00040	2
AX-157926440	3	59.618	KV452730.1	4982027	2-scf00040	2
AX-157926441	3	59.618	KV452730.1	4982396	2-scf00040	2
AX-157926442	3	59.618	KV452730.1	4983285	2-scf00040	2
AX-157926443	3	59.618	KV452730.1	4983548	2-scf00040	2
AX-157926450	3	59.618	KV452730.1	4989508	2-scf00040	2
AX-157926451	3	59.618	KV452730.1	4991622	2-scf00040	2
AX-158389704	3	59.618	KV452730.1	4992776	2-scf00040	2
AX-157926454	3	59.618	KV452730.1	4994481	2-scf00040	2
AX-157813703	3	59.618	KV452730.1	4996482	2-scf00040	2
AX-157926457	3	59.618	KV452730.1	5000890	2-scf00040	2
AX-157755508	3	59.618	KV452730.1	5004236	2-scf00040	2
AX-157926460	3	59.618	KV452730.1	5005472	2-scf00040	2
AX-157700540	3	59.618	KV452730.1	5007624	2-scf00040	2

AX-157926462	3	59.618	KV452730.1	5008544	2-scf00040	2
AX-157926463	3	59.618	KV452730.1	5009359	2-scf00040	2
AX-157926465	3	59.618	KV452730.1	5010753	2-scf00040	2
AX-157926466	3	59.618	KV452730.1	5011206	2-scf00040	2
AX-157926468	3	59.618	KV452730.1	5011773	2-scf00040	2
AX-158389705	3	59.618	KV452730.1	5013206	2-scf00040	2
AX-157926470	3	59.618	KV452730.1	5013999	2-scf00040	2
AX-157926471	3	59.618	KV452730.1	5015918	2-scf00040	2
AX-157926473	3	59.618	KV452730.1	5017634	2-scf00040	2
AX-157926474	3	59.618	KV452730.1	5018466	2-scf00040	2
AX-157926475	3	59.618	KV452730.1	5021465	2-scf00040	2
AX-157926476	3	59.618	KV452730.1	5021908	2-scf00040	2
AX-157926477	3	59.618	KV452730.1	5024697	2-scf00040	2
AX-157926480	3	59.618	KV452730.1	5026637	2-scf00040	2
AX-157926481	3	59.618	KV452730.1	5028503	2-scf00040	2
AX-157926482	3	59.618	KV452730.1	5029309	2-scf00040	2
AX-157926483	3	59.618	KV452730.1	5029830	2-scf00040	2
AX-157926484	3	59.618	KV452730.1	5030190	2-scf00040	2
AX-157926485	3	59.618	KV452730.1	5031033	2-scf00040	2
AX-157926486	3	59.618	KV452730.1	5033004	2-scf00040	2
AX-157926487	3	59.618	KV452730.1	5033593	2-scf00040	2
AX-157926488	3	59.618	KV452730.1	5034111	2-scf00040	2
AX-157926489	3	59.618	KV452730.1	5034322	2-scf00040	2
AX-157926490	3	59.618	KV452730.1	5038024	2-scf00040	2
AX-157926491	3	59.618	KV452730.1	5039093	2-scf00040	2
AX-157926493	3	59.618	KV452730.1	5041539	2-scf00040	2
AX-157926495	3	59.618	KV452730.1	5046792	2-scf00040	2
AX-157926496	3	59.618	KV452730.1	5048387	2-scf00040	2
AX-157926498	3	59.618	KV452730.1	5049008	2-scf00040	2
AX-157926500	3	59.618	KV452730.1	5051045	2-scf00040	2
AX-157926502	3	59.618	KV452730.1	5051708	2-scf00040	2
AX-157926503	3	59.618	KV452730.1	5052538	2-scf00040	2
AX-157926505	3	59.618	KV452730.1	5052946	2-scf00040	2
AX-158389706	3	59.618	KV452730.1	5053321	2-scf00040	2
AX-157926507	3	59.618	KV452730.1	5054680	2-scf00040	2
AX-157926508	3	59.618	KV452730.1	5055340	2-scf00040	2
AX-157926510	3	59.618	KV452730.1	5055864	2-scf00040	2
AX-158389707	3	59.618	KV452730.1	5056198	2-scf00040	2
AX-157926511	3	59.618	KV452730.1	5060412	2-scf00040	2
AX-157926513	3	59.618	KV452730.1	5061212	2-scf00040	2
AX-157726810	3	59.618	KV452730.1	5062598	2-scf00040	2
AX-157926514	3	59.618	KV452730.1	5063424	2-scf00040	2
AX-157926515	3	59.618	KV452730.1	5064400	2-scf00040	2
AX-157813706	3	59.618	KV452730.1	5065792	2-scf00040	2
AX-157926517	3	59.618	KV452730.1	5066091	2-scf00040	2
AX-157926519	3	59.618	KV452730.1	5066373	2-scf00040	2
AX-157926520	3	59.618	KV452730.1	5066777	2-scf00040	2
AX-157926521	3	59.618	KV452730.1	5067208	2-scf00040	2
AX-157926524	3	59.618	KV452730.1	5068340	2-scf00040	2
AX-157926525	3	59.618	KV452730.1	5068570	2-scf00040	2
AX-157926527	3	59.618	KV452730.1	5070174	2-scf00040	2

AX-157926528	3	59.618	KV452730.1	5070633	2-scf00040	2
AX-157926531	3	59.618	KV452730.1	5071288	2-scf00040	2
AX-157926533	3	59.618	KV452730.1	5072316	2-scf00040	2
AX-157813709	3	59.618	KV452730.1	5073471	2-scf00040	2
AX-157926534	3	59.618	KV452730.1	5073769	2-scf00040	2
AX-157926537	3	59.618	KV452730.1	5074181	2-scf00040	2
AX-157926538	3	59.618	KV452730.1	5074489	2-scf00040	2
AX-157926540	3	59.618	KV452730.1	5075248	2-scf00040	2
AX-157926541	3	59.618	KV452730.1	5075502	2-scf00040	2
AX-157926544	3	59.618	KV452730.1	5076246	2-scf00040	2
AX-157926547	3	59.618	KV452730.1	5077464	2-scf00040	2
AX-157926550	3	59.618	KV452730.1	5078486	2-scf00040	2
AX-157926552	3	59.618	KV452730.1	5079413	2-scf00040	2
AX-157926555	3	59.618	KV452730.1	5080466	2-scf00040	2
AX-157926556	3	59.618	KV452730.1	5081141	2-scf00040	2
AX-157926558	3	59.618	KV452730.1	5081420	2-scf00040	2
AX-157926559	3	59.618	KV452730.1	5081638	2-scf00040	2
AX-157926560	3	59.618	KV452730.1	5082313	2-scf00040	2
AX-157853876	3	59.618	KV452730.1	5083856	2-scf00040	2
AX-157926562	3	59.618	KV452730.1	5083930	2-scf00040	2
AX-157926563	3	59.618	KV452730.1	5084357	2-scf00040	2
AX-157926566	3	59.618	KV452730.1	5087152	2-scf00040	2
AX-157926568	3	59.618	KV452730.1	5088377	2-scf00040	2
AX-157926569	3	59.618	KV452730.1	5090361	2-scf00040	2
AX-157926571	3	59.618	KV452730.1	5092655	2-scf00040	2
AX-157926573	3	59.618	KV452730.1	5095506	2-scf00040	2
AX-157926574	3	59.618	KV452730.1	5096421	2-scf00040	2
AX-157926577	3	59.618	KV452730.1	5098042	2-scf00040	2
AX-157926578	3	59.618	KV452730.1	5100518	2-scf00040	2
AX-157926579	3	59.618	KV452730.1	5101440	2-scf00040	2
AX-157926580	3	59.618	KV452730.1	5101892	2-scf00040	2
AX-158405359	3	59.618	KV452730.1	5102007	2-scf00040	2
AX-157926583	3	59.618	KV452730.1	5109701	2-scf00040	2
AX-158389709	3	59.618	KV452730.1	5109803	2-scf00040	2
AX-157926586	3	59.618	KV452730.1	5111724	2-scf00040	2
AX-157926587	3	59.618	KV452730.1	5112726	2-scf00040	2
AX-157926588	3	59.618	KV452730.1	5113827	2-scf00040	2
AX-157693379	3	59.618	KV452730.1	5114635	2-scf00040	2
AX-157926590	3	59.618	KV452730.1	5117444	2-scf00040	2
AX-157926595	3	59.618	KV452730.1	5119677	2-scf00040	2
AX-157926597	3	59.618	KV452730.1	5120621	2-scf00040	2
AX-157926598	3	59.618	KV452730.1	5121297	2-scf00040	2
AX-157926602	3	59.618	KV452730.1	5122340	2-scf00040	2
AX-157926603	3	59.618	KV452730.1	5123981	2-scf00040	2
AX-157926609	3	59.618	KV452730.1	5128292	2-scf00040	2
AX-157718852	3	59.618	KV452730.1	5129230	2-scf00040	2
AX-157926616	3	59.618	KV452730.1	5132919	2-scf00040	2
AX-157926622	3	59.618	KV452730.1	5135335	2-scf00040	2
AX-157926625	3	59.618	KV452730.1	5138131	2-scf00040	2
AX-157926627	3	59.618	KV452730.1	5140390	2-scf00040	2
AX-157926629	3	59.618	KV452730.1	5140657	2-scf00040	2

AX-158389710	3	59.618	KV452730.1	5141852	2-scf00040	2
AX-157926631	3	59.618	KV452730.1	5142346	2-scf00040	2
AX-157926633	3	59.618	KV452730.1	5146154	2-scf00040	2
AX-157926634	3	59.618	KV452730.1	5146314	2-scf00040	2
AX-157926635	3	59.618	KV452730.1	5150123	2-scf00040	2
AX-157926636	3	59.618	KV452730.1	5150596	2-scf00040	2
AX-157708119	3	59.618	KV452730.1	5152177	2-scf00040	2
AX-157926638	3	59.618	KV452730.1	5152444	2-scf00040	2
AX-157926639	3	59.618	KV452730.1	5152915	2-scf00040	2
AX-157926641	3	59.618	KV452730.1	5158204	2-scf00040	2
AX-158389711	3	59.618	KV452730.1	5159465	2-scf00040	2
AX-157926642	3	59.618	KV452730.1	5159889	2-scf00040	2
AX-157926646	3	59.618	KV452730.1	5164551	2-scf00040	2
AX-157926648	3	59.618	KV452730.1	5165986	2-scf00040	2
AX-157926649	3	59.618	KV452730.1	5167307	2-scf00040	2
AX-157926651	3	59.618	KV452730.1	5168098	2-scf00040	2
AX-157926655	3	59.618	KV452730.1	5171915	2-scf00040	2
AX-157926659	3	59.618	KV452730.1	5175743	2-scf00040	2
AX-157813711	3	59.618	KV452730.1	5178234	2-scf00040	2
AX-157926663	3	59.618	KV452730.1	5178627	2-scf00040	2
AX-157926664	3	59.618	KV452730.1	5180768	2-scf00040	2
AX-157926667	3	59.618	KV452730.1	5183953	2-scf00040	2
AX-157926668	3	59.618	KV452730.1	5184287	2-scf00040	2
AX-157926669	3	59.618	KV452730.1	5185441	2-scf00040	2
AX-157926674	3	59.618	KV452730.1	5187896	2-scf00040	2
AX-157926676	3	59.618	KV452730.1	5188287	2-scf00040	2
AX-157926677	3	59.618	KV452730.1	5189613	2-scf00040	2
AX-157926679	3	59.618	KV452730.1	5190116	2-scf00040	2
AX-157813712	3	59.618	KV452730.1	5193542	2-scf00040	2
AX-157926684	3	59.618	KV452730.1	5194761	2-scf00040	2
AX-157749093	3	59.618	KV452730.1	5196630	2-scf00040	2
AX-157926687	3	59.618	KV452730.1	5198297	2-scf00040	2
AX-157813713	3	59.618	KV452730.1	5198445	2-scf00040	2
AX-157926689	3	59.618	KV452730.1	5199328	2-scf00040	2
AX-157926693	3	59.618	KV452730.1	5203027	2-scf00040	2
AX-157926695	3	59.618	KV452730.1	5203448	2-scf00040	2
AX-157711713	3	59.618	KV452730.1	5204459	2-scf00040	2
AX-157926697	3	59.618	KV452730.1	5205629	2-scf00040	2
AX-158389713	3	59.618	KV452730.1	5207134	2-scf00040	2
AX-158389714	3	59.618	KV452730.1	5208912	2-scf00040	2
AX-157926698	3	59.618	KV452730.1	5209321	2-scf00040	2
AX-157926701	3	59.618	KV452730.1	5210683	2-scf00040	2
AX-157926703	3	59.618	KV452730.1	5211132	2-scf00040	2
AX-157926704	3	59.618	KV452730.1	5211684	2-scf00040	2
AX-157926706	3	59.618	KV452730.1	5215271	2-scf00040	2
AX-157926707	3	59.618	KV452730.1	5216496	2-scf00040	2
AX-157926710	3	59.618	KV452730.1	5217362	2-scf00040	2
AX-157926712	3	59.618	KV452730.1	5218060	2-scf00040	2
AX-157926713	3	59.618	KV452730.1	5218970	2-scf00040	2
AX-157926714	3	59.618	KV452730.1	5220901	2-scf00040	2
AX-157926716	3	59.618	KV452730.1	5222210	2-scf00040	2

AX-157926717	3	59.618	KV452730.1	5222963	2-scf00040	2
AX-157926719	3	59.618	KV452730.1	5223531	2-scf00040	2
AX-157926720	3	59.618	KV452730.1	5224505	2-scf00040	2
AX-157926721	3	59.618	KV452730.1	5225899	2-scf00040	2
AX-157926723	3	59.618	KV452730.1	5226287	2-scf00040	2
AX-157926724	3	59.618	KV452730.1	5227027	2-scf00040	2
AX-157926725	3	59.618	KV452730.1	5227894	2-scf00040	2
AX-157926728	3	59.618	KV452730.1	5228727	2-scf00040	2
AX-157926730	3	59.618	KV452730.1	5230224	2-scf00040	2
AX-157926732	3	59.618	KV452730.1	5233255	2-scf00040	2
AX-157926733	3	59.618	KV452730.1	5233808	2-scf00040	2
AX-157926734	3	59.618	KV452730.1	5234730	2-scf00040	2
AX-157926736	3	59.618	KV452730.1	5235244	2-scf00040	2
AX-157926737	3	59.618	KV452730.1	5235751	2-scf00040	2
AX-157926739	3	59.618	KV452730.1	5237322	2-scf00040	2
AX-157926740	3	59.618	KV452730.1	5237977	2-scf00040	2
AX-158405362	3	59.618	KV452730.1	5239178	2-scf00040	2
AX-157926743	3	59.618	KV452730.1	5239881	2-scf00040	2
AX-157926744	3	59.618	KV452730.1	5241066	2-scf00040	2
AX-157926746	3	59.618	KV452730.1	5241838	2-scf00040	2
AX-157926747	3	59.618	KV452730.1	5242709	2-scf00040	2
AX-157926749	3	59.618	KV452730.1	5243626	2-scf00040	2
AX-157926750	3	59.618	KV452730.1	5244182	2-scf00040	2
AX-157926752	3	59.618	KV452730.1	5245469	2-scf00040	2
AX-157926754	3	59.618	KV452730.1	5247126	2-scf00040	2
AX-157926762	3	59.618	KV452730.1	5251383	2-scf00040	2
AX-157926764	3	59.618	KV452730.1	5254785	2-scf00040	2
AX-157727234	3	59.618	KV452730.1	5255277	2-scf00040	2
AX-157926768	3	59.618	KV452730.1	5259825	2-scf00040	2
AX-157926769	3	59.618	KV452730.1	5260496	2-scf00040	2
AX-157739468	3	59.618	KV452730.1	5261192	2-scf00040	2
AX-157926771	3	59.618	KV452730.1	5262259	2-scf00040	2
AX-157926773	3	59.618	KV452730.1	5265059	2-scf00040	2
AX-157708932	3	59.618	KV452730.1	5265903	2-scf00040	2
AX-157926780	3	59.618	KV452730.1	5267699	2-scf00040	2
AX-157926784	3	59.618	KV452730.1	5268989	2-scf00040	2
AX-157926785	3	59.618	KV452730.1	5269391	2-scf00040	2
AX-157926787	3	59.618	KV452730.1	5270189	2-scf00040	2
AX-157926789	3	59.618	KV452730.1	5272467	2-scf00040	2
AX-157926790	3	59.618	KV452730.1	5272920	2-scf00040	2
AX-157926792	3	59.618	KV452730.1	5274332	2-scf00040	2
AX-157926793	3	59.618	KV452730.1	5274839	2-scf00040	2
AX-157926794	3	59.618	KV452730.1	5275391	2-scf00040	2
AX-157926796	3	59.618	KV452730.1	5276376	2-scf00040	2
AX-157926797	3	59.618	KV452730.1	5277261	2-scf00040	2
AX-157926799	3	59.618	KV452730.1	5277550	2-scf00040	2
AX-157926810	3	59.618	KV452730.1	5286798	2-scf00040	2
AX-157926811	3	59.618	KV452730.1	5288636	2-scf00040	2
AX-157926813	3	59.618	KV452730.1	5291578	2-scf00040	2
AX-157926815	3	59.618	KV452730.1	5293039	2-scf00040	2
AX-158389716	3	59.618	KV452730.1	5293114	2-scf00040	2

AX-157926816	3	59.618	KV452730.1	5294607	2-scf00040	2
AX-158405363	3	59.618	KV452730.1	5295460	2-scf00040	2
AX-158405364	3	59.618	KV452730.1	5295499	2-scf00040	2
AX-157926817	3	59.618	KV452730.1	5296212	2-scf00040	2
AX-157926819	3	59.618	KV452730.1	5297289	2-scf00040	2
AX-157926820	3	59.618	KV452730.1	5298638	2-scf00040	2
AX-157926822	3	59.618	KV452730.1	5299116	2-scf00040	2
AX-157926824	3	59.618	KV452730.1	5299961	2-scf00040	2
AX-157926827	3	59.618	KV452730.1	5300787	2-scf00040	2
AX-157926828	3	59.618	KV452730.1	5301159	2-scf00040	2
AX-157926830	3	59.618	KV452730.1	5301527	2-scf00040	2
AX-157926832	3	59.618	KV452730.1	5302084	2-scf00040	2
AX-157926834	3	59.618	KV452730.1	5302572	2-scf00040	2
AX-157926836	3	59.618	KV452730.1	5306084	2-scf00040	2
AX-157926837	3	59.618	KV452730.1	5306740	2-scf00040	2
AX-157926840	3	59.618	KV452730.1	5308556	2-scf00040	2
AX-157926842	3	59.618	KV452730.1	5309019	2-scf00040	2
AX-157926843	3	59.618	KV452730.1	5310582	2-scf00040	2
AX-157926845	3	59.618	KV452730.1	5311601	2-scf00040	2
AX-157926847	3	59.618	KV452730.1	5312271	2-scf00040	2
AX-157926849	3	59.618	KV452730.1	5313046	2-scf00040	2
AX-157926850	3	59.618	KV452730.1	5314002	2-scf00040	2
AX-157926851	3	59.618	KV452730.1	5314593	2-scf00040	2
AX-157926852	3	59.618	KV452730.1	5315994	2-scf00040	2
AX-157926854	3	59.618	KV452730.1	5316407	2-scf00040	2
AX-158389717	3	59.618	KV452730.1	5316644	2-scf00040	2
AX-157926856	3	59.618	KV452730.1	5320061	2-scf00040	2
AX-157926857	3	59.618	KV452730.1	5321016	2-scf00040	2
AX-157813714	3	59.618	KV452730.1	5321371	2-scf00040	2
AX-157926858	3	59.618	KV452730.1	5321715	2-scf00040	2
AX-157926860	3	59.618	KV452730.1	5322381	2-scf00040	2
AX-157926861	3	59.618	KV452730.1	5322891	2-scf00040	2
AX-157926863	3	59.618	KV452730.1	5323457	2-scf00040	2
AX-157926865	3	59.618	KV452730.1	5326914	2-scf00040	2
AX-157926866	3	59.618	KV452730.1	5327948	2-scf00040	2
AX-157926867	3	59.618	KV452730.1	5328584	2-scf00040	2
AX-157926869	3	59.618	KV452730.1	5330221	2-scf00040	2
AX-158389718	3	59.618	KV452730.1	5331597	2-scf00040	2
AX-157926873	3	59.618	KV452730.1	5334210	2-scf00040	2
AX-157926874	3	59.618	KV452730.1	5334999	2-scf00040	2
AX-157926876	3	59.618	KV452730.1	5335499	2-scf00040	2
AX-157926877	3	59.618	KV452730.1	5335802	2-scf00040	2
AX-157926879	3	59.618	KV452730.1	5336337	2-scf00040	2
AX-158389719	3	59.618	KV452730.1	5336536	2-scf00040	2
AX-157926880	3	59.618	KV452730.1	5337867	2-scf00040	2
AX-157926882	3	59.618	KV452730.1	5338649	2-scf00040	2
AX-157926883	3	59.618	KV452730.1	5340563	2-scf00040	2
AX-157926885	3	59.618	KV452730.1	5341248	2-scf00040	2
AX-157926886	3	59.618	KV452730.1	5341831	2-scf00040	2
AX-157926888	3	59.618	KV452730.1	5342676	2-scf00040	2
AX-158389720	3	59.618	KV452730.1	5343728	2-scf00040	2

AX-157926890	3	59.618	KV452730.1	5343823	2-scf00040	2
AX-157926892	3	59.618	KV452730.1	5344548	2-scf00040	2
AX-157926893	3	59.618	KV452730.1	5345895	2-scf00040	2
AX-157926894	3	59.618	KV452730.1	5346807	2-scf00040	2
AX-157926895	3	59.618	KV452730.1	5347276	2-scf00040	2
AX-157926898	3	59.618	KV452730.1	5348113	2-scf00040	2
AX-157926899	3	59.618	KV452730.1	5348730	2-scf00040	2
AX-158405365	3	59.618	KV452730.1	5353887	2-scf00040	2
AX-157926901	3	59.618	KV452730.1	5356144	2-scf00040	2
AX-157926904	3	59.618	KV452730.1	5357973	2-scf00040	2
AX-157926906	3	59.618	KV452730.1	5359170	2-scf00040	2
AX-157926907	3	59.618	KV452730.1	5361026	2-scf00040	2
AX-157926908	3	59.618	KV452730.1	5361655	2-scf00040	2
AX-157926910	3	59.618	KV452730.1	5363390	2-scf00040	2
AX-157926911	3	59.618	KV452730.1	5363704	2-scf00040	2
AX-157926913	3	59.618	KV452730.1	5364628	2-scf00040	2
AX-157926915	3	59.618	KV452730.1	5365142	2-scf00040	2
AX-157926918	3	59.618	KV452730.1	5365709	2-scf00040	2
AX-157926920	3	59.618	KV452730.1	5366390	2-scf00040	2
AX-157926921	3	59.618	KV452730.1	5366805	2-scf00040	2
AX-157926923	3	59.618	KV452730.1	5367319	2-scf00040	2
AX-157926924	3	59.618	KV452730.1	5367819	2-scf00040	2
AX-157926927	3	59.618	KV452730.1	5372711	2-scf00040	2
AX-157926931	3	59.618	KV452730.1	5382159	2-scf00040	2
AX-157926933	3	59.618	KV452730.1	5383976	2-scf00040	2
AX-157926936	3	59.618	KV452730.1	5385343	2-scf00040	2
AX-157926940	3	59.618	KV452730.1	5386227	2-scf00040	2
AX-157926941	3	59.618	KV452730.1	5387131	2-scf00040	2
AX-157926943	3	59.618	KV452730.1	5387833	2-scf00040	2
AX-158405366	3	59.618	KV452730.1	5391411	2-scf00040	2
AX-157926946	3	59.618	KV452730.1	5396858	2-scf00040	2
AX-157926948	3	59.618	KV452730.1	5398325	2-scf00040	2
AX-157926950	3	59.618	KV452730.1	5400668	2-scf00040	2
AX-157926951	3	59.618	KV452730.1	5402289	2-scf00040	2
AX-157926953	3	59.618	KV452730.1	5403208	2-scf00040	2
AX-157926956	3	59.618	KV452730.1	5404185	2-scf00040	2
AX-157926959	3	59.618	KV452730.1	5408276	2-scf00040	2
AX-157926961	3	59.618	KV452730.1	5409810	2-scf00040	2
AX-157926963	3	59.618	KV452730.1	5410254	2-scf00040	2
AX-157926965	3	59.618	KV452730.1	5411263	2-scf00040	2
AX-157926967	3	59.618	KV452730.1	5411737	2-scf00040	2
AX-157926970	3	59.618	KV452730.1	5413849	2-scf00040	2
AX-157926972	3	59.618	KV452730.1	5415140	2-scf00040	2
AX-157926974	3	59.618	KV452730.1	5416895	2-scf00040	2
AX-157926975	3	59.618	KV452730.1	5417397	2-scf00040	2
AX-157926976	3	59.618	KV452730.1	5417905	2-scf00040	2
AX-157926977	3	59.618	KV452730.1	5419340	2-scf00040	2
AX-157813716	3	59.618	KV452730.1	5421542	2-scf00040	2
AX-157926978	3	59.618	KV452730.1	5424331	2-scf00040	2
AX-157926979	3	59.618	KV452730.1	5425824	2-scf00040	2
AX-157926980	3	59.618	KV452730.1	5426342	2-scf00040	2

AX-157926983	3	59.618	KV452730.1	5429069	2-scf00040	2
AX-157926985	3	59.618	KV452730.1	5430066	2-scf00040	2
AX-157926987	3	59.618	KV452730.1	5432650	2-scf00040	2
AX-157926989	3	59.618	KV452730.1	5433362	2-scf00040	2
AX-157721160	3	59.618	KV452730.1	5433632	2-scf00040	2
AX-157926991	3	59.618	KV452730.1	5436602	2-scf00040	2
AX-157926993	3	59.618	KV452730.1	5439562	2-scf00040	2
AX-157926994	3	59.618	KV452730.1	5440503	2-scf00040	2
AX-157926996	3	59.618	KV452730.1	5441934	2-scf00040	2
AX-157926998	3	59.618	KV452730.1	5443785	2-scf00040	2
AX-157926999	3	59.618	KV452730.1	5444300	2-scf00040	2
AX-157869190	3	59.618	KV452730.1	5445041	2-scf00040	2
AX-157927002	3	59.618	KV452730.1	5445480	2-scf00040	2
AX-157710237	3	59.618	KV452730.1	5446046	2-scf00040	2
AX-157927003	3	59.618	KV452730.1	5446286	2-scf00040	2
AX-157927006	3	59.618	KV452730.1	5447742	2-scf00040	2
AX-157927007	3	59.618	KV452730.1	5448725	2-scf00040	2
AX-158405368	3	59.618	KV452730.1	5449390	2-scf00040	2
AX-157927011	3	59.618	KV452730.1	5450403	2-scf00040	2
AX-157927012	3	59.618	KV452730.1	5451078	2-scf00040	2
AX-157927014	3	59.618	KV452730.1	5452590	2-scf00040	2
AX-157718200	3	59.618	KV452730.1	5453079	2-scf00040	2
AX-157927015	3	59.618	KV452730.1	5454940	2-scf00040	2
AX-158405369	3	59.618	KV452730.1	5455196	2-scf00040	2
AX-157927019	3	59.618	KV452730.1	5457821	2-scf00040	2
AX-157927021	3	59.618	KV452730.1	5458355	2-scf00040	2
AX-157927023	3	59.618	KV452730.1	5460906	2-scf00040	2
AX-157813717	3	59.618	KV452730.1	5460946	2-scf00040	2
AX-157813718	3	59.618	KV452730.1	5462260	2-scf00040	2
AX-157927024	3	59.618	KV452730.1	5463424	2-scf00040	2
AX-157927026	3	59.618	KV452730.1	5463847	2-scf00040	2
AX-157927030	3	59.618	KV452730.1	5465382	2-scf00040	2
AX-157927031	3	59.618	KV452730.1	5465741	2-scf00040	2
AX-158389722	3	59.618	KV452730.1	5466014	2-scf00040	2
AX-157927033	3	59.618	KV452730.1	5467433	2-scf00040	2
AX-157927037	3	59.618	KV452730.1	5469827	2-scf00040	2
AX-157927039	3	59.618	KV452730.1	5470454	2-scf00040	2
AX-157927040	3	59.618	KV452730.1	5471060	2-scf00040	2
AX-157927041	3	59.618	KV452730.1	5471588	2-scf00040	2
AX-157927042	3	59.618	KV452730.1	5472295	2-scf00040	2
AX-157927044	3	59.618	KV452730.1	5472768	2-scf00040	2
AX-157927045	3	59.618	KV452730.1	5473921	2-scf00040	2
AX-157927047	3	59.618	KV452730.1	5475156	2-scf00040	2
AX-157927049	3	59.618	KV452730.1	5475615	2-scf00040	2
AX-157869191	3	59.618	KV452730.1	5476717	2-scf00040	2
AX-157813719	3	59.618	KV452730.1	5478167	2-scf00040	2
AX-157813720	3	59.618	KV452730.1	5481798	2-scf00040	2
AX-157927052	3	59.618	KV452730.1	5482830	2-scf00040	2
AX-157927055	3	59.618	KV452730.1	5485491	2-scf00040	2
AX-158404508	3	59.618	KV452730.1	5486191	2-scf00040	2
AX-157927068	3	59.618	KV452730.1	5501063	2-scf00040	2

AX-157813722	3	59.618	KV452730.1	5501717	2-scf00040	2
AX-157927070	3	59.618	KV452730.1	5503895	2-scf00040	2
AX-157927071	3	59.618	KV452730.1	5506365	2-scf00040	2
AX-157927076	3	59.618	KV452730.1	5526558	2-scf00040	2
AX-157927078	3	59.618	KV452730.1	5526949	2-scf00040	2
AX-157927079	3	59.618	KV452730.1	5527622	2-scf00040	2
AX-157927081	3	59.618	KV452730.1	5528100	2-scf00040	2
AX-157723410	3	59.618	KV452730.1	5528895	2-scf00040	2
AX-157927082	3	59.618	KV452730.1	5529720	2-scf00040	2
AX-157927083	3	59.618	KV452730.1	5531361	2-scf00040	2
AX-157927085	3	59.618	KV452730.1	5532600	2-scf00040	2
AX-158389723	3	59.618	KV452730.1	5533042	2-scf00040	2
AX-157927086	3	59.618	KV452730.1	5533275	2-scf00040	2
AX-157927089	3	59.618	KV452730.1	5536491	2-scf00040	2
AX-157927091	3	59.618	KV452730.1	5538013	2-scf00040	2
AX-157927096	3	59.618	KV452730.1	5538916	2-scf00040	2
AX-157927098	3	59.618	KV452730.1	5539375	2-scf00040	2
AX-158405374	3	59.618	KV452730.1	5540837	2-scf00040	2
AX-158404509	3	59.618	KV452730.1	5541105	2-scf00040	2
AX-158405376	3	59.618	KV452730.1	5541146	2-scf00040	2
AX-157927099	3	59.618	KV452730.1	5541313	2-scf00040	2
AX-158389724	3	59.618	KV452730.1	5542346	2-scf00040	2
AX-157927102	3	59.618	KV452730.1	5543025	2-scf00040	2
AX-157927103	3	59.618	KV452730.1	5543494	2-scf00040	2
AX-157927105	3	59.618	KV452730.1	5544056	2-scf00040	2
AX-157927109	3	59.618	KV452730.1	5545820	2-scf00040	2
AX-157927110	3	59.618	KV452730.1	5546310	2-scf00040	2
AX-157927111	3	59.618	KV452730.1	5546758	2-scf00040	2
AX-157927113	3	59.618	KV452730.1	5553329	2-scf00040	2
AX-157927114	3	59.618	KV452730.1	5555024	2-scf00040	2
AX-157927117	3	59.618	KV452730.1	5555325	2-scf00040	2
AX-157927119	3	59.618	KV452730.1	5555636	2-scf00040	2
AX-157927120	3	59.618	KV452730.1	5555858	2-scf00040	2
AX-157813723	3	59.618	KV452730.1	5557984	2-scf00040	2
AX-157927121	3	59.618	KV452730.1	5560079	2-scf00040	2
AX-157927125	3	59.618	KV452730.1	5564157	2-scf00040	2
AX-157927126	3	59.618	KV452730.1	5568098	2-scf00040	2
AX-157927129	3	59.618	KV452730.1	5569540	2-scf00040	2
AX-157927132	3	59.618	KV452730.1	5570778	2-scf00040	2
AX-157813726	3	59.618	KV452730.1	5577134	2-scf00040	2
AX-157813727	3	59.618	KV452730.1	5577501	2-scf00040	2
AX-157927137	3	59.618	KV452730.1	5577901	2-scf00040	2
AX-157927139	3	59.618	KV452730.1	5578376	2-scf00040	2
AX-158389726	3	59.618	KV452730.1	5578507	2-scf00040	2
AX-157927140	3	59.618	KV452730.1	5579365	2-scf00040	2
AX-157927143	3	59.618	KV452730.1	5580389	2-scf00040	2
AX-157927144	3	59.618	KV452730.1	5580970	2-scf00040	2
AX-157927147	3	59.618	KV452730.1	5583089	2-scf00040	2
AX-157927148	3	59.618	KV452730.1	5583522	2-scf00040	2
AX-157869192	3	59.618	KV452730.1	5584903	2-scf00040	2
AX-157927149	3	59.618	KV452730.1	5585129	2-scf00040	2

AX-157813728	3	59.618	KV452730.1	5585446	2-scf00040	2
AX-157927151	3	59.618	KV452730.1	5588490	2-scf00040	2
AX-157927153	3	59.618	KV452730.1	5589463	2-scf00040	2
AX-157927158	3	59.618	KV452730.1	5592862	2-scf00040	2
AX-157927159	3	59.618	KV452730.1	5594170	2-scf00040	2
AX-157927166	3	59.618	KV452730.1	5597681	2-scf00040	2
AX-157927169	3	59.618	KV452730.1	5597936	2-scf00040	2
AX-157927170	3	59.618	KV452730.1	5598271	2-scf00040	2
AX-157927172	3	59.618	KV452730.1	5600062	2-scf00040	2
AX-157927173	3	59.618	KV452730.1	5601694	2-scf00040	2
AX-157718317	3	59.618	KV452730.1	5602071	2-scf00040	2
AX-157927175	3	59.618	KV452730.1	5602706	2-scf00040	2
AX-157927176	3	59.618	KV452730.1	5603429	2-scf00040	2
AX-157927178	3	59.618	KV452730.1	5604811	2-scf00040	2
AX-157813729	3	59.618	KV452730.1	5605981	2-scf00040	2
AX-157927180	3	59.618	KV452730.1	5606508	2-scf00040	2
AX-157927181	3	59.618	KV452730.1	5607718	2-scf00040	2
AX-157869193	3	59.618	KV452730.1	5608705	2-scf00040	2
AX-157927182	3	59.618	KV452730.1	5609613	2-scf00040	2
AX-157927186	3	59.618	KV452730.1	5611027	2-scf00040	2
AX-157927188	3	59.618	KV452730.1	5611961	2-scf00040	2
AX-157927191	3	59.618	KV452730.1	5612572	2-scf00040	2
AX-157927192	3	59.618	KV452730.1	5612878	2-scf00040	2
AX-157745219	3	59.618	KV452730.1	5613305	2-scf00040	2
AX-158405377	3	59.618	KV452730.1	5613507	2-scf00040	2
AX-157927193	3	59.618	KV452730.1	5617008	2-scf00040	2
AX-157927198	3	59.618	KV452730.1	5618845	2-scf00040	2
AX-157927199	3	59.618	KV452730.1	5619380	2-scf00040	2
AX-157754534	3	59.618	KV452730.1	5620570	2-scf00040	2
AX-157927201	3	59.618	KV452730.1	5621298	2-scf00040	2
AX-157927203	3	59.618	KV452730.1	5624059	2-scf00040	2
AX-157927207	3	59.618	KV452730.1	5626255	2-scf00040	2
AX-157927209	3	59.618	KV452730.1	5628597	2-scf00040	2
AX-157927210	3	59.618	KV452730.1	5629033	2-scf00040	2
AX-157927212	3	59.618	KV452730.1	5631192	2-scf00040	2
AX-157927213	3	59.618	KV452730.1	5632321	2-scf00040	2
AX-157927219	3	59.618	KV452730.1	5635337	2-scf00040	2
AX-157927220	3	59.618	KV452730.1	5635777	2-scf00040	2
AX-157927224	3	59.618	KV452730.1	5638164	2-scf00040	2
AX-157927232	3	59.618	KV452730.1	5643687	2-scf00040	2
AX-157927233	3	59.618	KV452730.1	5644155	2-scf00040	2
AX-157927235	3	59.618	KV452730.1	5644410	2-scf00040	2
AX-157927238	3	59.618	KV452730.1	5647021	2-scf00040	2
AX-157813732	3	59.618	KV452730.1	5650478	2-scf00040	2
AX-157927239	3	59.618	KV452730.1	5651518	2-scf00040	2
AX-157927240	3	59.618	KV452730.1	5651898	2-scf00040	2
AX-157927241	3	59.618	KV452730.1	5652421	2-scf00040	2
AX-157927245	3	59.618	KV452730.1	5653361	2-scf00040	2
AX-157927246	3	59.618	KV452730.1	5653882	2-scf00040	2
AX-157927249	3	59.618	KV452730.1	5656627	2-scf00040	2
AX-157813733	3	59.618	KV452730.1	5657118	2-scf00040	2

AX-157752613	3	59.618	KV452730.1	5659917	2-scf00040	2
AX-157927252	3	59.618	KV452730.1	5661832	2-scf00040	2
AX-157927254	3	59.618	KV452730.1	5662819	2-scf00040	2
AX-157927258	3	59.618	KV452730.1	5665257	2-scf00040	2
AX-157927259	3	59.618	KV452730.1	5667959	2-scf00040	2
AX-157927261	3	59.618	KV452730.1	5670708	2-scf00040	2
AX-157813734	3	59.618	KV452730.1	5671001	2-scf00040	2
AX-157927262	3	59.618	KV452730.1	5671510	2-scf00040	2
AX-157927263	3	59.618	KV452730.1	5671597	2-scf00040	2
AX-157927265	3	59.618	KV452730.1	5672131	2-scf00040	2
AX-157927267	3	59.618	KV452730.1	5674145	2-scf00040	2
AX-157927268	3	59.618	KV452730.1	5674764	2-scf00040	2
AX-157927270	3	59.618	KV452730.1	5679579	2-scf00040	2
AX-157927273	3	59.618	KV452730.1	5682019	2-scf00040	2
AX-157927275	3	59.618	KV452730.1	5682782	2-scf00040	2
AX-157927276	3	59.618	KV452730.1	5683370	2-scf00040	2
AX-157927279	3	59.618	KV452730.1	5683789	2-scf00040	2
AX-157927281	3	59.618	KV452730.1	5687587	2-scf00040	2
AX-157927283	3	59.618	KV452730.1	5688755	2-scf00040	2
AX-157927285	3	59.618	KV452730.1	5690359	2-scf00040	2
AX-157927286	3	59.618	KV452730.1	5690788	2-scf00040	2
AX-157927288	3	59.618	KV452730.1	5691276	2-scf00040	2
AX-157927289	3	59.618	KV452730.1	5691846	2-scf00040	2
AX-157927292	3	59.618	KV452730.1	5692442	2-scf00040	2
AX-158389727	3	59.618	KV452730.1	5693123	2-scf00040	2
AX-157927294	3	59.618	KV452730.1	5695388	2-scf00040	2
AX-157927303	3	59.618	KV452730.1	5701416	2-scf00040	2
AX-157927308	3	59.618	KV452730.1	5704017	2-scf00040	2
AX-157813735	3	59.618	KV452730.1	5714969	2-scf00040	2
AX-157927332	3	59.618	KV452730.1	5723006	2-scf00040	2
AX-157927360	3	59.618	KV452730.1	5742055	2-scf00040	2
AX-157753987	3	59.618	KV452730.1	5743756	2-scf00040	2
AX-157927409	3	59.618	KV452730.1	5776849	2-scf00040	2
AX-157813748	3	59.618	KV452730.1	5781642	2-scf00040	2
AX-157927412	3	59.618	KV452730.1	5783065	2-scf00040	2
AX-157813750	3	59.618	KV452730.1	5785361	2-scf00040	2
AX-158405383	3	59.618	KV452730.1	5785426	2-scf00040	2
AX-157927423	3	59.618	KV452730.1	5793744	2-scf00040	2
AX-157927425	3	59.618	KV452730.1	5795465	2-scf00040	2
AX-157927428	3	59.618	KV452730.1	5796987	2-scf00040	2
AX-157927432	3	59.618	KV452730.1	5800430	2-scf00040	2
AX-158405385	3	59.618	KV452730.1	5803013	2-scf00040	2
AX-158405386	3	59.618	KV452730.1	5803110	2-scf00040	2
AX-158404516	3	59.618	KV452730.1	5803298	2-scf00040	2
AX-157724521	3	59.618	KV452730.1	5810595	2-scf00040	2
AX-157927439	3	59.618	KV452730.1	5817345	2-scf00040	2
AX-157927453	3	59.618	KV452730.1	5835514	2-scf00040	2
AX-157927454	3	59.618	KV452730.1	5838269	2-scf00040	2
AX-157813764	3	59.618	KV452730.1	5844882	2-scf00040	2
AX-158379334	3	59.618	KV452730.1	5845441	2-scf00040	2
AX-157813765	3	59.618	KV452730.1	5848600	2-scf00040	2

AX-157927463	3	59.618	KV452730.1	5849276	2-scf00040	2
AX-157927468	3	59.618	KV452730.1	5854927	2-scf00040	2
AX-157813766	3	59.618	KV452730.1	5855517	2-scf00040	2
AX-157927472	3	59.618	KV452730.1	5859906	2-scf00040	2
AX-157927473	3	59.618	KV452730.1	5860786	2-scf00040	2
AX-157927475	3	59.618	KV452730.1	5867175	2-scf00040	2
AX-157927476	3	59.618	KV452730.1	5867595	2-scf00040	2
AX-157927477	3	59.618	KV452730.1	5871532	2-scf00040	2
AX-157927479	3	59.618	KV452730.1	5873152	2-scf00040	2
AX-157927483	3	59.618	KV452730.1	5878524	2-scf00040	2
AX-157927485	3	59.618	KV452730.1	5879585	2-scf00040	2
AX-157927487	3	59.618	KV452730.1	5880752	2-scf00040	2
AX-157927490	3	59.618	KV452730.1	5883365	2-scf00040	2
AX-157927493	3	59.618	KV452730.1	5887282	2-scf00040	2
AX-157853878	3	59.618	KV452730.1	5888673	2-scf00040	2
AX-157927497	3	59.618	KV452730.1	5897550	2-scf00040	2
AX-157927498	3	59.618	KV452730.1	5898293	2-scf00040	2
AX-157927507	3	59.618	KV452730.1	5907260	2-scf00040	2
AX-157927508	3	59.618	KV452730.1	5908308	2-scf00040	2
AX-157773098	3	59.618	KV452730.1	5912195	2-scf00040	2
AX-157927509	3	59.618	KV452730.1	5914429	2-scf00040	2
AX-157927510	3	59.618	KV452730.1	5915358	2-scf00040	2
AX-157927511	3	59.618	KV452730.1	5916241	2-scf00040	2
AX-157927512	3	59.618	KV452730.1	5919291	2-scf00040	2
AX-157927516	3	59.618	KV452730.1	5923961	2-scf00040	2
AX-157718798	3	59.618	KV452730.1	5924877	2-scf00040	2
AX-157927518	3	59.618	KV452730.1	5925697	2-scf00040	2
AX-157927519	3	59.618	KV452730.1	5926550	2-scf00040	2
AX-157927522	3	59.618	KV452730.1	5931400	2-scf00040	2
AX-157722825	3	59.618	KV452730.1	5934012	2-scf00040	2
AX-157927528	3	59.618	KV452730.1	5936892	2-scf00040	2
AX-157927539	3	59.618	KV452730.1	5946632	2-scf00040	2
AX-157927541	3	59.618	KV452730.1	5947406	2-scf00040	2
AX-157927542	3	59.618	KV452730.1	5947984	2-scf00040	2
AX-157927543	3	59.618	KV452730.1	5951360	2-scf00040	2
AX-157927548	3	59.618	KV452730.1	5953991	2-scf00040	2
AX-157927549	3	59.618	KV452730.1	5954547	2-scf00040	2
AX-157927551	3	59.618	KV452730.1	5954753	2-scf00040	2
AX-157752554	3	59.618	KV452730.1	5961515	2-scf00040	2
AX-157927565	3	59.618	KV452730.1	5965025	2-scf00040	2
AX-157927567	3	59.618	KV452730.1	5966403	2-scf00040	2
AX-157927568	3	59.618	KV452730.1	5968490	2-scf00040	2
AX-157927573	3	59.618	KV452730.1	5972284	2-scf00040	2
AX-157927576	3	59.618	KV452730.1	5974968	2-scf00040	2
AX-157927577	3	59.618	KV452730.1	5975350	2-scf00040	2
AX-157813774	3	59.618	KV452730.1	5982452	2-scf00040	2
AX-157927584	3	59.618	KV452730.1	5983023	2-scf00040	2
AX-157927594	3	59.618	KV452730.1	5991975	2-scf00040	2
AX-157754867	3	59.618	KV452730.1	5992483	2-scf00040	2
AX-157927599	3	59.618	KV452730.1	5993608	2-scf00040	2
AX-157775759	3	59.618	KV452730.1	5994810	2-scf00040	2

AX-157927601	3	59.618	KV452730.1	5995250	2-scf00040	2
AX-157927603	3	59.618	KV452730.1	5995967	2-scf00040	2
AX-157927606	3	59.618	KV452730.1	5997385	2-scf00040	2
AX-157927607	3	59.618	KV452730.1	5998168	2-scf00040	2
AX-157927608	3	59.618	KV452730.1	5998931	2-scf00040	2
AX-157927609	3	59.618	KV452730.1	6000092	2-scf00040	2
AX-157927618	3	59.618	KV452730.1	6005750	2-scf00040	2
AX-157927621	3	59.618	KV452730.1	6010375	2-scf00040	2
AX-157927624	3	59.618	KV452730.1	6015915	2-scf00040	2
AX-157927627	3	59.618	KV452730.1	6017147	2-scf00040	2
AX-157927630	3	59.618	KV452730.1	6020676	2-scf00040	2
AX-157927633	3	59.618	KV452730.1	6023698	2-scf00040	2
AX-157927636	3	59.618	KV452730.1	6026274	2-scf00040	2
AX-157927639	3	59.618	KV452730.1	6027901	2-scf00040	2
AX-157927642	3	59.618	KV452730.1	6029373	2-scf00040	2
AX-158389732	3	59.618	KV452730.1	6029934	2-scf00040	2
AX-157927643	3	59.618	KV452730.1	6031700	2-scf00040	2
AX-157927645	3	59.618	KV452730.1	6032023	2-scf00040	2
AX-157927649	3	59.618	KV452730.1	6033518	2-scf00040	2
AX-157927652	3	59.618	KV452730.1	6035590	2-scf00040	2
AX-157927653	3	59.618	KV452730.1	6036178	2-scf00040	2
AX-157927655	3	59.618	KV452730.1	6039881	2-scf00040	2
AX-157927658	3	59.618	KV452730.1	6041216	2-scf00040	2
AX-157927660	3	59.618	KV452730.1	6042930	2-scf00040	2
AX-157927661	3	59.618	KV452730.1	6043590	2-scf00040	2
AX-157927665	3	59.618	KV452730.1	6046872	2-scf00040	2
AX-157927666	3	59.618	KV452730.1	6047274	2-scf00040	2
AX-157927672	3	59.618	KV452730.1	6055022	2-scf00040	2
AX-157927676	3	59.618	KV452730.1	6056826	2-scf00040	2
AX-157927685	3	59.618	KV452730.1	6059988	2-scf00040	2
AX-157717747	3	59.618	KV452730.1	6060788	2-scf00040	2
AX-158379335	3	59.618	KV452730.1	6070271	2-scf00040	2
AX-157927693	3	59.618	KV452730.1	6071839	2-scf00040	2
AX-157927695	3	59.618	KV452730.1	6072270	2-scf00040	2
AX-157927703	3	59.618	KV452730.1	6074999	2-scf00040	2
AX-157927709	3	59.618	KV452730.1	6078674	2-scf00040	2
AX-157740527	3	59.618	KV452730.1	6079198	2-scf00040	2
AX-157927710	3	59.618	KV452730.1	6079705	2-scf00040	2
AX-157927715	3	59.618	KV452730.1	6086205	2-scf00040	2
AX-157742457	3	59.618	KV452730.1	6087439	2-scf00040	2
AX-157927723	3	59.618	KV452730.1	6090868	2-scf00040	2
AX-157813777	3	59.618	KV452730.1	6094049	2-scf00040	2
AX-157927725	3	59.618	KV452730.1	6094302	2-scf00040	2
AX-157727825	3	59.618	KV452730.1	6101825	2-scf00040	2
AX-157927735	3	59.618	KV452730.1	6102607	2-scf00040	2
AX-157773365	3	59.618	KV452730.1	6104551	2-scf00040	2
AX-157927740	3	59.618	KV452730.1	6106441	2-scf00040	2
AX-157927745	3	59.618	KV452730.1	6109891	2-scf00040	2
AX-157927746	3	59.618	KV452730.1	6110705	2-scf00040	2
AX-157927749	3	59.618	KV452730.1	6112317	2-scf00040	2
AX-157927752	3	59.618	KV452730.1	6114485	2-scf00040	2

AX-157927758	3	59.618	KV452730.1	6116628	2-scf00040	2
AX-157927761	3	59.618	KV452730.1	6118419	2-scf00040	2
AX-157927767	3	59.618	KV452730.1	6123403	2-scf00040	2
AX-157927769	3	59.618	KV452730.1	6124524	2-scf00040	2
AX-157927770	3	59.618	KV452730.1	6124998	2-scf00040	2
AX-157726982	3	59.618	KV452730.1	6129298	2-scf00040	2
AX-157927781	3	59.618	KV452730.1	6139185	2-scf00040	2
AX-157927783	3	59.618	KV452730.1	6140360	2-scf00040	2
AX-157927786	3	59.618	KV452730.1	6142294	2-scf00040	2
AX-157927818	3	59.618	KV452730.1	6172663	2-scf00040	2
AX-157928182	3	59.618	KV452730.1	6566368	2-scf00040	2
AX-157700539	3	59.618	FI862025.1	375	HORI212 Ictalu	UN
AX-157695484	3	59.618	FI884222.1	51	HORI212 Ictalu	UN
AX-157706473	3	59.618	3ML01032305	212	scf07921_1	UN
AX-157706474	3	59.618	3ML01032305	484	scf07921_1	UN
AX-157707005	3	59.618	3ML01033325	265	scf08971_1	UN
AX-157707494	3	59.618	3ML01034396	346	scf10070_1	UN
AX-157925900	3	59.836	KV452730.1	4400650	2-scf00040	2
AX-157926279	3	59.836	KV452730.1	4808342	2-scf00040	2
AX-157927272	3	59.836	KV452730.1	5680302	2-scf00040	2
AX-157927317	3	59.836	KV452730.1	5709574	2-scf00040	2
AX-157927503	3	59.836	KV452730.1	5901785	2-scf00040	2
AX-157927611	3	59.836	KV452730.1	6001141	2-scf00040	2
AX-157927775	3	59.836	KV452730.1	6131064	2-scf00040	2
AX-157773841	3	59.836	KV452730.1	6137217	2-scf00040	2
AX-157927779	3	59.836	KV452730.1	6138532	2-scf00040	2
AX-157741031	3	59.836	KV452730.1	6142628	2-scf00040	2
AX-157927788	3	59.836	KV452730.1	6144363	2-scf00040	2
AX-157927795	3	59.836	KV452730.1	6150346	2-scf00040	2
AX-157927799	3	59.836	KV452730.1	6156187	2-scf00040	2
AX-157927846	3	59.836	KV452730.1	6203304	2-scf00040	2
AX-157927849	3	59.836	KV452730.1	6205217	2-scf00040	2
AX-157927878	3	59.836	KV452730.1	6231281	2-scf00040	2
AX-157756115	3	59.836	KV452730.1	6237767	2-scf00040	2
AX-157927886	3	59.836	KV452730.1	6247261	2-scf00040	2
AX-157927887	3	59.836	KV452730.1	6247753	2-scf00040	2
AX-157927941	3	59.836	KV452730.1	6291523	2-scf00040	2
AX-157927942	3	59.836	KV452730.1	6292763	2-scf00040	2
AX-157927954	3	59.836	KV452730.1	6313210	2-scf00040	2
AX-158379337	3	59.836	KV452730.1	6337437	2-scf00040	2
AX-157928001	3	59.836	KV452730.1	6352538	2-scf00040	2
AX-157928002	3	59.836	KV452730.1	6353662	2-scf00040	2
AX-157928043	3	59.836	KV452730.1	6387679	2-scf00040	2
AX-157928052	3	59.836	KV452730.1	6396273	2-scf00040	2
AX-157928076	3	59.836	KV452730.1	6414508	2-scf00040	2
AX-157928106	3	59.836	KV452730.1	6460040	2-scf00040	2
AX-157928219	3	59.836	KV452730.1	6637286	2-scf00040	2
AX-157928271	3	59.836	KV452730.1	6731409	2-scf00040	2
AX-157883650	3	59.836	KV452730.1	6769793	2-scf00040	2
AX-157928507	3	59.836	KV452730.1	7057840	2-scf00040	2
AX-157928526	3	59.836	KV452730.1	7088050	2-scf00040	2

AX-157928696	3	59.836	KV452730.1	7349726	2-scf00040	2
AX-157928774	3	59.836	KV452730.1	7502660	2-scf00040	2
AX-157928835	3	59.836	KV452730.1	7655867	2-scf00040	2
AX-157813847	3	59.836	KV452730.1	7706174	2-scf00040	2
AX-157928918	3	59.836	KV452730.1	7792648	2-scf00040	2
AX-157813885	3	59.836	KV452730.1	8227444	2-scf00040	2
AX-157929325	3	59.836	KV452730.1	8389524	2-scf00040	2
AX-157813905	3	59.836	KV452730.1	8899308	2-scf00040	2
AX-157927790	3	59.858	KV452730.1	6145614	2-scf00040	2
AX-157927796	3	59.878	KV452730.1	6151417	2-scf00040	2
AX-157927798	3	59.899	KV452730.1	6155262	2-scf00040	2
AX-157927817	3	59.899	KV452730.1	6170909	2-scf00040	2
AX-157739773	3	59.922	KV452730.1	6165305	2-scf00040	2
AX-157927811	3	59.946	KV452730.1	6168173	2-scf00040	2
AX-157927812	3	59.946	KV452730.1	6168316	2-scf00040	2
AX-157927816	3	59.946	KV452730.1	6169561	2-scf00040	2
AX-157927964	3	59.946	KV452730.1	6320162	2-scf00040	2
AX-157928086	3	59.946	KV452730.1	6433309	2-scf00040	2
AX-157757163	3	59.946	KV452730.1	6557269	2-scf00040	2
AX-157928187	3	59.946	KV452730.1	6578030	2-scf00040	2
AX-157928334	3	59.946	KV452730.1	6825258	2-scf00040	2
AX-157928501	3	59.946	KV452730.1	7050132	2-scf00040	2
AX-157813833	3	59.946	KV452730.1	7082878	2-scf00040	2
AX-157928756	3	59.946	KV452730.1	7465966	2-scf00040	2
AX-157928762	3	59.946	KV452730.1	7472389	2-scf00040	2
AX-158405429	3	59.946	KV452730.1	7481726	2-scf00040	2
AX-157928825	3	59.946	KV452730.1	7632225	2-scf00040	2
AX-157928924	3	59.946	KV452730.1	7798464	2-scf00040	2
AX-157928935	3	59.946	KV452730.1	7819668	2-scf00040	2
AX-157928945	3	59.946	KV452730.1	7832834	2-scf00040	2
AX-157927825	3	60.02	KV452730.1	6180907	2-scf00040	2
AX-157927828	3	60.092	KV452730.1	6181958	2-scf00040	2
AX-157927838	3	60.157	KV452730.1	6190841	2-scf00040	2
AX-157755182	3	60.201	KV452730.1	6187566	2-scf00040	2
AX-157927842	3	60.201	KV452730.1	6195036	2-scf00040	2
AX-157739777	3	60.201	KV452730.1	6199057	2-scf00040	2
AX-157745679	3	60.201	KV452730.1	6200931	2-scf00040	2
AX-157927854	3	60.705	KV452730.1	6207167	2-scf00040	2
AX-157927858	3	60.712	KV452730.1	6209709	2-scf00040	2
AX-157928030	3	60.712	KV452730.1	6378967	2-scf00040	2
AX-157928525	3	60.712	KV452730.1	7087455	2-scf00040	2
AX-157928543	3	60.712	KV452730.1	7113985	2-scf00040	2
AX-157927859	3	60.734	KV452730.1	6212143	2-scf00040	2
AX-157927864	3	60.783	KV452730.1	6216698	2-scf00040	2
AX-157927868	3	60.839	KV452730.1	6219327	2-scf00040	2
AX-157927870	3	60.839	KV452730.1	6220567	2-scf00040	2
AX-157722219	3	61.146	KV452730.1	6223503	2-scf00040	2
AX-157927879	3	61.477	KV452730.1	6234166	2-scf00040	2
AX-157927881	3	61.477	KV452730.1	6234675	2-scf00040	2
AX-157927882	3	61.477	KV452730.1	6237080	2-scf00040	2
AX-157741118	3	61.477	KV452730.1	6240184	2-scf00040	2

AX-157813780	3	62.132	KV452730.1	6254676	2-scf00040	2
AX-157927894	3	62.132	KV452730.1	6256440	2-scf00040	2
AX-157927895	3	62.132	KV452730.1	6257138	2-scf00040	2
AX-157927896	3	62.132	KV452730.1	6257548	2-scf00040	2
AX-157927899	3	62.132	KV452730.1	6260667	2-scf00040	2
AX-157927904	3	62.132	KV452730.1	6264246	2-scf00040	2
AX-157927906	3	62.132	KV452730.1	6264603	2-scf00040	2
AX-157927918	3	62.132	KV452730.1	6270842	2-scf00040	2
AX-157813781	3	62.132	KV452730.1	6275493	2-scf00040	2
AX-157927934	3	62.132	KV452730.1	6287247	2-scf00040	2
AX-157927927	3	62.394	KV452730.1	6278726	2-scf00040	2
AX-157717653	3	62.504	KV452730.1	6283539	2-scf00040	2
AX-157927937	3	62.505	KV452730.1	6288278	2-scf00040	2
AX-157927939	3	62.511	KV452730.1	6290417	2-scf00040	2
AX-157927940	3	62.518	KV452730.1	6291015	2-scf00040	2
AX-157927930	3	63.411	KV452730.1	6279314	2-scf00040	2
AX-157927956	3	63.411	KV452730.1	6313731	2-scf00040	2
AX-157813859	3	63.411	KV452730.1	7853378	2-scf00040	2
AX-157928967	3	63.411	KV452730.1	7858067	2-scf00040	2
AX-157929244	3	63.411	KV452730.1	8270413	2-scf00040	2
AX-157929263	3	63.411	KV452730.1	8294103	2-scf00040	2
AX-157742741	3	63.411	KV452730.1	8877828	2-scf00040	2
AX-157927965	3	63.412	KV452730.1	6321955	2-scf00040	2
AX-157927979	3	63.493	KV452730.1	6333329	2-scf00040	2
AX-157927980	3	63.493	KV452730.1	6334177	2-scf00040	2
AX-157927984	3	63.588	KV452730.1	6336598	2-scf00040	2
AX-157927988	3	63.588	KV452730.1	6340369	2-scf00040	2
AX-157927990	3	63.588	KV452730.1	6341288	2-scf00040	2
AX-157927993	3	64.098	KV452730.1	6346811	2-scf00040	2
AX-157927997	3	64.098	KV452730.1	6348902	2-scf00040	2
AX-157927994	3	64.099	KV452730.1	6347237	2-scf00040	2
AX-157927998	3	64.099	KV452730.1	6350052	2-scf00040	2
AX-157927999	3	64.099	KV452730.1	6351175	2-scf00040	2
AX-157928003	3	64.099	KV452730.1	6354719	2-scf00040	2
AX-157928004	3	64.099	KV452730.1	6355911	2-scf00040	2
AX-157928005	3	64.099	KV452730.1	6356673	2-scf00040	2
AX-157928007	3	64.099	KV452730.1	6357144	2-scf00040	2
AX-157928026	3	64.168	KV452730.1	6370873	2-scf00040	2
AX-157928027	3	64.194	KV452730.1	6375284	2-scf00040	2
AX-157928044	3	64.194	KV452730.1	6390023	2-scf00040	2
AX-157928032	3	64.245	KV452730.1	6379631	2-scf00040	2
AX-157928038	3	64.295	KV452730.1	6385776	2-scf00040	2
AX-157928041	3	64.446	KV452730.1	6386588	2-scf00040	2
AX-157928054	3	64.446	KV452730.1	6396642	2-scf00040	2
AX-157928075	3	64.446	KV452730.1	6413872	2-scf00040	2
AX-157928024	3	64.537	KV452730.1	6368145	2-scf00040	2
AX-157928035	3	64.537	KV452730.1	6381468	2-scf00040	2
AX-157928046	3	64.537	KV452730.1	6393399	2-scf00040	2
AX-157928050	3	64.537	KV452730.1	6394663	2-scf00040	2
AX-157928055	3	64.537	KV452730.1	6397783	2-scf00040	2
AX-157928057	3	64.537	KV452730.1	6400940	2-scf00040	2

AX-157928058	3	64.537	KV452730.1	6401378	2-scf00040	2
AX-157928062	3	64.537	KV452730.1	6404915	2-scf00040	2
AX-157928064	3	64.537	KV452730.1	6405645	2-scf00040	2
AX-157928077	3	64.537	KV452730.1	6416949	2-scf00040	2
AX-157928082	3	64.537	KV452730.1	6422850	2-scf00040	2
AX-157813791	3	64.537	KV452730.1	6426744	2-scf00040	2
AX-157928084	3	64.537	KV452730.1	6429129	2-scf00040	2
AX-157928087	3	64.537	KV452730.1	6435227	2-scf00040	2
AX-157928089	3	64.537	KV452730.1	6435615	2-scf00040	2
AX-157928090	3	64.537	KV452730.1	6436272	2-scf00040	2
AX-157813793	3	64.537	KV452730.1	6436500	2-scf00040	2
AX-158405400	3	64.537	KV452730.1	6437227	2-scf00040	2
AX-157928098	3	64.537	KV452730.1	6443379	2-scf00040	2
AX-157928100	3	64.537	KV452730.1	6452074	2-scf00040	2
AX-157724669	3	64.537	KV452730.1	6454990	2-scf00040	2
AX-157928103	3	64.537	KV452730.1	6456817	2-scf00040	2
AX-157928104	3	64.537	KV452730.1	6458029	2-scf00040	2
AX-157862685	3	64.537	KV452730.1	6458907	2-scf00040	2
AX-157928105	3	64.537	KV452730.1	6458982	2-scf00040	2
AX-157928107	3	64.537	KV452730.1	6462145	2-scf00040	2
AX-157928108	3	64.537	KV452730.1	6463144	2-scf00040	2
AX-157928112	3	64.537	KV452730.1	6466519	2-scf00040	2
AX-157928113	3	64.537	KV452730.1	6466896	2-scf00040	2
AX-157928115	3	64.537	KV452730.1	6467219	2-scf00040	2
AX-157928117	3	64.537	KV452730.1	6467722	2-scf00040	2
AX-157928122	3	64.537	KV452730.1	6478548	2-scf00040	2
AX-157813796	3	64.537	KV452730.1	6478849	2-scf00040	2
AX-158421949	3	64.537	KV452730.1	6648070	2-scf00040	2
AX-157928235	3	64.537	KV452730.1	6649862	2-scf00040	2
AX-157928255	3	64.537	KV452730.1	6674540	2-scf00040	2
AX-157928266	3	64.537	KV452730.1	6705292	2-scf00040	2
AX-157928769	3	64.537	KV452730.1	7490896	2-scf00040	2
AX-157928814	3	64.537	KV452730.1	7613387	2-scf00040	2
AX-157929088	3	64.537	KV452730.1	7989372	2-scf00040	2
AX-157706732	3	64.537	3ML01032815	277	scf08445_1	UN
AX-157928125	3	64.574	KV452730.1	6483991	2-scf00040	2
AX-157928129	3	64.601	KV452730.1	6493789	2-scf00040	2
AX-157813808	3	64.601	KV452730.1	6593242	2-scf00040	2
AX-157813789	3	64.646	KV452730.1	6388666	2-scf00040	2
AX-157813794	3	64.646	KV452730.1	6443629	2-scf00040	2
AX-157928131	3	64.646	KV452730.1	6494613	2-scf00040	2
AX-157928133	3	64.646	KV452730.1	6494985	2-scf00040	2
AX-157928134	3	64.646	KV452730.1	6498514	2-scf00040	2
AX-157853880	3	64.646	KV452730.1	6498650	2-scf00040	2
AX-157813798	3	64.646	KV452730.1	6501041	2-scf00040	2
AX-157928144	3	64.646	KV452730.1	6515114	2-scf00040	2
AX-157813799	3	64.646	KV452730.1	6516983	2-scf00040	2
AX-157813800	3	64.646	KV452730.1	6517194	2-scf00040	2
AX-157928148	3	64.646	KV452730.1	6517335	2-scf00040	2
AX-157813801	3	64.646	KV452730.1	6519410	2-scf00040	2
AX-157928152	3	64.646	KV452730.1	6520971	2-scf00040	2

AX-157813803	3	64.646	KV452730.1	6522719	2-scf00040	2
AX-157928159	3	64.646	KV452730.1	6526075	2-scf00040	2
AX-158405402	3	64.646	KV452730.1	6535589	2-scf00040	2
AX-158405404	3	64.646	KV452730.1	6535979	2-scf00040	2
AX-157928176	3	64.646	KV452730.1	6555345	2-scf00040	2
AX-157813806	3	64.646	KV452730.1	6557355	2-scf00040	2
AX-157928190	3	64.646	KV452730.1	6582949	2-scf00040	2
AX-157928194	3	64.646	KV452730.1	6593664	2-scf00040	2
AX-157813810	3	64.646	KV452730.1	6607828	2-scf00040	2
AX-157702684	3	64.646	KV452730.1	6731254	2-scf00040	2
AX-157928382	3	64.646	KV452730.1	6892280	2-scf00040	2
AX-157813826	3	64.646	KV452730.1	6943550	2-scf00040	2
AX-157927943	3	64.755	KV452730.1	6295012	2-scf00040	2
AX-157927972	3	64.755	KV452730.1	6327083	2-scf00040	2
AX-157928137	3	64.755	KV452730.1	6502639	2-scf00040	2
AX-157928179	3	64.755	KV452730.1	6564325	2-scf00040	2
AX-158405406	3	64.755	KV452730.1	6609235	2-scf00040	2
AX-157928204	3	64.755	KV452730.1	6609957	2-scf00040	2
AX-157928205	3	64.755	KV452730.1	6610578	2-scf00040	2
AX-157813811	3	64.755	KV452730.1	6612155	2-scf00040	2
AX-157928213	3	64.755	KV452730.1	6626344	2-scf00040	2
AX-157928248	3	64.755	KV452730.1	6662054	2-scf00040	2
AX-157928249	3	64.755	KV452730.1	6666171	2-scf00040	2
AX-157928254	3	64.755	KV452730.1	6672324	2-scf00040	2
AX-157928257	3	64.755	KV452730.1	6675880	2-scf00040	2
AX-157928263	3	64.755	KV452730.1	6703407	2-scf00040	2
AX-157928268	3	64.755	KV452730.1	6708765	2-scf00040	2
AX-157717223	3	64.755	KV452730.1	6709360	2-scf00040	2
AX-157928289	3	64.755	KV452730.1	6751653	2-scf00040	2
AX-157928361	3	64.755	KV452730.1	6867357	2-scf00040	2
AX-157928444	3	64.755	KV452730.1	6975131	2-scf00040	2
AX-157928470	3	64.755	KV452730.1	7012127	2-scf00040	2
AX-157928476	3	64.755	KV452730.1	7019292	2-scf00040	2
AX-157928504	3	64.755	KV452730.1	7055907	2-scf00040	2
AX-157813834	3	64.755	KV452730.1	7090419	2-scf00040	2
AX-157928548	3	64.755	KV452730.1	7119998	2-scf00040	2
AX-157928555	3	64.755	KV452730.1	7127303	2-scf00040	2
AX-157928590	3	64.755	KV452730.1	7176215	2-scf00040	2
AX-157928656	3	64.755	KV452730.1	7263365	2-scf00040	2
AX-157813841	3	64.755	KV452730.1	7270095	2-scf00040	2
AX-157928727	3	64.755	KV452730.1	7412432	2-scf00040	2
AX-157928732	3	64.755	KV452730.1	7418932	2-scf00040	2
AX-157928734	3	64.755	KV452730.1	7427422	2-scf00040	2
AX-157928771	3	64.755	KV452730.1	7499100	2-scf00040	2
AX-157928773	3	64.755	KV452730.1	7501661	2-scf00040	2
AX-158405431	3	64.755	KV452730.1	7517573	2-scf00040	2
AX-157928784	3	64.755	KV452730.1	7545722	2-scf00040	2
AX-157928801	3	64.755	KV452730.1	7572687	2-scf00040	2
AX-157928812	3	64.755	KV452730.1	7608516	2-scf00040	2
AX-157928816	3	64.755	KV452730.1	7619054	2-scf00040	2
AX-157928817	3	64.755	KV452730.1	7620917	2-scf00040	2

AX-157928820	3	64.755	KV452730.1	7623912	2-scf00040	2
AX-157928822	3	64.755	KV452730.1	7624589	2-scf00040	2
AX-157928845	3	64.755	KV452730.1	7688629	2-scf00040	2
AX-157928847	3	64.755	KV452730.1	7693080	2-scf00040	2
AX-157928880	3	64.755	KV452730.1	7747801	2-scf00040	2
AX-157928883	3	64.755	KV452730.1	7750339	2-scf00040	2
AX-157928300	3	64.756	KV452730.1	6766410	2-scf00040	2
AX-157928466	3	64.756	KV452730.1	7006664	2-scf00040	2
AX-157928302	3	64.865	KV452730.1	6767368	2-scf00040	2
AX-157928560	3	64.865	KV452730.1	7135300	2-scf00040	2
AX-157928304	3	64.919	KV452730.1	6771465	2-scf00040	2
AX-157928483	3	64.919	KV452730.1	7022548	2-scf00040	2
AX-157928224	3	64.975	KV452730.1	6638614	2-scf00040	2
AX-157928229	3	64.975	KV452730.1	6642164	2-scf00040	2
AX-157928244	3	64.975	KV452730.1	6660407	2-scf00040	2
AX-157928298	3	64.975	KV452730.1	6765511	2-scf00040	2
AX-157928318	3	64.975	KV452730.1	6805729	2-scf00040	2
AX-157928321	3	64.975	KV452730.1	6808182	2-scf00040	2
AX-157928340	3	64.975	KV452730.1	6842167	2-scf00040	2
AX-157928457	3	64.975	KV452730.1	6995700	2-scf00040	2
AX-157928493	3	64.975	KV452730.1	7030349	2-scf00040	2
AX-157813837	3	64.975	KV452730.1	7102480	2-scf00040	2
AX-157928561	3	64.975	KV452730.1	7136360	2-scf00040	2
AX-157928861	3	64.975	KV452730.1	7712160	2-scf00040	2
AX-157928864	3	64.975	KV452730.1	7717601	2-scf00040	2
AX-157929099	3	64.975	KV452730.1	8011334	2-scf00040	2
AX-157772339	3	64.975	KV452730.1	8040308	2-scf00040	2
AX-157929131	3	64.975	KV452730.1	8042603	2-scf00040	2
AX-157813871	3	64.975	KV452730.1	8055800	2-scf00040	2
AX-157929142	3	64.975	KV452730.1	8060462	2-scf00040	2
AX-157853890	3	64.975	KV452730.1	8062205	2-scf00040	2
AX-157929144	3	64.975	KV452730.1	8064557	2-scf00040	2
AX-157929148	3	64.975	KV452730.1	8076171	2-scf00040	2
AX-157853891	3	64.975	KV452730.1	8077177	2-scf00040	2
AX-157929149	3	64.975	KV452730.1	8077861	2-scf00040	2
AX-157813875	3	64.975	KV452730.1	8104456	2-scf00040	2
AX-157929158	3	64.975	KV452730.1	8107715	2-scf00040	2
AX-157929159	3	64.975	KV452730.1	8110286	2-scf00040	2
AX-157853893	3	64.975	KV452730.1	8127805	2-scf00040	2
AX-157753157	3	64.975	KV452730.1	8139319	2-scf00040	2
AX-157929181	3	64.975	KV452730.1	8140589	2-scf00040	2
AX-157929185	3	64.975	KV452730.1	8147747	2-scf00040	2
AX-157929188	3	64.975	KV452730.1	8152713	2-scf00040	2
AX-157929189	3	64.975	KV452730.1	8153138	2-scf00040	2
AX-157929295	3	64.975	KV452730.1	8345730	2-scf00040	2
AX-157929297	3	64.975	KV452730.1	8346216	2-scf00040	2
AX-157813893	3	64.975	KV452730.1	8384250	2-scf00040	2
AX-157929627	3	64.975	KV452730.1	8855965	2-scf00040	2
AX-158405471	3	64.975	KV452730.1	8967435	2-scf00040	2
AX-157813920	3	64.975	KV452730.1	9053471	2-scf00040	2
AX-157928325	3	65.022	KV452730.1	6817677	2-scf00040	2

AX-157928022	3	65.083	KV452730.1	6366608	2-scf00040	2
AX-157874842	3	65.083	KV452730.1	6449015	2-scf00040	2
AX-157928161	3	65.083	KV452730.1	6526760	2-scf00040	2
AX-157813804	3	65.083	KV452730.1	6527077	2-scf00040	2
AX-157928189	3	65.083	KV452730.1	6581924	2-scf00040	2
AX-157928232	3	65.083	KV452730.1	6648116	2-scf00040	2
AX-157928238	3	65.083	KV452730.1	6651998	2-scf00040	2
AX-157928240	3	65.083	KV452730.1	6653579	2-scf00040	2
AX-158405411	3	65.083	KV452730.1	6655093	2-scf00040	2
AX-157744007	3	65.083	KV452730.1	6668603	2-scf00040	2
AX-157928258	3	65.083	KV452730.1	6676848	2-scf00040	2
AX-157813818	3	65.083	KV452730.1	6703753	2-scf00040	2
AX-157928277	3	65.083	KV452730.1	6736670	2-scf00040	2
AX-157928278	3	65.083	KV452730.1	6737023	2-scf00040	2
AX-157928279	3	65.083	KV452730.1	6743054	2-scf00040	2
AX-157883651	3	65.083	KV452730.1	6818830	2-scf00040	2
AX-157928326	3	65.083	KV452730.1	6820424	2-scf00040	2
AX-157928341	3	65.083	KV452730.1	6842718	2-scf00040	2
AX-157928347	3	65.083	KV452730.1	6853056	2-scf00040	2
AX-157928349	3	65.083	KV452730.1	6854412	2-scf00040	2
AX-157928383	3	65.083	KV452730.1	6896153	2-scf00040	2
AX-157813824	3	65.083	KV452730.1	6898693	2-scf00040	2
AX-157763927	3	65.083	KV452730.1	6898956	2-scf00040	2
AX-157928396	3	65.083	KV452730.1	6912389	2-scf00040	2
AX-157928401	3	65.083	KV452730.1	6919621	2-scf00040	2
AX-157928405	3	65.083	KV452730.1	6925374	2-scf00040	2
AX-157928407	3	65.083	KV452730.1	6925721	2-scf00040	2
AX-157928424	3	65.083	KV452730.1	6951366	2-scf00040	2
AX-157928425	3	65.083	KV452730.1	6952192	2-scf00040	2
AX-157928431	3	65.083	KV452730.1	6957170	2-scf00040	2
AX-157928433	3	65.083	KV452730.1	6958157	2-scf00040	2
AX-157928434	3	65.083	KV452730.1	6959567	2-scf00040	2
AX-157928435	3	65.083	KV452730.1	6960591	2-scf00040	2
AX-157928439	3	65.083	KV452730.1	6966530	2-scf00040	2
AX-157928453	3	65.083	KV452730.1	6987286	2-scf00040	2
AX-157813828	3	65.083	KV452730.1	6990559	2-scf00040	2
AX-157813829	3	65.083	KV452730.1	6992460	2-scf00040	2
AX-157928464	3	65.083	KV452730.1	7004331	2-scf00040	2
AX-157928468	3	65.083	KV452730.1	7008369	2-scf00040	2
AX-157928474	3	65.083	KV452730.1	7018079	2-scf00040	2
AX-157928496	3	65.083	KV452730.1	7042177	2-scf00040	2
AX-157928552	3	65.083	KV452730.1	7123728	2-scf00040	2
AX-157767841	3	65.083	KV452730.1	7177133	2-scf00040	2
AX-157928625	3	65.083	KV452730.1	7208084	2-scf00040	2
AX-157928626	3	65.083	KV452730.1	7210543	2-scf00040	2
AX-157928627	3	65.083	KV452730.1	7213091	2-scf00040	2
AX-157928631	3	65.083	KV452730.1	7214298	2-scf00040	2
AX-157928636	3	65.083	KV452730.1	7219175	2-scf00040	2
AX-157813840	3	65.083	KV452730.1	7268948	2-scf00040	2
AX-157813843	3	65.083	KV452730.1	7277215	2-scf00040	2
AX-157928703	3	65.083	KV452730.1	7362578	2-scf00040	2

AX-157928783	3	65.083	KV452730.1	7522936	2-scf00040	2
AX-157928787	3	65.083	KV452730.1	7552355	2-scf00040	2
AX-157853886	3	65.083	KV452730.1	7650291	2-scf00040	2
AX-158405438	3	65.083	KV452730.1	7728607	2-scf00040	2
AX-157928872	3	65.083	KV452730.1	7742560	2-scf00040	2
AX-157712756	3	65.083	KV452730.1	7754380	2-scf00040	2
AX-157928886	3	65.083	KV452730.1	7761043	2-scf00040	2
AX-157928899	3	65.083	KV452730.1	7770015	2-scf00040	2
AX-157813853	3	65.083	KV452730.1	7819548	2-scf00040	2
AX-157813855	3	65.083	KV452730.1	7823160	2-scf00040	2
AX-157928965	3	65.083	KV452730.1	7857441	2-scf00040	2
AX-157928971	3	65.083	KV452730.1	7860381	2-scf00040	2
AX-157712647	3	65.083	KV452730.1	7893145	2-scf00040	2
AX-158405443	3	65.083	KV452730.1	7895779	2-scf00040	2
AX-157853888	3	65.083	KV452730.1	7901783	2-scf00040	2
AX-157929013	3	65.083	KV452730.1	7910867	2-scf00040	2
AX-157929030	3	65.083	KV452730.1	7932194	2-scf00040	2
AX-157929035	3	65.083	KV452730.1	7936904	2-scf00040	2
AX-157755979	3	65.083	KV452730.1	7965070	2-scf00040	2
AX-157929070	3	65.083	KV452730.1	7967161	2-scf00040	2
AX-157929092	3	65.083	KV452730.1	7993082	2-scf00040	2
AX-157929097	3	65.083	KV452730.1	8003659	2-scf00040	2
AX-157722812	3	65.083	KV452730.1	8006300	2-scf00040	2
AX-157853889	3	65.083	KV452730.1	8023672	2-scf00040	2
AX-157768714	3	65.083	KV452730.1	8027724	2-scf00040	2
AX-157883656	3	65.083	KV452730.1	8031856	2-scf00040	2
AX-157708284	3	65.083	KV452730.1	8054434	2-scf00040	2
AX-157929154	3	65.083	KV452730.1	8085495	2-scf00040	2
AX-157929161	3	65.083	KV452730.1	8119880	2-scf00040	2
AX-157929162	3	65.083	KV452730.1	8120384	2-scf00040	2
AX-157929331	3	65.083	KV452730.1	8406007	2-scf00040	2
AX-157929417	3	65.083	KV452730.1	8529787	2-scf00040	2
AX-157929429	3	65.083	KV452730.1	8565055	2-scf00040	2
AX-157929433	3	65.083	KV452730.1	8569229	2-scf00040	2
AX-157929437	3	65.083	KV452730.1	8576688	2-scf00040	2
AX-157929442	3	65.083	KV452730.1	8587284	2-scf00040	2
AX-157929444	3	65.083	KV452730.1	8594582	2-scf00040	2
AX-157929446	3	65.083	KV452730.1	8598650	2-scf00040	2
AX-157929450	3	65.083	KV452730.1	8604946	2-scf00040	2
AX-157929456	3	65.083	KV452730.1	8611777	2-scf00040	2
AX-157929475	3	65.083	KV452730.1	8637415	2-scf00040	2
AX-157929637	3	65.083	KV452730.1	8874274	2-scf00040	2
AX-157929840	3	65.083	KV452730.1	9122258	2-scf00040	2
AX-157704623	3	65.083	BML01026798	1271	scf02400_1	UN
AX-157928497	3	65.105	KV452730.1	7044061	2-scf00040	2
AX-157928477	3	65.15	KV452730.1	7019844	2-scf00040	2
AX-157853882	3	65.15	KV452730.1	7052205	2-scf00040	2
AX-157928502	3	65.15	KV452730.1	7052326	2-scf00040	2
AX-157928503	3	65.15	KV452730.1	7052757	2-scf00040	2
AX-157928517	3	65.15	KV452730.1	7072539	2-scf00040	2
AX-157928588	3	65.15	KV452730.1	7169481	2-scf00040	2

AX-158405419	3	65.192	KV452730.1	7060722	2-scf00040	2
AX-157813830	3	65.192	KV452730.1	7065980	2-scf00040	2
AX-157928515	3	65.192	KV452730.1	7067672	2-scf00040	2
AX-157928516	3	65.192	KV452730.1	7069826	2-scf00040	2
AX-157813831	3	65.192	KV452730.1	7074044	2-scf00040	2
AX-157928467	3	65.235	KV452730.1	7007614	2-scf00040	2
AX-157928536	3	65.235	KV452730.1	7109907	2-scf00040	2
AX-157928549	3	65.249	KV452730.1	7122733	2-scf00040	2
AX-157704962	3	65.249	3ML01028083	991	scf03688_1	UN
AX-157928556	3	65.273	KV452730.1	7128043	2-scf00040	2
AX-157928518	3	65.301	KV452730.1	7073063	2-scf00040	2
AX-157928558	3	65.301	KV452730.1	7131385	2-scf00040	2
AX-157710617	3	65.301	KV452730.1	7137840	2-scf00040	2
AX-157928563	3	65.301	KV452730.1	7139107	2-scf00040	2
AX-157928564	3	65.301	KV452730.1	7143394	2-scf00040	2
AX-157928573	3	65.301	KV452730.1	7147900	2-scf00040	2
AX-157928622	3	65.301	KV452730.1	7205521	2-scf00040	2
AX-157928583	3	65.324	KV452730.1	7162656	2-scf00040	2
AX-157928836	3	65.324	KV452730.1	7657129	2-scf00040	2
AX-157928118	3	65.351	KV452730.1	6468979	2-scf00040	2
AX-157928589	3	65.351	KV452730.1	7171081	2-scf00040	2
AX-157715558	3	65.351	KV452730.1	7861329	2-scf00040	2
AX-157928989	3	65.351	KV452730.1	7888978	2-scf00040	2
AX-157929089	3	65.351	KV452730.1	7990053	2-scf00040	2
AX-157813870	3	65.351	KV452730.1	8053241	2-scf00040	2
AX-157929327	3	65.351	KV452730.1	8397122	2-scf00040	2
AX-157929771	3	65.351	KV452730.1	9041996	2-scf00040	2
AX-157929933	3	65.351	KV452730.1	9193441	2-scf00040	2
AX-157930009	3	65.351	KV452730.1	9247353	2-scf00040	2
AX-157928608	3	65.37	KV452730.1	7189417	2-scf00040	2
AX-157928638	3	65.392	KV452730.1	7223045	2-scf00040	2
AX-157813795	3	65.41	KV452730.1	6458343	2-scf00040	2
AX-157813817	3	65.41	KV452730.1	6699116	2-scf00040	2
AX-157928305	3	65.41	KV452730.1	6779144	2-scf00040	2
AX-157928545	3	65.41	KV452730.1	7115700	2-scf00040	2
AX-157928577	3	65.41	KV452730.1	7153018	2-scf00040	2
AX-157928578	3	65.41	KV452730.1	7153608	2-scf00040	2
AX-157928586	3	65.41	KV452730.1	7167021	2-scf00040	2
AX-157928649	3	65.41	KV452730.1	7249876	2-scf00040	2
AX-157759011	3	65.41	KV452730.1	7254928	2-scf00040	2
AX-157928664	3	65.41	KV452730.1	7294462	2-scf00040	2
AX-157928673	3	65.41	KV452730.1	7311312	2-scf00040	2
AX-157928679	3	65.41	KV452730.1	7317489	2-scf00040	2
AX-157928687	3	65.41	KV452730.1	7331292	2-scf00040	2
AX-157928708	3	65.41	KV452730.1	7372370	2-scf00040	2
AX-157928715	3	65.41	KV452730.1	7384083	2-scf00040	2
AX-157928717	3	65.41	KV452730.1	7384846	2-scf00040	2
AX-157928718	3	65.41	KV452730.1	7386399	2-scf00040	2
AX-157928721	3	65.41	KV452730.1	7388430	2-scf00040	2
AX-157928722	3	65.41	KV452730.1	7389650	2-scf00040	2
AX-157928733	3	65.41	KV452730.1	7424949	2-scf00040	2

AX-157928745	3	65.41	KV452730.1	7447930	2-scf00040	2
AX-157928781	3	65.41	KV452730.1	7521850	2-scf00040	2
AX-157928785	3	65.41	KV452730.1	7547173	2-scf00040	2
AX-157928807	3	65.41	KV452730.1	7604811	2-scf00040	2
AX-157928815	3	65.41	KV452730.1	7618003	2-scf00040	2
AX-157928834	3	65.41	KV452730.1	7655196	2-scf00040	2
AX-157928848	3	65.41	KV452730.1	7695070	2-scf00040	2
AX-157928850	3	65.41	KV452730.1	7696468	2-scf00040	2
AX-157928857	3	65.41	KV452730.1	7699265	2-scf00040	2
AX-157698333	3	65.41	KV452730.1	7718025	2-scf00040	2
AX-157928871	3	65.41	KV452730.1	7742186	2-scf00040	2
AX-157928874	3	65.41	KV452730.1	7743240	2-scf00040	2
AX-157928876	3	65.41	KV452730.1	7744666	2-scf00040	2
AX-157928877	3	65.41	KV452730.1	7746313	2-scf00040	2
AX-157928885	3	65.41	KV452730.1	7755665	2-scf00040	2
AX-157708734	3	65.41	KV452730.1	7762373	2-scf00040	2
AX-157928900	3	65.41	KV452730.1	7772159	2-scf00040	2
AX-157928903	3	65.41	KV452730.1	7775583	2-scf00040	2
AX-157874857	3	65.41	KV452730.1	7798035	2-scf00040	2
AX-157745951	3	65.41	KV452730.1	7799187	2-scf00040	2
AX-157928927	3	65.41	KV452730.1	7799629	2-scf00040	2
AX-157928929	3	65.41	KV452730.1	7808046	2-scf00040	2
AX-157716167	3	65.41	KV452730.1	7814861	2-scf00040	2
AX-157813852	3	65.41	KV452730.1	7816545	2-scf00040	2
AX-157813856	3	65.41	KV452730.1	7823609	2-scf00040	2
AX-157853887	3	65.41	KV452730.1	7829983	2-scf00040	2
AX-157928952	3	65.41	KV452730.1	7841304	2-scf00040	2
AX-157928982	3	65.41	KV452730.1	7870240	2-scf00040	2
AX-157929002	3	65.41	KV452730.1	7900148	2-scf00040	2
AX-157929043	3	65.41	KV452730.1	7941209	2-scf00040	2
AX-157929044	3	65.41	KV452730.1	7942863	2-scf00040	2
AX-157929061	3	65.41	KV452730.1	7952990	2-scf00040	2
AX-157929063	3	65.41	KV452730.1	7954949	2-scf00040	2
AX-157929086	3	65.41	KV452730.1	7988855	2-scf00040	2
AX-157741244	3	65.41	KV452730.1	7990986	2-scf00040	2
AX-157929094	3	65.41	KV452730.1	7997262	2-scf00040	2
AX-157929133	3	65.41	KV452730.1	8047953	2-scf00040	2
AX-157929146	3	65.41	KV452730.1	8074820	2-scf00040	2
AX-157929422	3	65.41	KV452730.1	8559106	2-scf00040	2
AX-157929460	3	65.41	KV452730.1	8620074	2-scf00040	2
AX-158405466	3	65.41	KV452730.1	8723837	2-scf00040	2
AX-157929682	3	65.41	KV452730.1	8944640	2-scf00040	2
AX-157704961	3	65.41	3ML01028083	458	scf03688_1	UN
AX-157928676	3	65.56	KV452730.1	7312548	2-scf00040	2
AX-157928705	3	65.56	KV452730.1	7367090	2-scf00040	2
AX-157928711	3	65.56	KV452730.1	7380024	2-scf00040	2
AX-157928737	3	65.56	KV452730.1	7442301	2-scf00040	2
AX-157928739	3	65.56	KV452730.1	7443322	2-scf00040	2
AX-157928957	3	65.56	KV452730.1	7843889	2-scf00040	2
AX-157749834	3	65.56	KV452730.1	7858797	2-scf00040	2
AX-157715849	3	65.56	KV452730.1	7859384	2-scf00040	2

AX-157883655	3	65.56	KV452730.1	7880370	2-scf00040	2
AX-157813863	3	65.56	KV452730.1	7885998	2-scf00040	2
AX-157928985	3	65.56	KV452730.1	7886347	2-scf00040	2
AX-157928994	3	65.56	KV452730.1	7891792	2-scf00040	2
AX-157929000	3	65.56	KV452730.1	7896940	2-scf00040	2
AX-157929011	3	65.56	KV452730.1	7909965	2-scf00040	2
AX-157929040	3	65.56	KV452730.1	7940289	2-scf00040	2
AX-157929041	3	65.56	KV452730.1	7940649	2-scf00040	2
AX-157750660	3	65.56	KV452730.1	7996733	2-scf00040	2
AX-157929096	3	65.56	KV452730.1	7999101	2-scf00040	2
AX-157749060	3	65.56	KV452730.1	8000438	2-scf00040	2
AX-157929108	3	65.56	KV452730.1	8014613	2-scf00040	2
AX-157714407	3	65.56	KV452730.1	8067421	2-scf00040	2
AX-157929157	3	65.56	KV452730.1	8107288	2-scf00040	2
AX-157929247	3	65.56	KV452730.1	8271028	2-scf00040	2
AX-157929254	3	65.56	KV452730.1	8277349	2-scf00040	2
AX-157929274	3	65.56	KV452730.1	8307305	2-scf00040	2
AX-157929412	3	65.56	KV452730.1	8525628	2-scf00040	2
AX-157929566	3	65.56	KV452730.1	8773479	2-scf00040	2
AX-157929607	3	65.56	KV452730.1	8818803	2-scf00040	2
AX-157929611	3	65.56	KV452730.1	8825183	2-scf00040	2
AX-157714749	3	65.56	KV452730.1	8894331	2-scf00040	2
AX-157929670	3	65.56	KV452730.1	8934443	2-scf00040	2
AX-157929735	3	65.56	KV452730.1	8992717	2-scf00040	2
AX-157929109	3	65.636	KV452730.1	8016664	2-scf00040	2
AX-157928898	3	65.71	KV452730.1	7769482	2-scf00040	2
AX-157813854	3	65.71	KV452730.1	7821883	2-scf00040	2
AX-157928948	3	65.71	KV452730.1	7838409	2-scf00040	2
AX-157929115	3	65.71	KV452730.1	8025783	2-scf00040	2
AX-157929116	3	65.71	KV452730.1	8026575	2-scf00040	2
AX-157929121	3	65.71	KV452730.1	8029179	2-scf00040	2
AX-157929125	3	65.71	KV452730.1	8036562	2-scf00040	2
AX-157929443	3	65.71	KV452730.1	8588175	2-scf00040	2
AX-157929467	3	65.71	KV452730.1	8628420	2-scf00040	2
AX-157929470	3	65.71	KV452730.1	8630081	2-scf00040	2
AX-157929474	3	65.71	KV452730.1	8636431	2-scf00040	2
AX-157929478	3	65.71	KV452730.1	8639802	2-scf00040	2
AX-157929484	3	65.71	KV452730.1	8646892	2-scf00040	2
AX-157929494	3	65.71	KV452730.1	8656582	2-scf00040	2
AX-157929511	3	65.71	KV452730.1	8696881	2-scf00040	2
AX-157929513	3	65.71	KV452730.1	8701004	2-scf00040	2
AX-157929515	3	65.71	KV452730.1	8707300	2-scf00040	2
AX-157929520	3	65.71	KV452730.1	8733161	2-scf00040	2
AX-157929535	3	65.71	KV452730.1	8742034	2-scf00040	2
AX-157929536	3	65.71	KV452730.1	8742890	2-scf00040	2
AX-157929547	3	65.71	KV452730.1	8752126	2-scf00040	2
AX-157929616	3	65.71	KV452730.1	8834248	2-scf00040	2
AX-157929621	3	65.71	KV452730.1	8845532	2-scf00040	2
AX-157813914	3	65.71	KV452730.1	8988709	2-scf00040	2
AX-157869201	3	65.71	KV452730.1	8996440	2-scf00040	2
AX-157929752	3	65.71	KV452730.1	9020230	2-scf00040	2

AX-157929772	3	65.71	KV452730.1	9042761	2-scf00040	2
AX-157701502	3	65.71	KV452730.1	9090561	2-scf00040	2
AX-157929132	3	65.725	KV452730.1	8046707	2-scf00040	2
AX-157929139	3	65.798	KV452730.1	8053907	2-scf00040	2
AX-157708285	3	65.828	KV452730.1	8054358	2-scf00040	2
AX-157928011	3	65.86	KV452730.1	6359479	2-scf00040	2
AX-157928307	3	65.86	KV452730.1	6779563	2-scf00040	2
AX-157928580	3	65.86	KV452730.1	7154734	2-scf00040	2
AX-157928629	3	65.86	KV452730.1	7213733	2-scf00040	2
AX-157928819	3	65.86	KV452730.1	7621992	2-scf00040	2
AX-157928907	3	65.86	KV452730.1	7780400	2-scf00040	2
AX-157929053	3	65.86	KV452730.1	7948545	2-scf00040	2
AX-157929091	3	65.86	KV452730.1	7992049	2-scf00040	2
AX-157813868	3	65.86	KV452730.1	8044327	2-scf00040	2
AX-157813872	3	65.86	KV452730.1	8059890	2-scf00040	2
AX-157745759	3	65.86	KV452730.1	8064208	2-scf00040	2
AX-157929147	3	65.86	KV452730.1	8075265	2-scf00040	2
AX-157853892	3	65.86	KV452730.1	8077456	2-scf00040	2
AX-157813873	3	65.86	KV452730.1	8096097	2-scf00040	2
AX-158382440	3	65.86	KV452730.1	8151675	2-scf00040	2
AX-157929192	3	65.86	KV452730.1	8154451	2-scf00040	2
AX-157929196	3	65.86	KV452730.1	8159581	2-scf00040	2
AX-157813881	3	65.86	KV452730.1	8200826	2-scf00040	2
AX-157929214	3	65.86	KV452730.1	8202073	2-scf00040	2
AX-157929227	3	65.86	KV452730.1	8239701	2-scf00040	2
AX-157929233	3	65.86	KV452730.1	8252680	2-scf00040	2
AX-157708332	3	65.86	KV452730.1	8257674	2-scf00040	2
AX-157929239	3	65.86	KV452730.1	8263102	2-scf00040	2
AX-157929242	3	65.86	KV452730.1	8268596	2-scf00040	2
AX-157929248	3	65.86	KV452730.1	8275247	2-scf00040	2
AX-157853908	3	65.86	KV452730.1	8316835	2-scf00040	2
AX-157929279	3	65.86	KV452730.1	8316879	2-scf00040	2
AX-157929282	3	65.86	KV452730.1	8317690	2-scf00040	2
AX-157813889	3	65.86	KV452730.1	8318542	2-scf00040	2
AX-157746406	3	65.86	KV452730.1	8320037	2-scf00040	2
AX-157929283	3	65.86	KV452730.1	8321185	2-scf00040	2
AX-157741182	3	65.86	KV452730.1	8322346	2-scf00040	2
AX-157929306	3	65.86	KV452730.1	8366490	2-scf00040	2
AX-157929307	3	65.86	KV452730.1	8368553	2-scf00040	2
AX-157929309	3	65.86	KV452730.1	8371762	2-scf00040	2
AX-157929317	3	65.86	KV452730.1	8381426	2-scf00040	2
AX-157929318	3	65.86	KV452730.1	8382301	2-scf00040	2
AX-157929329	3	65.86	KV452730.1	8400408	2-scf00040	2
AX-157813894	3	65.86	KV452730.1	8402301	2-scf00040	2
AX-157929337	3	65.86	KV452730.1	8411710	2-scf00040	2
AX-157929374	3	65.86	KV452730.1	8483354	2-scf00040	2
AX-157929378	3	65.86	KV452730.1	8489324	2-scf00040	2
AX-157929382	3	65.86	KV452730.1	8492784	2-scf00040	2
AX-158405455	3	65.86	KV452730.1	8495420	2-scf00040	2
AX-157929384	3	65.86	KV452730.1	8496098	2-scf00040	2
AX-157929386	3	65.86	KV452730.1	8497229	2-scf00040	2

AX-157883664	3	65.86	KV452730.1	8589110	2-scf00040	2
AX-157929453	3	65.86	KV452730.1	8608217	2-scf00040	2
AX-157929753	3	65.86	KV452730.1	9020712	2-scf00040	2
AX-157928960	3	65.861	KV452730.1	7852544	2-scf00040	2
AX-157928968	3	65.861	KV452730.1	7858330	2-scf00040	2
AX-157929014	3	65.861	KV452730.1	7913884	2-scf00040	2
AX-157929020	3	65.861	KV452730.1	7925109	2-scf00040	2
AX-157929023	3	65.861	KV452730.1	7926204	2-scf00040	2
AX-157929038	3	65.861	KV452730.1	7939335	2-scf00040	2
AX-157929049	3	65.861	KV452730.1	7944650	2-scf00040	2
AX-157718287	3	65.861	KV452730.1	7956610	2-scf00040	2
AX-157813866	3	65.861	KV452730.1	7965556	2-scf00040	2
AX-157929113	3	65.861	KV452730.1	8019429	2-scf00040	2
AX-157929151	3	65.861	KV452730.1	8079119	2-scf00040	2
AX-157929160	3	65.861	KV452730.1	8110720	2-scf00040	2
AX-157929209	3	65.861	KV452730.1	8177225	2-scf00040	2
AX-157929215	3	65.861	KV452730.1	8203466	2-scf00040	2
AX-157929240	3	65.861	KV452730.1	8265477	2-scf00040	2
AX-157883658	3	65.861	KV452730.1	8361678	2-scf00040	2
AX-157929334	3	65.861	KV452730.1	8409958	2-scf00040	2
AX-157929385	3	65.861	KV452730.1	8496923	2-scf00040	2
AX-157929387	3	65.861	KV452730.1	8498624	2-scf00040	2
AX-157929389	3	65.861	KV452730.1	8499511	2-scf00040	2
AX-157929392	3	65.861	KV452730.1	8501601	2-scf00040	2
AX-157929399	3	65.861	KV452730.1	8509772	2-scf00040	2
AX-157929405	3	65.861	KV452730.1	8516644	2-scf00040	2
AX-157929408	3	65.861	KV452730.1	8519298	2-scf00040	2
AX-157929411	3	65.861	KV452730.1	8522256	2-scf00040	2
AX-157929414	3	65.861	KV452730.1	8527910	2-scf00040	2
AX-157929415	3	65.861	KV452730.1	8528965	2-scf00040	2
AX-157929418	3	65.861	KV452730.1	8533124	2-scf00040	2
AX-157929461	3	65.861	KV452730.1	8623939	2-scf00040	2
AX-157929469	3	65.861	KV452730.1	8628606	2-scf00040	2
AX-157929477	3	65.861	KV452730.1	8638890	2-scf00040	2
AX-157929500	3	65.861	KV452730.1	8663714	2-scf00040	2
AX-157929502	3	65.861	KV452730.1	8664066	2-scf00040	2
AX-158405464	3	65.861	KV452730.1	8700249	2-scf00040	2
AX-158405465	3	65.861	KV452730.1	8700388	2-scf00040	2
AX-157929514	3	65.861	KV452730.1	8704560	2-scf00040	2
AX-157883666	3	65.861	KV452730.1	8714198	2-scf00040	2
AX-157929531	3	65.861	KV452730.1	8738845	2-scf00040	2
AX-157929539	3	65.861	KV452730.1	8743889	2-scf00040	2
AX-157929553	3	65.861	KV452730.1	8758504	2-scf00040	2
AX-158382446	3	65.861	KV452730.1	8764247	2-scf00040	2
AX-157929560	3	65.861	KV452730.1	8768437	2-scf00040	2
AX-157929580	3	65.861	KV452730.1	8790723	2-scf00040	2
AX-157929582	3	65.861	KV452730.1	8790949	2-scf00040	2
AX-157929587	3	65.861	KV452730.1	8796184	2-scf00040	2
AX-157929590	3	65.861	KV452730.1	8797363	2-scf00040	2
AX-157929643	3	65.861	KV452730.1	8882322	2-scf00040	2
AX-157929668	3	65.861	KV452730.1	8932802	2-scf00040	2

AX-157929728	3	65.861	KV452730.1	8990414	2-scf00040	2
AX-157929729	3	65.861	KV452730.1	8990732	2-scf00040	2
AX-157706547	3	65.861	3ML01032430	172	scf08050_1	UN
AX-157929428	3	65.88	KV452730.1	8563086	2-scf00040	2
AX-157929449	3	65.898	KV452730.1	8604334	2-scf00040	2
AX-157929525	3	65.905	KV452730.1	8735624	2-scf00040	2
AX-157929589	3	65.914	KV452730.1	8796667	2-scf00040	2
AX-157813819	3	66.017	KV452730.1	6709541	2-scf00040	2
AX-157928280	3	66.017	KV452730.1	6743700	2-scf00040	2
AX-157928284	3	66.017	KV452730.1	6748755	2-scf00040	2
AX-157928287	3	66.017	KV452730.1	6749015	2-scf00040	2
AX-157928313	3	66.017	KV452730.1	6794552	2-scf00040	2
AX-157928388	3	66.017	KV452730.1	6902766	2-scf00040	2
AX-157754236	3	66.017	KV452730.1	6967090	2-scf00040	2
AX-157874847	3	66.017	KV452730.1	6982133	2-scf00040	2
AX-157928535	3	66.017	KV452730.1	7103686	2-scf00040	2
AX-157853883	3	66.017	KV452730.1	7243025	2-scf00040	2
AX-157928648	3	66.017	KV452730.1	7249423	2-scf00040	2
AX-157813845	3	66.017	KV452730.1	7286033	2-scf00040	2
AX-157928666	3	66.017	KV452730.1	7295479	2-scf00040	2
AX-157928672	3	66.017	KV452730.1	7304276	2-scf00040	2
AX-157928683	3	66.017	KV452730.1	7321748	2-scf00040	2
AX-157928695	3	66.017	KV452730.1	7346780	2-scf00040	2
AX-157928849	3	66.017	KV452730.1	7696293	2-scf00040	2
AX-157928860	3	66.017	KV452730.1	7709774	2-scf00040	2
AX-157813848	3	66.017	KV452730.1	7730944	2-scf00040	2
AX-157813861	3	66.017	KV452730.1	7880513	2-scf00040	2
AX-157928996	3	66.017	KV452730.1	7892694	2-scf00040	2
AX-157929010	3	66.017	KV452730.1	7909029	2-scf00040	2
AX-157813867	3	66.017	KV452730.1	8018639	2-scf00040	2
AX-157929261	3	66.017	KV452730.1	8293269	2-scf00040	2
AX-157929427	3	66.017	KV452730.1	8562540	2-scf00040	2
AX-157929432	3	66.017	KV452730.1	8568693	2-scf00040	2
AX-158405459	3	66.017	KV452730.1	8584111	2-scf00040	2
AX-157929440	3	66.017	KV452730.1	8585328	2-scf00040	2
AX-157929447	3	66.017	KV452730.1	8600377	2-scf00040	2
AX-157929448	3	66.017	KV452730.1	8601627	2-scf00040	2
AX-157929569	3	66.017	KV452730.1	8778593	2-scf00040	2
AX-157929594	3	66.017	KV452730.1	8803726	2-scf00040	2
AX-157929596	3	66.017	KV452730.1	8804889	2-scf00040	2
AX-157929597	3	66.017	KV452730.1	8806557	2-scf00040	2
AX-157929599	3	66.017	KV452730.1	8807871	2-scf00040	2
AX-157929605	3	66.017	KV452730.1	8812901	2-scf00040	2
AX-157929619	3	66.017	KV452730.1	8843235	2-scf00040	2
AX-157929620	3	66.017	KV452730.1	8843788	2-scf00040	2
AX-157929623	3	66.017	KV452730.1	8846245	2-scf00040	2
AX-157929645	3	66.017	KV452730.1	8885417	2-scf00040	2
AX-157813906	3	66.017	KV452730.1	8900743	2-scf00040	2
AX-157813907	3	66.017	KV452730.1	8904456	2-scf00040	2
AX-157929657	3	66.017	KV452730.1	8911679	2-scf00040	2
AX-157929663	3	66.017	KV452730.1	8922407	2-scf00040	2

AX-157929672	3	66.017	KV452730.1	8935589	2-scf00040	2
AX-157813912	3	66.017	KV452730.1	8980665	2-scf00040	2
AX-157718615	3	66.017	KV452730.1	8980764	2-scf00040	2
AX-157813913	3	66.017	KV452730.1	8981385	2-scf00040	2
AX-157929718	3	66.017	KV452730.1	8981589	2-scf00040	2
AX-157929721	3	66.017	KV452730.1	8986555	2-scf00040	2
AX-157929724	3	66.017	KV452730.1	8989025	2-scf00040	2
AX-157929730	3	66.017	KV452730.1	8991145	2-scf00040	2
AX-157929733	3	66.017	KV452730.1	8992013	2-scf00040	2
AX-157929736	3	66.017	KV452730.1	8993292	2-scf00040	2
AX-157713019	3	66.017	KV452730.1	8996966	2-scf00040	2
AX-157813915	3	66.017	KV452730.1	8997017	2-scf00040	2
AX-157929740	3	66.017	KV452730.1	9000744	2-scf00040	2
AX-157929770	3	66.017	KV452730.1	9041081	2-scf00040	2
AX-157929809	3	66.017	KV452730.1	9078952	2-scf00040	2
AX-158404582	3	66.017	KV452730.1	9092192	2-scf00040	2
AX-157929825	3	66.017	KV452730.1	9094660	2-scf00040	2
AX-157929827	3	66.017	KV452730.1	9095409	2-scf00040	2
AX-157929828	3	66.017	KV452730.1	9095730	2-scf00040	2
AX-157929830	3	66.017	KV452730.1	9097061	2-scf00040	2
AX-157869203	3	66.017	KV452730.1	9106655	2-scf00040	2
AX-157813923	3	66.017	KV452730.1	9112672	2-scf00040	2
AX-157929901	3	66.017	KV452730.1	9166587	2-scf00040	2
AX-157929989	3	66.017	KV452730.1	9236968	2-scf00040	2
AX-157930034	3	66.017	KV452730.1	9273037	2-scf00040	2
AX-157930044	3	66.017	KV452730.1	9279715	2-scf00040	2
AX-157928291	3	66.176	KV452730.1	6752170	2-scf00040	2
AX-157928660	3	66.176	KV452730.1	7265907	2-scf00040	2
AX-157928724	3	66.176	KV452730.1	7400984	2-scf00040	2
AX-157929234	3	66.176	KV452730.1	8257038	2-scf00040	2
AX-157929570	3	66.176	KV452730.1	8779021	2-scf00040	2
AX-157929612	3	66.176	KV452730.1	8827309	2-scf00040	2
AX-157929662	3	66.176	KV452730.1	8922179	2-scf00040	2
AX-157813908	3	66.176	KV452730.1	8946129	2-scf00040	2
AX-157929746	3	66.176	KV452730.1	9007144	2-scf00040	2
AX-157929748	3	66.176	KV452730.1	9019384	2-scf00040	2
AX-157853934	3	66.176	KV452730.1	9027632	2-scf00040	2
AX-157929758	3	66.176	KV452730.1	9027974	2-scf00040	2
AX-157929759	3	66.176	KV452730.1	9030574	2-scf00040	2
AX-157929760	3	66.176	KV452730.1	9031480	2-scf00040	2
AX-157929762	3	66.176	KV452730.1	9031777	2-scf00040	2
AX-157929763	3	66.176	KV452730.1	9031967	2-scf00040	2
AX-157813916	3	66.176	KV452730.1	9032886	2-scf00040	2
AX-157929764	3	66.176	KV452730.1	9034758	2-scf00040	2
AX-157929765	3	66.176	KV452730.1	9035980	2-scf00040	2
AX-157929769	3	66.176	KV452730.1	9039964	2-scf00040	2
AX-157813917	3	66.176	KV452730.1	9041267	2-scf00040	2
AX-157929776	3	66.176	KV452730.1	9047589	2-scf00040	2
AX-157929780	3	66.176	KV452730.1	9050281	2-scf00040	2
AX-157813922	3	66.176	KV452730.1	9054967	2-scf00040	2
AX-157929788	3	66.176	KV452730.1	9061435	2-scf00040	2

AX-157929789	3	66.176	KV452730.1	9062995	2-scf00040	2
AX-157929791	3	66.176	KV452730.1	9063712	2-scf00040	2
AX-157929792	3	66.176	KV452730.1	9064364	2-scf00040	2
AX-157929794	3	66.176	KV452730.1	9065465	2-scf00040	2
AX-157929799	3	66.176	KV452730.1	9067582	2-scf00040	2
AX-157929800	3	66.176	KV452730.1	9068827	2-scf00040	2
AX-157929802	3	66.176	KV452730.1	9071643	2-scf00040	2
AX-157753934	3	66.176	KV452730.1	9074340	2-scf00040	2
AX-157929805	3	66.176	KV452730.1	9074923	2-scf00040	2
AX-157929806	3	66.176	KV452730.1	9075102	2-scf00040	2
AX-157929813	3	66.176	KV452730.1	9080782	2-scf00040	2
AX-157929817	3	66.176	KV452730.1	9085047	2-scf00040	2
AX-157929818	3	66.176	KV452730.1	9085619	2-scf00040	2
AX-157929820	3	66.176	KV452730.1	9087445	2-scf00040	2
AX-157929831	3	66.176	KV452730.1	9098850	2-scf00040	2
AX-157929838	3	66.176	KV452730.1	9113886	2-scf00040	2
AX-157715208	3	66.176	KV452730.1	9115889	2-scf00040	2
AX-157738372	3	66.176	KV452730.1	9123773	2-scf00040	2
AX-157929852	3	66.176	KV452730.1	9131228	2-scf00040	2
AX-157929902	3	66.176	KV452730.1	9168143	2-scf00040	2
AX-157930053	3	66.176	KV452730.1	9284873	2-scf00040	2
AX-157929866	3	66.363	KV452730.1	9135098	2-scf00040	2
AX-157929870	3	66.394	KV452730.1	9136314	2-scf00040	2
AX-157929871	3	66.394	KV452730.1	9138024	2-scf00040	2
AX-157929874	3	66.394	KV452730.1	9140717	2-scf00040	2
AX-157929876	3	66.394	KV452730.1	9142655	2-scf00040	2
AX-157929879	3	66.394	KV452730.1	9143697	2-scf00040	2
AX-157929882	3	66.394	KV452730.1	9144271	2-scf00040	2
AX-157813926	3	66.394	KV452730.1	9146343	2-scf00040	2
AX-157929884	3	66.394	KV452730.1	9146592	2-scf00040	2
AX-157929886	3	66.394	KV452730.1	9150144	2-scf00040	2
AX-158405483	3	66.394	KV452730.1	9150582	2-scf00040	2
AX-157929888	3	66.394	KV452730.1	9153526	2-scf00040	2
AX-157813927	3	66.394	KV452730.1	9163262	2-scf00040	2
AX-157929889	3	66.425	KV452730.1	9154968	2-scf00040	2
AX-157710114	3	66.454	KV452730.1	9157241	2-scf00040	2
AX-157929890	3	66.503	KV452730.1	9155496	2-scf00040	2
AX-157929897	3	66.503	KV452730.1	9160158	2-scf00040	2
AX-157929898	3	66.503	KV452730.1	9161858	2-scf00040	2
AX-157813928	3	66.503	KV452730.1	9168757	2-scf00040	2
AX-157929904	3	66.503	KV452730.1	9169252	2-scf00040	2
AX-157929915	3	66.503	KV452730.1	9174851	2-scf00040	2
AX-157929918	3	66.503	KV452730.1	9176582	2-scf00040	2
AX-157929919	3	66.503	KV452730.1	9177329	2-scf00040	2
AX-157929921	3	66.503	KV452730.1	9179322	2-scf00040	2
AX-157757023	3	66.503	KV452730.1	9181233	2-scf00040	2
AX-157751041	3	66.503	KV452730.1	9181587	2-scf00040	2
AX-157929924	3	66.503	KV452730.1	9186218	2-scf00040	2
AX-157929928	3	66.503	KV452730.1	9189351	2-scf00040	2
AX-157929981	3	66.503	KV452730.1	9229853	2-scf00040	2
AX-157930001	3	66.503	KV452730.1	9243070	2-scf00040	2

AX-157930040	3	66.503	KV452730.1	9275943	2-scf00040	2
AX-157853937	3	66.503	KV452730.1	9279180	2-scf00040	2
AX-157853938	3	66.503	KV452730.1	9279944	2-scf00040	2
AX-157930051	3	66.503	KV452730.1	9284328	2-scf00040	2
AX-157930055	3	66.503	KV452730.1	9286335	2-scf00040	2
AX-157853939	3	66.503	KV452730.1	9287039	2-scf00040	2
AX-157930057	3	66.503	KV452730.1	9287182	2-scf00040	2
AX-157930058	3	66.503	KV452730.1	9287850	2-scf00040	2
AX-157930077	3	66.503	KV452730.1	9305978	2-scf00040	2
AX-157930085	3	66.503	KV452730.1	9313900	2-scf00040	2
AX-157929930	3	66.576	KV452730.1	9190952	2-scf00040	2
AX-157929937	3	66.692	KV452730.1	9202120	2-scf00040	2
AX-157713512	3	66.722	KV452730.1	6644050	2-scf00040	2
AX-157928346	3	66.722	KV452730.1	6850316	2-scf00040	2
AX-157928469	3	66.722	KV452730.1	7010113	2-scf00040	2
AX-157928726	3	66.722	KV452730.1	7408202	2-scf00040	2
AX-157928831	3	66.722	KV452730.1	7649098	2-scf00040	2
AX-157773174	3	66.722	KV452730.1	7809754	2-scf00040	2
AX-157929016	3	66.722	KV452730.1	7918862	2-scf00040	2
AX-157929822	3	66.722	KV452730.1	9088501	2-scf00040	2
AX-157929823	3	66.722	KV452730.1	9088876	2-scf00040	2
AX-157929849	3	66.722	KV452730.1	9129508	2-scf00040	2
AX-157929935	3	66.722	KV452730.1	9201546	2-scf00040	2
AX-157929943	3	66.722	KV452730.1	9204447	2-scf00040	2
AX-157929946	3	66.722	KV452730.1	9209533	2-scf00040	2
AX-157929948	3	66.722	KV452730.1	9209954	2-scf00040	2
AX-157929951	3	66.722	KV452730.1	9212291	2-scf00040	2
AX-157929957	3	66.722	KV452730.1	9216998	2-scf00040	2
AX-157929959	3	66.722	KV452730.1	9218012	2-scf00040	2
AX-157929960	3	66.722	KV452730.1	9218546	2-scf00040	2
AX-157929963	3	66.722	KV452730.1	9220668	2-scf00040	2
AX-157929966	3	66.722	KV452730.1	9221995	2-scf00040	2
AX-157929977	3	66.722	KV452730.1	9227470	2-scf00040	2
AX-157929983	3	66.722	KV452730.1	9230554	2-scf00040	2
AX-157722770	3	66.722	KV452730.1	9239628	2-scf00040	2
AX-157929993	3	66.722	KV452730.1	9239754	2-scf00040	2
AX-157929994	3	66.722	KV452730.1	9240025	2-scf00040	2
AX-157929998	3	66.722	KV452730.1	9241678	2-scf00040	2
AX-157930005	3	66.722	KV452730.1	9244814	2-scf00040	2
AX-157930007	3	66.722	KV452730.1	9245085	2-scf00040	2
AX-157930014	3	66.722	KV452730.1	9251937	2-scf00040	2
AX-157853936	3	66.722	KV452730.1	9254083	2-scf00040	2
AX-157930016	3	66.722	KV452730.1	9254385	2-scf00040	2
AX-157930020	3	66.722	KV452730.1	9256667	2-scf00040	2
AX-157930022	3	66.722	KV452730.1	9257851	2-scf00040	2
AX-157930023	3	66.722	KV452730.1	9258574	2-scf00040	2
AX-157930024	3	66.722	KV452730.1	9260101	2-scf00040	2
AX-157930027	3	66.722	KV452730.1	9262863	2-scf00040	2
AX-157930028	3	66.722	KV452730.1	9263455	2-scf00040	2
AX-157930036	3	66.722	KV452730.1	9273556	2-scf00040	2
AX-157930038	3	66.722	KV452730.1	9274189	2-scf00040	2

AX-157930043	3	66.722	KV452730.1	9279015	2-scf00040	2
AX-157930047	3	66.722	KV452730.1	9280746	2-scf00040	2
AX-157930048	3	66.722	KV452730.1	9281818	2-scf00040	2
AX-158389737	3	66.722	KV452730.1	9285261	2-scf00040	2
AX-157930060	3	66.722	KV452730.1	9290258	2-scf00040	2
AX-157930063	3	66.722	KV452730.1	9292124	2-scf00040	2
AX-157930068	3	66.9	KV452730.1	9296313	2-scf00040	2
AX-157929945	3	67.05	KV452730.1	9207282	2-scf00040	2
AX-157929954	3	67.05	KV452730.1	9215196	2-scf00040	2
AX-157929999	3	67.05	KV452730.1	9242263	2-scf00040	2
AX-157874878	3	67.05	KV452730.1	9300039	2-scf00040	2
AX-157930074	3	67.05	KV452730.1	9303631	2-scf00040	2
AX-157930075	3	67.05	KV452730.1	9303983	2-scf00040	2
AX-157930080	3	67.05	KV452730.1	9308847	2-scf00040	2
AX-157930089	3	67.05	KV452730.1	9316875	2-scf00040	2
AX-157930092	3	67.05	KV452730.1	9318246	2-scf00040	2
AX-157813934	3	67.05	KV452730.1	9326621	2-scf00040	2
AX-157930099	3	67.05	KV452730.1	9327369	2-scf00040	2
AX-157930100	3	67.05	KV452730.1	9329078	2-scf00040	2
AX-158379344	3	67.05	KV452730.1	9329735	2-scf00040	2
AX-157930101	3	67.05	KV452730.1	9329865	2-scf00040	2
AX-157930102	3	67.05	KV452730.1	9330462	2-scf00040	2
AX-157720173	3	67.05	KV452730.1	9331638	2-scf00040	2
AX-157930125	3	67.05	KV452730.1	9356865	2-scf00040	2
AX-157930138	3	67.05	KV452730.1	9368345	2-scf00040	2
AX-157930173	3	67.05	KV452730.1	9396815	2-scf00040	2
AX-157930239	3	67.05	KV452730.1	9456443	2-scf00040	2
AX-157930241	3	67.05	KV452730.1	9458435	2-scf00040	2
AX-157930353	3	67.05	KV452730.1	9616690	2-scf00040	2
AX-157930354	3	67.05	KV452730.1	9617417	2-scf00040	2
AX-158389752	3	67.05	KV452730.1	10559891	2-scf00040	2
AX-157930989	3	67.05	KV452730.1	10560466	2-scf00040	2
AX-157930996	3	67.05	KV452730.1	10564580	2-scf00040	2
AX-157930997	3	67.05	KV452730.1	10566997	2-scf00040	2
AX-157931007	3	67.05	KV452730.1	10573592	2-scf00040	2
AX-157869224	3	67.05	KV452730.1	10580115	2-scf00040	2
AX-157931016	3	67.05	KV452730.1	10581864	2-scf00040	2
AX-158389753	3	67.05	KV452730.1	10591218	2-scf00040	2
AX-157931036	3	67.05	KV452730.1	10598572	2-scf00040	2
AX-157931039	3	67.05	KV452730.1	10600576	2-scf00040	2
AX-157931043	3	67.05	KV452730.1	10606553	2-scf00040	2
AX-157931051	3	67.05	KV452730.1	10611754	2-scf00040	2
AX-157931052	3	67.05	KV452730.1	10612395	2-scf00040	2
AX-157931054	3	67.05	KV452730.1	10613752	2-scf00040	2
AX-157854027	3	67.05	KV452730.1	10615901	2-scf00040	2
AX-157931061	3	67.05	KV452730.1	10618392	2-scf00040	2
AX-157931065	3	67.05	KV452730.1	10619934	2-scf00040	2
AX-157931075	3	67.05	KV452730.1	10628483	2-scf00040	2
AX-157931078	3	67.05	KV452730.1	10629801	2-scf00040	2
AX-158389756	3	67.05	KV452730.1	10640923	2-scf00040	2
AX-157931108	3	67.05	KV452730.1	10656206	2-scf00040	2

AX-157869225	3	67.05	KV452730.1	10659261	2-scf00040	2
AX-157869226	3	67.05	KV452730.1	10659557	2-scf00040	2
AX-157931111	3	67.05	KV452730.1	10662582	2-scf00040	2
AX-157931116	3	67.05	KV452730.1	10668061	2-scf00040	2
AX-157869229	3	67.05	KV452730.1	10669626	2-scf00040	2
AX-158389757	3	67.05	KV452730.1	10670327	2-scf00040	2
AX-157931119	3	67.05	KV452730.1	10670872	2-scf00040	2
AX-157931120	3	67.05	KV452730.1	10673169	2-scf00040	2
AX-157862713	3	67.05	KV452730.1	10673935	2-scf00040	2
AX-158389760	3	67.05	KV452730.1	10682101	2-scf00040	2
AX-157931127	3	67.05	KV452730.1	10683169	2-scf00040	2
AX-157931131	3	67.05	KV452730.1	10684062	2-scf00040	2
AX-157814025	3	67.05	KV452730.1	10691485	2-scf00040	2
AX-157931137	3	67.05	KV452730.1	10691533	2-scf00040	2
AX-157931146	3	67.05	KV452730.1	10696936	2-scf00040	2
AX-157931147	3	67.05	KV452730.1	10698824	2-scf00040	2
AX-157931150	3	67.05	KV452730.1	10700967	2-scf00040	2
AX-158389761	3	67.05	KV452730.1	10704193	2-scf00040	2
AX-157931154	3	67.05	KV452730.1	10704738	2-scf00040	2
AX-157814046	3	67.05	KV452730.1	10813947	2-scf00040	2
AX-157717479	3	67.05	KV452730.1	10888595	2-scf00040	2
AX-157814064	3	67.05	KV452730.1	11082266	2-scf00040	2
AX-157814065	3	67.05	KV452730.1	11084114	2-scf00040	2
AX-157869262	3	67.05	KV452730.1	11208534	2-scf00040	2
AX-157814083	3	67.05	KV452730.1	11209206	2-scf00040	2
AX-157869263	3	67.05	KV452730.1	11209588	2-scf00040	2
AX-157931594	3	67.05	KV452730.1	11209846	2-scf00040	2
AX-157862720	3	67.05	KV452730.1	11210074	2-scf00040	2
AX-157931595	3	67.05	KV452730.1	11213009	2-scf00040	2
AX-157854053	3	67.05	KV452730.1	11220232	2-scf00040	2
AX-157931599	3	67.05	KV452730.1	11220392	2-scf00040	2
AX-157931600	3	67.05	KV452730.1	11221042	2-scf00040	2
AX-157931605	3	67.05	KV452730.1	11224114	2-scf00040	2
AX-157931610	3	67.05	KV452730.1	11227331	2-scf00040	2
AX-157814084	3	67.05	KV452730.1	11228067	2-scf00040	2
AX-157931612	3	67.05	KV452730.1	11230802	2-scf00040	2
AX-157931644	3	67.05	KV452730.1	11273383	2-scf00040	2
AX-157931831	3	67.05	KV452730.1	11506027	2-scf00040	2
AX-157854116	3	67.05	KV452730.1	11638433	2-scf00040	2
AX-157854127	3	67.05	KV452730.1	11659007	2-scf00040	2
AX-157854142	3	67.05	KV452730.1	11713140	2-scf00040	2
AX-157854153	3	67.05	KV452730.1	11741191	2-scf00040	2
AX-157854158	3	67.05	KV452730.1	11751917	2-scf00040	2
AX-157814123	3	67.05	KV452730.1	11756739	2-scf00040	2
AX-157854160	3	67.05	KV452730.1	11769586	2-scf00040	2
AX-157854161	3	67.05	KV452730.1	11777456	2-scf00040	2
AX-157854162	3	67.05	KV452730.1	11777575	2-scf00040	2
AX-157932013	3	67.05	KV452730.1	11797566	2-scf00040	2
AX-157854166	3	67.05	KV452730.1	11809319	2-scf00040	2
AX-157862730	3	67.05	KV452730.1	11819121	2-scf00040	2
AX-157814128	3	67.05	KV452730.1	11820816	2-scf00040	2

AX-157932020	3	67.05	KV452730.1	11826139	2-scf00040	2
AX-157854172	3	67.05	KV452730.1	11832546	2-scf00040	2
AX-157854174	3	67.05	KV452730.1	11839387	2-scf00040	2
AX-157814135	3	67.05	KV452730.1	11885860	2-scf00040	2
AX-157932096	3	67.05	KV452730.1	11904137	2-scf00040	2
AX-157932097	3	67.05	KV452730.1	11905460	2-scf00040	2
AX-157932100	3	67.05	KV452730.1	11906625	2-scf00040	2
AX-157932102	3	67.05	KV452730.1	11907610	2-scf00040	2
AX-157932104	3	67.05	KV452730.1	11908315	2-scf00040	2
AX-157932148	3	67.05	KV452730.1	11933356	2-scf00040	2
AX-157932181	3	67.05	KV452730.1	11952742	2-scf00040	2
AX-157932184	3	67.05	KV452730.1	11953212	2-scf00040	2
AX-157932203	3	67.05	KV452730.1	11968412	2-scf00040	2
AX-157932210	3	67.05	KV452730.1	11972392	2-scf00040	2
AX-157932214	3	67.05	KV452730.1	11973958	2-scf00040	2
AX-157933520	3	67.05	KV452730.1	14011544	2-scf00040	2
AX-158390023	3	67.05	KV452732.1	392087	2-scf00042	2
AX-157813931	3	67.38	KV452730.1	9294633	2-scf00040	2
AX-157930117	3	67.38	KV452730.1	9349667	2-scf00040	2
AX-157930121	3	67.38	KV452730.1	9354359	2-scf00040	2
AX-157930122	3	67.38	KV452730.1	9355164	2-scf00040	2
AX-157930129	3	67.38	KV452730.1	9360702	2-scf00040	2
AX-157930132	3	67.38	KV452730.1	9362747	2-scf00040	2
AX-157930135	3	67.38	KV452730.1	9364924	2-scf00040	2
AX-158389738	3	67.38	KV452730.1	9366071	2-scf00040	2
AX-157930136	3	67.38	KV452730.1	9366317	2-scf00040	2
AX-157930137	3	67.38	KV452730.1	9367929	2-scf00040	2
AX-157930160	3	67.38	KV452730.1	9390999	2-scf00040	2
AX-157930161	3	67.38	KV452730.1	9391631	2-scf00040	2
AX-157930169	3	67.38	KV452730.1	9393612	2-scf00040	2
AX-157930171	3	67.38	KV452730.1	9394654	2-scf00040	2
AX-157930174	3	67.38	KV452730.1	9399706	2-scf00040	2
AX-157930175	3	67.38	KV452730.1	9400898	2-scf00040	2
AX-157930177	3	67.38	KV452730.1	9402117	2-scf00040	2
AX-157930178	3	67.38	KV452730.1	9403804	2-scf00040	2
AX-157930181	3	67.38	KV452730.1	9408888	2-scf00040	2
AX-157813936	3	67.38	KV452730.1	9424162	2-scf00040	2
AX-157930200	3	67.38	KV452730.1	9427407	2-scf00040	2
AX-157930201	3	67.38	KV452730.1	9427998	2-scf00040	2
AX-157930204	3	67.38	KV452730.1	9431091	2-scf00040	2
AX-157930207	3	67.38	KV452730.1	9435851	2-scf00040	2
AX-157930213	3	67.38	KV452730.1	9436372	2-scf00040	2
AX-157930214	3	67.38	KV452730.1	9437692	2-scf00040	2
AX-157930215	3	67.38	KV452730.1	9438081	2-scf00040	2
AX-157930217	3	67.38	KV452730.1	9439879	2-scf00040	2
AX-157930222	3	67.38	KV452730.1	9442746	2-scf00040	2
AX-157930231	3	67.38	KV452730.1	9452524	2-scf00040	2
AX-157930232	3	67.38	KV452730.1	9452843	2-scf00040	2
AX-157930237	3	67.38	KV452730.1	9454739	2-scf00040	2
AX-157874882	3	67.38	KV452730.1	9470763	2-scf00040	2
AX-157930250	3	67.38	KV452730.1	9473484	2-scf00040	2

AX-157930255	3	67.38	KV452730.1	9483225	2-scf00040	2
AX-158389740	3	67.38	KV452730.1	9488137	2-scf00040	2
AX-157930269	3	67.38	KV452730.1	9505857	2-scf00040	2
AX-157930286	3	67.38	KV452730.1	9538945	2-scf00040	2
AX-157930301	3	67.38	KV452730.1	9560386	2-scf00040	2
AX-157930311	3	67.38	KV452730.1	9582459	2-scf00040	2
AX-157930312	3	67.38	KV452730.1	9583024	2-scf00040	2
AX-157930313	3	67.38	KV452730.1	9583525	2-scf00040	2
AX-157930316	3	67.38	KV452730.1	9584952	2-scf00040	2
AX-157930323	3	67.38	KV452730.1	9589251	2-scf00040	2
AX-157930325	3	67.38	KV452730.1	9589690	2-scf00040	2
AX-157930327	3	67.38	KV452730.1	9590523	2-scf00040	2
AX-157930336	3	67.38	KV452730.1	9598103	2-scf00040	2
AX-157930341	3	67.38	KV452730.1	9608605	2-scf00040	2
AX-157930345	3	67.38	KV452730.1	9611510	2-scf00040	2
AX-157930346	3	67.38	KV452730.1	9612908	2-scf00040	2
AX-157930350	3	67.38	KV452730.1	9616207	2-scf00040	2
AX-157930359	3	67.38	KV452730.1	9630606	2-scf00040	2
AX-158414982	3	67.38	KV452730.1	9661144	2-scf00040	2
AX-157930451	3	67.38	KV452730.1	9822012	2-scf00040	2
AX-157930452	3	67.38	KV452730.1	9822417	2-scf00040	2
AX-157813966	3	67.38	KV452730.1	9832251	2-scf00040	2
AX-157930459	3	67.38	KV452730.1	9847883	2-scf00040	2
AX-157930462	3	67.38	KV452730.1	9858227	2-scf00040	2
AX-157930484	3	67.38	KV452730.1	9892610	2-scf00040	2
AX-157930515	3	67.38	KV452730.1	9956128	2-scf00040	2
AX-157930554	3	67.38	KV452730.1	10016148	2-scf00040	2
AX-157874889	3	67.38	KV452730.1	10018671	2-scf00040	2
AX-157930558	3	67.38	KV452730.1	10019373	2-scf00040	2
AX-157930560	3	67.38	KV452730.1	10019799	2-scf00040	2
AX-157930609	3	67.38	KV452730.1	10077291	2-scf00040	2
AX-157930686	3	67.38	KV452730.1	10176519	2-scf00040	2
AX-157930699	3	67.38	KV452730.1	10196947	2-scf00040	2
AX-157930763	3	67.38	KV452730.1	10291903	2-scf00040	2
AX-157930772	3	67.38	KV452730.1	10309729	2-scf00040	2
AX-157930778	3	67.38	KV452730.1	10314331	2-scf00040	2
AX-157717741	3	67.38	KV452730.1	10324911	2-scf00040	2
AX-157930794	3	67.38	KV452730.1	10325744	2-scf00040	2
AX-157930846	3	67.38	KV452730.1	10377001	2-scf00040	2
AX-157930870	3	67.38	KV452730.1	10428512	2-scf00040	2
AX-157930871	3	67.38	KV452730.1	10429213	2-scf00040	2
AX-157930986	3	67.38	KV452730.1	10556998	2-scf00040	2
AX-157814018	3	67.38	KV452730.1	10560567	2-scf00040	2
AX-157931031	3	67.38	KV452730.1	10589932	2-scf00040	2
AX-158405506	3	67.38	KV452730.1	10604797	2-scf00040	2
AX-157931080	3	67.38	KV452730.1	10630284	2-scf00040	2
AX-157931136	3	67.38	KV452730.1	10691038	2-scf00040	2
AX-157931138	3	67.38	KV452730.1	10692051	2-scf00040	2
AX-157931149	3	67.38	KV452730.1	10699599	2-scf00040	2
AX-157931162	3	67.38	KV452730.1	10715077	2-scf00040	2
AX-157883714	3	67.38	KV452730.1	10727863	2-scf00040	2

AX-157931203	3	67.38	KV452730.1	10763375	2-scf00040	2
AX-157869246	3	67.38	KV452730.1	10810153	2-scf00040	2
AX-157931248	3	67.38	KV452730.1	10811977	2-scf00040	2
AX-157869247	3	67.38	KV452730.1	10812985	2-scf00040	2
AX-157814045	3	67.38	KV452730.1	10813511	2-scf00040	2
AX-157931336	3	67.38	KV452730.1	10939516	2-scf00040	2
AX-157931419	3	67.38	KV452730.1	11012607	2-scf00040	2
AX-157931601	3	67.38	KV452730.1	11221829	2-scf00040	2
AX-157931616	3	67.38	KV452730.1	11241564	2-scf00040	2
AX-157931674	3	67.38	KV452730.1	11292209	2-scf00040	2
AX-157931680	3	67.38	KV452730.1	11298969	2-scf00040	2
AX-157931827	3	67.38	KV452730.1	11499417	2-scf00040	2
AX-157931937	3	67.38	KV452730.1	11683334	2-scf00040	2
AX-157814120	3	67.38	KV452730.1	11749770	2-scf00040	2
AX-157854171	3	67.38	KV452730.1	11832050	2-scf00040	2
AX-157932030	3	67.38	KV452730.1	11839679	2-scf00040	2
AX-157814129	3	67.38	KV452730.1	11843976	2-scf00040	2
AX-157932073	3	67.38	KV452730.1	11870186	2-scf00040	2
AX-157932149	3	67.38	KV452730.1	11933855	2-scf00040	2
AX-157932155	3	67.38	KV452730.1	11937340	2-scf00040	2
AX-157932156	3	67.38	KV452730.1	11938520	2-scf00040	2
AX-157932168	3	67.38	KV452730.1	11943475	2-scf00040	2
AX-157932186	3	67.38	KV452730.1	11957160	2-scf00040	2
AX-157932192	3	67.38	KV452730.1	11959758	2-scf00040	2
AX-157932224	3	67.38	KV452730.1	11979854	2-scf00040	2
AX-157814140	3	67.38	KV452730.1	11993850	2-scf00040	2
AX-157932295	3	67.38	KV452730.1	12057501	2-scf00040	2
AX-158405540	3	67.38	KV452730.1	12068882	2-scf00040	2
AX-157932343	3	67.38	KV452730.1	12141694	2-scf00040	2
AX-157932372	3	67.38	KV452730.1	12183831	2-scf00040	2
AX-157932373	3	67.38	KV452730.1	12184253	2-scf00040	2
AX-157932549	3	67.38	KV452730.1	12374930	2-scf00040	2
AX-157932606	3	67.38	KV452730.1	12444688	2-scf00040	2
AX-157932607	3	67.38	KV452730.1	12449419	2-scf00040	2
AX-157932623	3	67.38	KV452730.1	12464304	2-scf00040	2
AX-157932627	3	67.38	KV452730.1	12483178	2-scf00040	2
AX-157932839	3	67.38	KV452730.1	12918080	2-scf00040	2
AX-157874966	3	67.38	KV452730.1	13005166	2-scf00040	2
AX-157757920	3	67.38	KV452730.1	13040744	2-scf00040	2
AX-157932954	3	67.38	KV452730.1	13114179	2-scf00040	2
AX-157932960	3	67.38	KV452730.1	13124392	2-scf00040	2
AX-157932963	3	67.38	KV452730.1	13129167	2-scf00040	2
AX-158389914	3	67.38	KV452730.1	13186684	2-scf00040	2
AX-157933021	3	67.38	KV452730.1	13209953	2-scf00040	2
AX-157933203	3	67.38	KV452730.1	13529417	2-scf00040	2
AX-157933208	3	67.38	KV452730.1	13539379	2-scf00040	2
AX-157933218	3	67.38	KV452730.1	13546742	2-scf00040	2
AX-157933258	3	67.38	KV452730.1	13673377	2-scf00040	2
AX-157933262	3	67.38	KV452730.1	13678268	2-scf00040	2
AX-157933278	3	67.38	KV452730.1	13701229	2-scf00040	2
AX-157874980	3	67.38	KV452730.1	13776757	2-scf00040	2

AX-157874981	3	67.38	KV452730.1	13788625	2-scf00040	2
AX-157933343	3	67.38	KV452730.1	13788772	2-scf00040	2
AX-157874982	3	67.38	KV452730.1	13792099	2-scf00040	2
AX-157933360	3	67.38	KV452730.1	13820815	2-scf00040	2
AX-157933365	3	67.38	KV452730.1	13829913	2-scf00040	2
AX-157933369	3	67.38	KV452730.1	13837185	2-scf00040	2
AX-157933479	3	67.38	KV452730.1	13972922	2-scf00040	2
AX-157933503	3	67.38	KV452730.1	13994585	2-scf00040	2
AX-158382507	3	67.38	KV452730.1	14050442	2-scf00040	2
AX-157883790	3	67.38	KV452730.1	14079658	2-scf00040	2
AX-157933578	3	67.38	KV452730.1	14090162	2-scf00040	2
AX-157933611	3	67.38	KV452730.1	14139137	2-scf00040	2
AX-157933616	3	67.38	KV452730.1	14140627	2-scf00040	2
AX-157933619	3	67.38	KV452730.1	14143896	2-scf00040	2
AX-157933620	3	67.38	KV452730.1	14145530	2-scf00040	2
AX-157933645	3	67.38	KV452730.1	14163930	2-scf00040	2
AX-157933685	3	67.38	KV452730.1	14225391	2-scf00040	2
AX-157933687	3	67.38	KV452730.1	14229602	2-scf00040	2
AX-157933720	3	67.38	KV452730.1	14291684	2-scf00040	2
AX-157933726	3	67.38	KV452730.1	14301886	2-scf00040	2
AX-157933728	3	67.38	KV452730.1	14303803	2-scf00040	2
AX-157933735	3	67.38	KV452730.1	14342817	2-scf00040	2
AX-157933736	3	67.38	KV452730.1	14343780	2-scf00040	2
AX-157933745	3	67.38	KV452730.1	14365168	2-scf00040	2
AX-157933749	3	67.38	KV452730.1	14414923	2-scf00040	2
AX-157933751	3	67.38	KV452730.1	14415871	2-scf00040	2
AX-157933752	3	67.38	KV452730.1	14416944	2-scf00040	2
AX-157933806	3	67.38	KV452730.1	14527397	2-scf00040	2
AX-157933808	3	67.38	KV452730.1	14529379	2-scf00040	2
AX-157933812	3	67.38	KV452730.1	14537076	2-scf00040	2
AX-157933818	3	67.38	KV452730.1	14551097	2-scf00040	2
AX-157933825	3	67.38	KV452730.1	14572490	2-scf00040	2
AX-157933828	3	67.38	KV452730.1	14573363	2-scf00040	2
AX-157933831	3	67.38	KV452730.1	14575490	2-scf00040	2
AX-157933837	3	67.38	KV452730.1	14589780	2-scf00040	2
AX-157933839	3	67.38	KV452730.1	14596323	2-scf00040	2
AX-157933841	3	67.38	KV452730.1	14599004	2-scf00040	2
AX-157933842	3	67.38	KV452730.1	14604536	2-scf00040	2
AX-157933843	3	67.38	KV452730.1	14609823	2-scf00040	2
AX-157933845	3	67.38	KV452730.1	14611158	2-scf00040	2
AX-157933847	3	67.38	KV452730.1	14613544	2-scf00040	2
AX-157933850	3	67.38	KV452730.1	14620512	2-scf00040	2
AX-157933851	3	67.38	KV452730.1	14621067	2-scf00040	2
AX-157933860	3	67.38	KV452730.1	14633724	2-scf00040	2
AX-157814266	3	67.38	KV452730.1	14634425	2-scf00040	2
AX-157933865	3	67.38	KV452730.1	14644491	2-scf00040	2
AX-157933870	3	67.38	KV452730.1	14670146	2-scf00040	2
AX-157933871	3	67.38	KV452730.1	14672986	2-scf00040	2
AX-157933874	3	67.38	KV452730.1	14675055	2-scf00040	2
AX-157933876	3	67.38	KV452730.1	14682056	2-scf00040	2
AX-157933905	3	67.38	KV452733.1	30604	2-scf00041	2

AX-157933942	3	67.38	KV452733.1	69365	2-scf00041	2
AX-157933949	3	67.38	KV452733.1	74854	2-scf00041	2
AX-157934074	3	67.38	KV452733.1	196203	2-scf00041	2
AX-157934077	3	67.38	KV452733.1	197989	2-scf00041	2
AX-157934319	3	67.38	KV452733.1	456261	2-scf00041	2
AX-157934337	3	67.38	KV452733.1	474219	2-scf00041	2
AX-157744913	3	67.38	KV452733.1	475611	2-scf00041	2
AX-157934341	3	67.38	KV452733.1	482156	2-scf00041	2
AX-157934372	3	67.38	KV452733.1	500655	2-scf00041	2
AX-157934385	3	67.38	KV452733.1	520760	2-scf00041	2
AX-157934514	3	67.38	KV452733.1	634912	2-scf00041	2
AX-157934563	3	67.38	KV452733.1	687355	2-scf00041	2
AX-157934624	3	67.38	KV452733.1	762432	2-scf00041	2
AX-157740775	3	67.38	KV452733.1	892984	2-scf00041	2
AX-157934869	3	67.38	KV452733.1	999839	2-scf00041	2
AX-157883805	3	67.38	KV452733.1	1010692	2-scf00041	2
AX-157935005	3	67.38	KV452733.1	1123198	2-scf00041	2
AX-157745560	3	67.38	KV452733.1	1123824	2-scf00041	2
AX-157713147	3	67.38	KV452733.1	1205561	2-scf00041	2
AX-157883812	3	67.38	KV452733.1	1225009	2-scf00041	2
AX-158390005	3	67.38	KV452733.1	1266407	2-scf00041	2
AX-157814326	3	67.38	KV452733.1	1281162	2-scf00041	2
AX-157935165	3	67.38	KV452733.1	1287497	2-scf00041	2
AX-157935188	3	67.38	KV452733.1	1319796	2-scf00041	2
AX-157935189	3	67.38	KV452733.1	1320003	2-scf00041	2
AX-157935210	3	67.38	KV452733.1	1341323	2-scf00041	2
AX-157935223	3	67.38	KV452733.1	1357860	2-scf00041	2
AX-157709972	3	67.38	KV452733.1	1360839	2-scf00041	2
AX-157935343	3	67.38	KV452733.1	1476011	2-scf00041	2
AX-157935647	3	67.38	KV452733.1	1851442	2-scf00041	2
AX-157883820	3	67.38	KV452733.1	1920214	2-scf00041	2
AX-157935716	3	67.38	KV452733.1	1957766	2-scf00041	2
AX-157935800	3	67.38	KV452733.1	2086757	2-scf00041	2
AX-157710735	3	67.38	KV452733.1	2182019	2-scf00041	2
AX-158382549	3	67.38	KV452733.1	2287208	2-scf00041	2
AX-157814374	3	67.38	KV452733.1	2299453	2-scf00041	2
AX-157935922	3	67.38	KV452733.1	2309447	2-scf00041	2
AX-157935929	3	67.38	KV452733.1	2314053	2-scf00041	2
AX-157935938	3	67.38	KV452733.1	2333083	2-scf00041	2
AX-157936017	3	67.38	KV452733.1	2412650	2-scf00041	2
AX-157875089	3	67.38	KV452733.1	2420869	2-scf00041	2
AX-157869439	3	67.38	KV452733.1	2465770	2-scf00041	2
AX-157936138	3	67.38	KV452733.1	2521736	2-scf00041	2
AX-157936146	3	67.38	KV452733.1	2528450	2-scf00041	2
AX-157698550	3	67.38	KV452733.1	2530950	2-scf00041	2
AX-157936152	3	67.38	KV452733.1	2542408	2-scf00041	2
AX-157936180	3	67.38	KV452733.1	2571331	2-scf00041	2
AX-157936276	3	67.38	KV452733.1	2660607	2-scf00041	2
AX-157775122	3	67.38	KV452733.1	2706832	2-scf00041	2
AX-157936360	3	67.38	KV452733.1	2736158	2-scf00041	2
AX-157936408	3	67.38	KV452733.1	2779290	2-scf00041	2

AX-157936430	3	67.38	KV452733.1	2798183	2-scf00041	2
AX-157936456	3	67.38	KV452733.1	2825189	2-scf00041	2
AX-157814400	3	67.38	KV452733.1	2873063	2-scf00041	2
AX-157936532	3	67.38	KV452733.1	2912003	2-scf00041	2
AX-157814408	3	67.38	KV452733.1	2965872	2-scf00041	2
AX-157936608	3	67.38	KV452733.1	3000052	2-scf00041	2
AX-157936710	3	67.38	KV452733.1	3101952	2-scf00041	2
AX-157936718	3	67.38	KV452733.1	3109729	2-scf00041	2
AX-157875102	3	67.38	KV452733.1	3126745	2-scf00041	2
AX-157936745	3	67.38	KV452733.1	3140061	2-scf00041	2
AX-157936775	3	67.38	KV452733.1	3172990	2-scf00041	2
AX-158382568	3	67.38	KV452733.1	3251989	2-scf00041	2
AX-158382569	3	67.38	KV452733.1	3284437	2-scf00041	2
AX-157936990	3	67.38	KV452733.1	3360106	2-scf00041	2
AX-157937028	3	67.38	KV452733.1	3390995	2-scf00041	2
AX-157937064	3	67.38	KV452733.1	3420750	2-scf00041	2
AX-157937076	3	67.38	KV452733.1	3430778	2-scf00041	2
AX-157864719	3	67.38	KV452733.1	3487637	2-scf00041	2
AX-157937136	3	67.38	KV452733.1	3488577	2-scf00041	2
AX-157937196	3	67.38	KV452733.1	3541527	2-scf00041	2
AX-157937199	3	67.38	KV452733.1	3545734	2-scf00041	2
AX-157750580	3	67.38	KV452733.1	3675484	2-scf00041	2
AX-157937319	3	67.38	KV452733.1	3687578	2-scf00041	2
AX-157937329	3	67.38	KV452733.1	3702553	2-scf00041	2
AX-157864720	3	67.38	KV452733.1	3713931	2-scf00041	2
AX-157937448	3	67.38	KV452732.1	2140	2-scf00042	2
AX-157937450	3	67.38	KV452732.1	5857	2-scf00042	2
AX-157937451	3	67.38	KV452732.1	7653	2-scf00042	2
AX-157937453	3	67.38	KV452732.1	11965	2-scf00042	2
AX-157883846	3	67.38	KV452732.1	32498	2-scf00042	2
AX-157937478	3	67.38	KV452732.1	71791	2-scf00042	2
AX-157937521	3	67.38	KV452732.1	234056	2-scf00042	2
AX-158405657	3	67.38	KV452732.1	272362	2-scf00042	2
AX-157937548	3	67.38	KV452732.1	295552	2-scf00042	2
AX-157937561	3	67.38	KV452732.1	312260	2-scf00042	2
AX-157937610	3	67.38	KV452732.1	395193	2-scf00042	2
AX-157937755	3	67.38	KV452732.1	680904	2-scf00042	2
AX-157937761	3	67.38	KV452732.1	693499	2-scf00042	2
AX-157814477	3	67.38	KV452732.1	711655	2-scf00042	2
AX-157937800	3	67.38	KV452732.1	780910	2-scf00042	2
AX-157814491	3	67.38	KV452732.1	847350	2-scf00042	2
AX-157814514	3	67.38	KV452732.1	1167176	2-scf00042	2
AX-157938162	3	67.38	KV452732.1	1443134	2-scf00042	2
AX-157938164	3	67.38	KV452732.1	1449434	2-scf00042	2
AX-157938276	3	67.38	KV452735.1	33583	2-scf00044	2
AX-157722904	3	67.38	KV452735.1	36758	2-scf00044	2
AX-157938378	3	67.38	KV452735.1	118888	2-scf00044	2
AX-157814567	3	67.38	KV452742.1	142179	2-scf00046	2
AX-157869451	3	67.38	KV452742.1	142601	2-scf00046	2
AX-157814568	3	67.38	KV452742.1	143320	2-scf00046	2
AX-157938557	3	67.38	KV452742.1	146600	2-scf00046	2

AX-157712337	3	67.38	KV452742.1	156701	2-scf00046	2
AX-157938616	3	67.38	KV452742.1	206914	2-scf00046	2
AX-157938629	3	67.38	KV452742.1	220006	2-scf00046	2
AX-157938630	3	67.38	KV452742.1	221187	2-scf00046	2
AX-157938803	3	67.38	KV452742.1	452598	2-scf00046	2
AX-157938813	3	67.38	KV452742.1	465241	2-scf00046	2
AX-157938819	3	67.38	KV452742.1	470998	2-scf00046	2
AX-157938879	3	67.38	KV452742.1	521633	2-scf00046	2
AX-157814616	3	67.38	KV452742.1	541489	2-scf00046	2
AX-157938901	3	67.38	KV452742.1	550759	2-scf00046	2
AX-157774387	3	67.38	KV452742.1	563881	2-scf00046	2
AX-157938972	3	67.38	KV452742.1	615919	2-scf00046	2
AX-157938981	3	67.38	KV452742.1	625945	2-scf00046	2
AX-157939070	3	67.38	KV452742.1	766286	2-scf00046	2
AX-157939075	3	67.38	KV452742.1	780853	2-scf00046	2
AX-157939127	3	67.38	KV452742.1	911246	2-scf00046	2
AX-157939144	3	67.38	KV452742.1	956926	2-scf00046	2
AX-157875142	3	67.38	KV452742.1	1034416	2-scf00046	2
AX-157939180	3	67.38	KV452742.1	1077791	2-scf00046	2
AX-157875143	3	67.38	KV452742.1	1082676	2-scf00046	2
AX-157939411	3	67.38	KV452742.1	1379519	2-scf00046	2
AX-158382607	3	67.38	KV452742.1	1398805	2-scf00046	2
AX-157939433	3	67.38	KV452742.1	1402044	2-scf00046	2
AX-158382608	3	67.38	KV452742.1	1405300	2-scf00046	2
AX-157939541	3	67.38	KV452742.1	1512084	2-scf00046	2
AX-158382609	3	67.38	KV452742.1	1517240	2-scf00046	2
AX-157749580	3	67.38	KV452742.1	1527681	2-scf00046	2
AX-158382610	3	67.38	KV452742.1	1587119	2-scf00046	2
AX-158382611	3	67.38	KV452742.1	1590454	2-scf00046	2
AX-157939616	3	67.38	KV452742.1	1610110	2-scf00046	2
AX-157941332	3	67.38	KV452742.1	1690485	2-scf00046	2
AX-157751251	3	67.38	KV452742.1	1713582	2-scf00046	2
AX-157941253	3	67.38	KV452742.1	1755767	2-scf00046	2
AX-157939743	3	67.38	KV452742.1	2639166	2-scf00046	2
AX-157749400	3	67.38	KV452742.1	2787431	2-scf00046	2
AX-157939784	3	67.38	KV452742.1	2803161	2-scf00046	2
AX-157712264	3	67.38	KV452742.1	2912141	2-scf00046	2
AX-157939913	3	67.38	KV452742.1	2994629	2-scf00046	2
AX-157939939	3	67.38	KV452742.1	3017665	2-scf00046	2
AX-157939942	3	67.38	KV452742.1	3019520	2-scf00046	2
AX-157939985	3	67.38	KV452742.1	3057092	2-scf00046	2
AX-157939993	3	67.38	KV452742.1	3069906	2-scf00046	2
AX-157940100	3	67.38	KV452742.1	3173731	2-scf00046	2
AX-157940124	3	67.38	KV452742.1	3197817	2-scf00046	2
AX-158390047	3	67.38	KV452742.1	3204616	2-scf00046	2
AX-157940334	3	67.38	KV452742.1	3440808	2-scf00046	2
AX-157940349	3	67.38	KV452742.1	3474368	2-scf00046	2
AX-157940426	3	67.38	KV452742.1	3576805	2-scf00046	2
AX-157814733	3	67.38	KV452742.1	3621509	2-scf00046	2
AX-157940465	3	67.38	KV452742.1	3626820	2-scf00046	2
AX-158405756	3	67.38	KV452742.1	3794114	2-scf00046	2

AX-157940581	3	67.38	KV452742.1	3800341	2-scf00046	2
AX-158405762	3	67.38	KV452742.1	4070953	2-scf00046	2
AX-158390053	3	67.38	KV452742.1	4096639	2-scf00046	2
AX-157940703	3	67.38	KV452742.1	4097275	2-scf00046	2
AX-157875169	3	67.38	KV452742.1	4296579	2-scf00046	2
AX-157940875	3	67.38	KV452742.1	4388229	2-scf00046	2
AX-157940916	3	67.38	KV452742.1	4440797	2-scf00046	2
AX-157941405	3	67.38	KV452743.1	53712	2-scf00050	2
AX-157941512	3	67.38	KV452745.1	1894	2-scf00051	2
AX-157941556	3	67.38	KV452745.1	45873	2-scf00051	2
AX-157941657	3	67.38	KV452745.1	164286	2-scf00051	2
AX-157941662	3	67.38	KV452745.1	167085	2-scf00051	2
AX-157941735	3	67.38	KV452745.1	217258	2-scf00051	2
AX-157941942	3	67.38	KV452745.1	377673	2-scf00051	2
AX-157941996	3	67.38	KV452745.1	417566	2-scf00051	2
AX-157883903	3	67.38	KV452745.1	689776	2-scf00051	2
AX-157942433	3	67.38	KV452745.1	828059	2-scf00051	2
AX-157942591	3	67.38	KV452745.1	997191	2-scf00051	2
AX-157942597	3	67.38	KV452745.1	1002890	2-scf00051	2
AX-157875196	3	67.38	KV452745.1	1029398	2-scf00051	2
AX-157942615	3	67.38	KV452745.1	1037803	2-scf00051	2
AX-157942952	3	67.38	KV452744.1	19212	2-scf00052	2
AX-157942953	3	67.38	KV452744.1	19500	2-scf00052	2
AX-157943056	3	67.38	KV452744.1	184891	2-scf00052	2
AX-158390128	3	67.38	KV452747.1	521694	2-scf00053	2
AX-157943562	3	67.38	KV452747.1	525426	2-scf00053	2
AX-157814947	3	67.38	KV452747.1	533002	2-scf00053	2
AX-157943682	3	67.38	KV452747.1	639985	2-scf00053	2
AX-157943714	3	67.38	KV452747.1	684154	2-scf00053	2
AX-157943716	3	67.38	KV452747.1	687905	2-scf00053	2
AX-157814960	3	67.38	KV452747.1	688456	2-scf00053	2
AX-157943759	3	67.38	KV452747.1	749973	2-scf00053	2
AX-157943773	3	67.38	KV452747.1	774961	2-scf00053	2
AX-157943808	3	67.38	KV452747.1	812357	2-scf00053	2
AX-157943827	3	67.38	KV452747.1	824769	2-scf00053	2
AX-157943897	3	67.38	KV452747.1	888218	2-scf00053	2
AX-157943901	3	67.38	KV452747.1	893460	2-scf00053	2
AX-157945321	3	67.38	KV452746.1	173856	2-scf00054	2
AX-157889309	3	67.38	KV452740.1	286173	2-scf00586	2
AX-157703201	3	67.38	DX096831.1	75	IORI212 Ictalu	UN
AX-157704550	3	67.38	3ML01026525	342	scf02122_1	UN
AX-157706110	3	67.38	3ML01031624	73	scf07241_1	UN
AX-157706023	3	67.38	3ML01033587	256	scf09244_1	UN
AX-157930220	3	67.436	KV452730.1	9441544	2-scf00040	2
AX-157930113	3	67.487	KV452730.1	9347896	2-scf00040	2
AX-157930149	3	67.487	KV452730.1	9377450	2-scf00040	2
AX-158389739	3	67.487	KV452730.1	9408169	2-scf00040	2
AX-157930182	3	67.487	KV452730.1	9410347	2-scf00040	2
AX-157930189	3	67.487	KV452730.1	9416275	2-scf00040	2
AX-157930205	3	67.487	KV452730.1	9433264	2-scf00040	2
AX-157874881	3	67.487	KV452730.1	9445865	2-scf00040	2

AX-157930227	3	67.487	KV452730.1	9449253	2-scf00040	2
AX-157930229	3	67.487	KV452730.1	9451814	2-scf00040	2
AX-157930230	3	67.487	KV452730.1	9452298	2-scf00040	2
AX-157930234	3	67.487	KV452730.1	9453609	2-scf00040	2
AX-157930248	3	67.487	KV452730.1	9470465	2-scf00040	2
AX-157709596	3	67.487	KV452730.1	9472110	2-scf00040	2
AX-157930251	3	67.487	KV452730.1	9475553	2-scf00040	2
AX-157930256	3	67.487	KV452730.1	9485905	2-scf00040	2
AX-157813938	3	67.487	KV452730.1	9486036	2-scf00040	2
AX-157813939	3	67.487	KV452730.1	9486152	2-scf00040	2
AX-157813940	3	67.487	KV452730.1	9486279	2-scf00040	2
AX-157930257	3	67.487	KV452730.1	9486381	2-scf00040	2
AX-157930260	3	67.487	KV452730.1	9491614	2-scf00040	2
AX-157930261	3	67.487	KV452730.1	9492428	2-scf00040	2
AX-157930264	3	67.487	KV452730.1	9495798	2-scf00040	2
AX-157813943	3	67.487	KV452730.1	9496195	2-scf00040	2
AX-157813944	3	67.487	KV452730.1	9497371	2-scf00040	2
AX-157745330	3	67.487	KV452730.1	9499479	2-scf00040	2
AX-157930267	3	67.487	KV452730.1	9502772	2-scf00040	2
AX-157930268	3	67.487	KV452730.1	9503636	2-scf00040	2
AX-157930270	3	67.487	KV452730.1	9506115	2-scf00040	2
AX-157813945	3	67.487	KV452730.1	9506959	2-scf00040	2
AX-157930328	3	67.487	KV452730.1	9591144	2-scf00040	2
AX-157757772	3	67.487	KV452730.1	10038557	2-scf00040	2
AX-157813970	3	67.487	KV452730.1	10041729	2-scf00040	2
AX-157930582	3	67.487	KV452730.1	10047414	2-scf00040	2
AX-157930945	3	67.487	KV452730.1	10506470	2-scf00040	2
AX-157814026	3	67.487	KV452730.1	10700687	2-scf00040	2
AX-157709380	3	67.487	KV452730.1	10717161	2-scf00040	2
AX-157874926	3	67.487	KV452730.1	11572351	2-scf00040	2
AX-157854147	3	67.487	KV452730.1	11726461	2-scf00040	2
AX-157931996	3	67.487	KV452730.1	11777330	2-scf00040	2
AX-157932209	3	67.487	KV452730.1	11971791	2-scf00040	2
AX-157874984	3	67.487	KV452730.1	13867996	2-scf00040	2
AX-157934821	3	67.487	KV452733.1	946311	2-scf00041	2
AX-157937129	3	67.487	KV452733.1	3483063	2-scf00041	2
AX-157941074	3	67.487	KV452742.1	1966523	2-scf00046	2
AX-157930184	3	67.498	KV452730.1	9411068	2-scf00040	2
AX-157930235	3	67.498	KV452730.1	9454480	2-scf00040	2
AX-157930275	3	67.498	KV452730.1	9516016	2-scf00040	2
AX-157930334	3	67.498	KV452730.1	9597301	2-scf00040	2
AX-157813949	3	67.498	KV452730.1	9618329	2-scf00040	2
AX-157930910	3	67.498	KV452730.1	10475711	2-scf00040	2
AX-157930277	3	67.511	KV452730.1	9518427	2-scf00040	2
AX-157930284	3	67.528	KV452730.1	9537168	2-scf00040	2
AX-157930288	3	67.546	KV452730.1	9541141	2-scf00040	2
AX-157930296	3	67.562	KV452730.1	9552493	2-scf00040	2
AX-157930314	3	67.576	KV452730.1	9583992	2-scf00040	2
AX-157930508	3	67.576	KV452730.1	9942020	2-scf00040	2
AX-157930319	3	67.586	KV452730.1	9585499	2-scf00040	2
AX-157930331	3	67.596	KV452730.1	9592143	2-scf00040	2

AX-157930337	3	67.596	KV452730.1	9599032	2-scf00040	2
AX-157930339	3	67.596	KV452730.1	9605027	2-scf00040	2
AX-157930340	3	67.596	KV452730.1	9606589	2-scf00040	2
AX-157930343	3	67.596	KV452730.1	9611301	2-scf00040	2
AX-157931901	3	67.596	KV452730.1	11610243	2-scf00040	2
AX-157940497	3	67.596	KV452742.1	3658194	2-scf00046	2
AX-157930287	3	67.62	KV452730.1	9540415	2-scf00040	2
AX-157930347	3	67.62	KV452730.1	9614034	2-scf00040	2
AX-157930463	3	67.62	KV452730.1	9858551	2-scf00040	2
AX-157930464	3	67.62	KV452730.1	9859211	2-scf00040	2
AX-157930845	3	67.62	KV452730.1	10375524	2-scf00040	2
AX-157930960	3	67.62	KV452730.1	10533652	2-scf00040	2
AX-157930369	3	67.662	KV452730.1	9659657	2-scf00040	2
AX-157929971	3	67.705	KV452730.1	9223401	2-scf00040	2
AX-157930104	3	67.705	KV452730.1	9338689	2-scf00040	2
AX-157930141	3	67.705	KV452730.1	9368839	2-scf00040	2
AX-157813935	3	67.705	KV452730.1	9412129	2-scf00040	2
AX-157738293	3	67.705	KV452730.1	9458524	2-scf00040	2
AX-157930252	3	67.705	KV452730.1	9477230	2-scf00040	2
AX-157930253	3	67.705	KV452730.1	9479037	2-scf00040	2
AX-157721256	3	67.705	KV452730.1	9487984	2-scf00040	2
AX-157724552	3	67.705	KV452730.1	9563471	2-scf00040	2
AX-157930322	3	67.705	KV452730.1	9588651	2-scf00040	2
AX-157930357	3	67.705	KV452730.1	9620678	2-scf00040	2
AX-157813950	3	67.705	KV452730.1	9625510	2-scf00040	2
AX-157930362	3	67.705	KV452730.1	9644426	2-scf00040	2
AX-157930366	3	67.705	KV452730.1	9650500	2-scf00040	2
AX-157930370	3	67.705	KV452730.1	9663303	2-scf00040	2
AX-157930372	3	67.705	KV452730.1	9663523	2-scf00040	2
AX-157930374	3	67.705	KV452730.1	9664424	2-scf00040	2
AX-157930375	3	67.705	KV452730.1	9665144	2-scf00040	2
AX-157930378	3	67.705	KV452730.1	9673334	2-scf00040	2
AX-157930384	3	67.705	KV452730.1	9683435	2-scf00040	2
AX-157930390	3	67.705	KV452730.1	9689790	2-scf00040	2
AX-157930391	3	67.705	KV452730.1	9690586	2-scf00040	2
AX-157930392	3	67.705	KV452730.1	9697008	2-scf00040	2
AX-157930393	3	67.705	KV452730.1	9700877	2-scf00040	2
AX-157930396	3	67.705	KV452730.1	9703872	2-scf00040	2
AX-157930397	3	67.705	KV452730.1	9706136	2-scf00040	2
AX-157930401	3	67.705	KV452730.1	9717301	2-scf00040	2
AX-157930404	3	67.705	KV452730.1	9721473	2-scf00040	2
AX-157930406	3	67.705	KV452730.1	9722792	2-scf00040	2
AX-157930407	3	67.705	KV452730.1	9728673	2-scf00040	2
AX-157930408	3	67.705	KV452730.1	9729883	2-scf00040	2
AX-157813959	3	67.705	KV452730.1	9734949	2-scf00040	2
AX-157813962	3	67.705	KV452730.1	9782423	2-scf00040	2
AX-157930419	3	67.705	KV452730.1	9790006	2-scf00040	2
AX-157930420	3	67.705	KV452730.1	9790944	2-scf00040	2
AX-157813963	3	67.705	KV452730.1	9791086	2-scf00040	2
AX-157930421	3	67.705	KV452730.1	9791558	2-scf00040	2
AX-157930422	3	67.705	KV452730.1	9793125	2-scf00040	2

AX-157930424	3	67.705	KV452730.1	9794722	2-scf00040	2
AX-157748265	3	67.705	KV452730.1	9795112	2-scf00040	2
AX-157930425	3	67.705	KV452730.1	9796535	2-scf00040	2
AX-157930428	3	67.705	KV452730.1	9798475	2-scf00040	2
AX-157930430	3	67.705	KV452730.1	9798924	2-scf00040	2
AX-157771982	3	67.705	KV452730.1	9799737	2-scf00040	2
AX-157930431	3	67.705	KV452730.1	9801852	2-scf00040	2
AX-157930433	3	67.705	KV452730.1	9802296	2-scf00040	2
AX-157930435	3	67.705	KV452730.1	9803726	2-scf00040	2
AX-157930436	3	67.705	KV452730.1	9805587	2-scf00040	2
AX-157930438	3	67.705	KV452730.1	9806055	2-scf00040	2
AX-157930439	3	67.705	KV452730.1	9806849	2-scf00040	2
AX-157813964	3	67.705	KV452730.1	9808142	2-scf00040	2
AX-157930441	3	67.705	KV452730.1	9809105	2-scf00040	2
AX-157930444	3	67.705	KV452730.1	9810862	2-scf00040	2
AX-157930445	3	67.705	KV452730.1	9811131	2-scf00040	2
AX-157813965	3	67.705	KV452730.1	9815427	2-scf00040	2
AX-157930450	3	67.705	KV452730.1	9821199	2-scf00040	2
AX-157930453	3	67.705	KV452730.1	9825257	2-scf00040	2
AX-157930455	3	67.705	KV452730.1	9829929	2-scf00040	2
AX-157930456	3	67.705	KV452730.1	9833726	2-scf00040	2
AX-157930457	3	67.705	KV452730.1	9837136	2-scf00040	2
AX-157930460	3	67.705	KV452730.1	9857046	2-scf00040	2
AX-157930466	3	67.705	KV452730.1	9859703	2-scf00040	2
AX-157930470	3	67.705	KV452730.1	9876934	2-scf00040	2
AX-157930471	3	67.705	KV452730.1	9880275	2-scf00040	2
AX-157930473	3	67.705	KV452730.1	9881142	2-scf00040	2
AX-157930492	3	67.705	KV452730.1	9903463	2-scf00040	2
AX-157930511	3	67.705	KV452730.1	9947140	2-scf00040	2
AX-157930525	3	67.705	KV452730.1	9968735	2-scf00040	2
AX-157813967	3	67.705	KV452730.1	9979879	2-scf00040	2
AX-157930531	3	67.705	KV452730.1	9981173	2-scf00040	2
AX-157930533	3	67.705	KV452730.1	9985210	2-scf00040	2
AX-157930542	3	67.705	KV452730.1	9995422	2-scf00040	2
AX-157930550	3	67.705	KV452730.1	10005084	2-scf00040	2
AX-157930562	3	67.705	KV452730.1	10021695	2-scf00040	2
AX-157930563	3	67.705	KV452730.1	10023952	2-scf00040	2
AX-157930575	3	67.705	KV452730.1	10043625	2-scf00040	2
AX-157930577	3	67.705	KV452730.1	10044810	2-scf00040	2
AX-157930584	3	67.705	KV452730.1	10047928	2-scf00040	2
AX-157930592	3	67.705	KV452730.1	10052295	2-scf00040	2
AX-157930593	3	67.705	KV452730.1	10053978	2-scf00040	2
AX-157930595	3	67.705	KV452730.1	10055416	2-scf00040	2
AX-157930596	3	67.705	KV452730.1	10057438	2-scf00040	2
AX-157930601	3	67.705	KV452730.1	10067718	2-scf00040	2
AX-157813974	3	67.705	KV452730.1	10074624	2-scf00040	2
AX-157930607	3	67.705	KV452730.1	10075148	2-scf00040	2
AX-157930612	3	67.705	KV452730.1	10079214	2-scf00040	2
AX-157930613	3	67.705	KV452730.1	10082537	2-scf00040	2
AX-157930615	3	67.705	KV452730.1	10083704	2-scf00040	2
AX-157930619	3	67.705	KV452730.1	10094194	2-scf00040	2

AX-157930620	3	67.705	KV452730.1	10096069	2-scf00040	2
AX-157813979	3	67.705	KV452730.1	10121091	2-scf00040	2
AX-157930651	3	67.705	KV452730.1	10128261	2-scf00040	2
AX-157813985	3	67.705	KV452730.1	10155518	2-scf00040	2
AX-157930700	3	67.705	KV452730.1	10199397	2-scf00040	2
AX-157750874	3	67.705	KV452730.1	10231783	2-scf00040	2
AX-157930726	3	67.705	KV452730.1	10255191	2-scf00040	2
AX-157757104	3	67.705	KV452730.1	10280419	2-scf00040	2
AX-157930751	3	67.705	KV452730.1	10286523	2-scf00040	2
AX-157930758	3	67.705	KV452730.1	10289934	2-scf00040	2
AX-157813994	3	67.705	KV452730.1	10290362	2-scf00040	2
AX-157930760	3	67.705	KV452730.1	10290570	2-scf00040	2
AX-157930762	3	67.705	KV452730.1	10291120	2-scf00040	2
AX-157930765	3	67.705	KV452730.1	10293118	2-scf00040	2
AX-157930785	3	67.705	KV452730.1	10320087	2-scf00040	2
AX-157813997	3	67.705	KV452730.1	10327189	2-scf00040	2
AX-157930798	3	67.705	KV452730.1	10329023	2-scf00040	2
AX-157930799	3	67.705	KV452730.1	10330008	2-scf00040	2
AX-157930809	3	67.705	KV452730.1	10342743	2-scf00040	2
AX-157930810	3	67.705	KV452730.1	10343375	2-scf00040	2
AX-157930812	3	67.705	KV452730.1	10345537	2-scf00040	2
AX-157703018	3	67.705	KV452730.1	10347973	2-scf00040	2
AX-157930818	3	67.705	KV452730.1	10349265	2-scf00040	2
AX-157930822	3	67.705	KV452730.1	10353607	2-scf00040	2
AX-157930824	3	67.705	KV452730.1	10354582	2-scf00040	2
AX-157814000	3	67.705	KV452730.1	10354628	2-scf00040	2
AX-157930828	3	67.705	KV452730.1	10358581	2-scf00040	2
AX-157814002	3	67.705	KV452730.1	10358993	2-scf00040	2
AX-157930830	3	67.705	KV452730.1	10359524	2-scf00040	2
AX-157930831	3	67.705	KV452730.1	10362452	2-scf00040	2
AX-157930834	3	67.705	KV452730.1	10365060	2-scf00040	2
AX-157930835	3	67.705	KV452730.1	10366725	2-scf00040	2
AX-157930838	3	67.705	KV452730.1	10367266	2-scf00040	2
AX-158389745	3	67.705	KV452730.1	10367306	2-scf00040	2
AX-157930839	3	67.705	KV452730.1	10368105	2-scf00040	2
AX-157930840	3	67.705	KV452730.1	10370409	2-scf00040	2
AX-157930841	3	67.705	KV452730.1	10370850	2-scf00040	2
AX-157930843	3	67.705	KV452730.1	10371720	2-scf00040	2
AX-157930859	3	67.705	KV452730.1	10409443	2-scf00040	2
AX-157930860	3	67.705	KV452730.1	10411738	2-scf00040	2
AX-157930864	3	67.705	KV452730.1	10423602	2-scf00040	2
AX-157930866	3	67.705	KV452730.1	10424594	2-scf00040	2
AX-157930868	3	67.705	KV452730.1	10427739	2-scf00040	2
AX-157930874	3	67.705	KV452730.1	10431009	2-scf00040	2
AX-158379346	3	67.705	KV452730.1	10442125	2-scf00040	2
AX-157864641	3	67.705	KV452730.1	10451908	2-scf00040	2
AX-157930904	3	67.705	KV452730.1	10467031	2-scf00040	2
AX-157742761	3	67.705	KV452730.1	10476688	2-scf00040	2
AX-157930914	3	67.705	KV452730.1	10480094	2-scf00040	2
AX-157930918	3	67.705	KV452730.1	10484293	2-scf00040	2
AX-157930927	3	67.705	KV452730.1	10488746	2-scf00040	2

AX-157814008	3	67.705	KV452730.1	10490745	2-scf00040	2
AX-157757309	3	67.705	KV452730.1	10491947	2-scf00040	2
AX-157864642	3	67.705	KV452730.1	10496663	2-scf00040	2
AX-157930934	3	67.705	KV452730.1	10500743	2-scf00040	2
AX-157814010	3	67.705	KV452730.1	10506604	2-scf00040	2
AX-157930957	3	67.705	KV452730.1	10530510	2-scf00040	2
AX-157814013	3	67.705	KV452730.1	10532533	2-scf00040	2
AX-157930973	3	67.705	KV452730.1	10543082	2-scf00040	2
AX-157930978	3	67.705	KV452730.1	10548828	2-scf00040	2
AX-157930980	3	67.705	KV452730.1	10549414	2-scf00040	2
AX-157930981	3	67.705	KV452730.1	10549993	2-scf00040	2
AX-157930985	3	67.705	KV452730.1	10554623	2-scf00040	2
AX-157930990	3	67.705	KV452730.1	10561564	2-scf00040	2
AX-157931009	3	67.705	KV452730.1	10574805	2-scf00040	2
AX-157931010	3	67.705	KV452730.1	10575082	2-scf00040	2
AX-157814019	3	67.705	KV452730.1	10578014	2-scf00040	2
AX-157931033	3	67.705	KV452730.1	10593639	2-scf00040	2
AX-157931045	3	67.705	KV452730.1	10607948	2-scf00040	2
AX-157931100	3	67.705	KV452730.1	10646664	2-scf00040	2
AX-158389758	3	67.705	KV452730.1	10673631	2-scf00040	2
AX-157814029	3	67.705	KV452730.1	10722193	2-scf00040	2
AX-157814033	3	67.705	KV452730.1	10772712	2-scf00040	2
AX-157931231	3	67.705	KV452730.1	10793041	2-scf00040	2
AX-157931233	3	67.705	KV452730.1	10794124	2-scf00040	2
AX-157931251	3	67.705	KV452730.1	10817033	2-scf00040	2
AX-157931252	3	67.705	KV452730.1	10817488	2-scf00040	2
AX-157931275	3	67.705	KV452730.1	10831249	2-scf00040	2
AX-157814054	3	67.705	KV452730.1	10857164	2-scf00040	2
AX-157931325	3	67.705	KV452730.1	10916320	2-scf00040	2
AX-157931328	3	67.705	KV452730.1	10927405	2-scf00040	2
AX-157931329	3	67.705	KV452730.1	10928671	2-scf00040	2
AX-157931368	3	67.705	KV452730.1	10967141	2-scf00040	2
AX-157931440	3	67.705	KV452730.1	11025683	2-scf00040	2
AX-157723159	3	67.705	KV452730.1	11083246	2-scf00040	2
AX-157854051	3	67.705	KV452730.1	11216035	2-scf00040	2
AX-157854052	3	67.705	KV452730.1	11220185	2-scf00040	2
AX-158389778	3	67.705	KV452730.1	11225497	2-scf00040	2
AX-157814085	3	67.705	KV452730.1	11229503	2-scf00040	2
AX-157814087	3	67.705	KV452730.1	11235918	2-scf00040	2
AX-157869266	3	67.705	KV452730.1	11239764	2-scf00040	2
AX-157931723	3	67.705	KV452730.1	11359045	2-scf00040	2
AX-157931830	3	67.705	KV452730.1	11505213	2-scf00040	2
AX-157814112	3	67.705	KV452730.1	11566420	2-scf00040	2
AX-157931864	3	67.705	KV452730.1	11568850	2-scf00040	2
AX-157814116	3	67.705	KV452730.1	11724451	2-scf00040	2
AX-157854149	3	67.705	KV452730.1	11728628	2-scf00040	2
AX-157854157	3	67.705	KV452730.1	11747139	2-scf00040	2
AX-157854159	3	67.705	KV452730.1	11766405	2-scf00040	2
AX-157854163	3	67.705	KV452730.1	11781557	2-scf00040	2
AX-157862729	3	67.705	KV452730.1	11812824	2-scf00040	2
AX-157932085	3	67.705	KV452730.1	11880413	2-scf00040	2

AX-157932087	3	67.705	KV452730.1	11885020	2-scf00040	2
AX-157854178	3	67.705	KV452730.1	11886718	2-scf00040	2
AX-157932151	3	67.705	KV452730.1	11934249	2-scf00040	2
AX-157932164	3	67.705	KV452730.1	11942096	2-scf00040	2
AX-157932180	3	67.705	KV452730.1	11952364	2-scf00040	2
AX-157932187	3	67.705	KV452730.1	11957424	2-scf00040	2
AX-157932193	3	67.705	KV452730.1	11960558	2-scf00040	2
AX-157932264	3	67.705	KV452730.1	12022944	2-scf00040	2
AX-157932318	3	67.705	KV452730.1	12088717	2-scf00040	2
AX-157814146	3	67.705	KV452730.1	12120368	2-scf00040	2
AX-157932595	3	67.705	KV452730.1	12438329	2-scf00040	2
AX-157932997	3	67.705	KV452730.1	13168269	2-scf00040	2
AX-157933521	3	67.705	KV452730.1	14011774	2-scf00040	2
AX-157933622	3	67.705	KV452730.1	14146487	2-scf00040	2
AX-157934163	3	67.705	KV452733.1	272753	2-scf00041	2
AX-157936147	3	67.705	KV452733.1	2530633	2-scf00041	2
AX-157937628	3	67.705	KV452732.1	441892	2-scf00042	2
AX-158421983	3	67.705	KV452732.1	470228	2-scf00042	2
AX-157937756	3	67.705	KV452732.1	682721	2-scf00042	2
AX-157937908	3	67.705	KV452732.1	926247	2-scf00042	2
AX-157937987	3	67.705	KV452732.1	1094682	2-scf00042	2
AX-157945324	3	67.705	KV452746.1	176832	2-scf00054	2
AX-157703088	3	67.705	DX096210.1	331	IORI212 Ictalu	UN
AX-157707544	3	67.705	3ML01034543	267	scf10220_1	UN
AX-157930522	3	67.814	KV452730.1	9964213	2-scf00040	2
AX-157930967	3	67.814	KV452730.1	10540416	2-scf00040	2
AX-157930975	3	67.814	KV452730.1	10543834	2-scf00040	2
AX-157930983	3	67.814	KV452730.1	10554120	2-scf00040	2
AX-157814017	3	67.814	KV452730.1	10559644	2-scf00040	2
AX-157931021	3	67.814	KV452730.1	10583899	2-scf00040	2
AX-157931023	3	67.814	KV452730.1	10584899	2-scf00040	2
AX-157931024	3	67.814	KV452730.1	10585689	2-scf00040	2
AX-157931027	3	67.814	KV452730.1	10586351	2-scf00040	2
AX-157931038	3	67.814	KV452730.1	10599331	2-scf00040	2
AX-157931040	3	67.814	KV452730.1	10602039	2-scf00040	2
AX-157814021	3	67.814	KV452730.1	10606609	2-scf00040	2
AX-157931059	3	67.814	KV452730.1	10617060	2-scf00040	2
AX-157931077	3	67.814	KV452730.1	10629317	2-scf00040	2
AX-157931081	3	67.814	KV452730.1	10633112	2-scf00040	2
AX-157931087	3	67.814	KV452730.1	10635604	2-scf00040	2
AX-157931089	3	67.814	KV452730.1	10637782	2-scf00040	2
AX-157931092	3	67.814	KV452730.1	10639546	2-scf00040	2
AX-157931095	3	67.814	KV452730.1	10642559	2-scf00040	2
AX-157931096	3	67.814	KV452730.1	10645403	2-scf00040	2
AX-157931098	3	67.814	KV452730.1	10646126	2-scf00040	2
AX-157931102	3	67.814	KV452730.1	10650521	2-scf00040	2
AX-157931114	3	67.814	KV452730.1	10666363	2-scf00040	2
AX-157862714	3	67.814	KV452730.1	10676910	2-scf00040	2
AX-157931124	3	67.814	KV452730.1	10677845	2-scf00040	2
AX-157931134	3	67.814	KV452730.1	10689051	2-scf00040	2
AX-157931152	3	67.814	KV452730.1	10703609	2-scf00040	2

AX-157931160	3	67.814	KV452730.1	10709424	2-scf00040	2
AX-157931164	3	67.814	KV452730.1	10715429	2-scf00040	2
AX-157931170	3	67.814	KV452730.1	10721018	2-scf00040	2
AX-157931174	3	67.814	KV452730.1	10729378	2-scf00040	2
AX-157814031	3	67.814	KV452730.1	10734467	2-scf00040	2
AX-157931183	3	67.814	KV452730.1	10747932	2-scf00040	2
AX-157931185	3	67.814	KV452730.1	10748176	2-scf00040	2
AX-157931193	3	67.814	KV452730.1	10752423	2-scf00040	2
AX-157931205	3	67.814	KV452730.1	10764312	2-scf00040	2
AX-157766648	3	67.814	KV452730.1	10764900	2-scf00040	2
AX-157931209	3	67.814	KV452730.1	10771435	2-scf00040	2
AX-157869239	3	67.814	KV452730.1	10787963	2-scf00040	2
AX-157931227	3	67.814	KV452730.1	10791261	2-scf00040	2
AX-157931230	3	67.814	KV452730.1	10792313	2-scf00040	2
AX-157931247	3	67.814	KV452730.1	10809172	2-scf00040	2
AX-157869245	3	67.814	KV452730.1	10809488	2-scf00040	2
AX-157931255	3	67.814	KV452730.1	10819889	2-scf00040	2
AX-157931277	3	67.814	KV452730.1	10832227	2-scf00040	2
AX-157931280	3	67.814	KV452730.1	10833266	2-scf00040	2
AX-157931288	3	67.814	KV452730.1	10847689	2-scf00040	2
AX-157931295	3	67.814	KV452730.1	10851489	2-scf00040	2
AX-157814053	3	67.814	KV452730.1	10853460	2-scf00040	2
AX-157747998	3	67.814	KV452730.1	10880400	2-scf00040	2
AX-157931305	3	67.814	KV452730.1	10881826	2-scf00040	2
AX-157864644	3	67.814	KV452730.1	10889972	2-scf00040	2
AX-157931307	3	67.814	KV452730.1	10896626	2-scf00040	2
AX-157931310	3	67.814	KV452730.1	10900063	2-scf00040	2
AX-157931311	3	67.814	KV452730.1	10900552	2-scf00040	2
AX-157864645	3	67.814	KV452730.1	10903258	2-scf00040	2
AX-157931319	3	67.814	KV452730.1	10910160	2-scf00040	2
AX-157814055	3	67.814	KV452730.1	10912979	2-scf00040	2
AX-157931326	3	67.814	KV452730.1	10919204	2-scf00040	2
AX-157931332	3	67.814	KV452730.1	10934624	2-scf00040	2
AX-157931333	3	67.814	KV452730.1	10935469	2-scf00040	2
AX-157931337	3	67.814	KV452730.1	10941030	2-scf00040	2
AX-157931365	3	67.814	KV452730.1	10965437	2-scf00040	2
AX-157883719	3	67.814	KV452730.1	10972089	2-scf00040	2
AX-157931377	3	67.814	KV452730.1	10974101	2-scf00040	2
AX-157931385	3	67.814	KV452730.1	10977891	2-scf00040	2
AX-157814059	3	67.814	KV452730.1	10978067	2-scf00040	2
AX-157707778	3	67.814	KV452730.1	11254225	2-scf00040	2
AX-157931670	3	67.814	KV452730.1	11291406	2-scf00040	2
AX-157931728	3	67.814	KV452730.1	11365064	2-scf00040	2
AX-157931765	3	67.814	KV452730.1	11412891	2-scf00040	2
AX-157931828	3	67.814	KV452730.1	11501973	2-scf00040	2
AX-157931838	3	67.814	KV452730.1	11515664	2-scf00040	2
AX-157931974	3	67.814	KV452730.1	11742656	2-scf00040	2
AX-157931982	3	67.814	KV452730.1	11759002	2-scf00040	2
AX-157931990	3	67.814	KV452730.1	11768805	2-scf00040	2
AX-157932032	3	67.814	KV452730.1	11841525	2-scf00040	2
AX-157874938	3	67.814	KV452730.1	11898981	2-scf00040	2

AX-157932121	3	67.814	KV452730.1	11923156	2-scf00040	2
AX-157932413	3	67.814	KV452730.1	12226260	2-scf00040	2
AX-157932414	3	67.814	KV452730.1	12229411	2-scf00040	2
AX-157932416	3	67.814	KV452730.1	12230483	2-scf00040	2
AX-157932466	3	67.814	KV452730.1	12287415	2-scf00040	2
AX-157932501	3	67.814	KV452730.1	12309091	2-scf00040	2
AX-157932515	3	67.814	KV452730.1	12326275	2-scf00040	2
AX-157932578	3	67.814	KV452730.1	12420107	2-scf00040	2
AX-157883760	3	67.814	KV452730.1	12423059	2-scf00040	2
AX-157932616	3	67.814	KV452730.1	12457803	2-scf00040	2
AX-157932830	3	67.814	KV452730.1	12893736	2-scf00040	2
AX-157932835	3	67.814	KV452730.1	12910233	2-scf00040	2
AX-157933014	3	67.814	KV452730.1	13203394	2-scf00040	2
AX-157934003	3	67.814	KV452733.1	121823	2-scf00041	2
AX-157934027	3	67.814	KV452733.1	133000	2-scf00041	2
AX-157938049	3	67.814	KV452732.1	1240915	2-scf00042	2
AX-157938056	3	67.814	KV452732.1	1253684	2-scf00042	2
AX-157938057	3	67.814	KV452732.1	1265269	2-scf00042	2
AX-157938157	3	67.814	KV452732.1	1435947	2-scf00042	2
AX-157938168	3	67.814	KV452732.1	1459099	2-scf00042	2
AX-157945389	3	67.814	KV452746.1	243316	2-scf00054	2
AX-157945445	3	67.814	KV452746.1	294442	2-scf00054	2
AX-157945517	3	67.814	KV452746.1	374298	2-scf00054	2
AX-157931386	3	67.877	KV452730.1	10978721	2-scf00040	2
AX-157813842	3	68.033	KV452730.1	7274837	2-scf00040	2
AX-157928912	3	68.033	KV452730.1	7789365	2-scf00040	2
AX-157929548	3	68.033	KV452730.1	8754272	2-scf00040	2
AX-157743522	3	68.033	KV452730.1	8868435	2-scf00040	2
AX-157929676	3	68.033	KV452730.1	8939806	2-scf00040	2
AX-157929984	3	68.033	KV452730.1	9231574	2-scf00040	2
AX-157930084	3	68.033	KV452730.1	9311409	2-scf00040	2
AX-157930897	3	68.033	KV452730.1	10462991	2-scf00040	2
AX-157930966	3	68.033	KV452730.1	10539858	2-scf00040	2
AX-157931012	3	68.033	KV452730.1	10576307	2-scf00040	2
AX-157931013	3	68.033	KV452730.1	10579375	2-scf00040	2
AX-157931030	3	68.033	KV452730.1	10587940	2-scf00040	2
AX-157931056	3	68.033	KV452730.1	10614534	2-scf00040	2
AX-157931067	3	68.033	KV452730.1	10621587	2-scf00040	2
AX-157931113	3	68.033	KV452730.1	10665868	2-scf00040	2
AX-157931123	3	68.033	KV452730.1	10676464	2-scf00040	2
AX-157931173	3	68.033	KV452730.1	10725109	2-scf00040	2
AX-157931278	3	68.033	KV452730.1	10832583	2-scf00040	2
AX-157814060	3	68.033	KV452730.1	10978797	2-scf00040	2
AX-157757321	3	68.033	KV452730.1	10981580	2-scf00040	2
AX-157931401	3	68.033	KV452730.1	10993541	2-scf00040	2
AX-157746205	3	68.033	KV452730.1	10995166	2-scf00040	2
AX-157931409	3	68.033	KV452730.1	11001446	2-scf00040	2
AX-158389771	3	68.033	KV452730.1	11007836	2-scf00040	2
AX-157931415	3	68.033	KV452730.1	11010040	2-scf00040	2
AX-157931425	3	68.033	KV452730.1	11015140	2-scf00040	2
AX-157931445	3	68.033	KV452730.1	11029955	2-scf00040	2

AX-157931449	3	68.033	KV452730.1	11032977	2-scf00040	2
AX-157931457	3	68.033	KV452730.1	11056375	2-scf00040	2
AX-157931460	3	68.033	KV452730.1	11059208	2-scf00040	2
AX-157931462	3	68.033	KV452730.1	11061515	2-scf00040	2
AX-157931464	3	68.033	KV452730.1	11066973	2-scf00040	2
AX-158389773	3	68.033	KV452730.1	11069003	2-scf00040	2
AX-157931482	3	68.033	KV452730.1	11081817	2-scf00040	2
AX-157931486	3	68.033	KV452730.1	11088656	2-scf00040	2
AX-157931487	3	68.033	KV452730.1	11089843	2-scf00040	2
AX-157931488	3	68.033	KV452730.1	11091292	2-scf00040	2
AX-157931498	3	68.033	KV452730.1	11099298	2-scf00040	2
AX-157814066	3	68.033	KV452730.1	11101218	2-scf00040	2
AX-157931507	3	68.033	KV452730.1	11106235	2-scf00040	2
AX-157931509	3	68.033	KV452730.1	11106475	2-scf00040	2
AX-157814069	3	68.033	KV452730.1	11133169	2-scf00040	2
AX-157931533	3	68.033	KV452730.1	11133649	2-scf00040	2
AX-157741435	3	68.033	KV452730.1	11140324	2-scf00040	2
AX-157814070	3	68.033	KV452730.1	11140638	2-scf00040	2
AX-157931555	3	68.033	KV452730.1	11154696	2-scf00040	2
AX-158405517	3	68.033	KV452730.1	11156289	2-scf00040	2
AX-157814072	3	68.033	KV452730.1	11158788	2-scf00040	2
AX-157814076	3	68.033	KV452730.1	11170839	2-scf00040	2
AX-157814077	3	68.033	KV452730.1	11172274	2-scf00040	2
AX-157814078	3	68.033	KV452730.1	11173809	2-scf00040	2
AX-157931568	3	68.033	KV452730.1	11174260	2-scf00040	2
AX-157931569	3	68.033	KV452730.1	11175349	2-scf00040	2
AX-157931574	3	68.033	KV452730.1	11185075	2-scf00040	2
AX-157814088	3	68.033	KV452730.1	11242027	2-scf00040	2
AX-157864651	3	68.033	KV452730.1	11245912	2-scf00040	2
AX-157931628	3	68.033	KV452730.1	11256764	2-scf00040	2
AX-157931636	3	68.033	KV452730.1	11264772	2-scf00040	2
AX-157931683	3	68.033	KV452730.1	11300059	2-scf00040	2
AX-157931698	3	68.033	KV452730.1	11309888	2-scf00040	2
AX-157756180	3	68.033	KV452730.1	11311229	2-scf00040	2
AX-157931762	3	68.033	KV452730.1	11411005	2-scf00040	2
AX-158389810	3	68.033	KV452730.1	11427692	2-scf00040	2
AX-157931817	3	68.033	KV452730.1	11490469	2-scf00040	2
AX-157931821	3	68.033	KV452730.1	11494315	2-scf00040	2
AX-157755910	3	68.033	KV452730.1	11496134	2-scf00040	2
AX-157931835	3	68.033	KV452730.1	11510001	2-scf00040	2
AX-157931839	3	68.033	KV452730.1	11516636	2-scf00040	2
AX-157931849	3	68.033	KV452730.1	11541511	2-scf00040	2
AX-157931861	3	68.033	KV452730.1	11564315	2-scf00040	2
AX-157931867	3	68.033	KV452730.1	11575770	2-scf00040	2
AX-157931869	3	68.033	KV452730.1	11576264	2-scf00040	2
AX-158405528	3	68.033	KV452730.1	11578899	2-scf00040	2
AX-157931872	3	68.033	KV452730.1	11578979	2-scf00040	2
AX-157931874	3	68.033	KV452730.1	11582794	2-scf00040	2
AX-157814113	3	68.033	KV452730.1	11584060	2-scf00040	2
AX-157931877	3	68.033	KV452730.1	11589225	2-scf00040	2
AX-157931900	3	68.033	KV452730.1	11607587	2-scf00040	2

AX-157931926	3	68.033	KV452730.1	11650775	2-scf00040	2
AX-157931930	3	68.033	KV452730.1	11661827	2-scf00040	2
AX-157931934	3	68.033	KV452730.1	11676904	2-scf00040	2
AX-157931948	3	68.033	KV452730.1	11696806	2-scf00040	2
AX-157814121	3	68.033	KV452730.1	11752566	2-scf00040	2
AX-157814122	3	68.033	KV452730.1	11754913	2-scf00040	2
AX-157932000	3	68.033	KV452730.1	11783933	2-scf00040	2
AX-157854175	3	68.033	KV452730.1	11842561	2-scf00040	2
AX-157814134	3	68.033	KV452730.1	11883820	2-scf00040	2
AX-157932144	3	68.033	KV452730.1	11930729	2-scf00040	2
AX-157932211	3	68.033	KV452730.1	11972821	2-scf00040	2
AX-157932215	3	68.033	KV452730.1	11974638	2-scf00040	2
AX-157932228	3	68.033	KV452730.1	11982418	2-scf00040	2
AX-157932229	3	68.033	KV452730.1	11983659	2-scf00040	2
AX-157937812	3	68.033	KV452732.1	785915	2-scf00042	2
AX-157937840	3	68.033	KV452732.1	819018	2-scf00042	2
AX-157938126	3	68.033	KV452732.1	1397289	2-scf00042	2
AX-157938210	3	68.033	KV452732.1	1485347	2-scf00042	2
AX-157938212	3	68.033	KV452732.1	1487779	2-scf00042	2
AX-157704805	3	68.033	3ML01027464	1366	scf03070_1	UN
AX-157706823	3	68.033	3ML01032981	543	scf08614_1	UN
AX-157874886	3	68.075	KV452730.1	9899707	2-scf00040	2
AX-157814092	3	68.075	KV452730.1	11288092	2-scf00040	2
AX-157931693	3	68.075	KV452730.1	11307010	2-scf00040	2
AX-157758380	3	68.075	KV452730.1	11349863	2-scf00040	2
AX-157814097	3	68.075	KV452730.1	11407949	2-scf00040	2
AX-157931857	3	68.075	KV452730.1	11556210	2-scf00040	2
AX-157931936	3	68.075	KV452730.1	11680670	2-scf00040	2
AX-157940905	3	68.075	KV452742.1	4431125	2-scf00046	2
AX-157931677	3	68.083	KV452730.1	11297961	2-scf00040	2
AX-157883751	3	68.083	KV452730.1	12070363	2-scf00040	2
AX-157930732	3	68.142	KV452730.1	10268031	2-scf00040	2
AX-157931133	3	68.142	KV452730.1	10685961	2-scf00040	2
AX-157931299	3	68.142	KV452730.1	10854794	2-scf00040	2
AX-157931306	3	68.142	KV452730.1	10890809	2-scf00040	2
AX-157931343	3	68.142	KV452730.1	10948431	2-scf00040	2
AX-157814071	3	68.142	KV452730.1	11142916	2-scf00040	2
AX-157931542	3	68.142	KV452730.1	11143611	2-scf00040	2
AX-157931546	3	68.142	KV452730.1	11145942	2-scf00040	2
AX-157931550	3	68.142	KV452730.1	11149878	2-scf00040	2
AX-157931639	3	68.142	KV452730.1	11267839	2-scf00040	2
AX-157931661	3	68.142	KV452730.1	11285639	2-scf00040	2
AX-157931705	3	68.142	KV452730.1	11313712	2-scf00040	2
AX-157931722	3	68.142	KV452730.1	11358692	2-scf00040	2
AX-157931725	3	68.142	KV452730.1	11360841	2-scf00040	2
AX-157931727	3	68.142	KV452730.1	11363658	2-scf00040	2
AX-157883730	3	68.142	KV452730.1	11414960	2-scf00040	2
AX-157854088	3	68.142	KV452730.1	11427745	2-scf00040	2
AX-157931778	3	68.142	KV452730.1	11429253	2-scf00040	2
AX-157931799	3	68.142	KV452730.1	11455043	2-scf00040	2
AX-157726906	3	68.142	KV452730.1	11491564	2-scf00040	2

AX-157931823	3	68.142	KV452730.1	11495967	2-scf00040	2
AX-157931846	3	68.142	KV452730.1	11532952	2-scf00040	2
AX-157931850	3	68.142	KV452730.1	11545123	2-scf00040	2
AX-157931854	3	68.142	KV452730.1	11554477	2-scf00040	2
AX-157931866	3	68.142	KV452730.1	11575379	2-scf00040	2
AX-157931879	3	68.142	KV452730.1	11592030	2-scf00040	2
AX-157854117	3	68.142	KV452730.1	11638540	2-scf00040	2
AX-157854125	3	68.142	KV452730.1	11656804	2-scf00040	2
AX-157931928	3	68.142	KV452730.1	11661119	2-scf00040	2
AX-157931951	3	68.142	KV452730.1	11699215	2-scf00040	2
AX-157854141	3	68.142	KV452730.1	11703105	2-scf00040	2
AX-157931959	3	68.142	KV452730.1	11712301	2-scf00040	2
AX-157931967	3	68.142	KV452730.1	11727917	2-scf00040	2
AX-157932005	3	68.142	KV452730.1	11791413	2-scf00040	2
AX-157932014	3	68.142	KV452730.1	11797952	2-scf00040	2
AX-157932174	3	68.142	KV452730.1	11950848	2-scf00040	2
AX-157932212	3	68.142	KV452730.1	11973272	2-scf00040	2
AX-157932232	3	68.142	KV452730.1	11985147	2-scf00040	2
AX-157932270	3	68.142	KV452730.1	12032295	2-scf00040	2
AX-157932437	3	68.142	KV452730.1	12259475	2-scf00040	2
AX-157933101	3	68.142	KV452730.1	13336110	2-scf00040	2
AX-157933430	3	68.142	KV452730.1	13906288	2-scf00040	2
AX-157933903	3	68.142	KV452733.1	25382	2-scf00041	2
AX-157933938	3	68.142	KV452733.1	66383	2-scf00041	2
AX-157937482	3	68.142	KV452732.1	79265	2-scf00042	2
AX-157930127	3	68.361	KV452730.1	9357612	2-scf00040	2
AX-157930367	3	68.361	KV452730.1	9654986	2-scf00040	2
AX-157930495	3	68.361	KV452730.1	9905280	2-scf00040	2
AX-157930512	3	68.361	KV452730.1	9948530	2-scf00040	2
AX-157930703	3	68.361	KV452730.1	10206633	2-scf00040	2
AX-157930887	3	68.361	KV452730.1	10454395	2-scf00040	2
AX-157930889	3	68.361	KV452730.1	10454936	2-scf00040	2
AX-157814016	3	68.361	KV452730.1	10554530	2-scf00040	2
AX-157814023	3	68.361	KV452730.1	10651229	2-scf00040	2
AX-157752245	3	68.361	KV452730.1	10867022	2-scf00040	2
AX-157814056	3	68.361	KV452730.1	10926370	2-scf00040	2
AX-157775385	3	68.361	KV452730.1	10972415	2-scf00040	2
AX-157883722	3	68.361	KV452730.1	11054480	2-scf00040	2
AX-157931472	3	68.361	KV452730.1	11074698	2-scf00040	2
AX-158414992	3	68.361	KV452730.1	11100620	2-scf00040	2
AX-157931655	3	68.361	KV452730.1	11282595	2-scf00040	2
AX-157931706	3	68.361	KV452730.1	11314691	2-scf00040	2
AX-157931733	3	68.361	KV452730.1	11368641	2-scf00040	2
AX-157931847	3	68.361	KV452730.1	11533858	2-scf00040	2
AX-157931950	3	68.361	KV452730.1	11698821	2-scf00040	2
AX-157931978	3	68.361	KV452730.1	11743915	2-scf00040	2
AX-157931985	3	68.361	KV452730.1	11759415	2-scf00040	2
AX-157814124	3	68.361	KV452730.1	11762499	2-scf00040	2
AX-157814125	3	68.361	KV452730.1	11763063	2-scf00040	2
AX-157931986	3	68.361	KV452730.1	11763751	2-scf00040	2
AX-157931995	3	68.361	KV452730.1	11775407	2-scf00040	2

AX-157931998	3	68.361	KV452730.1	11780804	2-scf00040	2
AX-157932011	3	68.361	KV452730.1	11796914	2-scf00040	2
AX-157719649	3	68.361	KV452730.1	11804413	2-scf00040	2
AX-157932019	3	68.361	KV452730.1	11825058	2-scf00040	2
AX-157854170	3	68.361	KV452730.1	11831816	2-scf00040	2
AX-157932023	3	68.361	KV452730.1	11833708	2-scf00040	2
AX-157932028	3	68.361	KV452730.1	11838172	2-scf00040	2
AX-157932034	3	68.361	KV452730.1	11842247	2-scf00040	2
AX-157932036	3	68.361	KV452730.1	11844689	2-scf00040	2
AX-157814130	3	68.361	KV452730.1	11845077	2-scf00040	2
AX-157854176	3	68.361	KV452730.1	11847693	2-scf00040	2
AX-157932042	3	68.361	KV452730.1	11848879	2-scf00040	2
AX-157932043	3	68.361	KV452730.1	11849131	2-scf00040	2
AX-157932044	3	68.361	KV452730.1	11849693	2-scf00040	2
AX-157932045	3	68.361	KV452730.1	11849911	2-scf00040	2
AX-157932047	3	68.361	KV452730.1	11850821	2-scf00040	2
AX-157932049	3	68.361	KV452730.1	11851039	2-scf00040	2
AX-157932051	3	68.361	KV452730.1	11852028	2-scf00040	2
AX-157932053	3	68.361	KV452730.1	11853703	2-scf00040	2
AX-157814131	3	68.361	KV452730.1	11856044	2-scf00040	2
AX-157932054	3	68.361	KV452730.1	11857303	2-scf00040	2
AX-157932055	3	68.361	KV452730.1	11858093	2-scf00040	2
AX-157932058	3	68.361	KV452730.1	11861962	2-scf00040	2
AX-157854177	3	68.361	KV452730.1	11862257	2-scf00040	2
AX-157814132	3	68.361	KV452730.1	11862926	2-scf00040	2
AX-157932059	3	68.361	KV452730.1	11863628	2-scf00040	2
AX-157932060	3	68.361	KV452730.1	11865668	2-scf00040	2
AX-157932065	3	68.361	KV452730.1	11866681	2-scf00040	2
AX-157932069	3	68.361	KV452730.1	11867851	2-scf00040	2
AX-157932072	3	68.361	KV452730.1	11869730	2-scf00040	2
AX-157932078	3	68.361	KV452730.1	11872736	2-scf00040	2
AX-157932081	3	68.361	KV452730.1	11875057	2-scf00040	2
AX-157932082	3	68.361	KV452730.1	11875597	2-scf00040	2
AX-157932083	3	68.361	KV452730.1	11877349	2-scf00040	2
AX-157932088	3	68.361	KV452730.1	11885779	2-scf00040	2
AX-157932094	3	68.361	KV452730.1	11902367	2-scf00040	2
AX-157932101	3	68.361	KV452730.1	11907385	2-scf00040	2
AX-157932107	3	68.361	KV452730.1	11911194	2-scf00040	2
AX-157932109	3	68.361	KV452730.1	11912339	2-scf00040	2
AX-157932112	3	68.361	KV452730.1	11914383	2-scf00040	2
AX-157932117	3	68.361	KV452730.1	11920695	2-scf00040	2
AX-157932120	3	68.361	KV452730.1	11922668	2-scf00040	2
AX-157932124	3	68.361	KV452730.1	11925126	2-scf00040	2
AX-157932126	3	68.361	KV452730.1	11925383	2-scf00040	2
AX-157932132	3	68.361	KV452730.1	11926839	2-scf00040	2
AX-157932137	3	68.361	KV452730.1	11928372	2-scf00040	2
AX-157932139	3	68.361	KV452730.1	11929220	2-scf00040	2
AX-157932160	3	68.361	KV452730.1	11940375	2-scf00040	2
AX-157932162	3	68.361	KV452730.1	11940829	2-scf00040	2
AX-157932165	3	68.361	KV452730.1	11942742	2-scf00040	2
AX-157932169	3	68.361	KV452730.1	11944582	2-scf00040	2

AX-157932170	3	68.361	KV452730.1	11949186	2-scf00040	2
AX-157932171	3	68.361	KV452730.1	11949398	2-scf00040	2
AX-157932175	3	68.361	KV452730.1	11951235	2-scf00040	2
AX-157932178	3	68.361	KV452730.1	11951579	2-scf00040	2
AX-157932195	3	68.361	KV452730.1	11964690	2-scf00040	2
AX-157932207	3	68.361	KV452730.1	11970036	2-scf00040	2
AX-157932208	3	68.361	KV452730.1	11970867	2-scf00040	2
AX-157932223	3	68.361	KV452730.1	11976803	2-scf00040	2
AX-157932226	3	68.361	KV452730.1	11981318	2-scf00040	2
AX-157932238	3	68.361	KV452730.1	11990193	2-scf00040	2
AX-158405536	3	68.361	KV452730.1	12003258	2-scf00040	2
AX-157932251	3	68.361	KV452730.1	12004194	2-scf00040	2
AX-157883749	3	68.361	KV452730.1	12044271	2-scf00040	2
AX-157932302	3	68.361	KV452730.1	12066513	2-scf00040	2
AX-157874949	3	68.361	KV452730.1	12071648	2-scf00040	2
AX-157932312	3	68.361	KV452730.1	12083400	2-scf00040	2
AX-157932328	3	68.361	KV452730.1	12121313	2-scf00040	2
AX-157932344	3	68.361	KV452730.1	12142321	2-scf00040	2
AX-157932347	3	68.361	KV452730.1	12149681	2-scf00040	2
AX-157932349	3	68.361	KV452730.1	12154512	2-scf00040	2
AX-157932352	3	68.361	KV452730.1	12163649	2-scf00040	2
AX-157932354	3	68.361	KV452730.1	12165621	2-scf00040	2
AX-157713722	3	68.361	KV452730.1	12199127	2-scf00040	2
AX-157932400	3	68.361	KV452730.1	12215725	2-scf00040	2
AX-157932408	3	68.361	KV452730.1	12221236	2-scf00040	2
AX-157758419	3	68.361	KV452730.1	12870415	2-scf00040	2
AX-157932834	3	68.361	KV452730.1	12907938	2-scf00040	2
AX-157932890	3	68.361	KV452730.1	12993127	2-scf00040	2
AX-157932937	3	68.361	KV452730.1	13092064	2-scf00040	2
AX-157717633	3	68.361	KV452730.1	13279660	2-scf00040	2
AX-157933087	3	68.361	KV452730.1	13286191	2-scf00040	2
AX-157933200	3	68.361	KV452730.1	13526918	2-scf00040	2
AX-157933226	3	68.361	KV452730.1	13583609	2-scf00040	2
AX-157933414	3	68.361	KV452730.1	13892557	2-scf00040	2
AX-157874992	3	68.361	KV452730.1	14038553	2-scf00040	2
AX-157814261	3	68.361	KV452730.1	14268042	2-scf00040	2
AX-157933774	3	68.361	KV452730.1	14463423	2-scf00040	2
AX-157933895	3	68.361	KV452733.1	17078	2-scf00041	2
AX-157934035	3	68.361	KV452733.1	147888	2-scf00041	2
AX-157934094	3	68.361	KV452733.1	217324	2-scf00041	2
AX-157934251	3	68.361	KV452733.1	349079	2-scf00041	2
AX-157934445	3	68.361	KV452733.1	585769	2-scf00041	2
AX-157934473	3	68.361	KV452733.1	602654	2-scf00041	2
AX-157934554	3	68.361	KV452733.1	674351	2-scf00041	2
AX-157934587	3	68.361	KV452733.1	714712	2-scf00041	2
AX-157934598	3	68.361	KV452733.1	727872	2-scf00041	2
AX-157694707	3	68.361	KV452733.1	933439	2-scf00041	2
AX-157714629	3	68.361	KV452733.1	1064769	2-scf00041	2
AX-157742914	3	68.361	KV452733.1	1206889	2-scf00041	2
AX-157875090	3	68.361	KV452733.1	2425352	2-scf00041	2
AX-157709460	3	68.361	KV452733.1	2548839	2-scf00041	2

AX-158405650	3	68.361	KV452733.1	3459928	2-scf00041	2
AX-157937172	3	68.361	KV452733.1	3516192	2-scf00041	2
AX-157937287	3	68.361	KV452733.1	3643213	2-scf00041	2
AX-157937689	3	68.361	KV452732.1	553577	2-scf00042	2
AX-157937749	3	68.361	KV452732.1	656516	2-scf00042	2
AX-157937759	3	68.361	KV452732.1	691640	2-scf00042	2
AX-157937774	3	68.361	KV452732.1	723526	2-scf00042	2
AX-157937842	3	68.361	KV452732.1	828699	2-scf00042	2
AX-157694576	3	68.361	KV452732.1	850137	2-scf00042	2
AX-158405673	3	68.361	KV452732.1	935660	2-scf00042	2
AX-157937938	3	68.361	KV452732.1	989176	2-scf00042	2
AX-157937939	3	68.361	KV452732.1	989821	2-scf00042	2
AX-157937949	3	68.361	KV452732.1	998693	2-scf00042	2
AX-157937978	3	68.361	KV452732.1	1070953	2-scf00042	2
AX-157937994	3	68.361	KV452732.1	1111002	2-scf00042	2
AX-157938006	3	68.361	KV452732.1	1139936	2-scf00042	2
AX-157938059	3	68.361	KV452732.1	1271569	2-scf00042	2
AX-157938170	3	68.361	KV452732.1	1461170	2-scf00042	2
AX-157938338	3	68.361	KV452735.1	84840	2-scf00044	2
AX-157742901	3	68.361	KV452742.1	526061	2-scf00046	2
AX-157939773	3	68.361	KV452742.1	2768818	2-scf00046	2
AX-157939908	3	68.361	KV452742.1	2990196	2-scf00046	2
AX-157939934	3	68.361	KV452742.1	3008841	2-scf00046	2
AX-157940084	3	68.361	KV452742.1	3159294	2-scf00046	2
AX-157940130	3	68.361	KV452742.1	3208440	2-scf00046	2
AX-157940347	3	68.361	KV452742.1	3473977	2-scf00046	2
AX-157940355	3	68.361	KV452742.1	3488776	2-scf00046	2
AX-157940501	3	68.361	KV452742.1	3666534	2-scf00046	2
AX-157940531	3	68.361	KV452742.1	3729290	2-scf00046	2
AX-157721774	3	68.361	KV452742.1	3745922	2-scf00046	2
AX-157753193	3	68.361	KV452742.1	3769801	2-scf00046	2
AX-157940882	3	68.361	KV452742.1	4407192	2-scf00046	2
AX-157941387	3	68.361	KV452743.1	38275	2-scf00050	2
AX-157941523	3	68.361	KV452745.1	19148	2-scf00051	2
AX-158382650	3	68.361	KV452745.1	599540	2-scf00051	2
AX-157942316	3	68.361	KV452745.1	717041	2-scf00051	2
AX-158390087	3	68.361	KV452745.1	1048315	2-scf00051	2
AX-157943077	3	68.361	KV452747.1	12898	2-scf00053	2
AX-157943129	3	68.361	KV452747.1	75025	2-scf00053	2
AX-157943174	3	68.361	KV452747.1	132158	2-scf00053	2
AX-158376538	3	68.361	KV452740.1	287438	2-scf00586	2
AX-157696629	3	68.361	FI871124.1	141	ORI212 Ictalu	UN
AX-157693895	3	68.361	FI875480.1	131	ORI212 Ictalu	UN
AX-157705355	3	68.361	3ML01029487	603	scf05110_1	UN
AX-157930290	3	68.47	KV452730.1	9543460	2-scf00040	2
AX-157931058	3	68.47	KV452730.1	10616477	2-scf00040	2
AX-157931355	3	68.47	KV452730.1	10958257	2-scf00040	2
AX-157715469	3	68.47	KV452730.1	11869101	2-scf00040	2
AX-157932237	3	68.47	KV452730.1	11989662	2-scf00040	2
AX-157854181	3	68.47	KV452730.1	11993238	2-scf00040	2
AX-157932242	3	68.47	KV452730.1	11994601	2-scf00040	2

AX-158389821	3	68.47	KV452730.1	11997010	2-scf00040	2
AX-157932245	3	68.47	KV452730.1	11997543	2-scf00040	2
AX-157932246	3	68.47	KV452730.1	11999412	2-scf00040	2
AX-157932247	3	68.47	KV452730.1	12000496	2-scf00040	2
AX-157932260	3	68.47	KV452730.1	12018048	2-scf00040	2
AX-157932261	3	68.47	KV452730.1	12019155	2-scf00040	2
AX-157932263	3	68.47	KV452730.1	12022515	2-scf00040	2
AX-157932288	3	68.47	KV452730.1	12052364	2-scf00040	2
AX-157854202	3	68.47	KV452730.1	12053825	2-scf00040	2
AX-157854204	3	68.47	KV452730.1	12065914	2-scf00040	2
AX-157854205	3	68.47	KV452730.1	12066071	2-scf00040	2
AX-158389824	3	68.47	KV452730.1	12069016	2-scf00040	2
AX-157932304	3	68.47	KV452730.1	12069784	2-scf00040	2
AX-157932305	3	68.47	KV452730.1	12071448	2-scf00040	2
AX-157814141	3	68.47	KV452730.1	12072727	2-scf00040	2
AX-157932313	3	68.47	KV452730.1	12084045	2-scf00040	2
AX-157932315	3	68.47	KV452730.1	12084744	2-scf00040	2
AX-157932317	3	68.47	KV452730.1	12087924	2-scf00040	2
AX-157932335	3	68.47	KV452730.1	12129829	2-scf00040	2
AX-157854213	3	68.47	KV452730.1	12134975	2-scf00040	2
AX-157932338	3	68.47	KV452730.1	12135365	2-scf00040	2
AX-157932339	3	68.47	KV452730.1	12135681	2-scf00040	2
AX-157932342	3	68.47	KV452730.1	12141197	2-scf00040	2
AX-157932355	3	68.47	KV452730.1	12166002	2-scf00040	2
AX-157932360	3	68.47	KV452730.1	12168192	2-scf00040	2
AX-157854216	3	68.47	KV452730.1	12171671	2-scf00040	2
AX-157854217	3	68.47	KV452730.1	12173904	2-scf00040	2
AX-157854219	3	68.47	KV452730.1	12196254	2-scf00040	2
AX-157814151	3	68.47	KV452730.1	12196576	2-scf00040	2
AX-157814152	3	68.47	KV452730.1	12196874	2-scf00040	2
AX-157932381	3	68.47	KV452730.1	12200332	2-scf00040	2
AX-157779631	3	68.47	KV452730.1	12200667	2-scf00040	2
AX-157932382	3	68.47	KV452730.1	12202657	2-scf00040	2
AX-157779635	3	68.47	KV452730.1	12202750	2-scf00040	2
AX-157932389	3	68.47	KV452730.1	12207768	2-scf00040	2
AX-157779636	3	68.47	KV452730.1	12208795	2-scf00040	2
AX-157932394	3	68.47	KV452730.1	12210189	2-scf00040	2
AX-157932395	3	68.47	KV452730.1	12211551	2-scf00040	2
AX-157932396	3	68.47	KV452730.1	12212198	2-scf00040	2
AX-158389826	3	68.47	KV452730.1	12212324	2-scf00040	2
AX-158389827	3	68.47	KV452730.1	12224940	2-scf00040	2
AX-157932412	3	68.47	KV452730.1	12226044	2-scf00040	2
AX-157869293	3	68.47	KV452730.1	12229957	2-scf00040	2
AX-157932417	3	68.47	KV452730.1	12232051	2-scf00040	2
AX-157814154	3	68.47	KV452730.1	12233653	2-scf00040	2
AX-157745632	3	68.47	KV452730.1	12234598	2-scf00040	2
AX-157932419	3	68.47	KV452730.1	12234686	2-scf00040	2
AX-157869294	3	68.47	KV452730.1	12235742	2-scf00040	2
AX-157814155	3	68.47	KV452730.1	12236001	2-scf00040	2
AX-157932422	3	68.47	KV452730.1	12239335	2-scf00040	2
AX-157932428	3	68.47	KV452730.1	12246009	2-scf00040	2

AX-157883757	3	68.47	KV452730.1	12249291	2-scf00040	2
AX-157932432	3	68.47	KV452730.1	12249414	2-scf00040	2
AX-157932433	3	68.47	KV452730.1	12250621	2-scf00040	2
AX-157932436	3	68.47	KV452730.1	12251259	2-scf00040	2
AX-157814156	3	68.47	KV452730.1	12251852	2-scf00040	2
AX-157854220	3	68.47	KV452730.1	12252667	2-scf00040	2
AX-157854221	3	68.47	KV452730.1	12256254	2-scf00040	2
AX-158389828	3	68.47	KV452730.1	12256983	2-scf00040	2
AX-157854222	3	68.47	KV452730.1	12257954	2-scf00040	2
AX-158389830	3	68.47	KV452730.1	12260469	2-scf00040	2
AX-157932441	3	68.47	KV452730.1	12263474	2-scf00040	2
AX-157854223	3	68.47	KV452730.1	12264967	2-scf00040	2
AX-157854224	3	68.47	KV452730.1	12265126	2-scf00040	2
AX-157869295	3	68.47	KV452730.1	12279676	2-scf00040	2
AX-158389835	3	68.47	KV452730.1	12326019	2-scf00040	2
AX-158389839	3	68.47	KV452730.1	12420387	2-scf00040	2
AX-157767278	3	68.47	KV452730.1	12444212	2-scf00040	2
AX-157854254	3	68.47	KV452730.1	12705807	2-scf00040	2
AX-157854255	3	68.47	KV452730.1	12708912	2-scf00040	2
AX-157814184	3	68.47	KV452730.1	12758478	2-scf00040	2
AX-157932742	3	68.47	KV452730.1	12767695	2-scf00040	2
AX-157932837	3	68.47	KV452730.1	12913242	2-scf00040	2
AX-157932856	3	68.47	KV452730.1	12947163	2-scf00040	2
AX-157932874	3	68.47	KV452730.1	12967512	2-scf00040	2
AX-157932915	3	68.47	KV452730.1	13051188	2-scf00040	2
AX-157932926	3	68.47	KV452730.1	13070604	2-scf00040	2
AX-158389907	3	68.47	KV452730.1	13089417	2-scf00040	2
AX-157814203	3	68.47	KV452730.1	13096941	2-scf00040	2
AX-157932947	3	68.47	KV452730.1	13100024	2-scf00040	2
AX-158389909	3	68.47	KV452730.1	13101988	2-scf00040	2
AX-157933093	3	68.47	KV452730.1	13294747	2-scf00040	2
AX-157854341	3	68.47	KV452730.1	13375455	2-scf00040	2
AX-158389941	3	68.47	KV452730.1	13405988	2-scf00040	2
AX-157862760	3	68.47	KV452730.1	13472700	2-scf00040	2
AX-157937522	3	68.47	KV452732.1	234675	2-scf00042	2
AX-157937527	3	68.47	KV452732.1	244153	2-scf00042	2
AX-157937528	3	68.47	KV452732.1	247012	2-scf00042	2
AX-157937535	3	68.47	KV452732.1	266740	2-scf00042	2
AX-157937542	3	68.47	KV452732.1	279514	2-scf00042	2
AX-157937546	3	68.47	KV452732.1	294205	2-scf00042	2
AX-157937547	3	68.47	KV452732.1	294763	2-scf00042	2
AX-157937550	3	68.47	KV452732.1	296714	2-scf00042	2
AX-157937553	3	68.47	KV452732.1	298630	2-scf00042	2
AX-157937555	3	68.47	KV452732.1	300134	2-scf00042	2
AX-157937563	3	68.47	KV452732.1	312871	2-scf00042	2
AX-157937575	3	68.47	KV452732.1	333505	2-scf00042	2
AX-157937579	3	68.47	KV452732.1	339152	2-scf00042	2
AX-157937580	3	68.47	KV452732.1	339981	2-scf00042	2
AX-157937581	3	68.47	KV452732.1	341125	2-scf00042	2
AX-157937584	3	68.47	KV452732.1	343793	2-scf00042	2
AX-157937589	3	68.47	KV452732.1	355724	2-scf00042	2

AX-157937594	3	68.47	KV452732.1	363472	2-scf00042	2
AX-157937597	3	68.47	KV452732.1	367835	2-scf00042	2
AX-158390022	3	68.47	KV452732.1	374547	2-scf00042	2
AX-157937612	3	68.47	KV452732.1	398925	2-scf00042	2
AX-157757835	3	68.47	KV452732.1	413238	2-scf00042	2
AX-157937619	3	68.47	KV452732.1	424420	2-scf00042	2
AX-157937624	3	68.47	KV452732.1	440475	2-scf00042	2
AX-157883847	3	68.47	KV452732.1	448370	2-scf00042	2
AX-157937638	3	68.47	KV452732.1	462665	2-scf00042	2
AX-157883848	3	68.47	KV452732.1	465691	2-scf00042	2
AX-157937639	3	68.47	KV452732.1	465946	2-scf00042	2
AX-157937640	3	68.47	KV452732.1	466331	2-scf00042	2
AX-158405662	3	68.47	KV452732.1	471642	2-scf00042	2
AX-157937643	3	68.47	KV452732.1	476309	2-scf00042	2
AX-157937647	3	68.47	KV452732.1	482895	2-scf00042	2
AX-157937649	3	68.47	KV452732.1	486128	2-scf00042	2
AX-157937652	3	68.47	KV452732.1	495394	2-scf00042	2
AX-157937653	3	68.47	KV452732.1	496886	2-scf00042	2
AX-157814469	3	68.47	KV452732.1	498485	2-scf00042	2
AX-157937662	3	68.47	KV452732.1	509778	2-scf00042	2
AX-157937668	3	68.47	KV452732.1	522137	2-scf00042	2
AX-157937669	3	68.47	KV452732.1	523438	2-scf00042	2
AX-157937670	3	68.47	KV452732.1	525064	2-scf00042	2
AX-157937672	3	68.47	KV452732.1	526150	2-scf00042	2
AX-157937674	3	68.47	KV452732.1	528047	2-scf00042	2
AX-157937675	3	68.47	KV452732.1	531500	2-scf00042	2
AX-157937678	3	68.47	KV452732.1	532034	2-scf00042	2
AX-157937691	3	68.47	KV452732.1	555800	2-scf00042	2
AX-157937693	3	68.47	KV452732.1	556497	2-scf00042	2
AX-157937694	3	68.47	KV452732.1	558130	2-scf00042	2
AX-157937698	3	68.47	KV452732.1	559461	2-scf00042	2
AX-157937701	3	68.47	KV452732.1	571285	2-scf00042	2
AX-157937708	3	68.47	KV452732.1	582453	2-scf00042	2
AX-157937713	3	68.47	KV452732.1	585096	2-scf00042	2
AX-157937716	3	68.47	KV452732.1	586138	2-scf00042	2
AX-157937718	3	68.47	KV452732.1	594897	2-scf00042	2
AX-157883849	3	68.47	KV452732.1	599201	2-scf00042	2
AX-157937724	3	68.47	KV452732.1	600955	2-scf00042	2
AX-157937725	3	68.47	KV452732.1	605014	2-scf00042	2
AX-157937731	3	68.47	KV452732.1	615447	2-scf00042	2
AX-157937737	3	68.47	KV452732.1	634240	2-scf00042	2
AX-157937747	3	68.47	KV452732.1	650107	2-scf00042	2
AX-157938198	3	68.47	KV452732.1	1476772	2-scf00042	2
AX-157944424	3	68.47	KV452747.1	1397809	2-scf00053	2
AX-157928472	3	68.579	KV452730.1	7013431	2-scf00040	2
AX-157929600	3	68.579	KV452730.1	8808492	2-scf00040	2
AX-157929906	3	68.579	KV452730.1	9169735	2-scf00040	2
AX-157930188	3	68.579	KV452730.1	9413005	2-scf00040	2
AX-158405486	3	68.579	KV452730.1	9414056	2-scf00040	2
AX-157813946	3	68.579	KV452730.1	9516097	2-scf00040	2
AX-157813954	3	68.579	KV452730.1	9651698	2-scf00040	2

AX-157930409	3	68.579	KV452730.1	9755218	2-scf00040	2
AX-157930447	3	68.579	KV452730.1	9812707	2-scf00040	2
AX-157930490	3	68.579	KV452730.1	9902038	2-scf00040	2
AX-157930506	3	68.579	KV452730.1	9935137	2-scf00040	2
AX-157930572	3	68.579	KV452730.1	10041312	2-scf00040	2
AX-157813987	3	68.579	KV452730.1	10226175	2-scf00040	2
AX-157930717	3	68.579	KV452730.1	10236869	2-scf00040	2
AX-157930720	3	68.579	KV452730.1	10242503	2-scf00040	2
AX-157930779	3	68.579	KV452730.1	10314558	2-scf00040	2
AX-157930780	3	68.579	KV452730.1	10315758	2-scf00040	2
AX-157930856	3	68.579	KV452730.1	10402681	2-scf00040	2
AX-157930873	3	68.579	KV452730.1	10430211	2-scf00040	2
AX-157930882	3	68.579	KV452730.1	10444755	2-scf00040	2
AX-157814007	3	68.579	KV452730.1	10445528	2-scf00040	2
AX-157869215	3	68.579	KV452730.1	10458246	2-scf00040	2
AX-157930905	3	68.579	KV452730.1	10467328	2-scf00040	2
AX-157854018	3	68.579	KV452730.1	10480156	2-scf00040	2
AX-157930922	3	68.579	KV452730.1	10486904	2-scf00040	2
AX-157814009	3	68.579	KV452730.1	10492856	2-scf00040	2
AX-157930954	3	68.579	KV452730.1	10526756	2-scf00040	2
AX-157931019	3	68.579	KV452730.1	10583221	2-scf00040	2
AX-157931034	3	68.579	KV452730.1	10596297	2-scf00040	2
AX-158389759	3	68.579	KV452730.1	10678271	2-scf00040	2
AX-157931143	3	68.579	KV452730.1	10694670	2-scf00040	2
AX-157883715	3	68.579	KV452730.1	10736135	2-scf00040	2
AX-157931250	3	68.579	KV452730.1	10812564	2-scf00040	2
AX-157931297	3	68.579	KV452730.1	10852745	2-scf00040	2
AX-157931322	3	68.579	KV452730.1	10911723	2-scf00040	2
AX-157931324	3	68.579	KV452730.1	10915315	2-scf00040	2
AX-157931330	3	68.579	KV452730.1	10929309	2-scf00040	2
AX-157931334	3	68.579	KV452730.1	10937772	2-scf00040	2
AX-157931340	3	68.579	KV452730.1	10944167	2-scf00040	2
AX-157931342	3	68.579	KV452730.1	10946036	2-scf00040	2
AX-157931344	3	68.579	KV452730.1	10949517	2-scf00040	2
AX-157814057	3	68.579	KV452730.1	10950768	2-scf00040	2
AX-157931348	3	68.579	KV452730.1	10951669	2-scf00040	2
AX-157931350	3	68.579	KV452730.1	10952070	2-scf00040	2
AX-157931367	3	68.579	KV452730.1	10966016	2-scf00040	2
AX-157931373	3	68.579	KV452730.1	10970963	2-scf00040	2
AX-157931374	3	68.579	KV452730.1	10972864	2-scf00040	2
AX-157931405	3	68.579	KV452730.1	10997520	2-scf00040	2
AX-157931437	3	68.579	KV452730.1	11024504	2-scf00040	2
AX-157931549	3	68.579	KV452730.1	11148199	2-scf00040	2
AX-157931638	3	68.579	KV452730.1	11267027	2-scf00040	2
AX-157874921	3	68.579	KV452730.1	11380409	2-scf00040	2
AX-157931775	3	68.579	KV452730.1	11425307	2-scf00040	2
AX-158405525	3	68.579	KV452730.1	11426164	2-scf00040	2
AX-157931780	3	68.579	KV452730.1	11431335	2-scf00040	2
AX-157931781	3	68.579	KV452730.1	11433359	2-scf00040	2
AX-158389811	3	68.579	KV452730.1	11437184	2-scf00040	2
AX-157854089	3	68.579	KV452730.1	11437444	2-scf00040	2

AX-157931820	3	68.579	KV452730.1	11492994	2-scf00040	2
AX-157883731	3	68.579	KV452730.1	11496552	2-scf00040	2
AX-157931851	3	68.579	KV452730.1	11546018	2-scf00040	2
AX-157931852	3	68.579	KV452730.1	11549530	2-scf00040	2
AX-157738845	3	68.579	KV452730.1	11549834	2-scf00040	2
AX-158414998	3	68.579	KV452730.1	11552892	2-scf00040	2
AX-158379349	3	68.579	KV452730.1	11553723	2-scf00040	2
AX-157715977	3	68.579	KV452730.1	11553766	2-scf00040	2
AX-157931903	3	68.579	KV452730.1	11613800	2-scf00040	2
AX-157931973	3	68.579	KV452730.1	11738025	2-scf00040	2
AX-157931992	3	68.579	KV452730.1	11771154	2-scf00040	2
AX-157932004	3	68.579	KV452730.1	11788631	2-scf00040	2
AX-157932056	3	68.579	KV452730.1	11861263	2-scf00040	2
AX-157932145	3	68.579	KV452730.1	11931249	2-scf00040	2
AX-157932152	3	68.579	KV452730.1	11935587	2-scf00040	2
AX-157932230	3	68.579	KV452730.1	11984005	2-scf00040	2
AX-157932233	3	68.579	KV452730.1	11985502	2-scf00040	2
AX-157854180	3	68.579	KV452730.1	11988113	2-scf00040	2
AX-157932240	3	68.579	KV452730.1	11992265	2-scf00040	2
AX-157932241	3	68.579	KV452730.1	11993984	2-scf00040	2
AX-157932243	3	68.579	KV452730.1	11995211	2-scf00040	2
AX-157932248	3	68.579	KV452730.1	12001356	2-scf00040	2
AX-157932249	3	68.579	KV452730.1	12001835	2-scf00040	2
AX-157932250	3	68.579	KV452730.1	12003697	2-scf00040	2
AX-157932253	3	68.579	KV452730.1	12007499	2-scf00040	2
AX-157932254	3	68.579	KV452730.1	12014112	2-scf00040	2
AX-157932255	3	68.579	KV452730.1	12014566	2-scf00040	2
AX-157932258	3	68.579	KV452730.1	12016383	2-scf00040	2
AX-157854183	3	68.579	KV452730.1	12017121	2-scf00040	2
AX-157932259	3	68.579	KV452730.1	12017358	2-scf00040	2
AX-157854184	3	68.579	KV452730.1	12017512	2-scf00040	2
AX-157854185	3	68.579	KV452730.1	12018477	2-scf00040	2
AX-157854186	3	68.579	KV452730.1	12018687	2-scf00040	2
AX-157932262	3	68.579	KV452730.1	12020873	2-scf00040	2
AX-157854187	3	68.579	KV452730.1	12021448	2-scf00040	2
AX-157854188	3	68.579	KV452730.1	12023564	2-scf00040	2
AX-157854189	3	68.579	KV452730.1	12025534	2-scf00040	2
AX-157932266	3	68.579	KV452730.1	12025667	2-scf00040	2
AX-157932267	3	68.579	KV452730.1	12027556	2-scf00040	2
AX-157751757	3	68.579	KV452730.1	12028766	2-scf00040	2
AX-157932268	3	68.579	KV452730.1	12030036	2-scf00040	2
AX-157854191	3	68.579	KV452730.1	12030351	2-scf00040	2
AX-157932269	3	68.579	KV452730.1	12030787	2-scf00040	2
AX-157932272	3	68.579	KV452730.1	12033578	2-scf00040	2
AX-157854192	3	68.579	KV452730.1	12034048	2-scf00040	2
AX-157932274	3	68.579	KV452730.1	12034715	2-scf00040	2
AX-157854193	3	68.579	KV452730.1	12038891	2-scf00040	2
AX-157854194	3	68.579	KV452730.1	12039256	2-scf00040	2
AX-157854195	3	68.579	KV452730.1	12041924	2-scf00040	2
AX-157854196	3	68.579	KV452730.1	12042828	2-scf00040	2
AX-157854198	3	68.579	KV452730.1	12045832	2-scf00040	2

AX-157854199	3	68.579	KV452730.1	12046695	2-scf00040	2
AX-157862733	3	68.579	KV452730.1	12049594	2-scf00040	2
AX-157932285	3	68.579	KV452730.1	12049878	2-scf00040	2
AX-157854201	3	68.579	KV452730.1	12053784	2-scf00040	2
AX-157932294	3	68.579	KV452730.1	12055964	2-scf00040	2
AX-157932301	3	68.579	KV452730.1	12063971	2-scf00040	2
AX-157932306	3	68.579	KV452730.1	12073339	2-scf00040	2
AX-157932321	3	68.579	KV452730.1	12104912	2-scf00040	2
AX-157814144	3	68.579	KV452730.1	12109171	2-scf00040	2
AX-157814147	3	68.579	KV452730.1	12123490	2-scf00040	2
AX-157932331	3	68.579	KV452730.1	12123644	2-scf00040	2
AX-157932333	3	68.579	KV452730.1	12126510	2-scf00040	2
AX-157932348	3	68.579	KV452730.1	12152000	2-scf00040	2
AX-157779615	3	68.579	KV452730.1	12152429	2-scf00040	2
AX-157932361	3	68.579	KV452730.1	12172812	2-scf00040	2
AX-157932405	3	68.579	KV452730.1	12218168	2-scf00040	2
AX-157773989	3	68.579	KV452730.1	12222314	2-scf00040	2
AX-157932418	3	68.579	KV452730.1	12233362	2-scf00040	2
AX-157932421	3	68.579	KV452730.1	12238127	2-scf00040	2
AX-157932440	3	68.579	KV452730.1	12261951	2-scf00040	2
AX-157814157	3	68.579	KV452730.1	12265631	2-scf00040	2
AX-157932445	3	68.579	KV452730.1	12266382	2-scf00040	2
AX-157932447	3	68.579	KV452730.1	12266917	2-scf00040	2
AX-157932449	3	68.579	KV452730.1	12267854	2-scf00040	2
AX-157932450	3	68.579	KV452730.1	12268546	2-scf00040	2
AX-157932451	3	68.579	KV452730.1	12269650	2-scf00040	2
AX-157932453	3	68.579	KV452730.1	12270059	2-scf00040	2
AX-157932455	3	68.579	KV452730.1	12272161	2-scf00040	2
AX-157932459	3	68.579	KV452730.1	12273295	2-scf00040	2
AX-157727408	3	68.579	KV452730.1	12274186	2-scf00040	2
AX-157932460	3	68.579	KV452730.1	12276325	2-scf00040	2
AX-157744625	3	68.579	KV452730.1	12278008	2-scf00040	2
AX-157932462	3	68.579	KV452730.1	12279030	2-scf00040	2
AX-157932464	3	68.579	KV452730.1	12285029	2-scf00040	2
AX-157932473	3	68.579	KV452730.1	12294559	2-scf00040	2
AX-157932475	3	68.579	KV452730.1	12294890	2-scf00040	2
AX-157932477	3	68.579	KV452730.1	12295118	2-scf00040	2
AX-157932479	3	68.579	KV452730.1	12295814	2-scf00040	2
AX-157932482	3	68.579	KV452730.1	12297204	2-scf00040	2
AX-157932485	3	68.579	KV452730.1	12297737	2-scf00040	2
AX-157932487	3	68.579	KV452730.1	12298188	2-scf00040	2
AX-157932488	3	68.579	KV452730.1	12298295	2-scf00040	2
AX-157932492	3	68.579	KV452730.1	12300252	2-scf00040	2
AX-157932493	3	68.579	KV452730.1	12302095	2-scf00040	2
AX-157932494	3	68.579	KV452730.1	12302476	2-scf00040	2
AX-157854226	3	68.579	KV452730.1	12303733	2-scf00040	2
AX-157854227	3	68.579	KV452730.1	12304557	2-scf00040	2
AX-157883759	3	68.579	KV452730.1	12305377	2-scf00040	2
AX-157932497	3	68.579	KV452730.1	12305617	2-scf00040	2
AX-157932500	3	68.579	KV452730.1	12307023	2-scf00040	2
AX-158389832	3	68.579	KV452730.1	12307955	2-scf00040	2

AX-157932502	3	68.579	KV452730.1	12313399	2-scf00040	2
AX-157932505	3	68.579	KV452730.1	12314317	2-scf00040	2
AX-157932516	3	68.579	KV452730.1	12327139	2-scf00040	2
AX-157932521	3	68.579	KV452730.1	12329497	2-scf00040	2
AX-157771706	3	68.579	KV452730.1	12340016	2-scf00040	2
AX-157932533	3	68.579	KV452730.1	12348146	2-scf00040	2
AX-157932535	3	68.579	KV452730.1	12349303	2-scf00040	2
AX-157932538	3	68.579	KV452730.1	12350235	2-scf00040	2
AX-157932541	3	68.579	KV452730.1	12352641	2-scf00040	2
AX-157932542	3	68.579	KV452730.1	12359548	2-scf00040	2
AX-157814161	3	68.579	KV452730.1	12364408	2-scf00040	2
AX-157932548	3	68.579	KV452730.1	12369122	2-scf00040	2
AX-157932556	3	68.579	KV452730.1	12379185	2-scf00040	2
AX-157747418	3	68.579	KV452730.1	12382951	2-scf00040	2
AX-157869302	3	68.579	KV452730.1	12385299	2-scf00040	2
AX-157932559	3	68.579	KV452730.1	12387651	2-scf00040	2
AX-157932560	3	68.579	KV452730.1	12391257	2-scf00040	2
AX-157932561	3	68.579	KV452730.1	12391663	2-scf00040	2
AX-157932564	3	68.579	KV452730.1	12393290	2-scf00040	2
AX-157932567	3	68.579	KV452730.1	12397915	2-scf00040	2
AX-157702002	3	68.579	KV452730.1	12399690	2-scf00040	2
AX-157814166	3	68.579	KV452730.1	12408564	2-scf00040	2
AX-157932569	3	68.579	KV452730.1	12409700	2-scf00040	2
AX-157932572	3	68.579	KV452730.1	12411182	2-scf00040	2
AX-157932573	3	68.579	KV452730.1	12411684	2-scf00040	2
AX-157932576	3	68.579	KV452730.1	12418880	2-scf00040	2
AX-157932580	3	68.579	KV452730.1	12422002	2-scf00040	2
AX-157932581	3	68.579	KV452730.1	12422383	2-scf00040	2
AX-157932582	3	68.579	KV452730.1	12424140	2-scf00040	2
AX-157869307	3	68.579	KV452730.1	12425967	2-scf00040	2
AX-157932602	3	68.579	KV452730.1	12442947	2-scf00040	2
AX-157932603	3	68.579	KV452730.1	12443577	2-scf00040	2
AX-157932604	3	68.579	KV452730.1	12443796	2-scf00040	2
AX-157814167	3	68.579	KV452730.1	12447564	2-scf00040	2
AX-157932614	3	68.579	KV452730.1	12455454	2-scf00040	2
AX-157932615	3	68.579	KV452730.1	12455959	2-scf00040	2
AX-157814169	3	68.579	KV452730.1	12463456	2-scf00040	2
AX-157869310	3	68.579	KV452730.1	12473238	2-scf00040	2
AX-157814170	3	68.579	KV452730.1	12479791	2-scf00040	2
AX-157814172	3	68.579	KV452730.1	12480590	2-scf00040	2
AX-157932631	3	68.579	KV452730.1	12491444	2-scf00040	2
AX-157932634	3	68.579	KV452730.1	12496825	2-scf00040	2
AX-157932639	3	68.579	KV452730.1	12503131	2-scf00040	2
AX-157932641	3	68.579	KV452730.1	12508936	2-scf00040	2
AX-157814174	3	68.579	KV452730.1	12516142	2-scf00040	2
AX-157932649	3	68.579	KV452730.1	12519453	2-scf00040	2
AX-157932654	3	68.579	KV452730.1	12524837	2-scf00040	2
AX-157932656	3	68.579	KV452730.1	12525478	2-scf00040	2
AX-157932658	3	68.579	KV452730.1	12528749	2-scf00040	2
AX-157695025	3	68.579	KV452730.1	12530894	2-scf00040	2
AX-157814175	3	68.579	KV452730.1	12533152	2-scf00040	2

AX-157854231	3	68.579	KV452730.1	12535581	2-scf00040	2
AX-157932662	3	68.579	KV452730.1	12539140	2-scf00040	2
AX-158389842	3	68.579	KV452730.1	12542968	2-scf00040	2
AX-157932671	3	68.579	KV452730.1	12556039	2-scf00040	2
AX-157779736	3	68.579	KV452730.1	12582374	2-scf00040	2
AX-157854236	3	68.579	KV452730.1	12584679	2-scf00040	2
AX-157932677	3	68.579	KV452730.1	12584892	2-scf00040	2
AX-157932681	3	68.579	KV452730.1	12590783	2-scf00040	2
AX-157854237	3	68.579	KV452730.1	12596506	2-scf00040	2
AX-157932686	3	68.579	KV452730.1	12599043	2-scf00040	2
AX-157932689	3	68.579	KV452730.1	12605334	2-scf00040	2
AX-158389847	3	68.579	KV452730.1	12612642	2-scf00040	2
AX-158389848	3	68.579	KV452730.1	12616554	2-scf00040	2
AX-157932691	3	68.579	KV452730.1	12616665	2-scf00040	2
AX-158389849	3	68.579	KV452730.1	12618732	2-scf00040	2
AX-157869319	3	68.579	KV452730.1	12620434	2-scf00040	2
AX-157932693	3	68.579	KV452730.1	12623046	2-scf00040	2
AX-157932694	3	68.579	KV452730.1	12625416	2-scf00040	2
AX-157854242	3	68.579	KV452730.1	12627013	2-scf00040	2
AX-157932695	3	68.579	KV452730.1	12627211	2-scf00040	2
AX-158389851	3	68.579	KV452730.1	12630795	2-scf00040	2
AX-157704206	3	68.579	KV452730.1	12638737	2-scf00040	2
AX-158389854	3	68.579	KV452730.1	12644846	2-scf00040	2
AX-157932699	3	68.579	KV452730.1	12646651	2-scf00040	2
AX-158389857	3	68.579	KV452730.1	12662726	2-scf00040	2
AX-157854245	3	68.579	KV452730.1	12663104	2-scf00040	2
AX-158389858	3	68.579	KV452730.1	12663699	2-scf00040	2
AX-157932708	3	68.579	KV452730.1	12664144	2-scf00040	2
AX-157854247	3	68.579	KV452730.1	12668194	2-scf00040	2
AX-157932712	3	68.579	KV452730.1	12680151	2-scf00040	2
AX-158389861	3	68.579	KV452730.1	12684361	2-scf00040	2
AX-158389862	3	68.579	KV452730.1	12687372	2-scf00040	2
AX-157932719	3	68.579	KV452730.1	12692087	2-scf00040	2
AX-157932725	3	68.579	KV452730.1	12705093	2-scf00040	2
AX-157854253	3	68.579	KV452730.1	12705564	2-scf00040	2
AX-158421965	3	68.579	KV452730.1	12710043	2-scf00040	2
AX-157854256	3	68.579	KV452730.1	12716187	2-scf00040	2
AX-158389870	3	68.579	KV452730.1	12726960	2-scf00040	2
AX-157932733	3	68.579	KV452730.1	12742458	2-scf00040	2
AX-157814183	3	68.579	KV452730.1	12756834	2-scf00040	2
AX-157814185	3	68.579	KV452730.1	12759166	2-scf00040	2
AX-157854265	3	68.579	KV452730.1	12761549	2-scf00040	2
AX-157932744	3	68.579	KV452730.1	12768830	2-scf00040	2
AX-157814188	3	68.579	KV452730.1	12776803	2-scf00040	2
AX-157854269	3	68.579	KV452730.1	12783009	2-scf00040	2
AX-158389875	3	68.579	KV452730.1	12797207	2-scf00040	2
AX-157854271	3	68.579	KV452730.1	12798498	2-scf00040	2
AX-157932765	3	68.579	KV452730.1	12812104	2-scf00040	2
AX-158389877	3	68.579	KV452730.1	12821221	2-scf00040	2
AX-157932773	3	68.579	KV452730.1	12827057	2-scf00040	2
AX-157932777	3	68.579	KV452730.1	12832148	2-scf00040	2

AX-157854273	3	68.579	KV452730.1	12833479	2-scf00040	2
AX-158389878	3	68.579	KV452730.1	12834455	2-scf00040	2
AX-157932781	3	68.579	KV452730.1	12838417	2-scf00040	2
AX-158389879	3	68.579	KV452730.1	12842187	2-scf00040	2
AX-157932785	3	68.579	KV452730.1	12843027	2-scf00040	2
AX-157854275	3	68.579	KV452730.1	12849213	2-scf00040	2
AX-157854276	3	68.579	KV452730.1	12849359	2-scf00040	2
AX-157932794	3	68.579	KV452730.1	12850176	2-scf00040	2
AX-157932797	3	68.579	KV452730.1	12851248	2-scf00040	2
AX-157932799	3	68.579	KV452730.1	12852238	2-scf00040	2
AX-157932807	3	68.579	KV452730.1	12861989	2-scf00040	2
AX-157932808	3	68.579	KV452730.1	12862911	2-scf00040	2
AX-157932812	3	68.579	KV452730.1	12865332	2-scf00040	2
AX-158389883	3	68.579	KV452730.1	12869236	2-scf00040	2
AX-157932817	3	68.579	KV452730.1	12873699	2-scf00040	2
AX-157932818	3	68.579	KV452730.1	12874063	2-scf00040	2
AX-157932819	3	68.579	KV452730.1	12875207	2-scf00040	2
AX-157932821	3	68.579	KV452730.1	12876012	2-scf00040	2
AX-157854279	3	68.579	KV452730.1	12879790	2-scf00040	2
AX-157932824	3	68.579	KV452730.1	12879853	2-scf00040	2
AX-157932829	3	68.579	KV452730.1	12885609	2-scf00040	2
AX-157854280	3	68.579	KV452730.1	12890656	2-scf00040	2
AX-157869334	3	68.579	KV452730.1	12898661	2-scf00040	2
AX-157854281	3	68.579	KV452730.1	12899933	2-scf00040	2
AX-157869336	3	68.579	KV452730.1	12901439	2-scf00040	2
AX-157932836	3	68.579	KV452730.1	12912100	2-scf00040	2
AX-157854283	3	68.579	KV452730.1	12914595	2-scf00040	2
AX-157932842	3	68.579	KV452730.1	12921876	2-scf00040	2
AX-157883763	3	68.579	KV452730.1	12927757	2-scf00040	2
AX-157854284	3	68.579	KV452730.1	12928243	2-scf00040	2
AX-157814192	3	68.579	KV452730.1	12928722	2-scf00040	2
AX-158405552	3	68.579	KV452730.1	12945713	2-scf00040	2
AX-157932858	3	68.579	KV452730.1	12952319	2-scf00040	2
AX-157932859	3	68.579	KV452730.1	12953048	2-scf00040	2
AX-157932861	3	68.579	KV452730.1	12953705	2-scf00040	2
AX-157779759	3	68.579	KV452730.1	12955457	2-scf00040	2
AX-157932866	3	68.579	KV452730.1	12958686	2-scf00040	2
AX-157932869	3	68.579	KV452730.1	12961159	2-scf00040	2
AX-157932872	3	68.579	KV452730.1	12965412	2-scf00040	2
AX-157932876	3	68.579	KV452730.1	12969043	2-scf00040	2
AX-157932878	3	68.579	KV452730.1	12971486	2-scf00040	2
AX-157932881	3	68.579	KV452730.1	12974472	2-scf00040	2
AX-157932882	3	68.579	KV452730.1	12977800	2-scf00040	2
AX-157752286	3	68.579	KV452730.1	12984196	2-scf00040	2
AX-157932884	3	68.579	KV452730.1	12984641	2-scf00040	2
AX-157854290	3	68.579	KV452730.1	12986243	2-scf00040	2
AX-158404659	3	68.579	KV452730.1	12992181	2-scf00040	2
AX-157932896	3	68.579	KV452730.1	13005428	2-scf00040	2
AX-157854291	3	68.579	KV452730.1	13010749	2-scf00040	2
AX-157814193	3	68.579	KV452730.1	13010832	2-scf00040	2
AX-157869348	3	68.579	KV452730.1	13012174	2-scf00040	2

AX-157932903	3	68.579	KV452730.1	13014888	2-scf00040	2
AX-157814196	3	68.579	KV452730.1	13034176	2-scf00040	2
AX-157869351	3	68.579	KV452730.1	13039158	2-scf00040	2
AX-157869352	3	68.579	KV452730.1	13041795	2-scf00040	2
AX-157932912	3	68.579	KV452730.1	13047125	2-scf00040	2
AX-157712376	3	68.579	KV452730.1	13058725	2-scf00040	2
AX-157862752	3	68.579	KV452730.1	13060085	2-scf00040	2
AX-157814198	3	68.579	KV452730.1	13063462	2-scf00040	2
AX-158389904	3	68.579	KV452730.1	13074769	2-scf00040	2
AX-157932932	3	68.579	KV452730.1	13079867	2-scf00040	2
AX-157854301	3	68.579	KV452730.1	13080897	2-scf00040	2
AX-158415016	3	68.579	KV452730.1	13090667	2-scf00040	2
AX-157814199	3	68.579	KV452730.1	13094890	2-scf00040	2
AX-157932942	3	68.579	KV452730.1	13097031	2-scf00040	2
AX-157854305	3	68.579	KV452730.1	13098035	2-scf00040	2
AX-157932944	3	68.579	KV452730.1	13098862	2-scf00040	2
AX-157932952	3	68.579	KV452730.1	13104342	2-scf00040	2
AX-157932957	3	68.579	KV452730.1	13118313	2-scf00040	2
AX-157932961	3	68.579	KV452730.1	13124995	2-scf00040	2
AX-157932970	3	68.579	KV452730.1	13136339	2-scf00040	2
AX-157932971	3	68.579	KV452730.1	13138009	2-scf00040	2
AX-157932972	3	68.579	KV452730.1	13138611	2-scf00040	2
AX-157932978	3	68.579	KV452730.1	13145832	2-scf00040	2
AX-157932980	3	68.579	KV452730.1	13146702	2-scf00040	2
AX-157932984	3	68.579	KV452730.1	13148844	2-scf00040	2
AX-157932990	3	68.579	KV452730.1	13157989	2-scf00040	2
AX-157932993	3	68.579	KV452730.1	13160863	2-scf00040	2
AX-157814205	3	68.579	KV452730.1	13169259	2-scf00040	2
AX-157854316	3	68.579	KV452730.1	13172934	2-scf00040	2
AX-157933004	3	68.579	KV452730.1	13176055	2-scf00040	2
AX-157933007	3	68.579	KV452730.1	13183491	2-scf00040	2
AX-157779803	3	68.579	KV452730.1	13184007	2-scf00040	2
AX-157933008	3	68.579	KV452730.1	13185978	2-scf00040	2
AX-157814212	3	68.579	KV452730.1	13199600	2-scf00040	2
AX-157933012	3	68.579	KV452730.1	13201528	2-scf00040	2
AX-157854319	3	68.579	KV452730.1	13202286	2-scf00040	2
AX-157854320	3	68.579	KV452730.1	13203737	2-scf00040	2
AX-157854321	3	68.579	KV452730.1	13206314	2-scf00040	2
AX-157933020	3	68.579	KV452730.1	13208580	2-scf00040	2
AX-157731412	3	68.579	KV452730.1	13213560	2-scf00040	2
AX-157814214	3	68.579	KV452730.1	13216561	2-scf00040	2
AX-157933036	3	68.579	KV452730.1	13217490	2-scf00040	2
AX-157933039	3	68.579	KV452730.1	13219871	2-scf00040	2
AX-157748175	3	68.579	KV452730.1	13226978	2-scf00040	2
AX-157933040	3	68.579	KV452730.1	13227267	2-scf00040	2
AX-157933041	3	68.579	KV452730.1	13229057	2-scf00040	2
AX-157814215	3	68.579	KV452730.1	13230147	2-scf00040	2
AX-157814216	3	68.579	KV452730.1	13230832	2-scf00040	2
AX-157814217	3	68.579	KV452730.1	13233349	2-scf00040	2
AX-157933046	3	68.579	KV452730.1	13233583	2-scf00040	2
AX-157933047	3	68.579	KV452730.1	13233899	2-scf00040	2

AX-157933048	3	68.579	KV452730.1	13234366	2-scf00040	2
AX-157933052	3	68.579	KV452730.1	13236586	2-scf00040	2
AX-157814218	3	68.579	KV452730.1	13246073	2-scf00040	2
AX-158389919	3	68.579	KV452730.1	13248495	2-scf00040	2
AX-158389922	3	68.579	KV452730.1	13262811	2-scf00040	2
AX-157933069	3	68.579	KV452730.1	13263035	2-scf00040	2
AX-158389923	3	68.579	KV452730.1	13265962	2-scf00040	2
AX-157756163	3	68.579	KV452730.1	13270621	2-scf00040	2
AX-157933073	3	68.579	KV452730.1	13271198	2-scf00040	2
AX-157933075	3	68.579	KV452730.1	13272002	2-scf00040	2
AX-157933077	3	68.579	KV452730.1	13275294	2-scf00040	2
AX-157933079	3	68.579	KV452730.1	13276399	2-scf00040	2
AX-158389928	3	68.579	KV452730.1	13285681	2-scf00040	2
AX-158389929	3	68.579	KV452730.1	13286750	2-scf00040	2
AX-158389930	3	68.579	KV452730.1	13296876	2-scf00040	2
AX-157933095	3	68.579	KV452730.1	13300284	2-scf00040	2
AX-157779830	3	68.579	KV452730.1	13302268	2-scf00040	2
AX-158389934	3	68.579	KV452730.1	13317996	2-scf00040	2
AX-157933100	3	68.579	KV452730.1	13330038	2-scf00040	2
AX-158405560	3	68.579	KV452730.1	13339097	2-scf00040	2
AX-157779832	3	68.579	KV452730.1	13341609	2-scf00040	2
AX-157854336	3	68.579	KV452730.1	13351495	2-scf00040	2
AX-157933111	3	68.579	KV452730.1	13353170	2-scf00040	2
AX-157854338	3	68.579	KV452730.1	13359364	2-scf00040	2
AX-158389939	3	68.579	KV452730.1	13373495	2-scf00040	2
AX-157933130	3	68.579	KV452730.1	13376176	2-scf00040	2
AX-157869369	3	68.579	KV452730.1	13378376	2-scf00040	2
AX-157933136	3	68.579	KV452730.1	13379550	2-scf00040	2
AX-157933139	3	68.579	KV452730.1	13381081	2-scf00040	2
AX-157933142	3	68.579	KV452730.1	13384145	2-scf00040	2
AX-157862755	3	68.579	KV452730.1	13407602	2-scf00040	2
AX-158389943	3	68.579	KV452730.1	13411629	2-scf00040	2
AX-158389944	3	68.579	KV452730.1	13412224	2-scf00040	2
AX-157933158	3	68.579	KV452730.1	13417133	2-scf00040	2
AX-157869370	3	68.579	KV452730.1	13420719	2-scf00040	2
AX-157933159	3	68.579	KV452730.1	13431980	2-scf00040	2
AX-157854344	3	68.579	KV452730.1	13435358	2-scf00040	2
AX-157779845	3	68.579	KV452730.1	13436671	2-scf00040	2
AX-157862758	3	68.579	KV452730.1	13443983	2-scf00040	2
AX-157933166	3	68.579	KV452730.1	13451263	2-scf00040	2
AX-157814225	3	68.579	KV452730.1	13455207	2-scf00040	2
AX-158389947	3	68.579	KV452730.1	13462381	2-scf00040	2
AX-157933170	3	68.579	KV452730.1	13462653	2-scf00040	2
AX-157854349	3	68.579	KV452730.1	13463240	2-scf00040	2
AX-157779858	3	68.579	KV452730.1	13476551	2-scf00040	2
AX-157854351	3	68.579	KV452730.1	13483047	2-scf00040	2
AX-157933177	3	68.579	KV452730.1	13491268	2-scf00040	2
AX-157933182	3	68.579	KV452730.1	13496066	2-scf00040	2
AX-157933183	3	68.579	KV452730.1	13501861	2-scf00040	2
AX-157933186	3	68.579	KV452730.1	13504093	2-scf00040	2
AX-158389950	3	68.579	KV452730.1	13507412	2-scf00040	2

AX-157933202	3	68.579	KV452730.1	13528590	2-scf00040	2
AX-157933205	3	68.579	KV452730.1	13532872	2-scf00040	2
AX-157854356	3	68.579	KV452730.1	13541744	2-scf00040	2
AX-157869376	3	68.579	KV452730.1	13544571	2-scf00040	2
AX-157933217	3	68.579	KV452730.1	13544818	2-scf00040	2
AX-157869378	3	68.579	KV452730.1	13548953	2-scf00040	2
AX-157697977	3	68.579	KV452730.1	13552701	2-scf00040	2
AX-157869384	3	68.579	KV452730.1	13563176	2-scf00040	2
AX-157869385	3	68.579	KV452730.1	13564495	2-scf00040	2
AX-157854359	3	68.579	KV452730.1	13568864	2-scf00040	2
AX-157933223	3	68.579	KV452730.1	13574004	2-scf00040	2
AX-157933224	3	68.579	KV452730.1	13575643	2-scf00040	2
AX-157869386	3	68.579	KV452730.1	13576606	2-scf00040	2
AX-157854360	3	68.579	KV452730.1	13577811	2-scf00040	2
AX-157779874	3	68.579	KV452730.1	13578706	2-scf00040	2
AX-157814229	3	68.579	KV452730.1	13579178	2-scf00040	2
AX-157814230	3	68.579	KV452730.1	13579685	2-scf00040	2
AX-158389953	3	68.579	KV452730.1	13581292	2-scf00040	2
AX-158389954	3	68.579	KV452730.1	13583569	2-scf00040	2
AX-157933229	3	68.579	KV452730.1	13588931	2-scf00040	2
AX-157814231	3	68.579	KV452730.1	13592333	2-scf00040	2
AX-157862764	3	68.579	KV452730.1	13592514	2-scf00040	2
AX-158405564	3	68.579	KV452730.1	13594434	2-scf00040	2
AX-157933233	3	68.579	KV452730.1	13595901	2-scf00040	2
AX-157814232	3	68.579	KV452730.1	13601571	2-scf00040	2
AX-158389955	3	68.579	KV452730.1	13604704	2-scf00040	2
AX-157854364	3	68.579	KV452730.1	13606014	2-scf00040	2
AX-157814234	3	68.579	KV452730.1	13606146	2-scf00040	2
AX-157933238	3	68.579	KV452730.1	13606225	2-scf00040	2
AX-157692540	3	68.579	KV452730.1	13606937	2-scf00040	2
AX-157814235	3	68.579	KV452730.1	13610520	2-scf00040	2
AX-158389956	3	68.579	KV452730.1	13612046	2-scf00040	2
AX-157854365	3	68.579	KV452730.1	13613250	2-scf00040	2
AX-157854366	3	68.579	KV452730.1	13618584	2-scf00040	2
AX-157854367	3	68.579	KV452730.1	13619351	2-scf00040	2
AX-157869387	3	68.579	KV452730.1	13619575	2-scf00040	2
AX-157933244	3	68.579	KV452730.1	13621136	2-scf00040	2
AX-157933245	3	68.579	KV452730.1	13621670	2-scf00040	2
AX-157869390	3	68.579	KV452730.1	13623273	2-scf00040	2
AX-157933246	3	68.579	KV452730.1	13625384	2-scf00040	2
AX-157854368	3	68.579	KV452730.1	13626049	2-scf00040	2
AX-157933247	3	68.579	KV452730.1	13627367	2-scf00040	2
AX-158389957	3	68.579	KV452730.1	13628884	2-scf00040	2
AX-158389958	3	68.579	KV452730.1	13632186	2-scf00040	2
AX-157854370	3	68.579	KV452730.1	13632441	2-scf00040	2
AX-157933250	3	68.579	KV452730.1	13634327	2-scf00040	2
AX-157933251	3	68.579	KV452730.1	13638299	2-scf00040	2
AX-157854371	3	68.579	KV452730.1	13638769	2-scf00040	2
AX-157933252	3	68.579	KV452730.1	13640054	2-scf00040	2
AX-157814236	3	68.579	KV452730.1	13640151	2-scf00040	2
AX-157854372	3	68.579	KV452730.1	13641829	2-scf00040	2

AX-157854373	3	68.579	KV452730.1	13643839	2-scf00040	2
AX-157854374	3	68.579	KV452730.1	13644623	2-scf00040	2
AX-157814237	3	68.579	KV452730.1	13645135	2-scf00040	2
AX-157779890	3	68.579	KV452730.1	13645316	2-scf00040	2
AX-157779891	3	68.579	KV452730.1	13645381	2-scf00040	2
AX-157814238	3	68.579	KV452730.1	13648877	2-scf00040	2
AX-157933253	3	68.579	KV452730.1	13654514	2-scf00040	2
AX-157702052	3	68.579	KV452730.1	13658031	2-scf00040	2
AX-157854376	3	68.579	KV452730.1	13660715	2-scf00040	2
AX-157854377	3	68.579	KV452730.1	13660789	2-scf00040	2
AX-157933255	3	68.579	KV452730.1	13661789	2-scf00040	2
AX-157854378	3	68.579	KV452730.1	13662737	2-scf00040	2
AX-158389960	3	68.579	KV452730.1	13666458	2-scf00040	2
AX-157933257	3	68.579	KV452730.1	13667332	2-scf00040	2
AX-157854379	3	68.579	KV452730.1	13670714	2-scf00040	2
AX-157854380	3	68.579	KV452730.1	13672798	2-scf00040	2
AX-157854381	3	68.579	KV452730.1	13675557	2-scf00040	2
AX-157933259	3	68.579	KV452730.1	13675599	2-scf00040	2
AX-157933261	3	68.579	KV452730.1	13676957	2-scf00040	2
AX-157814239	3	68.579	KV452730.1	13677961	2-scf00040	2
AX-157933263	3	68.579	KV452730.1	13679738	2-scf00040	2
AX-157933265	3	68.579	KV452730.1	13680397	2-scf00040	2
AX-158389962	3	68.579	KV452730.1	13682173	2-scf00040	2
AX-157854382	3	68.579	KV452730.1	13683655	2-scf00040	2
AX-157933268	3	68.579	KV452730.1	13684330	2-scf00040	2
AX-157933269	3	68.579	KV452730.1	13687022	2-scf00040	2
AX-157933271	3	68.579	KV452730.1	13688598	2-scf00040	2
AX-157933272	3	68.579	KV452730.1	13689024	2-scf00040	2
AX-157869394	3	68.579	KV452730.1	13689352	2-scf00040	2
AX-157854383	3	68.579	KV452730.1	13690457	2-scf00040	2
AX-157854384	3	68.579	KV452730.1	13692043	2-scf00040	2
AX-157854385	3	68.579	KV452730.1	13692864	2-scf00040	2
AX-157869396	3	68.579	KV452730.1	13698679	2-scf00040	2
AX-158389963	3	68.579	KV452730.1	13709092	2-scf00040	2
AX-157854386	3	68.579	KV452730.1	13713794	2-scf00040	2
AX-157854387	3	68.579	KV452730.1	13714924	2-scf00040	2
AX-157933287	3	68.579	KV452730.1	13720954	2-scf00040	2
AX-157869397	3	68.579	KV452730.1	13722484	2-scf00040	2
AX-157854388	3	68.579	KV452730.1	13723417	2-scf00040	2
AX-158389965	3	68.579	KV452730.1	13725230	2-scf00040	2
AX-157933289	3	68.579	KV452730.1	13727396	2-scf00040	2
AX-157854389	3	68.579	KV452730.1	13728798	2-scf00040	2
AX-157814240	3	68.579	KV452730.1	13729308	2-scf00040	2
AX-157814241	3	68.579	KV452730.1	13729436	2-scf00040	2
AX-157933291	3	68.579	KV452730.1	13729682	2-scf00040	2
AX-157933292	3	68.579	KV452730.1	13730275	2-scf00040	2
AX-157862766	3	68.579	KV452730.1	13731625	2-scf00040	2
AX-157869398	3	68.579	KV452730.1	13732684	2-scf00040	2
AX-157869399	3	68.579	KV452730.1	13734119	2-scf00040	2
AX-157933293	3	68.579	KV452730.1	13737534	2-scf00040	2
AX-157933297	3	68.579	KV452730.1	13740428	2-scf00040	2

AX-157933298	3	68.579	KV452730.1	13740740	2-scf00040	2
AX-157869403	3	68.579	KV452730.1	13741865	2-scf00040	2
AX-157933301	3	68.579	KV452730.1	13743204	2-scf00040	2
AX-157869404	3	68.579	KV452730.1	13745163	2-scf00040	2
AX-157869405	3	68.579	KV452730.1	13745377	2-scf00040	2
AX-157933303	3	68.579	KV452730.1	13746144	2-scf00040	2
AX-157933304	3	68.579	KV452730.1	13746881	2-scf00040	2
AX-157869409	3	68.579	KV452730.1	13748649	2-scf00040	2
AX-157933311	3	68.579	KV452730.1	13750324	2-scf00040	2
AX-157854390	3	68.579	KV452730.1	13750823	2-scf00040	2
AX-157854391	3	68.579	KV452730.1	13752557	2-scf00040	2
AX-157854392	3	68.579	KV452730.1	13752855	2-scf00040	2
AX-157933313	3	68.579	KV452730.1	13754225	2-scf00040	2
AX-157933315	3	68.579	KV452730.1	13755749	2-scf00040	2
AX-157933321	3	68.579	KV452730.1	13758032	2-scf00040	2
AX-157933325	3	68.579	KV452730.1	13762184	2-scf00040	2
AX-157933327	3	68.579	KV452730.1	13763032	2-scf00040	2
AX-157933328	3	68.579	KV452730.1	13763805	2-scf00040	2
AX-157854394	3	68.579	KV452730.1	13764826	2-scf00040	2
AX-158389966	3	68.579	KV452730.1	13764913	2-scf00040	2
AX-157814243	3	68.579	KV452730.1	13767091	2-scf00040	2
AX-158389967	3	68.579	KV452730.1	13767319	2-scf00040	2
AX-157933330	3	68.579	KV452730.1	13772777	2-scf00040	2
AX-157933331	3	68.579	KV452730.1	13773341	2-scf00040	2
AX-157933335	3	68.579	KV452730.1	13776599	2-scf00040	2
AX-158389968	3	68.579	KV452730.1	13788038	2-scf00040	2
AX-157854396	3	68.579	KV452730.1	13789237	2-scf00040	2
AX-157933344	3	68.579	KV452730.1	13789464	2-scf00040	2
AX-157854397	3	68.579	KV452730.1	13790009	2-scf00040	2
AX-157933346	3	68.579	KV452730.1	13791807	2-scf00040	2
AX-157933347	3	68.579	KV452730.1	13794932	2-scf00040	2
AX-157933348	3	68.579	KV452730.1	13796180	2-scf00040	2
AX-157933349	3	68.579	KV452730.1	13796942	2-scf00040	2
AX-158389969	3	68.579	KV452730.1	13797321	2-scf00040	2
AX-157704058	3	68.579	KV452730.1	13797836	2-scf00040	2
AX-157814244	3	68.579	KV452730.1	13798430	2-scf00040	2
AX-157862767	3	68.579	KV452730.1	13798539	2-scf00040	2
AX-157854398	3	68.579	KV452730.1	13802300	2-scf00040	2
AX-158389970	3	68.579	KV452730.1	13806960	2-scf00040	2
AX-158389971	3	68.579	KV452730.1	13808914	2-scf00040	2
AX-157854399	3	68.579	KV452730.1	13809224	2-scf00040	2
AX-157854400	3	68.579	KV452730.1	13811306	2-scf00040	2
AX-157933352	3	68.579	KV452730.1	13811412	2-scf00040	2
AX-157854401	3	68.579	KV452730.1	13815660	2-scf00040	2
AX-157933357	3	68.579	KV452730.1	13816265	2-scf00040	2
AX-158389972	3	68.579	KV452730.1	13819296	2-scf00040	2
AX-157854404	3	68.579	KV452730.1	13819367	2-scf00040	2
AX-157779930	3	68.579	KV452730.1	13823279	2-scf00040	2
AX-157933361	3	68.579	KV452730.1	13824209	2-scf00040	2
AX-158389973	3	68.579	KV452730.1	13824428	2-scf00040	2
AX-157933362	3	68.579	KV452730.1	13827118	2-scf00040	2

AX-157862768	3	68.579	KV452730.1	13827628	2-scf00040	2
AX-157933363	3	68.579	KV452730.1	13828494	2-scf00040	2
AX-157933364	3	68.579	KV452730.1	13828945	2-scf00040	2
AX-157874983	3	68.579	KV452730.1	13829796	2-scf00040	2
AX-158405567	3	68.579	KV452730.1	13830771	2-scf00040	2
AX-158405568	3	68.579	KV452730.1	13831056	2-scf00040	2
AX-158389975	3	68.579	KV452730.1	13834536	2-scf00040	2
AX-157933366	3	68.579	KV452730.1	13834676	2-scf00040	2
AX-157933368	3	68.579	KV452730.1	13836713	2-scf00040	2
AX-157933370	3	68.579	KV452730.1	13839085	2-scf00040	2
AX-157933371	3	68.579	KV452730.1	13839598	2-scf00040	2
AX-157933372	3	68.579	KV452730.1	13840035	2-scf00040	2
AX-157933373	3	68.579	KV452730.1	13840561	2-scf00040	2
AX-157933374	3	68.579	KV452730.1	13841154	2-scf00040	2
AX-157854407	3	68.579	KV452730.1	13841924	2-scf00040	2
AX-158389976	3	68.579	KV452730.1	13842204	2-scf00040	2
AX-157933377	3	68.579	KV452730.1	13846888	2-scf00040	2
AX-157754343	3	68.579	KV452730.1	13850000	2-scf00040	2
AX-157933384	3	68.579	KV452730.1	13851916	2-scf00040	2
AX-158415026	3	68.579	KV452730.1	13852704	2-scf00040	2
AX-157854408	3	68.579	KV452730.1	13855760	2-scf00040	2
AX-158389977	3	68.579	KV452730.1	13856211	2-scf00040	2
AX-157933387	3	68.579	KV452730.1	13859648	2-scf00040	2
AX-157933392	3	68.579	KV452730.1	13865189	2-scf00040	2
AX-158389978	3	68.579	KV452730.1	13865910	2-scf00040	2
AX-157933396	3	68.579	KV452730.1	13868370	2-scf00040	2
AX-157933398	3	68.579	KV452730.1	13869150	2-scf00040	2
AX-158389979	3	68.579	KV452730.1	13870416	2-scf00040	2
AX-158389980	3	68.579	KV452730.1	13872761	2-scf00040	2
AX-157854410	3	68.579	KV452730.1	13873722	2-scf00040	2
AX-157933400	3	68.579	KV452730.1	13873951	2-scf00040	2
AX-158389981	3	68.579	KV452730.1	13875320	2-scf00040	2
AX-157814245	3	68.579	KV452730.1	13877730	2-scf00040	2
AX-157933406	3	68.579	KV452730.1	13881553	2-scf00040	2
AX-158389983	3	68.579	KV452730.1	13882512	2-scf00040	2
AX-157779935	3	68.579	KV452730.1	13884184	2-scf00040	2
AX-157933407	3	68.579	KV452730.1	13884334	2-scf00040	2
AX-157933409	3	68.579	KV452730.1	13888044	2-scf00040	2
AX-157854411	3	68.579	KV452730.1	13889135	2-scf00040	2
AX-157854412	3	68.579	KV452730.1	13889288	2-scf00040	2
AX-157933411	3	68.579	KV452730.1	13890441	2-scf00040	2
AX-157854413	3	68.579	KV452730.1	13890769	2-scf00040	2
AX-157933416	3	68.579	KV452730.1	13893616	2-scf00040	2
AX-158389984	3	68.579	KV452730.1	13897846	2-scf00040	2
AX-157933421	3	68.579	KV452730.1	13898195	2-scf00040	2
AX-157933424	3	68.579	KV452730.1	13899573	2-scf00040	2
AX-157933425	3	68.579	KV452730.1	13901381	2-scf00040	2
AX-157933428	3	68.579	KV452730.1	13903882	2-scf00040	2
AX-158389985	3	68.579	KV452730.1	13904022	2-scf00040	2
AX-157854415	3	68.579	KV452730.1	13906098	2-scf00040	2
AX-157862769	3	68.579	KV452730.1	13906370	2-scf00040	2

AX-157754179	3	68.579	KV452730.1	13908203	2-scf00040	2
AX-157933433	3	68.579	KV452730.1	13911181	2-scf00040	2
AX-157933435	3	68.579	KV452730.1	13912227	2-scf00040	2
AX-157933439	3	68.579	KV452730.1	13918623	2-scf00040	2
AX-157933440	3	68.579	KV452730.1	13923235	2-scf00040	2
AX-157779941	3	68.579	KV452730.1	13923939	2-scf00040	2
AX-158389987	3	68.579	KV452730.1	13924147	2-scf00040	2
AX-157933442	3	68.579	KV452730.1	13925048	2-scf00040	2
AX-157933443	3	68.579	KV452730.1	13925639	2-scf00040	2
AX-157933444	3	68.579	KV452730.1	13929423	2-scf00040	2
AX-157933445	3	68.579	KV452730.1	13930271	2-scf00040	2
AX-157933447	3	68.579	KV452730.1	13930466	2-scf00040	2
AX-157854418	3	68.579	KV452730.1	13933299	2-scf00040	2
AX-157933448	3	68.579	KV452730.1	13933611	2-scf00040	2
AX-157854419	3	68.579	KV452730.1	13936056	2-scf00040	2
AX-157933449	3	68.579	KV452730.1	13940272	2-scf00040	2
AX-157854420	3	68.579	KV452730.1	13942330	2-scf00040	2
AX-157933452	3	68.579	KV452730.1	13942422	2-scf00040	2
AX-157933458	3	68.579	KV452730.1	13943903	2-scf00040	2
AX-157933460	3	68.579	KV452730.1	13944380	2-scf00040	2
AX-157933465	3	68.579	KV452730.1	13945879	2-scf00040	2
AX-157869410	3	68.579	KV452730.1	13946272	2-scf00040	2
AX-157933466	3	68.579	KV452730.1	13946947	2-scf00040	2
AX-157814246	3	68.579	KV452730.1	13947325	2-scf00040	2
AX-157869412	3	68.579	KV452730.1	13955197	2-scf00040	2
AX-157933470	3	68.579	KV452730.1	13956836	2-scf00040	2
AX-157814247	3	68.579	KV452730.1	13958039	2-scf00040	2
AX-157730187	3	68.579	KV452730.1	13958600	2-scf00040	2
AX-157869414	3	68.579	KV452730.1	13964484	2-scf00040	2
AX-157869416	3	68.579	KV452730.1	13967633	2-scf00040	2
AX-157814248	3	68.579	KV452730.1	13975155	2-scf00040	2
AX-157933482	3	68.579	KV452730.1	13977123	2-scf00040	2
AX-157854422	3	68.579	KV452730.1	13978284	2-scf00040	2
AX-157854423	3	68.579	KV452730.1	13979241	2-scf00040	2
AX-157933485	3	68.579	KV452730.1	13982939	2-scf00040	2
AX-157869417	3	68.579	KV452730.1	13983253	2-scf00040	2
AX-157933490	3	68.579	KV452730.1	13984591	2-scf00040	2
AX-157854424	3	68.579	KV452730.1	13987213	2-scf00040	2
AX-157933494	3	68.579	KV452730.1	13989112	2-scf00040	2
AX-157933499	3	68.579	KV452730.1	13993057	2-scf00040	2
AX-157933500	3	68.579	KV452730.1	13993197	2-scf00040	2
AX-157862771	3	68.579	KV452730.1	13994259	2-scf00040	2
AX-157854425	3	68.579	KV452730.1	13997551	2-scf00040	2
AX-157869419	3	68.579	KV452730.1	13999770	2-scf00040	2
AX-157933506	3	68.579	KV452730.1	13999929	2-scf00040	2
AX-157933507	3	68.579	KV452730.1	14000830	2-scf00040	2
AX-157933509	3	68.579	KV452730.1	14002115	2-scf00040	2
AX-157933512	3	68.579	KV452730.1	14004799	2-scf00040	2
AX-157933516	3	68.579	KV452730.1	14007828	2-scf00040	2
AX-157869420	3	68.579	KV452730.1	14008211	2-scf00040	2
AX-157854427	3	68.579	KV452730.1	14010112	2-scf00040	2

AX-157869421	3	68.579	KV452730.1	14013159	2-scf00040	2
AX-158405572	3	68.579	KV452730.1	14014565	2-scf00040	2
AX-157854428	3	68.579	KV452730.1	14015571	2-scf00040	2
AX-157933529	3	68.579	KV452730.1	14017365	2-scf00040	2
AX-157854429	3	68.579	KV452730.1	14020119	2-scf00040	2
AX-157702632	3	68.579	KV452730.1	14022147	2-scf00040	2
AX-157854430	3	68.579	KV452730.1	14026121	2-scf00040	2
AX-157869424	3	68.579	KV452730.1	14028201	2-scf00040	2
AX-157933534	3	68.579	KV452730.1	14028303	2-scf00040	2
AX-157869425	3	68.579	KV452730.1	14028692	2-scf00040	2
AX-157854431	3	68.579	KV452730.1	14029133	2-scf00040	2
AX-157869426	3	68.579	KV452730.1	14029551	2-scf00040	2
AX-157933535	3	68.579	KV452730.1	14030707	2-scf00040	2
AX-157869427	3	68.579	KV452730.1	14031842	2-scf00040	2
AX-157933537	3	68.579	KV452730.1	14032424	2-scf00040	2
AX-157696693	3	68.579	KV452730.1	14035074	2-scf00040	2
AX-157933539	3	68.579	KV452730.1	14037505	2-scf00040	2
AX-157933549	3	68.579	KV452730.1	14043877	2-scf00040	2
AX-157933550	3	68.579	KV452730.1	14045514	2-scf00040	2
AX-157933565	3	68.579	KV452730.1	14065959	2-scf00040	2
AX-157933566	3	68.579	KV452730.1	14069014	2-scf00040	2
AX-158389990	3	68.579	KV452730.1	14076144	2-scf00040	2
AX-157814252	3	68.579	KV452730.1	14076910	2-scf00040	2
AX-157933593	3	68.579	KV452730.1	14114702	2-scf00040	2
AX-157933596	3	68.579	KV452730.1	14120994	2-scf00040	2
AX-157933607	3	68.579	KV452730.1	14135696	2-scf00040	2
AX-157933608	3	68.579	KV452730.1	14136814	2-scf00040	2
AX-157933609	3	68.579	KV452730.1	14137633	2-scf00040	2
AX-157933623	3	68.579	KV452730.1	14147346	2-scf00040	2
AX-157933626	3	68.579	KV452730.1	14149801	2-scf00040	2
AX-157933635	3	68.579	KV452730.1	14155843	2-scf00040	2
AX-157933648	3	68.579	KV452730.1	14167268	2-scf00040	2
AX-157933650	3	68.579	KV452730.1	14168604	2-scf00040	2
AX-157933651	3	68.579	KV452730.1	14169511	2-scf00040	2
AX-157814254	3	68.579	KV452730.1	14171413	2-scf00040	2
AX-157814255	3	68.579	KV452730.1	14172125	2-scf00040	2
AX-157874999	3	68.579	KV452730.1	14173588	2-scf00040	2
AX-157814256	3	68.579	KV452730.1	14175355	2-scf00040	2
AX-157933656	3	68.579	KV452730.1	14177904	2-scf00040	2
AX-157933667	3	68.579	KV452730.1	14194968	2-scf00040	2
AX-157933668	3	68.579	KV452730.1	14196359	2-scf00040	2
AX-157933670	3	68.579	KV452730.1	14197251	2-scf00040	2
AX-157933673	3	68.579	KV452730.1	14202407	2-scf00040	2
AX-157933676	3	68.579	KV452730.1	14207942	2-scf00040	2
AX-157875000	3	68.579	KV452730.1	14213977	2-scf00040	2
AX-157933681	3	68.579	KV452730.1	14218041	2-scf00040	2
AX-157933684	3	68.579	KV452730.1	14222615	2-scf00040	2
AX-157933688	3	68.579	KV452730.1	14230534	2-scf00040	2
AX-157933690	3	68.579	KV452730.1	14231668	2-scf00040	2
AX-158405573	3	68.579	KV452730.1	14236805	2-scf00040	2
AX-157933694	3	68.579	KV452730.1	14245035	2-scf00040	2

AX-157933695	3	68.579	KV452730.1	14247479	2-scf00040	2
AX-157933697	3	68.579	KV452730.1	14251133	2-scf00040	2
AX-157933699	3	68.579	KV452730.1	14257428	2-scf00040	2
AX-157933701	3	68.579	KV452730.1	14258110	2-scf00040	2
AX-157933702	3	68.579	KV452730.1	14258822	2-scf00040	2
AX-157933704	3	68.579	KV452730.1	14263231	2-scf00040	2
AX-157933705	3	68.579	KV452730.1	14263455	2-scf00040	2
AX-157933707	3	68.579	KV452730.1	14268156	2-scf00040	2
AX-157933711	3	68.579	KV452730.1	14276110	2-scf00040	2
AX-157933712	3	68.579	KV452730.1	14276706	2-scf00040	2
AX-157933727	3	68.579	KV452730.1	14302488	2-scf00040	2
AX-157933729	3	68.579	KV452730.1	14304407	2-scf00040	2
AX-157814262	3	68.579	KV452730.1	14342882	2-scf00040	2
AX-157933740	3	68.579	KV452730.1	14358849	2-scf00040	2
AX-157933741	3	68.579	KV452730.1	14360440	2-scf00040	2
AX-157933743	3	68.579	KV452730.1	14364000	2-scf00040	2
AX-157933748	3	68.579	KV452730.1	14406226	2-scf00040	2
AX-157933753	3	68.579	KV452730.1	14418815	2-scf00040	2
AX-157933755	3	68.579	KV452730.1	14419482	2-scf00040	2
AX-157933756	3	68.579	KV452730.1	14421385	2-scf00040	2
AX-157814264	3	68.579	KV452730.1	14422554	2-scf00040	2
AX-157933757	3	68.579	KV452730.1	14422995	2-scf00040	2
AX-157933761	3	68.579	KV452730.1	14431416	2-scf00040	2
AX-157933762	3	68.579	KV452730.1	14436190	2-scf00040	2
AX-157933764	3	68.579	KV452730.1	14440246	2-scf00040	2
AX-157933766	3	68.579	KV452730.1	14442023	2-scf00040	2
AX-157933771	3	68.579	KV452730.1	14457714	2-scf00040	2
AX-157933772	3	68.579	KV452730.1	14457870	2-scf00040	2
AX-157933775	3	68.579	KV452730.1	14467546	2-scf00040	2
AX-157933776	3	68.579	KV452730.1	14468756	2-scf00040	2
AX-157933779	3	68.579	KV452730.1	14474085	2-scf00040	2
AX-157933781	3	68.579	KV452730.1	14476227	2-scf00040	2
AX-157933782	3	68.579	KV452730.1	14481404	2-scf00040	2
AX-157933785	3	68.579	KV452730.1	14494271	2-scf00040	2
AX-157933786	3	68.579	KV452730.1	14496289	2-scf00040	2
AX-157933788	3	68.579	KV452730.1	14496761	2-scf00040	2
AX-157933792	3	68.579	KV452730.1	14498213	2-scf00040	2
AX-157933794	3	68.579	KV452730.1	14503797	2-scf00040	2
AX-157933798	3	68.579	KV452730.1	14507685	2-scf00040	2
AX-157933799	3	68.579	KV452730.1	14508591	2-scf00040	2
AX-157933800	3	68.579	KV452730.1	14514301	2-scf00040	2
AX-157854448	3	68.579	KV452730.1	14521663	2-scf00040	2
AX-157933809	3	68.579	KV452730.1	14532375	2-scf00040	2
AX-157933814	3	68.579	KV452730.1	14542059	2-scf00040	2
AX-157933815	3	68.579	KV452730.1	14543613	2-scf00040	2
AX-157933817	3	68.579	KV452730.1	14550525	2-scf00040	2
AX-157933822	3	68.579	KV452730.1	14563512	2-scf00040	2
AX-157933835	3	68.579	KV452730.1	14587825	2-scf00040	2
AX-157933838	3	68.579	KV452730.1	14590417	2-scf00040	2
AX-157854449	3	68.579	KV452730.1	14615108	2-scf00040	2
AX-157933848	3	68.579	KV452730.1	14616032	2-scf00040	2

AX-157933853	3	68.579	KV452730.1	14624221	2-scf00040	2
AX-157933859	3	68.579	KV452730.1	14628526	2-scf00040	2
AX-157933866	3	68.579	KV452730.1	14655287	2-scf00040	2
AX-157933869	3	68.579	KV452730.1	14661058	2-scf00040	2
AX-157933873	3	68.579	KV452730.1	14673413	2-scf00040	2
AX-157933875	3	68.579	KV452730.1	14676317	2-scf00040	2
AX-157933885	3	68.579	KV452733.1	7590	2-scf00041	2
AX-157933899	3	68.579	KV452733.1	21642	2-scf00041	2
AX-157933902	3	68.579	KV452733.1	24590	2-scf00041	2
AX-157933907	3	68.579	KV452733.1	32827	2-scf00041	2
AX-157933908	3	68.579	KV452733.1	34347	2-scf00041	2
AX-157933910	3	68.579	KV452733.1	36044	2-scf00041	2
AX-157933911	3	68.579	KV452733.1	37744	2-scf00041	2
AX-157933912	3	68.579	KV452733.1	38808	2-scf00041	2
AX-157933915	3	68.579	KV452733.1	42517	2-scf00041	2
AX-157933916	3	68.579	KV452733.1	43184	2-scf00041	2
AX-157933918	3	68.579	KV452733.1	44791	2-scf00041	2
AX-157933930	3	68.579	KV452733.1	54294	2-scf00041	2
AX-157933932	3	68.579	KV452733.1	55705	2-scf00041	2
AX-157933933	3	68.579	KV452733.1	56426	2-scf00041	2
AX-157933935	3	68.579	KV452733.1	60052	2-scf00041	2
AX-157933936	3	68.579	KV452733.1	60722	2-scf00041	2
AX-157933937	3	68.579	KV452733.1	65013	2-scf00041	2
AX-157933944	3	68.579	KV452733.1	70244	2-scf00041	2
AX-157933947	3	68.579	KV452733.1	72617	2-scf00041	2
AX-157933960	3	68.579	KV452733.1	84462	2-scf00041	2
AX-157814276	3	68.579	KV452733.1	85438	2-scf00041	2
AX-157814277	3	68.579	KV452733.1	87713	2-scf00041	2
AX-157751010	3	68.579	KV452733.1	89968	2-scf00041	2
AX-157933973	3	68.579	KV452733.1	98715	2-scf00041	2
AX-157933978	3	68.579	KV452733.1	99519	2-scf00041	2
AX-157933986	3	68.579	KV452733.1	107180	2-scf00041	2
AX-157814282	3	68.579	KV452733.1	107605	2-scf00041	2
AX-157933993	3	68.579	KV452733.1	113092	2-scf00041	2
AX-157869430	3	68.579	KV452733.1	115734	2-scf00041	2
AX-157740801	3	68.579	KV452733.1	120954	2-scf00041	2
AX-157814284	3	68.579	KV452733.1	122689	2-scf00041	2
AX-157713581	3	68.579	KV452733.1	124275	2-scf00041	2
AX-157744461	3	68.579	KV452733.1	125084	2-scf00041	2
AX-157934008	3	68.579	KV452733.1	128400	2-scf00041	2
AX-157934009	3	68.579	KV452733.1	128812	2-scf00041	2
AX-157934030	3	68.579	KV452733.1	139280	2-scf00041	2
AX-157934036	3	68.579	KV452733.1	154040	2-scf00041	2
AX-157755953	3	68.579	KV452733.1	166761	2-scf00041	2
AX-157753209	3	68.579	KV452733.1	178148	2-scf00041	2
AX-157709254	3	68.579	KV452733.1	180656	2-scf00041	2
AX-157934079	3	68.579	KV452733.1	199530	2-scf00041	2
AX-157934083	3	68.579	KV452733.1	205093	2-scf00041	2
AX-157934088	3	68.579	KV452733.1	211503	2-scf00041	2
AX-157934109	3	68.579	KV452733.1	229180	2-scf00041	2
AX-157934153	3	68.579	KV452733.1	262733	2-scf00041	2

AX-157934173	3	68.579	KV452733.1	283805	2-scf00041	2
AX-157934226	3	68.579	KV452733.1	321171	2-scf00041	2
AX-157934227	3	68.579	KV452733.1	322919	2-scf00041	2
AX-157934241	3	68.579	KV452733.1	327410	2-scf00041	2
AX-157814291	3	68.579	KV452733.1	327893	2-scf00041	2
AX-157934245	3	68.579	KV452733.1	334532	2-scf00041	2
AX-157726192	3	68.579	KV452733.1	339418	2-scf00041	2
AX-157934267	3	68.579	KV452733.1	374739	2-scf00041	2
AX-157934268	3	68.579	KV452733.1	375349	2-scf00041	2
AX-157934278	3	68.579	KV452733.1	390392	2-scf00041	2
AX-157934340	3	68.579	KV452733.1	480570	2-scf00041	2
AX-157934391	3	68.579	KV452733.1	524776	2-scf00041	2
AX-157934547	3	68.579	KV452733.1	666547	2-scf00041	2
AX-157934549	3	68.579	KV452733.1	666885	2-scf00041	2
AX-157934605	3	68.579	KV452733.1	732357	2-scf00041	2
AX-157934610	3	68.579	KV452733.1	740761	2-scf00041	2
AX-157935267	3	68.579	KV452733.1	1407920	2-scf00041	2
AX-157935550	3	68.579	KV452733.1	1729670	2-scf00041	2
AX-157935711	3	68.579	KV452733.1	1952123	2-scf00041	2
AX-157935924	3	68.579	KV452733.1	2311358	2-scf00041	2
AX-157814376	3	68.579	KV452733.1	2314617	2-scf00041	2
AX-157936393	3	68.579	KV452733.1	2767800	2-scf00041	2
AX-157936984	3	68.579	KV452733.1	3351701	2-scf00041	2
AX-157936985	3	68.579	KV452733.1	3354449	2-scf00041	2
AX-157937097	3	68.579	KV452733.1	3449018	2-scf00041	2
AX-157710815	3	68.579	KV452733.1	3476503	2-scf00041	2
AX-157937158	3	68.579	KV452733.1	3506442	2-scf00041	2
AX-157814440	3	68.579	KV452733.1	3572432	2-scf00041	2
AX-157937452	3	68.579	KV452732.1	9141	2-scf00042	2
AX-157937454	3	68.579	KV452732.1	12980	2-scf00042	2
AX-157937457	3	68.579	KV452732.1	14074	2-scf00042	2
AX-157937458	3	68.579	KV452732.1	16229	2-scf00042	2
AX-157937460	3	68.579	KV452732.1	22197	2-scf00042	2
AX-157937462	3	68.579	KV452732.1	23563	2-scf00042	2
AX-157937470	3	68.579	KV452732.1	39230	2-scf00042	2
AX-157937492	3	68.579	KV452732.1	119281	2-scf00042	2
AX-157937495	3	68.579	KV452732.1	140448	2-scf00042	2
AX-157937496	3	68.579	KV452732.1	149285	2-scf00042	2
AX-157814466	3	68.579	KV452732.1	156852	2-scf00042	2
AX-158405656	3	68.579	KV452732.1	161247	2-scf00042	2
AX-157937502	3	68.579	KV452732.1	166399	2-scf00042	2
AX-157814467	3	68.579	KV452732.1	173782	2-scf00042	2
AX-157937505	3	68.579	KV452732.1	177507	2-scf00042	2
AX-157937506	3	68.579	KV452732.1	180452	2-scf00042	2
AX-157937510	3	68.579	KV452732.1	193377	2-scf00042	2
AX-157937511	3	68.579	KV452732.1	195395	2-scf00042	2
AX-157937512	3	68.579	KV452732.1	198856	2-scf00042	2
AX-157937513	3	68.579	KV452732.1	203787	2-scf00042	2
AX-157714512	3	68.579	KV452732.1	210173	2-scf00042	2
AX-157937515	3	68.579	KV452732.1	214335	2-scf00042	2
AX-157937516	3	68.579	KV452732.1	217448	2-scf00042	2

AX-157937517	3	68.579	KV452732.1	218684	2-scf00042	2
AX-157937518	3	68.579	KV452732.1	224126	2-scf00042	2
AX-157937519	3	68.579	KV452732.1	226453	2-scf00042	2
AX-157937520	3	68.579	KV452732.1	229434	2-scf00042	2
AX-157937525	3	68.579	KV452732.1	239213	2-scf00042	2
AX-157937526	3	68.579	KV452732.1	242912	2-scf00042	2
AX-157937529	3	68.579	KV452732.1	255532	2-scf00042	2
AX-157937531	3	68.579	KV452732.1	258525	2-scf00042	2
AX-157937532	3	68.579	KV452732.1	264033	2-scf00042	2
AX-157937536	3	68.579	KV452732.1	268483	2-scf00042	2
AX-157937545	3	68.579	KV452732.1	290932	2-scf00042	2
AX-157937552	3	68.579	KV452732.1	297835	2-scf00042	2
AX-157937564	3	68.579	KV452732.1	313459	2-scf00042	2
AX-157937569	3	68.579	KV452732.1	318085	2-scf00042	2
AX-157937570	3	68.579	KV452732.1	326984	2-scf00042	2
AX-157937572	3	68.579	KV452732.1	327642	2-scf00042	2
AX-157937582	3	68.579	KV452732.1	342587	2-scf00042	2
AX-158390021	3	68.579	KV452732.1	346299	2-scf00042	2
AX-157937586	3	68.579	KV452732.1	346672	2-scf00042	2
AX-157937592	3	68.579	KV452732.1	361299	2-scf00042	2
AX-157937595	3	68.579	KV452732.1	364518	2-scf00042	2
AX-157937598	3	68.579	KV452732.1	373402	2-scf00042	2
AX-157937600	3	68.579	KV452732.1	375684	2-scf00042	2
AX-157937605	3	68.579	KV452732.1	388512	2-scf00042	2
AX-157937607	3	68.579	KV452732.1	389376	2-scf00042	2
AX-157937615	3	68.579	KV452732.1	417992	2-scf00042	2
AX-157937620	3	68.579	KV452732.1	426892	2-scf00042	2
AX-157937621	3	68.579	KV452732.1	437133	2-scf00042	2
AX-157937623	3	68.579	KV452732.1	438402	2-scf00042	2
AX-157937630	3	68.579	KV452732.1	442810	2-scf00042	2
AX-157937631	3	68.579	KV452732.1	446363	2-scf00042	2
AX-157937637	3	68.579	KV452732.1	460246	2-scf00042	2
AX-157937656	3	68.579	KV452732.1	500177	2-scf00042	2
AX-157937658	3	68.579	KV452732.1	503176	2-scf00042	2
AX-157937666	3	68.579	KV452732.1	519947	2-scf00042	2
AX-157937680	3	68.579	KV452732.1	533537	2-scf00042	2
AX-157937681	3	68.579	KV452732.1	537705	2-scf00042	2
AX-157937683	3	68.579	KV452732.1	546205	2-scf00042	2
AX-157937686	3	68.579	KV452732.1	547757	2-scf00042	2
AX-157937696	3	68.579	KV452732.1	558723	2-scf00042	2
AX-157937700	3	68.579	KV452732.1	563977	2-scf00042	2
AX-157814470	3	68.579	KV452732.1	570778	2-scf00042	2
AX-157814471	3	68.579	KV452732.1	576695	2-scf00042	2
AX-157718588	3	68.579	KV452732.1	580423	2-scf00042	2
AX-157937712	3	68.579	KV452732.1	584773	2-scf00042	2
AX-157937717	3	68.579	KV452732.1	589900	2-scf00042	2
AX-157937719	3	68.579	KV452732.1	596652	2-scf00042	2
AX-157937720	3	68.579	KV452732.1	599685	2-scf00042	2
AX-157937728	3	68.579	KV452732.1	607753	2-scf00042	2
AX-157937735	3	68.579	KV452732.1	633202	2-scf00042	2
AX-157937739	3	68.579	KV452732.1	634561	2-scf00042	2

AX-157937740	3	68.579	KV452732.1	641648	2-scf00042	2
AX-157937742	3	68.579	KV452732.1	642178	2-scf00042	2
AX-157937743	3	68.579	KV452732.1	643555	2-scf00042	2
AX-157937744	3	68.579	KV452732.1	643626	2-scf00042	2
AX-157937745	3	68.579	KV452732.1	645624	2-scf00042	2
AX-157937746	3	68.579	KV452732.1	646409	2-scf00042	2
AX-157814473	3	68.579	KV452732.1	652076	2-scf00042	2
AX-157937750	3	68.579	KV452732.1	660102	2-scf00042	2
AX-157937751	3	68.579	KV452732.1	661196	2-scf00042	2
AX-157937754	3	68.579	KV452732.1	666937	2-scf00042	2
AX-157814475	3	68.579	KV452732.1	687670	2-scf00042	2
AX-157937758	3	68.579	KV452732.1	689897	2-scf00042	2
AX-157937762	3	68.579	KV452732.1	697052	2-scf00042	2
AX-157937764	3	68.579	KV452732.1	697639	2-scf00042	2
AX-157937766	3	68.579	KV452732.1	702436	2-scf00042	2
AX-157814478	3	68.579	KV452732.1	722918	2-scf00042	2
AX-157709171	3	68.579	KV452732.1	724651	2-scf00042	2
AX-157937778	3	68.579	KV452732.1	741083	2-scf00042	2
AX-157937780	3	68.579	KV452732.1	742150	2-scf00042	2
AX-157937781	3	68.579	KV452732.1	743464	2-scf00042	2
AX-157937782	3	68.579	KV452732.1	744112	2-scf00042	2
AX-157937788	3	68.579	KV452732.1	753385	2-scf00042	2
AX-157814481	3	68.579	KV452732.1	754517	2-scf00042	2
AX-157937790	3	68.579	KV452732.1	757447	2-scf00042	2
AX-157814482	3	68.579	KV452732.1	760524	2-scf00042	2
AX-157937793	3	68.579	KV452732.1	764085	2-scf00042	2
AX-158379368	3	68.579	KV452732.1	766365	2-scf00042	2
AX-157746272	3	68.579	KV452732.1	766914	2-scf00042	2
AX-157744676	3	68.579	KV452732.1	769321	2-scf00042	2
AX-157814483	3	68.579	KV452732.1	769586	2-scf00042	2
AX-157701603	3	68.579	KV452732.1	772015	2-scf00042	2
AX-157937798	3	68.579	KV452732.1	776226	2-scf00042	2
AX-157937799	3	68.579	KV452732.1	776373	2-scf00042	2
AX-157937801	3	68.579	KV452732.1	781481	2-scf00042	2
AX-157937803	3	68.579	KV452732.1	782137	2-scf00042	2
AX-157937806	3	68.579	KV452732.1	784441	2-scf00042	2
AX-157937808	3	68.579	KV452732.1	784895	2-scf00042	2
AX-157937809	3	68.579	KV452732.1	785131	2-scf00042	2
AX-157937813	3	68.579	KV452732.1	794296	2-scf00042	2
AX-157814484	3	68.579	KV452732.1	796464	2-scf00042	2
AX-157937817	3	68.579	KV452732.1	796634	2-scf00042	2
AX-157774459	3	68.579	KV452732.1	796766	2-scf00042	2
AX-157937818	3	68.579	KV452732.1	796903	2-scf00042	2
AX-157937822	3	68.579	KV452732.1	798113	2-scf00042	2
AX-157814486	3	68.579	KV452732.1	799877	2-scf00042	2
AX-157814487	3	68.579	KV452732.1	801433	2-scf00042	2
AX-157718145	3	68.579	KV452732.1	801647	2-scf00042	2
AX-157937832	3	68.579	KV452732.1	810617	2-scf00042	2
AX-157937835	3	68.579	KV452732.1	811997	2-scf00042	2
AX-157937836	3	68.579	KV452732.1	813809	2-scf00042	2
AX-157937839	3	68.579	KV452732.1	815348	2-scf00042	2

AX-157937845	3	68.579	KV452732.1	838561	2-scf00042	2
AX-157937850	3	68.579	KV452732.1	850944	2-scf00042	2
AX-157709216	3	68.579	KV452732.1	859828	2-scf00042	2
AX-157937853	3	68.579	KV452732.1	860803	2-scf00042	2
AX-157814492	3	68.579	KV452732.1	862569	2-scf00042	2
AX-157937854	3	68.579	KV452732.1	863708	2-scf00042	2
AX-157937860	3	68.579	KV452732.1	875991	2-scf00042	2
AX-157937861	3	68.579	KV452732.1	879266	2-scf00042	2
AX-157814494	3	68.579	KV452732.1	880047	2-scf00042	2
AX-157937867	3	68.579	KV452732.1	883165	2-scf00042	2
AX-157937868	3	68.579	KV452732.1	884216	2-scf00042	2
AX-157937870	3	68.579	KV452732.1	884850	2-scf00042	2
AX-157937871	3	68.579	KV452732.1	886006	2-scf00042	2
AX-157937873	3	68.579	KV452732.1	886580	2-scf00042	2
AX-157937875	3	68.579	KV452732.1	887842	2-scf00042	2
AX-157814495	3	68.579	KV452732.1	888173	2-scf00042	2
AX-157937876	3	68.579	KV452732.1	888314	2-scf00042	2
AX-157814496	3	68.579	KV452732.1	889075	2-scf00042	2
AX-157937881	3	68.579	KV452732.1	893543	2-scf00042	2
AX-157937883	3	68.579	KV452732.1	896546	2-scf00042	2
AX-157814497	3	68.579	KV452732.1	897064	2-scf00042	2
AX-157937884	3	68.579	KV452732.1	897614	2-scf00042	2
AX-157937887	3	68.579	KV452732.1	899408	2-scf00042	2
AX-157937888	3	68.579	KV452732.1	899977	2-scf00042	2
AX-157937890	3	68.579	KV452732.1	900597	2-scf00042	2
AX-157937891	3	68.579	KV452732.1	900993	2-scf00042	2
AX-157814498	3	68.579	KV452732.1	905522	2-scf00042	2
AX-157937894	3	68.579	KV452732.1	910061	2-scf00042	2
AX-157814500	3	68.579	KV452732.1	911119	2-scf00042	2
AX-157814502	3	68.579	KV452732.1	912286	2-scf00042	2
AX-157740353	3	68.579	KV452732.1	913953	2-scf00042	2
AX-157717500	3	68.579	KV452732.1	915053	2-scf00042	2
AX-158405671	3	68.579	KV452732.1	919023	2-scf00042	2
AX-157937897	3	68.579	KV452732.1	919258	2-scf00042	2
AX-157937899	3	68.579	KV452732.1	921475	2-scf00042	2
AX-157937902	3	68.579	KV452732.1	922096	2-scf00042	2
AX-157937905	3	68.579	KV452732.1	922683	2-scf00042	2
AX-158390026	3	68.579	KV452732.1	923149	2-scf00042	2
AX-157937910	3	68.579	KV452732.1	931960	2-scf00042	2
AX-157937911	3	68.579	KV452732.1	932367	2-scf00042	2
AX-157937913	3	68.579	KV452732.1	937572	2-scf00042	2
AX-157714129	3	68.579	KV452732.1	938232	2-scf00042	2
AX-157937915	3	68.579	KV452732.1	939391	2-scf00042	2
AX-157718596	3	68.579	KV452732.1	940245	2-scf00042	2
AX-157937916	3	68.579	KV452732.1	940785	2-scf00042	2
AX-158405675	3	68.579	KV452732.1	943405	2-scf00042	2
AX-157937917	3	68.579	KV452732.1	944079	2-scf00042	2
AX-157937918	3	68.579	KV452732.1	946178	2-scf00042	2
AX-157937919	3	68.579	KV452732.1	946657	2-scf00042	2
AX-157937921	3	68.579	KV452732.1	947009	2-scf00042	2
AX-157814503	3	68.579	KV452732.1	950094	2-scf00042	2

AX-157937924	3	68.579	KV452732.1	950786	2-scf00042	2
AX-157937925	3	68.579	KV452732.1	951219	2-scf00042	2
AX-157937926	3	68.579	KV452732.1	951550	2-scf00042	2
AX-157937927	3	68.579	KV452732.1	955260	2-scf00042	2
AX-157937929	3	68.579	KV452732.1	955580	2-scf00042	2
AX-157937930	3	68.579	KV452732.1	955863	2-scf00042	2
AX-157937932	3	68.579	KV452732.1	956064	2-scf00042	2
AX-157814506	3	68.579	KV452732.1	974818	2-scf00042	2
AX-157937936	3	68.579	KV452732.1	979856	2-scf00042	2
AX-157814508	3	68.579	KV452732.1	979940	2-scf00042	2
AX-157937944	3	68.579	KV452732.1	994242	2-scf00042	2
AX-157937945	3	68.579	KV452732.1	995746	2-scf00042	2
AX-157814509	3	68.579	KV452732.1	996097	2-scf00042	2
AX-157937946	3	68.579	KV452732.1	996749	2-scf00042	2
AX-157937948	3	68.579	KV452732.1	997905	2-scf00042	2
AX-157937950	3	68.579	KV452732.1	1007173	2-scf00042	2
AX-157937951	3	68.579	KV452732.1	1008344	2-scf00042	2
AX-157937954	3	68.579	KV452732.1	1020635	2-scf00042	2
AX-157937956	3	68.579	KV452732.1	1031878	2-scf00042	2
AX-157883851	3	68.579	KV452732.1	1032442	2-scf00042	2
AX-157937958	3	68.579	KV452732.1	1035420	2-scf00042	2
AX-157937966	3	68.579	KV452732.1	1054755	2-scf00042	2
AX-157937968	3	68.579	KV452732.1	1055235	2-scf00042	2
AX-157937971	3	68.579	KV452732.1	1063868	2-scf00042	2
AX-157937973	3	68.579	KV452732.1	1067977	2-scf00042	2
AX-157937975	3	68.579	KV452732.1	1069563	2-scf00042	2
AX-157937980	3	68.579	KV452732.1	1079425	2-scf00042	2
AX-157937984	3	68.579	KV452732.1	1082828	2-scf00042	2
AX-157814511	3	68.579	KV452732.1	1101937	2-scf00042	2
AX-157937992	3	68.579	KV452732.1	1110014	2-scf00042	2
AX-157938000	3	68.579	KV452732.1	1126968	2-scf00042	2
AX-157938003	3	68.579	KV452732.1	1134582	2-scf00042	2
AX-157938010	3	68.579	KV452732.1	1145349	2-scf00042	2
AX-157938011	3	68.579	KV452732.1	1146130	2-scf00042	2
AX-157938013	3	68.579	KV452732.1	1147955	2-scf00042	2
AX-157938026	3	68.579	KV452732.1	1182873	2-scf00042	2
AX-157864729	3	68.579	KV452732.1	1191338	2-scf00042	2
AX-157938029	3	68.579	KV452732.1	1194126	2-scf00042	2
AX-157938031	3	68.579	KV452732.1	1194560	2-scf00042	2
AX-158405686	3	68.579	KV452732.1	1196749	2-scf00042	2
AX-157938036	3	68.579	KV452732.1	1208931	2-scf00042	2
AX-157938037	3	68.579	KV452732.1	1214257	2-scf00042	2
AX-157938039	3	68.579	KV452732.1	1218565	2-scf00042	2
AX-157938040	3	68.579	KV452732.1	1220163	2-scf00042	2
AX-158405690	3	68.579	KV452732.1	1224763	2-scf00042	2
AX-158405691	3	68.579	KV452732.1	1224800	2-scf00042	2
AX-157938043	3	68.579	KV452732.1	1226131	2-scf00042	2
AX-157814515	3	68.579	KV452732.1	1227380	2-scf00042	2
AX-157780232	3	68.579	KV452732.1	1229495	2-scf00042	2
AX-157869449	3	68.579	KV452732.1	1234832	2-scf00042	2
AX-157938048	3	68.579	KV452732.1	1236463	2-scf00042	2

AX-157938050	3	68.579	KV452732.1	1242533	2-scf00042	2
AX-157938055	3	68.579	KV452732.1	1248210	2-scf00042	2
AX-157814516	3	68.579	KV452732.1	1263977	2-scf00042	2
AX-157814517	3	68.579	KV452732.1	1270222	2-scf00042	2
AX-157862783	3	68.579	KV452732.1	1270314	2-scf00042	2
AX-157757114	3	68.579	KV452732.1	1270524	2-scf00042	2
AX-157938066	3	68.579	KV452732.1	1296277	2-scf00042	2
AX-157869450	3	68.579	KV452732.1	1298975	2-scf00042	2
AX-157938069	3	68.579	KV452732.1	1302163	2-scf00042	2
AX-157938072	3	68.579	KV452732.1	1311927	2-scf00042	2
AX-157938074	3	68.579	KV452732.1	1315406	2-scf00042	2
AX-157938075	3	68.579	KV452732.1	1315672	2-scf00042	2
AX-157938080	3	68.579	KV452732.1	1338057	2-scf00042	2
AX-157814519	3	68.579	KV452732.1	1342114	2-scf00042	2
AX-157938082	3	68.579	KV452732.1	1342302	2-scf00042	2
AX-157938085	3	68.579	KV452732.1	1347887	2-scf00042	2
AX-157938086	3	68.579	KV452732.1	1350959	2-scf00042	2
AX-158405693	3	68.579	KV452732.1	1354400	2-scf00042	2
AX-157938089	3	68.579	KV452732.1	1357083	2-scf00042	2
AX-157938091	3	68.579	KV452732.1	1357506	2-scf00042	2
AX-157854497	3	68.579	KV452732.1	1360311	2-scf00042	2
AX-157938096	3	68.579	KV452732.1	1366537	2-scf00042	2
AX-157938097	3	68.579	KV452732.1	1367470	2-scf00042	2
AX-157938099	3	68.579	KV452732.1	1368567	2-scf00042	2
AX-157938106	3	68.579	KV452732.1	1375475	2-scf00042	2
AX-157938107	3	68.579	KV452732.1	1376432	2-scf00042	2
AX-157938108	3	68.579	KV452732.1	1377551	2-scf00042	2
AX-157938111	3	68.579	KV452732.1	1378485	2-scf00042	2
AX-157938112	3	68.579	KV452732.1	1378913	2-scf00042	2
AX-157938116	3	68.579	KV452732.1	1385742	2-scf00042	2
AX-157938117	3	68.579	KV452732.1	1386536	2-scf00042	2
AX-157938125	3	68.579	KV452732.1	1396868	2-scf00042	2
AX-157938128	3	68.579	KV452732.1	1397755	2-scf00042	2
AX-157938134	3	68.579	KV452732.1	1403649	2-scf00042	2
AX-157938135	3	68.579	KV452732.1	1404451	2-scf00042	2
AX-157938137	3	68.579	KV452732.1	1407110	2-scf00042	2
AX-157938138	3	68.579	KV452732.1	1407674	2-scf00042	2
AX-157938139	3	68.579	KV452732.1	1414493	2-scf00042	2
AX-157938146	3	68.579	KV452732.1	1419550	2-scf00042	2
AX-157938148	3	68.579	KV452732.1	1421790	2-scf00042	2
AX-157938149	3	68.579	KV452732.1	1426129	2-scf00042	2
AX-157754107	3	68.579	KV452732.1	1430875	2-scf00042	2
AX-157938155	3	68.579	KV452732.1	1432068	2-scf00042	2
AX-157938158	3	68.579	KV452732.1	1437244	2-scf00042	2
AX-157938171	3	68.579	KV452732.1	1466544	2-scf00042	2
AX-157938173	3	68.579	KV452732.1	1466969	2-scf00042	2
AX-157702292	3	68.579	KV452732.1	1467069	2-scf00042	2
AX-157938189	3	68.579	KV452732.1	1468601	2-scf00042	2
AX-157938191	3	68.579	KV452732.1	1469698	2-scf00042	2
AX-157938192	3	68.579	KV452732.1	1472171	2-scf00042	2
AX-157938194	3	68.579	KV452732.1	1474511	2-scf00042	2

AX-157938196	3	68.579	KV452732.1	1475169	2-scf00042	2
AX-157938197	3	68.579	KV452732.1	1475615	2-scf00042	2
AX-157756532	3	68.579	KV452732.1	1477444	2-scf00042	2
AX-157938200	3	68.579	KV452732.1	1477957	2-scf00042	2
AX-157938201	3	68.579	KV452732.1	1478461	2-scf00042	2
AX-157938206	3	68.579	KV452732.1	1483495	2-scf00042	2
AX-157938207	3	68.579	KV452732.1	1483890	2-scf00042	2
AX-157938208	3	68.579	KV452732.1	1484337	2-scf00042	2
AX-157938211	3	68.579	KV452732.1	1486988	2-scf00042	2
AX-157938215	3	68.579	KV452732.1	1489700	2-scf00042	2
AX-157938217	3	68.579	KV452732.1	1494766	2-scf00042	2
AX-157938218	3	68.579	KV452732.1	1496615	2-scf00042	2
AX-157938224	3	68.579	KV452732.1	1500826	2-scf00042	2
AX-157938225	3	68.579	KV452732.1	1504566	2-scf00042	2
AX-157744739	3	68.579	KV452732.1	1504804	2-scf00042	2
AX-157938229	3	68.579	KV452732.1	1519689	2-scf00042	2
AX-157814523	3	68.579	KV452732.1	1521751	2-scf00042	2
AX-157814524	3	68.579	KV452732.1	1523554	2-scf00042	2
AX-157938232	3	68.579	KV452732.1	1527127	2-scf00042	2
AX-157938235	3	68.579	KV452732.1	1529708	2-scf00042	2
AX-157814527	3	68.579	KV452732.1	1543758	2-scf00042	2
AX-157938241	3	68.579	KV452732.1	1544828	2-scf00042	2
AX-157938242	3	68.579	KV452732.1	1546578	2-scf00042	2
AX-157682505	3	68.579	KV452731.1	2217	2-scf00043	2
AX-157938902	3	68.579	KV452742.1	551810	2-scf00046	2
AX-157875141	3	68.579	KV452742.1	976140	2-scf00046	2
AX-157941354	3	68.579	KV452742.1	1659747	2-scf00046	2
AX-157755810	3	68.579	KV452742.1	1660129	2-scf00046	2
AX-157941352	3	68.579	KV452742.1	1660623	2-scf00046	2
AX-157941095	3	68.579	KV452742.1	1940917	2-scf00046	2
AX-157939795	3	68.579	KV452742.1	2838003	2-scf00046	2
AX-157939992	3	68.579	KV452742.1	3068654	2-scf00046	2
AX-157814694	3	68.579	KV452742.1	3120336	2-scf00046	2
AX-158390048	3	68.579	KV452742.1	3235019	2-scf00046	2
AX-157940190	3	68.579	KV452742.1	3284013	2-scf00046	2
AX-157940205	3	68.579	KV452742.1	3298705	2-scf00046	2
AX-157940246	3	68.579	KV452742.1	3349094	2-scf00046	2
AX-158382627	3	68.579	KV452742.1	3656711	2-scf00046	2
AX-157814742	3	68.579	KV452742.1	3853542	2-scf00046	2
AX-157726411	3	68.579	KV452745.1	107676	2-scf00051	2
AX-157942347	3	68.579	KV452745.1	754305	2-scf00051	2
AX-157942504	3	68.579	KV452745.1	891848	2-scf00051	2
AX-157942584	3	68.579	KV452745.1	984823	2-scf00051	2
AX-157942682	3	68.579	KV452745.1	1160601	2-scf00051	2
AX-157943467	3	68.579	KV452747.1	440996	2-scf00053	2
AX-157943855	3	68.579	KV452747.1	845736	2-scf00053	2
AX-157945373	3	68.579	KV452746.1	229392	2-scf00054	2
AX-158405893	3	68.579	KV452746.1	334862	2-scf00054	2
AX-157706100	3	68.579	3ML01031607	202	scf07224_1	UN
AX-157706872	3	68.579	3ML01033060	151	scf08697_1	UN
AX-157707078	3	68.579	3ML01033463	59	scf09114_1	UN

AX-157746094	3	68.688	KV452730.1	10588023	2-scf00040	2
AX-157931855	3	68.688	KV452730.1	11555076	2-scf00040	2
AX-157814127	3	68.688	KV452730.1	11807901	2-scf00040	2
AX-157932398	3	68.688	KV452730.1	12213459	2-scf00040	2
AX-157933584	3	68.688	KV452730.1	14098103	2-scf00040	2
AX-157814265	3	68.688	KV452730.1	14425579	2-scf00040	2
AX-157933801	3	68.688	KV452730.1	14515963	2-scf00040	2
AX-157933922	3	68.688	KV452733.1	48039	2-scf00041	2
AX-157933923	3	68.688	KV452733.1	49732	2-scf00041	2
AX-157933925	3	68.688	KV452733.1	50219	2-scf00041	2
AX-157814275	3	68.688	KV452733.1	76567	2-scf00041	2
AX-157933951	3	68.688	KV452733.1	78591	2-scf00041	2
AX-157933956	3	68.688	KV452733.1	80397	2-scf00041	2
AX-158379354	3	68.688	KV452733.1	83975	2-scf00041	2
AX-157814278	3	68.688	KV452733.1	87992	2-scf00041	2
AX-157933961	3	68.688	KV452733.1	89060	2-scf00041	2
AX-157933968	3	68.688	KV452733.1	95800	2-scf00041	2
AX-157933970	3	68.688	KV452733.1	97512	2-scf00041	2
AX-157933974	3	68.688	KV452733.1	98998	2-scf00041	2
AX-157933980	3	68.688	KV452733.1	99824	2-scf00041	2
AX-157933982	3	68.688	KV452733.1	100873	2-scf00041	2
AX-157933983	3	68.688	KV452733.1	103414	2-scf00041	2
AX-157757048	3	68.688	KV452733.1	106995	2-scf00041	2
AX-157934000	3	68.688	KV452733.1	118243	2-scf00041	2
AX-157935718	3	68.688	KV452733.1	1958678	2-scf00041	2
AX-157937821	3	68.688	KV452732.1	797308	2-scf00042	2
AX-157814485	3	68.688	KV452732.1	797807	2-scf00042	2
AX-157814283	3	68.797	KV452733.1	121126	2-scf00041	2
AX-157874948	3	68.907	KV452730.1	12061345	2-scf00040	2
AX-157934006	3	68.907	KV452733.1	125952	2-scf00041	2
AX-157814285	3	68.907	KV452733.1	127253	2-scf00041	2
AX-157934011	3	68.907	KV452733.1	129111	2-scf00041	2
AX-157934025	3	68.907	KV452733.1	132279	2-scf00041	2
AX-157720477	3	68.907	KV452733.1	140809	2-scf00041	2
AX-157934031	3	68.907	KV452733.1	141570	2-scf00041	2
AX-157934033	3	68.907	KV452733.1	145772	2-scf00041	2
AX-157934034	3	68.907	KV452733.1	145956	2-scf00041	2
AX-157934050	3	68.907	KV452733.1	164185	2-scf00041	2
AX-157934052	3	68.907	KV452733.1	170013	2-scf00041	2
AX-157934062	3	68.907	KV452733.1	188118	2-scf00041	2
AX-157752708	3	68.999	KV452733.1	182290	2-scf00041	2
AX-157934071	3	69.234	KV452733.1	193451	2-scf00041	2
AX-157875012	3	69.235	KV452733.1	194875	2-scf00041	2
AX-157934073	3	69.235	KV452733.1	195164	2-scf00041	2
AX-157934082	3	69.235	KV452733.1	203072	2-scf00041	2
AX-157932651	3	69.453	KV452730.1	12520403	2-scf00040	2
AX-157932895	3	69.453	KV452730.1	13004023	2-scf00040	2
AX-157933276	3	69.453	KV452730.1	13695269	2-scf00040	2
AX-157933355	3	69.453	KV452730.1	13815215	2-scf00040	2
AX-157933525	3	69.453	KV452730.1	14013369	2-scf00040	2
AX-157934085	3	69.453	KV452733.1	206112	2-scf00041	2

AX-157934089	3	69.453	KV452733.1	212929	2-scf00041	2
AX-157937914	3	69.453	KV452732.1	938852	2-scf00042	2
AX-157938165	3	69.453	KV452732.1	1450717	2-scf00042	2
AX-157814521	3	69.453	KV452732.1	1452161	2-scf00042	2
AX-157758721	3	69.453	KV452732.1	1556308	2-scf00042	2
AX-157934092	3	69.563	KV452733.1	215187	2-scf00041	2
AX-157928986	3	69.672	KV452730.1	7887753	2-scf00040	2
AX-157929152	3	69.672	KV452730.1	8079473	2-scf00040	2
AX-157929609	3	69.672	KV452730.1	8821104	2-scf00040	2
AX-157930597	3	69.672	KV452730.1	10061018	2-scf00040	2
AX-157703711	3	69.672	KV452730.1	10425095	2-scf00040	2
AX-157931206	3	69.672	KV452730.1	10765526	2-scf00040	2
AX-157931952	3	69.672	KV452730.1	11699439	2-scf00040	2
AX-157709765	3	69.672	KV452730.1	12079845	2-scf00040	2
AX-157933683	3	69.672	KV452730.1	14221957	2-scf00040	2
AX-157710698	3	69.672	KV452733.1	215908	2-scf00041	2
AX-157934097	3	69.672	KV452733.1	218866	2-scf00041	2
AX-157934099	3	69.672	KV452733.1	220045	2-scf00041	2
AX-157755217	3	69.672	KV452733.1	221695	2-scf00041	2
AX-157934104	3	69.672	KV452733.1	225685	2-scf00041	2
AX-157934113	3	69.672	KV452733.1	231852	2-scf00041	2
AX-157934116	3	69.672	KV452733.1	234549	2-scf00041	2
AX-157934117	3	69.672	KV452733.1	235693	2-scf00041	2
AX-157934120	3	69.672	KV452733.1	236073	2-scf00041	2
AX-157934123	3	69.672	KV452733.1	237526	2-scf00041	2
AX-157709905	3	69.672	KV452733.1	253974	2-scf00041	2
AX-157814288	3	69.672	KV452733.1	260943	2-scf00041	2
AX-157934151	3	69.672	KV452733.1	261863	2-scf00041	2
AX-157883796	3	69.672	KV452733.1	267811	2-scf00041	2
AX-157937794	3	69.672	KV452732.1	769748	2-scf00042	2
AX-157934132	3	69.703	KV452733.1	243416	2-scf00041	2
AX-157934115	3	69.732	KV452733.1	232647	2-scf00041	2
AX-157934135	3	69.732	KV452733.1	245011	2-scf00041	2
AX-157934139	3	69.763	KV452733.1	247735	2-scf00041	2
AX-157934147	3	69.763	KV452733.1	256116	2-scf00041	2
AX-157771165	3	69.763	KV452733.1	271933	2-scf00041	2
AX-157934297	3	69.763	KV452733.1	426495	2-scf00041	2
AX-157934141	3	69.781	KV452733.1	250548	2-scf00041	2
AX-157934142	3	69.781	KV452733.1	254221	2-scf00041	2
AX-157934146	3	69.781	KV452733.1	255319	2-scf00041	2
AX-157934150	3	69.781	KV452733.1	260883	2-scf00041	2
AX-157814289	3	69.781	KV452733.1	262832	2-scf00041	2
AX-157934160	3	69.781	KV452733.1	267934	2-scf00041	2
AX-157934162	3	69.781	KV452733.1	268376	2-scf00041	2
AX-157934166	3	69.89	KV452733.1	279083	2-scf00041	2
AX-157934167	3	69.89	KV452733.1	280497	2-scf00041	2
AX-157934169	3	69.89	KV452733.1	281762	2-scf00041	2
AX-157934171	3	69.89	KV452733.1	282758	2-scf00041	2
AX-157772566	3	69.891	KV452733.1	285153	2-scf00041	2
AX-157934176	3	69.891	KV452733.1	286699	2-scf00041	2
AX-157934179	3	69.891	KV452733.1	294692	2-scf00041	2

AX-158405445	3	70.109	KV452730.1	7900826	2-scf00040	2
AX-157929019	3	70.109	KV452730.1	7919865	2-scf00040	2
AX-157929122	3	70.109	KV452730.1	8032202	2-scf00040	2
AX-157929556	3	70.109	KV452730.1	8764681	2-scf00040	2
AX-157929571	3	70.109	KV452730.1	8784318	2-scf00040	2
AX-157929574	3	70.109	KV452730.1	8785409	2-scf00040	2
AX-157748654	3	70.109	KV452730.1	8894849	2-scf00040	2
AX-157929656	3	70.109	KV452730.1	8911118	2-scf00040	2
AX-157930114	3	70.109	KV452730.1	9348846	2-scf00040	2
AX-157930152	3	70.109	KV452730.1	9377883	2-scf00040	2
AX-157930242	3	70.109	KV452730.1	9460141	2-scf00040	2
AX-157931144	3	70.109	KV452730.1	10695377	2-scf00040	2
AX-157931145	3	70.109	KV452730.1	10695931	2-scf00040	2
AX-157931400	3	70.109	KV452730.1	10989120	2-scf00040	2
AX-157931434	3	70.109	KV452730.1	11022670	2-scf00040	2
AX-157814089	3	70.109	KV452730.1	11244264	2-scf00040	2
AX-157749845	3	70.109	KV452730.1	11733715	2-scf00040	2
AX-157854152	3	70.109	KV452730.1	11740956	2-scf00040	2
AX-157932154	3	70.109	KV452730.1	11935673	2-scf00040	2
AX-157933945	3	70.109	KV452733.1	70682	2-scf00041	2
AX-157934129	3	70.109	KV452733.1	241630	2-scf00041	2
AX-157934144	3	70.109	KV452733.1	254795	2-scf00041	2
AX-157934184	3	70.109	KV452733.1	301109	2-scf00041	2
AX-157934189	3	70.109	KV452733.1	304558	2-scf00041	2
AX-157934207	3	70.109	KV452733.1	311393	2-scf00041	2
AX-157738473	3	70.109	KV452733.1	312171	2-scf00041	2
AX-157934212	3	70.109	KV452733.1	312474	2-scf00041	2
AX-157934218	3	70.109	KV452733.1	314286	2-scf00041	2
AX-157934219	3	70.109	KV452733.1	317825	2-scf00041	2
AX-157934223	3	70.109	KV452733.1	319607	2-scf00041	2
AX-157934232	3	70.109	KV452733.1	324335	2-scf00041	2
AX-157934249	3	70.109	KV452733.1	342368	2-scf00041	2
AX-157814292	3	70.109	KV452733.1	344146	2-scf00041	2
AX-157934252	3	70.109	KV452733.1	351742	2-scf00041	2
AX-157934254	3	70.109	KV452733.1	352517	2-scf00041	2
AX-158405588	3	70.109	KV452733.1	353169	2-scf00041	2
AX-157756144	3	70.109	KV452733.1	353533	2-scf00041	2
AX-157934256	3	70.109	KV452733.1	355627	2-scf00041	2
AX-157934259	3	70.109	KV452733.1	360019	2-scf00041	2
AX-157814293	3	70.109	KV452733.1	363587	2-scf00041	2
AX-157934262	3	70.109	KV452733.1	367174	2-scf00041	2
AX-157814294	3	70.109	KV452733.1	367229	2-scf00041	2
AX-157934264	3	70.109	KV452733.1	368312	2-scf00041	2
AX-157934272	3	70.109	KV452733.1	382113	2-scf00041	2
AX-157934273	3	70.109	KV452733.1	382474	2-scf00041	2
AX-157934277	3	70.109	KV452733.1	389540	2-scf00041	2
AX-157934284	3	70.109	KV452733.1	409580	2-scf00041	2
AX-157934285	3	70.109	KV452733.1	409951	2-scf00041	2
AX-157934295	3	70.109	KV452733.1	425576	2-scf00041	2
AX-157934296	3	70.109	KV452733.1	425959	2-scf00041	2
AX-157934298	3	70.109	KV452733.1	427108	2-scf00041	2

AX-157814296	3	70.109	KV452733.1	427457	2-scf00041	2
AX-158389997	3	70.109	KV452733.1	429305	2-scf00041	2
AX-157934299	3	70.109	KV452733.1	429837	2-scf00041	2
AX-157934302	3	70.109	KV452733.1	438072	2-scf00041	2
AX-157814297	3	70.109	KV452733.1	438942	2-scf00041	2
AX-157774646	3	70.109	KV452733.1	448096	2-scf00041	2
AX-157934308	3	70.109	KV452733.1	448177	2-scf00041	2
AX-157934311	3	70.109	KV452733.1	450219	2-scf00041	2
AX-157934313	3	70.109	KV452733.1	451310	2-scf00041	2
AX-157934314	3	70.109	KV452733.1	452067	2-scf00041	2
AX-157934325	3	70.109	KV452733.1	463452	2-scf00041	2
AX-157934326	3	70.109	KV452733.1	464219	2-scf00041	2
AX-157934328	3	70.109	KV452733.1	464880	2-scf00041	2
AX-157934330	3	70.109	KV452733.1	467036	2-scf00041	2
AX-157708124	3	70.109	KV452733.1	467691	2-scf00041	2
AX-157934333	3	70.109	KV452733.1	469084	2-scf00041	2
AX-157934336	3	70.109	KV452733.1	473615	2-scf00041	2
AX-157934349	3	70.109	KV452733.1	485053	2-scf00041	2
AX-157934350	3	70.109	KV452733.1	486371	2-scf00041	2
AX-157934352	3	70.109	KV452733.1	486937	2-scf00041	2
AX-157934354	3	70.109	KV452733.1	489108	2-scf00041	2
AX-157934361	3	70.109	KV452733.1	492107	2-scf00041	2
AX-157934395	3	70.109	KV452733.1	527792	2-scf00041	2
AX-157934396	3	70.109	KV452733.1	529155	2-scf00041	2
AX-157934398	3	70.109	KV452733.1	530120	2-scf00041	2
AX-157934401	3	70.109	KV452733.1	532728	2-scf00041	2
AX-157934406	3	70.109	KV452733.1	534967	2-scf00041	2
AX-157814300	3	70.109	KV452733.1	564202	2-scf00041	2
AX-157934464	3	70.109	KV452733.1	596369	2-scf00041	2
AX-157814480	3	70.109	KV452732.1	747524	2-scf00042	2
AX-157938045	3	70.109	KV452732.1	1232521	2-scf00042	2
AX-157943101	3	70.109	KV452747.1	31083	2-scf00053	2
AX-157945484	3	70.109	KV452746.1	339638	2-scf00054	2
AX-157934343	3	70.134	KV452733.1	483608	2-scf00041	2
AX-157727300	3	70.162	KV452733.1	490494	2-scf00041	2
AX-157934371	3	70.189	KV452733.1	499980	2-scf00041	2
AX-157814286	3	70.218	KV452733.1	239523	2-scf00041	2
AX-157934186	3	70.218	KV452733.1	301656	2-scf00041	2
AX-157934363	3	70.218	KV452733.1	492624	2-scf00041	2
AX-157934377	3	70.218	KV452733.1	503383	2-scf00041	2
AX-157934379	3	70.218	KV452733.1	505154	2-scf00041	2
AX-157934382	3	70.218	KV452733.1	510006	2-scf00041	2
AX-157934400	3	70.218	KV452733.1	532501	2-scf00041	2
AX-157775577	3	70.218	KV452733.1	533027	2-scf00041	2
AX-157934403	3	70.218	KV452733.1	533952	2-scf00041	2
AX-157934457	3	70.218	KV452733.1	592124	2-scf00041	2
AX-157937286	3	70.218	KV452733.1	3642746	2-scf00041	2
AX-157945344	3	70.218	KV452746.1	196018	2-scf00054	2
AX-157705697	3	70.218	BML01030637	261	scf06237_1	UN
AX-157934413	3	70.436	KV452733.1	540919	2-scf00041	2
AX-157934418	3	70.436	KV452733.1	547243	2-scf00041	2

AX-157934420	3	70.436	KV452733.1	548559	2-scf00041	2
AX-157934422	3	70.436	KV452733.1	552838	2-scf00041	2
AX-157934433	3	70.436	KV452733.1	564813	2-scf00041	2
AX-157934435	3	70.436	KV452733.1	570439	2-scf00041	2
AX-157934436	3	70.436	KV452733.1	571095	2-scf00041	2
AX-157934439	3	70.535	KV452733.1	575880	2-scf00041	2
AX-157814287	3	70.655	KV452733.1	251364	2-scf00041	2
AX-157934440	3	70.655	KV452733.1	576951	2-scf00041	2
AX-157934441	3	70.655	KV452733.1	579968	2-scf00041	2
AX-157724753	3	70.655	KV452733.1	584998	2-scf00041	2
AX-157934451	3	70.655	KV452733.1	589840	2-scf00041	2
AX-157934456	3	70.655	KV452733.1	591681	2-scf00041	2
AX-157934459	3	70.655	KV452733.1	593912	2-scf00041	2
AX-157934462	3	70.655	KV452733.1	594531	2-scf00041	2
AX-157934465	3	70.655	KV452733.1	596590	2-scf00041	2
AX-157934467	3	70.655	KV452733.1	596947	2-scf00041	2
AX-157934475	3	70.655	KV452733.1	603316	2-scf00041	2
AX-157934478	3	70.655	KV452733.1	605037	2-scf00041	2
AX-157934482	3	70.655	KV452733.1	609186	2-scf00041	2
AX-157934485	3	70.655	KV452733.1	611086	2-scf00041	2
AX-157934501	3	70.658	KV452733.1	620632	2-scf00041	2
AX-157723458	3	70.804	KV452733.1	624965	2-scf00041	2
AX-157723632	3	70.947	KV452733.1	630592	2-scf00041	2
AX-157934524	3	71.093	KV452733.1	644137	2-scf00041	2
AX-157934526	3	71.093	KV452733.1	648173	2-scf00041	2
AX-157934527	3	71.148	KV452733.1	649157	2-scf00041	2
AX-157934531	3	71.186	KV452733.1	650451	2-scf00041	2
AX-157934534	3	71.186	KV452733.1	661103	2-scf00041	2
AX-157934584	3	71.186	KV452733.1	713535	2-scf00041	2
AX-157814359	3	71.186	KV452733.1	1793213	2-scf00041	2
AX-157936046	3	71.186	KV452733.1	2434681	2-scf00041	2
AX-157941087	3	71.186	KV452742.1	1946881	2-scf00046	2
AX-157941081	3	71.186	KV452742.1	1953980	2-scf00046	2
AX-157814798	3	71.186	KV452742.1	1957291	2-scf00046	2
AX-157704687	3	71.186	3ML01027027	1138	scf02631_1	UN
AX-157706590	3	71.186	3ML01032531	263	scf08153_1	UN
AX-157932512	3	71.202	KV452730.1	12322939	2-scf00040	2
AX-157814253	3	71.202	KV452730.1	14164782	2-scf00040	2
AX-157934511	3	71.202	KV452733.1	631809	2-scf00041	2
AX-157934537	3	71.202	KV452733.1	663141	2-scf00041	2
AX-157934538	3	71.202	KV452733.1	663361	2-scf00041	2
AX-157934551	3	71.202	KV452733.1	667861	2-scf00041	2
AX-157757394	3	71.202	KV452733.1	668437	2-scf00041	2
AX-157934552	3	71.202	KV452733.1	671927	2-scf00041	2
AX-157934562	3	71.202	KV452733.1	686923	2-scf00041	2
AX-157936410	3	71.202	KV452733.1	2782043	2-scf00041	2
AX-157938533	3	71.202	KV452742.1	126032	2-scf00046	2
AX-157814776	3	71.202	KV452742.1	4430534	2-scf00046	2
AX-157883801	3	71.203	KV452733.1	682944	2-scf00041	2
AX-157934560	3	71.203	KV452733.1	684593	2-scf00041	2
AX-157934486	3	71.367	KV452733.1	611367	2-scf00041	2

AX-157934564	3	71.367	KV452733.1	688847	2-scf00041	2
AX-157933967	3	71.53	KV452733.1	94902	2-scf00041	2
AX-157934579	3	71.53	KV452733.1	710775	2-scf00041	2
AX-157934581	3	71.53	KV452733.1	711705	2-scf00041	2
AX-157934582	3	71.53	KV452733.1	712375	2-scf00041	2
AX-157943812	3	71.53	KV452747.1	816993	2-scf00053	2
AX-157934572	3	71.639	KV452733.1	702047	2-scf00041	2
AX-157934583	3	71.639	KV452733.1	713167	2-scf00041	2
AX-157934590	3	71.639	KV452733.1	719228	2-scf00041	2
AX-157934591	3	71.639	KV452733.1	719893	2-scf00041	2
AX-157934594	3	71.639	KV452733.1	720860	2-scf00041	2
AX-157934595	3	71.639	KV452733.1	725183	2-scf00041	2
AX-157723406	3	71.639	KV452733.1	725761	2-scf00041	2
AX-157934601	3	71.639	KV452733.1	729405	2-scf00041	2
AX-157934602	3	71.639	KV452733.1	730568	2-scf00041	2
AX-157934637	3	71.639	KV452733.1	775530	2-scf00041	2
AX-157934656	3	71.639	KV452733.1	798781	2-scf00041	2
AX-157934304	3	71.803	KV452733.1	441050	2-scf00041	2
AX-157934513	3	71.803	KV452733.1	633738	2-scf00041	2
AX-157934603	3	71.803	KV452733.1	731923	2-scf00041	2
AX-157934632	3	71.803	KV452733.1	771214	2-scf00041	2
AX-157934641	3	71.803	KV452733.1	782607	2-scf00041	2
AX-157934643	3	71.803	KV452733.1	783568	2-scf00041	2
AX-157934650	3	71.803	KV452733.1	791551	2-scf00041	2
AX-157934660	3	71.803	KV452733.1	800185	2-scf00041	2
AX-157934668	3	71.803	KV452733.1	805255	2-scf00041	2
AX-157854451	3	71.803	KV452733.1	819438	2-scf00041	2
AX-157934734	3	71.803	KV452733.1	876581	2-scf00041	2
AX-157719048	3	71.967	KV452733.1	745284	2-scf00041	2
AX-157934613	3	71.967	KV452733.1	745366	2-scf00041	2
AX-157934619	3	71.967	KV452733.1	753396	2-scf00041	2
AX-158382525	3	71.967	KV452733.1	798692	2-scf00041	2
AX-157934628	3	71.968	KV452733.1	769047	2-scf00041	2
AX-157934635	3	72.077	KV452733.1	771843	2-scf00041	2
AX-157934636	3	72.128	KV452733.1	773363	2-scf00041	2
AX-157934645	3	72.186	KV452733.1	785910	2-scf00041	2
AX-157934649	3	72.186	KV452733.1	790987	2-scf00041	2
AX-157934655	3	72.186	KV452733.1	796445	2-scf00041	2
AX-157934657	3	72.186	KV452733.1	799188	2-scf00041	2
AX-157934670	3	72.186	KV452733.1	809026	2-scf00041	2
AX-157934676	3	72.186	KV452733.1	819591	2-scf00041	2
AX-157934661	3	72.295	KV452733.1	801535	2-scf00041	2
AX-157934665	3	72.295	KV452733.1	803297	2-scf00041	2
AX-157940001	3	72.295	KV452742.1	3076801	2-scf00046	2
AX-157934667	3	72.338	KV452733.1	804530	2-scf00041	2
AX-157933123	3	72.405	KV452730.1	13363470	2-scf00040	2
AX-157814306	3	72.405	KV452733.1	822059	2-scf00041	2
AX-157934680	3	72.405	KV452733.1	824446	2-scf00041	2
AX-157937474	3	72.405	KV452732.1	58276	2-scf00042	2
AX-157937490	3	72.405	KV452732.1	104041	2-scf00042	2
AX-157939386	3	72.405	KV452742.1	1358206	2-scf00046	2

AX-157942501	3	72.405	KV452745.1	888938	2-scf00051	2
AX-157934683	3	72.624	KV452733.1	832963	2-scf00041	2
AX-157934688	3	72.711	KV452733.1	837099	2-scf00041	2
AX-157934692	3	72.847	KV452733.1	840744	2-scf00041	2
AX-157934693	3	72.847	KV452733.1	841657	2-scf00041	2
AX-157741461	3	72.847	KV452733.1	843351	2-scf00041	2
AX-157934696	3	72.847	KV452733.1	844091	2-scf00041	2
AX-157934265	3	72.964	KV452733.1	369198	2-scf00041	2
AX-157934697	3	72.964	KV452733.1	846326	2-scf00041	2
AX-157934720	3	72.996	KV452733.1	867555	2-scf00041	2
AX-157934723	3	72.996	KV452733.1	871555	2-scf00041	2
AX-157934724	3	73.114	KV452733.1	873067	2-scf00041	2
AX-157934740	3	73.114	KV452733.1	879995	2-scf00041	2
AX-157934727	3	73.225	KV452733.1	874145	2-scf00041	2
AX-157934733	3	73.225	KV452733.1	875560	2-scf00041	2
AX-157814308	3	73.368	KV452733.1	884514	2-scf00041	2
AX-157934745	3	73.511	KV452733.1	887029	2-scf00041	2
AX-157934746	3	73.511	KV452733.1	887876	2-scf00041	2
AX-157934748	3	73.511	KV452733.1	888323	2-scf00041	2
AX-157934752	3	73.934	KV452733.1	890873	2-scf00041	2
AX-157934754	3	73.939	KV452733.1	891742	2-scf00041	2
AX-157934758	3	73.939	KV452733.1	894024	2-scf00041	2
AX-157934759	3	73.939	KV452733.1	894383	2-scf00041	2
AX-157943757	3	73.939	KV452747.1	747721	2-scf00053	2
AX-157934769	3	74.23	KV452733.1	901402	2-scf00041	2
AX-157934773	3	74.23	KV452733.1	904481	2-scf00041	2
AX-157934799	3	74.23	KV452733.1	928974	2-scf00041	2
AX-157934807	3	74.23	KV452733.1	935849	2-scf00041	2
AX-157934781	3	74.376	KV452733.1	913510	2-scf00041	2
AX-157934785	3	74.704	KV452733.1	918354	2-scf00041	2
AX-157934787	3	74.704	KV452733.1	918754	2-scf00041	2
AX-157934788	3	74.733	KV452733.1	919994	2-scf00041	2
AX-157934797	3	74.733	KV452733.1	927526	2-scf00041	2
AX-157934790	3	74.753	KV452733.1	920521	2-scf00041	2
AX-157934791	3	74.774	KV452733.1	921605	2-scf00041	2
AX-157934792	3	74.793	KV452733.1	922455	2-scf00041	2
AX-157934806	3	74.793	KV452733.1	934276	2-scf00041	2
AX-157940202	3	74.793	KV452742.1	3297246	2-scf00046	2
AX-157723932	3	74.814	KV452733.1	924185	2-scf00041	2
AX-157934800	3	74.814	KV452733.1	930032	2-scf00041	2
AX-157934804	3	74.814	KV452733.1	932595	2-scf00041	2
AX-157934815	3	75.033	KV452733.1	939464	2-scf00041	2
AX-157934827	3	75.142	KV452733.1	953752	2-scf00041	2
AX-157934829	3	75.142	KV452733.1	958031	2-scf00041	2
AX-157720738	3	75.142	KV452733.1	967080	2-scf00041	2
AX-157756137	3	75.142	KV452733.1	967965	2-scf00041	2
AX-157934834	3	75.142	KV452733.1	969304	2-scf00041	2
AX-157934836	3	75.142	KV452733.1	972685	2-scf00041	2
AX-157934837	3	75.167	KV452733.1	974074	2-scf00041	2
AX-157934840	3	75.205	KV452733.1	977328	2-scf00041	2
AX-157934845	3	75.251	KV452733.1	978939	2-scf00041	2

AX-157934850	3	75.251	KV452733.1	981540	2-scf00041	2
AX-157934852	3	75.251	KV452733.1	982598	2-scf00041	2
AX-157934853	3	75.251	KV452733.1	984445	2-scf00041	2
AX-157934860	3	75.251	KV452733.1	988269	2-scf00041	2
AX-157854456	3	75.251	KV452733.1	995881	2-scf00041	2
AX-157934883	3	75.251	KV452733.1	1008128	2-scf00041	2
AX-157934892	3	75.251	KV452733.1	1018813	2-scf00041	2
AX-157939610	3	75.251	KV452742.1	1607245	2-scf00046	2
AX-157941998	3	75.251	KV452745.1	417941	2-scf00051	2
AX-157934861	3	75.36	KV452733.1	991321	2-scf00041	2
AX-157934888	3	75.36	KV452733.1	1017052	2-scf00041	2
AX-157934893	3	75.36	KV452733.1	1019249	2-scf00041	2
AX-157934895	3	75.36	KV452733.1	1020255	2-scf00041	2
AX-157934897	3	75.36	KV452733.1	1021007	2-scf00041	2
AX-158405597	3	75.36	KV452733.1	1021987	2-scf00041	2
AX-157752508	3	75.36	KV452733.1	1024951	2-scf00041	2
AX-157934914	3	75.426	KV452733.1	1039483	2-scf00041	2
AX-157934916	3	75.47	KV452733.1	1041290	2-scf00041	2
AX-157934917	3	75.508	KV452733.1	1042216	2-scf00041	2
AX-157934920	3	75.55	KV452733.1	1045792	2-scf00041	2
AX-157934922	3	75.579	KV452733.1	1047422	2-scf00041	2
AX-157725816	3	75.579	KV452733.1	1047736	2-scf00041	2
AX-157934933	3	75.579	KV452733.1	1054758	2-scf00041	2
AX-157934941	3	75.579	KV452733.1	1057316	2-scf00041	2
AX-157934943	3	75.579	KV452733.1	1058153	2-scf00041	2
AX-157934944	3	75.579	KV452733.1	1059032	2-scf00041	2
AX-157934946	3	75.579	KV452733.1	1059864	2-scf00041	2
AX-157934947	3	75.579	KV452733.1	1060136	2-scf00041	2
AX-157934948	3	75.579	KV452733.1	1061740	2-scf00041	2
AX-157934961	3	75.579	KV452733.1	1076055	2-scf00041	2
AX-157934973	3	75.579	KV452733.1	1092626	2-scf00041	2
AX-157934982	3	75.579	KV452733.1	1106100	2-scf00041	2
AX-157935035	3	75.579	KV452733.1	1148044	2-scf00041	2
AX-157935076	3	75.579	KV452733.1	1185742	2-scf00041	2
AX-157935113	3	75.579	KV452733.1	1236287	2-scf00041	2
AX-157935120	3	75.579	KV452733.1	1245178	2-scf00041	2
AX-157935122	3	75.579	KV452733.1	1245714	2-scf00041	2
AX-157935136	3	75.579	KV452733.1	1263043	2-scf00041	2
AX-157864682	3	75.59	KV452733.1	1060498	2-scf00041	2
AX-157814311	3	75.59	KV452733.1	1061471	2-scf00041	2
AX-157934958	3	75.59	KV452733.1	1074436	2-scf00041	2
AX-157934964	3	75.59	KV452733.1	1079648	2-scf00041	2
AX-157934966	3	75.59	KV452733.1	1080079	2-scf00041	2
AX-157934975	3	75.59	KV452733.1	1095606	2-scf00041	2
AX-157742589	3	75.59	KV452733.1	1151649	2-scf00041	2
AX-157935044	3	75.59	KV452733.1	1154938	2-scf00041	2
AX-157935138	3	75.59	KV452733.1	1263412	2-scf00041	2
AX-157814313	3	75.609	KV452733.1	1091328	2-scf00041	2
AX-157934969	3	75.632	KV452733.1	1091559	2-scf00041	2
AX-157934980	3	75.65	KV452733.1	1104313	2-scf00041	2
AX-157934987	3	75.678	KV452733.1	1112217	2-scf00041	2

AX-157934991	3	75.712	KV452733.1	1113445	2-scf00041	2
AX-157934993	3	75.758	KV452733.1	1114478	2-scf00041	2
AX-157934976	3	75.798	KV452733.1	1096115	2-scf00041	2
AX-157935003	3	75.798	KV452733.1	1121139	2-scf00041	2
AX-157935011	3	75.798	KV452733.1	1129083	2-scf00041	2
AX-157935013	3	75.798	KV452733.1	1129943	2-scf00041	2
AX-157935019	3	75.798	KV452733.1	1133021	2-scf00041	2
AX-157935031	3	75.798	KV452733.1	1144962	2-scf00041	2
AX-157935034	3	75.798	KV452733.1	1147741	2-scf00041	2
AX-157935039	3	75.798	KV452733.1	1151943	2-scf00041	2
AX-157935046	3	75.798	KV452733.1	1155864	2-scf00041	2
AX-157935052	3	75.798	KV452733.1	1158547	2-scf00041	2
AX-157717682	3	75.798	KV452733.1	1158861	2-scf00041	2
AX-157935060	3	75.798	KV452733.1	1162389	2-scf00041	2
AX-157935062	3	75.798	KV452733.1	1163944	2-scf00041	2
AX-157935063	3	75.846	KV452733.1	1164155	2-scf00041	2
AX-157935064	3	75.879	KV452733.1	1165843	2-scf00041	2
AX-157719264	3	75.907	KV452733.1	958847	2-scf00041	2
AX-157883806	3	75.907	KV452733.1	1063189	2-scf00041	2
AX-157883808	3	75.907	KV452733.1	1156410	2-scf00041	2
AX-157935050	3	75.907	KV452733.1	1158196	2-scf00041	2
AX-157814315	3	75.907	KV452733.1	1174791	2-scf00041	2
AX-157935067	3	75.907	KV452733.1	1176133	2-scf00041	2
AX-157935069	3	75.907	KV452733.1	1177892	2-scf00041	2
AX-157814317	3	75.907	KV452733.1	1178793	2-scf00041	2
AX-157935072	3	75.907	KV452733.1	1179905	2-scf00041	2
AX-157935073	3	75.907	KV452733.1	1180771	2-scf00041	2
AX-157935074	3	75.907	KV452733.1	1181177	2-scf00041	2
AX-157935075	3	75.907	KV452733.1	1183934	2-scf00041	2
AX-157740599	3	75.907	KV452733.1	1191093	2-scf00041	2
AX-157715250	3	75.907	KV452733.1	1196155	2-scf00041	2
AX-157935083	3	75.907	KV452733.1	1201155	2-scf00041	2
AX-157935085	3	75.907	KV452733.1	1202287	2-scf00041	2
AX-157714163	3	75.907	KV452733.1	1203142	2-scf00041	2
AX-157935089	3	75.907	KV452733.1	1205925	2-scf00041	2
AX-157935090	3	75.907	KV452733.1	1206184	2-scf00041	2
AX-157814320	3	75.907	KV452733.1	1207631	2-scf00041	2
AX-157714198	3	75.907	KV452733.1	1208303	2-scf00041	2
AX-157935092	3	75.907	KV452733.1	1209826	2-scf00041	2
AX-157935106	3	75.907	KV452733.1	1228858	2-scf00041	2
AX-157935108	3	75.907	KV452733.1	1230145	2-scf00041	2
AX-157935130	3	75.907	KV452733.1	1253348	2-scf00041	2
AX-157936370	3	75.907	KV452733.1	2740134	2-scf00041	2
AX-157741783	3	75.907	KV452742.1	935892	2-scf00046	2
AX-157883810	3	75.985	KV452733.1	1208562	2-scf00041	2
AX-157814323	3	76.052	KV452733.1	1215824	2-scf00041	2
AX-157717352	3	76.052	KV452733.1	1221096	2-scf00041	2
AX-157745876	3	76.125	KV452730.1	9844438	2-scf00040	2
AX-157814011	3	76.125	KV452730.1	10526212	2-scf00040	2
AX-157931171	3	76.125	KV452730.1	10723779	2-scf00040	2
AX-157934402	3	76.125	KV452733.1	533496	2-scf00041	2

AX-157934495	3	76.125	KV452733.1	616228	2-scf00041	2
AX-157934951	3	76.125	KV452733.1	1070313	2-scf00041	2
AX-157935042	3	76.125	KV452733.1	1154144	2-scf00041	2
AX-157814319	3	76.125	KV452733.1	1198665	2-scf00041	2
AX-157814324	3	76.125	KV452733.1	1219837	2-scf00041	2
AX-157935100	3	76.125	KV452733.1	1219873	2-scf00041	2
AX-157935101	3	76.125	KV452733.1	1221674	2-scf00041	2
AX-157697653	3	76.125	KV452733.1	1221997	2-scf00041	2
AX-157935102	3	76.125	KV452733.1	1222059	2-scf00041	2
AX-157935103	3	76.125	KV452733.1	1222418	2-scf00041	2
AX-157935104	3	76.125	KV452733.1	1223557	2-scf00041	2
AX-157935105	3	76.125	KV452733.1	1224502	2-scf00041	2
AX-157935107	3	76.125	KV452733.1	1229772	2-scf00041	2
AX-157935110	3	76.125	KV452733.1	1231512	2-scf00041	2
AX-157719337	3	76.125	KV452733.1	1240284	2-scf00041	2
AX-157935117	3	76.125	KV452733.1	1241417	2-scf00041	2
AX-157935119	3	76.125	KV452733.1	1244635	2-scf00041	2
AX-157935296	3	76.125	KV452733.1	1429590	2-scf00041	2
AX-157935814	3	76.125	KV452733.1	2105781	2-scf00041	2
AX-157936452	3	76.125	KV452733.1	2823678	2-scf00041	2
AX-158404759	3	76.125	KV452742.1	491635	2-scf00046	2
AX-157939835	3	76.125	KV452742.1	2893921	2-scf00046	2
AX-157727027	3	76.125	KV452742.1	2962905	2-scf00046	2
AX-157940326	3	76.125	KV452742.1	3422244	2-scf00046	2
AX-157943151	3	76.125	KV452747.1	99492	2-scf00053	2
AX-157935124	3	76.278	KV452733.1	1248183	2-scf00041	2
AX-157934968	3	76.344	KV452733.1	1086062	2-scf00041	2
AX-157715118	3	76.344	KV452733.1	1124743	2-scf00041	2
AX-157739766	3	76.344	KV452733.1	1188184	2-scf00041	2
AX-157935114	3	76.344	KV452733.1	1237241	2-scf00041	2
AX-157935126	3	76.344	KV452733.1	1251332	2-scf00041	2
AX-157935140	3	76.344	KV452733.1	1267641	2-scf00041	2
AX-157935143	3	76.344	KV452733.1	1268203	2-scf00041	2
AX-157935144	3	76.344	KV452733.1	1270230	2-scf00041	2
AX-157935148	3	76.344	KV452733.1	1271175	2-scf00041	2
AX-157935149	3	76.344	KV452733.1	1271697	2-scf00041	2
AX-157935151	3	76.344	KV452733.1	1275263	2-scf00041	2
AX-158405602	3	76.344	KV452733.1	1277561	2-scf00041	2
AX-157771415	3	76.344	KV452733.1	1280897	2-scf00041	2
AX-157935183	3	76.344	KV452733.1	1318179	2-scf00041	2
AX-157936264	3	76.344	KV452733.1	2643797	2-scf00041	2
AX-157773035	3	76.344	KV452733.1	2854279	2-scf00041	2
AX-157700662	3	76.344	KV452733.1	2953843	2-scf00041	2
AX-157935158	3	76.366	KV452733.1	1280261	2-scf00041	2
AX-157935159	3	76.393	KV452733.1	1281643	2-scf00041	2
AX-157814327	3	76.416	KV452733.1	1285598	2-scf00041	2
AX-157935163	3	76.453	KV452733.1	1286541	2-scf00041	2
AX-157935164	3	76.453	KV452733.1	1286911	2-scf00041	2
AX-157935168	3	76.453	KV452733.1	1292571	2-scf00041	2
AX-157935169	3	76.453	KV452733.1	1294033	2-scf00041	2
AX-157935179	3	76.453	KV452733.1	1310422	2-scf00041	2

AX-157935180	3	76.453	KV452733.1	1312716	2-scf00041	2
AX-157935185	3	76.453	KV452733.1	1318502	2-scf00041	2
AX-157935303	3	76.453	KV452733.1	1431597	2-scf00041	2
AX-157705726	3	76.453	3ML01030716	590	scf06316_1	UN
AX-157935196	3	76.562	KV452733.1	1326239	2-scf00041	2
AX-157935207	3	76.562	KV452733.1	1337594	2-scf00041	2
AX-157935208	3	76.562	KV452733.1	1340351	2-scf00041	2
AX-157935215	3	76.562	KV452733.1	1343802	2-scf00041	2
AX-157935217	3	76.562	KV452733.1	1344705	2-scf00041	2
AX-157935218	3	76.562	KV452733.1	1345283	2-scf00041	2
AX-157814330	3	76.562	KV452733.1	1346390	2-scf00041	2
AX-157935219	3	76.562	KV452733.1	1349175	2-scf00041	2
AX-157935220	3	76.562	KV452733.1	1352450	2-scf00041	2
AX-157935249	3	76.562	KV452733.1	1390202	2-scf00041	2
AX-157935250	3	76.562	KV452733.1	1390517	2-scf00041	2
AX-157935260	3	76.562	KV452733.1	1399797	2-scf00041	2
AX-157935268	3	76.562	KV452733.1	1408305	2-scf00041	2
AX-157935288	3	76.562	KV452733.1	1420470	2-scf00041	2
AX-157935310	3	76.562	KV452733.1	1436406	2-scf00041	2
AX-157935385	3	76.562	KV452733.1	1536886	2-scf00041	2
AX-157705688	3	76.562	3ML01030612	577	scf06212_1	UN
AX-157935225	3	76.616	KV452733.1	1359077	2-scf00041	2
AX-157935227	3	76.671	KV452733.1	1360392	2-scf00041	2
AX-157935228	3	76.671	KV452733.1	1361770	2-scf00041	2
AX-157935231	3	76.671	KV452733.1	1364857	2-scf00041	2
AX-157935241	3	76.671	KV452733.1	1380252	2-scf00041	2
AX-157935258	3	76.671	KV452733.1	1397129	2-scf00041	2
AX-157935271	3	76.671	KV452733.1	1409698	2-scf00041	2
AX-157935546	3	76.671	KV452733.1	1727790	2-scf00041	2
AX-157935600	3	76.671	KV452733.1	1813768	2-scf00041	2
AX-157935604	3	76.671	KV452733.1	1815580	2-scf00041	2
AX-157935610	3	76.671	KV452733.1	1819512	2-scf00041	2
AX-157935243	3	76.707	KV452733.1	1382310	2-scf00041	2
AX-157935244	3	76.741	KV452733.1	1382934	2-scf00041	2
AX-157945499	3	76.741	KV452746.1	357192	2-scf00054	2
AX-157935252	3	76.781	KV452733.1	1392413	2-scf00041	2
AX-157935255	3	76.781	KV452733.1	1396038	2-scf00041	2
AX-157935257	3	76.781	KV452733.1	1396764	2-scf00041	2
AX-157935270	3	76.781	KV452733.1	1409184	2-scf00041	2
AX-157935275	3	76.781	KV452733.1	1410617	2-scf00041	2
AX-157935276	3	76.781	KV452733.1	1411541	2-scf00041	2
AX-157935279	3	76.781	KV452733.1	1412438	2-scf00041	2
AX-157935285	3	76.781	KV452733.1	1417159	2-scf00041	2
AX-157935317	3	76.781	KV452733.1	1439261	2-scf00041	2
AX-157935616	3	76.781	KV452733.1	1821668	2-scf00041	2
AX-157935618	3	76.781	KV452733.1	1823121	2-scf00041	2
AX-157935623	3	76.781	KV452733.1	1826226	2-scf00041	2
AX-157935300	3	76.999	KV452733.1	1431147	2-scf00041	2
AX-157935331	3	76.999	KV452733.1	1460347	2-scf00041	2
AX-157935342	3	76.999	KV452733.1	1475026	2-scf00041	2
AX-157935354	3	76.999	KV452733.1	1503065	2-scf00041	2

AX-157935359	3	76.999	KV452733.1	1512194	2-scf00041	2
AX-157935381	3	76.999	KV452733.1	1533841	2-scf00041	2
AX-157935382	3	76.999	KV452733.1	1535745	2-scf00041	2
AX-157935394	3	76.999	KV452733.1	1546105	2-scf00041	2
AX-157935400	3	76.999	KV452733.1	1552731	2-scf00041	2
AX-157935416	3	76.999	KV452733.1	1562473	2-scf00041	2
AX-157814341	3	76.999	KV452733.1	1652455	2-scf00041	2
AX-157935552	3	76.999	KV452733.1	1730512	2-scf00041	2
AX-157935566	3	76.999	KV452733.1	1761051	2-scf00041	2
AX-157935606	3	76.999	KV452733.1	1818632	2-scf00041	2
AX-157935660	3	76.999	KV452733.1	1874610	2-scf00041	2
AX-157723102	3	76.999	KV452733.1	1895995	2-scf00041	2
AX-157935686	3	76.999	KV452733.1	1896335	2-scf00041	2
AX-157935724	3	76.999	KV452733.1	1965658	2-scf00041	2
AX-157935735	3	76.999	KV452733.1	1992455	2-scf00041	2
AX-157935765	3	76.999	KV452733.1	2039981	2-scf00041	2
AX-157935417	3	77.108	KV452733.1	1564839	2-scf00041	2
AX-157935424	3	77.108	KV452733.1	1579224	2-scf00041	2
AX-157935442	3	77.108	KV452733.1	1594634	2-scf00041	2
AX-157935445	3	77.108	KV452733.1	1597483	2-scf00041	2
AX-157935451	3	77.108	KV452733.1	1602506	2-scf00041	2
AX-157935314	3	77.218	KV452733.1	1438225	2-scf00041	2
AX-157935341	3	77.218	KV452733.1	1472853	2-scf00041	2
AX-157758002	3	77.218	KV452733.1	1528763	2-scf00041	2
AX-157935376	3	77.218	KV452733.1	1530670	2-scf00041	2
AX-157708731	3	77.218	KV452733.1	1532769	2-scf00041	2
AX-157935392	3	77.218	KV452733.1	1543394	2-scf00041	2
AX-157875048	3	77.218	KV452733.1	1543455	2-scf00041	2
AX-157935398	3	77.218	KV452733.1	1549718	2-scf00041	2
AX-157935399	3	77.218	KV452733.1	1550397	2-scf00041	2
AX-157875050	3	77.218	KV452733.1	1553578	2-scf00041	2
AX-157935401	3	77.218	KV452733.1	1554113	2-scf00041	2
AX-157935406	3	77.218	KV452733.1	1556531	2-scf00041	2
AX-157935408	3	77.218	KV452733.1	1558696	2-scf00041	2
AX-157935411	3	77.218	KV452733.1	1559369	2-scf00041	2
AX-157875052	3	77.218	KV452733.1	1561467	2-scf00041	2
AX-157935414	3	77.218	KV452733.1	1561794	2-scf00041	2
AX-157935420	3	77.218	KV452733.1	1565471	2-scf00041	2
AX-158404712	3	77.218	KV452733.1	1576928	2-scf00041	2
AX-157935423	3	77.218	KV452733.1	1578800	2-scf00041	2
AX-157935426	3	77.218	KV452733.1	1580290	2-scf00041	2
AX-157935429	3	77.218	KV452733.1	1582610	2-scf00041	2
AX-157814331	3	77.218	KV452733.1	1589658	2-scf00041	2
AX-157875057	3	77.218	KV452733.1	1594428	2-scf00041	2
AX-157935444	3	77.218	KV452733.1	1595745	2-scf00041	2
AX-157935446	3	77.218	KV452733.1	1599937	2-scf00041	2
AX-157935448	3	77.218	KV452733.1	1600355	2-scf00041	2
AX-157814333	3	77.218	KV452733.1	1604645	2-scf00041	2
AX-157935453	3	77.218	KV452733.1	1605175	2-scf00041	2
AX-157935456	3	77.218	KV452733.1	1605607	2-scf00041	2
AX-157814334	3	77.218	KV452733.1	1605981	2-scf00041	2

AX-157875058	3	77.218	KV452733.1	1607071	2-scf00041	2
AX-157814335	3	77.218	KV452733.1	1607804	2-scf00041	2
AX-157935458	3	77.218	KV452733.1	1609419	2-scf00041	2
AX-157875059	3	77.218	KV452733.1	1609873	2-scf00041	2
AX-157814336	3	77.218	KV452733.1	1612154	2-scf00041	2
AX-157935464	3	77.218	KV452733.1	1613246	2-scf00041	2
AX-157935468	3	77.218	KV452733.1	1614250	2-scf00041	2
AX-157935469	3	77.218	KV452733.1	1615216	2-scf00041	2
AX-157935471	3	77.218	KV452733.1	1615698	2-scf00041	2
AX-157935472	3	77.218	KV452733.1	1615982	2-scf00041	2
AX-157935474	3	77.218	KV452733.1	1616516	2-scf00041	2
AX-157935475	3	77.218	KV452733.1	1618156	2-scf00041	2
AX-157935477	3	77.218	KV452733.1	1618653	2-scf00041	2
AX-157814337	3	77.218	KV452733.1	1619153	2-scf00041	2
AX-157875060	3	77.218	KV452733.1	1619604	2-scf00041	2
AX-157814338	3	77.218	KV452733.1	1620232	2-scf00041	2
AX-157875061	3	77.218	KV452733.1	1622402	2-scf00041	2
AX-157935481	3	77.218	KV452733.1	1624179	2-scf00041	2
AX-157814339	3	77.218	KV452733.1	1624221	2-scf00041	2
AX-157935482	3	77.218	KV452733.1	1625434	2-scf00041	2
AX-158405607	3	77.218	KV452733.1	1627311	2-scf00041	2
AX-157935483	3	77.218	KV452733.1	1628610	2-scf00041	2
AX-157814340	3	77.218	KV452733.1	1628889	2-scf00041	2
AX-157935484	3	77.218	KV452733.1	1631061	2-scf00041	2
AX-157875062	3	77.218	KV452733.1	1637124	2-scf00041	2
AX-157935488	3	77.218	KV452733.1	1637454	2-scf00041	2
AX-157935489	3	77.218	KV452733.1	1638466	2-scf00041	2
AX-157935495	3	77.218	KV452733.1	1653674	2-scf00041	2
AX-157935496	3	77.218	KV452733.1	1654926	2-scf00041	2
AX-157935497	3	77.218	KV452733.1	1656251	2-scf00041	2
AX-157814343	3	77.218	KV452733.1	1671567	2-scf00041	2
AX-157935523	3	77.218	KV452733.1	1699036	2-scf00041	2
AX-157814349	3	77.218	KV452733.1	1701692	2-scf00041	2
AX-157935536	3	77.218	KV452733.1	1718568	2-scf00041	2
AX-157935538	3	77.218	KV452733.1	1718808	2-scf00041	2
AX-157935545	3	77.218	KV452733.1	1727486	2-scf00041	2
AX-158379360	3	77.218	KV452733.1	1727649	2-scf00041	2
AX-157935547	3	77.218	KV452733.1	1728519	2-scf00041	2
AX-157935558	3	77.218	KV452733.1	1744184	2-scf00041	2
AX-157935559	3	77.218	KV452733.1	1745234	2-scf00041	2
AX-157935560	3	77.218	KV452733.1	1748662	2-scf00041	2
AX-157814355	3	77.218	KV452733.1	1750778	2-scf00041	2
AX-157935563	3	77.218	KV452733.1	1752404	2-scf00041	2
AX-157935565	3	77.218	KV452733.1	1760655	2-scf00041	2
AX-157883817	3	77.218	KV452733.1	1762261	2-scf00041	2
AX-157935567	3	77.218	KV452733.1	1762542	2-scf00041	2
AX-157935569	3	77.218	KV452733.1	1763687	2-scf00041	2
AX-157935571	3	77.218	KV452733.1	1769132	2-scf00041	2
AX-157935574	3	77.218	KV452733.1	1772074	2-scf00041	2
AX-157935575	3	77.218	KV452733.1	1773411	2-scf00041	2
AX-157745214	3	77.218	KV452733.1	1784162	2-scf00041	2

AX-157935589	3	77.218	KV452733.1	1799169	2-scf00041	2
AX-157935617	3	77.218	KV452733.1	1822516	2-scf00041	2
AX-157935619	3	77.218	KV452733.1	1823879	2-scf00041	2
AX-157935622	3	77.218	KV452733.1	1825812	2-scf00041	2
AX-157935628	3	77.218	KV452733.1	1827544	2-scf00041	2
AX-157814361	3	77.218	KV452733.1	1838983	2-scf00041	2
AX-157935638	3	77.218	KV452733.1	1839407	2-scf00041	2
AX-157935641	3	77.218	KV452733.1	1840416	2-scf00041	2
AX-157935645	3	77.218	KV452733.1	1850671	2-scf00041	2
AX-157935646	3	77.218	KV452733.1	1851041	2-scf00041	2
AX-157814362	3	77.218	KV452733.1	1851125	2-scf00041	2
AX-157935648	3	77.218	KV452733.1	1851832	2-scf00041	2
AX-157935655	3	77.218	KV452733.1	1865048	2-scf00041	2
AX-157864690	3	77.218	KV452733.1	1868793	2-scf00041	2
AX-157739737	3	77.218	KV452733.1	1873117	2-scf00041	2
AX-157720787	3	77.218	KV452733.1	1877173	2-scf00041	2
AX-157935666	3	77.218	KV452733.1	1877576	2-scf00041	2
AX-157935678	3	77.218	KV452733.1	1886131	2-scf00041	2
AX-157935688	3	77.218	KV452733.1	1898765	2-scf00041	2
AX-157854463	3	77.218	KV452733.1	1908783	2-scf00041	2
AX-157935700	3	77.218	KV452733.1	1910431	2-scf00041	2
AX-157935701	3	77.218	KV452733.1	1910678	2-scf00041	2
AX-157757943	3	77.218	KV452733.1	1911240	2-scf00041	2
AX-157935702	3	77.218	KV452733.1	1911656	2-scf00041	2
AX-157864703	3	77.218	KV452733.1	1920786	2-scf00041	2
AX-157935708	3	77.218	KV452733.1	1927315	2-scf00041	2
AX-157935710	3	77.218	KV452733.1	1948572	2-scf00041	2
AX-157883822	3	77.218	KV452733.1	1951095	2-scf00041	2
AX-157935719	3	77.218	KV452733.1	1959520	2-scf00041	2
AX-157814365	3	77.218	KV452733.1	1959851	2-scf00041	2
AX-157935721	3	77.218	KV452733.1	1960892	2-scf00041	2
AX-157935722	3	77.218	KV452733.1	1961631	2-scf00041	2
AX-157775546	3	77.218	KV452733.1	1969433	2-scf00041	2
AX-157935726	3	77.218	KV452733.1	1970149	2-scf00041	2
AX-157935728	3	77.218	KV452733.1	1977610	2-scf00041	2
AX-157935730	3	77.218	KV452733.1	1979553	2-scf00041	2
AX-157712213	3	77.218	KV452733.1	1987004	2-scf00041	2
AX-157935734	3	77.218	KV452733.1	1988668	2-scf00041	2
AX-157935741	3	77.218	KV452733.1	1997571	2-scf00041	2
AX-157935743	3	77.218	KV452733.1	1998009	2-scf00041	2
AX-157935755	3	77.218	KV452733.1	2023101	2-scf00041	2
AX-158405613	3	77.218	KV452733.1	2028191	2-scf00041	2
AX-157935783	3	77.218	KV452733.1	2060920	2-scf00041	2
AX-157935792	3	77.218	KV452733.1	2078242	2-scf00041	2
AX-157935863	3	77.218	KV452733.1	2206739	2-scf00041	2
AX-158415035	3	77.218	KV452733.1	2226676	2-scf00041	2
AX-157758054	3	77.218	KV452733.1	2461472	2-scf00041	2
AX-157939955	3	77.218	KV452742.1	3026440	2-scf00046	2
AX-157814366	3	77.545	KV452733.1	2006866	2-scf00041	2
AX-157935759	3	77.545	KV452733.1	2032356	2-scf00041	2
AX-157935760	3	77.545	KV452733.1	2033922	2-scf00041	2

AX-157935918	3	77.545	KV452733.1	2298792	2-scf00041	2
AX-157935761	3	77.559	KV452733.1	2035981	2-scf00041	2
AX-157935763	3	77.575	KV452733.1	2038786	2-scf00041	2
AX-157935764	3	77.593	KV452733.1	2039409	2-scf00041	2
AX-157935767	3	77.607	KV452733.1	2042687	2-scf00041	2
AX-157936257	3	77.607	KV452733.1	2637147	2-scf00041	2
AX-157814769	3	77.607	KV452742.1	4300426	2-scf00046	2
AX-157750912	3	77.619	KV452733.1	1477967	2-scf00041	2
AX-157935775	3	77.619	KV452733.1	2051447	2-scf00041	2
AX-157935886	3	77.619	KV452733.1	2247230	2-scf00041	2
AX-157935977	3	77.619	KV452733.1	2369356	2-scf00041	2
AX-157693906	3	77.619	FI892229.1	383	HORI212 Ictalu	UN
AX-157935777	3	77.628	KV452733.1	2051880	2-scf00041	2
AX-157935704	3	77.639	KV452733.1	1913914	2-scf00041	2
AX-157935779	3	77.639	KV452733.1	2052330	2-scf00041	2
AX-157935431	3	77.654	KV452733.1	1583220	2-scf00041	2
AX-157935435	3	77.654	KV452733.1	1585169	2-scf00041	2
AX-157935762	3	77.654	KV452733.1	2038406	2-scf00041	2
AX-157935769	3	77.654	KV452733.1	2046716	2-scf00041	2
AX-157935781	3	77.654	KV452733.1	2058109	2-scf00041	2
AX-157935785	3	77.654	KV452733.1	2071658	2-scf00041	2
AX-157935787	3	77.654	KV452733.1	2072202	2-scf00041	2
AX-157935788	3	77.654	KV452733.1	2074158	2-scf00041	2
AX-157935789	3	77.654	KV452733.1	2075870	2-scf00041	2
AX-157935791	3	77.654	KV452733.1	2077001	2-scf00041	2
AX-157935796	3	77.654	KV452733.1	2079769	2-scf00041	2
AX-157935846	3	77.654	KV452733.1	2159636	2-scf00041	2
AX-157935802	3	77.69	KV452733.1	2091930	2-scf00041	2
AX-157935808	3	77.714	KV452733.1	2100792	2-scf00041	2
AX-157935810	3	77.722	KV452733.1	2101325	2-scf00041	2
AX-158390010	3	77.749	KV452733.1	2129715	2-scf00041	2
AX-157935963	3	77.749	KV452733.1	2361582	2-scf00041	2
AX-157935835	3	77.764	KV452733.1	2151493	2-scf00041	2
AX-157935836	3	77.764	KV452733.1	2153235	2-scf00041	2
AX-157935853	3	77.764	KV452733.1	2181742	2-scf00041	2
AX-157935854	3	77.764	KV452733.1	2182575	2-scf00041	2
AX-157935930	3	77.764	KV452733.1	2316535	2-scf00041	2
AX-157936004	3	77.764	KV452733.1	2399038	2-scf00041	2
AX-157936026	3	77.764	KV452733.1	2426585	2-scf00041	2
AX-157936030	3	77.764	KV452733.1	2427339	2-scf00041	2
AX-158382552	3	77.764	KV452733.1	2427868	2-scf00041	2
AX-157936031	3	77.764	KV452733.1	2428167	2-scf00041	2
AX-157936033	3	77.764	KV452733.1	2428752	2-scf00041	2
AX-157751541	3	77.764	KV452733.1	2444843	2-scf00041	2
AX-158382553	3	77.764	KV452733.1	2447289	2-scf00041	2
AX-157936075	3	77.764	KV452733.1	2458212	2-scf00041	2
AX-157936088	3	77.764	KV452733.1	2470205	2-scf00041	2
AX-157936106	3	77.764	KV452733.1	2492063	2-scf00041	2
AX-157936469	3	77.764	KV452733.1	2833865	2-scf00041	2
AX-157935874	3	77.795	KV452733.1	2221199	2-scf00041	2
AX-157935921	3	77.795	KV452733.1	2300951	2-scf00041	2

AX-157935951	3	77.795	KV452733.1	2346955	2-scf00041	2
AX-157936067	3	77.795	KV452733.1	2452628	2-scf00041	2
AX-157936068	3	77.795	KV452733.1	2452874	2-scf00041	2
AX-157936128	3	77.795	KV452733.1	2514733	2-scf00041	2
AX-158382555	3	77.795	KV452733.1	2562478	2-scf00041	2
AX-157936229	3	77.795	KV452733.1	2615580	2-scf00041	2
AX-157936245	3	77.795	KV452733.1	2629837	2-scf00041	2
AX-157935883	3	77.821	KV452733.1	2240781	2-scf00041	2
AX-157935754	3	77.873	KV452733.1	2019430	2-scf00041	2
AX-157935768	3	77.873	KV452733.1	2044101	2-scf00041	2
AX-157935812	3	77.873	KV452733.1	2103288	2-scf00041	2
AX-157935830	3	77.873	KV452733.1	2147669	2-scf00041	2
AX-157935852	3	77.873	KV452733.1	2179683	2-scf00041	2
AX-157935878	3	77.873	KV452733.1	2232175	2-scf00041	2
AX-157935884	3	77.873	KV452733.1	2241938	2-scf00041	2
AX-157935889	3	77.873	KV452733.1	2254811	2-scf00041	2
AX-157935901	3	77.873	KV452733.1	2279831	2-scf00041	2
AX-157935903	3	77.873	KV452733.1	2280591	2-scf00041	2
AX-157814370	3	77.873	KV452733.1	2282208	2-scf00041	2
AX-157935905	3	77.873	KV452733.1	2283395	2-scf00041	2
AX-157935908	3	77.873	KV452733.1	2288656	2-scf00041	2
AX-157701281	3	77.873	KV452733.1	2293175	2-scf00041	2
AX-157935915	3	77.873	KV452733.1	2297104	2-scf00041	2
AX-157935926	3	77.873	KV452733.1	2312345	2-scf00041	2
AX-157744862	3	77.873	KV452733.1	2317885	2-scf00041	2
AX-157935932	3	77.873	KV452733.1	2319925	2-scf00041	2
AX-157814379	3	77.873	KV452733.1	2325759	2-scf00041	2
AX-157935942	3	77.873	KV452733.1	2335405	2-scf00041	2
AX-157935945	3	77.873	KV452733.1	2337795	2-scf00041	2
AX-157935947	3	77.873	KV452733.1	2338604	2-scf00041	2
AX-157935948	3	77.873	KV452733.1	2338850	2-scf00041	2
AX-157935953	3	77.873	KV452733.1	2351945	2-scf00041	2
AX-157935957	3	77.873	KV452733.1	2356033	2-scf00041	2
AX-157935959	3	77.873	KV452733.1	2356849	2-scf00041	2
AX-157935961	3	77.873	KV452733.1	2360384	2-scf00041	2
AX-157935980	3	77.873	KV452733.1	2370715	2-scf00041	2
AX-157935983	3	77.873	KV452733.1	2373619	2-scf00041	2
AX-157935992	3	77.873	KV452733.1	2384238	2-scf00041	2
AX-157935994	3	77.873	KV452733.1	2385413	2-scf00041	2
AX-157936000	3	77.873	KV452733.1	2394042	2-scf00041	2
AX-157936001	3	77.873	KV452733.1	2394454	2-scf00041	2
AX-157936003	3	77.873	KV452733.1	2397547	2-scf00041	2
AX-157936006	3	77.873	KV452733.1	2400133	2-scf00041	2
AX-157936008	3	77.873	KV452733.1	2400630	2-scf00041	2
AX-157936012	3	77.873	KV452733.1	2409126	2-scf00041	2
AX-157936014	3	77.873	KV452733.1	2411848	2-scf00041	2
AX-157724356	3	77.873	KV452733.1	2423830	2-scf00041	2
AX-157936044	3	77.873	KV452733.1	2433756	2-scf00041	2
AX-157936053	3	77.873	KV452733.1	2438615	2-scf00041	2
AX-157936054	3	77.873	KV452733.1	2439049	2-scf00041	2
AX-157936055	3	77.873	KV452733.1	2439432	2-scf00041	2

AX-157936058	3	77.873	KV452733.1	2440580	2-scf00041	2
AX-157936059	3	77.873	KV452733.1	2441197	2-scf00041	2
AX-157936060	3	77.873	KV452733.1	2442001	2-scf00041	2
AX-157936062	3	77.873	KV452733.1	2446145	2-scf00041	2
AX-157936063	3	77.873	KV452733.1	2446413	2-scf00041	2
AX-157936065	3	77.873	KV452733.1	2447367	2-scf00041	2
AX-157697612	3	77.873	KV452733.1	2449699	2-scf00041	2
AX-157936069	3	77.873	KV452733.1	2454825	2-scf00041	2
AX-157936072	3	77.873	KV452733.1	2456798	2-scf00041	2
AX-157936073	3	77.873	KV452733.1	2457109	2-scf00041	2
AX-157743054	3	77.873	KV452733.1	2457433	2-scf00041	2
AX-157936076	3	77.873	KV452733.1	2458455	2-scf00041	2
AX-157936078	3	77.873	KV452733.1	2458889	2-scf00041	2
AX-157936079	3	77.873	KV452733.1	2460404	2-scf00041	2
AX-157772710	3	77.873	KV452733.1	2493689	2-scf00041	2
AX-157936118	3	77.873	KV452733.1	2500494	2-scf00041	2
AX-157936119	3	77.873	KV452733.1	2502476	2-scf00041	2
AX-157936179	3	77.873	KV452733.1	2563260	2-scf00041	2
AX-157936222	3	77.873	KV452733.1	2607964	2-scf00041	2
AX-157721732	3	77.873	KV452733.1	2612919	2-scf00041	2
AX-157936265	3	77.873	KV452733.1	2647742	2-scf00041	2
AX-157936319	3	77.873	KV452733.1	2700957	2-scf00041	2
AX-157936320	3	77.873	KV452733.1	2702135	2-scf00041	2
AX-157774663	3	77.873	KV452733.1	3780441	2-scf00041	2
AX-157941099	3	77.873	KV452742.1	1934326	2-scf00046	2
AX-157939873	3	77.873	KV452742.1	2945770	2-scf00046	2
AX-157943216	3	77.873	KV452747.1	175779	2-scf00053	2
AX-157936090	3	77.982	KV452733.1	2472422	2-scf00041	2
AX-157936091	3	77.982	KV452733.1	2475809	2-scf00041	2
AX-157936096	3	77.982	KV452733.1	2477044	2-scf00041	2
AX-157757747	3	77.982	KV452733.1	2479716	2-scf00041	2
AX-157936098	3	77.982	KV452733.1	2481113	2-scf00041	2
AX-157936103	3	77.982	KV452733.1	2491268	2-scf00041	2
AX-157936104	3	77.982	KV452733.1	2491797	2-scf00041	2
AX-157936109	3	77.982	KV452733.1	2493062	2-scf00041	2
AX-157814385	3	77.982	KV452733.1	2493505	2-scf00041	2
AX-157936113	3	77.982	KV452733.1	2496513	2-scf00041	2
AX-157745704	3	77.982	KV452733.1	2497034	2-scf00041	2
AX-157936195	3	77.982	KV452733.1	2583042	2-scf00041	2
AX-157936196	3	77.982	KV452733.1	2583801	2-scf00041	2
AX-157936197	3	77.982	KV452733.1	2584427	2-scf00041	2
AX-157936262	3	77.982	KV452733.1	2642487	2-scf00041	2
AX-157936314	3	77.982	KV452733.1	2696661	2-scf00041	2
AX-157814393	3	77.982	KV452733.1	2850116	2-scf00041	2
AX-157875093	3	78.013	KV452733.1	2497073	2-scf00041	2
AX-157936126	3	78.062	KV452733.1	2512877	2-scf00041	2
AX-157699473	3	78.062	KV452733.1	2513002	2-scf00041	2
AX-157935318	3	78.091	KV452733.1	1440003	2-scf00041	2
AX-157935319	3	78.091	KV452733.1	1440489	2-scf00041	2
AX-157935323	3	78.091	KV452733.1	1450068	2-scf00041	2
AX-157935325	3	78.091	KV452733.1	1451525	2-scf00041	2

AX-157935327	3	78.091	KV452733.1	1454617	2-scf00041	2
AX-157935329	3	78.091	KV452733.1	1458515	2-scf00041	2
AX-157935407	3	78.091	KV452733.1	1557070	2-scf00041	2
AX-157935561	3	78.091	KV452733.1	1750126	2-scf00041	2
AX-157814356	3	78.091	KV452733.1	1779383	2-scf00041	2
AX-157935694	3	78.091	KV452733.1	1906394	2-scf00041	2
AX-157936116	3	78.091	KV452733.1	2499378	2-scf00041	2
AX-157936145	3	78.091	KV452733.1	2527481	2-scf00041	2
AX-157814388	3	78.091	KV452733.1	2529023	2-scf00041	2
AX-157814389	3	78.091	KV452733.1	2533710	2-scf00041	2
AX-157936175	3	78.091	KV452733.1	2561917	2-scf00041	2
AX-157936177	3	78.091	KV452733.1	2562932	2-scf00041	2
AX-157936181	3	78.091	KV452733.1	2572045	2-scf00041	2
AX-157936185	3	78.091	KV452733.1	2577857	2-scf00041	2
AX-157936192	3	78.091	KV452733.1	2581801	2-scf00041	2
AX-157936193	3	78.091	KV452733.1	2582522	2-scf00041	2
AX-157726065	3	78.091	KV452733.1	2586343	2-scf00041	2
AX-157936208	3	78.091	KV452733.1	2595221	2-scf00041	2
AX-157936211	3	78.091	KV452733.1	2597394	2-scf00041	2
AX-157936214	3	78.091	KV452733.1	2599118	2-scf00041	2
AX-157754578	3	78.091	KV452733.1	2601947	2-scf00041	2
AX-157936218	3	78.091	KV452733.1	2604357	2-scf00041	2
AX-157936219	3	78.091	KV452733.1	2605167	2-scf00041	2
AX-157936224	3	78.091	KV452733.1	2612706	2-scf00041	2
AX-157936227	3	78.091	KV452733.1	2614395	2-scf00041	2
AX-157936228	3	78.091	KV452733.1	2614550	2-scf00041	2
AX-157936232	3	78.091	KV452733.1	2618175	2-scf00041	2
AX-157936238	3	78.091	KV452733.1	2623289	2-scf00041	2
AX-157753773	3	78.091	KV452733.1	2632158	2-scf00041	2
AX-157936254	3	78.091	KV452733.1	2635952	2-scf00041	2
AX-157936255	3	78.091	KV452733.1	2636552	2-scf00041	2
AX-157936267	3	78.091	KV452733.1	2648440	2-scf00041	2
AX-157936270	3	78.091	KV452733.1	2652857	2-scf00041	2
AX-157936274	3	78.091	KV452733.1	2656448	2-scf00041	2
AX-157936281	3	78.091	KV452733.1	2667766	2-scf00041	2
AX-157936284	3	78.091	KV452733.1	2671949	2-scf00041	2
AX-157936295	3	78.091	KV452733.1	2681275	2-scf00041	2
AX-157706171	3	78.091	KV452733.1	2681316	2-scf00041	2
AX-157936300	3	78.091	KV452733.1	2684716	2-scf00041	2
AX-157936317	3	78.091	KV452733.1	2699781	2-scf00041	2
AX-157936413	3	78.091	KV452733.1	2784230	2-scf00041	2
AX-157698254	3	78.091	FI894969.1	285	HORI212 Ictalu	UN
AX-157936323	3	78.2	KV452733.1	2705265	2-scf00041	2
AX-157936336	3	78.2	KV452733.1	2712031	2-scf00041	2
AX-157936131	3	78.31	KV452733.1	2518078	2-scf00041	2
AX-157740121	3	78.31	KV452733.1	2528314	2-scf00041	2
AX-157936242	3	78.31	KV452733.1	2625762	2-scf00041	2
AX-157936324	3	78.31	KV452733.1	2705857	2-scf00041	2
AX-157936328	3	78.31	KV452733.1	2708869	2-scf00041	2
AX-157936334	3	78.31	KV452733.1	2711277	2-scf00041	2
AX-157936339	3	78.31	KV452733.1	2713974	2-scf00041	2

AX-157936340	3	78.31	KV452733.1	2714457	2-scf00041	2
AX-157936348	3	78.31	KV452733.1	2728056	2-scf00041	2
AX-157936351	3	78.31	KV452733.1	2730651	2-scf00041	2
AX-157936353	3	78.31	KV452733.1	2731461	2-scf00041	2
AX-157936355	3	78.31	KV452733.1	2733550	2-scf00041	2
AX-157936359	3	78.31	KV452733.1	2735736	2-scf00041	2
AX-157936362	3	78.31	KV452733.1	2736941	2-scf00041	2
AX-157936363	3	78.31	KV452733.1	2737550	2-scf00041	2
AX-157936366	3	78.31	KV452733.1	2737863	2-scf00041	2
AX-157936367	3	78.31	KV452733.1	2738328	2-scf00041	2
AX-157936372	3	78.31	KV452733.1	2740881	2-scf00041	2
AX-157936373	3	78.31	KV452733.1	2741723	2-scf00041	2
AX-157936374	3	78.31	KV452733.1	2743564	2-scf00041	2
AX-157936375	3	78.31	KV452733.1	2744198	2-scf00041	2
AX-157936376	3	78.31	KV452733.1	2745584	2-scf00041	2
AX-157936377	3	78.31	KV452733.1	2745742	2-scf00041	2
AX-157936390	3	78.31	KV452733.1	2766493	2-scf00041	2
AX-157936398	3	78.31	KV452733.1	2773308	2-scf00041	2
AX-157936401	3	78.31	KV452733.1	2774198	2-scf00041	2
AX-157936411	3	78.31	KV452733.1	2782976	2-scf00041	2
AX-157936415	3	78.31	KV452733.1	2785801	2-scf00041	2
AX-157724050	3	78.31	KV452733.1	2789134	2-scf00041	2
AX-157936419	3	78.31	KV452733.1	2789516	2-scf00041	2
AX-157936420	3	78.31	KV452733.1	2791149	2-scf00041	2
AX-157741887	3	78.31	KV452733.1	2793617	2-scf00041	2
AX-157936436	3	78.31	KV452733.1	2803217	2-scf00041	2
AX-157936441	3	78.31	KV452733.1	2808936	2-scf00041	2
AX-157936448	3	78.31	KV452733.1	2818268	2-scf00041	2
AX-157936450	3	78.31	KV452733.1	2821471	2-scf00041	2
AX-157936457	3	78.31	KV452733.1	2825796	2-scf00041	2
AX-157936461	3	78.31	KV452733.1	2828029	2-scf00041	2
AX-157936475	3	78.31	KV452733.1	2842515	2-scf00041	2
AX-157814392	3	78.31	KV452733.1	2842566	2-scf00041	2
AX-157936477	3	78.31	KV452733.1	2845724	2-scf00041	2
AX-157726733	3	78.31	KV452733.1	2848322	2-scf00041	2
AX-157701923	3	78.31	KV452733.1	2849346	2-scf00041	2
AX-157936481	3	78.31	KV452733.1	2851513	2-scf00041	2
AX-157936483	3	78.31	KV452733.1	2852044	2-scf00041	2
AX-157814397	3	78.31	KV452733.1	2858607	2-scf00041	2
AX-157743640	3	78.31	KV452733.1	2861693	2-scf00041	2
AX-157814398	3	78.31	KV452733.1	2861778	2-scf00041	2
AX-157936493	3	78.31	KV452733.1	2862210	2-scf00041	2
AX-157854469	3	78.31	KV452733.1	2865342	2-scf00041	2
AX-157936544	3	78.31	KV452733.1	2921321	2-scf00041	2
AX-157814410	3	78.31	KV452733.1	2971008	2-scf00041	2
AX-157744419	3	78.31	KV452733.1	2972273	2-scf00041	2
AX-157936594	3	78.31	KV452733.1	2974943	2-scf00041	2
AX-157936740	3	78.31	KV452733.1	3137206	2-scf00041	2
AX-157936747	3	78.31	KV452733.1	3140611	2-scf00041	2
AX-157936754	3	78.31	KV452733.1	3145705	2-scf00041	2
AX-157936892	3	78.31	KV452733.1	3299001	2-scf00041	2

AX-157936902	3	78.31	KV452733.1	3304607	2-scf00041	2
AX-157936993	3	78.31	KV452733.1	3360975	2-scf00041	2
AX-157938549	3	78.31	KV452742.1	136343	2-scf00046	2
AX-157938655	3	78.31	KV452742.1	264814	2-scf00046	2
AX-157941030	3	78.31	KV452742.1	2069936	2-scf00046	2
AX-157936499	3	78.419	KV452733.1	2873753	2-scf00041	2
AX-158379364	3	78.419	KV452733.1	2874725	2-scf00041	2
AX-157936502	3	78.419	KV452733.1	2875452	2-scf00041	2
AX-157934819	3	78.516	KV452733.1	944144	2-scf00041	2
AX-157814312	3	78.516	KV452733.1	1088637	2-scf00041	2
AX-157936141	3	78.516	KV452733.1	2524298	2-scf00041	2
AX-157814401	3	78.516	KV452733.1	2880990	2-scf00041	2
AX-157930933	3	78.637	KV452730.1	10497754	2-scf00040	2
AX-158379347	3	78.637	KV452730.1	10724050	2-scf00040	2
AX-157933737	3	78.637	KV452730.1	14351813	2-scf00040	2
AX-157935950	3	78.637	KV452733.1	2340358	2-scf00041	2
AX-157936446	3	78.637	KV452733.1	2817726	2-scf00041	2
AX-158405624	3	78.637	KV452733.1	2885114	2-scf00041	2
AX-157936520	3	78.637	KV452733.1	2895521	2-scf00041	2
AX-157936521	3	78.637	KV452733.1	2898094	2-scf00041	2
AX-157814403	3	78.637	KV452733.1	2902109	2-scf00041	2
AX-158404719	3	78.637	KV452733.1	2905561	2-scf00041	2
AX-157875100	3	78.637	KV452733.1	2906918	2-scf00041	2
AX-157936530	3	78.637	KV452733.1	2911415	2-scf00041	2
AX-157936536	3	78.637	KV452733.1	2914552	2-scf00041	2
AX-157936537	3	78.637	KV452733.1	2914881	2-scf00041	2
AX-157746929	3	78.637	KV452733.1	2916384	2-scf00041	2
AX-157936539	3	78.637	KV452733.1	2916635	2-scf00041	2
AX-157936541	3	78.637	KV452733.1	2919163	2-scf00041	2
AX-157936542	3	78.637	KV452733.1	2920526	2-scf00041	2
AX-157936547	3	78.637	KV452733.1	2923852	2-scf00041	2
AX-157936549	3	78.637	KV452733.1	2925076	2-scf00041	2
AX-157747381	3	78.637	KV452733.1	2940562	2-scf00041	2
AX-157936565	3	78.637	KV452733.1	2944046	2-scf00041	2
AX-157936567	3	78.637	KV452733.1	2944139	2-scf00041	2
AX-157936569	3	78.637	KV452733.1	2944879	2-scf00041	2
AX-157936572	3	78.637	KV452733.1	2946737	2-scf00041	2
AX-157936573	3	78.637	KV452733.1	2947180	2-scf00041	2
AX-157936574	3	78.637	KV452733.1	2947832	2-scf00041	2
AX-157936586	3	78.637	KV452733.1	2963141	2-scf00041	2
AX-157814406	3	78.637	KV452733.1	2964868	2-scf00041	2
AX-157814407	3	78.637	KV452733.1	2965218	2-scf00041	2
AX-157936588	3	78.637	KV452733.1	2966457	2-scf00041	2
AX-157936591	3	78.637	KV452733.1	2970625	2-scf00041	2
AX-157814411	3	78.637	KV452733.1	2972327	2-scf00041	2
AX-157936600	3	78.637	KV452733.1	2977230	2-scf00041	2
AX-157864718	3	78.637	KV452733.1	3294167	2-scf00041	2
AX-157936891	3	78.637	KV452733.1	3294867	2-scf00041	2
AX-157936894	3	78.637	KV452733.1	3300348	2-scf00041	2
AX-157936895	3	78.637	KV452733.1	3300769	2-scf00041	2
AX-157936982	3	78.637	KV452733.1	3350633	2-scf00041	2

AX-157937050	3	78.637	KV452733.1	3413135	2-scf00041	2
AX-157937429	3	78.637	KV452733.1	3818299	2-scf00041	2
AX-157937487	3	78.637	KV452732.1	92670	2-scf00042	2
AX-157938377	3	78.637	KV452735.1	117796	2-scf00044	2
AX-157941040	3	78.637	KV452742.1	2043368	2-scf00046	2
AX-157940475	3	78.637	KV452742.1	3638069	2-scf00046	2
AX-157940548	3	78.637	KV452742.1	3756567	2-scf00046	2
AX-157941001	3	78.637	KV452742.1	4535366	2-scf00046	2
AX-157722644	3	78.637	KV452745.1	585317	2-scf00051	2
AX-157943131	3	78.637	KV452747.1	75538	2-scf00053	2
AX-157943800	3	78.637	KV452747.1	805445	2-scf00053	2
AX-157706757	3	78.637	3ML01032860	550	scf08491_1	UN
AX-157814413	3	78.701	KV452733.1	2991495	2-scf00041	2
AX-157936888	3	78.701	KV452733.1	3290746	2-scf00041	2
AX-157938286	3	78.701	KV452735.1	44156	2-scf00044	2
AX-157938288	3	78.701	KV452735.1	44942	2-scf00044	2
AX-157742985	3	78.746	KV452733.1	961590	2-scf00041	2
AX-157936335	3	78.746	KV452733.1	2711871	2-scf00041	2
AX-157936391	3	78.746	KV452733.1	2767516	2-scf00041	2
AX-157936472	3	78.746	KV452733.1	2834844	2-scf00041	2
AX-157936506	3	78.746	KV452733.1	2883137	2-scf00041	2
AX-157936508	3	78.746	KV452733.1	2886036	2-scf00041	2
AX-157936511	3	78.746	KV452733.1	2888336	2-scf00041	2
AX-157936515	3	78.746	KV452733.1	2890441	2-scf00041	2
AX-157936529	3	78.746	KV452733.1	2910420	2-scf00041	2
AX-157936535	3	78.746	KV452733.1	2912933	2-scf00041	2
AX-157936550	3	78.746	KV452733.1	2926140	2-scf00041	2
AX-157936560	3	78.746	KV452733.1	2936765	2-scf00041	2
AX-157936563	3	78.746	KV452733.1	2943499	2-scf00041	2
AX-157936570	3	78.746	KV452733.1	2945905	2-scf00041	2
AX-157936578	3	78.746	KV452733.1	2949985	2-scf00041	2
AX-157869442	3	78.746	KV452733.1	2963403	2-scf00041	2
AX-157814409	3	78.746	KV452733.1	2969455	2-scf00041	2
AX-157869443	3	78.746	KV452733.1	2972042	2-scf00041	2
AX-157936598	3	78.746	KV452733.1	2976107	2-scf00041	2
AX-157936601	3	78.746	KV452733.1	2977996	2-scf00041	2
AX-157869444	3	78.746	KV452733.1	2978453	2-scf00041	2
AX-157869445	3	78.746	KV452733.1	2988656	2-scf00041	2
AX-158404723	3	78.746	KV452733.1	2989670	2-scf00041	2
AX-157936605	3	78.746	KV452733.1	2995732	2-scf00041	2
AX-157814415	3	78.746	KV452733.1	2997347	2-scf00041	2
AX-157936610	3	78.746	KV452733.1	3000693	2-scf00041	2
AX-157814416	3	78.746	KV452733.1	3007853	2-scf00041	2
AX-157695368	3	78.746	KV452733.1	3009036	2-scf00041	2
AX-157936618	3	78.746	KV452733.1	3011443	2-scf00041	2
AX-157814417	3	78.746	KV452733.1	3014432	2-scf00041	2
AX-157814418	3	78.746	KV452733.1	3014705	2-scf00041	2
AX-157936623	3	78.746	KV452733.1	3016105	2-scf00041	2
AX-158405631	3	78.746	KV452733.1	3017431	2-scf00041	2
AX-157936640	3	78.746	KV452733.1	3030026	2-scf00041	2
AX-157936641	3	78.746	KV452733.1	3030912	2-scf00041	2

AX-157936644	3	78.746	KV452733.1	3035906	2-scf00041	2
AX-157936652	3	78.746	KV452733.1	3041166	2-scf00041	2
AX-157936654	3	78.746	KV452733.1	3042178	2-scf00041	2
AX-157936657	3	78.746	KV452733.1	3043356	2-scf00041	2
AX-157936660	3	78.746	KV452733.1	3050811	2-scf00041	2
AX-157936666	3	78.746	KV452733.1	3055305	2-scf00041	2
AX-157936673	3	78.746	KV452733.1	3061395	2-scf00041	2
AX-157936682	3	78.746	KV452733.1	3068020	2-scf00041	2
AX-157854470	3	78.746	KV452733.1	3069273	2-scf00041	2
AX-157936685	3	78.746	KV452733.1	3070439	2-scf00041	2
AX-157936686	3	78.746	KV452733.1	3070904	2-scf00041	2
AX-157936688	3	78.746	KV452733.1	3073377	2-scf00041	2
AX-157936695	3	78.746	KV452733.1	3081182	2-scf00041	2
AX-157740528	3	78.746	KV452733.1	3084367	2-scf00041	2
AX-157709400	3	78.746	KV452733.1	3085735	2-scf00041	2
AX-157936698	3	78.746	KV452733.1	3086719	2-scf00041	2
AX-158404725	3	78.746	KV452733.1	3095058	2-scf00041	2
AX-157936709	3	78.746	KV452733.1	3100288	2-scf00041	2
AX-157936711	3	78.746	KV452733.1	3103226	2-scf00041	2
AX-157936713	3	78.746	KV452733.1	3103880	2-scf00041	2
AX-157936714	3	78.746	KV452733.1	3106231	2-scf00041	2
AX-157854472	3	78.746	KV452733.1	3106792	2-scf00041	2
AX-157936716	3	78.746	KV452733.1	3106949	2-scf00041	2
AX-158390019	3	78.746	KV452733.1	3111273	2-scf00041	2
AX-157936722	3	78.746	KV452733.1	3120704	2-scf00041	2
AX-157936723	3	78.746	KV452733.1	3124567	2-scf00041	2
AX-158382562	3	78.746	KV452733.1	3129719	2-scf00041	2
AX-157936731	3	78.746	KV452733.1	3132601	2-scf00041	2
AX-157936733	3	78.746	KV452733.1	3133684	2-scf00041	2
AX-157936734	3	78.746	KV452733.1	3134907	2-scf00041	2
AX-157936735	3	78.746	KV452733.1	3135739	2-scf00041	2
AX-157936739	3	78.746	KV452733.1	3136154	2-scf00041	2
AX-157936742	3	78.746	KV452733.1	3137975	2-scf00041	2
AX-157936744	3	78.746	KV452733.1	3138497	2-scf00041	2
AX-157814420	3	78.746	KV452733.1	3139805	2-scf00041	2
AX-157936749	3	78.746	KV452733.1	3141454	2-scf00041	2
AX-157936756	3	78.746	KV452733.1	3146642	2-scf00041	2
AX-157936771	3	78.746	KV452733.1	3171170	2-scf00041	2
AX-157936774	3	78.746	KV452733.1	3172125	2-scf00041	2
AX-157883837	3	78.746	KV452733.1	3172913	2-scf00041	2
AX-157936778	3	78.746	KV452733.1	3174612	2-scf00041	2
AX-157854476	3	78.746	KV452733.1	3174984	2-scf00041	2
AX-158379365	3	78.746	KV452733.1	3180815	2-scf00041	2
AX-157936783	3	78.746	KV452733.1	3182453	2-scf00041	2
AX-157757910	3	78.746	KV452733.1	3183076	2-scf00041	2
AX-158405642	3	78.746	KV452733.1	3183552	2-scf00041	2
AX-157936784	3	78.746	KV452733.1	3183668	2-scf00041	2
AX-158405643	3	78.746	KV452733.1	3185617	2-scf00041	2
AX-157854478	3	78.746	KV452733.1	3187607	2-scf00041	2
AX-158421976	3	78.746	KV452733.1	3189998	2-scf00041	2
AX-157936788	3	78.746	KV452733.1	3193606	2-scf00041	2

AX-157936790	3	78.746	KV452733.1	3195930	2-scf00041	2
AX-157854480	3	78.746	KV452733.1	3197328	2-scf00041	2
AX-157936793	3	78.746	KV452733.1	3201719	2-scf00041	2
AX-157746024	3	78.746	KV452733.1	3203073	2-scf00041	2
AX-157854481	3	78.746	KV452733.1	3203388	2-scf00041	2
AX-157936796	3	78.746	KV452733.1	3203677	2-scf00041	2
AX-157936798	3	78.746	KV452733.1	3203989	2-scf00041	2
AX-157936799	3	78.746	KV452733.1	3204950	2-scf00041	2
AX-157854482	3	78.746	KV452733.1	3205129	2-scf00041	2
AX-157753366	3	78.746	KV452733.1	3206928	2-scf00041	2
AX-157936805	3	78.746	KV452733.1	3208929	2-scf00041	2
AX-157936807	3	78.746	KV452733.1	3215194	2-scf00041	2
AX-157936809	3	78.746	KV452733.1	3216152	2-scf00041	2
AX-157936810	3	78.746	KV452733.1	3216935	2-scf00041	2
AX-157936812	3	78.746	KV452733.1	3217768	2-scf00041	2
AX-157854483	3	78.746	KV452733.1	3219249	2-scf00041	2
AX-157936823	3	78.746	KV452733.1	3226086	2-scf00041	2
AX-157936824	3	78.746	KV452733.1	3226722	2-scf00041	2
AX-157936826	3	78.746	KV452733.1	3228762	2-scf00041	2
AX-157936827	3	78.746	KV452733.1	3229353	2-scf00041	2
AX-157936829	3	78.746	KV452733.1	3230409	2-scf00041	2
AX-157936831	3	78.746	KV452733.1	3231182	2-scf00041	2
AX-157936832	3	78.746	KV452733.1	3232687	2-scf00041	2
AX-157854484	3	78.746	KV452733.1	3233471	2-scf00041	2
AX-157854485	3	78.746	KV452733.1	3235601	2-scf00041	2
AX-157936837	3	78.746	KV452733.1	3235737	2-scf00041	2
AX-157936838	3	78.746	KV452733.1	3240050	2-scf00041	2
AX-157854486	3	78.746	KV452733.1	3241673	2-scf00041	2
AX-157936839	3	78.746	KV452733.1	3241734	2-scf00041	2
AX-158382565	3	78.746	KV452733.1	3242782	2-scf00041	2
AX-157936841	3	78.746	KV452733.1	3243964	2-scf00041	2
AX-157936842	3	78.746	KV452733.1	3244986	2-scf00041	2
AX-157936844	3	78.746	KV452733.1	3245754	2-scf00041	2
AX-157936847	3	78.746	KV452733.1	3247748	2-scf00041	2
AX-157936850	3	78.746	KV452733.1	3248375	2-scf00041	2
AX-157936852	3	78.746	KV452733.1	3251123	2-scf00041	2
AX-157936854	3	78.746	KV452733.1	3256272	2-scf00041	2
AX-157936857	3	78.746	KV452733.1	3264596	2-scf00041	2
AX-157936860	3	78.746	KV452733.1	3266872	2-scf00041	2
AX-157936862	3	78.746	KV452733.1	3267416	2-scf00041	2
AX-157708012	3	78.746	KV452733.1	3268410	2-scf00041	2
AX-157936867	3	78.746	KV452733.1	3271951	2-scf00041	2
AX-157936873	3	78.746	KV452733.1	3276903	2-scf00041	2
AX-157936875	3	78.746	KV452733.1	3277550	2-scf00041	2
AX-157936876	3	78.746	KV452733.1	3278201	2-scf00041	2
AX-157936878	3	78.746	KV452733.1	3278845	2-scf00041	2
AX-157936880	3	78.746	KV452733.1	3282007	2-scf00041	2
AX-157936882	3	78.746	KV452733.1	3282227	2-scf00041	2
AX-157936883	3	78.746	KV452733.1	3283956	2-scf00041	2
AX-157936885	3	78.746	KV452733.1	3284278	2-scf00041	2
AX-157936886	3	78.746	KV452733.1	3287509	2-scf00041	2

AX-157936887	3	78.746	KV452733.1	3289294	2-scf00041	2
AX-157936893	3	78.746	KV452733.1	3299753	2-scf00041	2
AX-157936897	3	78.746	KV452733.1	3302012	2-scf00041	2
AX-157936898	3	78.746	KV452733.1	3303141	2-scf00041	2
AX-157936904	3	78.746	KV452733.1	3306412	2-scf00041	2
AX-157936923	3	78.746	KV452733.1	3316521	2-scf00041	2
AX-157936931	3	78.746	KV452733.1	3321391	2-scf00041	2
AX-157936936	3	78.746	KV452733.1	3327515	2-scf00041	2
AX-157936937	3	78.746	KV452733.1	3328904	2-scf00041	2
AX-157936940	3	78.746	KV452733.1	3330239	2-scf00041	2
AX-157936946	3	78.746	KV452733.1	3333763	2-scf00041	2
AX-157936948	3	78.746	KV452733.1	3334913	2-scf00041	2
AX-157936949	3	78.746	KV452733.1	3335432	2-scf00041	2
AX-157936951	3	78.746	KV452733.1	3336648	2-scf00041	2
AX-157936955	3	78.746	KV452733.1	3337867	2-scf00041	2
AX-157936958	3	78.746	KV452733.1	3339721	2-scf00041	2
AX-157936960	3	78.746	KV452733.1	3340483	2-scf00041	2
AX-157936961	3	78.746	KV452733.1	3340845	2-scf00041	2
AX-157936965	3	78.746	KV452733.1	3341772	2-scf00041	2
AX-157775069	3	78.746	KV452733.1	3341981	2-scf00041	2
AX-157936967	3	78.746	KV452733.1	3342517	2-scf00041	2
AX-157936972	3	78.746	KV452733.1	3344168	2-scf00041	2
AX-157936974	3	78.746	KV452733.1	3344724	2-scf00041	2
AX-157936977	3	78.746	KV452733.1	3347063	2-scf00041	2
AX-157936979	3	78.746	KV452733.1	3348298	2-scf00041	2
AX-157936981	3	78.746	KV452733.1	3349610	2-scf00041	2
AX-157722090	3	78.746	KV452733.1	3364086	2-scf00041	2
AX-157937000	3	78.746	KV452733.1	3366466	2-scf00041	2
AX-157937002	3	78.746	KV452733.1	3367630	2-scf00041	2
AX-157937043	3	78.746	KV452733.1	3403931	2-scf00041	2
AX-157937044	3	78.746	KV452733.1	3404988	2-scf00041	2
AX-157937049	3	78.746	KV452733.1	3412485	2-scf00041	2
AX-157937056	3	78.746	KV452733.1	3415347	2-scf00041	2
AX-157937063	3	78.746	KV452733.1	3419955	2-scf00041	2
AX-157937068	3	78.746	KV452733.1	3422517	2-scf00041	2
AX-157883841	3	78.746	KV452733.1	3426706	2-scf00041	2
AX-157937099	3	78.746	KV452733.1	3455841	2-scf00041	2
AX-157854492	3	78.746	KV452733.1	3456602	2-scf00041	2
AX-157937105	3	78.746	KV452733.1	3461339	2-scf00041	2
AX-157938431	3	78.746	KV452735.1	214753	2-scf00044	2
AX-157939020	3	78.746	KV452742.1	672912	2-scf00046	2
AX-157943298	3	78.746	KV452747.1	261140	2-scf00053	2
AX-157815022	3	78.746	KV452747.1	1387989	2-scf00053	2
AX-157944438	3	78.746	KV452747.1	1407070	2-scf00053	2
AX-157689961	3	78.746		168	scf16407	UN
AX-157935749	3	78.856	KV452733.1	2009093	2-scf00041	2
AX-157936546	3	78.856	KV452733.1	2922053	2-scf00041	2
AX-157936579	3	78.856	KV452733.1	2950761	2-scf00041	2
AX-157936987	3	78.856	KV452733.1	3357500	2-scf00041	2
AX-157936994	3	78.856	KV452733.1	3362171	2-scf00041	2
AX-157937005	3	78.856	KV452733.1	3369292	2-scf00041	2

AX-157937006	3	78.856	KV452733.1	3370006	2-scf00041	2
AX-157937007	3	78.856	KV452733.1	3371469	2-scf00041	2
AX-157937008	3	78.856	KV452733.1	3372520	2-scf00041	2
AX-157715859	3	78.856	KV452733.1	3372871	2-scf00041	2
AX-158415044	3	78.856	KV452733.1	3375114	2-scf00041	2
AX-157937009	3	78.856	KV452733.1	3375175	2-scf00041	2
AX-157854489	3	78.856	KV452733.1	3375759	2-scf00041	2
AX-157937010	3	78.856	KV452733.1	3376199	2-scf00041	2
AX-157937017	3	78.856	KV452733.1	3384307	2-scf00041	2
AX-157937020	3	78.856	KV452733.1	3386214	2-scf00041	2
AX-157937022	3	78.856	KV452733.1	3386977	2-scf00041	2
AX-157755110	3	78.856	KV452733.1	3393443	2-scf00041	2
AX-157937037	3	78.856	KV452733.1	3398468	2-scf00041	2
AX-157741602	3	78.856	KV452733.1	3399332	2-scf00041	2
AX-157937040	3	78.856	KV452733.1	3400431	2-scf00041	2
AX-157937041	3	78.856	KV452733.1	3400849	2-scf00041	2
AX-157937045	3	78.856	KV452733.1	3408083	2-scf00041	2
AX-157937048	3	78.856	KV452733.1	3409977	2-scf00041	2
AX-157937053	3	78.856	KV452733.1	3414309	2-scf00041	2
AX-157937055	3	78.856	KV452733.1	3414983	2-scf00041	2
AX-157937062	3	78.856	KV452733.1	3419274	2-scf00041	2
AX-157937067	3	78.856	KV452733.1	3421788	2-scf00041	2
AX-157937070	3	78.856	KV452733.1	3423397	2-scf00041	2
AX-157937071	3	78.856	KV452733.1	3424645	2-scf00041	2
AX-157937073	3	78.856	KV452733.1	3426138	2-scf00041	2
AX-158405648	3	78.856	KV452733.1	3427152	2-scf00041	2
AX-158405649	3	78.856	KV452733.1	3427258	2-scf00041	2
AX-157757232	3	78.856	KV452733.1	3438282	2-scf00041	2
AX-158379366	3	78.856	KV452733.1	3471234	2-scf00041	2
AX-157937119	3	78.856	KV452733.1	3473787	2-scf00041	2
AX-157937157	3	78.856	KV452733.1	3505665	2-scf00041	2
AX-157937160	3	78.856	KV452733.1	3506809	2-scf00041	2
AX-157854494	3	78.856	KV452733.1	3508027	2-scf00041	2
AX-157937162	3	78.856	KV452733.1	3509385	2-scf00041	2
AX-157937165	3	78.856	KV452733.1	3511411	2-scf00041	2
AX-157937168	3	78.856	KV452733.1	3513039	2-scf00041	2
AX-157937169	3	78.856	KV452733.1	3514398	2-scf00041	2
AX-157937220	3	78.856	KV452733.1	3563720	2-scf00041	2
AX-157937254	3	78.856	KV452733.1	3602978	2-scf00041	2
AX-157937263	3	78.856	KV452733.1	3614892	2-scf00041	2
AX-157937264	3	78.856	KV452733.1	3615949	2-scf00041	2
AX-157937272	3	78.856	KV452733.1	3630954	2-scf00041	2
AX-157814454	3	78.856	KV452733.1	3639482	2-scf00041	2
AX-157937284	3	78.856	KV452733.1	3642314	2-scf00041	2
AX-157937295	3	78.856	KV452733.1	3649563	2-scf00041	2
AX-157937304	3	78.856	KV452733.1	3661282	2-scf00041	2
AX-157937308	3	78.856	KV452733.1	3666491	2-scf00041	2
AX-157938425	3	78.856	KV452735.1	210939	2-scf00044	2
AX-157682512	3	78.856	KV452734.1	8002	2-scf00045	2
AX-157938693	3	78.856	KV452742.1	323554	2-scf00046	2
AX-157938904	3	78.856	KV452742.1	553003	2-scf00046	2

AX-157814725	3	78.856	KV452742.1	3460301	2-scf00046	2
AX-157934823	3	78.922	KV452733.1	946921	2-scf00041	2
AX-157937074	3	78.922	KV452733.1	3427534	2-scf00041	2
AX-157689884	3	78.922		111	scf10483	UN
AX-157937091	3	78.965	KV452733.1	3442390	2-scf00041	2
AX-157937095	3	78.965	KV452733.1	3448224	2-scf00041	2
AX-157814424	3	78.965	KV452733.1	3450446	2-scf00041	2
AX-157937098	3	78.965	KV452733.1	3451299	2-scf00041	2
AX-157743256	3	78.965	KV452733.1	3452887	2-scf00041	2
AX-157814426	3	78.965	KV452733.1	3523930	2-scf00041	2
AX-157814428	3	78.965	KV452733.1	3525055	2-scf00041	2
AX-157814430	3	78.965	KV452733.1	3528168	2-scf00041	2
AX-157937226	3	78.965	KV452733.1	3570490	2-scf00041	2
AX-157937245	3	78.965	KV452733.1	3594213	2-scf00041	2
AX-157937280	3	78.965	KV452733.1	3638863	2-scf00041	2
AX-157937283	3	78.965	KV452733.1	3641817	2-scf00041	2
AX-157937323	3	78.965	KV452733.1	3695439	2-scf00041	2
AX-157937336	3	78.965	KV452733.1	3708248	2-scf00041	2
AX-157937395	3	78.965	KV452733.1	3779719	2-scf00041	2
AX-157780263	3	78.965	KV452735.1	94484	2-scf00044	2
AX-157938357	3	78.965	KV452735.1	106661	2-scf00044	2
AX-157938359	3	78.965	KV452735.1	107542	2-scf00044	2
AX-157682507	3	78.965	KV452734.1	5292	2-scf00045	2
AX-157682515	3	78.965	KV452734.1	12678	2-scf00045	2
AX-157814562	3	78.965	KV452742.1	121403	2-scf00046	2
AX-157938737	3	78.965	KV452742.1	398772	2-scf00046	2
AX-157814603	3	78.965	KV452742.1	458419	2-scf00046	2
AX-157938966	3	78.965	KV452742.1	614370	2-scf00046	2
AX-157939175	3	78.965	KV452742.1	1068021	2-scf00046	2
AX-157939515	3	78.965	KV452742.1	1486668	2-scf00046	2
AX-157939545	3	78.965	KV452742.1	1515446	2-scf00046	2
AX-157814647	3	78.965	KV452742.1	1548603	2-scf00046	2
AX-157939778	3	78.965	KV452742.1	2786751	2-scf00046	2
AX-157854528	3	78.965	KV452742.1	2794700	2-scf00046	2
AX-158405744	3	78.965	KV452742.1	2878788	2-scf00046	2
AX-157814667	3	78.965	KV452742.1	2892449	2-scf00046	2
AX-157939864	3	78.965	KV452742.1	2933951	2-scf00046	2
AX-158390044	3	78.965	KV452742.1	2941985	2-scf00046	2
AX-157939886	3	78.965	KV452742.1	2967705	2-scf00046	2
AX-157939972	3	78.965	KV452742.1	3043077	2-scf00046	2
AX-157940195	3	78.965	KV452742.1	3291067	2-scf00046	2
AX-157682526	3	78.965	KV452737.1	12511	2-scf00047	2
AX-157682538	3	78.965	KV452737.1	28426	2-scf00047	2
AX-157945422	3	78.965	KV452746.1	272511	2-scf00054	2
AX-158420600	3	78.965	KV452740.1	140166	2-scf00586	2
AX-157685757	3	78.965	KV452764.1	4804	2-scf00639	2
AX-157685760	3	78.965	KV452764.1	9835	2-scf00639	2
AX-157705050	3	78.965	3ML01028415	492	scf04022_1	UN
AX-157937115	3	79.074	KV452733.1	3469016	2-scf00041	2
AX-157937127	3	79.074	KV452733.1	3481904	2-scf00041	2
AX-157937133	3	79.074	KV452733.1	3486770	2-scf00041	2

AX-157937138	3	79.074	KV452733.1	3489053	2-scf00041	2
AX-157755732	3	79.074	KV452733.1	3489692	2-scf00041	2
AX-157937141	3	79.074	KV452733.1	3493260	2-scf00041	2
AX-157937144	3	79.074	KV452733.1	3494252	2-scf00041	2
AX-157937149	3	79.074	KV452733.1	3501077	2-scf00041	2
AX-157937156	3	79.074	KV452733.1	3505218	2-scf00041	2
AX-157937219	3	79.074	KV452733.1	3562531	2-scf00041	2
AX-157937351	3	79.074	KV452733.1	3723899	2-scf00041	2
AX-157937353	3	79.074	KV452733.1	3724387	2-scf00041	2
AX-157937389	3	79.074	KV452733.1	3770685	2-scf00041	2
AX-157937396	3	79.074	KV452733.1	3782072	2-scf00041	2
AX-157937398	3	79.074	KV452733.1	3782896	2-scf00041	2
AX-157937403	3	79.074	KV452733.1	3786250	2-scf00041	2
AX-157937404	3	79.074	KV452733.1	3786709	2-scf00041	2
AX-157814530	3	79.074	KV452735.1	29671	2-scf00044	2
AX-157938393	3	79.074	KV452735.1	142977	2-scf00044	2
AX-157938394	3	79.074	KV452735.1	143667	2-scf00044	2
AX-157938396	3	79.074	KV452735.1	145459	2-scf00044	2
AX-157938397	3	79.074	KV452735.1	146084	2-scf00044	2
AX-157814600	3	79.074	KV452742.1	388875	2-scf00046	2
AX-157938729	3	79.074	KV452742.1	389048	2-scf00046	2
AX-157814601	3	79.074	KV452742.1	389217	2-scf00046	2
AX-158376457	3	79.074	KV452740.1	129075	2-scf00586	2
AX-158376459	3	79.074	KV452740.1	129920	2-scf00586	2
AX-158376466	3	79.074	KV452740.1	138478	2-scf00586	2
AX-157749082	3	79.074	KV452740.1	140967	2-scf00586	2
AX-158376509	3	79.074	KV452740.1	245610	2-scf00586	2
AX-158376510	3	79.074	KV452740.1	247552	2-scf00586	2
AX-157853324	3	79.074	KV452740.1	254517	2-scf00586	2
AX-158405651	3	79.292	KV452733.1	3520236	2-scf00041	2
AX-157937400	3	79.292	KV452733.1	3785001	2-scf00041	2
AX-157938615	3	79.292	KV452742.1	206533	2-scf00046	2
AX-157938644	3	79.292	KV452742.1	242414	2-scf00046	2
AX-157869452	3	79.292	KV452742.1	243414	2-scf00046	2
AX-157814582	3	79.292	KV452742.1	271350	2-scf00046	2
AX-158405722	3	79.292	KV452742.1	491959	2-scf00046	2
AX-157937179	3	79.293	KV452733.1	3521024	2-scf00041	2
AX-157753119	3	79.293	KV452742.1	320495	2-scf00046	2
AX-157937185	3	79.402	KV452733.1	3530662	2-scf00041	2
AX-157716250	3	79.402	KV452733.1	3531742	2-scf00041	2
AX-157937190	3	79.402	KV452733.1	3533218	2-scf00041	2
AX-157931394	3	79.511	KV452730.1	10982369	2-scf00040	2
AX-157932543	3	79.511	KV452730.1	12359825	2-scf00040	2
AX-157935028	3	79.511	KV452733.1	1143589	2-scf00041	2
AX-157935289	3	79.511	KV452733.1	1421544	2-scf00041	2
AX-157935298	3	79.511	KV452733.1	1430400	2-scf00041	2
AX-157935693	3	79.511	KV452733.1	1901815	2-scf00041	2
AX-157935998	3	79.511	KV452733.1	2390109	2-scf00041	2
AX-157814412	3	79.511	KV452733.1	2984594	2-scf00041	2
AX-157936624	3	79.511	KV452733.1	3018338	2-scf00041	2
AX-157936772	3	79.511	KV452733.1	3171971	2-scf00041	2

AX-157936820	3	79.511	KV452733.1	3224712	2-scf00041	2
AX-157749766	3	79.511	KV452733.1	3242568	2-scf00041	2
AX-157936905	3	79.511	KV452733.1	3307194	2-scf00041	2
AX-157937026	3	79.511	KV452733.1	3389776	2-scf00041	2
AX-157937114	3	79.511	KV452733.1	3466746	2-scf00041	2
AX-157814433	3	79.511	KV452733.1	3537188	2-scf00041	2
AX-157747104	3	79.511	KV452733.1	3537309	2-scf00041	2
AX-157714052	3	79.511	KV452733.1	3540330	2-scf00041	2
AX-157814435	3	79.511	KV452733.1	3541488	2-scf00041	2
AX-157937204	3	79.511	KV452733.1	3548673	2-scf00041	2
AX-157937212	3	79.511	KV452733.1	3553684	2-scf00041	2
AX-157937216	3	79.511	KV452733.1	3559795	2-scf00041	2
AX-157714938	3	79.511	KV452733.1	3563201	2-scf00041	2
AX-157937223	3	79.511	KV452733.1	3565025	2-scf00041	2
AX-157937224	3	79.511	KV452733.1	3569930	2-scf00041	2
AX-157937233	3	79.511	KV452733.1	3582822	2-scf00041	2
AX-157937252	3	79.511	KV452733.1	3602369	2-scf00041	2
AX-157937270	3	79.511	KV452733.1	3625775	2-scf00041	2
AX-157937282	3	79.511	KV452733.1	3640261	2-scf00041	2
AX-157937289	3	79.511	KV452733.1	3645498	2-scf00041	2
AX-157937297	3	79.511	KV452733.1	3652630	2-scf00041	2
AX-157937299	3	79.511	KV452733.1	3653053	2-scf00041	2
AX-157937300	3	79.511	KV452733.1	3653461	2-scf00041	2
AX-157814457	3	79.511	KV452733.1	3654300	2-scf00041	2
AX-157937301	3	79.511	KV452733.1	3656484	2-scf00041	2
AX-157814458	3	79.511	KV452733.1	3656989	2-scf00041	2
AX-157713085	3	79.511	KV452733.1	3658294	2-scf00041	2
AX-157937303	3	79.511	KV452733.1	3660964	2-scf00041	2
AX-157937305	3	79.511	KV452733.1	3662667	2-scf00041	2
AX-158405652	3	79.511	KV452733.1	3665309	2-scf00041	2
AX-157937310	3	79.511	KV452733.1	3669225	2-scf00041	2
AX-157937311	3	79.511	KV452733.1	3673508	2-scf00041	2
AX-157937314	3	79.511	KV452733.1	3675100	2-scf00041	2
AX-157937315	3	79.511	KV452733.1	3681446	2-scf00041	2
AX-157814461	3	79.511	KV452733.1	3689147	2-scf00041	2
AX-157937321	3	79.511	KV452733.1	3694687	2-scf00041	2
AX-157937327	3	79.511	KV452733.1	3697842	2-scf00041	2
AX-158390020	3	79.511	KV452733.1	3704175	2-scf00041	2
AX-157937335	3	79.511	KV452733.1	3706697	2-scf00041	2
AX-157937346	3	79.511	KV452733.1	3715702	2-scf00041	2
AX-157937355	3	79.511	KV452733.1	3727510	2-scf00041	2
AX-157937364	3	79.511	KV452733.1	3732050	2-scf00041	2
AX-157937366	3	79.511	KV452733.1	3734735	2-scf00041	2
AX-157937370	3	79.511	KV452733.1	3737457	2-scf00041	2
AX-157937372	3	79.511	KV452733.1	3738996	2-scf00041	2
[truncated: 521,474 more chars]
